# A genome-wide comparative evolutionary analysis of zinc finger-BED transcription factor genes in land plants

**DOI:** 10.1038/s41598-022-16602-8

**Published:** 2022-07-19

**Authors:** Athar Hussain, Jinbao Liu, Binoop Mohan, Akif Burhan, Zunaira Nasim, Raveena Bano, Ayesha Ameen, Madiha Zaynab, M. Shahid Mukhtar, Karolina M. Pajerowska-Mukhtar

**Affiliations:** 1 Genomics Lab, School of Food and Agricultural Sciences (SFAS), University of Management and Technology (UMT), Lahore, 54770 Pakistan; 2 Department of Biology, University of Alabama at Birmingham, 1300 University Blvd, Birmingham, AL 35294 USA; 3 Department of Life Science, University of Management and Technology (UMT), Lahore, 54770 Pakistan; 4 Office of Research Innovation and Commercialization, University of Management and Technology, Lahore, 54770 Pakistan; 5 Shenzhen Key Laboratory of Marine Bioresource and Eco-Environmental Sciences, College of Life Sciences and Oceanography, Shenzhen University, Shenzhen, 51807 Guangdong China

**Keywords:** Plant evolution, Genome duplication, Phylogeny, Protein analysis

## Abstract

Zinc finger (Zf)-BED proteins are a novel superfamily of transcription factors that controls numerous activities in plants including growth, development, and cellular responses to biotic and abiotic stresses. Despite their important roles in gene regulation, little is known about the specific functions of Zf-BEDs in land plants. The current study identified a total of 750 Zf-BED-encoding genes in 35 land plant species including mosses, bryophytes, lycophytes, gymnosperms, and angiosperms. The gene family size was somewhat proportional to genome size. All identified genes were categorized into 22 classes based on their specific domain architectures. Of these, class I (Zf-BED_DUF-domain_Dimer_Tnp_hAT) was the most common in the majority of the land plants. However, some classes were family-specific, while the others were species-specific, demonstrating diversity at different classification levels. In addition, several novel functional domains were also predicated including WRKY and nucleotide-binding site (NBS). Comparative genomics, transcriptomics, and proteomics provided insights into the evolutionary history, duplication, divergence, gene gain and loss, species relationship, expression profiling, and structural diversity of Zf-BEDs in land plants. The comprehensive study of Zf-BEDs in *Gossypium* sp., (cotton) also demonstrated a clear footprint of polyploidization. Overall, this comprehensive evolutionary study of Zf-BEDs in land plants highlighted significant diversity among plant species.

## Introduction

The zinc finger (Zf) proteins are a superfamily of proteins involved in a wide range of plant functions, such as growth, development, and resistance to various biotic and abiotic stressors^1,2^. Zinc fingers are small peptide fragments stabilized with zinc ions that interact with nucleic acids and act as regulatory proteins that control transcription^3–5^. The Zf domains are often associated with other domains that modulate their biological functions. One of such domains is the BED domain, consisting of about 50–60 amino acid residues that contain an organized motif with two highly conserved aromatic positions and a shared pattern of cysteines and histidine^3,6^. The BED domain was named after the *Drosophila* proteins BEAF and DREF^7^, and can be found in one or more copies in the genomes of various animals, plants, and fungi^8^. BED-domain proteins have been shown to have DNA binding activity, and some are thought to be involved in chromatin insulation and ethylene regulation^9^.

The zinc finger-homeodomain proteins are categorized into nine types based on their structural and functional variation including C2H2, C8, C6, C3HC4, C2HC, C2HC5, C4, C4HC3, and CCCH (where C and H represent cysteine and histidine, respectively)^10–13^. Various zinc finger domains-containing proteins such as Lesion Simulating Disease Resistance 1 (LSD1) (C2C2), LSD One Like ( LOL1) (C2C2), ZAT12 (C2H2), ZAT7 (C2H2) and AtNFX1 (NF-X1) in *Arabidopsis*^14,15^, StZFP1 (Zinc Finger Protein) (C2H2) in *Solanum tuberosum* (potato) and OsLSD1 (C2C2), OsLOL1 (C2C2), OsRING-1 (RING H2, RING HC), OsRFP1, OsDOS (CCCH), OsZFP (C2H2) & SRZ1(C2C2) in *Oryza sativa* (rice)^11,13,16^. Recent reports indicate that zinc finger motifs have an important role in host–pathogen interactions^17^. For instance, multiple resistance proteins contain a Zf-BED domain, conferring immunity to various biotic stresses^18^. The Zf-BED domains also associate with the Nod-like receptor (NLR) proteins either at the N– or C– termini^17,19–21^. As such, NLR proteins play critical roles in plant immune responses^22–24^. Many defense proteins of *Arabidopsis* and rice containing zinc finger domains have also been shown to regulate programmed cell death (PCD)^2,25^. Likewise, Zf-BED proteins participate in numerous abiotic stresses including leaf senescence^26,27^.

In the current study, we have analyzed the evolutionary relationship of Zf-BED-containing genes in 35 land plant species including algae, bryophytes, gymnosperms, and flowering plants. Our study included identification, characterization, comparison, evolutionary relationship, orthologues, and species-based trees in all land plants. As a focal species for our analyses, we selected cotton (*Gossypium* sp.), which showed an unusually high number of Zf-BEDs and is an economically important crop plant worldwide that is also targeted by various deadly pathogens^28–30^. Here, we report a comprehensive genome-wide comparative study of Zf-BED among diverse plant species with a special focus on *Gossypium* sp. The presented results will provide a foundation for further functional analyses of the Zf-BED proteins in plants.

## Methods

### Plant genomes assemblies

In the current study, we have selected 35 land plants for genome-wide identification and evolutionary analysis of Zf-BED-containing genes. The selected plant species span the evolutionary spectrum from algae to higher plants including *Chlamydomonas reinhardtii* (Algae)*, Marchantia polymorpha* (Bryophytes), *Selaginella moellendorffii* (Lycophytes), *Picea abies* (Gymnosperms)*, Ginkgo biloba* (Gymnosperms)*, Spirodela polyrhiza* (Monocots), *Zea mays* (Monocots), *Sorghum bicolor* (Monocots), *Oryza sativa* (Monocots), *Brachypodium distachyon* (Monocots), *Hordeum vulgare* (Monocots), *Amborella trichopoda* (basal Angiosperms), *Nelumbo nucifera* (basal Angiosperms), *Aquilegia coerulea* (Dicots), *Solanum lycopersicum* (Dicots), *Solanum tuberosum* (Dicots), *Solanum melongena* (Dicots), *Vitis vinifera* (Dicots), *Citrus clementina* (Dicots), *Theobroma cacao* (Dicots), *Gossypium raimondii* (Dicots), *Gossypium arboreum* (Dicots), *Gossypium barbadence* (Dicots), *Gossypium hirsutum* (Dicots), *Gossypium turneri* (Dicots), *Gossypium herbaceum* (Dicots), *Gossypium thurberi* (Dicots), *Capsella rubella* (Dicots), *Arabidopsis thaliana* (Dicots), *Arabidopsis lyrata* (Dicots), *Prunus persica* (Dicots), *Populus trichocarpa* (Dicots), *Medicago truncatula* (Dicots), *Betula pendula* (Dicots) and *Cucumis sativus* (Dicots). We have downloaded publicly available latest genome assemblies from their respective genome databases as well as NCBI, Phytozom, Plaza, etc. (Table S1).

### Identification of Zf-BED and associated domains

To screen the Zf-BED domain-containing genes, the Pfam (protein family) databases were downloaded and installed on the local server. The pfamScan.pl algorithm was used to search for Zf-BED domains with e-value (1.1e−50) in Pfam-A. The *pfamscan.pl* output file was processed. This led to the filtering of all genes containing Zf-BED domains and considered *Zf-BED* genes. To find additional Zf-BED associated domains, we followed Hussain et al.^18^ protocol and used *domain.pl* to arrange domains at their respective position. Based on the additional associated domains, the identified *Zf-BED* containing genes were also classified.

### Evolution and diversity of Zf-BEDs in land plants

To provide an understanding of evolution and diversity in Zf-BED proteins among land plants, we used an advanced comparative genomics tool, OrthoFinder^31^. The DIAMOND tool was used for fast sequence similarity searches. The graph clustering was done with the MCL clustering algorithm. The gene tree inference and distance matrix of the orthogroups were constructed with DendroBLAST^32^. A distance-based phylogeny tree was constructed using FastME 2.0. For multiple sequence alignments, MAFTT 7.0^33^ was used. The maximum likelihood phylogenetic tree of large alignment was constructed using FastTreeMP^34^ with 1000 bootstrap values. Species based phylogenetic tree was also constructed using the same method.

### Class-wise gene ontology and 3D structure prediction

The ontology analysis was carried out using Gene Ontology Resource^35^. The class-wise ontology distribution was made in Microsoft PowerPoint. To predict the 3D structures of Zf-BED proteins, we have selected one representative protein sequence from each class. The 3D structures were predicted using the direct build model option with an open template. The server itself searches for the most similar template from PBD and predicts based on template information. The predicted structures were visualized through PyMol ^36^. The conserved motifs were predicted through the MEME suite motif analysis tool^37^ and graphically presented with TBtools.

### Chromosomal location, physical properties and *cis*-acting elements of Zf-BEDs

To determine the chromosomal distribution of Zf-BED encoding genes in *Gossypium* sp., *A. thaliana* and *Z. mays*, the GFF3 files were downloaded from Cottongen, TAIR, and MaizeGDB database^38–40^, and plots of gene density on chromosomes were generated. To identify and compare the *cis*-acting regulatory element of *Zf-BED* genes of *G. arboreum* , *G. barbadence,* and *G. hirsutum*, upstream sequences of 2 kb sequences were retrieved from CottonFGD^41^ and subjected to analyses in PlantCARE^42^. The physio-chemistry analysis of Zf-BED proteins was carried through the ExPASy ProtParam tool^43^ including molecular weight (MW), theoretical pI (IEP), polarity, aliphatic index, and grand average of hydropathicity index (GRAVY).

### Expression profiling of *Zf-BED* genes

To find the differential expression and response of *Zf-BED* genes in different tissues and stresses, we have retrieved FPKM values of Zf-BED genes from Cotton Functional Genomics Database (CottonFGD) under data-fetch and expression analysis with Bioproject PRJNA490626 and PRJNA594268. The collected data covered FPKM values of genes in the root, stems, leaf, oval fiber, and filament at different time intervals and under abiotic stresses including cold, heat, salt and drought^44^. For the biotic stresses, we downloaded RNA-seq SRA BioProject (PRJNA390823)^25^ and PRJNA398803^45^ SRA and processed them with transcriptomics pipelines. The FPKM values are used to generate a heatmap in TBTools under the heatmap graphics tool. The values were adjusted with log2 base and row and column clusters options^41^.

For the expression profiling of *Arabidopsis thaliana* and *Zea mays* *Zf-BED* genes, the FPKM values were collected from *Arabidopsis* and maize RNA-seq Database^46^ including tissue-specific expression (root, leaf, seedling, shoot, stem, meristem, flower, pollen, seed, embryo and endosperm), biotic stresses (*Acinetobacter radioresistens* strain SA188, *Blumeria graminis*, *Botrytis cinerea*, *Colletotrichum tofieldiae*, *Fusarium graminearum*, *Heterodera schachtii*, *Hyaloperonospora arabidopsidis*, *Microbacterium sp*, *Plutella xylostella*, *Pseudomonas syringae*, *Rhizobium sp*, *Rhizotonia solani*, *Scierotinia scierotiorum*, tobacco mosaic virus, *Verticillum dahliae*) and abiotic stresses (cold, dark, dehydration, drought, heat, hypoxia, irradiation, nutrient deficiency, osmotic, oxidative, ozone, salt, shade, UV, water, wounding).

### Plant material, growth conditions, and treatments

All work was performed with *Arabidopsis thaliana* ecotype Col-0 and *Solanum lycopersicum* L. (tomato) cultivar Micro-Tom. Col-0 seeds, purchased from the Ohio State University’s *Arabidopsis* Biological Resource Center, were directly sowed on Fafard Germination Mix in 90 mm × 68 mm Pöppelmann TEKU round pots and cold stratified in the cold room (4 ºC) for three days. Plants were grown on growth racks at 23 °C  with a 12 h light/12 h dark cycle with a light intensity of 120 µE/m^2^/s and watered every three days with tap water to keep soil moisture near field capacity. 10-day-old seedlings were transplanted to 12 × 6 Landmark Plastic flats with continuous irrigation till day 28. For biotic treatments, *Pseudomonas syringae* pv. tomato DC3000 (hereafter DC3000) or HrcC^−^ (a type III secretion system mutant of DC3000) with OD_600nm_ = 0.0002 in 10 mM MgCl_2_ was syringe-infiltrated into the leaves of 28-day-old *Arabidopsis* plants as described^47^. Six plants with four leaves per plant were infiltrated and collected at 48 h for the following quantitative real-time polymerase chain reaction (qRT-PCR). For drought stress, six plants were grown without further input of water since day28 while six plants were supplied with 3 ml tap water per plant every day till day35. Heat stress was performed on 4-week-old *Arabidopsis* for 1 h at 37 °C while plants were kept at room temperature as a control group. Samples were collected at each corresponding time point and then quickly frozen in liquid nitrogen followed by storing at − 80 °C for the next step. Three leaves were harvested for each replicate and three biological replicates were prepared for each experiment.

Micro-Tom seeds (Totally Tomatoes Seed Company, Randolph, WI, USA) were germinated on 150 mm Whatman filter papers saturated with tap water and placed on 150 mm × 15 mm Fisherbrand Petri Dishes. Plants were grown on growth racks at 23 °C with a 12 h light/12 h dark cycle with a light intensity of 120 µE/m^2^/s. Seven-day-old seedlings were transplanted to 12 × 6 Landmark Plastic flats with Fafard Germination Mix and watered every two days to keep moisture near field capacity till day 21. For biotic treatment, DC3000 or HrcC^−^ with OD600_nm_ = 0.0002 in 10 mM MgCl_2_ was syringe-infiltrated into the leaves of 21-day-old Micro-Tom plants. Six plants with three leaves per plant were infiltrated and collected at 48 h post-inoculation for downstream RT-qPCR analyses. For drought stress, six plants were grown without further input of water since day21 while six plants were supplied with 3 mL of tap water per plant every day till day28. Samples were collected at each time point and then quickly frozen in liquid nitrogen followed by storage at − 80 °C for the next step. Two leaves were harvested for each replicate and three biological replicates were prepared for each experiment.

### RNA extractions and qRT-PCR

Leaf samples of Col-0 and Micro-Tom were fine ground with Bead Ruptor 96 Well Plate Homogenizer. Total RNA of *Arabidopsis* was extracted using the TRIzol (Invitrogen) according to the manufacturer’s protocol^48^. 1 ml TRIzol was used per sample. The RNA pellet was dissolved in 20 μl DEPC-treated water and quantified by BioPhotometer Plus (Eppendorf). Total RNA of Micro-Tom was extracted using Direct-zol RNA MicroPrep kit (Zymo Research) according to the manufacturer’s protocol. 10 μg of *Arabidopsis* and Micro-Tom RNA were then treated with DNAse using the TURBO DNA-free Kit (Ambion) in a 20 μl reaction. cDNA was synthesized using SuperScript IV reverse transcriptase first-strand synthesis kit (Invitrogen) with 2 µg DNA-free RNA in a 10 μL reaction. PCR programs from both DNAse treatment and reverse transcription reaction were performed by using Applied Biosystems 96 Well Thermal Cycler. qRT-PCR was performed on an ABI 7500 Fast PCR System (ThermoFisher Scientific) with 2X PowerUp SYBR green master mix (Applied Biosystems, ThermoFisher Scientific) using the following settings: 50 °C for 2 min and 95 °C for 10 min followed by 40 cycles of 95 °C for 15 s, 55 °C for 15 s and 72 °C for 1 min.

### Ethics approval and consent to participate

The study is conducted in accordance with local and national regulations.

## Results

### Genome-wide identification and classification of Zf-BED encoding gene in land plants

**Figure 1:**
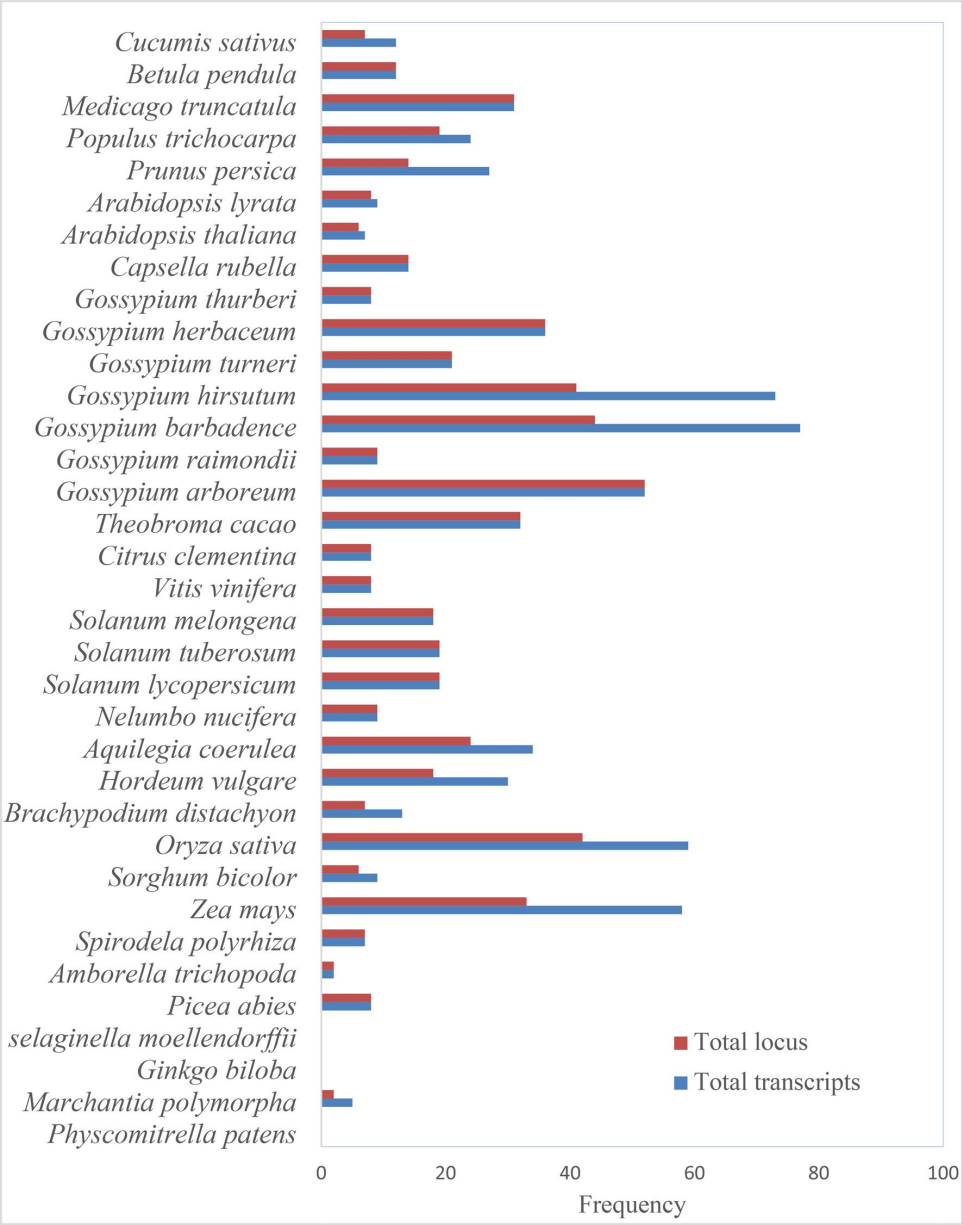
Distribution of Zf-BED encoding genes in land plant species.

The whole-genome screening of Zf-BED-encoding genes in 35 plant species demonstrated that three lower land plants do not contain any sequences resembling the *Zf-BED* gene family. In total, we identified 750 Zf-BED encoding genes, which were distributed across the remaining 32 plant species. Interestingly, the highest number of genes were observed in the cotton plants including *G. barbadence* (77 genes), *G. hirsutum* (73 genes), *G. arboreum* (52 genes), *G. herbaceum* (36 genes), however *Oryza sativa* (59 genes) and *Zea mays* (58 genes) also had large number of genes followed by *Aquilegia coerulea* (34 genes), *Theobroma cacao* (32 genes), *Medicago truncatula* (31 genes), *Hordeum vulgare* (30 genes), *Prunus persica* (27 genes), *Populus trichocarpa* (24 genes), *G. turneri* (21 genes), *Solanum lycopersicum* (19 genes), *S. tuberosum* (19 genes), *Solanum melongena* (18 genes), *Capsella rubella* (14 genes), *Brachypodium distachyon* (13 genes), *Betula pendula* (12 genes), *Cucumis sativus* (12 genes), *Sorghum bicolor* (9 genes), *Nelumbo nucifera* (9 genes), *G. raimondii* (9 genes)*, Arabidopsis lyrata* (9 genes), *Picea abies* (8 genes), *Vitis vinifera* (8 genes), *Citrus clementina* (8 genes), *G. thurberi* (8 genes), *Spirodela polyrhiza* (7 genes), *Arabidopsis thaliana* (7 genes), *Marchantia polymorpha* (5 genes) and *Amborella trichopoda* (2 genes) (Fig. 1, Tables S1–S2).

**Figure 2:**
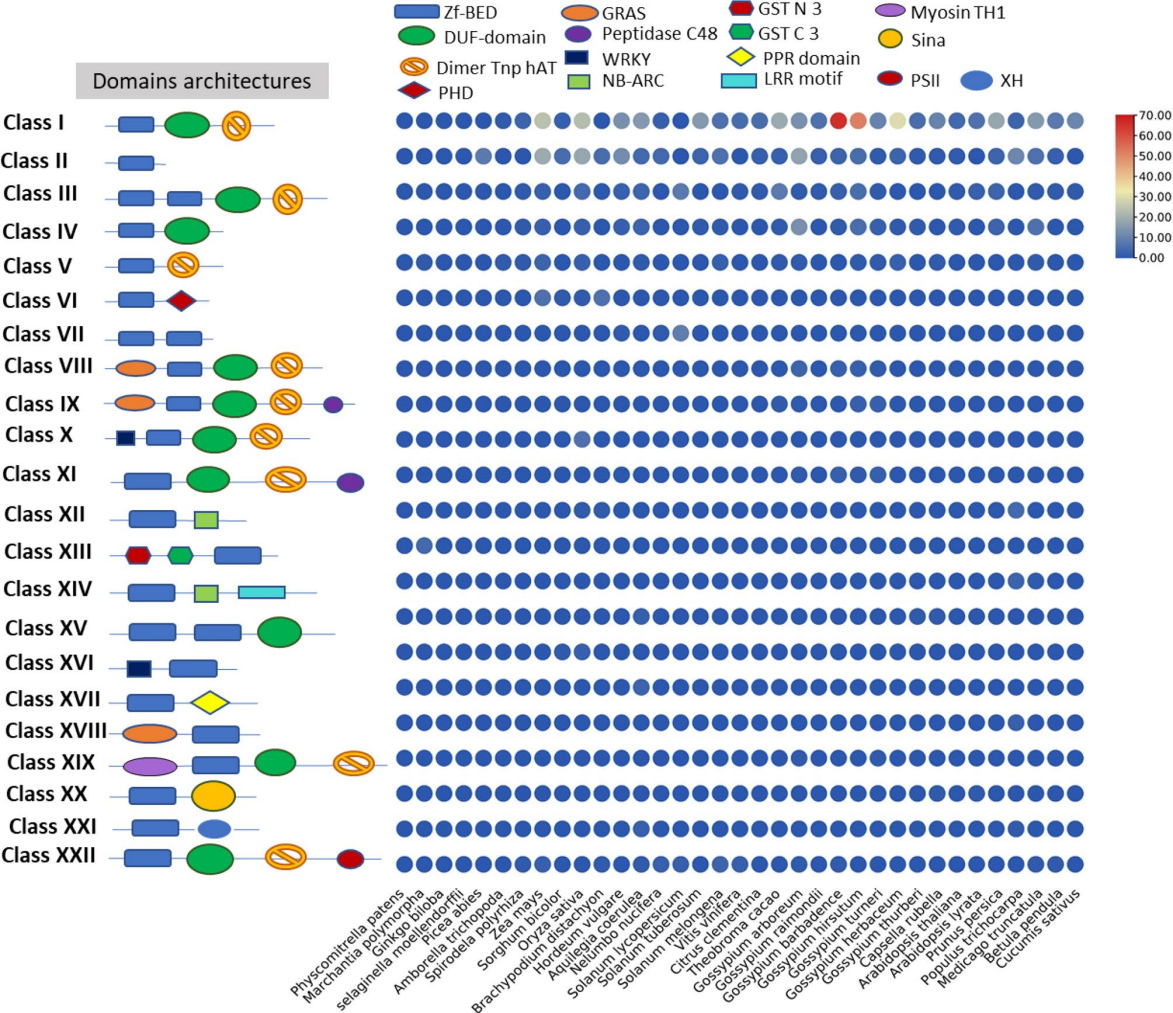
Classes of Zf-BED proteins based on conserved domains and their comparison among land plants.

Based on the conserved domains, motifs, and their patterns in the primary sequence of proteins, we have classified all 750 genes into 22 major classes named class I-XXII. Of these 22 classes, class I (Zf-BED–DUF-domain-Dimer_Tnp_hAT) and class II (Zf-BED ) were more common in all plant species, containing 390 and 144 genes, respectively, followed by class III (Zf-BED-Zf-BED-DUF-domain-Dimer_Tnp_hAT), IV (Zf-BED–DUF-domain), V (Zf-BED-Dimer_Tnp_hAT), and VI (Zf-BED-PHD). The classification of Zf-BED-encoding genes reported several new domains associated with Zf-BED domains. The vast majority of these domains are involved in plant defense functions, such as the GRAS domain (gibberellin signaling regulator), PHD (Cys4-His-Cys3 motif in the plant homeodomain), WRKY (DNA-binding domain and biotic and abiotic stress regulator), NBS (Nucleotide-Binding Site; one of the major superfamilies of plant resistance genes), Sina domain (N-terminal RING finger domain), F-box with LRR-motif, etc. We have also observed diversity in the presence and absence of these 22 classes in 32 plant species such as the *Marchantia polymorpha*, which is a liverwort (bryophyte) and has only five genes i.e., one gene from class V (Zf-BED-Dimer_Tnp_hAT) and four genes from class XIII (GST_N_3-GST_C_3-Zf-BED; Glutathione S-transferase, C-terminal domain). Similarly, in the *Picea abies* genome, a gymnosperm plant, there were only eight genes, all of which belonged to class II (Zf-BED). The *Amborella trichopoda*, considered a basal species among the flowering plants, has only two genes (one from class I and another from class V). The *Spirodela polyrhiza*, a monocot near to basal of angiosperms, has seven *Zf-BED* genes i.e., three genes in class I and one in each class III, IV, V and XXII. In summary, a distinct pattern could be identified among monocots since all studied species, except *Oryza sativa*, contained *Zf-BED* genes that belonged to the first six classes (I-VI), while the dicot plants showed a more diverse sampling of the *Zf-BED* genes across various classes. In the dicot plants, especially in *Gossypium* sp., the *G. raimondii* genome exhibited only the first three classes (I-III), which is consistent with its conserved genome, while other *Gossypium* species showed a large number of genes in other classes. It was also observed that most of the classes were only specific to *Gossypium* sp. For example, the class VIII (GRAS-Zf-BED-DUF-domain-Dimer_Tnp_hAT), IX (GRAS-Zf-BED-DUF-domain-Dimer_Tnp_hAT-Peptidase_C48) and XI (Zf-BED-DUF-domain-Dimer_Tnp_hAT-Peptidase_C48) were found only in *Gossypium* sp. (Fig. 2, Figure S1, Table S3–S4). Overall, we found that diverse plant species possess different *Zf-BED* gene classes, highlighting the genomic diversity in land plants.

### Evolutionary study of Zf-BEDs in land plants

To find the evolutionary relationships among the Zf-BED-encoding genes, we selected 25 land plants including *Aquilegia coerulea (Aqu), Arabidopsis thaliana (At), Betula pendula (Bpe), Brachypodium distachyon (Bra), Capsella rubella (Car), Citrus clementina (Cic), Cucumis sativus (Cuc), G. arboreum (Gar), Gossypium barbadence (Gba), Gossypium herbaceum (Ghe), G. hirsutum (Ghi), G. thurberi (Got), G. raimondii (Gra), Vitis vinifera (Gsv), Nelumbo nucifera (Nnu), Oryza sativa (OsR), Picea abies (Pab), Prunus persica (Pru), Solanum tuberosum (Sot), S. lycopersicum (Sol), S. melongena (Sme), Sorghum bicolor (Sob), Spirodela polyrhiza (Spi), Theobroma cacao (Tca),* and *G. turneri (Gtu),* and used their Zf-BED protein sequences to determine comparative genomics relationships, gene duplication events, gene trees, orthogroups, orthologs, putative xenologs, and species trees.

The comparative genomics analyses revealed that all *Zf-BED*s were divided into 9 orthogroups (OG0; 330 genes, OG1;168 genes, OG2; 23 genes, OG3; 20 genes, OG4; 15 genes and OG6 to OG8 each with 2 genes) (Figure S3, Table S7) covering 564 genes (99.8% of genes in orthogroups) with only one unassigned gene. Of these orthogroups, only one orthogroup was common to all 26 species, while three orthogroups were species-specific, containing only six genes each. Overall, the orthogroup mean and the median were computed as 62.7 and 15 genes, respectively (Figure S2, Table S5).

The species-wise orthogroups distribution showed that all species shared orthogroups (100% genes in orthogroups). Some orthogroups were species-specific; for instance, *O. sativa* had two species-specific orthogroups containing four genes (6.9% of genes). Similarly, *S. melongena* had one species-specific orthogroup containing two genes (13.3% of genes) (Figure S4, Table S6, Table S8, and Table S10–S13).

**Figure 3:**
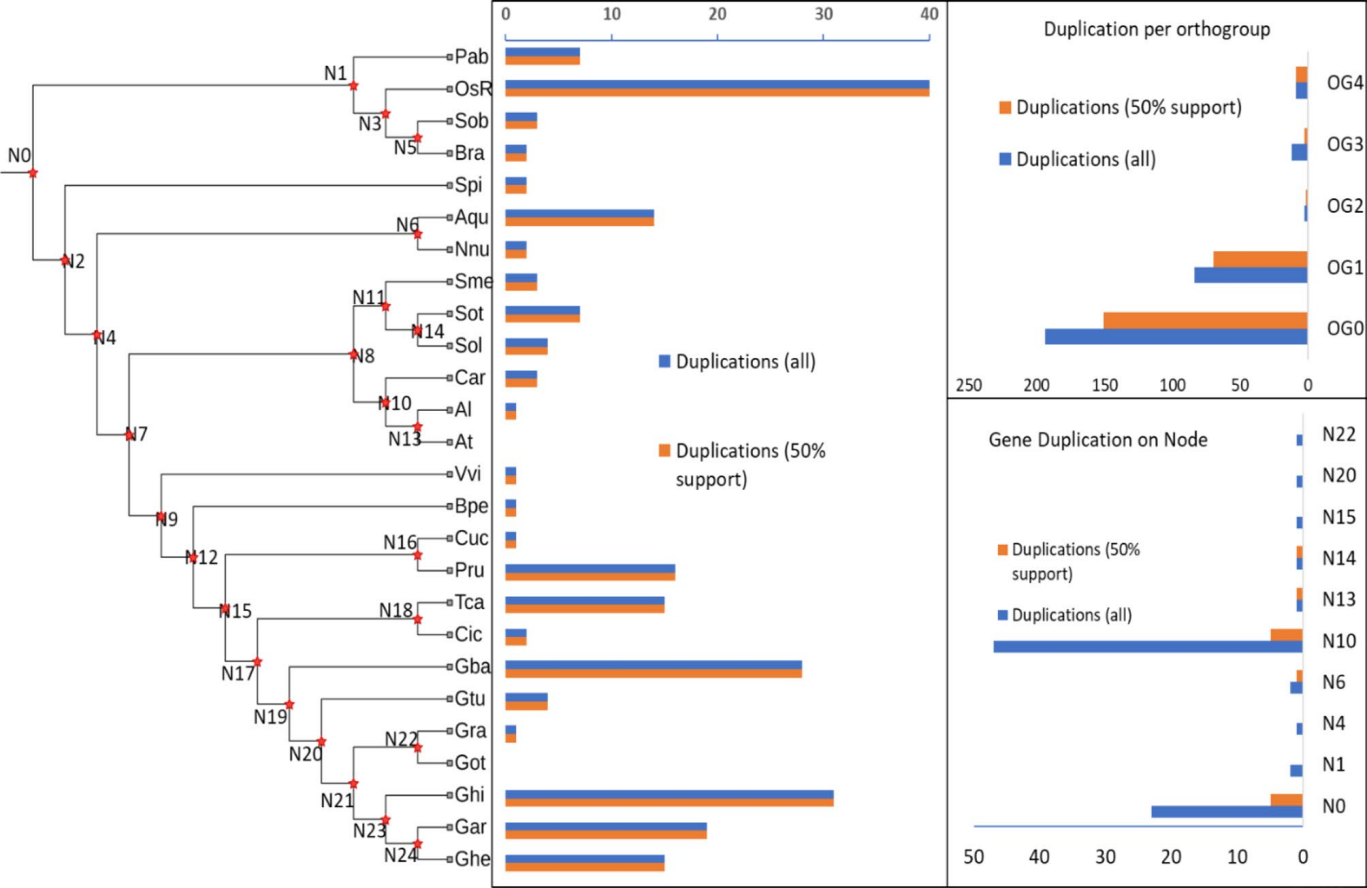
Evolutionary summary; species-tree with duplication events at the terminal and internal nodes and duplication at orthogroup level.

The orthogroup duplication events demonstrated that only five orthogroups (OG0 to OG4) passed through duplication events during the evolutionary time scale. The highest duplication numbers were recorded in OG0 (194 duplications) followed by OG1 (84 duplications), OG3 (12 duplications), OG4 (9 duplications), and OG2 (3 duplication events) (Fig. 3, Table S9).

For the species-based phylogenetic tree analysis, 26 representative species were included. The phylogenetic tree was divided into three major clades representing two clades of dicot plants and one clade of monocots. The gene duplication events at internal nodes and terminal nodes also demonstrated remarkable gene duplication in higher plants. For instance, in the case of the monocots clade, the highest gene duplication was observed in *O. sativa* (40 duplications) followed by *P. abies* (8 duplications). Similarly, in the dicot clades, the highest duplications are found in tetraploid species of cotton (*G. barbadence* and *G. hirsutum* with 29 and 31 gene duplication events, respectively). Furthermore, several gene duplication events were also found in the internal nodes, and the highest duplications occurred at N10 (49 duplications) (Fig. 3, Table S14). In summary, higher plants experienced more duplication events during evolutionary processes and different plant families carry species-specific orthogroups displaying their unique genetic makeup.

### Gene ontologies, conserved residues, and motifs in Zf-BEDs

**Figure 4:**
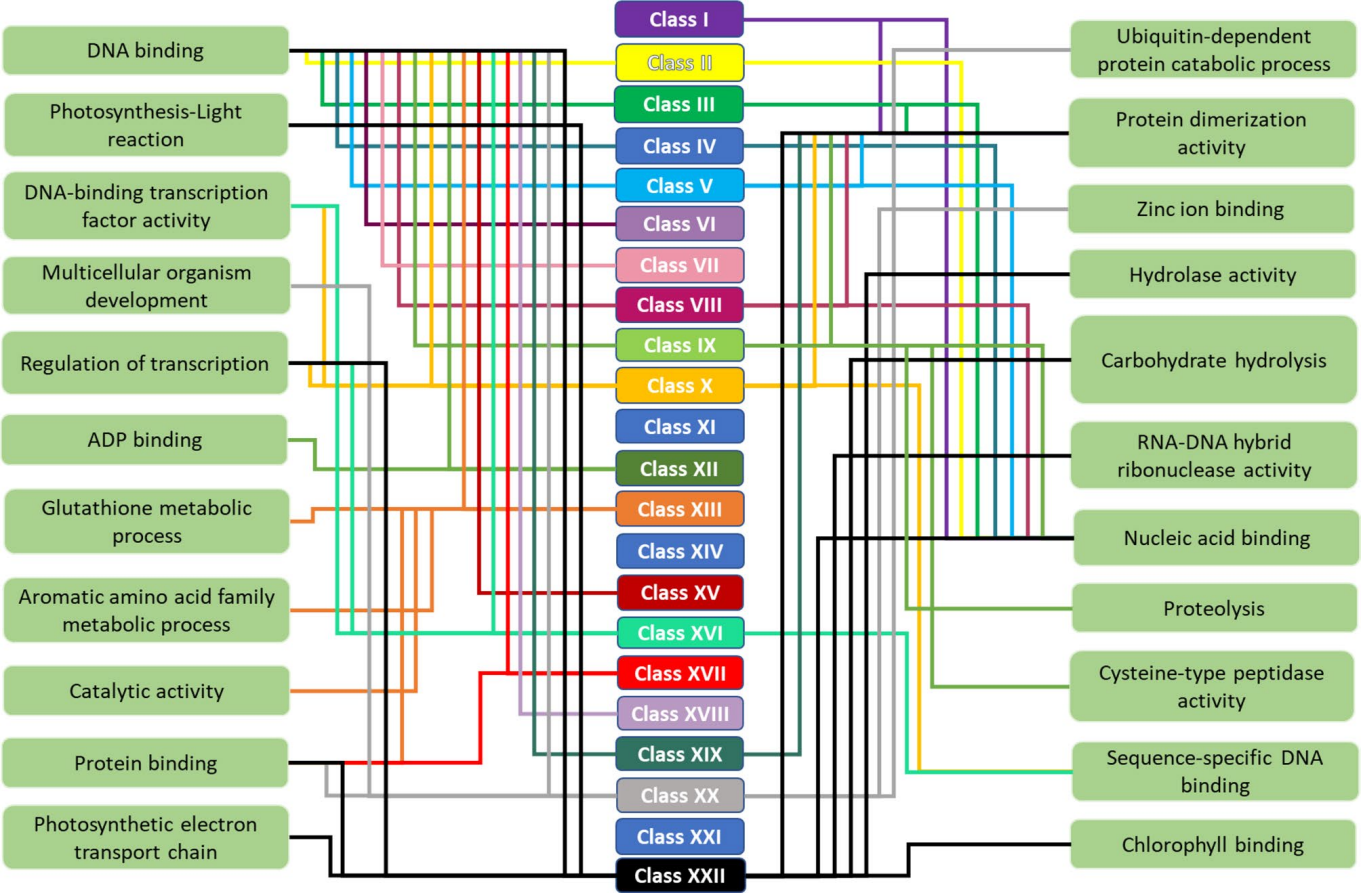
Zf-BEDs classes with their gene ontology-predicted molecular functions.

The gene ontology prediction of all *Zf-BED* genes indicated that all of them were involved in diverse molecular functions, mainly “DNA binding”, “nucleic acid binding”, and “protein dimerization” activities. However, different classes also exhibited additional diverse functions such as “hydrolase activity”, “regulation of transcription”, and “catabolic process”. The class-wise gene ontology demonstrated their putative roles in different molecular functions. For instance, class I is involved in protein dimerization and nucleic acid binding. Similarly, class II is implicated in DNA binding/nucleic acid binding activity. However, some classes were involved in more than then one molecular function, such as class XIII, which is required for DNA binding, metabolic processes, aromatic amino acid metabolism, catalytic activity, and protein binding activity. Class XXII contained different domain architectures, and consequently its members engaged in diverse functions including photosynthesis, chlorophyll binding, RNA–DNA hybrid ribonuclease activity, hydrolase activity, etc. (Fig. 4, Table S15). In summary, the *Zf-BED* gene ontology varied with the addition of associated domains. Therefore, the associated domains appear to have a high impact on the function of the Zf-BED proteins.

The multiple sequence alignment of Zf-BED proteins demonstrated highly conserved functional residues in the active sites. However, the lower plants showed highly diverse residues at functional sites. For instance, W^7^ was conserved in 87% sequences. Similarly, H^9^, F^10^, C^20^-C^23^ (X^2–4^ motif), G^33^, G^38^, T^39^, L^42^, K^43^, H^45^ (His motif), L^46^, C^52^, D^65^, P^83^, F^92^, E^124^, W^146^, C^247^, D^259^, L^318^, W^346^, P^372^, K^409^, and L^592^ sites were conserved in more than 60% of the Zf-BED proteins (Fig. S5). Most of the sequence variations at these sites were either genus-specific or species-specific. Conserved motif analysis also demonstrated the insertion or deletion of a specific motif in different classes of Zf-BED proteins. We also observed similar motif patterns in the same class members in the same genome. For instance, Class_I of GbaZf-BEDs had similar motifs with the same pattern. The CX2-4C motif (motif-5) was conserved in all classes and all land plants. WX[YH]F (motif_14) at the C-terminus was also found in most of the classes except classes XIII, XIV, XV, and XXI. Similarly, motif GTXXLXXH[LT] (motif_13) was also conserved in the majority of the proteins. In summary, most of the functional motifs were conserved in more than 90% of the analyzed sequences. However, some motifs were species-specific, while the others were Zf-BED classes-specific (Figures S5–S6).

### Homology-based modeling of Zf-BED classes

**Figure 5:**
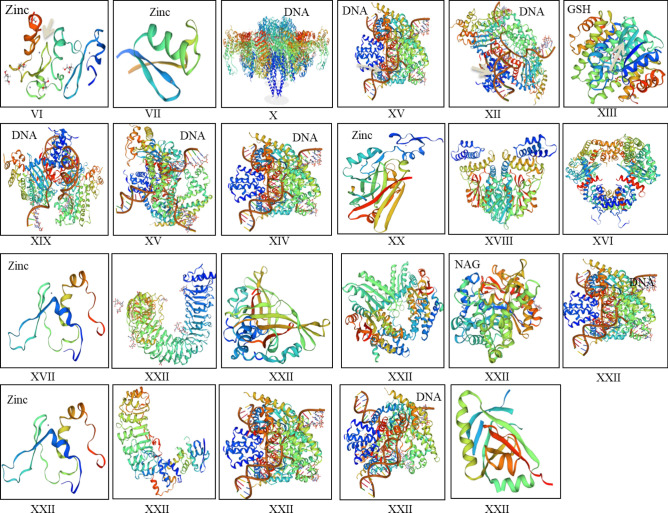
Protein 3D models for different classes of Zf-BED with their ligands; Zinc, DNA, GSH.

The homology-based 3D structures of different Zf-BED classes displayed different associations with their ligands. Most of the classes’ structures were associated with zinc ions in addition to DNA. Some other ligands exhibited a tendency to interact with different molecules. We have determined representative 3D structures with their associated ligands for each class. The comparative study provided clear differences in the structure, active sides, and ligands. For instance, class VI, VII, XX, and XXI proteins showed their association with zinc ion ligands in addition to some additional small molecules. Whereas, some classes e.g. X, XVI, and XVIII did not show any ligands in their structures. Furthermore, some classes (XV, XII, XIV, and XIX) were associated with DNA molecules. Similarly, class XIII was associated with glutathione (GSH) and NAG molecules (Fig. 5).

### Evolution of *Zf-BED* gene family in *Gossypium* sp.

To provide a detailed and comprehensive analysis of the *Zf-BED* genes in *Gossypium* species, we also presented the evolutionary relationship among five *Gossypium* sp., (*G. hirsutum, G. barbadence, G. arboreum, G. herbaceum* and *G. raimondii) with T. cacao.* The comparative genomics summarized that all *Zf-BEDs* from 6 species were divided into 15 orthogroups encompassing 179 genes (98.9% of genes in orthogroups). Of these orthogroups, only four orthogroups were common in all species, while two orthogroups were species-specific. Similarly, only four genes were in species-specific orthogroups. Overall, the orthogroup mean and medians were computed as 11.9 and 11 genes, respectively.

**Figure 6:**
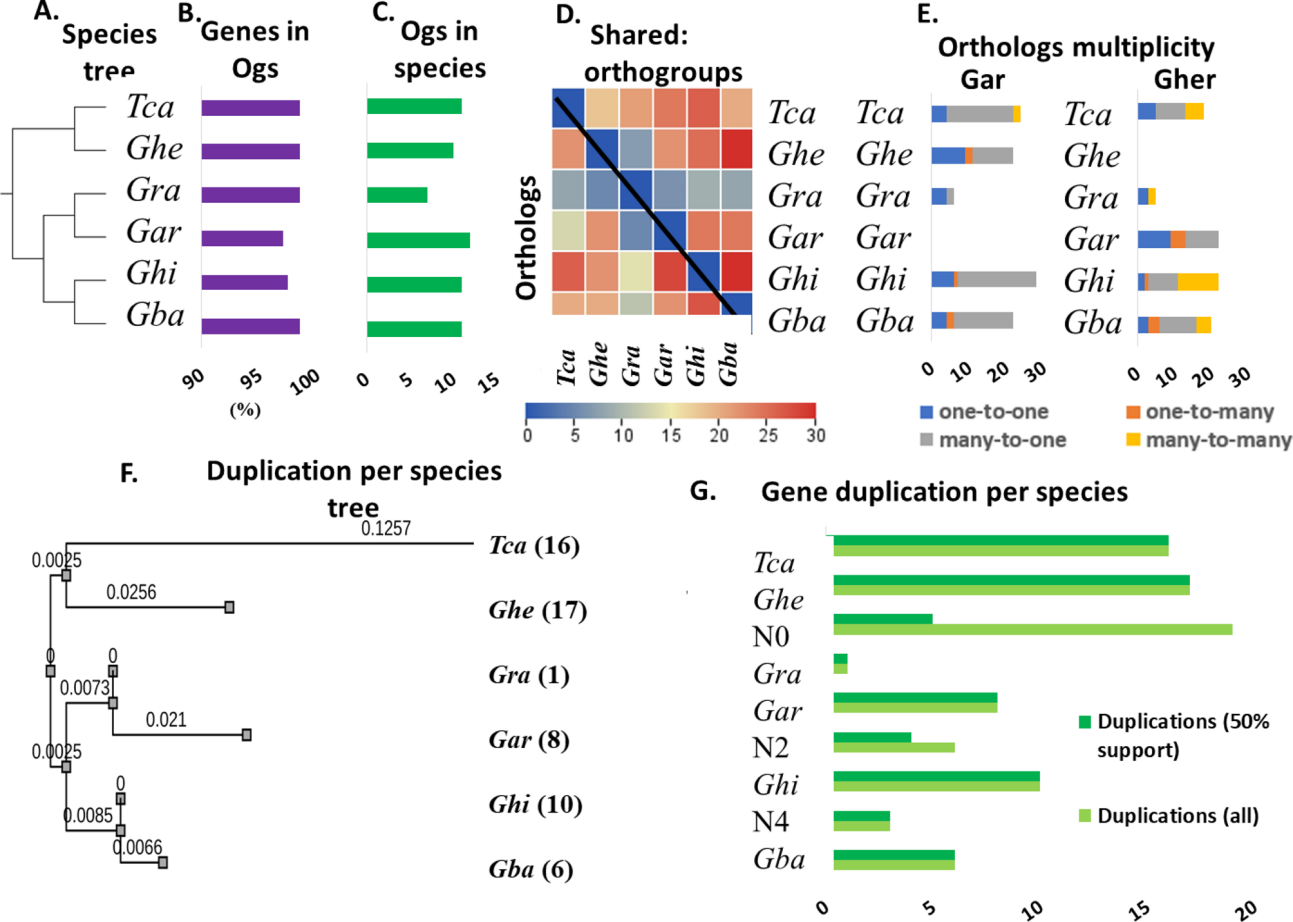
Summary of evolutionary analysis of *Zf-BED*s in *Gossypium* sp. (**A**) Species-based phylogenetic tree, (**B**) percentage of genes in orthogroups, (**C**) number of species-specific orthogroups, (**D**) heatmap of orthologs statistics one-to-one, (**E**) Heatmap of orthologs statistics one-to-many.

The species-wise orthogroups distribution represented that most of the identified genes belonged to orthogroups. For instance, 98.0% of genes were in orthogroups with all genes of *Tca, Ghe, Gra,* and *Gba* and the minimum genes in orthogroups were present in *Ghi* and *Gar.* The orthogroup sharing analysis demonstrated that the highest number of orthogroups were shared between *Ghe* and *Ghi* with *Gba*. Similarly, the orthologs multiplicity also depicted significant similarity and uniqueness among six species. The species-based phylogenetic tree showed that *Tca* is the common ancestor for all *Gossypium* species, and *Ghe* has the most conserved Zf-BED encoding genes with *Tca,* followed by the other four cotton species. The duplication events at each terminal node demonstrated that *Tca* has had the high duplication rate, followed by *Ghe, Ghi Gar,* and *Gba*, while *Gra* has experienced only a single duplication event (Fig. 6).

### Gene features and physio-chemistry of Zf-BED protein sequences

The analyzed gene features included physio-chemistry and gene-specific attributes. The physio-chemistry included molecular weight, protein charge, isoelectric point, and grand average of hydropathy. Similarly, gene features comprised transcript length, CDS length, GC contents in CDS sequence, number of exons and introns, lengths of exons and introns, etc. We provided a detailed study of the five species. In the case of *G. arboreum* *Zf-BED*, the transcript length ranged between 0.25 and 5.5 kb, the coding sequence length ranged from 0.25 to 5.5 kb, the percentage of GC content in the coding sequence was 34% to 46%, exon number of genes of *G. arboreum* ranged from a minimum of 1 to a maximum of 17 exons, while the mean exon length ranged between 1 and 6 kbp, mean intron length of genes of *G. arboreum* ranged from 0 to 7 kb, longest protein consisted of 1713 amino acids and the shortest protein had 95 amino acid residues, the molecular weight was observed between 10.58 to 197.52 kDa, protein charge range was − 20 to + 47, the isoelectric point range was 5.15 to 10.28 and the grand average of hydropathy range was − 0.75 to − 0.21. Similar results were also observed in other *Gossypium* species (Figures S7–S8).

### Chromosomal mapping of *Zf-BED* genes

To understand the effect of polyploidization on *Zf-BED* genes between diploid and tetraploid species, a genome-wide comparative distribution of *Zf-BED* genes on chromosomes was determined in all *Gossypium* species. Diverse species had a varied number of genes on different chromosomes. In A-like genomes (*Gar* and *Ghe*), all genes were relatively evenly distributed on all chromosomes except Chr#02, while in the D-like genome (*Gra*), all genes were localized to only six chromosomes. In the case of tetraploid species (*Ghi* and *Gba*), 20 chromosomes and 18 chromosomes possessed *Zf-BED* genes in *Ghi* and *Gba,* respectively. However, some chromosomes showed the highest number of genes in all species. For instance, D02 has the highest number of Zf-BED genes in *G. barbadense* and *G. hirsutum,* followed by Chr#07 in all species except *G. arboreum*. Similarly, some chromosomes did not contain any *Zf-BED* genes, such as Ga2, Ga5, Ga7, Ga10 did not bear any *Zf-BED* genes in *G. arboreum*. A similar pattern was also observed in *G. raimondii* (Gr2, Gr3, Gr5, Gr8, Gr10, Gr11, and Gr12), *G. barbadence* (GbA01, GbA04, GbD01, and GbD10), and in *G. hirsutum* (GhA01, GhA04, GhA09, GhA10, GhD07, and GhD10). In addition to *Gossypium* sp., the chromosomal locations of the *AtZf-BED* and *ZmZf-BED* genes were also mapped on their respective chromosomes. We observed that all *AtZf-BED* genes were localized only on three chromosomes (AT1, AT3, and AT4) while *ZmZf-BED* genes were distributed on all chromosomes except chromosome no. 6 (Zm6) (Figure S9).

### *Cis*-acting regulatory elements prediction of *Zf-BED* genes

The identified *cis*-acting regulatory elements were classified into different categories including response to hormones, response to stresses, growth, and during development. We observed that most of the gene promoters contained gibberellin, abscisic acid, salicylic acid, jasmonic acid, and auxin hormone-responsive elements. Similarly, in stress-responsive elements, we observed low temperature, light, wound, and drought, anoxic and anaerobic responsive factors. Furthermore, the growth and developmental *cis*-acting elements included zein metabolism regulation, seed-specific regulation, meristem expression, endosperm expression, circadian control, and cell cycle regulation. Some additional elements including MYBHv1 binding site, ATBP-1, a MYB binding site involved in flavonoid biosynthetic genes regulation, and CMA3 were also observed in some gene regulatory regions. The comparison of *cis*-acting elements among *G. arboreum*, *G. hirsutum*, and *G. barbadence* demonstrated common regulatory elements including gibberellin-responsive elements, low-temperature responsiveness, as well as wound-responsive elements, light-responsive, and salicylic acid responsive elements (Tables S24–S25).

### Expression profiling of *Zf-BED* genes in cotton, maize, and *Arabidopsis*

The expression profiling of *Zf-BED* genes showed variation with respect to tissues and stresses. In this study, we have included expression profiling of *Zf-BED* genes in *Gossypium* sp., maize, and *Arabidopsis* in different tissues and under various stresses. The expression profiling and cluster correlation of *Zf-BED* in *G. arboreum* demonstrated multiple clusters in different tissues including fiber, leaf, ovule, flower, stem, and root. For instance, a cluster containing *GaZf-BED20, GaZf-BED17,* and *GaZf-BED31* showed relatively high expression in leaf, flower, stem, and root. Similarly, another cluster (*GaZf-BED*11, *GaZf-BED*33, *GaZf-BED*30, *GaZf-BED*09, *GaZf-BED*16) had high transcript abundance in the ovule, flower, stem, and root. Some genes showed high expression levels in stem and root tissues, while other genes displayed little expression in ovule, flower, stem, and root. Interestingly, however, none of the genes displayed any expression in leaf and fiber. In summary, only a few *GaZf-BED* genes presumably play roles in tissue development (Figure S10, Table S27).

The expression profiling of *Zf-BED* genes in *G. hirsutum* in diverse tissues (like fiber, flower, ovule, and leaf at different time intervals) showed different expression patterns of genes. The gene clustering cladogram demonstrated that some genes have putative roles in all tissues. For instance, gene *Gh_A08G102900.1* had increased transcript levels in all four tissues. On the other hand, a set of genes exhibited tissue-specific expression such as *Gh_D08G097100.1, Gh_D07G157800.1, Gh_A08G102900.1,* and *Gh-D07G157800.1. Gh_D06G202500.1* had the highest expression in the leaf (Table S28). The stress-specific expression of *Zf-BED* demonstrated their putative functions under different abiotic stresses including cold, drought, heat, and salt at different time intervals. A cluster of genes such as *Gh_A08G102900.1, Gh_D08G097100.1, Gh_D07G157800.1, Gh_D06G202500.1,* and *Gh_D09G0005*00.1 showed high expression in all stresses including cold, drought, heat, and salt (Table S28). However, several genes exhibited tissue-specific expression. Moreover, we observed a cluster of genes that did not show any expression during the applied stresses.

The expression profiling of *Z. mays* Zf-BED genes also demonstrated tissue-specific and stress-specific. For instance, *ZmZf-BED43* (*Zm00001d033361*) gene is highly expressed only in anther and endosperm, while a cluster of genes including *ZmZf-BED43 (Zm00001d013336), ZmZf-BED26 (Zm00001d017846), ZmZf-BED34 (Zm00001d022534), ZmZf-BED38 (Zm00001d026358),* and *ZmZf-BED02* (*Zm00001d003128*) share a co-expression pattern in all tissues (Figure S11). In addition, the abiotic stress response of *ZmZf-BED* genes also followed the same pattern. A cluster of genes (*ZmZf-BED34, ZmZf-BED45, ZmZf-BED17*, and *ZmZf-BED26*) co-expressed under different abiotic stresses with a variable range of FPKM values (Figure S12). In contrast to biotic and tissues specific stresses, the response of *ZmZf-BED* was highly variable for biotic stresses. For instance, a pair of genes (*ZmZf-BED26* and *ZmZf-BED17*) displayed high expression under all biotic stresses indicating their key roles in biotic stress regulation in *Z. mays*. Whereas, other *ZmZf-BED* genes were highly treatment-specific e.g. *ZmZf-BED34* had high transcript levels under pathogen stresses including Maize Mosaic Virus, *Fusarium virgliforme,* etc. (Figure S13, Table S29).

**Figure 7:**
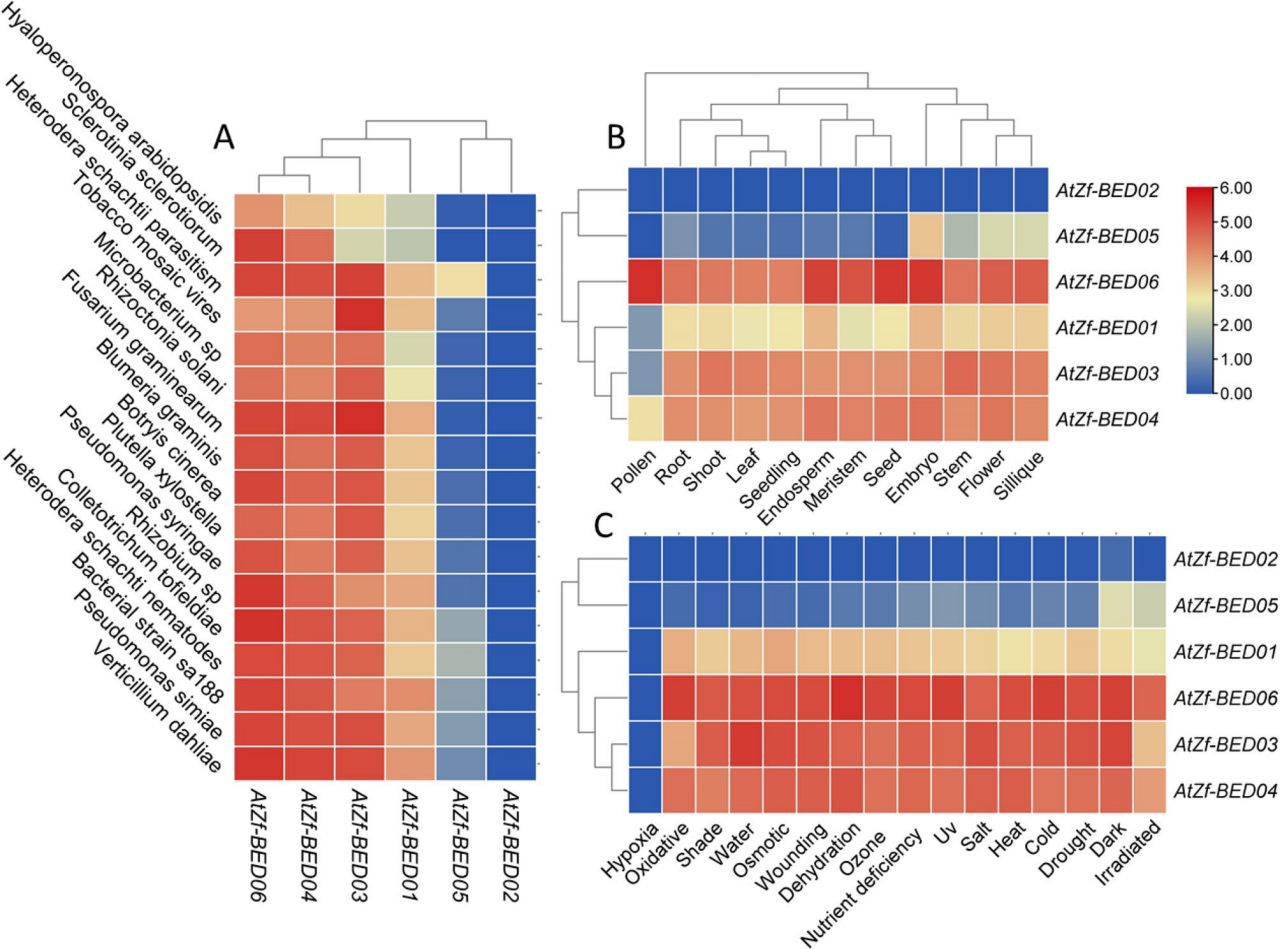
Expression profiling of *AtZF-BED*s. (**A**) under various biotic stresses, (**B**) in different tissues, (**C**) under different abiotic stresses.

The tissue-specific expression profiling of *AtZf-BED* in *Arabidopsis* demonstrated that all genes had high relative expression except *AtZf-BED05* (*AT3G48770*; class-II; Zf-BED) and *AT1G36095* (class-IV; Zf-BED-DUF-domain) forming a common cluster on the dendrogram. Another cluster, which included *AtZf-BED06* *(AT4G15020),* *AtZf-BED01 *(*AT1G18560*)*,* *AtZf-BED03* (*AT1G79740*)*,* and *AtZf-BED04* (*AT3G17450*), had high transcripts abundance in all tissues (pollen, root, shoot, leaf, seedling, endosperm, meristem, seed, embryo, stem, flower and silique). However, the level of transcripts varied among the tested tissues. In contrast to tissue-specific expression, the biotic stress-specific expression was highly variable depending on stress sources. For instance, *AtZf-BED01* (*AT1G18560*)*,* *AtZf-BED03* (*AT1G79740*)*,* *AtZf-BED04* (*AT3G17450*), and *AtZf-BED06* *(AT4G15020)* had increased expression when challenged with *Verticillium dahliae,* followed by *Fusarium graminearum*, Tobacco Mosaic Virus, and bacterial disease. Similarly, under abiotic stresses, *AtZf-BED06* *(AT4G15020),* *AtZf-BED03* (*AT1G79740*)*,* and *AtZf-BED04* (*AT3G17450*) showed heightened transcript accumulation under all stresses including cold, dark, drought, salt, wounding and water deficit (Fig. 7, Table S30).

### Experimental verification of *Zf-BED* genes expression in *Arabidopsis* and Micro-Tom tomato plants

**Figure 8:**
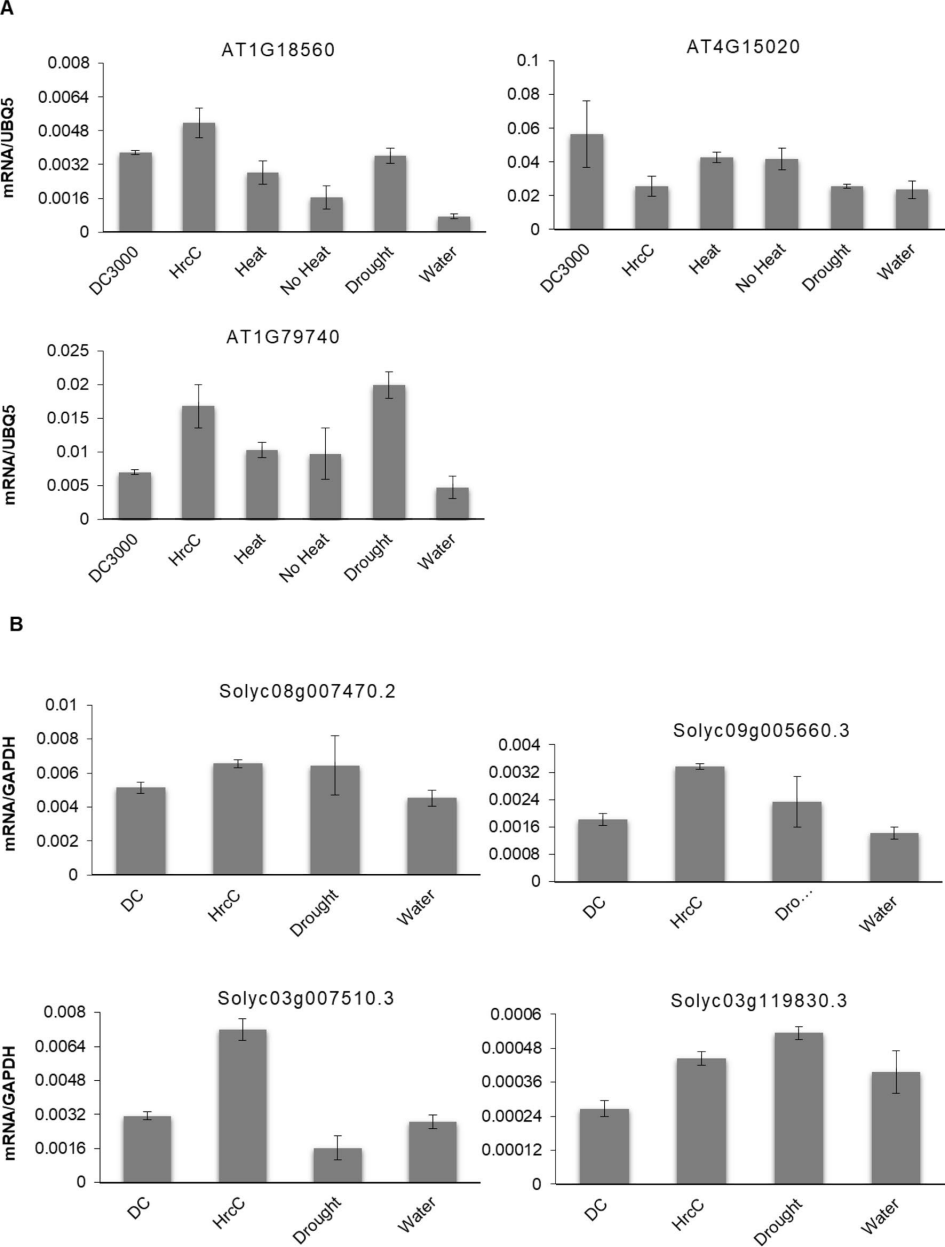
RT-qPCR analysis of *Zf-BED* mRNAs accumulation in Col-0 and Micro-Tom treated by biotic and abiotic stresses. (**A**) Transcript levels of *Zf-BED* genes in 4-week-old Col-0 genotype upon treatment with *Pseudomonas syringae* pv. *tomato* DC3000 (DC3000) and effector less mutant strain Pto DC3000 HrcC- (HrcC-) at 48 h, 37 °C (Heat) and room temperature (No heat) at 1 h, as well as no irrigation (Drought) and normal irrigation (Water) at 7 days. Gene expression was assessed using reference gene *UBQ5* in *AT1G18560, AT4G15020* and *AT1G79740*. (**B**) Transcript levels of *Zf-BED* genes in 3-week-old Micro-Tom genotype upon treatment with DC3000 and HrcC- at 48 h, as well as no irrigation (Drought) and normal irrigation (Water) at 7 days. Gene expression was assessed using reference gene GAPDH in *Solyc08g007470.2*, *Solyc09g005660.3, Solyc03g007510.3* and *Solyc03g119830.3*.

To decipher the relationship of *Zf-BED* family members in *Arabidopsis* and Micro-Tom in response to biotic and abiotic stress, three *Zf-BED* genes in *Arabidopsis* and four orthologous genes in Micro-Tom were selected to examine the transcript level changes under various stresses using qRT-PCR. In *Arabidopsis*, *AtZf-BED01* (*AT1G18560)* and *AtZf-BED03* (*AT1G79740)* were downregulated upon DC3000 infection compared to HrcC^−^, while *AT4G15020* showed the inverse trend (Fig. 8). Under heat treatment, *AtZf-BED01* (*AT1G18560)* transcripts were elevated whereas other *Zf-BED* genes didn’t display significant changes. Furthermore, *AtZf-BED01* (*AT1G18560)* and *AtZf-BED03* (*AT1G79740)* were upregulated significantly in response to drought stress. In Micro-Tom, all *Zf-BED* genes showed lower expression levels under DC3000 treatment compared to HrcC^−^. As for abiotic stress*, Solyc08g007470.2*, *Soly09g005660.3*, and *Solyc03g119830.1* expression was upregulated upon drought, while *Solyc03g007510.3* was downregulated (Fig. 8). Overall, the representative *Zf-BED* genes in *Arabidopsis* and Micro-Tom were shown to be involved in biotic and abiotic stresses.

## Discussion

The study of plant evolution provides evidence to explain the diversity of plants across the geologic time frame. Plant evolution has been shaped by key morphological and developmental novelties that improved plant adaptation, and studying morphological traits is a prime feature of paleobiology and leads to fundamental insights^50^. The gene family expansion and domain rearrangements in land plants hamper the identification of orthologous proteins in different plant species^50^. However, the comparative study on a genome-wide scale enables researchers to discover duplications and other events known to be major drivers of plant speciation^51^.

Owing to the evolution of diverse transcription factors, plants have acquired well-developed defense mechanisms and developed resilience to environmental stresses^52^. Zinc fingers execute their function via binding to nucleic acids and participate in transcriptional regulation processes. The *Zf-BED* genes are one of the important contributing factors in the plant defense system and have been functionally studied in many plant species including *Arabidopsis*, wheat, maize, *Medicago*, sorghum, etc.^15^.

The Zf-BED domains are widely spread in the eukaryotic genomes and actively participate in DNA binding and protein–protein interactions^53–56^. Recent studies demonstrated their integration as decoy domains with other resistance-related domains and their role in the regulation of diverse host functions. The Zf-BED domain with NLR conferred resistance to yellow rust and blast in rice^57,58^. With the identification of Zf-BED as a resistance domain, several experimental studies were carried out and validated their putative role in plant development and adaptation^58–61^. In contrast to previous gene-based studies, here we used genome-wide approaches for large-scale, unbiased identification and characterization of plant Zf-BEDs. In the current study, we have conducted an evolutionary study of the *Zf-BED* genes in 35 land plants including mosses, bryophytes, gymnosperms, and angiosperms. A total of 750 Zf-BED domain encoding genes were identified, representing 32 plant species. The lower plants such as *Physcomitrella patens* (mosses) and *Selaginella moellendorffii* (lycophyte) did not possess any Zf-BED encoding genes. Gene copy numbers varied from species to species, showing evolutionary diversity among land plants with higher plants showing numbers larger than those in lower plants. All identified genes were classified into 22 major classes, presenting the most comprehensive Zf-BED proteins analysis to date. In these classes, the presence of “Dimer_Tnp_hAT” as a decoy domain was common in all plants, pointing toward their essential roles in plant development^60^. Despite their important functionalities, very few classes of Zf-BED proteins have been reported in the literature to date; for instance, zfBED-NB-ARC(NLR) (class XII; ZfBED-NBS and class XIV; ZfBED-NBS-LRR) domain architecture was reported as conferring disease resistance in plants^19,57,61^. These classes were only found in *Brachypodium distachyon* (*Bradi5g22179.1*), *Oryza sativa* (*OsR498G0409301900.01.T01*), *Medicago truncatula* (*XP_013447741.2*), and *Populus trichocarpa* (*Potri.001G404800, Potri.001G405100, Potri.019G002700, Potri.T013632,* and *Potri.T107066*). However, they were only studied in wheat^57^ and rice^58^, while other identified ZfBED-NLRs may also have great potential in the plant immune system. Other than ZfBED-hAT and ZfBED-NRL, the remaining classes also have important decoy domains like DUF26, WRKY^62,63^, GRAS (transcription factors that regulate plant development)^64,65^, Sina, Glyco_hydro_1, and many others (Table S3). The comparative study of these 22 major classes among land plants demonstrated tandem duplications, deletions, and insertions of the domain during genome evolution. Similar evolutionary mechanisms were also observed in other protein families^66–68^. The molecular functions of 22 classes revealed their role in multiple biological processes and the functional diversity of classes appears to be due to the presence of decoy domains with the Zf-BED domain. For example, the hAT decoy domain has a potential role in plant development^60^. Similarly, the presence of the NLR domain provides an additional link to plant immunity^58^.

The sequence alignment and motif discovery identified important conserved signatures in land plants. We observed some highly conserved residues like W^7^, H^9^, and C^20^-C^23^ in all species. These conserved residues and signatures show their important roles in the structure and function of the Zf-BED domain^9^. Based on these signature variations, the Zf-BED proteins were divided into nine groups^11–15^. The expression profiling of *Zf-BED* genes in *Arabidopsis*, *Z. mays*, *G. arboreum,* and *G. hirsutum* also demonstrated their putative role in different tissues, as well as under biotic and abiotic stresses. Different orthogroups (OG1, OG2, and OG3) of Zf-BED responded differently in various tissues under diverse stresses. The OG1 (*AT4G15020.1, Solyc09g005660.2, Ghir_D05G011710.1, Ghir_D13G002100.1, Ghir_D13G002100.2, Zm00001d000412, Zm00001d004256, Zm00001d010895, Zm00001d016617*) was mainly involved in biotic and abiotic stresses as compared to other two orthogroups (Table S31). Several prior studies presented the responses of *Zf-BED* genes under biotic and abiotic stresses^59^. The overexpression of Zf-BED in rice lines increased drought tolerance^59^. The prediction of *cis*-regulatory elements also provides insight into the response of genes to different stresses. We also observed and identified several additional stress-responsive elements in the regulatory region of *Gossypium* sp. *Zf-BED* genes.

## Conclusions

Zf-BED encoding genes play important roles in plant development and adaptation. We identified a total of 750 Zf-BED encoding genes in 35 land plants and classified them into 22 major classes. The comparative study of land plants highlighted several duplications, deletions, and insertions in the genome during the evolutionary processes. A handful of genes were common in all plant species, while the shared and unique genes were different at different classification levels of kingdom Planta. The addition of the decoy domain with the Zf-BED domain provided an additional structural and functional role to the *Zf-BED* genes. The expression profiling also demonstrated that closely related genes have conserved stress-responsive functions in different plants. This is the first report of a genome-wide identification, characterization, and evolutionary study of Zf-BED encoding genes in land plants, which provides primary data for further functional studies that will help guide research efforts on plant adaptation under biotic and abiotic stresses.

## Supplementary Information

Supplementary Information.

## Data Availability

All data generated or analyzed during this study are included in this published article and its supplementary information files.
