# Supplementary material for: A genome-wide comparative evolutionary analysis of zinc finger-BED transcription factor genes in land plants
Source: Sci Rep. 2022 Jul 19;12:12328. doi: 10.1038/s41598-022-16602-8 (PMC9296551; doi:10.1038/s41598-022-16602-8)
Supplement: Supplementary file 1 — Supplementary Information. [file 41598_2022_16602_MOESM1_ESM.pdf]

Supplementary Information File

Comparative Study of Zf-BED Encoding Genes in Land Plants and Their Roles in Cotton

Athar Hussain[1†], Jinbao Liu[2†], Binoop Mohan[2†], Akif Burhan[3], Zunaira Naseem[3], Raveena Bano[3], Ayesha Ameen[4], Madiha Zaynab[5], M. Shahid Mukhtar[2*] and Karolina M. Pajerowska-Mukhtar[2*]

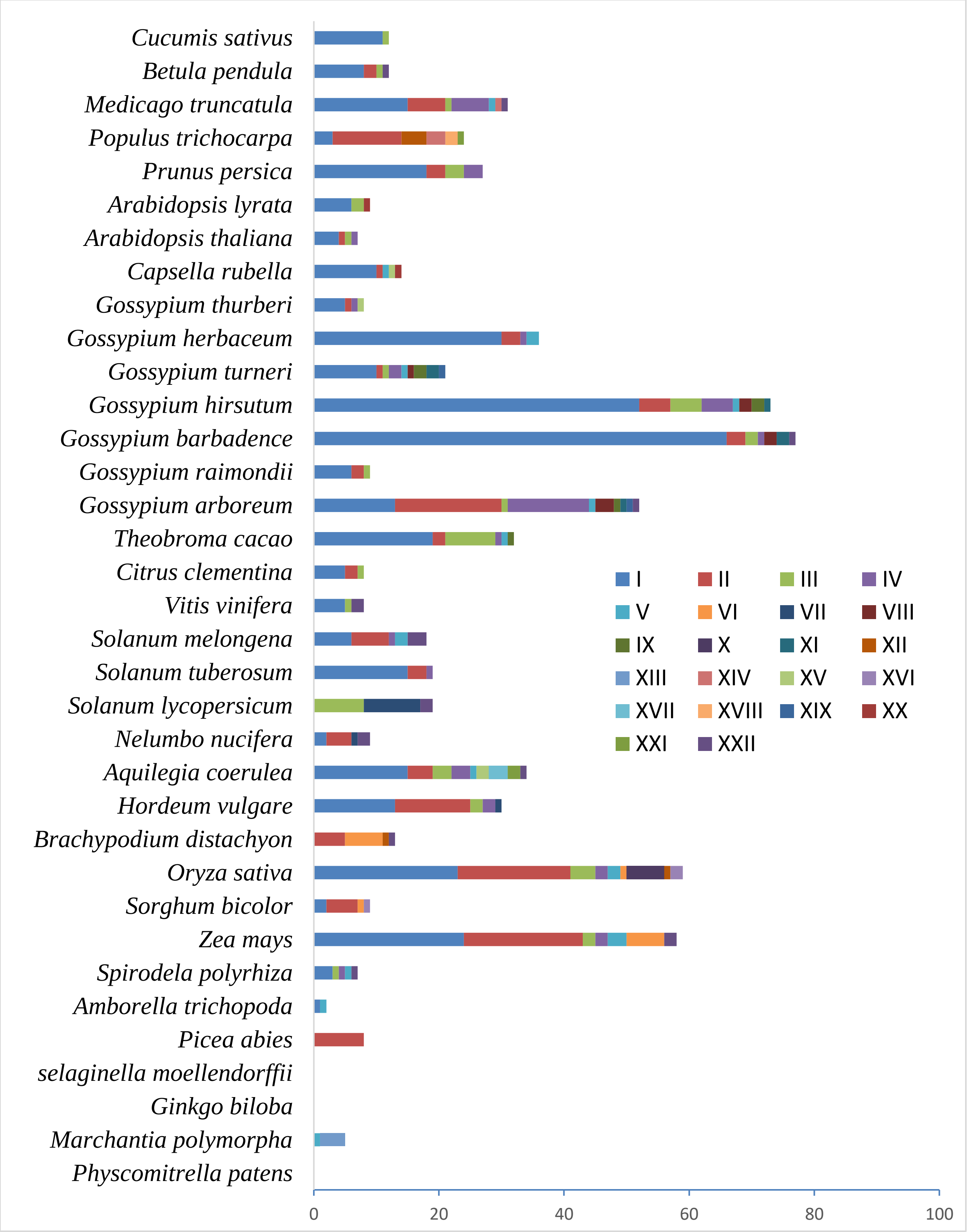

Figure S1: Distribution of Zf-BED genes in land plants and their classes

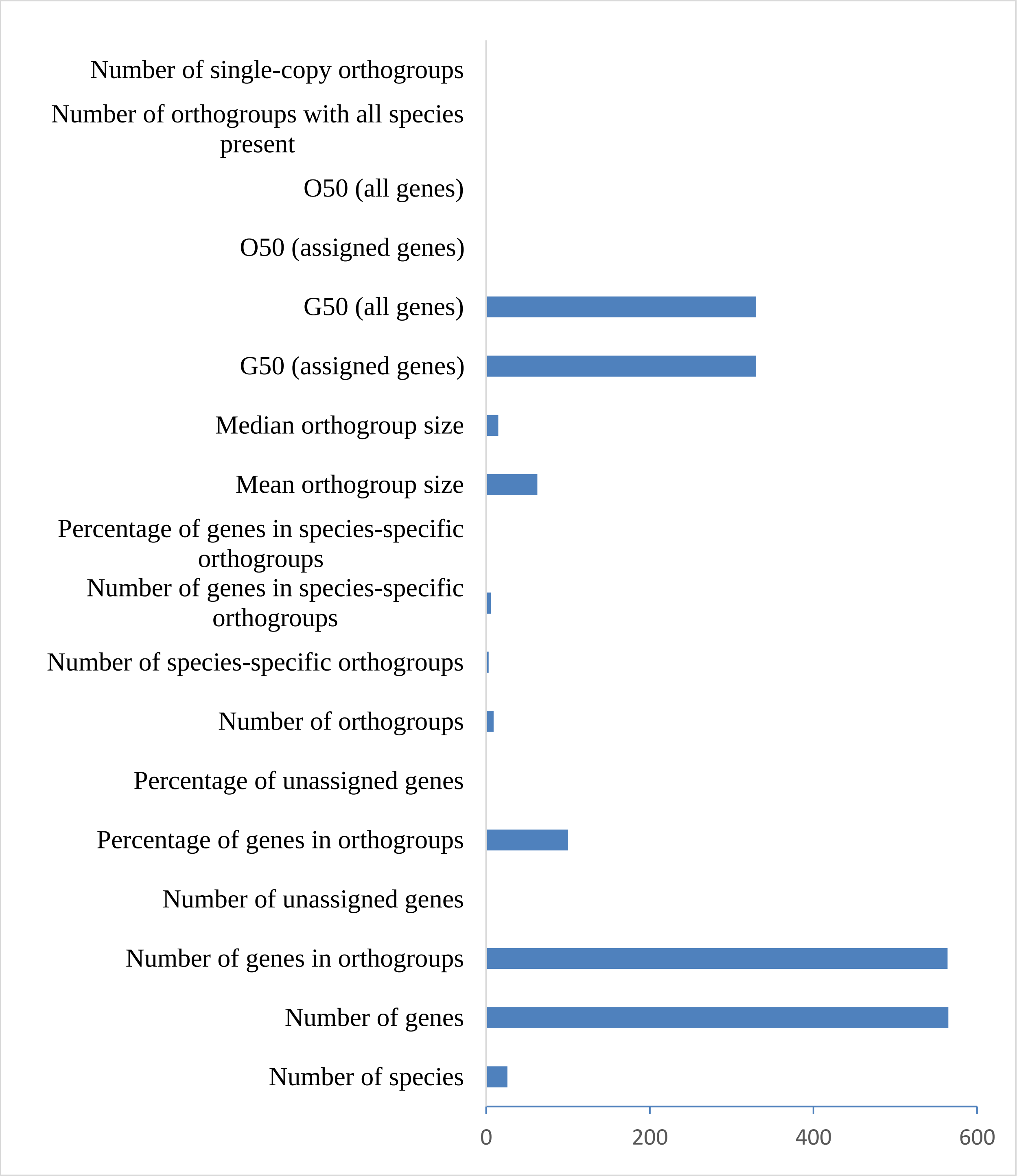

Figure S2: Statistical summary of comparative genomics of Zf-BEDs in land plants

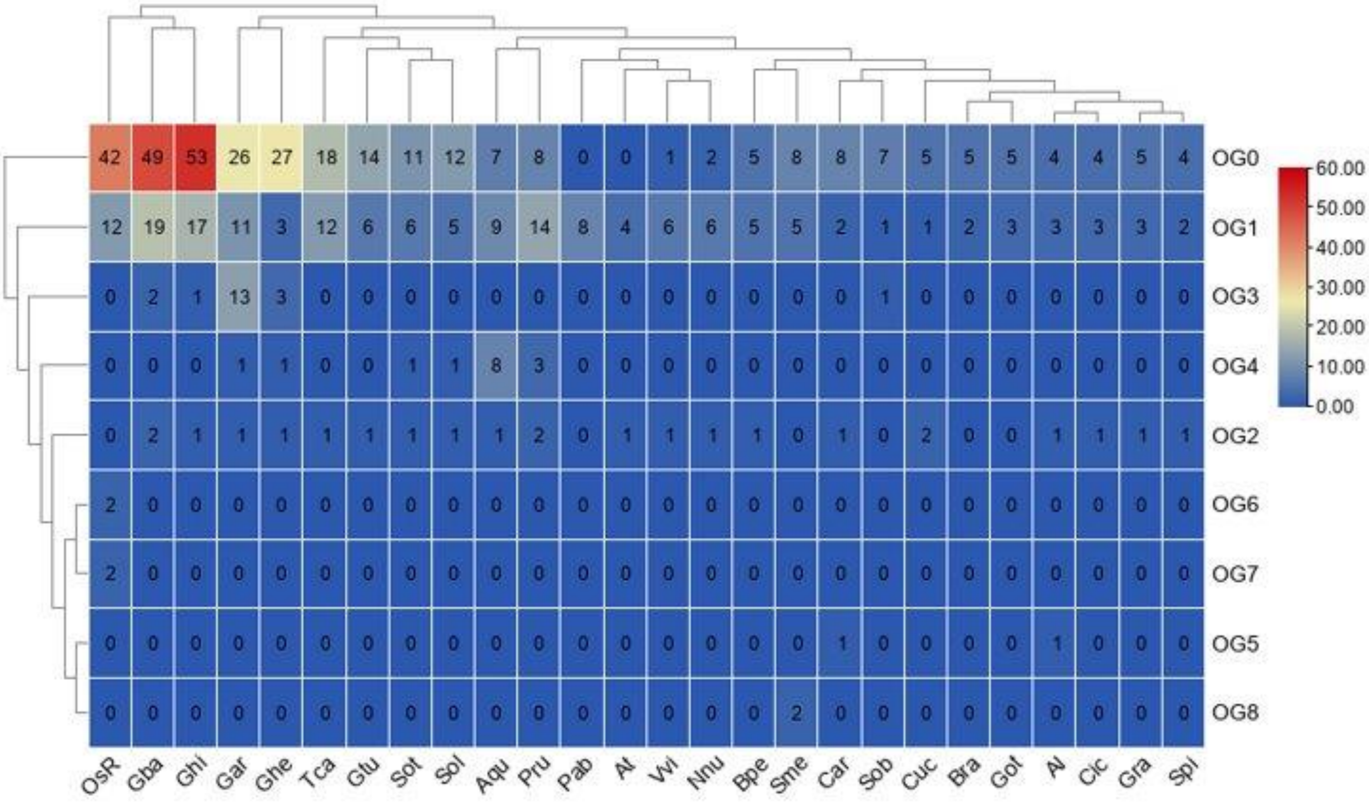

Figure S3: Number of genes in each orthogroup

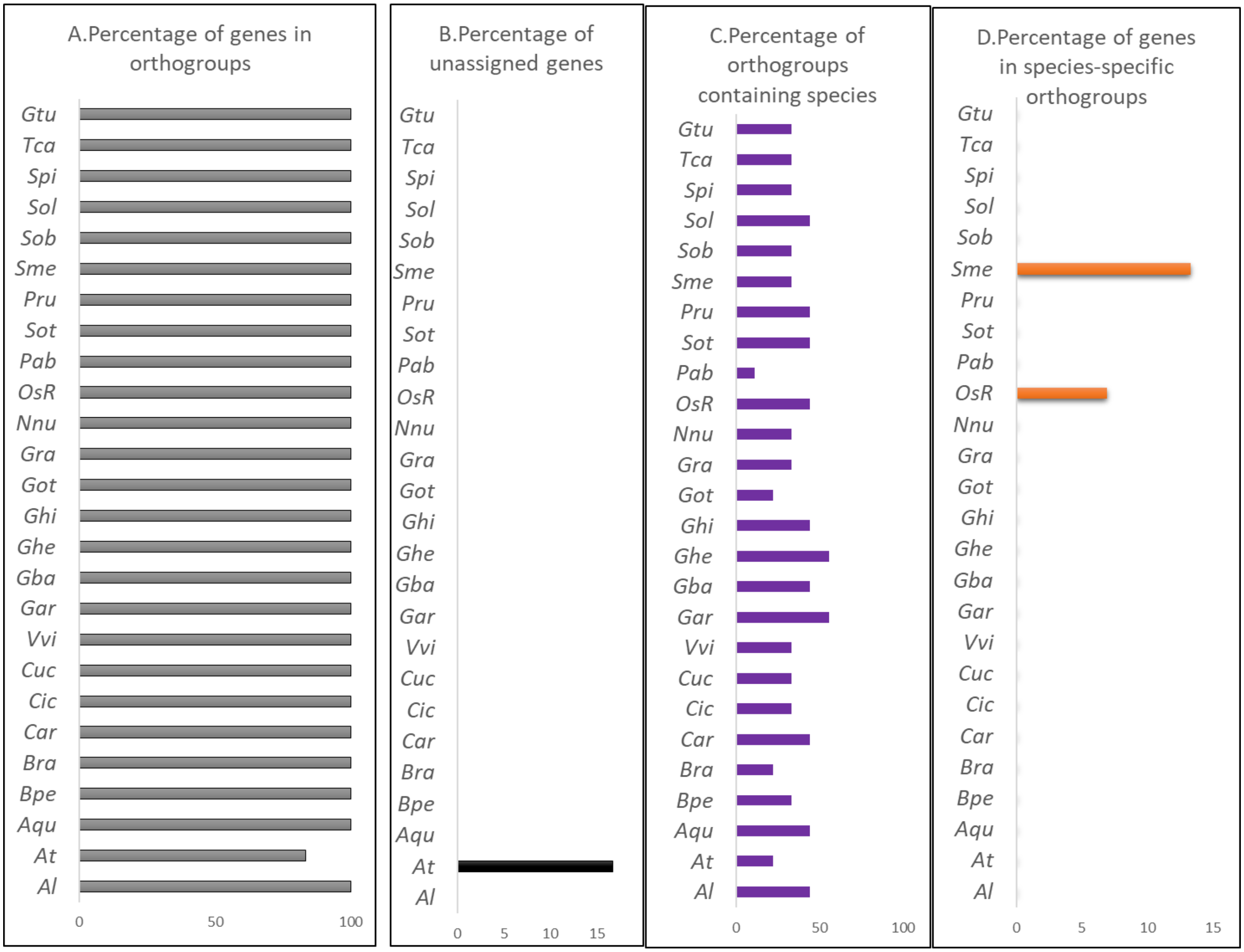

Figure S4: Species-wise orthologs comparative summary of Zf-BEDs in Land plants.

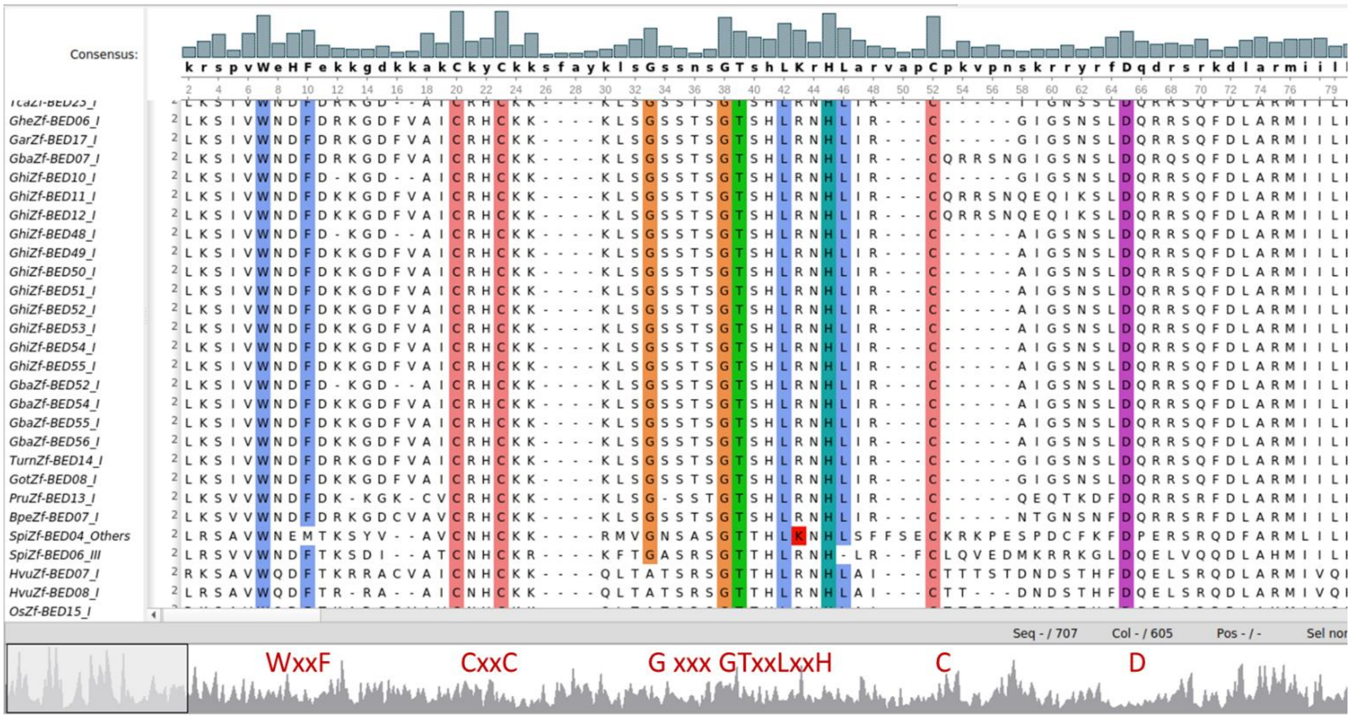

Figure S5: Important conserved residues in Zf-BEDs in land plants: W^{7}, H^{9}, F^{10}, C^{20}-C^{23} (X^{2-4} motif), G^{33}, G^{38}, T^{39}, L^{42}, K^{43}, H^{45} (His motif), L^{46}, C^{52}, D^{65}, P^{83}, F^{92}, E^{124}, W^{146}, C^{247}, D^{259}, L^{318}, W^{346}, P^{372}, K^{409}, W^{414}, L^{436}, P^{438}, W^{536}, W^{537}, P^{545}, A^{551}, L^{555}, S^{560} F^{571} and L^{592} are depicted in the alignment.

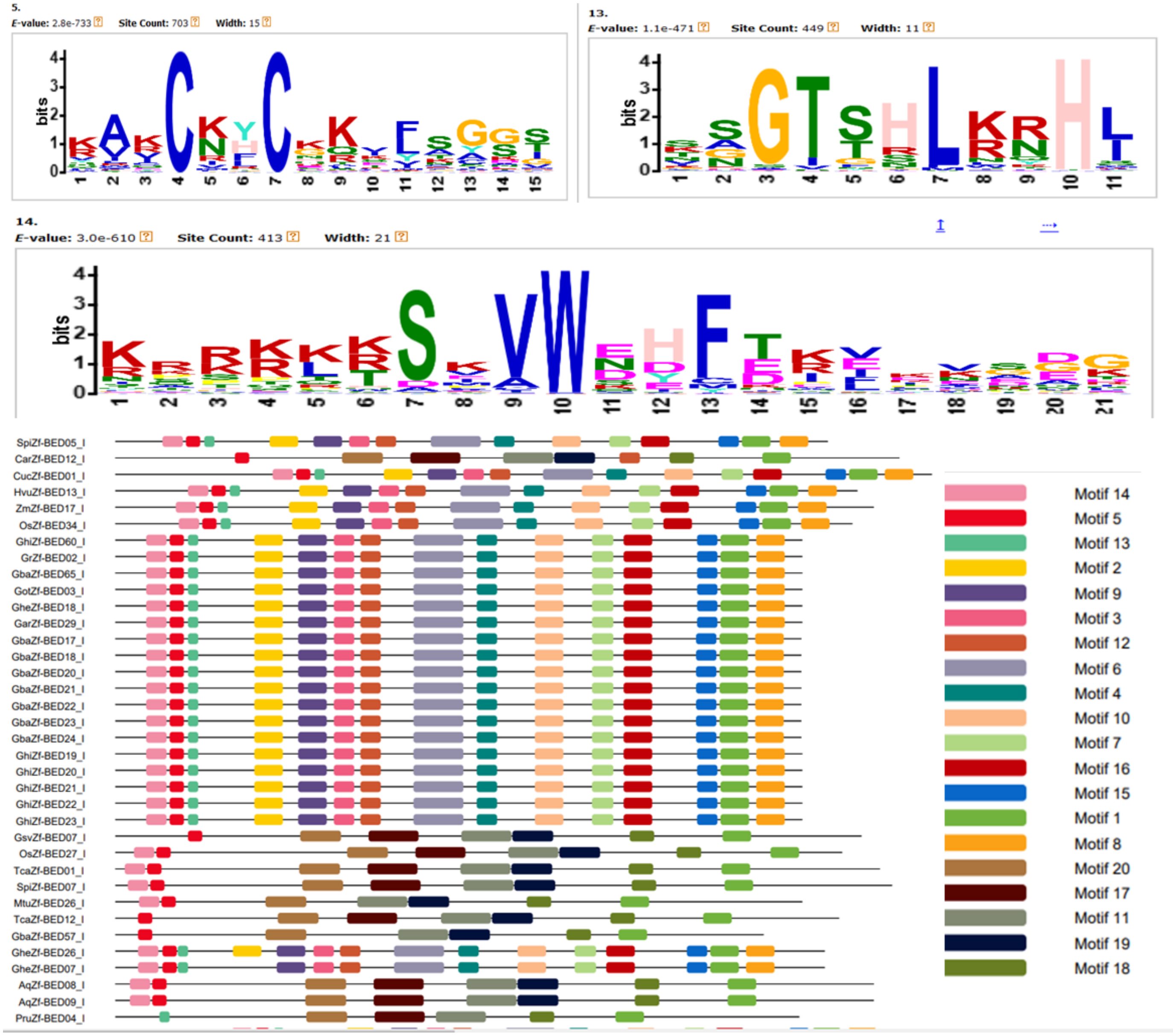

Figure S6: Conserved functional motifs found in Zf-BEDs of land plants. $CX_{2-4}C$; Motif_5, $GT_{XX}L_{XX}H$[LT]; Motif_13 and WX[YH]F; Motif_14. (See additional MEME motif file for detail)

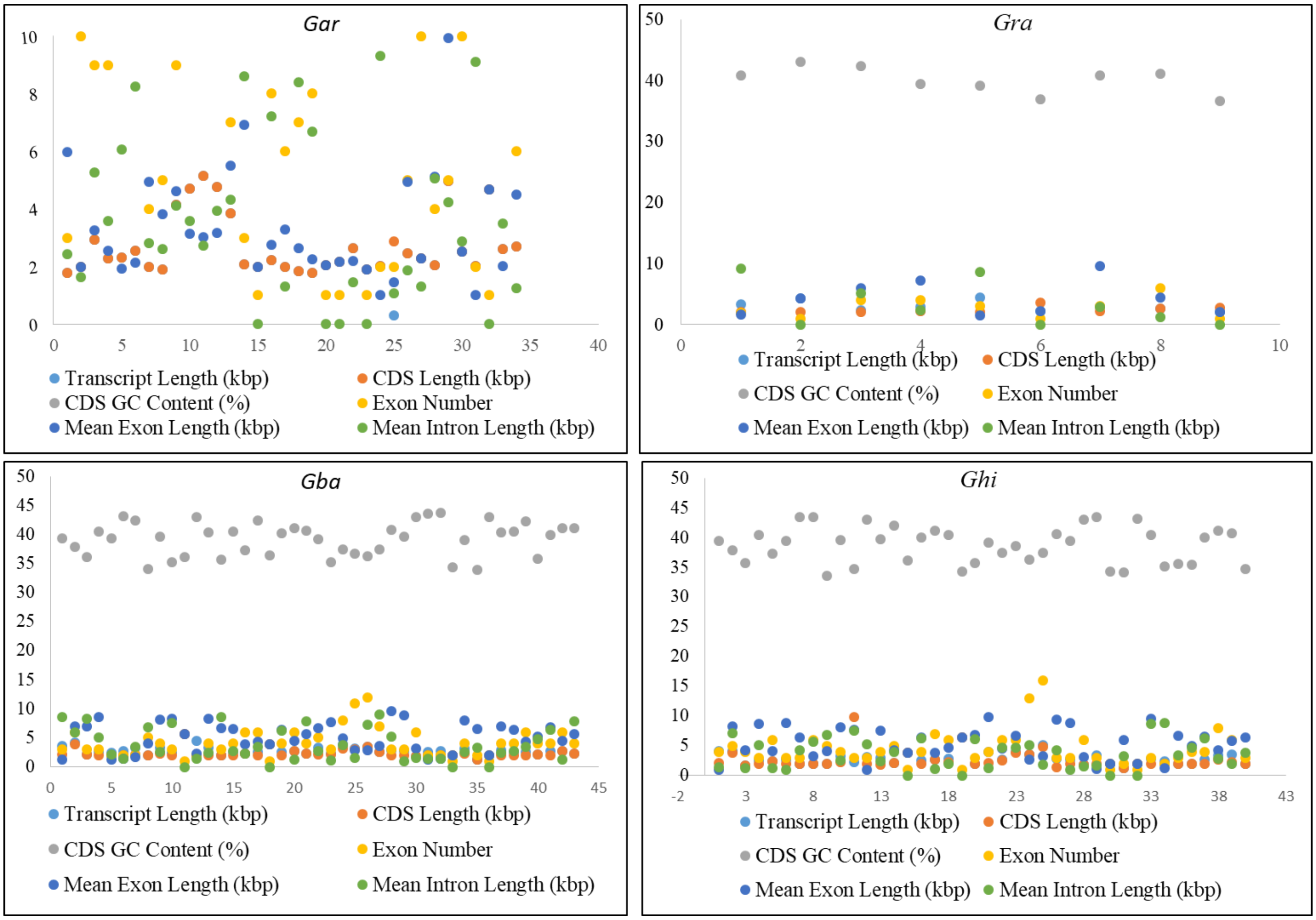

Figure S7: Gene features *of Zf-BED* including transcript length, CDS length, Exon number and GC content % in *Gossypium sp.*

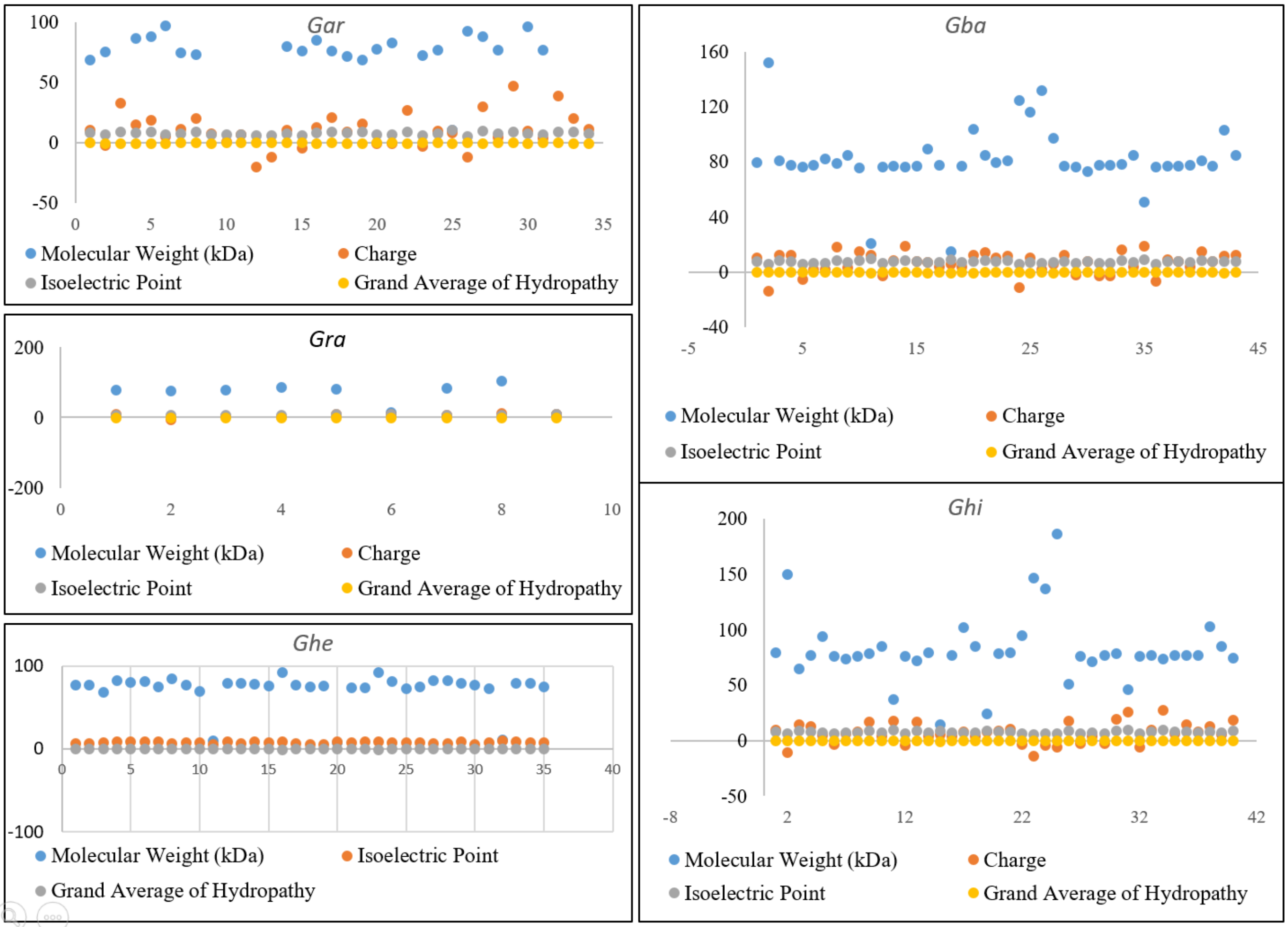

Figure S8: Physio-chemistry of cotton Zf-BED Proteins including molecular weight, isoelectric point, charge and GAVHY.

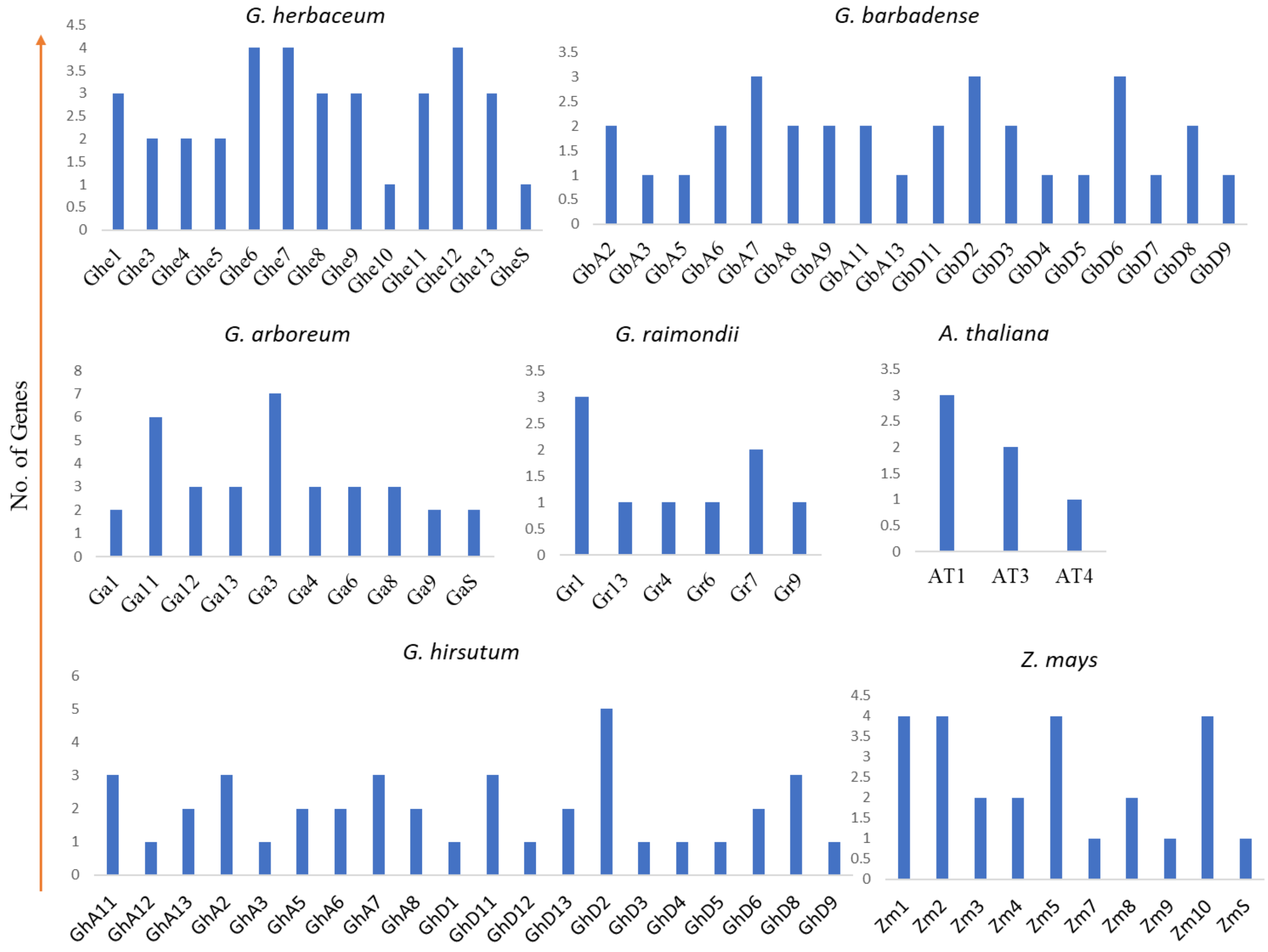

Figure S9: Chromosomal density of Zf-BED genes on *Ghe, Gba, Ga, Gr, Gh, At* and *Zm*.

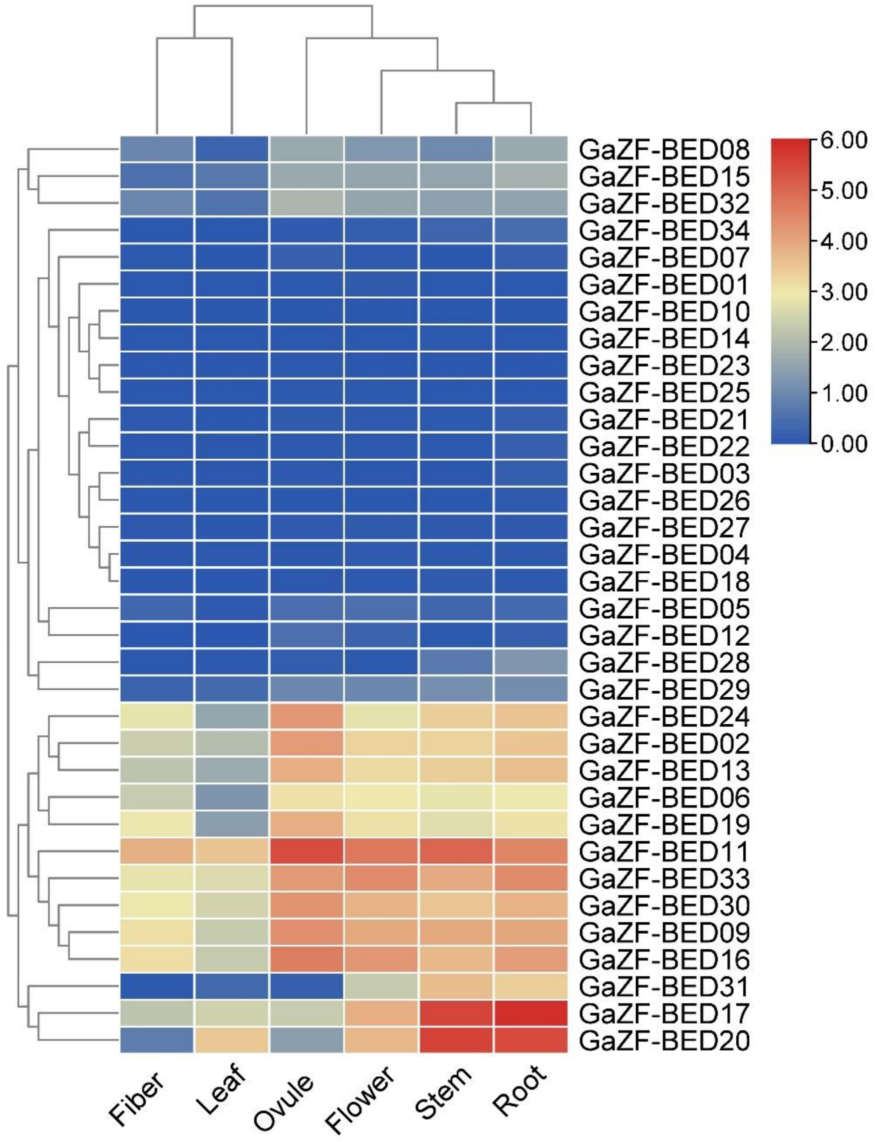

Figure S10: Tissue specific expression profiling of Zf-BED encoding genes in *G. arboreum* with log2 base value.

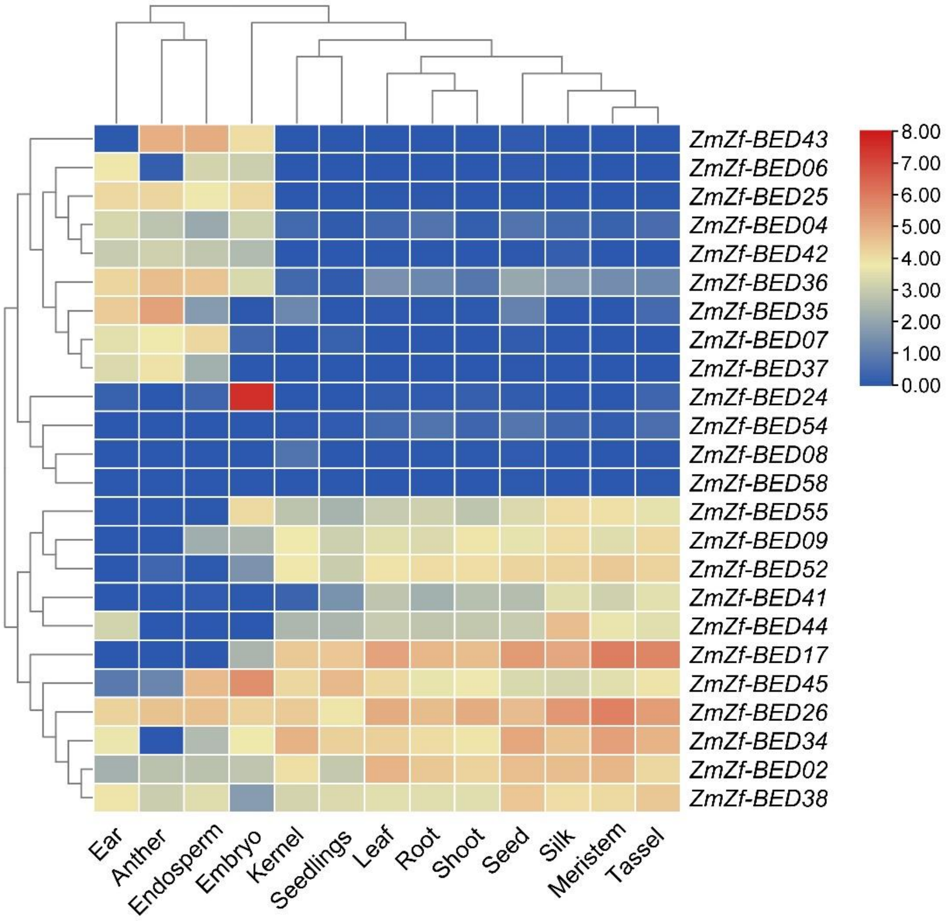

Figure S11: Tissue specific expression profiling of ZmZf-BED encoding genes in *Z. mays* with log2 base value

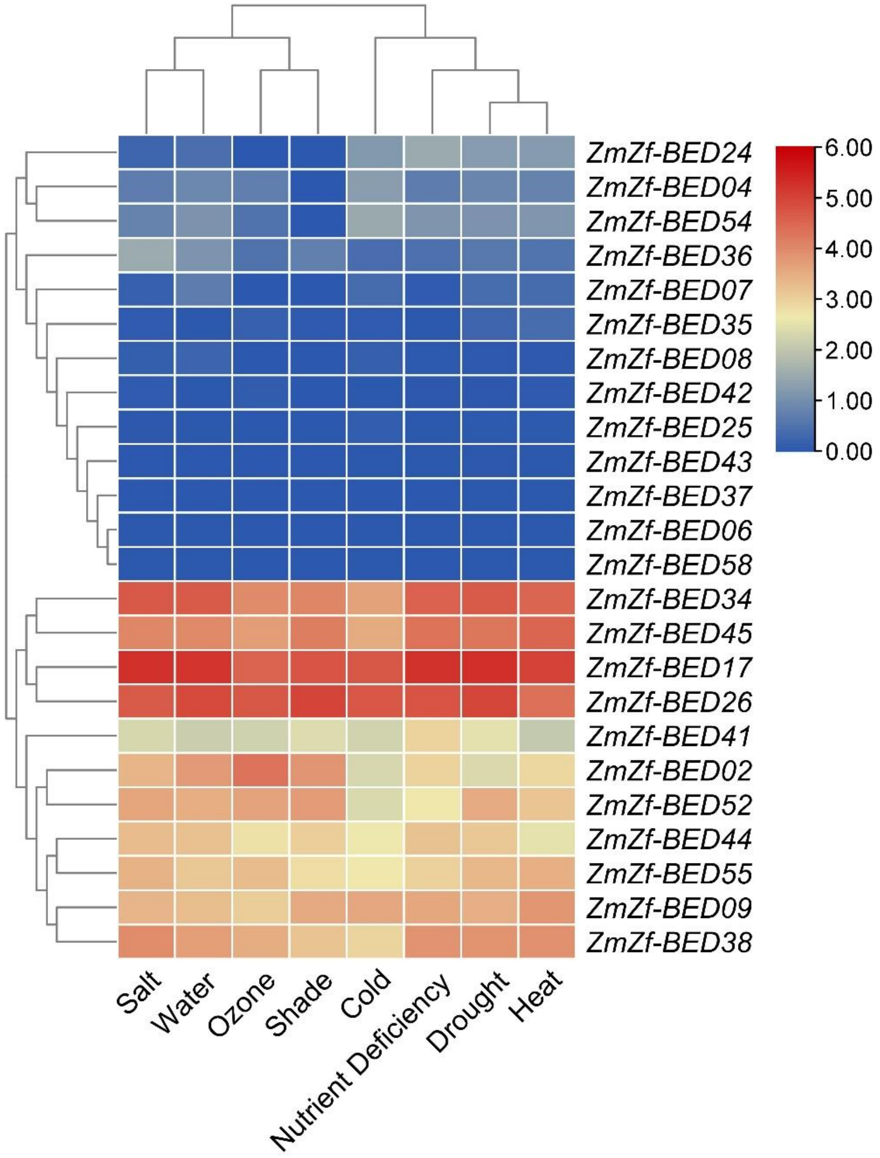

Figure S12: Abiotic stress-specific expression profiling of ZmZf-BED encoding genes in *Z. mays* with log2 base values.

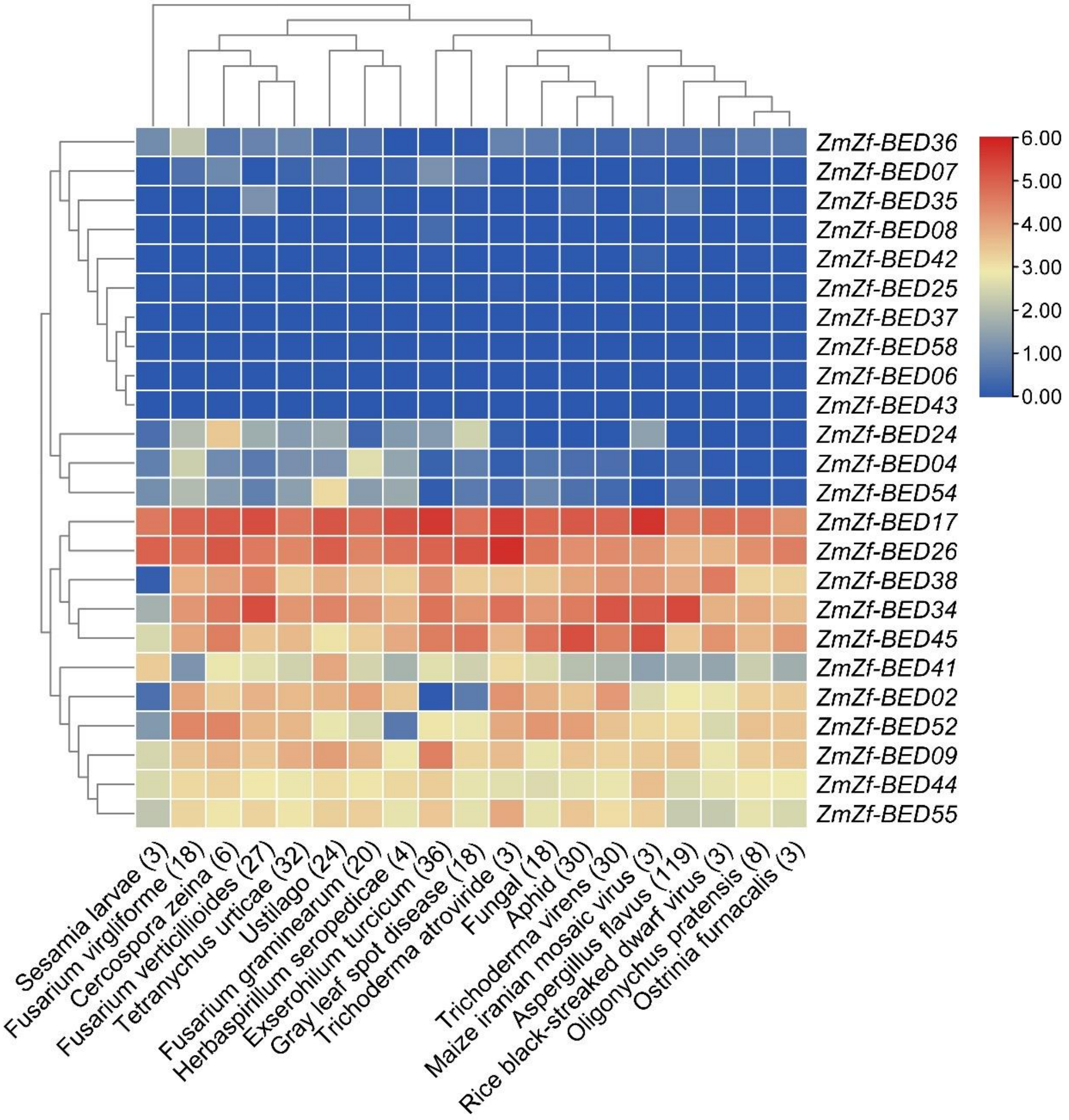

Figure 13: Biotic stress-specific expression profiling of ZmZf-BED encoding genes in *Z. mays* with log2 base values.

Table S1 List of selected plants and thier classes

Name of Plant Species	Family		Symbol	Total transcripts	Total locus	Genome_assembily Version
Physcomitrella patens	Mosses	Mosses	Ppa	0	0	Phypa V3
Marchantia polymorpha	bryophyte	bryophyte	Mpoly	5	2	JGI v3.1
Ginkgo biloba	Gynmosperm	Gynmosperm	Gbi	0	0	
selaginella moellendorffii	lycophyte	lycophyte	Smo	0	0	
Picea abies	Fern	Fern	Pab	8	8	ConGenIE v1.0
Amborella trichopoda	Angioseprms	M	Atr	2	2	JGI v1.0
Spirodela polyrhiza	Angioseprms	M	Spo	7	7	JGI v2.0
Zea mays	Angioseprms	m	Zma	58	33	AGP v4.0
Sorghum bicolor	Angioseprms	m	Sbi	9	6	JGI v3.1
Oryza sativa	Angioseprms	m	Osa	59	42	IRGSP-1.0
Brachypodium distachyon	Angioseprms	m	Bdi	13	7	JGI-PGF
Hordeum vulgare	Angioseprms	M	Hvu	30	18	MorexV3_pseudomolecules_assembly
Aquilegia coerulea	Angioseprms	D	Aco	34	24	JGI-PGF
Nelumbo nucifera	Angioseprms	D	Nnu	9	9	Chinese Lotus 1.1
Solanum lycopersicum	Angioseprms	D	Sly	19	19	Sol Genomics itag2.4
Solanum tuberosum	Angioseprms	D	Stu	19	19	JGI v4.03
Solanum melongena	Angioseprms	D	Sme	18	18	SME_r2.5.1
Vitis vinifera	Angioseprms	D	Vvi	8	8	Vitis vinifera_JGI 12x
Citrus clementina	Angioseprms	D	Cic	8	8	JGI v1.0
Theobroma cacao	Angioseprms	D	Tca	32	32	Criollo_cocoa_genome_V2
Gossypium arboreum	Angioseprms	D	Gar	52	52	Gossypium arboreum WHU-A2_V1
Gossypium raimondii	Angioseprms	D	Gra	9	9	JGI-AD4_v1.1
Gossypium barbadence	Angioseprms	D	Gba	77	44	HAU-AD2_v2.0
Gossypium hirsutum	Angioseprms	D	Ghi	73	41	HAU-AD1_v1
Gossypium turneri	Angioseprms	D	Gtu	21	21	D6_ISU_v1
Gossypium herbaceum	Angioseprms	D	Ghe	36	36	CRI-G2_v1.1
Gossypium thurberi	Angioseprms	D	Got	8	8	ISU-D1_v1
Capsella rubella	Angioseprms	D	Car	14	14	JGI v1.0
Arabidopsis thaliana	Angioseprms	D	At	7	6	Araport11
Arabidopsis lyrata	Angioseprms	D	Al	9	8	JGI v2.1
Prunus persica	Angioseprms	D	Pru	27	14	JGI v2.1
Populus trichocarpa	Angioseprms	D	Ptr	24	19	JGI v3.1
Medicago truncatula	Angioseprms	D	Mtr	31	31	MtrunA17r5.0-ANR,
Betula pendula	Angioseprms	D	Bpe	12	12	Bpev01
Cucumis sativus	Angioseprms	D	Cuc	12	7	JGI v1.0

Table S2 List of identified genes, thier domains architectures and classes

Gene_IDs	Gene_Name_Classes	Peptide_Sequence	Domain Architecture	Classes
AL1G27390.t1	AlZf-BED01_I	MDTNPSELVDVDVVALEMDSDTQQPTKRRKKKRSEVWNHFTTETVSPDTKKARCMHCNKSFAYMTANKCSGTSHLKRHIDFGICAKNGEINKLSQIKEETTSSPPKKSQISSPETKDVKPTACLPKKRQIPSPNVPLDQDRCNLEMAKMIIMHDYPLHMVEHPGFVGFLQALRPQFTVASFDTIHCDCVAMFLSQKQKTLDFIAEIPGGVNLTVDLWSSKQSVGYAFVAGHFVDKDWNLTHRLLNVAVVASPDSDFALNQPVTACLSDWHLEGKISSIAVSQSLVNKTSIDNLRGFLSFRNQHVLNGQLLMGKCYARLLSSMAQDALTAEQLQEPIKKVRDSIKYVQTSEACGDRFDELKRRFPTPLPYKDLLIDNQTRWDTSYNMLLAACEHRQVFSCLETCHPEYKISISPEEWIKIESLCSCLKVIFEAGKALTGPNQLTTNDLYHEMTKLQLELSPTAMGEDPDVRNLAKSLREKFDQYWRGCFLVLAVAVVMDPRFKMHLIKFTFNNSYGEDAERWTKSVDDAVHDLYLNYSEQNLLDAYVDHGFSEIEVAQESHFHQDIAEYANTGNGQSGDETSPQVEETTESHQQERQLKAEKQNVVGLWEGVSEESQLTEEDQQHGDDSQAHAMAEEDHPSFPVDVLLEEGSTLITIGESLSDFEMYMSDMKTELDQYLEENLIPRSEDFNVLSWWRVNSTNYPTLSKMAADLLSVPFSTVSPDSVFDTEVKQMDNYRTSLPGETLEALLCTKDWLKNGTL	zf-BED--DUF-domain--Dimer_Tnp_hAT--	I
AL1G31000.t1	AlZf-BED02_I	MEWNVNNAFKTYKELEPKAMMDMTLVPHSDPIDIGLGSSDKSNSVPPKRKKTMTSVYLKYFETAPDSKTRKCKFCGQSYSIATATGNLGRHLTNRHPGYDNSTDVVTSSSVPQTPPVVVKPSQSQSKAPQLDYDHLNWLVLKWLALSSLPPSTVDETWLGNSFKFLNPSIQLWPAKKYKAILHEVFRSMRGDVKTSMEHIQSKVSVTLSFWNSYENIFYMSVTGQWIDENWSSHRLLLDICRIPYPSGGSEIHNSLLKVLKIYAIEDRASKSLDSVIRKNEDVLENRMMLSSVEKNAVTIVHNYLDLDSFHRTTNDMCTNKDLTVGLALLFMDNISEMITTCQKSCHNPDWLRSCAESMAQKARSYNTQVCNVFTYITAILDPRIKTEYIPETINLESYIDEARNHFIRNYSSTHFTSSMSSGYRPQEIDEGGAGGGGNISFAEEIARRKRRGSMSNNVVDELTQYLSESIAPMQTDVLDWWKVNSGRYPRLSNMARDFLAVQATSAAPEEIFCGKGEEIDKQRYCMPHDSTQSVICIRSWIEAGMKLKYKCSEIDYERLMELAANVAADNSAGGLEKSQHR	zf-BED--DUF-domain--Dimer_Tnp_hAT--	I
AL2G29190.t1	AlZf-BED03_I	MKRRQKKLTINNELDLSDAVIVKSGRLKSVVWNDFDRVRKGETYIAICRHCKKRLSGSSASGTSHLRNHLIRCRRRSNGSNNGVAQYFVRGKKKELANERRKDEEVLSVVNVRYEHEKEELDDVNVVSTGLDQRRCRFDLARMIILHGYPLSMVEDVGFRMFIGNLQPLFELVAFERVESDCMEIYAKEKHKIFEALDKLPGKISISVDVWSGSDDFDEFLCLVAHYIDETWELKKRVLNFFMVDPSHTGEMLAEVIMTCLMEWDIDRKLFSMASSHSPPFGENVANKIRDRLSQNKFLYCYGQLFDVSCGVNVINQMAQDSLQTCCDTINMIRESIRYVKSSESIQDRFNQWIVETEAESERNLCIDDPTRWDSTCTMLEIALEQKSAFSLMNEHDPDSVLCPSDLEWERLGTIVEFLKVFVEVINAFTKSSCLPANMYFPEVCDVHLRLIEWNKNPDDFISSLAVNMRKKFDDFWEKNYLVLAIATILDPRFKMKLVEYYYPLFYGTSASELIEDISECIKALYDEHSVGSLLASSDQALGWQNHHHRSNGVAHGKEPDDRLIEFDRYINNTTTTPGQDSKSDLEKYLEEPLFPRNSDFDILNWWKVHTPKYPILSMMARNVLAVPMSNVSSSEDAFETCQRRQVSETWRSLRPSTVQALMCAQDWIQSELESS	zf-BED--DUF-domain--Dimer_Tnp_hAT--	I
AL2G41520.t1	AlZf-BED04_I	MDNNSLMLIDNNGSFEIDDQSHASLTLFTPTRPTEDLPNADTDDGLLPDPATLPTKRRRKKSMVWEHFTIETSSPGSTKACCKHCRKSFAYITGQKLAGTSHLKRHIQLGICPMSRDTSTHAQILGSKDPTTAPPKKRHRSSASYIPLDQDRCYNQMAKMIIMHDYPLHMVEHSGFTGFVQALRPQFTMASFNTIHADCVNMYLSEKHKLSNFISEIPGRVNLTVDLWTSNQSVGYAFVTGHFIDRDWNLSHRLLNVAVVPSPDSDFALNQPIAACLSDWNLERRLSSLTVGQSLVNKTSIENLRCCLSARNQNVLNGQLLLGNCYARLLSSMAQDLLGAEDFKTPIKKVRDSVKYVKTKDSCEERFDELKRQLQTPFTKDLLIDNQTKWDTSYNMLLAACEHKEVFSCLGNCDLDYKMSTSPEEWRKIEILCSCLKILFDAANVLTGSTRLTANDLYHEMTKLQLELSHSAMSEDPDVRNLANPLKEKFDEYWRECFLLLAVAVVMDPRFKMKLIEFSFSKAYGEDADKWIRSVDDAVHELYHDYAEQSHSLLDAYVGHGNDGFSETDMSQVHFHHEMPHEYHNSNGLSHDQIFEQPEDESLLNEKSLEGLSGEGQQTGTVSVGGNTTQGVEQKGHLGDAGTQKNHIVEGLSEESKPMEGLTQESLLTEGNLQSNGELTPAQESRLTDETSHENQAMEETHANQSAEEMSHETQLIEEFPCESQPSEDVPLQSQPVEEIPQESQLVEDTDMNQDSQSGEGYVPETQPMEEIVEDTQPVEEVVQEETSQMSRPVEDIPPESHPIEEIRLENQQVDDKDITHDIQPVEEMLEDTQPVEEVDQEAQQVEKHDDIQSVEELGHEIQPEAELLEDTQPVEEVAQEAELLEENHDDIQPVEEAGHGTQPVEEVAHEAQPVEQIPEHSQNPQNADGILSQEYSREGKSVQEEQQSDEGIASDQHPQSHAMPQEEEAQHDSQSHATPQEEATFTISQEGHQVDVLLQEGHHLEASSQEFPLITIGDGFSDFELYISEVGSHQQMKSELDQYLEESLIPRSQDFEVLGWWSLNRTKYPTLSKMAADVLSLPFCTVSPDSVFDTEVKKMDNYRSSLRHVTLEALFCAKDWFKHSSNSENNLKRES	zf-BED--DUF-domain--Dimer_Tnp_hAT--	I
AL3G27110.t1	AlZf-BED05_I	MMDESNEIILQKSKRLTSVVWNYFERVRKADVCYAVCIQCNKKLSGSSNSGTTHLRNHLMRCLKRTNHDMSQLLTPKRRKKENPVTVAATINFDEGQTKEEYLRPKFDQDQRRDEVVLSRGSGGRFSQERSQVDLARMIILHGYPLAMVDHVGFKVFARNLQPLFEAVPNSTIEDSCMEIYIREKQRVQHTLNHLYGKINLSVEMWSSRDNANYVCLASHYIDEEWRLHRNVLNFITLDPSHTEDMLSEVIIRCLIEWSLESKLFAVTFDSCSVNEEVVLRIKDHMSQSSQILINGQLFELKSAAHLLNSLVEVCLEAMRDVIQKIRGSVRYVKSSQSTQVRFNEIAHLAGINSQKILVLDSLVNSNSTFVMLETALEYKGAFCHLRDHDHRFDSSLTDEEWEWTRYVTGYLKLVFDIASDFSGNKCPTANVYFAEMCDIHIQLIEWCKNQDNFLSSLAANMKAKFDEYWNKCSLVLAIAAILDPRFKMKLVEYYYSKIYGSTALDRIKEVSHGVKELLDAYSMCSAIVGEDSSFSGSSLGHTRDRLKGFDKFLHETSQNLNTTSDLDKYLSEPIFPRSGEFNILNYWKVHTPRYPILSLMARDILGTPMSICAPDSTFNSGTPMIAESQSSLNPDIRQALFCAHDWLSTETEESLPLSRQYTQTL	zf-BED--DUF-domain--Dimer_Tnp_hAT--	I
AL3G30530.t1	AlZf-BED06_III	MAPPGSIGVVDPGWEHGVAQDERKKKVKCNYCGKIVSGGIYRLKQHLARVSGEVTYCDKSPEEVCLRMKENLVRSTRKLRQSEDNSGQSCSSFHQSNNDDEAEEDERPCWSRRSKGKLGLSDGSLLRSSGYIDPGWEHGVAQDERKKKVKCNYCNKIVSGGINRFKQHLARIPGEVAPCKNAPEEVYVKIKENMKWHRAGKRQNRPDDEMGALTFRTVSQDPEQEEDGADHDFYPISQDRLMLGNGRFSKDKRKSLDSMNMRSVSEANPKRVRMIPFQSPSSSKQRRLYSSCSNRVVSRKDVTSSISKFFHHVGVPTEAANSLYFQKMIELIGMYGEGFVVPSSHLFSGRLLQDEISTIKSYLREYRSSWLVTGCSIMADTWTNTEGKRMISFLVSCPRGVFFHSSIDAADIVEDALSLFKCLDKLVDDIGEENVVQVITQNTAICRSAGKLLEEKRKNMYWTPCAMHCTELVLEDISKLEFVSECLEKAQRITRFIYNQTWLLNLMKNEFTQGLDLLRPAAMRHASGFTTLQSLMDHKASLRGLFQSDGWILSQTAAKSEEGREVEKMVSSAAFWKKVQYVLKSVDPVMQVIHMINDGGDRLSLPYAYGYMCCAKMAIKSINGDDARKYGPFWRVIEYRWNPLFHHPLYVAAYFFNPAYKYRPDFMAQSEVVRGVNECIVRLEPDNTRRITALMQIPEYTSAKADFGTDIAIGTRTELDPAAWWQQHGISCLELQRVAVRILSHTCSSVGCEPKWSMYDQVNSQCQSRFGKKSMKDLTYVHYNLRLREKQLKRRLQHDDGPPPSLNYALLDQLLPEWLVATGKDEEETLQGEERAENEDHEDDEDEEENCYNMGSGNVDGEGEDNLDLYDDDLSDDD	zf-BED--zf-BED--DUF-domain--Dimer_Tnp_hAT--	III
AL3G30530.t2	AlZf-BED07_III	MAPPGSIGVVDPGWEHGVAQDERKKKVKCNYCGKIVSGGIYRLKQHLARVSGEVTYCDKSPEEVCLRMKENLVRSTRKLRQSEDNSGQSCSSFHQSNNDDEAEEDERPCWSRRSKGKLGLSDGSLLRSSGYIDPGWEHGVAQDERKKKVKCNYCNKIVSGGINRFKQHLARIPGEVAPCKNAPEEVYVKIKENMKWHRAGKRQNRPDDEMGALTFRTVSQDPEQEEDGADHDFYPISQDRLMLGNGRFSKDKRKSLDSMNMRSVSEANPKRVRMIPFQSPSSSKQRRLYSSCSNRVVSRKDVTSSISKFFHHVGVPTEAANSLYFQKMIELIGMYGEGFVVPSSHLFSGRLLQDEISTIKSYLREYRSSWLVTGCSIMADTWTNTEGKRMISFLVSCPRGVFFHSSIDAADIVEDALSLFKCLDKLVDDIGEENVVQVITQNTAICRSAGKLLEEKRKNMYWTPCAMHCTELVLEDISKLEFVSECLEKAQRITRFIYNQTWLLNLMKNEFTQGLDLLRPAAMRHASGFTTLQSLMDHKASLRGLFQSDGWILSQTAAKSEEGREVEKMVSSAAFWKKVQYVLKSVDPVMQVIHMINDGGDRLSLPYAYGYMCCAKMAIKSINGDDARKYGPFWRVIEYRWNPLFHHPLYVAAYFFNPAYKYRPDFMASEVVRGVNECIVRLEPDNTRRITALMQIPEYTSAKADFGTDIAIGTRTELDPAAWWQQHGISCLELQRVAVRILSHTCSSVGCEPKWSMYDQVNSQCQSRFGKKSMKDLTYVHYNLRLREKQLKRRLQHDDGPPPSLNYALLDQLLPEWLVATGKDEEETLQGEERAENEDHEDDEDEEENCYNMGSGNVDGEGEDNLDLYDDDLSDDD	zf-BED--zf-BED--DUF-domain--Dimer_Tnp_hAT--	III
AL7G40810.t1	AlZf-BED08_I	MDSELEPVALTPQKQDNAWKHCEIYKYGDRLQMRCLYCRKMFKGGGITRVKEHLAGKKGQGTICDQVPEDVRLFLQQCIDGTVRRQRKRHKSSSEPLSVAALPPIEGDMMVVQPDVNDGFKSPGSSDVVVQNESVSGGRTKQRTYRSRKNAFENGSASNNIDLIGRDMDNLIPVAISSVKNIVHPSFRDRESTIHMAIGRFLFGIGADFDAVNSVNFQPMIDAIASGGFGVSAPTHDDLRGWILKNCVEEMAKEVDECKAMWKRTGCSILVEELNSDNGLKVLNFLVYCPEKVVFLKSVDASEILSSADTLFELLSELVEEIGSTNVVQVITKCEDHYVDAGKKLMLVYPSLYWVPCAAHCIDQMLEEFGKLGWISETIEQARAITRFIYNRSGVLNLMWKFTSGNDILLPAFSSSATNFATLGRIAELKSNLQAMVTSAEWNECSYSEEQSGLVMNAISDEAFWKAVALVNHLTSPLLRALRIVCSEKRPAMGYVYAALYRAKDAIKTHLVNKEDYIIYWKIIDRWWEQQQHIPLLAAGFFLNPKFFYNANEEMRSELILSVL	zf-BED--DUF-domain--Dimer_Tnp_hAT--	I

		DCVERLVPDDKIQDKIIKELTSYKTAGGVFGRNLAIRARDTMLPAEWWSTYGESCLNLSRFAIRILSQTCSSSVSCRRNQIPVEQIFQSKNSIEQKRLSDLVFVQYNMRLRQLGPESGDDTLDPLSLNRIDVLKDWVSGDQACVEGNGSADWKSLESIHRTQVVPIIDDTEDLGSGFDDIEIFKGEKEVRDEGYYTNTSEKLFT		
AL7G53090.t1	AlZf-BED09_XX	MDLESVTLNVKHECIEMNEEDDDGETEGLVRSETQQTQTVFESNHCVKDDVWKEFVPIGKGEDGKERCRCIHCGKDLVTPTFTSNLWRHLRSCTKKTETKRGGNGCGQDRLNKDQNKCNSVDVVGASVYECSSDREGVSNNTLLKKRQRSSILEGKTRSGMLLGLDVLDCPICFEALTIPIFQCDNGHLACSSCCHKLSNKCPTCASPVGHNRCRAMESVLESVFVTCRNAKFGCAKNVSYGKVSIHEKECTFSQCSCPALDCNYTGSYNNIYSHFVDNHRNKSTSISFVCGGSVDVQMNISTGNILVLQESKKGLLFALQCFYKPHGLYVTVRCIAPSTPEVGKLAYCLYYSMDGHTLTYKSPEVKKVLEVSSETPQDNFMFVPHSLLRGEFLEMKIAIGLKVHAGS	zf-BED--Sina--	XX
Aquca_002_00025.1	AqZf-BED01_I	MDNGRVDGEVVPPLQFVKVELLPSPDAIRLPLPAESESLHPSNIINGSEATSSRKRKMLKSDAWNHFDIIDVKKDDGTLEPKAQCKYCGKHLSCQYASGTSHLNRHYDRCLSKITPNTRLGQVTCTPPEELVVDFTYDHTRACRELARLIVLFELSFSMGEDPHFQAMTQSFNSQAQPVSRFTIRDIVIESFEEERSKLHSLLQSIPGKICLTSAFWKTEEGDGYLCITAHFVDRDWKLKSRIISFPRVDYPHTEFAVYEAIYNSILGWNLNGKVFTLTLDDRNSIDVTRRLKESLFTNDNSLPKQLFHVSCSTYIVNIIVQGGLNEIHHSLENIRTSIRHIRTSKVNLRKFRELCEISGTTFRNLASDICIPYLWNTTYLMLDAAIPYQRTFELFFYDSILEFVPTTTDWKNAMVARDLLKVFYEASKSFSGLNYVSSSEFCHQLSNICLVLSKYENDPSCEGIVGSMKSKLKEYWTEISPVVELAACMDPRYKVTFLEVCLEVIFGDEETHGFGGMVEKIKKTLKQVFNEYTQKKGGTLLKEPFNSNEEKQVGGVLAYIFSKEEDPTFAIYAKRREASSQRCESELERYLAQSSVDLDINKPLDVLDWWKAQEGNYPLLSAMARDLLTVPVSSVPLELAFDSSERLINQCRSYISPKLLEASVCLRDWYKAQEGSQHAKLSELEDLMSEKEF	zf-BED--DUF-domain--Dimer_Tnp_hAT--	I
Aquca_002_01079.1	AqZf-BED02_I	MNFEASNGKSGQMVEWNANAFKSLKDMEPKSTMESISTIDPTVIALASEAIQKGTTVAAVKPRKKTMTSLYLRYFETAPDGKTRSCKFCKQSYSIATATGNLGRHLSHRHPGYDKVSDVITNGNPAQQPTTVIKKHQSPARPVSVDLDHLNWLLLKWLIGASHPPGTLEEEGLANSFKFANSLVKIWSTERFQAVILEVYKSMREDVRTSLEQVKSKISITLDFWTSYEQVFYMSVTGHWIDENWILHKVLLDICHIPYPCGGADIYHMLLKVLKMYNIENRILCCTHDNSQNAFQACHTLNKEDLDGRKTGSLCYIPCAARTLNLIIEDGLRTAKPVISKIREFALVMNTSAEISEDFRQITAAYQEGSWKFPIDASNRWSGSYTMLDIMRKASKSIDAVIRKHAETLASRNVLLSPPERNAVNIMHNYLEPFYKTTNNICTSKVLTIGLVLFFMDHVVEMINSCRDSRQSPDWLKSAADDMSKTARTYNSQVYNLYTYMAAILDPRIKSELVPENLNSESNLEDARSHFLRNYSSSHFPAMANGYTVQENEDGGSLSFAEEIARKRRRISMNTTTDELTQYLSESPAPFTTDVLEWWKGNTTRYPRLSVMARDFLAMQATSVSPEQLFCGKGDEIDKQRICLPHASMQAILCTRSWTDSGFKVRYRSTEIDYERLMESASAVTDNGTLHSEDKAK	zf-BED--DUF-domain--Dimer_Tnp_hAT--	I
Aquca_005_00314.1	AqZf-BED03_I	METVIESSTLPAKKRRKSDVWKYFDKVERPDETGKIVPKAACKVCDNSLVYGGTHGTSHLAKHAVRCCERTTEGGQQTVLAIVKNCSSRNCSILCFREELPFNKVDKPAFRRYISKSFGPQFKPPCRVTMRNDVMLLFKEEKTLLQNVLKQVPGRICFTSDLWTSNQKLGYMCITAHYINSEWELNKRVISFPMLPSPHTGKAISEEIYSNLLGWNICDKVGTLTLDNASNNDSAISRLKDSLFGLDLLRRDLFHVRCNAHIINLLVKDGLDHVVSSLDNIRESVRHVKTSQAKLQSFFECCQRLGMKEKRLPIDIQTRWDSTFLMLEAAIPYQKVFDLFFYHSDFQHIPTPTDWNNAVVLQDFPKVFKDSTKMFSGSGYVTSSLFCLQMSKISGVLLKYGNQTGFKVMCDSMKLKYNKYWQNIPLVLALASCMDPRYKLTILELCLDLNHGSESGLEVVSNESELKIGAYKIKLSELYEEYEKKVNSNATGHLEATSSLSVDDDIVAVMLSRRRESSIKTCKSDLQRYLDQPPVQVQPLPKFNVLEWWKAQESTYPVLSAMARDLLTIPVSTVASESAFSGSERVVSKCRSCLKPDIVEALMCLKDWFQAEEGLQDEDPDDIEELMREEP	zf-BED--DUF-domain--Dimer_Tnp_hAT--	I
Aquca_005_00564.1	AqZf-BED04_II	MLLSCLANNIRIQKSFYSKPDKERSEASCVQYNMPRKKDITWSHCKEVEGNHNAVICNYCGKQYMGGGITRFKKHLAGGNPNVETCTKCGKEVRDLFRKIADKMVEKKMQKQLLKKSSTMSSYTSQGRQKMNLMMKKPVICDELCRTRCIQLWIIQLYAFKVFSTVNLTQKPIGTNSNHIH	zf-BED	II
Aquca_005_00638.1	AqZf-BED05_XXII	MAKRDIGWEYGFVESSDETGIVCGFCEKKMRGGGVTRFKGHLAGVGNDVTSCTKVLPDVRLRIQASATSISATSSSSEDSKLLISFKLALQNPNLLSTWQTNQDPCYFTGVICKTSRVSSIDLSSINLNLDFNLVSSYLLSLQQLERLSLKKTNLTGNLTSAAWKSTSCSEMLAEIDLAENNLSGSISDISSLSGCKSLRFLNLSRNLGFSSNQK	zf-BED--LRR-motif--	XXII
Aquca_006_00358.1	AqZf-BED06_V	MKKKDKFWDYAEELKGRFLCNFCQKDFSGGVARLKSHLSKQTCRDIAICEKVTEDVQAAALLAVGRVDIYEEVCWVKKVFDDAKYVVDYIDRHVNILALQRYFTDNKELRKYNKTRFASYFLMLQLIIGLEDAPDFWLHGNDVIKALEPIVGVLRLVDGDGSNAGYLYEAMVRAQETLKKQKIVNPDKFSRIWELFEIRRNNNILNRVYATAAFLNPSLMYDGIITYDNQDVSKGLLFVGEKMVSFDQRDDFASQLLVYQGKHPKLFNFLSISQLRTAHPKIWWEVNGGFVPLFIKIAIRILSQPCISSACERNWSAFDAAQTKKRTRLLPQILDDLVYIRMNSLMMTRYDEVELQDRGPINLEELGDLPEDVDDGQDELMDEPIGEEPVDVVGDDDSWLSRRISIGTFSANREDLF	zf-BED--Dimer_Tnp_hAT--	V
Aquca_009_00684.1	AqZf-BED07_I	MPRARDIGWQHGKMIGGHRHHVQCNYCHRTMIGGITRFKKHLASKRGEIRGCEAVPKEVRELVKKHLAAWRKRKRMGKNKKKVNDEALNESSSEDKDTESNESDQDMESAKLESLQALHEVEVHHPTTNDNQLPMVGTREFFDAFSSGQCTNDQGSAPPRATDLGWAHGLMVNGDRQKIECKYCHKVILGGGISRLKQHLAGERGNIAPCEKVPEEVKAQMQQHLGFKVLQRLKKHKESEKVKKPLISSMQGKEEANNDDVQGSPNTTPQINNGRRRKKEGGEASYGKRKRHKRQNITFATPVNQRPSFISQESLDQADRAVAKFMYDAGIPFSAANSMYFQLMADAIAAVGPGYKMPSYDSLRGNLLNRSVEETGELCLELRKSWEVTGCSVMADRWTDRAGRTVINFYVYSSKGTMFFKSVDASEISKSSEALFNLFDSIVHDVGLKNIVHFVTDTTPNYKAAGKMLMSKYKSFFWSASAAHCIDLMLEELGYMDEIKEVLSKAKRVCQFIYNNDWLLSLMRKRTGGRDIVRPSITRCITNILTLQNIMSLKDPLHQMFTSTIWMQSDLSKQRAGLEVTDIVVDPQFWSSCGQVLKVTKPLLTVIHVADSEERPSMGYIYDALEKAKAEIASAFSNKESEYFPYLKVIDHIWEEELHNPLHAAAYYLNPSIFYNPGFSTSKLIQKGLLDCIETLEPNLTAQDMITRQITFYEDAVGDFSRPVALRGRESLAPATWWSLYAADYPDLQRFAVRILSQTCSATQCERNLSMFERTHLKKKNRLEHERLDNLIFVHYNLRLQERQSTVNKSMAKVVQDPTCLEEMDIDVSEWVEDLSSLDGDGLGWMDVAMPTEAICVNNTRTMKVDSSNYSANEGSSDETNSSDRNDEL	zf-BED--DUF-domain--Dimer_Tnp_hAT--	I
Aquca_009_00913.1	AqZf-BED08_I	MASSSAGSQSQPVPIGSLKHDPAWKHVQMYKNGDRVQLKCLYCLKIFSGGGIHRIKEHLACQKGNASCCPTVPPEVTRAMLQSLEGVIVKKKKKQQMVNGIKSNTPDTNDIDMDTFGTPSEMKLVVTPSSVIHSTIERFMKSDEGMSAQSLDTRKRGRVENSSSFVTPDTSPCPGNLVSRNEKDEVHMAISRFLYDVGLSPDAANSPYFQPMIDAIATVGPGFEAPSYHDLRGWILKNSVHNTNALVDKYKAAWGRTGCSVLADEWTTENNRTLINIFIYCPEGTMFLRSFDVTDTLQPSDVLYDLLKGVVEEIGVNHVVQVITDSGEHYINAGKKLTETFRTMYWTPCAARSINMMLEDMGRIEWINVILERAKSITRFVYNHGTVLNLIRRYTGGRDLVQPSVTRSATDFFTLKSMVHLKGNLQVMLNSQEWMDCPLSKHPDGMSMMDIILSQSFWEACSNIIRLTDPLVGVLRSVDSSKRPAMGHILASMHHVKEAIKNELFDEEAYLLYWNIINSRWDEQVQHPLHEAGFFLNPGLYYSLEGDVRDKIPSGMLDCIERLVPDINIQDKINKELISYKNAVGDFGRKMAIRAAHTLLPAEWWSTYGGGCPNLARLAIRILNQTCSASFCKLSSIPFEQVHRTKNHLERQRLSDLVFVQCNLRLQQTNLRKYKDPAVMDPISTDSIGLTEDWVTEKMDFFGSEDSDWVALHQPVNNSMLLDSPNAEPENLVAGFDYQERPN	zf-BED--DUF-domain--Dimer_Tnp_hAT--	I
Aquca_009_00913.2	AqZf-BED09_I	MASSSAGSQSQPVPIGSLKHDPAWKHVQMYKNGDRVQLKCLYCLKIFSGGGIHRIKEHLACQKGNASCCPTVPPEVTRAMLQSLEGVIVKKKKKQQMVNGIKSNTPDTNDIDMDTFGTPSEMKLVVTPSSVIHSTIERFMKSDEGMSAQSLDTRKRGRVENSSSFVTPDTSPCPGNLVSRNEKDEVHMAISRFLYDVGLSPDAANSPYFQPMIDAIATVGPGFEAPSYHDLRGWILKNSVHNTNALVDKYKAAWGRTGCSVLADEWTTENNRTLINIFIYCPEGTMFLRSFDVTDTLQPSDVLYDLLKGVVEEIGVNHVVQVITDSGEHYINAGKKLTETFRTMYWTPCAARSINMMLEDMGRIEWINVILERAKSITRFVYNHGTVLNLIRRYTGGRDLVQPSVTRSATDFFTLKSMVHLKGNLQVMLNSQEWMDCPLSKHPDGMSMMDIILSQSFWEACSNIIRLTDPLVGVLRSVDSSKRPAMGHILASMHHVKEAIKNELFDEEAYLLYWNIINSRWDEQVQHPLHEAGFFLNPGLYYSLEGDVRDKIPSGMLDCIERLVPDINIQDKINKELISYKNAVGDFGRKMAIRAAHTLLPAEWWSTYGGGCPNLARLAIRILNQTCSASFCKLSSIPFEQVHRTKNHLERQRLSDLVFVQCNLRLQQTNLRKYKDPAVMDPISTDSIGLTEDWVTEKMDFFGSEDSDWVALHQPVNNSMLLDSPNAEPENLVAGFDYQERPN	zf-BED--DUF-domain--Dimer_Tnp_hAT--	I
Aquca_010_00565.1	AqZf-BED10_II	MDASTIPVICYFGGNLSSPNVPDYIGGKSAASFINRSITLSELCDKIKDTCRIYEGTVIKLKCRYPLNQSTAITLDICDDSSLAVAMATIPTMSSSLVVYVEELAKQTVVAAKLTVTRASPSKIGTRKDIGWLHCTSLDGTTNKTQCNYCGKVMSGGGITRVKKHLAGGTNDVENCTKCGPEVRDMFRDLIRGKKQSKFEKRLKEQQYRKPSLLMRTAEDGSPAVQSQFSGEVSSCQANEEMLFVAEDFGSQ	zf-BED	II
Aquca_010_	AqZf-	MPKAKHMAWKHCSSVDGNHNAVTCKYCGRTFLGGGITRFKRHLASGDPNVKICAKTGKDVRNLFRQVVDQAVQRRNAKAVAEKNFQDALLHIDDSDEDEYSDEDPELRDFQRRRHSSFVGSNKERGSGSGSGSRRQTQSSINNLFRSGSVTSRSRRWGQKFKNLFTKMAHYDGINANQLVGSNPYTQPAIDAACRAGLGVRILTAYELLGPQLDIIVGECDKFIDELKQKKWAQY	zf-BED--DUF-domain--	I

00639.1	BED11_I	GVTAMCDGWTGPTKHSLINFMVYCDGDSCFIKSVDVSAFYKDASLILEQMKAVVDKVGKRYVVQIVTDNEANYKKAGEDLCKLLTGCAAHSIGLILKEFCRKHSVKKVIMRARKITKYIYNHSQVCDLMREICGGDIVRPALTRFVANFLALQRLLMKQECLKDMIIHPKWIAIRTRTKDQVMVEVEDLINSRPLWRKIKCICDSFEPMVETLRKVDDDKVPKMGFLYKWMENLKEKLRLFRQQTGSFGNKILVRAAEWWINYGYDAPSLRKVAVRILAQTVASSGCKRNWSTFDKLDKLVFSHYNLRLKQKNARRFAVLDNEPVDLTLLYPRVDASDSDDAPMDWVKFEVGGDLELDEAPRVPNAMIANELGVNVQDNILPVDPVHTQLNVPRSGKDDPLGDNPNIPGDGSDLFRVHVPSGSPSSSDDGGTGGVISSALGNGVAGSSRVYESFSDFRFTGESQFDHATQDDDHGATPIPKRHRQRRLHPTDETESSRVEDSPSTYQEQYGYNPSLAAYLGDNYTPDQGSSDSSWNTPYSTVGNYGSIHSTESPYGGLYNDGCYPNPLHPYSQSDYPPRDGAWFPDANQWLSSMTSVSDVPSLDTA	Dimer_Tnp_hAT--	
Aquca_010_00688.1	AqZf-BED12_XVII	MAKRNIGWEYGFVEPDDETGIVCGFCENKLRGGGVTRFKEHLVGVGNDVTSCTKVIYRNLLHDELFLGPHDEEDHDDDRRVEEVGRQEQEVRRREPVTSGASSHQIEIQDEDDETRAQRDLEEAVRRLTQDPNYSKQRDLEMAIEASRREHLSSSTKGVSSSRDFTDYDSDLEFKFVCFILVGLYILLILTQTSHARYLKLGIIPTIDTHLIRDYCYSKEFQHARLLFDEISNWDLVSTTTIIGHFAHHDRHQDAITLFSRMLYMDIRPNEYTFGTVIHSSTSLRALNVGKQLQAIAMKMGFCSNVFVGSSFVDLYVKLSTIEDARKAFEDTHHPNVVSYTSLICGYLKNERFEDAQMLFSRMPERNVISWNAMIGGYSQMGQNEEAVNLFIEMCRQGFQPNESTFPCVFSAAANIAALGMGKSFHACAFKLLGKYGVFVGNSLISFYAKCGNMEDSLLAFYRLHGKNTVSWNAIISGYAQNGKGKEALDFFQKMQFSGIRPNGVTLLCVLLACNHAGLVDEGYACFNLAQIEYPSVLRAEHYACMVDLLSRSGRFGEAEKFLQELPFNPGIGFWKALLGGCQIHSNMKLAELAVREIHALDPEDVSSYVMLSNASSAAGKWQSASLIRKEMKDKGMQRIPGCSWIEISGKVHVFTTRDKRHCEIDEVYTIMGFCLEHLKAIPNFNLFPED	zf-BED--PPR-domain--	XVII
Aquca_012_00053.1	AqZf-BED14_II	MASSYASSQATQASSERYRPPKSTDIGWDYGQRVDGDNTVISCDFCGHTSRGGIRGFKHHIVKGFSKSLSKYKDVQPCKQVSKDVEKVLKYMNEKEKVTQEREKQRRNA	zf-BED	II
Aquca_015_00235.1	AqZf-BED15_XXI	MVKGKDAAWAHALQVEGYPSGTQCKFCDKIIRGGGISRFKKHLAGGDPNVESCKKVEKEVSDLYREILDQSASKKKKNALTLIDLAELDDTKGLHNLVIVKQCDDNFQLKRARKELSNSLARKRSISEATYEIPTMDEGERLTMTVDEGKRTVLTKELEDVDTNNALIVRQHVDSDELIESRKELISGWKGFSDNLYVIGIKQMGELDIKPFKAACYRKYQSPHVADEKALQICSFWQSKLADPSWHPYGVTEELEEDEQLKELKLDLGSEVYDAVCRALMEINEYNASGRCPVDELWNFRENRKAKLQEAINILLKMKKE	zf-BED--XH--	XXI
Aquca_015_00235.2	AqZf-BED16_XXI	MVKGKDAAWAHALQVEGYPSGTQCKFCDKIIRGGGISRFKKHLAGGDPNVESCKKVEKEVSDLYREILDQSASKKKKNALTLIDLAELDDTKGLHNLVIVKQCDDNFQLKRARKELSNSLARKRSISEATYEIPTMDEGERLTMTVDEGKRTVLTKELEDVDTNNALIVRQHVDSDELIESRKELISGWKGFSDNLYVIGIKQMGELDIKPFKAACYRKYQSPHVADEKALQICSFWQSKLADPSWHPYGVTEELEEDEQLKELKLDLGSEVYDAVCRALMEINEYNASGRCPVDELWNFRENRKAKLQEAINILLKMKKE	zf-BED--XH--	XXI
Aquca_024_00047.1	AqZf-BED17_IV	MSKSNRDFAWKYAREIEGDKNHYCLYCNRKSSGGISRLKHHLADFDSLDDENMRSSGASVTQPKQRGPMDAFVSSKPKQTTFNSAYKKELRTESCRNIGRLFYHKVLPFNTVNSPFWLPAINVILKVEVDDIDLIKEEHKKVWKQYGCSIMSDGWTDGKNRVLVNFFINSSTDTIKNGENMFKYLDAVVDEVGEENVIQVIIDNAANMKNVGKRLMEKRNQGGGLRDIGKMKVFKETLDAAKNAIKFIYGHSIILAMMREFKGNKEIIRLAITRFATGFLTLQSRKNGGKGKKQKSIQVVEDEEETQDVGAMDSGGFPNIDTIDHFDSDVLRDEEFGGISLSQPMGDDFKGVDSDEDDLN	zf-BED--DUF-domain	IV
Aquca_029_00055.5	AqZf-BED22_XV	MVEKMAPLRSSGSVDPGWEHGSAQDDRKRKVKCNYCGKIVSGGIFRFKQHLARISGQVTHCNNAPEEVYLRMKENLKGCQSTKKHRQSRDVIASLYLHSSDYDEVEDESIDYGSKGKHVLDDKNMVVNLAPLRSLGYVDPGWEHGVAQDERKKKVKCNYCEKIVSGGINRFKQHLARIPGEVASCKNAPEEVYIKIKENMKWHRTGRRNRRPDTKDVPTFTLHSDNDEEEEQEEDPIYFRNKEKMLICDKSFAKDTRKRSRGRSPCSGAELQLKRPKLDYTTMKTPKIHTPAFYKPVKTVSDRRTQKELMSAICKFFYHAAVPLNVASSPYFYRMLELVSQYGQGLKGIYSPLICGQFLQDEITTIKQYLVEIKASWMITGCTIMADSWKDAQGKTLINFLVSCPRGMYFVSSVDVTDIVEDPSSLFKLFDKVVEEMGEENVVQVITENTAAYKAAGKMLEEKRRTLFWTPCAVYSIDRILEDFQKIKWVGECMDKGKKITKFIYNRIWLLNLMKKEFTGGREVICPSVTRFATSFVTLESLLNHRNDLKRMFESNKWFSSRFAKLDEGKEVENIVLNSTFWKKVQYVRKSVDPVLQVLLKMDSDGSLSMASIYNDMYRAKLAIKAIHGDDVRKYGPFWSVIDNHWSSFHHPLYMAAYFLNPSYRYRPDFVEHIEATRGNNECIVRLEPDSGRRISASMQVSPY	zf-BED--zf-BED--DUF-domain--	XV
Aquca_030_00025.1	AqZf-BED23_IV	MSVPSSMDSIDGVSNSSKRKKLSGSQNTLRSNVWLEFERSKDKQSAICKHCKETFRVNGTSSLRNHLNRCFMKKQNESGQERSRKDEGTPSTKMIKFCQERSRKDFSEMIVKHNYPFSMVEHEYFRKFVNNLQPHFDLKHRNTVRGDIVKLYKEEKNALYSILGKLSSRVSLTTDLWTAQCTRDSYMCLTVHYIDDDWVLRNKILNFRLMDCSQTGSELERIIMKCLFDWNVDKKVSTITVDNASSNDVMVSLLKANLNQDKLLVFNGKFFHIRCTTHILNLIVKDGLSAMGNTLNRIRESCKWNSTYQMISTALIYEKAFKRLHMKDEDFKTNPSPEEWVLAQKICTCLKNFYSSTMSLSGMDYPTADLYFSNVCEIHIALLEWKKSVTEEVKKMADSMYEKFKKYWSSCCLILAVAFVLNPGFKLKFVENYYSIIYGDYESAFQTRKVREAMIEIYSEYSPPTTNPLFTSQPSSSSSSQQLFEENSPNSNISIRFESWYQVDDDLCSTVAFSA	zf-BED--DUF-domain	IV
Aquca_039_00001.2	AqZf-BED25_I	MEAENKPPLKRTKRLTSVVWNDFERVRKNDLMVAICKHCNKKLSGSSTSGTSHLRNHLKRCLKKSNHDISQQLHLVREKKKDGTIDVGISKCDTAIGPLDLKFDEGHSRLELKFDQERSRLDLAHMIILHGYPLSMVEHVGFRRFVSNLQPFFQIMSGDGAKAVCMQIYQKQKQKMNEILDKLPGRISLSAELWTSYQDTRFLCLTAYFIDEAWQLQRKILNFIMVDPDTEQALSEAIMTCLTNWNIDRKLFSITVDSCSNNDNAVFRVRDRLSQNRSILRNIQLFHVRCATRILNFIIEDVLEALQEVTHKIRESVRYVKSSEAMQQKFDQMALQVSQKSLLIDSPTQWASTYVMLEAAIDYRGVFSRLQACDPCYLIAPSDVEWERTVALINYLKLVIEVTNVFSGAKYPTANIYFPEICDIHLKLIQLSKSKDSCISSMALKIKDKFDNYWSVSGLTLTVAVVLDPRFKMKLVEYYFQQMFETNAPECIKEVSGGIKDLFHEYSIRSTLTSFEQSLDGQIENSSGLDGILPNVSNEANDRLSGFDKFLHETSSIPHMKTELDKYLEEPIFPRNADFDVLNWWKVNSPKYPILSVMARDILGICVSIADSSAAEFDAGGKVLDMQRSSQSPDVLQAMVCTHDWLGTEFESELAASDSTAPLSVTAK	zf-BED--DUF-domain--Dimer_Tnp_hAT--	I
Aquca_040_00120.1	AqZf-BED28_XVII	MSVPSSMDSIDGVSNSSKRKKLSGSQNTLRSNVWLEFERSKDKQSAICKHCKETFRVNGTSSLRNHLNRCFMKKQNESGQERSRKDEGTPSTKMIKFCQERSRKDLSGMIVKHNYPFSMVEHEYFRKFVNNLQPQFDLKHRNTVRGDIVKLYKEEKNALYSILGKLSSRVSLTTDLWTTQCTRDSYMCLTVHYIDDDWVLRNKILNFRLMDCSHTGSELERIIMKCLLDWNVDKKVSTITVDNASSNDVMVSLLKANLNQDKLLVFNGKFFHIRCTTHILTLIVKDGLSAMGNTLEKVLESSRSSPRYSILPYCTNFMANLVSRYLLTTFLFQNPNYSLMVVRPYSSSSSVENYWKLLQRSNDSQTNLEKTLTNVRGKLDSSIVEIILKRCSINRSSLLGLRFFIWAGLQPDYRHTSYMFNKACKLFEIHQKPQTISDVLESYRMENRLVSVKTFKVVLNLCGEAKLADEALGLLRKMGEFGCRPDTTIFNVVIRMFSDKGGMDVAQGLMREMALIDLYPDLSTYITVIKGFCRVGRLEDACQLIEVMRNHGCVPNVVVYSILLDGYCKIGNFDRALEFLGEMEKEDNGRMPNIVTYTSLIQNFCEKGKTMEALTILDRMESRGCFPNRVTVSTLVKHLVFEGNIEAAYKLIDKVIANGIVSRNECYSSLVLSLLQNKKMDEAKKLFIWMLGNQIKPDGLACSILIKQLCLEGSILHGFEYYVEMEKRDFLASVDSDISSILLAGLCQQGHLVEAVRLINAMVERKIKLKAPYADSIFESLHKSGESELALHLMRVQELV	zf-BED--PPR-domain--	XVII
Aquca_046_00030.1	AqZf-BED29_II	MPRKKDIAWSHCKEVEGNHNAVICNYCGKQYMGGGITRFKKHLAGGNPNVETCTKCGSSEDESSDEEARDMRRAMQDSLVDQWNEDQGYRLSCMDPTQETSLITLIL	zf-BED	II
Aquca_056_00036.1	AqZf-BED30_IV	MPRKKDIVWFHCKEVEGNHNDVICNYCGKQYLGGGITRFKKHLAGGDPNVETCSKCGKEVRDLFRKIVDKQVERKNAKAIAGKEFHDVFLHVPGSSEDESGDEEARDIRRAMQDSLADQWNEDKAYRLSRSQSQFVNSQREAGSLKSSCDPELIDLDTERLPDTRQSTIPSKFDKQRNKGWGAKFRNLFAKMAHYRGINANQIVGNNPYTQPCIDAACRAGLGVKVPTRYELMGPELKNIVSECDMFIDSLRKKWSLYSVTIMCDGWTGPTRHSLMNFMIYYANLLRNEMKKVVDWVGKEYVVQIVTDNGANYKKEGEDLCKLPEFCRKDMVADLIAQARKITKYMYNHSQVCDLMRNCCSGDIVRPALTRFATNFMALQSMLNKMEERQRDRKMQEVEDIINKRQFWRDVKELVECFQPMVEILRKWMMNLKDKIKKTKKYPKWCLDLVERRWERQLSHTLHKAEHRGRGSPRTQPNEPRQRQAVTAGISTQQDAEVQEEDDTILEQRNLEEALRHSTQDPDYLEQEELERALQASRREQTLFSSRASSSRTPHEEIDYDSDDPLAFLDDL	zf-BED--DUF-domain	IV
Aquca_108_	AqZf-	MVRQKDQFWEYADDLKGRFLCKFCQKNYSGGIARVKSHLSRLQGRDIAICNSVPEDVQASAVLALQGTGPSYKKRKAALSLMNGEGSGVELPVRSAIPCAESLPKLSQTTVHAICDKEDKESNKKDKESVDRMVAQAFMMNNIGVDVIQSPSFISMVKSIAEFGSGYSLPSCATLCTKLLQDAKKEVDEYVSTVKGSWSLTGCTLMLDTSDSDVIAYSPKGAIYLKSWERTNDSSVFLADVLIPIIEEVGSENVVQIIVNNGFDIESENLVTERYPHIYRTRCVSRGIQLLLEDIFKEVEWIQSVVDDAKLIVDYVYKYPVVLKL	zf-BED--DUF-domain--	I

00016.1	BED33_I	MRVHTSDKDLKRPCKTSYVSYYMMLQSLIEVEDSLRMMVVSPEWSVVNESKIPTATQIAQIIQSTDFWSRIKEVISAAELIM<br>TILRLVDGDGSTAGYLYEAVERIGGEFKQRCSIDESKYSKLLKLYNSRRNSDIIQKIHAAAAFLHPSFMYEGKIKYEKSDIRD<br>GMNYVVEHMVNPDEMDDFAAQLLLYNGKNLKLFNTLSVLMMKKAHPRLWWEYNGGEVPLLRKLAIRILSQPCSSSCGR<br>NLSGFEVAQSENIHRSQHTYEDFIYTRINMKMMAKYNDLEMQDKFALGLENLGEYIKKNPGEHPEDIHDGYDKSQYEGGR<br>TGLNGDTIMLNEKSGPGSSSL	Dimer_Tnp_hAT--	
Aquca_124_00007.1	AqZf-BED34_I	METVIESSTLPAKKCRKSDVWKYFDKVERPDETGKIVPKAACKVCDNSLVYGGTHGTSHLARHAVRCCERTTEGGQQTV<br>LAIGPNGLICTFTFSQKTARQETVRYFVREELPFNKVDKPAFRRYISKSFGPQFKPPCRVTMRNDVMLLFKEEKTLLQNVL<br>KQVPGRICFISDLWTSNQKLGYMCITAHYINSEWELNKRVISFPMLPSPHTGKAISEEIYSNLLGWNISDKVGTLTLDNASN<br>NDSTISRLKDSLFGLDLLWRDLFHVRCNAHIINLLVKDGLDHVVSSLDNIRESVRHVKNSQAKLQSFFECCQRLGMKEKRL<br>PIDIQTRWDSTFLMLEAAIPYQKHVPTPTDWNNVVVLQDFLKVFKDSTKMFSGSGYVTSSLFCLQMSKISGVLLKYGNQT<br>GFKVMCDSMKLKYNKYWQNIPLVLALASCMDPRYKLTILELCLDLNHGSESGLEVVSNESELKIGAYKIKLSELYEEYEKK<br>VNSNATGHLEATSSLSVDDDIVAVMLSRRRESSIKTCKSDLQRYLDQPPVQVQSLPKFNVLEWWKAQESTYLVLSAMAR<br>DLLTIPVSTVASESAFSGSERVVSKCRSCLKPDIVEALMCLKDWFQAEEGLQDEDPDDIEELMREEP	zf-BED--DUF-domain--Dimer_Tnp_hAT--	I
AT1G18560.1	ATZf-BED01_I	MEWNVNNAFKTYKEMEPKAMMDMTLVPHSDPIDIGLGSSDKSNSVPPKRKKTMTSVYLKYFETAPDSKTRKCKFCGQS<br>YSIATATGNLGRHLTNRHPGYDKAAADVVTSSSVPQTPPAVVKPSQSQSKVPQLDYDHLNWLVLKWLALSSLPPSTVDE<br>TWLGNSFKFLKPSIQLWPAEKYKAILDEVFTSMRGDVKTTLEHIQSKVSVTLSFWNSYENIFYMSVTGQWIDENWSSHRL<br>LLDICRIPYPSGGSEIYNSLLKVLKTYAIEDRILCCTHDNSENAIHACHSLKEYFDGQKVLPFCYIPCAAQTLNDIIDEGLATIK<br>PIISKVREFTQELNASTELSDDFIQLTTAYQEGNWKLPIDASSRWSGNYQMVNILCKASKSLDSVIRKNEDALENRMMLSS<br>VEKNAVTIVHNYLDLDSFHKTTNDMCTNKDLTVGLALLFMDNISEMITTCQKSCHNPDWLRTCAESMAQKARSYNTQVCN<br>VFTYITAILDPRIKTEYIPETINLESYIDEARSHFIRNYSSSHFTSSMTSGYRPQEVDEGGGNISFAEEIARRKRRGSMSNNV<br>VDELTQYLSESIVPMQTDVLDWWKVNSGRYPRLSNMARDFLAVQATSAAPEEIFCGKGEEIDKQKYCMPHDSTQSVICIR<br>SWIEAGMKLKYKCSEIDYERLMELAATVAADNSAGGLEKIQQHR	zf-BED--DUF-domain--Dimer_Tnp_hAT--	I
AT1G36095.1	ATZf-BED02_IV	MSDELSHKNLDPVKKYAQPVPLKHGSWRCNYCHKVTNGGVKGAKQHILGGFRNVTQCSLVPPIMREEIKDSMLKKTEIK<br>ATTQMMPPPATSYDDYGEEEEAAEVLGNERRQPPVKKQKGLMDMFVCPTLPNVLKVLKDANMLYDHLDLMVDEVGEAN<br>VVKVVIDNASNYVKASQLSMANRPHLYWTPCAAHCIYLMLEDIGKISEVKTVITQCIFKNDYIYGHTSLVNMMRKIHKRWKS<br>AKIGCNTVCYVFHTIGQYHKQRKNLRNSATSQEWADSKWQKEIGARTVKRIIMQDSFWHKDG	zf-BED--DUF-domain	IV
AT1G79740.1	ATZf-BED03_I	MVREKDICWEYAEKLDGNKVKCKFCSRVLNGGISRLKHHLSRLPSKGVNPCAKVRDDVTDRVRSILSAKDDPPITNKYKP<br>PPPLSPPFDAPASKLVFPSSPPNAQDIAERSISLFFFENKIDFAVARSPSYHHMLDAVAKCGPGFVAPSPKTEWLDRVKSD<br>ISLQLKDTEKEWVTTGCTIIAEAWTDNKSRALINFSVSSPSRIFFHKSVDASSYFKNSKCLADLFDSVIQDIGQEHIVQIIMDN<br>SFCYTGISNHLLQNYATIFVSPCASQCLNIILEEFSKVDWVNQCISQAQVISKFVYNNSPVLDLLRKLTGGQDIIRSGVTRSV<br>SNFLSLQSMMKQKARLKHMFNCPEYTTNTNKPQSISCVNILEDNDFWRAVEESVAISEPILKVLREVSTGKPAVGSIYELM<br>SKAKESIRTYYIMDENKHKVFSDIVDTNWCEHLHSPLHAAAAFLNPSIQYNPEIKFLTSLKEDFFKVLEKLLPTSDLRRDITN<br>QIFTFTRAKGMFGCNLAMEARDSVSPGLWWEQFGDSAPVLQRVAIRILSQVCSGYNLERQWSTFQQMHWERRNKIDRE<br>ILNKLAYVNQNLKLGRMITLETDPIALEDIDMMSEWVEEAENPSPAQWLDRFGTALDGGDLNTRQFGGAIFSANDHNIFGL	zf-BED--DUF-domain--Dimer_Tnp_hAT--	I
AT3G17450.1	ATZf-BED04_III	MAPPGSIGVVDPGWEHGVAQDQRKKKVKCNYCGKIVSGGIYRLKQHLARVSGEVTYCDKSPEEVCMRMKENLVRSTKK<br>LRQSEDNSGQSCSSFHQSNNDDEADEEERRCWSIRSKGKLGLSDGSLLRSSGYIDPGWEHGIAQDERKKKVKCNYCNK<br>IVSGGINRFKQHLARIPGEVAPCKTAPEEVYVKIKENMKWHRAGKRQNRPDDEMGALTFRTVSQDPDQEEDREDHDFYP<br>TSQDRLMLGNGRFSKDKRKSFDSTNMRSVSEAKTKRARMIPFQSPSSSKQRKLYSSCSNRVVSRKDVTSSISKFLHHVG<br>VPTEAANSLYFQKMIELIGMYGEGFVVPSSQLFSGRLLQEEMSTIKSYLREYRSSWVVTGCSIMADTWTNTEGKKMISFLV<br>SCPRGVYFHSSIDATDIVEDALSLFKCLDKLVDDIGEENVVQVITQNTAIFRSAGKLLEEKRKNLYWTPCAIHCTELVLEDFS<br>KLEFVSECLEKAQRITRFIYNQTWLLNLMKNEFTQGLDLLRPAVMRHASGFTTLQSLMDHKASLRGLFQSDGWILSQTAA<br>KSEEGREVEKMVLSAVFWKKVQYVLKSVDPVMQVIHMINDGGDRLSMPYAYGYMCCAKMAIKSIHSDDARKYGPFWRVI<br>EYRWNPLFHHPLYVAAYFFNPAYKYRPDFMAQSEVVRGVNECIVRLEPDNTRRITALMQIPDYTCAKADFGTDIAIGTRTE<br>LDPSAWWQQHGISCLELQRVAVRILSHTCSSVGCEPKWSVYDQVNSQCQSQFGKKSTKDLTYVHYNLRLREKQLKQRL<br>HYEDEPPPTLNHALLDRLLPDWLVTSEKEEEEALHGEDRAEKEDHEDDEEEEEKFYMESGNVDGEGEDNLDPYDDDLS<br>DDD	zf-BED--zf-BED--DUF-domain--Dimer_Tnp_hAT--	III
AT3G48770.1	ATZf-BED05_II	MEKAFSPNTSNIKQEKSSNRSSHHKQDLAWNYVREAIDSRRRKVITCGFCGKQYVGSEINRMKHHLAGLNNRSACQKVS<br>SEVQHSMRKSLEENKEKPKKIIVVDLDMQDDEVNVGQSSHQSCSSSSRKRKVQHSMRKSLEETEEKLKQIIVVDLDMQD<br>NEVNVGQSSLQSFSSSNRKRKRETKTKPVIIETMTESAKQHIERIRRTKFSIGGAENPLTEDLHQAVKNLSAELYAKDVHFL<br>MELIQNAEDNEYPEGVDPSLEFVITSEDITNTGAPATLLIFNNEKGFSEKNIESICSVGRSTKKGNRKCGYIGEKGIGFKSVF<br>LITSQPYIFSNGYQIRFNEAPCSHCSLGYIVPEWVDQHPSLVDIQRMYGSGSALPTTTIILPLKSDKVKPVKEQLSNVHPEV<br>LLFLSKIKRLSIREHCLDPKLSTVNSIGIVSETNFVTRKSIDAESYTIHLSASEKGKNSEQECSYYMWRQKFPVKHENRVDR<br>RSEVEEWVITLAFPFGERLGHGNNSPGIYAFLPTEMVTNFPFIIQADFILASSREMILLDDIWNQGILNCVPLAFLNAFTSLV<br>KTTDAPVSSLLPAFRFLPVKESNYAKLNVVRESIRARVCAEEIVPSISHQGQKFFYKPCEVGRLIPTFWDILEKAGSEGASL<br>QNISSHGIYILNSAFDRTEYDNVLNFLGLKQVSNEWYVKCIQGCDLVTSVSEATYVEVLLFIAENWQCRFQNTNMGKVPLI<br>KYVVQKGVSSLSSLGGFSPRTLCLSTEKNQAWLLDWNDEFRCMSNFVFMPPTTRTALKVCSKKEIIHTWLKENVKVITLS<br>VSDYAKHLRENLNGDKRLVVAYAHFLHHSISKDFLSKEEAGKCCKDMPLVDNYGNVNISRNGVLVPASAGKWVSLVGSN<br>PWRHSGYIELSEEYLLSNRFAGLRSNKKDLLGFLKSSVEAGDIPDIEPPNVAIPALSGPLTKENVLLLLEWINKCNRHSLRS<br>NFLNSVRGGSWLRTTMNGVSDYRPPSQSFYHTSSWGSILQNGSILVDIPLSYYGNEIEKYKEELKIAGVMFEFSEVCRFV<br>GNHLMSLAETSTQSSANVFSILKFIRYLREKRLSPADFITAVKNGPWLKTISGYRSPDGAVLFSEEWKAASLISDIPFIDRGF<br>YGVVSLNGYKEELELLGVVVKFPDNYSLIVSHLNTAKLTYLTPDAMFLVLDCMRQLSPHRLINALWNSSQCFKTKKNGYKS<br>PAECFIPDPEWTCLLSVFDCFPLIDDDFYGSRIFAYKGELKQIGVKLQLEEAVKMFVSTFKQKAISSGLTRCTASSLLSCYK<br>KLMGSLYKYPEELMKSFKQFQWLHTKLGDFRAPKDCILFDSEWEPLRLIANLPFIDDGPNWYGKSIHEFRKELESLGVTVE<br>LRKGMSHVISSLSLPDPSRIAPSSALSLFRCIKFLREDRFQQLPKELLDKVSVKWLKTHAGYRSPEECLLFDRTWKLEPCD<br>GPFIDEEYYGSDINSFREELIAIGVGHDSDKACQLLARNVYKLSETDAISRVYRFLSEAEWKPEKGASSGRIWIPSDEKWA<br>DISSCVLFDKDKLFGSKFNVLENHYCSGKDHNLLGFFSSAFGVRINPSIEDYCELWKYWEKTKNRLSSHECCAFWSFVVR<br>HGDTVKAEKLLSESFSRLPVHSPDCNNNDGVMLSSISDVFIADDLLLKDMFIDSPVFVWYPTPSIPTLSRTRLIEIYRNIGVK<br>EVSKCVEIAEADLTGFKTELQEVVDPKKNLIGPGLVKLILAFLSDPSLKVETAERLRIIHSLVDIDVKETSETITTEYTLSLPSK<br>GEKLIAKAKRMIRWEREKGVVYAEKMEKTCGKRKLLEYATCFAEVIAKGVMWEREDLIGRLSELVKMAYLVEFDEEALEFL<br>MKSKNLQVYEEDEKLISDEFSQVN	zf-BED	II
AT4G15020.1	ATZf-BED06_I	MDAELEPVALTPQKQDNAWKHCEIYKYGDRLQMRCLYCRKMFKGGGITRVKEHLAGKKGQGTICDQVPEDVRLFLQQCI<br>DGTVRRQRKRHKSSSEPLSVASLPPIEGDMMVVQPDVNDGFKSPGSSDVVVQNESLLSGRTKQRTYRSKKNAFENGSA<br>SNNVDLIGRDMDNLIPVAISSVKNIVHPSFRDRENTIHMAIGRFLFGIGADFDAVNSVNFQPMIDAIASGGFGVSAPTHDDL<br>RGWILKNCVEEMAKEIDECKAMWKRTGCSILVEELNSDKGFKVLNFLVYCPEKVVFLKSVDASEVLSSADKLFELLSELVE<br>EVGSTNVVQVITKCDDYYVDAGKRLMLVYPSLYWVPCAAHCIDQMLEEFGKLGWISETIEQAQAITRFVYNHSGVLNLMW<br>KFTSGNDILLPAFSSSATNFATLGRIAELKSNLQAMVTSAEWNECSYSEEPSGLVMNALTDEAFWKAVALVNHLTSPLLRA<br>LRIVCSEKRPAMGYVYAALYRAKDAIKTHLVNREDYIIYWKIIDRWWEQQQHIPLLAAGFFLNPKLFYNTNEEIRSELILSVL<br>DCIERLVPDDKIQDKIIKELTSYKTAGGVFGRNLAIRARDTMLPAEWWSTYGESCLNLSRFAIRILSQTCSSSVSCRRNQIP<br>VEHIYQSKNSIEQKRLSDLVFVQYNMRLRQLGPGSGDDTLDPLSHNRIDVLKEWVSGDQACVEGNGSADWKSLESIHRN<br>QVAPIIDDTEDLGSGFDDIEIFKVEKEVRDEGYYTNTSEKLFT	zf-BED--DUF-domain--Dimer_Tnp_hAT--	I
ATR0598G059.1	ATRZf-BED01_I	MDAKPMMDIALLPSIDAIDIGLGSSEKGNVGPAGKPKKKSMTSFYLKFFETAPDGKSRRCKFCKQNYSIATATGNLGRHLS<br>HRHPGYDRQGDFVPQAPQAIPFNKKPSQPNVKSTNSVDNDHLSWLLLKWVINGPLPFSTFEDEGLADSFKFINSSTRFW<br>SKARAHSVLLEVFRSMREDVKAALDHVNCKVSITLDYWTNYEQVPYMSITGHWIDENWSLRKVLLDITHIPYPHGGTEIYH<br>SMLKVLESYNISGRVLACTHDNNQNVIIACRMLKDYLDGMKEPFTYIQCAAQTLNLIMEDGLRYVKPAIAKIRECVLEMNTS<br>VEIAQDFREMASACQEGSWNFPLDVSTRPSGHYTMLDVATKANTAMDAVIMKHEKMLGRNRMTTMEKNAVEKTRRYLD<br>SFFKTTNNLCGSELPTIGLVFFFMDHIMEMIKSCRESRYDPDWLKGAAVDMANKALIYSNQVYNLYTFISAILDPRIKKEFVP<br>VDLNTDLNQEAARNHFMMNYASGHFSSIPNGYTNPQDRDGGVQNVSFAEEIARKRRRVSMNNATDELTQYLSEPPAPL<br>SNTDVLDWWRGNSARFPKLSAMARDYLAVQSTAVPPDLVFSAAGDAVEKQRTSLSHDSVQAVMCIRSWVQNGFKFKFR<br>SNEIDYEKLVEPVVQDAPLFDRKTK	zf-BED--DUF-domain--Dimer_Tnp_hAT--	I

ATR0626G282.1	ATRZf-BED02_V	MSVNKVPDIAWDHCTLLGGKNRKVKCNYCGKGVTGTNRLKYHLACIRGDVMPCERVPLQVKTLMETSILERLRQRLTSEIGPEDQNFPWSRDLRKLYDKLQSKSEESKATKSTTNKKGKVDIENKLKRKMEIFNPVSLALQGSDDKWRHACRSIGRFFFESGVEISASESPAFQSMIDAIISCGFGFKSPSASELRGWILKEEVREVNERLKEVLLDDAKSITRFIYNKAQVLKMTRLHTKGRELVQPAKTGIVGCFLTLQMDFFSDAEVSSGLIECIDQMIMDLHLQDMVLQQLEEYKAAKDGFAEGMVISQRNECLPAEWWSSCGGHCPNLQRVAIRILSQTCTTSKCKLDWSLMRQLHSGKRNRIEQQRLADLVSVHYNLQLNNIKSPLRSYRPTTEMELNQMSDWLVGFQDHGETSNWMDLEFEETEVADKACDQGLPIFQPKEELDLEDYKNQGVS	zf-BED--Dimer_Tnp_hAT--	V
Bpev01.c0066.g0064.m0002	BpeZf-BED02_III	MIEFMARSCGIVDPGWEHGIAQDEKKKKVKCNYCGKIVSGGIYRLKQHLARLSGEVTYCEKAPEEVCLRMKENLEECRSNKKARQSEDVGQVNLNFHANNDEEEVHVRYRSKGKQLMVDRNPSSDINLAANLTPLRSLGYVDPGWEHGVAQDERKKKVKCNYCEKIVSGGINRFKQHLARIPGEVAPCKHAPEEVYLTIKENMKWHRTGRRHRQPDNSELSAFFMLADNENEEDEKEEVALHHSLIDGDRRLSKDLRKAVKGMSPNSGSEPSFKRSRLDSLFLKTPKTHTPQSYKQVKLNTGSNKKSGDEVFSAICKFFYYAGVPSEAADSIYFHKMLDLVGQYGQGLACPRNQLISDQSLQEEIATIKNYVVEYKASWAITGCSILADSWRDMQGRTLINFLVSCPDSVYFVTSVDATDVVEDASNLFKLLDKVVEEIGEENVVQVITEDTPSYKAAGKMLEEKRRKLFWTPCATYCIDRMLQDFLKIRCVGECMEKGQRITKVIYNQIWLLNLMKSEFTGGEELLRPAITRSASNFATLQSLQEHKVDLRKMFQSNKWISSRFSKSSLGKEVENIVLNATFWKKVQYVSKSVEPILQVLQEVDNGESLSMSSIYNDMYRAKLEIKSIHGDDARKYGPFWNAIDRHWSSLFYHPLYMAAYFLNPSYRYHPDFMAHSEVMRGLNECITRLEPDTMRRVSSSMQISDYNSAKADFGTELAISTRTELDPAAWWQQHGISCLELQRIAVRILSQTCSSFGCEHNWSIFDQIYSQRHNRLAQQRLDDLIYVHYNLRLRERQSRERFNEFSLDNVLVECLLDEWIVEAEKQSLQENEEILYNGTKQLDAYENDLVDYQDGTAEARKGCLELATMPDIKPLDVNPANASAASDDDDADIDFLDDDLSD	zf-BED--zf-BED--DUF-domain--Dimer_Tnp_hAT--	III
Bpev01.c0074.g0013.m0001	BpeZf-BED03_I	MVRGRDACWEHCVLVDATRQKVRCNYCQREFSGGVYRMKFHLAQIKNKDIIPCPDVPADVRDHIQSILSTPKKQKTPKKTKVDRAAAANGQQNSSSASGGFHPNHVSSGQNGSTCPSLLFPRPSPSEQPAVDDAQKQKQDDADKKVAVFFFHNSIPFGAAKSMYYQEMVNAIAECGVGYKAPSYEKLKYTLLDKVKGDIHDCYKKYRDEWKETGCTILCDSWSDGRSKSIVVFSVTCPKGTLFLKSVDISGHEDDAAHLFELLESVVSEVGVENVVQVITDTSASSVFAGRLLMSKYGSLFWSPCASYCVNKMLEDIGKQEWVATVLEEAKSITRYIYSNPWTLNMMRKFTCGSDLIRPRIARFVTNFLSLRSIVIQEDNLKHMFSHAEWLSSVYSKHPDAQAMKSLLYLDRFWKYAHEAVSVSEPLVKILRIVDGDMPAMGYMFEAIERAKVSIKTYYKGIGENYMPYWDIIDRRWNMQLHSSLHSAAAFLNPSIFYNPSLKTDFRMRNGFQEAMLKMAATEKEKLEITKEHPTYINSQGALGTDFAIMGRTVNAPGDWWAGYGYEIPTIQRAAIRILSQPCSSHWCRWNWNTFESMHSKKRNRVDVEKLNDMVFVHCNLWLHAICDGRDGKCKPIIFDEIDVTSEWPTESAESSAPLLDDSWLDTPLECRASP	zf-BED--DUF-domain--Dimer_Tnp_hAT--	I
Bpev01.c0129.g0012.m0001	BpeZf-BED04_XXII	MQGDEARALLGFPTDSRPTLSQVKAAYRRKVWESHPDLFPAQEKPHAESKFKSISEAYSCLLSGNSPISKKDQKVQQVMLSLAQAIHSRILKKIHKPTGYSGACKGRFALGKEPNQEKRVKFLVPSTCKMVEEMNPLGSSGYVDPGWEHGSAQDERKKKVKCNYCGKVVSGGIFRLKQHLARLTGEVTHCEKVPEDVCLRMRKNLEGCRSGRKQRQSEYEQGSLTFHSNEYNYAGEASVGYKQKGKKLVIDKKLFRGFSPFRALGCGDPGWEHCIPQDERKKRVKCNYCEKIISGGINRFKQHLARIPGEVAYCEKVPEDVYIKIKDNMKWHRTGRRPRKTDINTVSTCYLHSDTENEEEEDEGFLQCISKDILAIEDKVSDSDIRNNVKGRSPGTSVNGAEPKRSRLDSIFLKSLKYQTSPLFKQVKAKMICEKKTRKEVISAICKFFYHAGIPSNAANSPYFHKMLELVGQYGQGLKGPFSRLISGQFLQDEITTLEGYLTEFKASWAVTGCSIVADSWKDVQGRTLINFLVSCPRGLYFVSSTDATDMIEDAANLFKLFDKVVEEIGEENVVQVISKNTASFKTAGKMLEAKRRNLFWMPCAVYCIDQMLEDFLNIKWVGECIDKAKKITRFIYNSAWFVNFMKKEFTKGQELLRPSVTKFATSFFTLQSLLDQRIVLKRMFQSNNWLSSQLAKSDEGKEVEKIVLNVTFWKKMQYVKKSLEPIAQALQKIDSDETRSISFIYIDMYRAKLAIKAIHGDDARKYGPFWNVIDNHWNLLFHHPLCVAAYFLNPSYRYRPDFTMHPEVIRGLNECIVRLEPDSGKRISASMQIPDFVSAKADFGTDLAISTRTELDPAAWWQQHGISCLELQRIAIRILSQTCSSLGCEHTWSIYDQIHRIRRNCLSRKRWNELTYVHYNMRLRERQISKPDDLISFDSAMLESILDDWIVETEKQALQEDEEILYNEMEQLYGDEMDENENEENKHAEEVTSVGVVELLDVNPAAGVVITDDDALDFIADHLTD	DnaJ--zf-BED--zf-BED--DUF-domain--Dimer_Tnp_hAT--	XXII
Bpev01.c0167.g0005.m0001	BpeZf-BED05_I	MEDNFSMAPTGCLVPIHVEDHNNPATPSPLNAQNPTPSLPPRDKKSKRKAINQSIAWDHFTKVEPVDSDNPKAKCNYCSRLFGCHRRLGTSAMLSHVQFGCQKSPLKHQRLEKNQTLLQLGLKKGADGNVSSQIGFMKYDPDRVRMSIVRYLIKCELPFKHVDTEGFQEFVNNLEPRFIMPSRVTIQRDCMNLYREEKEKLKDLLTGQRVCLTTDTWTSLQNLNYMCLTAHYIDCNWKLHKKILKFCLVPNLKGETIGRILETALLEWEIDKVFTITVDNASSNDVAIDYMRMTIKDKDCTVLGGEFLHMRCVAHILNLVVTDGLKDVYDSITNVRNAVRYVKSSSARLSKFKEYVKREKIQSKKMVCLDVCARWNSTYLMLSIAEKFQRAFELMGEEDNELPVPGYLDWKNARIFAKFLKIFYDAILSLSGSLYVTSNMYFQQLCIIQNTLNDMCVSDDPVISVMAANMKSKYNKYWGSMDRINLMLYVAFVLDPRFKMKALVFWLKRCNGNEWSDNIEAKVRHLMNRLIEQYHKFHRDTSRVFDVAQRSLNATFLNIIDDDSSQAMDKFTSLFTQHLEEENGLECRSEVNRYLLDGCEASTPQFDVLAWWKINSVKYPILAEIALDVLAIPISTVASESAFSTRGRILDPFKSSLSPLTVQALICTQNWIRSKPIDIRELEELVESDDDDQGKCL	zf-BED--DUF-domain--Dimer_Tnp_hAT--	I
Bpev01.c0429.g0022.m0001	BpeZf-BED06_II	MDCTASPAVDSTPINDIKEDEPLSPMVVTQASSTANVPRKSAVPHPYSSKRKSSKVWDHFTIIEGCGANDPKATCNYCSKVYNCHSKRHGTSSMIFHLNGCKKYLDGRETLDKSQTTLNFERKPEGPSDKSQTTLSFERKPDGALRLSFVRKTG	zf-BED	II
Bpev01.c0502.g0006.m0001	BpeZf-BED07_I	MDLSDAVIVNSSRLKSVVWNDFDRIKKGDTCVAVCRHCKKKLSGSSTSGTSHLRNHLIRCQRRSNLGIPQYIAAREKKKAGTHALANFNMEQEQRKDEMLNLVNVRFEQEPIKDEAANTGNSNFDQRRSRFDLARMIILHGYPLAMVEHFGFRVFVKNLQPLFELVTFDRVEADCMEIYVKEKQKVNEVLDKLPGKISLSADMWAAYGNAKYLCLTAHYIDESWQLKKKILNFIMVDPSHTEDMHSDVIMTCLMDWDIDRKLFSMTFDSCSANDNIVCRIRDRLSQNRFLYCNGQFFDVRCVANVINLMVQDALEALCDVTKKIRESIRCVKSRQVMQTRFNEVAQEVKVESQKSLCLDNSLRWSSTHVMLEVALEYREVFCFLQENDPVYTMCPSNIEWERVSVVTSFLKLFVEVTNVLTRCKSSTANIYFPELCDVHLQLIEWCKNPDACISSVALKMRSKFEDYWERCSSGLAVAAMLDPRFKMKLVEYYYPQIYGSSAPERIDDVFHCVKELYNEHAICSPLASLDQGLAWQVGGGAGSSPASRDRLMGFDKFLNETSHSEGSKSDLDKYLEEPLFPRNVDFNILNWWKVHTPRYPILSMMARNVLAMPMSKFASEFAFSTEPRLLHSDWTSLTPPTVQALMCSQDWIQSELES	zf-BED--DUF-domain--Dimer_Tnp_hAT--	I
Bpev01.c0505.g0005.m0001	BpeZf-BED08_I	MVNIQEKQNNFYILMVREKDVCWEYAEKLEGNKVKCKFCQRVLNGGISRLKHHLSRLPSKGVNPCTKVRDDVTDRVRAIIAQKEDTKENSCVTKHKLTEARSTGNISAVKAPMLLDAASPISKVFPSVTPMAPPSLNNQENAEKSIALFFFENKLDFSVARSSSYQLMIEAIAKCGPGFTGPSAEMLKTAWLERIKSEVSLQSKDVEKEWSTTGCTIIADTWTDNKSRALINFLVSSPSRTFFHKSVDASSYFKNTKCLADLFDSVIQDFGPENVVQIIMDSSFNYTGIANHILQNYKTIFVSPCASQCLNLILEEFSKVDWVNRCILQAQTITKFIYNNASMLDLMKQFTGGQELIKTGITKSISNFLSLHSLLKQRPRSKLMFNSPEYSTNSSYTNKPQSITCISIVEDNDFWRAVEESVAISEPFLKVLREVSGGKPAVGSIYELMTRAKESIRTYYIMDENKCKTFLDIVDRKWRNQLHSPLHAAAAFLNPSIQYNPEMKFVPSMKEDLYKVLEILLPLPDMRRDITNQICTFTKVRGMFACNLAKEARDTVSPGLWWEQYGDSAPVLQRVAIRILSQVCSSFTFERHWNTFQQIHSEKRNKIDKETLNDLVYINYNLKLARQMRTKSLETDPIQFDDIDMTSEWVEESDSPSPTQWLDRFGPALDGSDLNTRQFNAAIFGGSDHIFGL	zf-BED--DUF-domain--Dimer_Tnp_hAT--	I
Bpev01.c1064.g0010.m0001	BpeZf-BED09_I	MLAVERVIRAIHTAVDAPRLTRFTLHAPKLVEVGFADGSAFNLSAEFLRVHSPAVDGKIRSIGGEKVISGRRHVGIMSAEPVGNYGGVLSSRYDLPTRILIVKTSKFRSPLKKPAAISPIGSPENSSIPIRLCISSDPLLLGPLVVSFSSSGPDVSDEDHDISAREFCTGIRKFSEGGRGRGKNLKLIDEISSGTVADEEGGEKSGSAGCSSQLVVEAEIKALEEGKLVDVMTEESLGVFVPVAVLIPTNCNDSTEDGKLEKLMKESAMKLGEKVMVKPPVEAIDRVLLAGIQVLWVNFWISRPLSSWSRRQWIKCGAVMSKLLHLPFECCDNECLDFIASGIENPLFAYSVTEEQQRLGFAKVLAEVDVDSALPKDLVVDVGDGKLITFGVEYPWLPIKCTQCKSFDHSVHACTQQEKQSTKEFIKKPGHSKFEFNLVARADGNVQKAVKEFKKPEFDRVIRRPTGHAKEKVAKDKGRLIKAITCEIEVDQGKKRWIQSFIYGSTRGLDRKALWQHLGFIKMQIAFVSWLIGALLGLAPPRASDPGWAHGIMVNGGRQKIKCKYCHKIMLGGGISRLKQHLAGERGNVAPCEEVPEEVRVQMQQHLGFKVLERLKRQKGMKSSKDSMSYIQGREEGDDGDVVQNKNMVSARGTSKRRGKEVVERISNRSKRQKKQYFSSVVPIVAEPIHQSFASQESMDQADMAVARFLYEAGIQFTAANSQYFQEMADAIAAVGPGYKMPSYHSLRGKLLNRSVQDVSEYVEELRKSWEVTGCSVMVDRWMDRTSRTVINFFVYCPKGTMFLKSVDASEITSPEALLNLFDGVVQEVGQKNIVNFLTDTSPSYKAAGKLLMEKYRTFFCTTCGVHCIDLMLEDIGKMDVIKEVLAKAKQVTQFIYNNAWVLILMRKKTSGRDIIQLATTRFASIFLTLQNMLSLKSHLHRMFTGSAWMHSTFSKERAGLEVAEVIVDPLFWSMCDQTLKVTKPLLSVLQLMDCGEKPSVGYIYDAMEKAKKSIIDALNNKESAYLPYLEVIDHIWQEEFHSSLHAAAYYLNPSIFYNPSFSSNKVIQKGLLDCIETLEPDLTAQVMITSNVNFYEEAVGDFGRPVALHSRDSLSPATWWSLYAADYPDLQRLAVRILSQTCSIARCDRSWSMFERIHLKKRNRLEHQRLNDLIFVHYNLHLQERRPEASKARIRRGTFDPLCLEAIDANMGDWVEDPGVLEGEDVSWIDVTVPSEPTFEIHKVKDLDDCNDDSTDDRGSDDMRGMDENDDL	zf-BED--DUF-domain--Dimer_Tnp_hAT--	I
Bpev01.c1348.g000	BpeZf-BED	MDGNSNSAAKNIGSIASASASSGPILIDSTMPPINNENVVCVGSGPTTNVGTQNPSSSLPPKPKKIEQTSIVWEHFTKIEGGDPEDPKSKCNYCSKIFSCHTRKQGTSSMLSHLKNSCKKYPGRFGKLDKSQTQLSFDAKKEGQVGEGSVGNLVIAKYNARKIRVAIAKMIILDELPFKFVEGEGFQDFMKTVESRFQIPSRCTVMKDCMKLFLSEKEKLRSMFLTTSPRVCLTTDTWTSVQNLNYMCITGHFIDSDWNLHKRILNFSLVPNHKGETIGKKVESCMLEWGITSIFTITVDNASSNDTAI	zf-BED	II

1.m0001	10_II			
Bpev01.c1420.g0003.m0002	BpeZf-BED11_I	MEISNDSAVKKPKRLTSVVWNHFERVRKADICYAVCIHCTKKLSGSSNSGTTHLRNHLMRCLKRSSTIDVSQLLAAKRRK<br>KDSTLSLTNITFDEGQRKDDYIKPTILKFDQDQKKEEIFNLGSSKFDQERSRLDLARMIILHGYPLAMVDHVGFKVFVKNLQ<br>PMFEVVPNNAVEQACMEIYGKEKLKVCETISKLHGRINLCVEMWSSPENLEYLCLTAHYIDEDWKLQKKILNFLTLDSSHT<br>EDMLSGVINKCLMDWDVDGKLFALTFDDCSTDDDIVKRIKEQISQKRPLLSNGQLFDVRSAAHVLTSIVQDAMEALQQVT<br>QKIRGSVKYVRSSQVTQGKFNEIAQQVGINSKKNLLLDCPIRWNSTYLMLETALEYRGAFSLLQDQDPSYTSALTDTEWE<br>WASSVTGYLKLFVEITNVFSSNKCPTANMYFPEICDVHIQLIEWCKSPDNFLSTMALKMKAKFDKYWSKCSLALAVAAILD<br>PRFKMKLVEYYYSQIYGSTALDRIKEVSDGIKELFNAYSICSTMVDQGSALPGSSLPSTSNDTRDRLRGFDKFLHETSLSQ<br>NAISDLDKYLEEPVFPRNCDFNILNWWKVHMPRYPILSMMARDVLGTPMSTVAPELAFSTGGRVLDNCRSSLNADTLQAL<br>ICTQDWMRMESRDNYAFRRDLHSLFLSSKKSCSSVDDALDIDDMLGVLPYIIRKRGGIGVVMVILMRKLVECQPACKLGN<br>SPMLAVPLMERSIVLYWKPCLLVMNMFLLRKYLLVFMILSLWSVVLNKF	zf-BED--DUF-domain--Dimer_Tnp_hAT--	I
Bpev01.c1974.g0006.m0001	BpeZf-BED12_I	MDWTANNTFKTFKDMEPKSVMDIAIIPNIGPIDIALSSSEKANANTSAKPRKKTMTSVYLKFFETAPDGKSRRCKFCGQSY<br>SIATATGNLGRHLSNRHPGYDKSGDVVSNSAPQPITVIRKSQPQGKAPQVDYDHLNWLLIKWLIVASLPPSTLEEKWLANS<br>YKFLNPSIQLWPGEKYKAVFREVFRSMQEDVRASLEQVSSKVSITLDFWTSYEQIYYMSVTCQWIDENWSFQKVLLDICHI<br>PHPCGDTEIYHSLVKVLKMYNIESRVLSCTHDNSQTAIHACHSLKEGLDGQKGGPFCYIPCAARTLNLIIDDGLRTTKPVIS<br>KIREFVLELNASSEMSDDFIEFTTAYQEGSWKFPLDASARWSGNYQMLDIVRKASKSMDAVIRKYEETLGNNRMLSSAEK<br>NAVSIVHQYLEPFYKTTTNICTNKVPTIGLVLFFMDHISETITVCRESRSPDWLKNAAEDMAIKARNYNNQVCNIFTYMTAIL<br>DPRIKGELIPESLSSGNYLEEARTHFMRNYSTSHFASMTSGYSAPEIEDGGSVSFAEEIARKKRRASMSTTTDELTQYLSE<br>TPAPIPTDVLEWWKVNSTRYPRLSLMARDFLTVQATSVAAEELFCSKGDEIDKQRICMPHDTAQALLCIRSWTHGGIKLKF<br>KSTEIDYERLMELAAAAAADNSNAGSDKKQK	zf-BED--DUF-domain--Dimer_Tnp_hAT--	I
Bradi2g25117.1	BraZf-BED01_II	TGLGWYRSKRGLDLVSPTSAFAPDWSPLSSRSTAPTSPPPATTLTTAPPCSLRPILPPLPRRSGGTEPDLSFGAWILKQP<br>LSNLPTRIRRPHLLRTRLLRGIGSDSCGLLPHDKPAETPISSSYGDLACKHLNNMSENYGTSNFADGTLVLPNARRSKAW<br>KHFEPNLVFVDGIRKAVCLYCGMKLTNTQKSGISSLINHVSKSCPKITAEDRNFFIASLKKKQAVVGYSRTSDIDMVQIFVC<br>NIYLCASENLQKLGQTGAGDYSLCYLRFQFLELKHLVIQVNMLQPHKFKGVKRL	zf-BED	II
Bradi2g40703.2	BraZf-BED03_VI	MDVDKDKRNGKVHLRDSSSQDALRTYKRRRQQVQTQEEPQPQHGQEQEPQQQQQPQQEQEPQPQPQPQPQPQPEP<br>KPNTVDVPEQQVTDSFWKSRDIGWKHGIMIDENRQHWKCMYCHLTRYGGGVSRLKRHLAGDLDVKMCPKVPPDVAEKI<br>REHLQKKRERRKKKAARKGDKVKTRSFANDTDAEKDPLPVDLELPTQIDTSVLEEVTNQTIEDHQEPTLLRAPLLLRGVRD<br>IGWEHAVDLDGNKRRWQCKWCDLCRSGGVTTLKAHLTDGSCPKIPMEMSKKVLNFLEEKRAARHLFNSNPWPAELKKN<br>DGPSVFSPKSEEEGTVSCKNDQQASRNPMHMQISEKCTTNELPAQSNQCGTEHSGQLVKEKIAAAHHLSNSNMSADVL<br>QSDCHSIFSTQLEEEGSVPCNKDHQPSKNYMHMHTSEKSAMNEMAGRSNQCDTEHSGQPLEDYDRSKISDQPDEQGPI<br>EHGRHHVLNNEHPVVDKNTENSGNKEILKHPRKTRFNIRKHIITIDEIARHWRCRYCGMDGHGKTSRLHYHLAGAFRHPK<br>CPSVPPEVFAKAKHHILIKRRLRMKKSGQQASPEPKIIGQSSVILQNNNPAFGNPPQLPIHDLSSEVHSNYPTRLRDNAWE<br>HSLIHDREKGHWKCKWCSLEGYHGITRLKWHLVGWKNRPQCRNIPEDSAKRIRDQMISREKKKGKRSGPHDGIDSCDM<br>LCSSKDLQPDQEDSAIAIQSRSSSQAFGEANRASNGCSTLSNTMPDPQSIMEMERPHKTLCSSTNKSEKQTGRSDCWTH<br>WKYVLNGLMLLPGVQEGDGIQSCIRDVLYGCAESGTVGDKVEMHSDITVSSNVNIAECKNVLVDILRSEIFALLCNVLRKA<br>VHQDEERTSYFDFGMIDSRMKNGDYGRAPGLFKDDLKLLWEDVKLAGQDIVDLANGLSSLTEASYTKKVGRERGSCDSE<br>KELKGAAVASSEPKNLMESSTLVPSTSQGFQQLDQPDPIDVSDVQMGTACDQCGKEAGGVNTITCNRCKLVCHISCIEPP<br>VPTGSWHCKSCSSTTWDESAEGGMSIVQYEPNLLHGNCVACKGLEVCRPAECKETVSERTPVDESRAIVISCTEPAEGE<br>ELPNIVVGGSCKMCGTPEDDDKRFLICGHSHCPYKYYHIRCLKSKQIASKVQRDKPCWYCPSCLCRVCLSDGDDEQTILC<br>DGCDEAYHLYCMTPRRTSVPKGKWYCSSCSVERAKAGMRQYEKKMLKLHRKDDARLPDRNFAAVDLLLSAAEKLSADE<br>DQLVSRPDQ	zf-BED--PHD--	VI
Bradi2g57020.1	BraZf-BED07_VI	MNAEQSNGKEKMHPRESTPEESLVHTCKRPRPPVSDPKPQDVPEQQNPHGYWKMRDKKGWEHCIMIDENRQHWKCK<br>YCYMVGYSGGVSKLKRHLIGYRGVKKCPAVPAQVCVEIKHLMDAKKERRQKLAALKGGNNVESRSFSGDDKDKMDLLA<br>PDSDALTGVNTHFLEEATNETNAELQNHVQERAIVTSSERKDLVVPSTSYGLISDVQMICSPTLKKSVEGPMVLAHEETSC<br>SHGNIVASKKPEVCKPPTYLESVSEEEQAESSRAIGVSGTKPTEVLTLSNISTTGCCKICGGSEEYNKRFLVCGDTLCGSK<br>YYHIRCLNFNQIASDAKRDKPCWYCPSCLCRVCHSNTDDDQIILCDDCDEGYHLYCLTPPYTSVPEGEWYCPFCTMERE<br>REGTEYGKILLKLHRKDDPMT	zf-BED--PHD--	VI
Bradi3g14893.1	BraZf-BED08_II	MGDEAEFAADQITQTKTSDASKKRRSESTVWEEFTKLSNGQQAECKHCLKRLSGKSTGGTSHLSRHLKICPARPGNTSK<br>QPQAPEAPSRPTVSNLCSFPIMSSAGFRQALAGIYHPTSSMIPQNVTEGQFITMFRNERLKLKDKIASAPGGVFLSVRRW<br>DLEESIFECLAVHFIDAEWKIHRRIIRYRLSMLGHADPGHYISIFPIRQNSSHPISVLSKETVVEYSLMDKLLGIALEHLTDVTI<br>TGRLENILTGKNYLLAKCKLITMQCSVDVLHRIFGYELNDVILETGQKWLSYMTCSARNINNFFRTCVYPDPLWLR	zf-BED	II
Bradi3g42745.2	BraZf-BED10_II	MSQFPWGAPLQQDPLPMEVDSSTGGWSWQLELAHPQITLPQQQDSLPMSIDPLGGGGGEDEEEWISLLRSCPLRSPPR<br>SLLPFSPASPPDIWVTPWDPYTWIPPCSHQIEQAVALIFARLHRQLRRLKMQMEIPLRRPKRSVIIQYNDDDDNTYDVLTVV<br>RKPYGPNDFLKHFKSKKDGELKYCIHCNDVFPSDTDLFDLSQNLRSVSRISIQEVYPPPKSSGGIPDMETALKLTEGSSEG<br>NILLSGNEKPKEIDAQKSASRHVESPVAFDQSNTPPERMLVDNGEAAASTDASWIDAVQNCFEETTKADKPKGTLKKKW<br>SAVWEHFVETIIDDTVWADCKHCKKRFAGGTKKAGTSTLRRHLDKLHGIETPKASSSSKNENLCGVPATSKSKTPTPSLL<br>PSLEPVASEPPGKTTQRKKTTDGVNDLATQEVCARGKRTRRRDNPSSSGVTPSEVPSSASAAPPPTVGHAAEATSTGM<br>VVASSQIALSAAGASGEVVRSIADTIINPGPKDLTNAILLMSQAAEAVAREKMLLQEMEKLRQENTDQATAVHSRLEALEAK<br>LVLAADENKKLKAENTALRSQPQESPHSRPASPPSKRS	zf-BED	II
Bradi4g03620.1	BraZf-BED12_XXII	MSSQIPILIPARKCASHPPPKSHLCSPLLPLPGRISPHQGAMGQEGSFEVGDKVFSRSILWIGVGYFDDFASTPLDWGFPI<br>DPLSHHPSPPPPPSPSHHMNIDLPQSSVAAEFQPPPADGDLVSTPSDAGFPIDQLWSSPPLPSYQMNVDPPKSPVAVEL<br>QPPPGGDLVPTPVRHVPPPSPSRHMNIDLPQSSLAELSSDAGFPIDQLSSPPPLPSYHMNIDLPRSSLAAELQPPSSGDS<br>VSSTVRPVPPPLPGQTDRPRSAIEEELQAWRLPDLPDVTDVELLLKHLDPACERLPRKYISQPRRRAVVNADLPVKNPQE<br>LPGVDRGEGRIGIHYLTDNLKLCFAQNTQLLENGQRKKFTLETPDGYWTLRDSAGERNDIWCIVGFKKTFEFRTKSGTKT<br>KWIMNVYSRVNGSYFVEDDLALCHVFEQDSPGYDPRPKCPTFHEGRCDGLSKVTVHHYQDQRSCPRFIPFTPNPGFLV<br>GDPDPDTSTQRGVPPTADHAESKKRKVGSDVWEYFTKIFARDIKGNVLTFAVCNHCSKVLTGGSSGGTTHLKNHTCACK<br>LKPVLAGRNQKASVSVSSS	NAM--zf-BED--	XXII
Bradi5g22179.1	BraZf-BED13_XII	MEPAEVDSLEAAIGWLVRTIIAVLLTDKMDVWIRGVKLDDDVKKLRFEIRRVGMVVATVKGRATGNEQLGESLAILKELLYD<br>ADDVVDELDYYRLQQQVEGDTVTRDDEISRGDADTPNSSVGKLRSAVWQHFIITEKIEGRPVRAKCSLCSEEFACDSVAN<br>GTSSMRKHLKNAHSVICENGKRSRNHSSTGDDATENATPIVIGSSSRGKRKRTNEDSAQITAANKRTHWDKAEISNRIKEI<br>TSQLKDIREEVSEVLKLHGSDLASSSNNHRSTASDQHLRTSSLVQRKVYGRDGEKNNIIKMITQDKSNGVTVLPIVGIAGV<br>GKTTLAQLVYNDPIVESKFDTKIWIWVSRNFDKVKLTREMLHFVSQETYEEINCYVKLQEILKSNANSKRLLLILDDVWDDM<br>DDCRWNQLLAPIKSDSANDNNVILVTTRNMTVAKMIRTVGPIQLGALENDDSWLLFKSCAFRDWNYEGPEILSSIGRQIAD<br>KLKGNPLAVVTAGALLRDHLTVDHWSNILKKENWKSLGLSGGIMPALKLSYDQLPYHLQKCFSYCSIFPDKYKFLGKDLRL<br>EETGWEYLIDLANLGFFQQILKEEEEDDDNEDGEGEGEESSLGSQIFYSMCDVMHDFARIISRTECATIDGLDCDKILPTIQ<br>HLSIVTDSAYNKDWQGNIPRNTMFEENLRNKVTSVRKLRTLVLLGDYDSYFLQLFHEIFQTAHNLRLLHTSATSADFNSSR<br>CSLVNTTHLRYLQHGSGKVNESFAHDLSKLYHLQVLDVRSDTYSPTIPDGINNLVSLRHLVLHKGVYPFIASIGKLTSLQEL<br>YGFKVQNSSGFEITQLQSMNGLVQLEVSQLDTVQTREEAYGAGLRNKGHLENLHLSWKDTLSDNESPADTARKHSEVLE<br>GLQPHKNLKDLQISGYSGTSPTWLASNMSATSLQTLHLDCCGEWKILPSLERFPCLRMLKLSNLWKVTEILVPSLEELVLV<br>RMPKLQRCTCTSVEGLNSSLRTLRIKNCEKLEVFDLFENGDKFIIEQRSWLSGVRELILIDCPLLGVLNRLPPSATFSELQIS<br>GVSTLPRMKGSYEKLHVLLPRVESFSEKDTSSERKSVGEVLAFHNLRSLKFLSINGYGENSMSIFFKDLSHLVSLKSLKIES<br>CEIVMPEPTREDMPAANCKVFPYLQSLVIKSCGITGKGLSLMLQHSPVLEKLVLFDSSDITLLSIEEEGNSLSNLTSDREPQ<br>DELFLHIPFNLSSSLKKITIEDCPSLRFNGSKKGFSGFTILEKLNIWGCPELLLSLPCFPSNLTSLKKLGLSSAYLKSLQLHSC<br>TALEELEIKPCYLLSIVEGMQSLGSLRHLTVLGCPCLPSYLESFSRQCNELLPRLGTLVIGDPAVLTTLSCKRLTSLHSLQLR<br>LRWMLVTRLTEEQERALVLLKSLQELTFYVCPHLMHLPAGLHTLPSLKRLKIDYCPSILRLPETGLPVSLEELELKCCSNEL<br>RDRCMPPATSKLK	zf-BED--NB-ARC--	XII

Carub v10002471m	Car Zf-BED 01_I	MDSSSPKPADDLKKEDIELDGEEVESPKAGHKKKKQETGSVPKVKPKIKKKCRVVPRSWVWDHFTRLAGVKDRCRCNY<br>CTREMGCATTNGTSCLKNHLNICKEYKIWQECRNQTQQVINPTSDGDGSACLQLSRVSEEVFREATNEMLVLGELPLSFI<br>ESLAWKHFCSKVKLYKPHSRRTSTRDIVEKYVKKKAEMMKIIAANKQRISLTTDIWVAPTTGASYMVITGHFVDANWRLKK<br>MIIGFKHVLDHTGETINSVLLDCLAEWGIRKIFTINVDNATANTNAMKKFKEAFSLVGHDALVLGGQFMHLRCCAHIINLVVK<br>DGLRDLNKSVEAIRTAVQYVRGSSKRVHAFELKVESGKMTRGSLSLDCQTRWNSTFLMLTRALKFRSAFDRMEAEDKLY<br>NDYFSESIDGIKRTGPPTKHDWDEIERLVRFLVIFYNSTLVVSASSNVCSYKCYNEIVTIERNLIALSTNSDEKLRSKANAMR<br>EKFNNVFDPRNKMKFAGLCFDMLYGKDSAESIELNKSVSDVLGKLFEEYSSQSQSQSGATPAQSGQESQCDLSQKMDL<br>DSDLEYLRMDTMYNEMVNEIGFQDASTELELYLKEKVECPKTNQLGIQFDVLGWWRINTPKYPVLALIAKDVLAMQVSSV<br>ASESAFSNSNRILDPSRSCLTHYMLEVLMCSEQWFKGEIQLNEKGVVTTQQMLAEIELQDNLERGKLLTSIICS	zf-BED--DUF-domain--Dimer_Tnp_hAT--	I
Carub v10006642m	Car Zf-BED 02_II	MDSSSPPNKANRDNEEHEQEEVITPTPDQRGKRKQTQENGATSEQPKAKKIIPPRSDVWTHFTRRENRDKCVCHHCKK<br>DYSCPSKSGTKNMWNHLEICSSYMVITAHYIDAQWRLKKLIIGFKYVTDHKGKTIAKVLLECLTDWGIEKVFCVSVDKATAN<br>SNALSLFESSFSLLSEDSLVMKGDFLHMRCAGHIINLIVRDGFNKIDDSVTAVRNAILYFRSGGRRQEAFDLKVDSGRMTR<br>GSLPLDVKTRWNSTYLMLLRAVKYKVAFDKMELEDKLYNDHFLEYENGAKRIGPPSMADWRAIERLNRFRIIFYDATLVVS<br>ASTTLNAHKCYGEIVNIASELQLLCTNYDSEKPEKPDPNPN	zf-BED	II
Carub v10006956m	Car Zf-BED 03_XX	MDLMNSGTCNVKTEEEMVKNTVFDSSDSEKTDAWKEFVSVGQGGDGRGRCRCIHCGKVLVKPTMTSNLWRHLRCCR<br>MRPKTISGGNEEQDRPIHDQRYSSIDVVGGSVSESNVVGGSVSESNSEREGVRENTHLKKRQRDSTGSGMLLGLNVLD<br>CPICFETLTIPIFQCDNGHLACSSCCPKLSNKCPTCASPVGHKRCTAMEKVIESVLIPCRNTKFGCTKKVSYGKESVHEKE<br>CTFTQCSCPALDCIYTGSYYDIYTHFVDNHSHVSKPMSFVCGGVVDVQMNIANEKILVLWESKKRLLFALQCFTEPDTIHQ<br>TYYRKKPEPRQLSIIEKTDDVEVVESIRVFAKKEGKKKVSDWEGGFAVTEEEGKEGALKSR	zf-BED--Sina--	XX
Carub v10006988m	Car Zf-BED 04_I	MDSSSFPNGEGLDNETADSGKQKAPDDPSGATSQPKKEKEQGERADVWDHFTKHKDNRDFCFCHYCQHNLSCPTKSG<br>TSHLKRHLKTCKAYLAWLDGSQDNTQQQKQINQEGGLKDARVSKAVFDEACDMMLVIGELPLSFIESEAFKNFCSMCNL<br>YQPHSRRTSTRNIVEMYVKRKAALKELFLSNKQRVSLTTDIWVASPTGASYMVITAHYIDTHWRLKKLIIGFKSVTDHKGQT<br>ISNVLLECLAEWGIKKVFCVTVDNATSNSLALRNFEKSFAEISHDSLVLSGEFLHMRCNAHILNLIVNDGLTDVSDSVAAIRN<br>AVTYVRSTTNRLNSFDLKVDAARIKRGSLPMDVKTRWNSTYLMLTKAIKFKAAFQKMYAEDKPYNDYFLELDKGNKKIGP<br>PEMLDWSKVERLEKFLAIFYESTLVVSGSTSVNSFKCYGEIVDICTSLRELSYSFDQELKKNATDMLKKFDKYWEGLKNM<br>NKMLIVATVFDPRKKMKFAELCFDDLFGKETVENKEMQTSVKDILRSLFDEYSLRLEQSTERWDDDLDLVSSRKRRKVES<br>RFTEMQNEKGVKVSTDELDVYLKESCETPDVMHGMEYDVLSWWKVNAHKYPILAEIARDVLAVQVSSVASESAFSTSGR<br>IISPARSSLTHYMVEVLMCLDQWMKQDISLSQKCLQFHKYLLT	zf-BED--DUF-domain--Dimer_Tnp_hAT--	I
Carub v10008481m	Car Zf-BED 05_I	MEWNVNNAFKTYKEMEPKAMMDMTLVPHSDPIDIGLASSDKASTAPPKRKKTMTSVYLKYFETAPDSKTRKCKFCGQSY<br>SIATATGNLGRHLANRHPGYDKATDIVTSSSVPQTPPVVVKPSQSQSKSLQLDYDHLNWLVLKWLALSSLPPSTVDETWL<br>GNSLKFLNPAVQLWPAKKYKAILHEVFRSMREDVKTSLEHIQSKVSVTLCFWSSYQNIFYMSVTGQWIDENWSSHRLLLD<br>ICRIPYPSGVSEIYSSLLKVLKIYAIDDRVLCCTHDNSQNAIHACHSLKEYLDGQKVLPFCYIPCAAQTLNEIIDEGLATIKPIIS<br>KVREFTQELNGSIELSDDFVQLTTAYQEGDWKLPIDASSRWSGNYQMVNILCKAGKSLDSVIRKTENGLENRMILTSAEK<br>NAVTIVHNYLDLDSFHKTTQDMCTNKDLTVGLALLFMDNISEMITTCQKSCHNPDWLRTCAESMAQKARSYNTQVCNVFT<br>YITAILDPRIKTEYIPETINLDSYIDEARTHFIRNYASSHFTSSMTSGYRPQDIDEGGGGNISFAEEIARRKRRGSMSNNVVD<br>ELTQYLSESIVPMQTDVLDWWKVNSGRYPRLSNMARDFLAVQATSAAPEEIFCGKGEEIDKQKYCMPQDSTQSVLCIRS<br>WIEAGMKLKYKSDEIDYERLMEFASTVAGDNTAGGLDKNQRR	zf-BED--DUF-domain--Dimer_Tnp_hAT--	I
Carub v10011179m	Car Zf-BED 06_I	MEDDTNPSDLVDVEVAVVEMDFDTQTNTRRKKKSDVWNHFVTEVVSPDTKKARCMHCNKSFAFMTATKASGTSHLKRH<br>IELGICPKRDEFKNLTPVKEETREVLQTTSYLPKKRRLSSPHVQPPLDQDRCNHEMAKMIIMHDYPLHMVEHPGFLGFLQA<br>LRPQFSVPNFTTIHHDCVSMFLSQKQKILDLIGEIPGGINLTIDLCSSKQSVGYAFLTGHFIDKDWNLTHRLLNVAVVASPDS<br>DFALNQPVAACLSDWKLEGKISSIAVSQSVVNKTSIDNLRGYLSVRNQHALNGQLLMGRCYARLLSSMAQDVLAAEQFQE<br>PIKKVRDSIKYVKTNEVCRDIFDDLKKQFPTPSTYKDLLIDSQTRWDTSYNMLLAACEHRQVFSSLETCHPEYKISLSPEEW<br>RKIESLCSCLKALFEAGNVLARPNQLTANDFYHEMTKLQLELSHTAMCEDPDVRSLANTMREKFDQYWRGCFLVLAVAV<br>VLDPRFKMHLIEFTFNKTYGADAKKWTKTVDDAVHDLYLNYSEQNLLDAYVDHGFSEIEVTQESHLHQDMAEDTNTGNG<br>QSGGEKSPPQLEKATESHQKEGHLKVDKKNVGGVWEGESEKSQLKEEDQQHCDDSQAHAMADEDKTTFPVDVLLVEG<br>NGQSAGETSPQLEKATESHQNEGQLKVDKKNVGGVWEGESEESQLKEEDQQHCDDSQAHAMADEDKTTLPVDVLLGE<br>GNGQSGDETSPQLEKTTESHQHEGHLKVDKQNVVGIWEGVSEESQPTEEDQQHCDDSQAHAMAEEDNTTFPVDVLLE<br>EGSTLITIGESLSDFEMYMSEMKTELDQYLEETLTPRSEDFNVLSWWRLNSTNYPTLSKMAVDLLSVPFTTVSPDSVFEAE<br>VKQMDSYMTSLSRVTLEAILCTKDWLKTGTL	zf-BED--DUF-domain--Dimer_Tnp_hAT--	I
Carub v10012565m	Car Zf-BED 07_V	MNHEADQNHEEEQDINCTGQQAAVTTKVLETPDCSGKRKDIPESSVANAKPAKEFKVILSRSPVWDHFTRNKEDRNKCI<br>CHYYKKEYCRKYKSGTSNLTMHMATCNQYQAYKENKSQQVIKGNGTVQSGKISEPLFREATNEMLVLGELPLSFVDNVA<br>WRHFSSHANLYRPHTKRTASRDIVHIASYLVITTHFIDAFWLLKKLIIGFKYVSNHKASTIATTLLDCLAEWGIQKVFAIIVDIA<br>TTNTLALKMFHRELSLVSNEAFFFDGDYMHVRCSAHIINLIVRDGLQELGKNVKAIRNGVSYVRSSHVRQKSFKLRVDSGK<br>LKRGSLTLDVKTIWNSTYTMLTNAVKYKFSFVKMLLEDRLFHDYFQELDNGNRRVGPPESTEWNAIERLVKFLVIFYNSTL<br>VVSASTNLHAHKCYGEIVTIERNHSFLSSSPDDELKNKADEMLKKFVKYWDGMKNNNKMLIIATICFEKLYGEDTIDSIAML<br>DSVFYVLDSMYKEYAALYSPRNCASSQTTRAKQHHSTQIREKMDLVDDLGYKRMDMAYKEMVADKADEEVRRELKIYLT<br>YPVENPKLIRGTELDVLSWWRVNSFKYPVLAEMVKDVLAMQVSSVASESAFSTSGRILDPHRSCLTHYMIEVLMCTEQW<br>MRQDIKSGADPTVITSAQLLSEFEMFDQLEKGNL	zf-BED--Dimer_Tnp_hAT--	V
Carub v10013143m	Car Zf-BED 09_I	MDESNEIILQKSKRLTSVVWNYFERVRKADVCYAVCIQCNKKLSGSSNSGTTHLRNHLMRCLKRTNHDMSQLLTPKRRK<br>KENPVTVATISFDEGQPKDEYLRPKFDQEQRRDEVVLSRGSGGRFSQERSQVDLARMIIMHGYPLAMVDHVGFKVFARN<br>LQPLFEAVPNSTIEDSCMEIYMREKQRVQHTLNNLYGKINLSVEMWSSRDNANYVCLASHYIDEEWRLHRNVLNFITLDPS<br>HTEDMLSEVIIRCLIEWRLESKLFAVTFDSFSVNEEIVLRIKDHMSQSSQILINGQLFELKSAAHLLNSLVQDCLEAMRDVIQ<br>KIRGSVRYVKSSQSAQVRFNEIAQLAGINSHKILVLDSLVNSNSTYVMLETVLEYKGAFCHLRDHDHGFDSSLTDEEWEW<br>TRYVTGYLKLVFDIASDFSGNKCPTANVYFPEMCDIHIQLIEWCKNQDNFLSSLAASMKAKFDEYWNKCSLVLAIAAILDPR<br>YKMKLVEYYYSKIYGSTALDRIKEVSNGVKELLDAYSMCSAIVGEDSSFSGSGLGRAMDTRDRLKGFDKFLHETSQNQNT<br>TSDLDKYLSEPNFPRSGEFNILNYWKVHTPRYPILSMMARDILGTPISIIAPDSTFNSGTPMIADSQSSLNPDIRQALFCAHD<br>WLSTETEEMLPLSRQYPQTL	zf-BED--DUF-domain--Dimer_Tnp_hAT--	I
Carub v10019720m	Car Zf-BED 11_I	MMIDNNGSLEIDDDQSHMDVTCMALSKDLPNADTDDGLLPEPAKRRRKKSMVWEHFTIETCSPGSTKACCKHCKKSFAY<br>ITGQKLAGTSHLKRHIQLGICPMTRDNTLLPHVLDSKDPTTAPPKKRPRSSATHLNFPLDQDHCYSQMAKMIIMHDYPLHM<br>VEHSGFTGFVQSLRPQFSIPSFNTIHGDCVNIYLSEKQKLSNFISEIPGRVNLTVDFWSSNQSVGYAFVTGHFIDRDWNLT<br>HRLLNVAVVPSPDSDFALNQPISACLSDWNLERRLCSLTVGQSVVNKTSIENLRCCLSARNQHVLNGQLLLGSCYARLLS<br>SMAQDLLGAEDLEAPIKKVRDSIKYVKTKDSCGERFDELKRQLQTPSTKDLLIDYQTRWDTSYNMLVAACEHKEVFSCLG<br>NCDLDYKLSTSPEEWRKIESLCSCLKIIFDAANVLTHSTRLTANDFYHEMTKLQLELSHTAMSEDPDVRNLANPLKEKFDE<br>YWRKCFLLLAVAVVMDPRFKMKLIEFSFSKAYGEDADKWIRSVDDAVHELYLDYAEQSHSLLDAYIGHENDGFSETYMSQ<br>VRFHNEMPREYHQSNGISHEEVYEQSEDGNQLNEKPRDHVEDTDMTQDSQPGEGKVPETQPMEEIVEDTQPVEEVVQ<br>EETSHVSRTVDDKDITHDMQPMDEMLEDTQPVEEVAQEAQQVEKHNITSLEEEGHEMQPVEDLLENTQPVGVVAQEEP<br>LVEEKHNDIQPVAGAGDETQVVEGILEDTQPVEELAQEVQPVEQIPEHSQNLQKPDSILCQEYSREGEEAVQQEQQSDG<br>RTDSDQHPQSDAIPQEEEAHHDSQSHAMPQEEATFTISQEGHHVDVLLQEGHHLEASSQEFPLITIGDGFSDFELYISEV<br>GSHQQMKSELDQYLEESLMPRSQDFEVLGWWSLNRTKYPTLSKMAADVLSLPFCTVSPDSVFDTEVKKMDNYRSSLPH<br>VTLEALFCAKDWFKHGSSSSSSNNNLKRES	zf-BED--DUF-domain--Dimer_Tnp_hAT--	I
Carub v10019846m	Car Zf-BED 12_I	HCLVIKLIYLLQNSILATWSSPLRSLNLFLSSSPGSKERHWSCVFLIFRQSSRGNIEYFLLDPEFCYLFIYAFCYRRQTLLGL<br>CVWQVEADEEHFIPFSMVREKDICWEYAEKLDGNKVKCKFCSRVLNGGISRLKHHLSRLPSKGVNPCAKVRDDVTDRVR<br>SILAAKDDPKDSPLTTNKYKPPEVKPPLSASLLPVTVSSGSKLFPTSILAPPTPNAQVIAERSISLFFFENKIDWCVARSPSY<br>HHMLDAIAKCGPAFFAPSPLSLKTEWLDRVKSEISLQLKDSEKEWVTTGCTIIAEAWTDNKSRALINFSVSSPSRIFFHKSV<br>DASSYFKNTKCLADLFDSVIQDIGQEHIVQIIMDNSFSYTGISNHILQNYGSIFVSPCASQCLSIILEEFSKVDWVNQCISQAQ<br>VISKFVYNNRPVLDLMRKLTGGQDIIRTGVTRSVSNFLSLQSMMKQKARLKHMFNSSEYTTQANKPQSMSCVNILEDNDF<br>WRALEESVAISEPILKVLREVSKGKPAVGSIYELMSKAKESIRTYYIMDENKHKVFSNIVDTKWCDHLHSPLHAAAAFLNPSI<br>QYNPEIKFLTSLKEDFFKVLEKLLPTSDLRRDITNQIFTFTRAKGMFGCNLAMEARDSVSPGLWWEQFGDSAPVLQRVAIR<br>ILSQVCSSYNLERQWSTFQQMHWERRNTIDREILNNLAYVNQNLKLGRMITLETDSISLEDIDMMSEWVEEAENPSPAQW<br>LDRFGSALDGGDLNTRQFGGAIFSANDHNIFGL	zf-BED--DUF-domain--Dimer_Tnp_hAT--	I

Carub v1002 2346m	CarZf-BED 13_I	MDLSDAVIVKSGRLKSVVWNDFDRVRKGETYVAICRHCKKRLSGSSASGTSHLRNHLIRCRRRSNGNGVAQYFGEEERR<br>EIEVANERRKDEEQLSIVNVRYEHEREHDDASAVSTGLDQRRSRFDLARMIILHGYPLTMVEDVGFRMFIRNLQPLFELVA<br>FERVESDCMEIYAKEKHKIFEALDKLPGKISISVDVWSGSDEFLCLAAHYIDEAWELKKRVLNFFVVDPSHTGEMLAEDIIT<br>CLMEWDIDRKLFSMVSSHSPPFGENVANKIRDRLSQNKFLYCNGQLFDVSCGVNVINQMAQDSLQTCCDIINMVRESIKY<br>VKSSESIQERFNQWTVEAEAESERNLFIDDPVRWDTTCTMLENALEQKSAFSLMNEHDPDSVLCPSDLEWDRLETIVEFL<br>KVFVEVTNAFTKSSCLPANMYFPEICDLHLRLIEWSKNPDDFISSLAANMRKKFDDFWDRNNMVLAIATILDPRFKMKLVD<br>YYYPLFYDTNASEFIEDVAEFIKALYDEHSIGSLLASSDQTLDWHNHHHGSNGVPHGKELDDRLIEFDRYINDTTTTTGQD<br>SKSDLEKYLEEPLFPRNSDFDILNWWKVHTPRYPILSMMARNVLAVPMSNVSLEEDAFETSQRRRVSQAWRSLRPSTVQ<br>ALMCAQDWIQSELESS	zf-BED--DUF-domain--Dimer_Tnp_hAT--	I
Carub v1002 2425m	CarZf-BED 14_I	MSDSISTKELDPARKHATPVVGKHGSWMCIYCKKVTSGGVQRAKQHIVGRQDRKEVFGKSDKELRDTACAEIARWFYDA<br>GLPFNAVSFDSFKNFLELVGQYGYGFKPPTMYELMVPFLKKEVKETEKPLVEHKTEWTTNGCSIMSDGWRDLVVQKDIIN<br>FLVNSPRGSVFIKSVDVSTVVKNANLLFDQLDRMVEEVGESDVVQVITDNASNYIKAGKLLLAKRPHLYWLPCAAHCIDLIC<br>NQELHLHKWLHLQSHFSCEYDAEIHKPRNLHRPAVTRFATSFITLSQYHKQRKNLRSSVTFQEWNDSKWSKDIGARNVK<br>KFIMQDMFWHHVLYALKLTGHLVKVLWLVDGEKKPAMGYIYEAMDRAKEAIAMTFNGRDEQYKDAFAIIDKRWNCQLHH<br>PLHAAGYFLNPEFQYKEDSGVNYEEVLKAFYSTIETLIPEIATQDKITTELEQFRNASGLFGIPMWWSSYGGSTPTLRDFAI<br>KVLSLTCSATGCERNWSVFQHLHTKKRNRLAQERLNDMVFVKYNRTLQRRYKRKDTIDPIIFEDIDKIQEDVAQSSLIVITA<br>QVVRSIHHSPSISPLPFQNHQSGPWSIDPRRGVYQSRAEKGKNIASSSTPLNLVDEDDENEIEEDFTGGDEDTEVLVEDD<br>EYDDEYDDKRKYRSFHIPKARSEVRLGSRLKPALNRITSPRAIKNITTTINGIHKRSSNHSHLTPFINCKRWSDGG	zf-BED--DUF-domain--Dimer_Tnp_hAT--	I
Ciclev 10004 311m	CicZf-BED 01_I	MRENLEGCSSSKKPRQDEGGKEAYLTFGSEEEEEEERAGHSSNRGKQLMVDRGVVSNLTPLRSLGCIDPGWEHGVAQ<br>DQRKKKVKCNYCGKVVSGGINRFKQHLARIQGEVAPCKDAPEDVYLKMKENMKWHRSGRRHKWCGTKEISDSHMQSNI<br>EDEVNEQEKDALHRMNSDRKMIGDKRQRKELRVAFNEMSPCRDIESLFNSTRFDSASLKTPCSQTTPSSKQVKIMRGSG<br>KKSQKEIISAICKFFYYAGVPLEAANSQYFHNMLELVAQYGQGLVGPPSQLIFGDLLQEEIETIRNNLAEHRASWAVTGCSV<br>MADSWTDSESRTLINLFVSSPHGLYFLSSVDATDIVEDASNLFKLLDKVVEEMGEENVVQVITQNTPSYKSAGKMLEEKR<br>KNLFWTPCATYCIDKMLDGFLNLKSVWKCMEMAQKITKFIYNHIRLLNLMKKEFTQGQELLMPSVTRFASSFTTLQNLLDH<br>RIDLRRMFQSEIWTSSWFSKSDKGKEVENIVLNAKFWKKVLYVVRSVNPIMKVLQKVDTGQSLSMPYVYNDMYRAKLAIQ<br>SFHGDDVRKYGPFWNVIDNHWDLLGHHPLYVAAYFLNPSYRYRPHFVAYPEVVRGLNECIVRLEPDKLRRIYAMSQISDY<br>TTAKADFGTELAISTRTELEPAAWWQQHGISSLQLQQIAVRILSQTCSSIGCEHKWSIYDQVHGQRHSRAAQKRINDLTYV<br>HYNLRLRECHIKRRSDDSISLEGALREHLLDDWIIEAEKQALPEDEEIFCDERGAAYDDDEYKNDLIENEDGNAEARNGST<br>EILTSSELLPLDVHPVKAGLATDDGADMNFSEDEESE	zf-BED--DUF-domain--Dimer_Tnp_hAT--	I
Ciclev 10004 312m	CicZf-BED 02_I	MRENLEGCSSSKKPRQDEGGKEAYLTFGSEEEEEEERAGHSSNRGKQLMVDRGVVSNLTPLRSLGCIDPGWEHGVAQ<br>DQRKKKVKCNYCGKVVSGGINRFKQHLARIQGEVAPCKDAPEDVYLKMKENMKWHRSGRRHKWCGTKEISDSHMQSNI<br>EDEVNEQEKDALHRMNSDRKMIGDKRQRKELRVAFNEMSPCRDIESLFNSTRFDSASLKTPCSQTTPSSKQVKIMRGSG<br>KKSQKEIISAICKFFYYAGVPLEAANSQYFHNMLELVAQYGQGLVGPPSQLIFGDLLQEEIETIRNNLAEHRASWAVTGCSV<br>MADSWTDSESRTLINLFVSSPHGLYFLSSVDATDIVEDASNLFKLLDKVVEEMGEENVVQVITQNTPSYKSAGKMLEEKR<br>KNLFWTPCATYCIDKMLDGFLNLKSVWKCMEMAQKITKFIYNHIRLLNLMKKEFTQGQELLMPSVTRFASSFTTLQNLLDH<br>RIDLRRMFQSEIWTSSWFSKSDKGKEVENIVLNAKFWKKVLYVVRSVNPIMKVLQKVDTGQSLSMPYVYNDMYRAKLAIQ<br>SFHGDDVRKYGPFWNVIDNHWDLLGHHPLYVAAYFLNPSYRYRPHFVAYPEVVRGLNECIVRLEPDKLRRIYAMSQISDY<br>TTAKADFGTELAISTRTELEPAAWWQQHGISSLQLQQIAVRILSQTCSSIGCEHKWSIYDQVHGQRHSRAAQKRINDLTYV<br>HYNLRLRECHIKRRSDDSISLEGALREHLLDDWIIEAEKQALPEDEEIFCDERGAAYDDDEYKNDLIENEDGNAEARNGST<br>EILTSSELLPLDVHPVKAGLATDDGADMNFSEDEESE	zf-BED--DUF-domain--Dimer_Tnp_hAT--	I
Ciclev 10011 114m	CicZf-BED 03_I	MTRSAQTPLLFRNASKKLRVVLLAPRRHPNKTPSHIYKQLTALEIYQCWWYPRGQGLRERKEGTSTDPDQIVDVEVDPSL<br>CIYLPRPLFLNHHSFAERFVRLSGTLRALGPFLISLSALTCSQLFVLLRTEVSMAKELSYTWPNGCTHVPGGSLPSLSSGK<br>SSDAEISTRPQQEININEAEEQNENQEKVMSLEININEAKEQNENQEKVMYLEININEAKEQNENQEKVMVNQDAKNSCR<br>NKRKKSEAWDHFSKRNGLAVCNYCGREFDGSSKKGTTHLKNHFQRCPGKRNGDVDKFIKSVIDAIGLGMNSEHWDPLV<br>LNSKKDDILQVYNKEKEKLRRFLNKLSCRFSLTIEWYANFCLLVVHYINDGWDPMWKVISVQDVRFPYSILLENMKLSCLD<br>WQIDRDICMVITTGDNVISEINSWVCERGSLPFGGQMMAIGGLVEILLTFVENLSSRTCDTVGIRRCFRDVLKSTPLNEHKF<br>GKISAAKVKSMGNKTYGFEQLEIAVGLKEVLCELVNADSNFKSMNLTKEDWQKATVAYEHIKALHDVACSLSESKCKTAN<br>AYFPKVCDIYMKLLQWERSEDDRVGKIALEAREIFFNRYWMKYELILVIAAVLDPRFKMDIVQLWYKEIYASAAHTHLEKIK<br>DEFNHVYNEYAKGSGKSRSGDASSSMSFKLLDAMGRPCTLLHGVFDDASPKSELDCYLNEPKFPRVEEFDILAWWRVN<br>TPKFPTLARMARDFLAMPISPSATTAYACFSKEAKDIFDTGLDNDLITALVCLKGWL	zf-BED--DUF-domain--Dimer_Tnp_hAT--	I
Ciclev 10019 100m	CicZf-BED 04_I	MNFAAGIVTGKAGGSMDWSVNTAYKTYKGVEVEPKHMMDMTLIPSIDPIDIGLGSSEKGNAAPSAKPRKKTMTSVYLKFF<br>ETAPDGKSRRCKFCGQSYSIATATGNLGRHLANRHPGYDKSGDAATSTATAPQTTVIVKKSQPQAKAHQVDYDHLNWLL<br>IRWLILASLPPSTLEEKWLMNSFRFLNPSIQLWPGDKYKAVFREVFRSMQEDVRLSLEQVSSKLSIILDFWTSYESFFYMS<br>VTCQWIDESWSFRKVLLDICHIPYPCGDSETYHSLEKVLENYNIENKVLSCTHDNSQNAIHACHTLKEKFDGQKVGPFCYI<br>PCAARTLSLIIDDGLRTTKPVISRVREFALQLNECTDFSEDFIQFSMAYREGSWKFPLDTSARWSGSYQMLDIVHKAGKTM<br>EAVVRKNGEKLGDRMILTAAEKTAVSIVHGYLEPFYKTTNNMCTTKMPTIGLILFFMDHISEMITVCRESRHTPEWLKSAAE<br>DMAKKARSYSSQVCNIFTYMTAILDPRIKCELIPENLNSENHLEEARAHFMRNYSTGHFPSVTSGYGAHEIEDGGSVSFAE<br>EIARKKRRGSIVSATDELTQYLSEPPAPMPTDVLEWWKVNSTRYPRLSVMARDFLAVQATSVAPEELFCSKGDEIDKLRF<br>SMPHDSTQAILCIKSWAQDGIKFKYRSTEIDYERLMELAAASVADNNVTSSDKKQK	zf-BED--DUF-domain--Dimer_Tnp_hAT--	I
Ciclev 10019 416m	CicZf-BED 05_I	MKGDPNAGSSSPSAIKKMKKPSDVLEEMREYRDADGNVKAECNHCKKTFDGSSKKGTTHLKNHLESCQANPKSKGRTE<br>ELKMLASLIKKKSVGGLIEHFSNTERDTTDEDFDHSSDREGDTSDEKRENSDGLDPSVLKSRKDEILQLYQEEKEKLHQFL<br>KNLSCRFSLQLVALVGLFEVPCSLVVHYIDDSWQRQSKIISFWVDDADIAMEYVKESCLSIHGKICSVLVPFQIPPAYDGES<br>WFSRQGSLPFAGCLRNADGLMDYIDGVLRSEDYFRDGIQSLKECAHYVASKSRSDERIQLDGDVQRAICWLNRVPDPW<br>GHLKYFATAVRVKEAFFELGQLDSDFRTINLTEKKWDEVTATLEHLKFLEDVAVGFSSGKCETPIIVDLPYVHKILKDRCNH<br>PVDDCLFCKKVMKGAIDLFFSKYYLIRVIAVILDPRFKMDGVQLHCKEIHGSDADRCLEKVNKDFRDVYDAYAATDTSNSK<br>SYEMLDAMGRPSSPKSELDLYMKELKVPAVEEFDILAWWRGNAPFYPTLARMARDYLALKIPFDHRAAEGAGALVHGGL<br>LCHYFYGEDIPELIRPLVCLKGFLGTAEN	zf-BED--DUF-domain--Dimer_Tnp_hAT--	I
Ciclev 10023 618m	CicZf-BED 06_II	MTSQFDKGKCVAVDSAPPSSKSSSNAEGTPNKSTTSEALEHFIQLSSDPLHPRSKCRYCGKEYKCGFNKGTSNLLDHIRE<br>GCLKYPGRARVDKKQKTLSYQPVKIGESCSLVSTTYSVEACREAIAKFIIKDEMSFRVVEGEGFREMLGVFENRFKVPSRT<br>TIARDILQLYKKTKKYLTDFFVSSHQRRAGETKAVLDKISIICRRE	zf-BED	II
Ciclev 10024 195m	CicZf-BED 07_III	MAPLRSTGYVDPGWEHGIAQDERKKKVKCNYCGKIVSGGIFRLKQHLARMSGEVTHCEKVPDDVCLNMRKNLEGCRSG<br>RKRSQSENEQASLSFHSSDYNDTEDALTGYKHRGKKVMSDKNLVIRFAPLRSLGYMDPGWEHCVAQDEKKKRVKCNYC<br>EKIISGGINRFKQHLARIPGEVAYCDKAPEDVYLKIKENMKWHRTGRRHRKPDTKEISAFYMQSDNEDEEEEDDNRFLQC<br>VTKDIVAIDDKVSDTEVRYNVKGRSPSSSGNGTEPPVRRSRLDSVFLKSLKSQTSPYSGHVKAKTGIEKKIRKEVISAICKF<br>FYHAGIPSNAANSPYFHNMLELVGQYGQGLQGPSSRLISGRFLQDEIATIKENLAEVKASWSITGCSVMADCWNDVQGR<br>TLINFLVSCPRGLYFISSMDATDSIEDAANIFKLLDKVVEEIGEENVVQVITKNTASFKAAGKMLEEKRRNLFWTPCAVDCID<br>RMLDDILNIKWVGECLDKAKKLTRFIYNSTWLLNVMKKEFTKGQELLRPATTKFATSFNTLQSLLDQRIGLKRLFQSNKWL<br>SSRFSKSDEGKEMEKIVLNLTFWKKMQYVKKSLGPIVQVLQKIDSTESRSISFLYNDMYRAKLAIKAIHGDDARKYGPFWS<br>VIDSQWNSLFHHPLHVAAYFLNPSYRYRPDFIMHPEIIRGLNECIVRLEVDNGKRISASMQIPDFVSARADFGTDLAISTRS<br>ELDPAAWWQQHGISCLELQRIAIRILSQTCSSVGCEHTWSTYDQVHSRRRNCLSRKRWNDLTYVHYNLRLRECQLGRKS<br>DDAISFDNAMLESILDDWLVESERQTIQEDEEILYNGMEPFYGDEIDENENEERRSAEMVALAGLVEPLEVNPAAGGVTTD<br>DDGLDFLDDDLTD	zf-BED--zf-BED--DUF-domain--Dimer_Tnp_hAT--	III
Ciclev 10024 286m	CicZf-BED 08_II	MDFCPNLLQSENDDNDGYDVVEVIEPSNGMELNSTSPIPSDETTDVSKKLKSVVWNHFTRQKINGQLKAICNKCNGKLVV<br>GSGTNHLHKHLLRCPRCKQPTIKNATLNATINKGKVQVGAFSFDHDTSRRELANMIIKHEYPLSMVEHSGFRKFVGSLQP<br>MFKVISRNTLKKDIFKQYEYE	zf-BED	II
Cotton_D_ge	GrZf-	MEVANETVIKKPKRLTSVVWNHFERVRKADLCYAVCVHCNKKLSGSSNSGTTHLRNHLMRCLKRFNYDVSQLLSAKKRK<br>KESTLTIANISYDEGQRKEEYLKPTIVKYEPEQRKDEVFNVQSSWFDQDRSRLDLARMIILHGYPLAMVEHVGFKVFVKNL<br>QPLFDVVPNSTVELSCMEIYGKERQKVHDMLSKLQGRINLAVEMWSSPENTNHVCMMAHYVGDDWKLQKKILNFVTLDS	zf-BED--DUF-domain--	I

ne_10000873	BED01_I	SHTDDLLSGVIIKCLMDWDIGSKLFAVTLDDFSTNDDIVLRIKEQISENKSRLSNGQLLDVRSAAHVLNSIVQDAMEALRVVL<br>QKIRGTVRYVKSSQSIQGKFKEMVLQTGINSQKNLVLDCPIRWNSTYLMLEAAIEYRNAFCQLPDLDLDLALSDEEWEWA<br>SSITGYLKLFVEIINVFSSNKCPTANIYFPEICHVHIQLIDWCKSPDNFLSSLAAKMKAKFDKYWSKCSLSLAVAAILDPRFKM<br>KLVEYYYSQIYGSTALERIKEVSDGLKELFNTYSICSTLMDQGSALPLGSLPSSSNDGRDRLKGFDKFLHETSQSQTAISDL<br>EKYLDEPVFPRNCNFNILNWWRVHTPRYPILSMMARDVLGTPMSTVSQESAFHAGGRVLDSCRCPLTPETQQALICTQD<br>WLRIQSDDPGPSSSHYALPLYVETN	Dimer_Tnp_hAT--	
Cotton_D_gene_10011977	GrZf-BED02_I	MAEITEATNMETTPVENNNELALITPETQPKRRKKKSMVWEYFTIETVSAGCRRACCNRCKQSFAYSTGSKVAGTSHLKR<br>HIAKGTCPALLRDQYNNQLTPYNPKTGGSEPRKRRYRSPSSPFIPFDQDRCRHEIARMIIMHEYPLHMVEHPGFIAFVQNL<br>QPRFDKVSFNTVQGDCVATYLREKQSLMKLIEGIPGRVCLTLDMWTSNQTLGYVFITGHFIDFEWKLQSRVLNVIMEPYP<br>DSDSALSHAVAACLSDWSLEGKLFSLTFNHPTSEAGLENLRPLLCTKNPLILNGQLLLGNCIARTLSSMAKDVLGAGHEIV<br>KKIRDSVKYVKTSESHDEKFVQVKNQLQVPSEKSLILDNQTQWNTTYQMLAAGTELKEVFNCLDTSDPDYKLAPSIEDWK<br>VAETLCTFLKPLFDAASILTTTTNPTAITFFHEAWKIHADLGRSITNEDPFISNIAKSMLEKIDKYWKDCSLILAIAVVMDPRFK<br>MKLVEFSFTKIFGEDAPTYIKIVDDGIHELFLEYVALPLPLTPTYTEEGNAGNNGKTDESQQGNLLSDQGLTDFDVYIMETS<br>SQQMKSELDQYLEESLLPRVQEFDVLGWWKLNKMKYPTLSKMARDILSIPVSAAAPDSVFDIIIKQLDEYRSSLRPETVEA<br>LICAKDWLHYGSEESNALVKMEF	zf-BED--DUF-domain--Dimer_Tnp_hAT--	I
Cotton_D_gene_10017869	GrZf-BED03_I	MEWSVNNAFKSYKDMEPKSTMDMVLIPNMDTIDIVLGSSEKGNVVPSAKPRKKTMTSVYLKYFETAPDGKTRRCKFCGQ<br>SYSIATATGNLGRHLSNRHPGYDKTGENVTSSAPQPSTTPTVIKKPQQQGRAPQVDYDHLNWLLIKWLILATLPPSTLEEK<br>WLANSFKFLNPSIQLWPGEKYKAVFREVFRSMREDVRASLEQVSSKVSIALDFWTSYEQIFYMSITCQWIDENWSFRKVL<br>LDICQVPYPCSDSEIYNSLVKVLKMYNIENKVLSCTHDNSQNAIHACHALKEDLDGQKMGPFCFIPCAARTLSLIIDDALRTT<br>KPVIAKVREFVQELNASLDISEDFIQLTTAYKEGSWQFPLDASARWSGSYQMLDLVQKAGKSMDAVVRKNEEMLGNRML<br>LNTAEKNVVNIVHNYLEPFYKVISEICVNTPPTIGMVIVYMDHISDTITTRQPPDWLKNPAEDMAKKLRSYNNQVCNIFIYMT<br>AILDPRIKCELIPESLNSENYLEEARAHFVRNYYTTPFSSMTSGYSSQDIEDGGAVSFAEEIARKKRRASMSNATDELTQYL<br>SESPAPTKTDVLEWWKVNSTRYPRLSAMARDFLAVQATSVKPDELFCSKGDEIDKQRFCMPHDSTQAILCIKSWTQGGL<br>KLKYKSTEIDYERLMEMAAAAAADISSAGIDKKQK	zf-BED--DUF-domain--Dimer_Tnp_hAT--	I
Cotton_D_gene_10023062	GrZf-BED04_I	MELNLVPISITRQKQDPAWNHCEVFKNGERLQIKCMYCGKLFKGGGIHRFKEHLAGRKGQGPICEQVPQGVRSVMQESL<br>NGILVKQDKKQKLIPKLLACGSSSSNPNIGGEVENLGSHDDMNFGIKPISVLNTLEGDSNVVSKVGRGRKRGRGRGRDW<br>NLIESNYPCVKTDLALVPNGGENPIHMAIGRFLYDIGVNLDAVNSVCFQPMIDGIASGGSGVVPPSCNDLRGWILKNVIEEV<br>KDDIDRNKAMWGKTGCSIIVEQCRTKNGRVLLSFLVYCPQATVFMKSVDASHAVYSADYLFELLKQVIEEVGSEKVVQVIT<br>NCEEPYLFTGKRLMESFPSLYWAPCLAHCVDLMLQDFSNLEWINETIEQAKSLTRFIYNQSSVLNMIRKFTSGNDVVEPAL<br>TCFATNFSTLKRMADLKLNLQAMVNSQDWLECPYAKKPGGQAMSDIVNNSSFWNSCMLIARITYPLLRVLEIVGSKKRSA<br>MGYVYAGIYRAKETIKKELVKQDDYMVYWNIIDNRWEQQRHLPLYAAGFFLNPKLFYNTEEHIHNDILSSVFDSIERLVPDT<br>NIQDQVVREINLYKNATGDLGRPMAVRARDNLLPGEWWSIYGGGCPNLQRLAIRILSQTCSSIGYKPSKISIEEIHNTRNFL<br>ERQRLSDLVFVQYNLYLRQMVLQKQEKDSLDPLAFNNKDILEDWIADTEVSPDNLESSDWKSLDPPVGNRTTLTPPSDEA<br>EDFLSTRFTDLDIFNGLKGVKEEI	zf-BED--DUF-domain--Dimer_Tnp_hAT--	I
Cotton_D_gene_10023084	GrZf-BED05_I	MDNFDQKLGPEFFKNLSAEAVTPLNVVHEEIYESSSKRPKTTSKVWDIFEKLPAQQGDSKAICKLCRRIYTAKTTSGTSHL<br>RRHIEACVKRGNHEVDQRSIEACFKPVKRNANRLTLSHDTLIAATTSLKNYKLDVDEIHRAIAMMIIVDEQPFSVVEDAGFR<br>RLLSAACPEFPVLSRSSIKRDIISIYVKERENIRELLATCPGRICLTSSTWKSDSDDHFNCVTTHFIDHEWRLQKRILSFKLM<br>PPPYDSLSVADEIALCMVQWNIEHKVFSVTLENLSSDDCVADMLRSRLAAKKYLPCKGVFFHVSCFFRILNSIVQAGLNLV<br>VDIIAKLRLGIKYVQQSPHRKKNFYIIAKTLNLDTQRKLCLDTPARWNSTYNMIEVAFCYNNAFMYLAEQDKNFLHKLSEDE<br>WEKLSVLYKFLKVFYEVTCVFFRNRQPTSNLYFKAAWKVHSRLFDMVRGPENFMTRMVREMHSKLNQYWSAYNLILSC<br>AAILDPRCKIKFVEYCYTKLYGSGAQKYVSVSVNTLYGLFDEYMQNSARPSQTTLLSTAASKISNDKDNNDGFEDYETFQS<br>ARFRTQVEKSQLDLYLEEPSHDLNSEIDVLEYWTLCSLRYPELSKMARDVLTIPVSTIASDSAFDISPQVISTDRSSLKPKM<br>LQALVCLQDWMLASDRTRGLGSMESKPEDDSSSSSDGDDDY	zf-BED--DUF-domain--Dimer_Tnp_hAT--	I
Cotton_D_gene_10023113	GrZf-BED06_II	MSTEPTSIKGSVTPPTSIDSENSGVGASSQTKGTTGKEKTAPQRLEVWSHFTKIINSEGASKAKCNYCQKEFCCDMKKNG<br>TGSLKYHIGSCKKNPSNVQGQLVLPRKRVEGGGRKFFNLEI	zf-BED	II
Cotton_D_gene_10024621	GrZf-BED07_I	MSSNLEPIPITSQKHDPAWKHCQMFKNGERVQLKCIYCGKIFKGGGIHRIKEHLAGHKGNAATCLRVPSDVRVLMQESLD<br>GVVVKKRKKQKIAEEITNVNQVSTEIQAYADQVDTNTGLLMIEKSDTLEPSSSLLVNQEGTSNVAGERRKRGRGKSLPAE<br>ANALSFVPVELGARRVNNHVHMAIGRFLFDIGATMDAVNSVYFQPMVDAIVSGGSGALMPSCNDLQGWILRKLVEEVKS<br>ENDKVMAAWVRTGCSILVNQWNTQTGRILLNFLVYCPEGTVFLKPIDASSVINSSDALYELLKQVVEEVGSKHVLQVITNG<br>EEQYIVAGRRLVETFPTLYWAPCAAHCVDLILEDFAKLEWINAIIEQARSITKFIYNHSVVLNMVRRYTFGNDIVEPAATRSA<br>TNFTTLTRMVDLKNNLQAMVTSQQWVDCPYSKKPGGLAMLDLVSNQSFWSSCVLIVRLTNPLLRVLRMVGSKKRPAMG<br>YVYAGMYRAKETIKKELVKRNEYMVYWNIIDHWWEQQWHHPLHAAGFYLNPRFFYSMEGDMPNEMLSGMLDCIEKLIPD<br>VTVQDKITKEINSYKNSVGDFGRKMAVRARDTLLPVEWWSTYGGSCPNLARLAIRVLSQTCSTLGLKHDHIPFEKLHETR<br>NCLEQQRLRDLIFVQCNLQLRQIGYESKQHDSMQPLSSESASIVEDWVTGIDAFLDDDTYPDWTTLETLSVNTMPLRPGD<br>EVEELGAGNFVSWIW	zf-BED--DUF-domain--Dimer_Tnp_hAT--	I
Cotton_D_gene_10035444	GrZf-BED08_III	MVEEMAPLRSIGYVDPGWEHGTAQDERKKKVKCNYCGKVVSGGIFRLKQHLARLSGEVTHCEKVPEEVCLNMRKNLEG<br>CRSGRKRRQLDYEQAALSIQSNEYSDGEDASASYKHKGKKVMGDKNLVIKFTPLRSLGYVDPGWEHCVAQDEKKRRVK<br>CNYCEKIISGGINRFKQHLARIPGEVAYCEKAPEEVYLKIKENMKWHRTGRRHRKPDTKEISTFYMHSDNEDEGGEEEGY<br>LQCISKDILAIDDKVSDNDIRNNVRGRSPGSSGNGAEPLLKKSRLDSVFLKSLKSQTSAHYKQPRARTGFEKKTHREVISAI<br>CKFFYHAGIPSNAANSPYFHKMLELVGQYGQGLQGPSSRLISGRLLQEEIANIKEYLVELKTSWAITGCSVMADSWNDAQ<br>GRMLINFLVSCPRGVYFLSSVDATDIIEDAVHLFKLLDKAVDEVGEEYVVQVITRNTLSFRNAGKMLEEKRRNLFWTPCAV<br>YCIDRMLEDFVNIKWVGECVDKAKKVTRFIYNNTWLLNFMKKEFTKGQELLQPAVTKFGTNFFTLQSLLDQRVGLKRMFQ<br>SNRWLSSRFSKSDEGKEVEKIVLNVSFWKKMQYVKKSFEPVAEVLQRIGSDKIRSLPFIYNDICRTKLAIKAIHGDDVRKYG<br>PFWSVIESNWSPLFHHPLYVAAYFLNPSYRYRPDFLMNPEVIRGLNGCIVRLEADNGKKIAASMQIPDFVSAKADFGTDLA<br>ISTRSELDPASWWQQHGISCLELQRIAIRILSQTCSSIGCEHNWSAFDQVHIKRHNCLSRKRLNDQTYVHYNLRLRERQLG<br>RKPDELVSFDSAMLESVLDDWLVETEKLAMHEDEEIIYTEVEQFCGDDMDEHESEEKRPAEMVTIAGFIEPLDVIPSAGGV<br>TTDDDGLDFLDDDLTD	zf-BED--zf-BED--DUF-domain--Dimer_Tnp_hAT--	III
Cotton_D_gene_10036775	GrZf-BED09_II	MSTEPTSIEGSVIPPTSIDSKNSGVGASSQTKGTTGKRKVAPQRSEVWSHFTKIINSQGASKAKCNYCQKKFCCDMKKW<br>YRVIEISYWFM	zf-BED	II
Cucsa.036140.1	CucZf-BED01_I	KKNPQLNPVAGLIEMPNFRSNPRSEKLESFNYNSQPISDIYLFIYLFSLLPILELRFIRLDFDYLDWKLESREFEDAFEGIIFP<br>DEMATPDGTNMESSGKDIMVIHHDEDHDLTTPNGENMVAREDELMETPDTGKLGSPDGHNELALVEVQPNKRRKKKSIV<br>WEHFTIESVSADCRRAYCKQCKQSFAYSTGSKVAGTSHLKRHIAKGTCSALLRGQDKNQMISTYTPPSRGTVSETPKRR<br>YKTSSSPYVVFDQDRCRQDIARMVIMHDYPLHMVEHPGFVSFVKNLQPRFNMVSFNTVQGDCVGTYLMEKQNLNKFIEA<br>IPGRICLTLDMWSSSRSVGYVFIMGSFIDSEWRLHRRVLNVVMEPYPESTNALSHAVAVCLSDWRLEGKLFSLTFNQRQN<br>EAALDNLRPLIAIKNPLILNGQFLVGSCIAWTLSDIAKDVLESGENTITKIRDSVKYVTTSDSHEEKFLELKQQLQVPSERNLF<br>LDDQTQWNTTYHMLVAASEMKEVFSCLDTSDPDYREAPSLEDWKLVEILCTYLKLLFDAANILTTTTHPSAITVFHEVWRI<br>HSELARAITNEDPFISSLSKMMLEKFDKYWKDCSIVLAAAVVMDPRFKMKLVEFSFTKVYGEEAPAYIKVVDDGIHELFDEY<br>VALPLPLTPTYAEDGSVDGNMKGEDSQGNLLSDNGFTDFDVYIMETTSQQMKSELDRYLEDSLLPRVQDFDLLGWWKL<br>NKFKYPTLSKMARDILSIPVSTLPPDSVFITRRKKMDQYRSSLRPETVEAMICSKDWMQCGLSEVSNALVRMEY	zf-BED--DUF-domain--Dimer_Tnp_hAT--	I
Cucsa.057910.1	CucZf-BED02_I	MEMSNDSSVKKPKRLTSVVWNHFERVRKADICYAVCVHCNKKLSGSSNSGTTHLRNHLMRCLKRSNYDVSQLLAAKRL<br>KKDSSSGLANIGFDEGQRKDEFKSAIVKYDPNQRRDEVVTIASSKFDQERSQNDLARMIILHGYPLSIVEHVGFKVFVKNV<br>QPLFEVLPNSTVEISCMEIYQKEKLKIFEIINRLPGRINVSVEMWSSSENIDYLCLTAHYIDEDWKLQKKLLNFITLDSSHTED<br>MLSDVIIKCLVDWGIDCKLFALTFDDFSTNYEIIRRIEEHISKQRSLLSNVRLFDVCSAAHTINAIAKDVIEAMREVNQKIRGSF<br>KYIRSSQVTQARFNEMAHQVGITSQKILVPDNPGQWNLTYFMLETAVEYKGAFSLLQKHDSHYASALTALEWEWTTSVA<br>DYLKLFVEIVNIFSSNKSPTANIYFPEMCDVHIQLIEWCKSSDSFLNSLASKIKAKYDKYWSKCSLALAVAAVLDPRFKMKLV<br>EYYYSQIYGSMALDRIKEVSDGIKELYDQYSICFSMVVQGSTLHGSGLPGTGNESRDRLKGFDKFLHETSQSQSVMSDLD	zf-BED--DUF-domain--Dimer_Tnp_hAT--	I

		KYLEEPVFPRNCDFNILNWWNVHTPRYPILSMMARDVIGTPMSSIVPEFAFCTTGRVLDRYRSSLSPDTRQALICAQDWL<br>CIELEGMFLMDSTSIRFSSFTCNCLFDFVSYQIQDHCLPLRTYRFCLKPINCT		
Cucsa.090680.1	CucZf-BED04_I	MDWSVNNAFKTFKDLEPKSMMDVSLIPHVEPIDIDLRSSDKGSPNTSAKPRKKTMTSVYLKFFETAADGKSRRCKFCGQS<br>YSIATATGNLGRHLSNRHPGYDKSGAIVVSNPAPQPISTMKKSQPQGKPQQIDYDHLNWLIIKWLILSSLPPSTLEEKWLA<br>NSYKFLNPSIQLWPTEKYKAVFREVYRSMQEDVRASLEHVTSKISVTLDFWNSYDQISFMSVTCQWIDESWSFQKVLLDI<br>THIPYPCGGLEIFHSIVKVLKMYNIESRILSCTHDNSQDAVHACHALKEHLDGQKVGPFCYIPCAARTLNLIIDDGLRPTKSII<br>AKVREFVLELNACLDISEDFVQFTTVYQEGNWKFPLDASVRWSGNYQMLDIVRKSGKSMEAVIRKYEETLGSKMLLNSAE<br>KNVVNIVHQYLEPFYKTTNNICTNKVATVGLVLFFMDHISETIAACRDSRHNPDWLKSAAEDMAKKAKNYSSQVCNIFTYM<br>TAILDPRIKGELIPESLNSGNHLEEARSHFMRYYSSNHFPSVTSGYSAQEIEDGGSVSFAEEIARKKRRASMSNATDELTQ<br>YLSEPPAPIPTDVLEWWKVNNTRYPRLSVMARDFLAVQATSLAPEELFCGRGDDIDKQRYCMPHDSTPALLCIKSWIQSG<br>FKLKYKSSEIDYERLMELSATSTVDSSTAGSDKKSK	zf-BED--DUF-domain--Dimer_Tnp_hAT--	I
Cucsa.090680.2	CucZf-BED05_I	MDWSVNNAFKTFKDLEPKSMMDVSLIPHVEPIDIDLRSSDKGSPNTSAKPRKKTMTSVYLKFFETAADGKSRRCKFCGQS<br>YSIATATGNLGRHLSNRHPGYDKSGAIVVSNPAPQPISTMKKSQPQGKPQQIDYDHLNWLIIKWLILSSLPPSTLEEKWLA<br>NSYKFLNPSIQLWPTEKYKAVFREVYRSMQEDVRASLEHVTSKISVTLDFWNSYDQISFMSVTCQWIDESWSFQKVLLDI<br>THIPYPCGGLEIFHSIVKVLKMYNIESRILSCTHDNSQDAVHACHALKEHLDGQKVGPFCYIPCAARTLNLIIDDGLRPTKSII<br>AKVREFVLELNACLDISEDFVQFTTVYQEGNWKFPLDASVRWSGNYQMLDIVRKSGKSMEAVIRKYEETLGSKMLLNSAE<br>KNVVNIVHQYLEPFYKTTNNICTNKVATVGLVLFFMDHISETIAACRDSRHNPDWLKSAAEDMAKKAKNYSSQVCNIFTYM<br>TAILDPRIKGELIPESLNSGNHLEEARSHFMRYYSSNHFPSVTSGYSAQEIEDGGSVSFAEEIARKKRRASMSNATDELTQ<br>YLSEPPAPIPTDVLEWWKVNNTRYPRLSVMARDFLAVQATSLAPEELFCGRGDDIDKQRYCMPHDSTPALLCIKSWIQSG<br>FKLKYKSSEIDYERLMELSATSTVDSSTAGSDKKSK	zf-BED--DUF-domain--Dimer_Tnp_hAT--	I
Cucsa.096030.1	CucZf-BED09_I	MELEPYLPLTPMDSSSKRRRKKSFVWDYFTVQTAEDGCIKAYCNQCNKAFAYINGSKLAGTSHLKRHIALGICPVSRERN<br>QSNGTHTTFAKRNTANPPRKRYRATPTFASFTFDQNRCNHDIAIMIIMHEYPLYMVEQPGFIDFVKNLQPQFNMMSYTTV<br>QEECTSIYLREKESLLNFVCGIPSRISLSIDVWTSSDTTCYVFLKGHFIDNNWNSHCRILNVIRIPSLKDDALILAVVTCLSNW<br>QLSGRVYALTVDQSFLSDTIAMNFRNFLVANDPNFLNGQLLVGNCLAQVLCQLAQNALSLTSEVVWKIRESVKYVKTSSV<br>REEKFLELKNQLKVPSNKELSIDNQTKWDTTFHMLVAACELKEVFSCLDASDPDYDLNMSVDDWKVAETLCTYLKYFFSV<br>ANILTSPCYPTANVFFLEVSKIQTELTNASMSQDPLVRDLTKPLKDKFDKYWNECCLVLALAVVMDPRFKLKLVEFIFSKIFV<br>QNAEEWIRIVDYGLHDLFLQFTMETLTLSETFGEDGNIGISITELQDEEHQGEIFLTTGDGLSDFDVYISEISEGQQTKSELD<br>RYLEEELSPKSQEFDVLSWWRLNKYRYPTLAKMACDILSMPVSTVTRDSVFDTEIRRMDSYRRSLPSATLEALICTRDWL<br>QYGSLQSSSSTETSDTTVKX	zf-BED--DUF-domain--Dimer_Tnp_hAT--	I
Cucsa.185330.1	CucZf-BED10_I	MQLLVESQGDEMDMSEAVIVKSSRLKSVVWNDFDRIKKGDTFVAVCRHCKKKLSGSSTSGTSHLRNHLIRCQRRSNHGI<br>SQFITSREKKKEATLAITNYVDQGQKKDDVLNLVNIRFDPEQVKEETNNTLSYNFDQRRSRFDLARMIILHGYPLAMVEHV<br>GFRVFVKNLHPLFELVTCNRVETDCLEIYVKEKQKVNELLDKLPGKISLSADMWSATDHLESSYLCLTAHYIDESWQLNKK<br>VLNFMFVDPSHTEDLHAETIMSCLMDWDIDRKLFSMTFDCSSTSDYISLQIRDRLSQNRFLYCNGQLFDVSCAIDLLNLMA<br>QDALEALCNILPKIRESIQHIKQSREGQAKFNEIAEQVKVQSQKLLSLDNPSMWNSTYVMLGAAIEYREAFSLLRENDLGYT<br>ACPTEEEWGRTCTITGYLKFLFEVTNVLTKANFSTANIFFPELCDLHLQLMEWSKNSDDYIKSLAFKMRTKFEEYWDKSGL<br>ALAVSAMLDPRFKMKLVEYYYPQIYGVVALQRIDDVLNCVKALYNEHSICSPLASIDQGLAWQVGSSSGSLPGSGKDARD<br>RLMGFDKFLHESSQSEGSKSDLDKYLEEPLFPRNVDFNVLNWWKVHTPRYPILSMMARNVLGIPMSKVAPDSAFKTGRK<br>VLDRDWSSLKPATIEALVCSQDWIRSELES	zf-BED--DUF-domain--Dimer_Tnp_hAT--	I
Cucsa.252180.1	CucZf-BED11_III	MMAPIRTSGFVDPGWEHGVAQDEKKKKVKCNYCGKIVSGGIYRLKQHLARVSGEVTYCDKAPEEVYLRMRENLEGCRS<br>NKKPRQSEDDEQSYLNFHSNDDEEDGSHVTYRNRGRQLMGNRNVGTNMTPLRSLRYVDPGWEHGVAQDERKKKVKC<br>NYCEKIVSGGINRFKQHLARIPGEVAPCKHAPEEVYLKIKENMKWHRTGRRHVQTDANEISAYFMQSDNEEEEEEKEESL<br>HHISKERFIDGDKRLSKDLKSTFRGMSPGGGSEPSVKRSRLDSVFLKTTKRQTEQVQKQALVKRGGNRRSRKEVMSAIC<br>KFFCYAGIPFQSANSVYFHKMLETVGQYGSGLVGPSCQLMSGRLLQEEVATIKSYLVELKASWAVTGCSILVDNWKDSD<br>GRAFINFLVSCPRGVYFVSSVDAMEIVDDPSNLFSVLDGVVDEIGEENVVQVITENTPIYKAAGKMLEEKRRNLFWTPCAT<br>YCVDHMLEDFLKLRSVEDCMEKCQKITKFIYNRSWLLNFMKNEFTQGLELLRPAVTRNASSFATLQCLLEHRGNLRRMFV<br>SNEWTSSRFSKSGEGQEVEMIVLNPSFWKKVQYVCKSVEPVLQVLQKVDSVQSLSISSIYNDMYRAKFAIQSIHGDEARK<br>YGPFWNVIDSNWNSLFCHSLHMAAFFLNPSYRYRPDFVAHSEVVRGLNECIVRLESDSSRRISASMQISDYNSAKSDFGT<br>ELAISTRTELDPAAWWQQHGISCLELQQIAVRILSQTCSSLCFEHNWTPFAKEHSQRHNSLSQRKMADLLYVHYNLQLRE<br>RQLRKQSNESISLDHILMEHLLDDWIVEPRKQGMQEDEEILCPGMEPLDAYENDLIDYEDGTSEGRKGCLQLVGLTDVDT<br>LDVNPANGGASTDNDADVKFYDNELSD	zf-BED--zf-BED--DUF-domain--Dimer_Tnp_hAT--	III
Cucsa.312740.1	CucZf-BED12_I	MEREEATTEETKETTRIKATINVDEEEVDETPTKRRRNMPVSIVWQHFERLKGDPNEPYARCKYCGATYACHSKRNGTG<br>TMKNHLESCKKYPFERKRDSFQKTSPFKPKEETAKTDPLQLVCEPKEETDGDGSSQVLCESINSESCRETLAEMIIMEALP<br>FKFVESKGFKRFVSKLTGSQEPKFVVPSRFTIARDILKIYVNEKQKLKDIFVKMKCKVCLTTDCWTSEQNINYMVLTAHFVD<br>CDWKLHKKILSFSQIESHNGHTIGKAIEKNLKDWGIKRIVTLTADNASSNDASIAFLIKRFNKGLICNGEYMHVKSYSQILNLI<br>VCDVFKDHNESIGRIRNAVRYVRSSPARLMKFKKCAENEKISSNSVVCLDFPNRWDSTYVMLEVALKFAKAFDRLENDED<br>AYRNDMPPTNEDWAIARLLIRFLKVFYNVTLKVSGSLYTTSNVVFHEICRIQNCLQLNGDSGNKMLSTMAKNMKAKFDKY<br>WEDDEKKNNILLCIAVVLDPRYKLKCLKYCWNNLFGPDIAMTKTRIVENALRRLFHEYNIGPSSSNCNSSSASCSTSVSAS<br>ASACDFANEIHSYDCMALDTIMDVDVKDEVETDVDLDYGITNSFKDVETTTLDKESEIDVYLLESLAPADSYFDILHWWKQ<br>NEHRFEVLSRISRDILAIPVSTVSSESTFNIGGCVVNSNRCSVAPKMMEALICTHNWLNTNPINLEIHNQDLEEDLRFEEGF<br>RAVMDGEKDVEDMEDFV	zf-BED--DUF-domain--Dimer_Tnp_hAT--	I
Gar01G02660	GarZf-BED01_IV	MPSPARDGSTQSSQNSSPSKDDQTTTNENVRKKITPRASCWNHFTKFVTKEGEKRARCNTCDVTYTMESTSGSTTNLN<br>NHLKTCLKRPRGNTSNPKQSELAFVKVSQETTDLSTWVFDKDAIRKALVRMIIVDELPFKFVEGEGFKYFLSIACPRFSLPS<br>RWTIRRDCLDLFNSMKSVMKNYFEKDISRVCLTTDTWTSLQRISYMVLTAHWVDDEWRLQKRIINFCPISAHRGESIGQAI<br>EKCLRDWGIERVFTITVDNASANSVAIEYLRKKFNHRNASVANGKFIHMRCVAHILNLIVQYGIKDASVSVDRVRGAVRYIR<br>ASPSRLTKFNQRVKEEMIDSKAQLCLDVPTRWNSTYMMLKVAEKYERAFESYVRDDHNFFLDLTAGDGVPTFDDWEIVR<br>RVIKVLEPFYHLTLKVSGSLHVTSHSLFEVLTDVHCLFDGWQDCGDLEIISMTSKMREKYNKYWGEGNKINMLVYLAVIFD<br>PRCKMSFLDFGVNLLFPNVANDIIKMIDKELHCLFNEYSSNAGRIELFEGRSSSLTNLCSSMEIDQSEMKTGLAKQKYLKKK<br>KQVGLESKSELDRYLGEDEEVNNSSSFDLLLWWKMNSPRFLFLHKWLEIFLLFLSQQLPRKVHLARVDVFSIVLEVL	zf-BED--DUF-domain	IV
Gar01G09140	GarZf-BED02_II	MPPREEFPTKGLEGAPSNDIGWHFGTPVPNTKGNVVCKLCGKVLKGGITRFKEHIAHKTDNVAPCPNVTDKQLEKVSNH<br>NTSGIEGKNSDEVLVVGITFMKKGDLLMDQPENIIEKEQVNPSQSFRRKISEAVSKFLIYEKLPFQLASSPWLYNLIQLSIEV<br>GQVVKLPTPYEVSDVYLESEYQRVCDWTVDLLKKYTQRKQILQPAFTRFATHFIQLEEITTQKQGLREIFNSKEFKESKWG<br>QQKSRPAYEAKKIVLGKDFWKKANDLIKVYEPLVKVLRLVDSDEKPTMGFIYEAVDRAKRAIQQDCRYFTEYDKIIDNRWN<br>FMHSDLHSAETLKDTRSVIERLEPSLDNQVRMANQLLLFRDKYETFGTPQAQRAWKQMNSDGENIDVLPVDTSDDEIDV<br>DQSQQQNLSHSSSSSTPSQNGDGPDGVGLSPIDKDDGYSGDRGEIKSSSQYGGEYGVGTASVHFHDRLEFDGNMFPE<br>PRRDRSSSSGRRSSSSNLGYSDSSTSIPSYFQPSHGYPQPYGSYPPFPNYGVPYQPQMHPPPPMYHPPPSLMYLPPQI<br>CPPYQLYENQA	zf-BED	II
Gar01G12850	GarZf-BED03_IV	MANKITTQIGGIYHNTGSKEFIKQNPSWHFGTPVPNAKANIVCKLCGKVVKGGIARFKEHIAHKIGNVAPCPNVTGIIRESM<br>MNILKESNTKKIDKKRRKDEFLSQLREDEDEHEEFIDEVFAIRQETRESIQSQHEWYRREEFRRRTGGWNNMYEEGRSS<br>HGSAREYHKERTSKSISSESEFALRGVIPELARNKSSKQPKVTDSFLKSFRRKIGEAVSKFLIYKRLHFQLASSPWLYNLIQ<br>LTTPYEVSNVYLESKYQRVRDWVNGLKTHWKELGATLMCDGWTNSLNQMHIINFLIYCSKGIIFWKSIDVLSVRNRDAEFY<br>YSLLDSTSCVAHCLDLCLEDIGKKPSVAKVLDEAKKMTCFVYNHIWTVDLMKKYTQGKQILRPPLVKVFKLVDSNEKPTMR<br>FIYEAYEKIIDNRWNFMHSDLHSAGYFLNPQFQFGVEHSENVLIETLEGTRSVIERLEPSLDTQVKMVNQLLLFRDKHETF<br>GTPQAQRAWKQMNPEWLHEKENPLLDGENAGVLPMDTSDDEMDVNQSQQQKLSHSSSSSTPSQSGNRPDGGDLSPI<br>DEDDGYSVDRGEIRYSSQYGGEYGFGTASGHFRDRSKFDGNMFPEPR	zf-BED--DUF-domain	IV
Gar01G18670	GarZf-BED04_I	MPPCEEFPTKGLEGAPSNDIGWHFGTPVPNTKGNIVCKLCGKVVKGGITRFKEHIAHKTGNVATCPNVTGVIRESMMNIL<br>KESKTKKIDKKRRKDEFLSQLREDEDEHEEFIDEVSAIRQATQESIQSQHEWHRREEFRRSTGGWNNIYEERRSSHGSAR<br>EYHRERTSKSIPSESEFTLEEAIPELARSKSSKQPKKIGEAVSKLLIYERLPFQLASSPWLYNLIQVSTEVGQGVKLPTPYEI<br>SDVYLESEYQRVRDWVNGLKTHWKELGATLMCDGWTNSLNQMHIINFLVYCNASSVRSRDAEFYYSLLDLVVEEIGESYI<br>VQIVTNNEAAMKAAGKKLMLKRKHLYWTSCAAHYLDLCLEDIGKKPSVAKVLDEAKKVTCFIYNHIWTVDLMKKYTQGKQI<br>LRPALTRFATHFIQLEEITRQKQGLREIQKIWKKTNDLIKVYEPLVKVLRLVDSDEKPTMGFIYEAVDRAKRAIQQDCRYFTE<br>YEKIIDNRWNFMHSDLHSAGYFLNPQFQFGVEHSENVLIETLEGTRSVIERLEPSLDTQVRMVNQVRFKYYYLLLTIKVLSQ<br>TTSASNCERNWSTFSYIHTKERNRLKYKKLEKLVFTYYNMRLQIRHQKRMSTDDINASFNPISLDHIFEDVDPLSEWLHEK<br>ENPLLDGENAGVLPVDTSDNEMDVDQSQQQNLSHSSSNSTPSQSGDGPDVGGLSPIDEDDGYSGDRGEIRSSSQYGG	zf-BED--DUF-domain--Dimer_Tnp_hAT--	I

		EYRVGTTSGHFRDRSEFDGNMFPEPRRDRSEPSVPSKGKGKKHTSIGSSSSRRSSSSNLGYSDSSTSTQGFYPPEQPS<br>YFQPSYGVPYQPQIYPPPPMYHPPPPLMYPPPQIYLPYQLYENQGQENQVKNVLKVKVNDLIFLVIPLIGEN		
Gar01<br>G2938<br>0	Gar<br>Zf-<br>BED<br>05_<br>V	MVRGRDACWEHCVLVDATRQKVRCNYCHREFSGGVYRMKFHLAQIKNKDIVPCAEVPDDVRDHIQSILNTPKKQKTPKK<br>PKMDKTVANGQQNSSSASGGLHPNHGSSGQHGSTCPSLLFPHPSPSEQPATDDAQKQKLDDADKKIAEDNLKHMFSHS<br>EWLSSIYSRRSDAQAIKSLLYLERFWKSAREAVKAMLKMATMDKDKIEITKEHPVYINAQGALGTDFAIMGRTLNAPGDW<br>WASYGYEIPTLQRVAIRILSQPCSFHWCRWNWSTFETVHTKKRNKVEMEKLNDLVFVHCNLWLQTICQGRDGKCKPIIFD<br>EIDVSSEWPTESEPSVPLLDDSWLDNLPLECRGSP	zf-BED--<br>Dimer_Tnp_hAT<br>--	V
Gar02<br>G1603<br>0	Gar<br>Zf-<br>BED<br>06_I<br>I	MSDPKRDSSTQSSQNSSPSKDDQTTTNENVRKKITPRSACWNHFTKFVTKEGEKRARCNTCDVTYTMESTSGSTTNLN<br>NHLKTCLKRPRGNISNPKQSELAFVKVSQETTDLSTCVFDKDAVRRALVRMIIVDELPFKIVEGEGFKYFLSIACPRFSLPS<br>RWTIRRDCLDLFNSMKSVMKNCFEKI	zf-BED	II
Gar02<br>G1686<br>0	Gar<br>Zf-<br>BED<br>07_I<br>V	MSDSKRDGSTQSSQNSSPSKDDQTTTNENVRKKITPRASCWNHFTKFVTKEGEKRARCNTCDVTYTMESTSGSTTNLN<br>NHLKTCLKRPRGNTSNPKQSELAFVKVSQETTDLSTWVFDKDAIRKALVRMIIVDELPFKFVEGEGFKYFLSIACPRFSLPS<br>RWTIRRDCLDLFNSMKSVMKNYFEKDISRVCLTTDTWTSLQRISYMVLTAHWVDDEWRLQKRIINFCPISAHRGESIGQAI<br>EKCLRDWGIERVFTITVDNASANSVAIEYLRKKFNHRNASVANGKFIHMRCVAHILNLIVQYGIKDASVSVDRVRGAVRYIR<br>ASPSRLTKFNQRVKEEMIDSKAQLCLDVPTRWNSTYMMLKVAEKYERAFESYVRDDHNFFLDLTAGDGVPTFDDWEIVR<br>RVIKVLEPFYHLTLKVSGSLHVTSHSLFEVLTDVHCLFDGWQDCGDLEIISMTSKMREKYNKYWGEGNKINMLVYLAVIFD<br>PRCKMSFLDFGVNLLFPNVANDIIK	zf-BED--DUF-<br>domain	I<br>V
Gar03<br>G0593<br>0	Gar<br>Zf-<br>BED<br>08_I<br>I	MPPLEEFPTKGLEGAPSNYIGWHFGTPVPNTKRNVVCKLCGKVVKGGITRFKEHIAHKIGNVAPCPNVTGVIRESMMNILK<br>ESNTTKIDKRGEQMNSYLN	zf-BED	II
Gar03<br>G0621<br>0	Gar<br>Zf-<br>BED<br>09_I<br>I	MSIEPTSIEGSVTPPTSIDSENSGVGASSQANVTTGKRKATPQRLEVWSHFTKIINNEGASKAKCNYCQKEFCCDVKKNG<br>TGSLKYHIGSCKKNPSNVVDPSQGQLVLPRKGVEGGEGHLSTWRFDQDACRKGLAQMIVIDELPFKFVESEVFKKFMFV<br>ACLKFHIPSRITMIRDVYQLYLDERVKIKQLLRSSCSRVCLTTDMDFFAKS	zf-BED	II
Gar03<br>G2865<br>0	Gar<br>Zf-<br>BED<br>10_<br>VIII	MASSFSHTALRLLLSCAEAIENGDLKRADKFLHNILILADERPYYFADESRVVRYFADALVRRAYGLHPASSYFPFPVDPA<br>QYYHYNCYHINGVIKKVIDDALMEKNALMGNRRLHLIDFSIPYYIIEGSVLRTLPNYSGDPLSVRVSYIPPPFLKKQVKLSRQ<br>REFLTKEAKEVNVKWEDELKVVYANSLAEMDECELDFKGSRDDEMVVVYYKFKLHKLLRDAKAMERELVRLKEINPMIVI<br>MLDFYANRSDSNFLRCLEDSFQYYSNTCRFWGESDFGFFGYLWECACEGYEQCPIFFLSAWKPKVEDRHLNSTPSNDK<br>FEDFNPNPLPLQSLQPFSEGLTLNRLAALAEIYDILKHLCYRYELPLALTRTSKTNDMNETMSDPNKKHTFLIQSSYCYVKD<br>WNAYRFINSCKIDTKSIIDKALKSRDGYHFEPSIKLDKLDYSYPIGPKYYNIDVAVAICLQNCYTSNDVYGVEFYWPITESEK<br>SKSLALNIFNDWKLMKTKFVTVKVQGTQFRFQEEVISNIPTSSNTTRTLEIAEEARDVHAKEINGHIEQKGVIPNCNSPIPIH<br>SSSKVVAAPFNTLEGPYNKIIFNDDPVKAKRNKQRKLWSAVWEHFDRFEVKGKQVARCKDCRKECTGSIKSGTTHLKNH<br>LDRCPAKKKQNQERQLIFPAYTNERSSTFDKERSCFKLAKMIIKHRSPLDMVDQEFFKNFVKDMQPGFEFQLKEILFYINQI<br>YKEEKKKLQLYFDRLACKLNLTLSLWKNNHGKTAYCCLIAHFIDDGWEPKMKILGLRNLEHIYDTKVVGGIIRSFVSKWNIS<br>RKVCSITVDNSFLNDGMIHQIKENCVSEQGSLSSTHWFISFTLLEDGFRDMDGILSKLRKSIEYVTETTHGKLNFQEAAKQV<br>KLQGGKSWDDLSFKPESDSDILDSALRSREIFCKLEQIDYNFMLNLSMEEWEKAVTLQSCFKCFDDIKGTQSLTANLYFPK<br>LCNMYEEFGQLKKSNHPFINLMKRKFDNYWSLCNVAFTIAATLDPRLKFRSSCNETYDLENMIKLIRFRKVLVDVYFEYAN<br>EAKNLSASSSVLIDSNSLTTETTNDCIVSYFSKFASPSNVKVVASQKSELDCYLEETLLPSDADILGWWRVNSQRFPTLAK<br>MARDFLAIPVSVSAPCSHISAMTRNPAYSRLDPESMEALVCSQNWLESTKENDGEHHEPMQNMDKRKRKVEENGTSTV<br>KVFKNRNHEKASSNGDIASDFNKNDGNLSFDNWMEPQCSSSESVGEKAEIMEASVRNRDRLESSIGKPNHGRNIAAAIEI<br>PNDEPSFNTNQLDEFQSSSSESDDETTLREQRSWYREDVRTYLVSSFTNKEKKRLNRWETSELSGK	GRAS--zf-BED--<br>DUF-domain--<br>Dimer_Tnp_hAT<br>--	V<br>II<br>I
Gar03<br>G2866<br>0	Gar<br>Zf-<br>BED<br>11_I<br>X	MASSISDTALRLLLSCAKAIEDGDLKSADALLHIILVLADERPYLYESRLVKYFADALVRRAYGLHPASSYNTFPGNPAPYY<br>HYNGYRINDIIKKVIDDALMGNRRLHLIDFSIPYENFEGSVLRTLPTFSGDPLPVRVSYILPPFLKKYVESFSQMEFLTKDAM<br>NLNVKLEAELKVVYANSLAEVDEYKLDFKRRREDEMVVVYYKFKLDKLLTDGKAMERELVRLKEINPTIVIMLDFYSNHTH<br>SNFLTCLEHSFQYYSNTCTFWCRYGIFLYSKYEWECNRDASEGNNIIRRHQTLSEWQRLFSIAGFTRIPLSHEKDDLIDFFF<br>FDDSSFLEIMREEEECLILGYKECPMFFLSAWKPKVEDGHFNSISTNHQFGQGFNPNPLPLQPLQPFLEGFALNRLAAFTE<br>IHDISKYLCCKYKLSLALTSASKVNNMNKPFWGPNKEFPFFIQSNSCYVKDLKSYQFMNHVVEVINSLIIEKAFESRDGYHF<br>EPSITKLDLEYYPYALVKNYNIDVVVAICLQNRHTSNEVYIVQFYWPPTESEISKSLALRIFDDLKHMKTTFVSVKVQGTEIK<br>FQEEAISSIPTSSNTAMPLKIVEEARNIDAIEINGHIEQVVAAPFNTLEGPYNQIGKTKGNKQRKSWSKVWVDFDKFEENGK<br>QVAKCKHCPKVLTGSSKSGTTHLNNHSKVCPGKKKQNQETQSILPVDTNERSSTFDQERSHLDLVKVVIKHQYPLDLAG<br>QEAFKNFVKGLQPMYEFQSRDKLLSDIHRIYNEEREKLQLYFDQLACKLNLIVSLWKNNHGKTAYCCLIAHFIDDGWELKM<br>KILGLRKLEHVYDTKVVGGIIRSFVSVWNIRKKVCSITVDNSFLNDGMVHQIRENCVREQGSLSSAYWFISFTLLEDGFREM<br>DSILSKLWKSIEYVTETTHGKLNFQEAVNQVKLQGGKSWDELSFKLESDSDILDSALRSREIFCKLEQIDDNFMLNLSMEE<br>WEKAATLQSCFKCFDDIKGTQSLTANLYFPKLCNMYEEFGQLKKSHPFVILMKRKFDNYWSLCNVAFTIAATLDPRLKFRS<br>SCDETYDLENTMKLRRFRKVLMDVYFEYANEAKNLSASSSVLDDSNSLTAETTKDCIVSYFSKFASPSNVKEVASQKSEL<br>DCYLEETLLPSDADILGWWRVNSQRFPTLAKMARDFLAIPVSVSAPCSNISAMTINPAYSSLDPESMEALVCSQNWLEST<br>KEKDGEHHEPMQNMDKRKRKMEENGTSTVKVFKNRNHEKASSNGDIASDFNKNDGSLSFDNWMEPQCSSSESVGEKA<br>EIMEASVRNRDRLESSIGKPNHGRNIAASIEIPNDEPSFNSNQLDEFQSSSSESDDETTLREQGSWCREDVRTYLVSSFT<br>NKEIKRLNRWKKSELSRKLIGRDKEFKLVGKKLKPLLMVPYCDETLIEYYIDDSVVNAFFKLLKGRSDKYPNVYIKNYSFDP<br>QIATCLIKGSKLEDEVLGWFKAEKLRGVHKLFLPMCLSAHWVLFCVDTREKKISWLDPIPSSRIKSNNVEKQIILQWFTSYL<br>LPEFGYNDADEWPFVVRTDIPKQENSIDCGVFVMKYGDCLTHGDFFPFTQKDMIHFRHRIFLDIYRGRLHGKR	GRAS--zf-BED--<br>DUF-domain--<br>Dimer_Tnp_hAT<br>--<br>Peptidase_C48-<br>-	I<br>X
Gar03<br>G2867<br>0	Gar<br>Zf-<br>BED<br>12_<br>XI	MLEEVCFRYELPVALTWACEATTDKIMLDGKKHTLFMERTSCYASNEGSQCFMEACAKHHIQEGQAIAGKAFQSSANFH<br>FKPSITKLMKSDYPLFNAAQLFGSHAVVAICLQNHYIIGDVYVVEFYWPEIESEKSESLALDIFSDLKNMKKKFVTIRVGGNE<br>VGFEREAISTTLQGTMHMRNAQPASSTNDLLSSNTTWSLNAVQQCDVHEMERHGLVEQVESAPFSTPNPMSYGGVLQT<br>QGPHKQEIGDKDFISQTVSIGDYEIVKAYMETCKVPRTKRRKYSSKVWLDFDKFEVNGKQVAKCKHCNKDFTGSSKSGT<br>THLKNHLERCQSDLITRDSDESNFTFDQERSRLDFAKMIIKHQSPLDMAEQEFFKIFVKNLQPMFEFQSKDILLSDIHRIYKE<br>EKEKLQLYFDQLACNFNLTISLCKNNHGKTAYCCLIAHFIDDNWEPKMKIIACKALEHIYDTKALNEIIQSSVLEWNISKKVFS<br>ITMDNPFLNDDMFQKIKETCFSDQGSFPSTHWFIGCTFIEDGFREMDLILLKLRKSIEYVSEIAQGKLKFEEVVNQVKLQGV<br>KSWDDLSLRLDSDFGVLHSALESREIFCQLEKIDGNFKLNPSVEEWEMVLAFHSCLKCFDDIEGTQSLTANLYFPKLCNIY<br>KKFLHLEKSNYPIVTLVKRKFDYYWSLCNSAFAVATILDPRLKFKFVEFSYTEIYGHDSKMHLNRFHKVLTDVYYEYANEAR<br>NLSKSTSDLDDSNSSTTEIVNDCILESFSKFASANNFNEVASWKSELDCYLDEPLLPLDGAFDILYWWCINTKRFPTLAKM<br>ARDFIAMPISILAPCLNFNAMITNPTYNNLNPESMEALVCSQNWLKIPKENDGENHGPMQNMYKRKRKMEDHSNVVKVSK<br>NWNREEANSSGDIAKGSIKNENIEASVCNRNRLEISTGKPNHGRNITALIEIPKDDSPFSNNKSGQFQSLSSESDNETTLKE<br>QGSWCKEDVRAYLVSRFTGKENKRLNRWQTNELIGKLIGRDKEFCLMGDKLAPLLMVPHGDETRKEYYIDDCVVNTFFK<br>LLKKRSDRFPKAYINHYSFDSQIATCLIQGSRSEHEVLAWFKAKKLRGAHKLFLPLCLSAHWVLFYVNTKEKKISWLDSNP<br>SSRIMSNNVEKQTILQWFTTFLLPEFGYYDANEWPFLVRTDIPVQKNWVDCGVFVMKYGDCLTHGDFFPFTQNDMVQTS<br>LVHMYCRCGRIDKAEVLGGVLYKDLAVWIPSHSRLVEDGLKYFKSMKDNFGIKPGIEHYTCLVDLLGRVGHFNLALKIENH<br>PRDAYASTSSSLGSIA	zf-BED--DUF-<br>domain--<br>Dimer_Tnp_hAT<br>--<br>Peptidase_C48-<br>-	X<br>I
Gar03<br>G2872<br>0	Gar<br>Zf-<br>BED<br>13_<br>VIII	MDFDLFDTETALRLLLSSAEAIEDGDLKRADELLQKILILADQSTFGFGDQRRVVKYFADALVRRAYGLHPASSYFTFPVDP<br>APYYHYNSYYINYVIKKVIDDEKNASNGNRRLHLIDFPVPYDNNYFEGSVLRTLTTFSGDPLPVRVSYILPPFLKKYVEFSR<br>KIMEFLTKDAMNLNVKLENELKVVYANSLAEVDEDKLDFKTRREDEMVVVYYKFKLHKLVRDAEAMKREFVRLKEINPTIVI<br>MLDFYSNHSDFNFLTCFKDSFQYSSNTCTCWGEADSGFGRNEYWWECNRDACEGNNLIRRHQTLSEWQRLFSMGGFS<br>RIPLNHTKDDLSNEEECLLLTERHILDNQTSLKIMGKEEECLILGYKGCPMFFLSAWKPKVEDGHFNSTSTNHQFRQDFNP<br>NPLPLQPLQPFSEGLILNRLAALAEIHDISKDLCCKYKLSLALTWASKVNNMNETISDPNKKHGLRFMEKLEFNMTEDKFIP<br>MYDIVTICLQNLYTSNDVYVVQFYWPAKMIEISKSLALRIFNDLKHMKKMFVEVKIQGTEIGSQKEVIPNISTSRCLQAVEET	GRAS--zf-BED--<br>DUF-domain--<br>Dimer_Tnp_hAT<br>--	V<br>II<br>I

		EEVQAVEINGVHVERGVDSNFPSPMPTHPSSKVVAAPFNTPEGPHNPIVSNGDPETGKANKEKLPITEQRSLTSEVWEHFDRLLVGGEQVAKCKHCSNVLTGESTSGSTHLKNHLNYRCSVMKKQNQERSRLNFVKMIIKHRYPLDMADQEFFKNFVKDMQPMFEFESKDISSYMRSIYMEEKEKLQLYFDKLASKFNLTVSLWKNNSGQTAYCCLIAHFIDDSWELKMKILGLRTLEHNNDTKAVGGIIQSLVSEWNIGSKVCSITVDNSMSDDSMVDQIKEICLSDQGSVSSDHWFISFTLLEDGFREMDGILFKLRKSIEYVTDTRHGKLKFQEAVDQVKLQVGKSWDDLSFRLESDLDILDSALRSREIFCKLEQIDGNFKLNPSMEEWENAVAIQSCLKCFDDIKGTQCLPVSLYFPKLCDTYKKLLQLEKSSHSFVTLMKRKFDRYWSLCNLALAVASVLDPRLKLKIVELSYRVIYGHDSKMRLNMFHKVLRDVYYEYASEAKNLTTSSSVLDDFNCSTVGLGNYSILDSLSKFASASNFNEEASWKLELELYLDEPLLPMDGAFFDILGWWCDKSQRFPILAKMAQDFLAIPVSISTSCSNISAMINNPAYSSLNPESMEALVCNENWLETPKGNDGENNEPTQTTDKGKRKLNEDSCFRKKSKPSNCEKAISIEDIVKDSNNNGN		
Gar03G28750	GarZf-BED14_VIII	MASSVFDTDTDTALRLLLSCAKAIEDGDLKSADAFLQNILILADERPYLYKSRVVKYFADALVRRAYGLHPASSYFTFPVDPSPYYHCGSYLINGVIENVIHDALMEKNALMGNRRLHLIDFSIPYSSFQNSVLRTLPTFSGDPLPVRVSYILPPFLKKYVKFSHQMEFLTMDAKEVNVKLEDEFKVVYGNTLAEVDEREIDFKRRRDDEMVVVYYKFKLGKLVRDAKVMERELVRLKEINPTIVIMLDFYSNHAHSNFLTCLEDSFQYSLKTVDCWAELDLYFDEEYEWECHIEAWEGNNVIRRHPTLTEWQHLFSMAGFSRIPLNHRKGIDLIVKNVNPLNDFFSTWNQSWLEIMGKEEECLIIGYKECPMFFLSAWKPKVEDEHLNFNSSNDKFGQGFNPYPSPLRPLQPFPEGLTLSRVAALAEIHDILNHLYCEHKFSWALTWASKVDNMNETMSDPNKKYTFTIQSNSCYLKDLNSYKFMRSCEYKIEETIIEKALESKDGYHFEPSITRFDIDDYMYLQRAKNCDVDVVVAICLQNRYLCNDVYVVEFYWPTTESEISKSFTPRIFNDLKYMEKKFVTVKVQSTEKAISNIPTSSYTARPLKIAEETEDVDAVEINGVNVQRGVIPNFHSPLTIQSSSKVVAAPSNTLEGPHNQIFPNGDPEIVKANKEKPSKATQRKLRSKVWDHFDGFEEDENQVAKCKHCPKMLTGSSKSGTTHLNSHSKVCPGKKKQNQESQLILPVDTNEGSLRFDKKRSHMDLAKMMIKLRCPLDMAEQETFKNFVKGLQPMLEFQSKDILSYIHHIYDEEKEKLQLYFDKLASKFNLTVSLWKNNAGKTTYCCLISHFIDDGWELKRKILALKTLEHINDTKALGENIRSLVLEWNISNKVCSITVDNSFLNDSMVDQIKEICLSDQGSVSSDHWFITFTLLEDGFREMDGILFKLSKSIEYVTETRHGKLKLQEAVDQVKLQGGKLWDDLSFRLESDFDILDSALRSREIFCELEQIDENFKLNPTMEEENAVALQSCLKCFDDIKGTQCLPVSLYFPKLCDAYKKFLQLEKSSHSFVTLMKRKFDRYWSLCNLALAVASVLDPRLKFKIVELSYRVIYGHDSKMRLNMFHKVLRDVYYEYASEAKYLTTSASVLDDFSCSTIGLGNDSILDSLSKCTSASNFNEEASWKLELELYLDEPLLPMDGAFFDILGWWCDKSQRFPILAKMAQDFLAIPVSISTSCSNISAMINNPAYSSLNPEIMEALVCSENWLETPKESC	GRAS--zf-BED--DUF-domain--Dimer_Tnp_hAT--	VIII
Gar03G28760	GarZf-BED15_XXII	MASSFFDIDIDTALGLLLSCAEAIEDGDLKSADKFLHNILILADERLYLYKRGVVKYFADALVRRAYGLHPASSNLTFPVDPSPYYHYNSNRINGVIENVIHGALMEKNALMGNRRFHLIDFSIPYYDSQNSVLRTLPTFSGDPLPVRVSYILPPFLKEHVKFSHQMEFLTKDAKEVNVKLEDELKVVYANSLAEADEFEIDFKRREDEMVVVYYKFKLDKMVRDAKAMERELVRLKEINPTIVIMLDFYSNHTHSNFLTCFKDSFQYSLKTLDFWAQLELFLEEEYEWDCNIEAWEGNNVIRRHPTLTEWQHLFSMAGFSRIPLNHRKDTLSVEDEDLLGIMGEEEECLILGKEGCRMFFLSAWKPKVEEEHLNFNSSKDKLGQGFNPFPSPLPPLQPFLEGLALNHVSALAEIYDILNHLCCKYKFSWAITWASKININQTMSNPNKKYTFLIQSNSCYLKDFNSFEFMHSCEYEMYFGKYEIEQTIIEKALESKYGYHSITNLDGEDNPYPVFVKQYDIDVVVAICLQNRYTSNDVYVVEFYWPATESETSKSLAPRIFNDLKHMEKKFVTVKVAGTKKAISNIPTSSNTSRRLIIAEETEDVDAVEINGVNVEIFPNGHPEIVKAIKEKPSKATQRKLRSKVWDHFDRFEEDGKQVAKCKHCPKVLTGSSKSGTTHLNNHLKVCPGKKKQNQESQLILPVDTNEGSLRFDKKRSHMDLVKMMIKLQCPLDMAEQETFKNFLKGLQPMFEFQSKDILSYIHHIYDEEKEKHQLYFDKLASKFNLTVSLWKNNSGKTTYCSLTSHFIDDGWELKRKILALKTLEHINDTKALGEIIGSLVLEWNISNKVCSITVDNSFLNDSMVDQIKEICLSDQGSVSSNHWLISFTLLEDGFREMDGILFKLRKSIEYVTETRQGKLKFQEAVDQVKLHGGKYWDDLSFRLESDFDVLDSALRSREIFCKLEQIDENFKLNPTMEEWENAVALQSCLKCFDDIKGSQCLPVSLYFPKLCDIYKKFLQLEKCHSFVTLMKRKFDHYWSLCNLAFAVASVLDPRLKFKIVELSYRVIYGHVSKMQLNKFHKVLRDVYYKYASEAKSLTTSASVFDDFDCSTIGLGNDSILDSLSKFASASNFNEEAPWKLELELYLDEPLLPMDGAFFDILGWWCDKSQRFPILAKMARDFLAIPISVSTPCSSISAIINNPAYSSLNPESMEALVCSENWLETPKENDGENHEPMQITDKRKRKMDEDSHPVKNSKPSNLEKAINTEDIAKDSNNNDFSLSFDNWMEPQFSSSESIGEKAEIMKALVCNENRLESSIGKPNHEKNVDVVIEIVNNDPLFDNNQSDEVQSSSSESEDEATLKEQGPWCEQDIKAYLLSRFTSKEHKRLDKWQRNELNGKLIGRDKKFKFQGEILAPLLMVPQSDETRKECYINDSVVNAFFELLKKRSDEFSNTYINHYSLDSQIAAQLIKGCRSELEVLNWFKAEKLRGVHKLFLPLCLSSHWVLFYVDIKEKKFSWLDPDPSSLILSYHFENHVKVLLQWFTSFLLPKLGYIDANKWPFIVRNDIPKQKKICGSFGDLNFGCHLCFPPLRLRLVFEKSLKIDEFVEEIPGFWEEDRESFSGSNCPYPFSSREKGDGYWVPPRTQMEVGEQFGGNTRNLLFEDPFNYSELMNLDAYGGWCSSSATTDQMFASFGFFSYPSMSHASFDSTNSIGQSSSTYVESGYALGGMDSSYNCADRTVFLKTDEHFGNTVDSKGVELGVRQHTDGNKQNDTSYLGNLLISRPIGRSLDEKMLRALSLFKDLSGEGILAQVWVPIKHGDQYMLTTSDQPYLLDQMLLGYREVSRTYTFSAEQKPGSFPGLPGRVFLSRVPEWTSNVIHYSKAEYLRITHAVNHEVRGSIGLPVFQPPEMSCCAVLELVTTKEKPNFHSEMEHVCHALEAVNLRTIARPRFLPQCLSKNQRAVLAEITDVLRAVCHAHSLPLALTWIPCNYTEEAENEYIEMRVREGNKSWDGKCVLCIEDTACYVNEREMQDFVHACVEHYLEGQGIAGKALQSNHPFFSADVKTYDVSGYPLVHHARKFNLNAAVAIRLRSTYTGDDDYILELFLPINMKGSLEQQLLLNNLSGTMQRICRTLRTVSDAEIVGERSKFDFQSVTVPTFPPMAMSQRSSETALSADSDMNSNHSIPFNESNSISDGKEADGPPEKAISGLPRQKRSTTEKNVSFSVLQQYFSGSLKDAAKNIGVCPTTLKRICRQHGISRWPSRKINKVNRSLKKIQTVLDSVQGVDGGLKFDPATGEFVAAGTVIQEHNPEKTLMFSNKNPPIGISEAFNHEKSSAPLASCLDGEDSVVKWDENECSFGGNNNEAARSLLIPSICQEVKKSIVPLNDCSEDSKSVAGAASWICPKNATIGSYFRQGDNMWGLNKGNPKVEIADCHFVSLNSSSLAATDEMDTRKEGDDGIDEYNRQHTSSSMTDTSNVSGSRLHGSSSSSEKSMEAKYSRMKTCVDSSSKITIKATYKENTVRFKFEPSAGCFQVYEEVAKRFKVQNGTFQLNYLDDEGEWVMLVSDSDLHECLEIMEYVGTRSVKFQVRDVPCTMGSSSSSNCFLSRNS	GRAS--zf-BED--DUF-domain--Dimer_Tnp_hAT--Peptidase_C48--RWP-RK--PB1--	XXII
Gar03G29280	GarZf-BED16_I	MDNFDQKLGPEFFKNLSAEAVTPLNVVHEEIYESSSKRPKTTSKVWDIFEKLPAQQGDSKAICKLCRRIYTAKTTSGTSHLRRHIEACVKRGNHEVDQRSIEACFKPVKRNANRLTLSHDTLISATTSLKNYKLDVDEIHRAIAMMIIVDEQPFSVVEDAGFRRLLSAACPEFPVLSRSSIKRDIISIYVKERENIRELLATCPGRICLTSSTWKSDSDDHFNCVTTHFIDHEWRLQKRILRFKLMPPPYDSLSVADEIALCMVQWNIEHKVFSVTLENLSSDDCVADMLRSRLAAKKYLPCKGVFFHVSCFFRILNSIVQAGLNLVVDIIAKLRLGIKYVQQSPHRKKNFYIVAKTLNLDTQRKLCLDTPARWNSTYDMIEVAFCYKNAFVYLAEQDKNFLHKLSEDEWEKMSVLYKFLKVFYEVTCVFFRNRQPTSNLYFKAAWKVHSRLFDMVRGPENFMTRMVREMHSKLNHYWSAYNLILSCAAILDPRYKIKFVEYCYTKLYGSGAQKYVSVSVNTLYGLFDEYMQTSARPSQTTLLSTAASKISNDKDENDGFEDYETFQSARFRTQVEKSQLDLYLEEPSHDLNSEIDVLEYWTLCSLRYPELSKMARDVLTIPVSTIASDSAFDITPQVISADRSSLKPKMLQALVSLQDWMLASDRTRGLGSMESKPEDDSSSSSDGDDDY	zf-BED--DUF-domain--Dimer_Tnp_hAT--	I
Gar04G04360	GarZf-BED17_I	MDMSDAVIVNSSRLKSIVWNDFDRVKKGDTFVAICRHCKKKLSGSSTSGTSHLRNHLIRCQRRSNHGVAQYFSAKDKKKEGSLALVTIDQEQKNDEVLSINLRYEQEQIKSEHVGIGSNSLDQRRSQFDLARMIILHNYPLAMVEHVGFKIFVRNLQPLFELATRNKVEADCMEIYAKEKQKVYEIFDKLPGKISVSADVWTASEDDAAYLSLAAHYIDENWQLKKKNLNFVTIDPSYTEDMHSEVIMNCLMDWDIDRKLFSMIFDSFTSDNIVERIRDRLSQNRFLYCNGQLFDVRCADLLNRMAHDALEALCEITQKIRESIRYVKSSEATQATFNELADEVQVETKKCLCIDNPLKWNSTYFMLEAALEYRKVFSCLRDRDPVNMKFLLSDPEWDRLITVTSFLKLFVEVTNVFTRSKYPTANIFFPEICDIHLQLIEWCKNPDEYISSLALKMRRKFEEYCYKCSSGLAVAAMLDPRFKMKLLEYYYPQLYGDSATELIDDVFECIKSLYNEHSIVSPLASSIDQGLDWQASGISGSGKDSRDRLMGFDKYLHETCQAEGSSSDLDKYLEEPLFPRNVDFNVLNWWKVHTPRYPILSMMARNILGIPISKVAAESRFDTGGRVLDHNWSSLPPTTIQALMCSQDWIRSELES	zf-BED--DUF-domain--Dimer_Tnp_hAT--	I
Gar04G06050	GarZf-BED18_II	MPPREEFPTKGLEGAPSNDIGWHFGTPVPNVKGNIVCKLCGKVVKGRITRFKEHIADKTGNVAPCPNVTSVIRESMMNVLKENNTKKIDKKRRKDDFLSQLREEEDEHEEFIDEISAIRQATRESIQSQHECHRREEFRRSTGGWDNIYEKGQSSQGSSIPTESEFTLRGTIPELVRSKSSKQPKVSDSFLKSFRKKIEVGQGVKLPTPYEVSDVYLESEYQRVHNWLLLFRDKHETFGTPQAQRAWKQMNPDGENAGVLPVDTSDDEMDVNQSQQQNLSHSSSSSMPSQSGDGPDGGGLSPVDEDDGYNGDRGEIRSSSQYRGEYGVGTTSRHFRDRSEFDGNMFPEPRRDRSEPRAPSKGKGKKHTSIGSSSGRRSGSSNLGYSDSSTSTQGFYPPEQPSYFQPSHGYPQPYGYYPPFPNYGVPYQPQMHPLPPMYHPPPPLMYPPPQIYPPHQLYENQCENVTFFGYIFGQRPRESSQERSQSEGDGSDLPRHSTNW	zf-BED	II
Gar04G07790	GarZf-BED19_II	MSTKPTFIKGSVTPPTLIDSENSGVRASSQTKGATGKRKVAPQRSEVWSHFTKIINSEGASKAKCNYCQKEFCCDMKKNGTGSLKYHIGSCKKNPSNVVDTSQGQIVLPRKGVERGEGHLSTWRFDQEACRKGLAQMIVIDELPFKFVESKGFNKFMVVACPRFHIPF	zf-BED	II
Gar04G09680	GarZf-BED	MPLHEEFPTKGLEGAPSNDIGWHFGTPVPNAKGNIVCKLCGKVVKGGITRFKEHITHKTCNVAPCPNVTSVIRESMMNILKESNTKKIDKKRRKNDFLSQLREDEDEHEEFCYKASNSRKYPITT	zf-BED	II

	20_II			
Gar04G15440	GarZf-BED21_II	MSTEPTSIKGSVTPPTSIDSENSGVGASSQANVTTGKRKATPQRSKVWSHFTKIINSEGASKAKCNYCQKEFCCDVKKNGTGSLKYHIGSCKKNPSNVVDPSQGQLVLPRKGVEGGEGHLSTWRFDQEACRKGLAQMIVIDELPFKFVESEGFKKFMFVACPRFHIPSRTTMTRDVYQLYLDERVKIKQLLKSSCSRVCLTTDTWTSLQRVNYLCITAHFIDNDWKLNKKILNFCPISSHKGESIGMVIEKCLLNWGLISCLLLLLIMQVQMMLLLVI	zf-BED	II
Gar05G35140	GarZf-BED22_II	MPRLRNKEDAAPSDDYGWRWGEPVEGNHNNVKCRFCGRFIKGGITRLKEHLAAKKGNVARYPHVSVEVRKTIVQHQEYHSEKAAKQRRKEELEERIRLGDRGDYGDSGGGDDEELTIARRESMISQVE	zf-BED	II
Gar06G06660	GarZf-BED23_I	MTEMTEIADMETIPGESNNQLALTTPEAQPIKRRKKKSMVWEYFTIENVSAGCRRAYCKRCKQSFAYSTGSKVAGTSHLKRHIAKGTCRALLRGQGQDNNQFITPYNPKMGGSEPPKRRYRSPSSPFIPFDQDRCRHEIARMIIMHEYPLHIVEHPGFIAFVQSLQPQFDKMSFNTVQGDCVATYLREKQSLMKFIEGIPGRFCLTLDMWSSNQTLGYVFITGHFVDSDWKLHRRVFNVVMEPYPDSHSALSHAIAACISDWSLEGKLFSLTFNHPLSEAGLENLRPLLCVKNPLILNGQLLIRNCIARTMSSMAKDVLGAGQEIIKKIRDSVKYVKMSESHDDKFIQVKNQLQVPSEKSLFLDNQTRWNTTYQMLAAASELKEVFDCLDTYDPDYKLAPSMEDWKLAETLCSFLKPLFDAASILTTTTLPTVITFFYEVWKIHVDLGRSITSEDPFISNLAKSMQEKIDKYCKDCSLVLAMAVVMDPRFKMKLVEFSFTKIYSEDAPTYIKTVDDGIHELFLEYVALPLPLTPTYAEEVNGANNGKTNESHYGNLLSDHGLTDFDVYIMETNSQQMKSELDQYLEESLLPRVQEFDVLGWWKLNKMKYPTLSKMARDILSIPVSAAAPESIFDITDKQLDEYRSSLRPETVEALICAKDWLHFGSSDVSNALVKMEF	zf-BED--DUF-domain--Dimer_Tnp_hAT--	I
Gar06G06670	GarZf-BED24_I	MTEMTEIADMETIPGESNNQLALTTPEAQPIKRRKKKSMVWEYFTIENVSAGCRRAYCKRCKQSFAYSTGSKVAGTSHLKRHIAKGTCRALLRGQGQDNNQFITPYNPKMGGSEPPKRRYRSPSSPFIPFDQDRCRHEIARMIIMHEYPLHIVEHPGFIAFVQSLQPQFDKMSFNTVQGDCVATYLREKQSLMKFIEGIPGRFCLTLDMWSSNQTLGYVFITGHFVDSDWKLHRRVFNVVMEPYPDSHSALSHAIAACLSDWSLEGKLFSLTFNHPLSEAGMENLRPLLCVKNPLILNGQLLIRNCIARTMSSMAKDVLGAGQEIIKKIRDSVNLFLDNQTRWNTTYQMLAAASELKEVFDCLDTYDPDYKLAPSMEDWKLAETLCSFLKPLFDAASILTTTTLPTVITFFYEVWKIHVDLGRSITSEDPFISNLAKSMQGKIDKYWKDCSLVLAMAVVMDPRFKMKLVEFSFTKIYSEDAPTYIKTVDDGIHELFLEYVALPLPLTPTYAEEVNGANNGKTNESHYGNLLSDHGLTDFDVYIMETNSQQMKSELDQYLEESLLPRVQEFDVVGWWKLNKMKYPTLSKMARDILSIPVSAAAPESIFDITDKQLDEYRSSLRPETVEALICAKDWLHFGSSDVSNALVKMEF	zf-BED--DUF-domain--Dimer_Tnp_hAT--	I
Gar07G08480	GarZf-BED25_IV	MSDSKRDGSTQSSQNSSPSKDDQTTTNENVRKKITPRASCWNHFTKFVTKEGEKRARCNTCDVTYTMESTSGSTTNLNNHLKTCLKRPRGNTSNPKQSELAFVKVSQETTDLSTWVFDKDAIRKALVRMIIVDELPFKFVEGEGFKYFLSIACPRFSLPSRWTIRRDCLDLFNSMKSVMKNYFEKDISRVCLTTDTWTSLQRISYMVLTAHWVDDEWRLQKRIINFCPISAHRGESIGQAIEKCLRDWGIERVFTITVDNASANSVAIEYLRKKFNHRNASVANGKFIHMRCVAHILNLIVQYGIKDASVSVDRVRGAVRYIRASPSRLTKFNQRVKEEMIDSKAQLCLDVPTRWNSTYMMLKVAEKYERAFESYVRDDHNFFLDLTAGDGVPTFDDWEIVRRVIKVLEPFYHLTLKVSGSLHVTSHSLFEVLTDVHCLFDGWQDCGDLEIISMTSKMREKYNKYWGEGNKINMLVYLAVIFDPRCKMSFLDFGVNLLFPNVANDIIK	zf-BED--DUF-domain	IV
Gar07G15520	GarZf-BED26_IV	MSDPKRDSSTQSSQNSSPSKDDQTTTNENVRKKITPRSACWNHFTKFVTKEGEKRARCNTCDVTYTMESTSGSTTNLNNHLKTCLKRPRGNISNPKQLELAFVKVSQETTDLSTWVFDKDAVRRALVRMIIVDELPFKIIEGEGFKYFLSIACPRFSLPSRWTIRRDCLDLFNSMKSVMKNYFEKDISRVCLTTDTWTSLQRISYMVLTAHWVDDEWRLQKRIINFCPISAHRGESIGQAIEKCLRDWGIERVFIITVDNASANSVAIEYLRKKFNHRNASIANGKFIHTRCVAHILNLIVQYGIKDASVSVDRVRGAVRYIKASPSRLTKFNQRVKEEMIDSKAQLCLDVPTRWNSTYMMLKVAEKYERTFESYVRDDHNFFLDLTAGDGVPTFDDWEIVRRVIKVLEPFYHLTLKVSGSLHVTSHSLFEVLTDVHCLFDGWQDCGDLDIISMTSKMREKYNKYWGEGNKINMLVYLAVIFDP	zf-BED--DUF-domain	IV
Gar07G18290	GarZf-BED27_II	MSPREEFPTKGLEGAPSNDIGWHFRTPVPNTKENIVCKLCGKVVKGGITRFKEHIAHKTCNVAPCPNVTGKQLEKVSNHNTSDIEGKNSDEVLVVGIPFMKKGDLIMDQLENVIEKEQVNPSQVSLSLP	zf-BED	II
Gar07G24040	GarZf-BED28_II	MPPREEFPTKGLEGAPSNDIDWHFGTPMPNAKGNIICKLCGKVVKGGITRFKEHIAHKIVNVAPCPNVSLEKV	zf-BED	II
Gar08G11360	GarZf-BED29_I	MAEMTEATNMETSPVENNNELALITPETQPKRRKKKSMVWEYFTIETVSAGCRRACCNRCKQSFAYSTGSKVAGTSHLKRHIAKGTCPALLRDQYNNQLTPYSPKTGGGEPRKRRYRSPSSPFIPFDQDRCRHEIARMIIMHEYPLHMVEHPGFIAFVQNLQPRFDKVSFNTVQGDCVATYLREKQSLMKLIEGIPGRVCLTLDMWTSNQTLGYVFITGHFIDFEWKLQRRVLNVIMEPYPDSDSALSHAVAACLSDWSLEGKLFSLIFNHPTSEAGLENLRPLLCTKNPLILNGQLLLGNCIARTLSSMAKDVLGAGHEIVKKIRDSVKYVKTSESHDEKFVQVKNQLQVPSEKSLILDNQNQWNTTYQMLAAASELKEVFNCLDTSDPDYKLAPSMEDWKVAETLCSFLKPLFDAASILMTTTNPTAITFFHEAWKIHADLGRSIANDDPFISNIAKSMLEKIDKYWKDCSLILAIAVVMDPRFKMKLVEFSFTKIFGEDAPTYIKIVDDGIHELFLEYVALPLPLTPTYTEEGNVGNNGKTNESQQGNLLSDQGLTDFDVYIMETSSQQMKSELDQYLEESLLPRVQEFDVLGWWKLNKMKYPTLSKMARDILSIPVSAAAPDSVFDIIIKQLDEYRSSLRPETVEALICAKDWLHHGSEESNALVKMEF	zf-BED--DUF-domain--Dimer_Tnp_hAT--	I
Gar08G30070	GarZf-BED30_I	MEVANETVIKKPKRLTSVVWNHFERVRKADLCYAVCVHCNKKLSGSSNSGTTHLRNHLMRCLKRFNYDVSQLLSAKKRKKDNTLTIANISYDEGQRKEEYLKPTIVKYEPEQRKDEVFNVQSSWFDQERSRLDLARMIILHGYPLAMVEHVGFKVFVKNLQPLFDVVPNSTVELSCMEIYGKERQKVHDMLSKLQGRINLAVEMWSSPENTNHVCMMAHYVGDDWKLQKKILNFVTLDSSHTNDLLSGVIIKCLMDWDIGSKLFAVTLDDFSTNDDIVLRIKEQISENKSRLSNGQLLDVRSAAHVLNSIVQDAMEALRLVIQKIRGTVRYVKSSQSIQGKFKEMVLQTGINSQKNLVLDCPIQWNSTYLMLETAIEYRNAFCQLPELDLDLALSDEEWEWASSITGYLKLFIEIINVFSSNKCPTANIYFPEICHVHIQLIDWCKSPDNFLSSLAAKMKAKFDKYWSKCSLSLAVAAILDPRFKMKLVEYYYSQIYGSTALERIKEVSDGLKELFNTYSICSTLMDQGSALPLSSLPSSSNDGRDRLKGFDKFLHETSQSQTAISDLEKYLDEPVFPRNCNFNILNWWRVHTPRYPILSMMARDVLGTPMSTVSQESAFHAGGRVLDSCRCPLTPETRQALICTQDWLRMQSDDTGPSSSHYALPLYVETN	zf-BED--DUF-domain--Dimer_Tnp_hAT--	I
Gar09G11510	GarZf-BED31_II	MPPREEFPTKGLEGAISNDIGWHFGTPMPNPKGNIVCKLCVKVVKGGITRFKEHIAHKTSNVAPCPNVTSVIRESMTNILKESNTKKIDKKEEKR	zf-BED	II
Gar09G22550	GarZf-BED32_XIX	MSRYRAQQQASVDEPEPNPQGEDHVPDDEQQDENDKDSQPSEPKGDYLNVASRPFLMKILQKQGDSKVLFADKVLKFTASGKMKRRNLIITDFAVYIVDPETDGLKRRIALAAVDKMCLSDLNDNFFSIIIPTEYDLLMASTRKTEIATCLFEAIKTSAQYELEVSFSSSFEYNATADLVKEIQFEEVEVVRERDVCWEYAEKLDGNKVRCKFCLRVLNGGISRLKHHLSRLPSKGVNPCNKVRDDVTDRVRAIISSKEDIKEIPSVKKQKIAEVRAPGNMSTGSKISPLETLSPAAKVFPTVLSIAASTLSDQETVERSIALFFFENKLDFSVARSSSYQAMIDAVGKFGPGLIAPSVETLKTTWLKRIKSEVTLHLKDAEKEWATTGCTIIADTWTDNKSKALINFLVSSPSRTFFHKSVDASSYFKNTKCLADLFDSVIQDFGQENVVQIIMDSSFNYTGISSHILQNYGTIFLSPCASQCLNLILEEFSRVDWVNRCILQAQTVSKFLYNNASMLDLMKKFTGGQELIRTGITKSVSCFLSLQSTLKQRSRLKHMFNSPEYSTNSSYANKPQSISCIAIVEDNDFWRAVEECVAISEPFLKVLREVSGGKPAVGSIYELMTRAKESIRTYYIMDESKCKTFLDIVDRQWRDQLHSPLHSAGAFLNPSIQYNPEVKFLGSIKEDFFKVLEKLLPTPELRRDITNQIFTFTRAKGMFACNLAMEARDTVSPGLWWEQFGDSAPVLQRVAIRILSQVCSTFTFERHWSTFQQIHTEKRNKIDKETLTDVVYINYNLKLAREMKTMPTDSDPIQFDDIDMTSEWVEESENPSPTQWLDRFGSALDGGDLNTRQFSAAMFGNDHIFGL	Myosin_TH1--zf-BED--DUF-domain--Dimer_Tnp_hAT--	XIX

Gar10G07380	Gar Zf-BED33_IV	MPPREEFPTKVLEGASVNNIGWHFGTPVPNAKGNIVCKLCGKVVKEGITRFKEHIAHKTGNVAPCPNVTGELYLNWLEAK<br>VQSNQRLHFQLASSPWLYNLIQVSTEVGQGVKLPTPYGVSDVYLESEYQRVRDWVNGLKAHWKEFGATITCDGLTNSLN<br>QMHITNFLVYCSKGTIFWKSVDVSNVRSRDAEFYYSLLDSVVEEIGEKYIVQIVTDNEVTMKAAEKN	zf-BED--DUF-domain	IV
Gar10G10440	Gar Zf-BED34_I	MNDPKRDGSTQSSQNSSPSRDDQTTTKENARKNITPRAACWSHFTKFVTKEGEKRARCNACDVTYTMESTSGSTTNLN<br>NHLKICLKKPRGNTSNPKQSELSFVKVSQEMTDLSTWVFDKDAIRKALVRMIIVDELPFKFVEGEGFKYFLSIACPRFSLSS<br>RWTIRRDCLDLFNFMKSVMKNCFEKDISRVCLTTDTWTSLQRISYMVLTAHWVDDEWRLQKRIINFCPISAHRGESIGQAI<br>EKCLRDWGIERVFTITVDNASANSVAIEYLRKKLNHRNASVANGKFIHMRCVAHILNLIVQYGIKDASVSVDRVRGAVRYIR<br>ASPSRLTKFNQQVKEEMIDSKAQLCLDVPTRWNSTYMMLKVAEKYERAFESYLRDDHNFFLDLTAGDGVPTFDDWDIVR<br>RVIKVLEPFYHLTLKVSGSFHVTSHSLFEVLTDVHCLFDRWQDCGDLEIISMTSKMREKYNKYWGEGNKINMLVYLAFIFD<br>PRCKMSFLDFGVNLLFPNVANDIMKMIDKELHCLFNEYSSNAGRIQLFEGRSIGLESKSELDRYLGEDEEVNNSSSFDLLL<br>WWKMNSPRFPILAQMARDILATPISTVASESAFSTGGRVLDSFRSSLTPLMVEALVCTQDWLRKSNDAINLEDYVDELQT<br>MEDGNT	zf-BED--DUF-domain--Dimer_Tnp_hAT--	I
Gar10G14600	Gar Zf-BED35_I	MPPREEFPPKGLEGAPSNDIGWHFGTPVPNTKGNIICKLCGKVVKGGITRFKEHISHKTGNVAPCPNVTGELYLNWPEAK<br>VQSNQSKGTIFWKSVDASSVRSRDAEFYYSLLDSVVEEIGENYIVQIVTDNEAAMKAAGKKLMLKRKHLYWTSCAAHCLD<br>LCLEDIGKKPSVAKVLDEAKKVTCFIYNHIWTVDLMKKYTQGKQILRPALTRFATHFIQLEEITRQKQGLREMFNSKEFKES<br>KWGQQKSGPAYEAKKIVLGKDFWKKANDLIKVYEPLVKVLRLVDSDEKPTMGFIYEAVDRAKREIQQDCRYFTEYEKIIDN<br>RWNFMHSDLHSVVIERLEHSLDTQVRMVNQLLLFRDKHETFGTPQAQRAWKQMNSAEWWIIYGTCVPELQKLAIKVLSQ<br>TTSASNCKRNWSTFSYIHTKARNRLKYKKLEKLVFTYYNMRLQIRHQKRMSTDDINASFNPISLDHIFEDVDPLSEWLHEK<br>GNPLLDGENAGVLPVDTSDDEMDVDQSQQQNLSHSSSSSMPSQSGDGPDGGGLSPIDEDDGYSGDRGEIRSSSQYGG<br>EYGVGTTSGHFRDRSEFDGNMFPEPRRDRSEPRAPSKGKGKKHTSIGSSSGRRSSSSNLGYSDSSTSTQGFYLPEQPS<br>YFQPSHGYPQPYGYYPLFPNYGVPYQPQMHPPPPMYHPPPPLMYPLPQIYPPYQLYENQGENVTFFGYIFGQRSREPS<br>QERSQSEGEGSDLPRHSTNW	zf-BED--DUF-domain--Dimer_Tnp_hAT--	I
Gar10G17150	Gar Zf-BED36_II	MSDPKRDGSTQSSQNSSPSRDDQTTTKENVRKKTTLRVACWNHFTKFMTKEGKKRARCNTCDVTYTMETTSGSTTNLN<br>NHLKACLKIPRGNTSNLKQLELTFVKVSQETMDLSTWVFDKDAVRKALVRMIIMNELPFKIVEGEGFKYFLSIACPRFSLPS<br>RWTIRRDCLDLFNSMKSVMKNYFEKDISRVCLMTDIWTSLQMISYMVLTAHWVDDEWRLQKRIINFFPISAHRGESIGQAI<br>GKCLRDWGIERVFTITVDNASANIIAIKYLRKKLNH	zf-BED	II
Gar10G24520	Gar Zf-BED37_IV	MPQREEVPTKGLEGAPSNDIGTPVPNAEGNIVCKLCGKVVKGGITRFKKHIAHKTGNVAPCPNVTEEEDEHEEFIDEVSAT<br>RQATRESIQSQHEWHRREEFRRSTGGWDNIYEEGGSSHGLAREYHRERTSKSIPISAKVGQSVKLPTPYEVSDVYLESE<br>YQRVHDWVNGLKTHWKELGATLMCDSWTNNLNQMHIINFLVYCSKGTNFWKSVDVSSVRSRDVEFYYSLLDSVVEEIGE<br>NYIVQIVTDNEAVMKAAGKSIHKGNKYFDPLLLDLQLISFNLKR	zf-BED--DUF-domain	IV
Gar11G00750	Gar Zf-BED38_I	MEWSVNNAFKSYKDMEPKSTMDMVLIPNMDTIDIVLGSSEKGNVVPSAKPRKKTMTSVYLKYFETAPDGKTRRCKFCGQ<br>SYSIATATGNLGRHLSNRHPGYDKTGENVSSSAPQPSTTPTVIKKPQPQGRAPQVDYDHLNWLLIKWLILATLPPSTLEEK<br>WLANSFKFLNPSIQLWPGEKYKAVFREVFRSMREDVRASLEQVSSKVSIALDFWSSYEQIFYMSITCQWIDENWSFQKVL<br>LDICQVPYPCTGSEIYNSLVKVLKMYNIENKVLSCTHDNSQNAIHACHALKEDLDGQKMGPFCFIPCAARTLSLIIDDALRTT<br>KPVIAKVREFVQELNASLDISEDFIQLATAYKEGSWQFPLDASARWSGSYQMLDIVQKAGKSMDAVVRKNEEMLGNRML<br>LNTAEKNVVNIVHNYLEPFYKVISEICVNTPPTIGMVIVYMDHISDTITTRQPPDWLKNPAEDMAKKLRSYNNQVCNIFIHMT<br>AILDPRIKCELIPESLNSENYLEEARAHFVRNYYTTPFSSMTSGYSSQDIEDGGAVSFAEEIARKKRRASMSNATDELTQYL<br>SESPAPTKTDVLEWWKVNSTRYPRLSAMARDFLAVQATSVKPDELFCSKGDEIDKQRFCMPHDSTQAILCIKSWTQGGL<br>KLKYKSTEIDYERLMEMAAAAAADISLAGMDKKQK	zf-BED--DUF-domain--Dimer_Tnp_hAT--	I
Gar11G24390	Gar Zf-BED39_IV	MPPCEEFLTNGLEGAPCNDIGLYFGTPVPNLKGNVVCKLCGKVVKGGITRFKRHIAHKIGNVAPCPNVTVKQLKKVSNHN<br>TNGIEGKNSDEVLVVGIKFMKKGDLLMDQLENIMEKEQMSQVFIVKMMNSTTVVRFSCRRNWRKLNCTNCVDNEAVMKA<br>VGKKLMLKRKQLYWTSCITNCIDLYLEDIGKKPSVAEVLDEPKKVTCFIYNHIWTVNLMKKYTQGKQILLPALTRFATHFIKL<br>EEITRQKQGLREMFNSKLLLVRDKYETCGTPQAQRA	zf-BED--DUF-domain	IV
Gar11G24510	Gar Zf-BED40_IV	MNDPKRDGSTQSSQNSSPSRDDQTKTKENARKKITPRAACWSHFTKFVTKEGEKRARCNACDVTYTMESTSGSTTNLN<br>NHLKICLKKPRGNTSNPKQSELSFVKVSQETTDLSTWVFDKNAIRKALVRMIIVDELPFKIVEGEGFKYFLSIACPRFSLTSR<br>WTIRRDCLDLFNSMKSMMKNYFEKDISRVCLTTDTWTSLQRISYMVLTAHWVDDEWRLQKRIINFCPISAHRGEKSIGQAI<br>EKCLQDWGIERAFNITVDNASANSVAIEYLRKKLNHRNASVANGKFIHMRCVAHILNLIVQYGIKDASVSVDRVRGAVRYIR<br>ASPSRLTKSNQRVKEEMIDSKAQLCLDVPTRWNSTYIMLKVAEKYERAFESYLRDDHNFFLDLTAGDGVPTFDDWDIVRR<br>VIKVLEPFCHLTLKVSGSLHVTSHSLFEVLTDVHCLFYGWQDCGDLEIISMTSKMREKYNKYWGEGNKINMLVYLAVIFDP<br>RCKMSFFDFVVNLLFPNVANDIMKMIDKELHCLFNEYSSNAERIQLFEGRSSSLTNICSSMEIDQSEMKTSFGQTKIP	zf-BED--DUF-domain	IV
Gar11G26400	Gar Zf-BED41_II	MPCSRNKEEVAPSDDYGWRWGELVEGNRNNVKCRFCGRIIKRGITQLKEYLAVKKGNVTPYPHGSVKVRKSIGQQLQE<br>YHREKAVRQRRKEELEERISLGNHGNYGDSGDDDEELTITRRKSVRSQVE	zf-BED	II
Gar11G27460	Gar Zf-BED42_II	MPPREEFPTKGLEGAPSNDIGWHFGTPVPNAKGNIVCKLCGKVVKGGITRFKEHIAHKTGNVAPCPNVTGVIRESMMNVL<br>KENNTKKIDKKRRKDDFLSQLREEEDEHEEFIDEISAIRQATRESIQSQHECHRREEFRRSTGGWDNIYEKGRSSQGSSIP<br>TESEFTLRGTIPELVRSKSSKQPKVSDSFLKSFRKKIGYFLNPQFQFGVEHSQNVLIETLEGTRSVIERLEPSLDTQVRMVN<br>QLLLFKDKHETFGTPQAQRAWKQMNPEWRWTDGGGLSPVDEDDGYNGDRGEIRSSSQYGGEYGVGTTSRHFRDRSE<br>FDGNMFPEPRRDRSEPRAPSKGKGKKHTSIGSSSGRRSGSSNLGYSDSSTSTQGFYPPEQPSYFQPSHGYPQPYGYY<br>PPFPNYGVPYQPQMHPPPPMYHPPPPLMYHPPQIYPPHQLYENQCENVTFFGYIFGQRPRESSQERSQNEGDGSDLPR<br>HSTNW	zf-BED	II
Gar11G28280	Gar Zf-BED43_IV	MPPREEFPTKGLEGAPSNDIGWHFGTPVPNAKGNIVCKLCGKVVKGGITRFKEHIAHKTGNVAPCPNVTEKVDKENNTKK<br>IDKKRRKDDFLSQLREEEDEHEEFIDEISAIRQATRESIQSQHECHRREEFRRSTGGWDNIYEKGRSSQESSIPTESEFTL<br>RGTIPELVRSKSSKQPKVSDSFLKSFRKKIGEAVSKFLIYERLPFQLASSPWLYNLIQLPTPYEVSDVYLESDYQRVHNWV<br>NGLKTHWKELGATLMCDGWTNSLNQMHIINFLVYCSKGTIFWKSVDVSSVRSRDAEFYYMTNNEAAMKAAGKKLMLKRE<br>HLYWTSCAAHCLDLCLEDIGKRPSVAKVLDEAKKVTCFIYNHTWTVDLMKKYTQGKQILRPALTRFVTHFIQLEEITRQKQ<br>GLREMFSSKEYKESKWGQQKSGPAYEAKKIILGKDFWKKANDLIKVYEPLVKVLRLVDSDEKPTMGFIYEAVNRAKRAIQ<br>QDCRYFTEYEKIIDKRWNFMHSDLHSAGYFLNPQFQFGVEHSHNVLIETLEGTRSVIERLEPSLDTQVRMVNQVRFNSNK<br>HETFGTPQAQRAWKQMNPDVDPLSEWLHEKENPLLDGENAGSGDGPDGGGLSPVDEDDGYNGDRGEIRSSSQYGGE<br>YGVGTTSRHFRDRSEFDGNMFPEPRRDRSELRAPSKGKGKKHTSIGSSSGRRSGSSNLRYSDSSTSTQGFYPPEQPSY<br>FQPSHGYPQPYGYYPPFPNYGVPYQPQMHPPPPMYHPLPPLMYPPPQIYPPHQLYENQCENVTFFGYIFGQRPRESSQ<br>ERSQSEGDGSDLPRHSTNW	zf-BED--DUF-domain	IV
Gar11G36310	Gar Zf-BED44_I	MPPREEFPTKGLEGAPSNDIGWHFGTPVPNTKGNIVCNLCGKVVKGGITRFKEHIAHKTGNVAPCPNVTGVIRESMMDIL<br>KESKTKKIDKKRRKDEFLSQLREDEDEHEEFIDEVSAIRQATRESIQSQHEWHRREEFSRSTGGWNNIYEEGRSSHGSAR<br>EYHRERTSKSIPSESEFTLRGAIPELARSKSSKQPKVSDSILKTFRKKIGEAVSKLLIYKRLSFQLASSPWLYNLIQVSTEVG<br>QGVKLPTPYEISDVYLESEYQRVRDWVNGLKTHWKELGTPVMCDGWTNSLNQMHIINFLVYCSKGTIFWKSVDASSVRS<br>RDAEFYYSLLDSVVEEIGENYIVQIVTDNEAAMKAAGKKLMLKRKHLYWTSCVAHCLDLCLEDIGKKPSVPKVLDEAKKVT<br>CFIYNHIWTVDLMKKYTQGKQILRPALTRFATHFIQLEEITRQKQGLREMFNSKEFKESKWGQQKSGPAYEAKKISPLVKV<br>LRLVDSDEKPTMGFIYEAVDRAKRAIQQDCRYFTEYEKIIDNRWNFMCSDLHSAGYFLNPQFQFGVEHSENVLKPSLDTQ<br>VRMVNQLLLFREKHETFGTPQAQRAWKQMNPAEWWIIYGTCVLELQKLAIKVLSQTTSASNLFTYYNMRLQIRHQKRMS<br>TDDINACFNPISLDYIFEDVDPLSEWLHEKENPLLDGENAGVLPVDTSDDEMDIDQSQQQNLSHSSSSSAPSQSGDGPDG<br>GGLSPIHEDDGYSGDRGEIRSSSQYGGEYGVGTTIGHFRDRSEFDGNMFPEPRRDRSEPKAPSKGKGKKHTSIGSSSG	zf-BED--DUF-domain--Dimer_Tnp_hAT--	I

		RRSSFSNLGYSDSSTSTQDFYPPEQPSYFQPSHGYPQPYGYYPPFPNYGCANLKKIHDRDTRSDRYVRNRDKRNATEN<br>TAATAI		
Gar11G38390	GarZf-BED45_I	MEVANESTAKKPKRLTSVVWNHFERVKKADICYAVCVHCNKKLSGSSNSGTTHLRNHLMRCLKRSNYDVSQLLAVKRRK<br>KENTLTIANISYDEGQRKEDYMKPTIVKYEQDQRKDEAFNLGSSWFDPERSRLDLARMIILHGYPLAMVEQVGFKVFVKN<br>MQPLFDVVHNSTIELSCVEIYMKEKQRIYDMLSKLQGRINLAIEMWSSPENSKYVCLTAHYVDDEWKLQKKILNFLTLDSS<br>HTEDVLSDVIIKCLMDWDIDCKLFAMTFDDCSTNDDIVSRIKDQVSESRPRLSNGQLLDVRSAAHVLNSIAQDAIEALQVVI<br>QKIRGSVKYVKSSQSILGKFNEIAQQQGIDNHKIVVLDYPIRWNSTYMMLEIAVEYRNVFHHLPELDPDFALSDEEWERAS<br>SIVSCLKLLIEIINVFSSNKCPTANIYFPEICHVHIQLIEWCKSSDAFLSSLATKMKAKFDKYWSKCSLALAVAAILDPRFKMKL<br>VEYYYSQIYGSTALERIKEASDGIKELFNAYSICSTLIDQGSALPGSSLPSSSNDTRDRLKGFDKFLHETSQSQTAISDLEKY<br>LDEPVFPRNCDFNILNWWRVHTPRYPILSMMARDVLGTPMSTIAQEFAFNAGGRMLDSNQSSLPPDTQQALICTRDWLR<br>TQSDDATPSSSHYALPLYVEAN	zf-BED--DUF-domain--Dimer_Tnp_hAT--	I
Gar12G06470	GarZf-BED46_IV	MDAISDRLVQNDGAIVSKQNLHSTIIPNIILEPKTLEPTPREEFPTKGLEGAPSNDIGWHFGTPMPNGKGNIVCKLCGKVVK<br>GGITRFKEHIAHKTGNVAPCPNVTGVIRESMMNVLKESNTKKIDKKRRKYDFLSQLREEEDEHEEFIDEISAIRQATRESIQL<br>QHEWHRREEFRRSTGGWDNIYEKRRSSQGSSIPIESEFTLRGAIPELVRSKTSKQPKKIGEAASKFLIYERLPFQLASSPW<br>LYNLIQVSTEVRQGVKLPTPYEVSDVYLESEYQRVPNWVNGLKTHWKELGATLMCDGWTNSLNQMHIINFLFYCSKGTIF<br>WKSIYVSNVRSRDAQFYYSLLDSVVEEIGENYIVQIVTDNEAAMKTAGTKLMLKRKHLYWTSCTAHCLDLYLEDIGKRPSV<br>TKVLNEAKKVTCFIYNHIWTVDLMKKYTQGKQILRPALTRFATHFIQLDKITRQKQGLREMFSSNEYKESKWGQQKSGPTY<br>EAKKIILGKDFWKKANDLIKVYEPLVKVLILVDSDEKPTIGFIYEAVDRAKRAIQQDCRYFTEYKKIIDKRWNFMHFDLHSAG<br>YFLNPQFQFGVEHSQNVLIETLEGIRSVIERLEPSLDTQVRMVNQLLLFRDKHETFGTLQAQRAWKQMNLDVDPLSKWLH<br>EKENSLLDGENAGMLPVDTSDDEMDVNQSQQPNLSHSSSSSTPSQSGDGPDGGGLSPVDEDDGYSSDRGEIRSSSQY<br>GREYGVGTTSGHFRDRSEFDGNMFPEPRRDRSEPRAPTKGKGKKHTSIGSSSGRRSSSSNLGYSDSSTSTQGFYPPEQ<br>PSYFQPSHGYPQPYGYYPPFPNYGVLYQPQMHPPPPMYHPPPPLMYPPPQIYPPHQLYENQCENFTFFGYIFGQRLRE<br>SSQERFQSKGEGSDLPRHSTNW	zf-BED--DUF-domain	IV
Gar12G08340	GarZf-BED47_IV	MPPCEEFPTKGLEGAPSNDIGWHFGTPVPNTKGNIVCKLCGKVMKGGITRFKKHIAHKTGNVAPCPNVTGVIRESMMNIL<br>KESKTKKIDKKRRKDEFLSQLREDEDKHEKFIDEVFAIRQATRESIQSQHEWHRREEFRRSTGERISKSILSESEFTLRGVI<br>PELARSKSSKQPKVSDSILKTFRKKIGEAVSKLLIYERLPFQLASSPWLYNLIQVSTEVGQGVKLPTPYEISDVYLESEYQR<br>VRDWVNGLKTHWKELGVTLMCDGWTNSLNQIHIINFLVYCNASSVRSRDVEFYYRLLDSVEAMKAAGKKLMLKRKHLYW<br>TLLNMTPIFTHCLDLCLEDIGKKPSVAKVLDEAKKVTCFIYNHIWTVDLMKKYTQGKQILRPALTRFATHFIQLEEITRQKQG<br>LREIQKIVLGKDFWKKANDLIKVYEPLVKVLRLVDSDEKPTMSFIYEAVDRAKRAIQQDCRYFIEYEKIIDNRWNFIHSDLHL<br>ADYFLNPQFQFGVEHSENVLIEILEGTRSIIERLEPSLNTQLRMVNQLLLFRDKHETFGTPQAQRAWKQMNPDVDPLSEW<br>LHEKENPLLDGENTGVLPVDTSDNEMDVDQSQQQNLSHSSSSSTPSQSGDGPDGNGLSPIDEDDGYSGDRGEIRSSSR<br>YGREYGVGTTSGHFCDRSEFDGNMFPEPRRDRSEPRAPSKG	zf-BED--DUF-domain	IV
Gar12G08690	GarZf-BED48_IV	MPPREEFPTKGLEGAPSNDIGWHFRTPVPNAKGNIVCKLCGKVVKEGITRFKEHIAHKTDNVAPCPNVTGIIRESMMNILK<br>ESNTKKIDKKRRKDDFLSQLREEEDEHEEFIDEISATRQATRESIQSQHEWHRREGFRRMSSYGSSIPTKSEFTLRGAIPE<br>LVRSKSSKQPKVSDSFLKSFRKKIGEAVSKFLIYERLPFQLASSPWLYNLIQVSTEFGQGVKLLTPNEVSDVYLESDLNQM<br>YIINFLVCCSKGTIFWKLVDVSNVRSRNAEFYYSLLNSVVEEIRENYIVYWKKKLMLKRKHLYWTSCVAHCLSLCLKDIGKR<br>PMTCFIYNHTWTVDLMKKYTQGKQILRPALTRFATHFIQLEKITRQKQGLREMFNSKEYKESKWGQQKSGPAHEPLVKVL<br>RLVNSEEKPTMAIQQAFRYFTEYKKIIDNRWNFMHSNLHPAGYFLNPQFQFGVEHSVNVLIETLEGTRSVIERLEPSLDTQ<br>VRMVNQLLLFRDKHERFDPPQPQRAWKQMNLDVDPLSGWLCEKENPLLDGENADVLLVDTSDDEMDVDQSQQKKLPH<br>SSSSSMPSQSGDGPDSGGLSPIDKDDGYSGDRGEIRSSSQYGGEYGVGTTSGHFCDRSEFDGNMFPEPRRDRSEPRA<br>PSKGKGKNHTFIGSSSSRRSSSSNLGYSDSSTSTQGFYPLEQPSYFQPSHGYLELYGYYPPFPNYGVPYQPQMHPPPP<br>MYHPPPPLMYPSPQIYAPYQLYENQGENVIFFGYIFGQRPRESSQEHSQSEGEGFDLPRHSTNW	zf-BED--DUF-domain	IV
Gar12G16420	GarZf-BED49_III	MVEEMAPLRSIGYVDPGWEHGTAQDERKKKVKCNYCGKVVSGGIFRLKQHLARLSGEVTHCEKVPEEVCLNMRKNLEG<br>CRSGRKRRQFDYEQAALSIQSNEYSDGEDASASYKHKGKKVMGDKNLVIKFTPLRSLGYVDPGWEHCVAQDEKKRRVK<br>CNYCEKIISGGINRFKQHLARIPGEVAYCEKAPEEVYLKIKENMKWHRTGRRHRKPDTKEISTFYMHSDNEDEGGEEEGY<br>LQCVSKDILAIDDKVSDNDIRNNVRGRSPGSSGNGAEPLLKKSRLDSVFLKSLKSQTSAHYKQPRARTGFEKKTHREVISA<br>ICKFFYHAGIPSNAANSPYFHKMLELVGQYGQGLQGPSSRLISGRLLQEEIANIKEYLVELKTSWAITGCSVMADSWNDAQ<br>GRMLINFLVSCPRGVYFLSSVDATDIIEDAVHLFKLLDKAVDEVGEEYVVQVITRNTLSFRNAGKMLEEKRRNLFWTPCAV<br>YCIDRMLEDFVNIKWVGECVDKAKKVTRFIYNNTWLLNFMKKEFTKGQELLQPAVTKFGTNFFTLQSLLDQRVGLKRMFQ<br>SNRWLSSRFSKSDEGKEVEKIVLNVSFWKKMQYVKKSFEPVAEVLQRIGSDKIRSMPFIYNDICRTKLAIKAIHGDDVRKY<br>GPFWSVIESNWSSLFHHPLYVAAYFLNPSYRYRPDFLMNPEVFRGLNECIVRLEADNGKKIAASMQIPDFVSAKADFGTD<br>LAISTRSELDPASWWQQHGISCLELQRIAIRILSQTCSSIGCEHNWSAFDQVHIKRHNCLSRKRLNDQTYVHYNLRLRERQ<br>LGRKPDELVSFDSAMLESVLDDWLVETEKLAMHEDEEIIYTEVEQFCGDDMDEHESEEKRPAEMVTIAGFIEPLDVIPSAG<br>GVTTDDDGLDFLDDDLTD	zf-BED--zf-BED--DUF-domain--Dimer_Tnp_hAT--	III
Gar13G06770	GarZf-BED50_II	MSTEPTSIKGSVTPPTSIDSENSGVGASSQANVTTGKRKATPQRSEVWSHFTKIINSEGASKAKCNYCQKEFCCDVKKNG<br>TGSLKYHIGSCKKNPSNVVDPSQGQLVLPRKGVEGGEGHFSTWRFDQKACRKGLAQMIVIDELPFKFVESEGFKKFMFV<br>ACPRFHIPSRTTMTRDVYQLYLD	zf-BED	II
Gar13G16090	GarZf-BED51_I	MPPREEFPTKGLEGAPSNDIGWHFGTPVPNAKGNIVCKLCGKVVKGGITRFKEHIAHKTGNVAPCPNVTGVIRESMMNVL<br>KENNTKKIDKKRRKDDFLSQLREEEDEHEEFIDEISAIRQATRESIQSQHECHRREEFRRSTGGWDNIYEKGRSSQGSSIP<br>TESEFTLRGTIPELVRSKSSKQLKVSDSFLKSFRKKIGEAVSKFLIYERLPFQLASSPWLYNLIQVSTEVGQGVKLPTPYEVL<br>DVYLESEYQRVHNWVNELKTHWKELGATLMCDGWTNSLNQMHIINFLVYCSKGTIFWKSVDVSSVRSRDAEFYYSLLDS<br>VVEEIGENYIVQIVTDNEAAMKAAGKKLMLKREHLYWTSCAAHCLDLCLEDIGKRPSVAKVLDEAKKVTCFIYNHTWTVDL<br>MKKHTQGKQILRPALTRFATHFIQLEEITRQNQGLREMFSSKEYKESKWGQQKSGPAYEAKKIILGKDFWKKSNDLIKVYE<br>PLVKVLRLVDNDEKPTMGFIYEAVDRAKRAIQQDCRYFTEYEKIIDKRCNFMHSDLHSAGYFLNPQFQFGVEHSHNVLIET<br>LEGTRSVIERLEPSLDTQVRMVNQLLLFRDKHETFGTPQAQRAWKQMNPAEWWIIYGTCVPELQKLAIKVLSQTTSASNC<br>ERNWSTFSYIHTKARNRWKYKKLEKLVFTYYNMRLQIRHQKRMSTDDINTSFNPISLDYIFEDVDPLSEWLHEKENPLLDG<br>ENTGVLPVDTSDDEMDVNQSQQQNLSHSSSSSTPSQSGDGPDGGGLSPVDEDDGYNGDRGEIRSSSKYGGEYGVGTT<br>SRHFRDRSEFDGNMFPEPRRDRSEPRAPSKGKGKKHTSIGSSSGRRSGSSNLGYSDSSTSTQGFYPPEQPSYFQPSH<br>GYPQPYGYYPPFPNYGVPYQLQMHPPPPMYHPPPPLMYPPPQIYPLHQLYENQCENVTFFGYIFGQRPRESSQERSQS<br>EGDGSDLPRHSTNW	zf-BED--DUF-domain--Dimer_Tnp_hAT--	I
Gar13G21920	GarZf-BED52_II	MSTEPTSIKGSVQPPTSIDSENSGVGASSQANVTTGKRKATPQRSEVWSHFTKIINSEGASKAKCNYCQKEFCCDVKKN<br>GTGSLKYHIGSCKKNPSNVVDPSQGQLVLPRKGVEGGEGHLSTWRFDQEACRKGLAQMIVIDELPFKFVESEGFKKFMF<br>VACPRFHIPSRTTMTRDVYQLYLDERVKIKQLLRSSCSRVCLTTDTWTSL	zf-BED	II
Gbar_A02G000610.1	GbaZf-BED01_I	MDNFDQKLGPEFFKNLSAEAVTPLNVVHEEIYESSSKRPKTTSKVWDIFEKLPAQQGDSKAICKLCRRIYTAKTTSGTSHL<br>RRHIEACVKRGNHEVDQRSIEACFKPVKRNANRLTLSHDTLISATTSLKNYKLDVDEIHRAIAMMIIVDEQPFSVVEDAGFR<br>RLLSAACPEFPVLSRSSIKRDIISIYVKERENIRELLATCPGRICLTSSTWKSDSDDHFNCVTTHFSDHEWRLQKRILRFKLM<br>PPPYDSLSVADEIALCMVQWNIEHKVFSVTLENLSSDDCVADMLRSRLAAKKYLPCKGVFFHVSCFFRILNSIVQAGLNLV<br>VDIIAKLRLGIKYVQQSPHRKKNFYIVAKTLNLDTQRKLCLDTPARWNSTYDMIEVAFCYKNAFVYLAEQDKNFLHKLSEDE<br>WEKMSVLYKFLKVFYEVTCVFFRNRQPTSNLYFKAAWKVHSRLFDMVRGPENFMTRMVREMHSKLNHYWSAYNLILSC<br>AAILDPRYKIKFVEYCYTKLYGSGAQKYVSVSVNTLYGLFDEYMQNSARPSQTTLLSTAASKISNDKDENDGFEDYETFQS<br>ARFRTQVEKSQLDLYLEEPSHDLNSEIDVLEYWTLCSLRYPELSKMARDVLTIPVSTIASDSAFDITPQVISADRSSLKPKM<br>LQALVSLQDWMLASDRTRGLGSMESKPEDDSSSSSDGDDDY	zf-BED--DUF-domain--Dimer_Tnp_hAT--	I
Gbar_A02G000610.2	GbaZf-BED02_I	MDNFDQKLGPEFFKNLSAEAVTPLNVVHEEIYESSSKRPKTTSKVWDIFEKLPAQQGDSKAICKLCRRIYTAKTTSGTSHL<br>RRHIEACVKRGNHEVDQRSIEACFKPVKRNANRLTLSHDTLISATTSLKNYKLDVDEIHRAIAMMIIVDEQPFSVVEDAGFR<br>RLLSAACPEFPVLSRSSIKRDIISIYVKERENIRELLATCPGRICLTSSTWKSDSDDHFNCVTTHFSDHEWRLQKRILRFKLM<br>PPPYDSLSVADEIALCMVQWNIEHKVFSVTLENLSSDDCVADMLRSRLAAKKYLPCKGVFFHVSCFFRILNSIVQAGLNLV<br>VDIIAKLRLGIKYVQQSPHRKKNFYIVAKTLNLDTQRKLCLDTPARWNSTYDMIEVAFCYKNAFVYLAEQDKNFLHKLSEDE<br>WEKMSVLYKFLKVFYEVTCVFFRNRQPTSNLYFKAAWKVHSRLFDMVRGPENFMTRMVREMHSKLNHYWSAYNLILSC	zf-BED--DUF-domain--Dimer_Tnp_hAT--	I

		AAILDPRYKIKFVEYCYTKLYGSGAQKYVSVSVNTLYGLFDEYMQNSARPSQTTLLSTAASKISNDKDENDGFEDYETFQS<br>ARFRTQVEKSQLDLYLEEPSHDLNSEIDVLEYWTLCSLRYPELSKMARDVLTIPVSTIASDSAFDITPQVISADRSSLKPKM<br>LQALVSLQDWMLASDRTRGLGSMESKPEDDSSSSSDGDDDY		
Gbar_A02G001010.1	GbaZf-BED03_VIII	MASSSSFSSADAALEILVSCAHAIEDGNLKTADSFLHQIWNTAAVELDLISKLVRYFAEALVRRAYGLHPPYYTHSNLQIPH<br>PLYYYYYYSRFDINEMVGEAIESATTGKKGFHLIDFHIPHLYGRGYLFKTLPNRSSDPLSVRITVVLPTFLKNTVDFQEEME<br>YLTEAGKLLKIELKKEDLRVVYANSLGEVDESTLDLRRTNDDEALVVYYNFKFHTLLAEAEAMKKELIKLRQINPEIVIMQEQ<br>YANDNDGNFIKRLEYSFRYYSNFFQYYSNLFKSGKPLGDNTAKYYMRQIHNIVACEGRDRIMRHQSLDEWRDLLLTAGFL<br>QIPFQKDVENLHALYWVEEIKEEKGCLVLSHKDCPILFVSCWRPRAGEEHFKFNLNSNKFGQGFNPRPFQPFPEGFILNR<br>LATFAEIYDMLEDVCFRYELPVALTWACEATTDKIMLDGKKHTLFMERTSCYASNEGSQCFMEACAKHHIQEGQAIAGKA<br>FQSSANFHFEPSITKLMKSDYPLFNAAQLFGSHAVVAICLQNHYIIGDVYVVEFYWPEIESEKSESLALDIFNDLKNMKKKF<br>VTIRVGGNEVGFEREAISTTLQGTMHMRNAQPASSTNDLLSSNTTWSLNAVQPCDVHEMERHGLVEQVESAPFSTPNP<br>MSHGGVLQTQGPHKQEIGEKDFISQTVSIGDYEIVKASMETCKVPRTKRRKYSSKVWLDFDKFEVNGKQVAKCKHCNKD<br>FTGSSKSGTTHLKNHLERCQSKKIKNQERQLITSEIGDLITRDSDESNFTFDQERSRLDFAKMIIKHQSPLDMAEQEFFKIF<br>VKNLQPMFEFQSKDILLSDIHRIYKEETEKLQLYFDHLACNFNLTISLCKNNHGKTAYCCLIAHFIDDNWEPRMKIIACKPLE<br>HIYDTKALNEIIQSSVLEWNISKKVFSITMDNPYLNDDMFQKIKETCFSDQGSFPSTHWFIGCTFIEDGFREMDLILLKLRKSI<br>EYVSEIAEGKLKFEEVVNQVKLQGGKSWDDLSLRLDSDFGVLHSALESREIFCQLEKIDGNFKLNPSVEEWEMVLAFHSC<br>LKCFDDIEGTQSLTANLYFPKLCNICKKFLHLEKSNYPIVTLMKRKFDYYWSLCNSAFAVATILDPRLKFKFVEFSYTEIYGH<br>DSKMHLNRFHKVLTDVYYEYANEARNLSKSTSDLDDSNYSTTEIVNDCILESFSKFASANNFNEVASWKSELDCYLDEPLL<br>PLDGAFDLLYWWCINNKRFPTLAKMARDFLAMPIPILAPCLNFNAMITNPTYNNLNTESMEALVCSQNWLKIPKENDGENH<br>GPMQNMVGITYEL	GRAS--zf-BED--DUF-domain--Dimer_Tnp_hAT--	VIII
Gbar_A02G014400.1	GbaZf-BED04_I	MSTKPTSIEGSVTPPTSIDSENSGVGASIQTKGTTGKRKAPPQRSEVWSHFTKFINSEGASKAKCNYCEKEFCCDMKKNG<br>TGSLKYHIGSCKKNPSNVVDTSQGQLVLPRKGVEGGEGNISTWRFDQEACRKGLAQMILIDELPFKFVESEGFKKFMFVA<br>CPRFHIPSRTTMTRDVYQLYLDERVKIKQLLRSSCSRVCLTTDTWTSLQRVNYLCLTAHFIDNDWKLNKKILNFCPISSHKG<br>ESIGMVIEKCLLNWGIDKLFTVTVDNASSNDVAIGYLRKKFNPRGGLVQNGKYLHMRCMAHIVNLIVVEGLKEMNKSVERV<br>RGAVRYVRQSPARLQKFKECVVVEKIECKKMLCLDVCTRWNSTYLMLDTAQNFERAFERFEEQDTNFRAELERERVGLV<br>DFLEHFYEVTLRISGTSYVTSNNFFDELSEIDILLRDAQLNSNIDFNVMAIKMKEKYDKYWGDIDKMNLLMFVACVLDPRQK<br>LKYLEFALSEMSSSEKACEMMQKLKESLYELFDEYKPPLHSTCSQSSVSTHVSIGEPQQKMKRRMQALYKKRELEICGED<br>KTSELDKYLAEANEEFVEDFDILLWWKVNSPRFPTLSKIARDVLAIPVSTVASESAFSTGGRVLDQYRSSLTPKIVQALVCT<br>QDWIRRSSSQEDIKKIEEQIQELDKIENELLASLSATMRIWCCDPTAKYHTSMSLLP	zf-BED--DUF-domain--Dimer_Tnp_hAT--	I
Gbar_A03G001880.1	GbaZf-BED05_I	MVRGRDACWEHCVLVDATRQKVRCNYCHREFSGGVYRMKFHLAQIKNKDIVPCAEVPDDVRDHIQSILNTPKKQKTPKK<br>PKMDKTVANGQQNSSSASGGLHPNHGSSGQHGSTCPSFLFPHPSPSEQPATDDAQKQKLDDADKKIAVFFFHNSIPFSA<br>AKSMYYQEMVDAIAECGVGYKAPSYEKLRSSLLEKVKGDIHDGYKKYREEWKETGCTVLCNSWSDGRTKSFVIFSVTYP<br>KGTLFLKSVDVSGHEDDASYLFELLESVVLEVGLENVIQVITDSTASYVCAGRHLMAKYSSLFWSPCASYCIDKMLEDISK<br>QEWVGIVLEEAKTIARYIYSHAWILNMIRKFTGGRELMRPRITRFVDNYLNLRSIVFQEDNLKHMFSHSEWLSSIYSRRSDA<br>QAIKSLLYLERFWKSAREAVSVSESLVKILRIVDGDMPAMGYIYEGIERAKGAIKAYYKGIEEKYMPIWDIIDRRWNMQLHS<br>PLHAAAAFLNPSIFYNPNFKIDLRMRNGFQEAMLKMATMDKDKIEITKEHPVYINAQGALGTDFAIMGRTLNAPGDWWAS<br>YSYEIPTLQRVAIRILSQPCSFHWCRWNWSTFETVHTKKRNKVEMEKLNDLVFVHCNLWLQTICQGRDGKCKPIIFDEIDV<br>SSEWPTESEPSVPLLDDSWLDNLPLECRGSP	zf-BED--DUF-domain--Dimer_Tnp_hAT--	I
Gbar_A03G001880.2	GbaZf-BED06_I	MVRGRDACWEHCVLVDATRQKVRCNYCHREFSGGVYRMKFHLAQIKNKDIVPCAEVPDDVRDHIQSILNTPKKQKTPKK<br>PKMDKTVANGQQNSSSASGGLHPNHGSSGQHGSTCPSFLFPHPSPSEQPATDDAQKQKLDDADKKIAVFFFHNSIPFSA<br>AKSMYYQEMVDAIAECGVGYKAPSYEKLRSSLLEKVKGDIHDGYKKYREEWKETGCTVLCNSWSDGRTKSFVIFSVTYP<br>KGTLFLKSVDVSGHEDDASYLFELLESVVLEVGLENVIQVITDSTASYVCAGRHLMAKYSSLFWSPCASYCIDKMLEDISK<br>QEWVGIVLEEAKTIARYIYSHAWILNMIRKFTGGRELMRPRITRFVDNYLNLRSIVFQEDNLKHMFSHSEWLSSIYSRRSDA<br>QAIKSLLYLERFWKSAREAVSVSESLVKILRIVDGDMPAMGYIYEGIERAKGAIKAYYKGIEEKYMPIWDIIDRRWNMQLHS<br>PLHAAAAFLNPSIFYNPNFKIDLRMRNGFQEAMLKMATMDKDKIEITKEHPVYINAQGALGTDFAIMGRTLNAPGDWWAS<br>YSYEIPTLQRVAIRILSQPCSFHWCRWNWSTFETVHTKKRNKVEMEKLNDLVFVHCNLWLQTICQGRDGKCKPIIFDEIDV<br>SSEWPTESEPSVPLLDDSWLDNLPLECRGSP	zf-BED--DUF-domain--Dimer_Tnp_hAT--	I
Gbar_A05G038120.1	GbaZf-BED07_I	MDMSDAVIVNSSRLKSIVWNDFDRVKKGDTFVAICRHCKKKLSGSSTSGTSHLRNHLIRCQRRSNHGVAQYFSAKDKKK<br>EGSLALVTIDQEQKNDEVLSIVNLRYEQEQIKSEHVGIGSNSLDQRQSQFDLARMIILHNYPLAMVEHVGFKIFVRNLQPLF<br>ELATRNKVEADCMEIYAKEKQKVYEIFDKLPGKISVSADVWTASEDDAAYLSLAAHYIDENWQLKKKNLNFVTIDPSYTED<br>MHSEVIMNCLMDWDIDRKLFSMIFDSFTSDNIVERIRDRLSQNRFLYCNGQLFDVRCAVDLLNRMAHDALEALCEITQKIR<br>ESIRYVKSSEATQATFNELADEVQVETKKCLCIDNPLKWNSTYFMLEAALEYRKVFSCLRDRDPVNMKFLLSDPEWDRLIT<br>VTSFLKLFVEVTNVFTRSKYPTANIFFPEICDIHLQLIEWCKNPDEYISSLALKMRKKFEEYWYKCSSGLAVAAMLDPRFKM<br>KLLEYYYPQLYGDSATELIDDVFECIKSLYNEHSIVSPLASSIDQGLDWQASGISGSGKDSRDRLMGFDKYLHETCQAEGS<br>SSDLDKYLEEPLFPRNVDFNVLNWWKVHTPRYPILSMMARNILGIPISKVAAESRFDTGGRVLDHNWSSLPPTTIQALMCS<br>QDWIRSELES	zf-BED--DUF-domain--Dimer_Tnp_hAT--	I
Gbar_A06G005450.1	GbaZf-BED11_I	MTEMTEIADMETIPGESNNQLALTTPEAQPIKRRKKKSMVWEYFTIENVSAGCRRAYCKRCKQSFAYSTGSKVAGTSHLK<br>RHIAKGTCRALLRGQGQDNNQFITPYNPKMGGSEPPKRRYRSPSSPFIPFDQDRCRHEIARMIIMHEYPLHIVEHPGFIAF<br>VQSLQPQFDKMSFNTVQGDCVATYLREKQSLMKFIEGIPGRFCLTLDMWSSNQTLGYVFITGHFVDSDWKLHRRVFNVV<br>MEPYPDSHSALSHAIAACISDWSLEGKLFSLTFNHPLSEAGLENLRPLLCVKNPLILNGQLLIRNCIARTMSSMAKDVLGAG<br>QEIIKKIRDSVKYVKMSESHDDKFIQVKNQLQVPSEKSLFLDNQTRWNTTYQMLAAASELKEVFDCLDTYDPDYKLAPSM<br>EDWKLAETLCSFLKPLFDAASILTTTTLPTVITFFYEVWKIHVDLGRSITSEDPFISKLAKSMQEKIDKYWKGCSLVLAMAVV<br>MDPRFKMKLVEFSFTKIYSEDAPTYIKTVDDGIHELFLEYVALPLPLTPTYAEEVNGANNGKTNESHYGNLLSDHGLTDFD<br>VYIMETNSQQMKSELDQYLEESLLPRVQEFDVLGWWKLNKMKYPTLSKMARDILSIPVSAAAPESIFDITDKQLDEYRSSL<br>RPETVEALICAKDWLHFGSSDVSNALVKMEF	zf-BED--DUF-domain--Dimer_Tnp_hAT--	I
Gbar_A06G005460.1	GbaZf-BED12_I	MSYLKLLFSYIYIYTHIHTYISWTTFLKFLFLSAAGFTSMTEMTEIADMETIPGESNNQLALTTPEAHPIKRRKKKSMVWEYF<br>TIENVSAGCRRAYCKRCKQSFAYSTGSKVAGTSHLKRHIAKGTCRALLRGQGQDNNQFITPYNPKMGGSEPPKRRYRSP<br>SSPFIPFDQDRCRHEIARMIIMHEYPLHIVEHPGFIAFVQSLQPQFDKMSFNTVQGDCVATYLREKQSLMKFIEGIPGRFCL<br>TLDMWSSNQTLGYVFITGHFVDSDWKLHRQVFNVVMEPYPDSHSALSHAIAACLSDWSLEGKLFSLTFNHPLSEAGLEN<br>LRPLLCVKNPLILNGQLLIRNCIARTMSSMAKDVLGAGQEIIKKIRDSVKYVKMSESHDDKFIQVKNQLQVPSEKSLFLDNQ<br>TRWNTTYQMLAAASELKEVFDCLDTYDPDYKLAPSMEDWKLAETLCSFLKPLFDAASILTTTTLPTVITFFYEVWKIHVDLG<br>RSITSEDPFISNLAKSMQEKIDKYWKDCSLVLAMAVVMDPRFKMKLVEFSFTKIYSEDAPTYIKTVDDGIHELFLEYVALPLP<br>LTPTYAEEVNGANNGKTNESHYGNLLSDHGLTDFDVYIMETNSQQMKSELDQYLEESLLPRVQEFDVVGWWKLNKMKY<br>PTLSKMARDILSIPVSAAAPESIFDITDKQLDEYRSSLRPETVEALICAKDWLHFGSSDVSNALVKMEF	zf-BED--DUF-domain--Dimer_Tnp_hAT--	I
Gbar_A07G007110.1	GbaZf-BED13_I	MSTEPTSIEGSITPPTSIDSENSRIRASSQAKGTTGKRKVTPQRSEVWSHFTKIINSEGASKAKCNYCQKEFCCDMKKNGT<br>RSLKYHIGSCKKNPSNVIDTRGRHLSTWRFDQEACRKGLTQMIVIDELPFKFVESEGFKKFMFVACPRFHIPSRTTMTRDV<br>YQLYLNERVKIKQLLKSSCSRVCLTTDTWTSLQSVNYLCITAHFIDNDWKLNKKILNFCPISSHKGESIGMVIEKCLLNWGID<br>KLFTVTVDNASSNNVAIGYLRKKFNPRGGLVQNGRYLPMRCMAHIVNLIVGAVRYVRQSPARLQKFKECVVVEKIECKKM<br>LFLDVCTRWNSTYLMLDTAQNFERAFKRFEEQDTNFRAELKRGEGWPSVDDWDNVRNLRDFLEHFYEVTLRISGTSYVT<br>SNNFFDELSEIDILLRDVQLNSNVDFNVMTIKMKEKYDKYWGDIDKMNLLMFVACVLDPRKKLKYLKFALSEMSSSEKACE<br>MMQKLKESLYELFDEYKPPLHSTCSQLSVPTHRELEICGEDKTSELDKYLAEANEEFVENFDILLWWKVNSPRFPTLSKM<br>AKDVLAIPVSTVASESAFSTGRRVLDQYRSSLTPKIVQALVCTQDWIRKLLSQEDIKKIEEQIQELDKIENGIFIDMLTFLFLLT<br>YNLFVCLYYLIFFKCVYFYYMLNFFL	zf-BED--DUF-domain--Dimer_Tnp_hAT--	I
Gbar_A07G008770.1	GbaZf-BED14_I	MELNLVPISITRQKQDPAWNHCEVFKNGERIQIKCMYCGKLFKGGGIHRFKEHLAGRKGQGPICEQVPQGVRSIMQESLN<br>GILVKQDKKQKLIPKLLACGSSSSNLNIGGEVENLGSHDDMNFGIKPISVLNTLEGDSNVVSKVGRGRKRGRDRDRNLIES<br>NRPCLKTDLALVPNGGENPIHMAIGRFLYDIGVNLDAVNSVCFQPMIDAIASGGSGVVPPSCHDLRGWILKNVIEEVKDDID<br>RNKAMWGKTGCSIIVEQCRTKNGRVLLSFLVYCPQATVFMKSVDASHAIYSADYLFELLKQVIEEVGSENVVQVITNCEEP<br>YLFTGKRLMESFPSLYWAPCLAHCVDLMLQDFSNLEWINETIEQAKSLTRFIYNQSSVLNTMRKFTSGNDVVEPALTCFAT<br>NFSTLRRMADLKLNLQAMVNSQDWLECPYAKEPGGQAMSDIVNNRSFWNSCVLIAHITYPLLRVLEIVGSKKRSAMGYVY<br>AGIYRAKETIKKELVKQDDYMVYWNIIDNRWEQQRHLPLYAAGFFLNPKFFYNTKEHIHNDILSAVFDSIERLVPDTNIQDQ<br>VVREINLYKNAMGDLGRPMAVRARDNLLPGEWWSIYGGGCPNLQRLAIRILSQTCSSIGYKPNKISIEEIHNTRNFLERRRL<br>SDLVFVQYNLYLRQMVLQNQEKDSLDPLVFNRKDILEDWIADNEVSPDNHESSDWKSLDPPVGNRTTLTPPGDEAEDFL<br>STRFMDLDIFNGLKGVKEEI	zf-BED--DUF-domain--Dimer_Tnp_hAT--	I

Gbar_A07G012480.1	GbaZf-BED15_I	MSTEPTSIKGSVTPPTSIDSENLGVGASSQANVTTGKRKATPQRSEVWSHFTKIINSEGASKAKCNYCQKEFCCDVKKNGTGSLKYHIGSCKKNPSNVVDPSQGQLVLPRKGVERGEGHISTWRFDQEACRKGLAQMIVIDELPFKFVESEGFKKFMFVACPRFHIPSQTTMTRDVYQLYLDERVKIKQLLRSSCSRVCLTTDTWTSLQRVNYLCITAHFIDNDWKLNKKILNFCPISSHKGESIGMVIEKCLLNWGIDKLFTITVDNASSNDVAIGYLRKKFNPRRGLVQNGKYLHMRCMAHIVSLIVVEGLKEMNKSVERVRGAVRYVRQSPARLQKFKECVVMEKIECKKMLCLDVCTRWNLTYLMLDTAQKFERAFEKFEEQDTNFRVELERGEGWPSVDDWANVRNLRDFLEHFYEVTLRISGTSYVTSNNFFDELSEIDILLRDAQLNSNVDFNVMAIKMKEKYDKYWGDIDKMNLLMFVACVLDPRQKLKYLEFALSEMSSSEKACEMMQKLKESLYELFDEYKPPLYSTCSQSSVPTHVSLGEPQQKMKRRMQALYKKRELEIGGEDKTSELDKYLAEANEDFIEDFDILLWWKMNNPRFPILSKMARDVLAIPVSTVASESTFSTGGRVLDQYRSSLTPKIVQALVCTQD	zf-BED--DUF-domain--Dimer_Tnp_hAT--	I
Gbar_A07G013730.1	GbaZf-BED16_II	MSTEPTSIRGSVTPPTSIDSENPGVGASSQANVTTRKRKATPQRSEVWSHFTKIINSEGASKAKCNYCQNEFCCDVKRNGTGSLKYHIGACKKNPSNVVDTSQGQLVLPRKGVEGGEGHLSTWRFDQEACRKLLAQMIVIDELPFKFVESEGFKKFMFVACPRFHIPSRTTMTKDVYKLYLDERVKIK	zf-BED	II
Gbar_A08G009840.1	GbaZf-BED17_I	MAEMTEATNMETSPVENNNELALITPETQPKRRKKKSMVWEYFTIETVSAGCRRACCNRCKQSFAYSTGSKVAGTSHLKRHIAKGTCPALLRDQYNNQLTPYSPKTGGGEPRKRRYRSPSSPFIPFDQDRCRHEIARMIIMHEYPLHMVEHPGFIAFVQNLQPRFDKVSFNTVQGDCVATYLREKQSLMKLIEGIPGRVCLTLDMWTSNQTLGYVFITGHFIDFEWKLQRRVLNVIMEPYPDSDSALSHAVAACLSDWSLEGKLFSLIFNHPTSEAGLENLRPLLCTKNPLILNGQLLLGNCIARNLSSMAKDVLGAGHEIVKKIRDSVKYVKTSESHDEKFVQVKNQLQVPSEKSLILDNQNQWNTTYQMLAAASELKEVFNCLDTSDPDYKLAPSMEDWKVAETLCTFLKPLFDAASILMTTTNPTAITFFHEAWKIHADLGRSIANDDPFISNIAKSMLEKIDKYWKDCSLILAIAVVMDPRFKMKLVEFSFTKIFGEDAPTYIKIVDDGIHELFLEYVALPLPLTPTYTEGNVGNNGKTDESQQGNLLSDQGLTDFDVYIMETSSQQMKSELDQYLEESLLPRVQEFDVLGWWKLNKMKYPTLSKMARDILSIPVSAAAPDSVFDIIIKQLDEYRSSLRPETVEALICAKDWLHHGSEESNALVKMEF	zf-BED--DUF-domain--Dimer_Tnp_hAT--	I
Gbar_A08G009840.2	GbaZf-BED18_I	MAEMTEATNMETSPVENNNELALITPETQPKRRKKKSMVWEYFTIETVSAGCRRACCNRCKQSFAYSTGSKVAGTSHLKRHIAKGTCPALLRDQYNNQLTPYSPKTGGGEPRKRRYRSPSSPFIPFDQDRCRHEIARMIIMHEYPLHMVEHPGFIAFVQNLQPRFDKVSFNTVQGDCVATYLREKQSLMKLIEGIPGRVCLTLDMWTSNQTLGYVFITGHFIDFEWKLQRRVLNVIMEPYPDSDSALSHAVAACLSDWSLEGKLFSLIFNHPTSEAGLENLRPLLCTKNPLILNGQLLLGNCIARNLSSMAKDVLGAGHEIVKKIRDSVKYVKTSESHDEKFVQVKNQLQVPSEKSLILDNQNQWNTTYQMLAAASELKEVFNCLDTSDPDYKLAPSMEDWKVAETLCTFLKPLFDAASILMTTTNPTAITFFHEAWKIHADLGRSIANDDPFISNIAKSMLEKIDKYWKDCSLILAIAVVMDPRFKMKLVEFSFTKIFGEDAPTYIKIVDDGIHELFLEYVALPLPLTPTYTEGNVGNNGKTDESQQGNLLSDQGLTDFDVYIMETSSQQMKSELDQYLEESLLPRVQEFDVLGWWKLNKMKYPTLSKMARDILSIPVSAAAPDSVFDIIIKQLDEYRSSLRPETVEALICAKDWLHHGSEESNALVKMEF	zf-BED--DUF-domain--Dimer_Tnp_hAT--	I
Gbar_A08G009840.4	GbaZf-BED20_I	MAEMTEATNMETSPVENNNELALITPETQPKRRKKKSMVWEYFTIETVSAGCRRACCNRCKQSFAYSTGSKVAGTSHLKRHIAKGTCPALLRDQYNNQLTPYSPKTGGGEPRKRRYRSPSSPFIPFDQDRCRHEIARMIIMHEYPLHMVEHPGFIAFVQNLQPRFDKVSFNTVQGDCVATYLREKQSLMKLIEGIPGRVCLTLDMWTSNQTLGYVFITGHFIDFEWKLQRRVLNVIMEPYPDSDSALSHAVAACLSDWSLEGKLFSLIFNHPTSEAGLENLRPLLCTKNPLILNGQLLLGNCIARNLSSMAKDVLGAGHEIVKKIRDSVKYVKTSESHDEKFVQVKNQLQVPSEKSLILDNQNQWNTTYQMLAAASELKEVFNCLDTSDPDYKLAPSMEDWKVAETLCTFLKPLFDAASILMTTTNPTAITFFHEAWKIHADLGRSIANDDPFISNIAKSMLEKIDKYWKDCSLILAIAVVMDPRFKMKLVEFSFTKIFGEDAPTYIKIVDDGIHELFLEYVALPLPLTPTYTEGNVGNNGKTDESQQGNLLSDQGLTDFDVYIMETSSQQMKSELDQYLEESLLPRVQEFDVLGWWKLNKMKYPTLSKMARDILSIPVSAAAPDSVFDIIIKQLDEYRSSLRPETVEALICAKDWLHHGSEESNALVKMEF	zf-BED--DUF-domain--Dimer_Tnp_hAT--	I
Gbar_A08G009840.5	GbaZf-BED21_I	MAEMTEATNMETSPVENNNELALITPETQPKRRKKKSMVWEYFTIETVSAGCRRACCNRCKQSFAYSTGSKVAGTSHLKRHIAKGTCPALLRDQYNNQLTPYSPKTGGGEPRKRRYRSPSSPFIPFDQDRCRHEIARMIIMHEYPLHMVEHPGFIAFVQNLQPRFDKVSFNTVQGDCVATYLREKQSLMKLIEGIPGRVCLTLDMWTSNQTLGYVFITGHFIDFEWKLQRRVLNVIMEPYPDSDSALSHAVAACLSDWSLEGKLFSLIFNHPTSEAGLENLRPLLCTKNPLILNGQLLLGNCIARNLSSMAKDVLGAGHEIVKKIRDSVKYVKTSESHDEKFVQVKNQLQVPSEKSLILDNQNQWNTTYQMLAAASELKEVFNCLDTSDPDYKLAPSMEDWKVAETLCTFLKPLFDAASILMTTTNPTAITFFHEAWKIHADLGRSIANDDPFISNIAKSMLEKIDKYWKDCSLILAIAVVMDPRFKMKLVEFSFTKIFGEDAPTYIKIVDDGIHELFLEYVALPLPLTPTYTEGNVGNNGKTDESQQGNLLSDQGLTDFDVYIMETSSQQMKSELDQYLEESLLPRVQEFDVLGWWKLNKMKYPTLSKMARDILSIPVSAAAPDSVFDIIIKQLDEYRSSLRPETVEALICAKDWLHHGSEESNALVKMEF	zf-BED--DUF-domain--Dimer_Tnp_hAT--	I
Gbar_A08G009840.6	GbaZf-BED22_I	MAEMTEATNMETSPVENNNELALITPETQPKRRKKKSMVWEYFTIETVSAGCRRACCNRCKQSFAYSTGSKVAGTSHLKRHIAKGTCPALLRDQYNNQLTPYSPKTGGGEPRKRRYRSPSSPFIPFDQDRCRHEIARMIIMHEYPLHMVEHPGFIAFVQNLQPRFDKVSFNTVQGDCVATYLREKQSLMKLIEGIPGRVCLTLDMWTSNQTLGYVFITGHFIDFEWKLQRRVLNVIMEPYPDSDSALSHAVAACLSDWSLEGKLFSLIFNHPTSEAGLENLRPLLCTKNPLILNGQLLLGNCIARNLSSMAKDVLGAGHEIVKKIRDSVKYVKTSESHDEKFVQVKNQLQVPSEKSLILDNQNQWNTTYQMLAAASELKEVFNCLDTSDPDYKLAPSMEDWKVAETLCTFLKPLFDAASILMTTTNPTAITFFHEAWKIHADLGRSIANDDPFISNIAKSMLEKIDKYWKDCSLILAIAVVMDPRFKMKLVEFSFTKIFGEDAPTYIKIVDDGIHELFLEYVALPLPLTPTYTEGNVGNNGKTDESQQGNLLSDQGLTDFDVYIMETSSQQMKSELDQYLEESLLPRVQEFDVLGWWKLNKMKYPTLSKMARDILSIPVSAAAPDSVFDIIIKQLDEYRSSLRPETVEALICAKDWLHHGSEESNALVKMEF	zf-BED--DUF-domain--Dimer_Tnp_hAT--	I
Gbar_A08G009840.7	GbaZf-BED23_I	MAEMTEATNMETSPVENNNELALITPETQPKRRKKKSMVWEYFTIETVSAGCRRACCNRCKQSFAYSTGSKVAGTSHLKRHIAKGTCPALLRDQYNNQLTPYSPKTGGGEPRKRRYRSPSSPFIPFDQDRCRHEIARMIIMHEYPLHMVEHPGFIAFVQNLQPRFDKVSFNTVQGDCVATYLREKQSLMKLIEGIPGRVCLTLDMWTSNQTLGYVFITGHFIDFEWKLQRRVLNVIMEPYPDSDSALSHAVAACLSDWSLEGKLFSLIFNHPTSEAGLENLRPLLCTKNPLILNGQLLLGNCIARNLSSMAKDVLGAGHEIVKKIRDSVKYVKTSESHDEKFVQVKNQLQVPSEKSLILDNQNQWNTTYQMLAAASELKEVFNCLDTSDPDYKLAPSMEDWKVAETLCTFLKPLFDAASILMTTTNPTAITFFHEAWKIHADLGRSIANDDPFISNIAKSMLEKIDKYWKDCSLILAIAVVMDPRFKMKLVEFSFTKIFGEDAPTYIKIVDDGIHELFLEYVALPLPLTPTYTEGNVGNNGKTDESQQGNLLSDQGLTDFDVYIMETSSQQMKSELDQYLEESLLPRVQEFDVLGWWKLNKMKYPTLSKMARDILSIPVSAAAPDSVFDIIIKQLDEYRSSLRPETVEALICAKDWLHHGSEESNALVKMEF	zf-BED--DUF-domain--Dimer_Tnp_hAT--	I
Gbar_A08G009840.8	GbaZf-BED24_I	MAEMTEATNMETSPVENNNELALITPETQPKRRKKKSMVWEYFTIETVSAGCRRACCNRCKQSFAYSTGSKVAGTSHLKRHIAKGTCPALLRDQYNNQLTPYSPKTGGGEPRKRRYRSPSSPFIPFDQDRCRHEIARMIIMHEYPLHMVEHPGFIAFVQNLQPRFDKVSFNTVQGDCVATYLREKQSLMKLIEGIPGRVCLTLDMWTSNQTLGYVFITGHFIDFEWKLQRRVLNVIMEPYPDSDSALSHAVAACLSDWSLEGKLFSLIFNHPTSEAGLENLRPLLCTKNPLILNGQLLLGNCIARNLSSMAKDVLGAGHEIVKKIRDSVKYVKTSESHDEKFVQVKNQLQVPSEKSLILDNQNQWNTTYQMLAAASELKEVFNCLDTSDPDYKLAPSMEDWKVAETLCTFLKPLFDAASILMTTTNPTAITFFHEAWKIHADLGRSIANDDPFISNIAKSMLEKIDKYWKDCSLILAIAVVMDPRFKMKLVEFSFTKIFGEDAPTYIKIVDDGIHELFLEYVALPLPLTPTYTEGNVGNNGKTDESQQGNLLSDQGLTDFDVYIMETSSQQMKSELDQYLEESLLPRVQEFDVLGWWKLNKMKYPTLSKMARDILSIPVSAAAPDSVFDIIIKQLDEYRSSLRPETVEALICAKDWLHHGSEESNALVKMEF	zf-BED--DUF-domain--Dimer_Tnp_hAT--	I
Gbar_A08G025510.1	GbaZf-BED25_I	MEVANETVIKKPKRLTSVVWNHFERVRKADLCYAVCVHCNKKLSGSSNSGTTHLRNHLMRCLKRFNYDVSQLLSAKKRKKDNTLTIANLSYDEGQRKEEYLKPTIVKYEPEQRKDEVFNVQSSWFDQERSRLDLARMIILHGYPLAMVEHVGFKVFVKNLQPLFDVMPNSTVELSCMEIYGKERQKVHDMLSKLQGRINLAVEMWSSPENTNHVCMMAHYVGDDWKLQKKILNFVTLDSSHTDDLLSGVIIKCLMDWDIGSKLFAVTLDDFSTNDDIVLRIKEQISENKSRLSNGQLLDVRSAAHVLNSIVQDAMEALRLVIQKIRGTVRYVKSSQSIQGKFKEMVLQTGINSQKNLVLDCPIQWNSTYLMLETAIEYRNAFCQLPELDLDLALSDEEWEWASSITGYLKLFIEIINVFSSNKCPTANIYFPEICHVHIQLIDWCKSPDNFLSSLAAKMKAKFDKYWSKCSLSLAVAAILDPRFKMKLVEYYYSQIYGSTALERIKEVSDGLKELFNTYSICSTLMDQGSALPLSSLPSSSNDGRDRLKGFDKFLHETSQSQTAISDLEKYLDEPVFPRNCNFNILNWWRVHTPRYPILSMMARDVLGTPMSTVSQESAFHAGGRVLDSCRCPLTPETRQALICTQDWLRMQSDDPGPSSSHYALPLYVETN	zf-BED--DUF-domain--Dimer_Tnp_hAT--	I
Gbar_A08G025510.2	GbaZf-BED26_I	MEVANETVIKKPKRLTSVVWNHFERVRKADLCYAVCVHCNKKLSGSSNSGTTHLRNHLMRCLKRFNYDVSQLLSAKKRKKDNTLTIANLSYDEGQRKEEYLKPTIVKYEPEQRKDEVFNVQSSWFDQERSRLDLARMIILHGYPLAMVEHVGFKVFVKNLQPLFDVMPNSTVELSCMEIYGKERQKVHDMLSKLQGRINLAVEMWSSPENTNHVCMMAHYVGDDWKLQKKILNFVTLDSSHTDDLLSGVIIKCLMDWDIGSKLFAVTLDDFSTNDDIVLRIKEQISENKSRLSNGQLLDVRSAAHVLNSIVQDAMEALRLVIQKIRGTVRYVKSSQSIQGKFKEMVLQTGINSQKNLVLDCPIQWNSTYLMLETAIEYRNAFCQLPELDLDLALSDEEWEWASSITGYLKLFIEIINVFSSNKCPTANIYFPEICHVHIQLIDWCKSPDNFLSSLAAKMKAKFDKYWSKCSLSLAVAAILDPRFKM	zf-BED--DUF-domain--Dimer_Tnp_hAT--	I

		KLVEYYYSQIYGSTALERIKEVSDGLKELFNTYSICSTLMDQGSALPLSSLPSSSNDGRDRLKGFDKFLHETSQSQTAISDL<br>EKYLDEPVFPRNCNFNILNWWRVHTPRYPILSMMARDVLGTPMSTVSQESAFHAGGRVLDSCRCPLTPETRQALICTQD<br>WLRMQSDDPGPSSSHYALPLYVETN		
Gbar_A09G008820.1	GbaZf-BED27_I	MSTEPTSIKGSVTPPTSIDSENSGVGASSQANVTTGKRKATPQRSEVWSHFTKIINSEGASKAKCNYCQKEFCCDVKKNG<br>TGSLKYHIGSCKKNSSNVVDPSQGQLVLPRKGVEEGEGHLSTWRFDQEACRKGLAQMIVIDELPFKFAESEGFKKFMFV<br>ACPRFHIPSRTTMTRDVYQLYLDERVKIKQLLRSSCSRVCLTTDTWTSLQRVNYLCITAHFIDNDWKLNKKILNFCPISSHK<br>GESIGMVIEKCFLNWGIDKLFTVTVDNASSNDVAIGYLRKKFNPRGGLVQNGKYLHMRCMAHIVNLIVVEGLKEMNKSVE<br>RVRGAVRYVRQSPARLQKFKKCVVMEKIECKKMLCLDVCTRWNSTYLMLDTAQKFERAFERFEEQDTNFRAELERGEG<br>WPNAQLNSNVGFNVMAIKMKEKYDKYWGDIDKMNLLMFVACILDPRQKLKYLEFALSEMSSSEKACEMMQKLKESLYEL<br>FDEYKPPLYSTCSRSSVPTHVSLGEPQQKMKRRMQALYKKRELEIGGEDKTSELDKYLAEANEDFIEDFDILLWWKMNSP<br>RFQILSKMARDVLAIPVSTVASESTFSTGGRVLDQYRSSLTPKIVQALVCTQDWIRKSSSQEDIKKIEEQIQELDMIENDLEI<br>MSSTLTSISLCIISWNKVTMAL	zf-BED--DUF-domain--Dimer_Tnp_hAT--	I
Gbar_A09G019340.1	GbaZf-BED28_I	MVRERDVCWEYAEKLDGNKVRCKFCLRVLNGGISRLKHHLSRLPSKGVNPCNKVRDDVTDRVRAIISSKEDIKEIPSVKK<br>QKIAEVRAPGNMSTGSKISPLETLSPAAKVFPTVLSIAASTLSDQETVERSIALFFFENKLDFSVARSSSYQAMIDAVGKFG<br>PGLIAPSVETLKTTWLKRIKSEVTLHLKDAEKEWATTGCTIIADTWTDNKSKALINFLVSSPSRTFFHKSVDASSYFKNTKCL<br>ADLFDSVIQDFGQENVVQIIMDSSFNYTGISSHILQNYGTIFLSPCASQCLNLILEEFSRVDWVNRCILQAQTVSKFLYNNAS<br>MLDLMKKFTGGQELIRTGITKSVSCFLSLQSTLKQRSRLKHMFNSPEYSTNSSYANKPQSISCIAIVEDNDFWRAVEECVAI<br>SEPFLKVLREVSGGKPAVGSIYELMTRAKESIRTYYIMDESKCKTFLDIVDRQWRDQLHSPLHSAGAFLNPSIQYNPEVKF<br>LGSIKEDFFKVLEKLLPTPELRRDITNQIFTFTRAKGMFACNLAMEARDTVSPGLWWEQFGDSAPVLQRVAIRILSQVCST<br>FTFERHWSTFQQIHTEKRNKIDKETLTDVVYINYNLKLAREMKTMPTDSDPIQFDDIDMTSEWVEESENPSPTQWLDRFG<br>SALDGGDLNTRQFSAAMFGNDHIFGL	zf-BED--DUF-domain--Dimer_Tnp_hAT--	I
Gbar_A09G019340.2	GbaZf-BED29_I	MIMYCSIFLAVVRERDVCWEYAEKLDGNKVRCKFCLRVLNGGISRLKHHLSRLPSKGVNPCNKVRDDVTDRVRAIISSKE<br>DIKEIPSVKKQKIAEVRAPGNMSTGSKISPLETLSPAAKVFPTVLSIAASTLSDQETVERSIALFFFENKLDFSVARSSSYQA<br>MIDAVGKFGPGLIAPSVETLKTTWLKRIKSEVTLHLKDAEKEWATTGCTIIADTWTDNKSKALINFLVSSPSRTFFHKSVDA<br>SSYFKNTKCLADLFDSVIQDFGQENVVQIIMDSSFNYTGISSHILQNYGTIFLSPCASQCLNLILEEFSRVDWVNRCILQAQT<br>VSKFLYNNASMLDLMKKFTGGQELIRTGITKSVSCFLSLQSTLKQRSRLKHMFNSPEYSTNSSYANKPQSISCIAIVEDNDF<br>WRAVEECVAISEPFLKVLREVSGGKPAVGSIYELMTRAKESIRTYYIMDESKCKTFLDIVDRQWRDQLHSPLHSAGAFLNP<br>SIQYNPEVKFLGSIKEDFFKVLEKLLPTPELRRDITNQIFTFTRAKGMFACNLAMEARDTVSPGLWWEQFGDSAPVLQRVA<br>IRILSQVCSTFTFERHWSTFQQIHTEKRNKIDKETLTDVVYINYNLKLAREMKTMPTDSDPIQFDDIDMTSEWVEESENPSP<br>TQWLDRFGSALDGGDLNTRQFSAAMFGNDHIFGL	zf-BED--DUF-domain--Dimer_Tnp_hAT--	I
Gbar_A09G019340.3	GbaZf-BED30_I	MVRERDVCWEYAEKLDGNKVRCKFCLRVLNGGISRLKHHLSRLPSKGVNPCNKVRDDVTDRVRAIISSKEDIKEIPSVKK<br>QKIAEVRAPGNMSTGSKISPLETLSPAAKVFPTVLSIAASTLSDQETVERSIALFFFENKLDFSVARSSSYQAMIDAVGKFG<br>PGLIAPSVETLKTTWLKRIKSEVTLHLKDAEKEWATTGCTIIADTWTDNKSKALINFLVSSPSRTFFHKSVDASSYFKNTKCL<br>ADLFDSVIQDFGQENVVQIIMDSSFNYTGISSHILQNYGTIFLSPCASQCLNLILEEFSRVDWVNRCILQAQTVSKFLYNNAS<br>MLDLMKKFTGGQELIRTGITKSVSCFLSLQSTLKQRSRLKHMFNSPEYSTNSSYANKPQSISCIAIVEDNDFWRAVEECVAI<br>SEPFLKVLREVSGGKPAVGSIYELMTRAKESIRTYYIMDESKCKTFLDIVDRQWRDQLHSPLHSAGAFLNPSIQYNPEVKF<br>LGSIKEDFFKVLEKLLPTPELRRDITNQIFTFTRAKGMFACNLAMEARDTVSPGLWWEQFGDSAPVLQRVAIRILSQVCST<br>FTFERHWSTFQQIHTEKRNKIDKETLTDVVYINYNLKLAREMKTMPTDSDPIQFDDIDMTSEWVEESENPSPTQWLDRFG<br>SALDGGDLNTRQFSAAMFGNDHIFGL	zf-BED--DUF-domain--Dimer_Tnp_hAT--	I
Gbar_A10G015300.1	GbaZf-BED32_IV	MPPREEFSTKGLEGAPSNDIGWHFGTPVPNTKGNIICKLCGKVVKGGITRFKEHISHKTGNVAPCPNVTEIGQGVKLPTPY<br>EISDVYLESEYQRVHDWVNGLKTHWKELGATLMCDGWTNSLNQMHIINFLVYCSKGTIFWKSVDASSVRSRDAEFYYSL<br>LDSVVEEIGENYIVQIVTDNEAAMKAAGKKLMLKRKHLYWTSCAAHCLDLCLEDIGKKPSVAKVLDEAKKVTCFIYNHIWTV<br>DLMKKYTQGKQILRPALTRFATHFIQLEEITRQKQGLREMFNSKEFKESKWGQQKSGPAYEVKKIVLGKDFWKKANDLIK<br>VYEPLVKVLRLVDSDEKPIMGFIYEAVDRAKRAIQQDCRYFTEYEKIIDNRWNFMHSDLHSAGYFLNPQFQFGVEYSENVL<br>IETLEGTRSVIEKLEHSLDTQVRMVNQLLLFRDKHETFGTPQAQRAWKQMNPGKHTFSYIHTKARNRLKYKKLEKLVFTYY<br>NMRLQIRHQKRMSTDDINASFNPISLDHIFEDVDPLSEWLHEKENPLLDGENAGVLPVDTSDDEMDVDQSQQQNLSHSS<br>SSSMPSQSGDGPDGGGLSPIDEDDGYSGDRGEIRSSSQYGGEYGVGTTSGHFRDRSEFDGNMFPEPRRDRSELRAPS<br>KGKGKKHTSIGSSSGRRSSSSNLGYSDSSTSTQGFYPPEQPSYFQPSHGYPQPYGYYPPFPNYGVPYQPQMHPPPPM<br>YHPPPPLMYPPPQIYPPYQLYENQGENVTFFGYIFGQRSREPSQERSQSEGEGSDLPCHSSNW	zf-BED--DUF-domain	IV
Gbar_A11G023000.1	GbaZf-BED34_II	MPCSRNKEEVAPSDDYGWRWGELVEGNRNNVKCRFCGRIIKRGITQLKEYLVVKKGNVAPCPHGSVKVRKSIGQQLQE<br>YHIEKAVRQRRKEELEERISLGDRGNYGDSGDDDEELTITRRKSVRSQVE	zf-BED	II
Gbar_A11G031170.1	GbaZf-BED35_I	MEVANESTAKKPKRLTSVVWNHFERVKKADICYAVCVHCNKKLSGSSNSGTTHLRNHLMRCLKRSNYDVSQLLAVKRRK<br>KENTLTIANISYDEGQRKEDYMKPTIVKYEQDQRKDEAFNLGSSWFDPERSRLDLARMIILHGYPLAMVEQVGFKVFVKN<br>MQPLFDVVHNSTIELSCVEIYMKEKQRIYDILSKLQGRINLAIEMWSSPENSKYVCLTAHYVDDEWKLQKKILNFLTLDSSH<br>TEDMLSDVIIKCLMDWDVDCKLFAMTFDDCSTNDDIVSRIKDQVSESRPRLSNGQLLDVRSAAHVLNSIAQDAIEALQVVIQ<br>KIRGSVKYVKSSQSILGKFNEIAQQQGIDNHKIVVLDYPIRWNSTYMMLETAVEYRNVFHHLPELDPDFALSDEEWERASS<br>IVSYLKLLIEIINVFSSNKCPTANIYFPEICHVHIQLIEWCKSSDAFLSSLATKMKAKFDKYWSKCSLALAVAAILDPRFKMKLV<br>EYYYSQIYGSTALERIKEASDGIKELFNAYSICSTLIDQGSALPGSSLPSSSNDTRDRLKGFDKFLHETSQSQTAISDLEKYL<br>DEPVFPRNCDFNILNWWRVHTPRYPILSMMARDVLGTPMSTVAQEFAFNAGGRMLDSNQSSPPPDTQQALICTRDWLR<br>TQSDDATPSSSHYALPLYVEAN	zf-BED--DUF-domain--Dimer_Tnp_hAT--	I
Gbar_A11G031170.2	GbaZf-BED36_I	MEVANESTAKKPKRLTSVVWNHFERVKKADICYAVCVHCNKKLSGSSNSGTTHLRNHLMRCLKRSNYDVSQLLAVKRRK<br>KENTLTIANISYDEGQRKEDYMKPTIVKYEQDQRKDEAFNLGSSWFDPERSRLDLARMIILHGYPLAMVEQVGFKVFVKN<br>MQPLFDVVHNSTIELSCVEIYMKEKQRIYDILSKLQGRINLAIEMWSSPENSKYVCLTAHYVDDEWKLQKKILNFLTLDSSH<br>TEDMLSDVIIKCLMDWDVDCKLFAMTFDDCSTNDDIVSRIKDQVSESRPRLSNGQLLDVRSAAHVLNSIAQDAIEALQVVIQ<br>KIRGSVKYVKSSQSILGKFNEIAQQQGIDNHKIVVLDYPIRWNSTYMMLETAVEYRNVFHHLPELDPDFALSDEEWERASS<br>IVSYLKLLIEIINVFSSNKCPTANIYFPEICHVHIQLIEWCKSSDAFLSSLATKMKAKFDKYWSKCSLALAVAAILDPRFKMKLV<br>EYYYSQIYGSTALERIKEASDGIKELFNAYSICSTLIDQGSALPGSSLPSSSNDTRDRLKGFDKFLHETSQSQTAISDLEKYL<br>DEPVFPRNCDFNILNWWRVHTPRYPILSMMARDVLGTPMSTVAQEFAFNAGGRMLDSNQSSPPPDTQQALICTRDWLR<br>TQSDDATPSSSHYALPLYVEAN	zf-BED--DUF-domain--Dimer_Tnp_hAT--	I
Gbar_A12G013140.1	GbaZf-BED37_III	MVEEMAPLRSIGYVDPGWEHGTAQDERKKKVKCNYCGKVVSGGIFRLKQHLARLSGEVTHCEKVPEEVCLNMRKNLEG<br>CRSGRKRRQFDYEQAALSIQSNEYSDGEDASASYKHKGKKVMGDKNLVIKFTPLRSLGYVDPGWEHCVAQDEKKRRVK<br>CNYCEKIISGGINRFKQHLARIPGEVAYCEKAPEEVYLKIKENMKWHRTGRRHRKPDTKEISTFYMHSDNEDEGGEEERY<br>LQCVSKDILAIDDKVSDNDIRNNVRGRSPGSSGNGAEPLLKKSRLDSVFLKSLKSQTSAHYKQPRARTGFEKKTHREVISA<br>ICKFFYHAGIPSNAANSPYFHKMLELVSQYGQGLQGPSSRLISGRLLQEEIANIKEYLVELKTSWAITGCSVMADSWNDAQ<br>GRMLINFLVSCPRGVYFLSSVDATDIIEDAVHLFKLLDKAVDEVGEEYVVQVITRNTLSFRNAGKMLEEKRRNLFWTPCAV<br>YCIDRMLEDFVNIKWVGECVDKAKKVTRFIYNNTWLLNFMKKEFTKGQELLQPAVTKFGTNFFTLQSLLDQRVGLKRMFQ<br>SNRWLSSRFSKSDEGKEVEKIVLNVSFWKKMQYVKKSFEPVAEVLQRIGSDKIRSMPFIYNDICRTKLAIKAIHGDDVRKY<br>GPFWSVIESNWSSLFHHPLYVAAYFLNPSYRYRPDFLMNPEVIRGLNECIVRLEADNGKKIAASMQIPDFVSAKADFGTDL<br>AISTRSELDPASWWQQHGISCLELQRIAIRILSQTCSSIGCEHNWSAFDQVHIKRHNCLSRKRLNDQTYVHYNLRLRERQL<br>GRKPDELVSFDSAMLESVLDDWLVETEKLAMHEDEEIIYTEVEQFCGDDMDEHESEEKRPAEMVTIAGFIEPLDVIPSAGG<br>VTTDDDGLDFLDDDLTD	zf-BED--zf-BED--DUF-domain--Dimer_Tnp_hAT--	III
Gbar_A13G002040.1	GbaZf-BED38_I	MSSNLEPIPITSQKHDPAWKHCQMFKNGERVQLKCIYCGKIFKGGGIHRIKEHLAGHKGNAATCLRVPSDVRVLMQESLD<br>GVVVKKRKKQKIAEEITNVNQVSTEIQAYGDQVDTNTGLLMIEKSDTLEPSSSLLVNREGTSNVAGERRKRGRGKNLPAE<br>ANALSFVPVELGARRVNNHVHMAIGRFLFDIGATMDAVNSVYFQPMVDAIVSGGSGALMPSCNDLQGWILRKLVEEVKS<br>ENDKVMGAWVRTGCSILVNQWNTQTGRILLNFLVYCPEGTVFLKSIDASSVINSSDALYELLKQVVEEVGSKHVLQVITNG<br>EEQYIVAGRRLAETFPTLYWTPCAAHCVDLILEDFAKLEWINAIIEQARSITKFIYNHSVVLNMVRRYTFGNDIVEPAATRSA<br>TNFTTLTRMVDLKNNLQAMVTSQQWVDCPYSKKPGGLAMLDLVSNQSFWSSCILIVRLTNPLLRVLRMVGSKKRPAMGY<br>VYAGMYRAKETIKKELVKRNEYMVYWNIIDHWWEQQWHHPLHAAGFYLNPRFFYSMEGDMPNEMLSGMLDCIEKLIPDV<br>TVQDKISKEINSYKNSVGDFGRKMAVRARDTLLPVEWWSTYGGSCPNLARLAIRVLSQTCSTFGLKHNHIPFEKLYETRN	zf-BED--DUF-domain--Dimer_Tnp_hAT--	I

		CLEQQRLRDLIFVQCNLQLRQIGYESKQHDSMQPLSSESASIVEDWVTGIDAFLDDDTYPDWTTLETLSVNTMLLRPGDEYLITVRFAQGGPLFPNCSKYAALGKHL		
Gbar_D02G000700.1	GbaZf-BED39_I	MDNFDQKLGPEFFKNLSAEAVTPLNVVHEEIYESSSKRPKTTSKVWDIFEKLPAQQGDSKAICKLCRRIYTAKTTSGTSHLRRHIEACVKRGNHEVDQRSIEACFKPVKRNANRLTLSHDTLIAATTSLKNYKLDVDEIHRAIAMMIIVDEQPFSVVEDAGFRRLLSAACPEFPVLSRSSIKRDIISIYVKERENIRELLATCPGRICLTSSTWKSDSDDHFNCVTTHFIDHEWRLQKRILSFKLMPPPYDSLSVADEIALCMVQWNIEHKVFSVTLENLSSDDCVADMLRSRLAAKKYLPCKGVFFHVSCFFRILNSIVQAGLNLVVDIIAKLRLGIKYVQQSPHRKKNFYIIAKTLNLDTQRKLCLDTPARWNSTYNMIEVAFCYNNAFMYLAEQDKNFLHKLSEDEWEKLSVLYKFLKVFYEVTCVFFRNRQPTSNLYFKAAWKVHSRLFDMVRGPENFMTRMVREMHSKLNQYWSAYNLILSCAAVLDPRCKIKFVEYCYTKLYGSGAQKYVSVSVNTLYGLFDEYMQNSARPSQTTLLSTAASKISNDKDNNDGFEDYETFQSARFRTQVEKSQLDLYLEEPSHDLNSEIDVLEYWTLCSLRYPELSKMARDVLTIPVSTIASDSAFDISPQVISTDRSSLKPKMLQALVCLQDWMLASDRTRGLGSMESKPEDDSSSSSDGDDDY	zf-BED--DUF-domain--Dimer_Tnp_hAT--	I
Gbar_D02G000700.2	GbaZf-BED40_I	MDNFDQKLGPEFFKNLSAEAVTPLNVVHEEIYESSSKRPKTTSKVWDIFEKLPAQQGDSKAICKLCRRIYTAKTTSGTSHLRRHIEACVKRGNHEVDQRSIEACFKPVKRNANRLTLSHDTLIAATTSLKNYKLDVDEIHRAIAMMIIVDEQPFSVVEDAGFRRLLSAACPEFPVLSRSSIKRDIISIYVKERENIRELLATCPGRICLTSSTWKSDSDDHFNCVTTHFIDHEWRLQKRILSFKLMPPPYDSLSVADEIALCMVQWNIEHKVFSVTLENLSSDDCVADMLRSRLAAKKYLPCKGVFFHVSCFFRILNSIVQAGLNLVVDIIAKLRLGIKYVQQSPHRKKNFYIIAKTLNLDTQRKLCLDTPARWNSTYNMIEVAFCYNNAFMYLAEQDKNFLHKLSEDEWEKLSVLYKFLKVFYEVTCVFFRNRQPTSNLYFKAAWKVHSRLFDMVRGPENFMTRMVREMHSKLNQYWSAYNLILSCAAVLDPRCKIKFVEYCYTKLYGSGAQKYVSVSVNTLYGLFDEYMQNSARPSQTTLLSTAASKISNDKDNNDGFEDYETFQSARFRTQVEKSQLDLYLEEPSHDLNSEIDVLEYWTLCSLRYPELSKMARDVLTIPVSTIASDSAFDISPQVISTDRSSLKPKMLQALVCLQDWMLASDRTSKSPYLIAYSNCCYTMHNCFV	zf-BED--DUF-domain--Dimer_Tnp_hAT--	I
Gbar_D02G000700.3	GbaZf-BED41_I	MDNFDQKLGPEFFKNLSAEAVTPLNVVHEEIYESSSKRPKTTSKVWDIFEKLPAQQGDSKAICKLCRRIYTAKTTSGTSHLRRHIEACVKRGNHEVDQRSIEACFKPVKRNANRLTLSHDTLIAATTSLKNYKLDVDEIHRAIAMMIIVDEQPFSVVEDAGFRRLLSAACPEFPVLSRSSIKRDIISIYVKERENIRELLATCPGRICLTSSTWKSDSDDHFNCVTTHFIDHEWRLQKRILSFKLMPPPYDSLSVADEIALCMVQWNIEHKVFSVTLENLSSDDCVADMLRSRLAAKKYLPCKGVFFHVSCFFRILNSIVQAGLNLVVDIIAKLRLGIKYVQQSPHRKKNFYIIAKTLNLDTQRKLCLDTPARWNSTYNMIEVAFCYNNAFMYLAEQDKNFLHKLSEDEWEKLSVLYKFLKVFYEVTCVFFRNRQPTSNLYFKAAWKVHSRLFDMVRGPENFMTRMVREMHSKLNQYWSAYNLILSCAAVLDPRCKIKFVEYCYTKLYGSGAQKYVSVSVNTLYGLFDEYMQNSARPSQTTLLSTAASKISNDKDNNDGFEDYETFQSARFRTQVEKSQLDLYLEEPSHDLNSEIDVLEYWTLCSLRYPELSKMARDVLTIPVSTIASDSAFDISPQVISTDRSSLKPKMLQALVCLQDWMLASDRTSKSPYLIAYSNCCYTMHNCFV	zf-BED--DUF-domain--Dimer_Tnp_hAT--	I
Gbar_D02G000700.4	GbaZf-BED42_I	MDNFDQKLGPEFFKNLSAEAVTPLNVVHEEIYESSSKRPKTTSKVWDIFEKLPAQQGDSKAICKLCRRIYTAKTTSGTSHLRRHIEACVKRGNHEVDQRSIEACFKPVKRNANRLTLSHDTLIAATTSLKNYKLDVDEIHRAIAMMIIVDEQPFSVVEDAGFRRLLSAACPEFPVLSRSSIKRDIISIYVKERENIRELLATCPGRICLTSSTWKSDSDDHFNCVTTHFIDHEWRLQKRILSFKLMPPPYDSLSVADEIALCMVQWNIEHKVFSVTLENLSSDDCVADMLRSRLAAKKYLPCKGVFFHVSCFFRILNSIVQAGLNLVVDIIAKLRLGIKYVQQSPHRKKNFYIIAKTLNLDTQRKLCLDTPARWNSTYNMIEVAFCYNNAFMYLAEQDKNFLHKLSEDEWEKLSVLYKFLKVFYEVTCVFFRNRQPTSNLYFKAAWKVHSRLFDMVRGPENFMTRMVREMHSKLNQYWSAYNLILSCAAVLDPRCKIKFVEYCYTKLYGSGAQKYVSVSVNTLYGLFDEYMQNSARPSQTTLLSTAASKISNDKDNNDGFEDYETFQSARFRTQVEKSQLDLYLEEPSHDLNSEIDVLEYWTLCSLRYPELSKMARDVLTIPVSTIASDSAFDISPQVISTDRSSLKPKMLQALVCLQDWMLASDRTRGLGSMESKPEDDSSSSSDGDDDY	zf-BED--DUF-domain--Dimer_Tnp_hAT--	I
Gbar_D02G000700.5	GbaZf-BED43_I	MDNFDQKLGPEFFKNLSAEAVTPLNVVHEEIYESSSKRPKTTSKVWDIFEKLPAQQGDSKAICKLCRRIYTAKTTSGTSHLRRHIEACVKRGNHEVDQRSIEACFKPVKRNANRLTLSHDTLIAATTSLKNYKLDVDEIHRAIAMMIIVDEQPFSVVEDAGFRRLLSAACPEFPVLSRSSIKRDIISIYVKERENIRELLATCPGRICLTSSTWKSDSDDHFNCVTTHFIDHEWRLQKRILSFKLMPPPYDSLSVADEIALCMVQWNIEHKVFSVTLENLSSDDCVADMLRSRLAAKKYLPCKGVFFHVSCFFRILNSIVQAGLNLVVDIIAKLRLGIKYVQQSPHRKKNFYIIAKTLNLDTQRKLCLDTPARWNSTYNMIEVAFCYNNAFMYLAEQDKNFLHKLSEDEWEKLSVLYKFLKVFYEVTCVFFRNRQPTSNLYFKAAWKVHSRLFDMVRGPENFMTRMVREMHSKLNQYWSAYNLILSCAAVLDPRCKIKFVEYCYTKLYGSGAQKYVSVSVNTLYGLFDEYMQNSARPSQTTLLSTAASKISNDKDNNDGFEDYETFQSARFRTQVEKSQLDLYLEEPSHDLNSEIDVLEYWTLCSLRYPELSKMARDVLTIPVSTIASDSAFDISPQVISTDRSSLKPKMLQALVCLQDWMLASDRTSKSPYLIAYSNCCYTMHNCFV	zf-BED--DUF-domain--Dimer_Tnp_hAT--	I
Gbar_D02G001040.1	GbaZf-BED44_I	MSTEPTSIEGSVTPPTSIDSKNSGVGASSQTKGTTGKRKATPQRSEVWSYFTKIINSEGASKAKCNYCQKEFCCDMKKNGIGSLKYHIGSCKKNPSNVQGQLVLPRKRVEGGEENLSTWRFDQETCRKGLAQMIVIDELTFKFVESESFKKFMFVACPRFHIPSRTTMIRDVYQLYLDERVKIKQLLRSSCSRVCLTTDTWTSLQRVNYLCITAHFIDNDWKLNKRILNFCPISSHKGESIGMVIEKCLLNWGIDKLFTVTVDNASLNDVAIGYLRKKFNLRGGLVQKGKYLHMRCMAHIMNLIVVEGLKEMNKSVERVRGAVRYVRQFPARLQKFKECVVVEKIECKKTLCLDVCTRWNSTYSMLDTALNFERAFERFEEQDTNFRAELERGEGWPSVDDWDNVRNLRDFLEHFYEVTLRISGTSYVTSNNFFDELSEIDILLRDAQLNSNVAFNVMAIKMKEKYDKYWGDIDKINLLMFVACVLDPRQKLKYLEFVLSEMSSSEKVCEMMQKLKGSLYELFDEYKPPLHSTCSQSSVPTHVSLGEPQQKMQRRIQALYKKCELEICGEDKTSELDKYLAEANEGFVEDFDILLWWKVNSPRFPTLSKMARDLLAIPVSTVASESAFSTGGRVLDQYRSSLTPKIVQALVCTQDWIRKSSSQEDIKKIEEQIQELDKIENGLMQLEIFWKEKMDTNGEY	zf-BED--DUF-domain--Dimer_Tnp_hAT--	I
Gbar_D02G001280.1	GbaZf-BED45_VIII	CEIDFKRRRDDEMVVVYYKFKLDKLVKDAKAMERELVRLKEINPTIVIMLDFYSNHTHSNFLTCFKDSFQYSLKTLDCWAELDLYFDEEYEWECHIEAWEGNNVIRRHPSLTEWQHLFSMAGFSRIALNHRKGIDLIVKDVNPLNDFFSMWNQSWLEIMGKEEECLILGYKECPMFFLSAWKPKVEEEHLNFNSSKDKFGQGFNPYPTPLRPLQPFPEGLTLSRVAVLAEIHDILNHLYCEHKFSSALIWASKVDNMNETKSDPNKKFTFSIQSNSCYSKDLNSYKFMRSCEYKIEQTIIEKALESKDGYHFEPSLTKSDIDDYMYLQRAKNCDVDVVVAICLQNRYTSNDVYVVEFYWPATESEISKSFTPRIFNDLKHMEKKFVTVKVQGTEKAISNIPTSSYTARPLKIAEETEDVDAVEINGVNVQRGVVPNFPSPITIQSSSKVVAAPSNTLEEPHNQIFPNGDPEIVRANKEEPSKATQRELRSKVWGHFDSFEEDEKQVAKCKHCPKVLTGSSKSGTTHLNNHSKVCPGKKKQNQESQLILPVDTNEGSLRFDKKRSHMDLAKMMIKLQCPLDMAEQETFKNFVKGLQPMFEFQSKDILSYIHRIYDEEKEKLQLYFDKLASKFNLTVSLLKNNSGKTIYCCLISHFIDDGWELKRKILALKILEHINDTKALGEIIRSLVLEWNISNKVCSITVDNSFLNDSMVDQIKEICLSDQGSVSSDHWFISFTLLEDGFREMDGILFKLRKSIEYVTETRHGKLKFQEAVDQVKLQGGKLWDDLSFRLESDFDILDSALRSREIFCKLEQIDDNFKLNPTMEEWENAVALQSCLKCFDDIKGTQCLPVSLYLPKLCDTYKKFLQLEKSSHSFVKLMKRKFDRYWSLCNLALAVASVLDPRLKFKIVELSYRVIYGHDSKMRLNMFQKVLRDVYYEYASEAKNLTSSASVLDDFNCSTIGLGNDSILDSLSKFASASNFNEEASWKLELELYLDEPLLPMDGAFFDILGWWCDKSQRFPILAKMAQDFLAIPVSISTSCSNISAMINNPAYSTLNPESMEALVCSENWLETPKER	GRAS--zf-BED--DUF-domain--Dimer_Tnp_hAT--	VIII
Gbar_D02G001280.2	GbaZf-BED46_I	MNETKSDPNKKFTFSIQSNSCYSKDLNSYKFMRSCEYKIEQTIIEKALESKDGYHFEPSLTKSDIDDYMYLQRAKNCDVDVVVAICLQNRYTSNDVYVVEFYWPATESEISKSFTPRIFNDLKHMEKKFVTVKVQGTEKAISNIPTSSYTARPLKIAEETEDVDAVEINGVNVQRGVVPNFPSPITIQSSSKVVAAPSNTLEEPHNQIFPNGDPEIVRANKEEPSKATQRELRSKVWGHFDSFEEDEKQVAKCKHCPKVLTGSSKSGTTHLNNHSKVCPGKKKQNQESQLILPVDTNEGSLRFDKKRSHMDLAKMMIKLQCPLDMAEQETFKNFVKGLQPMFEFQSKDILSYIHRIYDEEKEKLQLYFDKLASKFNLTVSLLKNNSGKTIYCCLISHFIDDGWELKRKILALKILEHINDTKALGEIIRSLVLEWNISNKVCSITVDNSFLNDSMVDQIKEICLSDQGSVSSDHWFISFTLLEDGFREMDGILFKLRKSIEYVTETRHGKLKFQEAVDQVKLQGGKLWDDLSFRLESDFDILDSALRSREIFCKLEQIDDNFKLNPTMEEWENAVALQSCLKCFDDIKGTQCLPVSLYLPKLCDTYKKFLQLEKSSHSFVKLMKRKFDRYWSLCNLALAVASVLDPRLKFKIVELSYRVIYGHDSKMRLNMFQKVLRDVYYEYASEAKNLTSSASVLDDFNCSTIGLGNDSILDSLSKFASASNFNEEASWKLELELYLDEPLLPMDGAFFDILGWWCDKSQRFPILAKMAQDFLAIPVSISTSCSNISAMINNPAYSTLNPESMEALVCSENWLETPKER	zf-BED--DUF-domain--Dimer_Tnp_hAT--	I
Gbar_D02G001290.1	GbaZf-BED47_XI	MKTTFVTVKVQGPEIKFQEEAISSIPTSSNTAMPLKIAEEARDIHAIEINAHIEQIVETKRNKQRKSWSKVWVDFDKFEEHGKQVAKCKHCPKVLTGSSKSGTTHLNNHSKVCPGKKKQNQESQLILPVDTNERSSTFDQERSHLDLVKMVIRHQYPLDLAGQEAFKNFVKGLQPMYEFQSRDKLLSDIHRIYNEEREKLQLYFDQLACKLNLTVSLWKNNHGKTAYCCLIAHFIDDSWELKMKTLGLRTLEHINDTKAVGGIIQSLVSEWNIGSKVCSITVDNSFLDDSMVQQIKENCLSNLVSLSSTHWFINCTLLEDGFREMDDLLFNLKKSIEYVTETKHGRLKFQEAVDQVKLQGGKSWDDLSLKLESDFGILDSALRSREIFCKLEQIDGNFKLNPSMEEWENAAALQSCLRCFDDIKGTQSLTVSLYFPKLCHIYKKFLQLEKSNPSFVTLMKRRFDHYWRLCNSALAVASVLDPRLKFKVVEFSYILIYGHDSKVQLNTFREVLRNVYNEYANETKNQTTSASVLDDINWLGNNSIWDSFSKFVTASNFNEASLKSELELYLDEPLLPKDGAIFDILGWWCDKSQKFPILAKMAQDFLAIPLSVFTPCSNIKATINNPAYNILNPESMEALVCSENWLETPNGNDGENHEPTQTTDKGKRKLDEDTCVRKKSKPSNCEKAISTEDIAKDFNNNEFNHGRNFKDVNEISSDDSSSDFDQSDQVQSSSSESDDETTLKEQGQWCEQDVRVYLVSNFTNKDYERLDKLERKKLNRKSIGRDKELNLTGDKLEHLLMVPRSTETQKKYHINEVVINAFFKLLKERSRKFSKAYINHYSFDSQAANLLIKGSRSEREVLARYKPDHLRGVHKLFLPLCLSDHWVLF	zf-BED--DUF-domain--Dimer_Tnp_hAT--Peptidase_C48--	XI

		YVDINAKKFSCLDPYQSSGILSFNSKNVDKILQWFKSFLLPEFGYKDANEWPYVERTDIPQQKNSVDCGVFVMKYGDCLT<br>YGDFFPFTQKDMIHFRRRIFLDLYRGRLHIKK		
Gbar_D02G001330.1	GbaZf-BED48_XI	MLEDVCFRYELPVALTWACEANTDKIMLDGKKYTLFMERTSCYTSNEGSQCFMEACAKHHIQEGQAIAGKALQSSANFH<br>FKPSITKLIKSDYPLFNAAQLFGSHAVVAICLQNHYIIGDVYVVEFYWPEIESEKSESLALDIFNDLKNMKKKFVTIRVGSNEG<br>TMHTRNAQPASSTNDLLSSNTTWSLNAVQPCDVHEMERHGLVESAPFSTPNPMSYGGVLQTQGPHKQEIGEKDFISQT<br>VSIGDYEIVKAYMETCKVPRTKQRKYLSKVWLAKCKHCNKDFTGSSKSGTTHLKNHLERCQSKKIKNQKRQLITSEIGDLIT<br>RDSDESNFTFDQERSRLDFAKMIIKHQCPLDMAEQEFFKIFVKNLQPMFEFQSKDILLSDIHRIYKEEKEKLQLYFDQVACN<br>FNLTISLWKNNLGKTAYCCLIAHFIDDNWGPKMKIIACKPLDHIYDTKALNEIIQSSVLEWNISKKVFSITMDNPYLSDDMFQ<br>KIKETCFSDQGSFPSTHWFIGCTFIKDGFREMDLILLKLRKSIEYVSEIAQGKLKFEEVVNQVKLQGGKSWDDLSLRLDSDF<br>GVLHSALESREIFCQLEKIDSNFKLNPSVEEWEMVLAFHSCLKCFDDIEGTQSLTANLYFPKLCNIYKKFLHLGKSNYPIVTL<br>MKRKFDYYWSLCNLAFSVATILDPRLKFKFVEFSYTEIYGHDSKINLNRFHKVLTDVYYEYANEARNLSKSTSDLDDSNSS<br>TTEIDNDCILESFSKFASASNFNEVASWKSELDCYLDEPLLPLDGAFDILYWWCINTKRFPTLAKMARDFLAMPISILAPCLN<br>FNAMITNPTYNNLNPESMEALVCSQNWLEIPKEKTNHGRNIAAAIEIPNDEPSFNGNQSDQFQSSSSESDDETSLREQGS<br>WCREDVRTYLVSNFTNKEVKRLNRWKRSELSGKKIGRDKDFQLMGENLTPLLMVPHCDETLIEYYIDDSKRSDKFPNGYI<br>KHYSFDSLIATCLIEGSKSEDEVLAWFKDEKLRGVHKLFLPMCLSAHWVLFCVDTKEKKISRLDPIPSSRIMSNSVEKQKIF<br>QWFTLYLLPQFGYNDAEKWAFEVRTDIPKQENSIDCGVFVMKYGDCLMHGDFFPFTQKDMIHFRRRIFLNIYRGRLHGKR	zf-BED--DUF-domain--Dimer_Tnp_hAT--Peptidase_C48--	XI
Gbar_D03G013660.1	GbaZf-BED49_I	MPPREEFPTKGLEGAPSNDIGWHFGTLVPNARGSIVCKLCGKVVKGGITRLKEHIAHKTGNVAPCPNVTGVIRESMMNVL<br>KESNTKKIDKKRRKDEFLSQLIEEEDEHEGFIDEVSAIRQATRESIQSQHEWHRREEFRRSTGGWDNIYEEGRSSHGSAR<br>EHNRERTSKSILGESEFTLRGAIPELVRSKSSKQPKVNDSFLKSFRRKIGEAVSKFIIYERLPFQLASSPWLYNLIQLPTPYE<br>VSDVYLESEYQRVHDWVNVLKTHWKELGATLMCDGWTNSLNQMHIINFLVYCSKGTIFWKSVDVSSVRSRDAEFYYRLL<br>DSVVEEIGENYIVQIVTDNEAAMKAAGKKLMLKRQHLYWTSCAAHCLDLCLEDIGKKPSVAKVLDEAKKVTCFIYNHIWTV<br>DLMKKYTQGKQILRPALTRFATHFIQLEEITRQKQGLREMFNSKEFKESKWGKQKSGPAYEAKKIVLGKDFWKKANDLIKV<br>YEPLVRVLRLVDSDEKPTMGFIYEAVDRAKRAIQQNCRYFTEYEKIIDNRWNFMHSDLHSAGYFLNPQFQFGVEHSENVLI<br>ETLEGTRSVIERLEPSMDTQVRMVNQLLLFRDKHETFGTPQAQRAWKQMNPAEWWMIYGTCVPELQKLAIKVLSQTTSA<br>SNCERNWSTFSYIHTKARNRLKYKKLEKLVFTYYNMRLKMRHQQRMSTDDINASFNPISLDYIFEDVDPLSEWLHEKENP<br>LLDGENAGVLPVDTSDDEMDVDQSQQQILSHSSSSSTPSQSGDGPDGGGLSPIDEDDGYSGDRGEIRSSSQYGGEYGV<br>VPLVDIFVTDQSLMEICFLNLGEIEVNLELHQREKARSILL	zf-BED--DUF-domain--Dimer_Tnp_hAT--	I
Gbar_D03G016590.1	GbaZf-BED50_I	MVRGRDACWEHCVLVDATRQKVRCNYCHREFSGGVYRMKFHLAQIKNKDIVPCAEVPDDVRDHIQSILNTPKKQKTPKK<br>PKMDKTVANGQQNSSSASGGLHPNHGSSGQHGSTCPSLLFPHPSPSEQPATDDAQKQKLDDADKKIAVFFFHNSIPFSA<br>AKSMYYQEMVDAIAECGVGYKAPSYEKLRSSLLEKVKGDIHDCYKKYREEWKETGCTVLCNSWSDGRTKSFVIFSVTYP<br>KGTLFLKSVDVSGHEDDASYLFELLESVVLEVGLENVIQVITDSTASYVCAGRHLMAKYSSLFWSPCASYCIDKMLEDISK<br>QEWVGIVLEEAKSIARYIYSHAWILNMMRKITGGRELMRPRITRFVDNYLTLRSIVIQEDNLKHMFSHSEWLSSIYSRRSDA<br>QAIKSLLYLERFWKSAREAVSVSESLVKILRIVDGDMPAMGYMYEGIERAKGAIKAYYKGIEEKYMPIWDIIDRRWNMQLH<br>SPLHAAAAFLNPSIFYNPNFKIDLRMRNGFQEAMLKMATMDKDKIEITKEHPVYINAQGALGTDFAIMGRTLNAPGDWWA<br>SYGYEIPTLQRVAIRILSQPCSFHWCRWNWSTFETVYTKKRNKVEMEKLNDLVFVHCNLWLQTICQGRDGKCKPIIFDEID<br>VSSEWPTESEPSVPLLDDSWLDNLPLECRGSP	zf-BED--DUF-domain--Dimer_Tnp_hAT--	I
Gbar_D03G016590.2	GbaZf-BED51_I	MVRGRDACWEHCVLVDATRQKVRCNYCHREFSGGVYRMKFHLAQIKNKDIVPCAEVPDDVRDHIQSILNTPKKQKTPKK<br>PKMDKTVANGQQNSSSASGGLHPNHGSSGQHGSTCPSLLFPHPSPSEQPATDDAQKQKLDDADKKIAVFFFHNSIPFSA<br>AKSMYYQEMVDAIAECGVGYKAPSYEKLRSSLLEKVKGDIHDCYKKYREEWKETGCTVLCNSWSDGRTKSFVIFSVTYP<br>KGTLFLKSVDVSGHEDDASYLFELLESVVLEVGLENVIQVITDSTASYVCAGRHLMAKYSSLFWSPCASYCIDKMLEDISK<br>QEWVGIVLEEAKSIARYIYSHAWILNMMRKITGGRELMRPRITRFVDNYLTLRSIVIQEDNLKHMFSHSEWLSSIYSRRSDA<br>QAIKSLLYLERFWKSAREAVSVSESLVKILRIVDGDMPAMGYMYEGIERAKGAIKAYYKGIEEKYMPIWDIIDRRWNMQLH<br>SPLHAAAAFLNPSIFYNPNFKIDLRMRNGFQEAMLKMATMDKDKIEITKEHPVYINAQGALGTDFAIMGRTLNAPGDWWA<br>SYGYEIPTLQRVAIRILSQPCSFHWCRWNWSTFETVYTKKRNKVEMEKLNDLVFVHCNLWLQTICQGRDGKCKPIIFDEID<br>VSSEWPTESEPSVPLLDDSWLDNLPLECRGSP	zf-BED--DUF-domain--Dimer_Tnp_hAT--	I
Gbar_D04G004060.1	GbaZf-BED52_I	MDMSDAVIVNSSRLKSIVWNDFDRVKKGDTFVAICRHCKKKLSGSSTSGTSHLRNHLIRCQRRSNHGVAQYFSAKDKKK<br>EGSLALVTIDQEQKNDEVLSIVNLRYEQEQIKSEHVAIGSNSLDQRRSQFDLARMIILHNYPLAMVEHVGFKIFVRNLQPLF<br>ELVTRNKVEADCMEIYAKEKQKVYEIFDKLPGKISVSADVWTASEDDAAYLSLAAHYIDENWQLKKKNLNFVTIDPSYAED<br>MHSEVIMNCLMDWDIDRKLFSMIFDSFTSDNIVERIRDRLSQNRFLHCNGQLFDVRCAVDLLNRMAHDALETLCEITQKIR<br>ESIRYVKSSEATQATFNELADEVQVETKKCLCIDNPLKWNSTYLMLEAASEYRKVFSCLRDRDPVNMKFLLSDPEWDRLIT<br>VTSFLKLFVEVTNVFTRSKYPTANIFFPEICDIHLQLIEWCKNPDEYISSLALKMRKKFEEYWYKCSSGLAVAAMLDPRFKM<br>KLLEYYYPQLYGDSATELIDDVFECIKSLYNEHSMVSPLASSIDQGLDWQASGIPGSGKDSRDRLMGFDKFLHETSQAEG<br>SSSDLDKYLEEPLFPRNVDFNVLNWWKVHTPRYPILSMMARNILGIPISKVAAESRFDTGGRMLNHNWSSLPPTTIQALM<br>CSRDWIRSELES	zf-BED--DUF-domain--Dimer_Tnp_hAT--	I
Gbar_D04G004060.3	GbaZf-BED54_I	MDMSDAVIVNSSRLKSIVWNDFDRVKKGDTFVAICRHCKKKLSGSSTSGTSHLRNHLIRCQRRSNHGVAQYFSAKDKKK<br>EGSLALVTIDQEQKNDEVLSIVNLRYEQEQIKSEHVAIGSNSLDQRRSQFDLARMIILHNYPLAMVEHVGFKIFVRNLQPLF<br>ELVTRNKVEADCMEIYAKEKQKVYEIFDKLPGKISVSADVWTASEDDAAYLSLAAHYIDENWQLKKKNLNFVTIDPSYAED<br>MHSEVIMNCLMDWDIDRKLFSMIFDSFTSDNIVERIRDRLSQNRFLHCNGQLFDVRCAVDLLNRMAHDALETLCEITQKIR<br>ESIRYVKSSEATQATFNELADEVQVETKKCLCIDNPLKWNSTYLMLEAASEYRKVFSCLRDRDPVNMKFLLSDPEWDRLIT<br>VTSFLKLFVEVTNVFTRSKYPTANIFFPEICDIHLQLIEWCKNPDEYISSLALKMRKKFEEYWYKCSSGLAVAAMLDPRFKM<br>KLLEYYYPQLYGDSATELIDDVFECIKSLYNEHSMVSPLASSIDQGLDWQASGIPGSGKDSRDRLMGFDKFLHETSQAEG<br>SSSDLDKYLEEPLFPRNVDFNVLNWWKVHTPRYPILSMMARNILGIPISKVAAESRFDTGGRMLNHNWSSLPPTTIQALM<br>CSRDWIRSELES	zf-BED--DUF-domain--Dimer_Tnp_hAT--	I
Gbar_D04G004060.4	GbaZf-BED55_I	MDMSDAVIVNSSRLKSIVWNDFDRVKKGDTFVAICRHCKKKLSGSSTSGTSHLRNHLIRCQRRSNHGVAQYFSAKDKKK<br>EGSLALVTIDQEQKNDEVLSIVNLRYEQEQIKSEHVAIGSNSLDQRRSQFDLARMIILHNYPLAMVEHVGFKIFVRNLQPLF<br>ELVTRNKVEADCMEIYAKEKQKVYEIFDKLPGKISVSADVWTASEDDAAYLSLAAHYIDENWQLKKKNLNFVTIDPSYAED<br>MHSEVIMNCLMDWDIDRKLFSMIFDSFTSDNIVERIRDRLSQNRFLHCNGQLFDVRCAVDLLNRMAHDALETLCEITQKIR<br>ESIRYVKSSEATQATFNELADEVQVETKKCLCIDNPLKWNSTYLMLEAASEYRKVFSCLRDRDPVNMKFLLSDPEWDRLIT<br>VTSFLKLFVEVTNVFTRSKYPTANIFFPEICDIHLQLIEWCKNPDEYISSLALKMRKKFEEYWYKCSSGLAVAAMLDPRFKM<br>KLLEYYYPQLYGDSATELIDDVFECIKSLYNEHSMVSPLASSIDQGLDWQASGIPGSGKDSRDRLMGFDKFLHETSQAEG<br>SSSDLDKYLEEPLFPRNVDFNVLNWWKVHTPRYPILSMMARNILGIPISKVAAESRFDTGGRMLNHNWSSLPPTTIQALM<br>CSRDWIRSELES	zf-BED--DUF-domain--Dimer_Tnp_hAT--	I
Gbar_D04G004060.5	GbaZf-BED56_I	MDMSDAVIVNSSRLKSIVWNDFDRVKKGDTFVAICRHCKKKLSGSSTSGTSHLRNHLIRCQRRSNHGVAQYFSAKDKKK<br>EGSLALVTIDQEQKNDEVLSIVNLRYEQEQIKSEHVAIGSNSLDQRRSQFDLARMIILHNYPLAMVEHVGFKIFVRNLQPLF<br>ELVTRNKVEADCMEIYAKEKQKVYEIFDKLPGKISVSADVWTASEDDAAYLSLAAHYIDENWQLKKKNLNFVTIDPSYAED<br>MHSEVIMNCLMDWDIDRKLFSMIFDSFTSDNIVERIRDRLSQNRFLHCNGQLFDVRCAVDLLNRMAHDALETLCEITQKIR<br>ESIRYVKSSEATQATFNELADEVQVETKKCLCIDNPLKWNSTYLMLEAASEYRKVFSCLRDRDPVNMKFLLSDPEWDRLIT<br>VTSFLKLFVEVTNVFTRSKYPTANIFFPEICDIHLQLIEWCKNPDEYISSLALKMRKKFEEYWYKCSSGLAVAAMLDPRFKM<br>KLLEYYYPQLYGDSATELIDDVFECIKSLYNEHSMVSPLASSIDQGLDWQASGIPGSGKDSRDRLMGFDKFLHETSQAEG<br>SSSDLDKYLEEPLFPRNVDFNVLNWWKVHTPRYPILSMMARNILGIPISKVAAESRFDTGGRMLNHNWSSLPPTTIQALM<br>CSRDWIRSELES	zf-BED--DUF-domain--Dimer_Tnp_hAT--	I
Gbar_D05G011800.1	GbaZf-BED57_I	MASSEVFINVRDHGKTVDVKKKRIKCNYCDKEMSGFSRLKYHLGGVRGNVLPCEKVPQDVKKLFRDMVQGREHLHNDA<br>PYLYRQPFPQKRNGCPHNNVAKKTRHQSSESSGDESREYGNTDSMSEDDLEDSVASCKRMVSQKSGKQNKRCIGRFF<br>FETGTDFKLVNSLSFQRLMNDIRGWILKDEVKEIQEYVQKIRQSWGNTGCRIFFIADCPEGPIYLHSCDVSASVDDVNTLQ<br>LLLDRVMYEVGAENVVQVIAFSTTGWVGDVGKQFMERWKSVFWTVNASHCIELLLDEVVNMGDVQRTLEKAKTISKFIHD<br>HVTVLNLWRDYMDGHDLIKPTKIKSAVPFVTLENIISEKRNITAMFTSSAWNNTTWSSTVEGKRVAKLVGDASFWRGAGM<br>VVKLTLPLIRVLCLMHGEDKPQMGYIYETIDQVKETIEGCNSRKSEYMPFWKAIDEIWDGHLHSPLHAAGYFFNPSFFYST<br>DFQSDFEVGFGLLCCMVPMWWSPYGGEYPELQRFATRILSQTCVGASKYRLNRSLAEKLLTKGRDRTEEQLLSDLTFVH<br>YNLQLQQQHSQLGVNYDIVADEIGPMNEWIVDDTAEIGSDNGDSNWKDLKSAVNGEGPSMYTSNFKRYYLFKMKLSKK<br>M	zf-BED--DUF-domain--Dimer_Tnp_hAT--	I
Gbar_D06G0	GbaZf-	MTEMTEITDMETIPGESNNQLALTTPEPQPIKRRKKKSMVWEYFTIENVSAGCRRAYCKRCKQSFAYSTGSKVAGTSHLK<br>RHIAKGTCRALLRGQGQDNNQFITPYNPKMGGSEPPKRRYRSPSSPFIPFDQDRCRHEIARMIIMHEYPLHIVEHPGFIAF<br>VQNLQPQFDKMSFNTVQGDCVATYLREKQSLMNFIEGIPGRFCLTLDMWSSNQTLGYVFITGHFVDSDWKLHRRVFNVV	zf-BED--DUF-domain--	I

05660. 1	BED 58_I	MEPYPDSHSALSHAIAACLSDWSLEGKLFSLTFNHPLSEAGLENLRPLLCVKNPLILNGQLLIRNCIARTMSSMAKDVLGAGQEIIKKIRDSVKYVKMSESHDDKFIQVKNQLQVPSEKSLFLDNQTQWNTTYQMLAAASELKEVFDCLDTYDPDYKLAPSMEDWKLAETLCSFLKPLFDAASILTTTTLPTVITFFYEVWKIHVDLGRSITCEDPFISNLAKSMQEKIDKYWKDCSLVLAMAVVMDPRFKMKLVEFSFTKIYGEDAPTYIKTVDDGIHELFLEYVALPLPLTPTYAEEVNGANNGKTNESHQGNLLSDHGLADFDVYIMETNSQQMKSELDQYLEESLLPRVQEFDVLGWWKLNKMKYPTLSKMARDILSIPVSAAATESIFDITDKQLDEYRSSLRPETVEALICAKDWLHYGSSDVSNALVRMEF	Dimer_Tnp_hAT --	
Gbar_ D06G0 05660. 3	Gba Zf- BED 60_I	MTEMTEITDMETIPGESNNQLALTTPEPQPIKRRKKKSMVWEYFTIENVSAGCRRAYCKRCKQSFAYSTGSKVAGTSHLKRHIAKGTCRALLRGQGQDNNQFITPYNPKMGGSEPPKRRYRSPSSPFIPFDQDRCRHEIARMIIMHEYPLHIVEHPGFIAFVQNLQPQFDKMSFNTVQGDCVATYLREKQSLMNFIEGIPGRFCLTLDMWSSNQTLGYVFITGHFVDSDWKLHRRVFNVVMEPYPDSHSALSHAIAACLSDWSLEGKLFSLTFNHPLSEAGLENLRPLLCVKNPLILNGQLLIRNCIARTMSSMAKDVLGAGQEIIKKIRDSVKYVKMSESHDDKFIQVKNQLQVPSEKSLFLDNQTQWNTTYQMLAAASELKEVFDCLDTYDPDYKLAPSMEDWKLAETLCSFLKPLFDAASILTTTTLPTVITFFYEVWKIHVDLGRSITCEDPFISNLAKSMQEKIDKYWKDCSLVLAMAVVMDPRFKMKLVEFSFTKIYGEDAPTYIKTVDDGIHELFLEYVALPLPLTPTYAEEVNGANNGKTNESHQGNLLSDHGLADFDVYIMETNSQQMKSELDQYLEESLLPRVQEFDVLGWWKLNKMKYPTLSKMARDILSIPVSAAATESIFDITDKQLDEYRSSLRPETVEALICAKDWLHYGSSDVSNALVRMEF	zf-BED--DUF-domain--Dimer_Tnp_hAT --	I
Gbar_ D06G0 05670. 1	Gba Zf- BED 61_I	MTEMTEITDMETIPGESNNQLALTTPEAQPIKRRKKKSMVWEYFTIENVSAGCRRAYCKRCKQSFAYSTGSKVAGTSHLKRHIAKGTCRALLRGQGQDNNQFITPYNPKMGGSEPPKRRYRSPSSPFIPFDQDRCRHEIARMIIMHEYPLHIVEHPGFIAFVQNLQPQFDKMSFNTVQGDCVATYLREKQSLMNFIEGIPGRFCLTLDMWSSNQTLGYVFITGHFVDSDWKLHRRVFNVVMEPYPDSHSALSHAIAACLSDWSLEGKLFSLTFNHPLSEAGLENLRPLLCVKNPLILNGQLLIRNCIARTMSSMAKDVLGAGQEIIKKIRDSVKYVKMSESHDDKFIQVKNQLQVPSEKSLFLDNQTQWNTTYQMLAAASELKEVFDCLDTYDPDYKLAPSMEDWKLAETLCSFLKPLFDAASILTTTTLPTVITFFYEVWKIHVDLGRSITCEDPFISNLAKSMQEKIDKYWKDCSLVLAMAVVMDPRFKMKLVEFSFTKIYGEDAPTYIKTVDDGIHELFLEYVALPLPLTPTYAEEVNGANNGKTNESHQGNLLSDHGLADFDVYIMETNSQQMKSELDQYLEESLLPRVQEFDVLGWWKLNKMKYPTLSKMARDILSIPVSAAATESIFDITDKQLDEYRSSLRPETVEALICAKDWLHYGSSDVSNALVRMEF	zf-BED--DUF-domain--Dimer_Tnp_hAT --	I
Gbar_ D06G0 19130. 1	Gba Zf- BED 62_I	MTMASSNTPIPVDDGFNEYESVVKRQKSTTSKVWDEMTKLECENKNELKAQCNHCKTIFSAKSSSETSHLRRHLNSCLKKVNKDIAQYIIATQPSPEGVPSIKIYKFDADECRQAISTFLVCGKHSFGTVEEPGFRYMMRIASPNFKNISRQTAIRDVLKYYAKERDHVKEELAKAPGLICLTSDNWNSEHTNDEYICITAHWVDKDWKLQKRIIRFRALFPPYDGLNIADELVLCLSQWGIDKKIFSITLDNASYNDVMVSCLKNCFRANQAILCDGVFFQVRCCAHILNLIVKAGLELADYVVCKIRNGIRYIKKSGIRRKRFYDVADKSFHLNVTKKLRQDVCVRWNSTYLMLESFLYYKDVLDYWGQRDKDYQMFALSSEEWRNVAILCKFLKVFYDVTCVFSGSNYPTANLYFRGVWKVYKLLIDIVKGPYSFLTPMVKQMQEKFNKYWAEYSLILSCAAILDPRYKLNYVQYCFKTIYGVHASDFVETILSNLRLLFDEYVKKSKSMSSSLAGSSNVSDKNPVDSGLDEHNDNSADFGGYFDESDDYKRYLNESSTRSEKSQLNIYLEESELELNSQIDVLDYWSKSSVRYNELSLLARDLLAIPISTVAFESAFSMGKKVITLIRSSLKPKTVQAVVCLDDWMRAKGFSAGNYYSHFIVVILYYFFINLII	zf-BED--DUF-domain--Dimer_Tnp_hAT --	I
Gbar_ D07G0 09120. 1	Gba Zf- BED 63_I	MELNLVPISITRQKQDPAWNHCEVFKNGERLQIKCMYCGKLFKGGGIHRFKEHLAGRKGQGPICEQVPQGVRSVMQESLNGILVKQDKKQKLIPKLLACGSSSSNPNIGGEVENLGSHDDMNFGIKPISVLNTLEGDSNVVSKVGRGRKRGRGRDWNLIESNYPCVKTDLALVPNGGDNPTHMAIGRFLYDIGVNLDVVNSVCFQPMIDAIASGGSGVVPPSCHDLRGWILKNVIEEVKDDIDRNKAMWGKTGCSIIVEQCRTKNGRVLLSFLVYCPQATVFMKSVDASHAFYSADYLFELLKQVIEEVGSEKVVQVITNCEEPYLFTGKRLMESFPSLYWAPCLAHCVDLMLQDFSNLEWINKTIEQAKSLTRFIYNQSLVLNMMRKFTSGNDVVEPALTCFATNFSTLKRMADLKLNLQAMVNSQDWLECPYAKKPGGQAMSDIVNNRSFWNSCMLIARITYPLLRVLEIVGSKKRSAMGYVYAGIYRAKETIKKELVKQDDYMVYWNIIDNRWEQQRHLPLYAAGFFLNPKLFYNTEEHIHNDFLSSVFDSIERLVPDTNIQDQVVREINLYKSATGDLGRPMAVRARDNLLPGEWWSIYGGGCPNLQRLAIRILSQTCSSIGYKPSKISIEEIHNTRNFLERQRLSDLVFVQYNLYLRQMVLQKQEKDSLDPLAFNNKDILEDWIADNEVSPDNLESSDWKSLDPPVGNRTTLTPPGDEAEDFLSTRFTDLDIFNGLKGVKEEI	zf-BED--DUF-domain--Dimer_Tnp_hAT --	I
Gbar_ D08G0 08570. 1	Gba Zf- BED 64_I I	MSTEPTSIEGNVTPPTSIDSENLGVGASSQTKGTTWNRKATPQKSKVWSHFTKIIISEDASKVKCNYCQKEFCCDMKRNVIGSLKYHIGSCKKNPSNVVDTSQGQLVLPRKGRFDQEACRKGLAQMIVIDELPFKFVESEGFKKFIFVAYPRFHIPSRTNMIRDVYQLYLDERVKIKQLLRSSCSRICLTIDTWTSLQRVNYLCITAHFIDNDWKLNKKILNFFPISSHKGESIGMVIEKCLLNWGIDKLFTITVHNASSNDVAIGYLRKKFNPRGGLVQNGKYLHMRCMAYILNLIVVEGLKEMNKSVERIRGAVRYVRQSPARLQKFKECVVAEKIECKKMLCLDVCTRWNLTYLMLNTAQNFERAFEIFEKQDTNFRAELERGEGWPRVDDWDNVRNLRDFLEHFYEVTLRIFGTSYVTSNNFFDDLSEIDILF	zf-BED	II
Gbar_ D08G0 10130. 1	Gba Zf- BED 65_I	MAEITEATNMETTPVENNNELALITPETQPKRRKKKSMVWEYFTIETVSAGCRRACCNRCKQSFAYSTGSKVAGTSHLKRHIAKGTCPALLRDQYNNQLTPYNPKTGGSEPRKRRYRSPSSPFIPFDQDRCRHEIARMIIMHEYPLHMVEHPGFIAFVQNLQPRFDKVSFNTVQGDCVATYLREKQSLMKLIEGIPGRVCLTLDMWTSNQTLGYVFITGHFIDFEWKLQSRVLNVIMEPYPDSDSALSHAVAACLSDWSLEGKLFSLTFNHPTSEAGLENLRPLLCTKNPLILNGQLLLGNCIARTLSSMAKDVLGAEHEVVKKIRDSVKYVKTSESHDEKFVQVKNQLQVPSEKSLILDNQTQWNTTYQMLAAGSELKEVFNCLDTSDPDYKLAPSIEDWKVAETLCTFLKPLFDAASILTTTTNPTAITFFHEAWKIHADLGRSITNEDPFISNIAKSMLEKIDKYWKDCSLILAIAVVMDPRFKMKLVEFSFTKIFGEDAPTYIKIVDDGIHELFLEYVALPLPLTPTYTEEGNAGNNGKTDESQQGNLLSDQGLTDFDVYIMETSSQQMKSELDQYLEESLLPRVQEFDVLGWWKLNKMKYPTLSKMARDILSIPVSAAAPDSVFDIIIKQLDEYRSSLRPETVEALICAKDWLHYGSEESNALVKMEF	zf-BED--DUF-domain--Dimer_Tnp_hAT --	I
Gbar_ D08G0 26120. 1	Gba Zf- BED 66_I	MEVANETVIKKPKRLTSVVWNHFERVRKADLCYAVCVHCNKKLSGSSNSGTTHLRNHLMRCLKRFNYDVSQLLSAKKRKKENTLTIANISYDEGQRKEEYLKPTIVKYEPEQRKDEVFNVQSSWFDQDRSRLDLARMIILHGYPLAMVEHVGFKVFVKNLQPLFDVVPNSTVELSCMEIYGKERQKVHDMLSKLQGRINLAVEMWSSPENTNHVCMMAHYIGDDWKLQKKILNFVTLDSSHTDDLLSGVIIKCLMDWDIGSKLFAVTLDDFSTNDDIVLRIKEQILENKSRLSNGQLLDVRSAAHVLNSIVQDAMEALRVVLQKIRGTVRYVKSSQSIQGKFKEMVLQTGINSQKNLVLDCPIRWNSTYLMLETAIEYRNAFCQLPDLDLDLALSDEEWEWASSITGYLKLFVEIINVFSSNKCPTANIYFPEICHVHIQLIDWCKSPDNFLSSLAAKMKAKFDKYWSKCSLSLAVAAILDPRFKMKLVEYYYSQIYGSTALERIKEVSDGLKELFSTYSICSTLMDQGSALPLGSLPSSSNDGRDRLKGFDKFLHETSQSQTAISDLEKYLDEPVFPHNCNFNILNWWRVHTPRYPILSMMARDVLGTPMSTVSQESAFHAGGRVLDSCRCPLTPETRQALICTQDWLRIQSDDPGPSSSHYALPLYVETN	zf-BED--DUF-domain--Dimer_Tnp_hAT --	I
Gbar_ D08G0 26120. 2	Gba Zf- BED 67_I	MEVANETVIKKPKRLTSVVWNHFERVRKADLCYAVCVHCNKKLSGSSNSGTTHLRNHLMRCLKRFNYDVSQLLSAKKRKKENTLTIANISYDEGQRKEEYLKPTIVKYEPEQRKDEVFNVQSSWFDQDRSRLDLARMIILHGYPLAMVEHVGFKVFVKNLQPLFDVVPNSTVELSCMEIYGKERQKVHDMLSKLQGRINLAVEMWSSPENTNHVCMMAHYIGDDWKLQKKILNFVTLDSSHTDDLLSGVIIKCLMDWDIGSKLFAVTLDDFSTNDDIVLRIKEQILENKSRLSNGQLLDVRSAAHVLNSIVQDAMEALRVVLQKIRGTVRYVKSSQSIQGKFKEMVLQTGINSQKNLVLDCPIRWNSTYLMLETAIEYRNAFCQLPDLDLDLALSDEEWEWASSITGYLKLFVEIINVFSSNKCPTANIYFPEICHVHIQLIDWCKSPDNFLSSLAAKMKAKFDKYWSKCSLSLAVAAILDPRFKMKLVEYYYSQIYGSTALERIKEVSDGLKELFSTYSICSTLMDQGSALPLGSLPSSSNDGRDRLKGFDKFLHETSQSQTAISDLEKYLDEPVFPHNCNFNILNWWRVHTPRYPILSMMARDVLGTPMSTVSQESAFHAGGRVLDSCRCPLTPETRQALICTQDWLRIQSDDPGPSSSHYALPLYVETN	zf-BED--DUF-domain--Dimer_Tnp_hAT --	I
Gbar_ D08G0 26120. 3	Gba Zf- BED 68_I	MEVANETVIKKPKRLTSVVWNHFERVRKADLCYAVCVHCNKKLSGSSNSGTTHLRNHLMRCLKRFNYDVSQLLSAKKRKKENTLTIANISYDEGQRKEEYLKPTIVKYEPEQRKDEVFNVQSSWFDQDRSRLDLARMIILHGYPLAMVEHVGFKVFVKNLQPLFDVVPNSTVELSCMEIYGKERQKVHDMLSKLQGRINLAVEMWSSPENTNHVCMMAHYIGDDWKLQKKILNFVTLDSSHTDDLLSGVIIKCLMDWDIGSKLFAVTLDDFSTNDDIVLRIKEQILENKSRLSNGQLLDVRSAAHVLNSIVQDAMEALRVVLQKIRGTVRYVKSSQSIQGKFKEMVLQTGINSQKNLVLDCPIRWNSTYLMLETAIEYRNAFCQLPDLDLDLALSDEEWEWASSITGYLKLFVEIINVFSSNKCPTANIYFPEICHVHIQLIDWCKSPDNFLSSLAAKMKAKFDKYWSKCSLSLAVAAILDPRFKMKLVEYYYSQIYGSTALERIKEVSDGLKELFSTYSICSTLMDQGSALPLGSLPSSSNDGRDRLKGFDKFLHETSQSQTAISDLEKYLDEPVFPHNCNFNILNWWRVHTPRYPILSMMARDVLGTPMSTVSQESAFHAGGRVLDSCRCPLTPETRQALICTQDWLRIQSDDPGPSSSHYALPLYVETN	zf-BED--DUF-domain--Dimer_Tnp_hAT --	I
Gbar_ D09G0 19140. 1	Gba Zf- BED 69_I	MVRERDVCWEYAEKLDGNKVRCKFCLRVLNGGISRLKHHLSRLPSKGVNPCNKVRDDVTDRVRAIISSKEDIKETPSVKKQKIAEVRAPGNMSTSSKISPLETLSPAAKVFPTVLSIAASTLSDQETVERSIALFFFENKLDFSVARSSSYQAMIDAVGKFGPGLIAPSVETLKTTWLKRIKSEVTLHLKDAEKEWATTGCTIIADTWTDNKSKALINFLVSSPSRTFFHKSVDASSYFKNTKCLADLFDSVIQDFGQENVVQIIMDSSFNYTGISSHILQNYGTIFLSPCASQCLNLILEEFSRVDWVNRCILQAQTVSKFLYNNASMLDLMKKFTGGQELIRTGITKSVSCFLSLQSMLKQRSRLKHMFNSPEYSTNSSYANKPQSISCIAIVEDNDFWRAVEECVAISEPFLKVLREVSGGKPVVGSIYELMTRAKESIRTYYIMDESKCKTFLDIVDRQWRDQLHSPLHSAGAFLNPSIQYNPEVKFLGSIKEDFFKVLEKLLPTPELRRDITNQIFTFTRAKGMFACNLAMEARDTVSPGLWWEQFGDSAPVLQRVAIRILSQVCST	zf-BED--DUF-domain--Dimer_Tnp_hAT --	I

		FMFERHWSTFQQIHTEKRNKIDKETLTDVVYINYNLKLAREMKTMPTESDPIQFDDIDMTSEWVEESENPSPTQWLDRFG<br>SALDGGDLNTRQFSAAMFGNDHIFGL		
Gbar_D09G019140.2	GbaZf-BED70_I	MIMYCSIFLAVVRERDVCWEYAEKLDGNKVRCKFCLRVLNGGISRLKHHLSRLPSKGVNPCNKVRDDVTDRVRAIISSKE<br>DIKETPSVKKQKIAEVRAPGNMSTSSKISPLETLSPAAKVFPTVLSIAASTLSDQETVERSIALFFFENKLDFSVARSSSYQA<br>MIDAVGKFGPGLIAPSVETLKTTWLKRIKSEVTLHLKDAEKEWATTGCTIIADTWTDNKSKALINFLVSSPSRTFFHKSVDA<br>SSYFKNTKCLADLFDSVIQDFGQENVVQIIMDSSFNYTGISSHILQNYGTIFLSPCASQCLNLILEEFSRVDWVNRCILQAQT<br>VSKFLYNNASMLDLMKKFTGGQELIRTGITKSVSCFLSLQSMLKQRSRLKHMFNSPEYSTNSSYANKPQSISCIAIVEDND<br>FWRAVEECVAISEPFLKVLREVSGGKPVVGSIYELMTRAKESIRTYYIMDESKCKTFLDIVDRQWRDQLHSPLHSAGAFLN<br>PSIQYNPEVKFLGSIKEDFFKVLEKLLPTPELRRDITNQIFTFTRAKGMFACNLAMEARDTVSPGLWWEQFGDSAPVLQRV<br>AIRILSQVCSTFMFERHWSTFQQIHTEKRNKIDKETLTDVVYINYNLKLAREMKTMPTESDPIQFDDIDMTSEWVEESENPS<br>PTQWLDRFGSALDGGDLNTRQFSAAMFGNDHIFGL	zf-BED--DUF-domain--Dimer_Tnp_hAT--	I
Gbar_D09G019140.3	GbaZf-BED71_I	MVRERDVCWEYAEKLDGNKVRCKFCLRVLNGGISRLKHHLSRLPSKGVNPCNKVRDDVTDRVRAIISSKEDIKETPSVKK<br>QKIAEVRAPGNMSTSSKISPLETLSPAAKVFPTVLSIAASTLSDQETVERSIALFFFENKLDFSVARSSSYQAMIDAVGKFG<br>PGLIAPSVETLKTTWLKRIKSEVTLHLKDAEKEWATTGCTIIADTWTDNKSKALINFLVSSPSRTFFHKSVDASSYFKNTKCL<br>ADLFDSVIQDFGQENVVQIIMDSSFNYTGISSHILQNYGTIFLSPCASQCLNLILEEFSRVDWVNRCILQAQTVSKFLYNNAS<br>MLDLMKKFTGGQELIRTGITKSVSCFLSLQSMLKQRSRLKHMFNSPEYSTNSSYANKPQSISCIAIVEDNDFWRAVEECVA<br>ISEPFLKVLREVSGGKPVVGSIYELMTRAKESIRTYYIMDESKCKTFLDIVDRQWRDQLHSPLHSAGAFLNPSIQYNPEVKF<br>LGSIKEDFFKVLEKLLPTPELRRDITNQIFTFTRAKGMFACNLAMEARDTVSPGLWWEQFGDSAPVLQRVAIRILSQVCST<br>FMFERHWSTFQQIHTEKRNKIDKETLTDVVYINYNLKLAREMKTMPTESDPIQFDDIDMTSEWVEESENPSPTQWLDRFG<br>SALDGGDLNTRQFSAAMFGNDHIFGL	zf-BED--DUF-domain--Dimer_Tnp_hAT--	I
Gbar_D11G000680.1	GbaZf-BED72_I	MEWSVNNAFKSYKDMEPKSTMDMVLIPNMDTIDIVLGSSEKGNVVPSAKPRKKTMTSVYLKYFETAPDGKTRRCKFCGQ<br>SYSIATATGNLGRHLSNRHPGYDKTGENVTSSAPQPSTTPTVIKKPQQQGRAPQVDYDHLNWLLIKWLILATLPPSTLEEK<br>WLANSFKFFNPSIQLWPGEKYKAVFREVFRSMREDVRASLEQVSSKVSIALDFWTSYEQIFYMSITCQWIDENWSFRKVL<br>LDICQVPYPCSDSEIYNSLVKVLKMYNIENKVLSCTHDNSQNAIHACHALKEDLDGQKMGPFCFIPCAARTLSLIIDDALRTT<br>KPVIAKVREFVQELNASLDISEDFIQLTTAYKEGSWQFPLDASARWSGSYQMLDLVQKAGKSMDAIVRKNEEMLGNRMLL<br>NTAEKNVVNIVHNYLEPFYKVISEICVNTPPTIGMVIVYMDHISDTITTRQPPDWLKNPAEDMAKKLRSYNNQVCNIFIYMTA<br>ILDPRIKCELIPESLNSENYLEEARAHFVRNYYTTPFSSMTSGYSSQDIEDGGAVSFAEEIARKKRRASMSNATDELTQYLS<br>ESPAPTKTDVLEWWKVNSTRYPRLSAMARDFLAVQATSVKPDELFCSKGDEIDKQRFCMPHDSTQAILCIKSWTQGGLK<br>LKYKSTEIDYERLMEMAAAAAADISSAGIDKKQK	zf-BED--DUF-domain--Dimer_Tnp_hAT--	I
Gbar_D11G027730.1	GbaZf-BED73_XXII	MSIEPTSIEGRVTPPTSIDSENSGVGASIQTKGTTGKRKAPPQRSAVWSHFTKFINSEGASKAKCNYCEKEFCCDMKKNG<br>TGSLKYHIGSCKKNPSNVVDTSQGQLVLPRKGVEGGEGNLSTWRFDQEACRKGLAQMILIDELLFKFVESEGFKKFMFVA<br>CPRFHIPPRTIMTRDVYQLYLDERVKIKKLLRSSCSRVCLTTDTWTSLQRVNYLCITAHFIDNDWKLNKKILNFCPISSHKGE<br>SIGMVIEKCLLNWGIDKLFTVTVDNASSNDVAIGYLRKKFNPRGGLVQNGKYLHMRCMAHIVNLIVVEGLKEMNKSVERVR<br>GAVRYVRQSPARLQKFKECVVVERIECKKMLCLDVCTRWNLTYLMLDTAQNFERALKRFEEQDTNFRAELERGEGWPS<br>VDDWDNVRKLRDFLEHFYEVTLRISGTSYLNSNIDFNVMAIKMKEKYDKYWGDIDKMNLLMFVACVLDPRQKLKYLEFAL<br>SEMSSSEKACEMMQKLKESLYELFDEYKPPLHSTCSQSSSSRELEICGEDKTSELDKYLAEANEEFVEDFDILLWWKVNS<br>PRFPTLSKIARDVLAIPVSTVASEFAFNTGGRVLDQYRSSLTPKIVQALVCTQDWIRRSSSQEDIKKIKEQIQELDKIENGIQ<br>YMFVIHFMTYLGIINSWGDWTIIGWTITNPSIWCYEGVARAHIVFFGLCFLAAMWH	zf-BED--DUF-domain--Dimer_Tnp_hAT--PSII--	XXII
Gbar_D11G031860.1	GbaZf-BED74_I	MEVANESTAKKPKRLTSVVWNHFERVKKADICYAVCVHCNKKLSGSSNSGTTHLRNHLMRCLKRSNYDVSQLLAVKRRK<br>KENTLTIANISYDEGQRKEDYMKPTIVKYEQDQRKDEAFNLGSSWFDPERSRLDLARMIILHGYPLAMVEHVGFKVFVKN<br>MQPLFDVVHNSTIELSCVEIYMKEKQRIYDMLSKLQGRINLAIEMWSSPENSKYVCLTAHYVDDEWKLQKKILNFLTLDSS<br>HTEDMLSDVIIKCLMDWDIDCKLFSMTFDDCSTNDDIVLRIKDQISESRPRLSNGQLLDVRSAAHVLNSIAQDAIEALQVVIQ<br>KIRGSVKYVKSSQSILGKFNEIAQQQGINNHKIVVLDYPIRWNSTYMMLETAVEYRNVFHHLPELDPDFALSDEEWKRASS<br>IVSYLKLLIEIINVFSSNKCPTANIYFPEICHVHIQLIEWCKSSDAFLSSLATKMKAKFDKYWSKCSLGLAVAAILDPRFKMKLV<br>EYYYSQIYGSTALERIKEASDGIKELFNAYSICSTLIDQGSALPGSSLPSSSNDTRDKLKGFDKFLHETSQSQTAISDLEKYL<br>DEPMFPRNCDFNILNWWRVHTPRYPILSMMARDVLGTPMSTVAQEFAFNAGGRILDSNQSSLPPDTRQALICTRDWLRT<br>QSDDATTSSSHYALPLYVEAN	zf-BED--DUF-domain--Dimer_Tnp_hAT--	I
Gbar_D11G031860.2	GbaZf-BED75_I	MEVANESTAKKPKRLTSVVWNHFERVKKADICYAVCVHCNKKLSGSSNSGTTHLRNHLMRCLKRSNYDVSQLLAVKRRK<br>KENTLTIANISYDEGQRKEDYMKPTIVKYEQDQRKDEAFNLGSSWFDPERSRLDLARMIILHGYPLAMVEHVGFKVFVKN<br>MQPLFDVVHNSTIELSCVEIYMKEKQRIYDMLSKLQGRINLAIEMWSSPENSKYVCLTAHYVDDEWKLQKKILNFLTLDSS<br>HTEDMLSDVIIKCLMDWDIDCKLFSMTFDDCSTNDDIVLRIKDQISESRPRLSNGQLLDVRSAAHVLNSIAQDAIEALQVVIQ<br>KIRGSVKYVKSSQSILGKFNEIAQQQGINNHKIVVLDYPIRWNSTYMMLETAVEYRNVFHHLPELDPDFALSDEEWKRASS<br>IVSYLKLLIEIINVFSSNKCPTANIYFPEICHVHIQLIEWCKSSDAFLSSLATKMKAKFDKYWSKCSLGLAVAAILDPRFKMKLV<br>EYYYSQIYGSTALERIKEASDGIKELFNAYSICSTLIDQGSALPGSSLPSSSNDTRDKLKGFDKFLHETSQSQTAISDLEKYL<br>DEPMFPRNCDFNILNWWRVHTPRYPILSMMARDVLGTPMSTVAQEFAFNAGGRILDSNQSSLPPDTRQALICTRDWLRT<br>QSDDATTSSSHYALPLYVEAN	zf-BED--DUF-domain--Dimer_Tnp_hAT--	I
Gbar_D12G013130.1	GbaZf-BED76_III	MVEEMAPLRSIGYVDPGWEHGTAQDERKKKVKCNYCGKVVSGGIFRLKQHLARLSGEVTHCEKVPEEVCLNMRKNLEG<br>CRSGRKRRQLDYEQAALSIQSNEYSDGEDASASYKHKGKKVMGDKNLVIKFTPLRSLGYVDPGWEHCVAQDEKKRRVK<br>CNYCEKIISGGINRFKQHLARIPGEVAYCEKAPEEVYLKIKENMKWHRTGRRHRKPDTKEISTFYMHSDNEDEGGEEEGY<br>LQCISKDILAIDDKVSDNDIRNNVRGRSPGSSGNGAEPLLKKSRLDSVFLKSLKSQTSTHHKQPRARTGFEKKTHREVISAI<br>CKFFYHAGIPSNAANSPYFHKMLELVGQYGQGLQGPSSRLISGRLLQEEIANIKEYLVELKTSWAITGCSVMADSWNDAQ<br>GRMLINFLVSCPRGVYFLSSVDATDIIEDAVHLFKLLDKAVDEVGEEYVVQVITRNTLSFRNAGKMLEEKRRNLFWTPCAV<br>YCIDRMLEDFVNIKWVGECVDKAKKVTRFIYNNTWLLNFMKKEFTKEQELLQPAVTKFGTNFFTLQSLLDQRVGLKRMFQ<br>SNRWLSSRFSKSDEGKEVEKIVLNVSFWKKMQYVKKSFEPVAEVLQRIGSDKIRSLPFIYNDICRTKLAIKAIHGDDVRKYG<br>PFWSVIESNWSPLFHHPLYVAAYFLNPSYRYRPDFLMNPEVIRGLNGCIVRLEADNGKKIAASMQIPDFVSAKADFGTDLA<br>ISTRSELDPASWWQQHGISCLELQRIAIRILSQTCSSIGCEHNWSAFDQVHIKRHNCLSRKRLNDQTYVHYNLRLRERQLG<br>RKPDELVSFDSAMLESVLDDWLVETEKLAMHEDEEIIYTEVEQFCGDDMDEHESEEKRPAEMVTIAGFIEPLDVIPSAGGV<br>TTDDDGLDFLDDDLTD	zf-BED--zf-BED--DUF-domain--Dimer_Tnp_hAT--	III
Gbar_D13G001890.1	GbaZf-BED77_I	MSSNLEPIPITSQKHDPAWKHCQMFKNGERVQLKCIYCGKIFKGGGIHRIKEHLAGHKGNAATCLRVPSNVRVLMQESLD<br>GVVVKKRKKQKIAEEITNVNQVSTEIQAYADQVDTNTGLLMIEKSDTLEPSSSLLVNQEGTSNVAGERRKRGRGKSLPAE<br>ANALSFVPVELGARRVNNHVHMAIGRFLFDIGATMDAVNSVYFQPMVDAIVSGGSGALMPSCNDLQGWILRKSVEEVKS<br>ENEKVMAAWVRTGCSILVNQWNTQTGRILLNFLVYCPEGTVFLKPIDASSVINSSDALYELLKQVVEEVGSKHVLQVITNG<br>EEQYIVAGRRLVETFPTLYWAPCAAHCVDLILEDFAKLEWINAIIEQARSITKFIYNHSVVLNMVRRYTFGNDIVEPAATRSA<br>TNFTTLTRMVDLKNNLQAMVTSQQWVDCPYSKKPGGLAMLDLVSNQSFWSSCVLIVRLTNPLLRVLRMAGSKKRPAMG<br>YVYAGMYRAKETIKKELVKRNEYMVYWNIIDHWWEQQWHHPLHAAGFYLNPRFFYSMEGDMPNEMLSGMLDCIEKLIPD<br>VTVQDKITKEINSYKNSIGDFGRKMAVRARDTLLPVEWWSTYGGSCPNLARLAIRVLSQTCSTLGLKHDHIPFEKLHETRN<br>CLEQQRLRDLIFVQCNLQLRQIGYESKQHDSMQPLSSESASIVEDWVTGIGAFLDDDTYPDWTTLETLSVNTMPLRPGDE<br>VEELGAVRFAQGGPLFPNCSKYAALGKHL	zf-BED--DUF-domain--Dimer_Tnp_hAT--	I
Ghe01G16520	GheZf-BED01_I	MTMASSNSPIHVDDGFNEYESAAKRQKSTTSKVWDEMTKLECENKNELKAQCNHCKTIFSAKSSSGTSHLRRHLNSCLK<br>KVNKDISQCTIATQPSLGGVPFIKNYKFDADECLEEPGFRYMMSIASPNFKNISRYTAARDVLMYYAKERDRVKEELARAP<br>GLICLTSDNWNSEHTNDEYICITAHWVDENWKLQKRIIRFRALFPPYDGLNIADELDNASYNDVMVSCLKNCFRANRAILC<br>DGAFFQVRCCAHILNLIVKAGLELADDVVAKIRNGIKYIKKSGTRRKRFYDVADKSFHLNVTKKLRQDVCVRWNSTYLMLE<br>SSLYYKDVLDYWGQRDKDYQLFALSNEEWRNVAILCKFLKVFYDVTCVFSGSNYPTANLYFRGVWKVHKVLLDTVKGPY<br>SFLTPMVKQMQEKFNKYWAEYSLILSCAAILDPRYKLNYVQYCFTTIYGIHASDFVETILSNLRLLFDEYVKKSKSTSSSLAG<br>SSNVSDKNPVDSSLGEHNVNNVDFGGDFDESDDYKRYLNESSTRCEKSQLDIYLEEPELELNSQIDVLDYWSKSSVRYN<br>ELSLLARDLLAIPISTVASESAFSMGKKVITPLRSSLKPKTVQAVDCLDDWMRAKGFSTEIGCKNDEDDEDDEEDEDDEDD<br>VSSIAF	zf-BED--DUF-domain--Dimer_Tnp_hAT--	I
Ghe01G16530	GheZf-BED02_I	MGLDKKRGPSNSPIHVDDGFNEYESAAKRQKSTTSKVWDEMTKLECENKNELKAQCNHCKTIFSAKSSSGTSHLRRHLN<br>SCLKKVNKDISQCTIATQPSLGGVPFIKNYKFDADECRKVVSTFLVCGKHSFRTVEEPGFRYMMSIASPNFKNISRYTAAR<br>DVLMYYAKERDRVKEELARAPGLICLTSDNWNSEHTNDEYICITAHWVDENWKLQKRIIRFRALFPPYDGLNIADELDNAS<br>YNDVMVSCLKNCFRANRAILCDGAFFQVRCCAHILNLIVKAGLELADDVVAKIRNGIKYIKKSGTRRKRFYDVADKSFHLNV<br>TKKLRQDVCVRWNSTYLMLESSLYYKDVLDYWGQRDKDYQLFALSNEEWRNVAILCKFLKVFYDVTCVFSGSNYPTANL<br>YFRGVWKVHKVLLDTVKGPYSFLTPMVKQMQEKFNKYWAEYSLILSCAAILDPRYKLNYVQYCFTTIYGIHASDFVETILSN	zf-BED--DUF-domain--Dimer_Tnp_hAT--	I

		LRLLFDEYVKKSKSTSSSLAGSSNVSDKNPVDSSLGEHNVNNVDFGGDFDESDDYKRYLNESSTRCEKSQLDIYLEEPELELNSQIDVLDYWSKSSVRYNELSLLARDLLAIPISTVASESAFSMGKKVITPLRSSLKPKTVQAVVCLDDWMRAKGFSTEIGCKNDEDDEDDEEDEDDEDDVSSIAF		
Ghe01 G2309 0	Ghe Zf- BED 03_I	MHFCSIFDNFYVFVIVTFIIAAGGWSYGLLQPASVSREAKDKVLVGWSKESNRMSTEPTSIEGSVTPPTSIDSENSGVGASSQANVTTGKRKATPQRSEVWSHFTKIINSEGASKAKCNYCQKEFCCDVKKNGTGSLKYHIGSCKKNPSNVVDPRHLSTWRFDQEACRKGLAQMIVIDELPFKFVESEGFKKFMFVARPRHIPSRTTMTKDVYQLYLDERVKIKQLLRSSCSRVCLTTDTWTSLQRVNYLCITAHFIDNDWKLNKKILNFCPISSHKGESIGMVIEKCLLTWGIDKLFTVTVDNASSNDVVIGCLRKKLNPRGGLVQNGKYLHMRCMAHIVNLIVVEGLKEMNKSVERVRGAVRYVRQSPARLQKFKECVVVEKIECKKMLCLDVCTRWNSTYLMLDTAQNIERAFERFEEQDTNFRAELERGEGWPSVDDWTNVRDLRDFLEHFYEVTLRISGTSYVTSNNFFDELSEIDILLRDAQLNSNVDFNVMAIKMKEKYDKINLLMFVACILDPRQKLKYLEFALSEMSSSEKASEMMQKLKESLYELFDEYKPSLYSTCSQSSVSTHVSLDEPQQKMKRRMQALYKKRELEICGEDKTSELDKYLAEANEEFVEDFDILLWWKVNSPRFPTLSKIARDVLAILVSTVVSESAFSTGGRVLDQYRSSLTPKIVQSLVCTQDWIRKSSSQEDIKKIEEQIQELDKIENGIFIILF	zf-BED--DUF-domain--Dimer_Tnp_hAT --	I
Ghe03 G0606 0	Ghe Zf- BED 04_I I	MPPLEEFPTKGLEGAPSNYIGWHFGTPVPNTKRNVVCKLCGKVVKGGITRFKEHIAHKIGNVAPCPNVTGVIRESMMNILKESNTTKIDKRGEQMNSYLN	zf-BED	II
Ghe03 G0632 0	Ghe Zf- BED 05_I	MSTEPTSIEGSVTPPTSIDSENSGVELQAKQMSEVWSHFTEIINNEGASKAKCNYCQKEFCCDVKKNGTRSLKYHIGSCKKNPSNVVDPSQGQLVLPRKGVEGGEGHLSTWRFDQEACRKGLAQMIVIDELPFKFVESEVFKKFMFVACSKFHITSRTTMTRDVYQLYLDERVKIKQLLRSSFNYLCITTHFIDNDWKLNKILNFCPISSHKGESIGMVIEKCLLNWGIDKLFTVTVDNASSNDVAIGYLRKKFNPRGGLVQNGKYLHMRCMAHIVNLIVVEGLKEMNKFVERVRGAARYVRQSPARLQKCKECVVVEKIECKKILCLDVCTRWNSTYLMLDTAQNFERAFERFEEQDTNFRAELERRKGWPSVDDWTIVRDLRDFLEHFYELSEIDILLRDAQLNSNVDFNVMAIKMKEKYDKYWGDIDKMNLLMFVACVLDPRQKLKYLEFALSEMSSSEKASEMMQKLKESLYELFDEYKPSLHSTCSQSSVSTHVSFGEPQQKMKRRMQALYKKRELEICGEDKTSELDKYLAEANEEFVKDFDILLWWKVNSPRFPTLSKMARDMLAIPVSTVASESSLTPKIVQALVCTQDWIRKSSSQEDIKKIEEQIQELDKIENDKNIAT	zf-BED--DUF-domain--Dimer_Tnp_hAT --	I
Ghe04 G0493 0	Ghe Zf- BED 06_I	MSDAVIVNSSRLKSIVWNDFDRVKKGDTFVAICRHCKKKLSGSSTSGTSHLRNHLIRCQRRSNHGVAQYFSAKDKKKEGSLALVTIDQEQKNDEVLSIVNLRYEQEQIKSEHVGIGSNSLDQRRSQFDLARMIILHNYPLAMVEHVGFKIFVRNLQPLFELATRNKVEADCMEIYAKEKQKVYEIFDKLPGKISVSADVWTASEDDAAYLSLAAHYIDENWQLKKKNLNFVTIDPSYTEDMHSEVIMNCLMDWDIDRKLFSMIFDSFTSDNIVERIRDRLSQNRFLYCNGQLFDVRCAVDLLNRMAHDALEALCEITQKIRESIRYVKSSEATQATFNELADEVQVETKKCLCIDNPLKWNSTYFMLEAALEYRKVFSCLRDRDPVNMKFLLSDPEWDRLITVTSFLKLFVEVTNVFTRSKYPTANIFFPEICDIHLQLIEWCKNPDEYISSLALKMRKKFEEYWYKCSSGLAVAAMLDPRFKMKLLEYYYPQLYGDSATELIDDVFECIKSLYNEHSIVSPLASSIDQGLDWQASGISGSGKDSRDRLMGFDKYLHETCQAEGSSSDLDKYLEEPLFPRNVDFNVLNWWKVHTPRYPILSMMARNILGIPISKVAAESRFDTGGRVLDHNWSSLPPTTIQALMCSQDWIRSELETNLIPLPQ	zf-BED--DUF-domain--Dimer_Tnp_hAT --	I
Ghe04 G2041 0	Ghe Zf- BED 07_I	MASSNTPIPVDDGFNEYESTVKRQKSTTSKVWDEMTKLECENKNELKAQCNHCKTVFSAKSSSGTSHLRRHLNSCLKKANKDITQYTIANQPSLGGVPFIKNYKFDADECRQAISIFLVCGKHSFRTVDEPGFRHMMRIASPNFKNISRHTAARDVLMYYAKERDRVKEELAKAPGLICLTSDNWNSEHTNDEYICITAHWVDKDWKLQKRIIRFRALFPPYDGLNIADELVLCLSQWGIDKKIFSITLDNASYNDVMVSCLKNRFRANRAILCDGAFFQVRCCAHILNLIVKAGLELADDVVAKIRNGIKYIKKSGIRRKRFYDVADKSFHLNVTKKLRQDVCVRWNSTYLMLESSLYYKDVLDYWAQRDKDYQMFALSSEEWRNVAILCKFLKVFYDVTCVFSGSNYPTANLYFRGVWKVHKVLLDTVKGPYSFVTPMVKQMQEKFNKYWAEYSLILSCAAILDPRYKLNYVQYCFTTIYGIHASDFVETILSNLRLLFDEYVKKSKSTSSSLAGSSNVSDKNPVDSSLDEHNVNSADFGRDFDESDDYKRYLNESSTRSEKSQLDIYLEEPELELNSQIDVLDYWSKSSVRYNELSLLARDLLAIPISTVASESAFSMGKKVITPLRSSLRNVRILGLTGQDRTEMSHVRIIHVSPCRKHGIDRILIDRNDLSILSIPRVLQYIHECKECIC	zf-BED--DUF-domain--Dimer_Tnp_hAT --	I
Ghe05 G2287 0	Ghe Zf- BED 08_I	MNDPKRDGSTQSSQNSSPSRDDQTTTKENARKKITPRAACWSHFTKFVTKEGEKRARCNACDVTYTMESTSGSTTNLNNHLKICLKKPRGNTSNTKQSELSFVKVSQETRDLSTWVFDKDAIRKALVRMIIVDELPFKIVEGEGFKYFLSIACPRFSLPSCWTIRKDCIDLFNSMKSVMKDCFEKDISKVCLTTDTWTSLQRTSYMVLTAHWVDDEWRLQKRIINFCPISAYRGESIGQAIEKCLRDWGIERVFTITVDNASANSVAIEYLRKKLNHRNASVANGKFIHMRCVAHILNLIVQYGIKDASMSVDRVRDAVRYIRASPSRLTKFNQWVKEEMIDSKAQLCLDVPTRWNSTYMMLKVAEKYEHAFESYLRDDHNFFLDLTAGDGVPTFDDWDIVRRVIKILEPFYHLTLKVSGSLHVTSHSLFEVLIDVHCLFDGWQDCGDLEIISMTSKMREKYNKYWGEGNKINMLVYLAIIFDPRCKMSFLDFGVNLLFPNVANDIMKMIDKELHCLFNEYSSNAGRIQLFEGRSSSLTNLCSSMEIDQSEMKTGLAKQKYLKKKKQVGLESKSELDRYLGEDEEVNNSSSFDLLLWWKMNSPRFPILAQMARDILATLISTVASESAFSTGGRVRDSFRSSLTPLMVEVLVCTQDWLRKSNDAINLEDYVDELQTMEDELTLNVVGQLRIDYSICFAFPFSELSSFCS	zf-BED--DUF-domain--Dimer_Tnp_hAT --	I
Ghe05 G2712 0	Ghe Zf- BED 09_I	MPPREEFPTKGLEGAPSNDIGWHFGTPVPNAKGNIVCKLCGKVVKGGITRFKEHIAHKTGNVAPCPNVTESMMNVLKENNTKKIEKKRRKDDFLSQLREEEDEHEEFIDEISAIRQATRESIQSQHECHRREEFRRSTGGWDNIYEKGRSSQGSSIPTESEFTLRGTIPKLVRSKSSKQPKVSDSFLKSFRKKIASSPWLYNLIQVSTEVGQGVKLPTPYEVSDVYLESEYQRVHNWVNGLKTHWKELGATLMCDGWTNSLNQMHIINFLVYCSKGTIFWKSVDVSSVRSRDAEFYYTMKAAGKKLMLKREHLYWTSCTAHCLDLCLEDIGKRPSVAKVLDEAKKVTCFIYNHTWTVDLMKKYTQGKQILRPALTRFATHFIQLEEITRQKQGLREMFSSKEYKESKWGQQKSGPAYEAKKIILGKDFWKKANDLIKVYEPLVKVLRLVDSDEKPTMGFIYEAVDRAKRAIQQDCRYFTEYEKIIDKRWNFMHSDLHSAGYFLNPQFQFGVEHSHNVLIETLEGTRSVIERLEPSLDTQVRMVNQVRFNSNKHETFGTPQAQRAWKQMNPAEWWIIYGTCVPELQKLAIKVLSQTTSASNCERNWSTFSYIHTKARNRLKYKKLEKLVHQKRMSTDDINTSFNPIKWLHEKENPLLDGENAGSGDGPDGGGLSPVDEDDGYNGDRGELRSSSQYRGEYGVGTTSRHFRDRSEFDGNMFPEPRRDRSEPRAPSKGKGKKHTSIGSSSGRRLGSSNLGYSDSSTSTQGFYPPEQPSYFQPSHGYPQPYGYYPPFPNYGVPYQPQMHPPPPMYHPPPPLI	zf-BED--DUF-domain--Dimer_Tnp_hAT --	I
Ghe06 G0654 0	Ghe Zf- BED 10_I	MFALDELLEAVVLLYIYIYTHIHTYISWTTFLKFLFLSAAGFTSMTEMTEIADMETIPGESNNQLALTTPEAQPIKRRKKKSMVWEYFTIENVSAGCRRAYCKRCKQSFAYSTGSKVAGTSHLKRHIAKGTCRALLRGQGQDNNQFITPYNPKMGGSEPPKRRYRSPSSPFIPFDQDRCRHDIARMIIMHEYPLHIVEHPGFIAFVQSLQPQFDKMSFNTVQGDCVATYLREKQSLMKFIEGIPGRFCLTLDMWSSNQTLGYVFITGHFVDSDWKLHRRVFNVVMEPYPDSHSALSHAIAACISDWSLEGKLFSLTFNHPLSEAGLENLRPLLCVKNPLILNGQLLIRNCIARTMSSMAKDVLGAGQEIIKKIRDSVKYVKMSESHDDKFIQVKNQLQVPSEKSLFLDNQTRWNTTYQMLAAASELKEVFDCLDTYDPDYKLAPSMEDWKLAETLCSFLKPLFDAASILTTTTLPTVITFFYEVWKIHVDLGRSITSEDPFISNLAKSMQEKIDKYWKDCSLVLAMAVVMDPRFKMKLVEFSFTKIYSEGAPTYIKTVDDGIHELFLEYVALPLPLTPTYAEEVNGANNGKTNESHYGNLLSDHGLTDFDVYIMETNSQQMKSELDQYLEESLLPRVQEFDVLGWWKLNKMKYPTLSKMARDILSIPVSAAAPESIFDITDKQLDEYRSSLRPETVEALICAKDWLHFGSSDVSNALVKMEF	zf-BED--DUF-domain--Dimer_Tnp_hAT --	I
Ghe06 G0655 0	Ghe Zf- BED 11_I	MFALDELLEAVVLLYIYIYTHIHTYISWTTFLKFLFLSAAGFTSMTEMTEIADMETIPGESNNQLALTTPEAQPIKRRKKKSMVWEYFTIENVSAGCRRAYCKRCKQSFAYSTGSKVAGTSHLKRHIAKGTCRALLRGQGQDNNQFITPYNPKMGGSEPPKRRYRSPSSPFIPFDQDRCRHEIARMIIMHEYPLHIVEHPGFIAFVQSLQPQFDKMSFNTVQGDCVATYLREKQSLMKFIEGIPGRFCLTLDMWSSNQTLGYVFITGHFVDSDWKLHRRVFNVVMEPYPDSHSALSHAIAACLSDWSLEGKLFSLTFNHPLSEAGLENLRPLLCVKNPLILNGQLLIRNCIARTMSSMAKDVLGAGQEIIKKIRESVKYVKMSESHDDKFIQVKNQLQVPSEKSLFLDNQTRWNTTYQMLAAASELKEVFDCLDTYDPDYKLAPSMEDWKLAETLCSFLKPLFDAASILTTTTLPTVITFFYEVWKIHVDLGRSITSEDPFISNLAKSMQEKIDKYWKDCSLVLAMAVVMDPRFKMKLVEFSFTKIYSEDAPTYIKTVDDGIHELFLEYVALPLPLTPTYAEEVNGANNGKTNESHYGNLLSDHGLTDFDVYIMETNSQQMKSELDQYLEESLLPRVQEFDVVGWWKLNKMKYPTLSKMARDILSIPVSAAAPESIFDITDKQLDEYRSSLRPETVEALICAKDWLHFGSSDVSNALVKMEF	zf-BED--DUF-domain--Dimer_Tnp_hAT --	I
Ghe06 G1301 0	Ghe Zf- BED 12_I	MTMASSNSPILVDDGFNKYESAPKRQKSTTSKVWDEMTKLECEDKNKLKAQCNHCKTIFSAKSSSGTSHLRRHLNSSTQPSLGGVPFIKNYKFDADKCRQAISTFLVYGKHSFRTVEESGFRYMMRIAIPNFKNISIYTATRDLAKAPGLICLTFDNWNSEHTNDEYIRITAHWVDNYWKLQRRIISFKALFPPYDGLNIVDELVLCLSQWSIDKKIFSITLDNASYNDLMVSCLKNQFRANRAILCDGAFFQVRCCAHILNLIVEAGLELADDVVGKIRNGIKYIKKSGIRKKRFYDVADKSFHLNVTKKLRQYVCVRWNSTYLMLESSLYYKDVLDYWGQRDKDYQIFALSNEEWRNVAILCKFFKVFYDVTCVFSGSNYPTTNLYFRGVWKVHKVLLGTVKGPYSFLTPMVKQMQEKFNKYWAEYSLILSCAAILDPRYKLDYVQYCFTTIYGIHASDFVETILSNLRLLFDEYVKKSKSMFSCLAGSSNVSNKNPVDSSLDEHNVNSVDFGGDFDESDDYKRYLNESSTMSEKAQLDIYLEESALELNSQIDVLDYWSKSSVRYNELSLLARDLLAIPISTKVITPLRSSLKPKTVPAVVCLDDWMRAKGFSTEIGFKNDDDDENDEGDEDDVSSIAF	zf-BED--DUF-domain--Dimer_Tnp_hAT --	I
Ghe06 G2108 0	Ghe Zf- BED	MTMASSNSPIPVDDEFNEYESAPKRQKSTTSMVWDEMTKLECENKNELKAQCNHCKTIFSAKSSSGTSHLRRHLNSCLKKVNKDITQYTIATQPSLGGVPFIKNYKFDADKCRQAISTFLVCSKHAFRTVEEPGFRYMMRISSPNFKNISRYTAARDLAKAPGLICLTSDNWNSEHTNDEYICITTHWVDKDWKLQKRIIRFRALFPPYDGLNIADELVLCLSQWGIDKKIFSITLDNASYNDV	zf-BED--Dimer_Tnp_hAT --	V

	13_V	MVSCLKNHFRANRAILCDGAFFQVRCCAHILNLIVKAGLELADDVVGKIRNGIKYIKKLRIRRKRFYDVADKSFHLNVTKKLRQDVCVGWNSTYLMLESSLYYKDVLDYWGQRDKDYQIFVLSNEEWRNVAILCKFLKVFYDVTCVFSGSNYPTANLYFRGVWKVHKLLLDTVKDFVETILSNLRLLFDEYVKRSKSTSSSLARSSNVSDKNLVDSSLDKHNVNSVDFGFGGDFDESDDYKRYLNESSTMSEKSQLDIYLEEPALELNSEIDLSLLVHDLLAILISTVASESAFSMGKKVITPLRSSLKPKMVQAIICLDDWMRAKGLSTEICYKNDDDDEDDEDDVSLITF		
Ghe07G15740	GheZf-BED14_I	MSTEPTSIEGSVTPPTSIDSENSGVGASSQANVTTGKRKATPQRSEVWSHFTKIINSEGASKAKCNYCQKEFCCDVKRNGTGSLKYHIGACKKNPSNVVDTSQGQLVLPRKGVEGGEGHLSTWRFDQEACRKLLAQMIKFMFVACPRFHIPSRTTMTKDVYKLYLDERVKIKQLLKSSCSRVCLTTDTWTSLQRVNYLCITAHFIDNDWKLNKKILNFCPISSHKGESIGMVIEKCLLNWGIDKLFTITVDNASSNDVAIGYLRKKFNLRGGLVQNEMNKSVERVRGAVRYVRQSPARLQKFKECVVLEKVECKKMLCLDVCTRWNSTYLMSDTEQDTNFRAELERGEGWPSVDDWANVRGLRDFLEHFYEVTLRISGTSYVTSNNFFDELSEIDILLRDAQLNSNVDFNVIAIKMKEKYDKYWGDIDKMNLLMFVACVLDPRQKLKYLEFALSEMSSSEKASEMMQKLKESLYELFDEYKPSLHSTCSQSSVPTHVSLGEPQQKMKRRMQALYKKRKLEICGEDKTSELDKYLAEANEEFVEDFDILLWWKVNSPRFPTLSKMARDVLAIPVSTVASESAFSTGGRVLDQYRSSLTPKIVQALVCTQDWIRKSSSQEDIKEIKEQIQELDKIENGIFITYVIFLIM	zf-BED--DUF-domain--Dimer_Tnp_hAT--	I
Ghe07G24060	GheZf-BED15_I	MGLDKKRGPFKNGPGLGLNSPIHVDDGFNEYESAAKRQKSTTSKVWDEMTKLECKNKNELKAQCNHCKTIFSSKSSSGTSHLRRHLNSCLKKVNKDISQYTIATQPSLGGVPFIKNYKFDADECRKAVSTFLVCGKHSFRTVEEPGFRYMMSIASPNFKNISRYTAARDVLMYYAKERDRVKEELARAPGLICLTSDNWNSEHTNDEYICITAHWVDENWKLQKRIIRFRALFPPYDGLNIADELVLCLSQWGIDKKIFSITLDNASYNDVMVSCLKNRFRANRAILCDGAFFQVRCCAHILNLIVKAGLELADDVVAKIRNGIKYIKKSGTRRKRFYDVADKSFHLNVTKKLRQDVCVRWNSTYLMLESSLYYKDVLDYWGQRDKDYQLFALSNEEWRNVDILCKFLKVFYDVTCVFSGSNYPMANLYFRGVWKVHKVLLDTVKGPYSFLTPMVKQMQEKFNKYWAEYSLILSCAAILDPRYKLNYVQYCFTTIYGIHASNFVETILSNLRLLFDEYVKKSKSTSSSLAGSSNVSDKNPVDSSLGEHNVNNVDFGGDFDESDDYKRYLNESSTRSEKSQLDIYLEDPELELNSQIDVLDYWSKSSVRYNELSLLARDLLAIPISTVASESAFSMGKKVITPLRSSLKPKMVQAVVCLDDWMRAKGFSTEIGCKNDEDDEDDVSSIDF	zf-BED--DUF-domain--Dimer_Tnp_hAT--	I
Ghe07G26640	GheZf-BED16_II	MADPDANTLTPSKPLTSASCTPISLDDDESPVDSGLEDPSILPLTSRHTSSVWTNFTRNRVGDAIKAECNHCSKLLASGKRVGTTHLKDC	zf-BED	II
Ghe07G30270	GheZf-BED17_I	MGLDKKRGPSNSSIHVDDGFNEYESAAKRQKSTTSKVWDEMTKLECENKNELKAQCNHCKTIFSAKSSSGTSNLRRHLNSCLKKVNKDISQYTIATQPSLGGVPFIKNYKFDADECRKAVSTFLVCGKHSFRTVEEPGFRYMMSIASPNFKNISRYTAARDVLMYYAKERDRVKEELARAPGLICLTSDNWNLEHTNDEYICITAHWVDENWKLQKRIIRFRALFPPYDGLNIADELVLCLSQWGIDKKIFSITLDNASYNDVMVSCLKNRFRANRAILCDGAFFQVRCCAHILNLIVKAGLELADDVVVKIRNGIKYIKKLGTRRKRFYDMADKSFHLNVTKKLRQDVCVRWNSTYLMLESSLYYKDVLDYWGQRDKDYQLFALSNEEWRNVAILCKFLKVFYDVTCVFSGSNYPTANLYFRGVWKVHKVLLDTVKGPYSFLTPMVKQMQEKFNKYWAEYSLILSCAAILDPRYKLNYVQYCFTTIYGIHASDFVETILSNLRLLFDEYVKKSKSTSSSLAGSSNVSDKNPVDSSLGEHNVNNVDFGGDFDESDDYKRYLNESSTRSEKSQLDIYLEEPELELNSQIDVLDYWSKSSVRYNELSLLARDLLAIPISTVASESAFSMGKKVITPLRSSLKPKTVQAVNDEDDEDDEDDEDDVSSIAF	zf-BED--DUF-domain--Dimer_Tnp_hAT--	I
Ghe08G12020	GheZf-BED18_I	MAEMTEATNMETSPVENNNELALITPETQPKRRKKKSMVWEYFTIETVSAGCRRACCNRCKQSFAYSTGSKVAGTSHLKRHIAKGTCPALLRDQYNNQLTPYSPKTGGGEPRKRRYRSPSSPFIPFDQDHCRHEIARMIIMHEYPLHMVEHPGFIAFVQNLQPRFDKVSFNTVQGDCVATYLREKQSLMKLIEGIPGRVCLTLDMWTSNQTLGYVFITGHFIDFEWKLQRRVLNVIMEPYPDSDSALSHAVAACLSDWSLEGKLFSLIFNHPTSEAGLENLRPLLCTKNPLILNGQLLLGNCMARTLSSMAKDVLGAGHEIVKKIRDSVKYVKTSESHDEKFVQVKNQLQVPSEKSLILDNQNQWNTTYQMLAAASELKEVFNCLDTSDPDYKLAPSMEDWKVAETLCSFLKPLFDAASILMTTTNPTAITFFHEAWKIHADLGRSIANDDPFISNIAKSMLEKIDKYWKDCSLILAIAVVMDPRFKMKLVEFSFTKIFGEDAPTYIKIVDDGIHELFLEYVALPLPLTPTYTEEGNVGNNGKTDESQQGNLLSDQGLTDFDVYIMETSSQQMKSELDQYLEESLLPRVQEFDVLGWWKLNKMKYPTLSKMARDILSIPVSAAAPDSVFDIIIKQLDEYRSSLRPETVEALICAKDWLHHGSEESNALVKMEF	zf-BED--DUF-domain--Dimer_Tnp_hAT--	I
Ghe08G16000	GheZf-BED19_V	MASSNSPIPVDDGFNVYESAPKHQKSTTSKVWDEMTKLECENKNELKAQCNHCKTIFSAKSSSGTFHLRRHLNICLKKVNKDITQYTIANQPSLGGCRQAVSTFLVCGKHSFRTVEESGIRYIMRIASPNFKNISRYTAARDVLMYYAKEGDRVKEELAKAPGLICLTSDNWNSKHTNDEYICITAHWVDKDWKLQKRIIRFRALFPPYDGLNIADELVLYLSQWGIDKKIFSITLDNASYNDVMVSCLKNRFRANRAILCNGAFFQVRCSAHIWNLIVKAGLELADGVVGKIRNGIKYIKKSGIRRKRFYDVADKSFHLNVAKKLRQDVCVRWNSTYLMVESSLYYKDVLDYWGQRDKDYQIFALSNEEWRNVAILCKFFKVFYDVTCVFSGSNYPTANLYFRGVWKVHKLLLDTVKDFVETILSNLRLLFDKYVKRSKSTSSSLAGSSNVSDKNPVDSSLDEHNVNSVDFGFGGDFDESDDYKWYLNESSTMSEKSQLDIYLEEPALELNSQIDVLDYWSKSSVRYNELSLLASSESAFSMGKKVITPLRSSLKPKTIQAVVCLDDWMRVKGFLTEIGCKNDDDDEDDEDDEDDVSLIAF	zf-BED--Dimer_Tnp_hAT--	V
Ghe08G30250	GheZf-BED20_I	MEVANETVIKKPKRLTSVVWNHFERVRKADLCYAVCVHCNKKLSGSSNSGTTHLRNHLMRCLKRFNYDVSQLLSAKKRKKDNTLTIANISYDEGQRKEEYLKPTIVKYEPEQRKDEVFNVQSSWFDQERSRLDLARMIILHGYPLAMVEHVGFKVFVKNLQPLFDVVPNSTVELSCMEIYGKERQKVHDMLSKLQGRINLAVEMWSSPENTNHVCMMAHYVGDDWKLQKKILNFVTLDSSHTDDLLSGVIIKCLMDWDIGSKLFAVTLDDFSANDDIVLRIKEQISENKSRLSNGQLLDVRSAAHVLNSIVQDAMEALRLVIQKIRGTVRYVKSSQSIQGKFKEMVLQTGINSQKNLVLDCPIQWNSTYLMLETAIEYRNAFCQLPELDLDLALSDEEWEWASSITGYLKLFIEIINVFSSNKCPTANIYFPEICHVHIQLIDWCKSPDNFLSSLAAKMKAKFDKYWSKCSLSLAVAAILDPRFKMKLVEYYYSQIYGSTALERIKEVSDGLKELFNTYSICSTLMDQGSALPLSSLPSSSNDGRDRLKGFDKFLHETSQSQTAISDLEKYLDEPVFPRNCNFNILNWWRVHTPRYPILSMMARDVLGTPMSTVSQESAFHAGGRVLDSCRCPLTPETQQALICTQDWLRMQSDDPGPSSSHYALPLYVETN	zf-BED--DUF-domain--Dimer_Tnp_hAT--	I
Ghe09G05740	GheZf-BED21_I	MSTEPTSIKGSVTPPTSIDSENSGVGASSQANVTTGKRKATPQRSEVWSHFTKIINSEGASKAKCNYCQKEFCCDVKKNGTGSLKYHIGSCKKNPSNVVDRSQGQLVLPRKGVEGGEGHLSTWRFDQEACRKGLAQMIVIDELPFKFVESEGFKKFMFVACPRFHIPSRTTMTRDVYQLYLDERVKIKQLLKSSCSRVCLTTDTWTSLQRVNYLCITAHFIDNNWKLNKKILNFCPISSHKGESIGMVIEKCLLNWGIDKLFTVTVDNASSNDVAIGYLRKKFNPRGGLVQNGKYLHMRCMAHIVNLIVVEGLKEMNKSVERVRGAVRYVRQSPARLQKFKECVVMEKIECKKMLCLDVCTRWNSTYLMLDTAQKFERAFERFEEQDTNFRAELERGEGWPSVDDWANVRNLKDFLEHFYEVTLRISGTSYVTSNNFFDELSEIDILLRDAQLNSNVDFNVMAIKMKEKYDKYWGDIDKMNLLMFVACVLDPRQKLKYLEFALSEMSSSEKACEMMQKLKESLYELFDEYKPPLYSTCTQSSVPTHVSLGEPQQKMKRRMQALYKKRELEIGGEDKTSELDKYLAEANEDFIEDFDILLDVLAIPVSTVASESTFSTGGRVLDQYRKLDMIENGIMQMEIFWREEMDTNGEY	zf-BED--DUF-domain--Dimer_Tnp_hAT--	I
Ghe09G23340	GheZf-BED22_I	MHQDKLKKRRRAGNLIGPNLKKNPRYLAQKEKRNQHVISNSNLLYLKENFDHLMFDEDVCWEYAEKLDGNKVRCKFCLRVLNGGISRLKHHLSRLPSKGVNPCNKVRDDVTDRVRAIISSKEDIKEIPSVKKQKIAEVRAPGNMSTGSKISPLETLSPAAKVFPTVLSIAASTLSDQETVERSIAFVARSSSYQAMIDAVGKFGPGLIAPSVETLKTTWLKRIKSEVTLHLKDAEKEWATTGCTIIADTWTDNKSKALINFLVSSPSRTFFHKSVDASSYFKNTKCLADLFDSVIQDFGQENVVQIIMDSSFNYTGISSHILQNYGTIFLSPCASQCLNLILEEFSRVDWVNRCILQAQTVSKFLYNNASMLDLMKKFTGGQELIRTGITKSVSCFLSLQSTLKQRSRLKHMFNSPEYSTNSSYANKPQSISCIAIVEDNDFWRAVEECVAISEPFLKVLREVSGGKPAVGSIYELMTRAKESIRTYYIMDESKCKTFLDIVDRQWRDQLHSPLHSAGAFLNPSIQYNPEVKFLGSIKEDFFKVLEKLLPTPELRRLWWEQFGDSAPVLQRVAIRILSQVCSTFTFERHWSTFQQIHTEKRNKIDKETLTDVVYINYNLKLAREMKTMPTDSDPIQFDDIDMTSEWVEESENPSPTQWLDRFGSALDGGDLNTRQFSAAMFGNDHIFGL	zf-BED--DUF-domain--Dimer_Tnp_hAT--	I
Ghe09G25140	GheZf-BED23_I	MGLDKKRGPSNSPIHVDDGFNEYESAAKRQKSTTSKVWDEMTKLECENKNELKAQCNHCKTIFSAKSSSGTSHLRRHLNSCLKKVNKDISQYTIATQPSLGGVPFIKNYKFDADECRKAVSTFLVCGKHSFRTVEEPGFRYMMSIASPNFKNISRYTAARDVLMYYAKERDRVKDELVRAPGLICLTSDNWNSEHTNDEYICITAHWVDENWKLQKRIIRFRALFPPYDGLNIADELVLCLSQWGIDKKIFSITLDNASYNDVMVSCLKNRFRANRAILCDGAFFQVRCCAHILNLIVKAGLELADDVVAKIRNGIKYIEKSGTRRKRFYDVADKSFHLNVTKKLRQDVCVRWNSTYLMLESSLYYKDVLDYWGQRDKDYQLFALSNEEWRNVAILCKFLKVFYDVTCVFSGSNYPTANLYFRGVWKVHKVLLDTVKGPYSFLTPMVKQMQEKFNKYWAEYSLILSCAAILDPRYKLNYVQYCFTTIYGIHASDFVETILSNLRLLFDEYVKKSKSTSSSLAGSSNVSDKNPVDSSLGEHNVNNVDFGGDFDESDDYKRYLNESSTRSEKSQLDIYLEEPELELNSQIDVLDYWSKSSVRYNELSLLARDLLAIPISTVASESAFSMDKKVITPLRSSLKPKTVQAVVCLDDWMRAKGFSTEIGCKNDEDDEDDEDDEDDVSSIAF	zf-BED--DUF-domain--Dimer_Tnp_hAT--	I

Ghe10G13030	Ghe Zf-BED 24_II	MPPREEFLAKGSEAAPSNDIGWHFGTPVPNTKGNFICKVCGKFVKGGITRFKEHIAHKTDNVAPCPNVTGM	zf-BED	II
Ghe11G00830	Ghe Zf-BED 25_I	MTFGLHLLQIVSFVGNKMEWSVNNAFKSYKDMEPKSTMDMVLIPNMDTIDIVLGSSEKGNVVPSAKPRKKTMTSVYLKYFETAPDGKTRRCKFCGQSYSIATATGNLGRHLSNRHPGYDKTGENVSSSAPQPSTTPTVIKKPQPQGRAPQVDYDHLNWLLIKWLILATLPPSTLEEKWLANSFKFLNPSIQLWPGEKYKAVFREVFRSMREDVRASLEQVSSKVSIALDFWSSYEQIFYMSITCQWIDENWSFQKVLLDICQVPYPCTGSEIYNSLVKVLKMYNIENKVLSCTHDNSQNAIHACHALKEDLDGQKMGPFCFIPCAARTLSLIIDDALRTTKPVIAKVREFVQELNASLDISEDFIQLATAYKEGSWQFPLDASARWSGSYQMLDIVQKAGKSMDAVVRKNEELLGNRMLLNTAEKNVVNIVHNYLEPFYKVISEICVNTPPTIGMVIVYMDHISDTITTRQPPDWLKNPAEDMAKKLRSYNNQVCNIFIHMTAILDPRIKCELIPESLNSKNYLEEARAHFVRNYYTTPFSSMTSGYSSQDIEDGGAVSFAEEIARKKRRVSMNNATDELTQYLSESPTPTKTDVLEWWKVNSTRYPRLSAMARDFLAVQATSVKPDELFCSKGDEIDKQRFCMPHDSTQAILCIKSWTQGGLKLKYKSTEIDYERLMEMAAAATADISLAGMDKKQK	zf-BED--DUF-domain--Dimer_Tnp_hAT--	I
Ghe11G11080	Ghe Zf-BED 26_I	MASSNSPIRVDDGFNEYESAAKRQKSTTSKVWDEMTKLECENKNELKAQCNHCKTIFSAKSSSGTSHLRRHLNSCLKKVNKDISQYTIATQPSLGGVPFIKNYKFDADECRKAVSTFLVCGKHSFRTVEEPGFRYMMSIASPNFKNISRYTAARDVLMYYAKERDRVKEELARAPGLICLTSDNWNSEHTNDEYICITAHWVDENWKLQKRIIRFRALFPPYDGLNIADELVLCLSQWGIDKKIFSITLDNASYNDVMVSCLKNRFRANRAILCDGAFFQVRCCAHILNLIVKAGLELADDVVAKIRNGIKYIKKSGTRRKRFYDVADKSFHLNVTKKLRQDVCVRWNSTYLMLESSLYYKDVLDYWGQRDKDYQLFALSNEEWRNVAILCKFLKVFYDVTCVFSGSNYPTANLYFRGVWKVHKVLLDTVKGPYSFLTPMVKQMQEKFNKYWAEYSLILSCAAILDPRYKLNYVQYCFTTIYGIHASDFVETILSNLRLLFDEYVKKSKSTSSSLAGSSNVSDKNPVDSSLGEHNVNNVDFGGDFDESDDYKRYLNESSTRSEKSQLDIYLEEPELELNSQIDVLDYWSKSSVRYNELSLLARDLLAIPISTVASESAFSMGKKVITPLRSSLKPKTVQAVVCLDDWIRAKGFSIGNYYSRFIVFGVVGDAVFCAVLLLFGCCFGAHEIEALICL	zf-BED--DUF-domain--Dimer_Tnp_hAT--	I
Ghe11G38980	Ghe Zf-BED 27_I	MEVANESTAKKPKRLTSVVWNHFERVKKADICYAVCVHCNKKLSGSSNSGTTHLRNHLMRCLKRSNYDVSQLLAVKRRKKENTLTIANISYDEGQRKEDYMKPTIVKYEQDQRKDEAFNLGSSWFDPERSRLDLARMIILHGYPLAMVEQVGFKVFVKNMQPLFDVVHNSTIELSCVEIYMKEKQRIYDMLSKLQGRINLAIEMWSSPENSKYVCLTAHYVDDEWKLQKKILNFLTLDSSHTEDVLSDVIIKCLMDWDIDCKLFAMTFDDCSTNDDIVSRIKDQVSESRPRLSNGQLLDVRSAAHVLNSIAQDAIEALQVVIQKIRGSVKYVKSSQSILGKFNEIAQQQGIDNHKIVVLDYPIRWNSTYMMLETAVEYRNVFHHLPELDPDFALSDEEWERASSIVSCLKLLIEIINVFSSNKCPTANIYFPEICHVHIQLIEWCKSSDAFLSSLATKMKAKFDKYWSKCSLALAVAAILDPRFKMKLVEYYYSQIYGSTALERIKEASDGIKELFNAYSICSTLIDQGSALPGSSLPSSSNDTRDRLKGFDKFLHETSQSQTAISDLEKYLDEPVFPRNCDFNILNWWRVHTPRYPILSMMARDVLGTPMSTIAQEFAFNAGGRMLDSNQSSLPPDTQQALICTRDWLRTQSDDATPSSSHYALPLYVEAN	zf-BED--DUF-domain--Dimer_Tnp_hAT--	I
Ghe12G03660	Ghe Zf-BED 29_I	MSTEPTSIKGSVTPPTSIDSENSGVGASSQANVTTGKRKATPQRSEVWSHFTKIINSEGASKAKCNYCQKEFCCDVKKNGTGSLKYHIGSCKKNPSNVVDPSQGQLVLPRKGVEGGEGHLSTWRFDQEACRKGLAQMIVIDELPFKFVESEGFKKFMFVACPRFHIPSRTTMTRDVYQLYLDERVKIKQLLKSSCSRVCLTTDTWTSLQRVNYLCITAHFIDNDWKLNKKILNFCPISSHKGESIGMVIEKCLLNWGIDKLFTVTVDNASSNDVAIGYLRKKFNPRGGLVQNGKYLHMRCMAHIVNLIVVEGLKEMNKSVERVRGAVRYVRQSPARLQKFKECVVMEKIECKKMLCLDVCTRWNSTYLMLDTAQKFERAFERFEEQDTNFRAELERGEGWPSVDDWANVRNLRDFLEHFYEVTLRISGTSYVTSNNFFDELSEIDILLRDAQLNSNVDFNVMAIKMKEKYDKYWGDIDKMNLLMFVACVLDPRQKLKYLEFALSEMSSSEKACEMMQKLKESLYELFDEYKPPLYSTCSQSSVPTHVSLGEPQQKMKRRMQALYKKCELEIGGEDKTSELDKYLAEANEDFIENFDILLWWKMNSPRFPILSKMARDVLAIPVSTVASESSLTPKIVQALVCTQDWIRKSSSQEDIKKIEEQIQELDMIENDVNAVKKFIREWEKGIWVS	zf-BED--DUF-domain--Dimer_Tnp_hAT--	I
Ghe12G12530	Ghe Zf-BED 30_IV	MPPCEEFPTKGLEGAPSNDIGWHFGTPVPNTKGNIVCKLCDKVMKGGITRFKKHIAHKTGNVAPCPNVTGVIRESMMNILKESKTKKIDKKRRKDEFLSQLREDEDKHEKFIDEVFAIRERISKSILSESEFTLRGVIPELAKSKSSKQPKVSDSILKTFRKKIGEVVSKLLIYERLPFQLASSPWLYNLIQVSTEVGQGVKLPTPYEISDVYLESEYQRVRDWVNGLKTHWKELGATLMCDGWTNSLNQIHIINFLVYCNASSVRSRDVEFYYRLLDSVEAMKATGKKLMLKRKHLYWTLLNMTPIFTHCLDLCLEDIGKKPSVAKVLDEAKKVTCFIYNHIWTVDLMKKYTQGKQILRPALTRFTTHFIQLEEITRQKQGLREMFDSKEFKESKWGQQKLGPAYEAKKIVLRKDFWKKANDLIKVYEPLVKVLRLVDSDEKPTMSFIYEAVDRAKRAIQQDCRYFIEYEKIIDNRWNFIHSDLHLADYFLNPQFQFGVEHSENVLIEILEGTRSVIERLEPSLNTQVRMVNQLLLFRDKHETFGTPQAQRAWKQMNPGKHTFSYIHTKARNRLKYKKLEKLVFTYYNMRLQIRHQKRMSTDDINASFNPISLDHIFEDVDPLSEWLHEKENPLLDGENTGVLPVDTSDNEMDVDQSQQQNLSHSSSSSTPSQSGDGPDGSGLSPIDEDDGYSGDRGEIRSSSRYGREYGVGTTSGHFYDRSKFDGNMFPEPRRDRSKKHTSIGFSSGRRSSSSNLGYNDSSTSTQGFYPPEQPLYFQPSHGYPQPYGYYPPFPNYGVPYQPQMHPPPPMYHPHPPLI	zf-BED--DUF-domain	IV
Ghe12G24770	Ghe Zf-BED 31_I	MSIEPTSIEGSVTPPTSIDSKNSGIGAPSQANVTTGKRKATPQRLEVWSHFTKIINSEGASKAKCNYCQKEFCCDVKRNSTGSLKYHIGACKKNPSNVVDTSQGQLVLPRKGVERGEGHLSTWRFDQEACRKGLAHFNEGFKKFMFVACPRFHIPSRTTMTRDVYQLYLDERVKIKQLLKSSCSRVCLTTDTWTSLQRVNYLCITAHFIDNDWKLNKKILNFCPISSHKGESIGMVIEKCLLNWGIDKLFTITVDNASSNDVAIGYLRKKFNPRGGLIQNGKYLHMRCMAHIGAIRYVRQSPARLQKFKECVVVEKIECKKMLCLDVCTRWNSTYLMLDTEQDTNFRAELERGEGWPSVDDWANVRDLRDFLEHFYEVTLRISGTSYVTSNNFFDELSEIDILLRDAQLNSNVDFNVMAIKMKKKYDKYWGDIDKMYLLMFVACVLDPRQKLKYLEFALSEISEKASEMMQKLKESLYELFDEYKPSLHSTCSQSSVPTHVSLGEPQQKMKRRMQALNKKRKLEICGEDKTSELDKYLAAANEEFVEDFDILLWWKVNSPRFPTLSKMARDVLAIPVFTVASESAFSTGGRVLDQYRSSLTPKIVQALVCTQDWIRKSSSQEDIKEIEEQIQELDKIENGIFITYVIFLIM	zf-BED--DUF-domain--Dimer_Tnp_hAT--	I
Ghe13G02340	Ghe Zf-BED 32_I	MSSNLEPIPITSQKHDPAWKHCQMFKNGERVQLKCIYCGKIFKGGGIHRIKEHLAGHKGNAATCLRVPSDVRVLMQESLDGVVVKKRKKQKIAEEITNVNQVSTEIQAYGDQVDTNTGLLMIEKSDTLEPSSSLLVNREGTSNVAGERRKRGRGKSLPAEANALSFVPVELGARRVNNHVHMAIGRFLFDIGATMDAVNSVYFQPMVDAIVSGGSGALMPSCNDLQGWILRKSVEEVKSENDKVMGAWVRTGCSILVNQWNTQTGQILLNFLVYCPEGTVFLKSIDASSVINSSDALYELLKQVVEEVGSKHVLQVITNGEEQYIVAGRRLAETFPTLYWTPCAAHCVDLILEDFAKLEWINAIIEQARSITKFIYNHSVVLNMVRRYTFGNDIVEPAATRSATNFTTLTRMVDLKNNLQAMVTSQQWVDCPYSKKPGGLAMLDLVSNQSFWSSCILIVRLTNPLLRVLRMVGSKKRPAMGYVYAGMYRAKETIKKELVKRNEYMVYWNIIDHWWEQQWHHPLHAAGFYLNPRFFYSMEGDMPNEMLSGMLDCIEKLIPDVTVQDKISKEINSYKNSVGDFGRKMAVRARDTLLPVEWWSTYGGSCPNLARLAIRVLSQTCSTLGLKHNHIPFEKLYETRNCLEQQRLRDLIFVQCNLQLRQIGYESKQHDSMQPLSSESASIVEDWVTGIDAFLDDDTYPDWTTLETLSVNTMLLRPGDEVEELGAGFNDHEIFNRMKEGDNENAEDNVVS	zf-BED--DUF-domain--Dimer_Tnp_hAT--	I
Ghe13G09140	Ghe Zf-BED 33_I	MSTEPTSIKGSVTPPTSIDSENSGVGASSQANMTIGKRKATPQRSEVWSHFTKIINSEGASKAKCNYCQKEFCCDVKRNGTGSLKYHIGACKKNPSNVIDASQGQLVLPRNGVEGGEGHLSTWRFDQEACRKGLAQMIKFMFVACPRFHIPSRTTMTRDVYQLYLDERVKIKQLLKSSCSRVCLTTDTWTSLQRVNYLCITAHFIDNDWKLNKKILKFCPISSHKVESIGMVIEKCLLNWGIDKLFTITVDNASSNDVAIGYLRKKFNPRGGLGAVRYVRQSPARLQKFKECVVVEKIECKKMLCLDVCTRWNSTYLMLDTEQDTNFRAELERGEGWPSVDDWANVRDLRDFLEHFYEVTLRISGTSYVTSNNFFDELSEIDILLRDAQLNSNVDFNVMAIKMKEKYDKYWGDIDKMNLLMFVACVLDPRQKLKYLEFALSEMSSSEKAFEMMQKLKESLYELFDEYKPSLHSTCSQSSVPTHVSLGEPQQKMKRRMQGLYKKRELEICGEDKTSELDKYLAEANEEFVEDFDILLWWKVNSPRFPTLSKMARDVLAIPVSTVASESTFSTGGRVLDQYRSSLTPKIVQALVCTQDWIRKSSLQEDIKEIEEQIQELDKIENGIFINTYII	zf-BED--DUF-domain--Dimer_Tnp_hAT--	I
Ghe13G14150	Ghe Zf-BED 34_I	MGLDKKRGPSNSPIHVDDGFNEYESAAKRQKSTTSKVWDEMTKLECENKNELKAQCNHCKTIFSAKSSSGTSHLRRHLNSCLKKVNKDISQYTIATQPSLGGVPFIKNYKFDADECRKAVSTFLECGKHSFRTVEEPGFRYMMSIASPNFKNISRYTAARDVLMYYAKERDRVKEELARALGLICLTSDNWNSEHTNDEYICITAHWVDENWKLQKRIIRFRALFPPYDGLNIADELVLCLSQWGIDKKIFSITLDNASYNDAMVSCLKNRFRANRAILCDGAFFQVRCCAHILNLIVKAGLELADDVVAKIRNGIKYIKKSGTRRKRFYDVADKSFHLNVTKKLRQDVCVRWNSTYLMLESSLYYKDVLDYWGQRDKDYQLFALSNEEWRNVAILCKFLKVFYDVTCVFSGSNYPMANLYFRGVWKVHKVLLDTVKGPYSFLTPMVKQMQEKFNKYWAEYSLILSCAAILDPRYKLNYVQYYFTTIYGIHASDFVETILSNLRLLFDEYVKKSKSTSSSLAGSSNVSDKNPVDSSLGEHNVNNSQLDIYLEEPELELNSQIDVLDYWSKSSVRYNELSLLARDLLAIPISTVASESAFSMGKKVITPLRSSLKPKTVQAVVCLDDWMRAKGFSTEIGCKNDEDDEDEDDEDDEDDVSSIAF	zf-BED--DUF-domain--Dimer_Tnp_hAT--	I
Ghe13G20100	Ghe Zf-BED 35_I	MSTEPTSIKGSVTPPTSIDSENSGVGASSQANVTTGKRKATPQRSEVWSHFTKIINSEGASKAKCNYCQKEFCCDVKKNGTGSLKYHIGSCKKNPSNVVDPSQGQLVLPRKGVEGGEGHLSTWRFDQEACRKGLAQMIVIDELPFKFVESEGFKNFMFVACPRFHIPSRTTMTRDVYQLYLDERVKIKQLLKSSCSRVCLTTDTWTSLQRVNYLCITAHFIDNDWKLNKKILNFCPISSHKGESIGMVIEKCLLNWGIDKLFTVTVTVDNASSNDVAIGYLRKKFNPRGGLVQNGKYLHMRCMAHIVNLIVVEGLKEMNKSV	zf-BED--DUF-domain--Dimer_Tnp_hAT--	I

		ERVRGAVRYVRQSPARLQKFKECVVMEKIECKKMLCLDVCTRWNSTYLMLDTAQKFERAFERFEEQDTNFRAELERGEWPSVDDWANVRNLRDFLEHFYEVTLRISGTSYVTSNNFFDELSEIDILLRDAQLNSNVDFNVMAIKMKEKYDKYWGDIDKMNLLMFVACVLDPRQKLKYLEFALSEMSSSEKACEMMQKLKESLYELFDEYKPPLYSTCSQSSVPTHVSLGEPQQKMKRRMQALYKKRELEIGGEDKTSELDKYLAKANEDFIEDFDILLWWKMNSPRFPILSKMARDVLAIPVSTVASESTFSTGGRVLDQYRSSLTPKIVQALVCTQDWIRKSSSQEDIKKIEEQIQELDMIENDVNAVKKFIREWEKGIWVS		
GheUnG14910	GheZf-BED36_I	MLRALAVSCFVLKIMVRGRDACWEHCVLVDATRQKVRCNYCHREFSGGVYRMKFHLAQIKNKDIVPCAEVPDDVRDHIQSILNTPKKQKTPKKPKMDKTVANGQQNSSSASGGLHPNHGSSGQHGSTCPSLLFPHPSPSEQPATDDAQKQKLDDADKKIAVFFFHNSIPFSAAKSMYYQEMVDAIAECGVGYKAPSYEKLRSSLLEKVKGDIHDGYKKYREEWKETGCTVLCNSWSDGRTKSFVIFSVTYPKGTLFLKSVDVSGHEDDASYLFELLESVVLEVGLENVIQVITDSTASYVCAGRHLMAKYSSLFWSPCASYCIDKMLEDISKQEWVGIVLEEAKTIARYIYSHAWILNMMRKFTGGRELMRPRITRFVDNYLNLRSIVFQEDNLKHMFSHSEWLSSIYSRRSDAQAIKSLLYLERFWKSAREAVSVSESLVKILRIVDGDMPAMGYIYEGIERAKGAIKAYYKGIEEKYMPIWDIIDRRWNMQLHSPLHAAAAFLNPSIFYNPNFKIDLRMRNGFQEAMLKMATMDKDKIEITKEHPVYINAQGALGTDFAIMGRTLNAPGDWWASYGYEIPTLQRVAIRILSQPCSFHWCRWNWSTFETGRDGKCKPIIFDEIDVSSEWPTESEPSVPLLDDSWLDNLPLECRGSP	zf-BED--DUF-domain--Dimer_Tnp_hAT--	I
Ghir_A02G000740.1	GhiZf-BED01_I	MDNFDQKLGPEFFKNLSAEAVTPLNVVHEEIYESSSKRPKTTSKVWDIFEKLPAQQGDSKAICKLCRRIYTAKTTSGTSHLRRHIEACVKRGNHEVDQRSIEACFKPVKRNANRLTLSHDTLISATTSLKNYKLDVDEIHRAIAMMIIVDEQPFSVVEDAGFRRLLSAACPEFPVLSRSSIKRDIISIYVKERENIRELLATCPGRICLTSSTWKSDSDDHFNCVTTHFIDHEWRLQKRILRFKLMPPPYDSLSVADEIALCMVQWNIEHKVFSVTLENLSSDDCVADMLRSRLAAKKYLPCKGVFFHVSCFFRILNSIVQAGLNLVVDIIAKLSLGIKYVQQSPHRKKNFYIVAKTLNLDTQRKLCLDTPARWNSTYDMIEVAFCYKNAFVYLAEQDKNFLHKLSEDEWEKMSVLYKFLKVFYEVTCVFFRNRQPTSNLYFKAAWKVHSRLFDMVRGPENFMTRMVREMHSKLNHYWSAYNLILSCAAILDPRYKIKFVEYCYTKLYGSGAQKYVSVSVNTLYGLFDEYMQNSARPSQTTLLSTAASKISNDKDENDGFEDYETFQSARFRTQVEKSQLDLYLEEPSHDLNSEIDVLEYWTLCSLRYPELSKMARDVLTIPVSTIASDSAFDITPQVISADRSSLKPKMLQALVSLQDWMLASDRTRGLGSMESKPEDDSSSSSDGDDDY	zf-BED--DUF-domain--Dimer_Tnp_hAT--	I
Ghir_A02G000740.2	GhiZf-BED02_I	MDNFDQKLGPEFFKNLSAEAVTPLNVVHEEIYESSSKRPKTTSKVWDIFEKLPAQQGDSKAICKLCRRIYTAKTTSGTSHLRRHIEACVKRGNHEVDQRSIEACFKPVKRNANRLTLSHDTLISATTSLKNYKLDVDEIHRAIAMMIIVDEQPFSVVEDAGFRRLLSAACPEFPVLSRSSIKRDIISIYVKERENIRELLATCPGRICLTSSTWKSDSDDHFNCVTTHFIDHEWRLQKRILRFKLMPPPYDSLSVADEIALCMVQWNIEHKVFSVTLENLSSDDCVADMLRSRLAAKKYLPCKGVFFHVSCFFRILNSIVQAGLNLVVDIIAKLSLGIKYVQQSPHRKKNFYIVAKTLNLDTQRKLCLDTPARWNSTYDMIEVAFCYKNAFVYLAEQDKNFLHKLSEDEWEKMSVLYKFLKVFYEVTCVFFRNRQPTSNLYFKAAWKVHSRLFDMVRGPENFMTRMVREMHSKLNHYWSAYNLILSCAAILDPRYKIKFVEYCYTKLYGSGAQKYVSVSVNTLYGLFDEYMQNSARPSQTTLLSTAASKISNDKDENDGFEDYETFQSARFRTQVEKSQLDLYLEEPSHDLNSEIDVLEYWTLCSLRYPELSKMARDVLTIPVSTIASDSAFDITPQVISADRSSLKPKMLQALVSLQDWMLASDRTRGLGSMESKPEDDSSSSSDGDDDY	zf-BED--DUF-domain--Dimer_Tnp_hAT--	I
Ghir_A02G000740.3	GhiZf-BED03_I	MDNFDQKLGPEFFKNLSAEAVTPLNVVHEEIYESSSKRPKTTSKVWDIFEKLPAQQGDSKAICKLCRRIYTAKTTSGTSHLRRHIEACVKRGNHEVDQRSIEACFKPVKRNANRLTLSHDTLISATTSLKNYKLDVDEIHRAIAMMIIVDEQPFSVVEDAGFRRLLSAACPEFPVLSRSSIKRDIISIYVKERENIRELLATCPGRICLTSSTWKSDSDDHFNCVTTHFIDHEWRLQKRILRFKLMPPPYDSLSVADEIALCMVQWNIEHKVFSVTLENLSSDDCVADMLRSRLAAKKYLPCKGVFFHVSCFFRILNSIVQAGLNLVVDIIAKLSLGIKYVQQSPHRKKNFYIVAKTLNLDTQRKLCLDTPARWNSTYDMIEVAFCYKNAFVYLAEQDKNFLHKLSEDEWEKMSVLYKFLKVFYEVTCVFFRNRQPTSNLYFKAAWKVHSRLFDMVRGPENFMTRMVREMHSKLNHYWSAYNLILSCAAILDPRYKIKFVEYCYTKLYGSGAQKYVSVSVNTLYGLFDEYMQNSARPSQTTLLSTAASKISNDKDENDGFEDYETFQSARFRTQVEKSQLDLYLEEPSHDLNSEIDVLEYWTLCSLRYPELSKMARDVLTIPVSTIASDSAFDITPQVISADRSSLKPKMLQALVSLQDWMLASDRTSKSPYLIAYGNCCYTMHNCFV	zf-BED--DUF-domain--Dimer_Tnp_hAT--	I
Ghir_A02G000740.4	GhiZf-BED04_I	MDNFDQKLGPEFFKNLSAEAVTPLNVVHEEIYESSSKRPKTTSKVWDIFEKLPAQQGDSKAICKLCRRIYTAKTTSGTSHLRRHIEACVKRGNHEVDQRSIEACFKPVKRNANRLTLSHDTLISATTSLKNYKLDVDEIHRAIAMMIIVDEQPFSVVEDAGFRRLLSAACPEFPVLSRSSIKRDIISIYVKERENIRELLATCPGRICLTSSTWKSDSDDHFNCVTTHFIDHEWRLQKRILRFKLMPPPYDSLSVADEIALCMVQWNIEHKVFSVTLENLSSDDCVADMLRSRLAAKKYLPCKGVFFHVSCFFRILNSIVQAGLNLVVDIIAKLSLGIKYVQQSPHRKKNFYIVAKTLNLDTQRKLCLDTPARWNSTYDMIEVAFCYKNAFVYLAEQDKNFLHKLSEDEWEKMSVLYKFLKVFYEVTCVFFRNRQPTSNLYFKAAWKVHSRLFDMVRGPENFMTRMVREMHSKLNHYWSAYNLILSCAAILDPRYKIKFVEYCYTKLYGSGAQKYVSVSVNTLYGLFDEYMQNSARPSQTTLLSTAASKISNDKDENDGFEDYETFQSARFRTQVEKSQLDLYLEEPSHDLNSEIDVLEYWTLCSLRYPELSKMARDVLTIPVSTIASDSAFDITPQVISADRSSLKPKMLQALVSLQDWMLASDRTRGLGSMESKPEDDSSSSSDGDDDY	zf-BED--DUF-domain--Dimer_Tnp_hAT--	I
Ghir_A02G001270.1	GhiZf-BED05_VIII	MASSSSFSSADAALEILVSCAHAIEDGNLKTADSFLHQIWNTAAVELDLISKLVRYFAEALVRRAYGLHPPYYTHSNLQIPHPLYYYYYYSRFDINEMVGEAIESATTGKKGFHLIDFHIPHLYGRGYLFKTLPNRSSDPLSVRITVVLPTFLKNTVDFQEEMEYLTEAGKLLKIELKKEDLRVVYANSLGEVDESTLDLRRTNDDEALVVYYNFKFHTLLAEAEAMKKELIKLRQINPEIVIMQEQYANDNDGNFIKRLEYSFRYYSNFFQYYSNLFKSGKPLGDNTAKYYMRQIHNIVACEGRDRIMRHQSLDEWRDLLLTAGFLQIPFQKDVENLHALYWVEEIKEEKGCLVLSHKDCPILFVSCWRPRAGEEHFKFNLNSNKFGQGFNPRPFQPFPEGFILNRLATFAEIYDMLEDVCFRYELPVALTWACEATTDKIMLDGKKHTLFMERTSCYASNEGSQCFMEACAKHHIQEGQAIAGKAFQSSANFHFEPSITKLMKSDYPLFNAAQLFGSHAVVAICLQNHYIIGDVYVVEFYWPEIESEKSESLALDIFNDLKNMKKKFVTIRVGGNEVGFEREAISTTLQGTMHMRNAQPASSTNDLLSSNTTWSLNAVQPCDVHEMERHGLVEQVESAPFSTPNPMSHGGVLQTQGPHKQEIGEKDFISQTVSIGDYEIVKASMETCKVPRTKRRKYSSKVWLDFDKFEVNGKQVAKCKHCNKDFTGSSKSGTTHLKNHLERCQSKKIKNQERQLITSEIGDLITRDSDESNFTFDQERSRLDFAKMIIKHQSPLDMAEQEFFKIFVKNLQPMFEFQSKDILLSDIHRIYKEETEKLQLYFDHLACNFNLTISLCKNNHGKTAYCCLIAHFIDDNWEPRMKIIACKPLEHIYDTKALNEIIQSSVLEWNISKKVFSITMDNPYLNDDMFQKIKETCFSDQGSFPSTHWFIGCTFIEDGFREMDLILLKLRKSIEYVSEIAEGKLKFEEVVNQVKLQGGKSWDDLSLRLDSDFGVLHSALESREIFCQLEKIDGNFKLNPSVEEWEMVLAFHSCLKCFDDIEGTQSLTANLYFPKLCNICKKFLHLEKSNYPIVTLMKRKFDYYWSLCNSAFAVATILDPRLKFKFVEFSYTEIYGHDSKMHLNRFHKVLTDVYYEYANEARNLSKSTSDLDDSNYSTTEIVNDCILESFSKFASANNFNEVASWKSELDCYLDEPLLPLDGAFDLLYWWCINNKRFPTLAKMARDFLAMPIPILAPCLNFNAMITNPTYNNLNTESMEALVCSQNWLKIPKESKSNF	GRAS--zf-BED--DUF-domain--Dimer_Tnp_hAT--	VIII
Ghir_A02G014680.1	GhiZf-BED06_V	MSTKPTSIEGSVTPPTSIDSENSGVGASIQTKGTTGKRKAPPQRSEVWSHFTKFINSEGASKAKCNYCEKEFCCDMKKNGTGSLKYHIGSCKKNPSNVVDTRNISTWRFDQEACRKGLAQMILIDELPFKFVESEGFKKFMFVACPRFHIPSRTTMTRDVYQLYLDERVKIKQLLRSSCSRVCLTTDTWTSLQRVNYLCLTAHFIDNDWKLNKKILNFCPISSHKGESIGMVIEKCLLNWGIDKLFTVTVDNASSNDVAIGYLRKKFNPRGGLVQNGKYLHMRCMAHIVNLIVVEGLKEMNKSVERVRGLLQKFKECIVVEKIECKKMLCLDVCTRWNSTYLMLDTAQNFERAFERFEEQDTNFRAELERERVGLVEMSSSEKACEMMQKLKESLYELFDEYKPPLHSTCSQSSVSTHVSIGEPQQKMKRRMQALYKKRELEICGEDKTSELDKYLAEANEEFVEDFDILLWWKVNSPRFPTLSKIARDVLAIPVSTVASESAFSTGGRVLDQYRSSLTPKIVQALVCTQDWIRRSSSQEDIKKIEEQIQELDKIENGMFIVLF	zf-BED--Dimer_Tnp_hAT--	V
Ghir_A03G001860.1	GhiZf-BED07_I	MVRGRDACWEHCVLVDATRQKVRCNYCHREFSGGVYRMKFHLAQIKNKDIVPCAEVPDDVRDHIQSILNTPKKQKTPKKPKMDKTVANGQQNSSSASGGLHPNHGSSGQHGSTCPSFLFPHPSPSEQPATDDAQKQKLDDADKKIAVFFFHNSIPFSAAKSMYYQEMVDAIAECGVGYKAPSYEKLRSSLLEKVKGDIHDGYKKYREEWKETGCTVLCNSWSDGRTKSFVIFSVTYPKGTLFLKSVDVSGHEDDASYLFELLESVVLEVGLENVIQVITDSTASYVCAGRHLMAKYSSLFWSPCASYCIDKMLEDISKQEWVGIVLEEAKTIARYIYSHAWILNMIRKFTGGRELMRPRITRFVDNYLNLRSIVFQEDNLKHMFSHSEWLSSIYSRRSDAQAIKSLLYLERFWKSAREAVSVSESLVKILRIVDGDMPAMGYIYEGIERAKGAIKAYYKGIEEKYMPIWDIIDRRWNMQLHSPLHAAAAFLNPSIFYNPNFKIDLRMRNGFQEAMLKMATMDKDKIEITKEHPVYINAQGALGTDFAIMGRTLNAPGDWWASYGYEIPTLQRVAIRILSQPCSFHWCRWNWSTFETVHTKKRNKVEMEKLNDLVFVHCNLWLQTICQGRDGKCKPIIFDEIDVSSEWPTESEPSVPLLDDSWLDNLPLECRGSP	zf-BED--DUF-domain--Dimer_Tnp_hAT--	I
Ghir_A03G001860.2	GhiZf-BED08_I	MVRGRDACWEHCVLVDATRQKVRCNYCHREFSGGVYRMKFHLAQIKNKDIVPCAEVPDDVRDHIQSILNTPKKQKTPKKPKMDKTVANGQQNSSSASGGLHPNHGSSGQHGSTCPSFLFPHPSPSEQPATDDAQKQKLDDADKKIAVFFFHNSIPFSAAKSMYYQEMVDAIAECGVGYKAPSYEKLRSSLLEKVKGDIHDGYKKYREEWKETGCTVLCNSWSDGRTKSFVIFSVTYPKGTLFLKSVDVSGHEDDASYLFELLESVVLEVGLENVIQVITDSTASYVCAGRHLMAKYSSLFWSPCASYCIDKMLEDISKQEWVGIVLEEAKTIARYIYSHAWILNMIRKFTGGRELMRPRITRFVDNYLNLRSIVFQEDNLKHMFSHSEWLSSIYSRRSDAQAIKSLLYLERFWKSAREAVSVSESLVKILRIVDGDMPAMGYIYEGIERAKGAIKAYYKGIEEKYMPIWDIIDRRWNMQLHSPLHAAAAFLNPSIFYNPNFKIDLRMRNGFQEAMLKMATMDKDKIEITKEHPVYINAQGALGTDFAIMGRTLNAPGDWWASYGYEIPTLQRVAIRILSQPCSFHWCRWNWSTFETVHTKKRNKVEMEKLNDLVFVHCNLWLQTICQGRDGKCKPIIFDEIDVSSEWPTESEPSVPLLDDSWLDNLPLECRGSP	zf-BED--DUF-domain--Dimer_Tnp_hAT--	I

Ghir_A05G030910.1	GhiZf-BED09_I	MPPREEFPTKGLEGAPSNDIGWHFGTLVPNARGSIVCKLCGKVVKGGITRLKEHIAHKTGNVAPCPNVTGVIRESMMNVLKESNTKKIDKKRRKDEFLSQLIEEEDEHEGFIDEVSAIRQATRESIQSQHEWHRREEFRRSTGGWDNIYEEGRSSHGSAREHNRERTSKSILGESEFTLRGAIPELVRSKSSKQPKVNDSFLKSFRRKIGEAVSKFIIYERLPFQLASSPWLYNLIQLPTPYEVSDVYLESEYQRVHDWVNVLKTHWKELGATLMCDGWTNSLNQMHIINFLVYCSKGTIFWKSVDVSSVRSRDAEFYYRLLDSVVEEIGENYIVQIVTDNEAAMKAAGKKLMLKRQHLYWTSCAAHCLDLCLEDIGKKPSVAKVLDEVKKVTCFIYNHIWTVDLMKKYTQGKQILRPALTRFATHFIQLEEITRQKQGLREMFNSKEFKESKWGKQKSGPAYEAKKIVLGKDFWKKANDLIKVYEPLVRVLRLVDSDEKPTMGFIYEAVDRAKRAIQQNCRYFTEYEKIIDNRWNFMHSDLHSAGYFLNPQFQFGVEHSENVLIETLEGTRSVIERLEPSMDTQVRMVNQWWMIYGTCVPELQKLAIKVLSQTTSASNCERNWSTFSYIHTKARNRLKYKKLEKLVFTYYNMRLKMRHQQRMSTDDINASFNPISLDYIFEDVDPLSEWLHEKENPLLDGENAGVLPVDTSDDEMDVDQSQQQILSHSSSSSTPSQSGDGPDGGGLSPIDEDDGYSGDRGEIRSSSQYGGEYGVVPLVDIFVTDQSLMEICFLNLGEIEVNLELHQREKARSILL	zf-BED--DUF-domain--Dimer_Tnp_hAT--	I
Ghir_A05G039010.1	GhiZf-BED10_I	MDMSDAVIVNSSRLKSIVWNDFDRVKKGDTFVAICRHCKKKLSGSSTSGTSHLRNHLIRCQRRSNHGVAQYFSAKDKKKEGSLALVTIDQEQKNDEVLSIVNLRYEQEQIKSEHVGIGSNSLDQRRSQFDLARMIILHNYPLAMVEHVGFKIFVRNLQPLFELATRNKVEADCMEIYAKEKQKVYEIFDKLPGKISVSADVWTASEDDAAYLSLAAHYIDENWQLKKKNLNFVTIDPSYTEDMHSEVIMNCLMDWDIDRKLFSMIFDSFTSDNIVERIRDRLSQNRFLYCNGQLFDVRCAVDLLNRMAHDALEALCEITQKIRESIRYVKSSEATQATFNELADEVQVETKKCLCIDNPLKWNSTYFMLEAALEYRKVFSCLRDRDPVNMKFLLSDPEWDRLITVTSFLKLFVEVTNVFTRSKYPTANIFFPEICDIHLQLIEWCKNPDEYISSLALKMRKKFEEYWYKCSSGLAVAAMLDPRFKMKLLEYYYPQLYGDSATELIDDVFECIKSLYNEHSIVSPLASSIDQGLDWQASGISGSGKDSRDRLMGFDKYLHETCQAEGSSSDLDKYLEEPLFPRNVDFNVLNWWKVHTPRYPILSMMARNILGIPISKVAAESRFDTGGRVLDHNWSSLPPTTIQALMCSQDWIRSGLES	zf-BED--DUF-domain--Dimer_Tnp_hAT--	I
Ghir_A05G039010.2	GhiZf-BED11_I	MDMSDAVIVNSSRLKSIVWNDFDRVKKGDTFVAICRHCKKKLSGSSTSGTSHLRNHLIRCQRRSNHGVAQYFSAKDKKKEGSLALVTIDQEQKNDEVLSIVNLRYEQEQIKSEHVGIGSNSLDQRRSQFDLARMIILHNYPLAMVEHVGFKIFVRNLQPLFELATRNKVEADCMEIYAKEKQKVYEIFDKLPGKISVSADVWTASEDDAAYLSLAAHYIDENWQLKKKNLNFVTIDPSYTEDMHSEVIMNCLMDWDIDRKLFSMIFDSFTSDNIVERIRDRLSQNRFLYCNGQLFDVRCAVDLLNRMAHDALEALCEITQKIRESIRYVKSSEATQATFNELADEVQVETKKCLCIDNPLKWNSTYFMLEAALEYRKVFSCLRDRDPVNMKFLLSDPEWDRLITVTSFLKLFVEVTNVFTRSKYPTANIFFPEICDIHLQLIEWCKNPDEYISSLALKMRKKFEEYWYKCSSGLAVAAMLDPRFKMKLLEYYYPQLYGDSATELIDDVFECIKSLYNEHSIVSPLASSIDQGLDWQASGISGSGKDSRDRLMGFDKYLHETCQAEGSSSDLDKYLEEPLFPRNVDFNVLNWWKVHTPRYPILSMMARNILGIPISKVAAESRFDTGGRVLDHNWSSLPPTTIQALMCSQDWIRSGLES	zf-BED--DUF-domain--Dimer_Tnp_hAT--	I
Ghir_A05G039010.3	GhiZf-BED12_I	MDMSDAVIVNSSRLKSIVWNDFDRVKKGDTFVAICRHCKKKLSGSSTSGTSHLRNHLIRCQRRSNHGVAQYFSAKDKKKEGSLALVTIDQEQKNDEVLSIVNLRYEQEQIKSEHVGIGSNSLDQRRSQFDLARMIILHNYPLAMVEHVGFKIFVRNLQPLFELATRNKVEADCMEIYAKEKQKVYEIFDKLPGKISVSADVWTASEDDAAYLSLAAHYIDENWQLKKKNLNFVTIDPSYTEDMHSEVIMNCLMDWDIDRKLFSMIFDSFTSDNIVERIRDRLSQNRFLYCNGQLFDVRCAVDLLNRMAHDALEALCEITQKIRESIRYVKSSEATQATFNELADEVQVETKKCLCIDNPLKWNSTYFMLEAALEYRKVFSCLRDRDPVNMKFLLSDPEWDRLITVTSFLKLFVEVTNVFTRSKYPTANIFFPEICDIHLQLIEWCKNPDEYISSLALKMRKKFEEYWYKCSSGLAVAAMLDPRFKMKLLEYYYPQLYGDSATELIDDVFECIKSLYNEHSIVSPLASSIDQGLDWQASGISGSGKDSRDRLMGFDKYLHETCQAEGSSSDLDKYLEEPLFPRNVDFNVLNWWKVHTPRYPILSMMARNILGIPISKVAAESRFDTGGRVLDHNWSSLPPTTIQALMCSQDWIRSGLES	zf-BED--DUF-domain--Dimer_Tnp_hAT--	I
Ghir_A06G005650.1	GhiZf-BED13_I	METIPGESNNQLALTTPEAQPIKRRKKKSMVWEYFTIENVSAGCRRAYCKRCKQSFAYSTGSKVAGTSHLKRHIAKGTCRALLRGQGQDNNQFITPYNPKMGGSEPPKRRYRSPSSPFIPFDQDRCRHEIARMIIMHEYPLHIVEHPGFIAFVQSLQPQFDKMSFNTVQGDCVATYLREKQSLMKFIEGIPGRFCLTLDMWSSNQTLGYVFITGHFVDSDWKLHRWVFNVVMEPYPDSHSALSHAIAACLSDWSLEGKLFSLTFNHPLSEAGLENLRPLLCVKNPLILNGQLLIRNCIARTMSSMAKDVLGAGQEIIKKIRDSVKYVKMSESHDDKFIQVKNQLQVPSEKSLFLDNQTRWNTTYQMLAAASELKEVPSMEDWKLAETLCSFLKPLFDAASILTTTTLPTVITFFYEVWKIHVDLGRSITSEDPFISNLAKSMQEKIDKYWKDCSLVLAMAVVMDPRFKMKLVEFSFTKIYSEDAPTYIKTVDDGIHELFLEYVALPLPLTPTYAEEVNGANNGKTNESHYGNLLSDHGLTDFDVYIMETNSQQMKSELDQYLEESLLPRVQEFDVLGWWKLNKMKYPTLSKMARDILSIPQLDEYRSSLRPETVEALICAKDWLHFGSSDVSNALVKME	zf-BED--DUF-domain--Dimer_Tnp_hAT--	I
Ghir_A06G005660.1	GhiZf-BED14_I	MDYYILYSHCLFQLKLSLGLQRRFLGFNSDPRLLLFGFLTAGTDDPEAHPIKRRKKKSMVWEYFTIENVSAGCRRAYCKRCKQSFAYSTGSKVAGTSHLKRHIAKGTCRALLRGQGQDNNQFITPYNPKMGGSEPPKRRYRSPSSPFIPFDQDRCRHEIARMIIMHEYPLSHTQFDKMSFNTVQGDCVATYLREKQSLMKFIEGIPGRFCLTLDMWSSNQTLGYVFITGHFVDSDWKLHRQVFNVVMEPYPDSHSALSHAIAACLSDWSLEGKLFSLTFNHPLSEAGLENLRPLLCKLYSSYYEQHMAKDVLGAGQEIIKKIRDSVKYVKMSESHDDKFIQVKNQLQVPSEKSLFLDNQTRWNTTYQMLAAASELKEVFDCLDTYDPDYKLAPSMEDWKLAETLCSFLKPLFDAASILTTTTLPTIITFFLKCGKFMWTWVQPCKINARKIDKYWKDCSLVLAMAVVMDPRFKMKLVEFSFTKIYSEDAPTYIKTVDDGIHELFLEYVALPLPLTPTYAEEVNGANNGKTNESHYGNLLSDHGLTDFDVYIMETNSQQMKSELDQYLEESLLPRVQEFDVVGWWKLNKMKYPTLSKMARDILSIPVSAAAPESIFDITDKQLDEYRSSLRPETVEALICAKDWLHFGSSDVSNALVKMEF	zf-BED--DUF-domain--Dimer_Tnp_hAT--	I
Ghir_A07G007310.1	GhiZf-BED15_I	MSTEPTFIEGSITPPTSIDSENSRIRASSQAKGTTGKRKVTSQRSEVWSHFTKIINSEGASKAKCNYCQKEFCCDMKKNGTRSLKYHIGSCKKNPSNVIDTRGRHLSTWRFDQEACRKGLTQMIVIDELPFKFVESEGFKKFMFVACPRFHIPSRTTMTRDVYQLYLNERVKIKQLLKISCSRVCLTTDTWISLQSVNYLCITAHFIDNNWKLNKKILNFCPISSYKGESIGMVIEKCLLNWGIDKLFTVTVDNASSNNVAIGYLRKKFNSRGGLVQNGRYLHMRCMAHIVNLIVGAVRYVRQSPARLQKFKECVVVEKIECKKMLFLDVCTRCNSTYLMLDTAQNFERAFKRFEEQDTNFRAELKRGEGWPSVDDWDNVRNLRDFLEHFYEVTLRISGTLYVTSNNFFDELSEIDILLRDVQLNSNVDFNVMTIKMKEKYDKYWGDIDKMNLLMFVACVLDPRKKLKYLEFALSEMSSSEKACEMMQKLKESLYELFDEYKPPLHSTCSQLSVPTHRELEICGEDKTSELDKYLAEANEEFVENFDILLWWKVNSPRFPTLSKMAKDVLAISVSTVASESAFSTGRRVLDQYRSSLTPKIVQALVCTQDWIRKSLSQEDIKKIEEQIQELDKIENGIFIDMLTFLFLLTYNLFVCLYYLIFFKCVYFYYMLNFFL	zf-BED--DUF-domain--Dimer_Tnp_hAT--	I
Ghir_A07G008970.1	GhiZf-BED16_I	MELNLVPISITRQKQDPAWNHCEVFKNGERIQIKCMYCGKLFKGGGIHRFKEHLAGRKGQGPICEQVPQGVRSIMQESLNGILVKQDKKQKLIPKLLACGSSSSNLNIGGEVENLGSHDDMNFGIKPISVLNTLEGDSNVVSKVGRGRKRGRGRDRNLIESNRPCLKTDLALVPNGGENPIHMAIGRFLYDIGVNLDAVNSVCFQPMIDAIASGGSGVVPPSCHDLRGWILKNVIEEVKDDIDRNKAMWGKTGCSIIVEQCRTKNGRVLLSFLVYCPQATVFMKSVDASHAIYSADYLFELLKQVIEEVGSENVVQVITNCEEPYLFTGKRLMESFPSLYWAPCLAHCVDLMLQDFSNLEWINETIEQAKSLTKFIYNQSSVLNTMRKFTSGNDIVEPALTCFATNFSTLRRMADLKLNLQAMVNSQDWLECPYAKEPGGQAMSDIVNNRSFWNSCVLIAHITYPLLRVLEIVGSKKRSAMGYVYAGIYRAKETIKKELVKQDDYMVYWNIIDNRWEQQRHLPLYAAGFFLNPKFFYNTKEHIHNDILSAVFDSIERLVPDTNIQDQVVREINLYKNAMGDLGRPMAVRARDNLLPGEWWSIYGGGCPNLQRLAIRILSQTCSSIGYKPNKISIEEIHNTRNFLERRRLSDLVFVQYNLYLRQMVLQNQEKDSLDPLVFNRKDILEDWIADNEVSPDNHESSDWKSLDPPVGNRTTLTPPGDEAEDFLSTRFMDLDIFNGLKGVKEEI	zf-BED--DUF-domain--Dimer_Tnp_hAT--	I
Ghir_A07G012610.1	GhiZf-BED17_II	MSTEPTSIKGSVTPPTLIDSENSGVGASSQANVTTGKRKATPQRSEVWSHFTKIINSEGASKAKCNYCQKEFCCDVKKNGTGSLKYHIDSCKKNPSNVVDSSQGQLVLPRKGVEGGEGHISTWRFDQEACRKGLAQMIVIDELPFKFVEIEGFMKFMFVACPRFHIPSQTTMTRDVYQLYLDERVKIKQLLRSSCSRVCLTTDTWTSLQRVNYLCITAHFIDNDWKLNKKILNFCPISSHKGESIGMVIEKCLLNWGIDKLFTVTVDNASSNDVAIGYLRKKFNPRRGLVQNGKYLHMRCMAHIVNLIVVEGLKEMNKSVERVRGLLDM	zf-BED	II
Ghir_A07G012610.2	GhiZf-BED18_II	MSTEPTSIKGSVTPPTLIDSENSGVGASSQANVTTGKRKATPQRSEVWSHFTKIINSEGASKAKCNYCQKEFCCDVKKNGTGSLKYHIDSCKKNPSNVVDSSQGQLVLPRKGVEGGEGHISTWRFDQEACRKGLAQMIVIDELPFKFVEIEGFMKFMFVACPRFHIPSQTTMTRDVYQLYLDERVKIKQLLRSSCSRVCLTTDTWTSLQRVNYLCITAHFIDNDWKLNKKILNFCPISSHKGESIGMVIEKCLLNWGIDKLFTVTVDNASSNDVAIGYLRKKFNPRRGLVQNGKYLHMRCMAHIVNLIVVEGLKEMNKSVERVRGLLDM	zf-BED	II
Ghir_A08G009690.1	GhiZf-BED19_I	MAEMTEATNMETSPVENNNELALITPETQPKRRKKKSMVWEYFTIETVSAGCRRACCNRCKQSFAYSTGSKVAGTSHLKRHIAKGTCPALLRDQYNNQLTPYSPKTGGGEPRKRRYRSPSSPFIPFDQDRCRHEIARMIIMYEYPLHMVEHPGFIAFVQNLQPRFDKVSFNTVQGDCVATYLREKQSLMKLIEGIPGRVCLTLDMWTSNQTLGYVFITGHFIDFEWKLQRRVLNVIMEPYPDSDSALSHAVAACLSDWSLEGKLFSLIFNHPTSEAGLENLRPLLCTKNPLILNGQLLLGNCIARNLSSMAKDVLGAGHEIVKKIRDSVKYVKTSESHDEKFVQVKNQLQVPSEKSLILDNQNQWNTTYQMLAAASELKEVFNCLDTSDPDYKLAPSMEDWKVAETLCTFLKPLFDAASILMTTTNPTAITFFHEAWKIHADLGRSIANDDPFISNIAKSMLEKIDKYWKDCSLILAIAVVMDPRFKMKLVEFSFTKIFGEDAPTYIKIVDDGIHELFLEYVALPLPLTPTYTEEGNVGNNGKTDESQQGNLLSDQGLTDFDVYIMET	zf-BED--DUF-domain--Dimer_Tnp_hAT--	I

		SSQQMKSELDQYLEESLLPRVQEFDVLGWWKLNKMKYPTLSKMARDILSIPVSAAAPDSVFDIIIKQLDEYRSSLRPETVE<br>ALICAKDWLHHGSEESNALVKMEF		
Ghir_A<br>08G00<br>9690.2	Ghi<br>Zf-<br>BED<br>20_I	MAEMTEATNMETSPVENNNELALITPETQPKRRKKKSMVWEYFTIETVSAGCRRACCNRCKQSFAYSTGSKVAGTSHLK<br>RHIAKGTCPALLRDQYNNQLTPYSPKTGGGEPRKRRYRSPSSPFIPFDQDRCRHEIARMIIMYEYPLHMVEHPGFIAFVQN<br>LQPRFDKVSFNTVQGDCVATYLREKQSLMKLIEGIPGRVCLTLDMWTSNQTLGYVFITGHFIDFEWKLQRRVLNVIMEPYP<br>DSDSALSHAVAACLSDWSLEGKLFSLIFNHPTSEAGLENLRPLLCTKNPLILNGQLLLGNCIARNLSSMAKDVLGAGHEIVK<br>KIRDSVKYVKTSESHDEKFVQVKNQLQVPSEKSLILDNQNQWNTTYQMLAAASELKEVFNCLDTSDPDYKLAPSMEDWK<br>VAETLCTFLKPLFDAASILMTTTNPTAITFFHEAWKIHADLGRSIANDDPFISNIAKSMLEKIDKYWKDCSLILAIAVVMDPRF<br>KMKLVEFSFTKIFGEDAPTYIKIVDDGIHELFLEYVALPLPLTPTYTEEGNVGNNGKTDESQQGNLLSDQGLTDFDVYIMET<br>SSQQMKSELDQYLEESLLPRVQEFDVLGWWKLNKMKYPTLSKMARDILSIPVSAAAPDSVFDIIIKQLDEYRSSLRPETVE<br>ALICAKDWLHHGSEESNALVKMEF	zf-BED--DUF-<br>domain--<br>Dimer_Tnp_hAT<br>--	I
Ghir_A<br>08G00<br>9690.3	Ghi<br>Zf-<br>BED<br>21_I	MAEMTEATNMETSPVENNNELALITPETQPKRRKKKSMVWEYFTIETVSAGCRRACCNRCKQSFAYSTGSKVAGTSHLK<br>RHIAKGTCPALLRDQYNNQLTPYSPKTGGGEPRKRRYRSPSSPFIPFDQDRCRHEIARMIIMYEYPLHMVEHPGFIAFVQN<br>LQPRFDKVSFNTVQGDCVATYLREKQSLMKLIEGIPGRVCLTLDMWTSNQTLGYVFITGHFIDFEWKLQRRVLNVIMEPYP<br>DSDSALSHAVAACLSDWSLEGKLFSLIFNHPTSEAGLENLRPLLCTKNPLILNGQLLLGNCIARNLSSMAKDVLGAGHEIVK<br>KIRDSVKYVKTSESHDEKFVQVKNQLQVPSEKSLILDNQNQWNTTYQMLAAASELKEVFNCLDTSDPDYKLAPSMEDWK<br>VAETLCTFLKPLFDAASILMTTTNPTAITFFHEAWKIHADLGRSIANDDPFISNIAKSMLEKIDKYWKDCSLILAIAVVMDPRF<br>KMKLVEFSFTKIFGEDAPTYIKIVDDGIHELFLEYVALPLPLTPTYTEEGNVGNNGKTDESQQGNLLSDQGLTDFDVYIMET<br>SSQQMKSELDQYLEESLLPRVQEFDVLGWWKLNKMKYPTLSKMARDILSIPVSAAAPDSVFDIIIKQLDEYRSSLRPETVE<br>ALICAKDWLHHGSEESNALVKMEF	zf-BED--DUF-<br>domain--<br>Dimer_Tnp_hAT<br>--	I
Ghir_A<br>08G00<br>9690.4	Ghi<br>Zf-<br>BED<br>22_I	MAEMTEATNMETSPVENNNELALITPETQPKRRKKKSMVWEYFTIETVSAGCRRACCNRCKQSFAYSTGSKVAGTSHLK<br>RHIAKGTCPALLRDQYNNQLTPYSPKTGGGEPRKRRYRSPSSPFIPFDQDRCRHEIARMIIMYEYPLHMVEHPGFIAFVQN<br>LQPRFDKVSFNTVQGDCVATYLREKQSLMKLIEGIPGRVCLTLDMWTSNQTLGYVFITGHFIDFEWKLQRRVLNVIMEPYP<br>DSDSALSHAVAACLSDWSLEGKLFSLIFNHPTSEAGLENLRPLLCTKNPLILNGQLLLGNCIARNLSSMAKDVLGAGHEIVK<br>KIRDSVKYVKTSESHDEKFVQVKNQLQVPSEKSLILDNQNQWNTTYQMLAAASELKEVFNCLDTSDPDYKLAPSMEDWK<br>VAETLCTFLKPLFDAASILMTTTNPTAITFFHEAWKIHADLGRSIANDDPFISNIAKSMLEKIDKYWKDCSLILAIAVVMDPRF<br>KMKLVEFSFTKIFGEDAPTYIKIVDDGIHELFLEYVALPLPLTPTYTEEGNVGNNGKTDESQQGNLLSDQGLTDFDVYIMET<br>SSQQMKSELDQYLEESLLPRVQEFDVLGWWKLNKMKYPTLSKMARDILSIPVSAAAPDSVFDIIIKQLDEYRSSLRPETVE<br>ALICAKDWLHHGSEESNALVKMEF	zf-BED--DUF-<br>domain--<br>Dimer_Tnp_hAT<br>--	I
Ghir_A<br>08G00<br>9690.5	Ghi<br>Zf-<br>BED<br>23_I	MAEMTEATNMETSPVENNNELALITPETQPKRRKKKSMVWEYFTIETVSAGCRRACCNRCKQSFAYSTGSKVAGTSHLK<br>RHIAKGTCPALLRDQYNNQLTPYSPKTGGGEPRKRRYRSPSSPFIPFDQDRCRHEIARMIIMYEYPLHMVEHPGFIAFVQN<br>LQPRFDKVSFNTVQGDCVATYLREKQSLMKLIEGIPGRVCLTLDMWTSNQTLGYVFITGHFIDFEWKLQRRVLNVIMEPYP<br>DSDSALSHAVAACLSDWSLEGKLFSLIFNHPTSEAGLENLRPLLCTKNPLILNGQLLLGNCIARNLSSMAKDVLGAGHEIVK<br>KIRDSVKYVKTSESHDEKFVQVKNQLQVPSEKSLILDNQNQWNTTYQMLAAASELKEVFNCLDTSDPDYKLAPSMEDWK<br>VAETLCTFLKPLFDAASILMTTTNPTAITFFHEAWKIHADLGRSIANDDPFISNIAKSMLEKIDKYWKDCSLILAIAVVMDPRF<br>KMKLVEFSFTKIFGEDAPTYIKIVDDGIHELFLEYVALPLPLTPTYTEEGNVGNNGKTDESQQGNLLSDQGLTDFDVYIMET<br>SSQQMKSELDQYLEESLLPRVQEFDVLGWWKLNKMKYPTLSKMARDILSIPVSAAAPDSVFDIIIKQLDEYRSSLRPETVE<br>ALICAKDWLHHGSEESNALVKMEF	zf-BED--DUF-<br>domain--<br>Dimer_Tnp_hAT<br>--	I
Ghir_A<br>08G02<br>4610.1	Ghi<br>Zf-<br>BED<br>24_I	MEVANETVIKKPKRLTSVVWNHFERVRKADLCYAVCVHCNKKLSGSSNSGTTHLRNHLMRCLKRFNYDVSQLLSAKKRK<br>KDNTLTIANISYDEGQRKEEYLKPTIVKYEPEQRKDEVFNVQSSWFDQERSRLDLARMIILHGYPLAMVEHVGFKVFVKNL<br>QPLFDVVPNSTVELSCMEIYGKERQKVHDMLSKLQGRINLAVEMWSSPENTNHVCMMAHYVGDDWKLQKKILNFVTLDS<br>SHTDDLLSGVIIKCLMDWDIGSKLFAMTLDDFSTNDDIVLRIKEQISENKSRLSNGQLLDVRSAVHVLNSIVQDAMEALRLVI<br>QKIRGTVRYVKSSQSIQGKFKEMVLQTGINSQKNLVLDCPIQWNSTYLMLETAIEYRNAFCQLPELDLDLALSDEEWEWA<br>SSITGYLKLFIEIINVFSSNKCPTANIYFPEICHVHIQLIDWCKSPDNFLSSLAAKMKAKFDKYWSKCSLSLAVAAILDPRFKM<br>KLVEYYYSQIYGSTALERIKEVSDGLKELFNTYSICSTLMDQGSALPLSSLPSSSNDGRDRLKGFDKFLHETSQSQNAISA<br>WKYLDEPVFPRNCNFNILNWWRVHTPRYPILSMMARDVLGTHVNRLTRVSIPRWR	zf-BED--DUF-<br>domain--<br>Dimer_Tnp_hAT<br>--	I
Ghir_A<br>08G02<br>4610.2	Ghi<br>Zf-<br>BED<br>25_I	MEVANETVIKKPKRLTSVVWNHFERVRKADLCYAVCVHCNKKLSGSSNSGTTHLRNHLMRCLKRFNYDVSQLLSAKKRK<br>KDNTLTIANISYDEGQRKEEYLKPTIVKYEPEQRKDEVFNVQSSWFDQERSRLDLARMIILHGYPLAMVEHVGFKVFVKNL<br>QPLFDVVPNSTVELSCMEIYGKERQKVHDMLSKLQGRINLAVEMWSSPENTNHVCMMAHYVGDDWKLQKKILNFVTLDS<br>SHTDDLLSGVIIKCLMDWDIGSKLFAMTLDDFSTNDDIVLRIKEQISENKSRLSNGQLLDVRSAVHVLNSIVQDAMEALRLVI<br>QKIRGTVRYVKSSQSIQGKFKEMVLQTGINSQKNLVLDCPIQWNSTYLMLETAIEYRNAFCQLPELDLDLALSDEEWEWA<br>SSITGYLKLFIEIINVFSSNKCPTANIYFPEICHVHIQLIDWCKSPDNFLSSLAAKMKAKFDKYWSKCSLSLAVAAILDPRFKM<br>KLVEYYYSQIYGSTALERIKEVSDGLKELFNTYSICSTLMDQGSALPLSSLPSSSNDGRDRLKGFDKFLHETSQSQNAISA<br>WKYLDEPVFPRNCNFNILNWWRVHTPRYPILSMMARDVLGTHVNRLTRVSIPRWR	zf-BED--DUF-<br>domain--<br>Dimer_Tnp_hAT<br>--	I
Ghir_A<br>11G00<br>0730.1	Ghi<br>Zf-<br>BED<br>26_I	MTFGLHLLQIVSFVGNKMEWSVNNAFKSYKDMEPKSTMDMVLIPNMDTIDIVLGSSEKGNVVPSAKPRKKTMTSVYLKYF<br>ETAPDGKTRRCKFCGQSYSIATATGNLGRHLSNRHPGYDKTGENVSSSAPQPSTTPTVIKKPQPQGRAPQVDYDHLNW<br>LLIKWLILATLPPSTLEEKWLANSFKFLNPSIQLWPGEKYKAVFCEVFRSMREDVRASLEQVSSKVSIALDFWSSYEQIFYM<br>SITCQWIDENWSFQKVLLDICQVPYPCTGSEIYNSLVKVLKMYNIENKVLSCTHDNSQNAIHACHALKEDLDGQKMGPFCF<br>IPCAARTLSLIIDDALRTTKPVIAKVREFVQELNASLDISEDFIQLATAYKEGSWQFPLDASARWSGSYQMLDIVQKAGKSM<br>DAVVRKNEEMLGNRMLLNTAEKNVVNIVHNYLEPFYKVISEIGVNTPPTIGMVIVYMDHISDTITTQQPPDWLKNPAEDMA<br>KKLRSYNNQVCNIFIYMTAILDPRIKCELIPESLNSENYLEEARAHFVRNYYTTPFSSMTSGYSSQDIEDGGAVSFAEEIARK<br>KRRASMSNATDELTQYLSESPAPTKTDVLEWWKVNSTRYPRLSAMARDFLAVQATSVKPDELFCSKGDEIDKQRFCMP<br>HDSTQAILCIKSWTQGGLKLKYKSTEIDYERLMEMAAAAAADISLAGMDKKQK	zf-BED--DUF-<br>domain--<br>Dimer_Tnp_hAT<br>--	I
Ghir_A<br>11G02<br>3640.1	Ghi<br>Zf-<br>BED<br>27_I<br>I	MPCSRNKEEVAPSDDYGWRWGELVEGNHNNVKCRFCGRIIKRGITQLKEYLAVKKGNVAPCPHGSVKVRKSIGQQLQE<br>YHIEKAVRQRRKEELEERISLGDRGNYGDSGDDDEELTITRRKSVRSQVE	zf-BED	II
Ghir_A<br>11G03<br>1630.1	Ghi<br>Zf-<br>BED<br>28_I	MEVANESTAKKPKRLTSVVWNHFERVKKANICYAVCVHCNKKLSGSSNSGTTHLRNHLMRCLKRSNYDVSQLLAVKRRK<br>KENTLTIANISYDEGQRKEDYMKPTIVKYEQDQRKDEAFNLGSSWFDPERSRLDLARMIILHGYPLAMVEQVGFKVFVKN<br>MQPLFDVVHNSTIELSCVEIYMKEKQRIYDILSKLQGRINLAIEMWSSPENSKYVCLTAHYVDDEWKLQKKILNFLTLDSSH<br>TEDMLSDVIIKCLMDWDIDCKLFAMTFDDCSTNDDIVSRIKDQVSESRPRLSNGQLLDVRSAAHVLNSIAQDAIEALQVVIQ<br>KIRGSVKYVKSSQSILGKFNEIAQQQGIDNHKIVVLDYPIRWNSTYMMLETAVEYRNVFHHLPELDPDFALSDEEWERASS<br>IVSYLKLLIEIINVFSSNKCPTANIYFPEICHVHIQLIEWCKSSDAFLSSLATKMKAKFDKYWSKCSLALAVAAILDPRFKMKLV<br>EYYYSQIYGSTALERIKEASDGIKELFNAYSICSTLIDQGSALPGSSLPSSSNDTRDRLKGFDKFLHETSQSQTAISDLEKYL<br>DEPVFPCNCDFNILNWWRVHTPRYPILSMMARDVLGTPMSTVAQEFAFNAGGRMLDSNQSSPPPDTQQALICTRDWLR<br>TQSDDATPSSSHYALPLYVEAN	zf-BED--DUF-<br>domain--<br>Dimer_Tnp_hAT<br>--	I
Ghir_A<br>11G03<br>1630.2	Ghi<br>Zf-<br>BED<br>29_I	MEVANESTAKKPKRLTSVVWNHFERVKKANICYAVCVHCNKKLSGSSNSGTTHLRNHLMRCLKRSNYDVSQLLAVKRRK<br>KENTLTIANISYDEGQRKEDYMKPTIVKYEQDQRKDEAFNLGSSWFDPERSRLDLARMIILHGYPLAMVEQVGFKVFVKN<br>MQPLFDVVHNSTIELSCVEIYMKEKQRIYDILSKLQGRINLAIEMWSSPENSKYVCLTAHYVDDEWKLQKKILNFLTLDSSH<br>TEDMLSDVIIKCLMDWDIDCKLFAMTFDDCSTNDDIVSRIKDQVSESRPRLSNGQLLDVRSAAHVLNSIAQDAIEALQVVIQ<br>KIRGSVKYVKSSQSILGKFNEIAQQQGIDNHKIVVLDYPIRWNSTYMMLETAVEYRNVFHHLPELDPDFALSDEEWERASS<br>IVSYLKLLIEIINVFSSNKCPTANIYFPEICHVHIQLIEWCKSSDAFLSSLATKMKAKFDKYWSKCSLALAVAAILDPRFKMKLV<br>EYYYSQIYGSTALERIKEASDGIKELFNAYSICSTLIDQGSALPGSSLPSSSNDTRDRLKGFDKFLHETSQSQTAISDLEKYL<br>DEPVFPCNCDFNILNWWRVHTPRYPILSMMARDVLGTPMSTVAQEFAFNAGGRMLDSNQSSPPPDTQQALICTRDWLR<br>TQSDDATPSSSHYALPLYVEAN	zf-BED--DUF-<br>domain--<br>Dimer_Tnp_hAT<br>--	I
Ghir_A<br>11G03<br>1630.3	Ghi<br>Zf-<br>BED<br>30_I	MEVANESTAKKPKRLTSVVWNHFERVKKANICYAVCVHCNKKLSGSSNSGTTHLRNHLMRCLKRSNYDVSQLLAVKRRK<br>KENTLTIANISYDEGQRKEDYMKPTIVKYEQDQRKDEAFNLGSSWFDPERSRLDLARMIILHGYPLAMVEQVGFKVFVKN<br>MQPLFDVVHNSTIELSCVEIYMKEKQRIYDILSKLQGRINLAIEMWSSPENSKYVCLTAHYVDDEWKLQKKILNFLTLDSSH<br>TEDMLSDVIIKCLMDWDIDCKLFAMTFDDCSTNDDIVSRIKDQVSESRPRLSNGQLLDVRSAAHVLNSIAQDAIEALQVVIQ<br>KIRGSVKYVKSSQSILGKFNEIAQQQGIDNHKIVVLDYPIRWNSTYMMLETAVEYRNVFHHLPELDPDFALSDEEWERASS<br>IVSYLKLLIEIINVFSSNKCPTANIYFPEICHVHIQLIEWCKSSDAFLSSLATKMKAKFDKYWSKCSLALAVAAILDPRFKMKLV	zf-BED--DUF-<br>domain--<br>Dimer_Tnp_hAT<br>--	I

		EYYYSQIYGSTALERIKEASDGIKELFNAYSICSTLIDQGSALPGSSLPSSSNDTRDRLKGFDKFLHETSQSQTAISDLEKYL<br>DEPVFPCNCDFNILNWWRVHTPRYPILSMMARDVLGTPMSTVAQEFAFNAGGRMLDSNQSSPPPDTQQALICTRDWLR<br>TQSDDATPSSSHYALPLYVEAN		
Ghir_A12G013020.1	GhiZf-BED31_III	MVEEMAPLRSIGYVDPGWEHGTAQDERKKKVKCNYCGKVVSGGIFRLKQHLARLSGEVTHCEKVPEEVCLNMRKNLEG<br>CRSGRKRRQFDYEQAALSIQSNEYSDGEDASASYKHKGKKVMGDKNLVIKFTPLRSLGYVDPGWEHCVAQDEKKRRVK<br>CNYCEKIISGGINRFKQHLARIPGEVAYCEKAPEEVYLKIKENMKWHRTGRRHRPDTKEISTFYMHSDNEDEGGEEEGYL<br>QCVSKDILAIDDKVSDNDIRNNVRGRSPGSSGNGAEPLLKKSRLDSVFLKSLKSQTSAHYKQPRARTGFEKKTHREVISAI<br>CKFFYHAGIPSNAANSPYFHKMLELVGQYGQGLQGPSSRLISGRLLQEEIANIKEYLVELKTSWAITGCSVMADSWNDAQ<br>GRMLINFLVSCPRGVYFLSSVDATDIIEDAVHLFKLLDKAVDEVGEEYVVQVITRNTLSFRNAGKMLEEKRRNLFWTPCAV<br>YCIDRMLEDFVNIKWVGECVDKAKKVTRFIYNNTWLLNFMKKEFTKGQELLQPAVTKFGTNFFTLQSLLDQRVGLKRMFQ<br>SNRWLSSRFSKSDEGKEVEKIVLNVSFWKKMQYVKKSFEPVAEVLQRIGSDKSDHAVYIYIHGDDVRKYGPFWSVIESN<br>WSSLFHHPLYVAAYFLNPSYRYRPDFLMNPEVIRGLNECIVRLEADNGKKIAASMQIPDFVSAKADFGTDLAISTRSELDPA<br>SWWQQHGISCLELQRIAIRILSQTCSSIGCEHNWSAFDQVHIKRHNCLSRKRLNDQTYVHYNLRLRERQLGRKPDELVSF<br>DSAMLESVLDDWLVETEKLAMHEDEEIIYTEVEQFCGDDMDEHESEEKRPAEMVTIAGFIEPLDVIPSAGGVTTDDDGLDF<br>LDDDLTD	zf-BED--zf-BED--DUF-domain--Dimer_Tnp_hAT--	III
Ghir_A13G001820.1	GhiZf-BED32_I	MSSNLEPIPITSQKHDPAWKHCQMFKNGERVQLKCIYCGKIFKGGGIHRIKEHLAGHKGNAATCLRVPSDVRVLMQESLD<br>GVVVKKRKKQKIAEEITNVNQVSTEIQAYGDQVDTNTGLLMIEKSDTLEPSSSLLVNREGTSNVAGERRKRGRGKNLPPE<br>ANALSFVPVELGARRVNNHVHMAIGRFLFDIGATMDAVNSVYFQPMVDAIVSGGSGALMPSCNDLQGWILRKSVEEVKS<br>ENDKVMGAWVRTGCSILVNQWNTQTGRILLNFLVYCPEGTVFLKSIDASSVINSSDALYELLKQVVEEVGSKHVLQVITNG<br>EEQYIVAGRRLAETFPTLYWTPCAAHCVDLILEDFAKLEWINAIIEQARSITKFIYNHSVVLNMVRRYTFGNDIVEPAATRSA<br>TNFTTLTRMVDLKNNLQAMVTSQQWVDCPYSKKPGGLAMLDLVSNQSFWSSCILIVRLTNPLLRVLRMVGSKKRPAMGY<br>VYAGMYRAKETIKKELVKRNEYMVYWNIIDHWWEQQWHHPLHAAGFYLNPRFFYSMEGDMPNEMLSGMLDCIEKLIPDV<br>TVQDKISKEINSYKNSVGDFGRKMAVRARDTLLPVEWWSTYGGSCPNLARLAIRVLSQTCSTFGLKHNHIPFEKLYETRN<br>CLEQQRLRDLIFVQCNLQLRQIGYESKQHDSMQPLSSESASIVEDWVTGIDAFLDDDTYPDWTTLETLSVNTMLLRPGDE<br>VEELGAGFNDHEIFNRMKEGDNENAEDNVVS	zf-BED--DUF-domain--Dimer_Tnp_hAT--	I
Ghir_A13G001820.2	GhiZf-BED33_I	MSSNLEPIPITSQKHDPAWKHCQMFKNGERVQLKCIYCGKIFKGGGIHRIKEHLAGHKGNAATCLRVPSDVRVLMQESLD<br>GVVVKKRKKQKIAEEITNVNQVSTEIQAYGDQVDTNTGLLMIEKSDTLEPSSSLLVNREGTSNVAGERRKRGRGKNLPPE<br>ANALSFVPVELGARRVNNHVHMAIGRFLFDIGATMDAVNSVYFQPMVDAIVSGGSGALMPSCNDLQGWILRKSVEEVKS<br>ENDKVMGAWVRTGCSILVNQWNTQTGRILLNFLVYCPEGTVFLKSIDASSVINSSDALYELLKQVVEEVGSKHVLQVITNG<br>EEQYIVAGRRLAETFPTLYWTPCAAHCVDLILEDFAKLEWINAIIEQARSITKFIYNHSVVLNMVRRYTFGNDIVEPAATRSA<br>TNFTTLTRMVDLKNNLQAMVTSQQWVDCPYSKKPGGLAMLDLVSNQSFWSSCILIVRLTNPLLRVLRMVGSKKRPAMGY<br>VYAGMYRAKETIKKELVKRNEYMVYWNIIDHWWEQQWHHPLHAAGFYLNPRFFYSMEGDMPNEMLSGMLDCIEKLIPDV<br>TVQDKISKEINSYKNSVGDFGRKMAVRARDTLLPVEWWSTYGGSCPNLARLAIRVLSQTCSTFGLKHNHIPFEKLYETRN<br>CLEQQRLRDLIFVQCNLQLRQMYAESHSFTDYAFGATKFYLQLMIFLSEVMRASNMILCSPCHLSLPLLWKTGLQELTHF<br>WMTIPIRIGQHLRHFL	zf-BED--DUF-domain--Dimer_Tnp_hAT--	I
Ghir_A13G001820.3	GhiZf-BED34_IV	MSSNLEPIPITSQKHDPAWKHCQMFKNGERVQLKCIYCGKIFKGGGIHRIKEHLAGHKGNAATCLRVPSDVRVLMQESLD<br>GVVVKKRKKQKIAEEITNVNQVSTEIQAYGDQVDTNTGLLMIEKSDTLEPSSSLLVNREGTSNVAGERRKRGRGKNLPPE<br>ANALSFVPVELGARRVNNHVHMAIGRFLFDIGATMDAVNSVYFQPMVDAIVSGGSGALMPSCNDLQGWILRKSVEEVKS<br>ENDKVMGAWVRTGCSILVNQWNTQTGRILLNFLVYCPEGTVFLKSIDASSVINSSDALYELLKQVVEEVGSKHVLQVITNG<br>EEQYIVAGRRLAETFPTLYWTPCAAHCVDLILEDFAKLEWINAIIEQARSITKFIYNHSVVLNMVRRYTFGNDIVEPAATRSA<br>TNFTTLTRMVDLKNNLQAMVTSQQWVDCPYSKKPGGLAMLDLVSNQSFWSSCILIVRLTNPLLRVLRMVGSKKRPAMGY<br>VYAGMYRAKETIKKELVKRNEYMVYWNIIDHWWEQQWHHPLHAAGFYLNPRFFYSMEGDMPNEMLSGMLDCIEKLIPDV<br>TVQDKISKEINSYKNSVGDFGRKMAVRARDTLLPGESLSYGCFLNFGCYIST	zf-BED--DUF-domain	IV
Ghir_A13G007540.1	GhiZf-BED35_II	MLLIYFNLLYNFFSCRISTEPTSIEGSVTPPTSIDSENSGVGASSQANVTIGKRKATPQRSEVWSHFTKIINSEGASKAKCNY<br>CQKEFCCDVKRNGTGSLKYHIGACKKNPSNVVDTSQGQLVLPRNGVEGGEGHLSTWRFDQEACRKGLAQMIVIDELPF<br>KFVESEGFKKFMFVACLRFHIPSQTTMTRDVYQLYLDERVKIKQLLKVLVLEFA	zf-BED	II
Ghir_D01G015550.1	GhiZf-BED36_I	MSTEPTSIEGSVTPPTSIDSENSGVGASIQTKGTTRKRKSPPQRSEVWSHFTKFINSEGASKAKCNYCEKEFCCDMKKNG<br>TGSLKYHIGSCKKNPSNVVDTSQGQLVLPRKGVEGGEGNLSTWRFDQETCRKGLAQMILIDELPFKFVESEGFKKFMFV<br>ACPRFHIPSRTTMTRDVYQLYLDERVKIKQLFRSSCSRVCLTTDTWTSLQRVNYLCITAHFIDNDWKLNKKILNFCPISSHK<br>GESIGMVIEKCLLNWGIDKLFTVTVDNASSNDVAIVQNGKYLHMRCMAHIVNLIVVEGLKEMNKSVERVRGAVRYVRQSP<br>ARLQKFKECVVVEKIECKKMLCLDVCTRWNSTYLMLDTTQNFERAFERFEEQDTNFRAELERGEGWPSVDDWDNVRKL<br>RDFLEHFYEVTLRISGTSYVTSNNFFDELSEIDILLRDAQLNSNIDFNVMAIKMKEKYDKYWGDIDKMNLLMFVACVLDPRQ<br>KLKYLEFALSEMSSSEKACEMMQKLKESLYELFDEYKPPLHSTCSQSSSSVSTHVSIGEPQQKMKRRMQALYKKRELEIC<br>GEDKTSELDKYLAEANEEFFEYFDILLWWKVNSPRFPTLSKIARDVLAIPVSTVASESAFSTGGRVLDQYRSSLTPKIVQAL<br>VCTQDWIRRSSSQEDIKKIEKQIQELDKIENGCWIL	zf-BED--DUF-domain--Dimer_Tnp_hAT--	I
Ghir_D02G000710.1	GhiZf-BED37_I	MDNFDQKLGPEFFKNLSAEAVTPLNVVHEEIYESSSKRPKTTSKVWDIFEKLPAQQGDSKAICKLCRRIYTAKTTSGTSHL<br>RRHIEACVKRGNHEVDQRSIEACFKPVKRNANRLTLSHDTLIAATTSLQNYKLDVDEIHRAIAMMIIVDEQPFSVVEDAGFR<br>RLLSAACPEFPVLSRSSIKRDIISIYVKERENIRELLATCPGRICLTSSTWKSDSDDHFSCVTTHFIDHEWRLQRRILRFKLM<br>PPPYDSLSVADEIALCMVQWNIEHKVFSVTLENLSSDDCVADMLRSRLAAKKYLPCKGVFFHVSCFFRILNSIVQAGLNLV<br>VDIIAKLRLGIKYVQQSPHRKKNFYIIAKTLNLDTQRKLCLDTPARWNSTYNMVEVAFCYKNAFVYLAEQDKNFLHKLSEDE<br>WEKMSVLYKFLKVFYEVTCVFFRNRQPTSNLYFKAAWKVHSRLFDMVRGPENFMTHMVREMHSKLNQYWSAYNLILSC<br>AAIDPRYKIKFVEYCYTKLYGSGAQKYVSVSVNTLYGLFDEYMQNSARPSQTTLLSTAASKISNDKDNNDGFEDYETFQS<br>ARFRTQVEKSQLDLYLEEPSHDLNSEIDVLEYWTLCSLRYPELSKMARDVLTIPVSTIASDSDFDISPQVISTDRSSLKPKM<br>LQSLVCLQDWMLASDRTRGLGSMESKPEDDSSSSSDGDDDY	zf-BED--DUF-domain--Dimer_Tnp_hAT--	I
Ghir_D02G000710.2	GhiZf-BED38_I	MDNFDQKLGPEFFKNLSAEAVTPLNVVHEEIYESSSKRPKTTSKVWDIFEKLPAQQGDSKAICKLCRRIYTAKTTSGTSHL<br>RRHIEACVKRGNHEVDQRSIEACFKPVKRNANRLTLSHDTLIAATTSLQNYKLDVDEIHRAIAMMIIVDEQPFSVVEDAGFR<br>RLLSAACPEFPVLSRSSIKRDIISIYVKERENIRELLATCPGRICLTSSTWKSDSDDHFSCVTTHFIDHEWRLQRRILRFKLM<br>PPPYDSLSVADEIALCMVQWNIEHKVFSVTLENLSSDDCVADMLRSRLAAKKYLPCKGVFFHVSCFFRILNSIVQAGLNLV<br>VDIIAKLRLGIKYVQQSPHRKKNFYIIAKTLNLDTQRKLCLDTPARWNSTYNMVEVAFCYKNAFVYLAEQDKNFLHKLSEDE<br>WEKMSVLYKFLKVFYEVTCVFFRNRQPTSNLYFKAAWKVHSRLFDMVRGPENFMTHMVREMHSKLNQYWSAYNLILSC<br>AAILDPRYKIKFVEYCYTKLYGSGAQKYVSVSVNTLYGLFDEYMQNSARPSQTTLLSTAASKISNDKDNNDGFEDYETFQS<br>ARFRTQVEKSQLDLYLEEPSHDLNSEIDVLEYWTLCSLRYPELSKMARDVLTIPVSTIASDSAFDISPQVISTDRSSLKPKM<br>LQSLVCLQDWMLASDRTSKSPYLIAYSNCCYTMHNCFV	zf-BED--DUF-domain--Dimer_Tnp_hAT--	I
Ghir_D02G001300.1	GhiZf-BED39_I	MNETMSDPNKKYTFSIQSNSCYSKDWNSYKFMRSCEYKIEQTIIEKALESKDGYHFEPSLTKSDIDDYMYLQRAKHCNVD<br>VVVAICLQNRYTSNDIYVVEFYWPTTESEISKSFTPRIFNDLKHMEKKFVTVKVQGTEQAISNIPTSSYTARPLKIAEETEDV<br>DAVELNGVNVQRGVVPNFPSPITIQSSSKVVAAPSNTLEGPHNQIFPNGDPEIVKANKQEPSKATQRELRSKVWDHFDRF<br>EEDEKQVAKCKHCPKVLTGSSKSGTTHLNNHSKVCPGKKKQNQESQLILPVDTNEGSLRFDKKRSHMDLAKMMIKLQCP<br>LDMAEQETFKNFVKGLQPMFEFQSKDILSYIHRIYDEEKEKLQLYFDKLASKFNLTVSLLKNNSGKTIYCCLISHFIDDGWEL<br>KRKILALKTLEHINDTKALGEIIRSLVLEWNISNKVCSITVDNSFLNDSMVDQIKEICLSDQGSVSSDHWFISFTLLEDGFREM<br>DGILFKLRKSIEYVTETRHGKLKFQEAVDQVKLQGGKLWDDLSFRLKSDFDILDSALRSREIFCKLEQIDDNFKLNPTMEE<br>WENAVALQSCLKCFDDVKGTQCLPVSLYLPKLCDTYKKFLQLEKSSHSFVTLMKRKFDRYWSLCNLALAVASVLDPRLKF<br>KIVELSYRVIYGHDSKMRLNMFHKVLRDVYYEYASEAKNLTSSASVLDDFNCSTIVLGNDSILDSLSKFASASNFNEEASW<br>KLELELYLDEPLLPMDGAFFDILGWWCDKSQRFPILAKMAQDFLAIPVSISTSCSNISAMINNPAYGSLNPESMEALVCSEN<br>WLETPKEMHYHFIG	zf-BED--DUF-domain--Dimer_Tnp_hAT--	I
Ghir_D02G001300.2	GhiZf-BED40_I	MEKKFVTVKVQGTEQAISNIPTSSYTARPLKIAEETEDVDAVELNGVNVQVIALSYIINDLFSISQIFPNGDPEIVKANKQEPS<br>KATQRELRSKVWDHFDRFEEDEKQVAKCKHCPKVLTGSSKSGTTHLNNHSKVCPGKKKQNQESQLILPVDTNEGSLRF<br>DKKRSHMDLAKMMIKLQCPLDMAEQETFKNFVKGLQPMFEFQSKDILSYIHRIYDEEKEKLQLYFDKLASKFNLTVSLLKN<br>NSGKTIYCCLISHFIDDGWELKRKILALKTLEHINDTKALGEIIRSLVLEWNISNKVCSITVDNSFLNDSMVDQIKEICLSDQGS<br>VSSDHWFISFTLLEDGFREMDGILFKLRKSIEYVTETRHGKLKFQEAVDQVKLQGGKLWDDLSFRLKSDFDILDSALRSREI<br>FCKLEQIDDNFKLNPTMEEWENAVALQSCLKCFDDVKGTQCLPVSLYLPKLCDTYKKFLQLEKSSHSFVTLMKRKFDRY	zf-BED--DUF-domain--Dimer_Tnp_hAT--	I

		WSLCNLALAVASVLDPRLKFKIVELSYRVIYGHDSKMRLNMFHKVLRDVYYEYASEAKNLTSSASVLDDFNCSTIVLGNDSILDSLSKFASASNFNEEASWKLELELYLDEPLLPMDGAFFDILGWWCDKSQRFPILAKMAQDFLAIPVSISTSCSNISAMINNPAYGSLNPESMEALVCSENWLETPKER		
Ghir_D02G001310.1	GhiZf-BED41_VIII	MASSLFDIDTDTALRLLLSCAEAIEDGDLKSADAYLQNILILADERPYLYKSRVVKYFADALVRRAYGLHPASSYFTFPVDPSPYYHCGSYLINGVIENVIHDALMEKNALMGNRKLHLIDFSIPYSSFQNSVVRTLPTFSGDPLPVRVSYILPPFLKKYVKFLRQMEFLTRDAKEVNVKLEDELKLVYGNSLAEVDECEIDLKRRRDDEMVVVYYKFKLDKLVRDAKAMERELVRLKEINPTIVIMLDFYSNHTHSNFLTCFKDSFQYSLKTLDCWEELDLYFDEEYAWECHIEAWEGNNVIRRHPTLTEWQHLFSMAGFSRIPLNHREGIDLIVKDVNPLNDFFSMSNQSWLEIMGKEEECLILGYKECPMFFLSAWKPKVEEEHLNFNSSNGFNPYPSPLRPLRPFPEGLTLSRVAAVAEIYDILNHLYCEHKFSLALTWVSKVDNMNETMSDPNKKYTFSIQSNSCYSKDWNSYKFMRSCEYKIEQTIIEKALESKDGYHFEPSLTKSDIDDYMYLQRAKHCNVDVVVAICLQNRYTSNDIYVVEFYWPTTESEISKSFTPRIFNDLKHMEKKFVTVKVQGTEQAISNIPTSSYTARPLKIAEETEDVDAVELNGVNVQRGVVPNFPSPITIQSSSKVVAAPSNTLEGPHNQIFPNGDPEIVKANKQEPSKATQRELRSKVWDHFDRFEEDEKQVAKCKHCPKVLTGSSKSGTTHLNNHSKVCPGKKKQNQESQLILPVDTNEGSLRFDKKRSHMDLAKMMIKLQCPLDMAEQETFKNFVKGLQPMFEFQSKDILSYIHRIYDEEKEKLQLYFDKLASKFNLTVSLLKNNSGKTIYCCLISHFIDDGWELKRKILALKTLEHINDTKALGEIIRSLVLEWNISNKVCSITVDNSFLNDSMVDQIKEICLSDQGSVSSDHWFISFTLLEDGFREMDGILFKLRKSIEYVTETRHGKLKFQEAVDQVKLQGGKLWDDLSFRLKSDFDILDSALRSREIFCKLEQIDDNFKLNPTMEEWENAVALQSCLKCFDDVKGTQCLPVSLYLPKLCDTYKKFLQLEKSSHSFVTLMKRKFDRYWSLCNLALAVASVLDPRLKFKIVELSYRVIYGHDSKMRLNMFHKVLRDVYYEYASEAKNLTSSASVLDDFNCSTIVLGNDSILDSLSKFASASNFNEEASWKLELELYLDEPLLPMDGAFFDILGWWCDKSQRFPILAKMAQDFLAIPVSISTSCSNISAMINNPAYGSLNPESMEALVCSENWLETPKER	GRAS--zf-BED--DUF-domain--Dimer_Tnp_hAT--	VIII
Ghir_D02G001340.1	GhiZf-BED42_XI	MLEDVCFRYELPVALTWACEANTDKIMLDGKKYTLFMEKTSFYASNEGSQCFMEACAKHHIQEGQAIAGKALQSSANFHFKPSITKLIKSDYPLFNAAQLFGNHAVVAICLQNHYIIGDVYVVEFYWPEIESEKSESLALDIFNDLKNMKKKFVTIRVGSNEGTMHTRNAQPASSTNDLLSSNTTWSLNTVQPCDVHEMERHGLVESAPFSTPNPMSYGGVLQTQGPHKQEIGEKDFISQTVSIGDYEIVKAYMETCKVPRTKRRKYLSKVWLDFDKFEVNGKQVAKCKHCNKDFTGSSKSGTTHLKNHLERCQSKKIKNQKRQLITSEIGDLITRDSDESNFTFDQERSRLDFAKMIIKHQCPLDMAEQEFFKIFVKNLQPMFEFQSKDILLSDIHRIYKEEKEKLQLYFDQVACNFNLTISLWKNNLGKTAYCCLIAHFIDDNWGPKMKIIACKPLEHIYDTKALNEIIQSSVLEWNISKKVFSITMDNPYLSDDMFQKIKETCFRDQGSFPLTHWFIGCTFIKDGFREMDLILLKLRKSIEYVSEIAQGKLKFEEVVNQVKLQGGKSWDDLSLRLDSDFGVLHSALESREIFCQLEKIDSNFKLNPSVEEWEMVLAFHSCLKCFDDIEGTQSLTANLYFPKLCNIYKKFLHLGKSNYPIVTLMKRKFDYYWSLCNLAFSVATILDPRLKFKFVEFSYTEIYGHDSKMHLNRFHKVLTDVYYEYANEARNLSKSTSDLDDSNSSTTEIDNDCILESFSKFASASNFNEVASWKSELDCYLDEPLLPLDGAFDILYWWCINTKRFPTLAKMARDFLAMPISILAPCLNFNAMITNPTYNNLNPESMEALVCSQNWLEIPKENDGENHGPMQNMSEPALPLDGDILETNHGRNIAAAIEIPNDEPSFNGNQSDQFQSSSSESDDETSLREQGSWCREDVRTYLVSNFTNKEVKRLNRWKRSELSGKKIGRDNDFQLMGENLTPLLMVPHCDETLIEYYIDDSVVNTYFKLLKKRSDKFPNGYIKHYSFDSLIATCLIEGSKSEDEVLAWFKDEKLRGVHKLFLPMCLSAHWVLFCVDTKEKKISWLDPIPSSRIMSNSVEKQKIFQWFTLYLLPQFGYNDAEKWAFEVRTDIPKQENSIDCGVFVIKYGDCLMHGDFFPFTQKDMIHFRRRIFLDIYRGRLHGKR	zf-BED--DUF-domain--Dimer_Tnp_hAT--Peptidase_C48--	XI
Ghir_D02G002620.1	GhiZf-BED43_IX	MDYSFSDTALRLLLFCAEAIENRDLKSADAFLLVILILADKRHYWFRDDSIVVKYFAYALVSRAYGLHPASSYFTFPVDPAPYYQYNSCHINGVIKKVIDDALMGNRRLHLIDFNIPYYGFEGSVLSTLPNFFCDRLRVRVSYILPPFLKEYVEFSRQMEFLTEDAKEVNVELEDELKVVYGNSLAEVDECEIDFKRRRDDEMVVVYYKFKLEKLVRDAKAMKRELVRLKEINPTIVIILDFYSNHSDSDFLTCFKDSFQYSLKTLDYWQELDRYLDGKKEWEFNIEAGEGNNIIRRHPTLTEWQHLFSTAGFSRIPLNHRKDNLSVEDNSFLKIMREEEECLILGYKGCPMFFLSAWKPKVEDGHFNSNSTNYKFDQGFNPNPLPLQPLQPFPEGSILNRLAALAEIHNISKDLCCKYKLSLALTWASKVNNMNGTISDPNKKHTFFIQSNYCYVKDRKSYDFMFGFERMISVPIFEKAFESRDGYHFEPSLTEVEDFKYFMLKDCNIDVALAICLQNLHTSDEVYVVEFYWPPTESEISKSLALRIFDDLKHMKTTFVTVKVQGPEIKFQEEAISSIPTSSNTAMPLKIAEEARGIRAKEINAHIEQIVETKRNKQRKLRSKVWVDFHKSEEEGKQVAKCKHCPKVLTGSSKSGTTHLNNHSKVCPGKKKQNQESQLILPVDTNERSSTFDQERSHLALVKMVIRQQYPLDLAGQEAFKNFVKGLQPMYEFQSRDKLLSDIHRIYNEEREKLQLYFDQLACKLNLTVSLSKNNHGKTAYCCLIAHFIDDSWELKMKTLGLRTLEHINDTKAVGGIIQSLVSEWNIGNKVCSITVDNSFLDDSMVQQIKENCLSNLVSLSSTHWFINCTLLEDGFREMDDLLFKLKKSIEYVTETKHGRLKFQEAVDQVKLQDGKSWDDLSLKLASDFGILDSALRSREIFCKLEQIDGNFKLNPSMEEWENAAALQSCLRCFDDIKGTQSLTVSLYLLKLCDIYMKFLQLEKSNPSFVTLMKRRFDHYWRLCNSALAVASVLDPRLKFKVVEFSYKVIYGHDSKVQLNTFREVLTNVYNEYANETKNQTTSASVLDDINWPGNNSIWDSFSKFVTASEASSKSELELYLDEHLIPMDGAIFDILGWWSDKSQMFPILAKMARDFLAIPVSIFIPCSNIKATINNPAYNILNPESMEALVCSENWLETPKGNDGENHEPTQTMDKGKRKLDEDTCVRKKSKPSNCEKAISTEDIDKDSNNNDEPAGEISIGKLQTENSSRNGCYGETSSGNKSKASNKMMGTISLRAIHQEKSSSELNHGRNVEDVSSGDSSSDNDQSDQLQSSSSESDVEITLKEQGSWSEQDIKAYLLSEFTEKENELIDKWQKNELKGKMIGRDKYFKIQGEKLAPLLMVPQGDETREEYYIEDLVVNTFFELLKKRSDKFPNVYINHYSFGSQIATQLIEGPRTEQEVLAWVKVDELRGVHKMFLPMSLSKHWVLFYVDTKEKKISWLDPIASSRIRSYNVEKDIILQWFTTLLLPKLGYVDAKEWPFLVRNDIPEQKNLVDCAVFVMKYGDCLTHGDYFPFKQEDMVHFRRRIFVDIYRGRIHKKKTKIDALFRNSKL	GRAS--zf-BED--DUF-domain--Dimer_Tnp_hAT--Peptidase_C48--	IX
Ghir_D02G002620.2	GhiZf-BED44_IX	MDYSFSDTALRLLLFCAEAIENRDLKSADAFLLVILILADKRHYWFRDDSIVVKYFAYALVSRAYGLHPASSYFTFPVDPAPYYQYNSCHINGVIKKVIDDALMGNRRLHLIDFNIPYYGFEGSVLSTLPNFFCDRLRVRVSYILPPFLKEYVEFSRQMEFLTEDAKEVNVELEDELKVVYGNSLAEVDECEIDFKRRRDDEMVVVYYKFKLEKLVRDAKAMKRELVRLKEINPTIVIILDFYSNHSDSDFLTCFKDSFQYSLKTLDYWQELDRYLDGKKEWEFNIEAGEGNNIIRRHPTLTEWQHLFSTAGFSRIPLNHRKDNLSVEDNSFLKIMREEEECLILGYKGCPMFFLSAWKPKVEDGHFNSNSTNYKFDQGFNPNPLPLQPLQPFPEGSILNRLAALAEIHNISKDLCCKYKLSLALTWASKVNNMNGTISDPNKKHTFFIQSNYCYVKDRKSYDFMFGFERMISVPIFEKAFESRDGYHFEPSLTEVEDFKYFMLKDCNIDVALAICLQNLHTSDEVYVVEFYWPPTESEISKSLALRIFDDLKHMKTTFVTVKVQGPEIKFQEEAISSIPTSSNTAMPLKIAEEARGIRAKEINAHIEQIIFNDDSQIVETKRNKQRKLRSKVWVDFHKSEEEGKQVAKCKHCPKVLTGSSKSGTTHLNNHSKVCPGKKKQNQESQLILPVDTNERSSTFDQERSHLALVKMVIRQQYPLDLAGQEAFKNFVKGLQPMYEFQSRDKLLSDIHRIYNEEREKLQLYFDQLACKLNLTVSLSKNNHGKTAYCCLIAHFIDDSWELKMKTLGLRTLEHINDTKAVGGIIQSLVSEWNIGNKVCSITVDNSFLDDSMVQQIKENCLSNLVSLSSTHWFINCTLLEDGFREMDDLLFKLKKSIEYVTETKHGRLKFQEAVDQVKLQDGKSWDDLSLKLASDFGILDSALRSREIFCKLEQIDGNFKLNPSMEEWENAAALQSCLRCFDDIKGTQSLTVSLYLLKLCDIYMKFLQLEKSNPSFVTLMKRRFDHYWRLCNSALAVASVLDPRLKFKVVEFSYKVIYGHDSKVQLNTFREVLTNVYNEYANETKNQTTSASVLDDINWPGNNSIWDSFSKFVTASEASSKSELELYLDEHLIPMDGAIFDILGWWSDKSQMFPILAKMARDFLAIPVSIFIPCSNIKATINNPAYNILNPESMEALVCSENWLETPKGNDGENHEPTQTMDKGKRKLDEDTCVRKKSKPSNCEKAISTEDIDKDSNNNDEPAGEISIGKLQTENSSRNGCYGETSSGNKSKASNKMMGTISLRAIHQEKSSSELNHGRNVEDVSSGDSSSDNDQSDQLQSSSSESDVEITLKEQGSWSEQDIKAYLLSEFTEKENELIDKWQKNELKGKMIGRDKYFKIQGEKLAPLLMVPQGDETREEYYIEDLVVNTFFELLKKRSDKFPNVYINHYSFGSQIATQLIEGPRTEQEVLAWVKVDELRGVHKMFLPMSLSKHWVLFYVDTKEKKISWLDPIASSRIRSYNVEKDIILQWFTTLLLPKLGYVDAKEWPFLVRNDIPEQKNLVDCAVFVMKYGDCLTHGDYFPFKQEDMVHFRRRIFVDIYRGRIHKKKTKIDALFRNSKL	GRAS--zf-BED--DUF-domain--Dimer_Tnp_hAT--Peptidase_C48--	IX
Ghir_D03G017110.1	GhiZf-BED45_IV	MVRGRDACWEHCVLVDATRQKVRCNYCHREFSGGVYRMKFHLAQIKNKDIVPCAEVPDDVRDHIQSILNTPKKQKTPKKPKMDKTVANGQQNSSSASGGLHPNHGSSGQHGSTCPSLLFPHPSPSEQPATDDAQKQKLDDADKKIAVFFFHNSIPFSAAKSMYYQEMVDAIAECGVGYKAPSYEKLRSSLLEKVKGDIHDCYKKYREEWKETGCTVLCNSWSDGRTKSFVIFSVTYPKGTLFLKSVDVSVMRIASYLFELLESVVLEVGLENVIQVITDSTASYVCAGRHLMAKYSSLFWSPCASYCIDKMLETLVNKVGGIVLEEAKSIARYIYSHAWILNMMRKITGGRELMRPRITRFVDNYLTLRSIVIQEDNLKHMFSHSEWLSSIYSRRSDAQAIKSLLYLERFWKSAREAVSVSESLVKILRIVDGDMPAMGYMYEGIERAKVL	zf-BED--DUF-domain	IV
Ghir_D03G017110.2	GhiZf-BED46_IV	MVRGRDACWEHCVLVDATRQKVRCNYCHREFSGGVYRMKFHLAQIKNKDIVPCAEVPDDVRDHIQSILNTPKKQKTPKKPKMDKTVANGQQNSSSASGGLHPNHGSSGQHGSTCPSLLFPHPSPSEQPATDDAQKQKLDDADKKIAVFFFHNSIPFSAAKSMYYQEMVDAIAECGVGYKAPSYEKLRSSLLEKVKGDIHDCYKKYREEWKETGCTVLCNSWSDGRTKSFVIFSVTYPKGTLFLKSVDVSVMRIASYLFELLESVVLEVGLENVIQVITDSTASYVCAGRHLMAKYSSLFWSPCASYCIDKMLETLVNKVGGIVLEEAKSIARYIYSHAWILNMMRKITGGRELMRPRITRFVDNYLTLRSIVIQEDNLKHMFSHSEWLSSIYSRRSDAQAIKSLLYLERFWKSAREAVSVSESLVKILRIVDGDMPAMGYMYEGIERAKVL	zf-BED--DUF-domain	IV
Ghir_D04G004090.1	GhiZf-BED48_I	MDMSDAVIVNSSRLKSIVWNDFDRVKKGDTFVAICRHCKKKLSGSSTSGTSHLRNHLIRCQRRSNHGVAQYFSAKDKKKEGSLALVTIDQEQKNDEVLSIVNLRYEQEQIKSEHVAIGSNSLDQRRSQFDLARMIILHNYPLAMVEHDGFKIFVRNLQPLFELVTRNKVEADCMEIYAKEKQKVYEIFDKLPGKISVSADVWTASEDDAAYLSLAAHYIDENWQLKKKNLNFVTIDPSYAEDMHSEVIMNCLMDWDIDRKLFSMIFDSFTSDNIVERIRDRLSQNRFLHCNGQLFDVRCAVDLLNRMAHDALETLCEITQKIRESIRYVKSSEATQATFNELADEVQVETKKCLCIDNPLKWNSTYLMLEAASEYRKVFSCLRDRDPVNMKFLLSDPEWDRLITVTSFLKLFVEVTNVFTRSKYPTANIFFPEICDIHLQLIEWCKNPDEYISSLALKMRKKFEEYWYKCSSGLAVAAMLDPRFKMKLLEYYYPQLYGDSATELIDDVFECIKSLYNEHSMVSPLASSIDQGLDWQASGIPGSGKDSRDRLMGFDKFLHETSQAEG	zf-BED--DUF-domain--Dimer_Tnp_hAT--	I

		SSSDLDKYLEEPLFPRNVDFNVLNWWKVHTPRYPILSMMARNILGIPISKVAAESRFDTGGRMLNHNWSSLPPTTIQALMCSRDWIRSELES		
Ghir_D04G004090.2	Ghi Zf-BED 49_I	MDMSDAVIVNSSRLKSIVWNDFDRVKKGDTFVAICRHCKKKLSGSSTSGTSHLRNHLIRCQRRSNHGVAQYFSAKDKKKEGSLALVTIDQEQKNDEVLSIVNLRYEQEQIKSEHVAIGSNSLDQRRSQFDLARMIILHNYPLAMVEHDGFKIFVRNLQPLFELVTRNKVEADCMEIYAKEKQKVYEIFDKLPGKISVSADVWTASEDDAAYLSLAAHYIDENWQLKKKNLNFVTIDPSYAEDMHSEVIMNCLMDWDIDRKLFSMIFDSFTSDNIVERIRDRLSQNRFLHCNGQLFDVRCAVDLLNRMAHDALETLCEITQKIRESIRYVKSSEATQATFNELADEVQVETKKCLCIDNPLKWNSTYLMLEAASEYRKVFSCLRDRDPVNMKFLLSDPEWDRLITVTSFLKLFVEVTNVFTRSKYPTANIFFPEICDIHLQLIEWCKNPDEYISSLALKMRKKFEEYWYKCSSGLAVAAMLDPRFKMKLLEYYYPQLYGDSATELIDDVFECIKSLYNEHSMVSPLASSIDQGLDWQASGIPGSGKDSRDRLMGFDKFLHETSQAEGSSSDLDKYLEEPLFPRNVDFNVLNWWKVHTPRYPILSMMARNILGIPISKVAAESRFDTGGRMLNHNWSSLPPTTIQALMCSRDWIRSELES	zf-BED--DUF-domain--Dimer_Tnp_hAT--	I
Ghir_D04G004090.3	Ghi Zf-BED 50_I	MDMSDAVIVNSSRLKSIVWNDFDRVKKGDTFVAICRHCKKKLSGSSTSGTSHLRNHLIRCQRRSNHGVAQYFSAKDKKKEGSLALVTIDQEQKNDEVLSIVNLRYEQEQIKSEHVAIGSNSLDQRRSQFDLARMIILHNYPLAMVEHDGFKIFVRNLQPLFELVTRNKVEADCMEIYAKEKQKVYEIFDKLPGKISVSADVWTASEDDAAYLSLAAHYIDENWQLKKKNLNFVTIDPSYAEDMHSEVIMNCLMDWDIDRKLFSMIFDSFTSDNIVERIRDRLSQNRFLHCNGQLFDVRCAVDLLNRMAHDALETLCEITQKIRESIRYVKSSEATQATFNELADEVQVETKKCLCIDNPLKWNSTYLMLEAASEYRKVFSCLRDRDPVNMKFLLSDPEWDRLITVTSFLKLFVEVTNVFTRSKYPTANIFFPEICDIHLQLIEWCKNPDEYISSLALKMRKKFEEYWYKCSSGLAVAAMLDPRFKMKLLEYYYPQLYGDSATELIDDVFECIKSLYNEHSMVSPLASSIDQGLDWQASGIPGSGKDSRDRLMGFDKFLHETSQAEGSSSDLDKYLEEPLFPRNVDFNVLNWWKVHTPRYPILSMMARNILGIPISKVAAESRFDTGGRMLNHNWSSLPPTTIQALMCSRDWIRSELES	zf-BED--DUF-domain--Dimer_Tnp_hAT--	I
Ghir_D04G004090.4	Ghi Zf-BED 51_I	MDMSDAVIVNSSRLKSIVWNDFDRVKKGDTFVAICRHCKKKLSGSSTSGTSHLRNHLIRCQRRSNHGVAQYFSAKDKKKEGSLALVTIDQEQKNDEVLSIVNLRYEQEQIKSEHVAIGSNSLDQRRSQFDLARMIILHNYPLAMVEHDGFKIFVRNLQPLFELVTRNKVEADCMEIYAKEKQKVYEIFDKLPGKISVSADVWTASEDDAAYLSLAAHYIDENWQLKKKNLNFVTIDPSYAEDMHSEVIMNCLMDWDIDRKLFSMIFDSFTSDNIVERIRDRLSQNRFLHCNGQLFDVRCAVDLLNRMAHDALETLCEITQKIRESIRYVKSSEATQATFNELADEVQVETKKCLCIDNPLKWNSTYLMLEAASEYRKVFSCLRDRDPVNMKFLLSDPEWDRLITVTSFLKLFVEVTNVFTRSKYPTANIFFPEICDIHLQLIEWCKNPDEYISSLALKMRKKFEEYWYKCSSGLAVAAMLDPRFKMKLLEYYYPQLYGDSATELIDDVFECIKSLYNEHSMVSPLASSIDQGLDWQASGIPGSGKDSRDRLMGFDKFLHETSQAEGSSSDLDKYLEEPLFPRNVDFNVLNWWKVHTPRYPILSMMARNILGIPISKVAAESRFDTGGRMLNHNWSSLPPTTIQALMCSRDWIRSELES	zf-BED--DUF-domain--Dimer_Tnp_hAT--	I
Ghir_D04G004090.5	Ghi Zf-BED 52_I	MDMSDAVIVNSSRLKSIVWNDFDRVKKGDTFVAICRHCKKKLSGSSTSGTSHLRNHLIRCQRRSNHGVAQYFSAKDKKKEGSLALVTIDQEQKNDEVLSIVNLRYEQEQIKSEHVAIGSNSLDQRRSQFDLARMIILHNYPLAMVEHDGFKIFVRNLQPLFELVTRNKVEADCMEIYAKEKQKVYEIFDKLPGKISVSADVWTASEDDAAYLSLAAHYIDENWQLKKKNLNFVTIDPSYAEDMHSEVIMNCLMDWDIDRKLFSMIFDSFTSDNIVERIRDRLSQNRFLHCNGQLFDVRCAVDLLNRMAHDALETLCEITQKIRESIRYVKSSEATQATFNELADEVQVETKKCLCIDNPLKWNSTYLMLEAASEYRKVFSCLRDRDPVNMKFLLSDPEWDRLITVTSFLKLFVEVTNVFTRSKYPTANIFFPEICDIHLQLIEWCKNPDEYISSLALKMRKKFEEYWYKCSSGLAVAAMLDPRFKMKLLEYYYPQLYGDSATELIDDVFECIKSLYNEHSMVSPLASSIDQGLDWQASGIPGSGKDSRDRLMGFDKFLHETSQAEGSSSDLDKYLEEPLFPRNVDFNVLNWWKVHTPRYPILSMMARNILGIPISKVAAESRFDTGGRMLNHNWSSLPPTTIQALMCSRDWIRSELES	zf-BED--DUF-domain--Dimer_Tnp_hAT--	I
Ghir_D04G004090.6	Ghi Zf-BED 53_I	MDMSDAVIVNSSRLKSIVWNDFDRVKKGDTFVAICRHCKKKLSGSSTSGTSHLRNHLIRCQRRSNHGVAQYFSAKDKKKEGSLALVTIDQEQKNDEVLSIVNLRYEQEQIKSEHVAIGSNSLDQRRSQFDLARMIILHNYPLAMVEHDGFKIFVRNLQPLFELVTRNKVEADCMEIYAKEKQKVYEIFDKLPGKISVSADVWTASEDDAAYLSLAAHYIDENWQLKKKNLNFVTIDPSYAEDMHSEVIMNCLMDWDIDRKLFSMIFDSFTSDNIVERIRDRLSQNRFLHCNGQLFDVRCAVDLLNRMAHDALETLCEITQKIRESIRYVKSSEATQATFNELADEVQVETKKCLCIDNPLKWNSTYLMLEAASEYRKVFSCLRDRDPVNMKFLLSDPEWDRLITVTSFLKLFVEVTNVFTRSKYPTANIFFPEICDIHLQLIEWCKNPDEYISSLALKMRKKFEEYWYKCSSGLAVAAMLDPRFKMKLLEYYYPQLYGDSATELIDDVFECIKSLYNEHSMVSPLASSIDQGLDWQASGIPGSGKDSRDRLMGFDKFLHETSQAEGSSSDLDKYLEEPLFPRNVDFNVLNWWKVHTPRYPILSMMARNILGIPISKVAAESRFDTGGRMLNHNWSSLPPTTIQALMCSRDWIRSELES	zf-BED--DUF-domain--Dimer_Tnp_hAT--	I
Ghir_D04G004090.7	Ghi Zf-BED 54_I	MDMSDAVIVNSSRLKSIVWNDFDRVKKGDTFVAICRHCKKKLSGSSTSGTSHLRNHLIRCQRRSNHGVAQYFSAKDKKKEGSLALVTIDQEQKNDEVLSIVNLRYEQEQIKSEHVAIGSNSLDQRRSQFDLARMIILHNYPLAMVEHDGFKIFVRNLQPLFELVTRNKVEADCMEIYAKEKQKVYEIFDKLPGKISVSADVWTASEDDAAYLSLAAHYIDENWQLKKKNLNFVTIDPSYAEDMHSEVIMNCLMDWDIDRKLFSMIFDSFTSDNIVERIRDRLSQNRFLHCNGQLFDVRCAVDLLNRMAHDALETLCEITQKIRESIRYVKSSEATQATFNELADEVQVETKKCLCIDNPLKWNSTYLMLEAASEYRKVFSCLRDRDPVNMKFLLSDPEWDRLITVTSFLKLFVEVTNVFTRSKYPTANIFFPEICDIHLQLIEWCKNPDEYISSLALKMRKKFEEYWYKCSSGLAVAAMLDPRFKMKLLEYYYPQLYGDSATELIDDVFECIKSLYNEHSMVSPLASSIDQGLDWQASGIPGSGKDSRDRLMGFDKFLHETSQAEGSSSDLDKYLEEPLFPRNVDFNVLNWWKVHTPRYPILSMMARNILGIPISKVAAESRFDTGGRMLNHNWSSLPPTTIQALMCSRDWIRSELES	zf-BED--DUF-domain--Dimer_Tnp_hAT--	I
Ghir_D04G004090.8	Ghi Zf-BED 55_I	MDMSDAVIVNSSRLKSIVWNDFDRVKKGDTFVAICRHCKKKLSGSSTSGTSHLRNHLIRCQRRSNHGVAQYFSAKDKKKEGSLALVTIDQEQKNDEVLSIVNLRYEQEQIKSEHVAIGSNSLDQRRSQFDLARMIILHNYPLAMVEHDGFKIFVRNLQPLFELVTRNKVEADCMEIYAKEKQKVYEIFDKLPGKISVSADVWTASEDDAAYLSLAAHYIDENWQLKKKNLNFVTIDPSYAEDMHSEVIMNCLMDWDIDRKLFSMIFDSFTSDNIVERIRDRLSQNRFLHCNGQLFDVRCAVDLLNRMAHDALETLCEITQKIRESIRYVKSSEATQATFNELADEVQVETKKCLCIDNPLKWNSTYLMLEAASEYRKVFSCLRDRDPVNMKFLLSDPEWDRLITVTSFLKLFVEVTNVFTRSKYPTANIFFPEICDIHLQLIEWCKNPDEYISSLALKMRKKFEEYWYKCSSGLAVAAMLDPRFKMKLLEYYYPQLYGDSATELIDDVFECIKSLYNEHSMVSPLASSIDQGLDWQASGIPGSGKDSRDRLMGFDKFLHETSQAEGSSSDLDKYLEEPLFPRNVDFNVLNWWKVHTPRYPILSMMARNILGIPISKVAAESRFDTGGRMLNHNWSSLPPTTIQALMCSRDWIRSELES	zf-BED--DUF-domain--Dimer_Tnp_hAT--	I
Ghir_D05G011710.1	Ghi Zf-BED 56_IV	MASSEVFINVRDHGKTVDVKKKRIKCNYCDKEMSGFSRLKYHLGGVRGNVLPCEKVPQDVKKLFRDMVQGREHLHNDAPYLYRQPFPQKRNGCPHNNVAKKTRHQSSESSGDESREYGNTDSMSEDDLEDSCCCYGDPNQESGKQNKRCIGRFFFETGTDFKLVNSLSFQRLMNDIRGPGWILKDEVKEIQEYVQKIRQSWGNTGCRIFFIADCPEGPIYLHSCDVSASVDDVNTLQLLLDRVMYEVGAENVVQVIAFSTTGWVGDVGKQFMERWKSVFWTVNASHCIELLLDEVVNMGDVQRTLEKAKTISKFIHDHVTVLNLWRDYMDGHDLIKPTKIKSAVPFVTLENIISEKRNITAMFTSSAWNNTTWSSTVEGKRVAKLVGDASFWRGAGMVVKLTLPLIRVLCLMHGEDKPQMGYIYETIDQVKETIEGCNSRKSEYMPFWKAIDEIWDGHLHSPLHAAGYFFNPSFFYSTDFQSDFEVGFGLLCCMVRMIQNQILSQTCVGASKYRLNRSLAEKLLTKGRDRTEEQLLSDLTFVHYNLQLQQQHSQLGVNYDIVADEIGPVNEWIVDDTAEIGSDNGDSNWKDLKSAVNGEGPSMYTSNFKRYHLFKMKLSKKM	zf-BED--DUF-domain	IV
Ghir_D06G005680.1	Ghi Zf-BED 57_I	MQSAGFTSMTEMTEITDMETIPGESNNQLALTTPEPQPIKRRKKKSMVWEYFTIENVSAGCRRAYCKRCKQSFAYSTGSKVAGTSHLKRHIAKGTCRALLRGQGQDNNQFITPYNPKMGGSEPPKRRYRSPSSPFIPFDQDRCRHEIARMIIMHEYPLHIVEHPGFIAFVQNLQPQFDKMSFNTVQGDCVATYLREKQSLMNFIEGIPGRFCLTLDMWSSNQTLGYVFITGHFVDSDWKLHRRVFNVVMEPYPDSHSALSHAIAACLSDWSLEGKLFSLTFNHPLSEAGLENLRPLLCVKNPLILNGQLLIRNCIARTMSSMAKDKIRDSVKYVKMSESHDDKFIQVKNQLQVPSEKSLFLDNQTQWNTTYQMLAAASELKEVFDCLDTYDPDYKLAPSMEDWKLAETLCSFLKPLFDAASILTTTTLPTVITFFYEVWKIHVDLGRSITCEDPFISNLAKSMQEKIDKYWKDCSLVLAMAVVMDPRFKMKLVEFSFTKIYGEDAPTYIKTVDDGIHELFLEYVALPLPLTPTYAEEVNGANNGKTNESHQGNLLSDHGLADFDVYIMETNSQQMKSELDQYLEESLLPRVQEFDVLGWWKLNKMKYPTLSKMARDILSIPVSAAATESIFDITDKQLDEYRSSLRPETVEALICAKDWLHYGSSDVSNALVRMEF	zf-BED--DUF-domain--Dimer_Tnp_hAT--	I
Ghir_D06G019310.1	Ghi Zf-BED 58_I	MTMASSNTPILVNDGFNEYESVVKRQKSTTSKVWDEMTKLECENKNELKVQCNHCKTIFSAKSSSETSHLRRHLNSCLKKVNKDIAQYIIATQPSPEGVPSIKNYKFDADECRQAISTFLVCGKHSFGTVEEPGFRYMMRIASPNFKNISRQTAIWDVLKYYAKERDHVKEELAKAPGLICLTSDNWNSEHTNDEYICITAHWVDKDWKLQKKIIRFRALFPPYDGLNIADELVLCLSQWGIDKKIFSITLDNASYNDVMVSCLKNYFRANRAILCDGAFFQVRCCVHILNLIVKAGLELADYVVCKIRNGIRYIKKSGIRRKRFYDVADKSFHLNVTKKLRQDVCVRWNSTYLMLESFLYYKDVLDYWGQRDKDYQMFALSSEEWRNVAILCKFLKVFYDVTCVFSGSNYPMANLYFRGVWKVHKLLIDIVKGPYSFLTSMVKQMQEKFNKYWAKYSLILSCAAILDPRYKLNYVQYCFKTIYGVHASDFVETILSNLRLLFDEYVKKSKSMSSSLAGSSNVSDKNPVDSGLDEHNDNSADFGGYFDESDDYKRYLNESSTRSEKSQLDIYLEESELELNSQIDVLDYWSKSSVRYNELSLLARDLLAIPISTVASESAFSMGKKVITPIRSSLKPKTVQAVVCLDDWMRAKGFSAGNYYSRFIVVILYYFFINLII	zf-BED--DUF-domain--Dimer_Tnp_hAT--	I

Ghir_D08G008310.1	GhiZf-BED59_II	MSTEPTSIEGNVTPPTSIDSENLGVGASSQTKGTTWNRKATPQKSEVWSHFTKIIISEDASKVKCNYCQKEFCCDMKRNVI<br>GSLKYHIGSCKKNPSNVVDTSQRQLVLPRKGRFDQEACRKGLAQMIVIDELPFKFVESEGFKKFIFVAYPRFHIPSRTNMI<br>RDVYQLYLDERVKIKQLLRSSCSRICLTIDTWTSLQRVNYLCITAHFIDNDWKLNKKILNFFPISSHKGESIGMVIEKCLLNW<br>GIDKLFTITVHNASSNDVAIGYLRKKFNPRGGLVQNGKYLHMRCMAYILNLIVVEGLKEMNKSVERIRGAVRYVRQSPARL<br>QKFKECVVAEKIKCKKMLCLDVCTRWNLTYLMLNTAQNFERAFEIFEKQDTNFRAELERERVGLVWMIGIMLET	zf-BED	II
Ghir_D08G009750.1	GhiZf-BED60_I	MAEITEATNMETTPVENNNELALITPETQPKRRKKKSMVWEYFTIETVSAGCRRACCNRCKQSFAYSTGSKVAGTSHLKR<br>HIAKGTCPALLHDQYNNQLTPYNPKTGGSEPRKRRYRSPSSPFIPFDQDRCRHEIARMIIMHEYPLHMVEHPGFIAFVQNL<br>QPRFDKVSFNTVQGDCVATYLREKQSLMKLIEGIPGRVCLTLDMWTSNQTLGYVFITGHFIDFEWKLQSRVLNVIMEPYP<br>DSDSALSHAVAACLSDWSLEGKLFSLTFNHPTSEAGLENLRPLLCTKNPLILNGQLLLGNCIARTLSSMAKDVLGAGHEVV<br>KKIRDSVKYVKTSESHDEKFVQVKNQLQVPSEKSLILDNQTQWNTTYQMLAAGSELKEVFNCLDTSDPDYKLAPSIEDWK<br>VAETLCTFLKPLFDAASILTTTTNPTAITFFHEAWKIHADLGRSITNEDPFISNIAKSMLEKIDKYWKDCSLILAIAVVMDPRFK<br>MKLVEFSFTKIFGEDAPTYIKIVDDGIHELFLEYVALPLPLTPTYTEEGNAGNNGKTDESQQGNLLSDQGLTDFDVYIMETS<br>SQQMKSELDQYLEESLLPRVQEFDVLGWWKLNKMKYPTLSKMARDILSIPVSAAAPDSVFDIIIKQLDEYRSSLRPETVEA<br>LICAKDWLHYGSEESNALVKMEF	zf-BED--DUF-domain--Dimer_Tnp_hAT--	I
Ghir_D08G025490.1	GhiZf-BED61_I	MEVANETVIKKPKRLTSVVWNHFERVRKADLCYAVCVHCNKKLSGSSNSGTTHLRNHLMRCLKRFNYDVSQLLSAKKRK<br>KENTLTIANISYDEGQRKEEYLKPTIVKYEPEQRKDEVFNVQSSWFDQDRSRLDLARMIILHGYPLAMVEHVGFKVFVKNL<br>QPLFDVVPNSTVELSCMEIYGKERQKVHDMLSKLQGRINLAVEMWSSPENTNHVCMMAHYIGDDWKLQKKILNFVTLDS<br>SHTDDLLSGVIIKCLMDWDIGSKLFAVTLDDFSTNDDIVLRIKEQILENKSRLSNGQLLDVRSAAHVLNSIVQDAMEALRVVL<br>QKIRGTVRYVKSSQSIQGKFKEMVLQTGINSQKNLVLDCPIRWNSTYLMLETAIEYRNAFCQLPDLDLDLALSDEEWEWA<br>SSITGYLKLFVEIINVFSSNKCPTANIYFPEICHVHIQLIDWCKSPDNFLSSLAAKMKAKFDKYWSKCSLSLAVAAILDPRFKM<br>KLVEYYYSQIYGSTALERIKEVSDGLKELFSTYSICSTLMDQGSALPLGSLPSSSNDGRDRLKGFDKFLHETSQSQTAISDL<br>EKYLDEPVFPRNCNFNILNWWRVHTPRYPILSMMARDVLGTPMSTVSQESAFHAGGRVLDSCRCPLTPETRQALICTQD<br>WLRIQSDDPGPSSSHYALPLYVETN	zf-BED--DUF-domain--Dimer_Tnp_hAT--	I
Ghir_D09G000090.1	GhiZf-BED62_I	MSKVWDEMTKLECENKNELKAQCNHCKTIFSAKSSSRTSHLRRHLNSCLKKVNKDITQYTIATQPSPEGVPSIKNYKFDP<br>DECRRAISTFIVCGKYSFRTVEEPGFRYMMRIASPNFKNISRHTAARDVLMYYAKERDRVKEELAKAPGLICLTSDNWNSE<br>HTNDEYICITAHWVDKDWKLQKRIIRFRALFPPYDGLNIADELVLCLSQWGIDKKIFSITLDNASYNDVMVSCLKNRFRANR<br>AILCDGAFFQVRCCAHILNLIVKAGLELADDVVGKIRNGIRYIKKSGIRRKRFYDVADKSFHLNVTKKLRQDVCVRWNSTYL<br>MIKSSLYYKDVLDYWGQRDKDYQMFALSNEEWRNVAILCKFLKVFYDVTCIFSGSNYPTTNLYFRGVWKVHKVLLDTVKG<br>PYSFLTPMVKQMQEKFNKYWAEYSLILSCAAILDPRYKLNYVQYCFNTIYGIHASDFVETILCNLRLLFDEYVKKSKSTSSS<br>LAESSNVSNKNPVDSGLDEHNDNSADFVGYFDESNDYKRYLNESSTRSEKSQLDIYLEEPELELNSQIDVLDYWSKSSVR<br>YNELSLLARDLLAIPISTVASKSAFSMGKKVITPLRSSLKPKTVQAVVCLDDWMRAKGFFNRNWLQKGR	zf-BED--DUF-domain--Dimer_Tnp_hAT--	I
Ghir_D11G020300.1	GhiZf-BED63_I	MSTKPTSIEGSVTPPTSIDSENSGVGASIQTKGTTGKRKAPPQRSEVWSHFTKFINSEGASKAKCNYCEKEFCCDMKKNG<br>TGSLKYHIGSCKKNPSNVVDTSQGQLVLPRKGVEGGEGNISTWRFDQEACRKGLAQMILIDELPFKFVESEGFKKFMFVA<br>CPRFHIPSRTTMTRDVYQLYLDERVKIKQLLRSSCSRLNLCLTAHFIDNDWKLNKKILNFCPISSHKGESIGMVIEKCLLNW<br>GIDKLFTVTVDNASSNDVAIGYLRKKFNPRGGLVQNGKYLHMRCMAHIVNLIVVEGLKEMNKSVERVRGLLQKFKECVVV<br>EKIECKKMLCLDVCTRWNSTYLMLDTAQNFERAFERFEEQDTNFRAELERGEGWPSVDDWDNVRNLRDFLEHFYEVTL<br>RISGTSYVTSNNFFDELSEIDILLRDAQLNSNIDFNVMAIKMKEKYDKYWGDIDKMNLLMFVACVLDPRQKLKYLEFALSEM<br>SSSEKACEMMQKLKESLYELFDEYKPPLHSTCSQSSVSTHVSIGEPQQKMKRRMQASYKKHELEICGEDKTSELDKYLA<br>ETNEEFVEDFDILLWWKVNSPRFPTLSKIARDVLAIPVSTVASESAFSTGGRVLDQYRSSLTPKIVQALVCTQDWIRRSSS<br>QEDIKKIEEQIQELDKIENGMFIVLF	zf-BED--DUF-domain--Dimer_Tnp_hAT--	I
Ghir_D11G027530.1	GhiZf-BED64_I	MSIEPTSIEGRVTPPTSIDSENSGVGASIQTKGTTGKRKAPPQRSAVWSHFTKFINSEGASKAKCNYCEKEFCCDMKKNG<br>TGSLKYHIGSCKKNPSNVVDTSQGQLVLPRKGVEGGEGNLSTWRFDQEACRKGLAQMILIDELLFKFVESEGFKKFMFVA<br>CPRFHIPPRTIMTRDVYQLYLDERVKIKKLLRSSCSRVCLTTDTWTSLQRVNYLCITAHFIDNDWKLNKKILNFCPISSHKGE<br>SIGMVIEKCLLNWGIDKLFTITVDNASSNDVAIGYLRKKFNPRGGLVQNGKYLHMRCMAHIVNLIVVEGLKEMNKSVERVR<br>GAVRYVRQSPARLQKFKECVVVERIECKKMLCLDVCTRWNLTYLMLDTAQNFERALKRFEEQDTNFRAELERGEGWPS<br>VDDWDNVRKLRDFLEHFYEVTLRISGTSYLNSNIDFNVMAIKMKEKYDKYWGDIDKMNLLMFVACVLDPRQKLKYLEFAL<br>SEMSSSEKACEMMQKLKESLYELFDEYKPPLHSTCSQSSSSRELEICGEDKTSELDKYLAEANEEFVEDFDILLWWKVNS<br>PRFPTLSKIARDVLAIPVSTVASEFAFNTGGRVLDQYRSSLTPKIVQALVCTQDWIRRSSSQEDIKKIKEQIQELDKIENGIQ<br>YMFVIHFMTYLGIINSWGDWTIIG	zf-BED--DUF-domain--Dimer_Tnp_hAT--	I
Ghir_D11G032010.1	GhiZf-BED65_I	MEVANESTAKKPKRLTSVVWNHFERVKKADICYAVCVHCNKKLSGSSNSGTTHLRNHLMRCLKRSNYDVSQLLAVKRRK<br>KENTLTIANISYDEGQRKEDYMKPTIVKYEQDQRKDEAFNLGSSWFDPERSRLDLARMIILHGYPLAMVEHVGFKVFVKN<br>MQPLFDVVHNSTIELSCVEIYMKEKQRIYDMLSKLQGRINLAIEMWSSPENSKYVCLTAHYVDDEWKLQKKILNFLTLDSS<br>HTEDMLSDVIIKCLMDWDIDCKLFSMTFDDCSTNDDIVLRIKDQISESRPRLSNGQLLDVRSAAHVLNSIAQDAIEALQVVIQ<br>KIRGSVKYVKSSQSILGKFNEIAQQQGINNHKIVVLDYPIRWNSTYMMLETAVEYRNVFHHLPELDPDFALSDEEWKRASS<br>IVSYLKLLIEIINVFSSNKCPTANIYFPEICHVHIQLIEWCKSSDAFLSSLATKMKAKFDKYWSKCSLGLAVAAILDPRFKMKLV<br>EYYYSQIYGSTALERIKEASDGIKELFNAYSICSTLIDQGSALPGSSLPSSSNDTRDKLKGFDKFLHETSQSQTAISDLEKYL<br>DEPMFPRNCDFNILNWWRVHTPRYPILSMMARDVLGTPMSTVAQEFAFNAGGRILDSNQSSLPPDTRQALICTRDWLRT<br>QSDDATPSSSHYALPLYVEAN	zf-BED--DUF-domain--Dimer_Tnp_hAT--	I
Ghir_D11G032010.2	GhiZf-BED66_I	MEVANESTAKKPKRLTSVVWNHFERVKKADICYAVCVHCNKKLSGSSNSGTTHLRNHLMRCLKRSNYDVSQLLAVKRRK<br>KENTLTIANISYDEGQRKEDYMKPTIVKYEQDQRKDEAFNLGSSWFDPERSRLDLARMIILHGYPLAMVEHVGFKVFVKN<br>MQPLFDVVHNSTIELSCVEIYMKEKQRIYDMLSKLQGRINLAIEMWSSPENSKYVCLTAHYVDDEWKLQKKILNFLTLDSS<br>HTEDMLSDVIIKCLMDWDIDCKLFSMTFDDCSTNDDIVLRIKDQISESRPRLSNGQLLDVRSAAHVLNSIAQDAIEALQVVIQ<br>KIRGSVKYVKSSQSILGKFNEIAQQQGINNHKIVVLDYPIRWNSTYMMLETAVEYRNVFHHLPELDPDFALSDEEWKRASS<br>IVSYLKLLIEIINVFSSNKCPTANIYFPEICHVHIQLIEWCKSSDAFLSSLATKMKAKFDKYWSKCSLGLAVAAILDPRFKMKLV<br>EYYYSQIYGSTALERIKEASDGIKELFNAYSICSTLIDQGSALPGSSLPSSSNDTRDKLKGFDKFLHETSQSQTAISDLEKYL<br>DEPMFPRNCDFNILNWWRVHTPRYPILSMMARDVLGTPMSTVAQEFAFNAGGRILDSNQSSLPPDTRQALICTRDWLRT<br>QSDDATPSSSHYALPLYVEAN	zf-BED--DUF-domain--Dimer_Tnp_hAT--	I
Ghir_D12G013240.1	GhiZf-BED67_III	MVEEMAPLRSIGYVDPGWEHGTAQDERKKKVKCNYCGKVVSGGIFRLKQHLARLSGEVTHCEKVPEEVCLNMRKNLEG<br>CRSGRKRRQLDYEQAALSIQSNEYSDGEDASASYKHKGKKVMGDKNLVIKFTPLRSLGYVDPGWEHCVAQDEKKRRVK<br>CNYCEKIISGGINRFKQHLARIPGEVAYCEKAPEEVYLKIKENMKWHRTGRRHRKPDTKEISTFYMHSDNEDEGGEEEGY<br>LQCISKDILAIDDKVSDNDIRNNVRGRSPGSSGNGAEPLLKKSRLDSVFLKSLKSQTSTHHKQPRARTGFEKKTHREVISAI<br>CKFFYHAGIPSNAANSPYFHKMLELVGQYGQGLQGPSSRLISGRLLQEEIANIKEYLVELKTSWAITGCSVMADSWNDAQ<br>GRMLINFLVSCPRGVYFLSSVDATDIIEDAVHLFKLLDKAVDEVGEEYVVQVITRNTLSFRNAGKMLEEKRRNLFWTPCAV<br>YCIDRMLEDFVNIKWVGECVDKAKKVTRFIYNNTWLLNFMKKEFTKGQELLQPAVTKFGTNFFTLQSLLDQRVGLKRMFQ<br>SNRWLSSRFSKSDEGKEVEKIVLNVSFWKKMQYVKKSFEPVAEVLQRIGSDKIRSLPFIYNDICRTKLAIKAIHGDDVRKYG<br>PFWSVIESNWSPLFHHPLYVAAYFLNPSYRYRPDFLMNPEVIRGLNGCIVRLEADNGKKIAASMQIPDFVSAKADFGTDLA<br>ISTRSELDPASWWQQHGISCLELQRIAIRILSQTCSSIGCEHNWSAFDQVHIKRHNCLSRKRLNDQTYVHYNLRLRERQLG<br>RKPDELVSFDSAMLESVLDDWLVETEKLAMHEDEEIIYTEVEQFCGDDMDEHESEEKRPAEMVTIAGFIEPLDVIPSAGGV<br>TTDDDGLDFLDDDLTD	zf-BED--zf-BED--DUF-domain--Dimer_Tnp_hAT--	III
Ghir_D12G013240.2	GhiZf-BED68_III	MVEEMAPLRSIGYVDPGWEHGTAQDERKKKVKCNYCGKVVSGGIFRLKQHLARLSGEVTHCEKVPEEVCLNMRKNLEG<br>CRSGRKRRQLDYEQAALSIQSNEYSDGEDASASYKHKGKKVMGDKNLVIKFTPLRSLGYVDPGWEHCVAQDEKKRRVK<br>CNYCEKIISGGINRFKQHLARIPGEVAYCEKAPEEVYLKIKENMKWHRTGRRHRKPDTKEISTFYMHSDNEDEGGEEEGY<br>LQCISKDILAIDDKVSDNDIRNNVRGRSPGSSGNGAEPLLKKSRLDSVFLKSLKSQTSTHHKQPRARTGFEKKTHREVISAI<br>CKFFYHAGIPSNAANSPYFHKMLELVGQYGQGLQGPSSRLISGRLLQEEIANIKEYLVELKTSWAITGCSVMADSWNDAQ<br>GRMLINFLVSCPRGVYFLSSVDATDIIEDAVHLFKLLDKAVDEVGEEYVVQVITRNTLSFRNAGKMLEEKRRNLFWTPCAV<br>YCIDRMLEDFVNIKWVGECVDKAKKVTRFIYNNTWLLNFMKKEFTKGQELLQPAVTKFGTNFFTLQSLLDQRVGLKRMFQ<br>SNRWLSSRFSKSDEGKEVEKIVLNVSFWKKMQYVKKSFEPVAEVLQRIGSDKIRSLPFIYNDICRTKLAIKAIHGDDVRKYG<br>PFWSVIESNWSPLFHHPLYVAAYFLNPSYRYRPDFLMNPEVIRGLNGCIVRLEADNGKKIAASMQIPDFVSAKADFGTDLA<br>ISTRSELDPASWWQQHGISCLELQRIAIRILSQTCSSIGCEHNWSAFDQVHIKRHNCLSRKRLNDQTYVHYNLRLRERQLG<br>RKPDELVSFDSAMLESVLDDWLVETEKLAMHEDEEIIYTEVEQFCGDDMDEHESEEKRPAEMVTIAGFIEPLDVIPSAGGV<br>TTDDDGLDFLDDDLTD	zf-BED--zf-BED--DUF-domain--Dimer_Tnp_hAT--	III

Ghir_D12G013240.3	GhiZf-BED69_III	MVEEMAPLRSIGYVDPGWEHGTAQDERKKKVKCNYCGKVVSGGIFRLKQHLARLSGEVTHCEKVPEEVCLNMRKNLEGCRSGRKRRQLDYEQAALSIQSNEYSDGEDASASYKHKGKKVMGDKNLVIKFTPLRSLGYVDPGWEHCVAQDEKKRRVKCNYCEKIISGGINRFKQHLARIPGEVAYCEKAPEEVYLKIKENMKWHRTGRRHRKPDTKEISTFYMHSDNEDEGGEEEGYLQCISKDILAIDDKVSDNDIRNNVRGRSPGSSGNGAEPLLKKSRLDSVFLKSLKSQTSTHHKQPRARTGFEKKTHREVISAICKFFYHAGIPSNAANSPYFHKMLELVGQYGQGLQGPSSRLISGRLLQEEIANIKEYLVELKTSWAITGCSVMADSWNDAQGRMLINFLVSCPRGVYFLSSVDATDIIEDAVHLFKLLDKAVDEVGEEYVVQVITRNTLSFRNAGKMLEEKRRNLFWTPCAVYCIDRMLEDFVNIKWVGECVDKAKKVTRFIYNNTWLLNFMKKEFTKGQELLQPAVTKFGTNFFTLQSLLDQRVGLKRMFQSNRWLSSRFSKSDEGKEVEKIVLNVSFWKKMQYVKKSFEPVAEVLQRIGSDKIRSLPFIYNDICRTKLAIKAIHGDDVRKYGPFWSVIESNWSPLFHHPLYVAAYFLNPSYRYRPDFLMNPEVIRGLNGCIVRLEADNGKKIAASMQIPDFVSAKADFGTDLAISTRSELDPASWWQQHGISCLELQRIAIRILSQTCSSIGCEHNWSAFDQVHIKRHNCLSRKRLNDQTYVHYNLRLRERQLGRKPDELVSFDSAMLESVLDDWLVETEKLAMHEDEEIIYTEVEQFCGDDMDEHESEEKRPAEMVTIAGFIEPLDVIPSAGGVTTDDDGLDFLDDDLTD	zf-BED--zf-BED--DUF-domain--Dimer_Tnp_hAT--	III
Ghir_D12G013240.4	GhiZf-BED70_III	MVEEMAPLRSIGYVDPGWEHGTAQDERKKKVKCNYCGKVVSGGIFRLKQHLARLSGEVTHCEKVPEEVCLNMRKNLEGCRSGRKRRQLDYEQAALSIQSNEYSDGEDASASYKHKGKKVMGDKNLVIKFTPLRSLGYVDPGWEHCVAQDEKKRRVKCNYCEKIISGGINRFKQHLARIPGEVAYCEKAPEEVYLKIKENMKWHRTGRRHRKPDTKEISTFYMHSDNEDEGGEEEGYLQCISKDILAIDDKVSDNDIRNNVRGRSPGSSGNGAEPLLKKSRLDSVFLKSLKSQTSTHHKQPRARTGFEKKTHREVISAICKFFYHAGIPSNAANSPYFHKMLELVGQYGQGLQGPSSRLISGRLLQEEIANIKEYLVELKTSWAITGCSVMADSWNDAQGRMLINFLVSCPRGVYFLSSVDATDIIEDAVHLFKLLDKAVDEVGEEYVVQVITRNTLSFRNAGKMLEEKRRNLFWTPCAVYCIDRMLEDFVNIKWVGECVDKAKKVTRFIYNNTWLLNFMKKEFTKGQELLQPAVTKFGTNFFTLQSLLDQRVGLKRMFQSNRWLSSRFSKSDEGKEVEKIVLNVSFWKKMQYVKKSFEPVAEVLQRIGSDKIRSLPFIYNDICRTKLAIKAIHGDDVRKYGPFWSVIESNWSPLFHHPLYVAAYFLNPSYRYRPDFLMNPEVIRGLNGCIVRLEADNGKKIAASMQIPDFVSAKADFGTDLAISTRSELDPASWWQQHGISCLELQRIAIRILSQTCSSIGCEHNWSAFDQVHIKRHNCLSRKRLNDQTYVHYNLRLRERQLGRKPDELVSFDSAMLESVLDDWLVETEKLAMHEDEEIIYTEVEQFCGDDMDEHESEEKRPAEMVTIAGFIEPLDVIPSAGGVTTDDDGLDFLDDDLTD	zf-BED--zf-BED--DUF-domain--Dimer_Tnp_hAT--	III
Ghir_D13G002100.1	GhiZf-BED71_I	MSSNLEPIPITSQKHDPAWKHCQMFKNGERVQLKCIYCGKIFKGGGIHRIKEHLAGHKGNAATCLRVPSNVRVLMQESLDGVVVKKRKKQKIAEEITNVNQVSTEIQAYADQVDTNTGLLMIEKSDTLEPSSSLLVNQEGTSNVAGERRKRGRGKSLPAEANALSFVPVELGARRVNNHVHMAIGRFLFDIGATMDAVNSVYFQPMVDAIVSGGSGALMPSCNDLQGWILRKSVEEVKSENEKVMAAWVRTGCSILVNQWNTQTGRILLNFLVYCPEGTVFLKPIDASSVINSSDALYELLKQVVEEVGSKHVLQVITNGEEQYIVAGRRLVETFPTLYWAPCAAHCVDLILEDFAKLEWINAIIEQARSITKFIYNHSVVLNMVRRYTFGNDIVEPAATRSATNFTTLTRMVDLKNNLQAMVTSQQWVDCPYSKKPGGLAMLDLVSNQSFWSSCVLIVRLTNPLLRVLRMAGSKKRPAMGYVYAGMYRAKETIKKELVKRNEYMVYWNIIDHWWEQQWHHPLHAAGFYLNPRFFYSMEGDMPNEMLSGMLDCIEKLIPDVTVQDKITKEINSYKNSIGDFGRKMAVRARDTLLPVEWWSTYGGSCPNLARLAIRVLSQTCSTLGLKHDHIPFEKLHETRNCLEQQRLRDLIFVQCNLQLRQIGYESKQHDSMQPLSSESASIVEDWVTGIGAFLDDDTYPDWTTLETLSVNTMPLRPGDEVEELGAGFNDHEIFNRMKEGDNEKAEDNVVS	zf-BED--DUF-domain--Dimer_Tnp_hAT--	I
Ghir_D13G002100.2	GhiZf-BED72_I	MSSNLEPIPITSQKHDPAWKHCQMFKNGERVQLKCIYCGKIFKGGGIHRIKEHLAGHKGNAATCLRVPSNVRVLMQESLDGVVVKKRKKQKIAEEITNVNQVSTEIQAYADQVDTNTGLLMIEKSDTLEPSSSLLVNQEGTSNVAGERRKRGRGKSLPAEANALSFVPVELGARRVNNHVHMAIGRFLFDIGATMDAVNSVYFQPMVDAIVSGGSGALMPSCNDLQGWILRKSVEEVKSENEKVMAAWVRTGCSILVNQWNTQTGRILLNFLVYCPEGTVFLKPIDASSVINSSDALYELLKQVVEEVGSKHVLQVITNGEEQYIVAGRRLVETFPTLYWAPCAAHCVDLILEDFAKLEWINAIIEQARSITKFIYNHSVVLNMVRRYTFGNDIVEPAATRSATNFTTLTRMVDLKNNLQAMVTSQQWVDCPYSKKPGGLAMLDLVSNQSFWSSCVLIVRLTNPLLRVLRMAGSKKRPAMGYVYAGMYRAKETIKKELVKRNEYMVYWNIIDHWWEQQWHHPLHAAGFYLNPRFFYSMEGDMPNEMLSGMLDCIEKLIPDVTVQDKITKEINSYKNSIGDFGRKMAVRARDTLLPVEWWSTYGGSCPNLARLAIRVLSQTCSTLGLKHDHIPFEKLHETRNCLEQQRLRDLIFVQCNLQLRQIGYESKQHDSMQPLSSESASIVEDWVTGIGAFLDDDTYPDWTTLETLSVNTMPLRPGDEVEELGAGFNDHEIFNRMKEGDNEKAEDNVVS	zf-BED--DUF-domain--Dimer_Tnp_hAT--	I
Ghir_D13G017940.1	GhiZf-BED73_I	MNFNLLYFFSCIRSIKPTSIEGSVTPPTSIDSENSGVGASSQTKGTTGKRKATQRSEVWSHFTKIINSEGASKAKCNYCQKEFCCDMKKNGTGSLKYHIGSCKKNPSNVVDNSQGQLVLPRKETCRKGLAQMIVIDELPFKFVESESFKKFTFVACPRFHIPSRTTMTRDVYQLYLDEKIKIKQLLRSSCSRVCLTTDTWTSLQRVNYLCITTHFIDNDWKLNKKILNFCPISSHKGESIGMVIEKCLLNWGIDKLFIVTVDNASSNHVAIGYLRNKFNPRGGLVQNGKYLHMRCMAHIVNLIVVQGLKEMNKSVERVRGLIQKFKECVVVEKIECKKMLCLDVCTRWNLTYLMLDTAQNFERAFERFEEQDTNFRAELERGEGWPSVDDWDNVRNLRDFLEHFYEVTLRISGTSYVTSNNFFDELSEIDILLRDAQLKSNVDFSVMAIKMKEKYDKYWGDIDKMNLLMFVACILDPRQKLKYLKFALSEMSSSEKACEMMQKLKESLYELFDEYKPPLHSTCSQLSVPTHVFLGEPQQKMKRRMQALYKKRELEICGEDKTSELDKYLAEANEEFVEDFEILLWWKVNSPRFPTLSKMARDVLAIPVSTVASESAFSTRGRVLDQYRSSLTPKSVQALACTQDWI	zf-BED--DUF-domain--Dimer_Tnp_hAT--	I
Gothu.00005298-RA	GotZf-BED01_IV	MYLQRAKHCDVDVVVAICLQNRYTSNDVYVVEFYWPATESEISKSFTPRIFNDLKYMEKKFVTVKVQGTEKAISNIPTSSYTARPLKIAEETEDVDAVEINGVNVQRGVVPNFPSPITIQSSSKVAAAPSNTLEGPHNQIVKSNKEEPSKATLRELRSKVWDHFDRFEEDEKQVAKCKHCPKVLTGSSKSGTTHLNNHSKVCPGKKKQNQESQLILPVDTNEGSLRFDKKRSHMDLAKMMIKLQCPLDMAEQETFKNFVKGLQPMFEFQSKDILSYIHRIYDEEKEKLQLYFDKLASKFNLTVSLLKNNSGKTIYCCLISHFIDDGWELKRKILALKTLEHINDTKALGEIIRSLVLEWNISNKVCSITVDNSFLNDSMVDQIKEICLSDQGSVSSDHWFISFTLLEDGFREMDGILFKLRKSIEYVTETRHGKLKFQEAVDQVKLQGGKLWDDLSFRLESDFDILDSALRSREIFCKLEQIDDNFKLNPTMEEWENAVALQSCLKCFDDIKGTQCLPRKFDRYWSLCNLALAVASVLDPRLKFKIVELSYRVIYGHDSKMRLNMFHKFLRDVYYEHASEAKNLTPSASVLDDFNCSTIGLGNDSILDSLSQFASASNFNEEASWKLELELYLDEPLLPMDGAFFDMVGGVINLKGFQYLQRWLKISLQF	zf-BED--DUF-domain	IV
Gothu.00005299-RA	GotZf-BED02_I	MLEDVCFRYELPVALTWACEANTDKIMLDGKKYTLFMERTSCYASNEGSQSFMEACAKHHIQEGQAIAGKALQSSANFHFEPSITKLIKSDYPLFNAAQLFGSHAVVAICLQNHYIIGDVYVVEFYWPEIESEKSESLALDIFNDLKNMKKKFVTIRVGSNEVGFEREAISTTLQGTMHTRNAQPASSTNDLLSSNTTWSLNAVQPCDVHEMERHGLVEQVESAPFSTPNPMSYGGVLQTQGPHKQEIGEKDFISQTVSIGDYEIVKAYMETCKVPRTKQRKYLSKVWLDFDKFEVNGKQVAKCKHCNKDFTGSSKSGTTHLKNHLERCQSKKIKNQERQLITSEIGDLITRDSDESNLTFDQERSRLDFAKMIIKHQCPLDMAEQEFFKIFVKNLQPMFEFQSKDILLSDIHRIYKEEKEKRQLYFDQLACNFNLTISLWKKNLGKTAYCCLIAHFIDDNWGPKMKIIACKPLEHIYDTKASNEIIQSSVLEWNISKKVFSITMDNPYLSDDMFQKIKETCFSDQGSFPSTHWFIGCTFIKDGFHEMDLILLKLRKSIEYVSEIAQGKLKFEEVVNQVKLQGGKSWDDLSLRLDSDFGVLHSALESREIFCQLEKIDSNFKLNPSVEEWEMVLAFHSCLKCFDDIEGTQSLTANLYFPKLCNIYKKFLHLGKSNYPIVTLMKRKFDYYWSLCNLAFAVATILDPRLKFKFVEFSYTEIYGHDSKMHLNRFLKVLTDVYYEYANEARNLSKSTSDLDDSNSSTTEIDNDCILESFSKFASASKFNEVASWKSELDYYLDEPLLPLDGAFDILYWWRINTKRFPTLAKMARDFLAMPISILAPCLNFNAMITNQTYNNLNPESMEALYKRKRKMEDHSNVVEVSKNWNREEANSSGDIAKGSIKNEPNHGRNITALNEIPKDDSPFNNNKSGQFQSLSSESDNEITLKEQGSWCKEDVRAYLVSRFTGKENKRLNRWKTNELIGKLIGRDKEFCLMGDKLVPLLMVPHGDETRKEYYIDDS	zf-BED--DUF-domain--Dimer_Tnp_hAT--	I
Gothu.00010173-RA	GotZf-BED03_I	MAEITEATNMETTPVENNNELALITPETQPKRRKKKSMVWEYFTIETVSAGCRRACCNRCKQSFAYSTGSKVAGTSHLKRHIAKGTCPALLRDQYNNQLTPYNPKTGGSEPRKRRYRSPSSPFIPFDQDRCRHEIARMIIMHEYPLHMVEHPGFIAFVQNLQPRFDKVSFNTVQGDCVATYLREKQSLMKLIEGIPGRVCLTLDMWTSNQTLGYVFITGHFIDFEWKLQSRVLNVIMEPYPDSDSALSHAVAACLSDWSLEGKLFSLTFNHPTSEAGLENLRPLLCTKNPLILNGQLLLGNCIARTLSSMAKDVLGAGHEIVKKIRDSVKYVKTSESHDEKFVQVKNQLQVPSEKSLILDNQTQWNTTYQMLATASELKEVFNCLDTSDPDYKLAPSMEDWKVAETLCTFLKPLFDAASILTTTTNPTAITFFHEAWKIHADLGRSITNEDPFISNIAKSMLEKIDKYWKDCSLILAIAVVMDPRFKMKLVEFSFTKIFGEDAPTYIKIVDDGIHELFLEYVALPLPLTPTYTEEGNAGNNGKTDESQQGNLLSDQGLTDFDVYIMETSSQQMKSELDQYLEESLLPRVQEFDVLGWWKLNKMKYPTLSKMARDILSIPVSAAAPDSVFDIIIKQLDEYRSSLRPETVEALICAKDWLHYGSEESNALVKMEF	zf-BED--DUF-domain--Dimer_Tnp_hAT--	I
Gothu.00025092-RA	GotZf-BED04_I	METTPVENNNELALITPETQPKRRKKKSMVWEYFTIETVSAGCRRACCNRCKQSFAYSTGSKVAGTSHLKRHIAKGTCPALLRDQYNNQLTPYNPKTGGSEPRKRRYRSPSSPFIPFDQDRCRHEIARMIIMHEYPLHMVEHPGFIAFVQNLQPRFDKVSFNTVQGDCVATYLREKQSLMKLIEGIPGRVCLTLDMWTSNQTLGYVFITGHFIDFEWKLQSRVLNVIMEPYPDSDSALSHAVAACLSDWSLEGKLFSLTFNHPTSEAGLENLRPLLCTKNPLILNGQLLLGNCIARTLSSMAKDVLGAGHEIVKKIRDSVKYVKTSESHDEKFVQVKNQLQVPSEKSLILDNQTQWNTTYQMLATASELKEVFNCLDTSDPDYKLAPSMEDWKVAETLCTFLKPLFDAASILTTTTNPTAITFFHEAWKIHADLGRSITNEDPFISNIAKSMLEKIDKYWKDCSLILAIAVVMDPRFKMKLVEFSFTKIFGEDAPTYIKIVDDGIHELFLEYVALPLPLTPTYTEEGNAGNNGKTDESQQGNLLSDQGLTDFDVYIMETSSQQMKSELDQYLEESLLPRVQEFDVLGWWKLNKMKYPTLSKMARDILSIPVSAAAPDSVFDIIIKQLDEYRSSLRPETVEALICAKDWLHYGSEESNALVKMEF	zf-BED--DUF-domain--Dimer_Tnp_hAT--	I

Gothu.00033101-RA	GotZf-BED05_XV	MAPLRSIGYVDPGWEHGTAQDERKKKVKCNYCGKVVSGGIFRLKQHLARLSGEVTHCEKVPEEVCLNMRKNLEGCRSGRKRRQLDYEQAALSIQSNDYSDGEDASASYKHKGKKVMGDKNLVIKFTPLRSLGYVDPGWEHCVAQDEKKRRVKCNYCEKIISGGINRFKQHLARIPGEVAYCEKAPEEVYLKIKENMKWHRTGRRHRKPDTKEISTFYMHSDNEDEGGEEEGYLQCISKDILAIDDKVSDNDIRNNVRGRSPGSSGNGAEPLLKKSRLDSVFLKSLKSQTSAHYKQPRARTGFEKKTHREVISAICKFFYHAGIPSNAANSPYFHKMLELVGQYGQGLQGPSSRLISGRLLQEEIANIKEYLVELKTSWAITGCSVMADSWNDAQGRMLINFLVSCPRGVYFLSSVDATDIMEDAVHLFKLLDKAVDEVGEEYVVQVITRNTLSFRNAGKMLEEKRRNLFWTPCAVYCIDRMLEDFVNIKWVGECVDKAKKVTRFIYNNTWLLNFMKKEFTKGQELLQPAVTKFGTNFFTLQSLLDQRVGLKRMFQSNRWLSSRFSKSDEGKEVEKIVLNVSFWKKMQYVKKSFEPVAEVLQRIGSDKIRSLPFIYNDICRTKLAIKAIHGDDVRKYGPFWSVIESNWSPLFHHPLYVAAYFLNPSYRYRPDFLTNPEVIRGLNGCIVRLEADNGKKIAASMQIPDFVSAKADFGTDLAISTRSELDPGDSIRTLCTNLLMVATTWDKLLRAATNRHTHTEPDMFINRVHIKRHNCLSRKRLNDQTYVHYNLRLRERQLGRKPDELVSFDSAMLESVLDDWLVETEKLTMHEDEEIIYTEVEQFCGDDMDEHESEEKRPAEMVTIAGFIEPLDVIPSAGGITTDDDGLVESKKRNVLTIHSSPSWTEFLSQKLCCFYREINAKASANVSPSLYWMSYQVLAQTESTVSVAIWKLQRKCYTKFPNLPSAKWNTLWSSVEHGSRSRYNSGTS	zf-BED--zf-BED--DUF-domain--	XV
Gothu.00036003-RA	GotZf-BED06_I	MELNLVPISITRQKQDPAWNHCEVFKNGERLQIKCMYCGKLFKGGGIHRFKEHLAGRKGQGPICEQVPQGVRSVMQESLNGILVKQDKKQKLIPKLLACGSSSSNPNIGGEVENLGSHDDMNFGIKPISVLNTLEGDSNVVSKVGRGRKRGRGRGRDRNLIESNYPCVKTDLALVPNGGENPIHMAIGRFLYDIGVNLDAVNSVCFQPMIDAIASGGSGVVPPSCHDLRGWILKNVIEEVKDDIDRNKAMWGKTGCSIIVEQCRTKSERVLLSFLVYCPQATVFMKSVDASHAVYSADYLFEVLKQVIEEVGSEKVVQVITNCEEPYLFTGKRLMESFPSLYWAPCLAHCVDLMLQDFSNLEWINETIEQAKSLTRFIYNQSSVLNMMRKFTSGNDVVEQALTCFATNFSTLKRMADLKLNLQAMVNSQDWLECPYAKKPGGQAMSDIVNNRSFWNSCMLIARITYPLLQVLEIVGSKKRSAMGYVYAGIYRAKETIKKELVKQDDYMVYWNIIDNRWEQQRHLPLYAAGFFLNPKLFYNTEEHIHNDILSSVFDSIERLVPDTNIQDQVVREINLYKNATGDLGRPMAVRARDNLLPGEWWSIYGGGCPNLQRLAIRILSQTCSSIGYKPSKISIEEIHNTRNFLERQRLSDLVFVQYNLYLRQMVLQKQEKDSLDPLAFNNKDILEDWIADNEVSPDNLESSDWKSLDPPVGNRTTLTPPGDEAEDFLSTKFTDLDIFNGLKGVKEEI	zf-BED--DUF-domain--Dimer_Tnp_hAT--	I
Gothu.00040989-RA	GotZf-BED07_II	MIMYCSIFLAVVRERDVCWEYAEKLDGNKVRCKFCLRVLNGGISRLKHHLSRLPSKGVNPCNKVRDDVTDRVRAIISSKEDIKETPSVKKQKIAEVRAPGNMSTSSKISPLETLSPAAKVFPTVLSIAASTLSDQETVERSIALFLFENKLDFSVARSSSYQAMIDAVGKFGPGLIAPSELIRTGITKSVSCFLSLQSMLKQRSRLKHMFNSPEYSTNSSYANKPQSISCIAIVEDNDFWRAVEECVAISEPFLKVLREVSGGKPAVGSIYELMTRAKESIRTYYIMDESKCKTFLDIVDRQWRDQLHSPLHSAGAFLNPSIQYNPEVKFLGSIKEDFFKVLEKLLPTPELRRDITNQIFTFTRAKGMFACNLAMEARDTVSPEESENPSPTQWLDRFGSALDGGDLNTRQFSAAMFGNDHIFGL	zf-BED	II
Gothu.00044750-RA	GotZf-BED08_I	MSDAVIVNSSRLKSIVWNDFDRVKKGDTFVAICRHCKKKLSGSSTSGTSHLRNHLIRCQRRSNHGVAQYFSAKDKKKEGSLALVTIDQEQKNDEVLSIVNLRYEQEQIKSEHVGIGSNSLDQRRSQFDLARMIILHNYPLAMVEHVGFKIFVRNLQPLFELVTRNKVEADCMEIYAKEKQKVYEIFDKLPGKISVSADVWTASEDDAAYLSLAAHYIDENWQLKKKNLNFVTIDPSYTEDMHSEVIMNCLMDWDIDRKLFSMIFDSFTSDNIVERIRDRLSQNRFLHCNGQLFDVRCAVDLLNRMAHDALEALCEITQKIRESIRYVKSSEATQATFNELADEAQVETKKCLCIDNPLKWNLTYFMLEAALEYRKVFSCLRDRDPVNMKFLLSDPEWDRLITVTSFLKLFVEVTNVFTRSKYPTANIFFPEICDIHLQLIEWCKNPDEYISSLALKMRKKFEEYWYKCSSGLAVAAMLDPRFKMKLLEYYYPQLYGDSATELIDDVFECIKSLYNEHSIVSPLASSIDQGLDWQASGIPGSGKDSRDRLMGFDKFLHETSQAEGSSSDLDKYLEEPLFPRNVDFNVLNWWKVHTPRYPILSMMARNILGIPISKVAAESRFDTGGRMLNHNWSSLSPTTIQALMCSQDWIRSELES	zf-BED--DUF-domain--Dimer_Tnp_hAT--	I
GSVIVT01007419001	GsvZf-BED01_I	MYNTKWSCLSTGSTWHELYIFASNTPEIGNLTEFVFYSKIMVRGRDACWEHCVLVDATRQKVRCNYCHREFSGGVYRMKFHLAQIKNKDIVSCTEVPNDVRDQIQSILSTPKKQKTPKKTKVDLAANGQQNSSSASGDFHPNHGSSGQHGSTCPLLFPRPSPSEQPAVDDEQKQKQDDADKKVAVFFFHNSVPFSAAKSMYYQEMVDAIAECGVGYKAPSYEKLRSTLMEKVKCDVNDCCKKLRDGWRATGCTILCDCWSDGRTKSLVVFSVTCPKGTLFLKSVDISGHADDAHYLYELLESVVLEVGLENVVQVITDSAASYVVYAGRLLMAKYTTLFWSPCASFCIDKMLEDISKQEWVSTVLEEAKTITHYIYSHAWILNMMRKFTGGRELIRPRITRFVTNFLSLRSIVVQEDNLKLMFSHMDWMSSVYSRRPDSQNVKSLLYLERFWKSAHEAVSVSEPLVKVLRIVDGDMPAMGYIYEGIERAKIAIKGYYNSIEEKYMPIWDIIDRRWNVQLHSPLHAAAAFLNPSIFYGPNFKVDLRMRNGFQEAMRKMATEDRDKIEITKEHPIYINAQGALGTEFAIMGRTLNAAGDWWAGYGYEIPTLQRAAIRILSQPCSSHWCGWNWSSFEALHTKKRNRMELEKLNDLVLVHCNLHLQAIIQSRDGKCKPIIFDEIDVGSEWPTEMESPSPLLDDSWLDNLPLECKGSP	zf-BED--DUF-domain--Dimer_Tnp_hAT--	I
GSVIVT01008224001	GsvZf-BED02_I	MDWSVNNAFKTYKDAEPKSVMDMALIPNIDPRDIGLGSSEKGNVGPAAKPRKKTMTSVYLKFFETAPDGKSRRCKFCGQSYSIATATGNLGRHLSNRHPGYDKSGDAVTSSAPQPITIVKKPQTQVKSPQVDFDHLNWLLIKWLILASLPPSTLEEKWLANSFKFLNPSIQLWPGEKYKAVFREVFRSMREDVRASLEQVSSKVSITVDFWTSYEQIFYMSVTCHWIDENWCFQKVLLDICHIPYPCGSNEIYHSLIKVLKMYNIESKVLSCTHDNSQTAMHACHSLKEDLDGQKVGPFCYLPCAARTLNMIIDDGLRTTKPVITKIREFVLEMNSSSEISEDFIQFTTVYQEGSWKIPLDASARWSGNYQMLDIVCKAGKSMDAVIRKYEETLSGRMLLNPAEKNAVNIVYAYLEAFYKITLNMINKVPTIGLVLFFMDHISEMIAGCRESLRSPDWLKNAAEEMAKKTRSYSNQVCNIFTYMTAILDPRIKAELIPESLNSETNLEEARTHFMRNYSTNHFPSIASGYSAQEIEDGASVSFAEEIARKKRRVSMSTATDELTQYLSEPPAPIPTDVLEWWKVNTTRYPRLSTMARDFLAVQATSVAPEEVFCGKGDEMDKQRFSMPHDSTQALLCIRSWTHGGIKLKYKSTEIDYESLMELATAAADNGTAGFDKKQK	zf-BED--DUF-domain--Dimer_Tnp_hAT--	I
GSVIVT01014094001	GsvZf-BED03_I	MEISNESAIKKPKRLTSVVWNHFERVRKADICYAVCIHCNKRLSGSSNSGTTHLRNHLMRCLKRSNYDVSQLLAAKRRKKEGALSLTAINYDEGQRKEENIKPTILKFDQEQKKDEPINLGSIRFDQERSRLDLARMIILHGYPLAMVNHVGFKVFVKDLQPLFEVNSAIELDCMEIYGKEKQKVYEVMSRSHGRINLAVDMWTSPEQAEYLCLTAHYIDEDWKLQKKILNFVSLDPSHTEDMLSEVIIKCLMEWEVGHKLFSMTFHDCATNDDVALRVKEHFSQDRPLLGSGQLLDVRCVGHVLNLIVQDCIEALREVTHKIRESVRYVKTSQATLGKFNEIAQQVGINSQQNLFLDCPTQWNSTYLMLDRVLEYKGAFSLLQEHDPGYTVALSDTEWEWASSITSYMKLLLEIIAVLSSNKCPTANIYFPEICDIHIQLIEWCKSPDDFISSLALKMKAKFDKYWSKCSLALAVAVILDPRFKMKLVEYYYPQIYGTDAADRIKDVSDGIKELFNVYCSTSASLHQGVALPGSSLPSTSNDSRDRLKGFDKFIHETSQNQNIVSDLDKYLEEPVFPRNCDFHILNWWKVQKPRYPILSMMVRDVLGIPMSTVAPEVVFSTGARVLDHYRSSLNPDTRQALICTQDWLQTGLEEPNQSSPHQTSPHPAIPLAIEAN	zf-BED--DUF-domain--Dimer_Tnp_hAT--	I
GSVIVT01017686001	GsvZf-BED04_III	MVEEMTSLRSPGYSDPGWEHGIAQDERKKKVKCNYCGKIVSGGIYRLKQHLARVSGEVTYCDKAPEEVYLKMRENLEGCRSNKKPRQSEDDGHTYLNFHQNDDEEEEEEHAGYRSKGKQLMSDRNLVINLAPLRSLGYVDPGWEHGVAQDERKKKVKCNYCEKIVSGGINRFKQHLARIPGEVAPCKNAPEEVYLKIKENMKWHRTGRRHRRPDAKEISAFYMNSDNDDEEDEQDEDALHRMNKENLIIGEKRLSKDLRKTFRGISPGSGSEPSLRRSRLDSVVPKTPKSQKALSYKQVKVKTGSSKKTRKEVISAICKFFYHAGVPLHAANSPYFHKMLELVGQYGQGLVGPPTQLISGRFLQEEIATIKNYLAEYKASWAITGCSIKADSWRDAQGRTLINILVSCPHGIYFVSSVDATDIVDDATNLFKLLDKVVEEMGEENVVQVITENTPSYKAAGKMLEEKRRSLFWTPCAAYCIDQMLEDFIGIKLVGECMEKGQKITKFIYNRIWLLNLMKKEFTQGQELLRPAVSRCASSFATLQSLLDHRIGLKRLFQSNKWLSSRFSKSEKGKEVEKIVLNATFWKKVQYVRKSVDPLVQVLQKVDSVESLSMPSIYNDMYRAKLAIRSTHGDDARKYGPFWAVIDNHWSSLFHHPLYMAAYFLNPSYRYRSDFLVHPEVVRGLNECIVRLEPDNMRRISASMQISDFNSAKADFGTELAISTRTELDPAAWWQQHGINCLELQRIAVRILSQTCSSFGCEHNWSTYDQIHRESHNRLAQKRLNDLIYVHYNLRLRERQLSKRSNDVMSLDSILLESLLDDWIVEAENPTVQEDEEIPYNEMDHTDAYENDLMEYEDGTADGRKASLEMVTLSSVEPLDIVNPASAGVATDDDTDLNFLGDDLSD	zf-BED--zf-BED--DUF-domain--Dimer_Tnp_hAT--	III
GSVIVT01021057001	GsvZf-BED05_XXII	MNIFRLAGDMTHLASVLVLLLKIHTIKSCAGISLKTQELYALVFATRYLDIFTDYISLYNTVMKLIFLGSSFSIVWYIRHHKIVRRSYDKDQDTFRHFFLVLPCLLLALVMNEKFTFLEVLWAFSLYLEAVAILPQLVLLQRTRNIDNLTGQYVFLLGIIRVLILKRRQGFRALYIKWKGLKEEKKISIFSGGDPSRSSPGMSSFDLKNSKKDFVWKYVIEVAGEQYLRCKFCNQRCTGGVNRLKHHLAGTHHGMKPCNKVSEDAKLECKEALANFKDQKTKRNELFQEIGMGPTSMHESALSKTIGTLGSGSGSGSVSGSGEPIPRGPMNKFTTSQPRQTTLNSKWKQEERKEGSVAYRALYILNWIYRYFTEPHYVHWITWISGLVQTLLYADFFYYYFHSWKNNEKLHLPA	ER_lumen_recept--zf-BED--	XXII
GSVIVT01027351001	GsvZf-BED06_I	MPFHTIGLYCPRVSSLAEQEEEDDDHQAAAQSLHCSTNRTTPHQQHMVETREFFDAFPANQHKDDQGTAPPRATDPGWAHGIMVNGGRQKIKCKYCHKVILGGGISRLKQHLAGERGNVAPCEEVPEDVKVQIQQHLGFKVLEKLKRQKGLKSSKNSLVPYYQDREGGADDVQRSPKAASARGISRKRRGKEIDEGTSYKKKRHKKQLFPTATPVAQVSIHNSFASQESMDQADMAVARFMYEAGVPFSAANSYYFQQMADAIAAVGPGYKMPSCHSLRGKLLNRSVQDVEGLCEELRRSWEVTGCSVMVDRCTDRTGHTVLNFYVYCPKGTVFLRSVYASDIANSTEALLSLFVSVVEEVGPKNIVNFVTDTTPTYKAAGKLLMGRYKTFFWSACGAHCIDLMLEEVGKRDEVKELLAKAKRITQFIYNNTWVLNLTRKRTGGRDIVQLAITRFASNFLTLQSIVSFKEALHQMFTSATWMQSAFSKQRAGVEVAEIIVDPTFWSMCDRALKVSKPLLAVLHLIDCEERPSVGYIYDAMEKAKKSIILAFDDKESDYSPYLKIIDCIWKEEFHSPLHAAAYYLNPSIFYNPSFSTNKVIQKGLLDCIESLEPNLSTQVMITSHINYYEEAVGDFSRPVALRGRESLAPATWWSLYAADYPDLQRLAVRILSQTCSVTRCETSWSMSERVHSKQRNRLEHQRLSDLIFVHYNLRLQEK	zf-BED--DUF-domain--Dimer_Tnp_hAT--	I

GSVIV<br>T0102<br>87260<br>01	Gsv<br>Zf-<br>BED<br>07_I	MAQIHLRPDTTHRCQSPKLIGLGPKNLIFSNEMRLYLKISEWRSGSVGISFISMVREKDVCWEYAEKLDGNKVRCKFCLRVLNGGISRLKHHLSRLPSKGVNPCSKVRDDVTDRVRAIISSKEDGKETSSAKKQRVAEAKSPGNYSAIKALMSVETPSPIAKIFPPITHMGPSSSNDGENAERSIALFFFENKLDFSVARSSSYQLMIEAVSKCGHGFRGPSAEILKTTWLERIKSEVSLQSKDIEKEWATTGCTIIADTWTDNKSRALINFLVSSPSRTFFHKSVDASSYFKNTKYLADLFDSVIQDLGPDNVVQIIMDSTLNYTGVASHIVQNYGTVFVSPCASQCLNLILEDFCKIDWVNRCILQAQTISKFIYNNASMLDLMKKSTGGQDLIRTGITKSVSNFLSLQSMLKQRPRLKHMFGSSEYSTNSYSNKPQNISCIAILEDNDFWRAVEECVAISEPFLKGLREVSGGKPAVGSIYELMTKAKESIRTYYIMDESKCKAFLDIVDGRWRNQLHSPLHAAAAFLNPSIQYNPEIKFIGAIKEDFFKVLEKLLPTSDMRRDITNQILLFTRATGMFGCNLAREARDTVPPGLWWEQFGDSAPVLQRVAIRILSQVCSTSTFERHWNTFQQIHSEKRNKIDKETLNDLVYINYNLKLARQMKMKSSEADPLQFDDIDMTSEWVEETENPSPTQWLDRFGSALDGSDLNTRQFNAAIFGSSDTIFGL	zf-BED--DUF-<br>domain--<br>Dimer_Tnp_hAT<br>--	I
GSVIV<br>T0103<br>20250<br>01	Gsv<br>Zf-<br>BED<br>08_<br>XXII	MTSSERIMDGSNGDVAEDFYHRYKEDVHTMKELGMDIFRFSISWFRVLPHEMSSSDSKNSRKDFVWKYVIEVSGEQYLRCKFCNQRCTGGVNRLKHHLAGTHHGRKPCSKVSEDARLECKEALANFKDQKTKRNELLQEIGMSPTSMHESALSKTIGTLGSGSGSVSGSGEPIPRGPMDKFTTSQPRQSTLNSKWKQEERKEVCRKFGRFIYSKGLPFNTVNDPYWFPMIDAVANFGPGFKPPSMHELRTWILKEEVNDLSIIMEDHKKAWKQYGCSIMSDGWTDGKSRCLINFLVNSPAGTWFMKSIDASDTIKNGENMFKYLDEVVEEIGEENVVQVITDNASNYVNAGMRLMEKRSRLWWTPCAAHCIDLMLEDIGKLNVHATTLSRARQVVKFIYRHTWVLSLMRTFTKNHELIRPAITRFATAFLTLQSGKLSGGVNKEGINFYNSLINELLSKGLQPYVTLFHWDLPQALEDEYGGFLSPHIINDFRDFAELCFKEFGDRVKYWITLNEPWSYSNGGYVEGNFAPGRCSKWVNGACRAGNSATEPYTVGHQLLLSHAAAVKVYKNKYQASQKGKIGITLVSHWMVPYSNQKVDKKEARRALDFMLGWFMNPLSYGDYPHSMRKLVGRRLPKFTPRQSLLIKGSFDFLGLNYYTANYAAHVPVANTVNVSYSTDSLVKLTNNTSQWNPHRSNDGFRLA	Glyco_hydro_1--<br>zf-BED--DUF-<br>domain--<br>Glyco_hydro_1--	X<br>X<br>II
HVU0<br>036G0<br>622.01	Hvu<br>Zf-<br>BED<br>01_I<br>I	MVVVEGDPKAVCKYCRGQFHTKFGTSSLRTHIAEACRSIEDACRKSFLLTMKKMPSEGLLVFDEKVSRELMVKFCIHAEIPFLKFEDPHLQPWIDSL	zf-BED	II
HVU0<br>037G0<br>661.01	Hvu<br>Zf-<br>BED<br>02_I<br>II	MEIEHAVIPQVVEYTEEHEVEVDVDVDNIMKTEVSYGSGIHNSAFRYKHKKRSKVWEEYKPIFLDGKVQFAECLYCHNRLSCKDSNGTSHLWRHQKICPGKTEVAAERQQKDSYFPYVLLNGDSPVSPIDPVNQIISETLDDIGSATPSRFKSKVWKEFTPIYVEGKLQAADCIHCQKRLSANKFGGRSHLSRHLLTCPGRLESIHNHQKFLFEPSSAPSFDSRVHDELSPALTNGKVQIAEYTNKFPRTSTSAHTIPIVQPIQMLPAHTPPTSDGASLKKQKTSFTNISTDKRAGKFCQETSYQELAKMIILHAYPFSIVEHEEMRRVIKNLNPVVGMVSHKDVEDHCIALFQKEKVNIKDVITLSSRRVSLSASIWTPDGSDPTVNYLCLTAHFVGDDWKVHRVIIKFGMFWSSPTNLERIIYCKEASVPESESGSYNVIWDAIRDWNLDQKILSLTSVGDVRNNANTVKLKEMLIEKKCLPIRGKLYNIACLDDILNSVVSAGQADILRFVGDVLMEFFVAHASSASAQQQLLEVVSQMSLKCPQEDAKWWHKFYFRLEVILHFKKSFPSEEVLNPEDMNVAESICKILRTFYRVIEVISCPSSVTANMYFNEVWKVRTVLQEEASNGNGEYSRLVMQMQESFHEYWQNSYLSLSIPVVLDPRFKISFIEFRLKRAFGTNSESYLSEIRDTVRELFNEYCNPTDRPSTASNSAALGADYNDSLEDWDQHLNEQAGSQISTELDDYLEEGLVPRKDEFDILNWWMNHTEKYPTLAAMAQDILAMPASAVQSEAAFSSSGPVIPKHYSTLSIKTIEALVCARDWMR	zf-BED--zf-BED-<br>-DUF-domain--<br>Dimer_Tnp_hAT<br>--	II<br>I
HVU0<br>037G0<br>661.02	Hvu<br>Zf-<br>BED<br>03_I<br>II	MEIEHAVIPQVVEYTEEHEVEVDVDVDNIMKTEVSYGSGIHNSAFRYKHKKRSKVWEEYKPIFLDGKVQFAECLYCHNRLSCKDSNGTSHLWRHQKICPGKTEVAAERQQKDSYFPYVLLNGDSPVSPIDPVNQIISETLDDIGSATPSRFKSKVWKEFTPIYVEGKLQAADCIHCQKRLSANKFGGRSHLSRHLLTCPGRLESIHNHQKFLFEPSSAPSFDSRVHDELSPALTNGKVQIAEYTNKFPRTSTSAHTIPIVQPIQMLPAHTPPTSDGASLKKQKTSFTNISTDKRAGKFCQETSYQELAKMIILHAYPFSIVEHEEMRRVIKNLNPVVGMVSHKDVEDHCIALFQKEKVNIKDVITLSSRRVSLSASIWTPDGSDPTVNYLCLTAHFVGDDWKVHRVIIKFGMFWSSPTNLERIIYCKEASVPESESGSYNVIWDAIRDWNLDQKILSLTSVGDVRNNANTVKLKEMLIEKKCLPIRGKLYNIACLDDILNSVVSAGQADILRFVGDVLMEFFVAHASSASAQQQLLEVVSQMSLKCPQEDAKWWHKFYFRLEVILHFKKSFPSEEVLNPEDMNVAESICKILRTFYRVIEVISCPSSVTANMYFNEVWKVRTVLQEEASNGNGEYSRLVMQMQESFHEYWQNSYLSLSIPVVLDPRFKISFIEFRLKRAFGTNSESYLSEIRDTVRELFNEYCNPTDRPSTASNSAALGADYNDSLEDWDQHLNEQAGSQISTELDDYLEEGLVPRKDEFDILNWWMNHTEKYPTLAAMAQDILAMPASAVQSEAAFSSSGPVIPKHYSTLSIKTIEALVCARDWMR	zf-BED--zf-BED-<br>-DUF-domain--<br>Dimer_Tnp_hAT<br>--	II<br>I
HVU0<br>038G0<br>256.01	Hvu<br>Zf-<br>BED<br>04_<br>VII	MIDENRQHWKCMYCHLTRYGGGVSRLKRHLAGDLDVKMCPKVPAEVSENIREHLQKKRKRRKQRAAQNGVNSVTRSFADDTKTEKDPVSVDLEVPTRIDTYILEEGTNLTNADHQEPTIFRPPLLLRGVRDIGWEHAVDLDGNKKRWQCKWCDLCRSGGVTTLKAHLTDSSCP	zf-BED--zf-BED-<br>-	V<br>II
HVU0<br>038G0<br>256.02	Hvu<br>Zf-<br>BED<br>05_I<br>I	MDANKDKGNDKVHLRDWSSLEALRTYKRRRQPEPEPEPEPEPEPEPNPVDVREQQVTDTFWKSRDIGWKHGIMIDENRQHWKCMYCHLTRYGGGVSRLKRHLAGDLDVKMCPKVPAEVSENIREHLQKKRKRRKQRAAQNGVNSVTRSFADDTKTEKDPVSVDLEVPTRIDTYILEEGTNLTNADHQEPTIFRPPLLLRGVRDIGWEHAVDLDGNKKRWQCKWCDLCRSGGVTTLKAHLTDSSCPKIPTEMSKQVLHFVEEKRAARQLFNRDPWPPYKKIDGASLFCSEGKEEGLVSCKTGQQPSNNGMHMQTSGNCTIDEVYEPNNLIPVDSFNLC	zf-BED	II
HVU0<br>038G0<br>256.04	Hvu<br>Zf-<br>BED<br>06_I<br>I	MDANKDKGNDKVHLRDWSSLEALRTYKRRRQPEPEPEPEPEPEPEPNPVDVREQQVTDTFWKSRDIGWKHGIMIDENRQHWKCMYCHLTRYGGGVSRLKRHLAGDLDVKMCPKVPAEVSENIREHLQKKRKRRKQRAAQNGVNSVTRSFADDTKTEKDPVSVDLEPPLLLRGVRDIGWEHAVDLDGNKKRWQCKWCDLCRSGGVTTLKAHLTDSSCPKIPTEMSKQVLHFVEEKRAARQLFNRDPWPPYKKIDGASLFCSEGKEEGLVSCKTGQQPSNNGMHMQTSGNCTIDEVYEPNNLIPVDSFNLC	zf-BED	II
HVU0<br>038G2<br>178.01	Hvu<br>Zf-<br>BED<br>07_I	MLAPAAMEVSVDGTVSTTVVSMPAVHNPRARKLRSAVWQDFTKERRADGNCVAICNHCKKQLTATSRSGTTHLRNHLAICTTTSTRRAGKRRKLIVRRILHNKTSTGQPGGGHASGDDNDNDSTHFDQELSRQDLARMIVQHGYRFSIVDDLGFQKFVKNLQPQFSMVSYDIVRADSMAIFESEKLKLQDVLLNTPCRVSISVDMWRSSTQMDYLCLTCHYIDHSGDEWKLRKKILNFVHVEEHFTASQIANLILEKLQRWGIERKLAAVVLDNCTAGDIVATELLRVMQPRRLLLLNGNLFHVRSCAHILNLTVEESLEQTSDIINRVREMIQNVKFSQERLQKFLDTAKLLQIDQKLLVLDSPNNWPSTYLMFDSACYYHDVLVRLAEQEAHYAAFLTAKEWADVKALTEILDALNNTMEKFPVENPTANLYFNDMCEIQVLLNTWRNSPSPVVAQVAGRMLKKFEGYWDLTRPVMAFASILDPRYKMKSIEYFFQLIYGNDQFTAKATIEAIKQTFTSLCNEYEHSADSLKNPAVLFYAGNSSSCMSSVYNNGNDSKTFSRITLSDARRGLDQYIQESSSGQSLKSDLDLYLEEAVYRQKEGNQDNFDILGWWKSFAAKYPVLSQMARDILAIPVSIIPLDSEARVLNEYLSTMDPSTVEGLVCAQDWLRADTEVANSDGHADDKVPRGDELIVAPN	zf-BED--DUF-<br>domain--<br>Dimer_Tnp_hAT<br>--	I
HVU0<br>038G2<br>178.02	Hvu<br>Zf-<br>BED<br>08_I	MLAPAAMEVSVDGTVSTTVVSMPAVHNPRARKLRSAVWQDFTKERRADGNCVAICNHCKKQLTATSRSGTTHLRNHLAICTTTSTRRAGKRRKLIVRRILHNKTSTGQPGGGHASGDDNDNDSTHFDQELSRQDLARMIVQHGYRFSIVDDLGFQKFVKNLQPQFSMVSYDIVRADSMAIFESEKLKLQDVLLNTPCRVSISVDMWRSSTQMDYLCLTCHYIDHSGDEWKLRKKILNFVHVEEHFTASQIANLILEKLQRWGIERKLAAVVLDNCTAGDIVATELLRVMQPRRLLLLNGNLFHVRSCAHILNLTVEESLEQTSDIINRVREMIQNVKFSQERLQKFLDTAKLLQIDQKLLVLDSPNNWPSTYLMFDSACYYHDVLVRLAEQEAHYAAFLTAKEWADVKALTEILDALNNTMEKFPVENPTANLYFNDMCEIQVLLNTWRNSPSPVVAQVAGRMLKKFEGYWDLTRPVMAFASILDPRYKMKSIEYFFQLIYGNDQFTAKATIEAIKQTFTSLCNEYEHSADSLKNPAVLFYAGNSSSCMSSVYNNGNDSKTFSRITLSDARRGLDQYIQESSSGQSLKSDLDLYLEEAVYRQKEGNQDNFDILGWWKSFAAKYPVLSQMARDILAIPVSIIPLDSEARVLNEYLSTMDPSTVEGLVCAQDWLRADTEVANSDGHADDKVPRGDELIVAPN	zf-BED--DUF-<br>domain--<br>Dimer_Tnp_hAT<br>--	I
HVU0<br>038G2<br>217.01	Hvu<br>Zf-<br>BED<br>09_I<br>V	MREKDACWEYGDKLDGNRVRCRFCQKVINGGISRFKFHLSQIPSKGVNPCVKVTDDVREKVIALIEAKESHRELELLKRKRVAELSVLPKRTRELPSQPSSPGLPASPAIIPAFEPNQLLGLEVPAPVLRLSSAVTKPRPASGLEVERCIAEFFFENKLDYSIADSISYRHMMETLVGQESQGPSADVLRTKWLQKLKSEILQRTQEIKKDWVTTGCTILADSWTDNKMKALINFSVSSPLGTFFLKTIDASPHIKNHRGMYELFDEVIQEVGPDNVVQIISDRNINYGNIDKLIMQNYNTIFWSPCASFCVNSMLDEFSKIDWVNQCICQAQTITRFVYNNNWILDLMRRCMAGQELVCSGITKSVSDFLTLQSLLKHRLKLKQMFHSSDYVSSSYANRSLSISCVEILNDDEFWRAVEEIAAVSEPLLRVMRDVSGGKAAIGYIYESMTKVMDSIRTYYIMDEGKCKSFLDIVEQKWQVELHSHLHSAAAFLNPSIQYNPEVKFFTSIKEEFYHVLDKVLTAPDQRHGITSELHAFRKAQGMFASNIAKEARNNTSPGLSLLL	zf-BED--DUF-<br>domain	I<br>V
HVU0<br>038G2<br>217.02	Hvu<br>Zf-<br>BED	MREKDACWEYGDKLDGNRVRCRFCQKVINGGISRFKFHLSQIPSKGVNPCVKVTDDVREKVIALIEAKESHRELELLKRKRVAELSVLPKRTRELPSQPSSPGLPASPAIIPAFEPNQLLGLEVPAPVLRLSSAVTKPRPASGLEVERCIAEFFFENKLDYSIADSISYRHMMETLVGQESQGPSADVLRTKWLQKLKSEILQRTQEIKKDWVTTGCTILADSWTDNKMKALINFSVSSPLGTFFLKTIDASPHIKNHRGMYELFDEVIQEVGPDNVVQIISDRNINYGNIDKLIMQNYNTIFWSPCASFCVNSMLDEFSKIDWVNQCICQAQTITRFVYNNNWILDLMRRCMAGQELVCSGITKSVSDFLTLQSLLKHRLKLKQMFHSSDYVSSSYANRSLSISCV	zf-BED--DUF-<br>domain	I<br>V

	10_I<br>V	EILNDDEFWRAVEEIAAVSEPLLRVMRDVSGGKAAIGYIYESMTKVMDSIRTYYIMDEGKCKSFLDIVEQKWQVELHSHLH<br>SAAAFLNPSIQYNPEVKFFTSIKEEFYHVLDKVLTAPDQRHGITSELHAFRKAQGMFASNIAKEARNNTSPGLSLLL		
HVU0<br>038G2<br>217.04	Hvu<br>Zf-<br>BED<br>11_I	MREKDACWEYGDKLDGNRVRCRFCQKVINGGISRFKFHLSQIPSKGVNPCVKVTDDVREKVIALIEAKESHRELELLKRK<br>RVAELSVLPKRTRELPSQPSSPGLPASPAIIPAFEPNQLLGLEVPAPVLRLSSAVTKPRPASGLEVERCIAEFFFENKLDYSI<br>ADSISYRHMMETLVGQESQGPSADVLRTKWLQKLKSEILQRTQEIKKDWVTTGCTILADSWTDNKMKALINFSVSSPLGT<br>FFLKTIDASPHIKNHRGMYELFDEVIQEVGPDNVVQIISDRNINYGNIDKLIMQNYNTIFWSPCASFCVNSMLDEFSKIDWVN<br>QCICQAQTITRFVYNNNWILDLMRRCMAGQELVCSGITKSVSDFLTLQSLLKHRLKLKQMFHSSDYVSSSYANRSLSISCV<br>EILNDDEFWRAVEEIAAVSEPLLRVMRDVSGGKAAIGYIYESMTKVMDSIRTYYIMDEGKCKSFLDIVEQKWQVELHSHLH<br>SAAAFLNPSIQYNPEVKFFTSIKEEFYHVLDKVLTAPDQRHGITSELHAFRKAQGMFASNIAKEARNNTSPGMWWEQYGD<br>SAPALQHCAVRIVSQVCSTLTFQRDWSIILQSHSEKRNKLNKEALADQAFVHYNLMLHSDSKTTTKKKGEGDPIALDDIDM<br>TSPWVEDSDGPSLTQWLDRFPSALDGGDLNTRQFGGSIFGTNDLFGL	zf-BED--DUF-<br>domain--<br>Dimer_Tnp_hAT<br>--	I
HVU0<br>038G2<br>217.05	Hvu<br>Zf-<br>BED<br>12_I	MREKDACWEYGDKLDGNRVRCRFCQKVINGGISRFKFHLSQIPSKGVNPCVKVTDDVREKVIALIEAKESHRELELLKRK<br>RVAELSVLPKRTRELPSQPSSPGLPASPAIIPAFEPNQLLGLEVPAPVLRLSSAVTKPRPASGLEVERCIAEFFFENKLDYSI<br>ADSISYRHMMETLVGQESQGPSADVLRTKWLQKLKSEILQRTQEIKKDWVTTGCTILADSWTDNKMKALINFSVSSPLGT<br>FFLKTIDASPHIKNHRGMYELFDEVIQEVGPDNVVQIISDRNINYGNIDKLIMQNYNTIFWSPCASFCVNSMLDEFSKIDWVN<br>QCICQAQTITRFVYNNNWILDLMRRCMAGQELVCSGITKSVSDFLTLQSLLKHRLKLKQMFHSSDYVSSSYANRSLSISCV<br>EILNDDEFWRAVEEIAAVSEPLLRVMRDVSGGKAAIGYIYESMTKVMDSIRTYYIMDEGKCKSFLDIVEQKWQVELHSHLH<br>SAAAFLNPSIQYNPEVKFFTSIKEEFYHVLDKVLTAPDQRHGITSELHAFRKAQGMFASNIAKEARNNTSPGMWWEQYGD<br>SAPALQHCAVRIVSQVCSTLTFQRDWSIILQSHSEKRNKLNKEALADQAFVHYNLMLHSDSKTTTKKKGEGDPIALDDIDM<br>TSPWVEDSDGPSLTQWLDRFPSALDGGDLNTRQFGGSIFGTNDLFGL	zf-BED--DUF-<br>domain--<br>Dimer_Tnp_hAT<br>--	I
HVU0<br>040G0<br>084.01	Hvu<br>Zf-<br>BED<br>13_I	MADETGNDSQMVEGNEIVDPGNEVVHGGELVEVDELAQGDELAQGEDLTQVDELLQGNEMAITEVATPPTTRRRRKKS<br>LVWEHFTIEDAAGGATRACCKLCKQTFAYSSGSKIAGTSHLKRHITLGSCPKIKGQGHKLAITIGTDNDGDGTVERRSKRR<br>YRYTGYANASFNQDRSCSYLAKMIILHDYPLHIVQQPAFTAFIDSLQPRFRVADVDAMEVEVYAVYQKEKENLLQAFSTMP<br>GRISLTIGLWTTSQTLGYVSVSGQFIDSEWKLHRRMLSFMMVSSPHSENALSEAISSSLTDWSMKEKLFTITLDNDCSSHD<br>IYSANLRDYLSSKNNHMLKGQLFVVRCYAHILNAVAEDVIALIHGVIYSIRESIKFIKASPSREEKFAEIALQLEIPSTKTLCLD<br>VTTQWNTTYLMLLAALDYRQAFITLETVDDNYNEAPSAEDWKKIEAACNYLRLLYDSAHSIMAAGNPTSNIFFHEAWKLQL<br>ELANASAHEDPIFSSVAKDMYEKFDKYWKDCNLVLAVAVVMDPRFKMKLVEFSYSKIYGVEAAKYVKVVNDSVHDLFKDY<br>VAQPLPLTPAYVEQGKTDNVTANGNNTQATQPSTGDGLQDFDIYLSEIATTQPSKSELEQYLDESLTPRIQEFDILNWWKL<br>NTLKYPTLSKMARDILSIPVSMVTGGSSIFSAGTGGHMLDDYRSSLRPEIVEALVCAKDWLPYSTTAPEALGSELLKID	zf-BED--DUF-<br>domain--<br>Dimer_Tnp_hAT<br>--	I
HVU0<br>040G0<br>084.02	Hvu<br>Zf-<br>BED<br>14_I	MLCMPDFGTSSMADETGNDSQMVEGNEIVDPGNEVVHGGELVEVDELAQGDELAQGEDLTQVDELLQGNEMAITEVAT<br>PPTTRRRRKKSLVWEHFTIEDAAGGATRACCKLCKQTFAYSSGSKIAGTSHLKRHITLGSCPKIKGQGHKLAITIGTDNDG<br>DGTVERRSKRRYRYTGYANASFNQDRSCSYLAKMIILHDYPLHIVQQPAFTAFIDSLQPRFRVADVDAMEVEVYAVYQKE<br>KENLLQAFSTMPGRISLTIGLWTTSQTLGYVSVSGQFIDSEWKLHRRMLSFMMVSSPHSENALSEAISSSLTDWSMKEKL<br>FTITLDNDCSSHDIYSANLRDYLSSKNNHMLKGQLFVVRCYAHILNAVAEDVIALIHGVIYSIRESIKFIKASPSREEKFAEIAL<br>QLEIPSTKTLCLDVTTQWNTTYLMLLAALDYRQAFITLETVDDNYNEAPSAEDWKKIEAACNYLRLLYDSAHSIMAAGNPT<br>SNIFFHEAWKLQLELANASAHEDPIFSSVAKDMYEKFDKYWKDCNLVLAVAVVMDPRFKMKLVEFSYSKIYGVEAAKYVK<br>VVNDSVHDLFKDYVAQPLPLTPAYVEQGKTDNVTANGNNTQATQPSTGDGLQDFDIYLSEIATTQPSKSELEQYLDESLT<br>PRIQEFDILNWWKLNTLKYPTLSKMARDILSIPVSMVTGGSSIFSAGTGGHMLDDYRSSLRPEIVEALVCAKDWLPYSTTA<br>PEALGSELLKID	zf-BED--DUF-<br>domain--<br>Dimer_Tnp_hAT<br>--	I
HVU0<br>040G1<br>748.01	Hvu<br>Zf-<br>BED<br>15_I	MGEPNGNENAMVHESEMDVGDEMIHGNEMVHSGDMIHEHDMVQGSEMVHGDEMVQGSGMVHDDEMIHGNEMIQVS<br>DMIHGHEMVQVNDMVNGDEMAHGHELVNAEATPPAITRRRRKKSLVWEHFTIEPVPGERGCQRACCNLCKATFAYSSG<br>TKIAGTSHLKRHITLGSCPMIKNQERKLAMTSIGGVTDNDGEGTVERPSKRRYRYTGFANATFDQDRSSSYLAKLIILHDYP<br>LHVVQQPAFTAFTESLQPRFRVADVETMEAEVYGVYQKEKHNLMQALNTMPGRISLTIGLWTTSQTLGYVSIAGQFIDTD<br>WKVHRRMLNFMMVSSPHSENALSEAISSSLTDWNMKDKLFTITLDNDCSSHDIYSANLRDHLSNKNNLMLKGQLFVVRC<br>YANILNAVAQDVIASIHGVIYSIRESIKFIKASSAREQRFAEIALQLEIPSTKTLCLDVTTQWNTTYLMLLAALDYRQAFTTLET<br>CDENYNESPSAEDWKKVEAACNYLKFLYDSAHGIMAAANPTSNLFFHEAWKLQLELSNAISHEDPVFSSIAKEMHERFDK<br>YWKDCNLVLAIGVVMDPRFKMKLVEFSYSKIYGVEAAKYVKVVDDALHDLYKDYIAQPQTLTPAYAVPGEINKNGPANGN<br>NNTVTPPSTGDGLLDFDMYLSEIAISQPSKSELEQYLEEALTPRIQDFEIVDWWKLNTLKFPTLSKMARDILAIPMSMVSSG<br>GNIFSAATTGSRMLDDYRSSLRPEIVEALFCAKDWLQYSPPPPAEAQGSVGVKME	zf-BED--DUF-<br>domain--<br>Dimer_Tnp_hAT<br>--	I
HVU0<br>040G1<br>748.02	Hvu<br>Zf-<br>BED<br>16_I	MGEPNGNENAMVHESEMDVGDEMIHGNEMVHSGDMIHEHDMVQGSEMVHGDEMVQGSGMVHDDEMIHGNEMIQVS<br>DMIHGHEMVQVNDMVNGDEMAHGHELVNAEATPPAITRRRRKKSLVWEHFTIEPVPGERGCQRACCNLCKATFAYSSG<br>TKIAGTSHLKRHITLGSCPMIKNQERKLAMTSIGGVTDNDGEGTVERPSKRRYRYTGFANATFDQDRSSSYLAKLIILHDYP<br>LHVVQQPAFTAFTESLQPRFRVADVETMEAEVYGVYQKEKHNLMQALNTMPGRISLTIGLWTTSQTLGYVSIAGQFIDTD<br>WKVHRRMLNFMMVSSPHSENALSEAISSSLTDWNMKDKLFTITLDNDCSSHDIYSANLRDHLSNKNNLMLKGQLFVVRC<br>YANILNAVAQDVIASIHGVIYSIRESIKFIKASSAREQRFAEIALQLEIPSTKTLCLDVTTQWNTTYLMLLAALDYRQAFTTLET<br>CDENYNESPSAEDWKKVEAACNYLKFLYDSAHGIMAAANPTSNLFFHEAWKLQLELSNAISHEDPVFSSIAKEMHERFDK<br>YWKDCNLVLAIGVVMDPRFKMKLVEFSYSKIYGVEAAKYVKVVDDALHDLYKDYIAQPQTLTPAYAVPGEINKNGPANGN<br>NNTVTPPSTGDGLLDFDMYLSEIAISQPSKSELEQYLEEALTPRIQDFEIVDWWKLNTLKFPTLSKMARDILAIPMSMVSSG<br>GNIFSAATTGSRMLDDYRSSLRPEIVEALFCAKDWLQYSPPPPAEAQGSVGVKME	zf-BED--DUF-<br>domain--<br>Dimer_Tnp_hAT<br>--	I
HVU0<br>041G0<br>949.01	Hvu<br>Zf-<br>BED<br>17_I	MEEVVPIGAQKHDPAWKHCLMVRLAGRDRLKCVYCGKHFLGGGIHRFKEHLARRPGNACCCPDVPADVEAVMHRSLDE<br>VAAKKLRKRALAAAMVAVAAASEPSSVAAASPSPSASNGDIASPIHVVPLNQAPRDEETPLSETGWTGGGATKRRKKALA<br>VRRAPAPAPAPQHHHHQQQQQPIHPATPAPQTQPPHQILMALDATAPQSSRHVDPAAAADREQVCMAVGRFLYDAGVP<br>LEAVNSVHFQPMVDAIASMGGRPEVFSYHDFRGCVLKKSLEEVTAQSEFYKGSWTRTGCSVLSDEWTTDKGRTLMTFS<br>VYCPEGTMFLKSVDATDIVTSSDALFELLKSVVEEVGERNVVQVITKNSQIHAAAGKKLGETFPTLFWSPCTFRCIDGMLE<br>DFSKATAVSEIISNAKTITGFLYSSALALSLMKKHLQGKDLLVPAETRAAMNFVTLKNMYSLKEDLQAMVSSDEWIHCLLPQ<br>IPGGVEVSGIVSNLQFWSSCALVVRATEPLVHLLKLVGSNKRPAMGYVYAGLYKAKAAIKKELVKKSDYMPYWNIIDQRW<br>DKHIQRPLHSAGFFLNPLFFDGIGDNISNEVFSGMLDCIERLVSDVKIQDKIQKELNMYRSEAAGDFRRQMAIRARRTLPPA<br>EWWYMYGGACPNLTRLAVRILSQTCSAKGRDRTHIPFERLHDQRMNIFERQRMHHLTFVQCNLRLQNRQQYKAKAFDPI<br>SADYIDIVDDWVVDRSALFSGPTEQPNWMEISQPFNWTPSAGPGDEFESFIEGVDDEMIQGASQGIQDDDDDKDDSNDE<br>ERTLSVGE	zf-BED--DUF-<br>domain--<br>Dimer_Tnp_hAT<br>--	I
HVU0<br>041G1<br>120.01	Hvu<br>Zf-<br>BED<br>18_I<br>I	MADGDGDAHEGAYDPMKYPAHRPWRSNDPGWNYGYWWKPTDVNIVVCNLCGKITKGGIKRHKEHLAATGGDATG	zf-BED	II
HVU0<br>041G1<br>207.01	Hvu<br>Zf-<br>BED<br>19_I	MDQEVDSANNMVAPEYNSGGLSRANPKRLRSKAWEDFTPIFVGGKVAKAECMHCHQVFNSNSSSGTSNLLKHQAMCA<br>PRAQKRPMQRKLMSAGSDPTQKKLSFFPTSQKKCVGTSDARPEKKDLVLLCNDRSKESSQNGSHEELGSPEQNDLALP<br>DVPTDTKTKSQQVDQNGSHDKLGTTEQENDAFPDNPIDKNVKNQSHEELALPEQKGIPTATKQKNQDVGQDGSHGELV<br>RKLSLHGYLPSMMDHGGLRKSVDFLNPMAKMPSYADLISAFLDLFDNEKAKLKEKFAAFCSRVCLSVHVWHYDPLSAFL<br>CLSVHYIDDEWERQQKIITFHAMDDTICIAEELGDAILLAIRDWGLCGKVFSIILDDAFIDDSVASSIKAQLMKENSTCANQSL<br>FVVRYGTHLLDQVIQVGLDEFEKIMEKSANCSKSMMGPNSCTAVRYPNYQYAPSQEDWGEASKMCETLEEFHQHMDTI<br>QHFRGPVHLFNSIEDVKRDLRHGLEGHQDGSFSNMLKKMQQKFQKHWKLCCLHLCMQIVMDPSDGLEHIKSKSYVNDV<br>HDTLLNLFYEYSGQVEDPSCTSGSKTSKEAAVNEDDMPLACCGHYGDKCSRGRPMTELDQYFHEPTYYCRGQTSVLQ<br>WWKEHNLTYPTVARMARDILAYHPEAITR	zf-BED--DUF-<br>domain--<br>Dimer_Tnp_hAT<br>--	I
HVU0<br>041G1<br>421.03	Hvu<br>Zf-<br>BED<br>20_I<br>I	MNLMCSSPLHQPILSVLRRSWKSIEDEPGNSNVMDEQEESTGSAPVPVFSGRKLKRLRSKVWDDFTPIYIDGKLARAEC<br>MHCHQVLSSGTGNLQKHQAKCTPGAQKRPMQQKPPFLSTGQNKSSDTAKVLPQKKAPVLLHILLADTNEEGQKVGQNL<br>SHGELATHERKNLASCDTPTDKDRKNQSHEELTVPDQEIPTDMNQRDPEVGQIEPHEELVRIFSVHGYPPSIKIHDRFTKF<br>VASLNPMVKMPAKVDMYRYSKELFDKEKTKLKEKLAALRSRVCLSAYVWHYDLTSAFLCLSVHYIDDEWEKQSHIIRFHS<br>VDPSCSAKQLSQDILYSIGNWGLRYKVFSITLDEAFLDDSVASDLKASLQEWNLLSSMSANRNSSMSANHTSSMSANHSI<br>FVIRCPTHLLNQVIHVGMDELHKTMEKSTKCSKYTKGHI	zf-BED	II

HVU0041G1421.04	Hvu Zf-BED 21_II	MDEQEESTGSAPVPVFSGRKLKRLRSKVWDDFTPIYIDGKLARAECMHCHQVLSSGTGNLQKHQAKCTPGAQKRPMQQKPPFLSTGQNKSSDTAKVLPQKKAPVLLHILLADTNEEGQKVGQNLSHGELATHERKNLASCDTPTDKDRKNQSHEELTVPDQEIPTDMNQRDPEVGQIEPHEELVRIFSVHGYPPSIKIHDRFTKFVASLNPMVKMPAKVDMYRYSKELFDKEKTKLKEKLAALRSRVCLSAYVWHYDLTSAFLCLSVHYIDDEWEKQSHIIRFHSVDPSCSAKQLSQDILYSIGNWGLRYKVFSITLDEAFLDDSVASDLKASLQEWNLLSSMSANRNSSMSANHTSSMSANHSIFVIRCPTHLLNQVIHVGMDELHKTMEKSTKCSKYTKGHI	zf-BED	II
HVU0042G1062.01	Hvu Zf-BED 22_I	MGEPKSNDNAMLHNGEMMMISSSEVVHCSEMLHGDNMVQESEILHGDEMVISGNEMVHGSEMIHGNEMVQVNDMIRSNDMDRVNNMVNGDEMAHGNALVSADLSPPTSSRRRKKKSPVWEHFTIQHVPGDKRCRIACCNLCKGTFAYSSGGKIAGTSHLKRHILQGSCPVIKNQERELALPLAEVAGNVGEGAIERPSKRRYRQTGFANATFDQDRSSLDLAKLIILHEYPLHVVQQPAFTALIGSLQPCFRVSDVDTMEGEVYAVYQKEKHNLLQALSTIPGRMSLTMGLWITSQTLGYVSLVGQFIDTDWRVHRRVLNFTMVASPHSQDALAEAISRSLFDWGMKEKLFAITLDNDCSSHDIYSANLRDHLSNKNGVMLKGQLFVVRCYAHILNAVAQDVIASAHGVIFSIRESIKFIKASDSYDQKFTEIALQLEIPSTKILCLDVTTQWDTTYLMLLAALDFRQVFTILETCDDNYNEAPSAEDWKKVEAASNCLKLLYDSAHSVMAVANPTSDIFFHEAWKLQLELANAAAHEDLVVSSTARDMHESFDKYWKDCNLVLAVAVVMDPRFKMKLVEFSYSKIYGVEAAKYVKVVNDSLHELYKEYLAQPLPARPVYVGQSASGNNNTQTTPPSIGDGLLDFDMYLSEIATAQPSKSELEQYLEEPLTPRSIQEFDILSWWKLNTLRFPTLSKMARDVLAIPMTMVSSIFSAETGIRTLDDYRSSLRPEIVEALVCAKDWLLHSPPASQTQSREMQ	zf-BED--DUF-domain--Dimer_Tnp_hAT--	I
HVU0042G1062.02	Hvu Zf-BED 23_I	MDGCLALGFDQIVFLSMGEPKSNDNAMLHNGEMMMISSSEVVHCSEMLHGDNMVQESEILHGDEMVISGNEMVHGSEMIHGNEMVQVNDMIRSNDMDRVNNMVNGDEMAHGNALVSADLSPPTSSRRRKKKSPVWEHFTIQHVPGDKRCRIACCNLCKGTFAYSSGGKIAGTSHLKRHILQGSCPVIKNQERELALPLAEVAGNVGEGAIERPSKRRYRQTGFANATFDQDRSSLDLAKLIILHEYPLHVVQQPAFTALIGSLQPCFRVSDVDTMEGEVYAVYQKEKHNLLQALSTIPGRMSLTMGLWITSQTLGYVSLVGQFIDTDWRVHRRVLNFTMVASPHSQDALAEAISRSLFDWGMKEKLFAITLDNDCSSHDIYSANLRDHLSNKNGVMLKGQLFVVRCYAHILNAVAQDVIASAHGVIFSIRESIKFIKASDSYDQKFTEIALQLEIPSTKILCLDVTTQWDTTYLMLLAALDFRQVFTILETCDDNYNEAPSAEDWKKVEAASNCLKLLYDSAHSVMAVANPTSDIFFHEAWKLQLELANAAAHEDLVVSSTARDMHESFDKYWKDCNLVLAVAVVMDPRFKMKLVEFSYSKIYGVEAAKYVKVVNDSLHELYKEYLAQPLPARPVYVGQSASGNNNTQTTPPSIGDGLLDFDMYLSEIATAQPSKSELEQYLEEPLTPRSIQEFDILSWWKLNTLRFPTLSKMARDVLAIPMTMVSSIFSAETGIRTLDDYRSSLRPEIVEALVCAKDWLLHSPPASQTQSREMQ	zf-BED--DUF-domain--Dimer_Tnp_hAT--	I
HVU0042G1062.03	Hvu Zf-BED 24_I	MGEPKSNDNAMLHNGEMMMISSSEVVHCSEMLHGDNMVQESEILHGDEMVISGNEMVHGSEMIHGNEMVQVNDMIRSNDMDRVNNMVNGDEMAHGNALVSADLSPPTSSRRRKKKSPVWEHFTIQHVPGDKRCRIACCNLCKGTFAYSSGGKIAGTSHLKRHILQGSCPVIKNQERELALPLAEVAGNVGEGAIERPSKRRYRQTGFANATFDQDRSSLDLAKLIILHEYPLHVVQQPAFTALIGSLQPCFRVSDVDTMEGEVYAVYQKEKHNLLQALSTIPGRMSLTMGLWITSQTLGYVSLVGQFIDTDWRVHRRVLNFTMVASPHSQDALAEAISRSLFDWGMKEKLFAITLDNDCSSHDIYSANLRDHLSNKNGVMLKGQLFVVRCYAHILNAVAQDVIASAHGVIFSIRESIKFIKASDSYDQKFTEIALQLEIPSTKILCLDVTTQWDTTYLMLLAALDFRQVFTILETCDDNYNEAPSAEDWKKVEAASNCLKLLYDSAHSVMAVANPTSDIFFHEAWKLQLELANAAAHEDLVVSSTARDMHESFDKYWKDCNLVLAVAVVMDPRFKMKLVEFSYSKIYGVEAAKYVKVVNDSLHELYKEYLAQPLPARPVYVGQSASGNNNTQTTPPSIGDGLLDFDMYLSEIATAQPSKSELEQYLEEPLTPRSIQEFDILSWWKLNTLRFPTLSKMARDVLAIPMTMVSSIFSAETGIRTLDDYRSSLRPEIVEALVCAKDWLLHSPPASQTQSREMQ	zf-BED--DUF-domain--Dimer_Tnp_hAT--	I
HVU0045G1107.01	Hvu Zf-BED 25_II	VWEHYEQELVVVEGDPKAVCKYCRGQFHTKFGTSSLRTHIAEACRSIEDACTKRFLLNIKKSHPKA	zf-BED	II
HVU0045G3460.01	Hvu Zf-BED 26_II	MLVLVSCSLVDVSLICCWLLHFAVMADGDGDAHEGVYDPMTDPTRMPRRSNDPGWKYGYWLKPPDINMVVCNLCGKITKGGIKRHKEHLTATGGDATGCPNATTQLRREMSEYLENNRRKIRQPNDDDDDVVEVHSTTMAPTVQCSTTRPSSGIAAKRNKKAFAVKITDKKCLAVAGTAA	zf-BED	II
HVU0600G0040.01	Hvu Zf-BED 27_II	MSTSRGSETADSTDDDTSQAPHARRSKVWEHYEQELVVVKGDLKAVCKYCRAQLHTKFGTSSLRTYIAEACRPIEDACRKRFELTMEKRPSEGMLVFDEKVSRELMVKFCIHAEIPFLKFEDPHLQPWIDSLQPTFQIKGRHMIHDDAMKMYKGMKKDIEVELQNLDYCICLTSDMWTSS	zf-BED	II
HVU0600G0040.02	Hvu Zf-BED 28_II	MSTSRGSETADSTDDDTSQAPHARRSKVWEHYEQELVVVKGDLKAVCKYCRAQLHTKFGTSSLRTYIAEACRPIEDACRKRFELTMEKRPSEGMLVFDEKVSRELMVKFCIHAEIPFLKFEDPHLQPWIDSLQPTFQIKGRHMIHDDAMKMYKGMKKDIEVELQNLDYCICLTSDMWTSS	zf-BED	II
HVU0600G0059.01	Hvu Zf-BED 29_II	MRFLPRFRRDPDLTMEAVEDDTTLEAAIGWLADTILANLPRIHQAAGLGNDIGKLKCEVEAVEMVVSAVQGRSAGNKPLARSLAAVKELLYDADDVIDELDGYRLQQEFQPETLLQTDGHGTLQTHENSRVNADHVQSSSNSRLRSKEWVHFDIIESEQNGGPSRARCNYCEKEIMCTTKMGTSVLSNHLKSKACSRKREETEPSSSTADATTTPAPVVTGSRKRMRTGQESSHITAANPNGRWNKDAFSERIQEITS	zf-BED	II
HVU0600G0059.03	Hvu Zf-BED 30_II	MEAVEDDTTLEAAIGWLADTILANLPRIHQAAGLGNDIGKLKCEVEAVEMVVSAVQGRSAGNKPLARSLAAVKELLYDADDVIDELDGYRLQQEFQPETLLQTDGHGTLQTHENSRVNADHVQSSSNSRLRSKEWVHFDIIESEQNGGPSRARCNYCEKEIMCTTKMGTSVLSNHLKSKACSRKREETEPSSSTADATTTPAPVVTGSRKRMRTGQESSHITAANPNGRWNKDAFSERIQ	zf-BED	II
Mapoly0048s0066.1	Map Zf-BED 01_XIII	MADTGKADKEKAECGKVVLHTYCHSSCGWRVRLALGLKGIPYEYKSWNLLTREHLSEEFKKISPLQLVPVLEIDGDVLVDSIAILEYLEERYPEKPLLPSDLKKRAAVRTVVNMIASSIQPIQNARVLQGIEKLAGPDARLEWAQEYITRGFTALESMLEKTSGKYCFGDEITLADVLLIPQMNNANRFKVDLSPFPTLVRLRDTLYELELVQESTVEKQPDFPKPTDSGVVDNRIVVHRASATGDHEEIVSQSEDDDCQETQPNEPQSGTSHSSRTEHTKEAKKHFDRYVVESGDNKHKEMARCKYCHREFVYNSTRMSQHLLGAKDSRNYVQTKACGAAPAHVVENLRRLTPGKLSSIRRPPHKYQRVAVDETAQRSNFSRQEREEIVRLVQLQLRQEQSVEVLQSKSA	GST_N_3--GST_C_3--zf-BED--	XIII
Mapoly0048s0066.2	Map Zf-BED 02_XIII	MADTGKADKEKAECGKVVLHTYCHSSCGWRVRLALGLKGIPYEYKSWNLLTREHLSEEFKKISPLQLVPVLEIDGDVLVDSIAILEYLEERYPEKPLLPSDLKKRAAVRTVVNMIASSIQPIQNARVLQGIEKLAGPDARLEWAQEYITRGFTALESMLEKTSGKYCFGDEITLADVLLIPQMNNANRFKVDLSPFPTLVRLRDTLYELELVQESTVEKQPDFPKPTDSGVVDNRIVVHRASATGDHEEIVSQSEDDDCQETQPNEPQSGTSHSSRTEHTKEAKKHFDRYVVESGDNKHKEMARCKYCHREFVYNSTRMSQHLLGAKDSRNYVQTKACGAAPAHVVENLRRLTPGKLSSIRRPPHKYQRVAVDETAQRSNFSRQEREEIVRLVQLQLRQEQSVEVLQSKSA	GST_N_3--GST_C_3--zf-BED--	XIII
Mapoly0048s0066.3	Map Zf-BED 03_XIII	MADTGKADKEKAECGKVVLHTYCHSSCGWRVRLALGLKGIPYEYKSWNLLTREHLSEEFKKISPLQLVPVLEIDGDVLVDSIAILEYLEERYPEKPLLPSDLKKRAAVRTVVNMIASSIQPIQNARVLQGIEKLAGPDARLEWAQEYITRGFTALESMLEKTSGKYCFGDEITLADVLLIPQMNNANRFKVDLSPFPTLVRLRDTLYELELVQESTVEKQPDFPKPTDSGVVDNRIVVHRASATGDHEEIVSQSEDDDCQETQPNEPQSGTSHSSRTEHTKEAKKHFDRYVVESGDNKHKEMARCKYCHREFVYNSTRMSQHLLGAKDSRNYVQTKACGAAPAHVVENLRRLTPGKLSSIRRPPHKYQRVAVDETAQRSNFSRQEREEIVRLVQLQLRQEQSVEVLQSKSA	GST_N_3--GST_C_3--zf-BED--	XIII
Mapoly0048	Map Zf-BED	MADTGKADKEKAECGKVVLHTYCHSSCGWRVRLALGLKGIPYEYKSWNLLTREHLSEEFKKISPLQLVPVLEIDGDVLVDSIAILEYLEERYPEKPLLPSDLKKRAAVRTVVNMIASSIQPIQNARVLQGIEKLAGPDARLEWAQEYITRGFTALESMLEKTSGKYCFGDEITLADVLLIPQMNNANRFKVDLSPFPTLVRLRDTLYELELVQESTVEKQPDFPKPTDSGVVDNRIVVHRASATGDHEEIVSQSEDDDCQETQPNEPQSGTSHSSRTEHTKEAKKHFDRYVVESGDNKHKEMARCKYCHREFVYNSTRMSQ	GST_N_3--GST_C_3--zf-BED--	XIII

s0066.<br>4	04_<br>XIII	HLLGAKDSRNYVQTKACGAAPAHVVENLRRLTPGKLSSIRRPPHKYQRVAVDETAQRSNFSRQEREEIVRLVQLQLRQE<br>QSVEVLQSKSA		
Mapol<br>y0152<br>s0005.<br>1	Map<br>Zf-<br>BED<br>05_<br>V	LGIVSSGRASGSTFATGRHSKKQKKTSWVFDHFYEDDDITGRVTCAVEGCGRSFSKGTSTTTLASHLTSKHRLTKGGVV<br>PGQVDQLTFSKNGGRLIAHNRLDDDKRARLFSKLVNWIVDNKQPIAVVENNFFKEFMHELKPFFNVSSCRTIGRGVDDAY<br>EEVKAKIALVLESIPGDVALTCDGWSSRIMRSYFVVTLHWINENWNLRSTVIEFIYFPPPHNQWTTSELLLSILKDLNLATRV<br>IAVTTDSGGEMPPAMRHVTAKLNEEFDDMLESDWLVRCVCHIINRAVKDCETIVKPMQVLLGRRNIVDLLCLDVETRWNS<br>MFEMVDNCFLVKDVFESMCNKEEFTAAGSSYVTLSIQPLIYESLKTHCLSTISGTLESGFTVPAAKRAAEAMLSKLEKYKE<br>NTNNPLCLLASGLDPSVPVLDEDIQELKFRIRQILIVRYGYQQQQIDEIPQRLGLLAAANKRFPLLSRLARDTFMVIGSSVPS<br>ESAFADSGSFVTPRRSSLSDENISKMMKLRSWNRLLKVLNK	zf-BED--<br>Dimer_Tnp_hAT<br>--	V
NNU_<br>00598-<br>RA	Nnu<br>Zf-<br>BED<br>01_<br>XXII	MFPFSISIEHQRCRCPARKPSSTLSFIGVDLKGEVTLPALSETFAASSMNFEAGTVSGKSGNLMEWNVNNAFKSLKDMEP<br>KAMMDMTLIPSIDPIDIGLGSSEKGNVAPPAKPRKKSMTSLYLKFFETAPDGKSRRCKFCKQSYSISTATGNLGRHLNHRH<br>PGYDKMGVGDAVNNTAPQPSPVTKKPQSQVKPPSVDLDHLNWLLLKFLILGSLPPSTLEEEWLSNSFKFLNSSVKFWTG<br>QKFQAVILEVFKSMRDDVKAYMEQVNSRVSITLDFWTSYEQVSYMSVKAHWIDENWSLHNILLDISHIPYPCGGTNIYQSL<br>VKVLKMYNIESRILSCTHDNSQNAMHLDGQKVVPFCYIPCAARTLNLIIEDGLRTVKPVISKIREFVLEMNASVEISEEFNKT<br>TAAYQEGSWKFPLDASTRWSGNYLMLDIVRKASKSMDAVIRNHEDTLGNRNMLLSPAEKNAINIMHAYLEPFYKTTNNIC<br>TSKVLTIGLVLFFMDHVSEMIAACRDSRHNPDWLKSTAEDMAKKSRSYNGQVYNVFTYMAAILDPRIKSDLIPESLNSENN<br>LEEARNHFMRNYSTSHFPAMANGYTAQDSEDGGSVSFAEEIARKRRRVSMSTATDELTQYLSEPPAPIPTDVLEWWKAN<br>STRYPRLSVMARDFLAVQATSVAPDELFCRKGDEVDKQKFCLPHGNMQSLLCIRSWIQSGFKLKYQSAEVDYEKLMESA<br>TAITDNGSTGFDKKPKSRTLMYRLDDNTGKANQENLSSKWAAAMAAVSSSLFPLLVCARISGLVVAVLVLIWALRFKTSFL<br>PHSSSQEDPIYAVLHPLLMVIGLIIISGEAILVHRWLPGSRNLKKSVHLCLQGVAVASGVFGIWTKFHGEKGIVENFYSLHS<br>WMGLICMFLFGAQWLMGFLSFWHRGEVRTVRLRVLPWHIFLGLYTYGLAVATAETGLLEKLTFLQTRRNVLKHSLESMV<br>VNSLGLGLALLSGVVILAAVSPKYQTPTKLMYSDTKCLSS	zf-BED--DUF-<br>domain--<br>Dimer_Tnp_hAT<br>--<br>Cytochrom_B56<br>1--	X<br>X<br>II
NNU_<br>01434-<br>RA	Nnu<br>Zf-<br>BED<br>02_<br>XXII	MDKIIFIKADRIGISLINIAEAWAAREAIFLSRELETKKIIVEGHSKLVIDCLNRRTKIPWSIKNIIEDCWEMTRVAELEITYQNTY<br>QEANQLVDFLANAGHLMSIYDAIAILDFHCQHSGGECNFYTIMVREKDVCWEYCDKLEGNKVRCKFCLKVLNGGISRLKH<br>HLSRVPSKGVHPCSKVRDEVTDRVRAIIAMKEEGKEAANAKKQRLAETKCPGNMSASKPLMPMEAMSPITKVFPSGSQIA<br>PPPSYDRDNAERSIALFFFENKLDFSVARSSSYQLMMDAVAKCGPGFRGPSSETLKTTWLERIKFEVSQQCKEIEKEWAT<br>TGCTIIADTWTDNKSRALINFLVSSPSGTFFHKSVDASTYFKNTKCLADLFDSVIQDFGPENVVQVIMDNALNYVGVGNHIM<br>QNYSTIFLSPCASHCLNLILEDFSKIDWVNRCILQAQTISRFIYNHTWVLDLMKKFTGGQELVRTGITKSVSNFLSLQSMLK<br>QRSRLKHMFNSPEYSSNPAYANKPQSISCIAILEDADFWRAVEESVAVSEPILKVLREVSGGKPAVGSIYEFMTRAKESIRT<br>YYIMDENKCKTFLDIVDRRWQNQLHSPLHAAAAFLNPSIQYNPEVKFLTLIKEEFFAVLERLLPTPELRHDIPAQIVVFKKAT<br>GMFGCNLAREARNTISPGLWWEQYGDSAPMLQRVAVRILSQVCSASTFERNWSTFQQIHSEKRNRLDKETLNDLLYINY<br>NLKLASRMKGKPIETDPILVDDIDMTSDWVEETENPSPTQWLDRFGSALDGGDLNSRQFNNSMFGANDHIF	RVT_3--zf-BED-<br>-DUF-domain--<br>Dimer_Tnp_hAT<br>--	X<br>X<br>II
NNU_<br>03623-<br>RA	Nnu<br>Zf-<br>BED<br>03_I	MMETELEPMPIGALKRDVAWKHCQMFKFENRTRLKCIYCGKMFSGGGIHRIKEHLAGQKGNGATCPRVHPDVRRIMRQ<br>SLEGSIVKSRRKQKRPEDIQNRLLGTGEMETMITQSEAITGLQLLAAPDAGEPHLRLLAKEEEGMTNRRMIGRTGNTSSPP<br>VTPHAHPTSNLTLGLPKGKDQVHMAIGRFLYDVGAPLDAVNSVYFQPMIDAIVSEGLGLKAPSYHDIRGWILKNSVEEVKG<br>VVDQYKGTWAKTGCSILADEWTTETGRILIHFLVYCPEGTVFLKFVDASDIVRSPDVLYELLKNVVEEVGVQNVLQVITDSA<br>DHYIIAGKKLTDTFPTMYWTPCAARCINLMLEDFGKFEWINAILEHVKSMTRFIYNHVVVLNMMRKYTYGKDLIQPALTRSA<br>TDFTILQNMVNLKSNLQDMVTSQEWMDCPYSKKPEGLTMIDIIYSQSFWSSCSSIIHLTEPLLRVLRMVGSHKRPAMGYIY<br>ECMYRAKEAIKKEFVEKKDYLVYWNVIDQRWNKQLTRPLQAAGFYLNPKFFYSIEGDVHNEIMSGMLDCIERLVSEIKIQD<br>KITKELSSYKNAVGDFGRKMAIRARNTMLPAEWWSTYGGGCPNLSRLAIRILNQTCSAVGFKQNEISFKQIHNVTRNQLE<br>HQRLSDLVFVQYNLRLRQRLLRKNQELDAMDPISFDNIDTVENWITEKEAVTMENGSSDWMALDQPLANAMLAPSPNDE<br>AYGLDAGFEDDEIFNGVRDEEENGRQLEEHINT	zf-BED--DUF-<br>domain--<br>Dimer_Tnp_hAT<br>--	I
NNU_<br>18735-<br>RA	Nnu<br>Zf-<br>BED<br>04_I<br>I	MASEPEPVPVSSQKHDPAWKHCQMFRNGDRYRLKCIYCGKMFSGGGIHRIKEHLAGQKGNAATCLNVQPEVRQLMQQ<br>SLDGGVLKRKKKLKIAEEKTDASPSPLLLEGTTTPAPPNELDTFATPGEATSGFQLITAPAPDTVEQNSGLLANREEEMGQ<br>VTDRSTDKRKRGRMENFSPPPVLPDAPPIGSNLTIGPGKGKEHVNLAIGRFLYDIGAPLDAVNSVYFQPMIEAIFESFWNS<br>CISIIRLTDPLLRVLRMVTSEKRPAMGYIYEAMYRAKQVIKKELVKKKDYVVYWNVIDQRWNQQLSRPLHAAGFYLNPSFF<br>YGIEGDVHNEIMSGMLDCIERLVPEIKTQDKITKELSSYKNAAGDFGRKMQLRRNKEADAMDPISIDNIDVVEDWVAEKEIL<br>LGEYVNSDWTSLVQPVANVMQSLSPNNEDEGMVEGYGDDEIYDGVNDDDVEEEDVQIQCQQEGYSDGNLEGQT	zf-BED	II
NNU_<br>21779-<br>RA	Nnu<br>Zf-<br>BED<br>05_<br>VII	MAPVRSTGFVDPGWEHGIAQDERKKKVKCNYCGKIVSGGIYRLKQHLARISGEVTYCKKAPEEVYLKMKENLEGCRSSK<br>KQRQSEDEEQASLDFHSNDDYEEEEGPVVFKRKGKQVTGDKNLVISLAPLRSLGYVDPGWEHGIAQDDRKKKVKCNYC<br>EKIVSGGINRFKQHLARIPGEVAYCKKAPEEVYLKMKENMKWHRTGRRQRRPDAKEIAAFYMHSDNDDEEEQDEDLLHK<br>EKMVIGDKSLGNDIRKRFRGRSPSTATPGSEPQLKRSRLDSVILRTPRSQTPTSYKQVKSKAASDKKTRKEVLSAICKFFY<br>HAAIPLNAANSPYFHKMLDLVAQHGQGLKGPSSRLISAKLAIKAIHGDDLRKYGSFWTVIDNHWNSLFHHPLYVAAYFLNP<br>SYRYRPDFLAHPEVIRGLNECIVRLEPDNGRRIAASMQISDFVSAKADFGTELAISTRTELDPEKQALQEDEEILYNEMEQT<br>EADENEVNENEDGNAEGRKGAVEMGVLPLVVTPMEVNPATMVAATDDDDDLDFLDDDLTD	zf-BED--zf-BED-<br>-	V<br>II
NNU_<br>23747-<br>RA	Nnu<br>Zf-<br>BED<br>06_I<br>I	MVAASENNNELAIANPEPQAQPNNKRRRKKSIVWEHFTIEAVGAGCTRACCKQCKQTFAYSTGSKLAGTSHLKRHIMLGT<br>CPATRRNQEKNQLTPYSPTKAGHCYAHVLSIIAQDALVALQETISKVRASVKYVKTSDTHEEKFIELKQQLQVPSTKSLSID<br>DLTKWNTTYLMLVAALELKEVFSCLDTSDPEYKDAPTMDDWKQVETLCIYLKLLYDAANILTSATYPTANIFFHEIKHFIFIIN<br>KIYNLEGSHAVV	zf-BED	II
NNU_<br>24793-<br>RA	Nnu<br>Zf-<br>BED<br>07_I<br>I	MDSSGASSNVHDHGTPVDEQKKRFRCNYCAKVVSGSTRLKQHLAGVRGDVVPCEQVPEDVKVQMRSCFLDTKKGVLT<br>REVGQLYHPDLPLKRNWCSNSANPNHSQPGSKGKGKRVILNSALDECVERKVPNKIFHSSTGIKSDEGEEDSLRHAQRE<br>NLRNMFASSAWNTSVWASSTEGKCVADLVGEPSFWSGAQMVLKAAIPLVRVLCLINGRDHRPQMGYIYETMDQMKETIK<br>TEFKDKKTKYLPFWGLIDDIWDNFLHSPLHSAGYFLNPSLFYSSDFLADAEVASGLLCCIVRMVKDRQVQDLISLQLDEYR<br>AANGSFGHGSAIDQRTRMPPDEVQDVMAQSEDSCAVDLDCGMGIAQRVISEEGPSDLQPKKEPR	zf-BED	II
NNU_<br>25566-<br>RA	Nnu<br>Zf-<br>BED<br>08_I	MMVNGDRQKIKCRYCHKVILGGGISRLKQHLAGERGNIAPCEKVPDDVKAQMQQHLGFKVLEKLKRQKESQTIKTPLISYI<br>HDREEGNYDEGQRSPKGTSARGSIGRKRGKEGEEGTSGRRKRHKKQLTPTTAPAQPLMHLNFASQESMDQADMAVAK<br>FMYDAGIPFSAANSFYFQLMADAIAAVGPGYKMPSYHSLRGKLLNKTVQETGELCQELKRSWEVTGCSVLVDRWTDRTD<br>RTVLNFFAYCPKGTMFLRSVDATETTKSSEALLDLFDSIVQDVGPKNIVHFVTDTTPNYKAAGKALMNKYKTFFWSACAA<br>HCIDLMLEEFGKMDQVKEVLARAKRISQFIYNHAWVLNLMRKKTGGRDIVRPAITRFATNFLALQSIVSLKDPLHEMFTSTS<br>WMQSAFSKQRAGIEVAETVVDPSFWSLCAEILKVTKPLLTVLHLIDTEERPSMGYIYDAMEKARKGIIVAFNNKESEYYPYL<br>KIIDQIWEEELHSPLHAAAYYLNPSIFYNPSFSTNKVIQKGLLDCIETLEPNLTAQEMITRHITFYEDAVGDFSRPVAVRGRE<br>SLAPATWWSLYAADYPDLQRFAIRISSQTCSGTRCERNWSMFERIHSKKRNRLEHERLNDLIFVHYNLRLQERWQEELMI<br>PYAWRGWVLMQEIGLTILESSRGRILVGWMWQYPVIHLMQVQS	zf-BED--DUF-<br>domain--<br>Dimer_Tnp_hAT<br>--	I
NNU_<br>26458-<br>RA	Nnu<br>Zf-<br>BED<br>09_I<br>I	MLATTENNEPISTEPTQSNKRRRKKSIVWEHFTIEPVGAGCTRACCKQCKQTFAYSTGTKLAGTSHLKRHIMLGTCPATR<br>RNQEKNQLTPYTPNSKPGYFSIKNPNMLNGQLLIGHCYAHVLSSVAQDALGALQEIINKVRTSVKFVKTSDAHEEKFIELK<br>QQLQVPSTKSNLLTSATYPTANTFFHEGIHELFLEYMAQPLPLTPTYVERETPKTESPGGILLSS	zf-BED	II
OsR49<br>8G010<br>03781<br>00.01.<br>T01	OsZ<br>f-<br>BED<br>01_I	MDDDPTSVNYKLRTMGMRGDDDDDVEEDRVEVFGNTSDIPIHVNVDDDDPPVDDGGNGTPTGSSATCTNKKTKTSKV<br>WDDFEELYETTNGNRVRVFAKCNYCHKTLSARSSAGTGHLLRHIKSCKPRKLGSNALPQSMLRFSADGSVIPWEYSPEV<br>ARFELCRLIAREDLPISFGQSPAFVNYIKAAHNPRFVPVSRQTTTRDFYKLFKDRRSVIIDRLNSASSIALTSDTWSGHAKE<br>DYLSVVAHFVNSDWQLEKRVLGLRLIDESHTGANIAERVIAVAEEYGITDKVFSITLDNASANSKAMDTLTPALSGYIGDLFL<br>HQRCACHIINLIVKAGLDKFKPMLNDIRAAISFLNASNQRIATYKNVCIAAGYRPRMFGLDMDVRWNSTYLMLKHLIPHREP<br>FTDLLVHNFVNDHPLLTDLHWACAESVLCFLKQFYDSTVVLSGVYYPTSPLIMHHILEIAGHLNTYGNVQNIANVVGPMKT<br>KFMNYWSKIPILYSFAFILDPRAKIRGFSKVLQIMAQLIGDDYSAYLTTVRASLSDTFAKYERKFGSVRLHSSTIPGPSTGKK<br>RTAWGKIFGSVVAAGLGAGNAGASPGAGNAGASPGAGLGAGSLSRMTSATALLQAASSTANLNSSELSAYLDSDTVNQ<br>YDDDFNILSWWQQHKLTYPVLSILAKDVMTVPVSTISSESTFSLTGRIIEDRRRRLNPRLVEILAVIKDWELAYAKSQHTTEN<br>VELQNAYENMYLDDEIDVNP	zf-BED--DUF-<br>domain--<br>Dimer_Tnp_hAT<br>--	I

OsR498G0100443400.01.T01	OsZf-BED02_I	MRERDVCWEYCDKMEGNKVRCRFCYKVLNGGISRLKFHLSQISSKGVNPCTKVKPDVIEKVKAVIAAKEEHRETQVLKRQRDTELSVRPRRIRDLPSQPTSPERATSPAITSTSDQTQFLALEVSTPVLKLSSVTNKARSAPQSEAERCIAEFFFENKLDYNIADSVSYRHMMEALGGQGFRGPSAEVLKTKWLHKLKSEVLQKTKEIEKDWATTGCTILADSWTDNKSKALINFSVSSPLGTFFLKTVDASPHIKSHQLYELFDDVIREVGPDNVVQIITDRNINYGSVDKLIMQNYNTIFWSPCASSCVNSMLDDFSKIDWVNRCICQAQTITRFVYNNKWVLDLMRKCIAGQELVCSGITKCVSDFLTLQSLLRHRPKLKQMFHSSDYASSSYANRSLSSSCVEILDDDEFWRAVEEIAAVSEPLLRVMRDVLGGKAAIGYIYESMTKVMDSIRTYYIMDEGKCKSFLDIVEQKWQVELHSPLHSAAAFLNPSIQYNPEVKFFTSIKEEFYHVLDKVLTVPDQRQGITVELHAFRKAQGMFGSNIAKEARNNTSPGMWWEQYGDSAPSLQHAAVRIVSQVCSTLTFQRDWSIIVRNHSEKRNKLDKEALADQAYVHYNFMLHSDSKMKKGDGDPIALDAIDMTSPWVEDSDSPNLAQWLDRFPSALDGDLNTRQFGGSIFGTNDTLFGL	zf-BED--DUF-domain--Dimer_Tnp_hAT--	I
OsR498G0100443400.01.T02	OsZf-BED03_I	MRERDVCWEYCDKMEGNKVRCRFCYKVLNGGISRLKFHLSQISSKGVNPCTKVKPDVIEKVKAVIAAKEEHRETQVLKRQRDTELSVRPRRIRDLPSQPTSPERATSPAITSTSDQTQFLALEVSTPVLKLSSVTNKARSAPQSEAERCIAEFFFENKLDYNIADSVSYRHMMEALGGQGFRGPSAEVLKTKWLHKLKSEVLQKTKEIEKDWATTGCTILADSWTDNKSKALINFSVSSPLGTFFLKTVDASPHIKSHQLYELFDDVIREVGPDNVVQIITDRNINYGSVDKLIMQNYNTIFWSPCASSCVNSMLDDFSKIDWVNRCICQAQTITRFVYNNKWVLDLMRKCIAGQELVCSGITKCVSDFLTLQSLLRHRPKLKQMFHSSDYASSSYANRSLSSSCVEILDDDEFWRAVEEIAAVSEPLLRVMRDVLGGKAAIGYIYESMTKVMDSIRTYYIMDEGKCKSFLDIVEQKWQVELHSPLHSAAAFLNPSIQYNPEVKFFTSIKEEFYHVLDKVLTVPDQRQGITVELHAFRKAQGMFGSNIAKEARNNTSPGMWWEQYGDSAPSLQHAAVRIVSQVCSTLTFQRDWSIIVRNHSEKRNKLDKEALADQAYVHYNFMLHSDSKMKKGDGDPIALDAIDMTSPWVEDSDSPNLAQWLDRFPSALDGDLNTRQFGGSIFGTNDTLFGL	zf-BED--DUF-domain--Dimer_Tnp_hAT--	I
OsR498G0100443400.01.T03	OsZf-BED04_I	MRERDVCWEYCDKMEGNKVRCRFCYKVLNGGISRLKFHLSQISSKGVNPCTKVKPDVIEKVKAVIAAKEEHRETQVLKRQRDTELSVRPRRIRDLPSQPTSPERATSPAITSTSDQTQFLALEVSTPVLKLSSVTNKARSAPQSEAERCIAEFFFENKLDYNIADSVSYRHMMEALGGQGFRGPSAEVLKTKWLHKLKSEVLQKTKEIEKDWATTGCTILADSWTDNKSKALINFSVSSPLGTFFLKTVDASPHIKSHQLYELFDDVIREVGPDNVVQIITDRNINYGSVDKLIMQNYNTIFWSPCASSCVNSMLDDFSKIDWVNRCICQAQTITRFVYNNKWVLDLMRKCIAGQELVCSGITKCVSDFLTLQSLLRHRPKLKQMFHSSDYASSSYANRSLSSSCVEILDDDEFWRAVEEIAAVSEPLLRVMRDVLGGKAAIGYIYESMTKVMDSIRTYYIMDEGKCKSFLDIVEQKWQVELHSPLHSAAAFLNPSIQYNPEVKFFTSIKEEFYHVLDKVLTVPDQRQGITVELHAFRKAQGMFGSNIAKEARNNTSPGMWWEQYGDSAPSLQHAAVRIVSQVCSTLTFQRDWSIIVRNHSEKRNKLDKEALADQAYVHYNFMLHSDSKMKKGDGDPIALDAIDMTSPWVEDSDSPNLAQWLDRFPSALDGDLNTRQFGGSIFGTNDTLFGL	zf-BED--DUF-domain--Dimer_Tnp_hAT--	I
OsR498G0100443400.01.T04	OsZf-BED05_I	MRERDVCWEYCDKMEGNKVRCRFCYKVLNGGISRLKFHLSQISSKGVNPCTKVKPDVIEKVKAVIAAKEEHRETQVLKRQRDTELSVRPRRIRDLPSQPTSPERATSPAITSTSDQTQFLALEVSTPVLKLSSVTNKARSAPQSEAERCIAEFFFENKLDYNIADSVSYRHMMEALGGQGFRGPSAEVLKTKWLHKLKSEVLQKTKEIEKDWATTGCTILADSWTDNKSKALINFSVSSPLGTFFLKTVDASPHIKSHQLYELFDDVIREVGPDNVVQIITDRNINYGSVDKLIMQNYNTIFWSPCASSCVNSMLDDFSKIDWVNRCICQAQTITRFVYNNKWVLDLMRKCIAGQELVCSGITKCVSDFLTLQSLLRHRPKLKQMFHSSDYASSSYANRSLSSSCVEILDDDEFWRAVEEIAAVSEPLLRVMRDVLGGKAAIGYIYESMTKVMDSIRTYYIMDEGKCKSFLDIVEQKWQVELHSPLHSAAAFLNPSIQYNPEVKFFTSIKEEFYHVLDKVLTVPDQRQGITVELHAFRKAQGMFGSNIAKEARNNTSPGMWWEQYGDSAPSLQHAAVRIVSQVCSTLTFQRDWSIIVRNHSEKRNKLDKEALADQAYVHYNFMLHSDSKMKKGDGDPIALDAIDMTSPWVEDSDSPNLAQWLDRFPSALDGDLNTRQFGGSIFGTNDTLFGL	zf-BED--DUF-domain--Dimer_Tnp_hAT--	I
OsR498G0100443400.01.T05	OsZf-BED06_I	MRERDVCWEYCDKMEGNKVRCRFCYKVLNGGISRLKFHLSQISSKGVNPCTKVKPDVIEKVKAVIAAKEEHRETQVLKRQRDTELSVRPRRIRDLPSQPTSPERATSPAITSTSDQTQFLALEVSTPVLKLSSVTNKARSAPQSEAERCIAEFFFENKLDYNIADSVSYRHMMEALGGQGFRGPSAEVLKTKWLHKLKSEVLQKTKEIEKDWATTGCTILADSWTDNKSKALINFSVSSPLGTFFLKTVDASPHIKSHQLYELFDDVIREVGPDNVVQIITDRNINYGSVDKLIMQNYNTIFWSPCASSCVNSMLDDFSKIDWVNRCICQAQTITRFVYNNKWVLDLMRKCIAGQELVCSGITKCVSDFLTLQSLLRHRPKLKQMFHSSDYASSSYANRSLSSSCVEILDDDEFWRAVEEIAAVSEPLLRVMRDVLGGKAAIGYIYESMTKVMDSIRTYYIMDEGKCKSFLDIVEQKWQVELHSPLHSAAAFLNPSIQYNPEVKFFTSIKEEFYHVLDKVLTVPDQRQGITVELHAFRKAQGMFGSNIAKEARNNTSPGMWWEQYGDSAPSLQHAAVRIVSQVCSTLTFQRDWSIIVRNHSEKRNKLDKEALADQAYVHYNFMLHSDSKMKKGDGDPIALDAIDMTSPWVEDSDSPNLAQWLDRFPSALDGDLNTRQFGGSIFGTNDTLFGL	zf-BED--DUF-domain--Dimer_Tnp_hAT--	I
OsR498G0100443400.01.T06	OsZf-BED07_I	MRERDVCWEYCDKMEGNKVRCRFCYKVLNGGISRLKFHLSQISSKGVNPCTKVKPDVIEKVKAVIAAKEEHRETQVLKRQRDTELSVRPRRIRDLPSQPTSPERATSPAITSTSDQTQFLALEVSTPVLKLSSVTNKARSAPQSEAERCIAEFFFENKLDYNIADSVSYRHMMEALGGQGFRGPSAEVLKTKWLHKLKSEVLQKTKEIEKDWATTGCTILADSWTDNKSKALINFSVSSPLGTFFLKTVDASPHIKSHQLYELFDDVIREVGPDNVVQIITDRNINYGSVDKLIMQNYNTIFWSPCASSCVNSMLDDFSKIDWVNRCICQAQTITRFVYNNKWVLDLMRKCIAGQELVCSGITKCVSDFLTLQSLLRHRPKLKQMFHSSDYASSSYANRSLSSSCVEILDDDEFWRAVEEIAAVSEPLLRVMRDVLGGKAAIGYIYESMTKVMDSIRTYYIMDEGKCKSFLDIVEQKWQVELHSPLHSAAAFLNPSIQYNPEVKFFTSIKEEFYHVLDKVLTVPDQRQGITVELHAFRKAQGMFGSNIAKEARNNTSPGMWWEQYGDSAPSLQHAAVRIVSQVCSTLTFQRDWSIIVRNHSEKRNKLDKEALADQAYVHYNFMLHSDSKMKKGDGDPIALDAIDMTSPWVEDSDSPNLAQWLDRFPSALDGDLNTRQFGGSIFGTNDTLFGL	zf-BED--DUF-domain--Dimer_Tnp_hAT--	I
OsR498G0100582800.01.T01	OsZf-BED08_II	MDDDPTSVNYELRTMGMRGDDDDDVEEDRVEVFGNTSDIPIHVNVDDDDPPVDDSGNGTPTDSSATCTNKKTKTSKVWDDFEELYETTNGNRVRVSAKCNYCHKTLSARSSAGTGHLLRHIKSCKPRKLGSNALPQSMLRFSADGSVIPWEYSPEDARFELCRLIARREDLPISFGQSPAFVNYIKAAHNPRFVPVSRQTTTRDFYKLFKDRRSVIIDRLNSASSIALTSDIWSGHAKEDYLSVVAHFVNSDWQLEKRVLGLRLIDESHTGANIAERV	zf-BED	II
OsR498G0101123100.01.T01	OsZf-BED09_II	MDEMIPKPLISNDNEMMHGHGYTTMVHGNNEILNGNELAVHAEVIPSASTRGQKRKSAIWEHFTLVDVSDGCKRASCIHCNQSLAYSSGSKNSGTSHLTRHIAEWCRVLKDRQKSRRYTTYNSSN	zf-BED	II
OsR498G0101261400.01.T01	OsZf-BED10_II	MDGVDKDKGNGKVHFRDSSPQEAFRTYKRRRQPRPEPQQQPQPQPQSEPEPQQQPQPQPQPQPQPEAEAEAEAEAKAADVLARQVTETFWKSRDIGWKHGIMIDENRQHWKCMYCHLTRYGGGVSRLKRHLAGDLDVKMCPKVPADVSEKIREHLRKKRERRKKRAAQNRDNCVTAKSTSDDIKSGKDPLPVDSEVLTGVDTVLEEVTNQTNHDNQDLTYPKATMLLRGIRDIGWEHAVDLDGNKRRWKCKWCSLCRSGGVTTLKAHLTDSSCPNIPKEISKKVLNFIEEKRAARHLFNSAAKSPFNVKFDEDAVNLSEIQVEGTPPLTDDRQPLGNSLHIQTSECTINEFEKVAAGSNQQGAEHSNQLLNHGEQLMKSSDQPEEHCTLEHGRCQVLDNNKQQTMDNKTDNPEHKEVLKHPKKTRFNIRKHIVIVDESARHWRCRYCGLDGYGKTSRLHFHLAAVFRHPKCPSVPKEVFAKARHHIHLKRRLNVKKAGQQARSRPHILGQSSQQQQNNNPVLSNYPTRLRDNAWEHSLIHDKEKGHWKCKWCSLEGYHGITRLKWHLVGWQNRPQCLNVPEDVAKTIRDKMISREKQKEGRLNLDVIDSCNMPCSSESLQFDQENFAEVMQGKGSSEDFNQAERQSNTLNTVCNTTHPPQNSNNYQGLQENGLYSSKNKSEKQTERYDCWSHWRYVLDGLMHLPGALEGPGIQSCIRDVLLYGSAEFGTVGDKVEMDSNRKVSSDGNIAKCQSVLVDVLKSENFALLCNVLGRTVHQDEQRTKYFDFTMIDSRMKNGDYGRAPLLFKHDLKMVTPLL	zf-BED	II
OsR498G0101261400.01.T02	OsZf-BED11_VI	MDGVDKDKGNGKVHFRDSSPQEAFRTYKRRRQPRPEPQQQPQPQPQSEPEPQQQPQPQPQPQPQPEAEAEAEAEAKAADVLARQVTETFWKSRDIGWKHGIMIDENRQHWKCMYCHLTRYGGGVSRLKRHLAGDLDVKMCPKVPADVSEKIREHLRKKRERRKKRAAQNRDNCVTAKSTSDDIKSGKDPLPVDSEVLTGVDTVLEEVTNQTNHDNQDLTYPKATMLLRGIRDIGWEHAVDLDGNKRRWKCKWCSLCRSGGVTTLKAHLTDSSCPNIPKEISKKVLNFIEEKRAARHLFNSAAKSPFNVKFDEDAVNLSEIQVEGTPPLTDDRQPLGNSLHIQTSECTINEFEKVAAGSNQQGAEHSNQLLNHGEQLMKSSDQPEEHCTLEHGRCQVLDNNKQQTMDNKTDNPEHKEVLKHPKKTRFNIRKHIVIVDESARHWRCRYCGLDGYGKTSRLHFHLAAVFRHPKCPSVPKEVFAKARHHIHLKRRLNVKKAGQQARSRPHILGQSSQQQQNNNPVLSNYPTRLRDNAWEHSLIHDKEKGHWKCKWCSLEGYHGITRLKWHLVGWQNRPQCLNVPEDVAKTIRDKMISREKQKEGRLNLDVIDSCNMPCSSESLQFDQENFAEVMQGKGSSEDFNQAERQSNTLNTVCNTTHPPQNSNNYQGLQENGLYSSKNKSEKQTERYDCWSHWRYVLDGLMHLPGALEGPGIQSCIRDVLLYGSAEFGTVGDKVEMDSNRKVSSDGNIAKCQSVLVDVLKSENFALLCNVLGRTVHQDEQRTKYFDFTMIDSRMKNGDYGRAPLLFKHDLKMLWEDLKMAGQDIIDLANNLSSLTETSYTKQVERERGSDDSEENLKGAAATNLEPMNMVKSNALVLSTSQGFNQLDQPDPMDVCDEQNGTNCNECGKVAKIDSILTCKRCMLAFHVSCIEPPVPSTSTGSWCCKTCSTICNESAEVGMALVHYEPNRLHGHCVACKDLEFCRPPRCEETASERAPADNSRAIVIPSAEPVEDVELSDIDVRGL	zf-BED--PHD--	VI
OsR498G01012614	OsZf-BED	MDGVDKDKGNGKVHFRDSSPQEAFRTYKRRRQPRPEPQQQPQPQPQSEPEPQQQPQPQPQPQPQPEAEAEAEAEAKAADVLARQVTETFWKSRDIGWKHGIMIDENRQHWKCMYCHLTRYGGGVSRLKRHLAGDLDVKMCPKVPADVSEKIREHLRKKRERRKKRAAQNRDNCVTAKSTSDDIKSGKDPLPVDSEVLTGVDTVLEEVTNQTNHDNQDLTYPKATMLLRGIRDIGWEHAVDLDGNKRRWKCKWCSLCRSGGVTTLKAHLTDSSCPNIPKEISKKVLNFIEEKRAARHLFNSAAKSPFNVKFDEDA	zf-BED	II

00.01.T03	12_II	VNLSEIQVEGTPPLTDDRQPLGNSLHIQTSECTINEFEKVAAGSNQQGAEHSNQLLNHGEQLMKSSDQPEEHCTLEHGRCQVLDNNKQQTMDNKTDNPEHKEVLKHPKKTRFNIRKHIVIVDESARHWRCRYCGLDGYGKTSRLHFHLAAVFRHPKCPSVPKEVFAKARHHIHLKRRLNVKKAGQQARSRPHILGQSSQQQQNNNPVLSNYPTRLRDNAWEHSLIHDKEKGHWKCKWCSLEGYHGITRLKWHLVGWQNRPQCLNVPEDVAKTIRDKMISREKQKEGRLNLDVIDSCNMPCSSESLQFDQENFAEVMQGKGSSEDFNQAERQSNTLNTVCNTTHPPQNSNNYQGLQENGLYSSKNKSEKQTERYDCWSHWRYVLDGLMHLPGALEGPGIQSCIRDVLLYGSAEFGTVGDKVEMDSNRKVSSDGNIAKCQSVLVDVLKSENFALLCNVLGRTVHQDEQRTKYFDFTMIDSRMKNGDYGRAPLLFKHDLKMVTPLL		
OsR498G0101261400.01.T04	OsZf-BED13_II	MDGVDKDKGNGKVHFRDSSPQEAFRTYKRRRQPRPEPQQQPQPQPQSEPEPQQQPQPQPQPQPQPEAEAEAEAEAKAADVLARQVTETFWKSRDIGWKHGIMIDENRQHWKCMYCHLTRYGGGVSRLKRHLAGDLDVKMCPKVPADVSEKIREHLRKKRERRKKRAAQNRDNCVTAKSTSDDIKSGKDPLPVDSEVLTGVDTVLEEVTNQTNHDNQDLTYPKATMLLRGIRDIGWEHAVDLDGNKRRWKCKWCSLCRSGGVTTLKAHLTDSSCPNIPKEISKKVLNFIEEKRAARHLFNSAAKSPFNVKFDEDAVNLSEIQVEGTPPLTDDRQPLGNSLHIQTSECTINEFEKVAAGSNQQGAEHSNQLLNHGEQLMKSSDQPEEHCTLEHGRCQVLDNNKQQTMDNKTDNPEHKEVLKHPKKTRFNIRKHIVIVDESARHWRCRYCGLDGYGKTSRLHFHLAAVFRHPKCPSVPKEVFAKARHHIHLKRRLNVKKAGQQARSRPHILGQSSQQQQNNNPVLSNYPTRLRDNAWEHSLIHDKEKGHWKCKWCSLEGYHGITRLKWHLVGWQNRPQCLNVPEDVAKTIRDKMISREKQKEGRLNLDVIDSCNMPCSSESLQFDQENFAEVMQGKGSSEDFNQAERQSNTLNTVCNTTHPPQNSNNYQGLQENGLYSSKNKSEKQTERYDCWSHWRYVLDGLMHLPGALEGPGIQSCIRDVLLYGSAEFGTVGDKVEMDSNRKVSSDGNIAKCQSVLVDVLKSENFALLCNVLGRTVHQDEQRTKYFDFTMIDSRMKNGDYGRAPLLFKHDLKMVTPLL	zf-BED	II
OsR498G0101827100.01.T01	OsZf-BED14_II	MLEPTAMEVPITSSVNTAVVPVPVVHNPRARKLRSAVWQDFTKERRADGSCVAVCNHCKKQLTATSRSGTTHLRNHLAICTTTSTRRAGKRRKLIVRRILHNKTSTDGQSGDGHASGEDHDNDGTHFDQELSRRDLAHMIVQHGYRFSIVDDVGFQKFVKNLQPQFRMVSYETVRADSMTIYESEKLKLQEASCHCTGQL	zf-BED	II
OsR498G0101827100.01.T02	OsZf-BED15_I	MLEPTAMEVPITSSVNTAVVPVPVVHNPRARKLRSAVWQDFTKERRADGSCVAVCNHCKKQLTATSRSGTTHLRNHLAICTTTSTRRAGKRRKLIVRRILHNKTSTDGQSGDGHASGEDHDNDGTHFDQELSRRDLAHMIVQHGYRFSIVDDVGFQKFVKNLQPQFRMVSYETVRADSMTIYESEKLKLQDALLKIPCRLSISVDMWRSNTQMDFLCLTCHYIDHANDEWKVRKKILNFVHVEAPFTDDQIASLILEKLREWGIDRKLAAIVLDNCASGEIVARELLVVLQPRRLLLLNGNLFQVRSCAHILNLTVQESLEQTSDIITRVREMIQNVKFSQERFEKFQDTAKLLQMDQKLLVLDSPNNWPSTYLMFDSACYYHDVLMRLAEQEAHYGAFLTAKEWADVKALTEILDALYHKMEKFPVENPTANLYFNDMCEVHVLLNTWRNSPSPVVAQVADRMLTKFEGYWDLTRPVMAFASILDPRYKMKSVEYFCRLIYAADQFRAKTTIDDIRQSFTNLCSEYEQSGNSFKNPSALFYSATSNSCMSSVYSNGDDFKTFSRITLSDARRGLDQYIQETSSGQSFKSDLDMYLEEPVYRQKEGHLDNFDILGWWKSFAAKYPVLSQMARDILAIPVSIIPLDSDARTLNEYLSTMDPSTVQGLVCAQDWLREDTEVASSDGHADDKAARGDELIVLPK	zf-BED--DUF-domain--Dimer_Tnp_hAT--	I
OsR498G0203238600.01.T01	OsZf-BED16_II	MDPNFPYQSPSFTLGDFDPNYMSGFDGTSGSAPTPPSVEEVPVHTAVVEEVPVQAATASEGFSGTASGSVSTHTGSKRSRTSVVWQNFDEIKETCPDGREVSKARCRICRQILSARSSGGTGHLKRHAESCAKKQGIQLRQQQLMVNPDGTVRSWEYDPMVARESLVRL	zf-BED	II
OsR498G0203342600.01.T01	OsZf-BED17_II	MAENNHQPTNAADHSKWSNCWDHFQEKAGQKAECRHCKKQLSFKNGTSRLNRHYQDTCPARKRARQQGSHGASVQAGIHDLQDAPAFSRSSSDNFGEQTA	zf-BED	II
OsR498G0203343400.01.T01	OsZf-BED18_II	MGSECWAHFEKKADNMAECRHCHNLLSYKNGTSRLNRHYQESCRKRGRQQGSHHGASAFSRSSSDDLGEQTAAAAAAEDQLIRMIALHGFPSSMVEDVEFTRFVRMLCPDFKMPSRDDVRKRCDELFDREMSSLKDAIGRTPGLASLSLGKTTTAWGKVAYLAAHFIDEEWNFHCRVIQVFKALE	zf-BED	II
OsR498G0204619100.01.T01	OsZf-BED19_I	MDMVAISDGGEDEKNSFDLNDEMSVSLPSSRSSSEGEDGDDTSKTQKRGAKRPKAGISLSPSKVKITRGKRASCWKYYKVINVPSKKEKGKMECKAKCRFCHHNYAYRPGGTTTTLNRHLDKCTIYLNKLAKPKAQGTLDFPLADGSMVVHPTEYDHDHTKLLIARMIILHDYPFRIVEHKGFNALMKWMNPSYEFIGRKAIKSECMKLYESEKEHLRKSLREAETISLTTDMWTSNQNLQYMCLVAHYIDVNWVLQCRVLNFVEVEPPHTGIVIAQAIFDCLVDWKIEDKVMIITLDNASNNDTAVSNLKSKLAARKNAQFDPDYFHVRCAAHIVNLVVNDGLQQIQSLITNVRNTVKYIMIIATILDPRFKMRYIRWCFSEFFGETRCVTEVAAITNEMEKLYRKYERICRHNQGGNSPHNGHSASSSISTTTSLASIIPSGFQSFLQSNAKESSKSELLIYLDEPNVSLEDSTFNLLNYWKVNAHRFPIVSNMAKRFLAVPASSVSSESTFSTEGRILDDYRSSLKPETVQALVCASSWIRASQNDNSAPIPVGENGDDDIEIVDFPNCVVASN	zf-BED--DUF-domain--Dimer_Tnp_hAT--	I
OsR498G0204839800.01.T01	OsZf-BED20_I	MDDDPTSVNYELRTMGMRGDDDDDVEEDRVEVFGNTSDIPIHVNVDDDDPLVDDSGNGTPTGSSATYTNKKTKTSKVWDDFEELYETTNGNRVRVSAKCNYCHKTLSARSSAGTGHLLRHIKSCKPRKLGSNALPQSMLRFSADGSVIPWEYSPEDARFELCRLISREDLPISFGQSPAFVNYIKAAHNPRFVPVSRQTTTRDFYKLFKDRRSVIIDRLNSASSIALTSDIWSGHAKEDYLSVVAHFVNSDWQLEKRVLGLRLIDESHTGANIAERVIAVAEEYGITDKVFSITDNASANSKAMITLTPALSGYIGDLFLHQRCACHIINLIVKAGLDKFKPMLNDIRAAISFLNASNQRIATYKNVCIAAGYRPRMFGLDMDVRWNSTYLMLKHLIPHREPFTVFISTQHPFVNDHPLLTYLHWACAESVLCFLEQFYDSTVVLSGVYYPTSPLIMHHILDIAGHLNTYGNVQNIANVVGPMKTKFMNYWSKIPILYSFAFILDPRAKIRGFSKVLQIMAQLIGDDYSAYLTTVRASLFDTFAKYERKFGSVRLHSSTIPGPSTGKKRTAWGKIFGSVVAAGLGAGNAGASPGAGNAGASPGAGLGAGSLSRMTSATALLQAASSTANLNSSELSAYLDSDTVNQYDDNFNILSWWQQHKLTYPVLSILAKDVMTVPVSTISSESTFSLTGRIIEDRRRRLNPRLVEILAVIKDWELADAKSQHTTENVELQNAYENMYLDDEIDVNP	zf-BED--DUF-domain--Dimer_Tnp_hAT--	I
OsR498G0305186600.01.T01	OsZf-BED21_IV	MDESNIPSFTLGDFDPNYVSRSFPTGEYDATGSAPTPPVMEPLAGSEASNTMSGSASTNTGSKRSRTSGVWQHFDEVAMTGPDGRQVTFARCRICKNKLSAKSSGGTGHLKRHAEACAKKQGIQLRQQQLLLNPDGTVRTWEYDPMVARENLARLIARQDLPLNFGESPAFENYIKNSHNPRFQAVSRQTTTRDLKNVHDKGYESLKELFSTCTFSVSVTSDIWSSRAKEDYLSVVVHFIDDDWQMQKRVLGLRLIDVSHTGENIAERIREVIDEFNLADKIFAVTMDNASANSRAMEILQPLFCIYAQSFLLHQRCACHIINLIVKCGFKRVNVHIDAVRQAITWLTASNPRIAQWKRYCCASGEPPRKFLTDADHRWNATYFMLKVVLPYKDLLTVFLQTRNGPKNSDGQPILTDHTWHIVERFNQFLETFHDCTLLLSLLLSQVYYPTANLILHNILEIATLLKEYENDDLLMPVVFNMKQKYLKYWKDIPMLYSFAFILDPRGKLRGFLNILSLIGDIINVDYSTYYADVKTKFYEVFRKYELKF	zf-BED--DUF-domain	IV
OsR498G0306283000.01.T01	OsZf-BED22_II	KRSRSHVWNDFEELYESRNGSQVRVRAKCNYCHKILFACSSGGTGHLIRHIKSCKPRNVGALSQSMLRFNADGFVSQWEYKPDVARTELVKLIAREDLPLCFGESAAFEEYIQKAHNPIFRSVSRQTTSRDVFK	zf-BED	II
OsR498G0306996300.01.T01	OsZf-BED23_I	MTEETGNDFQMVQGYEIVPSNEEAHAEEVQGDELVLAEDLAQGDEVQVNRLVSAEMSTPPTSRRRRKKSLVWEHFTIEAVSGGATRACCKLCKQTFAYSSGSKIAGTSHLKRHITLGSCPKIKNQEHKLALTPAVGTDNDGEGTVERPSKRRYRYTGYANAAFDQDRSCSYLAKMIILHDYPLHIVQQPAFTTFIDSLQPRFRVVDVETMEGEVYAVYQKEKENLMQAFSTMPGRISLTIGLWTTSQTLGYVSLAGQFIDSEWKIHRRMLNFMMVSSPHSENALSEAISTSLSDWNMKDKLFTITLDNDCSSHDIYSANLRDYLSNKNNLMLKGQLFVVRCYAHILNAVAQDVIASIHGVIYNIRESIKFIKASPSREEKFAEIALQLEIPSTKTLCLDVTTQWNTTYLMLLAALDYKQAFSTLETSDDNYNEAPSAEDWKKVEAACNYLKLLYDSAHSIMAAANPTSNLFFHEAWKLQLELSNATGHEDPVFSSIAKDMHERFDKYWKDCNLVLAIAVVMDPRFKMKLVEFSYSKIYGVEAAKYVKVVDDAVHELYKEYVAQPLPLTPAYVEQGDGNNAPASENGTQATAPSTGDGLVDFDMYLSEIATSQPTKSELEQYLDESLTPRIQEFDILNWWKLNTLKFPTLSRMARDILAIPMSMVSSGNSIFSAGTGTRMLDDYRSSLRPEIVEALVCAKDWLQYLPATPEAPSTTLVKVDAP	zf-BED--DUF-domain--Dimer_Tnp_hAT--	I
OsR498G030	OsZf-	MTEETGNDFQMVQGYEIVPSNEEAHAEEVQGDELVLAEDLAQGDEVQVNRLVSAEMSTPPTSRRRRKKSLVWEHFTIEAVSGGATRACCKLCKQTFAYSSGSKIAGTSHLKRHITLGSCPKIKNQEHKLALTPAVGTDNDGEGTVERPSKRRYRYTGYANAAFDQDRSCSYLAKMIILHDYPLHIVQQPAFTTFIDSLQPRFRVVDVETMEGEVYAVYQKEKENLMQAFSTMPGRISLTI	zf-BED--DUF-domain--	I

6996300.01.T02	BED24_I	GLWTTSQTLGYVSLAGQFIDSEWKIHRRMLNFMMVSSPHSENALSEAISTSLSDWNMKDKLFTITLDNDCSSHDIYSANLRDYLSNKNNLMLKGQLFVVRCYAHILNAVAQDVIASIHGVIYNIRESIKFIKASPSREEKFAEIALQLEIPSTKTLCLDVTTQWNTTYLMLLAALDYKQAFSTLETSDDNYNEAPSAEDWKKVEAACNYLKLLYDSAHSIMAAANPTSNLFFHEAWKLQLELSNATGHEDPVFSSIAKDMHERFDKYWKDCNLVLAIAVVMDPRFKMKLVEFSYSKIYGVEAAKYVKVVDDAVHELYKEYVAQPLPLTPAYVEQGDGNNAPASENGTQATAPSTGDGLVDFDMYLSEIATSQPTKSELEQYLDESLTPRIQEFDILNWWKLNTLKFPTLSRMARDILAIPMSMVSSGNSIFSAGTGTRMLDDYRSSLRPEIVEALVCAKDWLQYLPATPEAPSTTLVKVDAP	Dimer_Tnp_hAT--	
OsR498G0306997800.01.T01	OsZf-BED25_I	MTEETGNDFQMVQGYEIVPSNEEAHAEEVQGDELVLAEDLAQGDEVQVNRLVSAEMSTPPTSRRRRKKSLVWEHFTIEAVSGGATRACCKLCKQTFAYSSGSKIAGTSHLKRHITLGSCPKIKNQEHKLALTPAVGTDNDGEGTVERPSKRRYRYTGYANAAFDQDRSCSYLAKMIILHDYPLHIVQQPAFTTFIDSLQPRFRVVDVETMEGEVYAVYQKEKENLMQAFSTMPGRISLTIGLWTTSQTLGYVSLAGQFIDSEWKIHRRMLNFMMVSSPHSENALSEAISTSLSDWNMKDKLFTITLDNDCSSHDIYSANLRDYLSNKNNLMLKGQLFVVRCYAHILNAVAQDVIASIHGVIYNIRESIKFIKASPSREEKFAEIALQLEIPSTKTLCLDVTTQWNTTYLMLLAALDYKQAFSTLETSDDNYNEAPSAEDWKKVEAACNYLKLLYDSAHSIMAAANPTSNLFFHEAWKLQLELSNATGHEDPVFSSIAKDMHERFDKYWKDCNLVLAIAVVMDPRFKMKLVEFSYSKIYGVEAAKYVKVVDDAVHELYKEYVAQPLPLTPAYVEQGDGNNAPASENGTQATAPSTGDGLVDFDMYLSEIATSQPTKSELEQYLDESLTPRIQEFDILNWWKLNTLKFPTLSRMARDILAIPMSMVSSGNSIFSAGTGTRMLDDYRSSLRPEIVEALVCAKDWLQYLPATPEAPSTTLVKVDAP	zf-BED--DUF-domain--Dimer_Tnp_hAT--	I
OsR498G0306997800.01.T02	OsZf-BED26_I	MTEETGNDFQMVQGYEIVPSNEEAHAEEVQGDELVLAEDLAQGDEVQVNRLVSAEMSTPPTSRRRRKKSLVWEHFTIEAVSGGATRACCKLCKQTFAYSSGSKIAGTSHLKRHITLGSCPKIKNQEHKLALTPAVGTDNDGEGTVERPSKRRYRYTGYANAAFDQDRSCSYLAKMIILHDYPLHIVQQPAFTTFIDSLQPRFRVVDVETMEGEVYAVYQKEKENLMQAFSTMPGRISLTIGLWTTSQTLGYVSLAGQFIDSEWKIHRRMLNFMMVSSPHSENALSEAISTSLSDWNMKDKLFTITLDNDCSSHDIYSANLRDYLSNKNNLMLKGQLFVVRCYAHILNAVAQDVIASIHGVIYNIRESIKFIKASPSREEKFAEIALQLEIPSTKTLCLDVTTQWNTTYLMLLAALDYKQAFSTLETSDDNYNEAPSAEDWKKVEAACNYLKLLYDSAHSIMAAANPTSNLFFHEAWKLQLELSNATGHEDPVFSSIAKDMHERFDKYWKDCNLVLAIAVVMDPRFKMKLVEFSYSKIYGVEAAKYVKVVDDAVHELYKEYVAQPLPLTPAYVEQGDGNNAPASENGTQATAPSTGDGLVDFDMYLSEIATSQPTKSELEQYLDESLTPRIQEFDILNWWKLNTLKFPTLSRMARDILAIPMSMVSSGNSIFSAGTGTRMLDDYRSSLRPEIVEALVCAKDWLQYLPATPEAPSTTLVKVDAP	zf-BED--DUF-domain--Dimer_Tnp_hAT--	I
OsR498G0307295100.01.T02	OsZf-BED27_I	MASEAEAEVAAGPEVVLPIGAQKHDPAWKHCQMVRSAGRVRLKCVYCHKHFLGGGIHRFKEHLARRPGNACCCPKVPREVQETMLHSLDAVAAKKKRKQSLAEGIRRITHSAPAAAASASPPAPADAAEMESPIHMIPLNEVLDLGSVPLEETPPETREMKGSISKKRKKLAARQASTAPLAHQNQQPLQSTPAGLTQPFHQMVVAFDSAASQLRHFDQPGSNKEQVYMAIGRFLYDAGVSLEAVNSVYFQPMLEAVASAGGKPEAFSYHDFRGSILKKSLDEVTAQLEFYKGSWTRTGCTLLADEWTTDRGRTLINFSVYCPEGTMFLKSVDATDIVVSSDPLYELLKNVVEEVGEKNVVQVITNNSEIHAVAGKRLCETFPTLFWSQCSFQCIDGMLEDFSKVGAINEIICNAKVITGFIYNSAFAFNLMKRHLHGKDLLVPAETRAAMNFVTLKNMYNLKDSLEAMISSDEWIHYLLPKKPGGVEVTNLIGNLQFWSSCAAVVRITEPLVHLLKLVGSNKRPSMGYVYAGLYQAKAAIKKELVRKNDYMAYWDIIDWRWNKDAPRPLHLAGFFLNPLFFDGVRGGTSSEIFSGMLDCIERLVSDVKIQDKIQKELNVYRSEAAGDFRRQMAIRARRTLPPAEWWYTYGGACPNLTRLAVRILSQTCSAKGCDRRHISFEQIHDQRMNLFERQRMHHLTFVQYNLRLQHR	zf-BED--DUF-domain--Dimer_Tnp_hAT--	I
OsR498G0409301900.01.T01	OsZf-BED28_XII	MEEVEAGLLEGGIRWLAETILDNLDADKLDEWIRQIRLAADTEKLRAEIEKVDGVVAAVKGRAIGNRSLARSLGRLRGLLYDADDAVDELDYFRLQQQVEGGVTTRFEAEETVGDGAEDEDDIPMDNTDVPEAVAAGSSKKRSKAWEHFTTVEFTADGKDSKARCKYCHKDLCCTSKNGTSALRNHLNVCKRKRVTSTDQPVNPSSAGEGASNATGNSVGRKRMRMDGTSTHHEAVSTHPWNKAELSNRIQCMTHQLEEAVNEVMRLCRSSSSNQSRQGTPPATNATTSSYLPEPIVYGRAAEMETIKQLIMSNRSNGITVLPIVGNGGIGKTTLAQLVCKDLVIKSQFNVKIWVYVSDKFDVVKITRQILDHVSNQSHEGISNLDTLQQDLEEQMKSKKFLIVLDDVWEIRTDDWKKLLAPLR	zf-BED--NB-ARC--	XII
OsR498G0409324600.01.T01	OsZf-BED29_III	MDHDSLQNTMEHPVPGEEHEEDNSMKVDVAFDGVHSPFRYRHKKRSKVWEEYKPIFLNGKVQFAECLYCHNRLSCKDSNGTSHLWRHQKICPGKEEAAQRRQKDSYFPCVLVNENDPVSPHDPVNDVITETLSDINSVTPSGNRFTSKVWKEFTPVYIEGKLQAADCIHCQKRLSANKFGGRSHLSRHLITCAGRRGRGGGQIHQKGLFYPSSVPSLKSRVQDELSPALTNGKVQIAEYSSKLLRASSSGDSTPKPIRVVPAEHSVPTPDYTSLMKQRTSFVTTPAGQETSDPDLVGMIALHGYPLSIVEHEEMMRFVKKLNPVVNLVSRNAMEEHCLTLFQKEKENIKGKIAHFSRRVSLSASIWTPDGPETTVNYLCLTAHYIDEDWKVHRIIIKFGMFWSAPADLERMIHCMEACVPESESGSYNVILDAIRDWNLDQKLLSLTSVGEVRGDTNTSLLKEMLIEKKCLPIGGTLFNVACVDYVLNSIVFKVQADILRLVGDIVMDFLVSLTQQQLLEVISQTGLKCPQEDAKWWHKLYFRLEVLLHFKKSFPSEERVSPEDTKTAESVCKILRTIYRVIEVISSPSSPTANIYFNEIWKVRTVLQEEALNDHREIATVAMVMQEAFNEYWQNSYLWLAIPVVLDPRFKFSFIEFRLKRAFGTDSASYLSVIRETVRELFNEYCHSLNQASDVVSNSEALCADDNDSLEDWDQHLHEQASRQLSSELDDYLEDGLVPRKDDFDILNWWMTHSTKYPTLATIARDILAMPASAVQSEAAFSSSGPVIPKHQSTLNIRTIEALVCTRDWMR	zf-BED--zf-BED--DUF-domain--Dimer_Tnp_hAT--	III
OsR498G0409324600.01.T02	OsZf-BED30_III	MDHDSLQNTMEHPVPGEEHEEDNSMKVDVAFDGVHSPFRYRHKKRSKVWEEYKPIFLNGKVQFAECLYCHNRLSCKDSNGTSHLWRHQKICPGKEEAAQRRQKDSYFPCVLVNENDPVSPHDPVNDVITETLSDINSVTPSGNRFTSKVWKEFTPVYIEGKLQAADCIHCQKRLSANKFGGRSHLSRHLITCAGRRGRGGGQIHQKGLFYPSSVPSLKSRVQDELSPALTNGKVQIAEYSSKLLRASSSGDSTPKPIRVVPAEHSVPTPDYTSLMKQRTSFVTTPAGQETSDPDLVGMIALHGYPLSIVEHEEMMRFVKKLNPVVNLVSRNAMEEHCLTLFQKEKENIKGKIAHFSRRVSLSASIWTPDGPETTVNYLCLTAHYIDEDWKVHRIIIKFGMFWSAPADLERMIHCMEACVPESESGSYNVILDAIRDWNLDQKLLSLTSVGEVRGDTNTSLLKEMLIEKKCLPIGGTLFNVACVDYVLNSIVFKVQADILRLVGDIVMDFLVSLTQQQLLEVISQTGLKCPQEDAKWWHKLYFRLEVLLHFKKSFPSEERVSPEDTKTAESVCKILRTIYRVIEVISSPSSPTANIYFNEIWKVRTVLQEEALNDHREIATVAMVMQEAFNEYWQNSYLWLAIPVVLDPRFKFSFIEFRLKRAFGTDSASYLSVIRETVRELFNEYCHSLNQASDVVSNSEALCADDNDSLEDWDQHLHEQASRQLSSELDDYLEDGLVPRKDDFDILNWWMTHSTKYPTLATIARDILAMPASAVQSEAAFSSSGPVIPKHQSTLNIRTIEALVCTRDWMR	zf-BED--zf-BED--DUF-domain--Dimer_Tnp_hAT--	III
OsR498G0409324600.01.T03	OsZf-BED31_III	MDHDSLQNTMEHPVPGEEHEEDNSMKVDVAFDGVHSPFRYRHKKRSKVWEEYKPIFLNGKVQFAECLYCHNRLSCKDSNGTSHLWRHQKICPGKEEAAQRRQKDSYFPCVLVNENDPVSPHDPVNDVITETLSDINSVTPSGNRFTSKVWKEFTPVYIEGKLQAADCIHCQKRLSANKFGGRSHLSRHLITCAGRRGRGGGQIHQKGLFYPSSVPSLKSRVQDELSPALTNGKVQIAEYSSKLLRASSSGDSTPKPIRVVPAEHSVPTPDYTSLMKQRTSFVTTPAGQETSDPDLVGMIALHGYPLSIVEHEEMMRFVKKLNPVVNLVSRNAMEEHCLTLFQKEKENIKGKIAHFSRRVSLSASIWTPDGPETTVNYLCLTAHYIDEDWKVHRIIIKFGMFWSAPADLERMIHCMEACVPESESGSYNVILDAIRDWNLDQKLLSLTSVGEVRGDTNTSLLKEMLIEKKCLPIGGTLFNVACVDYVLNSIVFKVQADILRLVGDIVMDFLVSLTQQQLLEVISQTGLKCPQEDAKWWHKLYFRLEVLLHFKKSFPSEERVSPEDTKTAESVCKILRTIYRVIEVISSPSSPTANIYFNEIWKVRTVLQEEALNDHREIATVAMVMQEAFNEYWQNSYLWLAIPVVLDPRFKFSFIEFRLKRAFGTDSASYLSVIRETVRELFNEYCHSLNQASDVVSNSEALCADDNDSLEDWDQHLHEQASRQLSSELDDYLEDGLVPRKDDFDILNWWMTHSTKYPTLATIARDILAMPASAVQSEAAFSSSGPVIPKHQSTLNIRTIEALVCTRDWMR	zf-BED--zf-BED--DUF-domain--Dimer_Tnp_hAT--	III
OsR498G0409324600.01.T04	OsZf-BED32_III	MDHDSLQNTMEHPVPGEEHEEDNSMKVDVAFDGVHSPFRYRHKKRSKVWEEYKPIFLNGKVQFAECLYCHNRLSCKDSNGTSHLWRHQKICPGKEEAAQRRQKDSYFPCVLVNENDPVSPHDPVNDVITETLSDINSVTPSGNRFTSKVWKEFTPVYIEGKLQAADCIHCQKRLSANKFGGRSHLSRHLITCAGRRGRGGGQIHQKGLFYPSSVPSLKSRVQDELSPALTNGKVQIAEYSSKLLRASSSGDSTPKPIRVVPAEHSVPTPDYTSLMKQRTSFVTTPAGQETSDPDLVGMIALHGYPLSIVEHEEMMRFVKKLNPVVNLVSRNAMEEHCLTLFQKEKENIKGKIAHFSRRVSLSASIWTPDGPETTVNYLCLTAHYIDEDWKVHRIIIKFGMFWSAPADLERMIHCMEACVPESESGSYNVILDAIRDWNLDQKLLSLTSVGEVRGDTNTSLLKEMLIEKKCLPIGGTLFNVACVDYVLNSIVFKVQADILRLVGDIVMDFLVSLTQQQLLEVISQTGLKCPQEDAKWWHKLYFRLEVLLHFKKSFPSEERVSPEDTKTAESVCKILRTIYRVIEVISSPSSPTANIYFNEIWKVRTVLQEEALNDHREIATVAMVMQEAFNEYWQNSYLWLAIPVVLDPRFKFSFIEFRLKRAFGTDSASYLSVIRETVRELFNEYCHSLNQASDVVSNSEALCADDNDSLEDWDQHLHEQASRQLSSELDDYLEDGLVPRKDDFDILNWWMTHSTKYPTLATIARDILAMPASAVQSEAAFSSSGPVIPKHQSTLNIRTIEALVCTRDWMR	zf-BED--zf-BED--DUF-domain--Dimer_Tnp_hAT--	III
OsR498G0409481100.01.T01	OsZf-BED33_II	MDDDPTSVNYELRTMGMRGDDDDDVEEDRVEVFGNTSDIPIHVNVDDDDPPVDDSGNGTPTGSSATCTNKKTKTSKVWDDFEELYETTNGNRVRVSAKCNYCHKTLSARSSAGTGHLLRHIKSCKPRKLGSNALPQSMLRFSADGSVIPWEYSPEDARFELCRLIAREDLPISFGQSPAFVNYIKAAHNPRFVPVSRQTTTRDFYKLFKDRRSVIIDRLNSASSIALTSDIWSGHAKEDYLSVVAHFVNSDWQLEKRVLGLRLIDESHTGANIAERVIAVVEEYGITDKVFSITLDNASANSKAMITLTPALSGYIGDLFLHQRCACHIINLIVKAGLDKFKPMLNDIRAAISFLNASNQRIATYKNVCIAAGYRPRMFGLDMDVRWNSTYLMLKHLIPHREPFTVFISTQHPFVNDHPLLTDLHWACAESVLCFLEQFYDSTVVLSGVYYPTSPLIMHHILEIAGHLN	zf-BED	II
OsR498G05101166	OsZf-BED34_I	MAEETSNDNLVVQGNEIVPSNGEALAEEVQGDELVLAEDLIQGDEVQGNELVSAEMSIPPTSRRRRKKSLVWEHFTIEAVSGGATRACCKLCKQTFAYSSGSKIAGTSHLKRHITLGSCPKIKNQEHKLLLTPAGGTDNDGEGTVERPSKRRYRYTGYANAAFDQERSCSYLAKMIILHDYPLHIVQQPAFTTFIDSLQPRFRVVDVETMEWEVYAVYQKEKENLMQAFNTMPGRISLAIGLWTTSQTLGYVSLAGQFIDSEWKMHRRMLNFMMVSSPHSENALSEAISTSLSDWNMKDKLFTITLDNDCSSHDIYSANLR	zf-BED--DUF-domain--Dimer_Tnp_hAT--	I

00.01.T01		DYLSNKNNLMLKGQLFVVRCYAHILNAVAQDVIASIHGVIYNIRESIKFIKASPSCEEKFAEIALQLEIPSTKTLCLDVTTQWNTTYLMLLAALDYKQAFSTLETSDDNYNEAPSAEDWKKVEAACNYLKLLYDSAHSIMAAANPTSNLFFHEAWKLQLELSNATGREDPVFSSIAKDMHERFDKYWKDCNLVLAIAVVMDPRFKMKLVEFSYSKIYGVEAAKYVKVVDDAVHELYNEYVAQPLPLTPAYVEQGGGNNAPASENSTQATAPSTGDGLVDFDMYLSEIATSQPTKSELEQYLDESLTPRIQEFDILNWWKLNTLKYPTLSKMARDILAIPMSMVSSGNSIFSAGTGTRMLDDYRSSSRPEIVEALVCAKDWLQYLPATPEAPSTALVKVDAA		
OsR498G0511412700.01.T01	OsZf-BED35_II	MAEETGNDNQVVQGNEIVQSNEEAQAEEVQGDELVPAEDLTQGDEVQGNELVSAEISTPPTLRRRRKKSLVWEHFTIEAVSGGATRACCKLCKQTFAYSSGSKIAGTSHLKRHITLGSCPKIKNQEHKLALTPAGGTDNDGEGTVERPSKRRYRYTGYANAAFDQDRSCSCLAKMIILHDYPLHIVQQPAFTTFIDSLQPRFRVVDVETMEGEVYAVYQKEKENLTQAFSTMPGRISLTIGLWTTSQTLGYVSLAGQFIDSEWKIHRRMLNFMMVSSPHSENALSEAISASLSDWNMKDKLFTITLDNDCSSHDIYSANLRDYLSNKNNLMLKGQLFVVRCYAHILNAVAQDVIASIHGVIYNIRESIKFAEIALQLEIPSTKTLCLDVTTQWNTTYLMLLAALDYKQAFSTLETSDDNYNEAPSAEDWKKVEAACNYLKLLYDSAHSI	zf-BED	II
OsR498G0511413500.01.T01	OsZf-BED37_I	MCEPSGSDDAMVHASEMVDGDEMIHGNEMVVHDSVMIDGNEMVQENVMVHGSGEMVQGSEMVHNNEIIQVNDMIQVNEMVNGDKMAHGHELVGVELTTPTASRRRRKKSVVWEHFTIEEMPGGVSRASCNLCKQTFAYSCGSKISGTSHLKRHITLASCPMLKNEDMKLSLPLATVTNNDGEGCAERVAKRHYRSTGYANAMFDQDRTCSNLAKMIILHDYPLHIVEQRGFTAFIGSLQPRFRVIDVDTIEGQVHSVYQKERENLMHVFSTVPGRISLTVRLWATSQTLGYISLAAQFIDTEWRVHRRMVNFMMVSSPHSENSLSEAISTSLSDWNMKDKLFTITLDNDPSSHDIYSANMINYLSNKKDNIMIKGQLFVVRCYAHILNTVAQDVIASVHSVIYHIRESIKFIKASSVHEDKFAEIALQLEIPSAKTLCLDVTTQWNTTYLMLLAALDYQQVFASLETCDGDYNEAPSTEDWKKVEAACSYLSLLYDSAHNIMAAPNPTSNIFFHEAWKLQSELSNAIAHEDPIFRSTAKIMHERFDKYWKDCNLVLAIAVVMDPRFKMKLVEFSYSKIHSVEAAKYVKVVDDAIHELYSEYATQGEANRDAHVTDNSAAVTPPNGDELLDFDIYLSEIATSQPSISELEQYLEEALMPRIQDFEILEWWKLNTIKFPTLSKMARDVLAIPMSMVSSGSSIFSATATGSQMLDDYRSSLRPETVESLFCAKDWLQYPPATTEAPSTALVKME	zf-BED--DUF-domain--Dimer_Tnp_hAT--	I
OsR498G0611696400.01.T01	OsZf-BED38_I	MDDDPTSVNYELRTMGMRGDDDDDVEEDRVEVFGNTSDILIHVNVNDDDPPVDDSGNGTPTGSSATCTNKKTKTSKVWDDFEELYETTNGNRVRVSAKCNYCHKTLSARSSAGTGHLLRHIKSCKPRKLGSNALPQSMLRFSADGSVIPWEYSPEVARFELCRLIAREDLPISFGQSPAFVNYIKAAHNPRFVPVSRQTTTRDFYKLFKDRRSVIIDRLNSASSIALTSDIWSGHAKEDYLSVVAHFVSSYWQLEKRVLGLRLIDESHTGANIAERVIAVAEEYGITDKVFSITLDNASANSKAMDTLTPALSGYIGDLFLHQRCACHIINLIVKAGLDKFKPMLNDIRAAISFLNASNQRIATYKNVCIAAGYRPRMFGLDMDVRWNSTYLMLKHLIPHREPFTVFISTQHPFVNDHPLLTDLHWACAESVLCFLEQFYDSTVVLSGVYYPTSPLIMHHILEIAGHLNTYGNVQNLANVVGPMKTKFMNYWSKIPILYSFAFILDPRAKIRGFSKVLQIMAQLISDDYSAYLTTVRASLSDTFAKYERKFGSVRLHSSTIPGPSTGKKRTAWGKIFGSVVAAGLGAGNAGASPGAGNAGASPGAGLGAGSLSRMTSATALLQAASSTANLNPSELSAYLDSDTVNQYDNDFNILSWWQQHKLTYPVLSILAKDVMTVLVSTISSESTFSLTGRIIEDRRRRLNPRLVEILAVIKDWELADAKSQHTTENVELQNAYENMYLDDEIDVNP	zf-BED--DUF-domain--Dimer_Tnp_hAT--	I
OsR498G0611936800.01.T01	OsZf-BED39_I	MDDDPTSVNYELRTMGMRGDDNDDVEEDHVEVFGNTSDIPIHVNVDDDDPPVDDSGNGTPTGSSATCTNKKTKTSKVWDEFEELYETTNGNRVRVSAKCNYCHKTLSARSSAGTGHLLRHIKSCKPRKLGSNALPQSMLRFSADGSVIPWEYSPEVARFELCRLIAREDLPISFGQSPAFVNYIKAAHNPRFVPVSRQTTTRDFYKLFKDRRSIIIDRLNSASSIALTSDIWSGHAKEDYLSVVAHFVSSDWQLEKRVLGLRLINESHTGANIAECVIAVAEEYGITDKVFSITLDNASANSKAMDTLTPALSGYIGDLFLHQRCACHIINLIVKAGLDKFKPMLNDIRAAISFLNASNQRIATYKNVCIAAGYGPRMFGLDMDVRWNSTYLMLKHLIPHREPFTVFISTQHPFVNDHPLLTDLHWACAESVLCFLEQFYDSTVVLSGVYYPTSPLIMHHILEIAGHLNTYGNVQNLANVVGPMKTKFMNYWSKIPILYSFAFILDPRAKIRGFSKVLQIMAQLIGDDYSAYLTTVRASLSDTFAKYERKFGSVRLHSSTIPGPSTGKKRTAWGKIFGSVVAAGLGAGNAGASPGAGNAGASPGAGLGASSLSRMTSATALLQAASSTANLNPSELSAYLDSDTVNQYDDDFNILSWWQQHKLTYHVLSILAKDVMTVPVSTISSESTFSLTGRIIEDRRRRLNPRLVEILAVIKDWELADAKSQHTTENVELQNAYENMYLDDEIDVNP	zf-BED--DUF-domain--Dimer_Tnp_hAT--	I
OsR498G0612344300.01.T01	OsZf-BED40_V	MESSHASSSSASVCNAGIVALRASQPTLANVGDPTTNVVAAARVEVDLTQETTEDANVPPAKKAKKCSSEVWSHFDKYEKKVVGDDGTEIVELWAKCKKCSYTTRRESNRGTTIFWSHLDKKHQIKSGQQLLNLKKSESGTAVETYRFKRTLLEYYADKVYGVAAPKHMADFMVVINKLFDTYASSQASSKSPAATNVQNNFLVSKDYDGESDDELDADVQQYLRASTAPGIGTKSELEVYLEQPLLEWYTSDKTTFNILHWWSLKQHEFPILSFSLDLLVMFWLFKYQL	zf-BED--Dimer_Tnp_hAT--	V
OsR498G0612344300.01.T03	OsZf-BED41_V	MESSHASSSSASVCNAGIVALRASQPTLANVGDPTTNVVAAARVEVDLTQETTEDANVPPAKKAKKCSSEVWSHFDKYEKKVVGDDGTEIVELWAKCKKCSYTTRRESNRGTTIFWSHLDKKHQIKSGQQLLNLKKSESGTAVETYRFKRTLLEYYADKVYGVAAPKHMADFMVVINKLFDTYASSQASSKSPAATNVQNNFLVSKDYDGESDDELDADVQQYLRASTAPGIGTKSELEVYLEQPLLEWYTSDKTTFNILHWWSLKQHEFPILSFSLDLLVMFWLFKYQL	zf-BED--Dimer_Tnp_hAT--	V
OsR498G0613145100.01.T01	OsZf-BED42_I	MDPNFPYQSPSFTLGDFDPNYMSGFDGTSGSAPTPPSVEEVPVHTAVVEEVPVQEATASEGFSGTASGSVSTHTGSKRSRTSGVWQSFDEIKETCPDGREVSKARCRICRQILSARSSGGTGHLKRHAESCAKKQGIQLRQQQLMLNPDGTVRSWEYDPMVARESLVRLIARQDLPLNFGESPAFEHYIQQSHNPRFKAVSRQTSTRDLENVYHKEATALKELFSTCTFSVCVTSDIWSSRAREDYLSVVVHFVDDDWQLQKRVLGLRLIDVSHTGENIAERIREVINEFNLADKIFAVTLDNASANSRAIEILQPLFCVYAQSFLLHQRCACHIINLIVKTGMKRVGDHIDAVRQAIAWLTASNPRIAAWKRFCIAAGVEARKFATDAEHRWNATYLMLKVVLPYSSLLSDFVQSRGGPRNSDGSSVLNEHVWAIVQKFYQFLETFYDSTLTLSHVYYPTANIILHNLLEIATLFKEYENDDVLTEPVFHMKQKYLKYWKNIPMLYALAFVLDPRCKLRGLSAILSLVGDTIGVDYSSFYTEVRRKLYEVFGRYEVKFQEVRQRRPPPIPTTGKKKIQWGRIWGGSSSSSIQGGGSSSATSGDASSHVVAEELSGYLDSDAIHHEAQDFNVLGWWNDHKITYPVLSKLARDVLTVPVSTVSSESAFSLCGRIIEDRRTTLRSDHVEMLLSVKDWELARQHAQYT	zf-BED--DUF-domain--Dimer_Tnp_hAT--	I
OsR498G0713854400.01.T01	OsZf-BED43_II	MDESNIPSFTLGDFDPNYVSRSFPTGEYDATGSAPTPPVMEPPAGSEASGTMSGSASTNTGSKRSRTSGVWQHFDEVAMIGPDGRQVTFARCRICKNKLSAKSSGGTGHLKRHAETCAKKQGIQLRQQQLLLNPDGTVCTWEYDPMVARENLARLIARQDLPLNFGESPAFENYIKNSHNPRFQAVSRQTTTRDLKNVYDKGYESLKELFSTCTFSVSVTSDIWSSRAKEDYLSVVVHFIDDDWQMQKRVLGLRLIDVSHTGENIAERIREVIDEFNLADKIFAVTMDNASANSRAMEILQPLFCIYAQSFLLHQRCACHIINLIVKCGFKRVNVHIDAVRQAITWLTASNPRIAQWKRYCYASGEPPRKFLTDADHRWNATYFM	zf-BED	II
OsR498G0714056800.01.T01	OsZf-BED44_I	MEGENNASPHPPSLESRSKLRGRRGTSTRSSSNPTVSLEVVPLEKVSEDEDDDNSFDHRDDMSESLPIVDGGSGSQKGDDEFDDGGSKAKKAKTGGKKAGVTVSPKCKIARGKRAECWKHFKVINVPSKKERGVMVTKAKCKFCHRSYVYHPGGATTTLNRHLAGCTQYQNKLTKAKAQGTLNFGPEDDNSLIVNPTEYNHEHTRELIAKMIIAHEYSFRMVEHKWFNILMKWMNGNYEFIGRKSIKNECMRVYESEKNQLKRSLKEAESISLTTDLWTSNQNLQYMCLVAHYIDENWVMQCCVLNFIEVDPPHTGIVIAQAVFECMVEWKIEDKVTTITLDNATNNDTAVTNLKAKLLARKNSVFDPSYFHIRCAAHIVNLVVNDGLQPIDNLISCLRNTVKYFKRSPSRMYKFVEVCNNYSVKVGRGLALDVKTRWNSTYKMLDTCIDYKDAFGYYKEVDTSYVWKPSDSDWVSFGKIRPILGTMAEASTAFSGSLYPTANCFYPYIVKVKRALIEAQKSEDTYLRSMGAAMLDKFDKYWEEKNNVMVIATILDPRFKMRYIKWCFAQIFDPIRCEIEINDINQELERLYNKYEILHRQKMGENGTNRQSTSASVDTTSSMASIASDFQSFLQSTVTESSKSELLIYLDEANEAIDNKHFNLLRYWNVNCHRFPVVSSLAKRFLTVPVSSVSSECTFSAAGRVLDDYRSSLKPATVQALVCASSWIRGPYDDNNHPLLVIGDGEDDVESIEFPKCVVASN	zf-BED--DUF-domain--Dimer_Tnp_hAT--	I
OsR498G0815043800.01.T01	OsZf-BED45_IV	MRRSLSGRRQPPLRGTGVPAMDGDPTSCNYEAMVMGTQGDDEDDLEEERIEVFGNTASPLRDLSQPEPHDDGTGADGNGAPSGSSASNKRSRSEVWDDFEELFEERNGAQVRVSAKCNYCHKTLSARSTGGTGHLLRHIKSCKPRNVGALSQSMLRFNADGSVSQWEYKPDVARTELVRLIAREDLPLTFGQSAAFEEYIQNAHNPRFSVVSRQTISRDVFKVFDKRRAMLIDTLKSVSSVALTSDIWSGNAKEDYLSVVAHFVNSNWQLEKRILGLVLIDVSHNAENISERVLSVVQEYGLTNKIFSITLDNASANSKAMDSLKPALSGYIGDLYLHQRCACHIINLIVKAGLEIFKPMLQDFRTAISFINASNQHIALYKNFCIAKGIRPRKFGLDMDVRWNATYLMLKHLLPHRVIFSVFIASHHPMADGQPLLTDLHWTIAETVLLFLEQFYDSTVILSGVYYPTSPLIMHHILEIAGHLNTYENDWNLRNVVVPMKNKFLSYWSEIPFLYSFAFILDPRAKIRGFSNVLQIMGQLISGDYSAYLNEVRAALSDMYAKYESKFGAVRLQRATPSSSSGKKKTAWGKIFGAAACGSSLGAGLGAGNAGASASPGSGFGAGASPGSGFGASASPGSGLGDAG	zf-BED--DUF-domain	IV
OsR498G0815369600.01.T01	OsZf-BED46_XVI	MENNQSGQAQDGMADQRFRSWLTEQLNMQQQNAGCSSNPAPAVMQMPALPSHTDYGGIYPNPTEYDGYNWRMCGQKLVQGGCHQKFYYECSQANCGAEKSVTCSADGQIKKTVCKGSHNHPRSSERVFGDGSATLDAIPVGEILQAAGVIRPSVAMPRNEEEDGLQSGSSDSEDDDASEARAAGDDNAIRHVPAAAAQDTTAHNTIDVDVLGNSSQQRKNYHRSEKRKSKSKVWEDFTAVFSGGKVQSAECKHCKKCLSGKTSGGTSHLRRHLKICPAQFRTTRLQQEGSSSILDSSAANNRKFDQETSLELLIRGLVSNHCSYLVPSSANFR	WRKY--zf-BED--	XVI

OsR498G0815371000.01.T01	OsZf-BED47_X	MENQSGQPQYTMADQGFRPFSALMSAPSTAQQHVGSSFSTAVVQVAAAQQSHTDHGNICLADDDGYYWRMTGQSTTQGESSPTILSHYQCAQANCVVQKTVAYTADVETFYRGRHNHLRQSDRLEPMSQVGVLVEASDAAGAAAGPSVPETENGDDQSSGSSDRNEDDAGDVEMDEDAAAGDPNAMQRRKLKSKVWKEFKLVFKDGKLHTAICNHCKLRLVAETRNGTSHLRRHLKICPEKAGTSRVQKKRRSSTSQSQPDLPVSENLENGQENPSQNLENGQENPLEEFMRATVLKLCPFPAMYRASFASFLAGRNPAPNMVPQTTVEDKFISVYEKEKLKLKEKIIATPGGVFLSVNKWYSGSYETGIVCLTVHFIDEEWKINRKTIRCCLSESDGLDLNLFPHWQSEIANYEDDDKMVLKKVVRDWCLEPKLLGVTLEGSVDKKATISLEDDLTTGKNYLVAKCKLLTIPCMVDGLNDLMQYTVRREVPSMWCCYMTNTPERKLKCQEVVSQLQLDRPSFESKDWYLTFYWCEVAFHFIKYYPLSGNVYPSLDYLEASENFCKIARAIYHAMKAFYEPYNLTFNSYFHVIWRLRATLQELPSTKNIERVFKVKDMQEKFDKHWEKWYLWLSIAVVLDPRCKLAFIELRFREAFSQDAGTYISEVRAKLYELYIQYSHVNQQSNEILNQGNNGSDTQINAPLHKQRTNYTIAQAALEEFKELFEYLGGELCPQNDSFDILKWWKDNSSTYPTLAKMARDILAIPGCAVSAESAFNDDSDQRAELFNGKLGPETTEALICAQSWIIKSSGTADADNGNNITLS	WRKY--zf-BED--DUF-domain--Dimer_Tnp_hAT--	X
OsR498G0815371000.01.T02	OsZf-BED48_X	MENQSGQPQYTMADQGFRPFSALMSAPSTAQQHVGSSFSTAVVQVAAAQQSHTDHGNICLADDDGYYWRMTGQSTTQGESSPTILSHYQCAQANCVVQKTVAYTADVETFYRGRHNHLRQSDRLEPMSQVGVLVEASDAAGAAAGPSVPETENGDDQSSGSSDRNEDDAGDVEMDEDAAAGDPNAMQRRKLKSKVWKEFKLVFKDGKLHTAICNHCKLRLVAETRNGTSHLRRHLKICPEKAGTSRVQKKRRSSTSQSQPDLPVSENLENGQENPSQNLENGQENPLEEFMRATVLKLCPFPAMYRASFASFLAGRNPAPNMVPQTTVEDKFISVYEKEKLKLKEKIIATPGGVFLSVNKWYSGSYETGIVCLTVHFIDEEWKINRKTIRCCLSESDGLDLNLFPHWQSEIANYEDDDKMVLKKVVRDWCLEPKLLGVTLEGSVDKKATISLEDDLTTGKNYLVAKCKLLTIPCMVDGLNDLMQYTVRREVPSMWCCYMTNTPERKLKCQEVVSQLQLDRPSFESKDWYLTFYWCEVAFHFIKYYPLSGNVYPSLDYLEASENFCKIARAIYHAMKAFYEPYNLTFNSYFHVIWRLRATLQELPSTKNIERVFKVKDMQEKFDKHWEKWYLWLSIAVVLDPRCKLAFIELRFREAFSQDAGTYISEVRAKLYELYIQYSHVNQQSNEILNQGNNGSDTQINAPLHKQRTNYTIAQAALEEFKELFEYLGGELCPQNDSFDILKWWKDNSSTYPTLAKMARDILAIPGCAVSAESAFNDDSDQRAELFNGKLGPETTEALICAQSWIIKSSGTTFYF	WRKY--zf-BED--DUF-domain--Dimer_Tnp_hAT--	X
OsR498G0815371000.01.T03	OsZf-BED49_X	MENQSGQPQYTMADQGFRPFSALMSAPSTAQQHVGSSFSTAVVQVAAAQQSHTDHGNICLADDDGYYWRMTGQSTTQGESSPTILSHYQCAQANCVVQKTVAYTADVETFYRGRHNHLRQSDRLEPMSQVGVLVEASDAAGAAAGPSVPETENGDDQSSGSSDRNEDDAGDVEMDEDAAAGDPNAMQRRKLKSKVWKEFKLVFKDGKLHTAICNHCKLRLVAETRNGTSHLRRHLKICPEKAGTSRVQKKRRSSTSQSQPDLPVSENLENGQENPSQNLENGQENPLEEFMRATVLKLCPFPAMYRASFASFLAGRNPAPNMVPQTTVEDKFISVYEKEKLKLKEKIIATPGGVFLSVNKWYSGSYETGIVCLTVHFIDEEWKINRKTIRCCLSESDGLDLNLFPHWQSEIANYEDDDKMVLKKVVRDWCLEPKLLGVTLEGSVDKKATISLEDDLTTGKNYLVAKCKLLTIPCMVDGLNDLMQYTVRREVPSMWCCYMTNTPERKLKCQEVVSQLQLDRPSFESKDWYLTFYWCEVAFHFIKYYPLSGNVYPSLDYLEASENFCKIARAIYHAMKAFYEPYNLTFNSYFHVIWRLRATLQELPSTKNIERVFKVKDMQEKFDKHWEKWYLWLSIAVVLDPRCKLAFIELRFREAFSQDAGTYISERTNYTIAQAALEEFKELFEYLGGELCPQNDSFDILKWWKDNSSTYPTLAKMARDILAIPGCAVSAESAFNDDSDQRAELFNGKLGPETTEALICAQSWIIKSSGT	WRKY--zf-BED--DUF-domain--Dimer_Tnp_hAT--	X
OsR498G0815372200.01.T01	OsZf-BED50_XVI	MDNQSGQPQYGMADHGFRPSSASISAPSTAQQHTGSSSNTAVIQVATPPSHTDYGNIYLADDGYHWRMCGQNTIQGEPCQTIFYYQCAQANCMVQKSVARSADGQTTQTFSMGVHNHPQRSVRWLRDGSERLEPMSQVGVLVGASDAAGATVGPSVPETGNGNDQTSGSSDSDEDNDSDVGIDGDAAAAAAAAAADANALQRHVAAPIQGNIARTASEVGILDHRARHENSHPRKKRRFKSKVWEEFKAVLIDGKVQSAICNHCKNCLVGETTKGTSHLRRHLKSCPAKAGTSRVQKKQRISTLQPDSSVSKDLKYGQVNPLEEFMRAIVSKLCPFSAMYGASFAPFLAGRNPVLNMVPQATLEEKFLSVYEKEKQKLKEKIIATPGGVFLSLDSWYWDADVELYIICITVHFIDGDWKINRKTIRCSPKLVGSYMLGLLPRLKSNIVNVKMVLKEVVQDWGLQEKLLGVTLQPSEDSIKEASLHLEDDLTGRKYLVAKCKLLNIPCM	WRKY--zf-BED--	XVI
OsR498G0815372600.01.T01	OsZf-BED51_X	MENQSGQPQYAMADQGFHPFSPFMLAPSTTMQQHVGSSSSTPVIQVAALPSHAYYGNIDVADDGFHWRMCGQSTIQGGLCPTVFSYQCALPNCGVRKSITRSADGQTIETVCKGCHNHPRQSLRWLGDGSERLEPISQEIVLLEASDASGAAGGPSVPGTGNGHGQSSGSSDSCRDDDGDLGIDGNASVGDANAVKSGQVPAPAKEITVHSACEVDILNNIVRHENPQPRKKVRSKSTVWEEFEVVLIDGKVQTAECKHCKKGLSAKTSGGTSHLIRHLKICPAQHGTSRVQKKCSSLADLPIVKSWKDDQESSLDEIIRSIVSNLCPFSAMYSASFAQFLAGRNPVLNMVQQATVEEKFLSVFHNEKMKLKEKITATPGGVFLSLGEWQRLFYIQVRVACLTVHFIDEDWKINRKTIRCSLSVFGKSDILSLYPHWQSDIVLAEKVLKEVVQDWGLLDKLLGVTLQRSVDKKAPLHLEDDITGRNYLLSKCRLLSIPCMVDALNELMDSTVLDMESTWSHYMTSSPERKQKYQEILSQLHLDRPSLGSKGWYFTFYFSEAALQFIKSFPLPDAEPNCQSGPWEPSFDDLEATENYCKIARSAYRVIKVVSGPHNMTFNSYFHVIWSLRAAIQELPSIKNIGRVFDVAYMQKKFDRNWKKWYLWLSIAVVLDPRYKLGFIELCFRQAFSHVAGMYFSEVRAKLHELYIQYSYVNEQSKEILDHKNNCSDIQISAPLHNKGQNSTTAQAAVEEFKELYEYLGGGLCTQDDSFDILKWWRGNSSAYPTLAMMARDILAIPGCAVSTESAFDQCDQRAELFDGKLRPETTEALICAQSWIKSSADADDGNKNTSF	WRKY--zf-BED--DUF-domain--Dimer_Tnp_hAT--	X
OsR498G0815372600.01.T02	OsZf-BED52_X	MENQSGQPQYAMADQGFHPFSPFMLAPSTTMQQHVGSSSSTPVIQVAALPSHAYYGNIDVADDGFHWRMCGQSTIQGGLCPTVFSYQCALPNCGVRKSITRSADGQTIETVCKGCHNHPRQSLRWLGDGSERLEPISQEIVLLEASDASGAAGGPSVPGTGNGHGQSSGSSDSCRDDDGDLGIDGNASVGDANAVKSGQVPAPAKEITVHSACEVDILNNIVRHENPQPRKKVRSKSTVWEEFEVVLIDGKVQTAECKHCKKGLSAKTSGGTSHLIRHLKICPAQHGTSRVQKKCSSLADLPIVKSWKDDQESSLDEIIRSIVSNLCPFSAMYSASFAQFLAGRNPVLNMVQQATVEEKFLSVFHNEKMKLKEKITATPGGVFLSLGEWQRLFYIQVRVACLTVHFIDEDWKINRKTIRCSLSVFGKSDILSLYPHWQSDIVLAEKVLKEVVQDWGLLDKLLGVTLQRSVDKKAPLHLEDDITGRNYLLSKCRLLSIPCMVDALNELMDSTVLDMESTWSHYMTSSPERKQKYQEILSQLHLDRPSLGSKGWYFTFYFSEAALQFIKSFPLPDAEPNCQSGPWEPSFDDLEATENYCKIARSAYRVIKVVSGPHNMTFNSYFHVIWSLRAAIQELPSIKNIGRVFDVAYMQKKFDRNWKKWYLWLSIAVVLDPRYKLGFIELCFRQAFSHVAGMYFSEVRAKLHELYIQYSYVNEQSKEILDHKNNCSDIQISAPLHNKGQNSTTAQAAVEEFKELYEYLGGGLCTQDDSFDILKWWRGNSSAYPTLAMMARDILAIPGCAVSTESAFDQCDQRAELFDGKLRPETTEALICAQSWIKSSGTADADDGNKNTSF	WRKY--zf-BED--DUF-domain--Dimer_Tnp_hAT--	X
OsR498G0815372600.01.T03	OsZf-BED53_X	MENQSGQPQYAMADQGFHPFSPFMLAPSTTMQQHVGSSSSTPVIQVAALPSHAYYGNIDVADDGFHWRMCGQSTIQGGLCPTVFSYQCALPNCGVRKSITRSADGQTIETVCKGCHNHPRQSLRWLGDGSERLEPISQEIVLLEASDASGAAGGPSVPGTGNGHGQSSGSSDSCRDDDGDLGIDGNASVGDANAVKSGQVPAPAKEITVHSACEVDILNNIVRHENPQPRKKVRSKSTVWEEFEVVLIDGKVQTAECKHCKKGLSAKTSGGTSHLIRHLKICPAQHGTSRVQKKCSSLADLPIVKSWKDDQESSLDEIIRSIVSNLCPFSAMYSASFAQFLAGRNPVLNMVQQATVEEKFLSVFHNEKMKLKEKITATPGGVFLSLGEWQRLFYIQVRVACLTVHFIDEDWKINRKTIRCSLSVFGKSDILSLYPHWQSDIVLAEKVLKEVVQDWGLLDKLLGVTLQRSVDKKAPLHLEDDITGRNYLLSKCRLLSIPCMVDALNELMDSTVLDMESTWSHYMTSSPERKQKYQEILSQLHLDRPSLGSKGWYFTFYFSEAALQFIKSFPLPDAEPNCQSGPWEPSFDDLEATENYCKIARSAYRVIKVVSGPHNMTFNSYFHVIWSLRAAIQELPSIKNIGRVFDVAYMQKKFDRNWKKWYLWLSIAVVLDPRYKLGFIELCFRQAFSHVAGMYFSEVRAKLHELYIQYSYVNEQSKEILDHKNNCSDIQISAPLHNKGQNSTTAQAAVEEFKELYEYLGGGLCTQDDSFDILKWWRGNSSAYPTLAMMARDILAIPGCAVSTESAFDQCDQRAELFDGKLRPETTEALICAQSWIKSSADADDGNKNTSF	WRKY--zf-BED--DUF-domain--Dimer_Tnp_hAT--	X
OsR498G0815467500.01.T01	OsZf-BED54_II	MDQFRSAGVQGKLAGDGTIRRPAQRRRKFSSDVWVNSSSPFEYIFDDITGEEKAMCINCGLCMSAKSKNGTSHLRRHLETDGCKKKRQQGPISPAADSAAGPSPAGDGDQQQEAVDEDDDDAFVASICACYDKLLADDLVDVVKRNDVQQMPPVPSLTMTRFFGKRRERVAARSSSSQDEKSVDMDVVSSG	zf-BED	II
OsR498G0917323400.01.T01	OsZf-BED55_II	MDQDCDGANHGGTEEERALNGMADKLENADDMVEQEESSGSAPSPLFLGTRPKRLRSKVWDDFTPIYIDGKLARAECMHCHQVFNSNSTNGTSRLLKHQAKCSPHPQKRPMQQKLPFPPSSQKSLMEPSSDPTQKKLPFLPISQKRCSGTDDAMPHRKDPALPNTLNDINRRSQEIGKSLARKKLATREQKNPTSPDITNNDQKDQWDNEHPVLKQKCTPAGTNLKNPEVDQNGLIQTLAMCGYLPLMMHNDSFRKCVPCFDSMGKMPANTNIGGGFLQLFDKEKAKVKEKFSALSSRVCLSAHVWHYDPFLAFLCLSVHYIDDEWERQQKIITFRETRKVARVNTRACVVH	zf-BED	II
OsR498G0917324000.01.T01	OsZf-BED56_II	MDDQECDCPSGVGKQGDKELNGMKNEVDDANDMVEQEESSGSAPSPLFLGTRPKRLQSKVWDDFTPIFIDGKVARAECMHCHRVFNSGTSNLLKHQAKCSPQAQKRPMQQKLPISLSIENKSAEEHGAPVQDTPTDTNTK	zf-BED	II
OsR498G09173248	OsZf-BED	MGDNVDNANDMAEQEESSGSTPSPLFLGTRPKRLRSKAWDDFTPIYIDGKVAKAECMHCHQVFVSNSTSGTSSLLKHQSKCNPHAQKRAMQQKLPFLPSSQKNLTTLNSDPRQKKLLFLPISQKKCSDTA	zf-BED	II

00.01.<br>T01	57_I<br>I			
OsR49<br>8G091<br>77536<br>00.01.<br>T01	OsZ<br>f-<br>BED<br>58_I	MTSGFSPDSAQSPPGFPLSELAEQICRLESGEQKEEEAADADADAVGLYHLMVNERTEAVGGSDTIMASPPASASRPLDPAWVHAKVIGNSKNALACLHCGKKIGGGGITRFKYHLAGITGQVQACKKVPNDVRRQMKQLVNEHRSRHDTRDGHYSGFEEDSSDAVLSNGSSGVQLRPSRKRRTGCVSPQAAAGFERTGYVSPQAAAGFERIGYISPLAAAGCERTAYVSPQAAAGFEPSNDALLVSRDLVQHSMDGVEVPNDLLHNARVAMARWWYDANIPFSAAKSPFYQPMLDAIASAGAGLKGPLYHDLRGPLLKHLTDDIREYLHDMKKEWNACGCSLIADRKKNLGESSIINFFVYCRRGTMFLKSVDTSAEKANLLEIFDQVVREVGPENIVQFITDLDPRYKTTVKVLEERYKTFVWSPCAARCIDLMLENLADPRYFPMIDETLNKAKKITQFIYNHAWVLSLMRKEFTGGRDLCRPAISRFATHFLSLQCILKFEKELCQMVTSNKWVKSTYAKGGVGKEVAAMILNAHFWAQCKHVVKVTEPLLRVLRLVDSNEKPSMGYLYEAMEKAKELIRARMMHKVSLYGPYVRVIDARMEKQLNSPLQAAGLFFNPGIFFSPTFKMQSYAHRGLVKTISCLVPDDDIQDKIFLQLEEYKKGTGDFGLPIAIRQREKLDPVAWWDNFGNGTLELQGLAKRVLGQCCSATGCERNWDIFHHIHSRKISRLERSRLSDVVFLQYNQKLRERNLHKHRDAIDPISIDNIDVLDEWVSEEPSLLCRDDLNWERIDAPFAEPTSEDEEFVAIDDEEAPTASLSWPAAAAEDSYCPPPDQDPYQYVTQEDGILPF	zf-BED--DUF-domain--Dimer_Tnp_hAT--	I
OsR49<br>8G101<br>82914<br>00.01.<br>T01	OsZ<br>f-<br>BED<br>59_I<br>I	MGTVKAKCMHCSKKLSGATKNGTKHLQYHLKICVQKKIKLKGKTLTQSALRFSSANSKNLTVENYTFEQNVARRELCNMIVLHEYPSSIVDHVGFRRYVAALQSLFKIGTRNTIRVEIMAQYEFERKKDIQYMAGIQSRVAITTDLWTSNNQKRGYMAITTHFVDESWKLRSIIMRFIYVHVPHTADVIYEQLYEALVEWNLDEKISTVTFDNCTTNDAVIPMLVRSIGKRKLLNDGKLLYMRCAAHILNLIGKDEFVKELKAAIENIRESVAYWTATPKRIEKFEKIAKYAKVTIDKKIALDCKTRWNSTFKMLSLAVPYKAVFMRASRVDKQYETLPTEEEWNFAEDVVERLRL	zf-BED	II
PAB00<br>00047<br>2.1	Pab<br>Zf-<br>BED<br>01_I<br>I	MAPKEKRDPAWLHCQLIDGSMVCNYCKKGVGGGGIHRIKQHLANARGNIKPCLKVSDELKAEMMGLLEGFQADKAKKKKVKKEVGTSSGGTRFDSDPQDRMPSFEESSTFPIPGRDPYTNAREEVEHVGGSGAGASGSKRPRGNLDSFFYPALPLVLSPLLMPNGRR	zf-BED	II
PAB00<br>00478<br>0.1	Pab<br>Zf-<br>BED<br>02_I<br>I	MAPKEKKDPAWLHCQVIDGALVCNYCQKGVGGGGIHRIKQHLAHARGNVKPCKKVSDELKAEMMGHIENYQAEKAKTKKLKKEVGGSSSGTQTRTRFESDPQDRMPSFEESSVFPTHGYSYDPYTNPREVEHVGGSGAGASGSKRPRSNLDTFFFTPHHPWFSTHH	zf-BED	II
PAB00<br>01919<br>9.1	Pab<br>Zf-<br>BED<br>03_I<br>I	MAPKEKKDPAWLHCQLIDGSMVCNYCKKGVGGGGIHRIKQHLAHARGNVKPCLEVPDELKAEMMGLLEGYQADKAKNKKLQKEVGRSSGGTRTRFDSDPQDRMP	zf-BED	II
PAB00<br>02493<br>7.1	Pab<br>Zf-<br>BED<br>04_I<br>I	MAPKEKKDPAWLHCQLIDGVMVCNYCQKEVGGGGIHRIKQHLANARGNIKPCLKVSDEVKYEMLGLLEGFQAYKAKNKKVRKEVGRSSGGTRFDSDPQDRIPSFDESFAFPIPERDSYTNPREEVEHVSGSGAGASGSKRPRGYHR	zf-BED	II
PAB00<br>02774<br>5.1	Pab<br>Zf-<br>BED<br>05_I<br>I	MAPKTKDVTWVHVEVIEGNMYCNYCQKCIRGGGGGIRRLKEHLAGVKGQVKSCEVPLDVIGHIREEMQKVLNDYQVRKAREKAIQDEIGRKRATNPTFDYEDSPSLHSTGVRHRDPFHYIPPPIDSENTINLPKSKRRNNIQSYFTPPPTSGSDNANVSQVQPTQC	zf-BED	II
PAB00<br>02941<br>2.1	Pab<br>Zf-<br>BED<br>06_I<br>I	MAPKTKDAAWVHAEVIEGSMYCKYCQKCIRGGGIRRLKEHLASVKGQVKSCEAPLDVIRPIREEMQKVLNDYQVRKAREKAIQDEIGRKRAANPTFDYEDSPSLHSTGVRHRDPFHYIPPPIDSENTINPPKSKRRNSIQSYFTPPPTSGSNNANVSQIQPTQSQPTLDDHWKKQYREIAYEYIARWWYHAD	zf-BED	II
PAB00<br>03241<br>4.1	Pab<br>Zf-<br>BED<br>07_I<br>I	MAPKDKKDLAWLHCQLIDGSMVCNYCKKEVGRGGIHRIKQHLANARGNIKPCLEVPDELKAEMMGFIECYQENKAKNRKVQKEVGRNSGGTRTRFDSDPQDRMPSFEESSAFPIPGRDPYTNPREEVEHFGGSGVGASGSKRPRGHGSGQGGNTGSLSR	zf-BED	II
PAB00<br>06177<br>3.1	Pab<br>Zf-<br>BED<br>08_I<br>I	MAPKTKYVAWVHAEVIEGSMYCKYCQKCIGGGGGGIHTLKEHLVGVKGQVKSCEAPLDVIGPIREEMQKVLNDYQVHKAREKAIQDEIGRKRAANPTFDYKDSPSLHSTSVRHRDPFHYIPPPIDSENTINPPKSKRRNSIQSYFTPPPTX	zf-BED	II
PGSC<br>0003D<br>MT400<br>00206<br>5	Pgs<br>Zf-<br>BED<br>01_I<br>I	MKEWLLGTIRGGKAPQLQEIRTDFEAAPASKKSVRRGRPLDAAWDYATPVDAKRQRAVCKYCGFISSSGGITHLKAHLAGGDPKGPSKGCPNVPPEVKRAMAESLNRTVKGAKSMQPDEIRRYMKAENDWSPPRSDDYSLNQHRIVKNAQYSHFANGGNSVNDMTMLKQSETAHVDSYMEVMSSHNACKSSFLSKPSTRVGFI	zf-BED	II
PGSC<br>0003D<br>MT400<br>00754<br>1	Pgs<br>Zf-<br>BED<br>02_I	MRLCWEIVLENEMIHEHSHEIVPVNEMIHEHSHEIVPEKEMIHEHSHEIVPENEMIHEHSHETVPQNEMIHEHSHEMVPENEMTHEHSHAMVRENEMTHEHSHEMVRENEMTHEHNHEMVRENEMTHEHNHEMVPEHEMMHEHSHEMVPEHEMMHEHRHEMMPEHEMMHEHHMVLGNEIVPSNEMVPDDEMIPLNEMVLAEPQPNYIETPPNNPETQPSKRRKKKSIVWEHFTIENVGSGTRRAQCKQCKQSFAYSTGSKVAGTSHLKRHIAKGTCPVVLRNQQNNQLSPYSTPPKMSGYGGSTDAPKRRYRTASAPYLAFDPDRCRQEISKMIIMHDYPLHMVEHPGFLAFVQNLQPRFDMVSFNTVQGDCVATYLREKQAIQKVIEGVPGRICLTLDMWSSCYTVGYVFITGQYIDSEWKIHRKILNIIMEPYPDSETAFSHAVAACLSDWSMEGKLFSVTINQPLGDAAVDNLRALLSVKNPLVLNGQLLVGSCLARTLSSIAQGAFKLLQETVKKVRDSVKYVKTSEFHEEKFIELKQQLQVPSTKTLALDDQTQWNTTYEMLLAASELKEVFSCLDTSDPDYKDALTMEDWKQVEVLCTYLKILFDTANLLTAPTIPTTNTFFHEAWKIQLELARAAVSEDPSISSLTKTMQEEFDKYWKSCCLILAIAVVMDPRFKMKLVEFSFTKIYGEEAATYVKFVEEGIHELFLEYVALPLPLTPAYAEEVNDGTLKQENGGGGLTDFDAYIMETTSQQSRSELDQYLDESLLPRVHEFDVVGWWKLNRMKYPTLSKMARDILSVPVSTVTADSVFSTIGKEMDRYRCSLRPETVEALICAKDWLQNASVNTLHAPIKMEVPI	zf-BED--DUF-domain--Dimer_Tnp_hAT--	I
PGSC<br>0003D<br>MT400<br>01137<br>4	Pgs<br>Zf-<br>BED<br>03_I	MTRDKIDIHQHGVPVDQKKLKVKCNYCGKVVSGFSRLKQHLGGIRGDVTPCLETPILVKEALEAEILNKKNGNLIKEVGQLQHPNLPLKRNWCPRDGEPNKTSESVNKKHNGVNSKVAGTSVVDSSSQEISKSIGRFFYEAGIDLDAIRLPSFQRMVKATLSPGKTVKFPSCQELRGWILQDAVKEMQQYVMEIRNSWASTGCSILLDGWIDSNGRNLINILVYCPRGTIYLRSSDISSFNGNVDAMLLFFEEVLEEVGVETVVQIVAYSTSACMMEVGKKLMEKCKTVFWTVDASHCMELMLQNFTKIDPIQEALEKAKTLTQFIYSHATALKLLRDACPDELVKSSKIRSIVPFLTLENIVSQKDCLIRMFQSSDWRTSIMASTNEGKRISNMVKDESFWSEALMAVKATIPLVEVMKLLDGTNKPQVGFIYDTLDQAKETIKKEFQDKKSLYAKFWIAIDDIWDEYLHSHLHAAGYFLNPTLFYSSDFYTDVEVSCGLCCCVVRMAEDRHIQDLITLQIDEYRMGRGTFHFGSFKDKLSNISPALWWSQYGGQCPELQRLAVRILSQTCNGASHYRLKRSLVETLLTEGMNPIEKQRLQDLVFVHCNLQLQAFDPDGSNDDTDNVVDPMDEWIVGKGPNLVSENTELTWMDLELGSRNGKGKYCVEGPIIHVKKEEEDNC	zf-BED--DUF-domain--Dimer_Tnp_hAT--	I

PGSC0003DMT400011375	Pgs Zf-BED04_I	MTRDKIDIHQHGVPVDQKKLKVKCNYCGKVVSGFSRLKQHLGGIRGDVTPCLETPILVKEALEAEILNKKNGNLIKEVGQLQHPNLPLKRNWCPRDGEPNKTSESVNKKHNGVNSKVAGTSVVDSSSQEISKSIGRFFYEAGIDLDAIRLPSFQRMVKATLSPGKTVKFPSCQELRGWILQDAVKEMQQYVMEIRNSWASTGCSILLDGWIDSNGRNLINILVYCPRGTIYLRSSDISSFNGNVDAMLLFFEEVLEEVGVETVVQIVAYSTSACMMEVGKKLMEKCKTVFWTVDASHCMELMLQNFTKIDPIQEALEKAKTLTQFIYSHATALKLLRDACPDELVKSSKIRSIVPFLTLENIVSQKDCLIRMFQSSDWRTSIMASTNEGKRISNMVKDESFWSEALMAVKATIPLVEVMKLLDGTNKPQVGFIYDTLDQAKETIKKEFQDKKSLYAKFWIAIDDIWDEYLHSHLHAAGYFLNPTLFYSSDFYTDVEVSCGLCCCVVRMAEDRHIQDLITLQIDEYRMGRGTFHFGSFKDKLSNISPALWWSQYGVQFPELQRLAVRILSQTCNGASHYRLKRSLVETLHTEGMNPIEKQRLQDLVFVHCNLQLQAFDPDGSNDNTDYVDPMDEWIVGKEPNLVPENTQLTWMDLELASRNGKEKACVERAVIYVKEEAEEEEERGR	zf-BED--DUF-domain--Dimer_Tnp_hAT--	I
PGSC0003DMT400014322	Pgs Zf-BED05_I	MESKSLAVVEAPSTILNVEPIDIGPGSSEKDPTTKLRKKALTSVYLKHFETAPDGKTRKCKFCGQSYSIATATGNLGKHLSNRHPGYDITVNVASPAPQSVTVVKKLQPKPHVKGPQLELDHLNWLLVKWLILASLPPSTLDEHWLLNSFKFLNPTVKLWPEDKFQSVLCEVFRSMQEDVRVLVDQISSKVSITLDFWTSYEQLLYMSVTCQWIDENWSFQKLLLDICHISSPCGAAEVSHALLKVLKIYNIENRVLCCTHDNTPIALHACHTLKEDMDSQKMSPFYYLPCAAHTLNSVINDGLSSTKSIISKIREFVLKMNTSFEISQDFLQCCNAYQEGTWKFPLDASPRWSGNYQMLDIARKAGKSLETIVRKYDELLGSRVLLNNVEKNAVNIMHAFLEPFYKTINDICTNKVLTIGLVLFFMDHISETIAACRDSRHSPDWLKSAADEMATKARSYNDQMCNSFTYMTAILDPRIKVELIPESLNSENHLEEARSHFIRNYSTSHFPSISGSYAAHELEDGGSVSFAEEIARKKRKASMSSATDELTQYLSEPPAPIQTDVLEWWKVTNARYPRLSSMARDFLAAQPTALAPEDLFCSKGDEIDKQRFLTPYESTQALHCVKSWMQSGFKLKYKSTEIDYERLMELAAESSMAGSDKKQKS	zf-BED--DUF-domain--Dimer_Tnp_hAT--	I
PGSC0003DMT400032498	Pgs Zf-BED06_I	MVRGRDACWEHCVLVDATRQKVRCNYCRREFSGGVYRMKFHLAQIKNKDIVPCGQVPNEVRDHIKSILNNPKKQKNPKKAKLDQAANGQESSSSSASGGIRPPHDGFSGQNGSPCPPSIMFARCSPSSQPAVDDVQKQKQDNTDKKIAEFFYHNAIPFSVAKSFYYQEMVDAILECEAGYKAPCTEELGTKLLEKVKVDIDNGYKRLRDEWKETGCTILCDCWSDRSAKCLVVFSVTCSKGTMFLRSVDISDHADDPHYLFGLLESVVLEIGVKNVIQVMTDSSASYIYAGRLVMKKYPSVFWSPCASHCINKMLEDFSEHDWVNVVLKEANMITKYIYSNDWILDLMRKFSGGREFVLVRPRITNFVAMFLSLRALVVQEDNLKHMFSHAEWLSSIYSRHPEVQAIKSLLCLERFWKSAREAVMVSEPLLKLLRIVDGDMPAMAYMYEGVERAKLSIKAFYKDVDEKFVPIWDIIDSRWSTLLQSPLHAAAAFLNPSIFYNSSFKIDARIRNGFQEAMTKMAYEDKDKVEITKEHPMYMNAQGALGTEFAIKGRTLNAPADWWTGYGYEIPTLQRAAIRILSQPCSLHWCRWNWSTFDGVHEKRRERLELERFNDLVYVHCNLWLRAIIRSKDGKWKPINFDEIDVGAEWHTEAEVAPCTYLDDSWLQLAHFTP	zf-BED--DUF-domain--Dimer_Tnp_hAT--	I
PGSC0003DMT400032499	Pgs Zf-BED07_I	MVRGRDACWEHCVLVDATRQKVRCNYCRREFSGGVYRMKFHLAQIKNKDIVPCGQVPNEVRDHIKSILNNPKKQKNPKKAKLDQAANGQESSSSSASGGIRPPHDGFSGQNGSPCPPSIMFARCSPSSQPAVDDVQKQKQDNTDKKIAEFFYHNAIPFSVAKSFYYQEMVDAILECEAGYKAPCTEELGTKLLEKVKVDIDNGYKRLRDEWKETGCTILCDCWSDRSAKCLVVFSVTCSKGTMFLRSVDISDHADDPHYLFGLLESVVLEIGVKNVIQVMTDSSASYIYAGRLVMKKYPSVFWSPCASHCINKMLEDFSEHDWVNVVLKEANMITKYIYSNDWILDLMRKFSGGREFVLVRPRITNFVAMFLSLRALVVQEDNLKHMFSHAEWLSSIYSRHPEVQAIKSLLCLERFWKSAREAVMVSEPLLKLLRIVDGDMPAMAYMYEGVERAKLSIKAFYKDVDEKFVPIWDIIDSRWSTLLQSPLHAAAAFLNPSIFYNSSFKIDARIRNGFQEAMTKMAYEDKDKVEITKEHPMYMNAQGALGTEFAIKGRTLNAPADWWTGYGYEIPTLQRAAIRILSQPCSLHWCRWNWSTFDGVHEKRRERLELERFNDLVYVHCNLWLRAIIRSKDGKWKPINFDEIDVGAEWHTEAEVAPCTYLDDSWLQLAHFTP	zf-BED--DUF-domain--Dimer_Tnp_hAT--	I
PGSC0003DMT400032500	Pgs Zf-BED08_I	MVRGRDACWEHCVLVDATRQKVRCNYCRREFSGGVYRMKFHLAQIKNKDIVPCGQVPNEVRDHIKSILNNPKKQKNPKKAKLDQAANGQESSSSSASGGIRPPHDGFSGQNGSPCPPSIMFARCSPSSQPAVDDVQKQKQDNTDKKIAEFFYHNAIPFSVAKSFYYQEMVDAILECEAGYKAPCTEELGTKLLEKVKVDIDNGYKRLRDEWKETGCTILCDCWSDRSAKCLVVFSVTCSKGTMFLRSVDISDHADDPHYLFGLLESVVLEIGVKNVIQVMTDSSASYIYAGRLVMKKYPSVFWSPCASHCINKMLEDFSEHDWVNVVLKEANMITKYIYSNDWILDLMRKFSGGREFVLVRPRITNFVAMFLSLRALVVQEDNLKHMFSHAEWLSSIYSRHPEVQAIKSLLCLERFWKSAREAVMVSEPLLKLLRIVDGDMPAMAYMYEGVERAKLSIKAFYKDVDEKFVPIWDIIDSRWSTLLQSPLHAAAAFLNPSIFYNSSFKIDARIRNGFQEAMTKMAYEDKDKVEITKEHPMYMNAQGALGTEFAIKGRTLNAPADWWTGYGYEIPTLQRAAIRILSQPCSLHWCRWNWSTFDGVHEKRRERLELERFNDLVYVHCNLWLRAIIRSKDGKWKPINFDEIDVGAEWHTEAEVAPCTYLDDSWLQLAHFTP	zf-BED--DUF-domain--Dimer_Tnp_hAT--	I
PGSC0003DMT400032501	Pgs Zf-BED09_IV	MVRGRDACWEHCVLVDATRQKVRCNYCRREFSGGVYRMKFHLAQIKNKDIVPCGQVPNEVRDHIKSILNNPKKQKNPKKAKLDQAANGQESSSSSASGGIRPPHDGFSGQNGSPCPPSIMFARCSPSSQPAVDDVQKQKQDNTDKKIAEFFYHNAIPFSVAKSFYYQEMVDAILECEAGYKAPCTEELGTKLLEKVKVDIDNGYKRLRDEWKETGCTILCDCWSDRSAKCLVVFSVTCSKGTMFLRSVDISDHADDPHYLFGLLESVVLEIGVKNVIQVMTDSSASYIYAGRLVMKKYPSVFWSPCASHCINKMLEDFSEHDWVNVVLKEANMITKYIYSNDWILDLMRKFSGGREFVLVRPRITNFVAMFLSLRALVVQEDNLKHMFSHAEWLSSIYSRHPEVQAIKSLLCLERFWKSAREAVMVSEPLLKLLRIVDGDMPAMAYMYEGVERAKLSIKAFYKDVDEKFVPIWDIIDSRWSTLLQSPLHAAAAFLNPSIFYNSSFKIDARIRNGFQEAMTKMAYEDKDKVEITKEHPMYMNAQGALGTEFAIKGRTLNAPGTINSSTPEVHLHIVSML	zf-BED--DUF-domain	IV
PGSC0003DMT400042322	Pgs Zf-BED10_I	MTENIITDEVVVLVDESINSNATRQTKSVQLKVKKERKKRSRAWNHFRSFVDEERNKKSMCKHCGADYFADSGKNGTTSMLTHMLTCPKMPRIVDKNQTQIGFKTAQGGDTDDVVVVSWKFEQEQCRRALCRMVIVDELPFKFVEKEGFRNFMKVTQPHFKIPSRTTVTRDCFKLFDAEKQKLKRSFGEAQQRVSLTTDTWTSLQRINYMCITAHWIDKEWMMHKRIINFCQVSSHRGEDMANEISKCLRDWGLDKNFTITVDNASSNDVTVKELSKIFTKRGTNFMNGEHLHVRCMAHILNLVVQDGLKVSAASIERVRKAVKYIRLSPARCKRFHECAEDVDINCKKSLCLDVSTRWNSTYLMLNRAIEFENVFSSYVDRDIGLLHYLQFVEDEDGTAAGALSSDDWNNVKKVADFLQIFYDLTREVSGSQYVTSNLHFLKICEVSCYLKKLIVSEDDTDDLLGKIAKNMREKFDKYWGTPNKMNKMIFISCVLDPRHKFVSVGFALQMMFGKEKGLVLEHEVRGYMDLMFGEYVKSLSKDKDSHHSSFLSSSSFEKFSSLPSSSDSTVQSIGSLGSFMVDLMKHKAGNATIVKTELQKYLGEENEVETKNFNILSWWKINSPRFPILAEMARNVLAISISSVASECAFSTGGRILDSFRSSLTPKLVQTLVCLQDWIRSESRHVSVEEDIDVLEQDLANTSILDD	zf-BED--DUF-domain--Dimer_Tnp_hAT--	I
PGSC0003DMT400042323	Pgs Zf-BED11_I	MTENIITDEVVVLVDESINSNATRQTKSVQLKVKKERKKRSRAWNHFRSFVDEERNKKSMCKHCGADYFADSGKNGTTSMLTHMLTCPKMPRIVDKNQTQIGFKTAQGGDTDDVVVVSWKFEQEQCRRALCRMVIVDELPFKFVEKEGFRNFMKVTQPHFKIPSRTTVTRDCFKLFDAEKQKLKRSFGEAQQRVSLTTDTWTSLQRINYMCITAHWIDKEWMMHKRIINFCQVSSHRGEDMANEISKCLRDWGLDKNFTITVDNASSNDVTVKELSKIFTKRGTNFMNGEHLHVRCMAHILNLVVQDGLKVSAASIERVRKAVKYIRLSPARCKRFHECAEDVDINCKKSLCLDVSTRWNSTYLMLNRAIEFENVFSSYVDRDIGLLHYLQFVEDEDGTAAGALSSDDWNNVKKVADFLQIFYDLTREVSGSQYVTSNLHFLKICEVSCYLKKLIVSEDDTDDLLGKIAKNMREKFDKYWGTPNKMNKMIFISCVLDPRHKFVSVGFALQMMFGKEKGLVLEHEVRGYMDLMFGEYVKSLSKDKDSHHSSFLSSSSFEKFSSLPSSSDSTVQSIGSLGSFMVDLMKHKAGNATIVKTELQKYLGEENEVETKNFNILSWWKINSPRFPILAEMARNVLAISISSVASECAFSTGGRILDSFRSSLTPKLVQTLVCLQDWIRSESRHVSVEEDIDVLEQDLANTSILDD	zf-BED--DUF-domain--Dimer_Tnp_hAT--	I
PGSC0003DMT400042324	Pgs Zf-BED12_I	MTENIITDEVVVLVDESINSNATRQTKSVQLKVKKERKKRSRAWNHFRSFVDEERNKKSMCKHCGADYFADSGKNGTTSMLTHMLTCPKMPRIVDKNQTQIGFKTAQGGDTDDVVVVSWKFEQEQCRRALCRMVIVDELPFKFVEKEGFRNFMKVTQPHFKIPSRTTVTRDCFKLFDAEKQKLKRSFGEAQQRVSLTTDTWTSLQRINYMCITAHWIDKEWMMHKRIINFCQVSSHRGEDMANEISKCLRDWGLDKNFTITVDNASSNDVTVKELSKIFTKRGTNFMNGEHLHVRCMAHILNLVVQDGLKVSAASIERVRKAVKYIRLSPARCKRFHECAEDVDINCKKSLCLDVSTRWNSTYLMLNRAIEFENVFSSYVDRDIGLLHYLQFVEDEDGTAAGALSSDDWNNVKKVADFLQIFYDLTREVSGSQYVTSNLHFLKICEVSCYLKKLIVSEDDTDDLLGKIAKNMREKFDKYWGTPNKMNKMIFISCVLDPRHKFVSVGFALQMMFGKEKGLVLEHEVRGYMDLMFGEYVKSLSKDKDSHHSSFLSSSSFEKFSSLPSSSDSTVQSIGSLGSFMVDLMKHKAGNATIVKTELQKYLGEENEVETKNFNILSWWKINSPRFPILAEMARNVLAISISSVASECAFSTGGRILDSFRSSLTPKLVQTLVCLQDWIRSESRHVSVEEDIDVLEQDLANTSILDD	zf-BED--DUF-domain--Dimer_Tnp_hAT--	I
PGSC0003DMT400051036	Pgs Zf-BED13_I	MDTLTTLNEIADSEAQPNKFRRKKSIVWEHFTIERIGADCTRACCKKCKKSFAYISGSKLAGTSHLKRHIALGICPVGRTNQDKNQLTSFNSAAPTNGSAEATGKSRKRYRANPGPTNIPFDQARCYYDIAKMIIQHDYPLEMVEHSGFNKFVQNLQPLFSSVSVDTIQEHIFNIYLGEKQNLLNIIGAIPGRVSLTLNLRTSDQNLGYVFLTGYFVDSDWKLRCRLLNVIMVPFPDSDVAFNHAVAACLTDWCLETKLFTLTLDQSVANVNVRKNLGHLLSIKGGNILNGQLIIGSCCARVLSDLAQYALHYMRAIVEKVRQSVKFVKTADAHEEKFLELKRQLQVPSAKELIVDDQTKWDTTYQMLMTASELKEVFSCLDTSDPDYKVTPTMDEWKQAEILCEYLKLFFDAANLLTSPTYSTADVLFHEVWKIQLDLMQAARSQDRFIRDLTRPLQEKFEEYWNDCNLVLAVAVVMDPRFKMKLIEFTFNKIYGEEAESWIKIVDEGVHEIFCDYIVQSLPPPPASFVDEANDSFVIKSEFSQEDSFLANGDTFADFDVYLHDIMNNQQMKTELDQYLEESLMPRSQDFDVLGWWRINRCKYPTLSKMASDILSIPVCTVTPDSVFDTTLRDLDRHRSSLRPITIEALSCSKDWLQYESWELPYGTPDATVKMEY	zf-BED--DUF-domain--Dimer_Tnp_hAT--	I
PGSC0003DMT400	Pgs Zf-BED14_I	MEIEEEAVIVNSSRLKSVVWNDFDRVKKGDTFVAICRHCKRKLSGSSTSGTSHLRNHLIRCRRRSNHDISQLLTRGKKKEGPLAISNFSFDQEQRNGDAVSVVRTKFEQGHTRDGLFNNGIVNFDNRRSRLDLARMIILHGYPLSMVEHIGFRIFIRNLQPLFDIATFDGVEADCREIYLMERQKVYEELDKLPGKISLSADTWTANGDAEYLCLTAHYIDDSWHLKKKILNFLTTDPSQTEDMLSEVIMTSLRNWDIDRKLFSVTFDNYSTYDKIVSRIREQLCQHRFLYCDGQLFDTRCAANVIKLMVQDTLETASQIIHKVR	zf-BED--DUF-domain--Dimer_Tnp_hAT--	I

05470<br>8		ESIRYVRSSQATQEKFTEMAQIAGVDSQKCLNLDNSFYWNSTYIMIETALEYKDAFPLLQEHDSRYAMCPTVTEWDRISAI<br>ASFLKLFVEVSNVFAGSKYPTANTYFPDICDIHLQLIEWCQNSDDFVNSLALKLKSRFDEYWKKCSLALAIAAILDPRFKMQ<br>LVKYYYPQIYGDSAPDCINIVSDCMKALYNGHAIYSPLAPNGQAEASQVGGANNDRLTGFDKFIYETSVSNNIKSDLDNYL<br>EEKLFPRKDDFNILNWWKVHTPRYPILSMMARNILGMPMSKASLEYVFNTGNKALEPYRSSLRSDTLQALMCAQDWMRD<br>EFEDSKASSSTVTLALCYDAK		
PGSC<br>0003D<br>MT400<br>05470<br>9	Pgs<br>Zf-<br>BED<br>15_I	MEIEEEAVIVNSSRLKSVVWNDFDRVKKGDTFVAICRHCKRKLSGSSTSGTSHLRNHLIRCRRRSNHDISQLLTRGKKKE<br>GPLAISNFSFDQEQRNGDAVSVVRTKFEQGHTRDGLFNNGIVNFDNRRSRLDLARMIILHGYPLSMVEHIGFRIFIRNLQPL<br>FDIATFDGVEADCREIYLMERQKVYEELDKLPGKISLSADTWTANGDAEYLCLTAHYIDDSWHLKKKILNFLTTDPSQTED<br>MLSEVIMTSLRNWDIDRKLFSVTFDNYSTYDKIVSRIREQLCQHRFLYCDGQLFDTRCAANVIKLMVQDTLETASQIIHKVR<br>ESIRYVRSSQATQEKFTEMAQIAGVDSQKCLNLDNSFYWNSTYIMIETALEYKDAFPLLQEHDSRYAMCPTVTEWDRISAI<br>ASFLKLFVEVSNVFAGSKYPTANTYFPDICDIHLQLIEWCQNSDDFVNSLALKLKSRFDEYWKKCSLALAIAAILDPRFKMQ<br>LVKYYYPQIYGDSAPDCINIVSDCMKALYNGHAIYSPLAPNGQAEASQVGGANNDRLTGFDKFIYETSVSNNIKSDLDNYL<br>EEKLFPRKDDFNILNWWKVHTPRYPILSMMARNILGMPMSKASLEYVFNTGNKALEPYRSSLRSDTLQALMCAQDWMRD<br>EFEDSKASSSTVTLALCYDAK	zf-BED--DUF-<br>domain--<br>Dimer_Tnp_hAT<br>--	I
PGSC<br>0003D<br>MT400<br>05480<br>8	Pgs<br>Zf-<br>BED<br>16_I<br>I	MASPNPHSSPLVAVDQRRLQFTPVDLPLTPPMADAIPEGIVDLTSLTEASTGEKRKREGRLRSRVWQHFTKLIKEDGTCD<br>KCKCNHCHKIFTSSTRSGTTHLLRHISEGICPAFKKVKKENSPSIFSYIGGSIDRKVGINPWKFDQELGQASFEQSIDAHDD<br>LLPMGLDDIERQTCTASESDYVSQTSMPVFSKLPQQPAVKSHPIAEPWMTELKACVGKLVELTNERVPKPTSTDNKTCVA<br>VTTPDLSIASVVKCLNEMEDIPQSSAMYLDALDIVRDPEERECFICLNPEPRRRWLQRMLHRRFPLRYSTDV	zf-BED	II
PGSC<br>0003D<br>MT400<br>05486<br>3	Pgs<br>Zf-<br>BED<br>17_I	MEIPTETPIKKPKRLTSVVWNHFERVRKADICYAVCVHCKKKLSGSSNSGTTHLRNHLLRCLKRSNYDVTQILAAKRKKKD<br>PTLAVVTYEEGQRKEETISPVTTFKFDPEVKKEEVNVPINLGSVRFDQERSRLDLARMIMLHGYPLAMVDHIGFKIFVKNLQ<br>PQFEVLTNSAVELDCMTIYAKEKQKMYEAIHNLHGRISLSADVWDSSENARYMCLTAFYIDEDWKLQKKMLNFITLDPSHT<br>DDILSEVVIKSLTDWAIDRKLFSMTFDHCTGYEELIFRIKDWLSQNRPLLKNGELFDVRCAVQLMKSIVSDVMEAIRDVTHK<br>VRESIRHVKSSQVTLGKFNEIAQQAAISGERPLILDCGQQWSSTYLMLEAALDYRGAFCLLEEHDPTYTSALSETEWDHA<br>SAVAGYVKLFVEVTNVFTTNKYSTANIYFPEICDIHIQLIEWCKNPDNFLSDIALKMKEKFDRYWSKCSLTLAIAAILDPRFKM<br>KLVEYYYPQIYDSDAPNQIKAVSDAIRELSNEYAMGSSSLDPDTAGASGSLASTTIGTRDRLRGFDKFLHETSQSHNMTSD<br>LDKYLEEPVFPRNYDFSILNWWKVHTPRYPILSMMARDILGVPASTLGPELAFNNRGRVLDHHRSSLNPGAREALICGQD<br>WLRMESEEYNSPHIYTAVPLTVESK	zf-BED--DUF-<br>domain--<br>Dimer_Tnp_hAT<br>--	I
PGSC<br>0003D<br>MT400<br>06090<br>6	Pgs<br>Zf-<br>BED<br>18_I	MATPETPVTPATPTENHEMVHEHEMIPEHEMGHEHEMGHEHDMSHELDMGHEHEMGHEHEMMHEHEMVEHEIVLGN<br>EMVPGNGMVQDYEMIPGNEMVLAEPQPNNVETPETQPGKHRKKKSIVWEHFTIENVAVGTRRAQCKKCKQSFAYSTGS<br>KVAGTSHLKRHIAKGSCPVVLRNQQNDQLTPYSAPPKMTGYGGSNGAPKRRYRTASTPYVAFDPDRCRQEISKMIIMHD<br>YPLHMVEHPGFLTFVQNLQPRFDMVSFNTVQGDCVATYLREKQAIQKVIEGVPGWICLTLDMWSSHHTVGYVFITGQYV<br>DSEWKIHRKILNVIMEPYPDSEMAFSHAVAACLSDWSMEGKLFSVTINQPLGDAAIDNLRALLSVKNPLVLNGQLLVGNCL<br>ARTLSSIAQDAFKVVQGTVKKVRDSVKYVKTSESHEEKFIELKQQLQVPSTKMLTLDDRTQWNTTYEMLLAATELKEVFS<br>CLDALDPDFKDAPSLEDWKQVETLCTYLKIFFDTANLLTAPTIPTTNTFFHEAWKIQLELARAAASEDPFTSRLTKMMQEM<br>FDNYWKSCCLMLAIAVVMDPRFKMKLVEFSFSKIYGEEAAAYVKTVEEGIHELFNEYVSLPLPLTPTYVEEVNGSAMGQE<br>DGQGFDASCNGLGLTDFDVYIMETSSQQSRSELDQYLEESLLPRVHEFDVVGWWKLNRMKYPTLSKMARDILSVPVSTV<br>TADSIFSTVSKEMDHYRCSLRPETVEALICAKDWLQNASIDTSPVPIKMEVPI	zf-BED--DUF-<br>domain--<br>Dimer_Tnp_hAT<br>--	I
PGSC<br>0003D<br>MT400<br>06538<br>6	Pgs<br>Zf-<br>BED<br>19_I<br>I	MDPRSTVWQHFEKNFKNGLLVKAKCLHCKQNYAANTSRNGTSGLKQHLTNRCKVYKPPPVAPGIQKLLNIQSNSYSIET<br>WKFEQEVCRRALVEMIILDELPFSFVEKEGFKKFMSKVQPLFRIPSRRTITRDSYEVYGELRMNLKMSFREIQPRICLTTDT<br>WTLV	zf-BED	II
Potri.0<br>01G40<br>4800.3	Pot<br>Zf-<br>BED<br>01_<br>XIV	MDQERPQKKRVKHTDPFWDHVEKTSDGGPFKCKFCKSSFAASTSISRFKYHLSGESGKGVGICGRVPVDVKAAAYQAM<br>HKKQNAIPPIDHPVPNVEARRMEQEGRDLPDMAMEDWTESTRWEEFIEELMVINEAGGSQGQGVYEGMLDTENLTHSR<br>TVEGIELIDQVRVYEEQGADVSDGGVENLTDNFTGSVSIVTDESRVSEGLHAHKAKGEALLTTKLVGQASDRNKEMIWSW<br>LMKDDVLSVGIYGMGGVGKTSLVTHIHNQLLQRPSSFNYVFWVTVSQNFTISKLQYLIAKAINLDLSNEEDEKKRAAKLSK<br>ALVAKGKSVLILDDLWNHFLLEMVGIPVEVNACKLILTSRSLEVCRRMGCQKSIKVELLTKEEAWTLFVEKLGQRHYADLS<br>PEVADIAKSVAAECACLPLGIIAMAGSMREVNDLYEWRNALTELKQSEVGVEDMEPEVFHILRFSYMHLNDSALQQCLLY<br>CAFFPEDFTVDREDLIGYLIDEGIIQPMKSRQAEYDRGQAMLNKLENACLLESYISKEDYRCFKMHDLIRDMALQKLREKS<br>PIMVEVEEQLKELPDEDEWKVDVMRVSLMKNHLKEIPSGCSPMCPKLSTLFLFSNFKLEMIADSFFKHLQGLKVLDLSATA<br>IRELPSSFSDLVNLTALYLRRCHNLRYIPSLAKLRGLRKLDLRYTALEELPQGMEMLSNLRYLNLFGNSLKEMPAGILPKLS<br>QLQFLNANRASGIFKTVRVEEVACLNRMETLRYQFCDLVDFKKYLKSPEVRQYLTTYFFTIGQLGVDREMDSLLYMTPEE<br>VFYKEVLVHDCQIGEKGRFLELPEDVSSFSIGRCHDARSLCDVSPFKHATSLKSLGMWECDGIECLASMSESSTDIFESLE<br>SLYLKTLKNFCVFITREGAAPPSWQSNGTFSHLKKVTIGECPSMKNLFSLDLLPNLTNLEVIEVDDCDQMEEIIAIEDEEEG<br>MMVEDSSSSSHYAVTSLPNLKVLKLSNLPELKSIFHGEVICDSLQEIIVVNCPNLKRISLSHRNHANGQTPLRKIQAYPKEW<br>WESVEWGNSNSKNALEPLCVFWESLF	zf-BED--NB-<br>ARC--LRR-<br>motif--	X<br>I<br>V
Potri.0<br>01G40<br>4800.4	Pot<br>Zf-<br>BED<br>02_<br>XIV	MDQERPQKKRVKHTDPFWDHVEKTSDGGPFKCKFCKSSFAASTSISRFKYHLSGESGKGVGICGRVPVDVKAAAYQAM<br>HKKQNAIPPIDHPVPNVEARRMEQEGRDLPDMAMEDWTESTRWEEFIEELMVINEAGGSQGQGVYEGMLDTENLTHSR<br>TVEGIELIDQVRVYEEQGADVSDGGVENLTDNFTGSVSIVTDESRVSEGLHAHKAKGEALLTTKLVGQASDRNKEMIWSW<br>LMKDDVLSVGIYGMGGVGKTSLVTHIHNQLLQRPSSFNYVFWVTVSQNFTISKLQYLIAKAINLDLSNEEDEKKRAAKLSK<br>ALVAKGKSVLILDDLWNHFLLEMVGIPVEVNACKLILTSRSLEVCRRMGCQKSIKVELLTKEEAWTLFVEKLGQRHYADLS<br>PEVADIAKSVAAECACLPLGIIAMAGSMREVNDLYEWRNALTELKQSEVGVEDMEPEVFHILRFSYMHLNDSALQQCLLY<br>CAFFPEDFTVDREDLIGYLIDEGIIQPMKSRQAEYDRGQAMLNKLENACLLESYISKEDYRCFKMHDLIRDMALQKLREKS<br>PIMVEVEEQLKELPDEDEWKVDVMRVSLMKNHLKEIPSGCSPMCPKLSTLFLFSNFKLEMIADSFFKHLQGLKVLDLSATA<br>IRELPSSFSDLVNLTALYLRRCHNLRYIPSLAKLRGLRKLDLRYTALEELPQGMEMLSNLRYLNLFGNSLKEMPAGILPKLS<br>QLQFLNANRASGIFKTVRVEEVACLNRMETLRYQFCDLVDFKKYLKSPEVRQYLTTYFFTIGQLGVDREMDSLLYMTPEE<br>VFYKEVLVHDCQIGEKGRFLELPEDVSSFSIGRCHDARSLCDVSPFKHATSLKSLGMWECDGIECLASMSESSTDIFESLE<br>SLYLKTLKNFCVFITREGAAPPSWQSNGTFSHLKKVTIGECPSMKNLFSLDLLPNLTNLEVIEVDDCDQMEEIIAIEDEEEG<br>MMVEDSSSSSHYAVTSLPNLKVLKLSNLPELKSIFHGEVICDSLQEIIVVNCPNLKRISLSHRNHANGQTPLRKIQAYPKEW<br>WESVEWGNSNSKNALEPLCVFWESLF	zf-BED--NB-<br>ARC--LRR-<br>motif--	X<br>I<br>V
Potri.0<br>01G40<br>4950.1	Pot<br>Zf-<br>BED<br>03_I<br>I	MELFVETHMRSQDRQKEPQQFIDNRAQHFVERQQKKRVKHTDPFWDHVEKTNDGGPFKCKFCKSTFAASTSISRFKYH<br>LSGESGKGVGICGRVPVDVKAAAYQAMHKKQNAFPVRN	zf-BED	II
Potri.0<br>01G40<br>5100.3	Pot<br>Zf-<br>BED<br>05_<br>XIV	MDQERQQKKRVKHTDPFWDHVEKTNDGGPFKCKFCKSTFAASTSISRFKYHLSGESGKGVGICGRVPVDVKAAAYQAM<br>HKKQNPIPPIDHTVPNVQAQRIEQEGRDLPDMAMEDWTESMRSEELMVVNEAGGSRGSQGQGGYLLDMVDTENLTQS<br>RTMEGIALIDHVRVHEEQGTDVSDGVVENLTDNFTRGVSIVTEESRVSEGLDAHKAKGEALLTTKLVGQASDRNKETIWS<br>WLMKDDVLSVGIYGMGGVGKTSLATQIHNQLLQRPSSFNYVFWVTVSQNFTISKLQYLIAKAINLDLSNEEDEKKRAAKLS<br>KALVAKGKSVLILDDIWNHFLLETVGIPVGVNACKLILTSRSLEVCRRMGCQKSIKVELLTKEEAWTLFVEKLGNYATFSPE<br>VVQIAKSVAAECARLPLGIIAMAGSMRGVDDLHEWRNALTELKQSEVRAEDMETEVFHILRFSYMRLNDSALQQCLLYCA<br>YFPEDFTVDREDLIGYLIDEGIIQPMKSRQAEYDRGQAMLNKLENACLLESFFSNENYRVFKMHDLIRDMALQKLREKSPI<br>MVEGGEQLKELPDESEWKEEVVRVSLMENHVKEIPSGCAPMCPKLSTLFLSLNFKLEMIADSFFKHLQGLKVLDLSATAIR<br>ELPSSFSDLVNLTALYLRKCEKLRYIPSLAKLRELRKLDLRYTALEELPQGMEMLSNLRYLNLFGNSLKEMPAGILPKLSQL<br>QFLNVNRLFGIFKTVRVEEVACLKRMETLRYQFCDLVDFKKYLKSPEVRQPLTTYFFTIGQLGVDRVMDSLLYMTPDEVFY<br>KEVLVHDCQIGEKGRFLELPEDVSSFSIGRCHDARSLCDVSPFKHATSLKSLGMWECDGIEFLASMSESSTDIFESLESLY<br>LKTLKNFCVFITREGAAPPSWQSNGTFSHLKKLRIGECLSMKNLLALDLLPNLTNLEVIEVDDCDQMEEIIAAEDEEEGMMV<br>EDSSSSSHYAVTSLPNLKALKLSNLPELESIFHGEVICGSVQEILVVNCPNLKRISLSHRNHANGQTPLRKIQAYPKEWWE<br>SVEWGNSNSKNALEPLCVFRESLF	zf-BED--NB-<br>ARC--LRR-<br>motif--	X<br>I<br>V

Potri.002G221900.1	PotZf-BED06_XXII	QTKTHISISLATLYFCASRREDLVCLFVKGVTFIAKTDDNFICATLDWRPSEKCDYNQCPLEKAGLLNLMDNREDSTPNES<br>SPLSLEPNSSPIPVPVLVPVTTSSIDSNTKGNPPSKCNKRKTSRIWDHFKKLDSNPKALRAACMYCGKDYTCRTIFNGTSN<br>MWSHLGVCKKFSFVIDRKQKTLVLEPKPIIERGDNGEENLAIIKAVNYNYEECRKALEKLIILDEIPFNFVENQGFQSFCQVM<br>QPRFNVPSRLMN	Glyco_hydro_79n--zf-BED--	XXII
Potri.012G059200.1	PotZf-BED07_I	MDFGTGSVSGRAAANQMEWTVNNAFKTYKDMDHPKSMMDVALIQNVDPVDIGLGSSEKGTIVVPTKRKKTMTSVYLKFF<br>ETAPDGKSRRCKFCGQSYSIATATGNLGRHLSNRHPGYDKSGDSVTSSAPQPITVVKKAQQQGKQQMDYDHINWLLVK<br>WLILASLPPSTLEEKWLANSFKFLNPSIQLWPGERYKVKIREVFRSMQEDVMATLEKVSSKVSIILDFWSSYEQIFYMSVTC<br>QWIDENWSFQQVLLDICQIPYPCGGSEIYHSLEKVLKMYNIESRVLSCTHDNSQNAIHACHTLKEELDGQKLGMFCYIPCA<br>ARTLNLIIEDGLRTTKPVISKVREFVLELNSSAKMSEDFIQLTAAYQEGSWKFPLETSARWSGNYQMLDIVCKAGKSMDGV<br>MRKYEETIVGRTVLSPAEKNAVSIVHKYLEPFYKTTNNICTNKLLTIGLVLFFMDHISEMITLCKDSRLSSDWLKNAAEDMAT<br>KSRSYTTQVGNIFIFMTAILDPRIKCELIPESLSSGNYLEEARTLFIRNYSSSHFSSMTSGYGAQEIEDGGGVSFAEEIARKK<br>RRVSLSNATDELTQYLSEPPAPIPTDVLEWWKVNSTRYPRLSVMARDFLAVQPTSVAPEDLFCSKGDEIDKQRFCMPHD<br>STQAILCIRSWMQGGIKLKCKSDEIDYERLMEMAGATTAENTVGLDKKQR	zf-BED--DUF-domain--Dimer_Tnp_hAT--	I
Potri.012G059200.2	PotZf-BED08_I	MDFGVRKKDQTGSVSGRAAANQMEWTVNNAFKTYKDMDHPKSMMDVALIQNVDPVDIGLGSSEKGTIVVPTKRKKTMT<br>SVYLKFFETAPDGKSRRCKFCGQSYSIATATGNLGRHLSNRHPGYDKSGDSVTSSAPQPITVVKKAQQQGKQQMDYDHI<br>NWLLVKWLILASLPPSTLEEKWLANSFKFLNPSIQLWPGERYKVKIREVFRSMQEDVMATLEKVSSKVSIILDFWSSYEQIF<br>YMSVTCQWIDENWSFQQVLLDICQIPYPCGGSEIYHSLEKVLKMYNIESRVLSCTHDNSQNAIHACHTLKEELDGQKLGM<br>FCYIPCAARTLNLIIEDGLRTTKPVISKVREFVLELNSSAKMSEDFIQLTAAYQEGSWKFPLETSARWSGNYQMLDIVCKAG<br>KSMDGVMRKYEETIVGRTVLSPAEKNAVSIVHKYLEPFYKTTNNICTNKLLTIGLVLFFMDHISEMITLCKDSRLSSDWLKN<br>AAEDMATKSRSYTTQVGNIFIFMTAILDPRIKCELIPESLSSGNYLEEARTLFIRNYSSSHFSSMTSGYGAQEIEDGGGVSF<br>AEEIARKKRRVSLSNATDELTQYLSEPPAPIPTDVLEWWKVNSTRYPRLSVMARDFLAVQPTSVAPEDLFCSKGDEIDKQ<br>RFCMPHDSTQAILCIRSWMQGGIKLKCKSDEIDYERLMEMAGATTAENTVGLDKKQR	zf-BED--DUF-domain--Dimer_Tnp_hAT--	I
Potri.012G059200.3	PotZf-BED09_I	MEWTVNNAFKTYKDMDHPKSMMDVALIQNVDPVDIGLGSSEKGTIVVPTKRKKTMTSVYLKFFETAPDGKSRRCKFCGQ<br>SYSIATATGNLGRHLSNRHPGYDKSGDSVTSSAPQPITVVKKAQQQGKQQMDYDHINWLLVKWLILASLPPSTLEEKWLA<br>NSFKFLNPSIQLWPGERYKVKIREVFRSMQEDVMATLEKVSSKVSIILDFWSSYEQIFYMSVTCQWIDENWSFQQVLLDIC<br>QIPYPCGGSEIYHSLEKVLKMYNIESRVLSCTHDNSQNAIHACHTLKEELDGQKLGMFCYIPCAARTLNLIIEDGLRTTKPVI<br>SKVREFVLELNSSAKMSEDFIQLTAAYQEGSWKFPLETSARWSGNYQMLDIVCKAGKSMDGVMRKYEETIVGRTVLSPA<br>EKNAVSIVHKYLEPFYKTTNNICTNKLLTIGLVLFFMDHISEMITLCKDSRLSSDWLKNAAEDMATKSRSYTTQVGNIFIFMT<br>AILDPRIKCELIPESLSSGNYLEEARTLFIRNYSSSHFSSMTSGYGAQEIEDGGGVSFAEEIARKKRRVSLSNATDELTQYL<br>SEPPAPIPTDVLEWWKVNSTRYPRLSVMARDFLAVQPTSVAPEDLFCSKGDEIDKQRFCMPHDSTQAILCIRSWMQGGIK<br>LKCKSDEIDYERLMEMAGATTAENTVGLDKKQR	zf-BED--DUF-domain--Dimer_Tnp_hAT--	I
Potri.016G026900.1	PotZf-BED10_II	MNGGLQSTNKRKISTVWDRFVKHRGENGEVWATCKYCKKTYRAESKRGTSNLHKHLKKMFAKQTGRSRAANFG	zf-BED	II
Potri.017G018600.1	PotZf-BED11_XVIII	MATPGFNKLLDCAKAIEVGDLHLADSLIKDILTDNDDKLVKYCAEALVRRVYKLYPRNPRPLVPSCTDLRCNMDYQFFPFF<br>WFSELTTRNTIVDALTGKKRVQVIDFSLMANGRRWCLLLDDYLKQSSDAISFHLTSIGPILSKKGDYLNEILEKLPKEAKKLP<br>IEFEVKHMVASSPAEMVEAALKLERSSEDETIVVRWEFELHKLLALPGAIETVLSKLKELKPEIMIVVEQEASLNGQDFLECF<br>TKSFRYHSIIFDSLGKDNFEHGNHSKVLWEMYFRRQISNLVAQEGTDQIVRHQTFAEWRDRFCRSGFRHVRLQNQFKGT<br>FFGHLPEYHIEEMNRHPVLYRHDDQLLFTSAWKSYPTQLNSESLDNWNQESAMGDGNIMQIESSSLLILSGTVSSSAFLE<br>DQPDDEHIIMEGKVWSPECISINQVAASAKIFDMLEYICHVNYLPLALTWMSDRRILRLEKSACYLNDSTMVEFMEACGEH<br>HLEEGKGVAGKALQSNSMYFVSDISKLDVKDYPFIFEAWDFGLRGVVAIKLESVYVSSIDYVVEFFLPLEMKGISGQRLLIN<br>EITNILQKNSRNSWKVCTQELNGFNISSEVEVTMEEVGTSNIRPAAISDSPPLALSEIGSLNSSQIRELCNIFMPTGDGVEVP<br>EAHDQEFEEQNSVFGPQAYQFPDSDELVGMDSSHINTSRTSKRRRTSEVWQYFDEVREDGEVWAKCRSCSTKYRGES<br>TRGTTNLRKHLRSCPGKK	GRAS--zf-BED--	XVIII
Potri.017G020600.1	PotZf-BED12_II	MSKKYACDADIAILFIPGEKKEHGFPEWTSNVSYYRPDEYAHLSDAISCGVRGIIAFPVFESDQPKYCCAVLELVTMEEKQ<br>DFDLETEKVVQALQAANLGIKLPRPRPRPPQVQRAALTEIADVTRAVCQTHGLPLALTWIPWGDDDILKLGNLSFWWSGE<br>LAIERTACYADEEMQGFVHACEQLFLYPGQGAAGQACQTNLPSFEPNVKEKLVSKYPLAHHARKYNLNAAVAIQLRSTG<br>DDYIYILEFFLPILVKERSEQQELVEKLKLTIKQTCKSLRMLSKKPSIRKRGSKVSKNNDKIEPAPDPDGNMFIECADNKRQK<br>VSEVWKDFDKFIDKENKVWAICKGCKKKYTGDSKMGTSNLHKHLKSCSRKRQRDAKQHTLPSDLGGNFVFDQERSRLN<br>FVRMIMKLGFPLDMVQNEFFKTFVCDLQPKFQLCSQDISYGDNKITCCLTMHFIDDGWRMNKKILAFRSIEHDYMNEAVK<br>DVLVEWNIIEKVHFMFAEIAPPNSQMTREFRSKLVSQFPHMNGDLLCFSCYAQTLELLARDGFYEIKDVLNKIRAVLNM	zf-BED	II
Potri.017G021200.1	PotZf-BED13_II	MNLFRVFLKTLPLFVLQCISINQVAASAKIFDMLEYICHVNYLPLALTWMSDRRILRLEKSACYLNDSTMVEFMEACGEHHL<br>EEGKGVAGKALQSNSMYFVSDISKLDVKDYPFIFEAWDFGLRGVVAIKLESVYVSSIDYVVEFFLPLEMKGISGQRLLINEIT<br>NILQKNSRNSWKVCTQELNGFNISSEVEVTMEEVGTSNIRPAAISDSPPLALSEIGSLNSSQIRELCNIFMPTGDGVEVPEA<br>HDQEFEEQNSVFGPQAYQFPDSDELVGMDSSHINTSRTSKRRRTSEVWQYFDEVREDGEVWAKCRSCSTKYRGESTR<br>GTTNLRKHLRSCPGKK	zf-BED	II
Potri.017G021200.2	PotZf-BED14_II	MEGKVWSPECISINQVAASAKIFDMLEYICHVNYLPLALTWMSDRRILRLEKSACYLNDSTMVEFMEACGEHHLEEGKGV<br>AGKALQSNSMYFVSDISKLDVKDYPFIFEAWDFGLRGVVAIKLESVYVSSIDYVVEFFLPLEMKGISGQRLLINEITNILQKN<br>SRNSWKVCTQELNGFNISSEVEVTMEEVGTSNIRPAAISDSPPLALSEIGSLNSSQIRELCNIFMPTGDGVEVPEAHDQEF<br>EEQNSVFGPQAYQFPDSDELVGMDSSHINTSRTSKRRRTSEVWQYFDEVREDGEVWAKCRSCSTKYRGESTRGTTNL<br>RKHLRSCPGKK	zf-BED	II
Potri.017G021966.1	PotZf-BED15_II	MQCFVHSCEQLFLNTGHGAAGQAHRTYLPSFEPDVKEKHVSEYPRAHHTRKYNLNAAVAIVLRSTGTGCSYILEFFLPVL<br>MTDILEQQDLVTNLKLKLRETCKSLRMFSEAQLVGEQGSKASKKNAKMTPSPNADGNMFTVCANNKRRKVSEVWKWFD<br>KVKEKNGVVWSICKGCQKKHPGESKKGTSNLHKHLKRCSMGHM	zf-BED	II
Potri.017G021966.2	PotZf-BED16_II	MQCFVHSCEQLFLNTGHGAAGQAHRTYLPSFEPDVKEKHVSEYPRAHHTRKYNLNAAVAIVLRSTGTGCSYILEFFLPVL<br>MTDILEQQDLVTNLKLKLRETCKSLRMFSEAQLVGEQGSKASKKNAKMTPSPNADGNMFTVCANNKRRKVSEVWKWFD<br>KVKEKNGVVWSICKGCQKKHPGESKKGTSNLHKHLKRCSMGHM	zf-BED	II
Potri.017G033866.1	PotZf-BED17_II	MAGGSDPFQSYFEKVDGGLLKCIFCEKTLAGSTSTTRMKYHLARVGGGGVKICEKVTPDVQRAAFDKLPDRMRGSMPS<br>SSNNIIVTADSDPAQDLEMQQQGQSLLDDFSWMDSLTWEEIVLPEETMVPSMHMPDAPETGLGIEPAVQAFETDMNNIT<br>SS	zf-BED	II
Potri.019G002700.1	PotZf-BED18_XII	MLKMIRPNDPVWRHADRSSRAYVENMDDGRMKCKLCRQLFAQGTSISRIKFHLSGVKGRGVKICEDVPKEVQDAALAAI<br>DGPPEKKLKTVAGSSSNEVPNAQEQNNEGTHVEMAQHGEAVFTGEHAWANDFIGGGIELVHNTSERRPVIGGIEPMHNA<br>PETRPITEQTSQERERGICDFSSSSTVDNVIGNNIENPATGFMQPGAGASSSGGLKYYTSETRGDLLPTSSTKLVGRAFE<br>ENRNMIWSWLMDDEVLTIGVYGMGGVGKTTMLQYIHNELLKRSDISRHLDKVGISFQLNGCKPIIITRSERVCYRMNCQHE<br>IKAMPLSDGDAWTLLMEELGHDIPLSLEVELIVVAVARECSGLPLGIITMAGSLRGVEEY	zf-BED--NB-ARC--	XII

Potri.019G052900.1	Pot Zf-BED 19_II	KNSSSNRLDVGWQHGIDIDKNSRKVQCNYCQKIISEGIFHFKQHLACTRKDVEPCQQKRKAHQYSGNDDDDEIKEISSKD<br>KEKRATSGSGSTQTLNQLLKKDIRAEACRQIARFFYTSAIPFNYVKNLEFLKAFELVAKHGPGFKPPSYHDIREKYLSRKWI<br>KQ	zf-BED	II
Potri.T012100.1	Pot Zf-BED 20_II	MLRQNDPVLHHVDRSFWEYVEKMDDGRMRCKFCGHCFAERTSISRIKLHLAGVTGRGVKTCGQVPPDVQDAALAAIDG<br>PQEKKLKTLAGSSNNDVTNEISPSAQQQNNEMMMARQREDLWLEDLVTSITVEDMELLERGSFHERPSFYQADEPRGD<br>PSQPTNDQLCSPSVNNDVIVNDAQNVFRWEFRAWNMVGRKREFVRI	zf-BED	II
Potri.T013632.1	Pot Zf-BED 21_XII	MAKRKDRFWDHVEKLDDGRFICTFCGFKFAAAASISRIKWHLSGEEGHGVAVCGQVPQQVQEAAFLDMRHCNKRHKGI<br>ASSSNFNDNVISTTPQEQNNEVDNVAGDAGTTQAADRMGHPLGRSVEEFSRWLMEDDIENGTGGVVQPGAGASSSGG<br>LTDNTNETPGDPLPTSSTKLVGRAFEHNTNLIWSWLMDDEVSIIGIYGMGGVGKTTMMKHIYNKLLERLGISHCVCWVTVT<br>RDFSIERLQNLIARCLGMDLSSEDDDLCRAVKLSKELRRKQK	zf-BED--NB-ARC--	XII
Potri.T028400.1	Pot Zf-BED 22_II	MGRSDDPFWKEVEDMNDGSMKCKFCGHLFANGTSISRIKWHLSGERGHGVGICGQVPKEVQELSSLSSYAWWQQKT	zf-BED	II
Potri.T078300.1	Pot Zf-BED 23_XVIII	MATPGFNKLLDCAKAIEVGDLHLADSLIKDILTDNDDKLVKYCAEALVRRVYKLYPRNPRPLVPSCTDLRCNMDYQFFPFF<br>WFSELTTRNTIVDALTGKKRVQVIDFSLMANGRRWCLLLDDYLKQSSDAISFHLTSIGPILSKKGDYLNEILEKLPKEAKKLP<br>IEFEVKHMVASSPAEMVEAALKLERSSEDETIVVRWEFELHKLLALPGAIETVLSKLKELKPEIMIVVEQEASLNGQDFLECF<br>TKSFRYHSIIFDSLGKDNFEHGNHSKVLWEMYFRRQISNLVAQEGTDQIVRHQTFAEWRDRFCRSGFRHVRLQNQFKGT<br>FFGHLPEYHIEEMNRHPVLYRHDDQLLFTSAWKSYPTQLNSESLDNWNQESAMGDGNIMQIESSSLLILSGTVSSSAFLE<br>DQPDDEHIIMEGKVWSPECISINQVAASAKIFDMLEYICHVNYLPLALTWMSDRRILRLEKSACYLNDSTMVEFMEACGEH<br>HLEEGKGVAGKALQSNSMYFVSDISKLDVKDYPFIFEAWDFGLRGVVAIKLESVYVSSIDYVVEFFLPLEMKGISGQRLLIN<br>EITNILQKNSRNSWKVCTQELNGFNISSEVEVTMEEVGTSNIRPAAISDSPPLALSEIGSLNSSQIRELCNIFKPTGDGVEVP<br>EAHDQEFEEQNSVFGPQAYQFPDSDELVGMDSSHINTSRTSKRRRTSEVWQYFDEVREDGEVWAKCRSCSTKYRGES<br>TRGTTNLRKHLRSCPGKK	GRAS--zf-BED--	XVIII
Potri.T107066.1	Pot Zf-BED 24_XII	MVGGSDPFRRYFEKMDNGLWKCKFCEKKLAKDTKATRMKCHLARVGGGGVEICEKVTPDVQQAAFDKLPDRMRGSMP<br>CSSNNIIVTADSPPAQDLEMQQQGQSLLDEFPCTDSSTWEEIWHNVENGAPSMLMPDAPETGLGIEPPDQPFEMDMNNI<br>TSSFTRDAELSSGIESRELMQAVTERGSCSKMPVDKSVPSSSNNEGINAASTALRGLEMEQEEQPLSDERGWKGFLTGG<br>EITELVEEPRAPVVLMPDEPETRQRTEQAHQSFEMNLNNISSSSMRDFELRIGRLTTSLELMQSVVERRPSSKTPVHKKR<br>RTGRYVLPTTKLVGQAMERNMKDVWSWLLNDEVSCIGIRGMGGVGKTALATHIYNQLHEKLGMFHPVRWITMSQNFSIH<br>VLQGRIAEVLDLKPLDENDAMVRTGELLTELNVKKKGFLILDNLWSHFLPDEVGIPLRTDGWKLILTTRSLEICRKMDCQRII<br>KVEPLSEGEAWDLLEIMFRTAELEFYSFFNF	zf-BED--NB-ARC--	XII
Prupe.1G105600.1	Pru Zf-BED 01_III	MPFVFSNPDSYFLLPNSHCATMRSSGLVDPGWEHGMAQDERKKKVKCNYCGKIVSGGIYRLKQHLARVSGEVTYCDKA<br>PEDVYMSMKANMEGSRSNKKPRHSEDIGQAYLNFQSNDDEEEVHVGYRSKGKQLMGDRNLAMKLTPLRSLGYVDPGW<br>EHGVAQDEKKKKVKCIYCEKIVSGGINRFKQHLARIPGEVAPCKHAPEEVFLKIKENMKWHRTGRRQRQADSKDMSPFDL<br>QSDNEDQDDDQMEAALHHINKERLIDGDRRLGQNLRNTFKALPPSTGSEPLFKRSRLDSLFLTAPKSLTPHSYRQVRVRT<br>MSNKISRKEVISGICKFFYHAGVPLQAANSVYFHKMLELVGQYGQGLVAPPSQLISGRFLQEELATIKTYLADYKASWAITG<br>CSIMADSWRDTEGRILINFLASGPNGVYFVSSVDATEIVEDASNLFKLLDKVVEEMGEENVVQVITPITPSYKAAGNMLEEK<br>RKKLFWTPCATSCIDQMLEDFLKIRCVAECMEKGQKITKLIYNQIWLLNFLKSDFTQGKELLRPSITRFASSFATLQSLLDH<br>RTGLRRMFQSNKWISSQCSKSCEGKEVESIVLNATFWKKLQFVRNSVDPIMQVLQKVESGDCLSMSSIYNDMYRAKIAIK<br>TIHGDNVRKYEPFWSVIESHWNSLFYHPVYVAAYYLNPSYRYRPDFTAHTEAMRGLNECIVRLEPDSARRISASMQISDY<br>NSAKADFGTELAISTRTELDPAAWWQQHGISCLELQRIAVRILSQTCSSFGCEHNWSIYDQLYSLRNNRLAQKRLNDLIYV<br>HYNLRLREQQLQLRRRADNSISLDNVLLERLLDDWIVDAAENDMLENEEVLYNEIEQVDEYENDMVDYEGVNGNAETRN<br>GSVELVTLADADINPANAGVATDDDDEDDEDGDINFFDDDMSD	zf-BED--zf-BED--DUF-domain--Dimer_Tnp_hAT--	III
Prupe.1G105600.2	Pru Zf-BED 02_III	MRSSGLVDPGWEHGMAQDERKKKVKCNYCGKIVSGGIYRLKQHLARVSGEVTYCDKAPEDVYMSMKANMEGSRSNKK<br>PRHSEDIGQAYLNFQSNDDEEEVHVGYRSKGKQLMGDRNLAMKLTPLRSLGYVDPGWEHGVAQDEKKKKVKCIYCEKI<br>VSGGINRFKQHLARIPGEVAPCKHAPEEVFLKIKENMKWHRTGRRQRQADSKDMSPFDLQSDNEDQDDDQMEAALHHI<br>NKERLIDGDRRLGQNLRNTFKALPPSTGSEPLFKRSRLDSLFLTAPKSLTPHSYRQVRVRTMSNKISRKEVISGICKFFYHA<br>GVPLQAANSVYFHKMLELVGQYGQGLVAPPSQLISGRFLQEELATIKTYLADYKASWAITGCSIMADSWRDTEGRILINFL<br>ASGPNGVYFVSSVDATEIVEDASNLFKLLDKVVEEMGEENVVQVITPITPSYKAAGNMLEEKRKKLFWTPCATSCIDQMLE<br>DFLKIRCVAECMEKGQKITKLIYNQIWLLNFLKSDFTQGKELLRPSITRFASSFATLQSLLDHRTGLRRMFQSNKWISSQCS<br>KSCEGKEVESIVLNATFWKKLQFVRNSVDPIMQVLQKVESGDCLSMSSIYNDMYRAKIAIKTIHGDNVRKYEPFWSVIESH<br>WNSLFYHPVYVAAYYLNPSYRYRPDFTAHTEAMRGLNECIVRLEPDSARRISASMQISDYNSAKADFGTELAISTRTELDP<br>AAWWQQHGISCLELQRIAVRILSQTCSSFGCEHNWSIYDQLYSLRNNRLAQKRLNDLIYVHYNLRLREQQLQLRRRADNS<br>ISLDNVLLERLLDDWIVDAAENDMLENEEVLYNEIEQVDEYENDMVDYEGVNGNAETRNGSVELVTLADADINPANAGVA<br>TDDDDEDDEDGDINFFDDDMSD	zf-BED--zf-BED--DUF-domain--Dimer_Tnp_hAT--	III
Prupe.1G105600.3	Pru Zf-BED 03_III	MRSSGLVDPGWEHGMAQDERKKKVKCNYCGKIVSGGIYRLKQHLARVSGEVTYCDKAPEDVYMSMKANMEGSRSNKK<br>PRHSEDIGQAYLNFQSNDDEEEVHVGYRSKGKQLMGDRNLAMKLTPLRSLGYVDPGWEHGVAQDEKKKKVKCIYCEKI<br>VSGGINRFKQHLARIPGEVAPCKHAPEEVFLKIKENMKWHRTGRRQRQADSKDMSPFDLQSDNEDQDDDQMEAALHHI<br>NKERLIDGDRRLGQNLRNTFKALPPSTGSEPLFKRSRLDSLFLTAPKSLTPHSYRQVRVRTMSNKISRKEVISGICKFFYHA<br>GVPLQAANSVYFHKMLELVGQYGQGLVAPPSQLISGRFLQEELATIKTYLADYKASWAITGCSIMADSWRDTEGRILINFL<br>ASGPNGVYFVSSVDATEIVEDASNLFKLLDKVVEEMGEENVVQVITPITPSYKAAGNMLEEKRKKLFWTPCATSCIDQMLE<br>DFLKIRCVAECMEKGQKITKLIYNQIWLLNFLKSDFTQGKELLRPSITRFASSFATLQSLLDHRTGLRRMFQSNKWISSQCS<br>KSCEGKEVESIVLNATFWKKLQFVRNSVDPIMQVLQKVESGDCLSMSSIYNDMYRAKIAIKTIHGDNVRKYEPFWSVIESH<br>WNSLFYHPVYVAAYYLNPSYRYRPDFTAHTEAMRGLNECIVRLEPDSARRISASMQISDYNSAKADFGTELAISTRTELDP<br>AAWWQQHGISCLELQRIAVRILSQTCSSFGCEHNWSIYDQLYSLRNNRLAQKRLNDLIYVHYNLRLREQQLQLRRRADNS<br>ISLDNVLLERLLDDWIVDAAENDMLENEEVLYNEIEQVDEYENDMVDYEGVNGNAETRNGSVELVTLADADINPANAGVA<br>TDDDDEDDEDGDINFFDDDMSD	zf-BED--zf-BED--DUF-domain--Dimer_Tnp_hAT--	III
Prupe.1G199600.1	Pru Zf-BED 04_I	MASSSVPSGASVPGGLDVAWKYACPIEGNKHGTMCTFCESKFKSGGITRVKYHLAGFDPHKSVKKCKEVPQYIKKEKFV<br>EENIRSTARGDIWAIQVAILMMMMMMRMRMDMHILQTCTLRRKKITLLPFERRSKKNGGASGSSQPQYRRTQSLQDPLR<br>SPLVPMPHKNTTAKQRTIKGMIKNSVEVLGRYCSKFFILENVAPQNASSPHFKNMIATAQQASQGLATPSSYEIKHKYLDM<br>EYTYMQAYVEKVKEDWGVYGCTIMSDGWTGSMRLSIINFMDVIKEVGSSNVVHIVIDNGSAFVKAGEMMMEWYPIYWTP<br>CAAHCINLIFEDIRKQESVANVINKARKLTNYICNHGWLLAQMRIFLQSIYEPLYSTLLIVDTEVVPTMPILYDMFHIMKEKISK<br>LKGKKWLLKIINHKWDVILSRPLHQVAHYLNPRYQYETGVDHNKELLLGLQHVFERLNPTGDEEINFRDYRKTFRTEMAVK<br>SRKSIPPVEWWNLHGDSAPNLQKITMHILSQTTSSLARENRLAYTRLENIVFCYFNMKLKLRDEEAEMNKVAENDYIDLLDI<br>AGQPSDDDNNPIQQWIMTAHLDDEQGNPDTVIAQHAAQEGVDVDRVISEDVRSGDTSSFERDMLGPRQGQRRPLRDNA<br>SANRKESLSNSLDNDGGSNAGSGGNE	zf-BED--DUF-domain--Dimer_Tnp_hAT--	I
Prupe.1G215800.1	Pru Zf-BED 05_II	MASSSGLSGASVPGGLDVAWKYARPIEGNTHGTIYTFCESTFKSGGITRLKYHLAGFDPHKSVKKCKEVPPDTKNKMRIF<br>YQGDLVRPGATRFATNYITINNILNKKAGLRQLFRSKEWYNSRFSESEEGKIIESRVLDHRLWDAMEGVQSIYEPLYSILRII<br>DTETASSSACERNWSTFALFHMKQRNRLAYTRLEKIVFCYYNMKLKLHDEEAEMNKVAENDYIDLLDIARVISEDVRSGDT<br>SSFEKDMLGPRQGQRRPLRDDANASRKESSSNSSDNDGGSNVGSGDEDHGCREAGPGIGAIGKQYSKKRSQPINPSE<br>EDMAISFGSMSIGTRPSTNSNESYDGYGYVMSNYSGTSYGAEDDETYYGPTSWVNPNYPIYRRTVGSSRETYMHHVQT<br>WLTNYSGYMTWYDYCMNLDGCSSLFEPHRSFSFM	zf-BED	II

Prupe. 2G058 600.1	Pru Zf-BED 06_I	MTSFHYNEPEEKKKKKHASRLPRRVAANLQSRKISSGSEVSRSGAPCSASLRLATPCNASSRLPRAKRATRETADRLGR<br>FPAASLRRARRRARRHAFENTAQELSDTSPDMQYKDTQVSVPPRASDPGWAHGIMVNGGRQKIKCKYCHKVMLGGGIS<br>RLKQHLAGERGNVAPCEEVPEEVKVQIQQHLGFKVLEKLKKQKGLSNSKDSAPYLQYREGGENEDGMRTQKTVCTRGQ<br>RRRREKEAMECISNQMKKHKKRSFPAATTIVAQPLHQSFASQEIIAQADLAVARFMYESGIPFTAANSHFYQQMADAIAAA<br>GPGYKMPSYHALRGKLLNKSVQDAEEYEEELRKSWDVTGCSVMVDRWVDKTGNSVINFFVYCPKGTLFLKSADASDISE<br>CPDALLNLFDCVVQEVGHKRIVNFVTDASASCKAARKLLMEKYKTFFCSTCGGYGIELMLEEIGKMNEVKEVLAKAKKITQ<br>FIYNSAWMLNLVRRKTGGRDIVQLATTRFASTFLTLQNMVALKHRLEKIFASAAWMHSTFSKQKAGLELAEIIADQLFWSL<br>CDQILKVTKPLLSVFQLMDCEEKPSIGYVYDAMEKAKKSIIVSFDNKESDYVPYLEIIDHIWQEELHSPLHAAAYYLNPSIFY<br>NPSFSTNKVIQKGLLDCIETLEPNLTAQVVITSNINFYEEAVGDFGRPVALRCRESLAPATWWSLYAADYPDLQRLAVRILS<br>QTCSIIRYKRSRTMFERMCSKKKNRLEQQRFNHLAFVHYNLHLQHRRSEASKAIYTRGTLDPICLEAIDANMGDWVEDLG<br>AIGSDGLSWMDVTVPEVSIYFQLFGHWSSIAYSIYVQRKKEINFQNLVKKLIAVVDDGRSYNGRREERTTSWRELSLCMA<br>ALNSSKYNVNRNDLQPLSSVAFADSTEQ	zf-BED--DUF-domain--Dimer_Tnp_hAT --	I
Prupe. 2G058 600.2	Pru Zf-BED 07_I	MTSFHYNEPEEKKKKKHASRLPRRVAANLQSRKISSGSEVSRSGAPCSASLRLATPCNASSRLPRAKRATRETADRLGR<br>FPAASLRRARRRARRHAFENTAQELSDTSPDMQYKDTQVSVPPRASDPGWAHGIMVNGGRQKIKCKYCHKVMLGGGIS<br>RLKQHLAGERGNVAPCEEVPEEVKVQIQQHLGFKVLEKLKKQKGLSNSKDSAPYLQYREGGENEDGMRTQKTVCTRGQ<br>RRRREKEAMECISNQMKKHKKRSFPAATTIVAQPLHQSFASQEIIAQADLAVARFMYESGIPFTAANSHFYQQMADAIAAA<br>GPGYKMPSYHALRGKLLNKSVQDAEEYEEELRKSWDVTGCSVMVDRWVDKTGNSVINFFVYCPKGTLFLKSADASDISE<br>CPDALLNLFDCVVQEVGHKRIVNFVTDASASCKAARKLLMEKYKTFFCSTCGGYGIELMLEEIGKMNEVKEVLAKAKKITQ<br>FIYNSAWMLNLVRRKTGGRDIVQLATTRFASTFLTLQNMVALKHRLEKIFASAAWMHSTFSKQKAGLELAEIIADQLFWSL<br>CDQILKVTKPLLSVFQLMDCEEKPSIGYVYDAMEKAKKSIIVSFDNKESDYVPYLEIIDHIWQEELHSPLHAAAYYLNPSIFY<br>NPSFSTNKVIQKGLLDCIETLEPNLTAQVVITSNINFYEEAVGDFGRPVALRCRESLAPATWWSLYAADYPDLQRLAVRILS<br>QTCSIIRYKRSRTMFERMCSKKKNRLEQQRFNHLAFVHYNLHLQHRRSEASKAIYTRGTLDPICLEAIDANMGDWVEDLG<br>AIGSDGLSWMDVTVPGEWKFLNHRVENMDDCNDSTDDRGSDDGRGVDTNDDM	zf-BED--DUF-domain--Dimer_Tnp_hAT --	I
Prupe. 3G090 000.1	Pru Zf-BED 08_I	MDIPTTPTDNNGLPSSETQTNRRRRKKSIVWEYFTIETVGAGSTKAFCKQCKRSFAYITGSKLAGTSHLKRHIALGICPVSR<br>QKNQMTPFTPGSKTAATDAPKRRSRANSGYARVSFDQDRCNHDIAKMIIMHGYPLHIAEQLGFINFVQTLQPQFNLVSWN<br>TVQNECVGIYLREKQNLLNLISGIPGKVSLTVDLWTSNQNLGYVFLTGHFIDDHWNLYRQILNVIMVPSPDSGDTFSQAILT<br>CLSDWHLEGRLFTLTLDQSLSNETIIGNLKGLLSVKNPHMLNSQLLLRNCYARVLSSLACDVLVAMRETIAKVRESVKFVKT<br>SESHEEKFVQLKQQLQVPSTKNLSVDDLTKWDTTYHMLVAACELREVFACLDTYDPDYNINILLEEWKQVETLCTYLKLLF<br>DAANIVTAPVYPSANVFFQEVSRIQMELMHAAMSVDPFVSYLMRPLYEKFDKYWENCCLVLAVAVIMDPRYKMKIVELEF<br>NRIYGENAETWIRIVDDGIHELFLDYMMQMLTLPETPMDEGNDSIIKTEAPEEASQEGSLLSSVDGLQDFELYISDITGGQQ<br>MKSELDQYLEEAFTDRVEDFDVLVWWRLNRMKYPTLSRMASDILSISVSTVASDSVFDTEIKRMDSYQTSLGPATLEALIC<br>AKDWLKYGSFPQPPAL	zf-BED--DUF-domain--Dimer_Tnp_hAT --	I
Prupe. 3G090 000.2	Pru Zf-BED 09_I	MDIPTTPTDNNGLPSSETQTNRRRRKKSIVWEYFTIETVGAGSTKAFCKQCKRSFAYITGSKLAGTSHLKRHIALGICPVSR<br>QKNQMTPFTPGSKTAATDAPKRRSRANSGYARVSFDQDRCNHDIAKMIIMHGYPLHIAEQLGFINFVQTLQPQFNLVSWN<br>TVQNECVGIYLREKQNLLNLISGIPGKVSLTVDLWTSNQNLGYVFLTGHFIDDHWNLYRQILNVIMVPSPDSGDTFSQAILT<br>CLSDWHLEGRLFTLTLDQSLSNETIIGNLKGLLSVKNPHMLNSQLLLRNCYARVLSSLACDVLVAMRETIAKVRESVKFVKT<br>SESHEEKFVQLKQQLQVPSTKNLSVDDLTKWDTTYHMLVAACELREVFACLDTYDPDYNINILLEEWKQVETLCTYLKLLF<br>DAANIVTAPVYPSANVFFQEVSRIQMELMHAAMSVDPFVSYLMRPLYEKFDKYWENCCLVLAVAVIMDPRYKMKIVELEF<br>NRIYGENAETWIRIVDDGIHELFLDYMMQMLTLPETPMDEGNDSIIKTEAPEEASQEGSLLSSVDGLQDFELYISDITGGQQ<br>MKSELDQYLEEAFTDRVEDFDVLVWWRLNRMKYPTLSRMASDILSISVSTVASDSVFDTEIKRMDSYQTSLGPATLEALIC<br>AKDWLKYGSFPQPPAL	zf-BED--DUF-domain--Dimer_Tnp_hAT --	I
Prupe. 3G090 000.3	Pru Zf-BED 10_I	MDIPTTPTDNNGLPSSETQTNRRRRKKSIVWEYFTIETVGAGSTKAFCKQCKRSFAYITGSKLAGTSHLKRHIALGICPVSR<br>QKNQMTPFTPGSKTAATDAPKRRSRANSGYARVSFDQDRCNHDIAKMIIMHGYPLHIAEQLGFINFVQTLQPQFNLVSWN<br>TVQNECVGIYLREKQNLLNLISGIPGKVSLTVDLWTSNQNLGYVFLTGHFIDDHWNLYRQILNVIMVPSPDSGDTFSQAILT<br>CLSDWHLEGRLFTLTLDQSLSNETIIGNLKGLLSVKNPHMLNSQLLLRNCYARVLSSLACDVLVAMRETIAKVRESVKFVKT<br>SESHEEKFVQLKQQLQVPSTKNLSVDDLTKWDTTYHMLVAACELREVFACLDTYDPDYNINILLEEWKQVETLCTYLKLLF<br>DAANIVTAPVYPSANVFFQEVSRIQMELMHAAMSVDPFVSYLMRPLYEKFDKYWENCCLVLAVAVIMDPRYKMKIVELEF<br>NRIYGENAETWIRIVDDGIHELFLDYMMQMLTLPETPMDEGNDSIIKTEAPEEASQEGSLLSSVDGLQDFELYISDITGGQQ<br>MKSELDQYLEEAFTDRVEDFDVLVWWRLNRMKYPTLSRMASDILSISVSTVASDSVFDTEIKRMDSYQTSLGPATLEALIC<br>AKDWLKYGSFPQPPAL	zf-BED--DUF-domain--Dimer_Tnp_hAT --	I
Prupe. 3G104 200.1	Pru Zf-BED 11_I I	MASSSGPSGASVPGGLDVAWKYARLIEGNKHGTICTFCESTFKSGGITRLKYHLARFDPHKSVKKCKEVPLDIKKEVIAWI<br>KEKESSKQHKKSAEDKHKIYNKRGIHGQ	zf-BED	II
Prupe. 3G104 400.1	Pru Zf-BED 12_I I	MEGESETPISTNPPPPTQVHQSTPVQSAPLPSTLGKRKPSKNASGVWEHFTKIKCEDDTSEPRCICKYCKKGYACGSKN<br>NGTSTLWHHLRSRCKNSPLRHEKKQKVLSFEGQGKGGNLVAKEWHIHFVAPFEQS	zf-BED	II
Prupe. 3G170 500.1	Pru Zf-BED 13_I	MDMSDAVIVKSTRLKSVVWNDFDRIKKGDKCIAVCRHCKKKLSGSSTSGTSHLRNHLIRCQRRSNLGIPQLFAAREKKKE<br>GTYLNLDQEQKKDEAFNLVNIRFEQEQTKDDIINYGSGNFDQRRSRFDLARMIILHGYPLDMVEHVGFRVFVKNLQPLFEL<br>VTSERVEADCMEIYGKEKQKVKDMLGKLPGKISLTVDMWASLDGTEYLCLTAHYIDESWQLNKKILNFIVIDSSHTEDKHS<br>EIIMESLMDWDIDRNLFSMTFDSYSTNDNVVFRIRDRLSQNKLLSCDGQLFDVRCAANVINMMSQDALEALCEMTDKIRG<br>SIRYVKSSQVIQEKFNSIVHQVGGESRRCLCLDNPLQWNSTYVMVEIALEYRDAFALLQENDPVYAMCPSDVEWDRVNIIT<br>SYLKLFVGVTNVFTRFKSPTANLYFPELCEVYSQLNEWCKNADDYISSLALKMRSKFEEYWMRCSLSLAVAVMLDPRFK<br>MKPVDYYYAQFFGSGAPGRISDVFECVKTLYNEHSTCLAYVDQGLAWQVGGSSRLPGSGRDLRDRLTGFDKFLHETTEI<br>DGTKSDLDKYLEEPLFPRNAEFDILNWWKVHAPRYPILSMMARNVLGIPVSKVPIDSTFNTGGRVLDRDWSSMNPATIQA<br>LMCAQDWIRSELES	zf-BED--DUF-domain--Dimer_Tnp_hAT --	I
Prupe. 3G186 900.1	Pru Zf-BED 14_I	MKFIRMISLEGFEPVAVSPHKQDPAWKHCQLFIKDQPNGVKAELKKCIYCGKVFQGGGINRLKSHLAGRKGNGPTCDQT<br>PPDVRLSMLQSLDHGIAAFRHRKSQIVTNSHHSPSELDNSFAENGECKLMVGPDSTSLVNQEEDVGMSGNGVGLSGNLT<br>NVPLEVDNSYRSNAAMHRSAGVPILNFSVNQEEDAGMSVDRRVRWRGGTSSADTGSGSVDGVTKLSNVSAPIPIAVGS<br>AFSEDTQGRSTGFPNSYWLENEEEVGISNTNVSARKRVRGESAVGAANAGTVDNNYEVKEVGNQQIHMAIGRFLYEIQA<br>PLDVVKNSVYFQPMIDAIASGGKGTIAPSYDDLRGWILKNAVGEVKSDIHQHMETWARTGCSLLVNQWSSEKGKTLLNFA<br>VQCPEGTIYLKSVDASYFIFSPDALFEFLKEVVEEVGVGHVLQVITNTEEQFAVAGKRLMDTFPTLYWSPCVATSIDLILED<br>FGKVEWINSVIEQARSVTRFIYKHVVILNMMRRYTFGNDIVRLGVTRFATNFTTLKQMADLKFNLQSMVTSKEWMCCPYS<br>KTPEGSAVLDVLSNHSFWSACILVTHLTNPLLRVLRIVGSQKRAAMGYVFAGIYRAKETIKRELVKREEYMVYWDIIDYRW<br>KKLWPLPLHAAGFYLNPKFFYSVKGDLHNEIISRMFDCIERLVPDIKIQDEVIKEINLYKNAVGDLGRNLAVRARDNLLPAE<br>WWSTYGSSCPNLARLAIRILSQTCSIVQGQENQIPFELLHKTRNSLECQRLSDLVFVQYNLKLKQKVHKHKEQENVGPISF<br>DRNSIVEDWVTEMEMPLEDNENPDWMSLDPPSGNTRLLELSVDEAEDLGSGFDDNEIFIRLKVVQGEC	zf-BED--DUF-domain--Dimer_Tnp_hAT --	I
Prupe. 3G186 900.2	Pru Zf-BED 15_I	MISLEGFEPVAVSPHKQDPAWKHCQLFIKDQPNGVKAELKKCIYCGKVFQGGGINRLKSHLAGRKGNGPTCDQTPPDVR<br>LSMLQSLDHGIAAFRHRKSQIVTNSHHSPSELDNSFAENGECKLMVGPDSTSLVNQEEDVGMSGNGVGLSGNLTNVPLE<br>VDNSYRSNAAMHRSAGVPILNFSVNQEEDAGMSVDRRVRWRGGTSSADTGSGSVDGVTKLSNVSAPIPIAVGSAFSED<br>TQGRSTGFPNSYWLENEEEVGISNTNVSARKRVRGESAVGAANAGTVDNNYEVKEVGNQQIHMAIGRFLYEIQAPLDVV<br>KNSVYFQPMIDAIASGGKGTIAPSYDDLRGWILKNAVGEVKSDIHQHMETWARTGCSLLVNQWSSEKGKTLLNFAVQCP<br>EGTIYLKSVDASYFIFSPDALFEFLKEVVEEVGVGHVLQVITNTEEQFAVAGKRLMDTFPTLYWSPCVATSIDLILEDFGKVE<br>WINSVIEQARSVTRFIYKHVVILNMMRRYTFGNDIVRLGVTRFATNFTTLKQMADLKFNLQSMVTSKEWMCCPYSKTPEG<br>SAVLDVLSNHSFWSACILVTHLTNPLLRVLRIVGSQKRAAMGYVFAGIYRAKETIKRELVKREEYMVYWDIIDYRWKKLWP<br>LPLHAAGFYLNPKFFYSVKGDLHNEIISRMFDCIERLVPDIKIQDEVIKEINLYKNAVGDLGRNLAVRARDNLLPAEWWSTY<br>GSSCPNLARLAIRILSQTCSIVQGQENQIPFELLHKTRNSLECQRLSDLVFVQYNLKLKQKVHKHKEQENVGPISFDRNSIV<br>EDWVTEMEMPLEDNENPDWMSLDPPSGNTRLLELSVDEAEDLGSGFDDNEIFIRLKVVQGEC	zf-BED--DUF-domain--Dimer_Tnp_hAT --	I

Prupe.<br>3G186<br>900.3	Pru<br>Zf-<br>BED<br>16_I	MISLEGFEPVAVSPHKQDPAWKHCQLFIKDQPNGVKAELKKCIYCGKVFQGGGINRLKSHLAGRKGNGPTCDQTPPDVR<br>LSMLQSLDHGIAAFRHRKSQIVTNSHHSPSELDNSFAENGECKLMVGPDSTSLVNQEEDVGMSGNGVGLSGNLTNVPLE<br>VDNSYRSNAAMHRSAGVPILNFSVNQEEDAGMSVDRRVRWRGGTSSADTGSGSVDGVTKLSNVSAPIPIAVGSAFSED<br>TQGRSTGFPNSYWLENEEEVGISNTNVSARKRVRGESAVGAANAGTVDNNYEVKEVGNQQIHMAIGRFLYEIQAPLDVV<br>KNSVYFQPMIDAIASGGKGTIAPSYDDLRGWILKNAVGEVKSDIHQHMETWARTGCSLLVNQWSSEKGKTLLNFAVQCP<br>EGTIYLKSVDASYFIFSPDALFEFLKEVVEEVGVGHVLQVITNTEEQFAVAGKRLMDTFPTLYWSPCVATSIDLILEDFGKVE<br>WINSVIEQARSVTRFIYKHVVILNMMRRYTFGNDIVRLGVTRFATNFTTLKQMADLKFNLQSMVTSKEWMCCPYSKTPEG<br>SAVLDVLSNHSFWSACILVTHLTNPLLRVLRIVGSQKRAAMGYVFAGIYRAKETIKRELVKREEYMVYWDIIDYRWKKLWP<br>LPLHAAGFYLNPKFFYSVKGDLHNEIISRMFDCIERLVPDIKIQDEVIKEINLYKNAVGDLGRNLAVRARDNLLPAEWWSTY<br>GSSCPNLARLAIRILSQTCSIVQGQENQIPFELLHKTRNSLECQRLSDLVFVQYNLKLKQKVHKHKEQENVGPISFDRNSIV<br>EDWVTEMEMPLEDNENPDWMSLDPPSGNTRLLELSVDEAEDLGSGFDDNEIFIRLKVVQGEC	zf-BED--DUF-<br>domain--<br>Dimer_Tnp_hAT<br>--	I
Prupe.<br>3G186<br>900.4	Pru<br>Zf-<br>BED<br>17_I	MISLEGFEPVAVSPHKQDPAWKHCQLFIKDQPNGVKAELKKCIYCGKVFQGGGINRLKSHLAGRKGNGPTCDQTPPDVR<br>LSMLQSLDHGIAAFRHRKSQIVTNSHHSPSELDNSFAENGECKLMVGPDSTSLVNQEEDVGMSGNGVGLSGNLTNVPLE<br>VDNSYRSNAAMHRSAGVPILNFSVNQEEDAGMSVDRRVRWRGGTSSADTGSGSVDGVTKLSNVSAPIPIAVGSAFSED<br>TQGRSTGFPNSYWLENEEEVGISNTNVSARKRVRGESAVGAANAGTVDNNYEVKEVGNQQIHMAIGRFLYEIQAPLDVV<br>KNSVYFQPMIDAIASGGKGTIAPSYDDLRGWILKNAVGEVKSDIHQHMETWARTGCSLLVNQWSSEKGKTLLNFAVQCP<br>EGTIYLKSVDASYFIFSPDALFEFLKEVVEEVGVGHVLQVITNTEEQFAVAGKRLMDTFPTLYWSPCVATSIDLILEDFGKVE<br>WINSVIEQARSVTRFIYKHVVILNMMRRYTFGNDIVRLGVTRFATNFTTLKQMADLKFNLQSMVTSKEWMCCPYSKTPEG<br>SAVLDVLSNHSFWSACILVTHLTNPLLRVLRIVGSQKRAAMGYVFAGIYRAKETIKRELVKREEYMVYWDIIDYRWKKLWP<br>LPLHAAGFYLNPKFFYSVKGDLHNEIISRMFDCIERLVPDIKIQDEVIKEINLYKNAVGDLGRNLAVRARDNLLPAEWWSTY<br>GSSCPNLARLAIRILSQTCSIVQGQENQIPFELLHKTRNSLECQRLSDLVFVQYNLKLKQKVHKHKEQENVGPISFDRNSIV<br>EDWVTEMEMPLEDNENPDWMSLDPPSGNTRLLELSVDEAEDLGSGFDDNEIFIRLKVVQGEC	zf-BED--DUF-<br>domain--<br>Dimer_Tnp_hAT<br>--	I
Prupe.<br>3G186<br>900.5	Pru<br>Zf-<br>BED<br>18_I	MKFIRMISLEGFEPVAVSPHKQDPAWKHCQLFIKDQPNGVKAELKKCIYCGKVFQGGGINRLKSHLAGRKGNGPTCDQT<br>PPDVRLSMLQSLDHGIAAFRHRKSQIVTNSHHSPSELDNSFAENGECKLMVGPDSTSLVNQEEDVGMSGNGVGLSGNLT<br>NVPLEVDNSYRSNAAMHRSAGVPILNFSVNQEEDAGMSVDRRVRWRGGTSSADTGSGSVDGVTKLSNVSAPIPIAVGS<br>AFSEDTQGRSTGFPNSYWLENEEEVGISNTNVSARKRVRGESAVGAANAGTVDNNYEVKEVGNQQIHMAIGRFLYEIQA<br>PLDVVKNSVYFQPMIDAIASGGKGTIAPSYDDLRGWILKNAVGEVKSDIHQHMETWARTGCSLLVNQWSSEKGKTLLNFA<br>VQCPEGTIYLKSVDASYFIFSPDALFEFLKEVVEEVGVGHVLQVITNTEEQFAVAGKRLMDTFPTLYWSPCVATSIDLILED<br>FGKVEWINSVIEQARSVTRFIYKHVVILNMMRRYTFGNDIVRLGVTRFATNFTTLKQMADLKFNLQSMVTSKEWMCCPYS<br>KTPEGSAVLDVLSNHSFWSACILVTHLTNPLLRVLRIVGSQKRAAMGYVFAGIYRAKETIKRELVKREEYMVYWDIIDYRW<br>KKLWPLPLHAAGFYLNPKFFYSVKGDLHNEIISRMFDCIERLVPDIKIQDEVIKEINLYKNAVGDLGRNLAVRARDNLLPAE<br>WWSTYGSSCPNLARLAIRILSQTCSIVQGQENQIPFELLHKTRNSLECQRLSDLVFVQYNLKLKQKVHKHKEQENVGPISF<br>DRNSIVEDWVTEMEMPLEDNENPDWMSLDPPSGNTRLLELSVDEAEDLGSGFDDNEIFIRLKVVQGEC	zf-BED--DUF-<br>domain--<br>Dimer_Tnp_hAT<br>--	I
Prupe.<br>3G186<br>900.6	Pru<br>Zf-<br>BED<br>19_I	MKFIRMISLEGFEPVAVSPHKQDPAWKHCQLFIKDQPNGVKAELKKCIYCGKVFQGGGINRLKSHLAGRKGNGPTCDQT<br>PPDVRLSMLQSLDHGIAAFRHRKSQIVTNSHHSPSELDNSFAENGECKLMVGPDSTSLVNQEEDVGMSGNGVGLSGNLT<br>NVPLEVDNSYRSNAAMHRSAGVPILNFSVNQEEDAGMSVDRRVRWRGGTSSADTGSGSVDGVTKLSNVSAPIPIAVGS<br>AFSEDTQGRSTGFPNSYWLENEEEVGISNTNVSARKRVRGESAVGAANAGTVDNNYEVKEVGNQQIHMAIGRFLYEIQA<br>PLDVVKNSVYFQPMIDAIASGGKGTIAPSYDDLRGWILKNAVGEVKSDIHQHMETWARTGCSLLVNQWSSEKGKTLLNFA<br>VQCPEGTIYLKSVDASYFIFSPDALFEFLKEVVEEVGVGHVLQVITNTEEQFAVAGKRLMDTFPTLYWSPCVATSIDLILED<br>FGKVEWINSVIEQARSVTRFIYKHVVILNMMRRYTFGNDIVRLGVTRFATNFTTLKQMADLKFNLQSMVTSKEWMCCPYS<br>KTPEGSAVLDVLSNHSFWSACILVTHLTNPLLRVLRIVGSQKRAAMGYVFAGIYRAKETIKRELVKREEYMVYWDIIDYRW<br>KKLWPLPLHAAGFYLNPKFFYSVKGDLHNEIISRMFDCIERLVPDIKIQDEVIKEINLYKNAVGDLGRNLAVRARDNLLPAE<br>WWSTYGSSCPNLARLAIRILSQTCSIVQGQENQIPFELLHKTRNSLECQRLSDLVFVQYNLKLKQKVHKHKEQENVGPISF<br>DRNSIVEDWVTEMEMPLEDNENPDWMSLDPPSGNTRLLELSVDEAEDLGSGFDDNEIFIRLKVVQGEC	zf-BED--DUF-<br>domain--<br>Dimer_Tnp_hAT<br>--	I
Prupe.<br>3G186<br>900.7	Pru<br>Zf-<br>BED<br>20_I<br>V	MKFIRMISLEGFEPVAVSPHKQDPAWKHCQLFIKDQPNGVKAELKKCIYCGKVFQGGGINRLKSHLAGRKGNGPTCDQT<br>PPDVRLSMLQSLDHGIAAFRHRKSQIVTNSHHSPSELDNSFAENGECKLMVGPDSTSLVNQEEDVGMSGNGVGLSGNLT<br>NVPLEVDNSYRSNAAMHRSAGVPILNFSVNQEEDAGMSVDRRVRWRGGTSSADTGSGSVDGVTKLSNVSAPIPIAVGS<br>AFSEDTQGRSTGFPNSYWLENEEEVGISNTNVSARKRVRGESAVGAANAGTVDNNYEVKEVGNQQIHMAIGRFLYEIQA<br>PLDVVKNSVYFQPMIDAIASGGKGTIAPSYDDLRGWILKNAVGEVKSDIHQHMETWARTGCSLLVNQWSSEKGKTLLNFA<br>VQCPEGTIYLKSVDASYFIFSPDALFEFLKEVVEEVGVGHVLQVITNTEEQFAVAGKRLMDTFPTLYWSPCVATSIDLILED<br>FGKVEWINSVIEQARSVTRFIYKHVVILNMMRRYTFGNDIVRLGVTRFATNFTTLKQMADLKFNLQSMVTSKEWMCCPYS<br>KTPEGSAVLDVLSNHSFWSACILVTHLTNPLLRVLRIVGSQKRAAMGYVFAGIYRAKETIKRELVKREEYMVYWDIIDYRW<br>KKLWPLPLHAAGFYLNPKFFYSVKGDLHNEIISRMFDCIERLVPDIKIQDEVIKEINLYKNAVGDLGRNLAVRARDNLLPGFT<br>STKNRKMWVLYHLTAIALSKTG	zf-BED--DUF-<br>domain	I<br>V
Prupe.<br>3G186<br>900.8	Pru<br>Zf-<br>BED<br>21_I	MISLEGFEPVAVSPHKQDPAWKHCQLFIKDQPNGVKAELKKCIYCGKVFQGGGINRLKSHLAGRKGNGPTCDQTPPDVR<br>LSMLQSLDHGIAAFRHRKSQIVTNSHHSPSELDNSFAENGECKLMVGPDSTSLVNQEEDVGMSGNGVGLSGNLTNVPLE<br>VDNSYRSNAAMHRSAGVPILNFSVNQEEDAGMSVDRRVRWRGGTSSADTGSGSVDGVTKLSNVSAPIPIAVGSAFSED<br>TQGRSTGFPNSYWLENEEEVGISNTNVSARKRVRGESAVGAANAGTVDNNYEVKEVGNQQIHMAIGRFLYEIQAPLDVV<br>KNSVYFQPMIDAIASGGKGTIAPSYDDLRGWILKNAVGEVKSDIHQHMETWARTGCSLLVNQWSSEKGKTLLNFAVQCP<br>EGTIYLKSVDASYFIFSPDALFEFLKEVVEEVGVGHVLQVITNTEEQFAVAGKRLMDTFPTLYWSPCVATSIDLILEDFGKVE<br>WINSVIEQARSVTRFIYKHVVILNMMRRYTFGNDIVRLGVTRFATNFTTLKQMADLKFNLQSMVTSKEWMCCPYSKTPEG<br>SAVLDVLSNHSFWSACILVTHLTNPLLRVLRIVGSQKRAAMGYVFAGIYRAKETIKRELVKREEYMVYWDIIDYRWKKLWP<br>LPLHAAGFYLNPKFFYSVKGDLHNEIISRMFDCIERLVPDIKIQDEVIKEINLYKNAVGDLGRNLAVRARDNLLPAEWWSTY<br>GSSCPNLARLAIRILSQTCSIVQGQENQIPFELLHKTRNSLECQRLSDLVFVQYNLKLKQKVHKHKEQENVGPISFDRNSIV<br>EDWVTEMEMPLEDNENPDWMSLDPPSGNTRLLELSVDEAEDLGSGFDDNEIFIRLKVVQGEC	zf-BED--DUF-<br>domain--<br>Dimer_Tnp_hAT<br>--	I
Prupe.<br>3G186<br>900.9	Pru<br>Zf-<br>BED<br>22_I	MISLEGFEPVAVSPHKQDPAWKHCQLFIKDQPNGVKAELKKCIYCGKVFQGGGINRLKSHLAGRKGNGPTCDQTPPDVR<br>LSMLQSLDHGIAAFRHRKSQIVTNSHHSPSELDNSFAENGECKLMVGPDSTSLVNQEEDVGMSGNGVGLSGNLTNVPLE<br>VDNSYRSNAAMHRSAGVPILNFSVNQEEDAGMSVDRRVRWRGGTSSADTGSGSVDGVTKLSNVSAPIPIAVGSAFSED<br>TQGRSTGFPNSYWLENEEEVGISNTNVSARKRVRGESAVGAANAGTVDNNYEVKEVGNQQIHMAIGRFLYEIQAPLDVV<br>KNSVYFQPMIDAIASGGKGTIAPSYDDLRGWILKNAVGEVKSDIHQHMETWARTGCSLLVNQWSSEKGKTLLNFAVQCP<br>EGTIYLKSVDASYFIFSPDALFEFLKEVVEEVGVGHVLQVITNTEEQFAVAGKRLMDTFPTLYWSPCVATSIDLILEDFGKVE<br>WINSVIEQARSVTRFIYKHVVILNMMRRYTFGNDIVRLGVTRFATNFTTLKQMADLKFNLQSMVTSKEWMCCPYSKTPEG<br>SAVLDVLSNHSFWSACILVTHLTNPLLRVLRIVGSQKRAAMGYVFAGIYRAKETIKRELVKREEYMVYWDIIDYRWKKLWP<br>LPLHAAGFYLNPKFFYSVKGDLHNEIISRMFDCIERLVPDIKIQDEVIKEINLYKNAVGDLGRNLAVRARDNLLPAEWWSTY<br>GSSCPNLARLAIRILSQTCSIVQGQENQIPFELLHKTRNSLECQRLSDLVFVQYNLKLKQKVHKHKEQENVGPISFDRNSIV<br>EDWVTEMEMPLEDNENPDWMSLDPPSGNTRLLELSVDEAEDLGSGFDDNEIFIRLKVVQGEC	zf-BED--DUF-<br>domain--<br>Dimer_Tnp_hAT<br>--	I
Prupe.<br>4G156<br>200.1	Pru<br>Zf-<br>BED<br>23_I	MEIPIESAIKKPKRLTSIVWNHFERVRKADICYAVCVHCNKKLSGSSNSGTTHLRNHLMRCLKRSNFDVSQLLAAKRRKKD<br>NTVGLANINCDEAQRKDEYMKPALIKFDQDLKKDDIVTIASGKFDNDRSRLDLARMIILHGYPLTMVDHVGFKVFVKNLQPL<br>FEVVPNNDVEHFCMEIYRKEKRQVYQAINSLQGRINLSVEMWSSPENVEYLCLTAHYIDEDWKLQKKVLNFVTLDPTHTE<br>DSLSEVISKCLMDWDIHSKLFAFTLDDCSTDDDIVLRIKDRISQSRPLAGHGQLFDIRSAAHLLNSIVQDVLEALREVIQKIRG<br>SFKHVRSSQVVQGKFNEIAQQVGINSERRLILDFPVRWNSTYIMLETALEYRGAFSLLQEHDPSYASSLTDTEWEWTSFV<br>TGYLKLLVEITNVFSGNKSPTASIYFPEICHVHIQLIEWCKSPDDFLSCMALKMKAKFDKYWSKCSLALAVAAILDPRFKMK<br>LVEYYYSQIYGSTALDRIKEVSDGIKELFDAYSICSTMVDQGSALPGSSLPSTSSDTRDRLKGFDKFLYETSQSQNVISDLD<br>KYLEEPVFPRNCDFNILNWWKVHTPRYPILSMMARDVLGTPMSTVAPESAFSIGGRVLDQCRSSLNPDIRQALVCTQDW<br>LQVELKDVNPFSSHSAARPLLIESS	zf-BED--DUF-<br>domain--<br>Dimer_Tnp_hAT<br>--	I
Prupe.<br>5G216<br>200.1	Pru<br>Zf-<br>BED<br>24_I	MDWGANNAFKTFKDVEPKSMMDMGLIPTIDSVDIGLSSSEQGNATPSAKPRKKTMTSVYLKFFETAADGKSRRCKFCGQ<br>SYSIATATGNLGRHLSNRHPGYDKSGDVVTSSAPPPITVVRKHQPQSKAPQVDYNHLNWLLVKWLVLASLPPATLEEKW<br>LANSYKFLNPSIQLWSSEEYRKTFHEVFRSMKEVVRASLEHVSSKVSITLEFWTSYEEIYYMSVTCHWIDENWSFQKMML<br>DICHIPYPCGGAEIYHSLVKVLRLYNIENRVLSCTHDNSQSSMHGYVDGQKVGPFCYIPCSAHVLNLIIDDGLRTTKPLISKI<br>REFAIGLNASSEMSEDFTQFTAAYQESTWKMPLDTSTRWSGNYQMLDIVCKASKSMDAVIRKYETLGSRMLLSSAEKNA<br>VSNVHRYLQPFYKTTNNMCTNKLPTVGLVLFFMDHISETIAACRDSHLHPDLLKNAAKEMAEKVRGYNNQVCNIIIYMTAV<br>LDPRIKGELIPESLNAENFLDEARTHFIRNYSTSHFPSMTSGYSAQELEEGCNVSFAEEIARKKRRANMSSATDELTQYLS	zf-BED--DUF-<br>domain--<br>Dimer_Tnp_hAT<br>--	I

		EPPAPIATDVLEWWKVNSMRYPRLSLMARDFLAVQAVSVAPEELFCGKGDEIYKQRFCMPHDSTQALLCIRSWLQGGMK<br>LKYKTTEIDFERLMELATTAATADNTTPGSEKKQKY		
Prupe.5G216200.2	Pru Zf-BED25_I	MDWGANNAFKTFKDVEPKSMMDMGLIPTIDSVDIGLSSSEQGNATPSAKPRKKTMTSVYLKFFETAADGKSRRCKFCGQ<br>SYSIATATGNLGRHLSNRHPGYDKSGDVVTSSAPPPITVVRKHQPQSKAPQVDYNHLNWLLVKWLVLASLPPATLEEKW<br>LANSYKFLNPSIQLWSSEEYRKTFHEVFRSMKEVVRASLEHVSSKVSITLEFWTSYEEIYYMSVTCHWIDENWSFQKMML<br>DICHIPYPCGGAEIYHSLVKVLRLYNIENRVLSCTHDNSQSSMHGYVDGQKVGPFCYIPCSAHVLNLIIDDGLRTTKPLISKI<br>REFAIGLNASSEMSEDFTQFTAAYQESTWKMPLDTSTRWSGNYQMLDIVCKASKSMDAVIRKYETLGSRMLLSSAEKNA<br>VSNVHRYLQPFYKTTNNMCTNKLPTVGLVLFFMDHISETIAACRDSHLHPDLLKNAAKEMAEKVRGYNNQVCNIIIYMTAV<br>LDPRIKGELIPESLNAENFLDEARTHFIRNYSTSHFPSMTSGYSAQELEEGCNVSFAEEIARKKRRANMSSATDELTQYLS<br>EPPAPIATDVLEWWKVNSMRYPRLSLMARDFLAVQAVSVAPEELFCGKGDEIYKQRFCMPHDSTQALLCIRSWLQGGMK<br>LKYKTTEIDFERLMELATTAATADNTTPGSEKKQKY	zf-BED--DUF-domain--Dimer_Tnp_hAT--	I
Prupe.8G039800.1	Pru Zf-BED26_IV	MDNASGTVNEPANDSTTPSNNKPASAPRLTSKVWSYFKRVNKIENGKLVLKAECVICNTLFAGSPRSGTSHLSRHLKEHE<br>AAQARISPGHILLNADVAVNLTNFAYDHAVARKEVEDCIIRAELPFKFVESHDFTASTCKGDVMKKFGKEKSDLHALFEKLD<br>SKICLTSDVWSSQQKMGYMSLTAHFIDKDWFLNKRVICFKMIEYPHTGESLATHIFDELLSWRIHNKIFTLSLDNASNNDTA<br>ASILPSSLLLDSVQQKLFHVRCCCHILNLIVQDGLKVLSPSIDEITDIVRSMNSSSKRHEIWINIDNDVPHRWNSTYELLLVAI<br>KYKVPLHWYVKEINESISCNLQVPNEEDWKIAQLVSGFLQIFYSSTKILSGVYYPTSSCVISCLTDIHASFAAYSEFSVFKDA<br>LTEMKAKFDKYWEEFPTVFCFATIMDPRFKVFAIGEWLNMIGIDQLTIDSKLLALKTLLFQFFDVYKRSVMSYVGQNVQSA<br>NVTSSTLSQTPGSIVVPIVDYCLKPIVKSDGY	zf-BED--DUF-domain	IV
Prupe.8G075500.1	Pru Zf-BED27_IV	MKQVTTPTSTSKGNPTGNRSQPRTDAPSTSNGLDTRKPARPPSIVWENFIKMQDDPKNPKAKCKYCNKVYAYGSKKNG<br>TSNLLSHLANQCKEYPGRDIKKQKTLSFQPKKGEEEGKLIATSYSPESCREAIVRFIILDEQPFKVVEGEGFRDMLRVFEPR<br>LQVPSCVTIAKDVLKIYKKEKIKLKDYLTIMSQSPARSQKFKACVEQVKISSHKAICLDVPTRWNSTYLMLEKINMYLFVAVL<br>LDPCYKKRYSQYYFSLLCGEDKASEVTSKVRSKLNELYDQYKLLYNENAAYKDETHNPSEMEIDSNEVDFATAFTIGFMK<br>LVEKPDGEESKTEVDSTGGRILDLFRSSLSSRTVEALICTQNWLHSSPKTDVLKVVDEMEEIASGKYYFKISFIVEIN	zf-BED--DUF-domain	IV
Sme2.5_00456.1_g00001.1	Sme Zf-BED01_XXII	MNSLAISYGRNPRSDSLPEPEKLIGVEIPIWVLLRIKFRYLGFPQNSKFCYRTILFNFGSTVNSAWLGRVELVLVEVGSPEP<br>YRQRVPVRLNGTLGCDRRVMCMQTYVYLRVPPPTRAPVGFRHATLFSYWENPSTLFRIQETTLRDLFSFGKIHERNNQPI<br>DIGRLNGFFHCIISGSNGMHRRIDGAIRVRSNVDPTFDSLVGSGRSGGDHHGSSLLENPYIPYQCMDSYLSSTALSFWTV<br>MVTLETPATPATPTENHEIVPKNEMTHEHSHEMVPENEIINEHSHEMVLENEIINEHSHEMVPENEIINEHSHEMVPENEM<br>THEQSHEVVPENEMTHEQSHDVVPENEMTHEQSHEVVPENEMTDEQSHEVVPENEMTHEQNHEVVPENEMTHEHSHE<br>MVPENEMTHEQSHEVVLENEMTHEHSHEMVPENEMTHEQSHAVVPENEMTHEHSHEMVPENEMTHEHSHEMVPENE<br>MTHELSHEMVPENEMTHGHSHEMVPENEMTHEHSHEMVPENEMTHEHNHEMVPEHVMMHEHSHEMVPENEMMHEH<br>SHEMVPENEMVHEHHLVLGNEIVPSNEMVSDSEMIPLNEMVLAEPQPNYIETPPNNPETQPSKRRKKKSIVWEHFTIENV<br>GGGTRRAQCKQCKQSFAYSTGSKVAGTSHLKRHIAKGTCPVVLRQQQNTQLSPYSTPPKMSGYGGSTDAPKRRYRTAS<br>APYLAFDSDRCRQEISRMIIMHDYPLHMVEHPGFLAFVQNLQPRFDMVSFNTVQGDCVATYLREKQAIQKVIEGVPGCICL<br>TLDMWSSCYTVGYVFITGQYIDSEWKIHRKILNIIMEPYPDSDTAFSHAVAACLSDWSMEGKLFSVTINQPLGDAAADNLR<br>ALLSVKNPLVLNGQLLVGSCLARSLSSIALGVFKLLHGTVKKVRDSVKYVKTSESHEEKFIELKQQLQVPSTKTLALDDQTQ<br>WNTTYEMLLAASELKEVFSCLDTCDPDYKDAPSMEDWKQVEVLCTYLKILFDTANLLTAPTIPTTNTFFHAWKIQLELARG<br>AASEDPSISSLTKIMQEEFDKYWKSSCLILAIAVVMDPRFKMKLVEFSFTKIYGEEASTYVKIVEEGIHELFLEYVALPLPLTP<br>TYAEETTSQQSRSELDQYLDESLLPRVHEFDVVGWWKLNRMKYPTLSKMARDILSVPVSTVTADSVFSTVGKEMDRYRC<br>SLRPETVEALICAKDWLQNASAHFIFVLGPMRFRVVKSTIDTCHGARCYDDLMRKMCM	DUF2647--zf-BED--DUF-domain--Dimer_Tnp_hAT--	XXII
Sme2.5_00690.1_g00003.1	Sme Zf-BED02_I	MVRGRDACWEHCVLVDATRQKVRCNYCRREFSGGVYRMKFHLAQIKNKDVVTCGEVPNEVRDHIRTILNSPKKQKNPK<br>KAKPDQAANGQESSSSSASSRIRPPHDGFSGQNGNPCPPATMFACRSSSSQTAVDEVQKQKQDHADKKIAEFFYHNGI<br>PFSAAKSLYYQEMVDAILECEAGYKAPCTEKLGTNLLEKVKVDINDGFKRLRDEWKETGCSILCDSWSDGRDKCLVVISV<br>TCAKGTMFLRSVDVSGHANDPCCLGNLLDKVILEIGVENVVQVITEISDRFIYTGRLIMQMYPSVFWSPCASDCINKMLEDF<br>SKHDWVNAVLKEANMIIKYIYSNDWILDMMRKFSGGEEFVLVRPRITKFVAIFLFLRALVIQEDNLKHMFSHEEWLSSTYSR<br>HPEAQAIKSLLCLERFWRSAREAVAVSEPLLKLLRIVDGDMPAMAYMYEGVERAKISIKAFYKNVDEKFMPIWDIIDRRWS<br>MCLQTPLHAAAAFLNPSIFYNSSFKIDARIRNGFQEVMTSMASEDKDKVEITKEYPIYLNAQGALGTEFAIKGRTLNAPGVC<br>NFIAHVSSELRIFDDYIGYVIVVQIVIAFKSLPYVSSADWWTGYGYEIPTLQRAAIRILSQPCSLHWCRWNRSTFDGVYDKK<br>REGLELEKFCDLLYVRCNLWLRATISKDGKCKPINFDEIDVDAEWPTETEVPPCAHLDDSWLHTSPLEGKGIPSFSEFIYK	zf-BED--DUF-domain--Dimer_Tnp_hAT--	I
Sme2.5_01715.1_g00010.1	Sme Zf-BED03_II	METTSSASDTEVIINIPPTMPVTSQQGDKVTPHRCTPPKKQKRTTPSVFSKFENTGRETSEIWNHFSKFSAKCKAKCNYC<br>TKTYDAGSVNGTTTLWNHLNVKYHNSPFRFVAKRQTTLKPIKRGLLEDDLGSPARVIYNVEEIRRAIAEFVIIDEQPFRVVE<br>GEGFKKLKAKVLPNFEVPFRVTVARDCLRIYQKEKKKA	zf-BED	II
Sme2.5_01880.1_g00003.1	Sme Zf-BED04_II	MGFQDEQNQMCSFSNVVPIEIDGQMSSQANEDTNEVIEIEQGRFTSKAWQYFKPVKVDGVRYSVFKYCGGCVKSTRNC<br>GTKSMFEHIRRFCKKPSDLEIGDGSRTTGCSTPHYFD	zf-BED	II
Sme2.5_01985.1_g00003.1	Sme Zf-BED05_V	MEANETSCEMSSYISRKRAKRNTSIVWNTFSKLPRTSDSERLKAECQVCHEILVVDVGTSNMKCHVERHLDNIEERGYAP<br>SDQVIYREKLSVAILKLISFCYAPSDQVIYREKLSVAILKHNYPFSFDEYQGNRDIHLYLNPMCQMVMCLTNASCNNGVVD<br>HLKEHLSLMHSLVCDGKFFHVHCGNHILNLIVKAGLEKADEAIGKVREGENISDTSNNEIRDDLKEFDVFENQLESSETKTQ<br>LDLYLEEPKLDRKANPNLDVLAYWKENRGRYPELSLMARDVLSIPITTVASESSFSIGGRIIGKFQSSILPANAEAKLCVRD<br>WLCGQEDCDGSDTDEDEIAVELPPYLAELGSH	zf-BED--Dimer_Tnp_hAT--	V
Sme2.5_02480.1_g00019.1	Sme Zf-BED06_I	MNLEPVPVTSQKHDPAWKHCEMFKNGERVQLKCIYCGKIFKGGGIHRIKEHLAGQKGNASTCLRVQPEVRLLMQESLNG<br>VVMKKRKKQKLAEEITTYNAGTSDIAAFTDTCEVDLLPMPEAIEQTSNLFLNQEEGGNKTLGRKKKSRIRKPSSSNNNAML<br>LAINQSKRVNNHVHMAIARFLLDARVPLDAVNSVYFQPMIDVIASQGTQVVGPSYHDLRSWVLKASVQEVRNDIDQCSST<br>WARTGCSVLVDEWITGKGITLVNFLVYCPEGTMFLRSVDASSLINSMDSLYELLKEVVEEVGPRNVLQVVTSNEERYVIAG<br>KRLTDAYPTLFWTPCAAHSIDLMLEDIKKLEWIDTIMEQAKSISRFIYNNNVLLSMMRKFTLGVDLVDLGVTRSATDFLTLKR<br>MVNIKHNLQSMVTSVEWMDCPYSKRPEGFALLDYISNQSFWSTCSLISRLADPILRLLRIVCSEERPAMAYIYAGVYRAKE<br>TIKKELVDKKDYSVYWNIIDHRWESLQRHPLHAAGFYLNPKFFYTTEEEVHLHIRSIVYDCIEKLVPDPKVQDKIVKETTSYH<br>NSAGDFGRKMAVRARDTLFPAEWWSTYGGGCPNLARLAIRILSQTSSLIRSKPGRIPLEEMHETKNCIEHQRLNDLAFVQ<br>YNLWLRQRKNMEPDCMDSISYDKKELVHNWVSRKEQISEDMESSDWMTVDPPLGSIAPLGPLIDDIEALGAGFDDFEIFG<br>GPKDSEEEIGEENAVNE	zf-BED--DUF-domain--Dimer_Tnp_hAT--	I
Sme2.5_03304.1_g00001.1	Sme Zf-BED07_II	MLSGDIFTRNNLAILTKASIALPDDNFLSDGNYIFAPDMEVIANIPPTMPAISQQGDEVTLYQGTPKKQKRTTPLVPSKIGNT<br>GGETSEIWNHFTKFTVKGGQCRATCNYCPKTYAAGSANGTTTLWKHLNAKCPKSPFRVVDKRQFTFKPTKGGLLEVGL<br>GSPAKVIYNVEEIRKAIVEFVIIDEQPFRVV	zf-BED	II
Sme2.5_03433.1_g00003.1	Sme Zf-BED08_IV	MGEMAQDKLDVWQHGVAVDQKKKKIKCNYCSKVVSGSTRLRYHLGGIGGGVIPCVNAPTLVKEALKAEVLEKENGKLRE<br>EVGQLNHANLPLKRNWCPQDGDGDGEPTKTDISRSSESANKNHNGVNSKLAGSCVVNLSSQEISKSVGRFFYETGIDFD<br>AIRSPSFQRMVKPTLNPGQTIKFPSCQELKGWILQDAVKEMQQYVKDIRNSWANTGCSILLDGWVDSNGRNLINILVYCP<br>RGTIYLRSSNISYFNGNVDAMLLFFEEVGVETVVQIVAYSTSACMMEAGKKLMEKHRTVFWTVDASHFMELMLQELTKID<br>PIQEVLDKAKTLTQFIYSHATVLKLLRDVYPDELVKSSKIRSIVPFLTLENIVSQKEWFTRMFQSSDWQSSILASTGEGKRM<br>SEMVEGQSFWTEALMAVKATIPLHFGGHNMEVSDESDGEAEAAGLGIWPL	zf-BED--DUF-domain	IV
Sme2.5_03433.1_g	Sme Zf-BED09_I	MTRDKIDIHQHGVAVDQKKSKIKCNYCGKVVSGFSRLKHHLGGIRGDVTPCLEAPVPVKKALEAEILGKKNENLIKEVGQL<br>QHPNLPLKRNWCHRDGEPNNTNVTRTSESVNKKHNGVNSNVAGSSSSQEISKSIGRFFYEAGIDFDAIRSPSFQRMVKA<br>TLSPGQTIKFPSCQELNGWILEDAVKEMQQYVTEIRNSWASTGCSILLDGWVDSNGRNLINILVYCPRGTIYLRSSDISYFN<br>GNVDAMLLFYEEVLEEVGVETVVQIVAYSTSACMMEAGKKLMDKRKTVFWTVDASHCMELMLQELIKIDWIPEVLEKAKA<br>LVQFIYSHASVLKLLRDAFPGELVKSSKIRSIVPFLTLENIVSQKDGLIRMFESSDWQTSILASTSEGKRMSRIVKDQSFWTE	zf-BED--DUF-domain--Dimer_Tnp_hAT--	I

00004.1		ALMAVKATIPLVEVIKLLNGTNKPLVGYIYDTLDQAKETIKKELEDKKSLYAKFWKAIDDIWDEYLHSYIHAAGYFLNPILFYS<br>SDFYTDVEVSCGLCGCVVRMTEDRHVQDLITLQIDDYRMGRGMFHFGSFREILSDTSPALWWSQYGVEHPELQRLAIRIL<br>SQTCDGASHYRLKRSLIETLLAEGRNQIEQQRLRDLVFVHCNLQLQAFDPQGINDIADDVVDPMDEWIVGKEPNLVSHLT<br>WMDLSRNRNEKVCIYVKEEEEGG		
Sme2.5_05641.1_g00001.1	SmeZf-BED13_XXII	GTEIWRIEDFQPVPLPKSECGKFYSGDSYIILQTTSGKGGPYIYDIHFWLGKDTSQDEAGTAAIKTVELDAILGGRAVQHRE<br>IQGHESDKFLSYFKPCIIPLEGGIASGFKKPEEDEFETRLYVCKGKRVVRMKQVPFSRSSLNHDDVFILDSKDKIYQFNGAN<br>SNIQERAKALEVIQFLKDKYHEGTCDVAIVDDGNLQAETDSGSFWVLFGGFAPIGKKDVSEDDIVPDKTPAKLFSITDGQV<br>SLVDGELSKSSLENNKCYLLDCGAEVFVWVGRVTQLEERKAATQAAEEYLSNQNRPKSTHVTRLIQGYETHSFKSNFDS<br>WPSGSAPAAEEGRGKVAALLKQQGIGVKGASKSAPVNEEIPPLLEKGGKIEVWRINGNAKTLVPRDDIGKFHSGDCYIILY<br>TYHSNDRKEDYYLCWWIGKDSVEEDQNMAAKLASTMCNSLKGRPVLGRVYQGKEPPQFVAIFQPMLVLKGGLSAGYKN<br>YIADKGLNDETYTADSVALIRLSGTSVHNNKAVQVDAVATSLNSNECFLLQSGSSVFSWHGNQSTFEQHQLAAKVAEFLK<br>PGATVKHTKEGTESSSFWFGLGGKQSYTSKKVAVEIEEIYNFSQDDLLTEDVLLLDTRAEVFVWVGQSSDPKEKQSSLDI<br>GQKYIEMAACLEGLSPNVPLYKVTEGNEPCFFTTFFSWDPAKAIAYGNSFQKKVKLLFGVGHASENQPRSNGTNQGGST<br>QRASALAALNYAFNSPSPKKASSSPRSGKSPGSQRAAAVAALSSVLSAEKKQSSEGSSPLRLSRSSSVDPIPLVNGVTTT<br>EAIGSKEIPELKETAIVGHAETNGEDIGPKPEPEQEETGSDGSQTTFSYERLKAKSKNPVTRIDFKKREYNVKSVDENQQS<br>DLVRRRRPKNAVDPDSDLLKPELSRDLGFAQFRGFRMATPETPVTPANPTENHEMVHEHEMIPEHEMVHEHEMSHEHE<br>MGHEHEMSHEHEMGHEHEMIHEHEMVDHEIVLGNEMVPGNGMVSDYDMIPGNEMVLAEPQPNYVETPPSNIDSQPGK<br>RRKKKSIVWEHFTIENVAVGTRRAQCKQCKQSFAYSTGSKVAGTSHLKRHIAKGSCPVVLRNQQNDQLTPYSAPPKLTG<br>YGGSNGAPKRRYRTASTPYVAFDPDRCRQEISKMIIMHDYPLHMVEHQGFLTFVQNLQPRFDMVSFNTVQGDCVATYLR<br>EKQAIQKVIEGVPGWICLTLDMWSSRHTVGYLFITGQYVDSEWKIHRKILNVIMEPYPDSETAFSHAVAASLSDWSVEGKL<br>FSVTINQPLGDVAVDNLRALLSVKNPLVLNGQLLVGNCLARTLSSIAQDAFNFVHGTVKKVRDSVKYVKTSESHKEKFIELK<br>QQLQVPSTKMLTLDDRNQWNTTYEMLLAATELKEVFSCLDTIDPDYKDAPTLEDWKQVETLCTYLKIFFDTANLLTAPTIPT<br>TNTFFHEAWKIQLELARAAASEDPFTSRLTKMMQEKFDNYWKSCCLMLAIAVVMDPRFKMKLVEFSFSKIYGEEAATYVK<br>TVEEGIHELFNEYVALPLPLTPTYVEEVNGSAMRQEDGQGLDASCNGLGLTDFDVYIMETSSQQSRSELDQYLEESLLPR<br>VHEFDVVGWWKLNRMKYPTLSKMARDILSVPVSTVAADSIFGTVTKEMDSYRCSLRPETVEALICAKDWLQNASVDTLPA<br>PVKMEVPI	Gelsolin--Gelsolin--zf-BED--DUF-domain--Dimer_Tnp_hAT--	XXII
Sme2.5_06608.1_g00004.1	SmeZf-BED14_I	MSSNDNHIKLRSSKYLVVREKDVCWEYAEKLDGNKVRCKFCLRILNGGISRLKHHLSRLPSKGVNPCTKVRDDVTDRVRA<br>IIASKEETKEPPSTKKRKLIEAKTLVNISPEKPLLPVEPTTPIARIFPPIGQAVSLPGNNQENAERSIALFFFENKIDLAVARSS<br>SYHQMIEAVGKCGSGFIGPSPETLKATWLERIKSEVSLQSKDVEKEWAMTGCTLIAETWTDNKMKALINFSVSSPSRTFFY<br>KSVDASSYFKNLKCLSELFDSIIQDFGSENVVQVIVDNTLHCTGIVNHILQNYGSVFVSPCASQCINAVLDEFSKLDWVNRC<br>ILQAQSISKFIYNNSPLLDLMKKFTGGQEIIKTGITKSVSNFLSLQCLLKHRSRLKVIFNSPEFAANSAYINKSQSVNCIAILDD<br>NDFWRTAEECVAVSEPFLKVMREVSGGKPAVGTIYELLTRAKESIRTYYIMDEIKCKTFLDIVDKKWKNNLHSPLHSAAAFL<br>NPAIQYNTEVKFLGSIKEDFFGVLEKLLPTPELRRDITTQILLYTRASGMFGCNLAKEAIDTVHPDICSQFSTTWRHTMTEVK<br>WTAGIWWEQYGDAAPTLQRVAIKILSQVCSTFTFERHWSTFQQIHSEKRNKIDKETLLDLVYINYNLKLARYLLSKPTEEDP<br>LQLDDLDMTSEWVEEAENPSPTQWLDRFGSGLDGNDLNTRQFSAAIFGAGDHIFGL	zf-BED--DUF-domain--Dimer_Tnp_hAT--	I
Sme2.5_11021.1_g00002.1	SmeZf-BED15_V	MTERSIIWEHFEKFTGPNGELTVKCKYCAFPYPNFKIKNGTSGLKSHMGRCAKFPHNIETGQTRAALAKMIIIDELPFHFVE<br>KENEYVTYVLVNMLGEEIGTKLVDKVKKYMASLFNEYANKYAKESQSLAYSSISSSGGTLNLLNSHQTQSKKSYATFAQQ<br>FKKHKANCGSSNAKIELDKYLGEATENGSDEYDILVWWKINSPRFPILAEIDCDVLVISVSSVSSESTFITGGHILDSFRSSL<br>TPKLVQALVCCQDWLRSDSLSIEIEKDLNYLEQFELGFTPDACISLSNFSAS	zf-BED--Dimer_Tnp_hAT--	V
Sme2.5_11032.1_g00002.1	SmeZf-BED16_XXII	MLKSSKRCAHTISSQIQREKAIETFEIHLKFDRQRRQIQREKRSFSPASTDLRCRQIHLKSDLLLRFILSPSISNFSLSIFLDLK<br>SVVHGLVYSKCGVMGYAYDVFDEILEIMESQDEQNQMSSNAVPIENDGQMSSQANEGTNEVIEIEQGRFTSKAWQYFKP<br>LKVDGVHYSVCKYCGGRVKSTRNCGTKSMLEHIRRCRKKPSDLEIEDDSRITEEQFSGIQYSTSSQYFSKVCEVKLELEA<br>WIKSFNPLISNMAVAMLVKFKKYWDDMHIFMGVAAIFDPRYKMKLVEFYLPLIYGEEASTKIQIVRTHCHDLFQEYKSKIFA<br>PRKLVEVSCSNEAIGLLEGDRLSSFDRFVASSSAYTEKRSELDMYLEEGGGTGSGMGTLLISKIREEYPDRMMLIFSVFPS<br>PNVSDTVVEPYNATLFFHQLVENADEQAQAGGDKRALGAVRVLVFASVQNYHRPLLQEALMRVPFY	zf-BED--DUF-domain--Tubulin--	XXII
Sme2.5_14680.1_g00001.1	SmeZf-BED17_II	METTSSAPDTEVITNIPPTMPATSQQGDKVTPHRRTPPKKQKKDNSSGGQCRAKCNYCTKTYAAGSVNGTTTLWNHLNA<br>KCHNSPFRFVGKQQTTLKPIKRGLLEDDLGSLAKMIYNVEEIRRAIVEFMIIDEQPFRVVEGEGFKKLMAKALPNSEVPSRV<br>TVARDYLRIYQEEKEKLKELIRNQRVCLTSDTWTSLQNFTYMVVTAHWIDDQWYLQKKILNFFQTTNHKSETIAKGIEECLL<br>GWGINNLFTITLDNATANDAAIKILKVRIEEWKGIILENEFLRVRCNAHILNLILKEELDEQIGPISHIRSAVKYVRSSPSRFASF<br>KSFVEKVKLDTRGLVSLDVETRWNSMYTMLDKALKFEEAFTRMYVDDQNYQKYYRETSTMSKNPSADDWKDD	zf-BED	II
Sme2.5_16830.1_g00001.1	SmeZf-BED18_I	MPPRPSSSGKGGGFVAEFICSSPTPCKAKLGNWRRVEGRAQYPLSLEEVWFDVMYANKRFFCNSNGVFCNVNGREIRS<br>RFIGIATVLEAMEIEEEAVIVNSSRLKSVVWNDFDRVKKGDTFVAICRHCKRKLSGSSTSGTSHLRNHLIRCRRRSNHDISQ<br>LLTRGKKKEGPLAISNYSFDQEQRNGEAVSIVRTKFEQGHTRDGLFNNGIVNFDNRRSRLDLARMIILHGYPLSMVEHIGF<br>RIFVRNLQPLFDIATFDGVEADCREIYLMERQKVYEELDKLPGKISLSADTWTANGDAEYLCLTAHYIDDSWHLKRKILNFL<br>TTDPSQTEDMLSEVIMTSLRNWDIDRKLFSVTFDNYSTYDKIVSRIREQLCQHRFLYCDGQLFDTRCAANVIKLMVQDTLE<br>TASEIIHKVRESIRYVRSSQATQEKFNEMAQIAGADSQKCLNLDNSFYWNSTYIMIVTALEYKDAFPLLQEHDSRYAMCPT<br>VTEWDRISAIANFLKLFVEVSNVFAGSKYPTANSYFPDICDIHLQLIEWCQNSDDFVNSLALKLKSRFDEYWKKCSLALAIA<br>AILDPRFKMQLVKYYYPQIYGDSAPDCINIVSDCMKALYNGHAIYSPLAANGQAEASQVGDANNDRLTGFDKFIYETSVSN<br>NIKSDLDNYLEEKLFPRKDDFSILNWWKVHTPRYPILSMMARNILGMPMSKASLEHVFNTGNKALEPYRSSLRSDTLQAL<br>MCAQDWMRDEFEDSKASSSTVTLALCYDAK	zf-BED--DUF-domain--Dimer_Tnp_hAT--	I
Sobic.002G190000.1	SobZf-BED01_I	MVNGIEDMDAIANDLVEQESTGSPLFSGTNLNKRLRSKVWDDFIPTFAEGRIVRAKCMHCHQVFGGTNGTSSLLRHLASC<br>VPATLKRAKMQEHTSLPCTQKGTVSAGSDLKQKKLPFLPSNQKKCVGTDDMIPVQKELALLDTPTTMTRMNQEVVQNGS<br>HEDLAEQKNLALPGISTDNNGRNQSNKENTSAEQIGILADTSQKHEVDENASHEELIKILAMHGHLPKMVDQDGFRELVT<br>WLNPLVKMPSHDDLMMNTLNLFQKEKSKLMQEFTALRSRVCLSVYMWHYHPVLAFLCLRVHYIDGEWEKQQKVITFRAV<br>DSSCNAKELSDIISGAVEHWGLDGKVFCIILDDAFIDNSVALTVKANLHERNPLSANQSLFVVRYATHLVDRVIQVGLDELE<br>KTLEKQSKFSKCTNGRTPAAVQYPNCRYAPASEDWWTAGKISDKLDNLHKRVGSTKRYPTPAHLFNMLWNVKQDVHYE<br>SLIYCYKDDDTFSKILVKMQNKFKESWKICFFHCCMPMIMDPECRLERIKSSVWLFGSEKNDVLRTLASLFNEYLDQAENP<br>NNSSGSKTSKGTVVDADTLVEYYSHWGQCSERPMTELDQYLQAPHLTTSEPVGLKGAVGKPSDLQWWKEHSRNYPTV<br>ARMARDVLALPCISDWKAATRTATLAISESGSKQWVEELVCTQDWLTPAGANQESTDDL	zf-BED--DUF-domain--Dimer_Tnp_hAT--	I
Sobic.002G190000.2	SobZf-BED02_I	MDAIANDLVEQESTGSPLFSGTNLNKRLRSKVWDDFIPTFAEGRIVRAKCMHCHQVFGGTNGTSSLLRHLASCVPATLKR<br>AKMQEHTSLPCTQKGTVSAGSDLKQKKLPFLPSNQKKCVGTDDMIPVQKELALLDTPTTMTRMNQEVVQNGSHEDLAE<br>QKNLALPGISTDNNGRNQSNKENTSAEQIGILADTSQKHEVDENASHEELIKILAMHGHLPKMVDQDGFRELVTWLNPLV<br>KMPSHDDLMMNTLNLFQKEKSKLMQEFTALRSRVCLSVYMWHYHPVLAFLCLRVHYIDGEWEKQQKVITFRAVDSSCNA<br>KELSDIISGAVEHWGLDGKVFCIILDDAFIDNSVALTVKANLHERNPLSANQSLFVVRYATHLVDRVIQVGLDELEKTLEKQS<br>KFSKCTNGRTPAAVQYPNCRYAPASEDWWTAGKISDKLDNLHKRVGSTKRYPTPAHLFNMLWNVKQDVHYESLIYCYK<br>DDDTFSKILVKMQNKFKESWKICFFHCCMPMIMDPECRLERIKSSVWLFGSEKNDVLRTLASLFNEYLDQAENPNNSSGS<br>KTSKGTVVDADTLVEYYSHWGQCSERPMTELDQYLQAPHLTTSEPVGLKGAVGKPSDLQWWKEHSRNYPTVARMARD<br>VLALPCISDWKAATRTATLAISESGSKQWVEELVCTQDWLTPAGANQESTDDL	zf-BED--DUF-domain--Dimer_Tnp_hAT--	I
Sobic.003G188300.1	SobZf-BED03_VI	MDEGGSQGIKKVHFRDSSSQHPIVTYKRRRQQKPQTQQQLPQSPPPQQHQPEPQPLQQVEPQPQVEPEPNAGDVPAQ<br>QSKDTFWKSRDMGWKYGIMIDENRQHWKCMYCGLIRHGGGVSRLKRHLAGDLDVKMCPKVPADVVEEIREHLRKKRE<br>RRKKRAAQNGGDNVRTKSPSGDADVEKDLLPSDSVLSDGTGTNVPEEVTNQTSSVHHDTTPRFPILRARDIGWEHAVDL<br>DGNKRRWQCKFCSLCRSGGVTTLKAHLIDDSCPNVPKEISKKVSNFIEEKRATRLLLNNYVFNVDDDFNTQVQGEVTVEY<br>LNEQQPSRNVYVQTLDKGDINEVAAGSKQCGAESSGQPVERCDQPEEQCTMDYGRMDQVTSNKNQILDKNTENSKNT<br>KMLKPCRKSEFNTRKHIIIVDQSARHWRCRYCGMDGYGKKFWLHYHLAGAFHQPKCPNVPREVFAKARRHVLTKRRLKK<br>NKAEQQIPSSPHILGQSGEERQNSDPLCGNQSQLSINNEPREVHDYPAVLRDSAWEHSLIYEKENGSWKCKWCSIEGDH<br>GLTRLKWHLVGWQNHPQCPNIPMDVAEKMKDQMMSKEEQKARSGLFDGNGYCDVLCSSKSSQLDQDHLTATIHDRCS<br>SQAFANSELKGCNMLSSTTLLSQESSNPQVHHEDPQVCHEQERNEVATSSEPGCEKGQRMQWQSQNKPMMEEGPHG<br>NGLCGDTNKLEEQKSDFGSSDCWRYVLDRLMHLPDVQEDAGIGTCIRDALLYGCAEFGTVADKVEMDRDKTVNANTAR<br>CQNILMDVLRSENFALLCSVLCRTVHQDGERTRYFDFGVIDSRMKNGNYGHEPELFMHDLKLLWEDLKVAGQDIIHLANN<br>LSSLTEDSYEKLVGRERGSDDGELNGAVVARSEPKNLVQSNALVPLTSQGFNQLDQPGPSYLSDVYKDSICNQCGKEAR<br>VGSVLKCYRCMLPCHISCIQATDSFISTGRWCCKNCSAGSKEPVEGDMVLAHYYPNCLHENCVVCDRLAACRSPKCEDT	zf-BED--PHD--	VI

		PNENSRAMVISSVDSFADPELPEIDTCYSCKICGDTEEDEKRFLICGHVHCLYKYYHIRCLMSKQISSNVQRDQPCWYCPSCLCRVCLSDKDDHLTILCDGCDEAYHLYCITPRRTSVPKGHWYCSSCSVERAKEGMRQYERTLKLHQKDDAELQSWNYDGVDLLLSAAEQLREDELLVTRTN		
Sobic.005G129900.1	SobZf-BED04_II	MESSLLRRGVGSNSSSITQMRACASVGLGGATDGHPRAAAPLPPPPAGPAVAAASQDHNDGDINKVWMHGSKLAGQGFKCGYCGTTNKGGGATRFRDHLGCIVGEVKSCPSVPRVVRDAMRELRKTSMGNKREKRERMLRLERDLMQGLQGQEVVNLASD	zf-BED	II
Sobic.007G072133.1	SobZf-BED05_XVI	MRRISGLMENQSAHPRHGMVDQGFRPFTTTPLSSSVNAAPVMMPIWSADDGYIWKNSGLQEILGSGSGRLICYECSQANCMVKKSVVLSADGQILETVLRGSHNHPRPSEICPRDVPTGYIPDSQHYAYVPSEMYVPGASIPQTEGGEQEQLGSSSDSDEEDDGEQRDDGHVASASTTERPVVAPAERRTRSGTPHRKRPKSKVWEEFTKIFRDGKLQAAVCKHCESSLSAKTTGGTSHLKRHLQSCPGRPSTGRVQLQRPFSHPNSSVQKNLNLDQNKSLELLIKVLVSNLFSSPFTSSTIFRQFWAGISPTNDMVSPAAIEEKFLSIFQTEKLKLQEEIELAPGGVFLTVAGSSLELKCVIFLMVHFIDKEWNLNRKIIRCCFTGCEDSDAEYYASMFPFLKSYYYIFNGNVRAVEIEIVKEVVQNWKLEWKLLGISSSKSLCDASVPALEKNLTGQNYLLAKWINTKKSCHACK	WRKY--zf-BED--	XVI
Sobic.007G156000.1	SobZf-BED06_II	MANNDGEDGYSYLPTLADELRDCGMPGEDEDDMSDVADLLFGAVPSATSGAGRASAATGAGRGRGRGRGNAAGAGAAGRGRGAPAVAAPGAPAVAAPGAPAVAAPSAPASTAPSLSDAASLASSASNSKRRSPVWKHYDEVHDTVDGEDRCFAVCKLCKSRLSATSANGTGRLKRHHTSCQNKSDHASMIQTRLALNPDGL	zf-BED	II
Sobic.009G102000.1	SobZf-BED07_II	MAMPPIKGGPTSPTSLAGELRAVAEILLRLPTPAALVRAALASRRWLQVASSPGFLRRYRSRHASSPLLGLYVPRAHTGLPSFQLADSVRSDRALAKFVRGGDFGLTGLESHPEWRLLDCHNGRLLLSRGESRAVFDPASAREPVWLPQNSPLPSTFISECLLQGHSDDAASFRVVSVQQRGHDRLLRAAEYHSHTRQWHCHQWVKDINRPQHDQAMHAGRFIFWRYDDTSSLLLDTATMEFSILGIPFTFFQESMYAIGDTEDDMCCLVGLVGSINNIHLQVWLLKANGAANMWEPERKILVSQVLGRDIQLRQVHAVTNGLALLCWDQSRQFAINLKKMCIDSEFECSALGYPLQMPWPPAVLVETNEMDQGIEVIHGCDTNESATLTKSRCGSEMVSSDHGGEKIHCHETVIQGSEIFQGIETTQDNGTAQVTPPTTSRCGNDMDHSDEMIHCNQMVICGSEMVQGIDLTHGNYTGEAAPTTSRHCSETVQVSKNNHGGLMTRCNQMIIYDREMVQGIEMTLDKNNAEVTPPTIAKIRRKNSIIWKHFTTETDSDGCTRACCKYCRRSFACSRTSGTSHLKRHLTLGSCPALKGQVPPSAGGAQHCGSGAAEKPSKRQCTYADPADDALNQNYNISYLGKMDILTEPLTTKQGNEYSIPKCLKVLHDMDDVSDEMKHLAFHILKDATNREIFMSYESRLRGLWLKKEVNKLGT	zf-BED	II
Sobic.009G102000.2	SobZf-BED08_II	MAMPPIKGGPTSPTSLAGELRAVAEILLRLPTPAALVRAALASRRWLQVASSPGFLRRYRSRHASSPLLGLYVPRAHTGLPSFQLADSVRSDRALAKFVRGGDFGLTGLESHPEWRLLDCHNGRLLLSRGESRAVFDPASAREPVWLPQNSPLPSTFISECLLQGHSDDAASFRVVSVQQRGHDRLLRAAEYHSHTRQWHCHQWVKDINRPQHDQAMHAGRFIFWRYDDTSSLLLDTATMEFSILGIPFTFFQESMYAIGDTEDDMCCLVGLVGSINNIHLQVWLLKANGAANMWEPERKILVSQVLGRDIQLRQVHAVTNGLALLCWDQSRQFAINLKKMCIDSEFECSALGYPLQMPWPPAVLVETNEMDQGIEVIHGCDTNESATLTKSRCGSEMVSSDHGGEKIHCHETVIQGSEIFQGIETTQDNGTAQVTPPTTSRCGNDMDHSDEMIHCNQMVICGSEMVQGIDLTHGNYTGEAAPTTSRHCSETVQVSKNNHGGLMTRCNQMIIYDREMVQGIEMTLDKNNAEVTPPTIAKIRRKNSIIWKHFTTETDSDGCTRACCKYCRRSFACSRTSGTSHLKRHLTLGSCPALKGQVPPSAGGAQHCGSGAAEKPSKRQCTYADPADDALNQNYNISYLGKMDILTEPLTTKQGNEYSIPKCLKVLHDMDDVSDEMKHLAFHILKDATNREIFMSYESRLRGLWLKKEVNKLGT	zf-BED	II
Sobic.009G102000.3	SobZf-BED09_II	MAMPPIKGGPTSPTSLAGELRAVAEILLRLPTPAALVRAALASRRWLQVASSPGFLRRYRSRHASSPLLGLYVPRAHTGLPSFQLADSVRSDRALAKFVRGGDFGLTGLESHPEWRLLDCHNGRLLLSRGESRAVFDPASAREPVWLPQNSPLPSTFISECLLQGHSDDAASFRVVSVQQRGHDRLLRAAEYHSHTRQWHCHQWVKDINRPQHDQAMHAGRFIFWRYDDTSSLLLDTATMEFSILGIPFTFFQESMYAIGDTEDDMCCLVGLVGSINNIHLQVWLLKANGAANMWEPERKILVSQVLGRDIQLRQVHAVTNGLALLCWDQSRQFAINLKKMCIDSEFECSALGYPLQMPWPPAVLVETNEMDQGIEVIHGCDTNESATLTKSRCGSEMVSSDHGGEKIHCHETVIQGSEIFQGIETTQDNGTAQVTPPTTSRCGNDMDHSDEMIHCNQMVICGSEMVQGIDLTHGNYTGEAAPTTSRHCSETVQVSKNNHGGLMTRCNQMIIYDREMVQGIEMTLDKNNAEVTPPTIAKIRRKNSIIWKHFTTETDSDGCTRACCKYCRRSFACSRTSGTSHLKRHLTLGSCPALKGQVPPSAGGAQHCGSGAAEKPSKRQCTYADPADDALNQNYNISYLGKMDILTEPLTTKQGNEYSIPKCLKVLHDMDDVSDEMKHLAFHILKDATNREIFMSYESRLRGLWLKKEVNKLGT	zf-BED	II
Solyc01g010610.1.1	SolyZf-BED01_VII	MDPRSLVWQHFEKVFENGVLVKAKCLHCKQYYAANTTRNGTSGLKQYLTYRCKVYKPPTVAPGIQKLLNIQNNNLETWKFEQEF	zf-BED--zf-BED--	VII
Solyc03g007510.2.1	SolyZf-BED02_III	MMLYAHSAHLHKLQNSTKSRDKSNRSLSLSFCNFRLTVDNSFLHVSLCSISPLRRESLHNITMVREKDVCWEYAEKLEGNKVRCKFCLRILNGGISRLKHHLSRLPSKGVNPCTKVRDDVTDRVRDIIGSKEPPSTKKHKLIETKALANISPEKPLLSVEPITPIARIFPPIGQAISSSGNNQENAERSIALFFFENKIDFGVARSSSYHQMIEAVGKCGSGFIGPSPETLKATWLERIKSEVSLQSKDVEKEWAMTGCTLIAETWTDNKMKALINFLVSSPSRTFFYKSVDASSYFKNLKCLSELFDSIIQDFGPENVVQAQSLSKFIYNNSPLLDLMKKFTGGQEIIKTGITKSVSNFLSLQCLLKHRSRLKVIFNSPELAANSAYTNKSQSVNCITILDDNDFWRTAEECVAVSEPFLKVMREVSGGKPAVGTIYELLTRAKESIRTYYIMDEIKCKTFLDIVDKNWKNNLHSPLHSAAAFLNPGIQYNPEVKFLGSIKEDFFRVLEKLLPTPELRRDITTQILLYTRASGMFGCNLAKEAIDTVPPGIWWEQYGDAAPTLQRVAIKILSQVCSTFTCERHWSTFQQIHSEKRNKIDKETLLDLVYINYNLKLARYLVSKPPEEDPLQLDDIDMTSEWVEEAENPSPTQWLDRFGSGLDGNDLNTRQFTAAIFGPGDNIFGL	zf-BED--zf-BED--DUF-domain--Dimer_Tnp_hAT--	III
Solyc03g078070.1.1	SolyZf-BED03_VII	MRPTKSVGIDFDDKPLWNHVKVISMAPNGGENRTWSCNYCNKIVTGSYNRVKAHLLRLSGHGVQICKENSGDIYVILKMEHEQAERKRTNVQVDMADKLAARMFYASSGDRFDIDETTNDLTELSIDDPQIEGVIFEEEFEDLEEVEEDVEEIANLIK	zf-BED--zf-BED--	VII
Solyc03g116840.1.1	SolyZf-BED04_XXII	MNYARKKKQGVTMDTLTTLNEIADSEAQPNKFRRKKSIVWEHFTIERIGADCTRACCKKCKKSFAYISGSKLAGTSHLKRHIALGICPVGRTNQDKNQLTSFNSAAPTNGSAGATGKSRKRYRANPGPTSVPFDQARCYHDIAKMIIQHDYPLEMVEHSGFNKFVQNLQPLFSSVSVDTIQEHIFNIYLGEKQNLLNIIGAIPGRVSLTLNLRTSDQNLGYVFITGYFVDSDWKLRCRLLNVIMVPFPDSDVAFNHAVAACLTDWCLETKLFTLTLDQSVANVNVRKNLGHLLSIKGVNILNGQLIIGSCCARVLSDLAQYALHYMRAIVEKVRQSVKFVKTADAHEEKFLELKRQLQVPSAKELIVDDQTKWDTTYQMLMTASELKEVFSCLDTSDPDYKVTPTMDEWKQAEILCEYLKLFFDAANLLTSPTYSTADVLFHEVWKIQLDLMQAARSQDRFIRDLTRPLQEKFNEYWNDCNLVLAVAVVMDPRFKMKLVEFTFNKIYGEEAETWIKIVDEGVHEVFCDYIVQSLPPPPASFVEEANDNFVIKSEFSQEDSFLATNGDAFPDFEVYLDIINNQQMKTELDQYLEESLMPRSQDFDVLGWWRINRCKYPTLSKMASDILSIPVCTVTPDSVFDTVSRDLDRHRSSLRPITIEALSCSKDWLQYESWEPPYGTPDATVKMNMMLSSLRGLSNLTYTFHGKGVFLLASQCPNQVFQLAHLHLSPEIHLPLQSLIEKCKSMDQLRQIQSVIIQKGLISDPKLCSNMITFCSNNESGDMKYARSVFDIMPERGVFIWNTMIKGYSRENIPHDGVSIYREMLNNNVKPDNYTFPFLLKGFTREVSLKLGRSVHAHICKFGFELNEFVHHALIHVYCLCGQVDMARGVFDLSAKSDILIWNSMISGYNRSKQFGESRKLFYAMEEKQLQPTSVTLISVISALSQLKDLDTGNRVHQYVKDYKVQSSLVLDNAIVDLYASSGKMDVALGLFQSMKHKDVISWTTIVKGFVYIGQVDVARIYFDQMPKRDNISWTAMMDGYVKENRFKDVLMLFREMQAAKIRPDEFTMVSILTTCAHLGALELGEWIKTYIDKHKIYVDIHLGNAVIDMYFKCGSVEKALVMFTQMPSRDKFTWTAMIIGLASNGHEREALDMFFEMLRASETPDDVTYIGVLSACTHMGLVEEGKSFFANMASQHGIQPNVIHYGCLVDLLGRAGRLEGAYEVIMRMPVKPNSIVWGALLGACRIHKDVQMAEIAAQQLLQLEPGNGAVYVLLCNIYAACKKWDNLRETRRIMTDRGIKKTPGCSLIEMHGIVHEFVAGDQSHPQSKSIYSKLAELIGELKFSGYVPDTSEVSLDIGEEEKENSINRHSEKLAIAFALINSEPGFTIRIVKNLRICTDCHHVAKLISKRYNRKLIIRDRTRFHHFVQGSCSCKDYW	zf-BED--zf-BED--DUF-domain--Dimer_Tnp_hAT--PPR-domain--DYW_deaminase	XXII
Solyc03g119	SolyZf-BED	MDWNVNTAYKTLKEMESKSLAVVEAPSTTLNIESIDIGPGSSEKDPATKLRKKALTSVYLKYFETAPDGKTRKCKFCGQSYSIATATGNLGKHLSNRHPGYDITVNVASPAPQSVTVVKKLQPKPHVKGPQLELDHLNWLLVKWLILASLPPSTLDEHWLLNSFKFLNPTVKLWPEEKFQSVLCEVFRSIQEDVRVIVDQISSKVCITLDFWTSYEQLLYMSVTCQWIDENWSFQKLLLDICHISSPCGAAEVSHALLKVLKIYNIENRVLCCTHDNTPIALHACHTLKEDMDSQKMSPFYYLPCAAHTLNSVINDGLSSTKSIISK	zf-BED--zf-BED--DUF-domain--	III

830.2.1	05_III	IREIVLKMNTSFEISQDFLQCCNACQEGTWKFPLDASPRWSGNYQMLDIARKAGKSLEAMFRKYDELLGSRVLLNNAEKNAVNIMHAFLEPFYKTIHDICTNKVVTVGLVLFFMDHISETIAACRDSRHSPDWLRSAADEMATKARSYNEQMCNSFTYMTAILDPRIKVELIPESLNSENHLEEARSHFMRNYSTSHFPCISGSYAAHELEDGASVSFAEEIARKKRKASMSSATDELTQYLSEPPAPIPTDVLEWWKVNNARYPRLSSMARDFLAAQPTALAPQDLFCSKGDEIDKQRFSTSYGSTQALHCVKSWMQSGFKLKYKSTEIDYERLMELAAATAAESSMASSDKKQKS	Dimer_Tnp_hAT--	
Solyc04g051430.1.1	SolyZf-BED06_VII	MVRGWSMSFSQAKCAPAAARRSSSAVRPDVHQKKARSSSSREKQQRRRPAVHQKKKKQSRHTVKQPAAAAPCRPPSTFNQPGASSRAGVRTTADQSRISMVDSTDNTPSNSNDPSIDTEIIIKKKEMDPRSPVWQHFEKVFENGVFIKAKCLHCKQYYAANTTRNGTSGLKQHLIIGVKCINLRLLHPIFKNY	zf-BED--zf-BED--	VII
Solyc04g070990.2.1	SolyZf-BED07_XXII	MAAKPIDDTIWRGSFMISRPEMRLNLTAHLSSKACAKVWLAAKEMAEVIYPEFLPKRDVWPKSFKSAKLIDDNIAIYFFSVKGRDDQVFENLLYNLRHYDIALRALVGDSELLIFTSAHLPEKHHIAGFEGKVYLWGVFSGRQAPPQESPDDCFIHNTRVTQASIPNSANDGKDRSCKEFSNRKDPSTASKSARKGRPLDDAWQHATPVDGKKQRTVCNYCGFISSSGGITYLKTHLGGGDPTGSLKGCPNVPPEVKRVMKEWLLGTIRGGKAPQLQEIRTDFEVPASKKSVRRGRPLDAAWDYATPVDAKRQRAVCKYCGFISSSGGITHLKAHLAGGDPKGPSKGCPNVPPEVKRAMAESLNRTVKGAKSMQPDEIRRYMKAENDWSPPRSDDYSLNQHRIVKNAQYSHFANGNSVNDMTLSKQSETAHVDSYMEVMSSHNACKSSFLSKPSTRVGFI	zf-BED--zf-BED--zf-BED--zf-BED--	XXII
Solyc08g007470.1.1	SolyZf-BED08_III	MVRGRDACWEHCVLVDATKQKVRCNYCRREFSGGVYRMKFHLAQIKNKDIVPCGQVPNEVRDHIKNILNNPKKQKNPKKAKLDQAANGQESSSSSASGGIHPPHDGFSGQNGSPCPPSIMLARRSSSLQPAVDDVQKQKQDNADKKIAEFFYHNAIPFSVTKSFYYQEMVDAILECEAGYKAPCTEELGTKLLEKVKVDIDDGYKRLRDEWKETGCTILCDCWSDGRAKCLVVFSVTCSKGTMFLRSVDVSDHADDPHYLFGLLESVVLEIGVENVVQVMTDSSASYIYAGRLVMKKYPSVFWSPCASHCINKMLEDFSEHDWVNVVLKEANMITKYIYSNDWMLDMMRKFSGGGEFVLVRPRFTNFIAIFLSLRALVIQEDNLKHMFSHAEWLSSIYSRHPEVQAIKSLLCLERFWRSAREAVTVSEPLLKLLRIVDGDMPAMAYMYDGVERAKLSIKAFYKDVDEKFVPIWDIIDRRWSMLLQSPLHAAAAFLNPSIFYNSSFKIDARIRNGFQEAMTKMASEDKDKVEITKEHPMYINAQGALGTEFAIKGRTLNAPADWWTGYGYEIPTLQRAAIRILSQPCSLHWCRWNWSTFDGVHEKRRERLELDRFNDLVYVHCNLWLRALIRSKDGKWKPINFDEIDVGAEWPTEAEVAPCTYLDDSWLQLVG	zf-BED--zf-BED--DUF-domain--Dimer_Tnp_hAT--	III
Solyc09g005660.2.1	SolyZf-BED09_III	MAVDFPFSSLIKLKSATKTDTISLLLHTVEDQMTRDKIDIRQHGVPVDQKKLKVKCNYCGKVVSGFSRLKQHLGGIRGDVTPCLKTPILVKEALEAEILNKKNENLIKKVGQLQHPSLPLKRNWCPRDGEPNKTSESVNKKHNGVNSNVAGTSVVDSSSQEISKSIGRFFYEAGIDFDAIRLPSFQRMLKATLSPGKTIKFPSCQELKGWILQDAVKEMQQYVTEIRKSWASTGCSILLDGWIDSKGRNLINILVYCPRGTIYLRSSDISSFNGNVDAMLVFFEEVLEEVGVETVVQIVGYSTSACMMEAGKRLMEKCKTVFWTVDVSHCMELMLQKFTKMNPIQEALEKAKTLTQFIYNHATALKLLRDACPDELVKSSKIRSIVPFLTLENIVSQKDCLISMFQSSDWHTSIMASTNEGKRISEMVKNESFWSEALMAVKATIPLVKVIKLLNGTNKPQIGFIYDTLDQIKVTIKKEFQGKESLYAKFWAAIDDIWNGYLHSHLHAAGYFLNPIYFYSSDFYADAEVTSGLCCCVVRMTEDRHIQDLIALQIDEYRKGRSTFHFGSFKEKLINISPGALWWSQYGVQYPEIQRFAFRLLSQTCNGASHYRLKRSLVETLHTEGMNPIEKQRLQDLVFVHCNLQLQAFDPDGSNDNTDYVVDPMDEWIVRKEPNLVHENTQLTWMDLELASRNGKVKDVIYVKDEADEDEPKYI	zf-BED--zf-BED--DUF-domain--Dimer_Tnp_hAT--	III
Solyc09g064150.1.1	SolyZf-BED10_VII	MAYLFAVFGESIMAENVITDKVVGESGASNSNAIDQSQIVESQVKKGRKKRSRAWDHFSRKTDSDGNEKGVCNYCKKEYFVDTKEHGTTSMLNHTSKCPKMPYNIDIRQSRLAFQPMIGGNKGDVVVVPWKFDQEECRKALCRMKNIS	zf-BED--zf-BED--	VII
Solyc10g007190.2.1	SolyZf-BED11_VII	MASPNPHSSPPVAVDQRRLQFTPVDLPLTPPMADAIPEGIVDLTSLTEASTGEKRKREGRLRSRVWQHFTKLIKEDGTCDKCKCNHCHKIFTSSTRSGTTHLLRHISEGICPAFKKVKKENSPSIFSYIGGSIDRKVGINPWKFDQELGQASFEQSIDAHDDLLPLGLDDIERQTCTASESDYVSQASMPVFSKLPQQPAVKSHPISEPWMTELKACVGKLVELTNGGVSKPTSADNKTCVAVTTPDLSITSVVKCLNEMEDIPQSSAMYLDALDLVRDPEERECFICLNPEPRRRWLQRMLHRRFPLRYSTDV	zf-BED--zf-BED--	VII
Solyc10g007470.2.1	SolyZf-BED12_III	MEIPTETPIKKPKRLTSVVWNHFERVRKADICYAVCVHCKKKLSGSSNSGTTHLRNHLLRCLKRSNYDVTQILAAKRKKKDPTLAVVTYEEGQRKEETISPVSAFKFDPEVKKEEVNVPINLGSVRFDQERSRLDLARMIMLHGYPLAMVDHIGFKIFVKNLQPQFEVLTNSAVELDCMTIYAKEKQKMYEAIHNLHGRISLSADVWDSSENARYMCLTAFYIDEDWKLQKKMLNFITLDPSHTDDILSEVVVKSLTDWAIDRKLFSMTFDHCTGYEELIFRIKDWLSQNRPLLKNGELFDVRCAVQLMKSIVSDVMEAIRDVTHKVRESIRHVKSSQVTLGKFNEIAQQAAISGERPLILDCGQQWSSTYLMLEAALDYRGAFCLLEEHDPTYTSALSETEWDHASVVAGYVKLFVEVTNVFTTNKYSTANIYFPEICDIHIQLIEWCKNPDNFLSDIALKMKEKFDRYWSKCSLTLAIAAILDPRFKMKLVEYYYPQIYDSDAPNQIKAISDAIRELSNEYAMGSSSLDPDTAGASGSLASTTIGTRDRLRGFDKFLHETSHNHNMTSDLDKYLEEPVFPRNYDFSILNWWKVHTPRYPILSMMARDILGVPASTLGPELAFNNRGRVLDYHRSSLNPDAREALICGQDWLRMESEEYNSPHIYTAVPLTVESNLIGDQDTSV	zf-BED--zf-BED--DUF-domain--Dimer_Tnp_hAT--	III
Solyc10g007810.2.1	SolyZf-BED13_III	MEIEEEAVIVNSSRLKSVVWNDFDRVKKGDTFVAICRHCKRKLSGSSTSGTSHLRNHLIRCRRRSNHDISQLLTRGKKKEGPLAISNFSFDQEQRNGDAVSVVRTKFEQGHTRDGLFNNGIVNFDNRRSRLDLARMIILHGYPLSMVEHIGFRIFVRNLQPLFDIATFDGVEADCREIYLMERQKVYEELDKLPGKISLSADTWTANGDAEYLCLTAHYIDDSWHLKKKILNFLTTDPSQTEDMLSEVIMTSLRNWDIDRKLFSVTFDNYSTYDKIVSRIREQLCQHRFLYCDGQLFDTRCAANVIKLMVQDTLETASQIIHKVRESIRYVRSSQATQEKFTEMAQIAGVDSRKCLNLDNSFYWNSTYIMVETALEYKDAFPLLQEQDSRYAMCPTVTEWDRISAIASFLKLFVEVSNVFAGSKYPTANTYFPDICDIHLQLIEWCQNSDDFVNSLALKLKSRFDEYWKKCSLALAIAAILDPRFKMQLVKYYYPQIYGDSAPDCINIVSDCMKALYNGHAIYSPLAPNGQAEASQVGGVNNDRLTGFDKFIYETSVSNNIKSDLDNYLEEKLFPRKDDFNILNWWKVHTPRYPILSMMARNILGMPMSKASLEYVFNTGNKALEPYRSSLRSDTLQALMCAQDWMRDEFEDSKASSSTVTLALCYDAK	zf-BED--zf-BED--DUF-domain--Dimer_Tnp_hAT--	III
Solyc10g047760.1.1	SolyZf-BED14_VII	MKEDLSRGNRRDPGWKYNYMKYPNDTTRVTCNFCVETTLGGINRAKQHLIGNFRNAAKCKKS	zf-BED--zf-BED--	VII
Solyc10g080090.1.1	SolyZf-BED15_III	MATPKTPVTPAAPTENHEMVHEHEVIPEHEMGHEHEMGHEHDMRHELDMGHEHEMGHEHEMMQEHEMMQEHEMVEHEIVLGNEMVPGNGMVQDYEMIPGNEMVLAEPQPNYVETPETQPGKHRKKKSIVWEHFTIENVAVGTRRAQCKKCKQSFAYSTGSKVAGTSHLKRHIAKGSCPVVLRNQQNDQLTPYSAPPKMTGYGGNNGAPKRRYRTASTPYVAFDPDHCRQEISKMIIMHDYPLHMVEHPGFLTFVQNLQPRFDMVSFNTVQGDCVATYLREKQAIQKVIEGVPGWICLTLDMWSSHHTVGYVFITGQYVDSEWKIHRKILNVIMEPYPDSETAFSHAVAACLSDWSMEGKLFSVTINQPLGDAAIDNLRALLSVKNPLVLNGQLLVGNCLARTLGSIAQDAFKVVQGTVKKVRDIVKYVKTSESHEEKFIELKQQLQVPSTKMLTLDDQTQWNTTYEMLLAATELKEVFSCLDALDPDFKDAPSLEDWKQVETLCTYLKIFFDTANLLTAPTIPTTNTFFHEAWKIQLELARAAASEDPFTSRLTKMMQEMFDNYWKSCCLMLAIAVVMDPRFKMKLVEFSFSKIYGEEAAAYVKTVEEGIHELFNEYVALPLPLTPTYVEEVNGSGMRQEDGQGLDASCNGLGLTDFDVYIMETSSQQSRSELDQYLEESLLPRVHEFDVVGWWKLNRIKYPTLSKMARDILSVPVSTVTADSIFSTVSKEMDHYRCSLRPETVEALICAKDWLQNASLDTSPIPIKMEVPI	zf-BED--zf-BED--DUF-domain--Dimer_Tnp_hAT--	III
Solyc11g012630.1.1	SolyZf-BED16_VII	MPQLHGSNARSPIWNHYEKLEEKEDGSWTVKCIHCGRVAYYHSHYNGIASLRNHVKQCLETRNQNP	zf-BED--zf-BED--	VII
Solyc11g050940.1.1	SolyZf-BED17_VII	MGGADQSPISMADSTDNTPSNSNDLSIGTEITKKMDPRPLVWQHFEKVFENGVLVKAKCLHCKQYYAANTTRNGTRGLKQHLNYRYKVYKPLTVAPGIQKLLNIQNNNLETLKFEQEVCRRALVELIILDELPFSFVEKEGFKKFLSKVQPLFHFPSRRTITRDC	zf-BED--zf-BED--	VII

Solyc11g065860.1.1	SolyZf-BED18_VII	MAYLFAIFGESIMAENVITDKVVGESGDSNSNAIDQSQIVESQVKKGTKKRSRAWDHFSRKTDSDGNEKGVCNYCKKEYF<br>ADTKEHGTTSMLTHISKCPMMPYNIDIRQSRLAFQPMIGGNKGDVVVVPWKFDQEECRKALCRMVIIDELPFRFVEKKVL<br>NNL	zf-BED--zf-BED--	VII
Solyc12g009640.1.1	SolyZf-BED19_III	MATPETAAPPATPTENNEMVPKNEMIHEHGDEMVPQNEMIHEHGDEMVPQNEMIHEQSHEIVPENEMQNEMIHEIVPEN<br>EMQNEMIHEHSHDIVPENEMQNEHSHEIMPENEMIREHSHEIVRENEMIPEHNHEIVPENEMIHECSHETVPQNEMVHEH<br>SHEMVPDNEMTHEHTHEMVPENEMTHDHNHEMVQENEMTHEHNHEMVPENEMMHEHGHEMVPEHEMMHEHSHEM<br>MPEHEMMHEHHMVLGHEIVPSNEMVPDDEMIPLNEMVLAEPQPNYIETPPNNPETQPSKRRKKKSIVWEHFTIENVGGG<br>TRRAQCKQCKQSFAYSTGSKVAGTSHLKRHIAKGTCPVVLRNQQNNQLSPYSTPPKMSGYGGSTDAPKRRYRTASSPY<br>LAFDPDRCRQEISKMIIMHDYPLHMVEHPGFLAFVQNLQPRFDMVSFNTVQGDCVATYLREKQAIQKVIEGVPGRICLTLD<br>MWSSCYTVGYVFITGQYIDSEWKIHRKILNIIMEPYPDSDTAFSHAVAACLSDWSMEGKLFSVTINQPLGDASVDNLRALL<br>SVKNPLVLNGQLLVGSCLARTLSSIAQGAFNFLHETVKKVRDSVKYVKTSEFHEEKFIELKQQLQVPSTKTLALDDQTQWN<br>TTYEMLLAASELKEVFSCLDTSDPDYKDALSMDDWKQVEVLCTYLKILFDTANLLTAPTIPTTNTFFHEAWKIQLELARAAA<br>SEDPSISGLTKTMQEEFDKYWKSCCLILAIAVVMDPRFKMKLVEFSFTKIYGEEAATFVKFVEEGIHELFLEYVALPLPLTPA<br>YAEEVDDGALKQENGGGGLTDFDAYIMETTSQQSRSELDQYLDESLLPRVHEFDVVGWWKLNRMKYPTLSKMARDILS<br>VPVSTVTADSVFSTVGKEMDRYRCSLRPETVEALICAKDWLQNASVNTLHAPIKMEVPI	zf-BED--zf-BED--DUF-domain--Dimer_Tnp_hAT--	III
Spipo14G0037300	SpiZf-BED01_V	MDMTLTPNMDQIEYGVGSSDKESAGQMKPRKKSMTSLYLKFFETATDGKSRKCRFCKQSYSISTATGNLRRHLNHHHP<br>GYDRQGDGAHQAPQPGGAAAKKIQSQVKPAAVDSDQLNWLLLRWLIGASLPPSAMEDRALLASLRFLNPGVKLWSGER<br>AQLTASEVFSSMRDDVRASLENLSSKVSIALEFWTSSEQIFYMSVKGHWIDESWCLRRVLLGVPCIPHPCGGAEILHALLK<br>VLRMFGIEKNVLACTLDCSRDASQPAGHAGHALREELDARKAAFCYIPCAAQTLNLIIQDGLKTLRPTLTKIREFVREMNAS<br>PELAQDFRQLTAAYQEGAWQLPLDASPRWTGDYAMLDIARRAPSSMEVLSRKHEEVLGGKGQQLLSATEKSLVGFLHS<br>SFEPFYKVSANLCSCKVPTVGLVLFFMDHVFEIVSAFRESCRQEWLKAAADGMAERARSFSGTVYSAFTYMAAVLDPRIK<br>KDLIPENLNSEKNLEEARSYFSRNYAAGQFPSAANGYATAAAAAAQDGEAVSSVSFAEEIARKRRRLSVVPAADELTQYL<br>SEPPVPIATDVLEWWKVNSSRYPRLSVMARDYLAVQGTSVEPENVFSGKGDDLQRRRFCLPHGAVQAVLCIQSWVQSG<br>YKFKFRSVEVDFETLVDPHPLAAPEMAKSEKAT	zf-BED--Dimer_Tnp_hAT--	V
Spipo24G0000500	SpiZf-BED02_I	TALFFFFFGGFVVLGMVGIFDGDYHPLAIEAPPSTKRRGRKKSQVWEHFTVESVAGGSTRARCKRCDQTFAYSSGAKIA<br>GTSHLKRHIAMGSCPRIREEKKNQQQQQELALTPGSGGGGASSEPPAKRRYRSSGGGGGGLVGFDQEHCCEELAKMIA<br>MEEYPLHMVENPAFTAFVQGLQPRFKVPSFSALESEILAIYRREKQNLRQLVGGISSRVSLTVGLWTTAQTLGYVCLTGHF<br>IDGDWKLRRWMLSFTMVSSPHSENALSEVIGFSLSDWSLTTKLFTITLDNYCSSHDIYSANLRDHLSSKNTLVLKGQLFVV<br>RCYAHILSVIAQDLLASIHGVVYSIRESLKYVKASPEREQRFAEVHRRLGGGAAGDLRLDVQSRWDTTYLMLAGALELRHV<br>FAFLESSDADYNDAPSPEDWKKVEAVCGFIKLLYDSAMAITAVPEPTANLYFHEVWKIQLELARAAAGAGAAAGEDGGAA<br>KEMHEKFDKYWKDCSLVMAIAVVMDPRFKMKLVEFSFTKIYGDDAERYIKVVNDGIHDLFLDYVAQPLPLTPAYADQHSH<br>HAAAGGNSPEAGSLSSGDGLLDFDMYISATAVSHQGKSELDQYLEEALVPRGEGFEILEWWKLNELKYPTLAKMARDVL<br>AIPMSIVPMGSVFGDGGGSRLLDDYRSSLRPETVEALFCARDWLQHMPIKTEAPPPPSTAIVKTE	zf-BED--DUF-domain--Dimer_Tnp_hAT--	I
Spipo26G0011300	SpiZf-BED03_IV	MISPQKPSRPTRHPRAVDRAESMLTREDAETLYYSAGVAASMVREKDVCWEYCEKLEGNKVRCNFCQKILNGGISRLKH<br>HLSRVPSKGVNPCSKVRDEVSERVKTIIQSKEEGKEAANVKRQRLADGARSPSGNPLPAKAPSPPASAAPPPARFFPSH<br>ATTSGPQPAAKDVERCVAEFFFENRLDFGVARSASYQQMMEALGGAGFRGPSAEALKTTWLEKLKSEVNTKIKEIENEW<br>ATTGCTIIADTWTDNKSKAWINFFISSPSGTFFHRSVDASPYFKNAKHVSDLFDSVILGVGSENVVQIIVDDALSYMSVGNYI<br>MQKYGSIFWSPCASRCLGLILEDLCKVDWVGRCILQAQSVTKFIYNNAWVLALMRKFTGGQDIVRTSATRSASNFLSLQS<br>LVKQRSRLKQMFNSPEYSSSPYASRPHGIACVDVLDDNQFWRVAEELAAVSEALLKVLREVSGGKPAVGSIYESMTRAK<br>ESIRTYYIMDETKCKTFLDVVDKRWQHQLHSPIHAAAAYLNPSIQYNPEVKFLGSIKEEFLAVLDKLLPTPDLRQDITTQIFR<br>FRKAQGMFGSNLAREARSTAFPGRLPLPCLIETSPAPSLRCPHRAPP	zf-BED--DUF-domain	IV
Spipo2G0037800	SpiZf-BED04_XXII	MAEDDCCRVLDQESHPHVPKQRKLRSAVWNDMTKQPRGDGSYFAICNHCKKKMLGSSAMGTTHLKNHLRFCAAFKSA<br>KSGQTAPVEASNGEDRAQDLGERLSLPKRRKLRSAVWNEMTKEHRGDGSYVAVCNHCKKRMVGNSASGTTHLKNHLR<br>LCASFRNSGGGPADVGKFFSECGEKRKPEAGRLDCFGYDPDRGKQDFVTTSEDQSAGQYQEPRLRLPRRRKLRSAVW<br>NDMTKEQREDGSYFAICNHCKKKMVGNSTRGTTHLKNHLRFCAAFKRSKCGQTDVGQLFLEAGEFRRAGGSSPDCFKF<br>DPERSRQDFARMLILHDYPAAIVDHVGFRTFLRNLQPQFRLVPRDAVQADCMGIYRDEREKLQEALARARSRISLAGGLW<br>VTAGGSEHLSLTCHFVDDDWKLQKKTLNFVELKSGRDSSEISKAIEEEVAGWQITGKLFGLVLDDSLGKEGLVQDLVYSR<br>FGSELLLDPELFPMQCWARILDLIVQDGLKVEEVSGIIERIRGSVEHVGKTRSRRRRFQRCAQQFGGPGRPLLLDSPARW<br>QTTFSMLETALEYRDAFSHLPGLDRSYGLPPSAADWDTVRAIVDCVSLFHEATSRFSCSKRPAINFCFVDLCSVYVKLKE<br>WCNSLSPCIQAMAARMFEEFAGRLNGANLLLVIGSILDPRFKMKSVDYLFAQLYFDDFSQRIYDVHEAFLKLYKAYSAQYE<br>RAVMSQASETPADNGSPRGEQHHQPGSAPPSGKNTLDFMRKGLGRLLLETSLVQPRKSDVDLYLEEDVIVSDDDHFDVL<br>RWWMQHAERYPVLAMMARDILAVPVSVAASKSASGAQNMAVDHHLSSLDPHAAQSLLCAQDWLRGEIEESLQDGSAE<br>GLSSIFTLINLTNEAGEVDEEESGSEESDSPVFKLG	zf-BED--zf-BED--zf-BED--DUF-domain--Dimer_Tnp_hAT--	XXII
Spipo3G0068200	SpiZf-BED05_I	FMAFYQCLIVGQAWKLVPHRWVHLFIFIMEGVSDTEPLALEAAPTSRRRRKRSLVWEHFTIETVSDGCTKALCKQCNQSF<br>AYSTGKKIAGTSHLKRHITVGSCSKLRQEKRQLALTSGSKTDGNSTDPPKRRYRWRTSLNFDQDRSCLDLAKMIIMQEYP<br>LHMVEHTAFKAFIQGLQPQFKMPDHEAIEKEILGIYKKEKQSLTQLFGTIPGRVCLSIGLWTTSQTLGYVSVTGHFIDNDWK<br>LHSRVLNFSMVSSPHSENALSDVIGLCLSDWNIKSKLFTITLDNNCSSHDIYSANLRDHLSNKNTLTLKGQLFVVRCYAHLL<br>NVAAQDVIASIHGIIYNIRESLKYVKASLVREERFAEIVQQLEIPCTRPLSLDVQSHWDTTYLMLAGALEFRHAFAYLETCDP<br>HYNEAPSPDDWKKVDTVCTYLKLLYDSANVIMGAADPTANMYFHEAWKIHLELANAAVGEDGTVCSVAKEMHEKFDKY<br>WKDCSLVLAIAVVMDPRFKMKFVEFAFSKLYGEDAGRYIKVVDDGIHGLFDEYITQPISLAPAYEDHQRDLDNGVATPEGS<br>PQEHAPPTGDRLVDFDIYISEISVTQQTKSELEQYLEEALMPRIQGFDILNWWRLNSLKYPVLSRMAQDVLAIPMLTMPTS<br>SIFNTGSGSRLLDEYRSSLLPETVEALFCSRDWLQYAPIPEAPSTAVVKLE	zf-BED--DUF-domain--Dimer_Tnp_hAT--	I
Spipo4G0087300	SpiZf-BED06_III	MSEPIVKAEDDGNPQISHVTPRQRKLRSVVWNDFTKELKEDGSDIATCNHCKRKFTGASRSGTTHLRNHLRFCASYERA<br>KRKKLDIVRAQPLQLDDKTASAIVVHGVDGNALLQVEDGNSQIHEDGNAMEFEDEAGNAQLSNVTPILRCRKLRSLVWND<br>FSKEQREDGSLVAICNHCKKQMAGGSRSGTTHLKKHLGKCLLYQRMKRRKGEMGQIHSKWGDAEIDENVDLDNSLLDQ<br>ELVQQDLAHMIILHEYPFSIVNHVGFRAFVRDLQPQFRLMSSDALQESCLKVYEDRRLVLSEELGKLCCRVNLTVDVWSS<br>GSNVGYLHLRCHFIDDDWKLHHKVISFWHVDSPHESGIAKLILEKLCEWSIDGRVFSTFLENSATSDKVVDELLQLLRPKQ<br>ALLLDGALFHVHSCSHILNLIVQDGLEVISELANKVRYSVNFVRSSQARLESFQAAAKVVGAPQKPLLIDDPSRWHTTYLMI<br>ATACEFQDAFVSLAEGDGEFVHVPSPEDWESIKAINECLGAFYLMTEKASPGKSPTVNLCFNSMCGIHLSLKTWSGGSHP<br>LVSSMASKMLEQFEKYSGVTSIILAAASVLDPRYKMKSIEYFFRLIYTDGSEAELKIESVLQDFRSLYDAYAAKSGRSSGGH<br>GLSCKLVGGGDDDDDGTDDDDGTGGGVDCGNNGTSKASSQNPMADLRFGLDQYLQETSSSQPIKSDLDLYLEEAVHP<br>SLKGLDDDFDILAWWKSNAPKYPVLSEMARDILSIPVSIASPESGFRTKVLHHYHSSMDPVTLQGLVCAQDWLRSEIDESS<br>LNGFADGLSTIFTLDRWC	zf-BED--zf-BED--DUF-domain--Dimer_Tnp_hAT--	III
Spipo6G0073300	SpiZf-BED07_I	MASPPGPDIILPLSSQKHDPAWKHCHMIKRGDRVHLRCMYCHKLFSGGGIHRIKEHLACQKGNASSCPKVNMEVRRAMQ<br>QSLDGVVMRRRKKQKLAEEVKKLNPVKEADAADGHSEAEMGLQLVAMPISVEPEAVQLEKREEGAVARTHERKRRRAA<br>KNLSPPSRENGFVRANRGGAVDGDQVCLAIGRFLYEAGVPLDAVNSAHFQPMIDAIAAAGPGFKAPSYHDLRGWVLKNS<br>VEEMKGTVDFYKETWTRTGCSILADEWTTEADQTLINFMVYCPEGVMFLKSVDASQMVTSADALYELLKLVVEEVGVGN<br>VLQVITSNTETHALAGKRLTEAFPSMFWTPCASRCVDAMLEDIGKLEPASIILEHARSITRFVYNHAEVLNMVRRFADGKEL<br>VRPGRSPSETSFMTLKAVAALKENLTSMVGSEDWKNCSCSKNEAAAAMAELICSPEFWSSVDSLVQLTDPLVQVLRMVD<br>SIKRPAMGYIYRGLHRAKEVMRRVLKRKKDYMPYWNIIDWRWHKELPRPLQAAGFFLNPQFFYGIKGDVPNEIMTGVLDC<br>IERMVPEMKSQDKINKELTAYKNATGDFGRKMAIRARHSLLPGEWWSTYGGGCPNLARLAIRILSQTCSARGCERSRIPF<br>EQMHSQRKNHLEHQRLSDLIYVRYNLRLQQRHLLRNRWADPLSAINGVEDWTVERTGLFTREDEESSWVELIQPAPASE<br>AAHTDEDDAFFEIDGEEFNGCKEEEEEDDDDDDVKQEMYGFVKEEE	zf-BED--DUF-domain--Dimer_Tnp_hAT--	I
TCA.XM_007009203.1	TcaZf-BED01_I	MASNLEPIPITSQKHDPAWKHCQMFRNGERVQLKCIYCGKIFRGGGIHRIKEHLAGQKGNASTCFHVPSDVRLLMRESLD<br>GVEVKKRKKQKIAEEMSNANQVSSEIDTYDNQVDTNTGLLMIEGPDTLQPSSSLLVNREGTSNVSGDRRKRGKGKSSAA<br>ESNALVVNTVGLGAKRVNNHVHVAIGRFLFDIGAPLDAVNSVYFQPMVDAIISGGSGVLMPSCSDLQGWILKKSVEEVKS<br>DNDKVTAAWVRTGCSILVNQWNTQTGRILLNFLVYCPEGTVFLKSVDASSVINSSDALYELLKQVVEEVGSKHVLQVITNA<br>EEQYIVAGRRLAETFPTLYWTPCAAHCINLILEDFAKLEWINVIIEQARSITRFVYNHSVVLNMVRRYTLGNDIVEPAVTCSA<br>TNFTTLKQMIDLKNNLQAMVTSQEWMDCPYSKKPGGLEMLDLVSNPSFWSSSVLITQLTNPLLRVLRMVGSKKRPAMGY<br>VYAGMYRAKETIKKELVKRNEYMIYWNIIDHWWEQQWHHPLHGAGFYLNPKFFYSMEGDMPNEMLSGMLDCIEKLVPD<br>VKVQDKISKEINSYKNTVGDFGRKMAVRARDTLLPAEWWSTYGGSCPNLARLAIHVLSQTCSTLGLKQNSIPFEKLHETR	zf-BED--DUF-domain--Dimer_Tnp_hAT--	I

		NFLEQQRFRDLIFVQCNLQLRQIGCESKEQVSMQPMSFDATIEDWVMGNDAFLENYTHSDWTALDPLSVNTMLLGPSSDEVEELGAGFDDYEIFNGVKEQENAEDNVVG		
TCA.XM_007010687.1	Tca Zf-BED 02_I	MDQSVENCINVDDDVNILNEDEIPSQSHISESEQLNKRVKKETSNVWNYFTKIGKKQDGVERATCNGCKTEYKVGPKPGGSNYGTSHLRRHIDTCKFISYFNPHQMLIDYEGKVKARKFDPRISRDMLAEAIIKHDLPYAFVEYDKIRAWAKYVNPDVVMPSRNTTVSDVQRIHLREKEKLKQAMAKVPNRICLTSDVWTASTSEGYICLTAHFVNKNWKLCSKLLNFCRMPPPHTGVELAATIFDCLKEWGIDRKVFSLTLDNASANDNMQGVGLRLDASTRWNSTYLMFESAIKYQKAFASLQFVDRTYKYNPSDKEWGRAMIICEFLEPFYETINLISGSSYPTSNLYFMQVWKIESILNENLHNEDEVIKDMSQRMKMKFDKYWKDYSVVLAFGAILDPRMKLDFLRFCYSKIDASTCHEKLENVKTKLYELFEQYASNTSASSTSSHSTSNLPKQAGRGTKPKGLKIFSDNAKRFPDLSVMARDVLNISITTVASESAFSISGHVLTKFRSSLHHENVQMLVCTKNWLHGFSLAADDDDSELETSLLSKQDSNV	zf-BED--DUF-domain--Dimer_Tnp_hAT--	I
TCA.XM_007018892.1	Tca Zf-BED 03_I	MGIWLSASTHHLWYKFYHTTTFAVLPSALSFLLLDPLETRSIFGSTGKSLNTFNSNFVEKIKNVTIMDQSVENYINMDDDVNILNEDEILSQSHISESEQLNKRAKKETSNVWNYFTKIGKKQDRVERATCNGCKTEYKVGPKPGGSNYGTSHLRRHIDTCKFISYFNPHQMLMDYEGKVKARKFYPRISRDMLAEAIIKHYLPHAFVEYDKIRAWAKYVNPDVVMPSRNTAVSDVQRIHLREKEKLKQAMAKNLHNEDEVIKDMSQMMKMKFEKYWKDYSVVLTFGAILDPRMKLDFLRFCYSKIDASTCHEKLENVKTKLYELFEQYASNTGASGTFSHSTSNLPKQAGGGTKPKGLKIFSEFKMFQNETISIARKFEFDVYLGEAKLDYEVFEDLNVLNYWKDNAKRFPDLSVMARDVLSISITTVASESAFSIGGHVLTKFRSSLHHENVEMLVCTKNWLHGFSLAADDDDSELETSLLSK	zf-BED--DUF-domain--Dimer_Tnp_hAT--	I
TCA.XM_007019544.1	Tca Zf-BED 04_II	MESDNINVSFESNVYFLEEFDDCLQIEQLGATDEKKPCQPKKRKLTSKLWTFFERLPEKNSSDGKSKVKCKLCGYILNYESKYGIGNLKRHNDNCVRKDTRDIGQMIFSKEHNSMLMRSSKFDLEKFRELVVAAIVMHNLPLSFVEYTGIKSMLPYLREDVVLISRNTVKADIIKYLCLTAHFVNKNWVLQKRILNFSFMPPPHNGVALSEKIYALLVEWGIESKLFSITLDNASANDTFVDLLKVQLIMRKQLLGRGKFFHIRCCAHILNLIVQDGLKEIDSAIQKVRESIKYVAGSQGRKQKFLECVSLVNLNAKRGLKQDVPTRWNSTLLMLESALYFRLTFSHLEISDSNFKHSPSRDEWDRIEKLSKFLSVFYEITSVMFDPWYKIQFVEWSYTKLYGSDSAEFKKVKDHLFALYDEYAVEVPNTPSALNDTPFDEKNVHKGKNEFLKEFDNFQPYVETDMNSLTADVINLEISKEEMSSVECSNTVDVN	zf-BED	II
TCA.XM_007021936.1	Tca Zf-BED 05_I	MEVANESAIKKPKRLTSVVWNHFERVRKADVCYAVCVHCNKKLSGSSNSGTTHLRNHLMRCLKRSNYDVSQLLAAKRRKKDNTLTIANISYDEGQRKEDYIKPTIVKYEQDQRKDEVFNLGSSRFDQERSRLDLARMIILHGYPLAMVEHVGFKVFVKNLQPLFDLVPNSTIELFCMEIYGKEKQKVYDMLSKLQGRINLAVEMWSSPENSNYLCLTAHYIDDDWKLQKKILNFVTLDSSHTEDLLSEVIMKCLMDWDIECKLFAMTFDDCSTNDDIVLRIKEQISENRPRLSNGQLLDVRSAAHILNSLVQDAVEALQVVIQKIRGSVRYVKSSQSIQGKFNEIAQQTGIISQKSLVLDCPIRWNSTYVMLETAVEYRNAFCHLPELDPDLALSDDEWEWASSVTGYLKLFIEIINVFSGNKCPTANIYFPEICHVHIQLIEWCKSPDNFLSSLAAKMKAKFDKYWSKCSLALAVAAILDPRFKMKLVEYYYSQIYGSTALERIKEVSDGIKELFNAYSICSTLIDEGTALPGSSLPSSSNDSRDRLKGFDKFLHETAQSQSAISDLEKYLEEAVFPRNCDFNILNWWRVHTPRYPILSMMARDVLGTPMSTVAQESAFNAGGRVLDSCRSSLTADTRQALICTRDWLWMQSDDPSPSSSHYALPLYVEAN	zf-BED--DUF-domain--Dimer_Tnp_hAT--	I
TCA.XM_007021937.1	Tca Zf-BED 06_I	MEVANESAIKKPKRLTSVVWNHFERVRKADVCYAVCVHCNKKLSGSSNSGTTHLRNHLMRCLKRSNYDVSQLLAAKRRKKDNTLTIANISYDEGQRKEDYIKPTIVKYEQDQRKDEVFNLGSSRFDQERSRLDLARMIILHGYPLAMVEHVGFKVFVKNLQPLFDLVPNSTIELFCMEIYGKEKQKVYDMLSKLQGRINLAVEMWSSPENSNYLCLTAHYIDDDWKLQKKILNFVTLDSSHTEDLLSEVIMKCLMDWDIECKLFAMTFDDCSTNDDIVLRIKEQISENRPRLSNGQLLDVRSAAHILNSLVQDAVEALQVVIQKIRGSVRYVKSSQSIQGKFNEIAQQTGIISQKSLVLDCPIRWNSTYVMLETAVEYRNAFCHLPELDPDLALSDDEWEWASSVTGYLKLFIEIINVFSGNKCPTANIYFPEICHVHIQLIEWCKSPDNFLSSLAAKMKAKFDKYWSKCSLALAVAAILDPRFKMKLVEYYYSQIYGSTALERIKEVSDGIKELFNAYSICSTLIDEGTALPGSSLPSSSNDSRDRLKGFDKFLHETAQSQSAISDLEKYLEEAVFPRNCDFNILNWWRVHTPRYPILSMMARDVLGTPMSTVAQESAFNAGGRVLDSCRSSLTADTRQALICTRDWLWMQSDDPSPSSSHYALPLYVEAN	zf-BED--DUF-domain--Dimer_Tnp_hAT--	I
TCA.XM_007021938.1	Tca Zf-BED 07_I	MEVANESAIKKPKRLTSVVWNHFERVRKADVCYAVCVHCNKKLSGSSNSGTTHLRNHLMRCLKRSNYDVSQLLAAKRRKKDNTLTIANISYDEGQRKEDYIKPTIVKYEQDQRKDEVFNLGSSRFDQERSRLDLARMIILHGYPLAMVEHVGFKVFVKNLQPLFDLVPNSTIELFCMEIYGKEKQKVYDMLSKLQGRINLAVEMWSSPENSNYLCLTAHYIDDDWKLQKKILNFVTLDSSHTEDLLSEVIMKCLMDWDIECKLFAMTFDDCSTNDDIVLRIKEQISENRPRLSNGQLLDVRSAAHILNSLVQDAVEALQVVIQKIRGSVRYVKSSQSIQGKFNEIAQQTGIISQKSLVLDCPIRWNSTYVMLETAVEYRNAFCHLPELDPDLALSDDEWEWASSVTGYLKLFIEIINVFSGNKCPTANIYFPEICHVHIQLIEWCKSPDNFLSSLAAKMKAKFDKYWSKCSLALAVAAILDPRFKMKLVEYYYSQIYGSTALERIKEVSDGIKELFNAYSICSTLIDEGTALPGSSLPSSSNDSRDRLKGFDKFLHETAQSQSAISDLEKYLEEAVFPRNCDFNILNWWRVHTPRYPILSMMARDVLGTPMSTVAQESAFNAGGRVLDSCRSSLTADTRQALICTRDWLWMQSDDPSPSSSHYALPLYVEAN	zf-BED--DUF-domain--Dimer_Tnp_hAT--	I
TCA.XM_007021939.1	Tca Zf-BED 08_I	MEVANESAIKKPKRLTSVVWNHFERVRKADVCYAVCVHCNKKLSGSSNSGTTHLRNHLMRCLKRSNYDVSQLLAAKRRKKDNTLTIANISYDEGQRKEDYIKPTIVKYEQDQRKDEVFNLGSSRFDQERSRLDLARMIILHGYPLAMVEHVGFKVFVKNLQPLFDLVPNSTIELFCMEIYGKEKQKVYDMLSKLQGRINLAVEMWSSPENSNYLCLTAHYIDDDWKLQKKILNFVTLDSSHTEDLLSEVIMKCLMDWDIECKLFAMTFDDCSTNDDIVLRIKEQISENRPRLSNGQLLDVRSAAHILNSLVQDAVEALQVVIQKIRGSVRYVKSSQSIQGKFNEIAQQTGIISQKSLVLDCPIRWNSTYVMLETAVEYRNAFCHLPELDPDLALSDDEWEWASSVTGYLKLFIEIINVFSGNKCPTANIYFPEICHVHIQLIEWCKSPDNFLSSLAAKMKAKFDKYWSKCSLALAVAAILDPRFKMKLVEYYYSQIYGSTALERIKEVSDGIKELFNAYSICSTLIDEGTALPGSSLPSSSNDSRDRLKGFDKFLHETAQSQSAISDLEKYLEEAVFPRNCDFNILNWWRVHTPRYPILSMMARDVLGTPMSTVAQESAFNAGGRVLDSCRSSLTADTRQALICTRDWLWMQSDGACIIFDLFAQSFLIHYIYLTISYSYYCNRPQSIF	zf-BED--DUF-domain--Dimer_Tnp_hAT--	I
TCA.XM_007021940.1	Tca Zf-BED 09_I	MEVANESAIKKPKRLTSVVWNHFERVRKADVCYAVCVHCNKKLSGSSNSGTTHLRNHLMRCLKRSNYDVSQLLAAKRRKKDNTLTIANISYDEGQRKEDYIKPTIVKYEQDQRKDEVFNLGSSRFDQERSRLDLARMIILHGYPLAMVEHVGFKVFVKNLQPLFDLVPNSTIELFCMEIYGKEKQKVYDMLSKLQGRINLAVEMWSSPENSNYLCLTAHYIDDDWKLQKKILNFVTLDSSHTEDLLSEVIMKCLMDWDIECKLFAMTFDDCSTNDDIVLRIKEQISENRPRLSNGQLLDVRSAAHILNSLVQDAVEALQVVIQKIRGSVRYVKSSQSIQGKFNEIAQQTGIISQKSLVLDCPIRWNSTYVMLETAVEYRNAFCHLPELDPDLALSDDEWEWASSVTGYLKLFIEIINVFSGNKCPTANIYFPEICHVHIQLIEWCKSPDNFLSSLAAKMKAKFDKYWSKCSLALAVAAILDPRFKMKLVEYYYSQIYGSTALERIKEVSDGIKELFNAYSICSTLIDEGTALPGSSLPSSSNDSRDRLKGFDKFLHETAQSQSAISDLEKYLEEAVFPRNCDFNILNWWRVHTPRYPILSMMARDVLGTPMSTVAQESAFNAGGRVLDSCRSLCICRCG	zf-BED--DUF-domain--Dimer_Tnp_hAT--	I
TCA.XM_007021941.1	Tca Zf-BED 10_I	MEVANESAIKKPKRLTSVVWNHFERVRKADVCYAVCVHCNKKLSGSSNSGTTHLRNHLMRCLKRSNYDVSQLLAAKRRKKDNTLTIANISYDEGQRKEDYIKPTIVKYEQDQRKDEVFNLGSSRFDQERSRLDLARMIILHGYPLAMVEHVGFKVFVKNLQPLFDLVPNSTIELFCMEIYGKEKQKVYDMLSKLQGRINLAVEMWSSPENSNYLCLTAHYIDDDWKLQKKILNFVTLDSSHTEDLLSEVIMKCLMDWDIECKLFAMTFDDCSTNDDIVLRIKEQISENRPRLSNGQLLDVRSAAHILNSLVQDAVEALQVVIQKIRGSVRYVKSSQSIQGKFNEIAQQTGIISQKSLVLDCPIRWNSTYVMLETAVEYRNAFCHLPELDPDLALSDDEWEWASSVTGYLKLFIEIINVFSGNKCPTANIYFPEICHVHIQLIEWCKSPDNFLSSLAAKMKAKFDKYWSKCSLALAVAAILDPRFKMKLVEYYYSQIYGSTALERIKEVSDGIKELFNAYSICSTLIDEGTALPGSSLPSSSNDSRDRLKGFDKFLHETAQSQSAISDLEKYLEEAVFPRNCDFNILNWWRVHTPRYPILSMMARDVLGTPMSTVAQESAFNAGGRVLDSCRSSLTADTRQALICTRDWLWMQSDGACIIFDLFAQSFLIHYIYLTISYSYYCNRPQSIF	zf-BED--DUF-domain--Dimer_Tnp_hAT--	I
TCA.XM_007022827.1	Tca Zf-BED 11_IV	MDATDKCDDSVNYNCYKTRSQSSAFVGSSSQQIPIIELSRTPASGSQALISPTGSKPPLCSSSAKRHRKLTSEVWNHFEKKNIDREDVASCNYCKTILKANSKNGTTALKSHMNTCLEIFYDLTNIFSGIEYLTANYFFVKVRKLRIAIVKWMCSDDVTISATAKKMFDKFEKY	zf-BED--DUF-domain	IV
TCA.XM_007023515.1	Tca Zf-BED 12_I	MASSEASINVHDHGKAVDGKKQRVQCNYCGKEMSGFFRLKYHLGGVRGDVIPCEMVSEDVKELFKNMLPERGGRLSQEVRDLSRQDLPWKRNGCPNSNVAKKMRRQSCKSSGSRSGEDEIIDSMSEDDVKEPAILPSARIVSQSAVTGDPEEEPSCKQNKRCIGRFFYETGIDLTLVNSPSFQRMINDTHCPGQTNYKIPSCQELKGWILKDEVKEMQEYVEKIRQSWASSGCSILLDGWIDEKGRNLVSFIVDCPQGPIYLHSSDVSASVDDVDALQLLFDRVIDDVGVENVVQIIAFSTEGWVGAVGKQFMGRSKTVFWTVNASHCIELMLDKIAMMGEIRGTLENARTISKFIHGHLTVLNLLRDYTDGHDLIKPTKVRSAMPFVTLENIIAEKKNLKAMFASSEWNTSAWASRAEGKRVADLVGDPSFWKGAGRVVKTALPLIRVLCLINGDDKPQMGYIYETMDQMKETIKKECNSKESQYMPFWELIDKIWDGHLHSPLHAAGHFLNPSLFYSTDFQSDSEVAFGLLCCMVRMIQSQPIQDKIVQQLEAYRNSEGAFGEGSTVQQRTRFSSTMWWSTYGGRCPELQRFATRILSQTCVGASKYRLNRSLVEKLLTKGRNPVEQQLLSDLIFVHYNLQLQQQQRSQFGVNYDIAGDEIDAMDEWIVDDTPEIGSRDGDSAWKELDGAVNGGRPSSQVKEEYRQV	zf-BED--DUF-domain--Dimer_Tnp_hAT--	I
TCA.XM_007	Tca Zf-	MYDILEYVCLEYELPLGLTWTSWANIKPLPTEIMPDSYKKGTLFIPYHAFHDSDDQASQKFMDTCDAHELVEGQAIAGKLLQSNESLFIQDITELDGSEYPFAAAAREFGSRAALAISFCNRYVGDDVYVLEFYFPSSKKNLEDKELLKDRILHNLENMKKKFVAPRVVNGTGSVGVRKQTISNSIPVVTLATRTSLPVPASATCHNLFNSNETRSLNTTDAKDHHHVGTENLQAKQGAKKKN	zf-BED--DUF-domain--	I

033168.1	BED 13_I	RRDINPEMVKENRGTLTVPHTKRRKLVSKVWEHFTKFEENALQWAECKYCNKKFTGSSKSGTTHLKNHLERCPIKKIKFPTQISSDLTSPGANEGNSIFDPERTRLEFATTIIKNQCLLDVVEDECYKNFLRTLQPMFELQSRESILSDIDRIYKEEKKKLRQYFDQLGCNYSLAIRFGEDNLKKNVYCCLVAYFINDDWELKKNIIAFKLLDRVYDSQTVSGIIRSSLLEWNISKKICSITVNSLGLSDDIVEQIKENCLPAVGQASLPSGGCYNSCTLIEDGLHEIDDLLLKIRKLIEYVSEKPSERLKFQDAVNQLKLLGGKSRDDCPLRLDSGFEMLDWALESREVFFQVEQSDDNFGIILSKEEWDKALLMHSCLKESLSCFGGIDQSLTANVYFPKLCGMYRKFLQLEKTNYPRMKLMKMKFDNYWTTHHLVFAIATVLDPRLKFKFVEVMYGEIYGRNSKMELNKFHKLLMDVYDNYANELDNQISPTAIFGDSSCSTTQSADDSILKSFWRYASKKKFDDVASWKSELDCYLEEPLLVPLDNNDEFFDILDWWRSNTLKFPRLGRMARDILAIRVSTAPPHSSFSTLITNPKNSDLGPEIIEAFVCGQDWLETPQTDNKSNHANVQIMVGTFVNLGTIVNF	Dimer_Tnp_hAT--	
TCA.XM_007033314.1	Tca Zf-BED 14_I	MDNFDQKLGPDFLKNLSGETISPLSVVIHEDIYESSSKRPKTTSKVWDVFEKLPAQQGDSKAICKLCRRIYTAKTTSGTSHLRRHIEACLKRGNHDLDQRSTEACFKPVNRDANRHTVSQGTLIDATTPLKSYKLDVDEIRRAIAMMIIVDAQPFRVVEDTGFRHVLNVACPEFPLLSRKAIKRDIISIYVRERENIRELLGACPGRICLTSSTWKSNCDDHYNCVTAHFIDHEWRLQKRILRFKLIPPPYDSLSIADEIGLCMVQWNIEHKVFSVTLENLSSDDCVADILKTRLDAKKYHPFKGVFFNMSCSTRILNLIVQAGFNLIIDIIGKLRLGIKYVQQSPHRKKNFYIIAKTLNLDTQKKLCLDSPSRWNSTYNMIEVALCYKNAFLYLAEQDKNFIHKLSEDEWEKVSVSYKFLKVIFEVACIFFRNRQPTSNLYFKALWKVHRRLSDMVRGPENFMTRMVKEMQSKFNQYWSEYNLILSCAAILDPRYKIKFVEYCYTKLYGSGAQQYVSASVNTLYGLFHDYMQNSACPSHTATLSVLTTKISNDKDDNDGFEDYETFQSARFQTQVEKSQLDLYLDEPSHDLNSEIDVLEYWTLCSLRYPELSRMARDVLTIPVSTIASDNAFDIGPQVISTDRSSLKSKMIQALVCLQDWMLASDKTRGSGSMESRTEDDSSSSSDGDDDY	zf-BED--DUF-domain--Dimer_Tnp_hAT--	I
TCA.XM_007033315.1	Tca Zf-BED 15_I	MDNFDQKLGPDFLKNLSGETISPLSVVIHEDIYESSSKRPKTTSKVWDVFEKLPAQQGDSKAICKLCRRIYTAKTTSGTSHLRRHIEACLKRGNHDLDQRSTEACFKPVNRDANRHTVSQGTLIDATTPLKSYKLDVDEIRRAIAMMIIVDAQPFRVVEDTGFRHVLNVACPEFPLLSRKAIKRDIISIYVRERENIRELLGACPGRICLTSSTWKSNCDDHYNCVTAHFIDHEWRLQKRILRFKLIPPPYDSLSIADEIGLCMVQWNIEHKVFSVTLENLSSDDCVADILKTRLDAKKYHPFKGVFFNMSCSTRILNLIVQAGFNLIIDIIGKLRLGIKYVQQSPHRKKNFYIIAKTLNLDTQKKLCLDSPSRWNSTYNMIEVALCYKNAFLYLAEQDKNFIHKLSEDEWEKVSVSYKFLKVIFEVACIFFRNRQPTSNLYFKALWKVHRRLSDMVRGPENFMTRMVKEMQSKFNQYWSEYNLILSCAAILDPRYKIKFVEYCYTKLYGSGAQQYVSASVNTLYGLFHDYMQNSACPSHTATLSVLTTKISNDKDDNDGFEDYETFQSARFQTQVEKSQLDLYLDEPSHDLNSEIDVLEYWTLCSLRYPELSRMARDVLTIPVSTIASDNAFDIGPQVISTDRSSLKSKMIQALVCLQDWMLASDKTSSMESRTEDDSSSSSDGDDDY	zf-BED--DUF-domain--Dimer_Tnp_hAT--	I
TCA.XM_007033316.1	Tca Zf-BED 16_I	MDNFDQKLGPDFLKNLSGETISPLSVVIHEDIYESSSKRPKTTSKVWDVFEKLPAQQGDSKAICKLCRRIYTAKTTSGTSHLRRHIEACLKRGNHDLDQRSTEACFKPVNRDANRHTVSQGTLIDATTPLKSYKLDVDEIRRAIAMMIIVDAQPFRVVEDTGFRHVLNVACPEFPLLSRKAIKRDIISIYVRERENIRELLGACPGRICLTSSTWKSNCDDHYNCVTAHFIDHEWRLQKRILRFKLIPPPYDSLSIADEIGLCMVQWNIEHKVFSVTLENLSSDDCVADILKTRLDAKKYHPFKGVFFNMSCSTRILNLIVQAGFNLIIDIIGKLRLGIKYVQQSPHRKKNFYIIAKTLNLDTQKKLCLDSPSRWNSTYNMIEVALCYKNAFLYLAEQDKNFIHKLSEDEWEKVSVSYKFLKVIFEVACIFFRNRQPTSNLYFKALWKVHRRLSDMVRGPENFMTRMVKEMQSKFNQYWSEYNLILSCAAILDPRYKIKFVEYCYTKLYGSGAQQYVSASVNTLYGLFHDYMQNSACPSHTATLSVLTTKISNDKDDNDGFEDYETFQSARFQTQVEKSQLDLYLDEPSHDLNSEIDVLEYWTLCSLRYPELSRMARDVLTIPVSTIASDNAFDIGPQVISTDRSSLKSKMIQALVCLQDWMLASDKTRGSGSMESRTED	zf-BED--DUF-domain--Dimer_Tnp_hAT--	I
TCA.XM_007033790.1	Tca Zf-BED 17_IX	MSSSALDALVACAKAIQDENLTVADSLLERIWNLAAAQSWPGESDVVKYFAEALVRRAYGISSASANFNLLSPPPIYFLDNFSCDAINTACMGKKRFHLITFLFLPSDDWTYLFRSLANASGNFLSVRVSVIVSPFLEKIVKIQQEKSKHDLTTAAMERGIKLEDLRVVYANSLGDVDASKADFTRTTDEAVIVYYRYKLHELLADVRVMERELLKLRQINPEIVIIEEQYADHNDSNFIKRLEKSFQYYFNRFDFYEVTYCRQIVNIVGCEGTDRLERHQTLAQWRSLLRANGLLPVPLAPDIWSGEHEDNGCVVFQNDDGLLHFTSAWKLTDAVDHFNPISYNPIQGFNPNPALEDTVRTLQVDRQASSLNGLAAFAEIYDMLEDVCLKYELPLALTWVKGTPNGIMSGLNKKRSLSIETAYSYINCCYYYYYDYYVEKISQYRSFMQECAIYDIQEGQAIAGQALQSNEPFLFEPNITELRSNPFAEAAQKSGLHAALAICLVNHYTDDVYILEFFLSSSEEKLEEPKSLALRIFEDLKKMKTKFVKLRVHGTEVGLQEEAIPNIPWEEMPMRSSSPATSNDQFLNSNASRSLNVVELKDRHVVEIQGPNGQEAATSNFHPAYLSIHASSMAGTEHFNATNLRSYNGLLETHEPQLQEITEKNWISQTISNIDHEIVKANRENSALPRTKQRKLVSKVWKEFTKFEENGKQLAKCNHCSKEFTGSSKSGTTHLKNHLERCPRKKNEYQERQLKLSVKTGDLTNRDTSEGNSMFDQEKSRLDLVKMIIKHQYPLDVAEQEFFKSFVQNLQPMFEFQSQATIISDIHHIYEEEKKKLQQCFAQFACKFSLTISLWKDNLRKNAYCCLIAHFVDDDWELRRKILVFKNLEHNYGTGSIIRVIQNSISEWNMSEKVCSISVDNSSLNNGILQQIKESCLSDQVSLPSCHYYSSCTLIQDGLHEIDDILLKLRKSIEYVTELEHGKLKFQEAINQVTLQGGKSTDYGPLRLDSNFSILDSALESRQIFCQLEQIDGHFKVNPSIEEWERALILHSYLKGFYDNLSSFRQTHSSTANTYFPQLCDMYKKFLQMEKKNYPFMMKRKFDDHWSLCNLVFAIAALLDPRLKFKFVEFSYGEIYGRDSKRQLKRFHRDLMDIYFEYAYEPRNRTTSASVGCLTRQSTESANDSILDSFSRYASASNFNEVSSRKSDLDCYLEEPLLHLDGAFFDVLDWWRVNSERFPTLGRMAHDLLAMPVLVVPPCSDFSAVITNPAHNGLNPETMEALVCSHNWLEMPKGNDRANHAPMQNTAKRKWEEKETREVKSCKNWNSEETNNADKAKASYKMLTRALPLENDRQEGRPLKSSEPNHGKDTSGLIEIPNGSPSFDNQSEFQCYSSDESDGEIAGREQGEWREDDVRRYLLLPLTEKGRKRLNKWRNHKMSGKLIGRDKEFGVLDYKLAPLLTVPHGVETQVKYYIDDSVVNTFFKLLKKRSDRFPKAYVSHYSFDSWIATYLIEGSRSESQVFSWFKDEKLKDVQILFLPACLSAHWVLFCVDTKKRTFSWLDSNISSRTSNVAEKQAILGWFKRLLLPAFGYQNANEWPFEIRSDIPEQKNGVDCGLFVMKYADCLTHGEFFPFTQQHMPYFRLRTFLDIYRGRLHSQ	GRAS--zf-BED--DUF-domain--Dimer_Tnp_hAT--Peptidase_C48--	IX
TCA.XM_007035922.1	Tca Zf-BED 18_III	MVKLMAPARSSVFADPGWEHGVPQDEKKKKVKCNYCGKVVSGGIYRLKQHLARVSGEVTYCDKAPEEVFLRMKGNLEGCRSTKKSRQSNTGGHAYFNFHSNVIEEEEEERISYKSKGKLFMENSNPGLNLTPLRSLGYVDPGWEHGVPQDERKKKVKCNYCEKIVSGGINRFKQHLARIPGEVAPCKNAPEEVYLKIKENMKWHRTGKRHKQPYEKEIPTFDVGPNDEDEEQEEEDHILHQKSKEKLKIGDHGLGKDLRKTFRELSSSSGSEPLQKKSRLDSVFLKGVSDTALSCKKVREKIGFGKKSSREVYSAICKFFYHAGVPLQAANSVYFHKMLELVGQYGHGLAGPSSQLISGYFLQEEIKTIKNYLVEYKASWAITGCSVMADSWVDTEGRTFVNFLASCPYGIYFVSSVDVTYILEDALNLFKLLDKVVEEVGEENVVQVITENTPTYKAAGKMLEEKRRNLFWTPCAIYCIDRMLEDFLKLKCVGECIEKGQKVTKFIYNNVWLLNLMKKEFTQEQELLMPSLTQFASSFATLQNLLDHRTNVKRMFQSNKWISCRFSKSDEGKEMEKIIVNVTFWKKVQYVCKSVNPVMQVLQKVYNDQGLSMPFAYNDMYRAKLAIKAVHDNDARKYGPFWSVIENHWSLLFHHPLHTAAYFLNPSCRYRPDFVTHAEMVRGLNESIARLEPDNARRISASMQISDFNSAKADFGTELAISTRTELDPAAWWQQHGISCLELQRIAVRILSQTCSSSGCEYKWSIYDQIHTLRHSRLAQKRLNDLTYVHYNLRLRENQLKKRSNNSVSLDSTSAEHLLHDWIAEAEKRSWQEDEEIRYGENGMAYEDNNENDGVDYEGGTPEARKGSMEHLSLADVESQSLDIDPATDDEDDGDLNYYNDDVSD	zf-BED--zf-BED--DUF-domain--Dimer_Tnp_hAT--	III
TCA.XM_007036356.1	Tca Zf-BED 19_I	MEWNSNNTFKTYKDMEPKAMMDMALIPNIDPVDIGLGSSEKGSVVPTSKPRKKTMTSVYLKYFETAPDGKTRRCKFCGQSYSIATATGNLGRHLSNRHPGYDKTGDVVTTSSVPQPTTPVIKKSQPQGRAAQVDYDHLNWLLIKWLILASLPPSTLEEKWLANSFKFLNPSIQLWPGEKYKAVFREVFRSMREDVRVSLEQVSSKVSVTLDFWTSYEQIFYMSVTCQWIDENWSFQKVLLDICQVPYPCTGSEIYNTLFKVLKMYNIENKVLSCTHDNSQNAIHACHTLKEDLDGQKVGPFCYIPCAARTLSLIIDDALRTTKPVIAKVREFVQELNASLDISEDFIQLTTAYQEGSWQFPLDASARWSGNYQMLDLVHKAGKSMDAVVRKNEEILGSRILLNGAEKNVANIVHNYLEPFYKVINEICVNNPPTIGMVIVYMDHISDTIATRQTPDWLKSAAEDMAKKLRSYNNQVCNIFIYMTAILDPRIKCELIPESLNSENYLEEARAHFMRNYYTSHFSSMTSGYSAQDIEDGGSVSFAEEIARKKRRASMSNVADELTQYLSESPAPTKTDVLEWWKVNSTRYPRLSAMARDFLAVQATSVKPEELFCSKGDEIDKQRFCMPHDSTQAILCIKSWTQGGLKLKYKSSEIDYERLMELAAAAAADNISAGFDKKQK	zf-BED--DUF-domain--Dimer_Tnp_hAT--	I
TCA.XM_007036814.1	Tca Zf-BED 20_II	MGTTEKNKDVCLFDSVMTNTILCGKHFFLSVTLREANVRSISSLVEIIDNFMNATIVLPNGTTLHIEDHYKVVDQRGIYLASKMYIVMSTNLRHFMSKIRNVSTSPLIRNVSTIFRNFRLGLGSVERVSSAGFSLFTETEIDQLGATNEKKPCQPKKRKLTSKLWTFFERLPEKNSSDGKSKVKCKLCGYILNYESKYGTSNLKRHNDNCVRKYTRDIGQMIFSKEHNSMLMRSSKFDPKKFCELVVAAIVMHNLPLSFVEYTGIKSMLSYLREDVVLISRNTVKAHIIKMHKREKCKIQSLLQESPGRICLTFDLWTSIVIDGYMCLTAHFVDKN	zf-BED	II
TCA.XM_007038871.1	Tca Zf-BED 21_I	MFMAVVREKDVCWEYAEKLDGNKVRCKFCLRVLNGGISRLKHHLSRLPSKGVNPCSKVRDDVTDRVRAILSSKEEIKETSSVKKQKIAEARSPGNISTCSKIIPLEASSPVAKVFPATSPIAPPSLNSQENVERSIALFFFENKLDFSVARSSSYQAMIDAVGKFGPGFTGPSVETLKTMWLERIKSEVCLQSKDTEKEWATTGCTIIADTWTDNKSRALINFLVSSPSRTFFHKSVDASSYFKNTKCLADLFDSVIQDFGPENVVQIIMDSSFNYTGISNHILQNYGTIFVSPCASQCLNLILEEFSKVDWVNRCILQAQTLSKFLYNNASMLDLMKKFTGEQELIRTGITKSVSSFLSLQSMLKQRSRLKHMFNSPEYSTNSSYANKPQSISCIAIVEDNDFWRAVDECVAISEPFLKVLREVSGGKPAVGSIYELMTRAKESIRTYYIMDEGKCKTFLDIVDRKWRDQLHSPLHSAGAFLNPSIQYNQEIKFLGSIKEDFFKVLEKLLPTPELRRDITNQIFTFTRAKGMFACNLAMEARDTVSPGLWWEQFGDSAPVLQRVAIRILSQVCSTFTFERHWSTFQQIHSEKRNKIDKEILNDLVYINYNLRLARQMRTKSVEADPIQFDDIDMTSEWVEESENPSPTQWLDRFGSALDGGDLNTRQFNAAIFGNDHIFGL	zf-BED--DUF-domain--Dimer_Tnp_hAT--	I

TCA.XM_007039899.1	Tca Zf-BED 22_I	MVRGRDACWEHCVLVDATRQKVRCNYCHREFSGGVYRMKFHLAQIKNKDIVPCAEVPDDVRDHIQTILNSPKKQKTPKK<br>PKVDKAVANDQQNSSSASGGLHLNHGSSGQHGSTCPSLLFPRPSPSEQPAVDDGQKQKQEDADKKIAVFFFHNSIPFSA<br>AKSMYYQEMVDAIAKCGVGYKAPSYENLRSTLLEKVKGDIHDCYKKYRDEWKETGCTILCDSWSDGRTKSFVIFSVTCPK<br>GTLFLKSVDVSGHEDDASYLFELLESVVLEVGLENVIQVITDTAASYVYAGRLLMAKYSSLFWSPCASYCINKMLEDISKQE<br>WVGIVLEEAKSIVQYIYSHAWIVNMMRKFTGGRELMRPRITRFVANYLTLRSIIIQEDNLKHMFSHSEWLSSIYSRRSDAQAI<br>KSLLYLERFWKSAHEAVSVSEPLVKILRIVDGDMPAMGYIYEGIERAKVAIKAYYKGLEEKYMPIWDIIDRRWNMQLHSPLH<br>AAAAFLNPSIFYNPNFKIDLRMRNGFQEAMLKLATTDKDKIEITKEHPMYINAQGALGTDFAIMGRTLNAPGDWWASYGYE<br>IPTLQRVAIRILSQPCSSHWCRWNWSTFESIHTKKRNKVELEKFNDLVFVHCNLCLQAICHSRDGKCKPVIFDEIDVSSVW<br>SSELEPSAPLLDDSWLDTLPLECRGSP	zf-BED--DUF-domain--Dimer_Tnp_hAT--	I
TCA.XM_007048761.1	Tca Zf-BED 23_I	MDMSDAVVANSSRLKSIVWNDFDRVKKGDTFVAICRHCKKKLSGSSTSGTSHLRNHLIRCQRRSNHGIAQYFSGREKKK<br>EGSLAVVTIDQEQKKDEVLSLVNLRYEQEQIKNEPVTIGNSSLDQRRSQFDLARMIILHNYPLDMVDHVGFKIFVRNLQPLF<br>ELVTYNKVEADCMEIYAKEKQRVYEVLDKFPGKISVTADVWTASDDSAYLSLTAHYIDEDWQLKKRTLNFVTIDPSHTEDM<br>HSEVIMTCLMDWDIDRKLFSMIFDSYTSENIVDRIRDRLSQNRFLYCNGQLFDVRCAVDLLNRMVQDALDAVCEVTQKIRE<br>SIRYVKSSEATQSMFIELAHEVQVESQKCLRIDNPLKWNSTFLMLEVALEYRKVFCCLQDRDPVNMKFLPSDLEWDRVSV<br>IASFLKLFVEVTNVFTRSKYPTANIFFPEICDIHLQLIEWCKNPDDYINSLAVKMRKKFEDYWDKCSLGLAVAAMLDPRFKM<br>KLLEYYYPQLYGDSASELIDDVFECIKSLYNEHSMVSPLASSLDQGLSWQVSGIPGSGKDSRDRLMGFDKFLHETSQSDG<br>SNSDLDKYLEDPLFPRNVDFNILNWWKVHTPSYPILSMMAHNILGIPISKVAAESTFDTGGRVVDHNWSSLPPTTVQALMC<br>SQDWIRSELES	zf-BED--DUF-domain--Dimer_Tnp_hAT--	I
TCA.XM_007048965.1	Tca Zf-BED 24_I	MELNLTPISITKQKQDPAWNHCEAFKNGERLQIKCMYCGKMFKGGGIHRFKEHLAGRKGQGPICEQVPPGVRALMQESL<br>NGVLLKQDNKQNAIPELLACGGSSPHAGEIDKSAYSDDVNNGVKPIQVLNSLEPDSSLVLNGKGEVSQGIRDSKKRGRDR<br>SLLANSHSCAKSDLALVSIGAENPVHMAIGRFLYDIGVNLDAVNSVYFQPMIDAIASTGSGIVPPSSQDLRGWILKNVMEEV<br>KDDIDRNKTMWGKTGCSILVEQWSPKSGRTLLSFLVYCPQATVFLKSVDASRVIFSADHLNELLKQVVEEVGVENVVQVIT<br>NCEEQYFLAGKRLMESFPSLYWAPCLVHCVDMMLEDFANLEWISETIEQAKSVTRFVYNHSVVLNMMRRFTFHNDIVEP<br>AVTRFASNFATLKRMADLKLKLQAMVNSQDWSECPYAKKPGGLVMLDIVKNRSFWNSCILIVRLIYPLLQVLEIVGSKKRS<br>TMGYVYAGIYRAKETIKKELVKKDDYMVYWNIIDHRWEQQRHIPLYAAAFFLNPKFFYSIEGNIHNDILSSMFDCIERLVPDT<br>NVQDQIVREIHLYKNATGDLGRPMAVRARDNLLPGEWWSMYGGGCPNLQHLAIRILSQTCSSIGSKPNKISIEEIHDTRNF<br>LEHQRLSDLVYVRYNLYLRQMVLRSQDKDSADPLSFNSKEIRDDWIAYNAVCEEDYGSSDWMSLDPPVGSRMLSGTSG<br>DETEDFLGTGFADLEIFNGLNGVEDI	zf-BED--DUF-domain--Dimer_Tnp_hAT--	I
TCA.XM_007049409.1	Tca Zf-BED 25_V	MSEVWKHFTKFINNQGESKARCNYCGRELSVNTKYNGTNALKNHMNSCEKFSSALDYIQIELAFQSDDGVALLNELKIYKI<br>AISREEDKSKLEKYLSLNEPDVTDNDDFNVLILWKFNNHRYLTLALQAHDILVIPPSIIASESVFSTGGWVRAAYRSSLSPK<br>MVQALICAQD	zf-BED--Dimer_Tnp_hAT--	V
TCA.XM_007051201.1	Tca Zf-BED 26_III	MVEEMAPLRSTGYVDPGWEHGIAQDERKKKVKCNYCGKIVSGGIFRLKQHLARLSGEVTHCEKVPEEVCLNMRKNLEG<br>CRSGRKRRQSEYEQAALNFQSNEYNDAEEASAGYKHKGKKVMGDKNLVIKFTPLRSLGYVDPGWEHCVAQDEKKKRV<br>KCNYCEKIISGGINRFKQHLARIPGEVAYCEKAPEEVYLKIKENMKWHRTGRRHRKPDTKEISAFYLHSDNEDEGGEEDG<br>YLQCISKDILAIDDKVSDSDIRNNNVRGRSPGSSGNGAEPLLKRSRLDSVFLKSLKSQTSAHYKQTRAKIGFEKKTRREVIS<br>AICKFFYHAGIPSNAANSPYFHKMLEVVGQYGQGLHGPSSRIISGRLLQEEIANIKEYLAEFKASWAITGCSVMADSWNDA<br>QGRTLINFLVSCPRGVCFLSSVDATDMIEDAANLFKLLDKAVDEVGEEYVVQVITRNTLSFRNAGKMLEEKRRNLFWTPC<br>AVYCIDRMLEDFLNIKWVGECIDKAKKVTRFIYNNTWLLNFMKKEFTKGQELLKPAVTKFGTNFFTLQSMLDQRVGLKKMF<br>QSNRWLSSRFSKLDEGKEVEKIVLNVTFWKKMQYVKKSLEPVAEVLQKIGSDEIRSMPFIYNDICRTKLAIKAIHGDDVRKF<br>GPFWSVIENNWSSLFHHPLYVAAYFLNPSFRYCPDFLMNPEVIRGLNECIVRLESDNGKRISASMQIPDFVSAKADFGTDL<br>AISTRSELDPASWWQQHGISCLELQRIAIRILSQRCSSIGCQHTWSVFDQVHSKRRNCLSRKRLNDHTYVHYNLRLRERQ<br>LGRKPDDLVSFDSAMLESVLDDWLVESEKQAMQEDEEIIYNEVEQFYGDDMDEHFIFDVPHQTWSAEGEGSVSATYSHV<br>VRTGVPKAHGGRRNRAFIQIPFLIIVLGTIGLGGLNGTRAYRKQKEECPSHNPFLP	zf-BED--zf-BED--DUF-domain--Dimer_Tnp_hAT--	III
TCA.XM_007051202.1	Tca Zf-BED 27_III	MVEEMAPLRSTGYVDPGWEHGIAQDERKKKVKCNYCGKIVSGGIFRLKQHLARLSGEVTHCEKVPEEVCLNMRKNLEG<br>CRSGRKRRQSEYEQAALNFQSNEYNDAEEASAGYKHKGKKVMGDKNLVIKFTPLRSLGYVDPGWEHCVAQDEKKKRV<br>KCNYCEKIISGGINRFKQHLARIPGEVAYCEKAPEEVYLKIKENMKWHRTGRRHRKPDTKEISAFYLHSDNEDEGGEEDG<br>YLQCISKDILAIDDKVSDSDIRNNNVRGRSPGSSGNGAEPLLKRSRLDSVFLKSLKSQTSAHYKQTRAKIGFEKKTRREVIS<br>AICKFFYHAGIPSNAANSPYFHKMLEVVGQYGQGLHGPSSRIISGRLLQEEIANIKEYLAEFKASWAITGCSVMADSWNDA<br>QGRTLINFLVSCPRGVCFLSSVDATDMIEDAANLFKLLDKAVDEVGEEYVVQVITRNTLSFRNAGKMLEEKRRNLFWTPC<br>AVYCIDRMLEDFLNIKWVGECIDKAKKVTRFIYNNTWLLNFMKKEFTKGQELLKPAVTKFGTNFFTLQSMLDQRVGLKKMF<br>QSNRWLSSRFSKLDEGKEVEKIVLNVTFWKKMQYVKKSLEPVAEVLQKIGSDEIRSMPFIYNDICRTKLAIKAIHGDDVRKF<br>GPFWSVIENNWSSLFHHPLYVAAYFLNPSFRYCPDFLMNPEVIRGLNECIVRLESDNGKRISASMQIPDFVSAKADFGTDL<br>AISTRSELDPASWWQQHGISCLELQRIAIRILSQRCSSIGCQHTWSVFDQVHSKRRNCLSRKRLNDHTYVHYNLRLRERQ<br>LGRKPDDLVSFDSAMLESVLDDWLVESEKQAMQEDEEIIYNEVEQFYGDDMDEHVSEEKRPTEMVTLASLVEPLDVNPA<br>AGGVTTDDDGLDFLDDDLTD	zf-BED--zf-BED--DUF-domain--Dimer_Tnp_hAT--	III
TCA.XM_007051204.1	Tca Zf-BED 29_III	MVEEMAPLRSTGYVDPGWEHGIAQDERKKKVKCNYCGKIVSGGIFRLKQHLARLSGEVTHCEKVPEEVCLNMRKNLEG<br>CRSGRKRRQSEYEQAALNFQSNEYNDAEEASAGYKHKGKKVMGDKNLVIKFTPLRSLGYVDPGWEHCVAQDEKKKRV<br>KCNYCEKIISGGINRFKQHLARIPGEVAYCEKAPEEVYLKIKENMKWHRTGRRHRKPDTKEISAFYLHSDNEDEGGEEDG<br>YLQCISKDILAIDDKVSDSDIRNNNVRGRSPGSSGNGAEPLLKRSRLDSVFLKSLKSQTSAHYKQTRAKIGFEKKTRREVIS<br>AICKFFYHAGIPSNAANSPYFHKMLEVVGQYGQGLHGPSSRIISGRLLQEEIANIKEYLAEFKASWAITGCSVMADSWNDA<br>QGRTLINFLVSCPRGVCFLSSVDATDMIEDAANLFKLLDKAVDEVGEEYVVQVITRNTLSFRNAGKMLEEKRRNLFWTPC<br>AVYCIDRMLEDFLNIKWVGECIDKAKKVTRFIYNNTWLLNFMKKEFTKGQELLKPAVTKFGTNFFTLQSMLDQRVGLKKMF<br>QSNRWLSSRFSKLDEGKEVEKIVLNVTFWKKMQYVKKSLEPVAEVLQKIGSDEIRSMPFIYNDICRTKLAIKAIHGDDVRKF<br>GPFWSVIENNWSSLFHHPLYVAAYFLNPSFRYCPDFLMNPEVIRGLNECIVRLESDNGKRISASMQIPDFVSAKADFGTDL<br>AISTRSELDPASWWQQHGISCLELQRIAIRILSQRCSSIGCQHTWSVFDQVHSKRRNCLSRKRLNDHTYVHYNLRLRERQ<br>LGRKPDDLVSFDSAMLESVLDDWLVESEKQAMQEDEEIIYNEVEQFYGDDMDEHFIFDVPHQTWSAEGEGSVSATYSHV<br>VRTGVPKAHGGRRNRAFIQIPFLIIVLGTIGLGGLNGTRAYRKQKEECPSHNPFLP	zf-BED--zf-BED--DUF-domain--Dimer_Tnp_hAT--	III
TCA.XM_007051205.1	Tca Zf-BED 30_III	MVEEMAPLRSTGYVDPGWEHGIAQDERKKKVKCNYCGKIVSGGIFRLKQHLARLSGEVTHCEKVPEEVCLNMRKNLEG<br>CRSGRKRRQSEYEQAALNFQSNEYNDAEEASAGYKHKGKKVMGDKNLVIKFTPLRSLGYVDPGWEHCVAQDEKKKRV<br>KCNYCEKIISGGINRFKQHLARIPGEVAYCEKAPEEVYLKIKENMKWHRTGRRHRKPDTKEISAFYLHSDNEDEGGEEDG<br>YLQCISKDILAIDDKVSDSDIRNNNVRGRSPGSSGNGAEPLLKRSRLDSVFLKSLKSQTSAHYKQTRAKIGFEKKTRREVIS<br>AICKFFYHAGIPSNAANSPYFHKMLEVVGQYGQGLHGPSSRIISGRLLQEEIANIKEYLAEFKASWAITGCSVMADSWNDA<br>QGRTLINFLVSCPRGVCFLSSVDATDMIEDAANLFKLLDKAVDEVGEEYVVQVITRNTLSFRNAGKMLEEKRRNLFWTPC<br>AVYCIDRMLEDFLNIKWVGECIDKAKKVTRFIYNNTWLLNFMKKEFTKGQELLKPAVTKFGTNFFTLQSMLDQRVGLKKMF<br>QSNRWLSSRFSKLDEGKEVEKIVLNVTFWKKMQYVKKSLEPVAEVLQKIGSDEIRSMPFIYNDICRTKLAIKAIHGDDVRKF<br>GPFWSVIENNWSSLFHHPLYVAAYFLNPSFRYCPDFLMNPEVIRGLNECIVRLESDNGKRISASMQIPDFVSAKADFGTDL<br>AISTRSELDPASWWQQHGISCLELQRIAIRILSQRCSSIGCQHTWSVFDQVHSKRRNCLSRKRLNDHTYVHYNLRLRERQ<br>LGRKPDDLVSFDSAMLESVLDDWLVESEKQAMQEDEEIIYNEVEQFYGDDMDEHVSEEKRPTEMVTLASLVEPLDVNPA<br>AGGVTTDDDGLDFLDDDLTD	zf-BED--zf-BED--DUF-domain--Dimer_Tnp_hAT--	III
TCA.XM_007051206.1	Tca Zf-BED 31_III	MAPLRSTGYVDPGWEHGIAQDERKKKVKCNYCGKIVSGGIFRLKQHLARLSGEVTHCEKVPEEVCLNMRKNLEGCRSG<br>RKRRQSEYEQAALNFQSNEYNDAEEASAGYKHKGKKVMGDKNLVIKFTPLRSLGYVDPGWEHCVAQDEKKKRVKCNYC<br>EKIISGGINRFKQHLARIPGEVAYCEKAPEEVYLKIKENMKWHRTGRRHRKPDTKEISAFYLHSDNEDEGGEEDGYLQCIS<br>KDILAIDDKVSDSDIRNNNVRGRSPGSSGNGAEPLLKRSRLDSVFLKSLKSQTSAHYKQTRAKIGFEKKTRREVISAICKFF<br>YHAGIPSNAANSPYFHKMLEVVGQYGQGLHGPSSRIISGRLLQEEIANIKEYLAEFKASWAITGCSVMADSWNDAQGRTLI<br>NFLVSCPRGVCFLSSVDATDMIEDAANLFKLLDKAVDEVGEEYVVQVITRNTLSFRNAGKMLEEKRRNLFWTPCAVYCID<br>RMLEDFLNIKWVGECIDKAKKVTRFIYNNTWLLNFMKKEFTKGQELLKPAVTKFGTNFFTLQSMLDQRVGLKKMFQSNR<br>WLSSRFSKLDEGKEVEKIVLNVTFWKKMQYVKKSLEPVAEVLQKIGSDEIRSMPFIYNDICRTKLAIKAIHGDDVRKFGPFW<br>SVIENNWSSLFHHPLYVAAYFLNPSFRYCPDFLMNPEVIRGLNECIVRLESDNGKRISASMQIPDFVSAKADFGTDLAISTR<br>SELDPASWWQQHGISCLELQRIAIRILSQRCSSIGCQHTWSVFDQVHSKRRNCLSRKRLNDHTYVHYNLRLRERQLGRK<br>PDDLVSFDSAMLESVLDDWLVESEKQAMQEDEEIIYNEVEQFYGDDMDEHVSEEKRPTEMVTLASLVEPLDVNPAAGGV<br>TTDDDGLDFLDDDLTD	zf-BED--zf-BED--DUF-domain--Dimer_Tnp_hAT--	III

TCA.XM_007051207.1	Tca Zf-BED 32_III	MVEEMAPLRSTGYVDPGWEHGIAQDERKKKVKCNYCGKIVSGGIFRLKQHLARLSGEVTHCEKVPEEVCLNMRKNLEGCRSGRKRRQSEYEQAALNFQSNEYNDAEEASAGYKHKGKKVMGDKNLVIKFTPLRSLGYVDPGWEHCVAQDEKKKRVKCNYCEKIISGGINRFKQHLARIPGEVAYCEKAPEEVYLKIKENMKWHRTGRRHRKPDTKEISAFYLHSDNEDEGGEEDGYLQCISKDILAIDDKVSDSDIRNNNVRGRSPGSSGNGAEPLLKRSRLDSVFLKSLKSQTSAHYKQTRAKIGFEKKTRREVISAICKFFYHAGIPSNAANSPYFHKMLEVVGQYGQGLHGPSSRIISGRLLQEEIANIKEYLAEFKASWAITGCSVMADSWNDAQGRTLINFLVSCPRGVCFLSSVDATDMIEDAANLFKLLDKAVDEVGEEYVVQVITRNTLSFRNAGKMLEEKRRNLFWTPCAVYCIDRMLEDFLNIKWVGECIDKAKKVTRFIYNNTWLLNFMKKEFTKGQELLKPAVTKFGTNFFTLQSMLDQRVGLKKMFQSNRWLSSRFSKLDEGKEVEKIVLNVTFWKKMQYVKKSLEPVAEVLQKIGSDEIRSMPFIYNDICRTKLAIKAIHGDDVRKFGPFWSVIENNWSSLFHHPLYVAAYFLNPSFRYCPDFLMNPEVIRGLNECIVRLESDNGKRISASMQIPDFVSAKADFGTDLAISTRSELDPASWWQQHGISCLELQRIAIRILSQRCSSIGCQHTWSVFDQVHSKRRNCLSRKRLNDHTYVHYNLRLRERQLGRKPDDLVSFDSAMLESVLDDWLVESEKQAMQEDEEIIYNEVEQFYGDDMDEHVSEEKRPTEMVTLASLVEPLDVNPAAGGVTTDDDGLDFLDDDLTD	zf-BED--zf-BED--DUF-domain--Dimer_Tnp_hAT--	III
TURN.01g003420.m01.polypeptide	Turn Zf-BED 01_II	MASSNTPIPVDDGFNEYESAVKRQKFTTSKVWDEMTNLECENKNELKAQCNHCKTIFSAKSSSGTSHLRRHLNSYLKKVNKDITQYTIATQPSPEGVPSIKNYKFDADECRKAISTFLVCGKHSFRTVEEPGFRYMMRIASPNFKNISRHTATRDVLMYYAKERDRVKEELAKAPGLICLTSDNWNSEHTNDEYICVTAHWVDKDWKLQKRIIRFRALFPPYDGLNIADELVLCLSQWG	zf-BED	II
TURN.01g296250.m01.polypeptide	Turn Zf-BED 02_I	MEWSVNNAFKSYKDMEPKSTMDMVLIPNMDTIDIVLGSSEKGNVVPSAKPRKKTMTSVYLKYFETAPDGKTRRCKFCGQSYSIATATGNLGRHLSNRHPGYDKTGENVTSSAPQPSTTPTVIKKPQQQGRAPQVDYDHLNWLLIKWLILATLPPSTLEEKWLANSFKFLNPSIQLWPGEKYKAVFREVFRSMREDVRASLEQVSSKVSIALDFWTSYEQIFYMSITCQWIDENWSFRKVLLDICQVPYPCTSSEIYNSLVKVLKMYNIENKVLSCTHDNSQNAIHACHALKEDLDGQKMGPFCFIPCAARTLSLIIDDALRTTKPVIAKVREFVQELNASLDISEDFIQLATAYKEGSWQFPLDASARWSGSYQMLDLVQKAGKSMDAIVRKNEEMLGNRMLLNASEKNVVNIVHNYLEPFYKVISEICVNIPPTIGMVIVYMDHISDTITTRQPPDWLKNPAEDMAKKLRSYNNQVCNIFIYMTAILDPRIKCELIPESLNSENYLEEARAHFVRNYYTTPFSSMTSGYSSQDIEDGGAVSFAEEIARKKRRASMSNATDELTQYLSESPAPTKTDVLEWWKVNSTRYPRLSAMARDFLAVQATSVKPDELFCSKGDEIDKQRFCMPHDSTQAILCIKSWTQGGLKLKYKSTEIDYERLMEMAAAAAADISSAGIDKKQK	zf-BED--DUF-domain--Dimer_Tnp_hAT--	I
TURN.01g320270.m01.polypeptide	Turn Zf-BED 03_I	MTMASSNTPIPVDDGFNEYESSLKRQKSTTSKVWDEMTKLECENKNELKAQCNHCKTIFSAKSSSGTSHLRHHLNSCLKKVNKDITQYTIATQPSPEGVPSIKNYKFDADECRKAISTFLVCDKHSFRIVEEPGFRYMMRIASPNFKNISRHTAARDVLMYYAKERDRVKEELAKAPGLICLTSDNWNSEHTNDEYICITAHWVDKDWKLQKRIIRFRALFPPSDGLNIADELVLCLSQWVKAGLELADDVVGKIQNGIRYIRKSGIRRKRFYDVADKSFHLNVTKKLRQDVCVRWNSTYLMLESSLYYKDVLDYWGQRDKDYQMFALSNEEWRNVAILCKFLKVFYDVTCVFSGSNYPTANLYFRGVWKVHKVLLDTIKGPYSFLTPMVKRMQEKFNKYWAKYSLILSCAAILDPCYKLNYVQYCFNTIYGIHASDFVETILSNLRLLFDEYVKKSKSTSSSLAGSSNVSDKNPVDSGLDEHNDSSADFGGCFDESDDYKRYLNESSTRSEKSQLDIYLEEPELELNSQIDVLHYWSKSSVRYNELSLLARDLLAIPISPVAFELAFSMGKKVITPLRSSLKPKTVQAVVCLDDWMLAKGFSAEIG	zf-BED--DUF-domain--Dimer_Tnp_hAT--	I
TURN.01g330710.m01.polypeptide	Turn Zf-BED 04_I	MEVANESTAKKPKRLTSVVWNHFERVKKADICYAVCVHCNKKLSGSSNSGTTHLRNHLMRCLKRSNYDVSQLLAVKRRKKENSLTIANISYDEGQRKEDYMKPTIVKYEQDQRKDEAFNLGSSWFDPERSRLDLARMIILHGYPLAMVEHVGFKVFVKNMQPLFDVVHNSTIELSCVEIYMKEKQRIYDMLSKLQGRINLAIEMWSSPENSKYVCLTAHYVDDEWKLQKKILNFLTLDSSHTEDMLSDVIIKCLMDWDIDCKLFSMTFDDCSTNDDVVLRIKDQISESRSRLSNGQLLDVRSAAHVLNSIAQDAIEALQVVIQKIRGSVKYVKSSQSILGKFNEIAQQQGIDNHKIVVLDYPIRWNSTYMMLETAVEYRNVFHHLPELDPDFALSDEEWKRASSIVSYLKLLIEIINVFSSNKCPTANIYFPEICHVHIQLIEWCKSSDAFLSSLATKMKAKFDKYWSKCSLALAVAAILDPRFKMKLVEYYYSQIYGSTALERIKEASDGIKELFNAYSICSTLIDQGSALPGSSLPSSSNDTRDKLKGFDKFLHETSQSQTAISDLEKYLDEPMFPRNCDFNILNWWRVHTPRYPILSMMARDVLGTPMSTVAQEFAFNAGGRMLDSNQSSLPPDTRQALICTRDWLRTQPDDATPSSSHYALPLYVEAN	zf-BED--DUF-domain--Dimer_Tnp_hAT--	I
TURN.02g048360.m01.polypeptide	Turn Zf-BED 05_I	MVRGRDACWEHCVLVDATRQKVRCNYCHREFSGGVYRMKFHLAQIKNKDIVPCAEVPDDVRDHIQSILNTPKKQKTPKKPKMDKTVANGQQNSSSASGGLHPNHGSSGQHGSTCPSLLFPHPSPSEQPATDDAQKQKLDDADKKIAVFFFHNSIPFSAAKSMYYQEMVDAIAECGVGYKAPSYEKLRSSLLEKVKGDIHDCYKKYREEWKETGCTVLCNSWSDGRTKSFVIFSVTYPKGTLFLKSVDVSGHEDDASYLFELLESVVLEVGLENVIQVITDSTASYVCAGRHLMAKYSSLFWSPCASYCIDKMLEDISKQEWVGIVLEEAKSIARYIYSHAWILNMMRKITGGRELMRPRITRFVDNYLTLRSIVIQEDNLKHMFSHSEWLSSIYSRRSDAQAIKSLLYLERFWKSAREAVSVSESLVKILRIVDGDMPAMGYMYEGIERAKGAIKAYYKGIEEKYMPIWDIIDRRWNMQLHSPLHAAAAFLNPSIFYNPNFKIDLRMRNGFQEAMLKMATMDKDKIEITKEHPVYINAQGALGTDFAIMGRTLNAPGDWWASYGYEIPTLQRVAIRILSQPCSFHWCRWNWSTFETVHTKKRNKVEMEKLNDLVFVHCNLWIQTICQGRDGKCKPIIFDEIDVSSEWPTESEPSVPLLDDSWLDNLPLECRGSP	zf-BED--DUF-domain--Dimer_Tnp_hAT--	I
TURN.02g348510.m01.polypeptide	Turn Zf-BED 06_III	MAPLRSIGYVDPGWEHGTAQDERKKKVKCNYCGKVVSGGIFRLKQHLARLSGEVTHCEKVPEEVCLNMRKNLEGCRSGRKRRQLDYEQAALSIQSNEYSDGEDASASYKHKGKKVMGDKNLVIKFTPLRSLGYVDPGWEHCVAQDEKKRRVKCNYCEKIISGGINRFKQHLARIPGEVAYCEKAPEEVYLKIKENMKWHRTGRRHRKPDTKEISTFYMHSDNEDEGGEEEGYLQCISKDILAIDDKVSDNDIRNNVRGRSPGSSGNGAEPLLKKSRLDSVFLKSLKSQTSAHYKQPRARTGFEKKTHREVISAICKFFYHAGIPSNAANSPYFHKMLELVGQYGQGLQGPSSRLISGRLLQEEIANIKEYLVELKTSWAITGCSVMADSWNDAQGRMLINFLVSCPRGVYFLSSVDATDIIEDAVHLFKLLDKAVDEVGEEYVVQVITRNTLSFRNAGKMLEEKRRNLFWTPCAVYCIDRMLEDFVNIKWVGECVDKAKKVTRFIYNNTWLLNFMKKEFTKGQELLQPAVTKFGTNFFTLQSLLDQRVGLKRMFQSNRWLSSRFSKSDEGKEVEKIVLNVSFWKKMQYVKKSFEPVAEVLQRIGSDKIRSLPFIYNDICRTKLAIKAIHGDDVRKYGPFWSVIESNWSSLFHHPLYVAAYFLNPSYRYRPDFLMNPDVIRGLNGCIVRLEADNGKKIAASMQIPDFVSAKADFGTDLAISTRSELDPASWWQQHGISCLELQRIAIRILSQTCSSIGCEHNWSAFDQVHIKRHNCLSRKRLNDQTYVHYNLRLRERQLGRKPDELVSFDSAMLESVLDDWLVETEKLAMHEDEEIIYTEVEQFCGDDMDEHESEEKRPAEMVTIAGFIEPLDVIPSAGGVTTDDDGLDFLDDDLTD	zf-BED--zf-BED--DUF-domain--Dimer_Tnp_hAT--	III
TURN.03g077210.m01.polypeptide	Turn Zf-BED 07_V	MDNFDQKLGPEFFKNLSAEAVTPLNVVHEEIYESSSKRPKTTSKVWDIFEKLPAQQGDSKAICKLCRRIYTAKTTSGTSHLRRHIEACVKRDYETFQSARFRTQVEKSQLDLYLEEPSHDLNSEIDVLEYWTLCSLRYPELQNGPPMFLTIPVSTIASDSAFDLVPK	zf-BED--Dimer_Tnp_hAT--	V
TURN.03g077600.m01.polypeptide	Turn Zf-BED 08_IX	MASSFFDTALRFLPSCAEAIEDGDLKSADAFLHNILILADEIPYSNQSKLVKYFAEALVRRAYGLHPASSSLTFPVDPAPNYHYNSYLINGVIENVIHDALMGIRRFHLIDFSIPYYDRFQNSVLRTLPNFLDYPPPVRVSYILPPFLKKHVKFSRQMEFLTRDAKEVNVKLEDELKVVYGNSLAEVDECEIDFKEEMMKWWWFTINLNLISWEEMQKQWRESCFLTCFKDSFEYSLKTIDCWAVPGCYLGWEYAWECNIEAGEGNNIIRRHPTLTEWQHLFSMAGFSRIPLNHDKDILVLQDKRFLEIMGEEEECLILGKKGCPMFFLSAWKPKVEDGHFNSNSTNHKFGQGFNPNPLPRQPLQPIEGLILNRLAALAEIQDISKDLCCKYKLSLALTRASKVNNMNETISDPNNKHTFSFQSNSCYLKHSNSYLFMITSENMISGLITKKAFESRDGYHFEPSLTKVEDFRYFSLEHYNIDVVFAICLQNRHTSDEVYIVAFYWPPTESEISKSLALRIFDDLKHMKTTFVTVKVQGPQIKFQEEAISSIPTSSNTAMPLKIAENAHIEQIVETKRNKQRKSWSKVWVDFDKFEEHGKQVAKCKHCPKVLTGSSKSGTTHLNNHSKVCPGKKKHNQESQLILPVDTNERSSTFDQERSHLDLVKMVIRHQYPLDLAGQEAFKNFVKGLQPMYEFQSRDKLLSDIHRIYNEEREKLQLYFDQLACKLNLTVSLWKNDHGKTAYCCLIAHFIDDSWELKMKTLGLRTLEHINDTKAVGGIIQSLVSEWNIGSKVCSITVDNSFLDDSMVQQIKENCLSNLVSLSSTHWFINCTLLEDGFREMDDLLFNLKKSLEYVTETKHGRLKFQEAKDQVKLQDGKSWDDLSLKPESDFGILDSALRSREIFRKLEQIDGNFKLNPSMEEWENAAALQSCLRWFDDIKGTQSLTVSLYFPKLCHIYMKFLQLEKINPSFVTLMKRRFDHYWRVCNSALAVASVLDPRLKFKVVVFSYTLIYGHDSKVQLNTFREVLTNVYNEYANETKNQTTSASVLDDINWLGNNSIWDSFSKFVTASKFNEVSSKSELELYLDEPLLPKDGTTFDILGWWCDKSKKFPILAKMARDFLAIPVSIFTPCSSIKATINNPAYNILNPESMEALVCSENWLESPKGNDGENQESTQTTDKGKRKLDEDTCVRKKSKPSNCEKGISTEDIAKDSNNNDEPVGEISIGKLQTENSSKNGCYGETSSGNKSKASNKMMGTISLQDIHQEKSSSELNHGRNVEDVSSGDSSSDNDQSDQLQSSSSESDVEIELKEQGSWFEQDIKAYLLSEFTKKENELIDKWQKNEMKGKMIGRDKYFKIQGEKLAPLLMVPQGDETRKEYYIEDLIVNTFFELLKKRSDEFPNIYINHYSFGSQIATKLIEGTRTEQEVLAWFKVDELRGVHKMFLPMSLSKHWVLFYVDTKEKKISWLDPLASSRIRSYNVEKDIILQWFTTLLLPKLGYIDAKEWPFLVRNDIPEQKNSVDCAVFVMKYGDCLTHGDCFPFKQEDMVHFRRRIFVDIYRGRIHTKKINKDRCIV	GRAS--zf-BED--DUF-domain--Dimer_Tnp_hAT--Peptidase_C48--	IX

TURN.03g077610.m01.polypeptide	Turn Zf-BED 09_VIII	MAASFSDIDTDTALRLLLSCAKAIEDGDLKSADVFLENILILADERPYLYKSRVVKYFADALVRRACGLHPASSYFTFPVDPSPYYHCGSYLINGVIENVIHDALMEKNALMGNRRFHLIDFSIPYSSFQNSVVRTLPTFSGDPLPVRVSYILPPFLKKYVKFSRQMEFLTRDAKEVNVKLEEELKVVYGNSLGEVDECEMDFKRRRDDEMVVVYYKFKLDKLVRDAKAMERELVRLKEINPTIVIMLDFYSNHTHSNFLTCFKDSFQYSLKTLDCWAELDLYFDEEYERECHIEAWEGNNVIRRHPTLTEWQHLFSMAGFSRIPLNHREGIDLIVKDVNPLNDFFSMSNQSWLEIMGKEEECLILGYKECAMFFLSAWKPKVEEEPLNFNSSNDKFGQGFNPYPSPLRPLQPFPEGLTLSRVAALAEIYDILNHLYCEHKFSLALTWASKVDNINETMSDPNKKFTFSIQSNSCYSKDLNSYKFMRSCEYKIEQTIIEKALESKDGYHFEPSLTKFDIDDYMYLQRAKHCDVDVVVAICLQNRYTSNDVYVVEFYWPATESEISKSFTPHIFNDLKHMEKKFVTVKVQGTEKAISNIPTSSYTARPLKIAEETEDVDAVEINGVNVQRGVVPNFPSPITIQSSSKVVAAPSNTLEGPHNQIFPNGDPEIVKANKEEPSKATQRELRSKVWDHFDRFEEDEKQVAECKHCPKVLTGSSKSGTTHLNNHSKVCPGKKKQNQESQLILPVDTNEGSLRFDKKRSHKDLAKMMIKLQCPLDMAEQETFKNFVKGLQPMFEFQSKDILSYIHRIYDEEKEKLQLYFDNLASKFNLTVSLLKNNSGKTIYCCLISHFIDDGWELKRKILALKTLEHINDTKALGEIIRSLVLEWNIRNKVCSITVDNSFLNDSMVDQIKEICLSDQGSVSSDHWFISFTLLEDGVREMDGILFKLRKSIEYVTETRHGKLKFQEAVDQVKLQGGKLWDDLSFRLESDFDILDSALRSREIFCKLEQIDDNFKLNPTMEEWENAVALQSCLKCFDDIKGTQCLPVSLYLPKLCDTYKKFLQLEKSSHSFVTLMKRKFDRYWSLCNLALAVASVLDPRLKFKIVELSYRVIYGHDSKMRLNMFHKVLRDVYYEYASESKNLTSSASVLDDFNCSTIGLGNDSILDSLSKFASASNFNEEASWKLELELYLDEPLLPMDGAFFDILGWWCDKSQRFPILAKMAQDFLAVPVSISKSCSNISAMINNPAYSSLNPESMEALVCSENWLETPKENFSLSFDNWMEPQFSSSESIGEKAEILKALVCNDNRLESSIGKPDHEKNVDDSSSSESEDETTLKEEGPWCKQDIKAYLLSRFNSKEYKRLDKWRKNELNGVRYWYLYSWCPKVVKLEKSR	GRAS--zf-BED--DUF-domain--Dimer_Tnp_hAT--	VIII
TURN.03g077620.m01.polypeptide	Turn Zf-BED 10_XI	MLDSEAMISVPLSIEKAFESRDGYHFEPSLTEVEDIRYSILEDYNIDVVVAICLQNRHTSDEVYIVEFYWPPTKSEISKSLALSIFDDLKHMKTTFVTVKVQGPEIKFQEEAISSIPTSSNTAMPLKIAENVHIEQIVETKRNKQRKSWSKVWVDFDKFEEHGKQVAKCKHCLKVFTGSSKSGTTHLNNHSKVCPGKKKQNQESQLILPVDTNERSSTFDQERSHLDLVKMVIRHQYPLDLAGQEAFKNFVKGLQPMYEFQSRDKLLSDIHRIYKEEREKLQLYFDQLACKLNLTVSLWKNNHGKTAYCCFIAHFIDDGWELKMKILGLRKLEHVYDTKVVGGIIRSFVSEWNISKKVCSITVDSSFLNDGMVHQIRKNCVSEQGSLSSAHWFISFTLLEDGFREMDSILTKLWKSIEYVTETTHGKLNFQEAVNQVKLQGGKSWDELSFKLDSDSDILDSALRSREIFCKLEQIDDNFMLNLSKEEWETAVTLQSCFKCFDDIKGAQSLTANLYFPKLCNMYEEFGQLKKSNHPFVILMKRKFDNYWSLCNVAFTIAAALDPRLKFRSSCNETYDLESMMKLIRFRKVLMDVYSEYANEAKNLSASSSVLDDSNSLTAETTKDCIVSYFSKFASASNVKEVASQKSELDCYLEETLLPSDADILGWWRVNSQRFPTLAKMARDFLAIPVSVSSPCSNISAMTINPAYSSLGPESMEALICSQNWLESTKENDGEHHEPMQNMDKRKRKMEENDTSTVKVFKNRTHEKGSSNGDIASDFNKNDGSLSFDNWMEPQCSSSESVGEKAEIMEASVCNRDGLESSIGTKFLDTETNHGRNIAAAIEIPNDEPSFNSNQLDQFQSSSSESDDETTLREQGSWCIEDVRTYLVSSFTNKEKKRLNRWKRSQLSGKKIGRDKEFQLMGENLTPLLMVPHCDETLREYYIDDSVVNTYFKLLKKRSDKFPNVYIKHYSFDSLIATCLIEGSKSEDEVLAWFKDEKLRGVHKLFLPMCLSAHWVLLCVDTKEKKISWLDPIPSSRIMSNSVEKEKIFQWFTLYLLPEFGYNDAEKWPFEVRTDIPKQEKRVHSSIMTPKNTELQTSIYTLIFTLKNIENISSDILGFISDESTKRNIKSLIKSLCRML	zf-BED--DUF-domain--Dimer_Tnp_hAT--Peptidase_C48--	XI
TURN.03g077770.m01.polypeptide	Turn Zf-BED 11_XI	MKTTFVTVKVQGPEFKFQKECISSTPTSSNTAMPLEIAENAHIEQIAETKRNKQRKSWSKVWVDFDKFEEHGKQVAKCKHCLKVFTGSSKSGTTHLNNHSKVCPGKKKQNQESQLILPVDTNERSSTFDQERSHLDLVKMVIKHQFPLALVGQEAFKNFVKGLQPMFEFQSRDKLLSDIHRIYNEEREKLQLYFDQLACKLNLTVSLWKNNHGKTAYCCLIAHFIDDGWELKMKILGLRKLEHLYDTKVVGGIIRSFVSEWNISKKVCSITVDNSFLNDGMVHQIRENCVSEQGSFSLVHWFISFTLLEDGFREMDSILSKLWKSIEYVTETTHGKLIFQEAVNQVKLQGGKSWDELSFKLESDPDILDSALRSREIFCKLEQIDDNFMLNLSKEEWEKAVTLQSCFKCFDDIKGTQSLTANLYFPKLCNMYEEFGQLKKSNHPFVILMKRKFDNYWSLCNVAFTIAAALDPRLKFRSSCNETYDLESMMKLIRFRKVLMDVYSEYANEAKNLSASSSVLDDSNSLTAETTKDCIVSYFSKFASASNVKEVASQKSELDCYLEETLLPSDADILGWWRVHSQRFPTLAKMARDFLAIPVSVSSPCSNISAMTINPAYSSLDPESMEALVCSQNWLESTKENDEEHHEPMQNMDKRKRKMEENDTSTVKVFKNRTHEKASSNGDIASDFNKNDGCLSFDNWMEPQCSSSESVGEKAEIMESSVCNRDRLESSIGKTNHGRNIAAAIEIPNDEPSFNSNQLDQFQSSSSESDDKTTLTEQGSRCIEDVRIYLVSSFTNKEKKRLNRWKRSELSGKSNYKLKFTFHIKKIGRDKEFQLMGENLTPLLMVPHCDETLIEYYIDDSVVNTYFKLLKKRSDKFPNGYIKHYSFDSLIATCLIEGSKSEDEVLAWFKDEKLRGVHKLFLPMCLSAHWVLFCVDTKEKKISWLDPIPSSRIMSNSVEKQKIFQWFTLYLLPQFGYNDAEKWPFEVRTDIPKQENSVDCGVFVMKYGDCLVHGDFFPFTQKDMIHFRRRIFLDIYRGRLHGKR	zf-BED--DUF-domain--Dimer_Tnp_hAT--Peptidase_C48--	XI
TURN.03g079290.m01.polypeptide	Turn Zf-BED 12_IX	MKRELVRLKEINPTTVIILDFYSNHSDSDFLTCFKDSFQYSLKTLDYWQELDRYLDGKKEWEFNIEAGEGNNIIRRHPTLTEWQHLFSSAGFSRIPLNHRKDNLSVEDNSFLKIMREEEECLILGYKGCPMFFLSAWKPKVEDGHFNSSSTNHKFEQGFNPNPLPLQPLQPFLEGLILNRLAALAEIHNISKDLCCKYKLSLALTWASKVNNMNETISDPNKKHTFFIQSNYCYVKDRKSYDFMFGFERMISVPIFEKAFESRDGYHFEPSLTKVEDFKYFMLKDCNIDVALAICLQNRHTSGEVYIVEFYWPPTESEISKSLALRIFDDLKHMNTTFVTVKVQGPEIKFQEEAISSIPTSSNTAMPLKIAEEARGIRAIEINAHIEQIIFNDDSQTVETKRNKQRKLWSKVWVDFHKSEEEGKQVAKCKHCPKVLTGSSKSGTTHLNNHSKVCPGKKKQNQESQLILPVDTNERSSTFDQERSHLALVKMVIRQQYPLDLAGQEAFKNFVKGLQPMYEFQSRDKLLSDIHRIYNEEREKLQLYFDQLACKLNLTVSLSKNNHGKTAYCCLIAHFIDDSWELKMKTLGLRALEHINDTKAVGGIIQSLVSEWNIGNKVCSITVDNSFLDDSMVQQIKENCLSNLVSLSSTHWFINCTLLEDGFREMDDLLFKLKKSIEYVTETKHGKLKFQEAVDQVKLQDGKSWDDLSLKLESDFGILDSALRSREIFCKLEQIDGNFKLNPSMEEWENAAALQSCLRCFDDIKGTQSLTVSLYLPKLCDIYMKFLQLEKSNSSFVTLMKRRFDHYWRLCNSALAVASVLDPRLKFKVVEFSYILIYGHDSKVQLNTFREVLTNVYNEYANETKNQTTSASVLDDINWLGNNSIWDSFSKFVTASEASSKSELELYLDEHVIPMDGAIFDILGWWSDKSQKFPILAKMARDFLAIPVSIFIPCSNIKATINNPAYNILNPESMEALVCSENWLETPKGNDGENHEPTQTMDKGKRKLDEDTCVRKKSKPSNCEKAISTEDIAKDSNNNDEPAGEISIGKLQTENSSKNGCYGETSSGNKSKASNKMMGTICLRDNHQEKSSSELNHGRNVEYVSSGDSSSDNDQSDQLQSSSSESDVEITLKEQGSWSEQDIKAYLLSEFTKKENELIDKWQNNELKGKMIGRDKYFKIQGEKLAPLLMVPQGDETREEYYIEDLVVNTFFELLKKRSDKFPNIYINHYSFSSQIATKLIEGPRTEQEVLAWVKVDELRGVHKMFLPMSLSKHWVLFYVDTKEKKISWLDPIASSRIRSYNVEKDIILQWFTTLLLPKLGYVDAKEWPFLVRNDIPEQKKEREQGVLVKEGTRPTLSALGVAAVASISRRAVALPVLSLLLVRGHEQVPIGSAEVRAVFSSGSGRVAGCMVTEGKIVDGCGIRVIRNGRTVHVSVLDSLRRVKEIVKEVNAALECGMGVEDYDQWQEGDILEAFNTVQKKRTLEEASTSMAAALEGVGVEL	GRAS--zf-BED--DUF-domain--Dimer_Tnp_hAT--Peptidase_C48--	IX
TURN.03g368030.m01.polypeptide	Turn Zf-BED 13_I	MSSNLEPIPITSQKHDPAWKHCQMFKNGERVQLKCIYCGKIFKGGGIHRIKEHLAGHKGNAATCLRVPSDVRVLMQESLDGVVVKKRKKQKIAEEITNVNQVSTEIQAYADQVDTNTGLLMIEKSDTLEPSSSLLVNQEGTSNVAGERRKRGRGKSLPAEANALSFVPVELGARRVNNHVHMAIGRFLFDIGATMDAVNSVYFQPMVDAIVSGGSGALMPSCNDLQGWILRKSVEEVKSENDKVMAAWVRTGCSILVNQWNTQTGRILLNFLVYCPEGTVFLKPIDASSVINSSDALYELLKQVVEEVGSKHVLQVITNGEEQYIVAGRRLVETFPTLYWTPCAAHCVDLILEDFAKLEWINAIIEQARSITKFIYNHSVVLNMVRRYTFGNDIVEPAATRSATNFTTLTRMVDLKNNLQAMVTSQQWVDCPYSKKPGGLAMLDLVSNQSFWSSCVLIVRLTNPLLRVLRMVGSKKRPAMGYVYAGMYRAKETIKKELVKRNEYMVYWNIIDHWWEQQWHHPLHAAGFYLNPRFFYSMEGDMPNEMLSGMLDCIEKLIPDVTVQDKITKEINSYKNSVGDFGRKMAVRARDTLLPVEWWSTYGGSCPNLARLAIRVLSQTCSTLGLKHDHIPFEKLHETRNCLEQQRLRDLIFVQCNLQLRQIGYESKQHDSMQPLSSESASIVEDWVTGIDAFLDDDTYPDWTTLETLSVNTMLLRPGDEVEELGAGFNDHEIFNRMKEGDNEKAEDNVVS	zf-BED--DUF-domain--Dimer_Tnp_hAT--	I
TURN.04g084600.m01.polypeptide	Turn Zf-BED 14_I	MDMSDAVIVNSSRLKSIVWNDFDRVKKGDTFVAICRHCKKKLSGSSTSGTSHLRNHLIRCQRRSNHGVAQYFSAKDKKKEGSLALVTIDQEQKNDEVLSIVNLRYEQEQIKSEHVGIGSNSLDQRRSQFDLARMIILHNYPLAMVEHVGFKIFVRNLQPLFELVTRNKVEADCMEIYAKEKQKVYEIFDKLPGKISVSADMWTASEDDAAYLSLAAHYIDENWQLKKKNLNFVTIDPSYTEDMHSEVIMNCLMDWDIDRKLFSMIFDSFTSDNIVERIRDRLSQNRFLHCNGQLFDVRCAVDLLNRMAHDALEALCEITQKIRESIRYVKSSEATQATFNELADEVQVETKKCLCIDNPLKWNSTYFMLEAALEYRKVFSCLRDRDPVNRKFLLSDPEWDRLITVTSFLKLFVEVTNVFTRSKYPTANIFFPEICDIHLQLIEWCKNPDEYISSLALKMRKKFEEYWYKCSSGLAVAAMLDPRFKMKLLEYYYPQLYGDSATELIDDVFECIKSLYNEHSIVSPLASSIDQGLDWQASGIPGSGKDSRDRLMGFDKFLHETSQAEGSSSDLDKYLEEPLFPRNVDFNVLNWWKVHTPRYPILSMMARNILGIPISKVAAESRFDTGGRMLNHNWSSLPPTTIQALMCSQDWIRSELES	zf-BED--DUF-domain--Dimer_Tnp_hAT--	I
TURN.05g136810.m01.polypeptide	Turn Zf-BED 15_IV	MASSEVFINVRDHGKTVDVKKQRIKCNYCDKEMSGYSRLKYHLGGVRGNVLPCEKVPQDVKKLFRDMVQGREHLHNDAPYLYRQPFPQKRNGCPHNNVAKKTRHQSSESSGDESREYGNTDSMSEDDLEDSVASYKRMVSQSAAVIGDPNQESGTDFKLVNSLSFQRLMNDIRGPGQAEYKIPDCLELKGWILKDEVKEIQEYVQKIRQSWGNTGCSILLDGWID	zf-BED--DUF-domain	IV
TURN.06g16	Turn Zf-	MTEMTEITDMETIPGESNNQLALTTPEVQPIKRRKKKSMVWEYFTIENVSAGCRRAYCKRCKQSFAYSTGSKVAGTSHLKRHIAKGTCRALLRGQGQDNNQFITPYNPKMGGSEPPKRRYRSPSSPFIPFDQDRCRHEIATMIIMHEYPLHIVEHPGFIAFVQNLQPQFDKMSFNTVQGDCVATYLREKQSLMNFIEGIPGRFCLTLDMWSSNQTLGYVFITGHFVDSDWKLHRRVFNVV	zf-BED--DUF-domain--	I

9630.m01.polypeptide	BED16_I	MEPYPDSHSALSHAVAACLSDWSLEGKLFSLTFNHPLSEAGLENLRPLLCVKNPLILNGQLLIRNCIARAMSSMAKDVLGAGQEIIKKIRDSVKYVKMSESHDDKFIQVKNQLQVPSEKSLFLDNQTQWNTTYQMLAAASELKEVFDCLDTYDPDYKLAPSMEDWKLVETLCSFLKPLFDAASILTTTTLPTVITFFYEVWKIHVDLGRSITSEDPFISNLAKSMQEKIDKYWKDCSLVLAMAVVMDPRFKMKLVEFSFTKIYGEDAPTYIKTVDDGIHELFLEYVALPLPLTPTYAEEVNGANNGKTNESHQGNLLSDHGLADFDVYIMETNSQQMKSELDQYLEESLLPRVQEFDVLGWWKLNKMKYPTLSKMARDILSIPVSAAATESIFDITDKQLDEYRSSLRPETVEALICAKDWLHYGSSDVSNALVKMEF	Dimer_Tnp_hAT--	
TURN.06g169640.m01.polypeptide	TurnZf-BED17_I	MTEMTEITDMETIPGESNNQLALTTPEVQPIKRRKKKSMVWEYFTIENVSAGCRRAYCKRCKQSFAYSTGSKVAGTSHLKRHIAKGTCRALLRGQGQDNNQFITPYNPKMGGSEPPKRRYRSPSSPFIPFDQDRCRHEIARMIIMHEYPLHIVEHPGFIAFVQNLQPQFDKMSFNTVQGDCVATYLREKQSLMNFIEGIPGRFCLTLDMWSSNQTLGYVFITGHFVDSDWKLHRRVFNVVMEPYPDSHSALSHALAACLSDWSLEGNLFSLTFNHPLSEAGLENLRPLLCVKNPLILNGQLLIRNCIARAMSSMAKDVLGAGQEIIKKIRDSVKYVKMSESHDDKFIQVKNQLQVPSEKSLFLDNQTQWNTTYQMLAAASELKEVFDCLDTYDPDYKLAPSMEDWKLAETLCSFLKPLFDAASILTTTTLPTVITFFYEVWKIHVDLGRSITSEDPFISNLAKSMQEKIDKYWKDCSLVLAMAVVMDPRFKMKLVEFSFTKIYGEDAPTYIKTVDDGIHELFLEYVALPLALTPTYAEEVNGANNGKTNESHQGNLLSDHGLADFDVYIMETNSQQMKSELDQYLEESLLPRVQEFDVLGWWKLNKMKYPTLSKMARDILSIPVSAAATESIFDITDKQLDEYRSSLRPETVEALICAKDWLHYGSSDVSNALVKMEF	zf-BED--DUF-domain--Dimer_Tnp_hAT--	I
TURN.07g195520.m01.polypeptide	TurnZf-BED18_I	MELNLVPISITRQKQDPAWNHCEVFKNGERLQIKCMYCGKLFKGGGIHRFKEHLAGRKGQGPICEQVPQGVRSVMQESLNGILVKQDKKQKLIPKLLACGSSSSNPNISGEVENLGSHDDMNFGIKPISVLNTLEGDSNVVSKVGRGRKRGRGRDRNLIESNYPCVKTDLALVPNGGENPIHMAIGRFLYDIGVNLDAVNSVCFQPMIDAIASGGSGVVPPSCHDLRGWILKNVIKEVKDDIDRNKAMWGKTGCSIIVEQCRTKNGRVLLSFLVYCPQATVFMKSVDASHAVYSADYLFELLKQVIEEVGSEKVVQVITNCEEPYLFTGKRLMESFPSLYWAPCLAHCVDLMLQDFSNLEWINETIEQAKSLTRFIYNQSSVLNMMRKFTSGNDVVEPALTCFATNFSTLKRMADLKLNLQAMVNSQDWLECPYAKKPGGQAMSDIVNNRSFWNSCMLIARITYPLLRVLEIVGSKKRSAMGYVYAGIYRAKETIKKELVKQDDYMVYWNIIDNRWEQQRHLPLYAAGFFLNPKLFYNTEEHIQNDILSSVFDSIERLVPDTNIQDQVVREINLYKNATGDLGRPMAVRARDNLLPGEWWSIYGGGCPNLQRLAIRILSQTCSSIGYKPSKISIEEIHNTRNFLERQRLSDLVFVQYNLYLRQMVLQKQEKDSLDPLAFNNKDILEDWIADNEVSPDNLESSDWKSLDPPVGNRTTLTPPGDEAEDFLSTRFTDLDIFNGLKGVKEEI	zf-BED--DUF-domain--Dimer_Tnp_hAT--	I
TURN.08g206810.m01.polypeptide	TurnZf-BED19_IV	MEVANETIIKKPKRLTSVVWNHFERVRKADLCYAVCVHCNKKLSGSSNSGTTHLRNHLMRCLKRFNYDVSQLLSAKKRKKENTLTIANISYDEGQRKEEYLKPTIVKYEPEQRKDEVFNVQSSWFDQDRSRLDLARMIILHGYPLAMVEHVGFKVFVKNLQPLFDVVPNSTVGLSCMEIYGKERQKVHDMLSKLQGRINLALPELDLDLALSDEEWEWASSITGYLKLFVEIINVFSSNKCPTANIYFPEICHVHIQLIDWCKSPDNFLSSLAAKMKAKFDKYWSKCSLSLAVAAILDPRFKMKLVEYYYSQIYGSTALERIKEVSDGLKELFNTYSICSTLMDQGSALPLGSLPSSSNDGRDRLKGFDKFLHETSQNPGPSSSHYALPLYVETN	zf-BED--DUF-domain	IV
TURN.08g223180.m01.polypeptide	TurnZf-BED20_I	METTPVENNNELALITPETQPKRRKKKSMVWEYFTIETVSAGCRRACCNRCKQSFAYSTGSKVAGTSHLKRHIAKGTCPALLRDQYNNQLTPYNPKTGGSEPRKRRYRSPSSPFIPFDQDRCRHEIARMIIMHEYPLHMVEHPGFIAFVQNLQPRFDKVSFNTVQGDCVATYLREKQSLMKLIEGIPGRVCLTLDMWTSNQTLGYVFITGHFIDFEWKLQSRVLNVIMEPYPDSDSALSHAVAACLSDWSLEGKLFSLTFNHPTSEAGLENLRPLLCTKNPLILNGQLLLGNCIARTLSSMAKDVLGAGHEIVKKIRDSVKYVKTSESHDEKFVQVKNQLQVPSEKSLILDNQTQWNTTYQMLAAASELKEVFNCLDTSDPDYKLAPSMEDWKVAETLCTFLKPLFDAASILTTTTNPTAITFFHEAWKIHADLGRSITNEDPFISNIAKSMLEKIDKYWKDCSLILAIAVVMDPRFKMKLVEFSFTKIFGEDAPTYIKIVDDGIHELFLEYGLTDFDVYIMETSSQQMKSELDQYLEESLLPRVQEFDVLGWWKLNKMKYPTLSKMARDILSIPVSAAAPDSVFDIIIKQLDEYRSSLRPETVEALICAKDWLHYGSEESNALVKMEF	zf-BED--DUF-domain--Dimer_Tnp_hAT--	I
TURN.09g244120.m01.polypeptide	TurnZf-BED21_XIX	MSRYRAQQQAPVDEPEANPQGEDHVPDDEQQDENDKDSQPSEPKGDYLNVASRPFLMKILQKQGDSKVLFADKVLKFTASGKMKRRNLIITDFAVYIVDPETDGLKRRIALAAVDKMCLSDLNDNFLSIIIPTEYDLLMASTRKTEIATCLFEAIKTSAQYELEVSFSSSFEYNATADLVKEVQFEEVEVVRERDVCWEYAEKLDGNKVRCKFCLRVLNGGISRLKHHLSRLPSKGVNPCNKVRDDVTDRVRAIISSKEDIKETPSVKKQKIAEVRAPGNMSTSSKISPLETLSPAAKVFPTVLSIAASTLSDQETVERSIALFFFENKLDFSVARSSSYQAMIDAVGKFGPGLIAPSVETLKTTWLKRIKSEVTLHLKDAEKEWATTGCTIIADTWTDNKSKALINFLVSSPSRTFFHKSVDASSYFKNTKCLVDLFDSVIQDFGQENVVQIIMDSSFNYTGISSHILQNYGTIFLSPCASQCLNLILEEFSRVDWVNRCILQAQTVSKFLYNNASMLDLMKKFTGGQELIRTGITKSVSCFLSLQSMLKQRSRLKHMFNSPEYSTNSSYANKPQSISCIAIVEDNDFWRAVEECVAISEPFLKVLREVSGGKPAVGSIYELMTRAKESIRTYYIMDESKCKTFLDIVDRQWRDQLHSPLHSAGAFLNPSIQYNPEVKFLGSIKEDFFKVLEKLLPTPELRRDITNQIFTFTRAKGMFACNLAMEARDTVSPGLWWEQFGDSAPVLQRVAIRILSQVCSTFTFERHWSTFQQIHTEKRNKIDKETLTDVVYINYNLKLAREMKTMPTESDPIQFDDIDMTSEWVEESENPSPTQWLDRFGSALDGGDLNTRQFSAAMFGNDHIFGL	Myosin_TH1--zf-BED--DUF-domain--Dimer_Tnp_hAT--	XIX
XP_003593063.2	MtuZf-BED01_XXII	MSKQKISTSVIDRISSFPDDILIRILSSLPIEQACVTSILSKRWTHLWCFVPDLDFTKTKWKDQESYSRFEEFVFSVLYSREAAGNHSINTFILDIEYDSSELLLDGIKNIHKWVDIVVKSKVQNIHLYPHVPLNGEETILPKFPMSSILSCTTLVVLKLRWFNLTVVSDLSIRLPSLKTLHLKEIYFDQQRDFMMLLDGCPVLEDLQLCYIYMTRQSHHSLDDFESSSMLKKLNRADITDCECYFPVKSLSNLEFLRIKLSEVYHPRDFPTFNNLTWLVLNYDWDIVVQVLHHCPKLQNLDLYQVRGEDEWEYEYEFFAEKENWANPKFVPSCLTSNLTTCTMWDFAYAGQQLMDIRDFPTSETSEHEVSSPTPAANPLPPPYRICKNRSEAWDHFIVVLEEEKRAKCIYCPKEIKFNGGTSSMRNH	F-box--LRR-motif--zf-BED--	XXII
XP_013447741.2	MtuZf-BED02_XIV	MGRGKHEHCWKHVTRVENGLKWICNYCNDKFSGGASRIQTHLGGKGGGIRRCSNYHEGVHNNMASTSSNPPEAAVINRVYSTQDQGVHGSGGNRSFSESEINQLKQLVIDLKCEEKDIAKQLQWLESRGKKRKPEVDVWLRKLLDMKGSMYDMNNLNGTLDVSELIKNMKRHKKEKPIILSNEFVGRALDLDIQRVFYFLDDDKVFVIGICGMGGVGKTLLATLVEKEGKRKPTFKDVIWITVSHNHSISKLQHDIAKRIGLKLDADDERVRADNLSSVLEKKGKSILILDDVWKYIDLQKVGIHPKVNGIKVILTTRLKHVCDQMNCHPYAIIQIFPLSACEGWELFMLKLGHDETPRTLPHEIEEIVRCIVERFKGLPLAINVMARTMKGINDFHQWKHALNKLEKLEMGQMVEEEVFKVLKPSYDNLMEKNLQNCFLYCALLSINDCDWDRDWDEFEKDELIMKLVDHGKINDSMCLEEIFVEGNTILKKLKSHSLISFHYYLVATHPLVRNMACYIWKESQRNAIVRSGKGLTKMHLSHEWATDLELVHMRDYDIEEIPEGMSPNCPKLFALILNELSISRVPESFFMYMNNLSILDLSYNEDLESLPDSITKLRSLVSLVLKRCASLIHVPPLGELQALSRLVISVTSIEKLPLGLEKLTNLKWLDLSKNMSLNLELRSFSSYLIKLQYLDLRDTHALNMVEDVQGMNMLECFGGAFDYRCFYQSMQNNLDMSFERIKTYNLILGNVCSASLGDWWHNVNLKRFGAADHETKSIQFGDCDHFSHILPKDLTYLCIEKNGHWVCLCDALSYNTASSLRKIGAYDCQQLESLFCLSGSCSFCTKIHNLEDLELRSMESLIVVCKDVVDVRQSLTSGIFSYLKTFHICDCHLIEKLLTPRLVQQLQNLETISVKYCNSMKEIFAMSNNDDDESSSITLPKLTTLELYNLPQLKIVYKGSIRCGSSPLKLDISKCPSLERHPTIENEDVDIPCF	zf-BED--NB-ARC--LRR-motif--	XIV
XP_024625326.1	MtuZf-BED03_V	MANEEVQSPNDVSPTQQTQTALDETNVESQEPEAVREPSVSPNKRGLKSVYWRHYKRQKFDGKFKAICKYCDKKLGGETTNGTSHLKDHLSICAARNKRSPMQALLKVSEVPGKGKESFVAGTYTFNQDISRTVASESAFSTSGRDVTPQRSRLKEDTLEALMCSQNWFRTEMQGYSKIYASFECVDEDMDDDEEMSQT	zf-BED--Dimer_Tnp_hAT--	V
XP_024626091.1	MtuZf-BED04_I	MERKKWQCLHCKRTYVVVASGSTSHLKRHLREVCLIYKKLAAQQQKLNLKPTQSMIDEKLSGPLLMNLSAKYDHERQREAIAHWVMMHEHPFSIVEEEGFLFMMKCSNFSYEEISRKTLKNECVAVYESERKKLKSTLRIVNKICLTTDLWKSQNQKIEYMVLTGHFIDADWVLQKRVLGFVHVPPPRHGVDIADAIFKCLKEWGIENKIFSVSVDNAYYNDRCLKELKVLLSRHQKLVLDGKLFHVRCCAHILNLLVEDGIGRIREIVEKVHDSVRFINQSEARLRTFSEIVQQLKLGGKKLILDCPTRWNSTYQMLSVAMQFKEAFPHFQDREPSYTTLSDEDDWEKVEKVCKLLEVFNFVTHIIYGSEYPTSNLYLTEVFQIKQLLDQAVQDESNFMKDMANAMKEKFDKYWSQCNLVMSLASVLDPRIKMMGVNMCFPLIYPGDEARKNIEKVHKALNDMYLEYVDRLHEHREEGSSRTSAGQNGLTLEPTRPTRWSKLMNYVQEQQAIPAVKSEVQEYLNEPTYKPNNNGHMSFCALEWWKLNSGKYKVLSHMATDVLAISISTVASESTFSAGGRVIDYSELLYILLPLKL	zf-BED--DUF-domain--Dimer_Tnp_hAT--	I
XP_024626092.1	MtuZf-BED05_I	MERKKWQCLHCKRTYVVVASGSTSHLKRHLREVCLIYKKLAAQQQKLNLKPTQSMIDEKLSGPLLMNLSAKYDHERQREAIAHWVMMHEHPFSIVEEEGFLFMMKCSNFSYEEISRKTLKNECVAVYESERKKLKSTLRIVNKICLTTDLWKSQNQKIEYMVLTGHFIDADWVLQKRVLGFVHVPPPRHGVDIADAIFKCLKEWGIENKIFSVSVDNAYYNDRCLKELKVLLSRHQKLVLDGKLFHVRCCAHILNLLVEDGIGRIREIVEKVHDSVRFINQSEARLRTFSEIVQQLKLGGKKLILDCPTRWNSTYQMLSVAMQFKEAFPHFQDREPSYTTLSDEDDWEKVEKVCKLLEVFNFVTHIIYGSEYPTSNLYLTEVFQIKQLLDQAVQDESNFMKDMANAMKEKFDKYWSQCNLVMSLASVLDPRIKMMGVNMCFPLIYPGDEARKNIEKVHKALNDMYLEYVDRLHEHREEGSSRTSAGQNGLTLEPTRPTRWSKLMNYVQEQQAIPAVKSEVQEYLNEPTYKPNNNGHMSFCALEWWKLNSGKYKVLSHMATDVLAISISTVASESTFSAGGRVIDYSELLYILLPLKL	zf-BED--DUF-domain--Dimer_Tnp_hAT--	I

XP_024626093.1	Mtu Zf-BED06_I	MERKKWQCLHCKRTYVVVASGSTSHLKRHLREVCLIYKKLAAQQQKLNLKPTQSMIDEKLSGPLLMNLSAKYDHERQRE<br>AIAHWVMMHEHPFSIVEEEGFLFMMKCSNFSYEEISRKTLKNECVAVYESERKKLKSTLRIVNKICLTTDLWKSQNQKIEY<br>MVLTGHFIDADWVLQKRVLGFVHVPPPRHGVDIADAIFKCLKEWGIENKIFSVSVDNAYYNDRCLKELKVLLSRHQKLVLD<br>GKLFHVRCCAHILNLLVEDGIGRIREIVEKVHDSVRFINQSEARLRTFSEIVQQLKLGGKKLILDCPTRWNSTYQMLSVAMQ<br>FKEAFPHFQDREPSYTTLSDEDDWEKVEKVCKLLEQLLDQAVQDESNFMKDMANAMKEKFDKYWSQCNLVMSLASVLD<br>PRIKMMGVNMCFPLIYPGDEARKNIEKVHKALNDMYLEYVDRLHEHREEGSSRTSAGQNGLTLEPTRPTRWSKLMNYVQ<br>EQQAIPAVKSEVQEYLNEPTYKPNNNGHMSFCALEWWKLNSGKYKVLSHMATDVLAISISTVASESTFSAGGRVIDYSEL<br>LYILLPLKL	zf-BED--DUF-domain--Dimer_Tnp_hAT--	I
XP_024626644.1	Mtu Zf-BED07_I	MESISQNLSSTLTSGSANVAEPTEQVAVATQVPVVGLPPLPCLAKRRKPNAGGPRRTSPAWDHFIKLPDEPEPTAACIHC<br>HKRYLCDPKTHGTSNLLAHSKVCFKNPQNDPTQASLMFSNGEGGTLVAASQRFNPAACRKAIALFVLLDEHAFRVVEGE<br>GFKLLCRQLQPLLTIPSRRTVARDCFQLFLDENLRLKTYFKSDCVRVALTTDCWTSGQNFSYMTLTAHFINNDWKYEKRIL<br>SFCTVPNHKGDTIGRKVEEILKEWGIRNVSTITVDNASSNDVAVAYLKKRINNMGGLMGDGSFFHLRCCAHILNLVVGDGL<br>KQNELSISSIRNAVRFVRSSPQRSAKFKECIEFARITCKKMLCLDVQTRWNTAYLMLDGAEKFQPAFEKLEGEDSGYLEFF<br>GEAGPPSIHDWENVRCFVRFLKIFYDATKEFSSSQEVSLHKAFHQLASVHCELKRSAMNLNTVLASMGSDMKQKYNKYW<br>GKIENINKLIYFGVILDPRYKFSYVEWCFNDMYGDQPTFFTDLIAVIHTQLFKLFNWYKDAYDQQHNSGHPSASPSESSYV<br>SENVIPAEVPSHLARAEAFKEHLKLKESIVKKNELERYLDEERAEDVNFEILLWWKQNSCRYPVLSSMVRDVLATPVSTVA<br>SESAFSTGGRVLDTYRSSLNPQMAEALICAQNWLKPTLNQFKDLNINEEFELSATVVSEFDGPSANGSTSCGVRAAVVQ<br>GDASSSQSQPMSCD	zf-BED--DUF-domain--Dimer_Tnp_hAT--	I
XP_024629894.1	Mtu Zf-BED08_II	MEVEGVQEAHLQQGQDGEVTEAGAITEVGAATTVSEPKKTPQRRSKVWKHFSRNKNLVVCKYCGKEYAANSSSHGTTN<br>LGKHLKVCLKNPYRVVDKKQKTLAIEIGSEDDRNSVSFKLVDFSQEKTRLALAKMIIIDELPFKHVENEGFKMFMGKAQPKF<br>KIPSHDSVARDYLRLYFDEKETLKYMLSANNQMVSLTTDTWTSIQNMNYMCVTAHYIDDEWILKKKILSFNIIADHKGETIRI<br>ALENCMKD	zf-BED	II
XP_024630171.1	Mtu Zf-BED09_I	MDMSDAVIVKSSRLKSVVWNDFDRIKKGDTCVAVCRHCKKKLSGSSTSGTSHLRNHLIRCQRRSNHGLAQYITAREKRK<br>EGSLAITNFSLDQDPNKDDTVSLVNIKFEQAQLKDESVNTGNSNFDQRRSRFDLARMIILHGYPLAMVEHVGFRAFVKNL<br>QPLFELVTLNRVEADCIEIYDKERKKMNEMLDKLPGKISISADVWTANGDAEYLCLTSNYIDESWELRRRILNFIRIDPSHTG<br>DILSEAVMSCLMDWDIDRKLFSMILDGCSTCDNIATRIGERLMQNRFLYCNGQLFDIRCVANVLNVMSQLALGAVTEIVHKI<br>RETTRYIKSSQTVLAKFNEMAKEVGIISQKCLFLDNPMQWNSTYSMLEAALEFKDVLILLQENDIAYNISLSEVEWERLAIVT<br>SYLKLFVEVINIFTRSKYPTANIYFPELCDVKLHFIEWGKNSDPCISSLVVQLRSKFDEYWDKCSLGLAAAAMLDPRFKMKL<br>VDYYYPQIYGSMSASRIEEVFEGVKALYNEHSIGSPLASHDQGLAWQVENGSSSLSLPWSAKDSRDRLMGFDKFLHETS<br>QGEGAKSDLDKYLEEPLFPRNVDFNILNWWKVHTPRYPVLSMMARNVLGIPMSKVAPELAFNYSGRVLDRDWSSLNPAT<br>VQALVCSQDWIRSELEN	zf-BED--DUF-domain--Dimer_Tnp_hAT--	I
XP_024630464.1	Mtu Zf-BED10_I	MEPDPAQFHHTTIGSFLSSLPPLQTKPYHPSFSSGSDPSISSLSIECALNSQAYAMEISNDSEAKKPKRLTSVVWNHFERIK<br>KADICYAVCVHCNKRLSGSSNSGTTHLRNHLLRCLKRSNFDVSQLLAVKRKKKDTTVGLANISFDEGQRKEECIKPTFAKF<br>EQEHKKDEIINFVSSKFDQERSQHDLARMIILHGYPVTLAEQVGFKVFVKNLQPLFEFSPNSGVEISCIEIYRREKEKVFEMI<br>SKLCGRINLSIEMWSSAENTSYLCLSAHYIDENWTLQKKVLNFLTLHSSYTEDLLPEVIIKSLDEWDIDCKLFALTLDDCSVD<br>DSITLRIKERVSEKRPFLSTRQILDVRSAAHLITSIVQDAMDALHEVIQKIRESIRYIKSSQEVQGKFNEISQSAGINFQKALFL<br>DNPLQWKSTFIMLETALEYRSAFSLLQEHDTSYSSTLSDEEWEWASSVAGYLKLLVEIMNIFSGNRFPTSNIFFPEICDIHIQ<br>LIDWSRSSDHFLRLMALKMKSKFDKYWSKCSLALALAAVLDPRFKLKLVEYYYSLIYGSTALERIKEVSDGIKELFNAYSICS<br>TMVDQGSALPGSSLPSTSSGARDRLKGFDRFLHETSQSQSMTSDLDKYLEEPIFPRNSDFNILNWWKVHTPRYPILSMM<br>ARDVLGTPMSTLAPELAFNTGGRLLDSSRSSLNPDTREALICTHDWLRNESEGLNSSPIQSIPPILLESS	zf-BED--DUF-domain--Dimer_Tnp_hAT--	I
XP_024630465.1	Mtu Zf-BED11_I	MEPDPAQFHHTTIGSFLSSLPPLQTKPYHPSFSSGSDPSISSLSIECALNSQAYAMEISNDSEAKKPKRLTSVVWNHFERIK<br>KADICYAVCVHCNKRLSGSSNSGTTHLRNHLLRCLKRSNFDVSQLLAVKRKKKDTTVGLANISFDEGQRKEECIKPTFAKF<br>EQEHKKDEIINFVSSKFDQERSQHDLARMIILHGYPVTLAEQVGFKVFVKNLQPLFEFSPNSGVEISCIEIYRREKEKVFEMI<br>SKLCGRINLSIEMWSSAENTSYLCLSAHYIDENWTLQKKVLNFLTLHSSYTEDLLPEVIIKSLDEWDIDCKLFALTLDDCSVD<br>DSITLRIKERVSEKRPFLSTRQILDVRSAAHLITSIVQDAMDALHEVIQKIRESIRYIKSSQEVQGKFNEISQSAGINFQKALFL<br>DNPLQWKSTFIMLETALEYRSAFSLLQEHDTSYSSTLSDEEWEWASSVAGYLKLLVEIMNIFSGNRFPTSNIFFPEICDIHIQ<br>LIDWSRSSDHFLRLMALKMKSKFDKYWSKCSLALALAAVLDPRFKLKLVEYYYSLIYGSTALERIKEVSDGIKELFNAYSICS<br>TMVDQGSALPGSSLPSTSSGARDRLKGFDRFLHETSQSQSMTSDLDKYLEEPIFPRNSDFNILNWWKVHTPRYPILSMM<br>ARDVLGTPMSTLAPELAFNTGGRLLDSSRSSLNPDTREALICTHDWLRNESEGLNSSPIQSIPPILLESS	zf-BED--DUF-domain--Dimer_Tnp_hAT--	I
XP_024630466.1	Mtu Zf-BED12_I	MEISNDSEAKKPKRLTSVVWNHFERIKKADICYAVCVHCNKRLSGSSNSGTTHLRNHLLRCLKRSNFDVSQLLAVKRKKK<br>DTTVGLANISFDEGQRKEECIKPTFAKFEQEHKKDEIINFVSSKFDQERSQHDLARMIILHGYPVTLAEQVGFKVFVKNLQP<br>LFEFSPNSGVEISCIEIYRREKEKVFEMISKLCGRINLSIEMWSSAENTSYLCLSAHYIDENWTLQKKVLNFLTLHSSYTEDL<br>LPEVIIKSLDEWDIDCKLFALTLDDCSVDDSITLRIKERVSEKRPFLSTRQILDVRSAAHLITSIVQDAMDALHEVIQKIRESIR<br>YIKSSQEVQGKFNEISQSAGINFQKALFLDNPLQWKSTFIMLETALEYRSAFSLLQEHDTSYSSTLSDEEWEWASSVAGYL<br>KLLVEIMNIFSGNRFPTSNIFFPEICDIHIQLIDWSRSSDHFLRLMALKMKSKFDKYWSKCSLALALAAVLDPRFKLKLVEYY<br>YSLIYGSTALERIKEVSDGIKELFNAYSICSTMVDQGSALPGSSLPSTSSGARDRLKGFDRFLHETSQSQSMTSDLDKYLE<br>EPIFPRNSDFNILNWWKVHTPRYPILSMMARDVLGTPMSTLAPELAFNTGGRLLDSSRSSLNPDTREALICTHDWLRNES<br>EGLNSSPIQSIPPILLESS	zf-BED--DUF-domain--Dimer_Tnp_hAT--	I
XP_024632139.1	Mtu Zf-BED13_II	MANEEVQSPNDVSPTQQTQTALDETNVESQEPEAVREPSVSPNKRGLKFVYWRHYNRQKFDGKFKAICKYCEKKLGGE<br>TANDTTHLKDHLSICAARNKRSPMQILLKVSEVPGKGKESFVAGTYTFNQDISRTELAKMIILHEYPIMMVDHAGFRSFIHS<br>LQPLFKIPSRNTMKNDIMKLCESERAKQMNSFGKNKSRIAITTDMWTSSNQNKGYMAVIAHYIDNNWNLQNRIIR	zf-BED	II
XP_024632581.1	Mtu Zf-BED14_III	MAPIRSTGFVDPGWDHGIAQDERKKKVRCNYCGKVVSGGIYRLKQHLARVSGEVTYCEKAPEEVYLKMKENLEGCRSN<br>KKQKQVDAQAYMNFQSNDDEDDEEQVGCRSKGKQLMDGRNVSVNLTPLRSLGYVDPGWEHGVAQDERKKKVKCSYC<br>EKVVSGGINRFKQHLARIPGEVAPCKSAPEEVYLKIKENMKWHRTGKRHRQPEAKDLMPFYPKSDNEDDEYEQQEDTLH<br>HMNKEALIDIDRRYSKDTGKTFKGMSSNTSPEPALRRSRLDSFYLKHPTNQNLQTCKQLKVKTGPTKKLRKEVFSSICKFF<br>CHAGIPLQAADSVYFHKMLELAGQYGQGLACPSSQLISGRFLQEEINSIKNYLAEYKASWAITGCSIMADSWRDAQGRTII<br>NFLVSSPHGVYFVSSVDATNVVEDATYLFKLLDKVVEELGEENVVQVITENTPNYKAAGKMLEERRRNLFWTPCAIYCINQ<br>VLEDFLKIRCVEECMEKGQKITKLIYNQIWLLNLMKSEFTHGNELLKPAGTQCASSFATLQNLLDHRVSLRRMFLSNKWMS<br>SRFSSSSQGKEVQKIVLNVTFWKKMQSVRNSVYPILQVFQKVSSGESLSMPYIYNDLYRAKLAIKSIHGDDARKYEPFWK<br>VIDRHCNSLFCHPLYLAAYFLNPSYRYRQDFVSHSDVVRGLNECIVRLELDNMRRISASMQIPHYNSAQDDFGTELAISTR<br>TGLEPAAWWQQHGISCLELQRIAVRILSQTCSSFACEHDGSMYDQIYSKRKNRLSQKKLNDIMYVHYNLRLRECQVRKRS<br>RESKSTSAENVLQEHLLGDWIVDTTAQSSDSDKNIPFGVELDDEYENDSVDYDDGSERHLKGSHELVTMADGAVGSSDA<br>DHANIDGASDDESDLNYFDDDMSE	zf-BED--zf-BED--DUF-domain--Dimer_Tnp_hAT--	III
XP_024632694.1	Mtu Zf-BED15_II	MAEEQNTQNVERVIENAELPSPEPNEQQTTSKKTKVPKKDKKRKSGEPTAEEEEGEETEEDGGKRKPTKPRSWTWDHF<br>TKYNCPKSRKRRARCKWCEATYACDTHRNGTSNLKNHLLTQCKKFPRETTNDA	zf-BED	II
XP_024633643.1	Mtu Zf-BED16_I	MEQNLELVPITSQKHDPAWKHCQLFKNGEKVQLKCNYCLKFFKGGGIHRFKEHLACQKGNASMCSSVPADVRQHMQQS<br>LDGVVVKKRKRQKIEEEIMNVNPLATVLNGGNNQMDVNHHGVHVQPIGVHDPMEQNPVSCQMFHSPGEGMSRNVERR<br>KKIRATKNQNQNPAAVVYNTNYEPEPVVAPMEKNTLFPTKVDHQIHMAIGRFLYDIGAPFDAVNSIYFEQMVEAIASGGSG<br>FQRPSHHELRGWVLKNSVEDVKNDVDRCKMTWGRTGSSILVDQWTTEAGRILISFLAYCPEGVVFLKSLDATERLISEDF<br>LYGLMKEVVEEVGVGQVVQVITSAEKWYADAGRMLTETYPSLYWSPSAAHCIDLILEDFGNIEWISTVIEQAKSITRFVYNY<br>SAILNMVKRYTLGNDIVDPSFSRFTTDFTTLKRMVVLKHNLQAMVTSQEWMDCPYSKEAAGLEMLDILSNQTFWSSCEMI<br>VRLTHPLLRVLRTASSEMRPAMGYTYEGIYRAKEAIKKALIKREDYMVFWNIIHQRWDSLWHHPLHAAGFYLNPKFFYSIQ<br>GDVPNEIRSGMLDCIERLVPDTRVQDKISKELNLYKSAAGDFGRKMAIRARDNLLPSEWWSTYGGGCPNLSRLAIRILSQT<br>SSVMFCKRNQIPFEQIINTRNYIERQHFTDLVFVHYNLRLRQMFMNKEQESSDPLSFDNICNVEDWIGPRDLYFGEYGNS<br>DWMALDSSSINTMPLRSLNDEPEEMCEGFDDDEIFISLKDDEDVNSGDKFENH	zf-BED--DUF-domain--Dimer_Tnp_hAT--	I

XP_024633644.1	Mtu Zf-BED17_I	MEQNLELVPITSQKHDPAWKHCQLFKNGEKVQLKCNYCLKFFKGGGIHRFKEHLACQKGNASMCSSVPADVRQHMQQS<br>LDGVVVKKRKRQKIEEEIMNVNPLATVLNGGNNQMDVNHHGVHVQPIGVHDPMEQNPVSCQMFHSPGEGMSRNVERR<br>KKIRATKNQNQNPAAVVYNTNYEPEPVVAPMEKNTLFPTKVDHQIHMAIGRFLYDIGAPFDAVNSIYFEQMVEAIASGGSG<br>FQRPSHHELRGWVLKNSVEDVKNDVDRCKMTWGRTGSSILVDQWTTEAGRILISFLAYCPEGVVFLKSLDATERLISEDF<br>LYGLMKEVVEEVGVGQVVQVITSAEKWYADAGRMLTETYPSLYWSPSAAHCIDLILEDFGNIEWISTVIEQAKSITRFVYNY<br>SAILNMVKRYTLGNDIVDPSFSRFTTDFTTLKRMVVLKHNLQAMVTSQEWMDCPYSKEAAGLEMLDILSNQTFWSSCEMI<br>VRLTHPLLRVLRTASSEMRPAMGYTYEGIYRAKEAIKKALIKREDYMVFWNIIHQRWDSLWHHPLHAAGFYLNPKFFYSIQ<br>GDVPNEIRSGMLDCIERLVPDTRVQDKISKELNLYKSAAGDFGRKMAIRARDNLLPSEWWSTYGGGCPNLSRLAIRILSQT<br>SSVMFCKRNQIPFEQIINTRNYIERQHFTDLVFVHYNLRLRQMFMNKEQESSDPLSFDNICNVEDWIGPRDLYFGEYGNS<br>DWMALDSSSINTMPLRSLNDEPEEMCEGFDDDEIFISLKDDEDVNSGDKFENH	zf-BED--DUF-domain--Dimer_Tnp_hAT--	I
XP_024634393.1	Mtu Zf-BED18_IV	MSSQANEEPTQAGANTTQAEANATQEEETQAEPIVGRKRKKTSVIWKDFDEKEITKGVFRAVCKHCKAQYTTGTVGSST<br>SQMKRHLVSCTAKKLQDATEKRQAAIPFKRVSSGNPFLTSGVGYSNERMREIIATAVMVHEYPFNVVEDDVWMWAFEYA<br>NPEFRKVTHKTTRSDCLKLFENEKKILKKQLESVSKISLTTDMWKSSHQVVEYMVITGHFIDAGWNLQKRVLSFVKVPAPR<br>RGIDVADAIHKCLKTWRIESKIFTVSVDNAAYNDLCLKYLKDNISMSRKLILNGDLFHVRCCAHILNLLVQDGLSKIKDIIFNIR<br>ESVKYVNHNDARLKNFCDVVEQKGLKERKLVIDCPTRWNSTFNMASTALKFKIVFSAYKEREPHYDHAPSFEEWDKVEK<br>VCKLLEVFNSATHVISGSEYPTANLYLPEVWRVKQVLDMADEDEDLFMREMAKPMKKKFDKYWGESNLLMAIASVLDPR<br>CKFHSVCICFPKIYKSKEVSDENIEKVRRSLELLYDEYVALSLEESYLMPAVNLDNSSSSQTNVKNATGIDDLLQTIREQQAI<br>SPTKSELQDYLDQGVHVVPNSESFSALEWWRNNSMKVHIQCWR	zf-BED--DUF-domain	IV
XP_024634679.1	Mtu Zf-BED19_IV	MASSEAPTQPSAEASTQDSQTQRNVRAKTDIAWGHAKIVLDGEKEKPQCIYCNKVIKGGGINRLKLHLAGETGQVEACSQ<br>APEEVRFKMKQNREETRLKKRKTEHAIGNEEIGNEEQTQRGQPRCRSVVVASQKGTNNGSFDNYFLPRTTPGSQPTIKS<br>VLQTKEVVEKCDLSLAKWFIAASIPFNAANSPYFQSAVDALCCMGAGYKAPSIHDLREMLFKLFKEVVLYIGPENVVHIVTD<br>NAANYVAAGRLLEKEFPGLYWSPCAAHCINLMFQDIGKLPEVKEAVSHATNVTKYIYNHCYPLYLMRKFTHGREILRPAPT<br>RFASNFIALQSILSQKNALRAMVTSQEWTTSAYAKEAKAKQFVEQVLNTNFWTACADIVKLTEPLVGVLRLVDSEDKPSM<br>GFLYRNMYKAREEMVKRFQRNKTKVEPYLKIIDDRWDSQLRKNLHAAGYWLNPSCRFSPEFEKHKSTTSGLIDVIEKYAR<br>NNHELRANLYIFIMIYRNNKIRNYDPINDELLDDHHDNWVLEDSSPFLTVEELESSRNDLANMTIQPISNDIDGLNLDEDDDY<br>GNDAPDTNAENMDQSNVFDEAAGEDVEFLDELQIQSILTPWN	zf-BED--DUF-domain	IV
XP_024635336.1	Mtu Zf-BED20_I	MVREKDVCWEYAEKLDGNKVKCKFCQRVLNGGISRLKHHLSRFPSKGVNPCSKVRDDVTDRVRNIIASKEEVKETSSVK<br>KQKVSEVISPGSHSATKALISLDTTLPIGKMFPSSNPMTPSSTNNQENAERSIALFFFENKLDFSVARSSSYQLMIDAITKC<br>GPGFTGPSAEILKTIWLERIKSEVGLQSKDVEKEWATTGCTIIADTWTDYKSKAIINFLVSSPSRIFFHKSVDASAYFKNTKW<br>LADLFDSVIQEFGPENVVQIIMDSSFNYTGIGNHIVQNYGTIFVSPCASQCLNLILEEFTKIDWISRCILQAQTISKLIYNNASL<br>LDLMKSYSGGQELIRTGATKSVSTFLSLQTMLKLRTRLKHMFHSPEYALDTSYANKPQSLSCIAIAEDGDFWRTVEECVAI<br>SEPFLKVLREVSEGKPTVGSIYELMTRAKESIRTYYIMDENKCKTFLDIVDKKWRDQLHSPLHAAAAFLNPSIQYNPEIKFLS<br>SIKEDFYHVLEKLLPVPDMRRDITNQIYTFTKAHGMFGCSLAKEARNTVAPWLWWEQYGDSAPGLQRVAIRILSQVCSTF<br>SFQRQWSTFRQIHSEKKNKIDRETLNDLVYINYNLKLNRQMSAKSLEVDLLQFDDIDMTSEWVEENETVSPPTQWLDRFG<br>SALDGNDLNTRQFGSSIFGANDPIFGL	zf-BED--DUF-domain--Dimer_Tnp_hAT--	I
XP_024636168.1	Mtu Zf-BED21_IV	MASSEAPTQPSAEASTQESQTQRNVRAKTDIAWGHAKIVLHGDKEKPQCIYCNKVMKGGGINRLKLHLAGETGQVEACS<br>QAPEEVRFKMKQNREETRLKKRKTEHAIGNEEQTQREQPRSVVVASQKGTNNGSFDNYFLPRTTPGSQPTIKSVLQTKE<br>VVEKCNLALAKWFIAASIPFNAANSPYFQSAVDALCCMGAGYKAPSIHDLREMLFKLFKEVVLYIGPENVVQIVTDNAANY<br>VAAGRLLEKEFHGLYWSPCATHYINLMFQDIGKLPEVKEAVSHATNVTKYIYNHCYPLYLMRKFTHGREILRPAPTRFATN<br>FIALQSILSQKNALRAMVTSQEWTTSAYAKEAKAKQFVEQVLNTNFWTACADIVKPTEPLVGVLRLVDSEDKPAMGFLYR<br>NMYKAREEMVKRFQRNKTKVEPYLKIIDDRWDSQLRKNLHAAGYWLNPSCRFSPEFEKHKSTTSGLIDVIEKYARNNHEL<br>RANLYIFIMIYRNNKIRNYDPINDELLDDHHDNWVLEDSPPFLTVEELESLRNDLANMTIQPISNDIDGLNLDEDDDYGNDA<br>PDTNAENMDQSNVFDKAAREDVEFLDELQIQSILTPWN	zf-BED--DUF-domain	IV
XP_024636685.1	Mtu Zf-BED22_I	MEFETRIVTPINNNEKPDSGTQPSQKKKRSIVWEYFTVEAVEAGKSRAYCKHCNKSFHYISGSKTVGTTNLKKHISLGLCQ<br>GMQQKSQPNSHPENGGLQDNAIRPKKRQRETPAYAGNGISFDQERCINDIAKMIILHDYPLDIVKHQGFIAFVRTLHPMFN<br>PSCLNSVEGCVSSMYLREKQNLLDLINGIPGRLNLTLDLWTSNQETGYVFIRGHFIDSDCNVHHPILNVVKVPFPDSGDSL<br>NQTIMTCISDWHLEGRVLTLALDKSFSSETVKVNLRGNLSVNNPVILSGQLLNQNCYARVLSRLAVDVLRAMSKTISKVRE<br>CVKFVKYSESHEEKFIELKQQLQVPGLVNLLIDDQYNWDTTYHMLVAACELKEVFACFDATCPDFSMTLTMEDWKQVETL<br>CTYLKYMYDAAHILTAQQLPTANLFFLEVSKLHMELTNAAFSQDPFLSSLIFPLLKNFDQYWRDSCLILAVAVAMDPRHKM<br>KLVESTFTTIFGENAEPWIRIVEDGLHELFIDYNTEMLHFTATNGDDVDEIMLNTEPYEGPVDGSLFVDEGGLSDFEFNISD<br>FTSMQQFKSEMDEYLEEPLLSESKEFDILSWWRENRSKYPTLSRAASDILSMSISTVSADSVFDTEVRKMDDYRSSLDSQ<br>TLEALICAKDWFQHNSITKNVSNTPRK	zf-BED--DUF-domain--Dimer_Tnp_hAT--	I
XP_024637073.1	Mtu Zf-BED23_II	MDIRDFPTSETSEHEVSSPTPAANPLPPPSRVRKNRSEAWDHFIVVSEEEKRAKCLYCPKEIKFNGGTSSMRNHWLKHQ<br>EEDSNKKQKSGSCSTADMEGNSSAISKFDQMELRKALGKVFIGLELPFRKVDHEALHHFLNLGIPQFKIPSRTTLSRDILQ<br>MWGNEKVRLKTFLSQHCGRVCLTTDFWTSCQNYSYMSLTAHFVDNNWKLNKKILNFCQVPGHSGDVMAHTVWNCLTV<br>WGLNKVLTMTVDNASSNDVGISQLKKALMPDGLLMGGEYFHTRCCAHVLNLIVKEGLKDIEREVLRIRGAVRYVQASSSR<br>LQRFKACIDPQKVQYKGFVNLDIETRWNTSYLMLDAALKHMPTFALLEMQDHTYVKELRKGKGFPLPVDWEYVRSILPFL<br>ELFYNATVRLSGSSYVTSNMYMFEVLERR	zf-BED	II
XP_024639905.1	Mtu Zf-BED24_IV	MASSEAPTQPSAEASTQESQTQRNVRAKTDIAWGHAKIVLDSDKEKPQCIYCNKVMKGGGINRLKLHLAGETGQVEACS<br>QAPEEVRFKMKQNREETRLKKRKTEHAIGNEEQTQREQPRSVVVASQKGTNNGSFDNYFLPRTTPGSQPTIKSVLQTKE<br>VVEKCDLALAKWFIAASIPFNAANSPYFQSAVDALCCMGAGYKAPSIHDLREMLFKLFKEIVLYIGPENVVQIVTDNAANYV<br>AAGRLLEKEFPGLYWSPCAAHCINLMFQDIGKLPEVKEAVSHATNVTKYIYNHCYPLYLMRKFTHGREILRPAPTRFATNFI<br>ALQSILSQKNALRAMVTSQEWTTSAYAKEAKAKQFVEQVLNTNFWTACADIVKLT	zf-BED--DUF-domain	IV
XP_024639922.1	Mtu Zf-BED25_I	METGSGMIQVNDSLTQPGTTLSDNVDNSDRDQSQSPTDLIHPLTDDEDDVDACELTNEGKRKKIYDVWDHFKRKTIKGE<br>EKAVCNYFNKALTGKRTHGTSHLKKHYLSCKRKPYGDIRQAILLREQKKTDGSSSYLSNYHFDPEKSRKDLASMIIIHEYPL<br>SIVDHLGFRAYSEGLQPLFKVPSRNTVKSDIIKIYENEKLKTMGLQDKIESKIALTSDMWTTSNQKKGYMVITAHYIGDDWS<br>MQSRILRFVYVECPHTAEVISEKLFKCMIDWNIDRKLSTITLDNCNTNDCAIGMLINKLDCSTLMLDGQLFHMRCCAHILNII<br>AQDGLSVLEDGIEKVRNSVAFWTATPKREQSFREAARQIKVTITKKLILDCKTRWNSTYHMLIVALAYKDVFIRLRTKDHLY<br>TSLPATGEWETAKEICSRLEVFNRVTEMFSGTQYPTTNIFFPLVCEIKLSLKKWVTCSIESIRNMASKMIVKFEKCWSVIHGI<br>MGIAIVLDPRYKMKLLEYFYPLLYGSASSNEISSIKQLCFSLFEEYQAKINEMVEGSTEKSNDNDSNIDDVPGSNYLSGYDS<br>FVNDTSDIQSKSELDNYLEEKVLPRSAAFDILGWWKTNGIKYPTLQKLAKDILAIPVSTVASESAFSTSGRLLCPHRSRLHE<br>DTLEALMCAQSWMKQEKKASIGIVGPMQYLEVKDKRLQTFQDSDSEDETDNTSVIGDAIKHVKELQERLRVLEEENKNSH<br>IESVVTLNKLLLIYKSWSDDGSKAASANNESLPHVDAKILDKNVLIRIQCQKQKSYLLNKLVEIQKLHLFVVNSSVLAIGDSIL<br>DITIIARAHI	zf-BED--DUF-domain--Dimer_Tnp_hAT--	I
XP_024640524.1	Mtu Zf-BED26_I	MASSEAPTQPSAEASTQESQTQRNVRAKTDIAWGHAKIVLDGDKEKPQCIYCNKVMKGGGINRLKLHLAGETGQVEACS<br>QAPEEVRFKMKQNREEQTQREQPRSVVVASQKGTNNGSFDNYFLPRTTPGSQPTIKSVLQTKEVVEKCDLALAKWFIAA<br>SIPFNAANSPYFQSAVDALCCMGAGYKAPSIHDLREMLFKLFKEIVLYIGSENVVQIVTDNAANYVAAGRLLEKEFPGLYW<br>SPCAAHCINLMFQDIGKLPEVKEAVSHATNVTKYIYNHCYPLYLMRKFTHGREILRPAPTRFATNFIALQSILSQKNALRAM<br>VTSQEWTTSAYAKEAKAKQFVEQVLNTNFWTACADIVKLTEPLVCVLRLVDSEDKPAMGFLYRNMYKAREEMVKRFQRN<br>KTKVEPYLKIIDDRWDSQLRKNLHAAGYWLNPSCRFSPEFEKHKSTTSGLIDVIEKYARNNHELRAKLNTETSIFRNSEGD<br>FGRKSAVEARNSPFPDEWWELYGCQAPHLQKLAIRVLSQTCSSSGCERNWSVFEHIHSKKRNRLEHQRLNDLVFVRYN<br>LMLENRNNKIRNYDPINDELLDDHHDNWVLEDSPPFLTVEELESLRNDLANMTIQPISNDIDGLNLDEDDDYGNDAPDTNA<br>ENMDQSNVFDEAAGEDVEFLDELQIQSILTPWN	zf-BED--DUF-domain--Dimer_Tnp_hAT--	I
XP_024640749.1	Mtu Zf-BED27_I	MESISQNLSSTLTSGSANVAEPTEQVAVATQVPVVGLPPLPCLAKRRKPNAGGPRRTSPAWDHFIKLPDEPEPTAACIHC<br>HKRYLCDPKTHGTSNLLAHSKVCFKNPQNDPTQASLMFSNGEGGTLVAASQRFNPAACRKAIALFVLLDEHAFRVVEGE<br>GFKLLCRQLQPLLTIPSRRTVARDCFQLFLDENLRLKTYFKSDCVRVALTTDCWTSGQNFSYMTLTAHFINNDWKYEKRIL<br>SFCTVPNHKGDTIGRKVEEILKEWGIRNVSTITVDNASSNDVAVAYLKKRINNMGGLMGDGSFFHLRCCAHILNLVVGDGL<br>KQNELSISSIRNSVRFVRSSPQRSAKFKECIEFARITCKKMLCLDVQTRWNTAYLMLDGAEKFQPAFEKLEGEDSGYLEFF<br>GEAGPPSIHDWENVRCFVRFLKIFYDATKEFSSSQGVSLHKAFHQLASVHCELKRSAMNLNTVLASMGSDMKQKYNKY<br>WGKIENINKLIYFGVILDPRYKFSYVEWCFNDMYGDQPTFFTDLIAMIHTQLFKLFNWYKDAYDQQHNSGHPSASPSESSY<br>VSENVIPAEVPSHLARAEAFKEHLKLKESIVKKNELERYLDEERAEDVNFDILLSWKQNSCRYPVLSSMVRDVLATPVSTV	zf-BED--DUF-domain--Dimer_Tnp_hAT--	I

		ASESAFSTGGRVLDTYRSSLNPQMAEALICAQNWLKPTLNQFKDLNINEEFELSTTVVSEFDGPSANGSTSCGVRAAVVQGDASSSQSQPMSCD		
XP_024641085.1	MtuZf-BED28_II	MTNEITTSSEIDYECEDFNSSKRPKMTSKVWDEMERVQTTEGNKVLCKYCGKLLQDNCGTSHLKRHLVICPKRPKPLGVVTQDSMPSGYLRGIYSAKDSGSSKALMVRPLKAEPQSQVICFSPAPNYGAATVTIASVKNTSGTIGELNHKSSPTLLLPSVESPRNQEELSLDDGEMNAFYASLDVESPFKSPTQDTTVITESSNTTSPCEETSKALKTLQDLLSKEFSVLLQTGQCGTVKSTIEYLSKMSAVNGISSEMRLLILEVSREFTRWSCDYNDASKKIESASSHIMKADKVEESLEANKNEFKEVLSLENELCNQLATLEQRKKELEEQINAIKANISVFQSAKITATKRKREVFEEAKTLKAQRDELREQVPHLKDEREVAKKIQENIQAEWLKLGEKFNKSLNGVNSSEKLDGKSSIQACQSENSL	zf-BED	II
XP_024641086.1	MtuZf-BED29_II	MTNEITTSSEIDYECEDFNSSKRPKMTSKVWDEMERVQTTEGNKVLCKYCGKLLQDNCGTSHLKRHLVICPKRPKPLGVVTQDSMPSGYLRDSGSSKALMVRPLKAEPQSQVICFSPAPNYGAATVTIASVKNTSGTIGELNHKSSPTLLLPSVESPRNQEELSLDDGEMNAFYASLDVESPFKSPTQDTTVITESSNTTSPCEETSKALKTLQDLLSKEFSVLLQTGQCGTVKSTIEYLSKMSAVNGISSEMRLLILEVSREFTRWSCDYNDASKKIESASSHIMKADKVEESLEANKNEFKEVLSLENELCNQLATLEQRKKELEEQINAIKANISVFQSAKITATKRKREVFEEAKTLKAQRDELREQVPHLKDEREVAKKIQENIQAEWLKLGEKFNKSLNGVNSSEKLDGKSSIQACQSENSL	zf-BED	II
XP_024641497.1	MtuZf-BED30_IV	MASFEAPTQPSAEASTQESQTQRNVRAKTDIAWGHAKIVLDGDKEKPQCIYCNKVMKGGGINRLKLHLAGETGQVEACSQAPEEVRFKMKQNREETRLKKRKTEHAIGNEEQTQREQPRSVVVASQKGTNNGSFDNYFLPRTTPGSQPTIKSVLQTKEVVEKCDLALAKWFIAASIPFNAANSPYFQSAVDALCCMGAGYKAPSIHDLREMLFKLFKEIVLYIGPENVVQIVTDNAANYVAAGRLLEKEFPGLYWSPCAAHCINLMFQDIGKLPEVKEAVSHATNVTKYIYNHCYPLYLMRKFTHGREILRPAPTRFATNFIALQSILSQKNALRAMVTSQEWTTSAYAKEAKAKQFVEQVLNTNFWTACADIVKLTEPLVCVLRLVDSEDKPAMGFLYRNMYKAREEMVKRFQRNKTKVEPYLKIIDDRWDSQLRKNLHAAGYWLNPSCRFSPEFEKHKSTTSGLIDVIEKYARNNHELRANLYIFIMIYRNNKIRNYDPINDELLDDHHDNWVLEDSPPFLTVEELESLRNDLANMTIQPISNDIDGLNLDEDDDYGNDAPDTNAENMDQSNVFDEAAGEDVEFLDELQIQSILTPWN	zf-BED--DUF-domain	IV
XP_024642142.1	MtuZf-BED31_IV	MAVGGEDGDGGGSQITTMASENGNGGGGETGSSSSFRGRGRKNIRGNRTDIGWKYGTDVNGDARKVKCSFCAKVISGGVYRFKHHLAGTSDDSGPCAQVSDEVKMEMLKWVATLEEAAERKRKMAEIAQGNVTEDPAFEVEVSQHLQKVRGKASASGTQTKIDAIAKKPLKVEADDAVAEFFYTSAIAFNCIRNPAFAKMCVAIGKYGPDYKPPSYRDISDKLLVRAVDRTNEIVDKFKEEWKTTGCSIMSDGWTDRKRRSICNFMVNSPKGTVFLYSLDTSDISKTADKVFKMLDDVVEAVGEDNVIQVVTDNAANFKAGGELLMLKRTKLFWTPCAAHCIDLILEDFEKEMIIHNVTIKNARKLTTYIYNRTMLITMVRKFTNGRDLIRPALTRFATAYLTIGCLNDLKSSLINMFDSNDWKSSRFATTEEGKKMASGILDQRFWKNIGVCLKTAAPLMDVLHLVDSDEKPAMGYIYEAMDACKKQIQNNFNNVQKW	zf-BED--DUF-domain	IV
Zm00001d000412_P001	ZmZf-BED01_II	MEELVTRRRRGEPSSVNSASITQKISNAIKSDDPTWEHCFVPDMSKKQAIQCKYCDKVIHGGITRVKYHLANIGGFNVTKCKKVPAHVKQEMEAFLTKKTGEKEQRKKKKQRDRDEIDLDKSDGSNEEDNGHENDVIVLKSTRESNNCSSSRPPTNGGSGTIDNFYKPQSVEESDHKNKIQTKLSTHKREERRDRACEYICQFFYEAGIAHNTITLPSFGHMLEAIGAFGRGEWTVMYLPLVSSMDVCLRQRKKLLQDNKLKTPLHLDGYYLNPYFYYPNKSSIELDGSFRAAIISCITKMFDDEDTQDSIIEELSIYQDQQGAFGHDIAVRQRRNKTFNPDVVLDENVDQDEDHEGAHDEPSPEPSISKAQLPAKRKRHGHPRKKKLRSLKSLLSCDLERATCASSSESEDNESMQIEASNSDSRDE	zf-BED	II
Zm00001d003128_P001	ZmZf-BED02_I	MDLRISEGSGTNTTLDDQSIQSPTRRAKVWEYFLQELVEVDGVMKAVCKYCGAKLTSKRNSGTNSLRNHVADTCPKIPVEDRKQFIATMRKRTGEGSFVFDPRKTRECMVKWCISAEVAFNKFDDPFFATWMESLQPSFSGVGRQTMRNDCIARFKMMRQELRNELQSLNSRICLTSDLWTSNQKLGYLCLTAHYIDANFILKKKTIAFKDVKYPHTSLAVEEVITKCLIEWGIKEKVFTITLDNASNNQSACDLLRESGRSDMLFGGEHLHVRCCAHILNLLVQDGMAIAHGAIDKIRDLVRHIISSPSRIQAFNEIAERSGLPSKAGLILDVPNRWNSTHNMIMEAIEYKVVLKRYAEEQLEPSPDDEEWTNSEAIGEFLGAFEEATKAFSAHRSPTSHLFLHNVLCIHQALRNENWQINCVLEDLALAMDSKFDKYWEKGKYNMALVIATILDPSKKMDFLDFFYEKTCQQFIDIQINMSLAKQWFTKFFGEYAKIVQKDTTVSPIVATNTSTLGSPILGKRRLDKEFSQWSQTRGRRLAKSELDAYLEEEFVRTDERFEILSWWRTNTNKYPVLSAMARDLLAIPLSTVPSESAFSAGGRILADNRSSMTPETLECLVCCKDWLYEYPNIQVYPVVFGPS	zf-BED--DUF-domain--Dimer_Tnp_hAT--	I
Zm00001d003128_P002	ZmZf-BED03_II	MDLRISEGSGTNTTLDDQSIQSPTRRAKVWEYFLQELVEVDGVMKAVCKYCGAKLTSKRNSGTNSLRNHVADTCPKIPVEDRKQFIATMRKRTGEGSFVFDPRKTRECMVKWCISAEVAFNKFDDPFFATWMESLQPSFSGVGRQTMRNDCIARFKMMRQELRNELQSLNSRICLTSDLWTSNQKLGYLCLTAHYIDANFILKKKTIAFKDVKYPHTSLAVEEVITKCLIEWGIKEKVFTITLDNASNNQSACDLLRESGRSDMLFGGEHLHVRCCAHILNLLVQDGMAIAHGAIDKIRDLGDLCVVRWASDAI	zf-BED	II
Zm00001d003194_P001	ZmZf-BED04_IV	MDLRISEGSGTNTTLDDQSVQSPTRRAKVWEYFQQELVEVDGVMKAVCKYCGTKLTSKRNSGTNSLRNHVADTCSKIPVEDRKRFIATMRKRPGEGSFVFDPRKNRECMVKWCISVKVTFNKFDDPFFASWMESLQPSFSGVGRQTMRNDCIVRFKMMRQELRNELQSLNSRICLTSDLWTSNQKLGYLCLTAHYIDANFILKKKTIAFKDVKYPHTGLAIEEVITKCLIEWGIKEKVFTITLDNASNNQSACDLLRESGRSDMLFGGEHLHVRCCAHILNLLVQDGMAIAHGAIDKIRDLVRHIISSPSRIQAFNEIAERSGLSSKAGLILDVPNRWNSTHNMIMEAIEYKVVLKRYTEEQLEPSPDDEEWTNSEAIGEFLGAFEEATKAFSAHRSPTSHLFLHNVLCIHQALRNENWQINCVLEDLALAMDSKFDKY	zf-BED--DUF-domain	IV
Zm00001d003194_P002	ZmZf-BED05_II	MDLRISEGSGTNTTLDDQSVQSPTRRAKVWEYFQQELVEVDGVMKAVCKYCGTKLTSKRNSGTNSLRNHVADTCSKIPVEDRKRFIATMRKRPGEGSFVFDPRKNRECMVKWCISVKVTFNKFDDPFFASWMESLQPSFSGVGRQTMRNDCIVRFKMMRQELRNELQSLNSRICLTSDLWTSNQKLGYLCLTAHYIDANFILKKKTIAFKDVKYPHTGLAIEEVITKCLIEWGIKEKVFTITLDNASNNQSACDLLRESGRSDMLFGGEHLHVRCCAHILNLLVQDGMAIAHGAIDKIRDLVRHIISSPSRIQAFNEIAERSGLSSKAGLILDVPNRWNSTHNMIMEAIEYKVVLKRYTEEQLEPSPDDEEWTNSEAIGEFLGAFEEATKAFSAHRIYPVVFGPS	zf-BED	II
Zm00001d004256_P001	ZmZf-BED06_V	MGQLEATQIDTNTKLANVEMTVAHIDKSLVALLRRFDEMHANINGGRDEGAEDNPSSVASSSITQSTAATIKSDDPAWKHCYCPDLKKKHSLKCNYCDKLINAGITRVKYHLANIGGFNVSKCKKVPTPVKEDMVALLTKNCDAKEKKRKEKQRERDEIDLDNSGGDNSSEEESEHGNDVIVLKSTKGGSSSRLATTGGTIDKFYKPESIEESVQKNKRGLSTSQKIQTQLTTQKREERRDRACEYICQFFYEAGIAHNTVTLPSFAHMVEAIGAFGRANVLRRMDSDVPAMGFLHGCMLEAKKEIAMRFDNNENSFKVAWDIIDKRWDNKLKTPLHLAGYYLNPYFYYPNKSEIELDGSFRAAVIACITKTVEDEETQDNIIEELNVYQEQQGTFGHDIAVRQRRNKNFNPAKWWLNHGTSTPNLRLLATRILNLTCSSSGCERNWSDFEQVHTKKRNKLLHDRMRDLVFIKYNSRLRQKRENKSKDPLEREMNDVLEDDANEFITGLVPNANSDKDEEHGGAQVGETIHESQLSQPQPKRKRLVRPRKKKIRSLHSLLYGGLENEAVASSSESEDNGDGDISMHQYSDSDDLGDE	zf-BED--Dimer_Tnp_hAT--	V
Zm00001d006692_P001	ZmZf-BED07_XXII	MERVSSAPEMDDVISTDQSSRSTKRRAKVWDYVDSELVDGKEKAICKYCKAHLSSVAGKGTTHLNRHISTYCHAISQEERQRFLATQKTKPDEAHVFDPVVFRGLITNMAEKTIWGDDLVKHLIDVCKEEILAGNRPQGIFTRIGWKNVEDKFFARTKKKCTKTQLKNKLDNLKKDFTQFMELKIVATGLGWNEANQTVDCSNTWWDEHLEKCNNPERGTKCNHVRFRKHGPKHLDDLHFLFDKVHVTGATAMCPGDVSSCDSSSDDVLEVTEKTNEAWNKPKPKKRKQMSGAAQEKEEKSPFYQMYKNTCMKIESADEKISTSVEASSAPLQANLVPTIAEAMKMVKACGIQEKTAMMHTATSLIMKAEFREILFALETNEGRFDLIEREHKKSTT	zf-BED--MYB--	XXII
Zm00001d010895_P001	ZmZf-BED08_V	MSDEDRDREIERDRVWLHEDKIDTGFICKYCRETKSEGSDTRLKEHLAHRGKNDKKCPSVPPDIKDLWYPIGKFGTNYMFLESFMRRNDKFMVWFMSPEIRHSSYFLTEMERYAFDNITNVELWENMQYVLDEVEPLYMFLTFVDQEKSPTLGEVHMQYTNTKHTYQSKFENDSARYNTIMDVVDAKMNTVMTDTYVQPVCALHPYVNYVMGATNNLMTDLRKGVERMFDSNTAAMALQEYDFFKRKIGDFSSELARRMVVDRGTSPSSWWSMFGSDTPTLQRVAKRLLSLCASSSGCERNWSTFAFIHTKLRNKLDYLKLHELVFVNYNLHLRIQRATGTPEPSEFDPALAFMDLSLHRHNKAIRDWMERGRCNAPPTLDEDSTISDTPLPSTLFTLLVHEQGGTEKV	zf-BED--Dimer_Tnp_hAT--	V
Zm00001d011158_P001	ZmZf-BED09_VI	MIDDEESQGIKKVHFRDSFSQHPIVTYKRRRQQKPQTQQQLRQPPPSLQQHPEPQLQVKLEPKTEDVPEQQNNDTFWKSRDMGWKYGIMIDENRQHWKCMYCGLIRYGGGVSRLKRHLAGDLDVKMCPEVPADVVEEIREHLRKKRERRRKRAAQNGGDNVKAKNPSGDANVEKDLLPSNSVLPDGMDTNVLEEVTNQTGSVHHETTPRFPILRARDIGWEHAVDLDGNKRRWQCKFCSLCRSGGVTTLKAHLIDDSCPNVPKEISKKVSNFIEEKRATRLLLNNYVFNVDEDFNTILQEGTVEYVNEQQPSRKATYVQTLSKCAINEIAAGSKQCGAECSGQPVEHCDQPEEQCTMDYGRMDRLTSNKNQILDKNTENSKNTKMLKPCRKSEFNTRKHIIIVDKIGRHWKCRYCGMDVYGKKFQLHYHLAGAFRQAKCPNVPREVFAKARQHVLTKKMLKKSKAEQQIPSSPHILAQSGEERQNNDPFCGNQSQLSINNEPREVHNYPAVLRDSAWEHSLIYEKENGHWKCKWCSIEGDHGLTRLMWHLVGWQNRPQCPNIPKDVAEKMKDQMMSKKEQKARSGLFDGNGCCEVLCSSNSSQLDQNHLTARIHDRCSSQAFDHANSELKGCNMLSNTILSQQSSNPQVHHEDPQVCHEQERKEVATSSEPGCEQGQRMQWQSQNKPMMEGPHDNGLCGDTNQLEEQKSGFGISDCWRYVLDGQMHLPDVQEGAGIGTCIRDALLYGCAEFGTVPDKMEMDCDKTVDANTAKCQNILKDVLRSENFALLCSVLCRTVHQDGERTRYFDFGVIDSRMKNGNYGPEPELFVHDLKLLWEDLKVAGQDIVHLANNLSSLTEDSYEKLVGRERGSDDDELNEAVVARSEPKNLVQPNASVPLTSQGFNQLLDQPGPSDPSVVYKDSICNRCGKVAGAGSVLKCYR	zf-BED--PHD--	VI

		CMLPCHISCIEATGSPISTGRWCCKNCSAGTKEPVEGDMVLAHGNPNCLHESCVVCDRLAACRSPKCDNSRALVISSVDPEIDTCYSCKICGGTEEDEKRFLICGNVLCRYMYYHIGCLKSMQISTSVERGSPCWYCPSCLCRVCLCDKDDDLTILCDGCDEAYHIYCITPRHTSIPKGQWYCSSCSVERAEEGMRQYERRTLKLHRKEDAGLQSWNFDGVDLLLSAAEQLRIDEQLETRTD		
Zm00001d011158_P002	ZmZf-BED10_VI	MIDDEESQGIKKVHFRDSFSQHPIVTYKRRRQQKPQTQQQLRQPPPSLQQHPEPQLQVKLEPKTEDVPEQQNNDTFWKSRDMGWKYGIMIDENRQHWKCMYCGLIRYGGGVSRLKRHLAGDLDVKMCPEVPADVVEEIREHLRKKRERRRKRAAQNGGDNVKAKNPSGDANVEKDLLPSNSVLPDGMDTNVLEEVTNQTGSVHHETTPRFPILRARDIGWEHAVDLDGNKRRWQCKFCSLCRSGGVTTLKAHLIDDSCPNVPKEISKKVSNFIEEKRATRLLLNNYVFNVDEDFNTILQEGTVEYVNEQQPSRKATYVQTLSKCAINEKIAAGSKQCGAECSGQPVEHCDQPEEQCTMDYGRMDRLTSNKNQILDKNTENSKNTKMLKPCRKSEFNTRKHIIIVDKIGRHWKCRYCGMDVYGKKFQLHYHLAGAFRQAKCPNVPREVFAKARQHVLTKKMLKKSKAEQQIPSSPHILAQSGEERQNNDPFCGNQSQLSINNEPREVHNYPAVLRDSAWEHSLIYEKENGHWKCKWCSIEGDHGLTRLMWHLVGWQNRPQCPNIPKDVAEKMKDQMMSKKEQKARSGLFDGNGCCEVLCSSNSSQLDQNHLTARIHDRCSSQAFDHANSELKGCNMLSNTILSQQSSNPQVHHEDPQVCHEQERKEVATSSEPGCEQGQRMQWQSQNKPMMEGPHDNGLCGDTNQLEEQKSGFGISDCWRYVLDGQMHLPDVQEGAGIGTCIRDALLYGCAEFGTVPDKMEMDCDKTVDANTAKCQNILKDVLRSENFALLCSVLCRTVHQDGERTRYFDFGVIDSRMKNGNYGPEPELFVHDLKLVGRERGSDDDELNEAVVARSEPKNLVQPNASVPLTSQGFNQLLDQPGPSDPSVVYKDSICNRCGKVAGAGSVLKCYRCMLPCHISCIEATGSPISTGRWCCKNCSAGTKEPVEGDMVLAHGNPNCLHESCVVCDRLAACRSPKCDNSRALVISSVDPEIDTCYSCKICGGTEEDEKRFLICGNVLCRYMYYHIGCLKSMQISTSVERGSPCWYCPSCLCRVCLCDKDDDLTILCDGCDEAYHIYCITPRHTSIPKGQWYCSSCSVERAEEGMRQYERRTLKLHRKEDAGLQSWNFDGVDLLLSAAEQLRIDEQLETRTD	zf-BED--PHD--	VI
Zm00001d011158_P003	ZmZf-BED11_VI	MIDDEESQGIKKVHFRDSFSQHPIVTYKRRRQQKPQTQQQLRQPPPSLQQHPEPQLQVKLEPKTEDVPEQQNNDTFWKSRDMGWKYGIMIDENRQHWKCMYCGLIRYGGGVSRLKRHLAGDLDVKMCPEVPADVVEEIREHLRKKRERRRKRAAQNGGDNVKAKNPSGDANVEKDLLPSNSVLPDGMDTNVLEEVTNQTGSVHHETTPRFPILRARDIGWEHAVDLDGNKRRWQCKFCSLCRSGGVTTLKAHLIDDSCPNVPKEISKKVSNFIEEKRATRLLLNNYVFNVDEDFNTILQEGTVEYVNEQQPSRKATYVQTLSKCAINEIAAGSKQCGAECSGQPVEHCDQPEEQCTMDYGRMDRLTSNKNQILDKNTENSKNTKMLKPCRKSEFNTRKHIIIVDKIGRHWKCRYCGMDVYGKKFQLHYHLAGAFRQAKCPNVPREVFAKARQHVLTKKMLKKSKAEQQIPSSPHILAQSGEERQNNDPFCGNQSQLSINNEPREPMMEGPHDNGLCGDTNQLEEQKSGFGISDCWRYVLDGQMHLPDVQEGAGIGTCIRDALLYGCAEFGTVPDKMEMDCDKTVDANTAKCQNILKDVLRSENFALLCSVLCRTVHQDGERTRYFDFGVIDSRMKNGNYGPEPELFVHDLKLLWEDLKVAGQDIVHLANNLSSLTEDSYEKLVGRERGSDDDELNEAVVARSEPKNLVQPNASVPLTSQGFNQLLDQPGPSDPSVVYKDSICNRCGKVAGAGSVLKCYRCMLPCHISCIEATGSPISTGRWCCKNCSAGTKEPVEGDMVLAHGNPNCLHESCVVCDRLAACRSPKCDNSRALVISSVDPEIDTCYSCKICGGTEEDEKRFLICGNVLCRYMYYHIGCLKSMQISTSVERGSPCWYCPSCLCRVCLCDKDDDLTILCDGCDEAYHIYCITPRHTSIPKGQWYCSSCSVERAEEGMRQYERRTLKLHRKEDAGLQSWNFDGVDLLLSAAEQLRIDEQLETRTD	zf-BED--PHD--	VI
Zm00001d011158_P005	ZmZf-BED12_VI	MIDDEESQGIKKVHFRDSFSQHPIVTYKRRRQQKPQTQQQLRQPPPSLQQHPEPQLQVKLEPKTEDVPEQQNNDTFWKSRDMGWKYGIMIDENRQHWKCMYCGLIRYGGGVSRLKRHLAGDLDVKMCPEVPADVVEEIREHLRKKRERRRKRAAQNGGDNVKAKNPSGDANVEKDLLPSNSVLPDGMDTNVLEEVTNQTGSVHHETTPRFPILRARDIGWEHAVDLDGNKRRWQCKFCSLCRSGGVTTLKAHLIDDSCPNVPKEISKKVSNFIEEKRATRLLLNNYVFNVDEDFNTILQEGTVEYVNEQQPSRKATYVQTLSKCAINEIAAGSKQCGAECSGQPVEHCDQPEEQCTMDYGRMDRLTSNKNQILDKNTENSKNTKMLKPCRKSEFNTRKHIIIVDKIGRHWKCRYCGMDVYGKKFQLHYHLAGAFRQAKCPNVPREVFAKARQHVLTKKMLKKSKAEQQIPSSPHILAQSGEERQNNDPFCGNQSQLSINNEPREVHNYPAVLRDSAWEHSLIYEKENGHWKCKWCSIEGDHGLTRLMWHLVGWQNRPQCPNIPKDVAEKMKDQMMSKKEQKARSGLFDGNGCCEVLCSSNSSQLDQNHLTARIHDRCSSQAFDHANSELKGCNMLSNTILSQQSSNPQVHHEDPQVCHEQERKEVATSSEPGCEQGQRMQWQSQNKPMMEGPHDNGLCGDTNQLEEQKSGFGISDCWRYVLDGQMHLPDVQEGAGIGTCIRDALLYGCAEFGTVPDKMEMDCDKTVDANTAKCQNILKDVLRSENFALLCSVLCRTVHQDGERTRYFDFGVIDSRMKNGNYGPEPELFVHDLKLLWEDLKVAGQDIVHLANNLSSLTEDSYEKLVGRERGSDDDELNEAVVARSEPKNLVQPNASVPLTSQGFNQLLDQPGPSDPSVVYKDSICNRCGKVAGAGSVLKCYRCMLPCHISCIEATGSPISTGRWCCKNCSAGTKEPVEGDMVLAHGNPNCLHESCVVCDRLAACRSPKCDNSRALVISSVDPEIDTCYSCKICGGTEEDEKRFLICGNVLCRYMYYHIGCLKSMQISTSVERGSPCWYCPSCLCRVCLCDKDDDLTILCDGCDEAYHIYCITPRHTSIPKGQWYCSSCSVERAEEGMRQYERRTLKLHRKEDAGLQSWNFDGVDLLLSAAEQLRIDEQLETRTD	zf-BED--PHD--	VI
Zm00001d011158_P008	ZmZf-BED13_VI	MIDDEESQGIKKVHFRDSFSQHPIVTYKRRRQQKPQTQQQLRQPPPSLQQHPEPQLQVKLEPKTEDVPEQQNNDTFWKSRDMGWKYGIMIDENRQHWKCMYCGLIRYGGGVSRLKRHLAGDLDVKMCPEVPADVVEEIREHLRKKRERRRKRAAQNGGDNVKAKNPSGDANVEKDLLPSNSVLPDGMDTNVLEEVTNQTGSVHHETTPRFPILRARDIGWEHAVDLDGNKRRWQCKFCSLCRSGGVTTLKAHLIDDSCPNVPKEISKKVSNFIEEKRATRLLLNNYVFNVDEDFNTILQEGTVEYVNEQQPSRKATYVQTLSKCAINEIAAGSKQCGAECSGQPVEHCDQPEEQCTMDYGRMDRLTSNKNQILDKNTENSKNTKVHNYPAVLRDSAWEHSLIYEKENGHWKCKWCSIEGDHGLTRLMWHLVGWQNRPQCPNIPKDVAEKMKDQMMSKKEQKARSGLFDGNGCCEVLCSSNSSQLDQNHLTARIHDRCSSQAFDHANSELKGCNMLSNTILSQQSSNPQVHHEDPQVCHEQERKEVATSSEPGCEQGQRMQWQSQNKPMMEGPHDNGLCGDTNQLEEQKSGFGISDCWRYVLDGQMHLPDVQEGAGIGTCIRDALLYGCAEFGTVPDKMEMDCDKTVDANTAKCQNILKDVLRSENFALLCSVLCRTVHQDGERTRYFDFGVIDSRMKNGNYGPEPELFVHDLKLLWEDLKVAGQDIVHLANNLSSLTEDSYEKLVGRERGSDDDELNEAVVARSEPKNLVQPNASVPLTSQGFNQLLDQPGPSDPSVVYKDSICNRCGKVAGAGSVLKCYRCMLPCHISCIEATGSPISTGRWCCKNCSAGTKEPVEGDMVLAHGNPNCLHESCVVCDRLAACRSPKCDNSRALVISSVDPEIDTCYSCKICGGTEEDEKRFLICGNVLCRYMYYHIGCLKSMQISTSVERGSPCWYCPSCLCRVCLCDKDDDLTILCDGCDEAYHIYCITPRHTSIPKGQWYCSSCSVERAEEGMRQYERRTLKLHRKEDAGLQSWNFDGVDLLLSAAEQLRIDEQLETRTD	zf-BED--PHD--	VI
Zm00001d011158_P012	ZmZf-BED14_VI	MIDDEESQGIKKVHFRDSFSQHPIVTYKRRRQQKPQTQQQLRQPPPSLQQHPEPQLQVKLEPKTEDVPEQQNNDTFWKSRDMGWKYGIMIDENRQHWKCMYCGLIRYGGGVSRLKRHLAGDLDVKMCPEVPADVVEEIREHLRKKRERRRKRAAQNGGDNVKAKNPSGDANVEKDLLPSNSVLPDGMDTNVLEEVTNQTGSVHHETTPRFPILRARDIGWEHAVDLDGNKRRWQCKFCSLCRSGGVTTLKAHLIDDSCPNVPKEISKKVSNFIEEKRATRLLLNNYVFNVDEDFNTILQEGTVEYVNEQQPSRKATYVQTLSKCAINEIAAGSKQCGAECSGQPVEHCDQPEEQCTMDYGRMDRLTSNKNQILDKNTENSKNTKMLKPCRKSEFNTRKHIIIVDKIGRHWKCRYCGMDVYGKKFQLHYHLAGAFRQAKCPNVPREVFAKARQHVLTKKMLKKSKAEQQIPSSPHILAQSGEERQNNDPFCGNQSQLSINNEPREVHNYPAVLRDSAWEHSLIYEKENGHWKCKWCSIEGDHGLTRLMWHLVGWQNRPQCPNIPKDVAEKMKDQMMSKKEQKARSGLFDGNGCCEVLCSSNSSQLDQNHLTARIHDRCSSQAFDHANSELKGCNMLSNTILSQQSSNPQVHHEDPQVCHEQERKEVATSSEPGCEQGQRMQWQSQNKPMMEGPHDNGLCGDTNQLEEQKSGFGISDCWRYVLDGQMHLPDVQEGAGIGTCIRDALLYGCAEFGTVPDKMEMDCDKTVDANTAKCQNILKDVLRSENFALLCSVLCRTVHQDGERTRYFDFGVIDSRMKNGNYGPEPELFVHDLKLLWEDLKVAGQDIVHLANNLSSLTEDSYEKLVGRERGSDDDELNEAVVARSEPKNLVQPNASVPLTSQGFNQLLDQPGPSDPSVVYKDSICNRCGKVAGAGSVLKCYRCMLPCHISCIEATGSPISTGRWCCKNCSAGTKEPVEGDMVLAHGNPNCLHESCVVCDRLAACRSPKCDNSRALVISSVDPEIDTCYSCKICGGTEEDEKRFLICGNVLCRYMYYHIGCLKSMQISTSVERGSPCWYCPSCLCRVCLCDKDDDLTILCDGCDEAYHIYCITPRHTSIPKGQWYCSSCSVERAEEGMRQYERRTLKLHRKEDAGLQSWNFDGVDLLLSAAEQLRIDEQLETRTD	zf-BED--PHD--	VI
Zm00001d011158_P015	ZmZf-BED15_II	MIDDEESQGIKKVHFRDSFSQHPIVTYKRRRQQKPQTQQQLRQPPPSLQQHPEPQLQVKLEPKTEDVPEQQNNDTFWKSRDMGWKYGIMIDENRQHWKCMYCGLIRYGGGVSRLKRHLAGDLDVKMCPEVPADVVEEIREHLRKKRERRRKRAAQNGGDNVKAKNPSGDANVEKDLLPSNSVLPDGMDTNVLEEVTNQTGSVHHETTPRFPILRARDIGWEHAVDLDGNKRRWQCKFCSLCRSGGVTTLKAHLIDDSCPNVPKEISKKVSNFIEEKRATRLLLNNYVFNVDEDFNTILQEGTVEYVNEQQPSRKATYVQTLSKCAINEIAAGSKQCGAECSGQPVEHCDQPEEQCTMDYGRMDRLTSNKNQILDKNTENSKNTKMLKPCRKSEFNTRKHIIIVDKIGRHWKCRYCGMDVYGKKFQLHYHLAGAFRQAKCPNVPREVFAKARQHVLTKKMLKKSKAEQQIPSSPHILAQSGEERQNNDPFCGNQSQLSINNEPREVHNYPAVLRDSAWEHSLIYEKENGHWKCKWCSIEGDHGLTRLMWHLVGWQNRPQCPNIPKDVAEKMKDQMMSKKEQKARSGLFDGNGCCEVLCSSNSSQLDQNHLTARIHDRCSSQAFDHANSELKGCNMLSNTILSQQSSNPQVHHEDPQVCHEQERKEVATSSEPGCEQGQRMQWQSQNKPMMEGPHDNGLCGDTNQLEEQKSGFGISDCWRYVLDGQMHLPDVQEGAGIGTCIRDALLYGCAEFGTVPDKMEMDCDKTVDANTAKCQNILKDVLRSENFALLCSVLCRTVHQDGERTRYFDFGVIDSRMKNGNYGPEPELFVHDLKLEPLHSKSCSYSCSPFECG	zf-BED	II
Zm00001d011158_P016	ZmZf-BED	MIDDEESQGIKKVHFRDSFSQHPIVTYKRRRQQKPQTQQQLRQPPPSLQQHPEPQLQVKLEPKTEDVPEQQNNDTFWKSRDMGWKYGIMIDENRQHWKCMYCGLIRYGGGVSRLKRHLAGDLDVKMCPEVPADVVEEIREHLRKKRERRRKRAAQNGGDNVKAKNPSGDANVEKDLLPSNSVLPDGMDTNVLEEVTNQTGSVHHETTPRFPILRARDIGWEHAVDLDGNKRRWQCKFCSLCRSGGVTTLKAHLIDDSCPNVPKEISKKVSNFIEEKRATRLLLNNYVFNVDEDFNTILQEGTVEYVNEQQPSRKATYVQTLSKCAINEIAAGSKQCGAECSGQPVEHCDQPEEQCTMDYGRMDRLTSNKNQILDKNTENSKNTKMLKPCRKSEF	zf-BED	II

	16_I<br>I	NTRKHIIIVDKIGRHWKCRYCGMDVYGKKFQLHYHLAGAFRQAKCPNVPREVFAKARQHVLTKKMLKKSKAEQQIPSSPH<br>ILAQSGEERQNNDPFCGNQSQLSINNEPREVHNYPAVLRDSAWEHSLIYEKENGHWKCKWCSIEGDHGLTRLMWHLVG<br>WQNRPQCPNIPKDVAEKMKDQMMSKKEQKARSGLFDGNGCCEVLCSSNSSQLDQNHLTARIHDRCSSQAFDHANSEL<br>KGCNMLSNTILSQQSSNPQVHHEDPQVCHEQERKEVATSSEPGCEQGQRMQWQSQNKPMMEGPHDNGLCGDTNQL<br>EEQKSGFGISDCWRYLTKWKWIVIKQWMRTLLSARIFLRMF		
Zm000<br>01d01<br>3336_<br>P001	ZmZ<br>f-<br>BED<br>17_I	MAEETANDAQVAQDNEIALSNEAIQGDELVHVEELSQGDDLVQGNELVVSEVTTPTIGTRRRRKKSLVWEHFTIEAVAGG<br>ATRACCKLCKQTFAYSSGSKIAGTSHLKRHITLGSCPKIKNQEQRLALPSTGGTDNDGEGTIERRTKRRYRYTGYANAAF<br>DQDRSCSYLAKLIIQHDYPLHIVQQPSFAIFIESLQPRFKIVDVETMEGEVYAVYQKEKENLLQAFNSMPGRISLTIGLWTTS<br>QTLGYVSLAGQFIDSEWKVHRRMLNFMMVSSPHSENALSEAISMSLSDWNMKDRLFTITLDNECSSHDIYSANLRDHLSN<br>KNNLMLKGQLFVVRCYAHILNAVALDVIASIHGVVYSIRESIKFIKASSAREEKFAEIALQLEIPSTKTLCLDVTTQWNTTYLM<br>LLAALDYKQTFTTLETCDDNYNEAPSAEDWKKVEAACNYLKLLYDSAHSIMAAANPTANIFFHEAWKLQLELANGTGHED<br>PTFSSIAKDMHERFDKYWKDCSLVLAIAVVMDPRFKMKLVEFSYSKIYGAGAAKYVKVVDDAVHELYKEYVAQPLPLTPA<br>YVDQGEGNNGPANANSSQGAPASTGDGLLDFDMYLSEIQSSQPSKSELEQYLDESLTPRIQEFDILNWWKLNTVKFPTL<br>SKMARDILAIPVSMVSSGSSIFCAGTGSHMLDDYRSSLRPEIVEALVCAKDWLQYSPRRLKDRRFVVVTACLVEAKQESIY<br>PSVVSLSVVNCRCIVG	zf-BED--DUF-<br>domain--<br>Dimer_Tnp_hAT<br>--	I
Zm000<br>01d01<br>3336_<br>P002	ZmZ<br>f-<br>BED<br>18_<br>XXII	MAEETANDAQVAQDNEIALSNEAIQGDELVHVEELSQGDDLVQGNELVVSEVTTPTIGTRRRRKKSLVWEHFTIEAVAGG<br>ATRACCKLCKQTFAYSSGSKIAGTSHLKRHITLGSCPKIKNQEQRLALPSTGGTDNDGEGTIERRTKRRYRYTGYANAAF<br>DQDRSCSYLAKLIIQHDYPLHIVQQPSFAIFIESLQPRFKIVDVETMEGEVYAVYQKEKENLLQAFNSMPGRISLTIGLWTTS<br>QTLGYVSLAGQFIDSEWKVHRRMLNFMMVSSPHSENALSEAISMSLSDWNMKDRLFTITLDNECSSHDIYSANLRDHLSN<br>KNNLMLKGQLFVVRCYAHILNAVALDVIASIHGVVYSIRESIKFIKASSAREEKFAEIALQLEIPSTKTLCLDVTTQWNTTYLM<br>LLAALDYKQTFTTLETCDDNYNEAPSAEDWKKVEAACNYLKLLYDSAHSIMAAANPTANIFFHEAWKLQLELANGTGHED<br>PTFSSIAKDMHERFDKYWKDCSLVLAIAVVMDPRFKMKLVEFSYSKIYGAGAAKYVKVVDDAVHELYKEYVAQPLPLTPA<br>YVDQGEGNNGPANANSSQGAPASTGDGLLDFDMYSWIPLASIFSMDEPNNNFNATVHANEMFDSNGVIHEDEMAHDDE<br>MIHGHAMFLGDEMIRGTEMVEGSEMIHGHDMVQVNDLIHGNEMVPVHDMVNGDKIAHGNELVNSQMTPRISRRRKKKS<br>LVWEHFTIELMPGGSSRACCKLCKQTFAYSSGSKIAGTSHLKRHITLGSCPVMKDQDRKLALPLAGGHVTDNDGEGTAE<br>RPTKRRYRYTGYANATFDPQRSTSYLTKMIILHDYPLHIAQQSSFINFIESLQPRFRVVDVETMEGEVYAVFQKAKENLLQA<br>FSTMPGRISLTIGLWTTSQTLGYVSLAGQFIDSDWKVHRRMLNFMMVSSPHSENALSEAISSSLSEWNMKDKLFTITLDN<br>DCSSHDIYSANLRDHLSNKNNVMLKGQLFVVRCYAHILNVVAQDVIASIHGVIYSIRESIKFIKASPSREEKFAEIALQLEIPS<br>TKTLCLDVTTQWNTTYLMLLAALDYKQAFATLETCDDNYNESPSAEDWKKVEASCNYLKLLYDSAHSVMASANPTANIFF<br>HEAWKIQLELANGTEHGSPFFSCIAKDMHERFDKYWKDCSLVLAIAVVMDPRFKMKLVEFSYSKIYGAEAAKYVKVVNDS<br>LHELYKEYVAQTLPLAPAYVEANNVAANTNVNQGNPPPTSDGLLDFDMYLSEIQSSQPAKCELEQYLEESLTPRIQEFDIL<br>NWWKLNTLKFPTLSKMARDILAIPMSMVSSGSSIFSAGTGNRMLDDYRSSLRPETVEALVCAKDWLQYSPAATEAPGSE<br>MMKAEAL	zf-BED--DUF-<br>domain--zf-<br>BED--DUF-<br>domain--<br>Dimer_Tnp_hAT<br>--	X<br>X<br>II
Zm000<br>01d01<br>3336_<br>P003	ZmZ<br>f-<br>BED<br>19_I	MDEPNNNFNATVHANEMFDSNGVIHEDEMAHDDEMIHGHAMFLGDEMIRGTEMVEGSEMIHGHDMVQVNDLIHGNEMV<br>PVHDMVNGDKIAHGNELVNSQMTPRISRRRKKKSLVWEHFTIELMPGGSSRACCKLCKQTFAYSSGSKIAGTSHLKRHIT<br>LGSCPVMKDQDRKLALPLAGGHVTDNDGEGTAERPTKRRYRYTGYANATFDPQRSTSYLTKMIILHDYPLHIAQQSSFIN<br>FIESLQPRFRVVDVETMEGEVYAVFQKAKENLLQAFSTMPGRISLTIGLWTTSQTLGYVSLAGQFIDSDWKVHRRMLNFM<br>MVSSPHSENALSEAISSSLSEWNMKDKLFTITLDNDCSSHDIYSANLRDHLSNKNNVMLKGQLFVVRCYAHILNVVAQDVI<br>ASIHGVIYSIRESIKFIKASPSREEKFAEIALQLEIPSTKTLCLDVTTQWNTTYLMLLAALDYKQAFATLETCDDNYNESPSAE<br>DWKKVEASCNYLKLLYDSAHSVMASANPTANIFFHEAWKIQLELANGTEHGSPFFSCIAKDMHERFDKYWKDCSLVLAIA<br>VVMDPRFKMKLVEFSYSKIYGAEAAKYVKVVNDSLHELYKEYVAQTLPLAPAYVEANNVAANTNVNQGNPPPTSDGLLDF<br>DMYLSEIQSSQPAKCELEQYLEESLTPRIQEFDILNWWKLNTLKFPTLSKMARDILAIPMSMVSSGSSIFSAGTGNRMLDD<br>YRSSLRPETVEALVCAKDWLQYSPAATEAPGSEMMKAEAL	zf-BED--DUF-<br>domain--<br>Dimer_Tnp_hAT<br>--	I
Zm000<br>01d01<br>3336_<br>P004	ZmZ<br>f-<br>BED<br>20_I	MDEPNNNFNATVHANEMFDSNGVIHEDEMAHDDEMIHGHAMFLGDEMIRGTEMVEGSEMIHGHDMVQVNDLIHGNEMV<br>PVHDMVNGDKIAHGNELVNSQMTPRISRRRKKKSLVWEHFTIELMPGGSSRACCKLCKQTFAYSSGSKIAGTSHLKRHIT<br>LGSCPVMKDQDRKLALPLAGGHVTDNDGEGTAERPTKRRYRYTGYANATFDPQRSTSYLTKMIILHDYPLHIAQQSSFIN<br>FIESLQPRFRVVDVETMEGEVYAVFQKAKENLLQAFSTMPGRISLTIGLWTTSQTLGYVSLAGQFIDSDWKVHRRMLNFM<br>MVSSPHSENALSEAISSSLSEWNMKDKLFTITLDNDCSSHDIYSANLRDHLSNKNNVMLKGQLFVVRCYAHILNVVAQDVI<br>ASIHGVIYSIRESIKFIKASPSREEKFAEIALQLEIPSTKTLCLDVTTQWNTTYLMLLAALDYKQAFATLETCDDNYNESPSAE<br>DWKKVEASCNYLKLLYDSAHSVMASANPTANIFFHEAWKIQLELANGTEHGSPFFSCIAKDMHERFDKYWKDCSLVLAIA<br>VVMDPRFKMKLVEFSYSKIYGAEAAKYVKVVNDSLHELYKEYVAQTLPLAPAYVEANNVAANTNVNQGNPPPTSDGLLDF<br>DMYLSEIQSSQPAKCELEQYLEESLTPRIQEFDILNWWKLNTLKFPTLSKMARDILAIPMSMVSSGSSIFSAGTGNRMLDD<br>YRSSLRPETVEALVCAKDWLQYSPAATEAPGSEMMKAEAL	zf-BED--DUF-<br>domain--<br>Dimer_Tnp_hAT<br>--	I
Zm000<br>01d01<br>3336_<br>P005	ZmZ<br>f-<br>BED<br>21_I	MDEPNNNFNATVHANEMFDSNGVIHEDEMAHDDEMIHGHAMFLGDEMIRGTEMVEGSEMIHGHDMVQVNDLIHGNEMV<br>PVHDMVNGDKIAHGNELVNSQMTPRISRRRKKKSLVWEHFTIELMPGGSSRACCKLCKQTFAYSSGSKIAGTSHLKRHIT<br>LGSCPVMKDQDRKLALPLAGGHVTDNDGEGTAERPTKRRYRYTGYANATFDPQRSTSYLTKMIILHDYPLHIAQQSSFIN<br>FIESLQPRFRVVDVETMEGEVYAVFQKAKENLLQAFSTMPGRISLTIGLWTTSQTLGYVSLAGQFIDSDWKVHRRMLNFM<br>MVSSPHSENALSEAISSSLSEWNMKDKLFTITLDNDCSSHDIYSANLRDHLSNKNNVMLKGQLFVVRCYAHILNVVAQDVI<br>ASIHGVIYSIRESIKFIKASPSREEKFAEIALQLEIPSTKTLCLDVTTQWNTTYLMLLAALDYKQAFATLETCDDNYNESPSAE<br>DWKKVEASCNYLKLLYDSAHSVMASANPTANIFFHEAWKIQLELANGTEHGSPFFSCIAKDMHERFDKYWKDCSLVLAIA<br>VVMDPRFKMKLVEFSYSKIYGAEAAKYVKVVNDSLHELYKEYVAQTLPLAPAYVEANNVAANTNVNQGNPPPTSDGLLDF<br>DMYLSEIQSSQPAKCELEQYLEESLTPRIQEFDILNWWKLNTLKFPTLSKMARDILAIPMSMVSSGSSIFSAGTGNRMLDD<br>YRSSLRPETVEALVCAKDWLQYSPAATEAPGSEMMKAEAL	zf-BED--DUF-<br>domain--<br>Dimer_Tnp_hAT<br>--	I
Zm000<br>01d01<br>3336_<br>P006	ZmZ<br>f-<br>BED<br>22_I	MDEPNNNFNATVHANEMFDSNGVIHEDEMAHDDEMIHGHAMFLGDEMIRGTEMVEGSEMIHGHDMVQVNDLIHGNEMV<br>PVHDMVNGDKIAHGNELVNSQMTPRISRRRKKKSLVWEHFTIELMPGGSSRACCKLCKQTFAYSSGSKIAGTSHLKRHIT<br>LGSCPVMKDQDRKLALPLAGGHVTDNDGEGTAERPTKRRYRYTGYANATFDPQRSTSYLTKMIILHDYPLHIAQQSSFIN<br>FIESLQPRFRVVDVETMEGEVYAVFQKAKENLLQAFSTMPGRISLTIGLWTTSQTLGYVSLAGQFIDSDWKVHRRMLNFM<br>MVSSPHSENALSEAISSSLSEWNMKDKLFTITLDNDCSSHDIYSANLRDHLSNKNNVMLKGQLFVVRCYAHILNVVAQDVI<br>ASIHGVIYSIRESIKFIKASPSREEKFAEIALQLEIPSTKTLCLDVTTQWNTTYLMLLAALDYKQAFATLETCDDNYNESPSAE<br>DWKKVEASCNYLKLLYDSAHSVMASANPTANIFFHEAWKIQLELANGTEHGSPFFSCIAKDMHERFDKYWKDCSLVLAIA<br>VVMDPRFKMKLVEFSYSKIYGAEAAKYVKVVNDSLHELYKEYVAQTLPLAPAYVEANNVAANTNVNQGNPPPTSDGLLDF<br>DMYLSEIQSSQPAKCELEQYLEESLTPRIQEFDILNWWKLNTLKFPTLSKMARDILAIPMSMVSSGSSIFSAGTGNRMLDD<br>YRSSLRPETVEALVCAKDWLQYSPAATEAPGSEMMKAEAL	zf-BED--DUF-<br>domain--<br>Dimer_Tnp_hAT<br>--	I
Zm000<br>01d01<br>3336_<br>P008	ZmZ<br>f-<br>BED<br>23_I	MDEPNNNFNATVHANEMFDSNGVIHEDEMAHDDEMIHGHAMFLGDEMIRGTEMVEGSEMIHGHDMVQVNDLIHGNEMV<br>PVHDMVNGDKIAHGNELVNSQMTPRISRRRKKKSLVWEHFTIELMPGGSSRACCKLCKQTFAYSSGSKIAGTSHLKRHIT<br>LGSCPVMKDQDRKLALPLAGGHVTDNDGEGTAERPTKRRYRYTGYANATFDPQRSTSYLTKMIILHDYPLHIAQQSSFIN<br>FIESLQPRFRVVDVETMEGEVYAVFQKAKENLLQAFSTMPGRISLTIGLWTTSQTLGYVSLAGQFIDSDWKVHRRMLNFM<br>MVSSPHSENALSEAISSSLSEWNMKDKLFTITLDNDCSSHDIYSANLRDHLSNKNNVMLKGQLFVVRCYAHILNVVAQDVI<br>ASIHGVIYSIRESIKFIKASPSREEKFAEIALQLEIPSTKTLCLDVTTQWNTTYLMLLAALDYKQAFATLETCDDNYNESPSAE<br>DWKKVEASCNYLKLLYDSAHSVMASANPTANIFFHEAWKIQLELANGTEHGSPFFSCIAKDMHERFDKYWKDCSLVLAIA<br>VVMDPRFKMKLVEFSYSKIYGAEAAKYVKVVNDSLHELYKEYVAQTLPLAPAYVEANNVAANTNVNQGNPPPTSDGLLDF<br>DMYLSEIQSSQPAKCELEQYLEESLTPRIQEFDILNWWKLNTLKFPTLSKMARDILAIPMSMVSSGSSIFSAGTGNRMLDD<br>YRSSLRPETVEALVCAKDWLQYSPAATEAPGSEMMKAEAL	zf-BED--DUF-<br>domain--<br>Dimer_Tnp_hAT<br>--	I
Zm000<br>01d01<br>5283_<br>P002	ZmZ<br>f-<br>BED<br>24_I<br>I	MDLRISEGSGTSTTLDDQSVQSPTRRAKVWEYFEQELVEVDRVMKAVCKYCGTKLTSKRNSGTNSLRNLVADTCPKIPV<br>EDQKRFIATMRKRPGEGSFVFDPRKTRECMVKWCISAEVAFNKFDDPFFAPWMESLQPLFSGVGRQTMRNDCIARFKM<br>MRQELRNELQSLNSRICLTSDLWTSNQKLGYLCLTVHYIDANFILKKKTIAFKDVKYPHTGLAIEEVITKCLIEWGIKEKVFTI<br>TLDNASNNQSACDLIRESGRSDMLFGGEHLHVRCCAHILNLLVQDGMAIAHGAFDKIRDLFIPLCLDPASSNTLSASGNGE<br>IYASFSGLLMLSRCPLPLLLASSLITDSFHCC	zf-BED	II
Zm000<br>01d01<br>6617_<br>P001	ZmZ<br>f-<br>BED	MSASRPSSGRQPLCPPLCPPLAACPSARRPPPAHLCPPPSSGCVPSSGRRPDAHLQPSRARGLQPLRRPPLAAAHLQP<br>SRKPAHLCPDKSQQPVPPSQVTTSSRDMEDASADRPQGESGSQQPYDPAKDPKKKAKSKDPAWNYCYWLDLNKKDVV<br>KCILCGKIVHAGVRRLKQHLVGGHAKRCDYLKNNARQKPIELDDDKEGDKDDEVEFEASNEVRLAVYFSFVQFQTSKECS<br>IEGAGGSLPDEGLCSRRRLESGITVDVVCTEKPHIAPTWIIPWSPSFNQRAEELDRAIVLNVTVAINEAVEEAILANFARRLE<br>LVHDSLSLCRLGPASFLLILPSLEMADRAYDGGRIVNLSPTTKIHVMRWSRFLNSSVTASLPFHVEIEVSGIPAHAWELESV	zf-BED	II

	25_II	QALLNEWCWIADLHPDCERQREVFKVIAWCSSLSSIPREFKLQIVDPVTSDGGMLRRSLVYPIRVSVKVLDRPNLLQSLQP<br>SPPPVEDDPDQGRRRRRRRSRRSSFGINAPGKSSTTAVLPRASVHQRLGPCVQVFVKEGIRMPRLPVHNRLGPLEVFIA<br>EKGAIKKMKRVWIPKRKL		
Zm00001d017846_P001	ZmZf-BED26_I	MDLEVSEGSGTNATSDDQSVQTLPRRAKLLEYFQPELVEVNGVMKAICKYCGAMLRSSSTNSLRNHVADTCPKISVEDR<br>KQFIATMEKGPAEASFVFDSQKARECMVKWCISAEVAFNKFDDPFFAQWMESMQPSFSGVGRQTMQNDCITRFKMMR<br>QELRNELQSLNSRICLTTNLWTSNQKLGYLCLTAHYINADFILKKKIIAFKYLKYPHTGLAIKEGITECLKEWGIKEKMFTITLD<br>NVSNNLSVRDLRESGLFGGDLRIVQCCAHILILLVQDGMAIAHGAIYKIRELVRHINSSPSRIQAFNGIAERSGLPSKDRLILD<br>VPNRWNSTHDMIMEAIEYKVVLKRYAEEQLEPSPNDEEWTNSEAIGEFLEAFEKATKAFSTHRSPTSHLFLHNVLCIHRTL<br>RNTNWQINCVLKDSARAMDAKFDKYWEKGKYNMTLVLATILDPSKKMDFLDFFYEKMSQYFVDIQINVVLVKQCLTKLFE<br>KYATLVQIDNKSSPFIDIRTSNLGSTVLGKRRLDEEFSQWSQIRGRRFPKSELDTYLEEELVRIDERFEILNWWRTNANKYP<br>VLSAMARDILAIPLSTVPSEFAFSAGGRILDDNRSTMTPETLECFVCCKDWLYEYPNIQA	zf-BED--DUF-domain--Dimer_Tnp_hAT--	I
Zm00001d017846_P002	ZmZf-BED27_I	MRSPAAPRRSPISSRSAPALRLVVRRRSSRTPARPSRRLEAKTVPFPVSVVKMDLEVSEGSGTNATSDDQSVQTLPRRA<br>KLLEYFQPELVEVNGVMKAICKYCGAMLRSSSTNSLRNHVADTCPKISVEDRKQFIATMEKGPAEASFVFDSQKARECMV<br>KWCISAEVAFNKFDDPFFAQWMESMQPSFSGVGRQTMQNDCITRFKMMRQELRNELQSLNSRICLTTNLWTSNQKLGY<br>LCLTAHYINADFILKKKIIAFKYLKYPHTGLAIKEGITECLKEWGIKEKMFTITLDNVSNNLSVRDLRESGLFGGDLRIVQCCA<br>HILILLVQDGMAIAHGAIYKIRELVRHINSSPSRIQAFNGIAERSGLPSKDRLILDVPNRWNSTHDMIMEAIEYKVVLKRYAEE<br>QLEPSPNDEEWTNSEAIGEFLEAFEKATKAFSTHRSPTSHLFLHNVLCIHRTLRNTNWQINCVLKDSARAMDAKFDKYWE<br>KGKYNMTLVLATILDPSKKMDFLDFFYEKMSQYFVDIQINVVLVKQCLTKLFEKYATLVQIDNKSSPFIDIRTSNLGSTVLGK<br>RRLDEEFSQWSQIRGRRFPKSELDTYLEEELVRIDERFEILNWWRTNANKYPVLSAMARDILAIPLSTVPSEFAFSAGGRIL<br>DDNRSTMTPETLECFVCCKDWLYEYPNIQA	zf-BED--DUF-domain--Dimer_Tnp_hAT--	I
Zm00001d017846_P005	ZmZf-BED28_I	MRSPAAPRRSPISSRSAPALRLVVRRRSSRTPARPSRRLEAKTVPFPVSVVKMDLEVSEGSGTNATSDDQSVQTLPRRA<br>KLLEYFQPELVEVNGVMKAICKYCGAMLRSSSTNSLRNHVADTCPKISVEDRKQFIATMEKGPAEASFVFDSQKARECMV<br>KWCISAEVAFNKFDDPFFAQWMESMQPSFSGVGRQTMQNDCITRFKMMRQELRNELQSLNSRICLTTNLWTSNQKLGY<br>LCLTAHYINADFILKKKIIAFKYLKYPHTGLAIKEGITECLKEWGIKEKMFTITLDNVSNNLSVRDLRESGLFGGDLRIVQCCA<br>HILILLVQDGMAIAHGAIYKIRELVRHINSSPSRIQAFNGIAERSGLPSKDRLILDVPNRWNSTHDMIMEAIEYKVVLKRYAEE<br>QLEPSPNDEEWTNSEAIGEFLEAFEKATKAFSTHRSPTSHLFLHNVLCIHRTLRNTNWQINCVLKDSARAMDAKFDKYWE<br>KGKYNMTLVLATILDPSKKMDFLDFFYEKMSQYFVDIQINVVLVKQCLTKLFEKYATLVQIDNKSSPFIDIRTSNLGSTVLGK<br>RRLDEEFSQWSQIRGRRFPKSELDTYLEEELVRIDERFEILNWWRTNANKYPVLSAMARDILAIPLSTVPSEFAFSAGGRIL<br>DDNRSTMTPETLECFVCCKDWLYEYPNIQA	zf-BED--DUF-domain--Dimer_Tnp_hAT--	I
Zm00001d017846_P006	ZmZf-BED29_I	MRSPAAPRRSPISSRSAPALRLVVRRRSSRTPARPSRRLEAKTVPFPVSVVKMDLEVSEGSGTNATSDDQSVQTLPRRA<br>KLLEYFQPELVEVNGVMKAICKYCGAMLRSSSTNSLRNHVADTCPKISVEDRKQFIATMEKGPAEASFVFDSQKARECMV<br>KWCISAEVAFNKFDDPFFAQWMESMQPSFSGVGRQTMQNDCITRFKMMRQELRNELQSLNSRICLTTNLWTSNQKLGY<br>LCLTAHYINADFILKKKIIAFKYLKYPHTGLAIKEGITECLKEWGIKEKMFTITLDNVSNNLSVRDLRESGLFGGDLRIVQCCA<br>HILILLVQDGMAIAHGAIYKIRELVRHINSSPSRIQAFNGIAERSGLPSKDRLILDVPNRWNSTHDMIMEAIEYKVVLKRYAEE<br>QLEPSPNDEEWTNSEAIGEFLEAFEKATKAFSTHRSPTSHLFLHNVLCIHRTLRNTNWQINCVLKDSARAMDAKFDKYWE<br>KGKYNMTLVLATILDPSKKMDFLDFFYEKMSQYFVDIQINVVLVKQCLTKLFEKYATLVQIDNKSSPFIDIRTSNLGSTVLGK<br>RRLDEEFSQWSQIRGRRFPKSELDTYLEEELVRIDERFEILNWWRTNANKYPVLSAMARDILAIPLSTVPSEFAFSAGGRIL<br>DDNRSTMTPETLECFVCCKDWLYEYPNIQA	zf-BED--DUF-domain--Dimer_Tnp_hAT--	I
Zm00001d017846_P007	ZmZf-BED30_I	MRSPAAPRRSPISSRSAPALRLVVRRRSSRTPARPSRRLEAKTVPFPVSVVKMDLEVSEGSGTNATSDDQSVQTLPRRA<br>KLLEYFQPELVEVNGVMKAICKYCGAMLRSSSTNSLRNHVADTCPKISVEDRKQFIATMEKGPAEASFVFDSQKARECMV<br>KWCISAEVAFNKFDDPFFAQWMESMQPSFSGVGRQTMQNDCITRFKMMRQELRNELQSLNSRICLTTNLWTSNQKLGY<br>LCLTAHYINADFILKKKIIAFKYLKYPHTGLAIKEGITECLKEWGIKEKMFTITLDNVSNNLSVRDLRESGLFGGDLRIVQCCA<br>HILILLVQDGMAIAHGAIYKIRELVRHINSSPSRIQAFNGIAERSGLPSKDRLILDVPNRWNSTHDMIMEAIEYKVVLKRYAEE<br>QLEPSPNDEEWTNSEAIGEFLEAFEKATKAFSTHRSPTSHLFLHNVLCIHRTLRNTNWQINCVLKDSARAMDAKFDKYWE<br>KGKYNMTLVLATILDPSKKMDFLDFFYEKMSQYFVDIQINVVLVKQCLTKLFEKYATLVQIDNKSSPFIDIRTSNLGSTVLGK<br>RRLDEEFSQWSQIRGRRFPKSELDTYLEEELVRIDERFEILNWWRTNANKYPVLSAMARDILAIPLSTVPSEFAFSAGGRIL<br>DDNRSTMTPETLECFVCCKDWLYEYPNIQGRFMHHSAGSCSCLGMTLPLLLASSLIRYSFY	zf-BED--DUF-domain--Dimer_Tnp_hAT--	I
Zm00001d017846_P008	ZmZf-BED31_I	MRSPAAPRRSPISSRSAPALRLVVRRRSSRTPARPSRRLEAKTVPFPVSVVKMDLEVSEGSGTNATSDDQSVQTLPRRA<br>KLLEYFQPELVEVNGVMKAICKYCGAMLRSSSTNSLRNHVADTCPKISVEDRKQFIATMEKGPAEASFVFDSQKARECMV<br>KWCISAEVAFNKFDDPFFAQWMESMQPSFSGVGRQTMQNDCITRFKMMRQELRNELQSLNSRICLTTNLWTSNQKLGY<br>LCLTAHYINADFILKKKIIAFKYLKYPHTGLAIKEGITECLKEWGIKEKMFTITLDNVSNNLSVRDLRESGLFGGDLRIVQCCA<br>HILILLVQDGMAIAHGAIYKIRELVRHINSSPSRIQAFNGIAERSGLPSKDRLILDVPNRWNSTHDMIMEAIEYKVVLKRYAEE<br>QLEPSPNDEEWTNSEAIGEFLEAFEKATKAFSTHRSPTSHLFLHNVLCIHRTLRNTNWQINCVLKDSARAMDAKFDKYWE<br>KGKYNMTLVLATILDPSKKMDFLDFFYEKMSQYFVDIQINVVLVKQCLTKLFEKYATLVQIDNKSSPFIDIRTSNLGSTVLGK<br>RRLDEEFSQWSQIRGRRFPKSELDTYLEEELVRIDERFEILNWWRTNANKYPVLSAMARDILAIPLSTVPSEFAFSAGGRIL<br>DDNRSTMTPETLECFVCCKDWLYEYPNIQA	zf-BED--DUF-domain--Dimer_Tnp_hAT--	I
Zm00001d017846_P009	ZmZf-BED32_II	MRSPAAPRRSPISSRSAPALRLVVRRRSSRTPARPSRRLEAKTVPFPVSVVKMDLEVSEGSGTNATSDDQSVQTLPRRA<br>KLLEYFQPELVEVNGVMKAICKYCGAMLRSSSTNSLRNHVADTCPKISVEDRKQFIATMEKGPAEASFVFDSQKARECMV<br>KWCISAEVAFNKFDDPFFAQWMESMQPSFSGVGRQTMQNDCITRFKMMRQELRNELQSLNSRICLTTNLWTSNQKLGY<br>LCLTAHYINADFILKKKIIAFKYLKYPHTGLAIKEGITECLKEWGIKEKMFTITLDNVSNNLSVRDLRESGLFGGDLRIVQCCA<br>HILILLVQDGMAIAHGAIYKIRELVRHINSSPSRIQAFNGIAERSGLPSKDRLILDVPNRWNSTHDMIMEAIEYKVVLKRYAEE<br>QLEPSPNDEEWTNSEAIGEFLEAFEKATKAFSTHRSPTSHLFLHNVLYKNERSNFNRTCGSSDWTDRYLQTAEIESMGR<br>GRGRGKKLTTVRSHEDKGSSGEEVVPAKKRRGRPQKRFASKIDQAVDVENFVEKVDGDQEEADDGKLKNSAAAGGNK<br>RGRPLKEGSNIVVEDNNSIVPSSSDESTRTNGFRQIGSRRKNKPRRAAEAGLECK	zf-BED	II
Zm00001d017846_P010	ZmZf-BED33_I	MRSPAAPRRSPISSRSAPALRLVVRRRSSRTPARPSRRLEAKTVPFPVSVVKMDLEVSEGSGTNATSDDQSVQTLPRRA<br>KLLEYFQPELVEVNGVMKAICKYCGAMLRSSSTNSLRNHVADTCPKISVEDRKQFIATMEKGPAEASFVFDSQKARECMV<br>KWCISAEVAFNKFDDPFFAQWMESMQPSFSGVGRQTMQNDCITRFKMMRQELRNELQSLNSRICLTTNLWTSNQKLGY<br>LCLTAHYINADFILKKKIIAFKYLKYPHTGLAIKEGITECLKEWGIKEKMFTITLDNVSNNLSVRDLRESGLFGGDLRIVQCCA<br>HILILLVQDGMAIAHGAIYKIRELVRHINSSPSRIQAFNGIAERSGLPSKDRLILDVPNRWNSTHDMIMEAIEYKVVLKRYAEE<br>QLEPSPNDEEWTNSEAIGEFLEAFEKATKAFSTHRSPTSHLFLHNVLCIHRTLRNTNWQINCVLKDSARAMDAKFDKYWE<br>KGKYNMTLVLATILDPSKKMDFLDFFYEKMSQYFVDIQINVVLVKQCLTKLFEKYATLVQIDNKSSPFIDIRTSNLGSTVLGK<br>RRLDEEFSQWSQIRGRRFPKSELDTYLEEELVRIDERFEILNWWRTNANKYPVLSAMARDILAIPLSTVPSEFAFSAGGRIL<br>DDNRSTMTPETLECFVCS	zf-BED--DUF-domain--Dimer_Tnp_hAT--	I
Zm00001d022534_P001	ZmZf-BED34_II	MAVETASDAQVAQDNEISPSNEAIQGDELAHGEELFQGDDLVQGNELVVSEVTTPPTTGIRRRRKKKSLVWEHFTIEAVA<br>GGATRACCNLCKQTFAYSSGSKIAGTSHLKRHITLGSCPKIKDQDQRLALPSTGGTDNYGEATSERPTKRRYRYTGYANA<br>VFDQDRSCSYLARMIIQHDYPLHIVQQQAFSVFIESLQPRFKIVDVDTMEGEVYAVYQKEKENLLRSFNTMPGRISLTIGLW<br>TTSQTLGYVSLAGQFIDSEWKVRRRMLNFMMVSSPHSENALSEAISLSLSDWSMKDKLFTITLDNECSSHDIYSANLRDH<br>LSNKNSLMLKGQLFVVRCYAHILNAVAQDVIASVHGVVYNIRESIKFIKASSAREEKCVDSDVLVVVM	zf-BED	II
Zm00001d023717_P001	ZmZf-BED35_II	MGDPNSNDSSMVHGTATETVFEGYEIFLGGNEMVTGCEAIISDKMGHAAETVLDYKMVDIHDNKMVHRNEIVPSGSKIDR<br>NGQTVLRRCSQIVRGNEIGNDNVTTEVKPPTSSKRKRKTSMVWEHFTTEDSEGCTRACCNHCKRIFAYSSGLKMSGTSH<br>LKRHITQGHCPEIEVQKPTAGGRENDCQGTVQKPSMRCRSTCTGYANAPFNPDRCQSYLAKMIILHGYPLQIVQQAAFIS<br>FVESLQPSFKVINKDAVEAEVYAVYLKERKSLLKQVENIPGRINLTVQTWTTSQTLGYVSLAGQFIDSEWKLHRRMLNFMM<br>VPWSCSEDDAVTEAISRSLHQWNMSDKLFTITRDYESSSHDIYSLNLREELSKNNITMLGGQFSVVRCYAHMLTAVAIDVT<br>ALVQSVIYKIRESIKFIKCRIGHEEMFADIILQLQIPSNQILCLDIKTQWNTTYLMLQAALDYKEAFTMLEKCDGNYSQAPSAV<br>DWEKVEVACR	zf-BED	II
Zm00001d025207_P001	ZmZf-BED36_II	MPRYSLLSRHIEATVVPRYNTEERGRKSSVRSGARSSSRVRAAAREWGSCWAVAGKKEPERQMAVAGKKEPENEPTG<br>GEAAGALAGRGGDKELVDCWSWKSAMERVSSALEMDEAASTDQSSRLTKRRAKVWDYVDTEVVDGKEKAVCKYCKV<br>HLSSAVGKGTTHLNRHIFVYCHAIPQEERQMFLATQKTKPGDDHVFDPVVFHGLIAKYFLSAEISFRKCEDPSWKEMIIYY<br>QPSFRLVGRQTVRSDCVLLYEEEKLQLIEQFTKLKSHVSLTADL	zf-BED	II

Zm00001d025324_P001	ZmZf-BED37_II	MGGDCSPSSNLPRGISWMRRPRFPEHRLRLGRRLQRVHGQDSVTTSEFEASICSVYGTKSTTSSLLQQDGNSGEPSSVNSASVTQKTSNAIKSDDPTWEHCFVPDMSKKHAIQCKYCDKVIHGGITRVKYHLANIGGFNVTKCKKVPAPVNQEMEALLTKKTSEKEQRKKEKQRERDEIDLDKSDGSNEEDNEHENDIVVLKSTRNDRMRDLVFVKFNSKLREKRNNKSKDPIEKELNDILEDGGNEFITGVVPDENVDQDEDHEGAHDEPSQEPSISKAQLPAKRKRHGHPRKKKLRSLKSLLSGDLEPATCASSSESEDNESMQIEASNSDSGDE	zf-BED	II
Zm00001d026358_P001	ZmZf-BED38_III	MEHHIIQQVGEHEGEEEEDGMGTEAAFEGVHNHDTFRYKHKKRSKVWEEYKPIFLNGKVQFAECLYCHSRMSCKDSNGTSHLWRHQKICPGKRDVVFRRLKDSYFPCVLVNQSEPVTPGDSVNQIISETLDDISSVIPNRFKSKVWREFSPIYVEGKLQAADCVHCRKRLSANKFGGRSHLSRHLQTCQARRGYNNQKGTLYPSSVPDLKSIGQDELSPALANGKKILSLTSVGEVRNALSSSKLKEILVEKKCLPIRGKLYDVACVDDVLNTIASKVQQNIIHLVGDMVTEFFVAHTSSSLNQQQLMEVISQMSLKCPQEDAKWWYKFYFRLEVFLHFNKPFPFEEAASPEHVIVAESICKILRTFYRVIEVISASSSPTANMYFNEIWKVRTVLQEEASSEHAEITAMVAEMQETFNEYWQHSYLWLCIPVILDPRFKFGFIEFRLKRAFGLKSASYLSGIRETLQELFNEYCSPVDQPNVRVPKSESFSLDDNDSFEDWDQHLNEQASSQKSTELDNYLADGLVPRKDDFDNLNWWMCHATKYPTLAAIVQDILAMPASAVPSEAAFTSSGPVIPKHHSMLSIKTIEALVCTRDWMR	zf-BED--zf-BED--DUF-domain--Dimer_Tnp_hAT--	III
Zm00001d026358_P002	ZmZf-BED39_III	MEHHIIQQVGEHEGEEEEDGMGTEAAFEGVHNHDTFRYKHKKRSKVWEEYKPIFLNGKVQFAECLYCHSRMSCKDSNGTSHLWRHQKICPGKRDVVFRRLKDSYFPCVLVNQSEPVTPGDSVNQIISETLDDISSVIPNRFKSKVWREFSPIYVEGKLQAADCVHCRKRLSANKFGGRSHLSRHLQTCQARRGYNNQKGTLYPSSVPDLKSIGQDELSPALANGKKILSLTSVGEVRNALSSSKLKEILVEKKCLPIRGKLYDVACVDDVLNTIASKVQQNIIHLVGDMVTEFFVAHTSSSLNQQQLMEVISQMSLKCPQEDAKWWYKFYFRLEVFLHFNKPFPFEEAASPEHVIVAESICKILRTFYRVIEVISASSSPTANMYFNEIWKVRTVLQEEASSEHAEITAMVAEMQETFNEYWQHSYLWLCIPVILDPRFKFGFIEFRLKRAFGLKSASYLSGIRETLQELFNEYCSPVDQPNVRVPKSESFSLDDNDSFEDWDQHLNEQASSQKSTELDNYLADGLVPRKDDFDNLNWWMCHATKYPTLAAIVQDILAMPASAVPSEAAFTSSGPVIPKHHSMLSIKTIEALVCTRDWMR	zf-BED--zf-BED--DUF-domain--Dimer_Tnp_hAT--	III
Zm00001d026358_P003	ZmZf-BED40_II	MEHHIIQQVGEHEGEEEEDGMGTEAAFEGVHNHDTFRYKHKKRSKVWEEYKPIFLNGKVQFAECLYCHSRMSCKDSNGTSHLWRHQKICPGKRDVVFRRLKDSYFPCGMLRFCIFRSRALVPR	zf-BED	II
Zm00001d028972_P001	ZmZf-BED41_II	MDLGVCEGSSTNITSDYHSVQMSTRTTKVWEYFQQELVEVDGVMKAICKYCGTKLTTRKRNSCTNSLRNHVADTCPKILVEDRKRFIATMRKKPGEGSFVFDPRKTHECMVKWCISAEVAFNKFDDPFFAPWMESLQPSFSAFNEIAERSGFPSKAGLILDVPNCCNSTHNMIMEAIEYKVVLKRYAEEQLEPSPDDEEWTNSEAIGEFLGGI	zf-BED	II
Zm00001d029192_P001	ZmZf-BED42_II	MLPVDEEVLMLRPASALSAAADSKSLAEKAIAALPPELSAQATDPKRKARSQDPGWKFEWWPDTTKKDFVQCIFCSKIVPSGIKRFKQHLAGGFGDTMKCARVPELVSKEMHMYLKRNMRLVITANTEEGEEGEERNDEGAASSSRPQQTYVRTRKRSRNAAAQDIGEDDSDAQED	zf-BED	II
Zm00001d033361_P001	ZmZf-BED43_V	MTLGWAAKRSAPRAHQRDNPSSVASASITQSTAATIKSDDPAWKHCYCPDLKKKHSLKCNYCDKLINAGITRVKYHLANIAGFNVSKCKKVPTPVKEDMVALLTKNCDAKEKKRKEKQRERDEIDLDNSGGDNSSEEESEHGNDVIVFKSTKGGSSSRLATTGGTIDKFYKPESIEESVQKNKRGLSTSQKIQTQLTTQKREERRDRACEYICQFFYEAGIAHNTVTLPSFAHMVEAIGAFGRGLRGPSSYEMSGPFLKKCKEKLSTVLEDLAKIGAVEETISSAKHVTAFLYAHTRVLDLMRKFLGKDLVHSGVTRFATAYLNLKSLQDNKKDISRLFRSDELNELGYLKKAKGKKANKVVRSEGFWKNVDMVVNFFEPLANVLRRMDSDVPAMGFLHGCMLEAKKEIAMRFDNNENSFKVAWDITDKRWDNKLKTPLHLAGYYLNPYFYYPNKSEIELDGSFRAVVIACITKTVEDEETQDNIIEELNVYQEQQGTFGHDIAVRQRRNKNFNPAKWWLNHGTSTPNLRLLATRILNLTCSSSGCERNWSDFEQVHTKKRNKLLHDRMRDLVFIKFNSRLRQKRENKSKDPLEREMNDVLEDDANEFITGLVPNANSDKDEEHGGAQVGATIHEPQLSQPQPKRKRLVRPRKKKIRSLHSLLYGGLENEAVASSSESEDNGDGDISMHQYSDSDDLGDE	zf-BED--Dimer_Tnp_hAT--	V
Zm00001d033903_P001	ZmZf-BED44_II	MGEPNSNDNAMVHDNEMIDGNGVIHGSEMIHGSVMIHGDEMPHGNEMIHGNEMIHGTEMVEGSEMIHGHEMVQVNDLIHGNEMVAVNVMVNGDEMPHVNEFINTEVTPRRRKKKSLVWEHFTIEPMPGGNSRACCNLCKQTFAYSSGSKIAGTSHLKRHITLGSCPVMKDQDRRLTLPLAGGHATDNDGEGTVERPTKRRYRYTGYANATFDQERSSSYLAKMIILHDYPLHIVQQSSFTKFIESLQPRFRVVDVETMEGEVYAVFQKAKENLLQAFSTMPGRISLTIGLWTTSQTLGYVSLAGQFIDSDWKVHRRMLNFMMVSSPHSENALSEAISSSLLEWNMKDRLFTITLDNDCSSHDIYSANLRDHLSNKNNLMLKGQLFVVRCYAHILNVVAQDVIASIHGVIYNIRESIKFIKASPNRGLLFSMGLLGYIFSKSTISLVLGITPGLATLLLGTQSEVLEERQIQLPAYLGSGR	zf-BED	II
Zm00001d039328_P001	ZmZf-BED45_I	MRERDACWEYCDKLDGNKVRCRFCHKVLNGGISRLKFHLSQIPSKGVNPCTKVKEDVVDRVKAVISAKEEYKEFQLLKRQRVADLSSAAAPAKWAPEAPPSLSTSPGRVASPAAVITRAAEQSRLLAPEASAPVPRPSAAAANNKPRAAAASEWETERCIAEFFFENKLDYSIADSVSYQQMLEALGGPGFRGPPADVLRDKWLQRLKSEILQKTKEIEKDWATTGCTILADSWTDNKLKALINFSVASPMGTFFLKTVDASSHFKTHRGLYDLFDEVIQEVGPENVVQIIADRNINYGSTDKLVAQNYGGAIFWSPCASFCVNAMLDDFSKIDWVNQCICQAQTVTRFIYNSRWVLDLARKCVAGQELVCSGITKSVSDFLTLKSLLRHRPKLKQMFHSAEFSSSSYASRSIPCVEILDDDELWRAVEEIAAVSEPLLRVMRDVSGGRQAIGYIYESMTKVTDSIRTYYIMDEGKCKSFLDIVEQRWQTELHSPLHSAAAFLSPGIQYNPEVKFFRTIKEEFYQVLDKVLTTPDQRHDITAQLHAFRKAQGLFGSNIAKEARNNTPPGMWWEQYGDSAPSLQRAAVRITSQVCSTLTFQRDWGVILQNHYEKRNKLDKEALADQAYVHYNLTLHSEPKARRRPDADPIALDAVDMTSAWVEDSDGPILTQWLDRFPSALDGGDLNTRQFGGSIFGTNDNLFGL	zf-BED--DUF-domain--Dimer_Tnp_hAT--	I
Zm00001d039328_P002	ZmZf-BED46_I	MRERDACWEYCDKLDGNKVRCRFCHKVLNGGISRLKFHLSQIPSKGVNPCTKVKEDVVDRVKAVISAKEEYKEFQLLKRQRVADLSSAAAPAKWAPEAPPSLSTSPGRVASPAAVITRAAEQSRLLAPEASAPVPRPSAAAANNKPRAAAASEWETERCIAEFFFENKLDYSIADSVSYQQMLEALGGPGFRGPPADVLRDKWLQRLKSEILQKTKEIEKDWATTGCTILADSWTDNKLKALINFSVASPMGTFFLKTVDASSHFKTHRGLYDLFDEVIQEVGPENVVQIIADRNINYGSTDKLVAQNYGGAIFWSPCASFCVNAMLDDFSKIDWVNQCICQAQTVTRFIYNSRWVLDLARKCVAGQELVCSGITKSVSDFLTLKSLLRHRPKLKQMFHSAEFSSSSYASRSIPCVEILDDDELWRAVEEIAAVSEPLLRVMRDVSGGRQAIGYIYESMTKVTDSIRTYYIMDEGKCKSFLDIVEQRWQTELHSPLHSAAAFLSPGIQYNPEVKFFRTIKEEFYQVLDKVLTTPDQRHDITAQLHAFRKAQGLFGSNIAKEARNNTPPGMWWEQYGDSAPSLQRAAVRITSQVCSTLTFQRDWGVILQNHYEKRNKLDKEALADQAYVHYNLTLHSEPKARRRPDADPIALDAVDMTSAWVEDSDGPILTQWLDRFPSALDGGDLNTRQFGGSIFGTNDNLFGL	zf-BED--DUF-domain--Dimer_Tnp_hAT--	I
Zm00001d039328_P003	ZmZf-BED47_I	MRERDACWEYCDKLDGNKVRCRFCHKVLNGGISRLKFHLSQIPSKGVNPCTKVKEDVVDRVKAVISAKEEYKEFQLLKRQRVADLSSAAAPAKWAPEAPPSLSTSPGRVASPAAVITRAAEQSRLLAPEASAPVPRPSAAAANNKPRAAAASEWETERCIAEFFFENKLDYSIADSVSYQQMLEALGGPGFRGPPADVLRDKWLQRLKSEILQKTKEIEKDWATTGCTILADSWTDNKLKALINFSVASPMGTFFLKTVDASSHFKTHRGLYDLFDEVIQEVGPENVVQIIADRNINYGSTDKLVAQNYGGAIFWSPCASFCVNAMLDDFSKIDWVNQCICQAQTVTRFIYNSRWVLDLARKCVAGQELVCSGITKSVSDFLTLKSLLRHRPKLKQMFHSAEFSSSSYASRSIPCVEILDDDELWRAVEEIAAVSEPLLRVMRDVSGGRQAIGYIYESMTKVTDSIRTYYIMDEGKCKSFLDIVEQRWQTELHSPLHSAAAFLSPGIQYNPEVKFFRTIKEEFYQVLDKVLTTPDQRHDITAQLHAFRKAQGLFGSNIAKEARNNTPPGMWWEQYGDSAPSLQRAAVRITSQVCSTLTFQRDWGVILQNHYEKRNKLDKEALADQAYVHYNLTLHSEPKARRRPDADPIALDAVDMTSAWVEDSDGPILTQWLDRFPSALDGGDLNTRQFGGSIFGTNDNLFGL	zf-BED--DUF-domain--Dimer_Tnp_hAT--	I
Zm00001d039328_P004	ZmZf-BED48_I	MRERDACWEYCDKLDGNKVRCRFCHKVLNGGISRLKFHLSQIPSKGVNPCTKVKEDVVDRVKAVISAKEEYKEFQLLKRQRVADLSSAAAPAKWAPEAPPSLSTSPGRVASPAAVITRAAEQSRLLAPEASAPVPRPSAAAANNKPRAAAASEWETERCIAEFFFENKLDYSIADSVSYQQMLEALGGPGFRGPPADVLRDKWLQRLKSEILQKTKEIEKDWATTGCTILADSWTDNKLKALINFSVASPMGTFFLKTVDASSHFKTHRGLYDLFDEVIQEVGPENVVQIIADRNINYGSTDKLVAQNYGGAIFWSPCASFCVNAMLDDFSKIDWVNQCICQAQTVTRFIYNSRWVLDLARKCVAGQELVCSGITKSVSDFLTLKSLLRHRPKLKQMFHSAEFSSSSYASRSIPCVEILDDDELWRAVEEIAAVSEPLLRVMRDVSGGRQAIGYIYESMTKVTDSIRTYYIMDEGKCKSFLDIVEQRWQTELHSPLHSAAAFLSPGIQYNPEVKFFRTIKEEFYQVLDKVLTTPDQRHDITAQLHAFRKAQGLFGSNIAKEARNNTPPGMWWEQYGDSAPSLQRAAVRITSQVCSTLTFQRDWGVILQNHYEKRNKLDKEALADQAYVHYNLTLHSEPKARRRPDADPIALDAVDMTSAWVEDSDGPILTQWLDRFPSALDGGDLNTRQFGGSIFGTNDNLFGL	zf-BED--DUF-domain--Dimer_Tnp_hAT--	I
Zm00001d03	ZmZf-	MRERDACWEYCDKLDGNKVRCRFCHKVLNGGISRLKFHLSQIPSKGVNPCTKVKEDVVDRVKAVISAKEEYKEFQLLKRQRVADLSSAAAPAKWAPEAPPSLSTSPGRVASPAAVITRAAEQSRLLAPEASAPVPRPSAAAANNKPRAAAASEWETERCIAEFFFENKLDYSIADSVSYQQMLEALGGPGFRGPPADVLRDKWLQRLKSEILQKTKEIEKDWATTGCTILADSWTDNKL	zf-BED--DUF-domain--	I

9328_P005	BED49_I	KALINFSVASPMGTFFLKTVDASSHFKTHRGLYDLFDEVIQEVGPENVVQIIADRNINYGSTDKLVAQNYGGAIFWSPCASFCVNAMLDDFSKIDWVNQCICQAQTVTRFIYNSRWVLDLARKCVAGQELVCSGITKSVSDFLTLKSLLRHRPKLKQMFHSAEFSSSSYASRSIPCVEILDDDELWRAVEEIAAVSEPLLRVMRDVSGGRQAIGYIYESMTKVTDSIRTYYIMDEGKCKSFLDIVEQRWQTELHSPLHSAAAFLSPGIQYNPEVKFFRTIKEEFYQVLDKVLTTPDQRHDITAQLHAFRKAQGLFGSNIAKEARNNTPPGMWWEQYGDSAPSLQRAAVRITSQVCSTLTFQRDWGVILQNHYEKRNKLDKEALADQAYVHYNLTLHSEPKARRRPDADPIALDAVDMTSAWVEDSDGPILTQWLDRFPSALDGGDLNTRQFGGSIFGTNDNLFGL	Dimer_Tnp_hAT--	
Zm00001d039328_P006	ZmZf-BED50_I	MGVGLKGNGDESIAAPSCCSARKSKDTPIRSPAAAPQMRERDACWEYCDKLDGNKVRCRFCHKVLNGGISRLKFHLSQIPSKGVNPCTKVKEDVVDRVKAVISAKEEYKEFQLLKRQRVADLSSAAAPAKWAPEAPPSLSTSPGRVASPAAVITRAAEQSRLLAPEASAPVPRPSAAAANNKPRAAAASEWETERCIAEFFFENKLDYSIADSVSYQQMLEALGGPGFRGPPADVLRDKWLQRLKSEILQKTKEIEKDWATTGCTILADSWTDNKLKALINFSVASPMGTFFLKTVDASSHFKTHRGLYDLFDEVIQEVGPENVVQIIADRNINYGSTDKLVAQNYGGAIFWSPCASFCVNAMLDDFSKIDWVNQCICQAQTVTRFIYNSRWVLDLARKCVAGQELVCSGITKSVSDFLTLKSLLRHRPKLKQMFHSAEFSSSSYASRSIPCVEILDDDELWRAVEEIAAVSEPLLRVMRDVSGGRQAIGYIYESMTKVTDSIRTYYIMDEGKCKSFLDIVEQRWQTELHSPLHSAAAFLSPGIQYNPEVKFFRTIKEEFYQVLDKVLTTPDQRHDITAQLHAFRKAQGLFGSNIAKEARNNTPPGMWWEQYGDSAPSLQRAAVRITSQVCSTLTFQRDWGVILQNHYEKRNKLDKEALADQAYVHYNLTLHSEPKARRRPDADPIALDAVDMTSAWVEDSDGPILTQWLDRFPSALDGGDLNTRQFGGSIFGTNDNLFGL	zf-BED--DUF-domain--Dimer_Tnp_hAT--	I
Zm00001d039328_P007	ZmZf-BED51_I	MRERDACWEYCDKLDGNKVRCRFCHKVLNGGISRLKFHLSQIPSKGVNPCTKVKEDVVDRVKAVISAKEEYKEFQLLKRQRVADLSSAAAPAKWAPEAPPSLSTSPGRVASPAAVITRAAEQSRLLAPEASAPVPRPSAAAANNKPRAAAASEWETERCIAEFFFENKLDYSIADSVSYQQMLEALGGPGFRGPPADVLRDKWLQRLKSEILQKTKEIEKDWATTGCTILADSWTDNKLKALINFSVASPMGTFFLKTVDASSHFKTHRGLYDLFDEVIQEVGPENVVQIIADRNINYGSTDKLVAQNYGGAIFWSPCASFCVNAMLDDFSKIDWVNQCICQAQTVTRFIYNSRWVLDLARKCVAGQELVCSGITKSVSDFLTLKSLLRHRPKLKQMFHSAEFSSSSYASRSIPCVEILDDDELWRAVEEIAAVSEPLLRVMRDVSGGRQAIGYIYESMTKVTDSIRTYYIMDEGKCKSFLDIVEQRWQTELHSPLHSAAAFLSPGIQYNPEVKFFRTIKEEFYQVLDKVLTTPDQRHDITAQLHAFRKAQGLFGSNIAKEARNNTPPGMWWEQYGDSAPSLQRAAVRITSQVCSTLTFQRDWGVILQNHYEKRNKLDKEALADQAYVHYNLTLHSEPKARRRPDADPIALDAVDMTSAWVEDSDGPILTQWLDRFPSALDGGDLNTRQFGGSIFGTNDNLFGL	zf-BED--DUF-domain--Dimer_Tnp_hAT--	I
Zm00001d043354_P002	ZmZf-BED52_II	MEGVSSAPEMDEAISTDQSSRSTKRRAKVWDHVDSELIDGKEKAVCKYCKAHLSSAAGKGEPMTGKRMFDSDDEEYDWEI	zf-BED	II
Zm00001d043354_P006	ZmZf-BED53_I	MEGVSSAPEMDEAISTDQSSRSTKRRAKVWDHVDSELIDGKEKAVCKYCKAHLSSAAGKGTTHLNRHISVYCHAIPPEERQRFLATQKTKPDVAHVFDPVVFRGLIAKYFLSAEISFRKCEDPSWKEMINYCQPSFRLVGRQTVRSDCMLLYEEEKLQLIEQFTKLKSHVSLTADLWSSNQNLGYLGVTAHFISEDFELHKKIIAFKKISFPHTSYAVQDGITSCLLEWGLVGDLFTLTLDNASVNNRAMKDMRDALGSQMFFSGEHLHVRCSSHVLNIMVQAGLKVVPNAVGKVRDIIKVVISTPSRLQIFNSIVQTLGLRSKSGLILDVPHRWNATYDMLNEALKYKAALNRYAVEQHHECPTEEDWSKAEALHGFLQEFSDATKAFSADRHPTAHLFLKMLLVIRGVLLDENYNSNELLNEMANVMYTKFQKYWINPNIVLLIAAVLDPSMKTEFVKFYFYTVGDNVDMKMRELKRYLKKYYLEYEKIMRSHSLHVFITPDDQMRSEPSTSSPLCGKRRVEHAFAQFASQNANARPERTEMDTYLEDPRILIKPDECFNVLGWWKKNSDAYPILSLMARDFMAIPVSTVSSESAFSAAGRVLGKNRTSLSPETLEALVCAKDWLIGFNDEEEGKLSYKSRLLYDNELNCLRHFTR	zf-BED--DUF-domain--Dimer_Tnp_hAT--	I
Zm00001d047922_P001	ZmZf-BED54_II	MDLRISEGSGTNTTLDDQSVQSPTRRAKVWEYFQQELVEVDGVMKAVCKYCGTKLTSKRNSGTNSLRNHVADTCPKISVEDRKRFIATMRKKPGEGSFVFDPRKTRECMVKWCISAEVAFNKFDDPFFAPWMESLQPSFSGVGRQTMRNDCIARFKMMRQELRNELQSLNSRICLTSDLWTSN	zf-BED	II
Zm00001d049450_P005	ZmZf-BED55_I	MDHSDEHDSDDMKVGAASDGPSRLRKKRSKVWDEYEPIIVDGAIQSAECRYCHMHMSCRGADGQSNGTSHLWRHQKICRAKDEFCPSQLQQDADFSSVINEVEPLEQILPDSLDEIKLVTHSENSKFRSKVWKDFIPFYVQGRVQGADCVHCHKRLTADKGRSHLNRHTQTCPARSGNILNHQKGVSFQSNLPSSKSSLQDELSPALTNGKIQIAEYASKFLKGSSSDASLVERHVLALPAMYDMNPSEKSTPSAQTAADRTRKTDQEASYLELTRMVISHGYPLSIVEHEEMRRFAKSLNPTFNMASSIDIEEYSTLLFQKEKADLKERIALLSRRVSLSASVWAPDGAEASVKYLCLAVHFIDSDWKLQRKTIKFGVFWSLPTSLERMIQFKEACVLDSDIGPFNVIQEALRDWNLDQKHFSLTSGSEIRNDEGTSKLMDLLIQRKCLPIRGELYNIDCVNDVLNNIVSKGQQVLCHVGNILETFIRAHMSSSLTRQQLLEAVAHMGLKCPHEDAKWWHIIYFRLEVLLHFKKAFPSEELLSAGDNKAVESVCRILRAFYHAVEVICSPICPTSNVYFNELWKVRTVLQEEASTDLIELANMVWEMQEAFNEYWQNSYLWLSIPVVLDPRFKITFIEFRLKRAFGTNAEKYVSAIRDTIRELFHEYCGLSNNLGGDTSTREVELDEFDSDSLEDWDEHLNAQTRNQQLRELDNYLEDGIVPRKDDFDILNWWMSNSTKYPTLSIMARDVLAVPASAVNFEAALSGKVNVIHKQWSTLNIKTIEALVCTRDWVK	zf-BED--DUF-domain--Dimer_Tnp_hAT--	I
Zm00001d049450_P007	ZmZf-BED56_I	MDHSDEHDSDDMKVGAASDGPSRLRKKRSKVWDEYEPIIVDGAIQSAECRYCHMHMSCRGADGQSNGTSHLWRHQKICRAKDEFCPSQLQQDADFSYVVINEVEPLEQILPDSLDEIKLVTHSENSKFRSKVWKDFIPFYVQGRVQGADCVHCHKRLTADKGRSHLNRHTQTCPARSGNILNHQKGVSFQSNLPSSKSSLQDELSPALTNGKIQIAEYASKFLKGSSSDASLVERHVLALPAMYDMNPSEKSTPSAQTAADRTRKTDQEASYLELTRMVISHGYPLSIVEHEEMRRFAKSLNPTFNMASSIDIEEYSTLLFQKEKADLKERIALLSRRVSLSASVWAPDGAEASVKYLCLAVHFIDSDWKLQRKTIKFGVFWSLPTSLERMIQFKEACVLDSDIGPFNVIQEALRDWNLDQKHFSLTSGSEIRNDEGTSKLMDLLIQRKCLPIRGELYNIDCVNDVLNNIVSKGQQVLCHVGNILETFIRAHMSSSLTRQQLLEAVAHMGLKCPHEDAKWWHIIYFRLEVLLHFKKAFPSEELLSAGDNKAVESVCRILRAFYHAVEVICSPICPTSNVYFNELWKVRTVLQEEASTDLIELANMVWEMQEAFNEYWQNSYLWLSIPVVLDPRFKITFIEFRLKRAFGTNAEKYVSAIRDTIRELFHEYCGLSNNLGGDTSTREVELDEFDSDSLEDWDEHLNAQTRNQQLRELDNYLEDGIVPRKDDFDILNWWMSNSTKYPTLSIMARDVLAVPASAVNFEAALSGKVNVIHKQWSTLNIKTIEALVCTRDWVK	zf-BED--DUF-domain--Dimer_Tnp_hAT--	I
Zm00001d049450_P008	ZmZf-BED57_IV	MGSLHVASLAWLPPLHWSGACSTATVMGHPCPHTAVSSVRGSSWLRRSTSGSRTALTAAAAGRGITPQIWRWRCAGGRIRYMFEQVDNIGLSTDMDHSDEHDSDDMKVGAASDGPSRLRKKRSKVWDEYEPIIVDGAIQSAECRYCHMHMSCRGADGQSNGTSHLWRHQKICRAKDEFCPSQLQQDADFSSVLRPYFNVVKDIKSLIVINEVEPLEQILPDSLDEIKLVTHSENSKFRSKVWKDFIPFYVQGRVQGADCVHCHKRLTADKGRSHLNRHTQTCPARSGNILNHQKGVSFQSNLPSSKSSLQDELSPALTNGKIQIAEYASKFLKGSSSDASLVERHVLALPAMYDMNPSEKSTPSAQTAADRTRKTDQEASYLELTRMVISHGYPLSIVEHEEMRRFAKSLNPTFNMASSIDIEEYSTLLFQKEKADLKERIALLSRRVSLSASVWAPDGAEASVKYLCLAVHFIDSDWKLQRKTIKFGVFWSLPTSLERMIQFKEACVLDSDIGPFNVIQEALRDWNLDQKHFSLTSGSEIRNDEGTSKLMDLLIQRKCLPIRGELYNIDCVNDVLNNIVSKGQQVLCHVGNILETFIRAHMSSSLTRQQLLEAVAHMGLKCPHEDAKWWHIIYFRLEVLLHFKKAFPSEELLSAGDNKAVESVCRILRAFYHAVEVICSPICPTSNVYFNELWKVRTVLQEEASTDLIELANMVWEMQEAFNEYWQNSYLWLSIPVVLDPRFKITFIEFRLKRAFGTNAEKAD	zf-BED--DUF-domain	IV
Zm00001d050879_P001	ZmZf-BED58_II	MSDEDRDRERDRDKVWLHGEKVGAGFKCKYCRETKSGGGGTRLKEHLVHRGKNVKKCPSVPPDIKAYFQLDIDKTKENKSSRFRQQLRADEAARTHFGDDEYEDELKAALHQSQEEEDEDSDDNTTDPSAGDDGSHGDAGLYGVGGSGAGGSGTGGSGAGDWRSTGGSRFTHSTQDSYHDALYSQRETISGRRRSVSFPVQDGIRSSSSSGSSNYPTGQDLVYNPYAWQWQPSQRTYGPPPPLGEAPPSIMYRYGNYGPPPPPYSNLSNMYPPGPQYGYGQVPAAPTFHYQYDGLDQYHDPSNMPEYYHYDSS	zf-BED	II

Table S3 List of classes, domain architectures and number of genes

Class No.	Domain Architecture	Total no. of genes
I	zf-BED--DUF-domain--Dimer_Tnp_hAT	390
II	zf-BED	144
III	zf-BED--zf-BED--DUF-domain--Dimer_Tnp_hAT	49
IV	zf-BED--DUF-domain	46
V	zf-BED--Dimer_Tnp_hAT	19
VI	zf-BED--PHD	14
VII	zf-BED--zf-BED	11
VIII	GRAS--zf-BED--DUF-domain--Dimer_Tnp_hAT	8
IX	GRAS--zf-BED--DUF-domain--Dimer_Tnp_hAT--Peptidase_C48	6
X	WRKY--zf-BED--DUF-domain--Dimer_Tnp_hAT	6
XI	zf-BED--DUF-domain--Dimer_Tnp_hAT--Peptidase_C48	6
XII	zf-BED--NB-ARC	6
XIII	GST_N_3--GST_C_3--zf-BED	4
XIV	zf-BED--NB-ARC--LRR-motif	4
XV	zf-BED--zf-BED--DUF-domain	4
XVI	WRKY--zf-BED	3
XVII	zf-BED--PPR-domain	3
XVIII	GRAS--zf-BED	2
XIX	Myosin_TH1--zf-BED--DUF-domain--Dimer_Tnp_hAT	2
XX	zf-BED--Sina	2
XXI	zf-BED--XH	2
XXII	DnaJ--zf-BED--zf-BED--DUF-domain--Dimer_Tnp_hAT	1
	DUF2647--zf-BED--DUF-domain--Dimer_Tnp_hAT	1
	ER_lumen_recept--zf-BED	1
	F-box--LRR-motif--zf-BED	1
	Gelsolin--Gelsolin--zf-BED--DUF-domain--Dimer_Tnp_hAT	1
	Glyco_hydro_1--zf-BED--DUF-domain--Glyco_hydro_1	1
	Glyco_hydro_79n--zf-BED	1
	GRAS--zf-BED--DUF-domain--Dimer_Tnp_hAT--Peptidase_C48--RWP-RK--PB1	1
	NAM--zf-BED	1
	RVT_3--zf-BED--DUF-domain--Dimer_Tnp_hAT	1
	zf-BED--DUF-domain	1
	Total	11

Table S4 major classes and their genes with respect to species.

Species name	I	II	III	IV	V	VI	VII	VIII	IX	X	XI	XII	XIII	XIV	XV	XVI	XVII	XVIII	XIX	XX	XXI	XXII	Total Transcripts	Gene Locus
Physcomitrella patens	0	0	0	0	0	0	0	0	0	0	0	0	0	0	0	0	0	0	0	0	0	0	0	0
Marchantia polymorpha	0	0	0	0	1	0	0	0	0	0	0	0	4	0	0	0	0	0	0	0	0	0	5	2
Ginkgo biloba	0	0	0	0	0	0	0	0	0	0	0	0	0	0	0	0	0	0	0	0	0	0	0	0
selaginella moellendorffii	0	0	0	0	0	0	0	0	0	0	0	0	0	0	0	0	0	0	0	0	0	0	0	0
Picea abies	0	8	0	0	0	0	0	0	0	0	0	0	0	0	0	0	0	0	0	0	0	0	8	8
Amborella trichopoda	1	0	0	0	1	0	0	0	0	0	0	0	0	0	0	0	0	0	0	0	0	0	2	2
Spirodela polyrhiza	3	0	1	1	1	0	0	0	0	0	0	0	0	0	0	0	0	0	0	0	0	1	7	7
Zea mays	24	19	2	2	3	6	0	0	0	0	0	0	0	0	0	0	0	0	0	0	0	2	58	33
Sorghum bicolor	2	5	0	0	0	1	0	0	0	0	0	0	0	0	0	1	0	0	0	0	0	0	9	6
Oryza sativa	23	18	4	2	2	1	0	0	0	6	0	1	0	0	0	2	0	0	0	0	0	0	59	42
Brachypodium distachyon	0	5	0	0	0	6	0	0	0	0	0	1	0	0	0	0	0	0	0	0	0	1	13	7
Hordeum vulgare	13	12	2	2	0	0	1	0	0	0	0	0	0	0	0	0	0	0	0	0	0	0	30	18
Aquilegia coerulea	15	4	3	3	1	0	0	0	0	0	0	0	0	0	2	0	3	0	0	0	2	1	34	24
Nelumbo nucifera	2	4	0	0	0	0	1	0	0	0	0	0	0	0	0	0	0	0	0	0	0	2	9	9
Solanum lycopersicum	0	0	8	0	0	0	9	0	0	0	0	0	0	0	0	0	0	0	0	0	0	2	19	19
Solanum tuberosum	15	3	0	1	0	0	0	0	0	0	0	0	0	0	0	0	0	0	0	0	0	0	19	19
Solanum melongena	6	6	0	1	2	0	0	0	0	0	0	0	0	0	0	0	0	0	0	0	0	3	18	18
Vitis vinifera	5	0	1	0	0	0	0	0	0	0	0	0	0	0	0	0	0	0	0	0	0	2	8	8
Citrus clementina	5	2	1	0	0	0	0	0	0	0	0	0	0	0	0	0	0	0	0	0	0	0	8	8
Theobroma cacao	19	2	8	1	1	0	0	0	1	0	0	0	0	0	0	0	0	0	0	0	0	0	32	32
Gossypium arboreum	13	17	1	13	1	0	0	3	1	0	1	0	0	0	0	0	0	0	1	0	0	1	52	52
Gossypium raimondii	6	2	1	0	0	0	0	0	0	0	0	0	0	0	0	0	0	0	0	0	0	0	9	9
Gossypium barbadence	66	3	2	1	0	0	0	2	0	0	2	0	0	0	0	0	0	0	0	0	0	1	77	44
Gossypium hirsutum	52	5	5	5	1	0	0	2	2	0	1	0	0	0	0	0	0	0	0	0	0	0	73	41
Gossypium turneri	10	1	1	2	1	0	0	1	2	0	2	0	0	0	0	0	0	0	1	0	0	0	21	21
Gossypium herbaceum	30	3	0	1	2	0	0	0	0	0	0	0	0	0	0	0	0	0	0	0	0	0	36	36
Gossypium thurberi	5	1	0	1	0	0	0	0	0	0	0	0	0	0	1	0	0	0	0	0	0	0	8	8
Capsella rubella	10	1	0	0	1	0	0	0	0	0	0	0	0	0	1	0	0	0	0	1	0	0	14	14
Arabidopsis thaliana	4	1	1	1	0	0	0	0	0	0	0	0	0	0	0	0	0	0	0	0	0	0	7	6

Arabidopsis lyrata	6	0	2	0	0	0	0	0	0	0	0	0	0	0	0	0	0	0	0	1	0	0	9	8
Prunus persica	18	3	3	3	0	0	0	0	0	0	0	0	0	0	0	0	0	0	0	0	0	0	27	14
Populus trichocarpa	3	11	0	0	0	0	0	0	0	0	0	4	0	3	0	0	0	2	0	0	1	0	24	19
Medicago truncatula	15	6	1	6	1	0	0	0	0	0	0	0	0	1	0	0	0	0	0	0	0	1	31	31
Betula pendula	8	2	1	0	0	0	0	0	0	0	0	0	0	0	0	0	0	0	0	0	0	1	12	12
Cucumis sativus	11	0	1	0	0	0	0	0	0	0	0	0	0	0	0	0	0	0	0	0	0	0	12	7

Table S5 Statistical summary of comparative genomics

Number of species	26
Number of genes	565
Number of genes in orthogroups	564
Number of unassigned genes	1
Percentage of genes in orthogroups	99.8
Percentage of unassigned genes	0.2
Number of orthogroups	9
Number of species-specific orthogroups	3
Number of genes in species-specific orthogroups	6
Percentage of genes in species-specific orthogroups	1.1
Mean orthogroup size	62.7
Median orthogroup size	15
G50 (assigned genes)	330
G50 (all genes)	330
O50 (assigned genes)	1
O50 (all genes)	1
Number of orthogroups with all species present	1
Number of single-copy orthogroups	0

Table S6 Species wise statistical summary of Zf-BED genes in land plants

	Al	At	Aqu	Bpe	Bra	Car	Cic	Cuc	Vvi	Gar	Gba	Ghe	Ghi	Got	Gra	Nnu	OsR	Pab	Sot	Pru	Sme	Sob	Sol	Spi	Tca	Gtu
Number of genes	9	6	25	11	7	12	8	8	8	52	72	35	72	8	9	9	58	8	19	27	15	9	19	7	31	21
Number of genes in orthogroups	9	5	25	11	7	12	8	8	8	52	72	35	72	8	9	9	58	8	19	27	15	9	19	7	31	21
Number of unassigned genes	0	1	0	0	0	0	0	0	0	0	0	0	0	0	0	0	0	0	0	0	0	0	0	0	0	0
Percentage of genes in orthogroups	100	83.3	100	100	100	100	100	100	100	100	100	100	100	100	100	100	100	100	100	100	100	100	100	100	100	100
Percentage of unassigned genes	0	16.7	0	0	0	0	0	0	0	0	0	0	0	0	0	0	0	0	0	0	0	0	0	0	0	0
Number of orthogroups containing species	4	2	4	3	2	4	3	3	3	5	4	5	4	2	3	3	4	1	4	4	3	3	4	3	3	3
Percentage of orthogroups containing species	44.4	22.2	44.4	33.3	22.2	44.4	33.3	33.3	33.3	55.6	44.4	55.6	44.4	22.2	33.3	33.3	44.4	11.1	44.4	44.4	33.3	33.3	44.4	33.3	33.3	33.3
Number of species-specific orthogroups	0	0	0	0	0	0	0	0	0	0	0	0	0	0	0	0	2	0	0	0	1	0	0	0	0	0
Number of genes in species-specific orthogroups	0	0	0	0	0	0	0	0	0	0	0	0	0	0	0	0	4	0	0	0	2	0	0	0	0	0
Percentage of genes in species-specific orthogroups	0	0	0	0	0	0	0	0	0	0	0	0	0	0	0	0	6.9	0	0	0	13.3	0	0	0	0	0

Table S7 Orthogroups and gene count per plant species

Orthogroup	Al	At	Aqu	Bpe	Bra	Car	Cic	Cuc	Vvi	Gar	Gba	Ghe	Ghi	Got	Gra	Nnu	OsR	Pab	Sot	Pru	Sme	Sob	Sol	Spi	Tca	Gtu	Total
OG0	4	0	7	5	5	8	4	5	1	26	49	27	53	5	5	2	42	0	11	8	8	7	12	4	18	14	330
OG1	3	4	9	5	2	2	3	1	6	11	19	3	17	3	3	6	12	8	6	14	5	1	5	2	12	6	168
OG2	1	1	1	1	0	1	1	2	1	1	2	1	1	0	1	1	0	0	1	2	0	0	1	1	1	1	23
OG3	0	0	0	0	0	0	0	0	0	13	2	3	1	0	0	0	0	0	0	0	0	1	0	0	0	0	20
OG4	0	0	8	0	0	0	0	0	0	1	0	1	0	0	0	0	0	0	1	3	0	0	1	0	0	0	15
OG5	1	0	0	0	0	1	0	0	0	0	0	0	0	0	0	0	0	0	0	0	0	0	0	0	0	0	2
OG6	0	0	0	0	0	0	0	0	0	0	0	0	0	0	0	0	2	0	0	0	0	0	0	0	0	0	2
OG7	0	0	0	0	0	0	0	0	0	0	0	0	0	0	0	0	2	0	0	0	0	0	0	0	0	0	2
OG8	0	0	0	0	0	0	0	0	0	0	0	0	0	0	0	0	0	0	0	0	2	0	0	0	0	0	2

Table S8 Orthogroups and species overlap

	Al	At	Aqu	Bpe	Bra	Car	Cic	Cuc	Vvi	Gar	Gba	Ghe	Ghi	Got	Gra	Nnu	OsR	Pab	Sot	Pru	Sme	Sob	Sol	Spi	Tca	Gtu
**Al**	4	2	3	3	2	4	3	3	3	3	3	3	3	2	3	3	2	1	3	3	2	2	3	3	3	3
**At**	2	2	2	2	1	2	2	2	2	2	2	2	2	1	2	2	1	1	2	2	1	1	2	2	2	2
**Aqu**	3	2	4	3	2	3	3	3	3	4	3	4	3	2	3	3	2	1	4	4	2	2	4	3	3	3
**Bpe**	3	2	3	3	2	3	3	3	3	3	3	3	3	2	3	3	2	1	3	3	2	2	3	3	3	3
**Bra**	2	1	2	2	2	2	2	2	2	2	2	2	2	2	2	2	2	1	2	2	2	2	2	2	2	2
**Car**	4	2	3	3	2	4	3	3	3	3	3	3	3	2	3	3	2	1	3	3	2	2	3	3	3	3
**Cic**	3	2	3	3	2	3	3	3	3	3	3	3	3	2	3	3	2	1	3	3	2	2	3	3	3	3
**Cuc**	3	2	3	3	2	3	3	3	3	3	3	3	3	2	3	3	2	1	3	3	2	2	3	3	3	3
**Vvi**	3	2	3	3	2	3	3	3	3	3	3	3	3	2	3	3	2	1	3	3	2	2	3	3	3	3
**Gar**	3	2	4	3	2	3	3	3	3	5	4	5	4	2	3	3	2	1	4	4	2	3	4	3	3	3
**Gba**	3	2	3	3	2	3	3	3	3	4	4	4	4	2	3	3	2	1	3	3	2	3	3	3	3	3
**Ghe**	3	2	4	3	2	3	3	3	3	5	4	5	4	2	3	3	2	1	4	4	2	3	4	3	3	3
**Ghi**	3	2	3	3	2	3	3	3	3	4	4	4	4	2	3	3	2	1	3	3	2	3	3	3	3	3
**Got**	2	1	2	2	2	2	2	2	2	2	2	2	2	2	2	2	2	1	2	2	2	2	2	2	2	2
**Gra**	3	2	3	3	2	3	3	3	3	3	3	3	3	2	3	3	2	1	3	3	2	2	3	3	3	3
**Nnu**	3	2	3	3	2	3	3	3	3	3	3	3	3	2	3	3	2	1	3	3	2	2	3	3	3	3
**OsR**	2	1	2	2	2	2	2	2	2	2	2	2	2	2	2	2	4	1	2	2	2	2	2	2	2	2
**Pab**	1	1	1	1	1	1	1	1	1	1	1	1	1	1	1	1	1	1	1	1	1	1	1	1	1	1
**Sot**	3	2	4	3	2	3	3	3	3	4	3	4	3	2	3	3	2	1	4	4	2	2	4	3	3	3
**Pru**	3	2	4	3	2	3	3	3	3	4	3	4	3	2	3	3	2	1	4	4	2	2	4	3	3	3
**Sme**	2	1	2	2	2	2	2	2	2	2	2	2	2	2	2	2	2	1	2	2	3	2	2	2	2	2
**Sob**	2	1	2	2	2	2	2	2	2	3	3	3	3	2	2	2	2	1	2	2	2	3	2	2	2	2
**Sol**	3	2	4	3	2	3	3	3	3	4	3	4	3	2	3	3	2	1	4	4	2	2	4	3	3	3
**Spi**	3	2	3	3	2	3	3	3	3	3	3	3	3	2	3	3	2	1	3	3	2	2	3	3	3	3
**Tca**	3	2	3	3	2	3	3	3	3	3	3	3	3	2	3	3	2	1	3	3	2	2	3	3	3	3
**Gtu**	3	2	3	3	2	3	3	3	3	3	3	3	3	2	3	3	2	1	3	3	2	2	3	3	3	3

Table S9: Duplication events in ZfBED genes per orthogroup in land plants

Orthogroup	Duplications (all)	Duplications (50% support)
OG0	194	151
OG1	84	70
OG2	3	2
OG3	12	3
OG4	9	9

Table S10. Orthologues one-to-one plant species

	Al	At	Aqu	Bpe	Bra	Car	Cic	Cuc	Vvi	Gar	Gba	Ghe	Ghi	Got	Gra	Nnu	OsR	Pab	Sot	Pru	Sme	Sob	Sol	Spi	Tca	Gtu
**Al**	0	2	1	3	0	6	1	2	2	2	0	3	1	2	2	1	1	0	2	2	2	0	3	2	2	2
**At**	2	0	2	2	0	3	1	1	3	3	1	3	2	3	2	3	1	0	1	0	2	1	2	3	2	3
**Aqu**	1	2	0	3	0	1	2	1	3	2	0	1	1	1	2	3	1	0	1	0	0	0	1	1	1	2
**Bpe**	3	2	3	0	1	4	2	2	5	5	0	4	1	3	3	4	1	0	2	2	4	0	5	1	4	5
**Bra**	0	0	0	1	0	0	0	0	0	0	0	0	0	0	0	0	2	0	1	1	0	1	2	0	1	0
**Car**	6	3	1	4	0	0	1	2	3	3	1	3	2	2	2	2	0	0	2	2	2	1	4	2	3	3
**Cic**	1	1	2	2	0	1	0	1	1	2	0	1	1	1	2	2	0	0	1	1	0	0	1	1	1	2
**Cuc**	2	1	1	2	0	2	1	0	2	2	0	1	0	2	3	1	0	0	2	3	1	0	3	0	1	2
**Vvi**	2	3	3	5	0	3	1	2	0	4	0	3	1	2	3	4	1	0	2	1	2	0	4	1	3	4
**Gar**	2	3	2	5	0	3	2	2	4	0	8	7	7	4	4	3	0	0	2	1	3	0	5	1	4	9
**Gba**	0	1	0	0	0	1	0	0	0	8	0	4	15	2	2	1	0	0	0	0	0	1	1	0	1	2
**Ghe**	3	3	1	4	0	3	1	1	3	7	4	0	4	1	3	2	1	0	2	1	5	1	5	2	5	6
**Ghi**	1	2	1	1	0	2	1	0	1	7	15	4	0	3	4	2	0	0	1	0	0	2	2	1	3	3
**Got**	2	3	1	3	0	2	1	2	2	4	2	1	3	0	3	2	1	0	0	1	3	0	2	2	3	5
**Gra**	2	2	2	3	0	2	2	3	3	4	2	3	4	3	0	2	0	0	2	1	0	0	2	1	3	6
**Nnu**	1	3	3	4	0	2	2	1	4	3	1	2	2	2	2	0	1	0	1	0	1	0	3	2	3	4
**OsR**	1	1	1	1	2	0	0	0	1	0	0	1	0	1	0	1	0	0	1	1	1	0	1	1	0	0
**Pab**	0	0	0	0	0	0	0	0	0	0	0	0	0	0	0	0	0	0	0	0	0	0	0	0	0	0
**Sot**	2	1	1	2	1	2	1	2	2	2	0	2	1	0	2	1	1	0	0	2	2	0	7	1	1	1
**Pru**	2	0	0	2	1	2	1	3	1	1	0	1	0	1	1	0	1	0	2	0	2	0	3	0	1	1
**Sme**	2	2	0	4	0	2	0	1	2	3	0	5	0	3	0	1	1	0	2	2	0	0	5	1	5	3
**Sob**	0	1	0	0	1	1	0	0	0	0	1	1	2	0	0	0	0	0	0	0	0	0	1	0	1	0
**Sol**	3	2	1	5	2	4	1	3	4	5	1	5	2	2	2	3	1	0	7	3	5	1	0	1	6	5
**Spi**	2	3	1	1	0	2	1	0	1	1	0	2	1	2	1	2	1	0	1	0	1	0	1	0	1	1
**Tca**	2	2	1	4	1	3	1	1	3	4	1	5	3	3	3	3	0	0	1	1	5	1	6	1	0	7
**Gtu**	2	3	2	5	0	3	2	2	4	9	2	6	3	5	6	4	0	0	1	1	3	0	5	1	7	0

Table S11 Orthologues one-to-many plant species

	Al	At	Aqu	Bpe	Bra	Car	Cic	Cuc	Vvi	Gar	Gba	Ghe	Ghi	Got	Gra	Nnu	OsR	Pab	Sot	Pru	Sme	Sob	Sol	Spi	Tca	Gtu
**AL**	0	0	1	0	0	0	0	1	0	1	4	1	3	0	1	1	0	0	1	2	0	0	0	0	2	2
**At**	1	0	3	1	1	0	1	1	1	1	4	0	2	0	1	1	2	1	0	3	0	0	1	0	2	1
**Aqu**	1	0	0	0	0	0	0	1	0	0	2	0	1	0	0	0	0	0	0	2	0	0	1	0	1	0
**Bpe**	0	0	0	0	0	0	0	1	0	1	6	1	4	0	0	0	0	0	2	3	0	0	1	0	2	1
**Bra**	0	0	0	0	0	0	0	0	0	0	0	0	0	0	0	1	0	0	0	0	0	1	1	0	0	0
**Car**	0	0	2	0	1	0	0	1	1	2	4	1	2	0	0	0	2	1	1	1	0	0	1	0	1	1
**Cic**	0	0	1	1	0	0	0	1	0	1	3	1	2	0	1	0	1	0	1	1	1	0	0	1	1	0
**Cuc**	2	0	0	2	0	2	1	0	0	3	5	3	5	1	1	0	0	0	3	2	2	0	1	0	2	2
**Vvi**	1	0	1	1	0	0	2	1	0	1	5	1	3	0	0	0	1	0	1	3	0	0	0	0	2	1
**Gar**	1	0	1	0	0	0	0	1	0	0	7	1	7	1	1	0	0	0	2	2	0	0	0	0	2	0
**Gba**	0	0	1	0	1	0	0	0	1	3	0	4	5	0	0	0	1	1	1	0	1	0	1	0	1	2
**Ghe**	0	0	2	0	1	0	0	1	0	3	7	0	5	1	0	1	1	0	2	3	0	0	0	0	0	0
**Ghi**	0	0	1	0	1	0	0	1	1	2	5	2	0	0	0	0	1	1	1	1	1	0	1	0	0	0
**Got**	1	0	2	0	0	0	0	0	0	1	6	0	4	0	0	1	2	0	1	1	0	0	0	0	1	0
**Gra**	1	0	0	0	0	0	0	1	0	1	5	1	3	0	0	0	2	0	1	1	1	0	1	1	3	1
**Nnu**	1	0	1	0	0	0	0	1	0	0	3	0	1	0	0	0	1	0	1	2	1	0	0	0	1	0
**OsR**	0	0	1	0	0	0	0	0	0	0	1	0	1	0	1	2	0	0	0	2	0	1	0	0	1	1
**Pab**	0	0	0	0	0	0	0	0	0	0	0	0	0	0	0	0	0	0	0	0	0	0	0	0	0	0
**Sot**	1	0	1	0	0	1	0	1	0	1	2	1	1	0	0	1	1	0	0	3	0	2	1	0	1	1
**Pru**	0	0	1	1	0	0	0	0	0	2	3	2	3	0	1	1	1	0	2	0	1	1	0	0	1	1
**Sme**	0	0	1	0	1	0	0	0	0	0	4	0	3	0	1	2	2	0	3	1	0	1	1	0	1	1
**Sob**	0	0	1	0	2	0	0	0	1	2	1	1	0	0	0	0	3	1	0	0	0	0	1	0	0	0
**Sol**	1	0	1	0	0	1	0	1	0	1	5	1	3	0	0	1	1	0	3	3	1	1	0	0	1	1
**Spi**	0	0	2	1	0	0	0	1	1	1	3	1	2	0	1	1	1	0	1	2	1	0	1	0	2	2
**Tca**	0	0	0	0	0	0	0	1	0	0	6	0	3	0	0	0	1	0	3	1	1	0	0	0	0	0
**Gtu**	1	0	0	0	0	0	0	1	0	1	11	0	9	1	0	0	0	0	3	1	1	0	0	0	2	0

Table S12: Orthologues many-to-one plant species

	Al	At	Aqu	Bpe	Bra	Car	Cic	Cuc	Vvi	Gar	Gba	Ghe	Ghi	Got	Gra	Nnu	OsR	Pab	Sot	Pru	Sme	Sob	Sol	Spi	Tca	Gtu
**Al**	0	2	2	0	0	0	0	4	2	2	0	0	0	2	2	2	0	0	2	0	0	0	2	0	0	2
**At**	0	0	0	0	0	0	0	0	0	0	0	0	0	0	0	0	0	0	0	0	0	0	0	0	0	0
**Aqu**	2	7	0	0	0	5	2	0	2	8	3	10	3	4	0	2	2	0	8	3	2	3	8	4	0	0
**Bpe**	0	2	0	0	0	0	2	4	2	0	0	0	0	0	0	0	0	0	0	2	0	0	0	2	0	0
**Bra**	0	2	0	0	0	2	0	0	0	0	2	2	2	0	0	0	0	0	0	0	5	4	0	0	0	0
**Car**	0	0	0	0	0	0	0	6	0	0	0	0	0	0	0	0	0	0	2	0	0	0	2	0	0	0
**Cic**	0	3	0	0	0	0	0	3	5	0	0	0	0	0	0	0	0	0	0	0	0	0	0	0	0	0
**Cuc**	2	2	2	2	0	2	2	0	2	2	0	2	2	0	2	2	0	0	2	0	0	0	2	2	2	2
**Vvi**	0	2	0	0	0	2	0	0	0	0	2	0	2	0	0	0	0	0	0	0	0	2	0	2	0	0
**Gar**	2	8	0	2	0	10	13	18	2	0	18	12	16	2	2	0	0	0	2	15	0	21	2	2	0	2
**Gba**	20	15	4	29	0	23	12	38	24	33	0	26	12	22	17	11	4	0	11	22	20	2	27	17	22	44
**Ghe**	2	0	0	2	0	2	9	14	2	2	19	0	5	0	2	0	0	0	2	11	0	3	2	2	0	0
**Ghi**	25	11	5	28	0	19	15	43	17	26	13	27	0	24	18	5	6	0	8	29	21	0	23	10	20	46
**Got**	0	0	0	0	0	0	0	2	0	2	0	2	0	0	0	0	0	0	0	0	0	0	0	0	0	2
**Gra**	2	2	0	0	0	0	2	2	0	2	0	0	0	0	0	0	2	0	0	2	2	0	0	2	0	0
**Nnu**	2	2	0	0	2	0	0	0	0	0	0	2	0	2	0	0	4	0	2	2	4	0	2	2	0	0
**OsR**	0	10	0	0	0	10	3	0	4	0	4	8	4	10	11	6	0	0	3	12	18	14	2	6	2	0
**Pab**	0	8	0	0	0	8	0	0	0	0	8	0	8	0	0	0	0	0	0	0	0	8	0	0	0	0
**Sot**	2	0	0	6	0	2	3	7	4	6	2	6	2	2	2	2	0	0	0	5	8	0	8	4	8	8
**Pru**	11	14	4	7	0	2	2	6	7	5	0	14	2	9	2	4	11	0	8	0	9	0	8	11	2	2
**Sme**	0	0	0	0	0	0	3	5	0	0	2	0	2	0	2	2	0	0	0	3	0	0	2	2	2	2
**Sob**	0	0	0	0	3	0	0	0	0	0	0	0	0	0	0	0	3	0	5	3	7	0	3	0	0	0
**Sol**	0	2	2	2	2	2	0	2	0	0	2	0	2	0	2	0	0	0	3	0	7	2	0	2	0	0
**Spi**	0	0	0	0	0	0	2	0	0	0	0	0	0	0	2	0	0	0	0	0	0	0	0	0	0	0
**Tca**	8	9	6	12	0	6	6	13	13	9	2	0	0	6	15	6	2	0	6	6	2	0	6	4	0	9
**Gtu**	4	2	0	2	0	2	0	5	2	0	6	0	0	0	2	0	2	0	2	2	2	0	2	4	0	0

Table S13: Orthologues many-to-many plant species

	Al	At	Aqu	Bpe	Bra	Car	Cic	Cuc	Vvi	Gar	Gba	Ghe	Ghi	Got	Gra	Nnu	OsR	Pab	Sot	Pru	Sme	Sob	Sol	Spi	Tca	Gtu
**Al**	0	0	4	2	0	0	6	0	0	0	2	0	2	0	0	0	4	0	0	4	0	0	0	2	2	0
**At**	0	0	0	0	0	0	0	0	0	0	0	0	0	0	0	0	0	0	0	0	0	0	0	0	0	0
**Aqu**	2	0	0	4	3	7	2	2	5	7	6	4	6	2	4	2	12	3	4	12	4	0	4	4	6	6
**Bpe**	2	0	4	0	0	3	2	0	0	2	2	2	2	0	2	0	4	0	2	0	2	0	0	0	0	0
**Bra**	0	0	2	0	0	5	0	0	2	2	0	0	0	0	0	0	4	2	0	0	0	0	2	0	0	0
**Car**	0	0	8	4	4	0	8	0	0	4	4	4	4	0	4	4	8	0	4	6	4	4	4	6	4	4
**Cic**	5	0	2	2	0	4	0	2	0	2	2	2	2	2	2	0	2	0	2	2	2	0	2	2	2	2
**Cuc**	0	0	4	0	0	0	4	0	0	0	2	0	0	0	0	0	4	0	0	2	0	0	0	2	0	0
**Vvi**	0	0	4	0	2	0	0	0	0	2	0	0	0	0	0	0	4	2	0	0	0	0	2	0	0	0
**Gar**	0	0	22	13	8	14	12	0	8	0	0	0	2	0	0	0	16	8	16	0	16	0	11	3	8	4
**Gba**	2	0	48	8	0	16	33	2	0	0	0	0	26	0	0	4	39	0	31	6	22	0	14	14	23	5
**Ghe**	0	0	8	9	0	21	6	0	0	0	0	0	11	0	0	0	8	0	12	0	12	0	3	3	13	13
**Ghi**	5	0	45	10	0	18	35	0	0	2	30	2	0	0	0	6	32	0	34	6	19	0	9	9	28	7
**Got**	0	0	5	0	0	0	5	0	0	0	0	0	0	0	0	0	2	0	2	0	2	0	2	2	2	0
**Gra**	0	0	4	2	0	3	2	0	0	0	0	0	0	0	0	2	0	0	2	2	2	0	0	0	0	0
**Nnu**	0	0	2	0	0	2	0	0	0	0	2	0	2	0	2	0	0	0	0	2	0	2	0	0	2	2
**OsR**	11	0	36	10	12	42	11	11	10	21	17	17	17	7	0	0	0	4	17	11	13	3	24	10	10	17
**Pab**	0	0	8	0	8	0	0	0	8	8	0	0	0	0	0	0	8	0	0	0	0	0	8	0	0	0
**Sot**	0	0	10	3	0	5	6	0	0	5	11	5	11	2	3	0	10	0	0	0	5	0	0	3	0	2
**Pru**	6	0	17	0	0	6	5	2	0	0	11	0	9	0	9	9	5	0	0	0	0	0	0	3	9	9
**Sme**	0	0	5	3	0	5	3	0	0	5	5	5	5	2	3	0	4	0	5	0	0	0	0	2	0	2
**Sob**	0	0	0	0	0	7	0	0	0	0	0	0	0	0	0	3	2	0	0	0	0	0	2	0	0	0
**Sol**	0	0	7	0	2	7	5	0	2	4	2	2	2	2	0	0	12	2	0	0	0	3	0	3	0	2
**Spi**	2	0	4	0	0	4	2	2	0	2	2	2	2	2	0	0	4	0	2	2	2	0	2	0	0	2
**Tca**	7	0	13	0	0	9	9	0	0	8	17	8	19	2	0	2	9	0	0	2	0	0	0	0	0	10
**Gtu**	0	0	15	0	0	3	11	0	0	6	2	4	6	0	0	2	8	0	3	2	3	0	3	3	9	0

Table S14 Duplications events per species-based phylogenetic tree nodes

Species tree node	Duplications (all)	Duplications (50% support)
Pab	7	7
OsR	40	40
Sob	3	3
Bra	2	2
Spi	2	2
Aqu	14	14
Nnu	2	2
Sme	3	3
Sot	7	7
Sol	4	4
Car	3	3
Al	1	1
At	0	0
Vvi	1	1
Bpe	1	1
Cuc	1	1
Pru	16	16
Tca	15	15
Cic	2	2
Gba	28	28
Gtu	4	4
Gra	1	1
Got	0	0
Ghi	31	31
Gar	19	19
Ghe	15	15
N0	23	5
N1	2	0
N10	47	5
N13	1	1
N14	1	1
N15	1	0
N20	1	0
N22	1	0
N4	1	0
N6	2	1

Table S15 List of reference genes, classes and thier ontologies

Domain architecture	Class	Gene Ontologies			
zf-BED--DUF-domain--Dimer_Tnp_hAT--	I	nucleic acid binding	Interacting selectively and non-covalently with any nucleic acid	protein dimerization activity	
zf-BED	II	nucleic acid binding	DNA binding		
zf-BED--zf-BED--DUF-domain--Dimer_Tnp_hAT--	III	nucleic acid binding	DNA binding	protein dimerization activity	
zf-BED--DUF-domain	IV	DNA binding	nucleic acid binding		
zf-BED--Dimer_Tnp_hAT--	V	nucleic acid binding	DNA binding	protein dimerization activity	
zf-BED--PHD--	VI	DNA binding			
zf-BED--zf-BED--	VII	DNA binding			
GRAS--zf-BED--DUF-domain--Dimer_Tnp_hAT--	VIII	Moecular	nucleic acid binding	DNA binding	protein dimerization activity
GRAS--zf-BED--DUF-domain--Dimer_Tnp_hAT--Peptidase_C48--	IX	Biological Process: proteolysis	cysteine-type peptidase activity	protein dimerization activity	nucleic acid binding
WRKY--zf-BED--DUF-domain--Dimer_Tnp_hAT--	X	DNA-binding transcription factor activity, sequence-specific DNA binding, DNA binding , protein dimerization activity, regulation of transcription regulation of transcription,			
zf-BED--NB-ARC--	XII	ADP binding, DNA binding			
GST_N_3--GST_C_3--zf-BED--	XIII	glutathione metabolic process, aromatic amino acid family metabolic process , protein binding, DNA binding, catalytic activity,			
zf-BED--zf-BED--DUF-domain--	XV	DNA binding			
WRKY--zf-BED--	XVI	regulation of transcription, DNA-binding transcription factor activity, seuquence specific DNA binding, DNA binding			
WRKY--zf-BED--	XVI	regulation of transcription, DNA binding, DNA binding transcription activity, sequence-specific DNA binding			
zf-BED--PPR-domain--	XVII	DNA binding and protein binding			
GRAS--zf-BED--	XVIII	DNA binding			
Myosin_TH1--zf-BED--DUF-domain--Dimer_Tnp_hAT--	XIX	DNA binding, motor activity, protein dimerization activity, and myosin complex			
zf-BED--Sina--	XX	ubiquitin-dependent protein catabolic process, multicellular organism development,			

		DNA binding<br>zinc ion binding<br>protein binding, and cytoplasm			
zf-BED--LRR-motif--	XXII	DNA binding			
DnaJ--zf-BED--zf-BED--DUF-domain--Dimer_Tnp_hAT--	XXII	DNA binding and protein dimerzation activity			
NAM--zf-BED--	XXII	regulation of transcription, DNA-templated and DNA binding			
GRAS--zf-BED--DUF-domain--Dimer_Tnp_hAT--Peptidase_C48--RWP-RK--PB1--	XXII	DNA binding, protein bindng, and protein dimerization activity			
Glyco_hydro_1--zf-BED--DUF-domain--Glyco_hydro_1--	XXII	DNA binding<br>hydrolase activity, hydrolyzing O-glycosyl compounds<br>carbohydrate metabolic process ,			
F-box--LRR-motif--zf-BED--	XXII	DNA binding,<br>protein binding			
RVT_3--zf-BED--DUF-domain--Dimer_Tnp_hAT--	XXII	RNA-DNA hybrid ribonuclease activity, nucleic acid binding, DNA binding, protein dimerization activity			
Glyco_hydro_79n--zf-BED--	XXII	hydrolase activity, DNA binding			
zf-BED--DUF-domain--Tubulin--	XXII	no GO terms			
zf-BED--zf-BED--zf-BED--zf-BED--	XXII	DNA binding			
zf-BED--zf-BED--zf-BED--DUF-domain--Dimer_Tnp_hAT--	XXII	protein dimerization activity, DNA binding			
zf-BED--DUF-domain--zf-BED--DUF-domain--Dimer_Tnp_hAT--	XXII	protein dimerization activity, DNA binding			
zf-BED--DUF-domain--Dimer_Tnp_hAT--PSII--	XXII	photosynthesis, light reaction	photosynthetic electron transport chain	photosystem	membrane, protein dimerization activity, nucleic acid binding, chlorophyll binding

Table S16 List of GrZF-BED genes and their features

Chromosome	Gene_ID	Start	End	Protein_sequence	Protein Length (aa)	Molecular Weight (kDa)	Charge	Isoelectric Point	Grand Average of Hydropathy
Gr7	Cotton_D_gene_10000873	47147766	47151161	MEVANETVIKKPKRLTSVVWNHFERVRKADLCYAVCVHCNKKLSGSSNSGTTHLRNHLMRCLKRFNYDVSQLLSAKKRKKESTLTIANISYDEGQRKEEYLKPTIVKYEPEQRKDEVFNVQSSWFDQDRSRLDLARMIILHGYPLAMVEHVGFKVFVKNLQPLFDVVPNSTVELSCMEIYGKERQKVHDMLSKLQGRINLAVEMWSSPENTNHVCMMAHYVGDDWKLQKKILNFVTLDSSHTDDLLSGVIIKCLMDWDIGSKLFAVTLDDFSTNDDIVLRIKEQISENKSRLSNGQLLDVRSAAHVLNSIVQDAMEALRVVLQKIRGTVRYVKSSQSIQGKFKEMVLQTGINSQKNLVLDCPIRWNSTYLMLEAAIEYRNAFCQLPDLDLDLALSDEEWEWASSITGYLKLFVEIINVFSSNKCPTANIYFPEICHVHIQLIDWCKSPDNFLSSLAAKMKAKFDKYWSKCSLSLAVAAILDPRFKMKLVEYYYSQIYGSTALERIKEVSDGLKELFNTYSICSTLMDQGSALPLGSLPSSSNDGRDRLKGFDKFLHETSQSQTAISDLEKYLDEPVFPRNCNFNILNWWRVHTPRYPILSMMARDVLGTPMSTVSQESAFHAGGRVLDSCRCPLTPETQQALICTQDWLRIQSDDPGPSSSHYALPLYVETN	672	76.846	8.5	7.469	-0.282
Gr4	Cotton_D_gene_10011977	11099244	11103492	MAEITEATNMETTPVENNNELALITPETQPKRRKKKSMVWEYFTIETVSAGCRRACCNRCKQSFAYSTGSKVAGTSHLKRHIAKGTCPALLRDQYNNQLTPYNPKTGGSEPRKRRYRSPSSPFIPFDQDRCRHEIARMIIMHEYPLHMVEHPGFIAFVQNLQPRFDKVSFNTVQGDCVATYLREKQSLMKLIEGIPGRVCLTLDMWTSNQTLGYVFITGHFIDFEWKLQSRVLNVIMEPYPDSDSALSHAVAACLSDWSLEGKLFSLTFNHPTSEAGLENLRPLLCTKNPLILNGQLLLGNCIARTLSSMAKDVLGAGHEIVKKIRDSVKYVKTSESHDEKFVQVKNQLQVPSEKSLILDNQTQWNTTYQMLAAGTELKEVFNCLDTSDPDYKLAPSIEDWKVAETLCTFLKPLFDAASILTTTTNPTAITFFHEAWKIHADLGRSITNEDPFISNIAKSMLEKIDKYWKDCSLILAIAVVMDPRFKMKLVEFSFTKIFGEDAPTYIKIVDDGIHELFLEYVALPLPLTPTYTEEGNAGNNGKTDESQQGNLLSDQGLTDFDVYIMETSSQQMKSELDQYLEESLLPRVQEFDVLGWWKLNKMKYPTLSKMARDILSIPVSAAAPDSVFDIIIKQLDEYRSSLRPETVEALICAKDWLHYGSEESNALVKMEF	673	76.346	-5.5	5.909	-0.304
Gr7	Cotton_D_gene_10017869	54829127	54833062	MEWSVNNAFKSYKDMEPKSTMDMVLIPNMDTIDIVLGSSEKGNVVPSAKPRKKTMTSVYLKYFETAPDGKTRRCKFCGQSYSIATATGNLGRHLSNRHPGYDKTGENVTSSAPQPSTTPTVIKKPQQQGRAPQVDYDHLNWLLIKWLILATLPPSTLEEKWLANSFKFLNPSIQLWPGEKYKAVFREVFRSMREDVRASLEQVSSKVSIALDFWTSYEQIFYMSITCQWIDENWSFRKVLLDICQVPYPCSDSEIYNSLVKVLKMYNIENKVLSCTHDNSQNAIHACHALKEDLDGQKMGPFCFIPCAARTLSLIIDDALRTTKPVIAKVREFVQELNASLDISEDFIQLTTAYKEGSWQFPLDASARWSGSYQMLDLVQKAGKSMDAVVRKNEEMLGNRMLLNTAEKNVVNIVHNYLEPFYKVISEICVNTPPTIGMVIVYMDHISDTITTRQPPDWLKNPAEDMAKKLRSYNNQVCNIFIYMTAILDPRIKCELIPESLNSENYLEEARAHFVRNYYTTPFSSMTSGYSSQDIEDGGAVSFAEEIARKKRRASMSNATDELTQYLSESPAPTKTDVLEWWKVNSTRYPRLSAMARDFLAVQATSVKPDELFCSKGDEIDKQRFCMPHDSTQAILCIKSWTQGGLKLKYKSTEIDYERLMEMAAAAAADISSAGIDKKQK	681	77.382	4	7.183	-0.388
Gr1	Cotton_D_gene_10023062	29849665	29853289	MELNLVPISITRQKQDPAWNHCEVFKNGERLQIKCMYCGKLFKGGGIHRFKEHLAGRKGQGPICEQVPQGVRSVMQESLNGILVKQDKKQKLIPKLLACGSSSSNPNIGGEVENLGSHDDMNFGIKPISVLNTLEGDSNVVSKVGRGRKRGRGRGRDWNLIESNYPCVKTDLALVPNGGENPIHMAIGRFLYDIGVNLDAVNSVCFQPMIDGIASGGSGVVPPSCNDLRGWILKNVIEEVKDDIDRNKAMWGKTGCSIIVEQCRTKNGRVLLSFLVYCPQATVFMKSVDASHAVYSADYLFELLKQVIEEVGSEKVVQVITNCEEPYLFTGKRLMESFPSLYWAPCLAHCVDLMLQDFSNLEWINETIEQAKSLTRFIYNQSSVLNMIRKFTSGNDVVEPALTCFATNFSTLKRMADLKLNLQAMVNSQDWLECPYAKKPGGQAMSDIVNNSSFWNSCMLIARITYPLLRVLEIVGSKKRSAMGYVYAGIYRAKETIKKELVKQDDYMVYWNIIDNRWEQQRHLPLYAAGFFLNPKLFYNTEEHIHNDILSSVFDSIERLVPDTNIQDQVVREINLYKNATGDLGRPMAVRARDNLLPGEWWSIYGGGCPNLQRLAIRILSQTCSSIGYKPSKISIEEIHNTRNFLERQRLSDLVFVQYNLYLRQMVLQKQEKDSLDPLAFNNKDILEDWIADTEVSPDNLESSDWKSLDPPVGNRTTLTPPSDEAEDFLSTRFTDLDIFNGLKGVKEEI	750	84.753	1.5	6.717	-0.296
Gr1	Cotton_D_gene_10023084	189964	194599	MDNFDQKLGPEFFKNLSAEAVTPLNVVHEEIYESSSKRPKTTSKVWDIFEKLPAQQGDSKAICKLCRRIYTAKTTSGTSHLRRHIEACVKRGNHEVDQRSIEACFKPVKRNANRLTLSHDTLIAATTSLKNYKLDVDEIHRAIAMMIIVDEQPFSVVEDAGFRRLLSAACPEFPVLSRSSIKRDIISIYVKERENIRELLATCPGRICLTSSTWKSDSDDHFNCVTTHFIDHEWRLQKRILSFKLMPPPYDSLSVADEIALCMVQWNIEHKVFSVTLENLSSDDCVADMLRSRLAAKKYLPCKGVFFHVSCFFRILNSIVQAGLNLVVDIIAKLRLGIKYVQQSPHRKKNFYIIAKTLNLDTQRKLCLDTPARWNSTYNMIEVAFCYNNAFMYLAEQDKNFLHKLSEDEWEKLSVLYKFLKVFYEVTCVFFRNRQPTSNLYFKAAWKVHSRLFDMVRGPENFMTRMVREMHSKLNQYWSAYNLILSCAAILDPRCKIKFVEYCYTKLYGSGAQKYVSVSVNTLYGLFDEYMQNSARPSQTTLLSTAASKISNDKDNNDGFEDY	691	79.517	10	7.697	-0.325

				ETFQSARFRTQVEKSQLDLYLEEPSHDLNSEIDVLEYWTLCSLRYPELSKMARDVLTIPVSTIASDSAFDISPQVISTDRSSLKPKMLQALVCLQDWMLASDRTRGLGSMESKPEDDSSSSSDGDDDY					
Gr 1	Cotton_D_gene_10023113	4233318	4255888	MSTEPTSIKGSVTPPTSIDSENSGVGASSQTKGTTGKEKTAPQRLEVWSHFTKIINSEGASKAKCNYCQKEFCCDMKKNGTGSLKYHIGSCKKNPSNVQGQLVLPRKRVEGGGRKFFNLEI	121	13.1	10	10.042	-0.748
Gr 13	Cotton_D_gene_10024621	41591701	41595156	MSSNLEPIPITSQKHDPAWKHCQMFKNGERVQLKCIYCGKIFKGGGIHRIKEHLAGHKGNAATCLRVPSDVRVLMQESLDGVVVKKRKKQKIAEEITNVNQVSTEIQAYADQVDTNTGLLMIEKSDTLEPSSSLLVNQEGTSNVAGERRKRGRGKSLPAEANALSFVPVELGARRVNNHVHMAIGRFLFDIGATMDAVNSVYFQPMVDAIVSGGSGALMPSCNDLQGWILRKLVEEVKSENDKVMAAWVRTGCSILVNQWNTQTGRILLNFLVYCPEGTVFLKPIDASSVINSSDALYELLKQVVEEVGSKHVLQVITNGEEQYIVAGRRLVETFPTLYWAPCAAHCVDLILEDFAKLEWINAIIEQARSITKFIYNHSVVLNMVRRYTFGNDIVEPAATRSATNFTTLTRMVDLKNNLQAMVTSQQWVDCPYSKKPGGLAMLDLVSNQSFWSSCVLIVRLTNPLLRVLRMVGSKKRPAMGYVYAGMYRAKETIKKELVKRNEYMVYWNIIDHWWEQQWHHPLHAAGFYLNPRFFYSMEGDMPNEMLSGMLDCIEKLIPDVTVQDKITKEINSYKNSVGDFGRKMAVRARDTLLPVEWWSTYGGSCPNLARLAIRVLSQTCSTLGLKHDHIPFEKLHETRNCLEQQRLRDLIFVQCNLQLRQIGYESKQHDSMQPLSSESASIVEDWVTGIDAFLDDDTYPDWTTLETLSVNTMPLRPGDEVEELGAGNFVSWIW	735	83.079	7	7.227	-0.212
Gr 9	Cotton_D_gene_10035444	41067388	41070709	MVEEMAPLRSIGYVDPGWEHGTAQDERKKKVKCNYCGKVVSGGIFRLKQHLARLSGEVTHCEKVPEEVCLNMRKNLEGCRSGRKRRQLDYEQAALSIQSNEYSDGEDASASYKHKGKKVMGDKNLVIKFTPLRSLGYVDPGWEHCVAQDEKKRRVKCNYCEKIISGGINRFKQHLARIPGEVAYCEKAPEEVYLKIKENMKWHRTGRRHRKPDTKEISTFYMHSDNEDEGGEEEGYLQCISKDILAIDDKVSDNDIRNNVRGRSPGSSGNGAEPLLKKSRLDSVFLKSLKSQTSAHYKQPRARTGFEKKTHREVISAICKFFYHAGIPSNAANSPYFHKMLELVGQYGQGLQGPSSRLISGRLLQEEIANIKEYLVELKTSWAITGCSVMADSWNDAQGRMLINFLVSCPRGVYFLSSVDATDIIEDAVHLFKLLDKAVDEVGEEYVVQVITRNTLSFRNAGKMLEEKRRNLFWTPCAVYCIDRMLEDFVNIKWVGECVDKAKKVTRFIYNNTWLLNFMKKEFTKGQELLQPAVTKFGTNFFTLQSLLDQRVGLKRMFQSNRWLSSRFSKSDEGKEVEKIVLNVSFWKKMQYVKKSFEPVAEVLQRIGSDKIRSLPFIYNDICRTKLAIKAIHGDDVRKYGPFWSVIESNWSPLFHHPLYVAAYFLNPSYRYRPDFLMNPEVIRGLNGCIVRLEADNGKKIAASMQIPDFVSAKADFGTDLAISTRSELDPASWWQQHGISCLELQRIAIRILSQTCSSIGCEHNWSAFDQVHIKRHNCLSRKRLNDQTYVHYNLRLRERQLGRKPDELVSFDSAMLESVLDDWLVETEKLAMHEDEEIIYTEVEQFCGDDMDEHESEEKRPAEMVTIAGFIEPLDVIPSAGGVTTDDDGLDFLDDDLTD	900	103.125	12	7.495	-0.505
Gr 6	Cotton_D_gene_10036775	40594489	40596511	MSTEPTSIEGSVIPPTSIDSKNSGVGASSQTKGTTGKRKVAPQRSEVWSHFTKIINSQGASKAKCNYCQKKFCCDMKKWYRVIEISYWFM	90	10.074	8.5	10.061	-0.578

Table S17 List of GaZF-BED genes and their features

Chromosome	Gene_ID	Start	End	Protein_sequence	Protein Length (aa)	Molecular Weight (kDa)	Charge	Isoelectric Point	Grand Average of Hydropathy
Ga3	Ga03G2181	129461115	129464068	MSIEPTSIEGSVTPPTSIDSENSGVGASSQANVTTGKRKATPQRLEVWSHFTKIINNEGASKAKCNYCQKEFCCDVKKNGTGSLKYHIGSCKKNPSNVVDPSQGQLVLPRKGVEGGEGHLSTWRFDQDACRKGLAQMIVIDELPFKFVESEVFKKFMFVACLKFHIPSRITMIRDVYQLYLDERVKIKQLLRSSFNYLCITAHFIDNDWKLNKNILNFCPISSHKGESIRMVIEKCLLNWGIDKLFTVTVDNASSNDVVIGYLRKIFNPRWGLVQNGKYLHMRCMAHIVNLIVVEGLKEMNKFVERVRGAARYVRQSPARLQKFKECVVVEKIECKKMLCLDVCTRWNSTYLMLDTAQNFERAFERFEEQDTNFRAELERRKGWPSVDDWTIVRDLRDFLEHFYELSEIDILLRDAQLNSNVDFNVMAIKMKEKYDKYWGDIDKMNLLMFVGCVLDPRQKLKYLEFALSEMSSSEKASEMMQKLKESLYELFDEYKPSLHSICSQSSVSTHVSFSEPQQKMKRRMQALYKKRELEICGEDKTSELDKYLAEANEEFVEDFDILLWWKVNSPRFPTLSKMARDVLAIPVSTVVSESSLTPKIVQALVCTQDWIENHHHKKTSKRLKNKSKNKNIAT	635	73.483	20	8.681	-0.411
Ga8	Ga08G2756	127731453	127733564	MEVANETVIKKPKRLTSVVWNHFERVRKADLCYAVCVHCNKKLSGSSNSGTTHLRNHLMRCLKRFNYDVSQLLSAKKRKKDNTLTIANISYDEGQRKEEYLKPTIVKYEPEQRKDEVFNVQSSWFDQERSRLDLARMIILHGYPLAMVEHVGFKVFVKNLQPLFDVVPNSTVELSCMEIYGKERQKVHDMLSKLQGRINLAVEMWSSPENTNHVCMMAHYVGDDWKLQKKILNFVTLDSSHTNDLLSGVIIKCLMDWDIGSKLFAVTLDDFSTNDDIVLRIKEQISENKSRLSNGQLLDVRSAAHVLNSIVQDAMEALRLVIQKIRGTVRYVKSSQSIQGKFKEMVLQTGINSQKNLVLDCPIQWNSTYLMLETAIEYRNAFCQLPELDLDLALSDEEWEWASSITGYLKLFIEIINVFSSNKCPTANIYFPEICHVHIQLIDWCKSPDNFLSSLAAKMKAKFDKYWSKCSLSLAVAAILDPRFKMKLVEYYYSQIYGSTALERIKEVSDGLKELFNTYSICSTLMDQGSALPLSSLPSSSNDGRDRLKGFDKFLHETSQSQTAISDLEKYLDEPVFPRNCNFNILNWWRVHTPRYPILSMMARDVLGTPMSTVSQESAFHAGGRVLDSCRCPLTPETRQALICTQDWLRMQSDDTGPSSSHYALPLYVETN	672	76.996	9.5	7.609	-0.292
Ga11	Ga11G4055	123821312	123824877	MEWSVNNAFKSYKDMEPKSTMDMVLIPNMDTIDIVLGSSEKGNVVPSAKPRKKTMTSVYLKYFETAPDGKTRRCKFCGQSYSIATATGNLGRHLSNRHPGYDKTGENVSSSAPQPSTTPTVIKKPQPQGRAPQVDYDHLNWLLIKWLILATLPPSTLEEKWLANSFKFLNPSIQLWPGEKYKAVFREVFRSMREDVRASLEQVSSKVSIALDFWSSYEQIFYMSITCQWIDENWSFQKVLLDICQVPYPCTGSEIYNSLVKVLKMYNIENKVLSCTHDNSQNAIHACHALKEDLDGQKMGPFCFIPCAARTLSLIIDDALRTTKPVIAKVREFVQELNASLDISEDFIQLATAYKEGSWQFPLDASARWSGSYQMLDIVQKAGKSMDAVVRKNEEMLGNRMLLNTAEKNVVNIVHNYLEPFYKVISEICVNTPPTIGMVIVYMDHISDTITTRQPPDWLKNPAEDMAKKLRSYNNQVCNIFIHMTAILDPRIKCELIPESLNSENYLEEARAHFVRNYYTTPFSSMTSGYSSQDIEDGGAVSFAEEIARKKRRASMSNATDELTQYLSESPAPTKTDVLEWWKVNSTRYPRLSAMARDFLAVQATSVKPDELFCSKGDEIDKQRFCMPHDSTQAILCIKSWTQGGLKLKYKSTEIDYERLMEMAAAAAADISLAGMDKKQK	681	77.239	4.5	7.215	-0.375
Ga11	Ga11G3315	117624397	117626751	MSTEPTSIEGNITPPTSIDSENSGIGASSQAKGTIGKRKATPQRSEVWSHFTKIINSEGASKAKCNYCQKEFCCDMKKNGTGSLKYHIGSCKKNSSNVVDTSQGQLVLPRKRVEGGGGHLSTWRFDQEACRKGLAQMIVIDELPFKFVESEGFKKFMFVACPRFHIPSRTTVTRDVYQLYLDKKVKIKQLLKSSCSRVCLSTDTWTSLQIVNYLCITAHFIDNDWKLNKKILNFCPISSHKGESIGMVFEKCLLNWGIDKLFTVTVDNASSNDVAIGYLRKKFNPRGGLVQNDKYLHMRCMTHIVNLIVVEGLKEMNKSVEPRLQKFKECVVLEKIEWNSTYLMLDTAQNFERAFERFEEQDTNFRAELEKGEGWPSVDDWDNVRNLRDFLEHFYEVTLHISGTSYLNSNVDFNVMVIKMKDKCDKYWGDIDKMNLLMFVACVLDPRQKLKYLEFALSEMSRSEKACKMMQKLKESLYELFDEYKPPLHTLYKKRELEICGEDKTSELDKYLAEANEEFVEDFNILLWWKVNSPRFPTLSKMAKCHRGRVLDQYRSTLTPKIVQALVWTQDWIRKSLSQEDIKKIEEQIQELDKIENGLMQMEIFWREETDTNGEY	616	71.448	9	7.817	-0.499
Ga1	Ga01G2645	110642070	110644889	MLRALAVSCFVLKIMVRGRDACWEHCVLVDATRQKVRCNYCHREFSGGVYRMKFHLAQIKNKDIVPCAEVPDDVRDHIQSILNTPKKQKTPKKPKMDKTVANGQQNSSSASGGLHPNHGSSGQHGSTCPSLLFPHPSPSEQPATDDAQKQKLDDADKKIAVFFFHNSIPFSAAKSMYYQEMVDAIAECGVGYKAPSYEKLRSSLLEKVKGDIHDGYKKYREEWKETGCTVLCNSWSDGRTKSFVIFSVTYPKGTLFLKSVDVSGHEDDASYLFELLESVVLEVGLENVIQVITDSTASYVCAGRHLMAKYSSLFWSPCASYCIDKMLEDISKQEWVGIVLEEAKTIARYIYSHAWILNMMRKFTGGRELMRPRITRFVDNYLTLRSIVFQEDNLKHMFSHSEWLSSIYSRRSDAQAIKSLLYLERFWKSAREAVSVSESLVKILRIVDGDMPAMGYIYEGIERAKGAIKAYYKGIEEKYMPIWDIIDRRWNMQLHSPLHAAAAFLNPSIFYNPNFKIDLRMRNGFQEAMLKMATMDKDKIEITKEHPVYINAQGALGTDFAIMGRTLNAPGDWWASYGYEIPTLQRVAIRILSQPCSFHWCRWNWSTFETGRDGKCKPIIFDEIDVSSEWPTESEPSVPLLDDSWLDNLPLECRGSP	657	74.967	11.5	7.772	-0.342
Ga8	Ga08G1	103418	103425	MSPREEFPTRGLEGAPSNDIGWHFGTPVPNAKGNIVCKLCGKVLKGGITRFKKHIAHKIGCVIRESMMNILKESNMKKIDKKRRKDEFLSQLREEEDKHEEFIDEVSAIRQATRESIQSQHEWHRREEFRRSTGGWNNMYEEGRSSHGSAREYHRERRSKSISSESEFTLRGAIPELVRSKSSKQPKVNDSFLKSFRRKIAEAVSKFLIYEILHFQLASSLWLYNLIQVSIEVGQGVKLPTPYEVSDVYLESDLNQMHIINFRIYCSKGTIFWKSVDVSSVRNKDAEFYYSLLDSVVEEIGENYILLEKKLILKRKHLFWTSCAAYCLDLCLEDIGKKPVTRFIYNHISTVDLMKKYTQGKQILQPALTRFATHFIQLEEIIRQKQGLIEMFNSKEFKESKWGQQKSGPAYKAKKIPLVKVLRLVDSDEKPTIAIQQDCRYFIEYEKIIDNRWNFMQSDLHSAGYFLNPQFQFGVEYSENVLIETLEGTRS	872	100.6	20	8.555	-0.753

	539	251	072	VIERLEPSLDTQVRMVNQWLLFRDKHETFGTPQAQRAWKQMNLTEWWIIYDTCVPELQKLAIKVLSQTTSASNCERNWSTFSYIHTKARNRLKYKKLEKLIRHQKTMSTDDINVSFNPISLDHIFEDKENPLLDGENAGVLLVDTSDDEMDVNQSQQQNLSHSSSSSTPSRSGDRPDGGGLSPIDEDDGYSVDRGEIRSSSQYGGEYGVGTAIGHFRDRSEFDGNMFPKPRRDRSEPRAPSKGKGKKHTSIGSSSGRRSSSSNLGYSDSSTSTQGFYPPEQPSYFQPSHGYPQPYGYYPPFPNYGVSYQPQMHPPPPMYHPPPPLIYPPPQIYPSYQLYENQGENVTFFGYIFGQRPRESNQKRSQSEGEGSDLPHHSTNW					
Ga13	Ga13G1499	95084810	95089972	MPPREEFPTKGLEGAPSNDIGWHFGTPVPNAKGNIVCKLCGKVVKGGITRFKEHIAHKTGNVAPCPNVTGVIRESMMNVLKENNTKKIDKKRRKDDFLSQLREEEDEHEEFIDEISAIRQATRESIQSQHECHRREEFRRSTGGWDNIYEKGRSSQGSSIPTESEFTLRGTIPELVRSKSSKQLKVSDSFLKSFRKKIGEAVSKFLIYERLPFQLASSPWLYNLIQVSTEVGQGVKLPTPYETHWKELGATLMCDGWTNSLNQMHIINFLVYCSKGTIFWKSVDVSSVRSRDAEFYYSLLDSVVEEIGENYIVQIVTDNEAAMKAAGKKLMLKREHLYWTSCAAHCLDLCLEDIGKRPSVAKVLDEAKKVTCFIYNHTWTVDLMKKHTQGKQILRPALTRFATHFIQLEEITRQNKEYKESKWGQQKSGPAYEAKKIILGKDFWKKSNDLIKVYEPLVKVLRLVDNDEKPTMGFIYEAVDRAKRAIQQDCRYFTEYEKIIDKRCNFMHSDLHSAGYFLNPQFQFGVEHSHNVLIETLEGTRSVIERLEPSLDTQVRMVNQLLLFRDKHETFGTPQAQRAWKQMNPDVDPLSEWLHEKENPLLDGENTGSGDGPDGGGLSPVDEDDGYNGDRGEIRSSSKYGGEYGVGTTSRHFRDRSEFDGNMFPEPRRDRSEPRAPSKGKGKKHTSIGSSSGRRSGSSNLGYSDSSTSTQGFYPPEQPSYFQPSHGYPQPYGYYPPFPNYGVPYQLQMHPPPPMYHPPPPLI	763	86.995	15	8.042	-0.734
Ga12	Ga12G2516	93757254	93764397	MCLIIFFTEKKQVKRTRTSETINQFRPISLCNVLYKIITKTIVIHLRQAMKTLVKQNQSRITRFKEHIAHKTGNVAPCPNVTGVIRESMMNVLKESNTKKIDKKRRKYDFLSQLREEEDEHEEFIDEISAIRQATRESIQLQHEWHRREEFRRSTGGWDNIYEKRRSSQGSSIPIESEFTLRGAIPELVRSKTSKQPKKIGEAASKFLIYERLPFQLASSPWLYNLIQVSTEVRQGVKLPTPYEVSDVYLESEYQRVPNWVNGLKTHWKELGATLMCDGWTNSLNQMHIINFLFYCSKGTIFWKSIYVSNVRSRDAQFYYSLLDSVVEEIGENYIVQIVTDNEAAMKTAGTKLMLKRKHLYWTSCTAHCLDLYLEDIGKRPSVTKVLNEAKKVTCFIYNHIWTVDLMKKYTQGKQILRPALTRFATHFIQLDKITRQKQGLREMFSSNEYKESKWGQQKSGPTYEAKKIILGKDFWKKANDLIKVYEPLVKVLILVDSDEKPTIGFIYEAVDRAKRAIQQDCRYFTEYKKIIDKRWNFMHFDLHSAGYFLNPQFQFGVEHSQNVLIETLEGIRSVIERLEPSLDTQVRMVNQLLLFRDKHETFGTLQAQRAWKQMNLAEWWIIYGTCVPELQKLAIKVLSQTTSASNCERNWSTFSYIHTKARNRLKYKKLEKLVFTYYNMRLQIRHQKRMSTDDINTSFNPISLDYIFEDVDPLSKWLHEKENSLLDGENAGMLPVDTSDDEMDVNQSQQPNLSHSSSSSTPSQSGDGPDGGGLSPVDEDDGYSSDRGEIRSSSQYGREYGVGTTSGHFRDRSEFDGNMFPEPRRDRSEPRAPTKGKGKKHTSIGSSSGRRSSSSNLGYSDSSTSTQGFYPPEQPSYFQPSHGYPQPYGYYPPFPNYGVLYQPQMHPPPPMYHPPPPLMYPPPQIYPPHQLYENQCENFTFFGYIFGQRLRESSQERFQSKGEGSDLPRHSTNW	975	113.311	32.5	9.262	-0.702
Ga4	Ga04G1716	92039516	92041492	MDMSDAVIVNSSRLKSIVWNDFDRVKKGDTFVAICRHCKKKLSGSSTSGTSHLRNHLIRCQRRSNHGVAQYFSAKDKKKEGSLALVTIDQEQKNDEVLSIVNLRYEQEQIKSEHVGIGSNSLDQRRSQFDLARMIILHNYPLAMVEHVGFKIFVRNLQPLFELATRNKVEADCMEIYAKEKQKVYEIFDKLPGKISVSADVWTASEDDAAYLSLAAHYIDENWQLKKKNLNFVTIDPSYTEDMHSEVIMNCLMDWDIDRKLFSMIFDSFTSDNIVERIRDRLSQNRFLYCNGQLFDVRCAVDLLNRMAHDALEALCEITQKIRESIRYVKSSEATQATFNELADEVQVETKKCLCIDNPLKWNSTYFMLEAALEYRKVFSCLRDRDPVNMKFLLSDPEWDRLITVTSFLKLFVEVTNVFTRSKYPTANIFFPEICDIHLQLIEWCKNPDEYISSLALKMRRKFEEYCYKCSSGLAVAAMLDPRFKMKLLEYYYPQLYGDSATELIDDVFECIKSLYNEHSIVSPLASSIDQGLDWQASGISGSGKDSRDRLMGFDKYLHETCQAEGSSSDLDKYLEEPLFPRNVDFNVLNWWKVHTPRYPILSMMARNILGIPISKVAAESRFDTGGRVLDHNWSSLPPTTIQALMCSQDWIRSELES	658	76.053	-4.5	6.032	-0.339
Ga4	Ga04G1537	87547213	87550655	MPPREEFPTKGLEGAPSNDIGWHFGTPVPNVKGNIVCKLCGKVVKGRITRFKEHIADKTGNVAPCPNVTSVIRESMMNVLKENNTKKIDKKRRKDDFLSQLREEEDEHEEFIDEISAIRQATRESIQSQHECHRREEFRRVLVKQKFKATKGQLPFQLASSPWLYNLIQVSTEVGQGVKLPTPYEVSDVYLESEYQRVHNWVNGLKTHWKELGATLMCDGWTNSLNQMHIINFLVYCSKGTIFWKSVDVSSVHSRDAEFYYSSNESCWKKLMLKREHLYWTSCVAHCLDLCLEDIGKRPSVAKVLDEAKKVTCFIYNHTWTVDLMKKYTQGKQILRPALTRFATHFIQLEEITRQKQGLREMFSSKEYKESKWGQQKSGPAYEAKKIILGKDFWKKANDLIKVYEPLVKVLRLAVDRAKRAIQQDCRYFTEYEKIIDKRWNFMHSDLHSAGYFLNPQFQFGVEHSHNVLIETLEGTRSVIERLEPSLDTQVRMVNQARNRLKYKKLEKLVFTYYNMRLQIRHQKRMSTDDINTSFNPISLDYIFEDSGDGPDGGGLSPVDEDDGYNGDRGEIRSSSQYRGEYGVGTTSRHFRDRSEFDGNMFPEPRRDRSEPRAPSKGKGKKHTSIGSSSGRRSGSSNLGYSDSSTSTQGFYPPEQPSYFQPSHGYPQPYGYYPPFPNYGVPYQPQMHPLPPMYHPPPPLMYPPPKYILHINYMKTNVKMLLFLDIFLDKGQENQVKNALKVKVMDLIFLVILLIGEN	758	87.796	30	9.298	-0.685
Ga12	Ga12G2323	84672933	84691458	MPPCEEFPTKGLEGAPSNDIGWHFGTPVPNTKGNIVCKLCGKVMKGGITRFKKHIAHKTGNVAPCPNVTGVIRESMMNILKESKTKKIDKKRRKDEFLSQLREDEDKHEKFIDEVFAIRQATRESIQSQHEWHRREEFRRSTGERISKSILSESEFTLRGVIPELARSKSSKQPKVSDSILKTFRKKIGEAVSKLLIYERLPFQLASSPWLYNLIQVSTEVGQGVKLPTPYEISDVYLESEYQRVRDWVNGLKTHWKELGVTLMCDGWTNSLNQIHIINFLVYCNASSVRSRDVEFYYRLLDSVEAMKAAGKKLMLKRKHLYWTLLNMTPIFTHCLDLCLEDIGKKPSVAKVLDEAKKVTCFIYNHIWTVDLMKKYTQGKQILRPALTRFATHFIQLEEITRQKQGLREMFNSKEFKESKWGQQKLGPAYEAKKIVLGKDFWKKANDLIKVYEPLVKVLRLVDSDEKPTMSFIYEAVDRAKRAIQQDCRYFIEYEKIIDNRWNFIHSDLHLADYFLNPQFQFGVEHSENVLIEILEGTRSIIERLEPSLNTQLRMVNQLLLFRDKHETFGTPQAQRAWKQMNPAEWWIIYGTNVPELQKLAIKVLSQTTSASNYERNWSTFSYIHTKARNRLKYKKLEKLVFTYYNMRLQRRHQKRMSTDDINASFNPISLDHIFEDVDPLSEWLHEKENPLLDGENTGVLPVDTSDNEMDVDQSQQQNLSHSSSSSTPSQSGDGPDGNGLSPIDEDDGYSGDRGEIRSSSRYGREYGVGTTSGHFCDRSEFDGNMFPEPRRDRSKKHTSIGFSSGRRSSSSNLGYNDSSTSTQGFYPPEQPSYFQPSHGYPQPYGYYPPFPNYGVPYQPQMHPPPPMYHPHPPLI	876	101.345	26.5	9.02	-0.689
Ga4	Ga04G1387	83573290	83575913	MSTKPTFIKGSVTPPTLIDSENSGVRASSQTKGATGKRKVAPQRSEVWSHFTKIINSEGASKAKCNYCQKEFCCDMKKNGTGSLKYHIGSCKKNPSNVVDTSQGQIVLPRKGVERGEGHLSTWRFDQEACRKGLAQMIVIDELPFKFVESKGFNKFMVVACPRSSCSRVCLTTDTWTFLQRVNYLCITAHFIDNDWKLNKKILNFCPISSHKGESIGMLFTVTVDNASSNDVAIGYLRKKFNPRGGLVQNGKYLHMRCMAHIVNLIVVEGLKEMNKSVEHVRGVVRYVRQSPARLQKFKECVVVEKIKCKKMLCLDVCTRWNSTYLMLDTAQNFERAFERFEEQDTNFRAELERGEGWPSVDDWANVRDLRDFLKHFYEVTLRISGTSYVTPNNFFDELSKIDILLRDAQLNSNVDFNVMAIKMKEKYDKYWGDIDKMNLLMFVACVLDPRQKLKYLEFAHSEMSSSEKAFEMIQNLKESLYELFDDVPTHVSLGEPQQKMKRRMQALYKKRELEICGEDKTSELDKYLAEANEEFVEDFDILLWWKVNSPRFPALSKMARDVLAIPVSTVALESSLTPKIVQALVCTQDWIQKSSSQEDIKKIEEQIQELDKIENDLCNVFNYVMIQIWVIWVIRLIRIIRVIRVIRLIRLIFNQK	657	75.914	20.5	8.783	-0.325
Ga9	Ga09G	78346	78356	MSRYRAQQQASVDEPEPNPQGEDHVPDDEQQDENDKDSQPSEPKGDYLNVASRPFLMKILQKQGDSKVLFADKVLKFTASGKMKRRNLIITDFAVYIVDPETDGLKRRIALAAVDKMCLSDLNDNFFSIIIPTEYDLLMASTRKTEIATCLFEAIKTSAQYELEVSFSSSFEYNATADLVKEIQFEEVEGVNPCNKVRDDVTDRVRAIISSKEDIKEIPSVKKQKIAEVRAPGNMSTGSKISPLETLSPAAKVFPTVLSIAASTLSDQETVERSIALFFFENKLDFSVARSSSYQAMIDAVGKFGPGLIAPSVETLKTTWLKRIKSEVTLHLKDAEKEWATTGCTIIADTWTDNKSKALINFLVSSPSRTFFHKSVDASSYFKNTKCLADLFDSVIQDFGQENVVQIIMDSSFNYTGISSHI	821	92.8	-12	5.154	-0.309

	2053	843	817	LQNYGTIFLSPCASQCLNLILEEFSRVDWVNRCILQAQTVSKFLYNNASMLDLMKKFTGGQELIRTGITKSVSCFLSLQSTLKQRSRLKHMFNSPEYSTNSSYANKPQSISCIAIVEDNDFWRAVEECVAISEPFLKVLREVSGGKPAVGSIYELMTRAKESIRTYYIMDESKCKTFLDIVDRQWRDQLHSPLHSAGAFLNPSIQYNPEVKFLGSIKEDFFKVLEKLLPTPELRRDITNQIFTFTRAKGMFACNLAMEARDTVSPGLWWEQFGDSAPVLQRVAIRILSQVCSTFTFERHWSTFQQIHTEKRNKIDKETLTDVVYINYNLKLAREMKTMPTDSDPIQFDDIDMTSEWVEESENPSPTQWLDRFGSALDGGDLNTRQFSAAMFGNDHIFGL		29			
Ga11	Ga11G17702	76598915	76605560	MESTSGSTTNLNNHLKICLKKPRGNTSNPKQSELSFVKVSQETTDLSTWVFDKNAIRKALVRMIIVDELPFKIVEGEGFKYFLSIACPRFSLTSRWTIRRDCLDLFNSMKSMMKNYFEKDISRVCLTTDTWTSLQRISYMVLTAHWVDDEWRLQKRIINFCPISAHRGEKSIGQAIEKCLQDWGIERAFNITVDNASANSVAIEYLRKKLNHRNASVANGKFIHMRCVAHILNLIVQYGIKDASVSVDRVRGAVRYIRASPSRLTKSNQRVKEEMIDSKAQLCLDVPTRWNSTYIMLKVAEKYERAFESYLRDDHNFFLDLTAGDGVPTFDDWDIVRRVIKVLEPFCHLTLKVSGSLHVTSHSLFEVLTDVHCLFYGWQDCGDLEIISMTSKMREKYNKYWGEGNKINMLVYLAVIFDPRCKMSFFDFVVNLLFPNVANDIMKMIDKELHCLFNEYSSNAERIQLFEALAKQKYLKKKKQVGLESKSELDRYLGEDKEVNNSSSFDLLLWWKMNSPRFPILAQMARDILATPISTVASESAFSTGGHVLDSFKSSLTPLMVEALVCTQDWLRKSNDAINLEDYVDELQTMEDGNT	595	68.55	10.5	8.081	-0.258
Ga1	Ga01G1640	73240389	73245477	MPPCEEFPTKGLEGAPSNDIGWHFGTPVPNTKGNIVCKLCGKVVKGGITRFKEHIAHKTGNVATCPNVTGIKYDEYIKESKTKKIDKKRRKDEFLSQLREDEDEHEEFIDEVSAIRQATQESIQSQHEWHRREEFRRSTGAREYHRERTSKSIPSESEFTLEEAIPELARSKSSKQPKKIGEAVSKLLIYERLPFQLASSPWLYNLIQVSTEVGQGVKLPTPYEISDVYLESEYQRVRDWVNGLKTHWKELGATLMCDGWTNSLNQMHIINFLVYCNASSVRSRDAEFYYSLLDLVVEEIGESYIVQIVTNNEAAMKAAGKKLMLKRKHLYWTSCAAHYLDLCLEDIGKKPSVAKVLDEAKKVTCFIYNHIWTVDLMKKYTQGKQILRPALTRFATHFIQLEEITRQKQGLREMFNSKEFKESKWGQQKSRPAYEAKKIWKKTNDLIKVYEPLVKVLRLVDSDEKPTMGFIYEAVDRAKRAIQQDCRYFTEYEKIIDNRWNFMHSDLHSAGYFLNPQFQFGVEHSENVLIETLEGTRSVIERLEPSLDTQVRMVNQVRFKYYYLLLTIKVLSQTTSASNCERNWSTFSYIHTKERNRLKYKKLEKLVFTYYNMRLQIRHQKRMSTDDINASFNPISLDHIFEDVDPLSEWLHEKENPLLDGENAGVLPVDTSDNEMDVDQSQQQNLSHSSSNSTPSQSGDGPDVGGLSPIDEDDGYSGDRGEIRSSSQYGGEYRVGTTSGHFRDRSEFDGNMFPEPRRDRSEPSVPSKGKGKKHTSIGSSSSRRSSSSNLGYSDSSTSTQGFYPPEQPSYFQPSYGVPYQPQIYPPPPMYHPPPPLI	837	96.344	10	7.369	-0.745
Ga9	Ga09G0974	61840791	61841186	MPPREEFPTKGLEGAISNDIGWHFGTPMPNPKGNIVCKLCVKVVKGGITRFKEHIAHKTSNVAPCPNVTSVIRESMTNILKESNTKKIDKKEEKR	95	10.584	8.5	10.285	-0.625
Ga8	Ga08G1068	48506008	48507918	MVWEYFTIETVSAGCRRACCNRCKQSFAYSTGSKVAGTSHLKRHIAKGTCPALLRDQYNNQLTPYSPKTGGGEPRKRRYRSPSSPFIPFDQDRCRHEIARMIIMHEYPLHMVEHPGFIAFVQNLQPRFDKVSFNTVQGDCVATYLREKQSLMKLIEGIPGRVCLTLDMWTSNQTLGYVFITGHFIDFEWKLQRRVLNVIMEPYPDSDSALSHAVAACLSDWSLEGKLFSLIFNHPTSEAGLENLRPLLCTKNPLILNGQLLLGNCIARTLSSMAKDVLGAGHEIVKKIRDSVKYVKTSESHDEKFVQVKNQLQVPSEKSLILDNQNQWNTTYQMLAAASELKEVFNCLDTSDPDYKLAPSMEDWKVAETLCSFLKPLFDAASILMTTTNPTAITFFHEAWKIHADLGRSIANDDPFISNIAKSMLEKIDKYWKDCSLILAIAVVMDPRFKMKLVEFSFTKIFGEDAPTYIKIVDDGIHELFLEYVALPLPLTPTYTEEGNVGNNGKTNESQQGNLLSDQGLTDFDVYIMETSSQQMKSELDQYLEESLLPRVQEFDVLGWWKLNKMKYPTLSKMARDILSIPVSAAAPDSVFDIIIKQLDEYRSSLRPETVEALICAKDWLHHGSEESNALVKMEF	636	72.178	- 3	6.209	-0.244
Ga11	Ga11G1486	43119625	43126879	MFSKEINILIRLKLNLLFRRRPKPLLSKITVHSNRIKYIDSKSVIRESMMNVLKENNTKKIDKKRRKDDFLSQLREEEDEHEEFIDEISAIRQATRESIQSQHECHRREEFRRSTGGWDNIYEKGRSSQGSSIPTESEFTLRGTIPELVRSKSSKQPKVSDSFLKSFRKKIGEAVSKFLIYERLPFQLASSPWLYNLIQVSTEVGQGVKLPTPYEVSDVYLESEYQRVHNWEYKESKWGQQKSGPAYEAKKIILGKDFWKKANDLIKVYEPLVKVLRLVDSDEKPTMGFIYEAVDRAKRAIQQDCRYFTEYEKIIDKRWNFMHSDLHSAGYFLNPQFQFGVEHSQNVLIETLEGTRSVIERLEPSLDTQVRMVNQLLLFRDKHETFGTPQAQRAWKQMNPVKQLQHQIERNWSTFSYIHTKARNRLKYKKLEKLVFTYYNMRLQIRHQKRMSTDDINTSFNPISLDYIFEDVDPLSEWLHEKENPLLDGENAGVLPVDTSDDEMDVNQSQQQNLSHSSSSSTPSQSGDGPDGGGLSPVDEDDGYNGDRGEIRSSSQYGGEYGVGTTSRHFRDRSEFDGNMFPEPRRDRSEPRAPSKGKGKKHTSIGSSSGRRSGSSNLGYSDSSTSTQGFYPPEQPSYFQPSHGYPQPYGYYPPFPNYGVPYQPQMHPPPPMYHPPPPLMYHPPQIYPPHQLYENQCENVTFFGYIFGQRPRESSQERSQNEGDGSDLPRHSTNW	735	85.364	13	8.329	-0.959
Ga12	Ga12G1697	26411725	26415047	MVEEMAPLRSIGYVDPGWEHGTAQDERKKKVKCNYCGKVVSGGIFRLKQHLARLSGEVTHCEKVPEEVCLNMRKNLEGCRSGRKRRQFDYEQAALSIQSNEYSDGEDASASYKHKGKKVMGDKNLVIKFTPLRSLGYVDPGWEHCVAQDEKKRRVKCNYCEKIISGGINRFKQHLARIPGEVAYCEKAPEEVYLKIKENMKWHRTGRRHRKPDTKEISTFYMHSDNEDEGGEEEGYLQCVSKDILAIDDKVSDNDIRNNVRGRSPGSSGNGAEPLLKKSRLDSVFLKSLKSQTSAHYKQPRARTGFEKKTHREVISAICKFFYHAGIPSNAANSPYFHKMLELVGQYGQGLQGPSSRLISGRLLQEEIANIKEYLVELKTSWAITGCSVMADSWNDAQGRMLINFLVSCPRGVYFLSSVDATDIIEDAVHLFKLLDKAVDEVGEEYVVQVITRNTLSFRNAGKMLEEKRRNLFWTPCAVYCIDRMLEDFVNIKWVGECVDKAKKVTRFIYNNTWLLNFMKKEFTKGQELLQPAVTKFGTNFFTLQSLLDQRVGLKRMFQSNRWLSSRFSKSDEGKEVEKIVLNVSFWKKMQYVKKSFEPVAEVLQRIGSDKIRSMPFIYNDICRTKLAIKAIHGDDVRKYGPFWSVIESNWSSLFHHPLYVAAYFLNPSYRYRPDFLMNPEVFRGLNECIVRLEADNGKKIAASMQIPDFVSAKADFGTDLAISTRSELDPASWWQQHGISCLELQRIAIRILSQTCSSIGCEHNWSAFDQVHIKRHNCLSRKRLNDQTYVHYNLRLRERQLGRKPDELVSFDSAMLESVLDDWLVETEKLAMHEDEEIIYTEVEQFCGDDMDEHESEEKRPAEMVTIAGFIEPLDVIPSAGGVTTDDDGLDFLDDDLTD	900	103.259	11	7.389	-0.513
Ga11	Ga11G10	179786	179876	MIPKNVGETTAQRERNSKQPLKIRDRKWCFGSKNRVLIPLPNGVNLSLVNTNSHSHPLHLLTCVIRESMMNILKESKTKKIDKKRRKDEFLPQLREDEDEHEEFIDEVSAIRQATRESIQSQHDIGGWNNIYEEGRSSHGSAREYHRERTSKSILSESEFTLRGAIPELVRSKSSKQAKVNDSFLKSFRRKIGEAVSKFLIYERLPFQLASSPWLYNLIQVSTEVGQGVKLLTPYEISNVYLESEYQRVRDWINGLKTYWKELGATLMCNGWTNSLNQMHIINFLVYCSKGTIFWKSVDVSSVRSRDAEFYYSLLDSVVEEIGENYIVQIVTDNEVAMEAAGKKLMLKRKHLYWTSCVAHCLDLCLEDIEKKPSVAKVLDKAKKVTCFIHNRIWTVDLMKKYTQGKQILRPSLTRFVTHFIQLEEITRQKQGLREMNLKNQNGDSKSQGQLMKPKKLFWEKIFGKKANDLIKVYEPLVIVLRLYEKIIDNRWNFMHSDLHSAGYFLDPQFQFGVEHSENVLIETLEGTRSVIERLEPSLILKSEWLIRLYSSTIICNLLLLFRDKHETFGTPQAQRAWKQMNLDVDPLSEWLHEKENPLLDGENTGSGDGPDGGGLSPIDEDDGYSV	767	88.256	18.5	8.741	-0.622

	22	63	27	DRGEIRSSSQYGGEYGVGTTSGVGTTSGYFRDRSEFDGNMFPEPRRDRSELRAPSKRKGNKHTSIGSLSGRRSSSSNLGYNDSSTSTQGFYPPEQPLYFQPSHGYPQPYGYYPPFPNYGVPYQPQMHPLPPMYHLPPPLM					
Ga6	Ga06G0799	15036141	15042793	MDPDVERTLADDVESVAAAPAQGTPPVEPQSSANNQGEGAKQAFFTMMNEWVAQYARTNPAVQQFPNLNNPPQEPVMPSVTDPVRLSKPPVDLIRKRGAEEFRAVITDDAERAEFWLDNTIRVFDELSCTPDECLKCAISLLRDSAYYWWRTLISIVPNERVTWDFFQTEFRKKYISQRFIDQKRKEFLELKQGRMTVSEYEHEFVRLSRYARECVADEVAMCKRFEEGLNEDLRLLVGILEIKEFVTLVERACKAEELGKEKKKAEFEARDYRKRSTGKAPFSAVKKFREDTNRSRTTAGISIRPRPLTDSRATSVASVGNNRQEKPECSQCGRRHIGECWGKSTNKACYGCGSKDHLIRDCTELDEKNRMQGARPSGMTARGRPPRISGGRGGSQRGATDTAVRSETRAPARAYAIRAREEASSPDVITGTFTLFDTNVIALIDPGSTHSYICETLASSKTLPIESTEFVIRVSNPLGHYVLVNKVCKKSPLVFRGSCFPADLMLLPFDEFDVILGLDWLTMHDAVVNCKGKTIDLRCANNEIIRVESTDLKGLPAVISAMLAQKYVRKGYEAYLAYVLDDKESEKKPESVPVVCEYPDVFPEELPGLPPVREIEFSIELVPGTTPISIAPYRMAPTELKELKAQLQELVDRGFARPSFSPWGAPVLFVKKKDGTMRLCIDYRQLNKATIKNKYPLPRIDDLFDQLKGASVFSKIDLRSGYYQLRIRDSDIPKTAFRTRYGHYEFLVMPFGLTNAPAVFMDLMNRIFRPYLDRFVVVFIDDILVYSQNENEHAEHLRLVLQILRDKQLYAKFNKCEFWLREVSFLGHVVSASGIRVDPSKISAILNWKPPRNITEVRSFLGLAGYYRRFVKGFSMIATPMTKLLQKDVKFEWTEKCQKSFDQLKTHLTKAPVLVQPESGKEFVIYSDASLLGLGCVLMQEGRVVAYASRQLKPHEKNYPTHDLELAAIVFALKIWRHYLFGEKCHVYSDHKSLKYLMTQRDLNLRQRRWLELLKDYELVIDYHPGKANVVADALSRKSLFALRAMNVHLSILPDNVLVAELKAKPLLAHQIREAQKVDEELLAKRAECVLNKESEFQIDDDDCLRFRSRLCVPKNSELISIILNEAHCSRMAIHPGSTKMYNDLKRRFWWHGMKRDISDFVSRCLICQQVKAEHQVPSGLLQPIMIPEWKWDRVTMDFVSGLPLSASKKDAIWVVVDRLTKSAHFIPVRTDFSLDKLAELYVSQIVRLHGVPISVVSDRDPRFTSRFWKKLQEALGTKLHFSTAFHPQTDGQSERIIQILEDMLRCCILEFSGSWERYLPLIEFAYNNSFQSSIKMAPYEALYGRKCRTPLFWTELGESKIFGVDLIKDAEQKVRVIRENLKIASDRQKSYADLKRKDIEYQVGDKVFLKVSPWKKILRFGRKGKLSPRFIGPYEISERVGPVAYRLILPPELEKVHDVFHVSMLRRYRSDPSHVISPSEVEIQANMSYEEEPIRILAREMKELRNKRVPLVKVLWLRHGMEEATWEPENSMKERYPNLFTGKIFGDENFPKNQVKGKEKSFGEVGHTSIQVEPEKLKKEKQARAKPRTSSSRVGTVILNASSMCANIISTMCATFGCGNKLCMNEVLDPFCVLGTVIEVITPSSDFLVSSS	1,654	189.269	47	8.885	-0.358
Ga6	Ga06G0549	8098009	8100183	MFALDELLEAVVLLYIYIYIYTHIHTYISWTTFLKFLFLSAAGFTSMTEMTEIADMETIPGESNNQLALTTPEAQPIKRRKKKSMVWEYFTIENVSAGCRRAYCKRCKQSFAYSTGSKVAGTSHLKRHIAKGTCRALLRGQGQDNNQFITPYNPKMGGSEPPKRRYRSPSSPFIPFDQDRCRHEIARMIIMHEYPLHIVEHPGFIAFVQSLQPQFDKMSFNTVQGDCVATYLREKQSLMKFIEGIPGRFCLTLDMWSSNQTLGYVFITGHFVDSDWKLHRRVFNVVMEPYPDSHSALSHAIAACLSDWSLEGKLFSLTFNHPLSEAGMENLRPLLCVKNPLILNGQLLIRNCIARTMSSMAKDVLGAGQEIIKKIRDSVKYVKMSESHDDKFIQVKNQLQVPSEKSLFLDNQTRWNTTYQMLAAASELKEVFDCLDTYDPDYKLAPSMEDWKLAETLCSFLKPLFDAASILTTTTLPTVITFFYEVWKIHVDLGRSITSEDPFISNLAKSMQGKIDKYWKDCSLVLAMAVVMDPRFKMKLVEFSFTKIYSEDAPTYIKTVDDGIHELFLEYVALPLPLTPTYAEEVNGANNGKTNESHYGNLLSDHGLTDFDVYIMETNSQQMKSELDQYLEESLLPRVQEFDVVGWWKLNKMKYPTLSKMARDILSIPVSAAAPESIFDITDKQLDEYRSSLRPETVEALICAKDWLHFGSSDVSNALVKMEF	724	82.782	-0.5	6.469	-0.21
Ga6	Ga06G0548	8091818	8093854	MTEMTEIADMETIPGESNNQLALTTPEAQPIKRRKKKSMVWEYFTIENVSAGCRRAYCKRCKQSFAYSTGSKVAGTSHLKRHIAKGTCRALLRGQGQDNNQFITPYNPKMGGSEPPKRRYRSPSSPFIPFDQDRCRHEIARMIIMHEYPLHIVEHPGFIAFVQSLQPQFDKMSFNTVQGDCVATYLREKQSLMKFIEGIPGRFCLTLDMWSSNQTLGYVFITGHFVDSDWKLHRRVFNVVMEPYPDSHSALSHAIAACISDWSLEGKLFSLTFNHPLSEAGLENLRPLLCVKNPLILNGQLLIRNCIARTMSSMAKDVLGAGQEIIKKIRDSVKYVKMSESHDDKFIQVKNQLQVPSEKSLFLDNQTRWNTTYQMLAAASELKEVFDCLDTYDPDYKLAPSMEDWKLAETLCSFLKPLFDAASILTTTTLPTVITFFYEVWKIHVDLGRSITSEDPFISNLAKSMQEKIDKYCKDCSLVLAMAVVMDPRFKMKLVEFSFTKIYSEDAPTYIKTVDDGIHELFLEYVALPLPLTPTYAEEVNGANNGKTNESHYGNLLSDHGLTDFDVYIMETNSQQMKSELDQYLEESLLPRVQEFDVLGWWKLNKMKYPTLSKMARDILSIPVSAAAPESIFDITDKQLDEYRSSLRPETVEALICAKDWLHFGSSDVSNALVKMEF	678	77.345	-0.5	6.464	-0.294
Ga11	Ga11G0526	7330760	7332869	MEVANESTAKKPKRLTSVVWNHFERVKKADICYAVCVHCNKKLSGSSNSGTTHLRNHLMRCLKRSNYDVSQLLAVKRRKKENTLTIANISYDEGQRKEDYMKPTIVKYEQDQRKDEAFNLGSSWFDPERSRLDLARMIILHGYPLAMVEQVGFKVFVKNMQPLFDVVHNSTIELSCVEIYMKEKQRIYDMLSKLQGRINLAIEMWSSPENSKYVCLTAHYVDDEWKLQKKILNFLTLDSSHTEDVLSDVIIKCLMDWDIDCKLFAMTFDDCSTNDDIVSRIKDQVSESRPRLSNGQLLDVRSAAHVLNSIAQDAIEALQVVIQKIRGSVKYVKSSQSILGKFNEIAQQQGIDNHKIVVLDYPIRWNSTYMMLEIAVEYRNVFHHLPELDPDFALSDEEWERASSIVSCLKLLIEIINVFSSNKCPTANIYFPEICHVHIQLIEWCKSSDAFLSSLATKMKAKFDKYWSKCSLALAVAAILDPRFKMKLVEYYYSQIYGSTALERIKEASDGIKELFNAYSICSTLIDQGSALPGSSLPSSSNDTRDRLKGFDKFLHETSQSQTAISDLEKYLDEPVFPRNCDFNILNWWRVHTPRYPILSMMARDVLGTPMSTIAQEFAFNAGGRMLDSNQSSLPPDTQQALICTRDWLRTQSDDATPSSSHYALPLYVEAN	672	76.952	3.5	6.846	-0.312
Ga13	Ga13G0405	4739633	4743091	MPPREEFPTKGLKGAPSNDIGWHFGTLVPNTKEISYVNFHIAHKTGNVAPCPNVIGVIRESMMNILKESKTKKIDKKRRKDEFLSQLREYEDEHEEFIDEVSAIRQATRETSSTWLYSLIQVSTEVGQGVKLITPYEISDVYLESEYQRKSVDASSVHSGDAEFYYSLLDSVVEEIRENYIVQIVTDNEAAMKAAGKNLMLKRKHLYWTSCAAHCLDLCLEDIGKKPSVAKVLDEAKKVTCFIYNHIWTVDLMKKYTQGKQILRPALTRFATHFIQLEEITRQKQGLREMFNSKQKSGPAYEAKKIILEKDFWKKANDLIKVYEPLVKVLKLVDSDEKPTMGFIYEAVVRAKRAIQQDCRYFTEYEKIIDNRWNFMHSNLHSAGYFLNPQFQFGMEHSENVLIETLEDKHETFGTPQAQRKENPLLDGENVGVLPVDTPDDEMDVDQSQQQNLSHSSSSSTLSQSGNGPDGGGLSPIDEDDGYSGDKGEIRSFSQYGGEYGVGTTSGYFRDRSEFDGNLFPEPRKDRSEPRAPSKGKGKKHTSIGSSSGRRSSSSNLGYSDSSTSTQGFNPPEQPSYFQPSHGYPQPYGYYPPFPNYGVPYQPQMHPPPPMYHLPPPLMYPPPQIYPPYQLYENKGENNQVNNALKVKVKDLIFLVIPLIGEN	663	75.294	-2	6.342	-0.704
Ga13	Ga13G0357	4027246	4033725	MPPREEFPTKGLEGAPSYDIGWHFGTPVPNAKGNIICKLCGKVVKGGITRFKEHIAHKTGNVAPCPNVTGVIRESMMNVLKESNTKKTDKKRRKDDLLSQLREEEDGHEEFIDEISAIRQATRESIQSQHERYMYNLIQEYKESKWGQQKSGPAYEAKKIILGKDFWKKANDLIKVYKPLVKVLRLMDSDEKPTMGFIYEAVDRAKRAIQQDCRYFTEYEKIIDKRWNFMHSDLHSAEPSFDTQVRMVNQLILFRDKHETFGTLQAQRAWKQMNPAEWWIIYGTCVPKLQKLAIKVLSQTTSASNCERNWSTFSYIHTKARNRLKYKKLEKLVFTYYNMRLQIRHQKRMSTDDVNTSFNPISLDYIFEDVDPLSKWLHEKENPLLDGENAGVSSSTPSQSGDGPNGGGLSPVDKDDGYNGDRGEIRYSSQYRGEYGVGTTSGYFRDRSEFDGNMFPEPRRDRSEPKAPSKGKGKKHTSIGSSSDRRSGSSNLGYSDSFTSTQGFYPPEQPSYFQPSHGYPQPYGYYPPFPNYGVPYQPQMHPPPQIYPPHQLYENQCENVTFLGYIFGQRPRESSQERSQSEGDGSDLPRHSTIGEN	597	68.568	16	8.889	-0.941

Ga3	Ga03G0125	1040564	1048005	MASSFSHTALRLLLSCAEAIENGDLKRADKFLHNILILADERPYYFADESRVVRYFADALVRRAYGLHPASSYFPFPVDPAQYYHYNCYHINGVIKKVIDDALMEKNALMGNRRLHLIDFSIPYYIIEGSVLRTLPNYSGDPLSVRVSYIPPPFLKKQVKLSRQREFLTKEAKEVNVKWEDELKVVYANSLAEMDECELDFKGSRDDEMVVVYYKFKLHKLLRDAKAMERELVRLKEINPMIVIMLDFYANRSDSNFLRCLEDSFQYYSNTCRFWGESDFGFFGYLWECACEGNNLIRRHQTLSEWQRLFSMGGFSRIPLNHTKDWLIIMQHTLVNQSFLEIMGEEEECLVLGYEQCPIFFLSAWKPKVEDRHLNSTPSNDKFEDFNPNPLPLQSLQPFSEGLTLNRLAALAEIYDILKHLCYRYELPLALTRTSKTNDMNETMSDPNKKHTFLIQSSYCYVKDWNAYRFINSCKIDTKSIIDKALKSRDGYHFEPSIKLDKLDYSYPIGPKYYNIDVAVAICLQNCYTSNDVYGVEFYWPITESEKSKSLALNIFNDWKLMKTKFVTVKVQGTQFRFQEEVISNIPTSSNTTRTLEIAEEARDVHAKEINGHIEQIIFNDDPVKAKRNKQRKLWSAVWEHFDRFEVKGKQVARCKDCRKECTGSIKSGTTHLKNHLDRCPAKKKQNQERQLIFPAYTNERSSTFDKERSCFKLAKMIIKHRSPLDMVDQEFFKNFVKDMQPGFEFQLKEILFYINQIYKEEKKKLQLYFDRLACKLNLTLSLWKNNHGKTAYCCLIAHFIDDGWEPKMKILGLRNLEHIYDTKVVGGIIRSFVSKWNISRKVCSITVDNSFLNDGMIHQIKENCVSEQGSLSSTHWFISFTLLEDGFRDMDGILSKLRKSIEYVTETTHGKLNFQEAAKQVKLQGGKSWDDLSFKPESDSDILDSALRSREIFCKLEQIDYNFMLNLSMEEWEKAVTLQSCFKCFDDIKGTQSLTANLYFPKLCNMYEEFGQLKKSNHPFINLMKRKFDNYWSLCNVAFTIAATLDPRLKFRSSCNETYDLENMIKLIRFRKVLVDVYFEYANEAKNLSASSSVLIDSNSLTTETTNDCIVSYFSKFASPSNVKVVASQKSELDCYLEETLLPSDADILGWWRVNSQRFPTLAKMARDFLAIPVSVSAPCSHISAMTRNPAYSRLDPESMEALVCSQNWLESTKENDGEHHEPMQNMDKRKRKVEENGTSTVKVFKNRNHEKASSNGDIASDFNKNDGNLSFDNWMEPQCSSSESVGEKAEIMEASVRNRDRNIAAAIEIPNDEPSFNTNQLDEFQSSSSESDDETTLREQRSWYREDVRTYLVSSFTNKEKKRLNRWETSELSGYVASFN	1,382	160.667	7.5	6.909	-0.501
Ga3	Ga03G0123	1026224	1035935	MASSISDTALRLLLSCAKAIEDGDLKSADALLHIILVLADERPYLYESRLVKYFADALVRRAYGLHPASSYNTFPGNPAPYYHYNGYRINDIIKKVIDDALMGNRRLHLIDFSIPYENFEGSVLRTLPTFSGDPLPVRVSYILPPFLKKYVESFSQMEFLTKDAMNLNVKLEAELKVVYANSLAEVDEYKLDFKRRREDEMVVVYYKFKLDKLLTDGKAMERELVRLKEINPTIVIMLDFYSNHTHSNFLTCLEHSFQYYSNTCTFWCRYGIFLYSKYEWECNRDASEGNNIIRRHQTLSEWQRLFSIAGFTRIPLSHEKDDLIDFFFFDDSSFLEIMREEEECLILGYKECPMFFLSAWKPKVEDGHFNSISTNHQFGQGFNPNPLPLQPLQPFLEGFALNRLAAFTEIHDISKYLCCKYKLSLALTSASKVNNMNKPFWGPNKEFPFFIQSNSCYVKDLKSYQFMNHVVEVINSLIIEKAFESRDGYHFEPSITKLDLEYYPYALVKNYNIDVVVAICLQNRHTSNEVYIVQFYWPPTESEISKSLALRIFDDLKHMKTTFVSVKVQGTEIKFQEEAISSIPTSSNTAMPLKIVEEARNIDAIEINGHIEQVVAAPFNTLEGPYNQIGKTKGNKQRKSWSKVWVDFDKFEENGKQVAKCKHCPKVLTGSSKSGTTHLNNHSKVCPGKKKQNQETQSILPVDTNERSSTFDQERSHLDLVKVVIKHQYPLDLAGQEAFKNFVKGLQPMYEFQSRDKLLSDIHRIYNEEREKLQLYFDQLACKLNLIVSLWKNNHGKTAYCCLIAHFIDDGWELKMKILGLRKLEHVYDTKVVGGIIRSFVSVWNIRKKVCSITVDNSFLNDGMVHQIRENCVREQGSLSSAYWFISFTLLEDGFREMDSILSKLWKSIEYVTETTHGKLNFQEAVNQVKLQGGKSWDELSFKLESDSDILDSALRSREIFCKLEQIDDNFMLNLSMEEWEKAATLQSCFKCFDDIKGTQSLTANLYFPKLCNMYEEFGQLKKSNHPFVILMKRKFDNYWSLCNVAFTIAATLDPRLKFRSSCDETYDLENTMKLRRFRKVLMDVYFEYANEAKNLSASSSVLDDSNSLTAETTKDCIVSYFSKFASPSNVKEVASQKSELDCYLEETLLPSDADILGWWRVNSQRFPTLAKMARDFLAIPVSVSAPCSNISAMTINPAYSSLDPESMEALVCSQNWLESTKEKDGEHHEPMQNMDKRKRKMEENGTSTVKVFKNRNHEKASSNGDIASDFNKNDGSLSFDNWMEPQCSSSESVGEKAEIMEASVRNRDRLESSIGKPNHGRNIAASIEIPNDEPSFNSNQLDEFQSSSSESDDETTLREQGSWCREDVRTYLVSSFTNKEIKRLNRWKKSELSRKLIGRDKEFKLVGKKLKPLLMVPYCDETLIEYYIDDSVVNAFFKLLKGRSDKYPNVYIKNYSFDPQIATCLIKGSKLEDEVLGWFKAEKLRGVHKLFLPMCLSAHWVLFCVDTREKKISWLDPIPSSRIKSNNVEKQIILQWFTSYLLPEFGYNDADEWPFVVRTDIPKQEN	1,567	181.089	0.5	6.537	-0.421
Ga3	Ga03G0122	1011005	1020517	MASSSSFSSADAALEILVSCAHAIEDGNLKIADSFLHQIWNTAAVELDLISKLVRYFAEALVRRAYGLHPPYYTHSNLQIPHPLYYYYYYSRFDINEMVGEAIESATTGKKGFHLIDFHIPHLYGRGYLFKTLPNRSSDPLSVRITVVLPTFLKNTVDFQEEMEYLTEAGKLLKIELKKEDLRVVYANSLGEVDESTLDLRRTNDDEALVVYYNFKFHTLLAEAEAMKKELIKLRQLNPEIVIMQEQYANDNDGNFIKRLEYSFRYYSNFFQYYSNLFKSGKPLGYNTAKYYMRQIHNIVACEGRDRIMRHQSLDEWRDLLLTAGFLQIPFQKDVENLHALYWVEEIKEEKGCLVLSHKDCPILFVSCWRPRAGEEHFKFNLNSNNLGQEVCFRYELPVALTWACEATTDKIMLDGKKHTLFMERTSCYASNEGSQCFMEACAKHHIQEGQAIAGKAFQSSANFHFKPSITKLMKSDYPLFNAAQLFGSHAVVAICLQNHYIIGDVYVVEFYWPEIESEKSESLALDIFSDLKNMKKKFVTIRVGGNEVGFEREAISTTLQGTMHMRNAQPASSTNDLLSSNTTWSLNAVQQCDVHEMERHGLVEQVESAPFSTPNPMSYGGVLQTQGPHKQEIGDKDFISQTVSIGDYEIVKAYMETCKVPRTKRRKYSSKVWLDFDKFEVNGKQVAKCKHCNKDFTGSSKSGTTHLKNHLERCQSDLITRDSDESNFTFDQERSRLDFAKMIIKHQSPLDMAEQEFFKIFVKNLQPMFEFQSKDILLSDIHRIYKEEKEKLQLYFDQLACNFNLTISLCKNNHGKTAYCCLIAHFIDDNWEPKMKIIACKALEHIYDTKALNEIIQSSVLEWNISKKVFSITMDNPFLNDDMFQKIKETCFSDQGSFPSTHWFIGCTFIEDGFREMDLILLKLRKSIEYVSEIAQGKLKFEEVVNQVKLQGVKSWDDLSLRLDSDFGVLHSALESREIFCQLEKIDGNFKLNPSVEEWEMVLAFHSCLKCFDDIEGTQSLTANLYFPKLCNIYKKFLHLEKSNYPIVTLVKRKFDYYWSLCNSAFAVATILDPRLKFKFVEFSYTEIYGHDSKMHLNRFHKVLTDVYYEYANEARNLSKSTSDLDDSNSSTTEIVNDCILESFSKFASANNFNEVASWKSELDCYLDEPLLPLDGAFDILYWWCINTKRFPTLAKMARDFIAMPISILAPCLNFNAMITNPTYNNLNPESMEALVCSQNWLKIPKENDGENHGPMQNMYKRKRKMEDHSNVVKVSKNWNREEANSSGDIAKGSIKNENIEASVCNRNRLEISTGKPNHGRNITALIEIPKDDSPFSNNKSGQFQSLSSESDNETTLKEQGSWCKEDVRAYLVSRFTGKENKRLNRWQTNELIGKLIGRDKEFCLMGDKLAPLLMVPHGDETRKEYYIDDCVVNTFFKLLKKRSDRFPKAYINHYSFDSQIATCLIQGSRSEHEVLAWFKAKKLRGAHKLFLPLCLSAHWVLFYVNTKEKKISWLDSNPSSRIMSNNVEKQTILQWFTTFLLPEFGYYDANEWPFLVRTDIPVQKNWVDCGVFVMKYGDCLTHGDFFPFTQNDMVQTSLVHMYCRCGRIDKAEVLGGVLYKDLAMQITEPCSLHHVVFMSILLACSHSRLVEDGLKYFKSMKDNFGIKPGIEHYTCLVDLLGRVGHFNLALKIENHPRDAYASTSSSLGSIA	1,713	197.528	6.5	6.699	-0.361
Ga3	Ga03G0116	951176	957620	MASSVFDTDTDTALRLLLSCAKAIEDGDLKSADAFLQNILILADERPYLYKSRVVKYFADALVRRAYGLHPASSYFTFPVDPSPYYHCGSYLINGVIENVIHDALMEKNALMGNRRLHLIDFSIPYSSFQNSVLRTLPTFSGDPLPVRVSYILPPFLKKYVKFSHQMEFLTMDAKEVNVKLEDEFKVVYGNTLAEVDEREIDFKRRRDDEMVVVYYKFKLGKLVRDAKVMERELVRLKEINPTIVIMLDFYSNHAHSNFLTCLEDSFQYSLKTVDCWAELDLYFDEEYEWECHIEAWEGNNVIRRHPTLTEWQHLFSMAGFSRIPLNHRKEIMGKEEECLIIGYKECPMFFLSAWKPKVEDEHLNFNSSNDKFGQGFNPYPSPLRPLQPFPEGLTLSRVAALAEIHDILNHLYCEHKFSWALTWASKVDNMNETMSDPNKKYTFTIQSNSCYLKDLNSYKFMRSCEYKIEETIIEKALESKDGYHFEPSITRFDIDDYMYLQRAKNCDVDVVVAICLQNRYLCNDVYVVEFYWPTTESEISKSFTPRIFNDLKYMEKKFVTVKVQSTEKAISNIPTSSYTARPLKIAEETEDVDAVEINGVNVQIFPNGDPEIVKANKEKPSKATQRKLRSKVWDHFDGFEEDENQVAKCKHCPKMLTGSSKSGTTHLNSHSKVCPGKKKQNQESQLILPVDTNEGSLRFDKKRSHMDLAKMMIKLRCPLDMAEQETFKNFVKGLQPMLEFQSKDILSYIHHIYDEEKEKLQLYFDKLASKFNLTVSLWKNNAGKTTYCCLISHFIDDGWELKRKILALKTLEHINDTKALGENIRSLVLEWNISNKVCSITVDNSFLNDSMVDQIKEICLSDQGSVSSDHWFITFTLLEDGFREMDGILFKLSKSIEYVTETRHGKLKLQEAVDQVKLQGGKLWDDLSFRLESDFDILDSALRSREIFCELEQIDENFKLNPTMEEWENAVALQSCLKCFDDIKGTQCLPVSLYFPKLCDAYKKFLQLEKSSHSFVTLMKRKFDRYWSLCNLALAVASVLDPRLKFKIVELSYRVIYGHDSKMRLNMFHKVLRDVYYEYASEAKYLTTSASVLDDFSCSTIGLGNDSILDSLSKCTSASNFNEEASWKLELELYLDEPLLPMDGAFFDILGWWCDKSQRFPILAKMAQDFLAIPVSISTSCSNISAMINNPAYSSLNPEIMEALVCSENWLETPKEKFNSLNAFSFHRIKGKRPWCEQDIKAYLLSRFTKKEHKRLDKWQRNELNGYIASFDVLILEYEMIIII	1,285	149.091	-12	5.895	-0.338

Ga3	Ga03G01115	937148	947448	MASSFFDIDIDTALGLLLSCAEAIEDGDLKSADKFLHNILILADERLYLYKRGVVKYFADALVRRAYGLHPA<br>SSNLTFPVDPSPYYHYNSNRINGVIENVIHGALMEKNALMGNRRFHLIDFSIPYYDSQNSVLRTLPTFSG<br>DPLPVRVSYILPPFLKEHVKFSHQMEFLTKDAKEVNVKLEDELKVVYANSLAEADEFEIDFKRREDEMVV<br>VYYKFKLDKMVRDAKAMERELVRLKEINPTIVIMLDFYSNHTHSNFLTCFKDSFQYSLKTLDFWAQLELF<br>LEEEYEWDCNIEAWEGNNVIRRHPTLTEWQHLFSMAGFSRIPLNHRKDTLSVEDEDLLGIMGEEEECLI<br>LGKEGCRMFFLSAWKPKVEEEHLNFNSSKDKLGQGFNPFPSPLPPLQPFLEGLALNHVSALAEIYDILN<br>HLCCKYKFSWAITWASKININQTMSNPNKKYTFLIQSNSCYLKDFNSFEFMHSCEYEMYFGKYEIEQTIIE<br>KALESKYGYHSITNLDGEDNPYPVFVKQYDIDVVVAICLQNRYTSNDVYVVEFYWPATESETSKSLAPRI<br>FNDLKHMEKKFVTVKVAGTKKAISNIPTSSNTSRRLIIAEETEDVDAVEINGVNVEIFPNGHPEIVKAIKEK<br>PSKATQRKLRSKVWDHFDRFEEDGKQVAKCKHCPKVLTGSSKSGTTHLNNHLKVCPGKKKQNQESQL<br>ILPVDTNEGSLRFDKKRSHMDLVKMMIKLQCPLDMAEQETFKNFLKGLQPMFEFQSKDILSYIHHIYDEE<br>KEKHQLYFDKLASKFNLTVSLWKNNSGKTTYCSLTSHFIDDGWELKRKILALKTLEHINDTKALGEIIGSL<br>VLEWNISNKVCSITVDNSFLNDSMVDQIKEICLSDQGSVSSNHWLISFTLLEDGFREMDGILFKLRKSIEY<br>VTETRQGKLKFQEAVDQVKLHGGKYWDDLSFRLESDFDVLDSALRSREIFCKLEQIDENFKLNPTMEE<br>WENAVALQSCLKCFDDIKGSQCLPVSLYFPKLCDIYKKFLQLEKSCHSFVTLMKRKFDHYWSLCNLAFA<br>VASVLDPRLKFKIVELSYRVIYGHVSKMQLNKFHKVLRDVYYKYASEAKSLTTSASVFDDFDCSTIGLGN<br>DSILDSLSKFASASNFNEEAPWKLELELYLDEPLLPMDGAFFDILGWWCDKSQRFPILAKMARDFLAIPI<br>SVSTPCSSISAIINNPAYSSLNPESMEALVCSENWLETPKENDGENHEPMQITDKRKRKMDEDSHPVKN<br>SKPSNLEKAINTEDIAKDSNNNDFSLSFDNWMEPQFSSSESIGEKAEIMKALVCNENRLESSIGKPNHEK<br>NVDVVIEIVNNDPLFDNNQSDEVQSSSSESEDEATLKEQGPWCEQDIKAYLLSRFTSKEHKRLDKWQR<br>NELNGKLIGRDKKFKFQGEILAPLLMVPQSDETRKECYINDSVVNAFFELLKKRSDEFSNTYINHYSLDS<br>QIAAQLIKGCRSELEVLNWFKAEKLRGVHKLFLPLCLSSHWVLFYVDIKEKKFSWLDPDPSSLILSYHFE<br>NHVKVLLQWFTSFLLPKLGYIDANKWPFINLRQLWGLKFWLSSLFSPSEVEVGV	1,590	183.821	-20	5.732	-0.381
Ga3	Ga03G00771	520967	523214	MDNFDQKLGPEFFKNLSAEAVTPLNVVHEEIYESSSKRPKTTSKVWDIFEKLPAQQGDSKAICKLCRRIY<br>TAKTTSGTSHLRRHIEACVKRGNHEVDQRSIEACFKPVKRNANRLTLSHDTLISATTSLKNYKLDVDEIH<br>RAIAMMIIVDEQPFSVVEDAGFRRLLSAACPEFPVLSRSSIKRDIISIYVKERENIRELLATCPGRICLTSST<br>WKSDSDDHFNCVTTHFIDHEWRLQKRILRFKLMPPPYDSLSVADEIALCMVQWNIEHKVFSVTLENLSS<br>DDCVADMLRSRLAAKKYLPCKGVFFHVSCFFRILNSIVQAGLNLVVDIIAKLRLGIKYVQQSPHRKKNFYI<br>VAKTLNLDTQRKLCLDTPARWNSTYDMIEVAFCYKNAFVYLAEQDKNFLHKLSEDEWEKMSVLYKFLK<br>VFYEVTCVFFRNRQPTSNLYFKAAWKVHSRLFDMVRGPENFMTRMVREMHSKLNHYWSAYNLILSCA<br>AILDPRYKIKFVEYCYTKLYGSGAQKYVSVSVNTLYGLFDEYMQTSARPSQTTLLSTAASKISNDKDEND<br>GFEDYETFQSARFRTQVEKSQLDLYLEEPSHDLNSEIDVLEYWTLCSLRYPELSKMARDVLTIPVSTIAS<br>DSAFDITPQVISADRSSLKPKMLQALVSLQDWMLASDRTRGLGSMESKPEDDSSSSSDGDDDY	691	79.628	10.5	7.739	-0.337
GaS	Ga14G2397	188851	235529	MEPGVERPLADDVESVAAAPAQGTPPVEPQSSANNQNEGAKQAFFTMMNEWVAQYARTNPAVQPFP<br>NLNTPPQEPAMPPVTDPVRLSKPPVDLIRKRGAEEFKAIVTDDAEKAEFWLDNTIRVLDELSCTPDECLK<br>CAISLLRDSAYYWWRTLISIVPNERVTWDFFQSEFRKKYISQRFIDQKRKEFLELKQGRMTVSEYEHEFV<br>RLSRYARECVADEVAMCKRFEEGLNEELKLLVGILEIKEFVTLVERACKAEELGKEKKKAEFEARDYRK<br>RSTSKAPFSAVKRFREDTSKSRTTAGISIRARPLTDSRATSVASVGNNRQERPECPQCGRRHLGECW<br>GKSVNRACYGCGSKDHFIRDCTELDERNKTQGARPSGTTAGGRPPRISGGRGGNQRGASNTAVRSE<br>TRAPARAYAIRAREEASSPDVITGTFTLFDTIVIALIDPGSTHSYVCETLASSKTLPVESTEFVIRVSNPLG<br>QYVLVDKVCKRCPLIIRESCFPADLMLLPFNEFDVILGMDWLTVHDAVVDCKRKTIDLRSANNEVVRVES<br>TDLKGAPAIISSMTARRYVKKGCETYLAYVFGSKETERKLESVPVVCEYSDVFPEELPGLPPVREVEFGI<br>EVVPGTTPISIAPYRMALTELKELKVQLQELTDRGFARPSFSPWGAPVLFVKKKDGTMRLCIDYRQLNK<br>VTIKNKYPLPRIDDLFDQLKGASVFSKIDLRSGYYQLRVRESDIPKTAFRTRYGHYEFLVMPFGLTNAPA<br>VFMDLMNRIFRPYLDRFVVVFIDDILVYSRDETEHAEHLRLVLQILRDKQLYAKFSKCEFWLKEVSFLGH<br>VVSASGVRVDPNKILAIVDWKPPRNVTEVRSFLGLAGYYRRFVKGFSTIATPMTKLLQKDVKFEWTEKC<br>QKSFDQLKAYLTEAPILVQPESGKEFVIYSDASLLGLGCVLMQEGRVVAYASRQLKPHEKNYPTHDLEL<br>AAIVFALKIWRHYLFGEKCHVYSDHKSLKYLMTQRDLNLRQRRWLELLKDYELVIDYHPGKANVVADAL<br>SRKSLFALRAMNVHLSILPDNVLVAELKAKPLLAHQIQEAQKVDEELLAKRAECVLNKESEFQIDDDDCL<br>RFRSRLCVPKNSELISIILNEAHCSRMSIHPGSTKMYNDLKRQFWWPGMKRDISEFVSRCLICQQVKAE<br>HQVPSGLLQPIMIPEWKWDRVTMDFVSGLPLTQNKKDSVWVIVDRLTKSAHFIPVRTDFTLDKLAELYV<br>SQIVRLHGVPISIVSDRDPRFTSRFWKKLQEALGTKLHFSTAFHPQTDGQSERIIQILEDMLRCCILEFSG<br>SWERYLPLIEFAYNNSFQSSIKMAPYEALYGRKCRTPLFWTELSEGKFFGVDLVKDAEQKVRVIRESLK<br>AASDRQKSYADLKRKDIEYQVGDKVFLKVSPWKKVLRFGRKGKLSPRFIGPYEVSERIGPVAYRLILPPE<br>LERIHNVFHVSMLRRYRSDPSHVIAPSEIEIQPNLSYEEEPVHIMRREVKELRNRKIPLVKVLWHKHGME<br>EATWELEDSMKERYPSLFTGKIFGDENFLSGGEL	1,558	178.817	39	8.726	-0.364
GaS	Ga14G2037	139944	174405	MPPREEFPTKGLEGAPSNDIGWHFGTPVPNTKGNIVCNLCGKVVKGGITRFKEHIAHKTGNVAPCPNV<br>TGVIRESMMDILKESKTKKIDKKRRKDEFLSQLREDEDEHEEFIDEVSAIRQATRESIQSQHEWHRREEF<br>SRSTGGWNNIYEEGRSSHGSAREYHRERTSKSIPSESEFTLRGAIPELARSKSSKQPKIGEAVSKLLIYK<br>RLSFQLASSPWLYNLIQVSTEVGQGVKLPTPYEISDVYLESEYHEFVIGLNQMHIINFLVYCSKGTIFWKS<br>VDASSVRSRDAEFYYSLLDSVVEEIGENYIVQIVTDNEAAMKAAGKKLMLKRKHLYWTSCVAHCLDLCL<br>EDIGKKPSVPKVLDEAKKVTCFIYNHIWTVDLMKKYTQGKQILRPALTRFATHFIQLEEITRQKQGLREMF<br>NSKEFKESKWGQQKSGPAYEAKKIPLVKVLRLVDSDEKPTMGFIYEAVDRAKRAIQQDCRYFTEYEKIID<br>NRWNFMHSDLHSAGYFLNPQFQFGVEHSENIKPSLDTQVRMVNQLLLFREKHETFGTPQAQRAWKQ<br>MNPAEWWIIYGTCVLELQKLAIKVLSQTTSASNLFTYYNMRLQIRHQKRMSTDDINACFNPISLDYIFEDV<br>DPLSEWLHEKENPLLDGENAGVLPVDTSDDEMDIDQSQQQNLSHSSSSSAPSQSGDGPDGGGLSPIH<br>EDDGYSGDRGEIRSSSQYGGEYGVGTTIGHFRDRSEFDGNMFPEPRRDRSEPKAPSKGKGKKHTSIG<br>SSSGRRSSFSNLGYSDSSTSTQDFYPPEQPSYFQPSHGYPQPYGYYPPFPNYGENITFFGYIFGQRSR<br>ESSQERSQSEGEGSNLPRHSTNW	851	97.198	5	6.844	-0.734

Table S18 List of GbZF-BED genes and their features

Gene_ID	Chromosome	Gene_ID	Start	End	Protein_sequence	Protein Length (aa)	Molecular Weight (kDa)	Charge	Isoelectric Point	Grand Average of Hydropathy
Gbar_A08G025510	GbA8	Gbar_A08G025510	118150598	118154629	MEVANETVIKKPKRLTSVVWNHFERVRKADLCYAVCVHCNKKLSGSSNSGTTHLRNHLMRCLKRFNYDVSQLLSAKKRKKDNTLTIANLSYDEGQRKEEYLKPTIVKYEPEQRKDEVFNVQSSWFDQERSRLDLARMIILHGYPLAMVEHVGFKVFVKNLQPLFDVMPNSTVELSCMEIYGKERQKVHDMLSKLQGRINLAVEMWSSPENTNHVCMMAHYVGDDWKLQKKILNFVTLDSSHTDDLLSGVIIKCLMDWDIGSKLFAVTLDDFSTNDDIVLRIKEQISENKSRLSNGQLLDVRSAAHVLNSIVQDAMEALRLVIQKIRGTVRYVKSSQSIQGKFKEMVLQTGINSQKNLVLDCPIQWNSTYLMLETAIEYRNAFCQLPELDLDLALSDEEWEWASSITGYLKLFIEIINVFSSNKCPTANIYFPEICHVHIQLIDWCKSPDNFLSSLAAKMKAKFDKYWSKCSLSLAVAAILDPRFKMKLVEYYYSQIYGSTALERIKEVSDGLKELFNTYSICSTLMDQGSALPLSSLPSSSNDGRDRLKGFDKFLHETSQSQTAISDLEKYLDEPVFPRNCNFNILNWWRVHTPRYPILSMMARDVLGTPMSTVSQESAFHAGGRVLDSCRCPLTPETRQALICTQDWLRMQSDDPGPSSSHYALPLYVETN	188	21.026	12	9.702	-0.548
Gbar_A11G031170	GbA11	Gbar_A11G031170	106789145	106793605	MEVANESTAKKPKRLTSVVWNHFERVKKADICYAVCVHCNKKLSGSSNSGTTHLRNHLMRCLKRSNYDVSQLLAVKRRKKENTLTIANISYDEGQRKEDYMKPTIVKYEQDQRKDEAFNLGSSWFDPERSRLDLARMIILHGYPLAMVEQVGFKVFVKNMQPLFDVVHNSTIELSCVEIYMKEKQRIYDILSKLQGRINLAIEMWSSPENSKYVCLTAHYVDDEWKLQKKILNFLTLDSSHTEDMLSDVIIKCLMDWDVDCKLFAMTFDDCSTNDDIVSRIKDQVSESRPRLSNGQLLDVRSAAHVLNSIAQDAIEALQVVIQKIRGSVKYVKSSQSILGKFNEIAQQQGIDNHKIVVLDYPIRWNSTYMMLETAVEYRNVFHHLPELDPDFALSDEEWERASSIVSYLKLLIEIINVFSSNKCPTANIYFPEICHVHIQLIEWCKSSDAFLSSLATKMKAKFDKYWSKCSLALAVAAILDPRFKMKLVEYYYSQIYGSTALERIKEASDGIKELFNAYSICSTLIDQGSALPGSSLPSSSNDTRDRLKGFDKFLHETSQSQTAISDLEKYLDEPVFPRNCDFNILNWWRVHTPRYPILSMMARDVLGTPMSTVAQEFAFNAGGRMLDSNQSSPPPDTQQALICTRDWLRTQSDDATPSSSHYALPLYVEAN	678	76.811	7.5	7.767	-0.267
Gbar_A05G038120	GbA5	Gbar_A05G038120	96290111	96294801	MDMSDAVIVNSSRLKSIVWNDFDRVKKGDTFVAICRHCKKKLSGSSTSGTSHLRNHLIRCQRRSNHGVAQYFSAKDKKKEGSLALVTIDQEQKNDEVLSIVNLRYEQEQIKSEHVGIGSNSLDQRQSQFDLARMIILHNYPLAMVEHVGFKIFVRNLQPLFELATRNKVEADCMEIYAKEKQKVYEIFDKLPGKISVSADVWTASEDDAAYLSLAAHYIDENWQLKKKNLNFVTIDPSYTEDMHSEVIMNCLMDWDIDRKLFSMIFDSFTSDNIVERIRDRLSQNRFLYCNGQLFDVRCAVDLLNRMAHDALEALCEITQKIRESIRYVKSSEATQATFNELADEVQVETKKCLCIDNPLKWNSTYFMLEAALEYRKVFSCLRDRDPVNMKFLLSDPEWDRLITVTSFLKLFVEVTNVFTRSKYPTANIFFPEICDIHLQLIEWCKNPDEYISSLALKMRKKFEEYWYKCSSGLAVAAMLDPRFKMKLLEYYYPQLYGDSATELIDDVFECIKSLYNEHSIVSPLASSIDQGLDWQASGISGSGKDSRDRLMGFDKYLHETCQAEGSSSDLDKYLEEPLFPRNVDFNVLNWWKVHTPRYPILSMMARNILGIPISKVAAESRFDTGGRVLDHNWSSLPPTTIQALMCSQDWIRSELES	674	77.23	12.5	7.765	-0.39
Gbar_A02G014400	GbA2	Gbar_A02G014400	91345392	91349157	MSTKPTSIEGSVTPPTSIDSENSGVGASIQTKGTTGKRKAPPQRSEVWSHFTKFINSEGASKAKCNYCEKEFCCDMKKNGTGSLKYHIGSCKKNPSNVVDTSQGQLVLPRKGVEGGEGNISTWRFDQEACRKGLAQMILIDELPFKFVESEGFKKFMFVACPRFHIPSRTTMTRDVYQLYLDERVKIKQLLRSSCSRVCLTTDTWTSLQRVNYLCLTAHFIDNDWKLNKKILNFCPISSHKGESIGMVIEKCLLNWGIDKLFTVTVDNASSNDVAIGYLRKKFNPRGGLVQNGKYLHMRCMAHIVNLIVVEGLKEMNKSVERVRGAVRYVRQSPARLQKFKECVVVEKIECKKMLCLDVCTRWNSTYLMLDTAQNFERAFERFEEQDTNFRAELERERVGLVDFLEHFYEVTLRISGTSYVTSNNFFDELSEIDILLRDAQLNSNIDFNVMAIKMKEKYDKYWGDIDKMNLLMFVACVLDPRQKLKYLEFALSEMSSSEKACEMMQKLKESLYELFDEYKPPLHSTCSQSSVSTHVSIGEPQQKMKRRMQALYKKRELEICGEDKTSELDKYLAEANEEFVEDFDILLWWKVNSPRFPTLSKIARDVLAIPVSTVASESAFSTGGRVLDQYRSSLTPKIVQALVCTQDWIRRSSSQEDIKKIEEQIQELDKIENELLASLSATMRIWCCDPTAKYHTSMSLLP	1,313	151.682	-14	5.939	-0.348
Gbar_A09G019340	GbA9	Gbar_A09G019340	71544691	71548153	MVRERDVCWEYAEKLDGNKVRCKFCLRVLNGGISRLKHHLSRLPSKGVNPCNKVRDDVTDRVRAIISSKEDIKEIPSVKKQKIAEVRAPGNMSTGSKISPLETLSPAAKVFPTVLSIAASTLSDQETVERSIALFFFENKLDFSVARSSSYQAMIDAVGKFGPGLIAPSVETLKTTWLKRIKSEVTLHLKDAEKEWATTGCTIIADTWTDNKSKALINFLVSSPSRTFFHKSVDASSYFKNTKCLADLFDSVIQDFGQENVVQIIMDSSFNYTGISSHILQNYGTIFLSPCASQCLNLILEEFSRVDWVNRCILQAQTVSKFLYNNASMLDLMKKFTGGQELIRTGITKSVSCFLSLQSTLKQRSRLKHMFNSPEYSTNSSYANKPQSISCIAIVEDNDFWRAVEECVAISEPFLKVLREVSGGKPAVGSIYELMTRAKESIRTYYIMDESKCKTFLDIVDRQWRDQLHSPLHSAGAFLNPSIQYNPEVKFLGSIKEDFFKVLEKLLPTPELRRDITNQIFTFTRAKGMFACNLAMEARDTV	672	77.025	8.5	7.469	-0.298

					SPGLWWEQFGDSAPVLQRVAIRILSQVCSTFTFERHWSTFQQIHTEKRNKIDKETLTDVVYINYNLKLAREMKTMPTDSDPIQFDDIDMTSEWVEESENPSPTQWLDRFGSALDGGDLNTRQFSAAMFGNDHIFGL					
Gbar_D08G026120	GbD8	Gbar_D08G026120	64331222	64334757	MEVANETVIKKPKRLTSVVWNHFERVRKADLCYAVCVHCNKKLSGSSNSGTTHLRNHLMRCLKRFNYDVSQLLSAKKRKKENTLTIANISYDEGQRKEEYLKPTIVKYEPEQRKDEVFNVQSSWFDQDRSRLDLARMIILHGYPLAMVEHVGFKVFVKNLQPLFDVVPNSTVELSCMEIYGKERQKVHDMLSKLQGRINLAVEMWSSPENTNHVCMMAHYIGDDWKLQKKILNFVTLDSSHTDDLLSGVIIKCLMDWDIGSKLFAVTLDDFSTNDDIVLRIKEQILENKSRLSNGQLLDVRSAAHVLNSIVQDAMEALRVVLQKIRGTVRYVKSSQSIQGKFKEMVLQTGINSQKNLVLDCPIRWNSTYLMLETAIEYRNAFCQLPDLDLDLALSDEEWEWASSITGYLKLFVEIINVFSSNKCPTANIYFPEICHVHIQLIDWCKSPDNFLSSLAAKMKAKFDKYWSKCSLSLAVAAILDPRFKMKLVEYYYSQIYGSTALERIKEVSDGLKELFSTYSICSTLMDQGSALPLGSLPSSSNDGRDRLKGFDKFLHETSQSQTAISDLEKYLDEPVFPHNCNFNILNWWRVHTPRYPILSMMARDVLGTPMSTVSQESAFHAGGRVLDSCRCPLTPETRQALICTQDWLRIQSDDPGPSSSHYALPLYVETN	658	76.119	-2	6.317	-0.354
Gbar_D11G031860	GbD11	Gbar_D11G031860	63030188	63034788	MEVANESTAKKPKRLTSVVWNHFERVKKADICYAVCVHCNKKLSGSSNSGTTHLRNHLMRCLKRSNYDVSQLLAVKRRKKENTLTIANISYDEGQRKEDYMKPTIVKYEQDQRKDEAFNLGSSWFDPERSRLDLARMIILHGYPLAMVEHVGFKVFVKNMQPLFDVVHNSTIELSCVEIYMKEKQRIYDMLSKLQGRINLAIEMWSSPENSKYVCLTAHYVDDEWKLQKKILNFLTLDSSHTEDMLSDVIIKCLMDWDIDCKLFSMTFDDCSTNDDIVLRIKDQISESRPRLSNGQLLDVRSAAHVLNSIAQDAIEALQVVIQKIRGSVKYVKSSQSILGKFNEIAQQQGINNHKIVVLDYPIRWNSTYMMLETAVEYRNVFHHLPELDPDFALSDEEWKRASSIVSYLKLLIEIINVFSSNKCPTANIYFPEICHVHIQLIEWCKSSDAFLSSLATKMKAKFDKYWSKCSLGLAVAAILDPRFKMKLVEYYYSQIYGSTALERIKEASDGIKELFNAYSICSTLIDQGSALPGSSLPSSSNDTRDKLKGFDKFLHETSQSQTAISDLEKYLDEPMFPRNCDFNILNWWRVHTPRYPILSMMARDVLGTPMSTVAQEFAFNAGGRILDSNQSSLPPDTRQALICTRDWLRTQSDDATTSSSHYALPLYVEAN	678	77.412	-2.5	6.277	-0.305
Gbar_D06G019130	GbD6	Gbar_D06G019130	57827962	57829998	MTMASSNTPIPVDDGFNEYESVVKRQKSTTSKVWDEMTKLECENKNELKAQCNHCKTIFSAKSSSETSHLRRHLNSCLKKVNKDIAQYIIATQPSPEGVPSIKIYKFDADECRQAISTFLVCGKHSFGTVEEPGFRYMMRIASPNFKNISRQTAIRDVLKYYAKERDHVKEELAKAPGLICLTSDNWNSEHTNDEYICITAHWVDKDWKLQKRIIRFRALFPPYDGLNIADELVLCLSQWGIDKKIFSITLDNASYNDVMVSCLKNCFRANQAILCDGVFFQVRCCAHILNLIVKAGLELADYVVCKIRNGIRYIKKSGIRRKRFYDVADKSFHLNVTKKLRQDVCVRWNSTYLMLESFLYYKDVLDYWGQRDKDYQMFALSSEEWRNVAILCKFLKVFYDVTCVFSGSNYPTANLYFRGVWKVYKLLIDIVKGPYSFLTPMVKQMQEKFNKYWAEYSLILSCAAILDPRYKLNYVQYCFKTIYGVHASDFVETILSNLRLLFDEYVKKSKSMSSSLAGSSNVSDKNPVDSGLDEHNDNSADFGGYFDESDDYKRYLNESSTRSEKSQLNIYLEESELELNSQIDVLDYWSKSSVRYNELSLLARDLLAIPISTVAFESAFSMGKKVITLIRSSLKPKTVQAVVCLDDWMRAKGFSAGNYYSHFIVVILYYFFINLII	1,139	131.775	2	6.611	-0.4
Gbar_A09G008820	GbA9	Gbar_A09G008820	55171820	55175529	MSTEPTSIKGSVTPPTSIDSENSGVGASSQANVTTGKRKATPQRSEVWSHFTKIINSEGASKAKCNYCQKEFCCDVKKNGTGSLKYHIGSCKKNSSNVVDPSQGQLVLPRKGVEEGEGHLSTWRFDQEACRKGLAQMIVIDELPFKFAESEGFKKFMFVACPRFHIPSRTTMTRDVYQLYLDERVKIKQLLRSSCSRVCLTTDTWTSLQRVNYLCITAHFIDNDWKLNKKILNFCPISSHKGESIGMVIEKCFLNWGIDKLFTVTVDNASSNDVAIGYLRKKFNPRGGLVQNGKYLHMRCMAHIVNLIVKEGLKEMNKSVERVRGAVRYVRQSPARLQKFKKCVVMEKIECKKMLCLDVCTRWNSTYLMLDTAQKFERAFERFEEQDTNFRAELERGEGWPNAQLNSNVGFNVMAIKMKEKYDKYWGDIDKMNLLMFVACILDPRQKLKYLEFALSEMSSSEKACEMMQKLKESLYELFDEYKPPLYSTCSRSSVPTHVSLGEPQQKMKRRMQALYKKRELEIGGEDKTSELDKYLAEANEDFIEDFDILLWWKMNSPRFQILSKMARDVLAIPVSTVASESTFSTGGRVLDQYRSSLTPKIVQALVCTQDWIRKSSSQEDIKKIEEQIQELDMIENDLEIMSSTLTSISLCIISWNKVTMAL	672	76.277	-3	6.214	-0.297
Gbar_D03G016590	GbD3	Gbar_D03G016590	48868436	48872364	MVRGRDACWEHCVLVDATRQKVRCNYCHREFSGGVYRMKFHLAQIKNKDIVPCAEVPDDVRDHIQSILNTPKKQKTPKKPKMDKTVANGQQNSSSASGGLHPNHGSSGQHGSTCPSLLFPHPSPSEQPATDDAQKQKLDDADKKIAVFFFHNSIPFSAAKSMYYQEMVDAIAECGVGYKAPSYEKLRSSLLEKVKGDIHDCYKKYREEWKETGCTVLCNSWSDGRTKSFVIFSVTYPKGTLFLKSVDVSGHEDDASYLFELLESVVLEVGLENVIQVITDSTASYVCAGRHLMAKYSSLFWSPCASYCIDKMLEDISKQEWVGIVLEEAKSIARYIYSHAWILNMMRKITGGRELMRPRITRFVDNYLTLRSIVIQEDNLKHMFSHSEWLSSIYSRRSDAQAIKSLLYLERFWKSAREAVSVSESLVKILRIVDGDMPAMGYMYEGIERAKGAIKAYYKGIEEKYMPIWDIIDRRWNMQLHSPLHAAAAFLNPSIFYNPNFKIDLRMRNGFQEAMLKMATMDKDKIEITKEHPVYINAQGALGTDFAIMGRTLNAPGDWWASYGYEIPTLQRVAIRILSQPCSFHWCRWNWSTFETVYTKKRNKVEMEKLNDLVFVHCNLWLQTICQGRDGKCKPIIFDEIDVSSEWPTESEPSVPLLDDSWLDNLPLECRGSP	748	84.532	14	8.104	-0.217
Gbar_D09G019140	GbD9	Gbar_D09G019140	45648211	45651626	MVRERDVCWEYAEKLDGNKVRCKFCLRVLNGGISRLKHHLSRLPSKGVNPCNKVRDDVTDRVRAIISSKEDIKETPSVKKQKIAEVRAPGNMSTSSKISPLETLSPAAKVFPTVLSIAASTLSDQETVERSIALFFFENKLDFSVARSSSYQAMIDAVGKFGPGLIAPSVETLKTTWLKRIKSEVTLHLKDAEKEWATTGCTIIADTWTDNKSKALINFLVSSPSRTFFHKSVDASSYFKNTKCLADLFDSVIQDFGQENVVQIIMDSSFNYTGISSHILQNYGTIFLSPCASQCLNLILEEFSRVDWVNRCILQAQTVSKFLYNNASMLDLMKKFTGGQELIRTGITKSVSCFLSLQSMLKQRSRLKHMFNSPEYSTNSSYANKPQSISCIAIVEDNDFWRAVEECVAISEPFLKVLREVSGGKPVVGSIYELMTRAKESIRTYYIMDESKCKTFLDIVDRQWRDQLHSPLHSAGAFLNPSIQYNPEVKFLGSIKEDFFKVLEKLLPTPELRRDITNQIFTFTRAKGMFACNLAMEARDTVSPGLWWEQFGDSAPVLQRVAIRILSQVCSTFMFERHWSTFQQIHTEKRNKIDKETLTDVVYINYNLKLAREMKTMPTESDPIQFDDIDMTSEWVEESENPSPTQWLDRFGSALDGGDLNTRQFSAAMFGNDHIFGL	635	72.824	7.5	7.356	-0.455

Gbar_D03G013660	GbD3	Gbar_D03G013660	44530279	44533352	MPPREEFPTKGLEGAPSNDIGWHFGTLVPNARGSIVCKLCGKVVKGGITRLKEHIAHKTGNVAPCPNVTGVIRESMMNVLKESNTKKIDKKRRKDEFLSQLIEEEDEHEGFIDEVSAIRQATRESIQSQHEWHRREEFRRSTGGWDNIYEEGRSSHGSAREHNRERTSKSILGESEFTLRGAIPELVRSKSSKQPKVNDSFLKSFRRKIGEAVSKFIIYERLPFQLASSPWLYNLIQLPTPYEVSDVYLESEYQRVHDWVNVLKTHWKELGATLMCDGWTNSLNQMHIINFLVYCSKGTIFWKSVDVSSVRSRDAEFYYRLLDSVVEEIGENYIVQIVTDNEAAMKAAGKKLMLKRQHLYWTSCAAHCLDLCLEDIGKKPSVAKVLDEAKKVTCFIYNHIWTVDLMKKYTQGKQILRPALTRFATHFIQLEEITRQKQGLREMFNSKEFKESKWGKQKSGPAYEAKKIVLGKDFWKKANDLIKVYEPLVRVLRLVDSDEKPTMGFIYEAVDRAKRAIQQNCRYFTEYEKIIDNRWNFMHSDLHSAGYFLNPQFQFGVEHSENVLIETLEGTRSVIERLEPSMDTQVRMVNQLLLFRDKHETFGTPQAQRAWKQMNPAEWWMIYGTCVPELQKLAIKVLSQTTSASNCERNWSTFSYIHTKARNRLKYKKLEKLVFTYYNMRLKMRHQQRMSTDDINASFNPISLDYIFEDVDPLSEWLHEKENPLLDGENAGVLPVDTSDDEMDVDQSQQQILSHSSSSSTPSQSGDGPDGGGLSPIDEDDGYSGDRGEIRSSSQYGGEYGVVPLVDIFVTDQSLMEICFLNLGEIEVNLELHQREKARSILL	900	103.354	12	7.495	-0.516
Gbar_A08G009840	GbA8	Gbar_A08G009840	41718084	41723968	MAEMTEATNMETSPVENNNELALITPETQPKRRKKKSMVWEYFTIETVSAGCRRACCNRCKQSFAYSTGSKVAGTSHLKRHIAKGTCPALLRDQYNNQLTPYSPKTGGGEPRKRRYRSPSSPFIPFDQDRCRHEIARMIIMHEYPLHMVEHPGFIAFVQNLQPRFDKVSFNTVQGDCVATYLREKQSLMKLIEGIPGRVCLTLDMWTSNQTLGYVFITGHFIDFEWKLQRRVLNVIMEPYPDSDSALSHAVAACLSDWSLEGKLFSLIFNHPTSEAGLENLRPLLCTKNPLILNGQLLLGNCIARNLSSMAKDVLGAGHEIVKKIRDSVKYVKTSESHDEKFVQVKNQLQVPSEKSLILDNQNQWNTTYQMLAAASELKEVFNCLDTSDPDYKLAPSMEDWKVAETLCTFLKPLFDAASILMTTTNPTAITFFHEAWKIHADLGRSIANDDPFISNIAKSMLEKIDKYWKDCSLILAIAVVMDPRFKMKLVEFSFTKIFGEDAPTYIKIVDDGIHELFLEYVALPLPLTPTYTEGNVGNNGKTDESQQGNLLSDQGLTDFDVYIMETSSQQMKSELDQYLEESLLPRVQEFDVLGWWKLNKMKYPTLSKMARDILSIPVSAAAPDSVFDIIIKQLDEYRSSLRPETVEALICAKDWLHHGSEESNALVKMEF	659	75.723	15	8.388	-0.399
Gbar_D08G010130	GbD8	Gbar_D08G010130	23217231	23219252	MAEITEATNMETTPVENNNELALITPETQPKRRKKKSMVWEYFTIETVSAGCRRACCNRCKQSFAYSTGSKVAGTSHLKRHIAKGTCPALLRDQYNNQLTPYNPKTGGSEPRKRRYRSPSSPFIPFDQDRCRHEIARMIIMHEYPLHMVEHPGFIAFVQNLQPRFDKVSFNTVQGDCVATYLREKQSLMKLIEGIPGRVCLTLDMWTSNQTLGYVFITGHFIDFEWKLQSRVLNVIMEPYPDSDSALSHAVAACLSDWSLEGKLFSLTFNHPTSEAGLENLRPLLCTKNPLILNGQLLLGNCIARTLSSMAKDVLGAEHEVVKKIRDSVKYVKTSESHDEKFVQVKNQLQVPSEKSLILDNQTQWNTTYQMLAAGSELKEVFNCLDTSDPDYKLAPSIEDWKVAETLCTFLKPLFDAASILTTTTNPTAITFFHEAWKIHADLGRSITNEDPFISNIAKSMLEKIDKYWKDCSLILAIAVVMDPRFKMKLVEFSFTKIFGEDAPTYIKIVDDGIHELFLEYVALPLPLTPTYTEEGNAGNNGKTDESQQGNLLSDQGLTDFDVYIMETSSQQMKSELDQYLEESLLPRVQEFDVLGWWKLNKMKYPTLSKMARDILSIPVSAAAPDSVFDIIIKQLDEYRSSLRPETVEALICAKDWLHYGSEESNALVKMEF	674	77.179	12	7.74	-0.38
Gbar_A07G012480	GbA7	Gbar_A07G012480	22713096	22717070	MSTEPTSIKGSVTPPTSIDSENLGVGASSQANVTTGKRKATPQRSEVWSHFTKIINSEGASKAKCNYCQKEFCCDVKKNGTGSLKYHIGSCKKNPSNVVDPSQGQLVLPRKGVERGEGHISTWRFDQEACRKGLAQMIVIDELPFKFVESEGFKKFMFVACPRFHIPSQTTMTRDVYQLYLDERVKIKQLLRSSCSRVCLTTDTWTSLQRVNYLCITAHFIDNDWKLNKKILNFCPISSHKGESIGMVIEKCLLNWGIDKLFTITVDNASSNDVAIGYLRKKFNPRRGLVQNGKYLHMRCMAHIVSLIVVEGLKEMNKSVERVRGAVRYVRQSPARLQKFKECVVMEKIECKKMLCLDVCTRWNLTYLMLDTAQKFERAFEKFEEQDTNFRVELERGEGWPSVDDWANVRNLRDFLEHFYEVTLRISGTSYVTSNNFFDELSEIDILLRDAQLNSNVDFNVMAIKMKEKYDKYWGDIDKMNLLMFVACVLDPRQKLKYLEFALSEMSSSEKACEMMQKLKESLYELFDEYKPPLYSTCSQSSVPTHVSLGEPQQKMKRRMQALYKKRELEIGGEDKTSELDKYLAEANEDFIEDFDILLWWKMNNPRFPILSKMARDVLAIPVSTVASESTFSTGGRVLDQYRSSLTPKIVQALVCTQD	748	84.872	3	6.851	-0.305
Gbar_A07G008770	GbA7	Gbar_A07G008770	12900777	12904796	MELNLVPISITRQKQDPAWNHCEVFKNGERIQIKCMYCGKLFKGGGIHRFKEHLAGRKGQGPICEQVPQGVRSIMQESLNGILVKQDKKQKLIPKLLACGSSSSNLNIGGEVENLGSHDDMNFGIKPISVLNTLEGDSNVVSKVGRGRKRGRDRDRNLIESNRPCLKTDLALVPNGGENPIHMAIGRFLYDIGVNLDAVNSVCFQPMIDAIASGGSGVVPPSCHDLRGWILKNVIEEVKDDIDRNKAMWGKTGCSIIVEQCRTKNGRVLLSFLVYCPQATVFMKSVDASHAIYSADYLFELLKQVIEEVGSENVVQVITNCEEPYLFTGKRLMESFPSLYWAPCLAHCVDLMLQDFSNLEWINETIEQAKSLTRFIYNQSSVLNTMRKFTSGNDVVEPALTCFATNFSTLRRMADLKLNLQAMVNSQDWLECPYAKEPGGQAMSDIVNNRSFWNSCVLIAHITYPLLRVLEIVGSKKRSAMGYVYAGIYRAKETIKKELVKQDDYMVYWNIIDNRWEQQRHLPLYAAGFFLNPKFFYNTKEHIHNDILSAVFDSIERLVPDTNIQDQVVREINLYKNAMGDLGRPMAVRARDNLLPGEWWSIYGGGCPNLQRLAIRILSQTCSSIGYKPNKISIEEIHNTRNFLERRRLSDLVFVQYNLYLRQMVLQNQEKDSLDPLVFNRKDILEDWIADNEVSPDNHESSDWKSLDPPVGNRTTLTPPGDEAEDFLSTRFMDLDIFNGLKGVKEEI	676	78.721	18	8.517	-0.301
Gbar_D07G009120	GbD7	Gbar_D07G009120	10732450	10736393	MELNLVPISITRQKQDPAWNHCEVFKNGERLQIKCMYCGKLFKGGGIHRFKEHLAGRKGQGPICEQVPQGVRSVMQESLNGILVKQDKKQKLIPKLLACGSSSSNPNIGGEVENLGSHDDMNFGIKPISVLNTLEGDSNVVSKVGRGRKRGRGRDWNLIESNYPCVKTDLALVPNGGDNPTHMAIGRFLYDIGVNLDVVNSVCFQPMIDAIASGGSGVVPPSCHDLRGWILKNVIEEVKDDIDRNKAMWGKTGCSIIVEQCRTKNGRVLLSFLVYCPQATVFMKSVDASHAFYSADYLFELLKQVIEEVGSEKVVQVITNCEEPYLFTGKRLMESFPSLYWAPCLAHCVDLMLQDFSNLEWINKTIEQAKSLTRFIYNQSLVLNMMRKFTSGNDVVEPALTCFATNFSTLKRMADLKLNLQAMVNSQDWLECPYAKKPGGQAMSDIVNNRSFWNSCMLIARITYPLLRVLEIVGSKKRSAMGYVYAGIYRAKETIKKELVKQDDYMVYWNIIDNRWEQQRHLPLYAAGFFLNPKLFYNTEEHIHNDFLSSVFDSIERLVPDTNIQDQVVREINLYKSATGDLGRPMAVRARDNLLPGEWWSIYGGGCPNLQRLAIRILSQTCSSIGYKPSKISIEEIHNTRNFLERQRLSDLVFVQYNLYLRQMVLQKQEKDSLDPLAFNNKDILEDWIADNEVSPDNLESSDWKSLDPPVGNRTTLTPPGDEAEDFLSTRFTDLDIFNGLKGVKEEI	843	97.18	6	6.952	-0.57

Gbar_D05G011800	GbD5	Gbar_D05G011800	9808536	9811206	MASSEVFINVRDHGKTVDVKKKRIKCNYCDKEMSGFSRLKYHLGGVRGNVLPCEKVPQDVKKLFRDMVQGREHLHNDAPYLYRQPFPQKRNGCPHNNVAKKTRHQSSESSGDESREYGNTDSMSEDDLEDSVASCKRMVSQKSGKQNKRCIGRFFFETGTDFKLVNSLSFQRLMNDIRGWILKDEVKEIQEYVQKIRQSWGNTGCRIFFIADCPEGPIYLHSCDVSASVDDVNTLQLLLDRVMYEVGAENVVQVIAFSTTGWVGDVGKQFMERWKSVFWTVNASHCIELLLDEVVNMGDVQRTLEKAKTISKFIHDHVTVLNLWRDYMDGHDLIKPTKIKSAVPFVTLENIISEKRNITAMFTSSAWNNTTWSSTVEGKRVAKLVGDASFWRGAGMVVKLTLPLIRVLCLMHGEDKPQMGYIYETIDQVKETIEGCNSRKSEYMPFWKAIDEIWDGHLHSPLHAAGYFFNPSFFYSTDFQSDFEVGFGLLCCMVPMWWSPYGGEYPELQRFATRILSQTCVGASKYRLNRSLAEKLLTKGRDRTEEQLLSDLTFVHYNLQLQQQHSQLGVNYDIVADEIGPMNEWIVDDTAEIGSDNGDSNWKDLKSAVNGEGPSMYTSNFKRYYLFKMKLSKKM	700	80.711	11.5	8.109	-0.41
Gbar_A06G005460	GbA6	Gbar_A06G005460	9554656	9558369	MSYLKLLFSYIYIYTHIHTYISWTTFLKFLFLSAAGFTSMTEMTEIADMETIPGESNNQLALTTPEAHPIKRRKKKSMVWEYFTIENVSAGCRRAYCKRCKQSFAYSTGSKVAGTSHLKRHIAKGTCRALLRGQGQDNNQFITPYNPKMGGSEPPKRRYRSPSSPFIPFDQDRCRHEIARMIIMHEYPLHIVEHPGFIAFVQSLQPQFDKMSFNTVQGDCVATYLREKQSLMKFIEGIPGRFCLTLDMWSSNQTLGYVFITGHFVDSDWKLHRQVFNVVMEPYPDSHSALSHAIAACLSDWSLEGKLFSLTFNHPLSEAGLENLRPLLCVKNPLILNGQLLIRNCIARTMSSMAKDVLGAGQEIIKKIRDSVKYVKMSESHDDKFIQVKNQLQVPSEKSLFLDNQTRWNTTYQMLAAASELKEVFDCLDTYDPDYKLAPSMEDWKLAETLCSFLKPLFDAASILTTTTLPTVITFFYEVWKIHVDLGRSITSEDPFISNLAKSMQEKIDKYWKDCSLVLAMAVVMDPRFKMKLVEFSFTKIYSEDAPTYIKTVDDGIHELFLEYVALPLPLTPTYAEEVNGANNGKTNESHYGNLLSDHGLTDFDVYIMETNSQQMKSELDQYLEESLLPRVQEFDVVGWWKLNKMKYPTLSKMARDILSIPVSAAAPESIFDITDKQLDEYRSSLRPETVEALICAKDWLHFGSSDVSNALVKMEF	678	77.384	1.5	6.652	-0.295
Gbar_A06G005450	GbA6	Gbar_A06G005450	9546728	9550872	MTEMTEIADMETIPGESNNQLALTTPEAQPIKRRKKKSMVWEYFTIENVSAGCRRAYCKRCKQSFAYSTGSKVAGTSHLKRHIAKGTCRALLRGQGQDNNQFITPYNPKMGGSEPPKRRYRSPSSPFIPFDQDRCRHEIARMIIMHEYPLHIVEHPGFIAFVQSLQPQFDKMSFNTVQGDCVATYLREKQSLMKFIEGIPGRFCLTLDMWSSNQTLGYVFITGHFVDSDWKLHRRVFNVVMEPYPDSHSALSHAIAACISDWSLEGKLFSLTFNHPLSEAGLENLRPLLCVKNPLILNGQLLIRNCIARTMSSMAKDVLGAGQEIIKKIRDSVKYVKMSESHDDKFIQVKNQLQVPSEKSLFLDNQTRWNTTYQMLAAASELKEVFDCLDTYDPDYKLAPSMEDWKLAETLCSFLKPLFDAASILTTTTLPTVITFFYEVWKIHVDLGRSITSEDPFISKLAKSMQEKIDKYWKGCSLVLAMAVVMDPRFKMKLVEFSFTKIYSEDAPTYIKTVDDGIHELFLEYVALPLPLTPTYAEEVNGANNGKTNESHYGNLLSDHGLTDFDVYIMETNSQQMKSELDQYLEESLLPRVQEFDVLGWWKLNKMKYPTLSKMARDILSIPVSAAAPESIFDITDKQLDEYRSSLRPETVEALICAKDWLHFGSSDVSNALVKMEF	658	76.08	-5.5	5.915	-0.342
Gbar_A07G007110	GbA7	Gbar_A07G007110	9107387	9109688	MSTEPTSIEGSITPPTSIDSENSRIRASSQAKGTTGKRKVTPQRSEVWSHFTKIINSEGASKAKCNYCQKEFCCDMKKNGTRSLKYHIGSCKKNPSNVIDTRGRHLSTWRFDQEACRKGLTQMIVIDELPFKFVESEGFKKFMFVACPRFHIPSRTTMTRDVYQLYLNERVKIKQLLKSSCSRVCLTTDTWTSLQSVNYLCITAHFIDNDWKLNKKILNFCPISSHKGESIGMVIEKCLLNWGIDKLFTVTVDNASSNNVAIGYLRKKFNPRGGLVQNGRYLPMRCMAHIVNLIVGAVRYVRQSPARLQKFKECVVVEKIECKKMLFLDVCTRWNSTYLMLDTAQNFERAFKRFEEQDTNFRAELKRGEGWPSVDDWDNVRNLRDFLEHFYEVTLRISGTSYVTSNNFFDELSEIDILLRDVQLNSNVDFNVMTIKMKEKYDKYWGDIDKMNLLMFVACVLDPRKKLKYLKFALSEMSSSEKACEMMQKLKESLYELFDEYKPPLHSTCSQLSVPTHRELEICGEDKTSELDKYLAEANEEFVENFDILLWWKVNSPRFPTLSKMAKDVLAIPVSTVASESAFSTGRRVLDQYRSSLTPKIVQALVCTQDWIRKLLSQEDIKKIEEQIQELDKIENGIFIDMLTFLFLLTYNLFVCLYYLIFFKCVYFYYMLNFFL	717	82.066	2	6.672	-0.238
Gbar_D06G005670	GbD6	Gbar_D06G005670	7621396	7625517	MTEMTEITDMETIPGESNNQLALTTPEAQPIKRRKKKSMVWEYFTIENVSAGCRRAYCKRCKQSFAYSTGSKVAGTSHLKRHIAKGTCRALLRGQGQDNNQFITPYNPKMGGSEPPKRRYRSPSSPFIPFDQDRCRHEIARMIIMHEYPLHIVEHPGFIAFVQNLQPQFDKMSFNTVQGDCVATYLREKQSLMNFIEGIPGRFCLTLDMWSSNQTLGYVFITGHFVDSDWKLHRRVFNVVMEPYPDSHSALSHAIAACLSDWSLEGKLFSLTFNHPLSEAGLENLRPLLCVKNPLILNGQLLIRNCIARTMSSMAKDVLGAGQEIIKKIRDSVKYVKMSESHDDKFIQVKNQLQVPSEKSLFLDNQTQWNTTYQMLAAASELKEVFDCLDTYDPDYKLAPSMEDWKLAETLCSFLKPLFDAASILTTTTLPTVITFFYEVWKIHVDLGRSITCEDPFISNLAKSMQEKIDKYWKDCSLVLAMAVVMDPRFKMKLVEFSFTKIYGEDAPTYIKTVDDGIHELFLEYVALPLPLTPTYAEEVNGANNGKTNESHQGNLLSDHGLADFDVYIMETNSQQMKSELDQYLEESLLPRVQEFDVLGWWKLNKMKYPTLSKMARDILSIPVSAAATESIFDITDKQLDEYRSSLRPETVEALICAKDWLHYGSSDVSNALVRMEF	1,001	115.749	10.5	7.23	-0.542
Gbar_D06G005660	GbD6	Gbar_D06G005660	7578934	7582966	MTEMTEITDMETIPGESNNQLALTTPEPQPIKRRKKKSMVWEYFTIENVSAGCRRAYCKRCKQSFAYSTGSKVAGTSHLKRHIAKGTCRALLRGQGQDNNQFITPYNPKMGGSEPPKRRYRSPSSPFIPFDQDRCRHEIARMIIMHEYPLHIVEHPGFIAFVQNLQPQFDKMSFNTVQGDCVATYLREKQSLMNFIEGIPGRFCLTLDMWSSNQTLGYVFITGHFVDSDWKLHRRVFNVVMEPYPDSHSALSHAIAACLSDWSLEGKLFSLTFNHPLSEAGLENLRPLLCVKNPLILNGQLLIRNCIARTMSSMAKDVLGAGQEIIKKIRDSVKYVKMSESHDDKFIQVKNQLQVPSEKSLFLDNQTQWNTTYQMLAAASELKEVFDCLDTYDPDYKLAPSMEDWKLAETLCSFLKPLFDAASILTTTTLPTVITFFYEVWKIHVDLGRSITCEDPFISNLAKSMQEKIDKYWKDCSLVLAMAVVMDPRFKMKLVEFSFTKIYGEDAPTYIKTVDDGIHELFLEYVALPLPLTPTYAEEVNGANNGKTNESHQGNLLSDHGLADFDVYIMETNSQQMKSELDQYLEESLLPRVQEFDVLGWWKLNKMKYPTLSKMARDILSIPVSAAATESIFDITDKQLDEYRSSLRPETVEALICAKDWLHYGSSDVSNALVRMEF	1,077	124.582	-11.5	5.755	-0.389
Gbar_D04G004060	GbD4	Gbar_D04G004060	5449486	5454214	MDMSDAVIVNSSRLKSIVWNDFDRVKKGDTFVAICRHCKKKLSGSSTSGTSHLRNHLIRCQRRSNHGVAQYFSAKDKKKEGSLALVTIDQEQKNDEVLSIVNLRYEQEQIKSEHVAIGSNSLDQRRSQFDLARMIILHNYPLAMVEHVGFKIFVRNLQPLFELVTRNKVEADCMEIYAKEKQKVYEIFDKLPGKISVSADVWTASEDDAAYLSLAAHYIDENWQLKKKNLNFVTIDPSYAEDMHSEVIMNCLMDWDIDRKLFSMIFDSFTSDNIVERIRDRLSQNRFLHCNGQLFDVRCAVDLLNRMAHDALETLCEITQKIRESIRYVKSSEATQATFNELADEVQVETKKCLCIDNPLKWNSTYLMLEAASEYRKVFSCLRDRDPVNMKFLLSDPEWDRLITVTSFLKLFVEVTNVFTRSKYPTANIFFPEICDIHLQLIEWCKNPDEYISSLALKMRKKFEEYWYKCSSGLAVA	691	79.503	10	7.697	-0.326

					AMLDPRFKMKLLEYYYPQLYGDSATELIDDVFECIKSLYNEHSMVSPLASSIDQGLDWQASGIPGSGKDSRDRLMGFDKFLHETSQAEGSSSDLDKYLEEPLFPRNVDFNVLNWWKVHTPRYPILSMMARNILGIPISKVAAESRFDTGGRMLNHNWSSLPPTTIQALMCSRDWIRSELES					
Gbar_A03G001880	GbA3	Gbar_A03G001880	2268671	2272252	MVRGRDACWEHCVLVDATRQKVRCNYCHREFSGGVYRMKFHLAQIKNKDIVPCAEVPDDVRDHIQSILNTPKKQKTPKKPKMDKTVANGQQNSSSASGGLHPNHGSSGQHGSTCPSFLFPHPSPSEQPATDDAQKQKLDDADKKIAVFFFHNSIPFSAAKSMYYQEMVDAIAECGVGYKAPSYEKLRSSLLEKVKGDIHDGYKKYREEWKETGCTVLCNSWSDGRTKSFVIFSVTYPKGTLFLKSVDVSGHEDDASYLFELLESVVLEVGLENVIQVITDSTASYVCAGRHLMAKYSSLFWSPCASYCIDKMLEDISKQEWVGIVLEEAKTIARYIYSHAWILNMIRKFTGGRELMRPRITRFVDNYLNLRSIVFQEDNLKHMFSHSEWLSSIYSRRSDAQAIKSLLYLERFWKSAREAVSVSESLVKILRIVDGDMPAMGYIYEGIERAKGAIKAYYKGIEEKYMPIWDIIDRRWNMQLHSPLHAAAAFLNPSIFYNPNFKIDLRMRNGFQEAMLKMATMDKDKIEITKEHPVYINAQGALGTDFAIMGRTLNAPGDWWASYSYEIPTLQRVAIRILSQPCSFHWCRWNWSTFETVHTKKRNKVEMEKLNDLVFVHCNLWLQTICQGRDGKCKPIIFDEIDVSSEWPTESEPSVPLLDDSWLDNLPLECRGSP	703	80.579	12.5	8.091	-0.391
Gbar_A13G002040	GbA13	Gbar_A13G002040	2110025	2114630	MSSNLEPIPITSQKHDPAWKHCQMFKNGERVQLKCIYCGKIFKGGGIHRIKEHLAGHKGNAATCLRVPSDVRVLMQESLDGVVVKKRKKQKIAEEITNVNQVSTEIQAYGDQVDTNTGLLMIEKSDTLEPSSSLLVNREGTSNVAGERRKRGRGKNLPAEANALSFVPVELGARRVNNHVHMAIGRFLFDIGATMDAVNSVYFQPMVDAIVSGGSGALMPSCNDLQGWILRKLVEEVKSENDKVMGAWVRTGCSILVNQWNTQTGRILLNFLVYCPEGTVFLKSIDASSVINSSDALYELLKQVVEEVGSKHVLQVITNGEEQYIVAGRRLAETFPTLYWTPCAAHCVDLILEDFAKLEWINAIIEQARSITKFIYNHSVVLNMVRRYTFGNDIVEPAATRSATNFTTLTRMVDLKNNLQAMVTSQQWVDCPYSKKPGGLAMLDLVSNQSFWSSCILIVRLTNPLLRVLRMVGSKKRPAMGYVYAGMYRAKETIKKELVKRNEYMVYWNIIDHWWEQQWHHPLHAAGFYLNPRFFYSMEGDMPNEMLSGMLDCIEKLIPDVTVQDKISKEINSYKNSVGDFGRKMAVRARDTLLPVEWWSTYGGSCPNLARLAIRVLSQTCSTFGLKHNHIPFEKLYETRNCLEQQRLRDLIFVQCNLQLRQIGYESKQHDSMQPLSSESASIVEDWVTGIDAFLDDDTYPDWTTLETLSVNTMLLRPGDEYLITVRFAQGGPLFPNCSKYAALGKHL	780	89.064	7	6.996	-0.724
Gbar_D02G001280	GbD2	Gbar_D02G001280	11218115	11282899	MNETKSDPNKKFTFSIQSNSCYSKDLNSYKFMRSCEYKIEQTIIEKALESKDGYHFEPSLTKSDIDDYMYLQRAKNCDVDVVVAICLQNRYTSNDVYVVEFYWPATESEISKSFTPRIFNDLKHMEKKFVTVKVQGTEKAISNIPTSSYTARPLKIAEETEDVDAVEINGVNVQRGVVPNFPSPITIQSSSKVVAAPSNTLEEPHNQIFPNGDPEIVRANKEEPSKATQRELRSKVWGHFDSFEEDEKQVAKCKHCPKVLTGSSKSGTTHLNNHSKVCPGKKKQNQESQLILPVDTNEGSLRFDKKRSHMDLAKMMIKLQCPLDMAEQETFKNFVKGLQPMFEFQSKDILSYIHRIYDEEKEKLQLYFDKLASKFNLTVSLLKNNSGKTIYCCLISHFIDDGWELKRKILALKILEHINDTKALGEIIRSLVLEWNISNKVCSITVDNSFLNDSMVDQIKEICLSDQGSVSSDHWFISFTLLEDGFREMDGILFKLRKSIEYVTETRHGKLKFQEAVDQVKLQGGKLWDDLSFRLESDFDILDSALRSREIFCKLEQIDDNFKLNPTMEEWENAVALQSCLKCFDDIKGTQCLPVSLYLPKLCDTYKKFLQLEKSSHSFVKLMKRKFDRYWSLCNLALAVASVLDPRLKFKIVELSYRVIYGHDSKMRLNMFQKVLRDVYYEYASEAKNLTSSASVLDDFNCSTIGLGNDSILDSLSKFASASNFNEEASWKLELELYLDEPLLPMDGAFFDILGWWCDKSQRFPILAKMAQDFLAIPVSISTSCSNISAMINNPAYSTLNPESMEALVCSENWLETPKER	672	76.97	3.5	6.848	-0.333
Gbar_D02G001040	GbD2	Gbar_D02G001040	7980735	8025177	MSTEPTSIEGSVTPPTSIDSKNSGVGASSQTKGTTGKRKATPQRSEVWSYFTKIINSEGASKAKCNYCQKEFCCDMKKNGIGSLKYHIGSCKKNPSNVQGQLVLPRKRVEGGEENLSTWRFDQETCRKGLAQMIVIDELTFKFVESESFKKFMFVACPRFHIPSRTTMIRDVYQLYLDERVKIKQLLRSSCSRVCLTTDTWTSLQRVNYLCITAHFIDNDWKLNKRILNFCPISSHKGESIGMVIEKCLLNWGIDKLFTVTVDNASLNDVAIGYLRKKFNLRGGLVQKGKYLHMRCMAHIMNLIVVEGLKEMNKSVERVRGAVRYVRQFPARLQKFKECVVVEKIECKKTLCLDVCTRWNSTYSMLDTALNFERAFERFEEQDTNFRAELERGEGWPSVDDWDNVRNLRDFLEHFYEVTLRISGTSYVTSNNFFDELSEIDILLRDAQLNSNVAFNVMAIKMKEKYDKYWGDIDKINLLMFVACVLDPRQKLKYLEFVLSEMSSSEKVCEMMQKLKGSLYELFDEYKPPLHSTCSQSSVPTHVSLGEPQQKMQRRIQALYKKCELEICGEDKTSELDKYLAEANEGFVEDFDILLWWKVNSPRFPTLSKMARDLLAIPVSTVASESAFSTGGRVLDQYRSSLTPKIVQALVCTQDWIRKSSSQEDIKKIEEQIQELDKIENGLMQLEIFWKEKMDTNGEY	128	14.789	6	9.245	-1.052
Gbar_D11G000680	GbD11	Gbar_D11G000680	6255574	6298558	MEWSVNNAFKSYKDMEPKSTMDMVLIPNMDTIDIVLGSSEKGNVVPSAKPRKKTMTSVYLKYFETAPDGKTRRCKFCGQSYSIATATGNLGRHLSNRHPGYDKTGENVTSSAPQPSTTPTVIKKPQQQGRAPQVDYDHLNWLLIKWLILATLPPSTLEEKWLANSFKFFNPSIQLWPGEKYKAVFREVFRSMREDVRASLEQVSSKVSIALDFWTSYEQIFYMSITCQWIDENWSFRKVLLDICQVPYPCSDSEIYNSLVKVLKMYNIENKVLSCTHDNSQNAIHACHALKEDLDGQKMGPFCFIPCAARTLSLIIDDALRTTKPVIAKVREFVQELNASLDISEDFIQLTTAYKEGSWQFPLDASARWSGSYQMLDLVQKAGKSMDAIVRKNEEMLGNRMLLNTAEKNVVNIVHNYLEPFYKVISEICVNTPPTIGMVIVYMDHISDTITTRQPPDWLKNPAEDMAKKLRSYNNQVCNIFIYMTAILDPRIKCELIPESLNSENYLEEARAHFVRNYYTTPFSSMTSGYSSQDIEDGGAVSFAEEIARKKRRASMSNATDELTQYLSESPAPTKTDVLEWWKVNSTRYPRLSAMARDFLAVQATSVKPDELFCSKGDEIDKQRFCMPHDSTQAILCIKSWTQGGLKLKYKSTEIDYERLMEMAAAAAADISSAGIDKKQK	678	77.438	-2.5	6.277	-0.31
Gbar_D02G000700	GbD2	Gbar_D02G000700	5254335	5304401	MDNFDQKLGPEFFKNLSAEAVTPLNVVHEEIYESSSKRPKTTSKVWDIFEKLPAQQGDSKAICKLCRRIYTAKTTSGTSHLRRHIEACVKRGNHEVDQRSIEACFKPVKRNANRLTLSHDTLIAATTSLKNYKLDVDEIHRAIAMMIIVDEQPFSVVEDAGFRRLLSAACPEFPVLSRSSIKRDIISIYVKERENIRELLATCPGRICLTSSTWKSDSDDHFNCVTTHFIDHEWRLQKRILSFKLMPPPYDSLSVADEIALCMVQWNIEHKVFSVTLENLSSDDCVADMLRSRLAAKKYLPCKGVFFHVSCFFRILNSIVQAGLNLVVDIIAKLRLGIKYVQQSPHRKKNFYIIAKTLNLDTQRKLCLDTPARWNSTYNMIEVAFCYNNAFMYLAEQDKNFLHKLSEDEWEKLSVLYKFLKVFYEVTCVFFRNRQPTSNLYFKAAWKVHSRLFDMVRGPENFMTRMVREMHSKLNQYWSAYNLILSCAAVLDPRCKIKFVEYCYTKLYGSGAQKYVSVSVNTLYGLFDEYMQNSARPSQTTLLSTAASKISNDKDNNDGFEDYETFQSARFRTQVEKSQLDLYLEEPSHDLNSEIDVLEYWTLCSLRYPELSKMARDVLTIPVSTIASDSAFDISPQVISTDRSSLKPKMLQALVCLQDWMLASDRTRGLGSMESKPEDDSSSSSDGDDDY	681	77.251	4	7.183	-0.36

Gbar_A02G000610	GbA2	Gbar_A02G000610	426160	430416	MDNFDQKLGPEFFKNLSAEAVTPLNVVHEEIYESSSKRPKTTSKVWDIFEKLPAQQGDSKAICKLCRRIYTAKTTSGTSHLRRHIEACVKRGNHEVDQRSIEACFKPVKRNANRLTLSHDTLISATTSLKNYKLDVDEIHRAIAMMIIVDEQPFSVVEDAGFRRLLSAACPEFPVLSRSSIKRDIISIYVKERENIRELLATCPGRICLTSSTWKSDSDDHFNCVTTHFSDHEWRLQKRILRFKLMPPPYDSLSVADEIALCMVQWNIEHKVFSVTLENLSSDDCVADMLRSRLAAKKYLPCKGVFFHVSCFFRILNSIVQAGLNLVVDIIAKLRLGIKYVQQSPHRKKNFYIVAKTLNLDTQRKLCLDTPARWNSTYDMIEVAFCYKNAFVYLAEQDKNFLHKLSEDEWEKMSVLYKFLKVFYEVTCVFFRNRQPTSNLYFKAAWKVHSRLFDMVRGPENFMTRMVREMHSKLNHYWSAYNLILSCAAILDPRYKIKFVEYCYTKLYGSGAQKYVSVSVNTLYGLFDEYMQNSARPSQTTLLSTAASKISNDKDENDGFEDYETFQSARFRTQVEKSQLDLYLEEPSHDLNSEIDVLEYWTLCSLRYPELSKMARDVLTIPVSTIASDSAFDITPQVISADRSSLKPKMLQALVSLQDWMLASDRTRGLGSMESKPEDDSSSSSDGDDDY	691	79.615	10.5	7.739	-0.349
Gbar_A11G000320	GbA11	Gbar_A11G000320	326960	331238	MEWSVNNAFKSYKDMEPKSTMDMVLIPNMDTIDIVLGSSEKGNVVPSAKPRKKTMTSVYLKYFETAPDGKTRRCKFCGQSYSIATATGRHLSNRHPGYDKTGENVSSSAPQLSTTPTVIKKPQPQGRAPQVDYDHLNWLLIKWLILATLPPSTLEEKWLANSFKFLNPSIQLWPGEKYKAVFREVFRSMREDVRASLEQVSSKVSIALDFWSSYEQIFYMSITCQWIDENWSFQKVLLDICQVPYPCTGSEIYNSLVKVLKMYNIENKVLSCTHDNSQNAIHACHALKEDLDGQKMGPFCFIPCAARTLSLIIDDALRTTKPVIAKVREFVQELNASLDISEDFIQLATAYKEGSWQFPLDASARWSGSYQMLDIVQKAGKSMDAVVRKNEEMLGNRMLLNTAEKNVVNIVHNYLEPFYKVISEICVNTPPTIGMVIVYMDHISDTITARQPPDWLKNPAEDMAKKLRSYNNQVCNIFIYMTAILDPRIKCELIPESLNSENYLEEARAHFVRNYYTTPFSSMTSGYSSQDIEDGGAVSFAEEIARKKRRASMSNATDELTQYLSESPAPTKTDVLEWWKVNSTRYPRLSAMARDFLAVQATSVKPDELFCSKGDEIDKQRFCMPHDSTQAILCIKSWTQGGLKLKYKSTEIDYERLMEMAAAAAADISLAGMDKKQK	663	75.9	18.5	8.624	-0.407

Table S19 List of GhZF-BED genes and their features

Chromosome	Gene_ID	Start	End	Protein_sequence	Protein Length (aa)	Molecular Weight (kDa)	Charge	Isoelectric Point	Grand Average of Hydropathy
GhA8	Ghir_A08G024610	120957667	120961437	MEVANETVIKKPKRLTSVVWNHFERVRKADLCYAVCVHCNKKLSGSSNSGTTHLRNHLMRCLKRFNYDVSQLLSAKKRKKDNTLTIANISYDEGQRKEEYLKPTIVKYEPEQRKDEVFNVQSSWFDQERSRLDLARMIILHGYPLAMVEHVGFKVFVKNLQPLFDVVPNSTVELSCMEIYGKERQKVHDMLSKLQGRINLAVEMWSSPENTNHVCMMAHYVGDDWKLQKKILNFVTLDSSHTDDLLSGVIIKCLMDWDIGSKLFAMTLDDFSTNDDIVLRIKEQISENKSRLSNGQLLDVRSAVHVLNSIVQDAMEALRLVIQKIRGTVRYVKSSQSIQGKFKEMVLQTGINSQKNLVLDCPIQWNSTYLMLETAIEYRNAFCQLPELDLDLALSDEEWEWASSITGYLKLFIEIINVFSSNKCPTANIYFPEICHVHIQLIDWCKSPDNFLSSLAAKMKAKFDKYWSKCSLSLAVAAILDPRFKMKLVEYYYSQIYGSTALERIKEVSDGLKELFNTYSICSTLMDQGSALPLSSLPSSSNDGRDRLKGFDKFLHETSQSQNAISAWKYLDEPVFPRNCNFNILNWWRVHTPRYPILSMMARDVLGTHVNRLTRVSIPRWR	622	71.804	17	8.541	-0.287
GhA11	Ghir_A11G031630	116728011	116732464	MEVANESTAKKPKRLTSVVWNHFERVKKANICYAVCVHCNKKLSGSSNSGTTHLRNHLMRCLKRSNYDVSQLLAVKRRKKENTLTIANISYDEGQRKEDYMKPTIVKYEQDQRKDEAFNLGSSWFDPERSRLDLARMIILHGYPLAMVEQVGFKVFVKNMQPLFDVVHNSTIELSCVEIYMKEKQRIYDILSKLQGRINLAIEMWSSPENSKYVCLTAHYVDDEWKLQKKILNFLTLDSSHTEDMLSDVIIKCLMDWDIDCKLFAMTFDDCSTNDDIVSRIKDQVSESRPRLSNGQLLDVRSAAHVLNSIAQDAIEALQVVIQKIRGSVKYVKSSQSILGKFNEIAQQQGIDNHKIVVLDYPIRWNSTYMMLETAVEYRNVFHHLPELDPDFALSDEEWERASSIVSYLKLLIEIINVFSSNKCPTANIYFPEICHVHIQLIEWCKSSDAFLSSLATKMKAKFDKYWSKCSLALAVAAILDPRFKMKLVEYYYSQIYGSTALERIKEASDGIKELFNAYSICSTLIDQGSALPGSSLPSSSNDTRDRLKGFDKFLHETSQSQTAISDLEKYLDEPVFPCNCDFNILNWWRVHTPRYPILSMMARDVLGTPMSTVAQEFAFNAGGRMLDSNQSSPPPDTQQALICTRDWLRTQSDDATPSSSHYALPLYVEAN	672	76.93	3.5	6.846	-0.323
GhA5	Ghir_A05G039010	102699209	102703926	MDMSDAVIVNSSRLKSIVWNDFDRVKKGDTFVAICRHCKKKLSGSSTSGTSHLRNHLIRCQRRSNHGVAQYFSAKDKKKEGSLALVTIDQEQKNDEVLSIVNLRYEQEQIKSEHVGIGSNSLDQRRSQFDLARMIILHNYPLAMVEHVGFKIFVRNLQPLFELATRNKVEADCMEIYAKEKQKVYEIFDKLPGKISVSADVWTASEDDAAYLSLAAHYIDENWQLKKKNLNFVTIDPSYTEDMHSEVIMNCLMDWDIDRKLFSMIFDSFTSDNIVERIRDRLSQNRFLYCNGQLFDVRCAVDLLNRMAHDALEALCEITQKIRESIRYVKSSEATQATFNELADEVQVETKKCLCIDNPLKWNSTYFMLEAALEYRKVFSCLRDRDPVNMKFLLSDPEWDRLITVTSFLKLFVEVTNVFTRSKYPTANIFFPEICDIHLQLIEWCKNPDEYISSLALKMRKKFEEYWYKCSSGLAVAAMLDPRFKMKLLEYYYPQLYGDSATELIDDVFECIKSLYNEHSIVSPLASSIDQGLDWQASGISGSGKDSRDRLMGFDKYLHETCQAEGSSSDLDKYLEEPLFPRNVDFNVLNWWKVHTPRYPILSMMARNILGIPISKVAAESRFDTGGRVLDHNWSSLPPTTIQALMCSQDWIRSGLES	658	76.036	-3.5	6.144	-0.339
GhA2	Ghir_A02G014680	98819881	98821955	MSTKPTSIEGSVTPPTSIDSENSGVGASIQTKGTTGKRKAPPQRSEVWSHFTKFINSEGASKAKCNYCEKEFCCDMKKNGTGSLKYHIGSCKKNPSNVVDTRNISTWRFDQEACRKGLAQMILIDELPFKFVESEGFKKFMFVACPRFHIPSRTTMTRDVYQLYLDERVKIKQLLRSSCSRVCLTTDTWTSLQRVNYLCLTAHFIDNDWKLNKKILNFCPISSHKGESIGMVIEKCLLNWGIDKLFTVTVDNASSNDVAIGYLRKKFNPRGGLVQNGKYLHMRCMAHIVNLIVVEGLKEMNKSVERVRGLLQKFKECIVVEKIECKKMLCLDVCTRWNSTYLMLDTAQNFERAFERFEEQDTNFRAELERERVGLVEMSSSEKACEMMQKLKESLYELFDEYKPPLHSTCSQSSVSTHVSIGEPQQKMKRRMQALYKKRELEICGEDKTSELDKYLAEANEEFVEDFDILLWWKVNSPRFPTLSKIARDVLAIPVSTVASESAFSTGGRVLDQYRSSLTPKIVQALVCTQDWIRRSSSQEDIKKIEEQIQELDKIENGMFIVLF	564	64.703	14.5	8.398	-0.415
GhA12	Ghir_A12G013020	82539958	82543276	MVEEMAPLRSIGYVDPGWEHGTAQDERKKKVKCNYCGKVVSGGIFRLKQHLARLSGEVTHCEKVPEEVCLNMRKNLEGCRSGRKRRQFDYEQAALSIQSNEYSDGEDASASYKHKGKKVMGDKNLVIKFTPLRSLGYVDPGWEHCVAQDEKKRRVKCNYCEKIISGGINRFKQHLARIPGEVAYCEKAPEEVYLKIKENMKWHRTGRRHRPDTKEISTFYMHSDNEDEGGEEEGYLQCVSKDILAIDDKVSDNDIRNNVRGRSPGSSGNGAEPLLKKSRLDSVFLKSLKSQTSAHYKQPRARTGFEKKTHREVISAICKFFYHAGIPSNAANSPYFHKMLELVGQYGQGLQGPSSRLISGRLLQEEIANIKEYLVELKTSWAITGCSVMADSWNDAQGRMLINFLVSCPRGVYFLSSVDATDIIEDAVHLFKLLDKAVDEVGEEYVVQVITRNTLSFRNAGKMLEEKRRNLFWTPCAVYCIDRMLEDFVNIKWVGECVDKAKKVTRFIYNNTWLLNFMKKEFTKGQELLQPAVTKFGTNFFTLQSLLDQRVGLKRMFQSNRWLSSRFSKSDEGKEVEKIVLNVSFWKKMQYVKKSFEPVAEVLQRIGSDKSDHAVYIYIHGDDVRKYGPFWSVIESNWSSLFHHPLYVAAYFLNPSYRYRPDFLMNPEVIRGLNECIVRLEADNGKKIAASMQIPDFVSAKADFGTDLAISTRSELDPASWWQQHGISCLELQRIAIRILSQTCSSIGCEHNWSAFDQVHIKRHNCLSRKRLNDQTYVHYNLRLRERQLGRKPDELVSFDSAMLESVL	888	101.838	7.5	7.033	-0.522

				DDWLVETEKLAMHEDEEIIYTEVEQFCGDDMDEHESEEKRPAEMVTIAGFIEPLDVIPSAGGV TTDDDGLDFLDDDLTD					
GhA11	Ghir_A11G023640	70017247	70017633	MPCSRNKEEVAPSDDYGWRWGELVEGNHNNVKCRFCGRIIKRGITQLKEYLAVKKGNVAPC PHGSVKVRKSIGQQLQEYHIEKAVRQRRKEELEERISLGDRGNYGDSGDDDEELTITRRKSV RSQVE	128	14.742	5.5	8.933	-1.06
GhD8	Ghir_D08G025490	67158310	67161330	MEVANETVIKKPKRLTSVVWNHFERVRKADLCYAVCVHCNKKLSGSSNSGTTHLRNHLMRC LKRFNYDVSQLLSAKKRKKENTLTIANISYDEGQRKEEYLKPTIVKYEPEQRKDEVFNVQSSW FDQDRSRLDLARMIILHGYPLAMVEHVGFKVFVKNLQPLFDVVPNSTVELSCMEIYGKERQKV HDMLSKLQGRINLAVEMWSSPENTNHVCMMAHYIGDDWKLQKKILNFVTLDSSHTDDLLSG VIIKCLMDWDIGSKLFAVTLDDFSTNDDIVLRIKEQILENKSRLSNGQLLDVRSAAHVLNSIVQD AMEALRVVLQKIRGTVRYVKSSQSIQGKFKEMVLQTGINSQKNLVLDCPIRWNSTYLMLETAI EYRNAFCQLPDLDLDLALSDEEWEWASSITGYLKLFVEIINVFSSNKCPTANIYFPEICHVHIQL IDWCKSPDNFLSSLAAKMKAKFDKYWSKCSLSLAVAAILDPRFKMKLVEYYYSQIYGSTALER IKEVSDGLKELFSTYSICSTLMDQGSALPLGSLPSSSNDGRDRLKGFDKFLHETSQSQTAISD LEKYLDEPVFPRNCNFNILNWWRVHTPRYPILSMMARDVLGTPMSTVSQESAFHAGGRVLD SCRCPLTPETRQALICTQDWLRIQSDDPGPSSSHYALPLYVETN	672	76.944	9.5	7.609	-0.28
GhD11	Ghir_D11G032010	66356920	66361443	MEVANESTAKKPKRLTSVVWNHFERVKKADICYAVCVHCNKKLSGSSNSGTTHLRNHLMRC LKRSNYDVSQLLAVKRRKKENTLTIANISYDEGQRKEDYMKPTIVKYEQDQRKDEAFNLGSS WFDPERSRLDLARMIILHGYPLAMVEHVGFKVFVKNMQPLFDVVHNSTIELSCVEIYMKEKQR IYDMLSKLQGRINLAIEMWSSPENSKYVCLTAHYVDDEWKLQKKILNFLTLDSSHTEDMLSDVI IKCLMDWDIDCKLFSMTFDDCSTNDDIVLRIKDQISESRPRLSNGQLLDVRSAAHVLNSIAQDA IEALQVVIQKIRGSVKYVKSSQSILGKFNEIAQQQGINNHKIVVLDYPIRWNSTYMMLETAVEY RNVFHHLPELDPDFALSDEEWKRASSIVSYLKLLIEIINVFSSNKCPTANIYFPEICHVHIQLIEW CKSSDAFLSSLATKMKAKFDKYWSKCSLGLAVAAILDPRFKMKLVEYYYSQIYGSTALERIKE ASDGIKELFNAYSICSTLIDQGSALPGSSLPSSSNDTRDKLKGFDKFLHETSQSQTAISDLEKY LDEPMFPRNCDFNILNWWRVHTPRYPILSMMARDVLGTPMSTVAQEFAFNAGGRILDSNQS SLPPDTRQALICTRDWLRTQSDDATPSSSHYALPLYVEAN	672	77.081	8	7.363	-0.329
GhD6	Ghir_D06G019310	61373003	61375039	MTMASSNTPILVNDGFNEYESVVKRQKSTTSKVWDEMTKLECENKNELKVQCNHCKTIFSAK SSSETSHLRRHLNSCLKKVNKDIAQYIIATQPSPEGVPSIKNYKFDADECRQAISTFLVCGKHS FGTVEEPGFRYMMRIASPNFKNISRQTAIWDVLKYYAKERDHVKEELAKAPGLICLTSDNWN SEHTNDEYICITAHWVDKDWKLQKKIIRFRALFPPYDGLNIADELVLCLSQWGIDKKIFSITLDN ASYNDVMVSCLKNYFRANRAILCDGAFFQVRCCVHILNLIVKAGLELADYVVCKIRNGIRYIKK SGIRRKRFYDVADKSFHLNVTKKLRQDVCVRWNSTYLMLESFLYYKDVLDYWGQRDKDYQ MFALSSEEWRNVAILCKFLKVFYDVTCVFSGSNYPMANLYFRGVWKVHKLLIDIVKGPYSFLT SMVKQMQEKFNKYWAKYSLILSCAAILDPRYKLNYVQYCFKTIYGVHASDFVETILSNLRLLFD EYVKKSKSMSSSLAGSSNVSDKNPVDSGLDEHNDNSADFGGYFDESDDYKRYLNESSTRSE KSQLDIYLEESELELNSQIDVLDYWSKSSVRYNELSLLARDLLAIPISTVASESAFSMGKKVITPI RSSLKPKTVQAVVCLDDWMRAKGFSAGNYYSRFIVVILYYFFINLII	678	78.442	19	8.592	-0.263
GhD11	Ghir_D11G027530	58862079	58865506	MSIEPTSIEGRVTPPTSIDSENSGVGASIQTKGTTGKRKAPPQRSAVWSHFTKFINSEGASKA KCNYCEKEFCCDMKKNGTGSLKYHIGSCKKNPSNVVDTSQGQLVLPRKGVEGGEGNLSTW RFDQEACRKGLAQMILIDELLFKFVESEGFKKFMFVACPRFHIPPRTIMTRDVYQLYLDERVKI KKLLRSSCSRVCLTTDTWTSLQRVNYLCITAHFIDNDWKLNKKILNFCPISSHKGESIGMVIEK CLLNWGIDKLFTITVDNASSNDVAIGYLRKKFNPRGGLVQNGKYLHMRCMAHIVNLIVVEGLK EMNKSVERVRGAVRYVRQSPARLQKFKECVVVERIECKKMLCLDVCTRWNLTYLMLDTAQN FERALKRFEEQDTNFRAELERGEGWPSVDDWDNVRKLRDFLEHFYEVTLRISGTSYLNSNID FNVMAIKMKEKYDKYWGDIDKMNLLMFVACVLDPRQKLKYLEFALSEMSSSEKACEMMQKL KESLYELFDEYKPPLHSTCSQSSSSRELEICGEDKTSELDKYLAEANEEFVEDFDILLWWKVN SPRFPTLSKIARDVLAIPVSTVASEFAFNTGGRVLDQYRSSLTPKIVQALVCTQDWIRRSSSQE DIKKIKEQIQELDKIENGIQYMFVIHFMTYLGIINSWGDWTIIG	669	76.981	14	8.287	-0.358
GhD13	Ghir_D13G017940	54847433	54849449	MNFNLLYFFSCIRSIKPTSIEGSVTPPTSIDSENSGVGASSQTKGTTGKRKATQRSEVWSHFT KIINSEGASKAKCNYCQKEFCCDMKKNGTGSLKYHIGSCKKNPSNVVDNSQGQLVLPRKETC RKGLAQMIVIDELPFKFVESESFKKFTFVACPRFHIPSRTTMTRDVYQLYLDEKIKIKQLLRSSC SRVCLTTDTWTSLQRVNYLCITTHFIDNDWKLNKKILNFCPISSHKGESIGMVIEKCLLNWGID KLFIVTVDNASSNHVAIGYLRNKFNPRGGLVQNGKYLHMRCMAHIVNLIVVQGLKEMNKSVE RVRGLIQKFKECVVVEKIECKKMLCLDVCTRWNLTYLMLDTAQNFERAFERFEEQDTNFRAE LERGEGWPSVDDWDNVRNLRDFLEHFYEVTLRISGTSYVTSNNFFDELSEIDILLRDAQLKSN VDFSVMAIKMKEKYDKYWGDIDKMNLLMFVACILDPRQKLKYLKFALSEMSSSEKACEMMQK LKESLYELFDEYKPPLHSTCSQLSVPTHVFLGEPQQKMKRRMQALYKKRELEICGEDKTSEL DKYLAEANEEFVEDFEILLWWKVNSPRFPTLSKMARDVLAIPVSTVASESAFSTRGRVLDQY RSSLTPKSVQALACTQDWI	646	74.523	18.5	8.526	-0.361
GhD3	Ghir_D03G017110	50004036	50007681	MVRGRDACWEHCVLVDATRQKVRCNYCHREFSGGVYRMKFHLAQIKNKDIVPCAEVPDDV RDHIQSILNTPKKQKTPKKPKMDKTVANGQQNSSSASGGLHPNHGSSGQHGSTCPSLLFPH PSPSEQPATDDAQKQKLDDADKKIAVFFFHNSIPFSAAKSMYYQEMVDAIAECGVGYKAPSY EKLRSSLLEKVKGDIHDCYKKYREEWKETGCTVLCNSWSDGRTKSFVIFSVTYPKGTLFLKS VDVSVMRIASYLFELLESVVLEVGLENVIQVITDSTASYVCAGRHLMAKYSSLFWSPCASYCID KMLETLVNKVGGIVLEEAKSIARYIYSHAWILNMMRKITGGRELMRPRITRFVDNYLTLRSIVIQ EDNLKHMFSHSEWLSSIYSRRSDAQAIKSLLYLERFWKSAREAVSVSESLVKILRIVDGDMPA MGYMYEGIERAKVL	451	51.115	18	8.847	-0.294
Gh	Ghir_D0	439	439	MSTEPTSIEGSVTPPTSIDSENSGVGASIQTKGTTRKRKSPPQRSEVWSHFTKFINSEGASKA KCNYCEKEFCCDMKKNGTGSLKYHIGSCKKNPSNVVDTSQGQLVLPRKGVEGGEGNLSTW RFDQETCRKGLAQMILIDELPFKFVESEGFKKFMFVACPRFHIPSRTTMTRDVYQLYLDERVK	679	78.2	9	7.8	-0.4

D1	1G015550	72661	75937	IKQLFRSSCSRVCLTTDTWTSLQRVNYLCITAHFIDNDWKLNKKILNFCPISSHKGESIGMVIEKCLLNWGIDKLFTVTVDNASSNDVAIVQNGKYLHMRCMAHIVNLIVVEGLKEMNKSVERVRGAVRYVRQSPARLQKFKECVVVEKIECKKMLCLDVCTRWNSTYLMLDTTQNFERAFERFEEQDTNFRAELERGEGWPSVDDWDNVRKLRDFLEHFYEVTLRISGTSYVTSNNFFDELSEIDILLRDAQLNSNIDFNVMAIKMKEKYDKYWGDIDKMNLLMFVACVLDPRQKLKYLEFALSEMSSSEKACEMMQKLKESLYELFDEYKPPLHSTCSQSSSSVSTHVSIGEPQQKMKRRMQALYKKRELEICGEDKTSELDKYLAEANEEFFEYFDILLWWKVNSPRFPTLSKIARDVLAIPVSTVASESAFSTGGRVLDQYRSSLTPKIVQALVCTQDWIRRSSSQEDIKKIEKQIQELDKIENGCWIL		79		41	42
GhA8	Ghir_A08G009690	43167380	43171512	MAEMTEATNMETSPVENNNELALITPETQPKRRKKKSMVWEYFTIETVSAGCRRACCNRCKQSFAYSTGSKVAGTSHLKRHIAKGTCPALLRDQYNNQLTPYSPKTGGGEPRKRRYRSPSSPFIPFDQDRCRHEIARMIIMYEYPLHMVEHPGFIAFVQNLQPRFDKVSFNTVQGDCVATYLREKQSLMKLIEGIPGRVCLTLDMWTSNQTLGYVFITGHFIDFEWKLQRRVLNVIMEPYPDSDSALSHAVAACLSDWSLEGKLFSLIFNHPTSEAGLENLRPLLCTKNPLILNGQLLLGNCIARNLSSMAKDVLGAGHEIVKKIRDSVKYVKTSESHDEKFVQVKNQLQVPSEKSLILDNQNQWNTTYQMLAAASELKEVFNCLDTSDPDYKLAPSMEDWKVAETLCTFLKPLFDAASILMTTTNPTAITFFHEAWKIHADLGRSIANDDPFISNIAKSMLEKIDKYWKDCSLILAIAVVMDPRFKMKLVEFSFTKIFGEDAPTYIKIVDDGIHELFLEYVALPLPLTPTYTEEGNVGNNGKTDESQQGNLLSDQGLTDFDVYIMETSSQQMKSELDQYLEESLLPRVQEFDVLGWWKLNKMKYPTLSKMARDILSIPVSAAAPDSVFDIIIKQLDEYRSSLRPETVEALICAKDWLHHGSEESNALVKMEF	673	76.433	-4.5	6.028	-0.299
GhD12	Ghir_D12G013240	43026876	43032264	MVEEMAPLRSIGYVDPGWEHGTAQDERKKKVKCNYCGKVVSGGIFRLKQHLARLSGEVTHCEKVPEEVCLNMRKNLEGCRSGRKRRQLDYEQAALSIQSNEYSDGEDASASYKHKGKKVMGDKNLVIKFTPLRSLGYVDPGWEHCVAQDEKKRRVKCNYCEKIISGGINRFKQHLARIPGEVAYCEKAPEEVYLKIKENMKWHRTGRRHRKPDTKEISTFYMHSDNEDEGGEEEGYLQCISKDILAIDDKVSDNDIRNNVRGRSPGSSGNGAEPLLKKSRLDSVFLKSLKSQTSTHHKQPRARTGFEKKTHREVISAICKFFYHAGIPSNAANSPYFHKMLELVGQYGQGLQGPSSRLISGRLLQEEIANIKEYLVELKTSWAITGCSVMADSWNDAQGRMLINFLVSCPRGVYFLSSVDATDIIEDAVHLFKLLDKAVDEVGEEYVVQVITRNTLSFRNAGKMLEEKRRNLFWTPCAVYCIDRMLEDFVNIKWVGECVDKAKKVTRFIYNNTWLLNFMKKEFTKGQELLQPAVTKFGTNFFTLQSLLDQRVGLKRMFQSNRWLSSRFSKSDEGKEVEKIVLNVSFWKKMQYVKKSFEPVAEVLQRIGSDKIRSLPFIYNDICRTKLAIKAIHGDDVRKYGPFWSVIESNWSPLFHHPLYVAAYFLNPSYRYRPDFLMNPEVIRGLNGCIVRLEADNGKKIAASMQIPDFVSAKADFGTDLAISTRSELDPASWWQQHGISCLELQRIAIRILSQTCSSIGCEHNWSAFDQVHIKRHNCLSRKRLNDQTYVHYNLRLRERQLGRKPDELVSFDSAMLESVLDDWLVETEKLAMHEDEEIIYTEVEQFCGDDMDEHESEEKRPAEMVTIAGFIEPLDVIPSAGGVTTDDDGLDFLDDDLTD	900	103.129	12.5	7.504	-0.51
GhA5	Ghir_A05G030910	39914397	39917469	MPPREEFPTKGLEGAPSNDIGWHFGTLVPNARGSIVCKLCGKVVKGGITRLKEHIAHKTGNVAPCPNVTGVIRESMMNVLKESNTKKIDKKRRKDEFLSQLIEEEDEHEGFIDEVSAIRQATRESIQSQHEWHRREEFRRSTGGWDNIYEEGRSSHGSAREHNRERTSKSILGESEFTLRGAIPELVRSKSSKQPKVNDSFLKSFRRKIGEAVSKFIIYERLPFQLASSPWLYNLIQLPTPYEVSDVYLESEYQRVHDWVNVLKTHWKELGATLMCDGWTNSLNQMHIINFLVYCSKGTIFWKSVDVSSVRSRDAEFYYRLLDSVVEEIGENYIVQIVTDNEAAMKAAGKKLMLKRQHLYWTSCAAHCLDLCLEDIGKKPSVAKVLDEVKKVTCFIYNHIWTVDLMKKYTQGKQILRPALTRFATHFIQLEEITRQKQGLREMFNSKEFKESKWGKQKSGPAYEAKKIVLGKDFWKKANDLIKVYEPLVRVLRLVDSDEKPTMGFIYEAVDRAKRAIQQNCRYFTEYEKIIDNRWNFMHSDLHSAGYFLNPQFQFGVEHSENVLIETLEGTRSVIERLEPSMDTQVRMVNQWWMIYGTCVPELQKLAIKVLSQTTSASNCERNWSTFSYIHTKARNRLKYKKLEKLVFTYYNMRLKMRHQQRMSTDDINASFNPISLDYIFEDVDPLSEWLHEKENPLLDGENAGVLPVDTSDDEMDVDQSQQQILSHSSSSSTPSQSGDGPDGGGLSPIDEDDGYSGDRGEIRSSSQYGGEYGVVPLVDIFVTDQSLMEICFLNLGEIEVNLELHQREKARSILL	816	94.013	4.5	6.846	-0.554
GhD11	Ghir_D11G020300	24150836	24152911	MSTKPTSIEGSVTPPTSIDSENSGVGASIQTKGTTGKRKAPPQRSEVWSHFTKFINSEGASKAKCNYCEKEFCCDMKKNGTGSLKYHIGSCKKNPSNVVDTSQGQLVLPRKGVEGGEGNISTWRFDQEACRKGLAQMILIDELPFKFVESEGFKKFMFVACPRFHIPSRTTMTRDVYQLYLDERVKIKQLLRSSCSRLNLCLTAHFIDNDWKLNKKILNFCPISSHKGESIGMVIEKCLLNWGIDKLFTVTVDNASSNDVAIGYLRKKFNPRGGLVQNGKYLHMRCMAHIVNLIVVEGLKEMNKSVERVRGLLQKFKECVVVEKIECKKMLCLDVCTRWNSTYLMLDTAQNFERAFERFEEQDTNFRAELERGEGWPSVDDWDNVRNLRDFLEHFYEVTLRISGTSYVTSNNFFDELSEIDILLRDAQLNSNIDFNVMAIKMKEKYDKYWGDIDKMNLLMFVACVLDPRQKLKYLEFALSEMSSSEKACEMMQKLKESLYELFDEYKPPLHSTCSQSSVSTHVSIGEPQQKMKRRMQASYKKHELEICGEDKTSELDKYLAETNEEFVEDFDILLWWKVNSPRFPTLSKIARDVLAIPVSTVASESAFSTGGRVLDQYRSSLTPKIVQALVCTQDWIRRSSSQEDIKKIEEQIQELDKIENGMFIVLF	668	76.667	6.5	7.496	-0.421
GhD8	Ghir_D08G009750	23456204	23458225	MAEITEATNMETTPVENNNELALITPETQPKRRKKKSMVWEYFTIETVSAGCRRACCNRCKQSFAYSTGSKVAGTSHLKRHIAKGTCPALLHDQYNNQLTPYNPKTGGSEPRKRRYRSPSSPFIPFDQDRCRHEIARMIIMHEYPLHMVEHPGFIAFVQNLQPRFDKVSFNTVQGDCVATYLREKQSLMKLIEGIPGRVCLTLDMWTSNQTLGYVFITGHFIDFEWKLQSRVLNVIMEPYPDSDSALSHAVAACLSDWSLEGKLFSLTFNHPTSEAGLENLRPLLCTKNPLILNGQLLLGNCIARTLSSMAKDVLGAGHEVVKKIRDSVKYVKTSESHDEKFVQVKNQLQVPSEKSLILDNQTQWNTTYQMLAAGSELKEVFNCLDTSDPDYKLAPSIEDWKVAETLCTFLKPLFDAASILTTTTNPTAITFFHEAWKIHADLGRSITNEDPFISNIAKSMLEKIDKYWKDCSLILAIAVVMDPRFKMKLVEFSFTKIFGEDAPTYIKIVDDGIHELFLEYVALPLPLTPTYTEEGNAGNNGKTDESQQGNLLSDQGLTDFDVYIMETSSQQMKSELDQYLEESLLPRVQEFDVLGWWKLNKMKYPTLSKMARDILSIPVSAAAPDSVFDIIIKQLDEYRSSLRPETVEALICAKDWLHYGSEESNALVKMEF	673	76.299	-6	5.886	-0.303
GhA7	Ghir_A07G012610	23076597	23080402	MSTEPTSIKGSVTPPTLIDSENSGVGASSQANVTTGKRKATPQRSEVWSHFTKIINSEGASKAKCNYCQKEFCCDVKKNGTGSLKYHIDSCKKNPSNVVDSSQGQLVLPRKGVEGGEGHISTWRFDQEACRKGLAQMIVIDELPFKFVEIEGFMKFMFVACPRFHIPSQTTMTRDVYQLYLDERVKIKQLLRSSCSRVCLTTDTWTSLQRVNYLCITAHFIDNDWKLNKKILNFCPISSHKGESIGMVIEKCLLNWGIDKLFTVTVDNASSNDVAIGYLRKKFNPRRGLVQNGKYLHMRCMAHIVNLIVVEGLKEMNKSVERVRGLLDM	329	37.059	18	9.313	-0.305

GhA13	Ghir_A13G007540	20086078	20086725	MLLIYFNLLYNFFSCRISTEPTSIEGSVTPPTSIDSENSGVGASSQANVTIGKRKATPQRSEVWSHFTKIINSEGASKAKCNYCQKEFCCDVKRNGTGSLKYHIGACKKNPSNVVDTSQGQLVLPRNGVEGGEGHLSTWRFDQEACRKGLAQMIVIDELPFKFVESEGFKKFMFVACLRFHIPSQTTMTRDVYQLYLDERVKIKQLLKVLVLEFA	215	24.138	8	8.734	-0.244
GhD8	Ghir_D08G008310	12833829	12835063	MSTEPTSIEGNVTPPTSIDSENLGVGASSQTKGTTWNRKATPQKSEVWSHFTKIIISEDASKVKCNYCQKEFCCDMKRNVIGSLKYHIGSCKKNPSNVVDTSQRQLVLPRKGRFDQEACRKGLAQMIVIDELPFKFVESEGFKKFIFVAYPRFHIPSRTNMIRDVYQLYLDERVKIKQLLRSSCSRICLTIDTWTSLQRVNYLCITAHFIDNDWKLNKKILNFFPISSHKGESIGMVIEKCLLNWGIDKLFTITVHNASSNDVAIGYLRKKFNPRGGLVQNGKYLHMRCMAYILNLIVVEGLKEMNKSVERIRGAVRYVRQSPARLQKFKECVVAEKIKCKKMLCLDVCTRWNLTYLMLNTAQNFERAFEIFEKQDTNFRAELERERVGLVWMIGIMLET	400	46.207	25.5	9.785	-0.282
GhA7	Ghir_A07G008970	12686257	12690271	MELNLVPISITRQKQDPAWNHCEVFKNGERIQIKCMYCGKLFKGGGIHRFKEHLAGRKGQGPICEQVPQGVRSIMQESLNGILVKQDKKQKLIPKLLACGSSSSNLNIGGEVENLGSHDDMNFGIKPISVLNTLEGDSNVVSKVGRGRKRGRGRDRNLIESNRPCLKTDLALVPNGGENPIHMAIGRFLYDIGVNLDAVNSVCFQPMIDAIASGGSGVVPPSCHDLRGWILKNVIEEVKDDIDRNKAMWGKTGCSIIVEQCRTKNGRVLLSFLVYCPQATVFMKSVDASHAIYSADYLFELLKQVIEEVGSENVVQVITNCEEPYLFTGKRLMESFPSLYWAPCLAHCVDLMLQDFSNLEWINETIEQAKSLTKFIYNQSSVLNTMRKFTSGNDIVEPALTCFATNFSTLRRMADLKLNLQAMVNSQDWLECPYAKEPGGQAMSDIVNNRSFWNSCVLIAHITYPLLRVLEIVGSKKRSAMGYVYAGIYRAKETIKKELVKQDDYMVYWNIIDNRWEQQRHLPLYAAGFFLNPKFFYNTKEHIHNDILSAVFDSIERLVPDTNIQDQVVREINLYKNAMGDLGRPMAVRARDNLLPGEWWSIYGGGCPNLQRLAIRILSQTCSSIGYKPNKISIEEIHNTRNFLERRRLSDLVFVQYNLYLRQMVLQNQEKDSLDPLVFNRKDILEDWIADNEVSPDNHESSDWKSLDPPVGNRTTLTPPGDEAEDFLSTRFMDLDIFNGLKGVKEEI	748	84.8	4	6.977	-0.3
GhA6	Ghir_A06G005660	9953835	9958683	MDYYILYSHCLFQLKLSLGLQRRFLGFNSDPRLLLFGFLTAGTDDPEAHPIKRRKKKSMVWEYFTIENVSAGCRRAYCKRCKQSFAYSTGSKVAGTSHLKRHIAKGTCRALLRGQGQDNNQFITPYNPKMGGSEPPKRRYRSPSSPFIPFDQDRCRHEIARMIIMHEYPLSHTQFDKMSFNTVQGDCVATYLREKQSLMKFIEGIPGRFCLTLDMWSSNQTLGYVFITGHFVDSDWKLHRQVFNVVMEPYPDSHSALSHAIAACLSDWSLEGKLFSLTFNHPLSEAGLENLRPLLCKLYSSYYEQHMAKDVLGAGQEIIKKIRDSVKYVKMSESHDDKFIQVKNQLQVPSEKSLFLDNQTRWNTTYQMLAAASELKEVFDCLDTYDPDYKLAPSMEDWKLAETLCSFLKPLFDAASILTTTTLPTIITFFLKCGKFMWTWVQPCKINARKIDKYWKDCSLVLAMAVVMDPRFKMKLVEFSFTKIYSEDAPTYIKTVDDGIHELFLEYVALPLPLTPTYAEEVNGANNGKTNESHYGNLLSDHGLTDFDVYIMETNSQQMKSELDQYLEESLLPRVQEFDVVGWWKLNKMKYPTLSKMARDILSIPVSAAAPESIFDITDKQLDEYRSSLRPETVEALICAKDWLHFGSSDVSNALVKMEF	660	75.93	8	7.362	-0.318
GhA6	Ghir_A06G005650	9947674	9949681	METIPGESNNQLALTTPEAQPIKRRKKKSMVWEYFTIENVSAGCRRAYCKRCKQSFAYSTGSKVAGTSHLKRHIAKGTCRALLRGQGQDNNQFITPYNPKMGGSEPPKRRYRSPSSPFIPFDQDRCRHEIARMIIMHEYPLHIVEHPGFIAFVQSLQPQFDKMSFNTVQGDCVATYLREKQSLMKFIEGIPGRFCLTLDMWSSNQTLGYVFITGHFVDSDWKLHRWVFNVVMEPYPDSHSALSHAIAACLSDWSLEGKLFSLTFNHPLSEAGLENLRPLLCVKNPLILNGQLLIRNCIARTMSSMAKDVLGAGQEIIKKIRDSVKYVKMSESHDDKFIQVKNQLQVPSEKSLFLDNQTRWNTTYQMLAAASELKEVPSMEDWKLAETLCSFLKPLFDAASILTTTTLPTVITFFYEVWKIHVDLGRSITSEDPFISNLAKSMQEKIDKYWKDCSLVLAMAVVMDPRFKMKLVEFSFTKIYSEDAPTYIKTVDDGIHELFLEYVALPLPLTPTYAEEVNGANNGKTNESHYGNLLSDHGLTDFDVYIMETNSQQMKSELDQYLEESLLPRVQEFDVLGWWKLNKMKYPTLSKMARDILSIPQLDEYRSSLRPETVEALICAKDWLHFGSSDVSNALVKME	640	73.229	6.5	7.226	-0.301
GhD5	Ghir_D05G011710	9898762	9901432	MASSEVFINVRDHGKTVDVKKKRIKCNYCDKEMSGFSRLKYHLGGVRGNVLPCEKVPQDVKKLFRDMVQGREHLHNDAPYLYRQPFPQKRNGCPHNNVAKKTRHQSSESSGDESREYGNTDSMSEDDLEDSCCCYGDPNQESGKQNKRCIGRFFFETGTDFKLVNSLSFQRLMNDIRGPGWILKDEVKEIQEYVQKIRQSWGNTGCRIFFIADCPEGPIYLHSCDVSASVDDVNTLQLLLDRVMYEVGAENVVQVIAFSTTGWVGDVGKQFMERWKSVFWTVNASHCIELLLDEVVNMGDVQRTLEKAKTISKFIHDHVTVLNLWRDYMDGHDLIKPTKIKSAVPFVTLENIISEKRNITAMFTSSAWNNTTWSSTVEGKRVAKLVGDASFWRGAGMVVKLTLPLIRVLCLMHGEDKPQMGYIYETIDQVKETIEGCNSRKSEYMPFWKAIDEIWDGHLHSPLHAAGYFFNPSFFYSTDFQSDFEVGFGLLCCMVRMIQNQILSQTCVGASKYRLNRSLAEKLLTKGRDRTEEQLLSDLTFVHYNLQLQQQHSQLGVNYDIVADEIGPVNEWIVDDTAEIGSDNGDSNWKDLKSAVNGEGPSMYTSNFKRYHLFKMKLSKKM	622	71.132	4	6.877	-0.459
GhA7	Ghir_A07G007310	9297323	9299624	MSTEPTFIEGSITPPTSIDSENSRIRASSQAKGTTGKRKVTSQRSEVWSHFTKIINSEGASKAKCNYCQKEFCCDMKKNGTRSLKYHIGSCKKNPSNVIDTRGRHLSTWRFDQEACRKGLTQMIVIDELPFKFVESEGFKKFMFVACPRFHIPSRTTMTRDVYQLYLNERVKIKQLLKISCSRVCLTTDTWISLQSVNYLCITAHFIDNNWKLNKKILNFCPISSYKGESIGMVIEKCLLNWGIDKLFTVTVDNASSNNVAIGYLRKKFNSRGGLVQNGRYLHMRCMAHIVNLIVGAVRYVRQSPARLQKFKECVVVEKIECKKMLFLDVCTRCNSTYLMLDTAQNFERAFKRFEEQDTNFRAELKRGEGWPSVDDWDNVRNLRDFLEHFYEVTLRISGTLYVTSNNFFDELSEIDILLRDVQLNSNVDFNVMTIKMKEKYDKYWGDIDKMNLLMFVACVLDPRKKLKYLEFALSEMSSSEKACEMMQKLKESLYELFDEYKPPLHSTCSQLSVPTHRELEICGEDKTSELDKYLAEANEEFVENFDILLWWKVNSPRFPTLSKMAKDVLAISVSTVASESAFSTGRRVLDQYRSSLTPKIVQALVCTQDWIRKSLSQEDIKKIEEQIQELDKIENGIFIDMLTFLFLLTYNLFVCLYYLIFFKCVYFYYMLNFFL	676	78.772	17	8.425	-0.271
GhD6	Ghir_D06G00	81440	81477	MQSAGFTSMTEMTEITDMETIPGESNNQLALTTPEPQPIKRRKKKSMVWEYFTIENVSAGCRRAYCKRCKQSFAYSTGSKVAGTSHLKRHIAKGTCRALLRGQGQDNNQFITPYNPKMGGSEPPKRRYRSPSSPFIPFDQDRCRHEIARMIIMHEYPLHIVEHPGFIAFVQNLQPQFDKMSFNTVQGDCVATYLREKQSLMNFIEGIPGRFCLTLDMWSSNQTLGYVFITGHFVDSDWKLHRRVFNVVMEPYPDSHSALSHAIAACLSDWSLEGKLFSLTFNHPLSEAGLENLRPLLCVKNPLILNGQLLIRNCIARTMSSMAKDKIRDSVKYVKMSESHDDKFIQVKNQLQVPSEKSLFLDNQTQWNTTYQM	676	77.238	-2.5	6.276	-0.321

	5680	20	53	LAAASELKEVFDCLDTYDPDYKLAPSMEDWKLAETLCSFLKPLFDAASILTTTTLPTVITFFYEVWKIHVDLGRSITCEDPFISNLAKSMQEKIDKYWKDCSLVLAMAVVMDPRFKMKLVEFSFTKIYGEDAPTYIKTVDDGIHELFLEYVALPLPLTPTYAEEVNGANNGKTNESHQGNLLSDHGLADFDVYIMETNSQQMKSELDQYLEESLLPRVQEFDVLGWWKLNKMKYPTLSKMARDILSIPVSAAATESIFDITDKQLDEYRSSLRPETVEALICAKDWLHYGSSDVSNALVRMEF					
GhD4	Ghir_D04G004090	5891689	5896393	MDMSDAVIVNSSRLKSIVWNDFDRVKKGDTFVAICRHCKKKLSGSSTSGTSHLRNHLIRCQRRSNHGVAQYFSAKDKKKEGSLALVTIDQEQKNDEVLSIVNLRYEQEQIKSEHVAIGSNSLDQRRSQFDLARMIILHNYPLAMVEHDGFKIFVRNLQPLFELVTRNKVEADCMEIYAKEKQKVYEIFDKLPGKISVSADVWTASEDDAAYLSLAAHYIDENWQLKKKNLNFVTIDPSYAEDMHSEVIMNCLMDWDIDRKLFSMIFDSFTSDNIVERIRDRLSQNRFLHCNGQLFDVRCAVDLLNRMAHDALETLCEITQKIRESIRYVKSSEATQATFNELADEVQVETKKCLCIDNPLKWNSTYLMLEAASEYRKVFSCLRDRDPVNMKFLLSDPEWDRLITVTSFLKLFVEVTNVFTRSKYPTANIFFPEICDIHLQLIEWCKNPDEYISSLALKMRKKFEEYWYKCSSGLAVAAMLDPRFKMKLLEYYYPQLYGDSATELIDDVFECIKSLYNEHSMVSPLASSIDQGLDWQASGIPGSGKDSRDRLMGFDKFLHETSQAEGSSSDLDKYLEEPLFPRNVDFNVLNWWKVHTPRYPILSMMARNILGIPISKVAAESRFDTGGRMLNHNWSSLPPTTIQALMCSRDWIRSELES	658	76.135	-3	6.217	-0.366
GhD2	Ghir_D02G002620	3008558	3016452	MDYSFSDTALRLLLFCAEAIENRDLKSADAFLLVILILADKRHYWFRDDSIVVKYFAYALVSRAYGLHPASSYFTFPVDPAPYYQYNSCHINGVIKKVIDDALMGNRRLHLIDFNIPYYGFEGSVLSTLPNFFCDRLRVRVSYILPPFLKEYVEFSRQMEFLTEDAKEVNVELEDELKVVYGNSLAEVDECEIDFKRRRDDEMVVVYYKFKLEKLVRDAKAMKRELVRLKEINPTIVIILDFYSNHSDSDFLTCFKDSFQYSLKTLDYWQELDRYLDGKKEWEFNIEAGEGNNIIRRHPTLTEWQHLFSTAGFSRIPLNHRKDNLSVEDNSFLKIMREEEECLILGYKGCPMFFLSAWKPKVEDGHFNSNSTNYKFDQGFNPNPLPLQPLQPFPEGSILNRLAALAEIHNISKDLCCKYKLSLALTWASKVNNMNGTISDPNKKHTFFIQSNYCYVKDRKSYDFMFGFERMISVPIFEKAFESRDGYHFEPSLTEVEDFKYFMLKDCNIDVALAICLQNLHTSDEVYVVEFYWPPTESEISKSLALRIFDDLKHMKTTFVTVKVQGPEIKFQEEAISSIPTSSNTAMPLKIAEEARGIRAKEINAHIEQIVETKRNKQRKLRSKVWVDFHKSEEEGKQVAKCKHCPKVLTGSSKSGTTHLNNHSKVCPGKKKQNQESQLILPVDTNERSSTFDQERSHLALVKMVIRQQYPLDLAGQEAFKNFVKGLQPMYEFQSRDKLLSDIHRIYNEEREKLQLYFDQLACKLNLTVSLSKNNHGKTAYCCLIAHFIDDSWELKMKTLGLRTLEHINDTKAVGGIIQSLVSEWNIGNKVCSITVDNSFLDDSMVQQIKENCLSNLVSLSSTHWFINCTLLEDGFREMDDLLFKLKKSIEYVTETKHGRLKFQEAVDQVKLQDGKSWDDLSLKLASDFGILDSALRSREIFCKLEQIDGNFKLNPSMEEWENAAALQSCLRCFDDIKGTQSLTVSLYLLKLCDIYMKFLQLEKSNPSFVTLMKRRFDHYWRLCNSALAVASVLDPRLKFKVVEFSYKVIYGHDSKVQLNTFREVLTNVYNEYANETKNQTTSASVLDDINWPGNNSIWDSFSKFVTASEASSKSELELYLDEHLIPMDGAIFDILGWWSDKSQMFPILAKMARDFLAIPVSIFIPCSNIKATINNPAYNILNPESMEALVCSENWLETPKGNDGENHEPTQTMDKGKRKLDEDTCVRKKSKPSNCEKAISTEDIDKDSNNNDEPAGEISIGKLQTENSSRNGCYGETSSGNKSKASNKMMGTISLRAIHQEKSSSELNHGRNVEDVSSGDSSSDNDQSDQLQSSSSESDVEITLKEQGSWSEQDIKAYLLSEFTEKENELIDKWQKNELKGKMIGRDKYFKIQGEKLAPLLMVPQGDETREEYYIEDLVVNTFFELLKKRSDKFPNVYINHYSFGSQIATQLIEGPRTEQEVLAWVKVDELRGVHKMFLPMSLSKHWVLFYVDTKEKKISWLDPIASSRIRSYNVEKDIILQWFTTLLLPKLGYVDAKEWPFLVRNDIPEQKNLVDCAVFVMKYGDCLTHGDYFPFKQEDMVHFRRRIFVDIYRGRIHKKKTKIDALFRNSKL	1,614	186.598	-6	6.27	-0.445
GhA3	Ghir_A03G001860	2879623	2883248	MVRGRDACWEHCVLVDATRQKVRCNYCHREFSGGVYRMKFHLAQIKNKDIVPCAEVPDDVRDHIQSILNTPKKQKTPKKPKMDKTVANGQQNSSSASGGLHPNHGSSGQHGSTCPSFLFPHPSPSEQPATDDAQKQKLDDADKKIAVFFFHNSIPFSAAKSMYYQEMVDAIAECGVGYKAPSYEKLRSSLLEKVKGDIHDGYKKYREEWKETGCTVLCNSWSDGRTKSFVIFSVTYPKGTLFLKSVDVSGHEDDASYLFELLESVVLEVGLENVIQVITDSTASYVCAGRHLMAKYSSLFWSPCASYCIDKMLEDISKQEWVGIVLEEAKTIARYIYSHAWILNMIRKFTGGRELMRPRITRFVDNYLNLRSIVFQEDNLKHMFSHSEWLSSIYSRRSDAQAIKSLLYLERFWKSAREAVSVSESLVKILRIVDGDMPAMGYIYEGIERAKGAIKAYYKGIEEKYMPIWDIIDRRWNMQLHSPLHAAAAFLNPSIFYNPNFKIDLRMRNGFQEAMLKMATMDKDKIEITKEHPVYINAQGALGTDFAIMGRTLNAPGDWWASYGYEIPTLQRVAIRILSQPCSFHWCRWNWSTFETVHTKKRNKVEMEKLNDLVFVHCNLWLQTICQGRDGKCKPIIFDEIDVSSEWPTESEPSVPLLDDSWLDNLPLECRGSP	674	77.2	12.5	7.765	-0.39
GhA13	Ghir_A13G001820	2060042	2063794	MSSNLEPIPITSQKHDPAWKHCQMFKNGERVQLKCIYCGKIFKGGGIHRIKEHLAGHKGNAATCLRVPSDVRVLMQESLDGVVVKKRKKQKIAEEITNVNQVSTEIQAYGDQVDTNTGLLMIEKSDTLEPSSSLLVNREGTSNVAGERRKRGRGKNLPPEANALSFVPVELGARRVNNHVHMAIGRFLFDIGATMDAVNSVYFQPMVDAIVSGGSGALMPSCNDLQGWILRKSVEEVKSENDKVMGAWVRTGCSILVNQWNTQTGRILLNFLVYCPEGTVFLKSIDASSVINSSDALYELLKQVVEEVGSKHVLQVITNGEEQYIVAGRRLAETFPTLYWTPCAAHCVDLILEDFAKLEWINAIIEQARSITKFIYNHSVVLNMVRRYTFGNDIVEPAATRSATNFTTLTRMVDLKNNLQAMVTSQQWVDCPYSKKPGGLAMLDLVSNQSFWSSCILIVRLTNPLLRVLRMVGSKKRPAMGYVYAGMYRAKETIKKELVKRNEYMVYWNIIDHWWEQQWHHPLHAAGFYLNPRFFYSMEGDMPNEMLSGMLDCIEKLIPDVTVQDKISKEINSYKNSVGDFGRKMAVRARDTLLPVEWWSTYGGSCPNLARLAIRVLSQTCSTFGLKHNHIPFEKLYETRNCLEQQRLRDLIFVQCNLQLRQIGYESKQHDSMQPLSSESASIVEDWVTGIDAFLDDDTYPDWTTLETLSVNTMLLRPGDEVEELGAGFNDHEIFNRMKEGDNENAEDNVVS	752	85.046	4	6.881	-0.276
GhD13	Ghir_D13G002100	1833146	1837660	MSSNLEPIPITSQKHDPAWKHCQMFKNGERVQLKCIYCGKIFKGGGIHRIKEHLAGHKGNAATCLRVPSNVRVLMQESLDGVVVKKRKKQKIAEEITNVNQVSTEIQAYADQVDTNTGLLMIEKSDTLEPSSSLLVNQEGTSNVAGERRKRGRGKSLPAEANALSFVPVELGARRVNNHVHMAIGRFLFDIGATMDAVNSVYFQPMVDAIVSGGSGALMPSCNDLQGWILRKSVEEVKSENEKVMAAWVRTGCSILVNQWNTQTGRILLNFLVYCPEGTVFLKPIDASSVINSSDALYELLKQVVEEVGSKHVLQVITNGEEQYIVAGRRLVETFPTLYWAPCAAHCVDLILEDFAKLEWINAIIEQARSITKFIYNHSVVLNMVRRYTFGNDIVEPAATRSATNFTTLTRMVDLKNNLQAMVTSQQWVDCPYSKKPGGLAMLDLVSNQSFWSSCVLIVRLTNPLLRVLRMAGSKKRPAMGYVYAGMYRAKETIKKELVKRNEYMVYWNIIDHWWEQQWHHPLHAAGFYLNPRFFYSMEGDMPNEMLSGMLDCIEKLIPDVTVQDKITKEINSYKNSIGDFGRKMAVRARDTLLPVEWWSTYGGSCPNLARLAIRVLSQTCSTLGLKHDHIPFEKLHETRNCLEQQRLRDLIFVQCNLQLRQIGYESKQHDSMQPLSSESASIVEDWVTGIGAFLDDDTYPDWTTLETLSVNTMPLRPGDEVEELGAGFNDHEIFNRMKEGDNEKAEDNVVS	752	84.881	5.5	7.013	-0.263

GhD2	Ghir_D02G001340	1244716	1309397	MLEDVCFRYELPVALTWACEANTDKIMLDGKKYTLFMEKTSFYASNEGSQCFMEACAKHHIQEGQAIAGKALQSSANFHFKPSITKLIKSDYPLFNAAQLFGNHAVVAICLQNHYIIGDVYVVEFYWPEIESEKSESLALDIFNDLKNMKKKFVTIRVGSNEGTMHTRNAQPASSTNDLLSSNTTWSLNTVQPCDVHEMERHGLVESAPFSTPNPMSYGGVLQTQGPHKQEIGEKDFISQTVSIGDYEIVKAYMETCKVPRTKRRKYLSKVWLDFDKFEVNGKQVAKCKHCNKDFTGSSKSGTTHLKNHLERCQSKKIKNQKRQLITSEIGDLITRDSDESNFTFDQERSRLDFAKMIIKHQCPLDMAEQEFFKIFVKNLQPMFEFQSKDILLSDIHRIYKEEKEKLQLYFDQVACNFNLTISLWKNNLGKTAYCCLIAHFIDDNWGPKMKIIACKPLEHIYDTKALNEIIQSSVLEWNISKKVFSITMDNPYLSDDMFQKIKETCFRDQGSFPLTHWFIGCTFIKDGFREMDLILLKLRKSIEYVSEIAQGKLKFEEVVNQVKLQGGKSWDDLSLRLDSDFGVLHSALESREIFCQLEKIDSNFKLNPSVEEWEMVLAFHSCLKCFDDIEGTQSLTANLYFPKLCNIYKKFLHLGKSNYPIVTLMKRKFDYYWSLCNLAFSVATILDPRLKFKFVEFSYTEIYGHDSKMHLNRFHKVLTDVYYEYANEARNLSKSTSDLDDSNSSTTEIDNDCILESFSKFASASNFNEVASWKSELDCYLDEPLLPLDGAFDILYWWCINTKRFPTLAKMARDFLAMPISILAPCLNFNAMITNPTYNNLNPESMEALVCSQNWLEIPKENDGENHGPMQNMSEPALPLDGDILETNHGRNIAAAIEIPNDEPSFNGNQSDQFQSSSSESDDETSLREQGSWCREDVRTYLVSNFTNKEVKRLNRWKRSELSGKKIGRDNDFQLMGENLTPLLMVPHCDETLIEYYIDDSVVNTYFKLLKKRSDKFPNGYIKHYSFDSLIATCLIEGSKSEDEVLAWFKDEKLRGVHKLFLPMCLSAHWVLFCVDTKEKKISWLDPIPSSRIMSNSVEKQKIFQWFTLYLLPQFGYNDAEKWAFEVRTDIPKQENSIDCGVFVIKYGDCLMHGDFFPFTQKDMIHFRRRIFLDIYRGRLHGKR	1,185	137.157	-4	6.311	-0.405
GhD2	Ghir_D02G001310	1184479	1190848	MASSLFDIDTDTALRLLLSCAEAIEDGDLKSADAYLQNILILADERPYLYKSRVVKYFADALVRRAYGLHPASSYFTFPVDPSPYYHCGSYLINGVIENVIHDALMEKNALMGNRKLHLIDFSIPYSSFQNSVVRTLPTFSGDPLPVRVSYILPPFLKKYVKFLRQMEFLTRDAKEVNVKLEDELKLVYGNSLAEVDECEIDLKRRRDDEMVVVYYKFKLDKLVRDAKAMERELVRLKEINPTIVIMLDFYSNHTHSNFLTCFKDSFQYSLKTLDCWEELDLYFDEEYAWECHIEAWEGNNVIRRHPTLTEWQHLFSMAGFSRIPLNHREGIDLIVKDVNPLNDFFSMSNQSWLEIMGKEEECLILGYKECPMFFLSAWKPKVEEEHLNFNSSNGFNPYPSPLRPLRPFPEGLTLSRVAAVAEIYDILNHLYCEHKFSLALTWVSKVDNMNETMSDPNKKYTFSIQSNSCYSKDWNSYKFMRSCEYKIEQTIIEKALESKDGYHFEPSLTKSDIDDYMYLQRAKHCNVDVVVAICLQNRYTSNDIYVVEFYWPTTESEISKSFTPRIFNDLKHMEKKFVTVKVQGTEQAISNIPTSSYTARPLKIAEETEDVDAVELNGVNVQRGVVPNFPSPITIQSSSKVVAAPSNTLEGPHNQIFPNGDPEIVKANKQEPSKATQRELRSKVWDHFDRFEEDEKQVAKCKHCPKVLTGSSKSGTTHLNNHSKVCPGKKKQNQESQLILPVDTNEGSLRFDKKRSHMDLAKMMIKLQCPLDMAEQETFKNFVKGLQPMFEFQSKDILSYIHRIYDEEKEKLQLYFDKLASKFNLTVSLLKNNSGKTIYCCLISHFIDDGWELKRKILALKTLEHINDTKALGEIIRSLVLEWNISNKVCSITVDNSFLNDSMVDQIKEICLSDQGSVSSDHWFISFTLLEDGFREMDGILFKLRKSIEYVTETRHGKLKFQEAVDQVKLQGGKLWDDLSFRLKSDFDILDSALRSREIFCKLEQIDDNFKLNPTMEEWENAVALQSCLKCFDDVKGTQCLPVSLYLPKLCDTYKKFLQLEKSSHSFVTLMKRKFDRYWSLCNLALAVASVLDPRLKFKIVELSYRVIYGHDSKMRLNMFHKVLRDVYYEYASEAKNLTSSASVLDDFNCSTIVLGNDSILDSLSKFASASNFNEEASWKLELELYLDEPLLPMDGAFFDILGWWCDKSQRFPILAKMAQDFLAIPVSISTSCSNISAMINNPAYGSLNPESMEALVCSENWLETPKER	1,270	146.602	-14	5.732	-0.334
GhD2	Ghir_D02G001300	1184457	1189497	MEKKFVTVKVQGTEQAISNIPTSSYTARPLKIAEETEDVDAVELNGVNVQVIALSYIINDLFSISQIFPNGDPEIVKANKQEPSKATQRELRSKVWDHFDRFEEDEKQVAKCKHCPKVLTGSSKSGTTHLNNHSKVCPGKKKQNQESQLILPVDTNEGSLRFDKKRSHMDLAKMMIKLQCPLDMAEQETFKNFVKGLQPMFEFQSKDILSYIHRIYDEEKEKLQLYFDKLASKFNLTVSLLKNNSGKTIYCCLISHFIDDGWELKRKILALKTLEHINDTKALGEIIRSLVLEWNISNKVCSITVDNSFLNDSMVDQIKEICLSDQGSVSSDHWFISFTLLEDGFREMDGILFKLRKSIEYVTETRHGKLKFQEAVDQVKLQGGKLWDDLSFRLKSDFDILDSALRSREIFCKLEQIDDNFKLNPTMEEWENAVALQSCLKCFDDVKGTQCLPVSLYLPKLCDTYKKFLQLEKSSHSFVTLMKRKFDRYWSLCNLALAVASVLDPRLKFKIVELSYRVIYGHDSKMRLNMFHKVLRDVYYEYASEAKNLTSSASVLDDFNCSTIVLGNDSILDSLSKFASASNFNEEASWKLELELYLDEPLLPMDGAFFDILGWWCDKSQRFPILAKMAQDFLAIPVSISTSCSNISAMINNPAYGSLNPESMEALVCSENWLETPKER	827	95.05	-3.5	6.224	-0.393
GhA2	Ghir_A02G001270	1062867	1069866	MASSSSFSSADAALEILVSCAHAIEDGNLKTADSFLHQIWNTAAVELDLISKLVRYFAEALVRRAYGLHPPYYTHSNLQIPHPLYYYYYYSRFDINEMVGEAIESATTGKKGFHLIDFHIPHLYGRGYLFKTLPNRSSDPLSVRITVVLPTFLKNTVDFQEEMEYLTEAGKLLKIELKKEDLRVVYANSLGEVDESTLDLRRTNDDEALVVYYNFKFHTLLAEAEAMKKELIKLRQINPEIVIMQEQYANDNDGNFIKRLEYSFRYYSNFFQYYSNLFKSGKPLGDNTAKYYMRQIHNIVACEGRDRIMRHQSLDEWRDLLLTAGFLQIPFQKDVENLHALYWVEEIKEEKGCLVLSHKDCPILFVSCWRPRAGEEHFKFNLNSNKFGQGFNPRPFQPFPEGFILNRLATFAEIYDMLEDVCFRYELPVALTWACEATTDKIMLDGKKHTLFMERTSCYASNEGSQCFMEACAKHHIQEGQAIAGKAFQSSANFHFEPSITKLMKSDYPLFNAAQLFGSHAVVAICLQNHYIIGDVYVVEFYWPEIESEKSESLALDIFNDLKNMKKKFVTIRVGGNEVGFEREAISTTLQGTMHMRNAQPASSTNDLLSSNTTWSLNAVQPCDVHEMERHGLVEQVESAPFSTPNPMSHGGVLQTQGPHKQEIGEKDFISQTVSIGDYEIVKASMETCKVPRTKRRKYSSKVWLDFDKFEVNGKQVAKCKHCNKDFTGSSKSGTTHLKNHLERCQSKKIKNQERQLITSEIGDLITRDSDESNFTFDQERSRLDFAKMIIKHQSPLDMAEQEFFKIFVKNLQPMFEFQSKDILLSDIHRIYKEETEKLQLYFDHLACNFNLTISLCKNNHGKTAYCCLIAHFIDDNWEPRMKIIACKPLEHIYDTKALNEIIQSSVLEWNISKKVFSITMDNPYLNDDMFQKIKETCFSDQGSFPSTHWFIGCTFIEDGFREMDLILLKLRKSIEYVSEIAEGKLKFEEVVNQVKLQGGKSWDDLSLRLDSDFGVLHSALESREIFCQLEKIDGNFKLNPSVEEWEMVLAFHSCLKCFDDIEGTQSLTANLYFPKLCNICKKFLHLEKSNYPIVTLMKRKFDYYWSLCNSAFAVATILDPRLKFKFVEFSYTEIYGHDSKMHLNRFHKVLTDVYYEYANEARNLSKSTSDLDDSNYSTTEIVNDCILESFSKFASANNFNEVASWKSELDCYLDEPLLPLDGAFDLLYWWCINNKRFPTLAKMARDFLAMPIPILAPCLNFNAMITNPTYNNLNTESMEALVCSQNWLKIPKESKSNF	1,299	150.144	-10.5	6.085	-0.344
GhA11	Ghir_A11G000730	687773	691437	MTFGLHLLQIVSFVGNKMEWSVNNAFKSYKDMEPKSTMDMVLIPNMDTIDIVLGSSEKGNVVPSAKPRKKTMTSVYLKYFETAPDGKTRRCKFCGQSYSIATATGNLGRHLSNRHPGYDKTGENVSSSAPQPSTTPTVIKKPQPQGRAPQVDYDHLNWLLIKWLILATLPPSTLEEKWLANSFKFLNPSIQLWPGEKYKAVFCEVFRSMREDVRASLEQVSSKVSIALDFWSSYEQIFYMSITCQWIDENWSFQKVLLDICQVPYPCTGSEIYNSLVKVLKMYNIENKVLSCTHDNSQNAIHACHALKEDLDGQKMGPFCFIPCAARTLSLIIDDALRTTKPVIAKVREFVQELNASLDISEDFIQLATAYKEGSWQFPLDASARWSGSYQMLDIVQKAGKSMDAVVRKNEEMLGNRMLLNTAEKNVVNIVHNYLEPFYKVISEIGVNTPPTIGMVIVYMDHISDTITTQQPPDWLKNPAEDMAKKLRSYNNQVCNIFIYMT	698	79.025	3.5	7.036	-0.334

				AILDPRIKCELIPESLNSENYLEEARAHFVRNYYTTPFSSMTSGYSSQDIEDGGAVSFAEEIARKKRRASMSNATDELTQYLSESPAPTKTDVLEWWKVNSTRYPRLSAMARDFLAVQATSVKPDELFCSKGDEIDKQRFCMPHDSTQAILCIKSWTQGGLKLKYKSTEIDYERLMEMAAAAAADISLAGMDKKQK					
GhD2	Ghir_D02G000710	5628847	5676779	MDNFDQKLGPEFFKNLSAEAVTPLNVVHEEIYESSSKRPKTTSKVWDIFEKLPAQQGDSKAICKLCRRIYTAKTTSGTSHLRRHIEACVKRGNHEVDQRSIEACFKPVKRNANRLTLSHDTLIAATTSLQNYKLDVDEIHRAIAMMIIVDEQPFSVVEDAGFRRLLSAACPEFPVLSRSSIKRDIISIYVKERENIRELLATCPGRICLTSSTWKSDSDDHFSCVTTHFIDHEWRLQRRILRFKLMPPPYDSLSVADEIALCMVQWNIEHKVFSVTLENLSSDDCVADMLRSRLAAKKYLPCKGVFFHVSCFFRILNSIVQAGLNLVVDIIAKLRLGIKYVQQSPHRKKNFYIIAKTLNLDTQRKLCLDTPARWNSTYNMVEVAFCYKNAFVYLAEQDKNFLHKLSEDEWEKMSVLYKFLKVFYEVTCVFFRNRQPTSNLYFKAAWKVHSRLFDMVRGPENFMTHMVREMHSKLNQYWSAYNLILSCAAIDPRYKIKFVEYCYTKLYGSGAQKYVSVSVNTLYGLFDEYMQNSARPSQTTLLSTAASKISNDKDNNDGFEDYETFQSARFRTQVEKSQLDLYLEEPSHDLNSEIDVLEYWTLCSLRYPELSKMARDVLTIPVSTIASDSDFDISPQVISTDRSSLKPKMLQSLVCLQDWMLASDRTRGLGSMESKPEDDSSSSSDGDDDY	691	79.63	10.5	7.721	-0.335
GhA2	Ghir_A02G000740	5390667	5441136	MDNFDQKLGPEFFKNLSAEAVTPLNVVHEEIYESSSKRPKTTSKVWDIFEKLPAQQGDSKAICKLCRRIYTAKTTSGTSHLRRHIEACVKRGNHEVDQRSIEACFKPVKRNANRLTLSHDTLISATTSLKNYKLDVDEIHRAIAMMIIVDEQPFSVVEDAGFRRLLSAACPEFPVLSRSSIKRDIISIYVKERENIRELLATCPGRICLTSSTWKSDSDDHFNCVTTHFIDHEWRLQKRILRFKLMPPPYDSLSVADEIALCMVQWNIEHKVFSVTLENLSSDDCVADMLRSRLAAKKYLPCKGVFFHVSCFFRILNSIVQAGLNLVVDIIAKLSLGIKYVQQSPHRKKNFYIVAKTLNLDTQRKLCLDTPARWNSTYDMIEVAFCYKNAFVYLAEQDKNFLHKLSEDEWEKMSVLYKFLKVFYEVTCVFFRNRQPTSNLYFKAAWKVHSRLFDMVRGPENFMTRMVREMHSKLNHYWSAYNLILSCAAILDPRYKIKFVEYCYTKLYGSGAQKYVSVSVNTLYGLFDEYMQNSARPSQTTLLSTAASKISNDKDENDGFEDYETFQSARFRTQVEKSQLDLYLEEPSHDLNSEIDVLEYWTLCSLRYPELSKMARDVLTIPVSTIASDSAFDITPQVISADRSSLKPKMLQALVSLQDWMLASDRTRGLGSMESKPEDDSSSSSDGDDDY	691	79.572	9.5	7.607	-0.336
GhD9	Ghir_D09G000090	2169555	2194770	MSKVWDEMTKLECENKNELKAQCNHCKTIFSAKSSSRTSHLRRHLNSCLKKVNKDITQYTIATQPSPEGVPSIKNYKFDPDECRRAISTFIVCGKYSFRTVEEPGFRYMMRIASPNFKNISRHTAARDVLMYYAKERDRVKEELAKAPGLICLTSDNWNSEHTNDEYICITAHWVDKDWKLQKRIIRFRALFPPYDGLNIADELVLCLSQWGIDKKIFSITLDNASYNDVMVSCLKNRFRANRAILCDGAFFQVRCCAHILNLIVKAGLELADDVVGKIRNGIRYIKKSGIRRKRFYDVADKSFHLNVTKKLRQDVCVRWNSTYLMIKSSLYYKDVLDYWGQRDKDYQMFALSNEEWRNVAILCKFLKVFYDVTCIFSGSNYPTTNLYFRGVWKVHKVLLDTVKGPYSFLTPMVKQMQEKFNKYWAEYSLILSCAAILDPRYKLNYVQYCFNTIYGIHASDFVETILCNLRLLFDEYVKKSKSTSSSLAESSNVSNKNPVDSGLDEHNDNSADFVGYFDESNDYKRYLNESSTRSEKSQLDIYLEEPELELNSQIDVLDYWSKSSVRYNELSLLARDLLAIPISTVASKSAFSMGKKVITPLRSSLKPKTVQAVVCLDDWMRAKGFFNRNWLQKGR	636	73.898	27.5	9.169	-0.399

Table S20 List of GheZF-BED genes and their features

Chromosome	Gene_ID	Start	End	Protein Length (aa)	Molecular Weight (kDa)	Isoelectric Point	Grand Average of Hydropathy	Protein_sequence
Ghe8	Ghe08G302500	130345203	130347313	672	76.93533	6.98	-0.288	MEVANETVIKKPKRLTSVVWNHFERVRKADLCYAVCVHCNKKLSGSSNSGTTHLRNHLMRCLKRFNYDVSQLLSAKKRKKDNTLTIANISYDEGQRKEEYLKPTIVKYEPEQRKDEVFNVQSSWFDQERSRLDLARMIILHGYPLAMVEHVGFKVFVKNLQPLFDVVPNSTVELSCMEIYGKERQKVHDMLSKLQGRINLAVEMWSSPENTNHVCMMAHYVGDDWKLQKKILNFVTLDSSHTDDLLSGVIIKCLMDWDIGSKLFAVTLDDFSANDDIVLRIKEQISENKSRLSNGQLLDVRSAAHVLNSIVQDAMEALRLVIQKIRGTVRYVKSSQSIQGKFKEMVLQTGINSQKNLVLDCPIQWNSTYLMLETAIEYRNAFCQLPELDLDLALSDEEWEWASSITGYLKLFIEIINVFSSNKCPTANIYFPEICHVHIQLIDWCKSPDNFLSSLAAKMKAKFDKYWSKCSLSLAVAAILDPRFKMKLVEYYYSQIYGSTALERIKEVSDGLKELFNTYSICSTLMDQGSALPLSSLPSSSNDGRDRLKGFDKFLHETSQSQTAISDLEKYLDEPVFPRNCNFNILNWWRVHTPRYPILSMMARDVLGTPMSTVSQESAFHAGGRVLDSCRCPLTPETQQALICTQDWLRMQSDDPGPSSSHYALPLYVETN
Ghe11	Ghe11G389800	124508348	124510457	672	76.93997	6.41	-0.319	MEVANESTAKKPKRLTSVVWNHFERVKKADICYAVCVHCNKKLSGSSNSGTTHLRNHLMRCLKRSNYDVSQLLAVKRRKKENTLTIANISYDEGQRKEDYMKPTIVKYEQDQRKDEAFNLGSSWFDPERSRLDLARMIILHGYPLAMVEQVGFKVFVKNMQPLFDVVHNSTIELSCVEIYMKEKQRIYDMLSKLQGRINLAIEMWSSPENSKYVCLTAHYVDDEWKLQKKILNFLTLDSSHTEDVLSDVIIKCLMDWDIDCKLFAMTFDDCSTNDDIVSRIKDQVSESRPRLSNGQLLDVRSAAHVLNSIAQDAIEALQVVIQKIRGSVKYVKSSQSILGKFNEIAQQQGIDNHKIVVLDYPIRWNSTYMMLETAVEYRNVFHHLPELDPDFALSDEEWERASSIVSCLKLLIEIINVFSSNKCPTANIYFPEICHVHIQLIEWCKSSDAFLSSLATKMKAKFDKYWSKCSLALAVAAILDPRFKMKLVEYYYSQIYGSTALERIKEASDGIKELFNAYSICSTLIDQGSALPGSSLPSSSNDTRDRLKGFDKFLHETSQSQTAISDLEKYLDEPVFPRNCDFNILNWWRVHTPRYPILSMMARDVLGTPMSTIAQEFAFNAGGRMLDSNQSSLPPDTQQALICTRDWLRTQSDDATPSSSHYALPLYVEAN
Ghe6	Ghe06G210800	116734022	116736154	596	68.40111	7.13	-0.331	MTMASSNSPIPVDDEFNEYESAPKRQKSTTSMVWDEMTKLECENKNELKAQCNHCKTIFSAKSSSGTSHLRRHLNSCLKKVNKDITQYTIATQPSLGGVPFIKNYKFDADKCRQAISTFLVCSKHAFRTVEEPGFRYMMRISSPNFKNISRYTAARDLAKAPGLICLTSDNWNSEHTNDEYICITTHWVDKDWKLQKRIIRFRALFPPYDGLNIADELVLCLSQWGIDKKIFSITLDNASYNDVMVSCLKNHFRANRAILCDGAFFQVRCCAHILNLIVKAGLELADDVVGKIRNGIKYIKKLRIRRKRFYDVADKSFHLNVTKKLRQDVCVGWNSTYLMLESSLYYKDVLDYWGQRDKDYQIFVLSNEEWRNVAILCKFLKVFYDVTCVFSGSNYPTANLYFRGVWKVHKLLLDTVKDFVETILSNLRLLFDEYVKRSKSTSSSLARSSNVSDKNLVDSSLDKHNVNSVDFGFGGDFDESDDYKRYLNESSTMSEKSQLDIYLEEPALELNSEIDLSLLVHDLLAILISTVASESAFSMGKKVITPLRSSLKPKMVQAIICLDDWMRAKGLSTEICYKNDDDDEDDEDDVSLITF
Ghe1	Ghe01G230900	113462087	113467331	721	82.79617	8.29	-0.329	MHFCSIFDNFYVFVIVTFIIAAGGWSYGLLQPASVSREAKDKVLVGWSKESNRMSTEPTSIEGSVTPPTSIDSENSGVGASSQANVTTGKRKATPQRSEVWSHFTKIINSEGASKAKCNYCQKEFCCDVKKNGTGSLKYHIGSCKKNPSNVVDPRHLSTWRFDQEACRKGLAQMIVIDELPFKFVESEGFKKFMFVARPRHIPSRTTMTKDVYQLYLDERVKIKQLLRSSCSRVCLTTDTWTSLQRVNYLCITAHFIDNDWKLNKKILNFCPISSHKGESIGMVIEKCLLTWGIDKLFTVTVDNASSNDVVIGCLRKKLNPRGGLVQNGKYLHMRCMAHIVNLIVVEGLKEMNKSVERVRGAVRYVRQSPARLQKFKECVVVEKIECKKMLCLDVCTRWNSTYLMLDTAQNIERAFERFEEQDTNFRAELERGEGWPSVDDWTNVRDLRDFLEHFYEVTLRISGTSYVTSNNFFDELSEIDILLRDAQLNSNVDFNVMAIKMKEKYDKINLLMFVACILDPRQKLKYLEFALSEMSSSEKASEMMQKLKESLYELFDEYKPSLYSTCSQSSVSTHVSLDEPQQKMKRRMQALYKKRELEICGEDKTSELDKYLAEANEEFVEDFDILLWWKVNSPRFPTLSKIARDVLAILVSTVVSESAFSTGGRVLDQYRSSLTPKIVQSLVCTQDWIRKSSSQEDIKKIEEQIQELDKIENGIFIILF
Ghe4	Ghe04G204100	104518054	104521395	695	80.10171	8.77	-0.354	MASSNTPIPVDDGFNEYESTVKRQKSTTSKVWDEMTKLECENKNELKAQCNHCKTVFSAKSSSGTSHLRRHLNSCLKKANKDITQYTIANQPSLGGVPFIKNYKFDADECRQAISIFLVCGKHSFRTVDEPGFRHMMRIASPNFKNISRHTAARDVLMYYAKERDRVKEELAKAPGLICLTSDNWNSEHTNDEYICITAHWVDKDWKLQKRIIRFRALFPPYDGLNIADELVLCLSQWGIDKKIFSITLDNASYNDVMVSCLKNRFRANRAILCDGAFFQVRCCAHILNLIVKAGLELADDVVAKIRNGIKYIKKSGIRRKRFYDVADKSFHLNVTKKLRQDVCVRWNSTYLMLESSLYYKDVLDYWAQRDKDYQMFALSSEEWRNVAILCKFLKVFYDVTCVFSGSNYPTANLYFRGVWKVHKVLLDTVKGPYSFVTPMVKQMQEKFNKYWAEYSLILSCAAILDPRYKLNYVQYCFTTIYGIHASDFVETILSNLRLLFDEYVKKSKSTSSSLAGSSNVSDKNPVDSSLDEHNVNSADFGRDFDESDDYKRYLNESSTRSEKSQLDIYLEEPELELNSQIDVLDYWSKSSVRYNELSLLARDLLAIPISTVASESAFSMGKKVITPLRSSLRNVRILGLTGQDRTEMSHVRIIHVSPCRKHGIDRILIDRNDLSILSIPRVLQYIHECKECIC
Ghe13	Ghe13G201000	102139410	102142029	705	81.04616	8.46	-0.427	MSTEPTSIKGSVTPPTSIDSENSGVGASSQANVTTGKRKATPQRSEVWSHFTKIINSEGASKAKCNYCQKEFCCDVKKNGTGSLKYHIGSCKKNPSNVVDPSQGQLVLPRKGVEGGEGHLSTWRFDQEACRKGLAQMIVIDELPFKFVESEGFKNFMFVACPRFHIPSRTTMTRDVYQLYLDERVKIKQLLKSSCSRVCLTTDTWTSLQRVNYLCITAHFIDNDWKLNKKILNFCPISSHKGESIGMVIEKCLLNWGIDKLFTVTVTVDNASSNDVAIGYLRKKFNPRGGLVQNGKYLHMRCMAHIVNLIVVEGLKEMNKSVERVRGAVRYVRQSPARLQKFKECVVMEKIECKKMLCLDVCTRWNSTYLMLDTAQKFERAFERFEEQDTNFRAELERGEWPSVDDWANVRNLRDFLEHFYEVTLRISGTSYVTSNNFFDELSEIDILLRDAQLNSNVDFNVMAIKMKEKYDKYWGDIDKMNLLMFVACVLDPRQKLKYLEFALSEMSSSEKACEMMQKLKESLYELFDEYKPPLYSTCSQSSVPTHVSLGEPQQKMKRRMQALYKKRELEIGGEDKTSELDKYLAKANEDFIEDFDILLWWKMNSPRFPILSKMARDVLAIPVSTVASESTFSTGGRVLDQYRSSLTPKIVQALVCTQDWIRKSSSQEDIKKIEEQIQELDMIENDVNAVKKFIREWEKGIWVS
Ghe12	Ghe12G	1011	1011	651	74.77409	8.66	-0.382	MSIEPTSIEGSVTPPTSIDSKNSGIGAPSQANVTTGKRKATPQRLEVWSHFTKIINSEGASKAKCNYCQKEFCCDVKRNSTGSLKYHIGACKKNPSNVVDTSQGQLVLPRKGVERGEGHLSTWRFDQEACRKGLAHFNEGFKKFMFVACPRFHIPSRTTMTRDVYQLYLDERVKIKQLLKSSCSRVCLTTDTWTSLQRVNYLCITAHFIDNDWKLNKKILNFCPISSHKGESIGMVIEKCLLNWGIDKLFTITVDNASSNDVAIGYLRKKFNPRGGLIQNGKYLHMRCMAHIGAIRYVRQSPARLQKFKECVVVEKIECKKMLC

	24770	490887	516339					LDVCTRWNSTYLMLDTEQDTNFRAELERGEGWPSVDDWANVRDLRDFLEHFYEVTLRISGTSYVTSNNFFDELSEIDILLRDAQLNSNVDFNVMAIKMKKKYDKYWGDIDKMYLLMFVACVLDPRQKLKYLEFALSEISEKASEMMQKLKESLYELFDEYKPSLHSTCSQSSVPTHVSLGEPQQKMKRRMQALNKKRKLEICGEDKTSELDKYLAAANEEFVEDFDILLWWKVNSPRFPTLSKMARDVLAIPVFTVASESAFSTGGRVLDQYRSSLTPKIVQALVCTQDWIRKSSSQEDIKEIEEQIQELDKIENGIFITYVIFLIM
Ghe12	Ghe13G02340	101149087	101151639	752	84.93123	6.35	-0.265	MSSNLEPIPITSQKHDPAWKHCQMFKNGERVQLKCIYCGKIFKGGGIHRIKEHLAGHKGNAATCLRVPSDVRVLMQESLDGVVVKKRKKQKIAEEITNVNQVSTEIQAYGDQVDTNTGLLMIEKSDTLEPSSSLLVNREGTSNVAGERRKRGRGKSLPAEANALSFVPVELGARRVNNHVHMAIGRFLFDIGATMDAVNSVYFQPMVDAIVSGGSGALMPSCNDLQGWILRKSVEEVKSENDKVMGAWVRTGCSILVNQWNTQTGQILLNFLVYCPEGTVFLKSIDASSVINSSDALYELLKQVVEEVGSKHVLQVITNGEEQYIVAGRRLAETFPTLYWTPCAAHCVDLILEDFAKLEWINAIIEQARSITKFIYNHSVVLNMVRRYTFGNDIVEPAATRSATNFTTLTRMVDLKNNLQAMVTSQQWVDCPYSKKPGGLAMLDLVSNQSFWSSCILIVRLTNPLLRVLRMVGSKKRPAMGYVYAGMYRAKETIKKELVKRNEYMVYWNIIDHWWEQQWHHPLHAAGFYLNPRFFYSMEGDMPNEMLSGMLDCIEKLIPDVTVQDKISKEINSYKNSVGDFGRKMAVRARDTLLPVEWWSTYGGSCPNLARLAIRVLSQTCSTLGLKHNHIPFEKLYETRNCLEQQRLRDLIFVQCNLQLRQIGYESKQHDSMQPLSSESASIVEDWVTGIDAFLDDDTYPDWTTLETLSVNTMLLRPGDEVEELGAGFNDHEIFNRMKEGDNENAEDNVVS
Ghe7	Ghe07G30270	100223819	100227287	667	76.6581	7.43	-0.42	MGLDKKRGPSNSSIHVDDGFNEYESAAKRQKSTTSKVWDEMTKLECENKNELKAQCNHCKTIFSAKSSSGTSNLRRHLNSCLKKVNKDISQYTIATQPSLGGVPFIKNYKFDADECRKAVSTFLVCGKHSFRTVEEPGFRYMMSIASPNFKNISRYTAARDVLMYYAKERDRVKEELARAPGLICLTSDNWNLEHTNDEYICITAHWVDENWKLQKRIIRFRALFPPYDGLNIADELVLCLSQWGIDKKIFSITLDNASYNDVMVSCLKNRFRANRAILCDGAFFQVRCCAHILNLIVKAGLELADDVVVKIRNGIKYIKKLGTRRKRFYDMADKSFHLNVTKKLRQDVCVRWNSTYLMLESSLYYKDVLDYWGQRDKDYQLFALSNEEWRNVAILCKFLKVFYDVTCVFSGSNYPTANLYFRGVWKVHKVLLDTVKGPYSFLTPMVKQMQEKFNKYWAEYSLILSCAAILDPRYKLNYVQYCFTTIYGIHASDFVETILSNLRLLFDEYVKKSKSTSSSLAGSSNVSDKNPVDSSLGEHNVNNVDFGGDFDESDDYKRYLNESSTRSEKSQLDIYLEEPELELNSQIDVLDYWSKSSVRYNELSLLARDLLAIPISTVASESAFSMGKKVITPLRSSLKPKTVQAVNDEDDEDDEDDEDDVSSIAF
Ghe8	Ghe08G16000	97239473	97241611	602	68.98536	7.77	-0.392	MASSNSPIPVDDGFNVYESAPKHQKSTTSKVWDEMTKLECENKNELKAQCNHCKTIFSAKSSSGTFHLRRHLNICLKKVNKDITQYTIANQPSLGGCRQAVSTFLVCGKHSFRTVEESGIRYIMRIASPNFKNISRYTAARDVLMYYAKEGDRVKEELAKAPGLICLTSDNWNSKHTNDEYICITAHWVDKDWKLQKRIIRFRALFPPYDGLNIADELVLYLSQWGIDKKIFSITLDNASYNDVMVSCLKNRFRANRAILCNGAFFQVRCSAHIWNLIVKAGLELADGVVGKIRNGIKYIKKSGIRRKRFYDVADKSFHLNVAKKLRQDVCVRWNSTYLMVESSLYYKDVLDYWGQRDKDYQIFALSNEEWRNVAILCKFFKVFYDVTCVFSGSNYPTANLYFRGVWKVHKLLLDTVKDFVETILSNLRLLFDKYVKRSKSTSSSLAGSSNVSDKNPVDSSLDEHNVNSVDFGFGGDFDESDDYKWYLNESSTMSEKSQLDIYLEEPALELNSQIDVLDYWSKSSVRYNELSLLASSESAFSMGKKVITPLRSSLKPKTIQAVVCLDDWMRVKGFLTEIGCKNDDDDEDDEDDEDDVSLIAF
Ghe7	Ghe07G26640	95448207	95448479	90	9.59669	5.42	-0.482	MADPDANTLTPSKPLTSASCTPISLDDDESPVDSGLEDPSILPLTSRHTSSVWTNFTRNRVGDAIKAECNHCSKLLASGKRVGTTHLKDC
Ghe7	Ghe07G24060	88300084	88303669	688	78.94703	8.53	-0.405	MGLDKKRGPFKNGPGLGLNSPIHVDDGFNEYESAAKRQKSTTSKVWDEMTKLECKNKNELKAQCNHCKTIFSSKSSSGTSHLRRHLNSCLKKVNKDISQYTIATQPSLGGVPFIKNYKFDADECRKAVSTFLVCGKHSFRTVEEPGFRYMMSIASPNFKNISRYTAARDVLMYYAKERDRVKEELARAPGLICLTSDNWNSEHTNDEYICITAHWVDENWKLQKRIIRFRALFPPYDGLNIADELVLCLSQWGIDKKIFSITLDNASYNDVMVSCLKNRFRANRAILCDGAFFQVRCCAHILNLIVKAGLELADDVVAKIRNGIKYIKKSGTRRKRFYDVADKSFHLNVTKKLRQDVCVRWNSTYLMLESSLYYKDVLDYWGQRDKDYQLFALSNEEWRNVDILCKFLKVFYDVTCVFSGSNYPMANLYFRGVWKVHKVLLDTVKGPYSFLTPMVKQMQEKFNKYWAEYSLILSCAAILDPRYKLNYVQYCFTTIYGIHASNFVETILSNLRLLFDEYVKKSKSTSSSLAGSSNVSDKNPVDSSLGEHNVNNVDFGGDFDESDDYKRYLNESSTRSEKSQLDIYLEDPELELNSQIDVLDYWSKSSVRYNELSLLARDLLAIPISTVASESAFSMGKKVITPLRSSLKPKMVQAVVCLDDWMRAKGFSTEIGCKNDEDDEDDVSSIDF
Ghe9	Ghe09G25140	79488057	79491528	686	78.79341	6.66	-0.426	MGLDKKRGPSNSPIHVDDGFNEYESAAKRQKSTTSKVWDEMTKLECENKNELKAQCNHCKTIFSAKSSSGTSHLRRHLNSCLKKVNKDISQYTIATQPSLGGVPFIKNYKFDADECRKAVSTFLVCGKHSFRTVEEPGFRYMMSIASPNFKNISRYTAARDVLMYYAKERDRVKDELVRAPGLICLTSDNWNSEHTNDEYICITAHWVDENWKLQKRIIRFRALFPPYDGLNIADELVLCLSQWGIDKKIFSITLDNASYNDVMVSCLKNRFRANRAILCDGAFFQVRCCAHILNLIVKAGLELADDVVAKIRNGIKYIEKSGTRRKRFYDVADKSFHLNVTKKLRQDVCVRWNSTYLMLESSLYYKDVLDYWGQRDKDYQLFALSNEEWRNVAILCKFLKVFYDVTCVFSGSNYPTANLYFRGVWKVHKVLLDTVKGPYSFLTPMVKQMQEKFNKYWAEYSLILSCAAILDPRYKLNYVQYCFTTIYGIHASDFVETILSNLRLLFDEYVKKSKSTSSSLAGSSNVSDKNPVDSSLGEHNVNNVDFGGDFDESDDYKRYLNESSTRSEKSQLDIYLEEPELELNSQIDVLDYWSKSSVRYNELSLLARDLLAIPISTVASESAFSMDKKVITPLRSSLKPKTVQAVVCLDDWMRAKGFSTEIGCKNDEDDEDDEDDEDDVSSIAF
Ghe9	Ghe09G23340	77827619	77831095	689	78.38064	8.7	-0.356	MHQDKLKKRRRAGNLIGPNLKKNPRYLAQKEKRNQHVISNSNLLYLKENFDHLMFDEDVCWEYAEKLDGNKVRCKFCLRVLNGGISRLKHHLSRLPSKGVNPCNKVRDDVTDRVRAIISSKEDIKEIPSVKKQKIAEVRAPGNMSTGSKISPLETLSPAAKVFPTVLSIAASTLSDQETVERSIAFVARSSSYQAMIDAVGKFGPGLIAPSVETLKTTWLKRIKSEVTLHLKDAEKEWATTGCTIIADTWTDNKSKALINFLVSSPSRTFFHKSVDASSYFKNTKCLADLFDSVIQDFGQENVVQIIMDSSFNYTGISSHILQNYGTIFLSPCASQCLNLILEEFSRVDWVNRCILQAQTVSKFLYNNASMLDLMKKFTGGQELIRTGITKSVSCFLSLQSTLKQRSRLKHMFNSPEYSTNSSYANKPQSISCIAIVEDNDFWRAVEECVAISEPFLKVLREVSGGKPAVGSIYELMTRAKESIRTYYIMDESKCKTFLDIVDRQWRDQLHSPLHSAGAFLNPSIQYNPEVKFLGSIKEDFFKVLEKLLPTPELRRLWWEQFGDSAPVLQRVAIRILSQVCSTFTFERHWSTFQQIHTEKRNKIDKETLTDVVYINYNLKLAREMKTMPTDSDPIQFDDIDMTSEWVEESENPSPTQWLDRFGSALDGGDLNTRQFSAAMFGNDHIFGL
Ghe13	Ghe13	732	732	663	76.14566	7.43	-0.402	MGLDKKRGPSNSPIHVDDGFNEYESAAKRQKSTTSKVWDEMTKLECENKNELKAQCNHCKTIFSAKSSSGTSHLRRHLNSCLKKVNKDISQYTIATQPSLGGVPFIKNYKFDADECRKAVSTFLECGKHSFRTVEEPGFRYMMSIASPNFKNISRYTAARDVLMYYAKERDRVKEELARALGLICLTSDNWNSEHTNDEYICITAHWVDENWKLQKRIIRFRALFPPYDGLNIADELVLCLSQWGIDKKIFSITLDNAS

	G14150	14982	18460					YNDAMVSCLKNRFRANRAILCDGAFFQVRCCAHILNLIVKAGLELADDVVAKIRNGIKYIKKSGTRRKRFYDVADKSFHLNVTKKLRQDVCVRWNSTYLMLESSLYYKDVLDYWGQRDKDYQLFALSNEEWRNVAILCKFLKVFYDVTCVFSGSNYPMANLYFRGVWKVHKVLLDTVKGPYSFLTPMVKQMQEKFNKYWAEYSLILSCAAILDPRYKLNYVQYYFTTIYGIHASDFVETILSNLRLLFDEYVKKSKSTSSSLAGSSNVSDKNPVDSSLGEHNVNNSQLDIYLEEPELELNSQIDVLDYWSKSSVRYNELSLLARDLLAIPISTVASESAFSMGKKVITPLRSSLKPKTVQAVVCLDDWMRAKGFSTEIGCKNDEDDEDDEDDEDDVSSIAF
Ghe12	Ghe12G12530	71490159	71497284	800	92.41117	8.9	-0.659	MPPCEEFPTKGLEGAPSNDIGWHFGTPVPNTKGNIVCKLCDKVMKGGITRFKKHIAHKTGNVAPCPNVTGVIRESMMNILKESKTKKIDKKRRKDEFLSQLREDEDKHEKFIDEVFAIRERISKSILSESEFTLRGVIPELAKSKSSKQPKVSDSILKTFRKKIGEVVSKLLIYERLPFQLASSPWLYNLIQVSTEVGQGVKLPTPYEISDVYLESEYQRVRDWVNGLKTHWKELGATLMCDGWTNSLNQIHIINFLVYCNASSVRSRDVEFYYRLLDSVEAMKATGKKLMLKRKHLYWTLLNMTPIFTHCLDLCLEDIGKKPSVAKVLDEAKKVTCFIYNHIWTVDLMKKYTQGKQILRPALTRFTTHFIQLEEITRQKQGLREMFDSKEFKESKWGQQKLGPAYEAKKIVLRKDFWKKANDLIKVYEPLVKVLRLVDSDEKPTMSFIYEAVDRAKRAIQQDCRYFIEYEKIIDNRWNFIHSDLHLADYFLNPQFQFGVEHSENVLIEILEGTRSVIERLEPSLNTQVRMVNQLLLFRDKHETFGTPQAQRAWKQMNPGKHTFSYIHTKARNRLKYKKLEKLVFTYYNMRLQIRHQKRMSTDDINASFNPISLDHIFEDVDPLSEWLHEKENPLLDGENTGVLPVDTSDNEMDVDQSQQQNLSHSSSSSTPSQSGDGPDGSGLSPIDEDDGYSGDRGEIRSSSRYGREYGVGTTSGHFYDRSKFDGNMFPEPRRDRSKKHTSIGFSSGRRSSSSNLGYNDSSTSTQGFYPPEQPLYFQPSHGYPQPYGYYPPFPNYGVP
Ghe1	Ghe01G16530	57552276	57555756	671	76.97826	6.36	-0.45	MGLDKKRGPSNSPIHVDDGFNEYESAAKRQKSTTSKVWDEMTKLECENKNELKAQCNHCKTIFSAKSSSGTSHLRRHLNSCLKKVNKDISQCTIATQPSLGGVPFIKNYKFDADECRKVVSTFLVCGKHSFRTVEEPGFRYMMSIASPNFKNISRYTAARDVLMYYAKERDRVKEELARAPGLICLTSDNWNSEHTNDEYICITAHWVDENWKLQKRIIRFRALFPPYDGLNIADELDNASYNDVMVSCLKNCFRANRAILCDGAFFQVRCCAHILNLIVKAGLELADDVVAKIRNGIKYIKKSGTRRKRFYDVADKSFHLNVTKKLRQDVCVRWNSTYLMLESSLYYKDVLDYWGQRDKDYQLFALSNEEWRNVAILCKFLKVFYDVTCVFSGSNYPTANLYFRGVWKVHKVLLDTVKGPYSFLTPMVKQMQEKFNKYWAEYSLILSCAAILDPRYKLNYVQYCFTTIYGIHASDFVETILSNLRLLFDEYVKKSKSTSSSLAGSSNVSDKNPVDSSLGEHNVNNVDFGGDFDESDDYKRYLNESSTRCEKSQLDIYLEEPELELNSQIDVLDYWSKSSVRYNELSLLARDLLAIPISTVASESAFSMGKKVITPLRSSLKPKTVQAVVCLDDWMRAKGFSTEIGCKNDEDDEDDEEDEDDEDDVSSIAF
Ghe1	Ghe01G16520	57522251	57524396	650	74.59936	5.66	-0.453	MTMASSNSPIHVDDGFNEYESAAKRQKSTTSKVWDEMTKLECENKNELKAQCNHCKTIFSAKSSSGTSHLRRHLNSCLKKVNKDISQCTIATQPSLGGVPFIKNYKFDADECLEEPGFRYMMSIASPNFKNISRYTAARDVLMYYAKERDRVKEELARAPGLICLTSDNWNSEHTNDEYICITAHWVDENWKLQKRIIRFRALFPPYDGLNIADELDNASYNDVMVSCLKNCFRANRAILCDGAFFQVRCCAHILNLIVKAGLELADDVVAKIRNGIKYIKKSGTRRKRFYDVADKSFHLNVTKKLRQDVCVRWNSTYLMLESSLYYKDVLDYWGQRDKDYQLFALSNEEWRNVAILCKFLKVFYDVTCVFSGSNYPTANLYFRGVWKVHKVLLDTVKGPYSFLTPMVKQMQEKFNKYWAEYSLILSCAAILDPRYKLNYVQYCFTTIYGIHASDFVETILSNLRLLFDEYVKKSKSTSSSLAGSSNVSDKNPVDSSLGEHNVNNVDFGGDFDESDDYKRYLNESSTRCEKSQLDIYLEEPELELNSQIDVLDYWSKSSVRYNELSLLARDLLAIPISTVASESAFSMGKKVITPLRSSLKPKTVQAVDCLDDWMRAKGFSTEIGCKNDEDDEDDEEDEDDEDDVSSIAF
Ghe8	Ghe08G12020	46653114	46655135	673	76.37856	5.79	-0.3	MAEMTEATNMETSPVENNNELALITPETQPKRRKKKSMVWEYFTIETVSAGCRRACCNRCKQSFAYSTGSKVAGTSHLKRHIAKGTCPALLRDQYNNQLTPYSPKTGGGEPRKRRYRSPSSPFIPFDQDHCRHEIARMIIMHEYPLHMVEHPGFIAFVQNLQPRFDKVSFNTVQGDCVATYLREKQSLMKLIEGIPGRVCLTLDMWTSNQTLGYVFITGHFIDFEWKLQRRVLNVIMEPYPDSDSALSHAVAACLSDWSLEGKLFSLIFNHPTSEAGLENLRPLLCTKNPLILNGQLLLGNCMARTLSSMAKDVLGAGHEIVKKIRDSVKYVKTSESHDEKFVQVKNQLQVPSEKSLILDNQNQWNTTYQMLAAASELKEVFNCLDTSDPDYKLAPSMEDWKVAETLCSFLKPLFDAASILMTTTNPTAITFFHEAWKIHADLGRSIANDDPFISNIAKSMLEKIDKYWKDCSLILAIAVVMDPRFKMKLVEFSFTKIFGEDAPTYIKIVDDGIHELFLEYVALPLPLTPTYTEEGNVGNNGKTDESQQGNLLSDQGLTDFDVYIMETSSQQMKSELDQYLEESLLPRVQEFDVLGWWKLNKMKYPTLSKMARDILSIPVSAAAPDSVFDIIIKQLDEYRSSLRPETVEALICAKDWLHHGSEESNALVKMEF
Ghe10	Ghe10G13030	41275117	41275332	71	7.68286	8.96	-0.39	MPPREEFLAKGSEAAPSNDIGWHFGTPVPNTKGNFICKVCGKFVKGGITRFKEHIAHKTDNVAPCPNVTGM
Ghe6	Ghe06G13010	41233386	41235521	642	73.8672	7.74	-0.323	MTMASSNSPILVDDGFNKYESAPKRQKSTTSKVWDEMTKLECEDKNKLKAQCNHCKTIFSAKSSSGTSHLRRHLNSSTQPSLGGVPFIKNYKFDADKCRQAISTFLVYGKHSFRTVEESGFRYMMRIAIPNFKNISIYTATRDLAKAPGLICLTFDNWNSEHTNDEYIRITAHWVDNYWKLQRRIISFKALFPPYDGLNIVDELVLCLSQWSIDKKIFSITLDNASYNDLMVSCLKNQFRANRAILCDGAFFQVRCCAHILNLIVEAGLELADDVVGKIRNGIKYIKKSGIRKKRFYDVADKSFHLNVTKKLRQYVCVRWNSTYLMLESSLYYKDVLDYWGQRDKDYQIFALSNEEWRNVAILCKFFKVFYDVTCVFSGSNYPTTNLYFRGVWKVHKVLLGTVKGPYSFLTPMVKQMQEKFNKYWAEYSLILSCAAILDPRYKLDYVQYCFTTIYGIHASDFVETILSNLRLLFDEYVKKSKSMFSCLAGSSNVSNKNPVDSSLDEHNVNSVDFGGDFDESDDYKRYLNESSTMSEKAQLDIYLEESALELNSQIDVLDYWSKSSVRYNELSLLARDLLAIPISTKVITPLRSSLKPKTVPAVVCLDDWMRAKGFSTEIGFKNDDDDENDEGDEDDVSSIAF
Ghe7	Ghe07G15740	28158246	28160769	647	73.98794	8.49	-0.399	MSTEPTSIEGSVTPPTSIDSENSGVGASSQANVTTGKRKATPQRSEVWSHFTKIINSEGASKAKCNYCQKEFCCDVKRNGTGSLKYHIGACKKNPSNVVDTSQGQLVLPRKGVEGGEGHLSTWRFDQEACRKLLAQMIKFMFVACPRFHIPSRTTMTKDVYKLYLDERVKIKQLLKSSCSRVCLTTDTWTSLQRVNYLCITAHFIDNDWKLNKKILNFCPISSHKGESIGMVIEKCLLNWGIDKLFTITVDNASSNDVAIGYLRKKFNLRGGLVQNEMNKSVERVRGAVRYVRQSPARLQKFKECVVLEKVECKKMLCLDVCTRWNSTYLMSDTEQDTNFRAELERGEGWPSVDDWANVRGLRDFLEHFYEVTLRISGTSYVTSNNFFDELSEIDILLRDAQLNSNVDFNVIAIKMKEKYDKYWGDIDKMNLLMFVACVLDPRQKLKYLEFALSEMSSSEKASEMMQKLKESLYELFDEYKPSLHSTCSQSSVPTHVSLGEPQQKMKRRMQALYKKRKLEICGEDKTSELDKYLAEANEEFVEDFDILLWWKVNSPRFPTLSKMARDVLAIPVSTVASESAFSTGGRVLDQYRSSLTPKIVQALVCTQDWIRKSSSQEDIKEIKEQIQELDKIENGIFITYVIFLIM
Ghe5	Ghe05	280	280	800	91.93383	9.14	-0.802	MPPREEFPTKGLEGAPSNDIGWHFGTPVPNAKGNIVCKLCGKVVKGGITRFKEHIAHKTGNVAPCPNVTESMMNVLKENNTKKIEKKRRKDDFLSQLREEEDEHEEFIDEISAIRQATRESIQSQHECHRREEFRRSTGGWDNIYEKGRSSQGSSIPTESEFTLRGTIPKLVRSKSSKQPKVSDSFLKSFRKKI

	G27120	42174	46780					ASSPWLYNLIQVSTEVGQGVKLPTPYEVSDVYLESEYQRVHNWVNGLKTHWKELGATLMCDGWTNSLNQMHIINFLVYCSKGTIFWKSVDVSSVRSRDAEFYYTMKAAGKKLMLKREHLYWTSCTAHCLDLCLEDIGKRPSVAKVLDEAKKVTCFIYNHTWTVDLMKKYTQGKQILRPALTRFATHFIQLEEITRQKQGLREMFSSKEYKESKWGQQKSGPAYEAKKIILGKDFWKKANDLIKVYEPLVKVLRLVDSDEKPTMGFIYEAVDRAKRAIQQDCRYFTEYEKIIDKRWNFMHSDLHSAGYFLNPQFQFGVEHSHNVLIETLEGTRSVIERLEPSLDTQVRMVNQVRFNSNKHETFGTPQAQRAWKQMNPAEWWIIYGTCVPELQKLAIKVLSQTTSASNCERNWSTFSYIHTKARNRLKYKKLEKLVHQKRMSTDDINTSFNPIKWLHEKENPLLDGENAGSGDGPDGGGLSPVDEDDGYNGDRGELRSSSQYRGEYGVGTTSRHFRDRSEFDGNMFPEPRRDRSEPRAPSKGKGKKHTSIGSSSGRRLGSSNLGYSDSSTSTQGFYPPEQPSYFQPSHGYPQPYGYYPPFPNYGVPYQ
Ghe5	Ghe05G22870	22163248	22166624	709	81.47332	7.44	-0.316	MNDPKRDGSTQSSQNSSPSRDDQTTTKENARKKITPRAACWSHFTKFVTKEGEKRARCNACDVTYTMESTSGSTTNLNNHLKICLKKPRGNTSNTKQSELSFVKVSQETRDLSTWVFDKDAIRKALVRMIIVDELPFKIVEGEGFKYFLSIACPRFSLPSCWTIRKDCIDLFNSMKSVMKDCFEKDISKVCLTTDTWTSLQRTSYMVLTAHWVDDEWRLQKRIINFCPISAYRGESIGQAIEKCLRDWGIERVFTITVDNASANSVAIEYLRKKLNHRNASVANGKFIHMRCVAHILNLIVQYGIKDASMSVDRVRDAVRYIRASPSRLTKFNQWVKEEMIDSKAQLCLDVPTRWNSTYMMLKVAEKYEHAFESYLRDDHNFFLDLTAGDGVPTFDDWDIVRRVIKILEPFYHLTLKVSGSLHVTSHSLFEVLIDVHCLFDGWQDCGDLEIISMTSKMREKYNKYWGEGNKINMLVYLAIIFDPRCKMSFLDFGVNLLFPNVANDIMKMIDKELHCLFNEYSSNAGRIQLFEGRSSSLTNLCSSMEIDQSEMKTGLAKQKYLKKKKQVGLESKSELDRYLGEDEEVNNSSSFDLLLWWKMNSPRFPILAQMARDILATLISTVASESAFSTGGRVRDSFRSSLTPLMVEVLVCTQDWLRKSNDAINLEDYVDELQTMEDELTLNVVGQLRIDYSICFAFPFSELSSFCS
Ghe13	Ghe13G09140	21072784	21076326	631	72.27188	7.94	-0.402	MSTEPTSIKGSVTPPTSIDSENSGVGASSQANMTIGKRKATPQRSEVWSHFTKIINSEGASKAKCNYCQKEFCCDVKRNGTGSLKYHIGACKKNPSNVIDASQGQLVLPRNGVEGGEGHLSTWRFDQEACRKGLAQMIKFMFVACPRFHIPSRTTMTRDVYQLYLDERVKIKQLLKSSCSRVCLTTDTWTSLQRVNYLCITAHFIDNDWKLNKKILKFCPISSHKVESIGMVIEKCLLNWGIDKLFTITVDNASSNDVAIGYLRKKFNPRGGLGAVRYVRQSPARLQKFKECVVVEKIECKKMLCLDVCTRWNSTYLMLDTEQDTNFRAELERGEGWPSVDDWANVRDLRDFLEHFYEVTLRISGTSYVTSNNFFDELSEIDILLRDAQLNSNVDFNVMAIKMKEKYDKYWGDIDKMNLLMFVACVLDPRQKLKYLEFALSEMSSSEKAFEMMQKLKESLYELFDEYKPSLHSTCSQSSVPTHVSLGEPQQKMKRRMQGLYKKRELEICGEDKTSELDKYLAEANEEFVEDFDILLWWKVNSPRFPTLSKMARDVLAIPVSTVASESTFSTGGRVLDQYRSSLTPKIVQALVCTQDWIRKSSLQEDIKEIEEQIQELDKIENGIFINTYII
Ghe9	Ghe09G05740	15868365	15870737	652	74.89293	7.71	-0.438	MSTEPTSIKGSVTPPTSIDSENSGVGASSQANVTTGKRKATPQRSEVWSHFTKIINSEGASKAKCNYCQKEFCCDVKKNGTGSLKYHIGSCKKNPSNVVDRSQGQLVLPRKGVEGGEGHLSTWRFDQEACRKGLAQMIVIDELPFKFVESEGFKKFMFVACPRFHIPSRTTMTRDVYQLYLDERVKIKQLLKSSCSRVCLTTDTWTSLQRVNYLCITAHFIDNNWKLNKKILNFCPISSHKGESIGMVIEKCLLNWGIDKLFTVTVDNASSNDVAIGYLRKKFNPRGGLVQNGKYLHMRCMAHIVNLIVVEGLKEMNKSVERVRGAVRYVRQSPARLQKFKECVVMEKIECKKMLCLDVCTRWNSTYLMLDTAQKFERAFERFEEQDTNFRAELERGEGWPSVDDWANVRNLKDFLEHFYEVTLRISGTSYVTSNNFFDELSEIDILLRDAQLNSNVDFNVMAIKMKEKYDKYWGDIDKMNLLMFVACVLDPRQKLKYLEFALSEMSSSEKACEMMQKLKESLYELFDEYKPPLYSTCTQSSVPTHVSLGEPQQKMKRRMQALYKKRELEIGGEDKTSELDKYLAEANEDFIEDFDILLDVLAIPVSTVASESTFSTGGRVLDQYRKLDMIENGIMQMEIFWREEMDTNGEY
Ghe6	Ghe06G06550	9767914	9770082	722	82.57394	5.99	-0.216	MFALDELLEAVVLLYIYIYTHIHTYISWTTFLKFLFLSAAGFTSMTEMTEIADMETIPGESNNQLALTTPEAQPIKRRKKKSMVWEYFTIENVSAGCRRAYCKRCKQSFAYSTGSKVAGTSHLKRHIAKGTCRALLRGQGQDNNQFITPYNPKMGGSEPPKRRYRSPSSPFIPFDQDRCRHEIARMIIMHEYPLHIVEHPGFIAFVQSLQPQFDKMSFNTVQGDCVATYLREKQSLMKFIEGIPGRFCLTLDMWSSNQTLGYVFITGHFVDSDWKLHRRVFNVVMEPYPDSHSALSHAIAACLSDWSLEGKLFSLTFNHPLSEAGLENLRPLLCVKNPLILNGQLLIRNCIARTMSSMAKDVLGAGQEIIKKIRESVKYVKMSESHDDKFIQVKNQLQVPSEKSLFLDNQTRWNTTYQMLAAASELKEVFDCLDTYDPDYKLAPSMEDWKLAETLCSFLKPLFDAASILTTTTLPTVITFFYEVWKIHVDLGRSITSEDPFISNLAKSMQEKIDKYWKDCSLVLAMAVVMDPRFKMKLVEFSFTKIYSEDAPTYIKTVDDGIHELFLEYVALPLPLTPTYAEEVNGANNGKTNESHYGNLLSDHGLTDFDVYIMETNSQQMKSELDQYLEESLLPRVQEFDVVGWWKLNKMKYPTLSKMARDILSIPVSAAAPESIFDITDKQLDEYRSSLRPETVEALICAKDWLHFGSSDVSNALVKMEF
Ghe6	Ghe06G06540	9761589	9763757	722	82.50187	6.06	-0.212	MFALDELLEAVVLLYIYIYTHIHTYISWTTFLKFLFLSAAGFTSMTEMTEIADMETIPGESNNQLALTTPEAQPIKRRKKKSMVWEYFTIENVSAGCRRAYCKRCKQSFAYSTGSKVAGTSHLKRHIAKGTCRALLRGQGQDNNQFITPYNPKMGGSEPPKRRYRSPSSPFIPFDQDRCRHDIARMIIMHEYPLHIVEHPGFIAFVQSLQPQFDKMSFNTVQGDCVATYLREKQSLMKFIEGIPGRFCLTLDMWSSNQTLGYVFITGHFVDSDWKLHRRVFNVVMEPYPDSHSALSHAIAACISDWSLEGKLFSLTFNHPLSEAGLENLRPLLCVKNPLILNGQLLIRNCIARTMSSMAKDVLGAGQEIIKKIRDSVKYVKMSESHDDKFIQVKNQLQVPSEKSLFLDNQTRWNTTYQMLAAASELKEVFDCLDTYDPDYKLAPSMEDWKLAETLCSFLKPLFDAASILTTTTLPTVITFFYEVWKIHVDLGRSITSEDPFISNLAKSMQEKIDKYWKDCSLVLAMAVVMDPRFKMKLVEFSFTKIYSEGAPTYIKTVDDGIHELFLEYVALPLPLTPTYAEEVNGANNGKTNESHYGNLLSDHGLTDFDVYIMETNSQQMKSELDQYLEESLLPRVQEFDVLGWWKLNKMKYPTLSKMARDILSIPVSAAAPESIFDITDKQLDEYRSSLRPETVEALICAKDWLHFGSSDVSNALVKMEF
Ghe11	Ghe11G11080	9597257	9600949	695	79.55106	8.6	-0.251	MASSNSPIRVDDGFNEYESAAKRQKSTTSKVWDEMTKLECENKNELKAQCNHCKTIFSAKSSSGTSHLRRHLNSCLKKVNKDISQYTIATQPSLGGVPFIKNYKFDADECRKAVSTFLVCGKHSFRTVEEPGFRYMMSIASPNFKNISRYTAARDVLMYYAKERDRVKEELARAPGLICLTSDNWNSEHTNDEYICITAHWVDENWKLQKRIIRFRALFPPYDGLNIADELVLCLSQWGIDKKIFSITLDNASYNDVMVSCLKNRFRANRAILCDGAFFQVRCCAHILNLIVKAGLELADDVVAKIRNGIKYIKKSGTRRKRFYDVADKSFHLNVTKKLRQDVCVRWNSTYLMLESSLYYKDVLDYWGQRDKDYQLFALSNEEWRNVAILCKFLKVFYDVTCVFSGSNYPTANLYFRGVWKVHKVLLDTVKGPYSFLTPMVKQMQEKFNKYWAEYSLILSCAAILDPRYKLNYVQYCFTTIYGIHASDFVETILSNLRLLFDEYVKKSKSTSSSLAGSSNVSDKNPVDSSLGEHNVNNVDFGGDFDESDDYKRYLNESSTRSEKSQLDIYLEEPELELNSQIDVLDYWSKSSVRYNELSLLARDLLAIPISTVASESAFSMGKKVITPLRSSLKPKTVQAVVCLDDWIRAKGFSIGNYYSRFIVFGVVGDAVFCAVLLLFGCCFGAHEIEALICL
Ghe4	Ghe04G04930	7010148	7013266	663	76.65161	5.85	-0.335	MSDAVIVNSSRLKSIVWNDFDRVKKGDTFVAICRHCKKKLSGSSTSGTSHLRNHLIRCQRRSNHGVAQYFSAKDKKKEGSLALVTIDQEQKNDEVLSIVNLRYEQEQIKSEHVGIGSNSLDQRRSQFDLARMIILHNYPLAMVEHVGFKIFVRNLQPLFELATRNKVEADCMEIYAKEKQKVYEIFDKLPGKISVSADVWTASEDDAAYLSLAAHYIDENWQLKKKNLNFVTIDPSYTEDMHSEVIMNCLMDWDIDRKLFSMIFDSFTSDNIVERIRDRLSQNRFLYCNGQLFDVRCAVDLLNRMAHDALEALCEITQKIRESIRYVKSSEATQATFNELADEVQVETKKCLCIDNPLKWNSTYFMLEAALEYRKVFSCLRDRDPVNMKFLLSDPEWDRLITVTSFLKLFVEVTNVFTRSKYPTANIFFPEICDIHLQLIEWCKNPDEYISSLALKMRKKFEEYWYKCSSGLAVAAMLDPRFKMKLLEYYYPQLYGDSATELIDDVFECIKSLYNEHSIVSPLASSIDQGLDWQASGISGSGKDSRDRLMGFDKYLHETCQAEGSSSDLDKYLEEPLFPRNVDFNV

								LNWWKVHTPRYPILSMMARNILGIPISKVAAESRFDTGGRVLDHNWSSLPPTTIQALMCSQDWIRSELETNLIPLPQ
Ghe3	Ghe03G06320	6237648	6240605	629	72.77772	7.43	-0.438	MSTEPTSIEGSVTPPTSIDSENSGVELQAKQMSEVWSHFTEIINNEGASKAKCNYCQKEFCCDVKKNGTRSLKYHIGSCKKNPSNVVDPSQGQLVLPRKGVEGGEGHLSTWRFDQEACRKGLAQMIVIDELPFKFVESEVFKKFMFVACSKFHITSRTTMTRDVYQLYLDERVKIKQLLRSSFNYLCITTHFIDNDWKLNKILNFCPISSHKGESIGMVIEKCLLNWGIDKLFTVTVDNASSNDVAIGYLRKKFNPRGGLVQNGKYLHMRCMAHIVNLIVVEGLKEMNKFVERVRGAARYVRQSPARLQKCKECVVVEKIECKKILCLDVCTRWNSTYLMLDTAQNFERAFERFEEQDTNFRAELERRKGWPSVDDWTIVRDLRDFLEHFYELSEIDILLRDAQLNSNVDFNVMAIKMKEKYDKYWGDIDKMNLLMFVACVLDPRQKLKYLEFALSEMSSSEKASEMMQKLKESLYELFDEYKPSLHSTCSQSSVSTHVSFGEPQQKMKRRMQALYKKRELEICGEDKTSELDKYLAEANEEFVKDFDILLWWKVNSPRFPTLSKMARDMLAIPVSTVASESSLTPKIVQALVCTQDWIRKSSSQEDIKKIEEQIQELDKIENDKNIAT
Ghe3	Ghe03G06060	5907043	5907454	100	11.06588	9.47	-0.45	MPPLEEFPTKGLEGAPSNYIGWHFGTPVPNTKRNVVCKLCGKVVKGGITRFKEHIAHKIGNVAPCPNVTGVIRESMMNILKESNTTKIDKRGEQMNSYLN
Ghe12	Ghe12G03660	5275686	5278299	690	79.29524	8.28	-0.417	MSTEPTSIKGSVTPPTSIDSENSGVGASSQANVTTGKRKATPQRSEVWSHFTKIINSEGASKAKCNYCQKEFCCDVKKNGTGSLKYHIGSCKKNPSNVVDPSQGQLVLPRKGVEGGEGHLSTWRFDQEACRKGLAQMIVIDELPFKFVESEGFKKFMFVACPRFHIPSRTTMTRDVYQLYLDERVKIKQLLKSSCSRVCLTTDTWTSLQRVNYLCITAHFIDNDWKLNKKILNFCPISSHKGESIGMVIEKCLLNWGIDKLFTVTVDNASSNDVAIGYLRKKFNPRGGLVQNGKYLHMRCMAHIVNLIVVEGLKEMNKSVERVRGAVRYVRQSPARLQKFKECVVMEKIECKKMLCLDVCTRWNSTYLMLDTAQKFERAFERFEEQDTNFRAELERGEGWPSVDDWANVRNLRDFLEHFYEVTLRISGTSYVTSNNFFDELSEIDILLRDAQLNSNVDFNVMAIKMKEKYDKYWGDIDKMNLLMFVACVLDPRQKLKYLEFALSEMSSSEKACEMMQKLKESLYELFDEYKPPLYSTCSQSSVPTHVSLGEPQQKMKRRMQALYKKCELEIGGEDKTSELDKYLAEANEDFIENFDILLWWKMNSPRFPILSKMARDVLAIPVSTVASESSLTPKIVQALVCTQDWIRKSSSQEDIKKIEEQIQELDMIENDVNAVKKFIREWEKGIWVS
Ghe11	Ghe11G00830	7383305	7419771	698	79.22183	7.79	-0.349	MTFGLHLLQIVSFVGNKMEWSVNNAFKSYKDMEPKSTMDMVLIPNMDTIDIVLGSSEKGNVVPSAKPRKKTMTSVYLKYFETAPDGKTRRCKFCGQSYSIATATGNLGRHLSNRHPGYDKTGENVSSSAPQPSTTPTVIKKPQPQGRAPQVDYDHLNWLLIKWLILATLPPSTLEEKWLANSFKFLNPSIQLWPGEKYKAVFREVFRSMREDVRASLEQVSSKVSIALDFWSSYEQIFYMSITCQWIDENWSFQKVLLDICQVPYPCTGSEIYNSLVKVLKMYNIENKVLSCTHDNSQNAIHACHALKEDLDGQKMGPFCFIPCAARTLSLIIDDALRTTKPVIAKVREFVQELNASLDISEDFIQLATAYKEGSWQFPLDASARWSGSYQMLDIVQKAGKSMDAVVRKNEELLGNRMLLNTAEKNVVNIVHNYLEPFYKVISEICVNTPPTIGMVIVYMDHISDTITTRQPPDWLKNPAEDMAKKLRSYNNQVCNIFIHMTAILDPRIKCELIPESLNSKNYLEEARAHFVRNYYTTPFSSMTSGYSSQDIEDGGAVSFAEEIARKKRRVSMNNATDELTQYLSESPTPTKTDVLEWWKVNSTRYPRLSAMARDFLAVQATSVKPDELFCSKGDEIDKQRFCMPHDSTQAILCIKSWTQGGLKLKYKSTEIDYERLMEMAAAATADISLAGMDKKQK
GheS	GheUnG14910	3014446	3042265	657	74.97991	7.79	-0.347	MLRALAVSCFVLKIMVRGRDACWEHCVLVDATRQKVRCNYCHREFSGGVYRMKFHLAQIKNKDIVPCAEVPDDVRDHIQSILNTPKKQKTPKKPKMDKTVANGQQNSSSASGGLHPNHGSSGQHGSTCPSLLFPHPSPSEQPATDDAQKQKLDDADKKIAVFFFHNSIPFSAAKSMYYQEMVDAIAECGVGYKAPSYEKLRSSLLEKVKGDIHDGYKKYREEWKETGCTVLCNSWSDGRTKSFVIFSVTYPKGTLFLKSVDVSGHEDDASYLFELLESVVLEVGLENVIQVITDSTASYVCAGRHLMAKYSSLFWSPCASYCIDKMLEDISKQEWVGIVLEEAKTIARYIYSHAWILNMMRKFTGGRELMRPRITRFVDNYLNLRSIVFQEDNLKHMFSHSEWLSSIYSRRSDAQAIKSLLYLERFWKSAREAVSVSESLVKILRIVDGDMPAMGYIYEGIERAKGAIKAYYKGIEEKYMPIWDIIDRRWNMQLHSPLHAAAAFLNPSIFYNPNFKIDLRMRNGFQEAMLKMATMDKDKIEITKEHPVYINAQGALGTDFAIMGRTLNAPGDWWASYGYEIPTLQRVAIRILSQPCSFHWCRWNWSTFETGRDGKCKPIIFDEIDVSSEWPTESEPSVPLLDDSWLDNLPLECRGSP

Table S21 List of ZmZF-BED genes and their features

Chromosome no.	Gene_ID	Start	End	Protein_sequence
Zm1	Zm00001d033903	278102123	278108344	MGEPNSNDNAMVHDNEMIDGNGVIHGSEMIHGSVMIHGDEMPHGNEMIHGNEMIHGTEMVEGSEMIHGHEMVQVNDLIHGNEMVAVNVMVNGDEMPHVNEFINTEVTPRRRRKKKSLVWEHFTIEPMPGGNSRACCNLCKQTFAYSSGSKIAGTSHLKRHITLGSCPVMKDQDRRLTLPLAGGHATDNDGEGTVERPTKRRYRYTGYANATFDQERSSSYLAKMIILHDYPLHIVQQSSFTKFIESLQPRFRVVDVETMEGEVYAVFQKAKENLLQAFSTMPGRISLTIGLWTTSQTLGYVSLAGQFIDSDWKVHRRMLNFMMVSSPHSENALSEAISSSLLEWNMKDRLFTITLDNDCSSHDIYSANLRDHLSNKNNLMLKGQLFVVRCYAHILNVVAQDVIASIHGVIYNIRESIKFIKASPNRGLLFSMGLLGYIFSKSTISLVLGITPGLATLLLGTQSEVLEERQIQLPAYLGSGR
Zm1	Zm00001d033361	260215313	260294650	MTLGWAAKRSAPRAHQRDNPSSVASASITQSTAATIKSDDPAWKHCYCPDLKKKHSLKCNYCDKLINAGITRVKYHLANIAGFNVSKCKKVPTPVKEDMVALLTKNCDAKEKKRKEKQRERDEIDLDNSGGDNSSEEESEHGNDVIVFKSTKGGSSSRLATTGGTIDKFYKPESIEESVQKNKRGLSTSQKIQTQLTTQKREERRDRACEYICQFFYEAGIAHNTVTLPSFAHMVEAIGAFGRGLRGPSSYEMSGPFLKKCKEKLSTVLEDLAKIGAVEETISSAKHVTAFLYAHTRVLDLMRKFLGKDLVHSGVTRFATAYLNLKSLQDNKKDISRLFRSDELNELGYLKKAKGKKANKVVRSEGFWKNVDMVVNFFEPLANVLRRMDSDVPAMGFLHGCMLEAKKEIAMRFDNNENSFKVAWDITDKRWDNKLKTPLHLAGYYLNPYFYYPNKSEIELDGSFRAVVIACITKTVEDEETQDNIIEELNVYQEQQGTFGHDIAVRQRRNKNFNPAKWWLNHGTSTPNLRLLATRILNLTCSSSGCERNWSDFEQVHTKKRNKLLHDRMRDLVFIKFNSRLRQKRENKSKDPLEREMNDVLEDDANEFITGLVPNANSDKDEEHGGAQVGATIHEPQLSQPQPKRKRLVRPRKKKIRSLHSLLYGGLENEAVASSSESEDNGDGDISMHQYSDSDDLGDE
Zm2	Zm00001d006692	214487406	214491116	MERVSSAPEMDDVISTDQSSRSTKRRAKVWDYVDSELVDGKEKAICKYCKAHLSSVAGKGTTHLNRHISTYCHAISQEERQRFLATQKTKPDEAHVFDPVVFRGLITNMAEKTIWGDDLVKHLIDVCKEEILAGNRPQGIFTRIGWKNVEDKFFARTKKKCTKTQLKNKLDNLKKDFTQFMELKIVATGLGWNEANQTVDCSNTWWDEHLEKCNNPERGTKCNHVRFRKHGPKHLDDLHFLFDKVHVTGATAMCPGDVSSCDSSSDDVLEVTEKTNEAWNKPKPKKRKQMSGAAQEKEEKSPFYQMYKNTCMKIESADEKISTSVEASSAPLQANLVPTIAEAMKMVKACGIQEKTAMMHTATSLIMKAEFREILFALETNEGRFDLIEREHKKSTT
Zm5	Zm00001d017846	208632549	208641965	MDLEVSEGSGTNATSDDQSVQTLPRRAKLLEYFQPELVEVNGVMKAICKYCGAMLRSSSTNSLRNHVADTCPKISVEDRKQFIATMEKGPAEASFVFDSQKARECMVKWCISAEVAFNKFDDPFFAQWMESMQPSFSGVGRQTMQNDCITRFKMMRQELRNELQSLNSRICLTTNLWTSNQKLGYLCLTAHYINADFILKKKIIAFKYLKYPHTGLAIKEGITECLKEWGIKEKMFTITLDNVSNNLSVRDLRESGLFGGDLRIVQCCAHILILLVQDGMAIAHGAIYKIRELVRHINSSPSRIQAFNGIAERSGLPSKDRLILDVPNRWNSTHDMIMEAIEYKVVLKRYAEEQLEPSPNDEEWTNSEAIGEFLEAFEKATKAFSTHRSPTSHLFLHNVLCIHRTLRNTNWQINCVLKDSARAMDAKFDKYWEKGKYNMTLVLATILDPSKKMDFLDFFYEKMSQYFVDIQINVVLVKQCLTKLFEKYATLVQIDNKSSPFIDIRTSNLGSTVLGKRRLDEEFSQWSQIRGRRFPKSELDTYLEEELVRIDERFEILNWWRTNANKYPVLSAMARDILAIPLSTVPSEFAFSAGGRILDDNRSTMTPETLECFVCCKDWLYEYPNIQA
Zm3	Zm00001d043354	197463158	197466335	MEGVSSAPEMDEAISTDQSSRSTKRRAKVWDHVDSELIDGKEKAVCKYCKAHLSSAAGKGEPMTGKRMFDSDDEEYDWEI
Zm7	Zm00001d022534	179612706	179615009	MAVETASDAQVAQDNEISPSNEAIQGDELAHGEELFQGDDLVQGNELVVSEVTTPPTTGIRRRRKKKSLVWEHFTIEAVAGGATRACCNLCKQTFAYSSGSKIAGTSHLKRHITLGSCPKIKDQDQRLALPSTGGTDNYGEATSERPTKRRYRYTGYANAVFDQDRSCSYLARMIIQHDYPLHIVQQQAFSVFIESLQPRFKIVDVDTMEGEVYAVYQKEKENLLRSFNTMPGRISLTIGLWTTSQTLGYVSLAGQFIDSEWKVRRRMLNFMMVSSPHSENALSEAISLSLSDWSMKDKLFTITLDNECSSHDIYSANLRDHLSNKNSLMLKGQLFVVRCYAHILNAVAQDVIASVHGVVYNIRESIKFIKASSAREEKCVDSDVLVVVM
Zm5	Zm00001d016617	170299068	170321300	MSASRPSSGRQPLCPPLCPPLAACPSARRPPPAHLCPPPSSGCVPSSGRRPDAHLQPSRARGLQPLRRPPLAAAHLQPSRKPAHLCPDKSQQPVPPSQVTTSSRDMEDASADRPQGESGSQQPYDPAKDPKKKAKSKDPAWNYCYWLDLNKKDVVKCILCGKIVHAGVRRLKQHLVGGHAKRCDYLKNNARQKPIELDDDKEGDKDDEVEFEASNEVRLAVYFSFVQFQTSKECSIEGAGGSLPDEGLCSRRRLESGITVDVVCTEKPHIAPTWIIPWSPSFNQRAEELDRAIVLNVTVAINEAVEEAILANFARRLELVHDSLSLCRLGPASFLLILPSLEMADRAYDGGRIVNLSPTTKIHVMRWSRFLNSSVTASLPFHVEIEVSGIPAHAWELESVQALLNEWCWIADLHPDCERQREVFKVIAWCSSLSSIPREFKLQIVDPVTSDGGMLRRSLVYPIRVSVKVLDRPNLLQSLQPSPPPVEDDPDQGRRRRRRRSRRSSFGINAPGKSSTTAVLPRASVHQRLGPCVQVFVKEGIRMPRLPVHNRLGPLEVFIAEKGAIKKMKRVWIPKRKL
Zm9	Zm00001d047922	145490841	145491392	MDLRISEGSGTNTTLDDQSVQSPTRRAKVWEYFQQELVEVDGVMKAVCKYCGTKLTSKRNSGTNSLRNHVADTCPKISVEDRKRFIATMRKKPGEGSFVFDPRKTRECMVKWCISAEVAFNKFDDPFFAPWMESLQPSFSGVGRQTMRNDCIARFKMMRQELRNELQSLNSRICLTSDLWTSN
Zm10	Zm00001d026358	144194271	144197480	MEHHIIQQVGEHEGEEEEDGMGTEAAFEGVHNHDTFRYKHKKRSKVWEEYKPIFLNGKVQFAECLYCHSRMSCKDSNGTSHLWRHQKICPGKRDVVFRRLKDSYFPCVLVNQSEPVTPGDSVNQIISETLDDISSVIPNRFKSKVWREFSPIYVEGKLQAADCVHCRKRLSANKFGGRSHLSRHLQTCQARRGYNNQKGTLYPSSVPDLKSIGQDELSPALANGKKILSLTSVGEVRNALSSSKLKEILVEKKCLPIRGKLYDVACVDDVLNTIASKVQQNIIHLVGDMVTEFFVAHTSSSLNQQQLMEVISQMSLKCPQEDAKWWYKFYFRLEVFLHFNKPFPFEEAASPEHVIVAESICKILRTFYRVIEVISASSSPTANMYFNEIWKVRTVLQEEASSEHAEITAMVAEMQETFNEYWQHSYLWLCIPVILDPRFKFGFIEFRLKRAFGLKSASYLSGIRETLQELFNEYCSPVDQPNVRVPKSESFSLDDNDSFEDWDQHLNEQASSQKSTELDNYLADGLVPRKDDFDNLNWWMCHATKYPTLAAIVQDILAMPASAVPSEAAFTSSGPVIPKHHSMLSIKTIEALVCTRDWMR
Zm8	Zm00001d011158	141169109	141194917	MIDDEESQGIKKVHFRDSFSQHPIVTYKRRRQQKPQTQQQLRQPPPSLQQHPEPQLQVKLEPKTEDVPEQQNNDTFWKSRDMGWKYGIMIDENRQHWKCMYCGLIRYGGGVSRLKRHLAGDLDVKMCPEVPADVVEEIREHLRKKRERRRKRAAQNGGDNVKAKNPSGDANVEKDLLPSNSVLPDGMDTNVLEEVTNQTGSVHHETTPRFPILRARDIGWEHAVDLDGNKRRWQCKFCSLCRSGGVTTLKAHLIDDSCPNVPKEISKKVSNFIEEKRATRLLLNNYVFNVDEDFNTILQEGTVEYVNEQQPSRKATYVQTLSKCAINEIAAGSKQCGAECSGQPVEHCDQPEEQCTMDYGRMDRLTSNKNQILDKNTENSKNTKMLKPCRKSEFNTRKHIIIVDKIGRHWKCRYCGMDVYGKKFQLHYHLAGAFRQAKCPNVPREVFAKARQHVLTKKMLKKSKAEQQIPSSPHILAQSGEERQNNDPFCGNQSQLSINNEPREVHNYPAVLRDSAWEHSLIYEKENGHWKCKWCSIEGDHGLTRLMWHLVGWQNRPQCPNIPKDVAEKMKDQMMSKKEQKARSGLFDGNGCCEVLCSSNSSQLDQNHLTARIH

				DRCSSQAFDHANSELKGCNMLSNTILSQQSSNPQVHHEDPQVCHEQERKEVATSSEPGCEQGQRMQWQSQNKPMMEGPHDNGLCGDTNQLEEQKSGFGISDCWRYVLDGQMHLPDVQEGAGIGTCIRDALLYGCAEFGTVPDKMEMDCDKTVDANTAKCQNILKDVLRSENFALLCSVLCRTVHQDGERTRYFDFGVIDSRMKNGNYGPEPELFVHDLKLLWEDLKVAGQDIVHLANNLSSLTEDSYEKLVGRERGSDDDELNEAVVARSEPKNLVQPNASVPLTSQGFNQLLDQPGPSDPSVVYKDSICNRCGKVAGAGSVLKCYRCMLPCHISCIEATGSPISTGRWCCKNCSAGTKEPVEGDMVLAHGNPNCLHESCVVCDRLAACRSPKCDNSRALVISSVDPEIDTCYSCKICGGTEEDEKRFLICGNVLCRYMYYHIGCLKSMQISTSVERGSPCWYCPSCLCRVCLCDKDDDLTILCDGCDEAYHIYCITPRHTSIPKGQWYCSSCSVERAEEGMRQYERRTLKLHRKEDAGLQSWNFDGVDLLLSAAEQLRIDEQLETRTD
Zm8	Zm00001d010895	132443170	132444536	MSDEDRDREIERDRVWLHEDKIDTGFICKYCRETKSEGSDTRLKEHLAHRGKNDKKCPSVPPDIKDLWYPIGKFGTNYMFLESFMRRNDKFMVWFMSPEIRHSSYFLTEMERYAFDNITNVELWENMQYVLDEVEPLYMFLTFVDQEKSPTLGEVHMQYTNTKHTYQSKFENDSARYNTIMDVVDAKMNTVMTDTYVQPVCALHPYVNYVMGATNNLMTDLRKGVERMFDSNTAAMALQEYDFFKRKIGDFSSELARRMVVDRGTSPSSWWSMFGSDTPTLQRVAKRLLSLCASSSGCERNWSTFAFIHTKLRNKLDYLKLHELVFVNYNLHLRIQRATGTPEPSEFDPALAFMDLSLHRHNKAIRDWMERGRCNAPPTLDEDSTISDTPLPSTLFTLLVHEQGGTEKV
Zm4	Zm00001d050879	128119733	128121029	MSDEDRDRERDRDKVWLHGEKVGAGFKCKYCRETKSGGGGTRLKEHLVHRGKNVKKCPSVPPDIKAYFQLDIDKTKENKSSRFRQQLRADEAARTHFGDDEYEDELKAALHQSQEEEDEDSDDNTTDPSAGDDGSHGDAGLYGVGGSGAGGSGTGGSGAGDWRSTGGSRFTHSTQDSYHDALYSQRETISGRRRSVSFPVQDGIRSSSSSGSSNYPTGQDLVYNPYAWQWQPSQRTYGPPPPLGEAPPSIMYRYGNYGPPPPPYSNLSNMYPPGPQYGYGQVPAAPTFHYQYDGLDQYHDPSNMPEYYHYDSS
Zm10	Zm00001d025324	114125068	114128392	MGGDCSPSSNLPRGISWMRRPRFPEHRLRLGRRLQRVHGQDSVTTSEFEASICSVYGTKSTTSSLLQQDGNSGEPSSVNSASVTQKTSNAIKSDDPTWEHCFVPDMSKKHAIQCKYCDKVIHGGITRVKYHLANIGGFNVTKCKKVPAPVNQEMEALLTKKTSEKEQRKKEKQRERDEIDLDKSDGSNEEDNEHENDIVVLKSTRNDRMRDLVFVKFNSKLREKRNNKSKDPIEKELNDILEDGGNEFITGVVPDENVDQDEDHEGAHDEPSQEPSISKAQLPAKRKRHGHPRKKKLRSLKSLLSGDLEPATCASSSESEDNESMQIEASNSDSGDE
Zm10	Zm00001d025207	109142174	109143177	MPRYSLLSRHIEATVVPRYNTEERGRKSSVRSGARSSSRVRAAAREWGSCWAVAGKKEPERQMAVAGKKEPENEPTGGEAAGALAGRGGDKELVDCWSWKSAMERVSSALEMDEAASTDQSSRLTKRRAKVWDYVDTEVVDGKEKAVCKYCKVHLSSAVGKGTTHLNRHIFVYCHAIPQEERQMFLATQKTKPGDDHVFDPVVFHGLIAKYFLSAEISFRKCEDPSWKEMIIYYQPSFRLVGRQTVRSDCVLLYEEEKLQLIEQFTKLKSHVSLTADL
Zm2	Zm00001d004256	97279244	97293487	MGQLEATQIDTNTKLANVEMTVAHIDKSLVALLRRFDEMHANINGGRDEGAEDNPSSVASSSITQSTAATIKSDDPAWKHCYCPDLKKKHSLKCNYCDKLINAGITRVKYHLANIGGFNVSKCKKVPTPVKEDMVALLTKNCDAKEKKRKEKQRERDEIDLDNSGGDNSSEEESEHGNDVIVLKSTKGGSSSRLATTGGTIDKFYKPESIEESVQKNKRGLSTSQKIQTQLTTQKREERRDRACEYICQFFYEAGIAHNTVTLPSFAHMVEAIGAFGRANVLRRMDSDVPAMGFLHGCMLEAKKEIAMRFDNNENSFKVAWDIIDKRWDNKLKTPLHLAGYYLNPYFYYPNKSEIELDGSFRAAVIACITKTVEDEETQDNIIEELNVYQEQQGTFGHDIAVRQRRNKNFNPAKWWLNHGTSTPNLRLLATRILNLTCSSSGCERNWSDFEQVHTKKRNKLLHDRMRDLVFIKYNSRLRQKRENKSKDPLEREMNDVLEDDANEFITGLVPNANSDKDEEHGGAQVGETIHESQLSQPQPKRKRLVRPRKKKIRSLHSLLYGGLENEAVASSSESEDNGDGDISMHQYSDSDDLGDE
Zm5	Zm00001d015283	82651101	82653444	MDLRISEGSGTSTTLDDQSVQSPTRRAKVWEYFEQELVEVDRVMKAVCKYCGTKLTSKRNSGTNSLRNLVADTCPKIPVEDQKRFIATMRKRPGEGSFVFDPRKTRECMVKWCISAEVAFNKFDDPFFAPWMESLQPLFSGVGRQTMRNDCIARFKMMRQELRNELQSLNSRICLTSDLWTSNQKLGYLCLTVHYIDANFILKKKTIAFKDVKYPHTGLAIEEVITKCLIEWGIKEKVFTITLDNASNNQSACDLIRESGRSDMLFGGEHLHVRCCAHILNLLVQDGMAIAHGAFDKIRDLFIPLCLDPASSNTLSASGNGEIYASFSGLLMLSRCPLPLLLASSLITDSFHCC
Zm1	Zm00001d029192	61646122	61648831	MLPVDEEVLMLRPASALSAAADSKSLAEKAIAALPPELSAQATDPKRKARSQDPGWKFEWWPDTTKKDFVQCIFCSKIVPSGIKRFKQHLAGGFGDTMKCARVPELVSKEMHMYLKRNMRLVITANTEEGEEGEERNDEGAASSSRPQQTYVRTRKRSRNAAAQDIGEDDSDAQED
Zm1	Zm00001d028972	53302218	53302435	MDLGVCEGSSTNITSDYHSVQMSTRTTKVWEYFQQELVEVDGVMKAICKYCGTKLTRKRNSCTNSLRNHVADTCPKILVEDRKRFIATMRKKPGEGSFVFDPRKTHECMVKWCISAEVAFNKFDDPFFAPWMESLQPSFSAFNEIAERSGFPSKAGLILDVPNCCNSTHNMIMEAIEYKVVLKRYAEEQLEPSPDDEEWTNSEAIGEFLGGI
Zm2	Zm00001d003194	35276371	35286449	MDLRISEGSGTNTTLDDQSVQSPTRRAKVWEYFQQELVEVDGVMKAVCKYCGTKLTSKRNSGTNSLRNHVADTCSKIPVEDRKRFIATMRKRPGEGSFVFDPRKNRECMVKWCISVKVTFNKFDDPFFASWMESLQPSFSGVGRQTMRNDCIVRFKMMRQELRNELQSLNSRICLTSDLWTSNQKLGYLCLTAHYIDANFILKKKTIAFKDVKYPHTGLAIEEVITKCLIEWGIKEKVFTITLDNASNNQSACDLLRESGRSDMLFGGEHLHVRCCAHILNLLVQDGMAIAHGAIDKIRDLVRHIISSPSRIQAFNEIAERSGLSSKAGLILDVPNRWNSTHNMIMEAIEYKVVLKRYTEEQLEPSPDDEEWTNSEAIGEFLGAFEEATKAFSAHRSPTSHLFLHNVLCIHQALRNENWQINCVLEDLALAMDSKFDKY
Zm2	Zm00001d003128	33305015	33307758	MDLRISEGSGTNTTLDDQSIQSPTRRAKVWEYFLQELVEVDGVMKAVCKYCGAKLTSKRNSGTNSLRNHVADTCPKIPVEDRKQFIATMRKRTGEGSFVFDPRKTRECMVKWCISAEVAFNKFDDPFFATWMESLQPSFSGVGRQTMRNDCIARFKMMRQELRNELQSLNSRICLTSDLWTSNQKLGYLCLTAHYIDANFILKKKTIAFKDVKYPHTSLAVEEVITKCLIEWGIKEKVFTITLDNASNNQSACDLLRESGRSDMLFGGEHLHVRCCAHILNLLVQDGMAIAHGAIDKIRDLVRHIISSPSRIQAFNEIAERSGLPSKAGLILDVPNRWNSTHNMIMEAIEYKVVLKRYAEEQLEPSPDDEEWTNSEAIGEFLGAFEEATKAFSAHRSPTSHLFLHNVLCIHQALRNENWQINCVLEDLALAMDSKFDKYWEKGKYNMALVIATILDPSKKMDFLDFFYEKTCQQFIDIQINMSLAKQWFTKFFGEYAKIVQKDTTVSPIVATNTSTLGSPILGKRRLDKEFSQWSQTRGRRLAKSELDAYLEEEFVRTDERFEILSWWRTNTNKYPVLSAMARDLLAIPLSTVPSESAFSAGGRILADNRSSMTPETLECLVCCKDWLYEYPNIQVYPVVFGPS
Zm4	Zm00001d049450	30698430	30698788	MDHSDEHDSDDMKVGAASDGPSRLRKKRSKVWDEYEPIIVDGAIQSAECRYCHMHMSCRGADGQSNGTSHLWRHQKICRAKDEFCPSQLQQDADFSSVINEVEPLEQILPDSLDEIKLVTHSENSKFRSKVWKDFIPFYVQGRVQGADCVHCHKRLTADKGRSHLNRHTQTCPARSGNILNHQKGVSFQSNLPSSKSSLQDELSPALTNGKIQIAEYASKFLKGSSSDASLVERHVLALPAMYDMNPSEKSTPSAQTAADRTRKTDQEASYLELTRMVISHGYPLSIVEHEEMRRFAKSLNPTFNMASSIDIEEYSTLLFQKEKADLKERIALLSRRVSLSASVWAPDGAEASVKYLCLAVHFIDSDWKLQRKTIKFGVFWSLPTSLERMIQFKEACVLDSDIGPFNVIQEALRDWNLDQKHFSLTSGSEIRNDEGTSKLMDLLIQRKCLPIRGELYNIDCVNDVLNNIVSKGQQVLCHVGNILETFIRAHMSSSLTRQQLLEAVAHMGLKCPHEDAKWWHIIYFRLEVLLHFKKAFPSEELLSAGDNKAVESVCRILRAFYHAVEVICSPICPTSNVYFNELWKVRTVLQEEASTDLIELANMVWEMQEAFNEYWQNSYLWLSIPVVLDPRFKITFIEFRLKRAFGTNAEKYVSAIRDTIRELFHEYCGLSNNLGGDTSTREVELDEFDSDSLEDWDEHLNAQTRNQQ

				LRELDNYLEDGIVPRKDDFDILNWWMSNSTKYPTLSIMARDVLAVPASAVNFEAALSGKVNVIHKQWSTLNIKTIEALVC TRDWVK
Zm 10	Zm 000 01d 023 717	16 70 99 98	16 71 00 01	MGDPNSNDSSMVHGTATETVFEGYEIFLGGNEMVTGCEAIISDKMGHAAETVLDYKMVDIHDNKMVHRNEIVPSGSKI DRNGQTVLRRCSQIVRGNEIGNDNVTTEVKPPTSSKRKRKTSMVWEHFTTEDSEGCTRACCNHCKRIFAYSSGLKMS GTSHLKRHITQGHCPEIEVQKPTAGGRENDCQGTVQKPSMRCRSTCTGYANAPFNPDRCQSYLAKMIILHGYPLQIVQ QAAFISFVESLQPSFKVINKDAVEAEVYAVYLKERKSLLKQVENIPGRINLTVQTWTTSQTLGYVSLAGQFIDSEWKLHR RMLNFMMVPWSCSEDDAVTEAISRSLHQWNMSDKLFTITRDYESSSHDIYSLNLREELSKNNITMLGGQFSVVRCYAH MLTAVAIDVTALVQSVIYKIRESIKFIKCRIGHEEMFADIILQLQIPSNQILCLDIKTQWNTTYLMLQAALDYKEAFTMLEKC DGNYSQAPSAVDWEKVEVACR
Zm 5	Zm 000 01d 013 336	88 65 28 8	88 65 47 3	MAEETANDAQVAQDNEIALSNEAIQGDELVHVEELSQGDDLVQGNELVVSEVTTPTIGTRRRRKKSLVWEHFTIEAVA GGATRACCKLCKQTFAYSSGSKIAGTSHLKRHITLGSCPKIKNQEQRLALPSTGGTDNDGEGTIERRTKRRYRYTGYA NAAFDQDRSCSYLAKLIIQHDYPLHIVQQPSFAIFIESLQPRFKIVDVETMEGEVYAVYQKEKENLLQAFNSMPGRISLTI GLWTTSQTLGYVSLAGQFIDSEWKVHRRMLNFMMVSSPHSENALSEAISMSLSDWNMKDRLFTITLDNECSSHDIYSA NLRDHLSNKNNLMLKGQLFVVRCYAHILNAVALDVIASIHGVVYSIRESIKFIKASSAREEKFAEIALQLEIPSTKTLCLDVT TQWNTTYLMLLAALDYKQTFTTLETCDDNYNEAPSAEDWKKVEAACNYLKLLYDSAHSIMAAANPTANIFFHEAWKLQ LELANGTGHEDPTFSSIAKDMHERFDKYWKDCSLVLAIAVVMDPRFKMKLVEFSYSKIYGAGAAKYVKVVDDAVHELY KEYVAQPLPLTPAYVDQGEGNNGPANANSSQGAPASTGDGLLDFDMYLSEIQSSQPSKSELEQYLDESLTPRIQEFDI LNWWKLNTVKFPTLSKMARDILAIPVSMVSSGSSIFCAGTGSHMLDDYRSSLRPEIVEALVCAKDWLQYSPRRLKDRR FVVVTACLVEAKQESIYPSVVSLSVVNCRCIVG
Zm 3	Zm 000 01d 039 328	19 58 21 8	19 65 69 4	MRERDACWEYCDKLDGNKVRCRFCHKVLNGGISRLKFHLSQIPSKGVNPCTKVKEDVVDRVKAVISAKEEYKEFQLLK RQRVADLSSAAAPAKWAPEAPPSLSTSPGRVASPAAVITRAAEQSRLLAPEASAPVPRPSAAAANNKPRAAAASEWE TERCIAEFFFENKLDYSIADSVSYQQMLEALGGPGFRGPPADVLRDKWLQRLKSEILQKTKEIEKDWATTGCTILADSW TDNKLKALINFSVASPMGTFFLKTVDASSHFKTHRGLYDLFDEVIQEVGPENVVQIIADRNINYGSTDKLVAQNYGGAIF WSPCASFCVNAMLDDFSKIDWVNQCICQAQTVTRFIYNSRWVLDLARKCVAGQELVCSGITKSVSDFLTLKSLLRHRP KLKQMFHSAEFSSSSYASRSIPCVEILDDDELWRAVEEIAAVSEPLLRVMRDVSGGRQAIGYIYESMTKVTDSIRTYYIM DEGKCKSFLDIVEQRWQTELHSPLHSAAAFLSPGIQYNPEVKFFRTIKEEFYQVLDKVLTTPDQRHDITAQLHAFRKAQ GLFGSNIAKEARNNTPPGMWWEQYGDSAPSLQRAAVRITSQVCSTLTFQRDWGVILQNHYEKRNKLDKEALADQAYV HYNLTLHSEPKARRRPDADPIALDAVDMTSAWVEDSDGPILTQWLDRFPSALDGGDLNTRQFGGSIFGTNDNLFGL
Zm S	Zm 000 01d 000 412	46 36 4	46 62 6	MEELVTRRRRGEPSSVNSASITQKISNAIKSDDPTWEHCFVPDMSKKQAIQCKYCDKVIHGGITRVKYHLANIGGFNVT KCKKVPAHVKQEMEAFLTKKTGEKEQRKKKKQRDRDEIDLDKSDGSNEEDNGHENDVIVLKSTRESNNCSSSRPPTN GGSGTIDNFYKPQSVEESDHKNKIQTKLSTHKREERRDRACEYICQFFYEAGIAHNTITLPSFGHMLEAIGAFGRGEWT VMYLPLVSSMDVCLRQRKKLLQDNKLKTPLHLDGYYLNPYFYYPNKSSIELDGSFRAAIISCITKMFDDEDTQDSIIEELS IYQDQQGAFGHDIAVRQRRNKTFNPDVVLDENVDQDEHEGAHDEPSPEPSISKAQLPAKRKRHGHPRKKKLRSLKS LLSCDLERATCASSSESEDNESMQIEASNSDSRDE

Table S22 List of AtZF-BED genes and their features

Gene_ID	Chromosome no.	Gene_ID	Start	End		Protein_sequence
AT1G18560.1	AT1	AT1G18560.1	6385449	6388080	ATZf-BED01_I	MEWNVNNAFKTYKEMEPKAMMDMTLVPHSDPIDIGLGSSDKSNSVPPKRKKTMTSVYLKYFETAPDSKTRKCKFCGQSYSIATATGNLGRHLTNRHPGYDKAAADVVTSSSVPQTPPAVVKPSQSQSKVPQLDYDHLNWLVLKWLALSSLPPSTVDETWLGNSFKFLKPSIQLWPAEKYKAILDEVFTSMRGDVKTTLEHIQSKVSVTLSFWNSYENIFYMSVTGQWIDENWSSHRLLLDICRIPYPSGGSEIYNSLLKVLKTYAIEDRILCCTHDNSENAIHACHSLKEYFDGQKVLPFCYIPCAAQTLNDIIDEGLATIKPIISKVREFTQELNASTELSDDFIQLTTAYQEGNWKLPIDASSRWSGNYQMVNILCKASKSLDSVIRKNEDALENRMMLSSVEKNAVTIVHNYLDLDSFHKTTNDMCTNKDLTVGLALLFMDNISEMITTCQKSCHNPDWLRTCAESMAQKARSYNTQVCNVFTYITAILDPRIKTEYIPETINLESYIDEARSHFIRNYSSSHFTSSMTSGYRPQEVDEGGGNISFAEEIARRKRRGSMSNNVVDELTQYLSESIVPMQTDVLDWWKVNSGRYPRLSNMARDFLAVQATSAAPEEIFCGKGEEIDKQKYCMPHDSTQSVICIRSWIEAGMKLKYKCSEIDYERLMELAATVAADNSAGGLEKIQQHR
AT1G36095.1	AT1	AT1G36095.1	13491370	13492725	ATZf-BED02_IV	MSDELSHKNLDPVKKYAQPVPLKHGSWRCNYCHKVTNGGVKGAKQHILGGFRNVTQCSLVPPIMREEIKDSMLKKTEIKATTQMMPPPATSYDDYGEEEEAAEVLGNERRQPPVKKQKGLMDMFVCPTLPNVLKVLKDANMLYDHLDLMVDEVGEANVVKVVIDNASNYVKASQLSMANRPHLYWTPCAAHCIYLMLEDIGKISEVKTVITQCIFKNDYIYGHTSLVNMMRKIHKRWKSAKIGCNTVCYVFHTIGQYHKQRKNLRNSATSQEWADSKWQKEIGARTVKRIIMQDSFWHKDG
AT1G79740.1	AT1	AT1G79740.1	30004367	30007020	ATZf-BED03_I	MVREKDICWEYAEKLDGNKVKCKFCSRVLNGGISRLKHHLSRLPSKGVNPCAKVRDDVTDRVRSILSAKDDPPITNKYKPPPPLSPPFDAPASKLVFPSSPPNAQDIAERSISLFFFENKIDFAVARSPSYHHMLDAVAKCGPGFVAPSPKTEWLDRVKSDISLQLKDTEKEWVTTGCTIIAEAWTDNKSRALINFSVSSPSRIFFHKSVDASSYFKNSKCLADLFDSVIQDIGQEHIVQIIMDNSFCYTGISNHLLQNYATIFVSPCASQCLNIILEEFSKVDWVNQCISQAQVISKFVYNNSPVLDLLRKLTGGQDIIRSGVTRSVSNFLSLQSMMKQKARLKHMFNCPEYTTNTNKPQSISCVNILEDNDFWRAVEESVAISEPILKVLREVSTGKPAVGSIYELMSKAKESIRTYYIMDENKHKVFSDIVDTNWCEHLHSPLHAAAAFLNPSIQYNPEIKFLTSLKEDFFKVLEKLLPTSDLRRDITNQIFTFTRAKGMFGCNLAMEARDSVSPGLWWEQFGDSAPVLQRVAIRILSQVCSGYNLERQWSTFQQMHWERRNKIDREILNKLAYVNQNLKLGRMITLETDPIALEDIDMMSEWVEEAENPSPAQWLDRFGTALDGGDLNTRQFGGAIFSANDHNIFGL
AT3G17450.1	AT3	AT3G17450.1	5972671	5976118	ATZf-BED04_III	MAPPGSIGVVDPGWEHGVAQDQRKKKVKCNYCGKIVSGGIYRLKQHLARVSGEVTYCDKSPEEVCMRMKENLVRSTKKLRQSEDNSGQSCSSFHQSNNDDEADEEERRCWSIRSKGKLGLSDGSLLRSSGYIDPGWEHGIAQDERKKKVKCNYCNKIVSGGINRFKQHLARIPGEVAPCKTAPEEVYVKIKENMKWHRAGKRQNRPDDEMGALTFRTVSQDPDQEEDREDHDFYPTSQDRLMLGNGRFSKDKRKSFDSTNMRSVSEAKTKRARMIPFQSPSSSKQRKLYSSCSNRVVSRKDVTSSISKFLHHVGVPTEAANSLYFQKMIELIGMYGEGFVVPSSQLFSGRLLQEEMSTIKSYLREYRSSWVVTGCSIMADTWTNTEGKKMISFLVSCPRGVYFHSSIDATDIVEDALSLFKCLDKLVDDIGEENVVQVITQNTAIFRSAGKLLEEKRKNLYWTPCAIHCTELVLEDFSKLEFVSECLEKAQRITRFIYNQTWLLNLMKNEFTQGLDLLRPAVMRHASGFTTLQSLMDHKASLRGLFQSDGWILSQTAAKSEEGREVEKMVLSAVFWKKVQYVLKSVDPVMQVIHMINDGGDRLSMPYAYGYMCCAKMAIKSIHSDDARKYGPFWRVIEYRWNPLFHHPLYVAAYFFNPAYKYRPDFMAQSEVVRGVNECIVRLEPDNTRRITALMQIPDYTCAKADFGTDIAIGTRTELDPSAWWQQHGISCLELQRVAVRILSHTCSSVGCEPKWSVYDQVNSQCQSQFGKKSTKDLTYVHYNLRLREKQLKQRLHYEDEPPPTLNHALLDRLLPDWLVTSEKEEEEALHGEDRAEKEDHEDDEEEEEKFYMESGNVDGEGEDNLDPYDDDLSDDD
AT3G48770.1	AT3	AT3G48770.1	18078474	18086817	ATZf-BED05_II	MEKAFSPNTSNIKQEKSSNRSSHHKQDLAWNYVREAIDSRRRKVITCGFCGKQYVGSEINRMKHHLAGLNNRSACQKVSSEVQHSMRKSLEENKEKPKKIIVVDLDMQDDEVNVGQSSHQSCSSSSRKRKVQHSMRKSLEETEEKLKQIIVVDLDMQDNEVNVGQSSLQSFSSSNRKRKRETKTKPVIIETMTESAKQHIERIRRTKFSIGGAENPLTEDLHQAVKNLSAELYAKDVHFLMELIQNAEDNEYPEGVDPSLEFVITSEDITNTGAPATLLIFNNEKGFSEKNIESICSVGRSTKKGNRKCGYIGEKGIGFKSVFLITSQPYIFSNGYQIRFNEAPCSHCSLGYIVPEWVDQHPSLVDIQRMYGSGSALPTTTIILPLKSDKVKPVKEQLSNVHPEVLLFLSKIKRLSIREHCLDPKLSTVNSIGIVSETNFVTRKSIDAESYTIHLSASEKGKNSEQECSYYMWRQKFPVKHENRVDRRSEVEEWVITLAFPFGERLGHGNNSPGIYAFLPTEMVTNFPFIIQADFILASSREMILLDDIWNQGILNCVPLAFLNAFTSLVKKTTDAPVSSLLPAFRFLPVKESNYAKLNVVRESIRARVCAEEIVPSISHQGQKFFYKPCEVGRLIPTFWDILEKAGSEGASLQNISSHGIYILNSAFDRTEYDNVLNFLGLKQVSNEWYVKCIQGCDLVTSVSEATYVEVLLFIAENWQCRFQNTNMGKVPLIKYVVQKGVSSLSSLGGFSPRTLCLSTEKNQAWLLDWNDEFRCMSNFVFMPPTTRTALKVCSKKEIIHTWLKENVKVITLSVSDYAKHLRENLNGDKRLVVAYAHFLHHSISKDFLSKEEAGKCCKDMPLVDNYGNVNISRNGVLVPASAGKWVSLVGSNPWRHSGYIELSEEYLLSNRFAGLRSNKKDLLGFLKSSVEAGDIPDIEPPN

						VAIPALSGPLTKENVLLLLEWINKCNRHSLRSNFLNSVRGGSWLRTTMNGVSDYRPPSQSFYHTSSWGSILQNGSILVDIPLSYYGNEIEKYKEELKIAGVMFEFSEVCRFVGNHLMSLAETSTQSSANVFSILKFIRYLREKRLSPADFITAVKNGPWLKTISGYRSPDGAVLFSEEWKAASLISDIPFIDRGFYGVVSLNGYKEELELLGVVVKFPDNYSLIVSHLNTAKLTYLTPDAMFLVLDCMRQLSPHRLINALWNSSQCFKTKKNGYKSPAECFIPDPEWTCLLSVFDCFPLIDDDFYGSRIFAYKGELKQIGVKLQLEEAVKMFVSTFKQKAISSGLTRCTASSLLSCYKKLMGSLYKYPEELMKSFKQFQWLHTKLGDFRAPKDCILFDSEWEPLRLIANLPFIDDGPNWYGKSIHEFRKELESLGVTVELRKGMSHVISSLSLPDPSRIAPSSALSLFRCIKFLREDRFQQLPKELLDKVSVKWLKTHAGYRSPEECLLFDRTWKLEPCDGPFIDEEYYGSDINSFREELIAIGVGHDSDKACQLLARNVYKLSETDAISRVYRFLSEAEWKPEKGASSGRIWIPSDEKWADISSCVLFDKDKLFGSKFNVLENHYCSGKDHNLLGFFSSAFGVRINPSIEDYCELWKYWEKTKNRLSSHECCAFWSFVVRHGDTVKAEKLLSESFSRLPVHSPDCNNNDGVMLSSISDVFIADDLLLKDMFIDSPVFVWYPTPSIPTLSRTRLIEIYRNIGVKEVSKCVEIAEADLTGFKTELQEVVDPKKNLIGPGLVKLILAFLSDPSLKVETAERLRIIHSLVDIDVKETSETITTEYTLSLPSKGEKLIAKAKRMIRWEREKGVVYAEKMEKTCGKRKLLEYATCFAEVIAKGVMWEREDLIGRLSELVKMAYLVEFDEEALEFLMKSKNLQVYEEDEKLISDEFSQVN
AT4G15020.1	AT4	AT4G15020.1	8575409	8578688	ATZf-BED06_I	MDAELEPVALTPQKQDNAWKHCEIYKYGDRLQMRCLYCRKMFKGGGITRVKEHLAGKKGQGTICDQVPEDVRLFLQQCIDGTVRRQRKRHKSSSEPLSVASLPPIEGDMMVVQPDVNDGFKSPGSSDVVVQNESLLSGRTKQRTYRSKKNAFENGSASNNVDLIGRDMDNLIPVAISSVKNIVHPSFRDRENTIHMAIGRFLFGIGADFDAVNSVNFQPMIDAIASGGFGVSAPTHDDLRGWILKNCVEEMAKEIDECKAMWKRTGCSILVEELNSDKGFKVLNFLVYCPEKVVFLKSVDASEVLSSADKLFELLSELVEEVGSTNVVQVITKCDDYYVDAGKRLMLVYPSLYWVPCAAHCIDQMLEEFGKLGWISETIEQAQAITRFVYNHSGVLNLMWKFTSGNDILLPAFSSSATNFATLGRIAELKSNLQAMVTSAEWNECSYSEEPSGLVMNALTDEAFWKAVALVNHLTSPLLRALRIVCSEKRPAMGYVYAALYRAKDAIKTHLVNREDYIIYWKIIDRWWEQQQHIPLLAAGFFLNPKLFYNTNEEIRSELILSVLDCIERLVPDDKIQDKIIKELTSYKTAGGVFGRNLAIRARDTMLPAEWWSTYGESCLNLSRFAIRILSQTCSSSVSCRRNQIPVEHIYQSKNSIEQKRLSDLVFVQYNMRLRQLGPGSGDDTLDPLSHNRIDVLKEWVSGDQACVEGNGSADWKSLESIHRNQVAPIIDDTEDLGSGFDDIEIFKVEKEVRDEGYYTNTSEKLFT

Table S23 Chromosomal Mapping of ZfBED genes on Ghi, Gr, Ga, Gba, Zm and At.

Chr	No. of genes
GhA11	3
GhA12	1
GhA13	2
GhA2	3
GhA3	1
GhA5	2
GhA6	2
GhA7	3
GhA8	2
GhD1	1
GhD11	3
GhD12	1
GhD13	2
GhD2	5
GhD3	1
GhD4	1
GhD5	1
GhD6	2
GhD8	3
GhD9	1

Chr	No. of genes
Gr1	3
Gr13	1
Gr4	1
Gr6	1
Gr7	2
Gr9	1

Chr	No. of genes
Ghe1	3
Ghe3	2
Ghe4	2
Ghe5	2
Ghe6	4
Ghe7	4
Ghe8	3
Ghe9	3
Ghe10	1

Ghe11	3
Ghe12	4
Ghe13	3
GheS	1

Chr	No. of genes
Ga1	2
Ga11	6
Ga12	3
Ga13	3
Ga3	7
Ga4	3
Ga6	3
Ga8	3
Ga9	2
GaS	2

Chr	No. of genes
GbA2	2
GbA3	1
GbA5	1
GbA6	2
GbA7	3
GbA8	2
GbA9	2
GbA11	2
GbA13	1
GbD11	2
GbD2	3
GbD3	2
GbD4	1
GbD5	1
GbD6	3
GbD7	1
GbD8	2
GbD9	1

Chr	No. of genes
Zm1	4
Zm2	4
Zm3	2

Zm4	2
Zm5	4
Zm7	1
Zm8	2
Zm9	1
Zm10	4
ZmS	1

**Chr**	**No. of genes**
AT1	3
AT3	2
AT4	1

Table S24 Cis-acting elements (top 100 hits) of Zf-BED encoding genes in *G. arboreum*.

Gene IDs	Cis-acting elements name	Cis-acting elements sequence	Posi tion	Lent ght	Stra nds	Reference species	Descriptions
Ga01G1 640.1	Unnamed__4	CTCC	37	4	+	*Petroselinum hortense*	
Ga01G1 640.1	Unnamed__4	CTCC	199	4	+	*Petroselinum hortense*	
Ga01G1 640.1	Unnamed__4	CTCC	611	4	+	*Petroselinum hortense*	
Ga01G1 640.1	Unnamed__4	CTCC	1188	4	-	*Petroselinum hortense*	
Ga01G1 640.1	Unnamed__4	CTCC	1265	4	-	*Petroselinum hortense*	
Ga01G1 640.1	Unnamed__4	CTCC	1587	4	+	*Petroselinum hortense*	
Ga01G1 640.1	Unnamed__4	CTCC	1646	4	-	*Petroselinum hortense*	
Ga01G1 640.1	MYB recognition site	CCGTTG	410	6	+	*Arabidopsis thaliana*	
Ga01G1 640.1	P-box	CCTTTTG	85	8257 3.94	-	*Oryza sativa*	gibberellin-responsive element
Ga01G1 640.1	Myb	TAACTG	739	6	+	*Arabidopsis thaliana*	
Ga01G1 640.1	TATA-box	TATA	1	4	+	*Arabidopsis thaliana*	core promoter element around -30 of transcription start
Ga01G1 640.1	TATA-box	TATAA	10	5	-	*Arabidopsis thaliana*	core promoter element around -30 of transcription start
Ga01G1 640.1	TATA-box	TATA	11	4	+	*Arabidopsis thaliana*	core promoter element around -30 of transcription start
Ga01G1 640.1	TATA-box	ATTATA	735	6	+	*Brassica napus*	core promoter element around -30 of transcription start
Ga01G1 640.1	TATA-box	TATAA	736	5	-	*Arabidopsis thaliana*	core promoter element around -30 of transcription start
Ga01G1 640.1	TATA-box	TATA	737	4	+	*Arabidopsis thaliana*	core promoter element around -30 of transcription start
Ga01G1 640.1	TATA-box	ATATAT	751	6	+	*Brassica napus*	core promoter element around -30 of transcription start
Ga01G1 640.1	TATA-box	TATATA	752	6	+	*Arabidopsis thaliana*	core promoter element around -30 of transcription start
Ga01G1 640.1	TATA-box	ATATAT	753	6	+	*Brassica napus*	core promoter element around -30 of transcription start
Ga01G1 640.1	TATA-box	TATATA	754	6	+	*Arabidopsis thaliana*	core promoter element around -30 of transcription start
Ga01G1 640.1	TATA-box	ATATAT	755	6	+	*Brassica napus*	core promoter element around -30 of transcription start
Ga01G1 640.1	TATA-box	TATATA	756	6	+	*Arabidopsis thaliana*	core promoter element around -30 of transcription start
Ga01G1 640.1	TATA-box	ATATAT	757	6	+	*Brassica napus*	core promoter element around -30 of transcription start
Ga01G1 640.1	TATA-box	TATATA	758	6	+	*Arabidopsis thaliana*	core promoter element around -30 of transcription start
Ga01G1 640.1	TATA-box	ATATAT	759	6	+	*Brassica napus*	core promoter element around -30 of transcription start
Ga01G1 640.1	TATA-box	TATATA	760	6	+	*Arabidopsis thaliana*	core promoter element around -30 of transcription start
Ga01G1 640.1	TATA-box	ATATAT	761	6	+	*Brassica napus*	core promoter element around -30 of transcription start
Ga01G1 640.1	TATA-box	TATATA	762	6	+	*Arabidopsis thaliana*	core promoter element around -30 of transcription start
Ga01G1 640.1	TATA-box	ATATAT	763	6	+	*Brassica napus*	core promoter element around -30 of transcription start
Ga01G1 640.1	TATA-box	TATATA	764	6	+	*Arabidopsis thaliana*	core promoter element around -30 of transcription start
Ga01G1 640.1	TATA-box	ATATAT	765	6	+	*Brassica napus*	core promoter element around -30 of transcription start

Ga01G1 640.1	TATA-box	TATATA	766	6	+	*Arabidopsis thaliana*	core promoter element around -30 of transcription start
Ga01G1 640.1	TATA-box	ATATAA	767	6	+	*Brassica oleracea*	core promoter element around -30 of transcription start
Ga01G1 640.1	TATA-box	TATA	768	4	+	*Arabidopsis thaliana*	core promoter element around -30 of transcription start
Ga01G1 640.1	TATA-box	TATATA	794	6	+	*Arabidopsis thaliana*	core promoter element around -30 of transcription start
Ga01G1 640.1	TATA-box	ATATAT	795	6	+	*Brassica napus*	core promoter element around -30 of transcription start
Ga01G1 640.1	TATA-box	TATA	796	4	+	*Arabidopsis thaliana*	core promoter element around -30 of transcription start
Ga01G1 640.1	TATA-box	TATA	826	4	+	*Arabidopsis thaliana*	core promoter element around -30 of transcription start
Ga01G1 640.1	TATA-box	ATATAA	843	6	+	*Brassica oleracea*	core promoter element around -30 of transcription start
Ga01G1 640.1	TATA-box	TATA	844	4	+	*Arabidopsis thaliana*	core promoter element around -30 of transcription start
Ga01G1 640.1	TATA-box	TATTTAAA	894	8	-	*Arabidopsis thaliana*	core promoter element around -30 of transcription start
Ga01G1 640.1	TATA-box	TATA	923	4	+	*Arabidopsis thaliana*	core promoter element around -30 of transcription start
Ga01G1 640.1	TATA-box	TACAAAA	960	7	-	*Oryza sativa*	core promoter element around -30 of transcription start
Ga01G1 640.1	TATA-box	TATACA	963	6	-	*Helianthus annuus*	core promoter element around -30 of transcription start
Ga01G1 640.1	TATA-box	TATA	965	4	+	*Arabidopsis thaliana*	core promoter element around -30 of transcription start
Ga01G1 640.1	TATA-box	TATAAAA	983	7	-	*Pisum sativum*	core promoter element around -30 of transcription start
Ga01G1 640.1	TATA-box	TATAAA	984	6	-	*Helianthus annuus*	core promoter element around -30 of transcription start
Ga01G1 640.1	TATA-box	TATAA	985	5	-	*Arabidopsis thaliana*	core promoter element around -30 of transcription start
Ga01G1 640.1	TATA-box	TATA	986	4	+	*Arabidopsis thaliana*	core promoter element around -30 of transcription start
Ga01G1 640.1	TATA-box	TATA	1091	4	-	*Arabidopsis thaliana*	core promoter element around -30 of transcription start
Ga01G1 640.1	TATA-box	ATATAA	1095	6	+	*Brassica oleracea*	core promoter element around -30 of transcription start
Ga01G1 640.1	TATA-box	TATA	1096	4	-	*Arabidopsis thaliana*	core promoter element around -30 of transcription start
Ga01G1 640.1	TATA-box	TATAAATA	1102	8	-	*Daucus carota*	core promoter element around -30 of transcription start
Ga01G1 640.1	TATA-box	TATAAAT	1103	7	-	*Brassica juncea*	core promoter element around -30 of transcription start
Ga01G1 640.1	TATA-box	TATAAA	1104	6	-	*Helianthus annuus*	core promoter element around -30 of transcription start
Ga01G1 640.1	TATA-box	TATAA	1105	5	-	*Arabidopsis thaliana*	core promoter element around -30 of transcription start
Ga01G1 640.1	TATA-box	TATA	1106	4	-	*Arabidopsis thaliana*	core promoter element around -30 of transcription start
Ga01G1 640.1	TATA-box	ATATAA	1133	6	+	*Brassica oleracea*	core promoter element around -30 of transcription start
Ga01G1 640.1	TATA-box	TATA	1134	4	-	*Arabidopsis thaliana*	core promoter element around -30 of transcription start
Ga01G1 640.1	TATA-box	ATTATA	1213	6	+	*Brassica napus*	core promoter element around -30 of transcription start
Ga01G1 640.1	TATA-box	TATATAA	1214	7	-	*Arabidopsis thaliana*	core promoter element around -30 of transcription start
Ga01G1 640.1	TATA-box	TATATA	1215	6	-	*Arabidopsis thaliana*	core promoter element around -30 of transcription start
Ga01G1 640.1	TATA-box	TATA	1217	4	-	*Arabidopsis thaliana*	core promoter element around -30 of transcription start
Ga01G1 640.1	TATA-box	TATA	1250	4	-	*Arabidopsis thaliana*	core promoter element around -30 of transcription start
Ga01G1 640.1	TATA-box	TATA	1255	4	-	*Arabidopsis thaliana*	core promoter element around -30 of transcription start

Ga01G1640.1	TATA-box	TATAAAA	1333	7	-	*Pisum sativum*	core promoter element around -30 of transcription start
Ga01G1640.1	TATA-box	TATAAA	1334	6	-	*Helianthus annuus*	core promoter element around -30 of transcription start
Ga01G1640.1	TATA-box	TATAA	1335	5	-	*Arabidopsis thaliana*	core promoter element around -30 of transcription start
Ga01G1640.1	TATA-box	TATA	1336	4	-	*Arabidopsis thaliana*	core promoter element around -30 of transcription start
Ga01G1640.1	TATA-box	taTATAAAtc	1363	9	-	*Arabidopsis thaliana*	core promoter element around -30 of transcription start
Ga01G1640.1	TATA-box	TATAAAT	1364	7	-	*Brassica juncea*	core promoter element around -30 of transcription start
Ga01G1640.1	TATA-box	TATAAA	1365	6	-	*Helianthus annuus*	core promoter element around -30 of transcription start
Ga01G1640.1	TATA-box	TATATAA	1366	7	-	*Arabidopsis thaliana*	core promoter element around -30 of transcription start
Ga01G1640.1	TATA-box	TATATA	1367	6	-	*Arabidopsis thaliana*	core promoter element around -30 of transcription start
Ga01G1640.1	TATA-box	ATATAT	1368	6	-	*Brassica napus*	core promoter element around -30 of transcription start
Ga01G1640.1	TATA-box	TATA	1369	4	-	*Arabidopsis thaliana*	core promoter element around -30 of transcription start
Ga01G1640.1	TATA-box	TATATTTATATTT	1371	12	+	*Avena sativa*	core promoter element around -30 of transcription start
Ga01G1640.1	TATA-box	TATAAAA	1374	7	-	*Pisum sativum*	core promoter element around -30 of transcription start
Ga01G1640.1	TATA-box	TATAAA	1375	6	-	*Helianthus annuus*	core promoter element around -30 of transcription start
Ga01G1640.1	TATA-box	TATAA	1376	5	-	*Arabidopsis thaliana*	core promoter element around -30 of transcription start
Ga01G1640.1	TATA-box	TATA	1377	4	-	*Arabidopsis thaliana*	core promoter element around -30 of transcription start
Ga01G1640.1	TATA-box	ATATAA	1410	6	+	*Brassica oleracea*	core promoter element around -30 of transcription start
Ga01G1640.1	TATA-box	TATA	1411	4	-	*Arabidopsis thaliana*	core promoter element around -30 of transcription start
Ga01G1640.1	TATA-box	TATAAAA	1497	7	-	*Pisum sativum*	core promoter element around -30 of transcription start
Ga01G1640.1	TATA-box	TATAAA	1498	6	-	*Helianthus annuus*	core promoter element around -30 of transcription start
Ga01G1640.1	TATA-box	TATAA	1499	5	-	*Arabidopsis thaliana*	core promoter element around -30 of transcription start
Ga01G1640.1	TATA-box	TATA	1500	4	-	*Arabidopsis thaliana*	core promoter element around -30 of transcription start
Ga01G1640.1	TATA-box	ATATAT	1505	6	-	*Brassica napus*	core promoter element around -30 of transcription start
Ga01G1640.1	TATA-box	TATA	1506	4	-	*Arabidopsis thaliana*	core promoter element around -30 of transcription start
Ga01G1640.1	TATA-box	TATA	1538	4	-	*Arabidopsis thaliana*	core promoter element around -30 of transcription start
Ga01G1640.1	TATA-box	TATTTAAA	1552	8	+	*Arabidopsis thaliana*	core promoter element around -30 of transcription start
Ga01G1640.1	TATA-box	TATA	1619	4	-	*Arabidopsis thaliana*	core promoter element around -30 of transcription start
Ga01G1640.1	TATA-box	ATTATA	1692	6	+	*Brassica napus*	core promoter element around -30 of transcription start
Ga01G1640.1	TATA-box	TATAA	1693	5	-	*Arabidopsis thaliana*	core promoter element around -30 of transcription start
Ga01G1640.1	TATA-box	TATA	1694	4	-	*Arabidopsis thaliana*	core promoter element around -30 of transcription start
Ga01G1640.1	TATA-box	TATAAA	1730	6	-	*Helianthus annuus*	core promoter element around -30 of transcription start
Ga01G1640.1	TATA-box	TATAA	1731	5	-	*Arabidopsis thaliana*	core promoter element around -30 of transcription start
Ga01G1640.1	TATA-box	TATA	1732	4	-	*Arabidopsis thaliana*	core promoter element around -30 of transcription start

**Identified Cis-Acting Motif**	**Functions**
3-AF1 binding site	light responsive element
3-AF3 binding site	part of a conserved DNA module array (CMA3)
4cl-CMA1b	light responsive element
AAAC-motif	light responsive element
AACA_motif	involved in endosperm-specific negative expression
A-box	cis-acting regulatory element
ABRE	cis-acting element involved in the abscisic acid responsiveness
ACA-motif	part of gapA in (gapA-CMA1) involved with light responsiveness
ACE	cis-acting element involved in light responsiveness
AE-box	part of a module for light response
ARE	cis-acting regulatory element essential for the anaerobic induction
AT1-motif	part of a light responsive module
ATC-motif	part of a conserved DNA module involved in light responsiveness
ATCT-motif	part of a conserved DNA module involved in light responsiveness
AT-rich element	binding site of AT-rich DNA binding protein (ATBP-1)
AT-rich sequence	element for maximal elicitor-mediated activation (2copies)
AuxRE	part of an auxin-responsive element
AuxRR-core	cis-acting regulatory element involved in auxin responsiveness
Box 4	part of a conserved DNA module involved in light responsiveness
Box II	part of a light responsive element
Box III	protein binding site
CAAT-box	common cis-acting element in promoter and enhancer regions
CAG-motif	part of a light response element
CAT-box	cis-acting regulatory element related to meristem expression
CCAAT-box	MYBHv1 binding site
CGTCA-motif	cis-acting regulatory element involved in the MeJA-responsiveness
chs-CMA1a	part of a light responsive element
chs-CMA2a	part of a light responsive element
chs-CMA2b	part of a light responsive element
chs-Unit 1 m1	part of a light responsive element
Circadian	cis-acting regulatory element involved in circadian control
GA-motif	part of a light responsive element
Gap-box	part of a light responsive element
GARE-motif	gibberellin-responsive element
GATA-motif	part of a light responsive element
G-box	cis-acting regulatory element involved in light responsiveness
GC-motif	enhancer-like element involved in anoxic specific inducibility
GCN4_motif	cis-regulatory element involved in endosperm expression
GT1-motif	light responsive element
GTGGC-motif	part of a light responsive element
HD-Zip 1	element involved in differentiation of the palisade mesophyll cells
HD-Zip 3	protein binding site
I-box	part of a light responsive element
LAMP-element	part of a light responsive element

L-box	part of a light responsive element
LS7	part of a light responsive element
LTR	cis-acting element involved in low-temperature responsiveness
MBS	MYB binding site involved in drought-inducibility
MBSI	MYB binding site involved in flavonoid biosynthetic genes regulation
motif I	cis-acting regulatory element root specific
MRE	MYB binding site involved in light responsiveness
MSA-like	cis-acting element involved in cell cycle regulation
NON-box	cis-acting regulatory element related to meristem specific activation
O2-site	cis-acting regulatory element involved in zein metabolism regulation
P-box	gibberellin-responsive element
RY-element	cis-acting regulatory element involved in seed-specific regulation
SARE	cis-acting element involved in salicylic acid responsiveness
Sp1	light responsive element
TATA-box	core promoter element around -30 of transcription start
TATC-box	cis-acting element involved in gibberellin-responsiveness
TCA-element	cis-acting element involved in salicylic acid responsiveness
TCCC-motif	part of a light responsive element
TC-rich repeats	cis-acting element involved in defense and stress responsiveness
TCT-motif	part of a light responsive element
TGA-box	part of an auxin-responsive element
TGACG-motif	cis-acting regulatory element involved in the MeJA-responsiveness
TGA-element	auxin-responsive element
WUN-motif	wound-responsive element

Table S25 Cis-acting elements (top 100 hits) of Zf-BED encoding genes in *G. barbadence*.

Gene IDs	Cis-acting elements name	Cis-acting elements sequence	Posi tion	Len tght	Stra nds	Reference species	Descriptions
Gbar_A02 G000610.1	ABRE4	CACGTA	998	6	-	*Zea mays*	
Gbar_A02 G000610.1	W box	TTGACC	615	6	-	*Arabidopsis thaliana*	
Gbar_A02 G000610.1	as-1	TGACG	198 0	5	-	*Arabidopsis thaliana*	
Gbar_A02 G000610.1	MYB-like sequence	TAACCA	980	6	+	*Arabidopsis thaliana*	
Gbar_A02 G000610.1	AT1-motif	AATTATTTTTTA TT	184 7	13	+	*Solanum tuberosum*	part of a light responsive module
Gbar_A02 G000610.1	AuxRR-core	GGTCCAT	373	7	+	*Nicotiana tabacum*	cis-acting regulatory element involved in auxin responsiveness
Gbar_A02 G000610.1	CAAT-box	CAAT	43	4	-	*Nicotiana glutinosa*	
Gbar_A02 G000610.1	CAAT-box	CAAAT	264	5	+	*Pisum sativum*	common cis-acting element in promoter and enhancer regions
Gbar_A02 G000610.1	CAAT-box	CAAT	273	4	+	*Nicotiana glutinosa*	
Gbar_A02 G000610.1	CAAT-box	CAAT	325	4	-	*Nicotiana glutinosa*	
Gbar_A02 G000610.1	CAAT-box	CAAAT	352	5	+	*Pisum sativum*	common cis-acting element in promoter and enhancer regions
Gbar_A02 G000610.1	CAAT-box	CAAT	465	4	-	*Nicotiana glutinosa*	
Gbar_A02 G000610.1	CAAT-box	CAAT	505	4	+	*Nicotiana glutinosa*	
Gbar_A02 G000610.1	CAAT-box	CAAT	574	4	+	*Nicotiana glutinosa*	
Gbar_A02 G000610.1	CAAT-box	CAAT	592	4	+	*Nicotiana glutinosa*	
Gbar_A02 G000610.1	CAAT-box	CCAAT	643	5	-	*Arabidopsis thaliana*	common cis-acting element in promoter and enhancer regions
Gbar_A02 G000610.1	CAAT-box	CCAAT	655	5	-	*Arabidopsis thaliana*	common cis-acting element in promoter and enhancer regions
Gbar_A02 G000610.1	CAAT-box	CAAAT	783	5	+	*Pisum sativum*	common cis-acting element in promoter and enhancer regions
Gbar_A02 G000610.1	CAAT-box	CAAT	815	4	-	*Nicotiana glutinosa*	
Gbar_A02 G000610.1	CAAT-box	CAAAT	839	5	-	*Pisum sativum*	common cis-acting element in promoter and enhancer regions
Gbar_A02 G000610.1	CAAT-box	CAAT	851	4	+	*Nicotiana glutinosa*	
Gbar_A02 G000610.1	CAAT-box	CAAAT	943	5	+	*Pisum sativum*	common cis-acting element in promoter and enhancer regions
Gbar_A02 G000610.1	CAAT-box	CAAT	104 5	4	-	*Nicotiana glutinosa*	
Gbar_A02 G000610.1	CAAT-box	CAAAT	107 9	5	+	*Pisum sativum*	common cis-acting element in promoter and enhancer regions
Gbar_A02 G000610.1	CAAT-box	CAAT	108 6	4	+	*Nicotiana glutinosa*	
Gbar_A02 G000610.1	CAAT-box	CAAAT	114 5	5	+	*Pisum sativum*	common cis-acting element in promoter and enhancer regions
Gbar_A02 G000610.1	CAAT-box	CAAT	114 8	4	-	*Nicotiana glutinosa*	
Gbar_A02 G000610.1	CAAT-box	CAAT	116 7	4	-	*Nicotiana glutinosa*	
Gbar_A02 G000610.1	CAAT-box	CAAT	120 2	4	+	*Nicotiana glutinosa*	
Gbar_A02 G000610.1	CAAT-box	CCAAT	123 8	5	+	*Arabidopsis thaliana*	common cis-acting element in promoter and enhancer regions
Gbar_A02 G000610.1	CAAT-box	CAAT	123 9	4	+	*Nicotiana glutinosa*	

Gbar_A02 G000610.1	CAAT-box	CAAT	1263	4	-	*Nicotiana glutinosa*	
Gbar_A02 G000610.1	CAAT-box	CAAT	1364	4	+	*Nicotiana glutinosa*	
Gbar_A02 G000610.1	CAAT-box	CAAT	1371	4	-	*Nicotiana glutinosa*	
Gbar_A02 G000610.1	CAAT-box	CAAT	1412	4	-	*Nicotiana glutinosa*	
Gbar_A02 G000610.1	CAAT-box	CAAT	1443	4	+	*Nicotiana glutinosa*	
Gbar_A02 G000610.1	CAAT-box	CAAT	1475	4	-	*Nicotiana glutinosa*	
Gbar_A02 G000610.1	CAAT-box	CAAT	1491	4	+	*Nicotiana glutinosa*	
Gbar_A02 G000610.1	CAAT-box	CAAT	1553	4	-	*Nicotiana glutinosa*	
Gbar_A02 G000610.1	CAAT-box	CAAT	1686	4	-	*Nicotiana glutinosa*	
Gbar_A02 G000610.1	CAAT-box	CAAT	1750	4	+	*Nicotiana glutinosa*	
Gbar_A02 G000610.1	CAAT-box	CAAT	1757	4	-	*Nicotiana glutinosa*	
Gbar_A02 G000610.1	CAAT-box	CAAT	1821	4	-	*Nicotiana glutinosa*	
Gbar_A02 G000610.1	CAAT-box	CAAT	1839	4	+	*Nicotiana glutinosa*	
Gbar_A02 G000610.1	CAAT-box	CAAAT	1841	5	-	*Pisum sativum*	common cis-acting element in promoter and enhancer regions
Gbar_A02 G000610.1	CAAT-box	CAAAT	1863	5	+	*Pisum sativum*	common cis-acting element in promoter and enhancer regions
Gbar_A02 G000610.1	CAAT-box	CAAAT	1904	5	-	*Pisum sativum*	common cis-acting element in promoter and enhancer regions
Gbar_A02 G000610.1	CAAT-box	CAAT	1987	4	-	*Nicotiana glutinosa*	
Gbar_A02 G000610.1	ATC-motif	AGTAATCT	1344	8	-	*Spinacia oleracea*	part of a conserved DNA module involved in light responsiveness
Gbar_A02 G000610.1	MYC	CATGTG	317	6	+	*Arabidopsis thaliana*	
Gbar_A02 G000610.1	MYC	CATGTG	801	6	-	*Arabidopsis thaliana*	
Gbar_A02 G000610.1	MYC	CATTTG	1079	6	-	*Arabidopsis thaliana*	
Gbar_A02 G000610.1	ATCT-motif	AATCTAATCC	743	9	+	*Pisum sativum*	part of a conserved DNA module involved in light responsiveness
Gbar_A02 G000610.1	Unnamed__4	CTCC	723	4	+	*Petroselinum hortense*	
Gbar_A02 G000610.1	CGTCA-motif	CGTCA	1980	5	+	*Hordeum vulgare*	cis-acting regulatory element involved in the MeJA-responsiveness
Gbar_A02 G000610.1	TCT-motif	TCTTAC	424	6	-	*Arabidopsis thaliana*	part of a light responsive element
Gbar_A02 G000610.1	TATA	TATAAAAT	1091	8	-	*Arabidopsis thaliana*	
Gbar_A02 G000610.1	TATA	TATAAAAT	1257	8	+	*Arabidopsis thaliana*	
Gbar_A02 G000610.1	TATA	TATAAAAT	1502	8	-	*Arabidopsis thaliana*	
Gbar_A02 G000610.1	TATA	TATAAAAT	1643	8	+	*Arabidopsis thaliana*	
Gbar_A02 G000610.1	G-box	tgACACGTGGCA	314	11	+	*Lycopersicon esculentum*	cis-acting regulatory element involved in light responsiveness
Gbar_A02 G000610.1	G-box	TACGTG	998	6	+	*Arabidopsis thaliana*	cis-acting regulatory element involved in light responsiveness
Gbar_A02 G000610.1	G-box	CACGTG	1004	6	-	*Arabidopsis thaliana*	cis-acting regulatory element involved in light responsiveness
Gbar_A02 G000610.1	MBS	CAACTG	1433	6	-	*Arabidopsis thaliana*	MYB binding site involved in drought-inducibility
Gbar_A02 G000610.1	ABRE3a	TACGTG	998	6	+	*Zea mays*	

Gbar_A02 G000610.1	MYB	CAACCA	676	6	+	*Arabidopsis thaliana*	
Gbar_A02 G000610.1	MYB	TAACCA	980	6	+	*Arabidopsis thaliana*	
Gbar_A02 G000610.1	TATA-box	TATAA	51	5	-	*Arabidopsis thaliana*	core promoter element around -30 of transcription start
Gbar_A02 G000610.1	TATA-box	TATA	52	4	+	*Arabidopsis thaliana*	core promoter element around -30 of transcription start
Gbar_A02 G000610.1	TATA-box	TATATA	186	6	+	*Arabidopsis thaliana*	core promoter element around -30 of transcription start
Gbar_A02 G000610.1	TATA-box	ATATAT	187	6	+	*Brassica napus*	core promoter element around -30 of transcription start
Gbar_A02 G000610.1	TATA-box	TATATA	188	6	+	*Arabidopsis thaliana*	core promoter element around -30 of transcription start
Gbar_A02 G000610.1	TATA-box	ATATAT	189	6	+	*Brassica napus*	core promoter element around -30 of transcription start
Gbar_A02 G000610.1	TATA-box	TATA	190	4	+	*Arabidopsis thaliana*	core promoter element around -30 of transcription start
Gbar_A02 G000610.1	TATA-box	TACATAAA	250	8	+	*Oryza sativa*	core promoter element around -30 of transcription start
Gbar_A02 G000610.1	TATA-box	TATA	259	4	+	*Arabidopsis thaliana*	core promoter element around -30 of transcription start
Gbar_A02 G000610.1	TATA-box	ATATAT	280	6	+	*Brassica napus*	core promoter element around -30 of transcription start
Gbar_A02 G000610.1	TATA-box	TATA	281	4	+	*Arabidopsis thaliana*	core promoter element around -30 of transcription start
Gbar_A02 G000610.1	TATA-box	TATTTAAA	291	8	-	*Arabidopsis thaliana*	core promoter element around -30 of transcription start
Gbar_A02 G000610.1	TATA-box	TATACA	327	6	-	*Helianthus annuus*	core promoter element around -30 of transcription start
Gbar_A02 G000610.1	TATA-box	TATA	329	4	+	*Arabidopsis thaliana*	core promoter element around -30 of transcription start
Gbar_A02 G000610.1	TATA-box	TATA	436	4	+	*Arabidopsis thaliana*	core promoter element around -30 of transcription start
Gbar_A02 G000610.1	TATA-box	TATATTTATATT T	528	12	-	*Avena sativa*	core promoter element around -30 of transcription start
Gbar_A02 G000610.1	TATA-box	ATATAT	536	6	+	*Brassica napus*	core promoter element around -30 of transcription start
Gbar_A02 G000610.1	TATA-box	TATATA	537	6	+	*Arabidopsis thaliana*	core promoter element around -30 of transcription start
Gbar_A02 G000610.1	TATA-box	ATATAT	538	6	+	*Brassica napus*	core promoter element around -30 of transcription start
Gbar_A02 G000610.1	TATA-box	TATA	539	4	+	*Arabidopsis thaliana*	core promoter element around -30 of transcription start
Gbar_A02 G000610.1	TATA-box	TATAAA	694	6	-	*Helianthus annuus*	core promoter element around -30 of transcription start
Gbar_A02 G000610.1	TATA-box	TATAA	695	5	-	*Arabidopsis thaliana*	core promoter element around -30 of transcription start
Gbar_A02 G000610.1	TATA-box	TATA	696	4	+	*Arabidopsis thaliana*	core promoter element around -30 of transcription start
Gbar_A02 G000610.1	TATA-box	ATTATA	786	6	+	*Brassica napus*	core promoter element around -30 of transcription start
Gbar_A02 G000610.1	TATA-box	TATATAA	787	7	-	*Arabidopsis thaliana*	core promoter element around -30 of transcription start
Gbar_A02 G000610.1	TATA-box	TATATA	788	6	+	*Arabidopsis thaliana*	core promoter element around -30 of transcription start
Gbar_A02 G000610.1	TATA-box	ATATAA	789	6	+	*Brassica oleracea*	core promoter element around -30 of transcription start
Gbar_A02 G000610.1	TATA-box	TATA	790	4	+	*Arabidopsis thaliana*	core promoter element around -30 of transcription start
Gbar_A02 G000610.1	TATA-box	ATATAA	794	6	+	*Brassica oleracea*	core promoter element around -30 of transcription start
Gbar_A02 G000610.1	TATA-box	TATA	795	4	+	*Arabidopsis thaliana*	core promoter element around -30 of transcription start
Gbar_A02 G000610.1	TATA-box	TACAAAA	860	7	+	*Oryza sativa*	core promoter element around -30 of transcription start

Table S26 Cis-acting elements of Zf-BED encoding genes in *G. hirsutum*.

Gene IDs	Cis-acting elements name	Cis-acting elements sequence	Posi tion	Len tght	Stra nds	Reference species	Descriptions
Ghir_A02G 001270.1	MYB	CAACCA	129 3	6	+	*Arabidopsis thaliana*	
Ghir_A02G 001270.1	MYB	CAACCA	148 8	6	+	*Arabidopsis thaliana*	
Ghir_A02G 001270.1	MYB	CAACAG	176 5	6	+	*Arabidopsis thaliana*	
Ghir_A02G 001270.1	AAGAA-motif	GAAAGAA	121 1	7	-	*Avena sativa*	
Ghir_A02G 001270.1	AAGAA-motif	GAAAGAA	141 0	7	-	*Avena sativa*	
Ghir_A02G 001270.1	as-1	TGACG	414	5	-	*Arabidopsis thaliana*	
Ghir_A02G 001270.1	CAAT-box	CAAAT	82	5	+	*Pisum sativum*	common cis-acting element in promoter and enhancer regions
Ghir_A02G 001270.1	CAAT-box	CAAT	85	4	-	*Nicotiana glutinosa*	
Ghir_A02G 001270.1	CAAT-box	CAAT	108	4	+	*Nicotiana glutinosa*	
Ghir_A02G 001270.1	CAAT-box	CAAT	152	4	-	*Nicotiana glutinosa*	
Ghir_A02G 001270.1	CAAT-box	CAAAT	203	5	-	*Pisum sativum*	common cis-acting element in promoter and enhancer regions
Ghir_A02G 001270.1	CAAT-box	CAAAT	242	5	-	*Pisum sativum*	common cis-acting element in promoter and enhancer regions
Ghir_A02G 001270.1	CAAT-box	CAAAT	253	5	-	*Pisum sativum*	common cis-acting element in promoter and enhancer regions
Ghir_A02G 001270.1	CAAT-box	CAAAT	263	5	-	*Pisum sativum*	common cis-acting element in promoter and enhancer regions
Ghir_A02G 001270.1	CAAT-box	CAAAT	329	5	-	*Pisum sativum*	common cis-acting element in promoter and enhancer regions
Ghir_A02G 001270.1	CAAT-box	CAAT	357	4	+	*Nicotiana glutinosa*	
Ghir_A02G 001270.1	CAAT-box	CAAAT	496	5	-	*Pisum sativum*	common cis-acting element in promoter and enhancer regions
Ghir_A02G 001270.1	CAAT-box	CAAAT	528	5	-	*Pisum sativum*	common cis-acting element in promoter and enhancer regions
Ghir_A02G 001270.1	CAAT-box	CAAT	559	4	+	*Nicotiana glutinosa*	
Ghir_A02G 001270.1	CAAT-box	CAAAT	588	5	-	*Pisum sativum*	common cis-acting element in promoter and enhancer regions
Ghir_A02G 001270.1	CAAT-box	CAAAT	671	5	-	*Pisum sativum*	common cis-acting element in promoter and enhancer regions
Ghir_A02G 001270.1	CAAT-box	CAAT	703	4	+	*Nicotiana glutinosa*	
Ghir_A02G 001270.1	CAAT-box	CAAT	724	4	-	*Nicotiana glutinosa*	
Ghir_A02G 001270.1	CAAT-box	CAAT	794	4	-	*Nicotiana glutinosa*	
Ghir_A02G 001270.1	CAAT-box	CAAAT	111 3	5	+	*Pisum sativum*	common cis-acting element in promoter and enhancer regions
Ghir_A02G 001270.1	CAAT-box	CAAAT	112 1	5	+	*Pisum sativum*	common cis-acting element in promoter and enhancer regions
Ghir_A02G 001270.1	CAAT-box	CAAT	113 6	4	-	*Nicotiana glutinosa*	
Ghir_A02G 001270.1	CAAT-box	CAAT	116 3	4	-	*Nicotiana glutinosa*	
Ghir_A02G 001270.1	CAAT-box	CAAT	119 6	4	+	*Nicotiana glutinosa*	
Ghir_A02G 001270.1	CAAT-box	CAAT	131 4	4	-	*Nicotiana glutinosa*	
Ghir_A02G 001270.1	CAAT-box	CAAT	136 1	4	-	*Nicotiana glutinosa*	

Ghir_A02G 001270.1	CAAT-box	CAAT	139 5	4	+	*Nicotiana glutinosa*	
Ghir_A02G 001270.1	CAAT-box	CAAT	139 7	4	-	*Nicotiana glutinosa*	
Ghir_A02G 001270.1	CAAT-box	CAAT	151 3	4	-	*Nicotiana glutinosa*	
Ghir_A02G 001270.1	CAAT-box	CAAT	156 3	4	+	*Nicotiana glutinosa*	
Ghir_A02G 001270.1	CAAT-box	CAAT	174 4	4	-	*Nicotiana glutinosa*	
Ghir_A02G 001270.1	CAAT-box	CAAT	177 8	4	+	*Nicotiana glutinosa*	
Ghir_A02G 001270.1	CAAT-box	CCAAT	179 0	5	-	*Arabidopsis thaliana*	common cis-acting element in promoter and enhancer regions
Ghir_A02G 001270.1	CAAT-box	CAAT	184 3	4	+	*Nicotiana glutinosa*	
Ghir_A02G 001270.1	CAAT-box	CAAT	184 9	4	+	*Nicotiana glutinosa*	
Ghir_A02G 001270.1	CAAT-box	CAAAT	188 4	5	-	*Pisum sativum*	common cis-acting element in promoter and enhancer regions
Ghir_A02G 001270.1	chs-CMA2c	ATGTACGTGGA GG	104 2	12	-	*Petroselinu m crispum*	part of a light responsive element
Ghir_A02G 001270.1	TATA-box	ATATAT	27	6	+	*Brassica napus*	core promoter element around -30 of transcription start
Ghir_A02G 001270.1	TATA-box	TATATA	28	6	+	*Arabidopsis thaliana*	core promoter element around -30 of transcription start
Ghir_A02G 001270.1	TATA-box	ATATAT	29	6	+	*Brassica napus*	core promoter element around -30 of transcription start
Ghir_A02G 001270.1	TATA-box	TATATA	30	6	+	*Arabidopsis thaliana*	core promoter element around -30 of transcription start
Ghir_A02G 001270.1	TATA-box	TATA	32	4	+	*Arabidopsis thaliana*	core promoter element around -30 of transcription start
Ghir_A02G 001270.1	TATA-box	TACAAAA	50	7	-	*Oryza sativa*	core promoter element around -30 of transcription start
Ghir_A02G 001270.1	TATA-box	TATAA	76	5	-	*Arabidopsis thaliana*	core promoter element around -30 of transcription start
Ghir_A02G 001270.1	TATA-box	TATA	77	4	+	*Arabidopsis thaliana*	core promoter element around -30 of transcription start
Ghir_A02G 001270.1	TATA-box	TATA	276	4	+	*Arabidopsis thaliana*	core promoter element around -30 of transcription start
Ghir_A02G 001270.1	TATA-box	ATATAT	295	6	+	*Brassica napus*	core promoter element around -30 of transcription start
Ghir_A02G 001270.1	TATA-box	TATA	296	4	+	*Arabidopsis thaliana*	core promoter element around -30 of transcription start
Ghir_A02G 001270.1	TATA-box	taTATAAAtc	337	9	+	*Arabidopsis thaliana*	core promoter element around -30 of transcription start
Ghir_A02G 001270.1	TATA-box	ATATAA	338	6	+	*Brassica oleracea*	core promoter element around -30 of transcription start
Ghir_A02G 001270.1	TATA-box	TATA	339	4	+	*Arabidopsis thaliana*	core promoter element around -30 of transcription start
Ghir_A02G 001270.1	TATA-box	TATAA	534	5	-	*Arabidopsis thaliana*	core promoter element around -30 of transcription start
Ghir_A02G 001270.1	TATA-box	TATA	535	4	+	*Arabidopsis thaliana*	core promoter element around -30 of transcription start
Ghir_A02G 001270.1	TATA-box	TATA	543	4	+	*Arabidopsis thaliana*	core promoter element around -30 of transcription start
Ghir_A02G 001270.1	TATA-box	TATACA	683	6	-	*Helianthus annuus*	core promoter element around -30 of transcription start
Ghir_A02G 001270.1	TATA-box	TATATA	685	6	+	*Arabidopsis thaliana*	core promoter element around -30 of transcription start
Ghir_A02G 001270.1	TATA-box	ATATAT	686	6	+	*Brassica napus*	core promoter element around -30 of transcription start
Ghir_A02G 001270.1	TATA-box	TATA	687	4	+	*Arabidopsis thaliana*	core promoter element around -30 of transcription start
Ghir_A02G 001270.1	TATA-box	TATA	790	4	+	*Arabidopsis thaliana*	core promoter element around -30 of transcription start
Ghir_A02G 001270.1	TATA-box	TATATA	105 6	6	-	*Arabidopsis thaliana*	core promoter element around -30 of transcription start

Ghir_A02G 001270.1	TATA-box	ATATAA	1057	6	+	*Brassica oleracea*	core promoter element around -30 of transcription start
Ghir_A02G 001270.1	TATA-box	TATA	1058	4	-	*Arabidopsis thaliana*	core promoter element around -30 of transcription start
Ghir_A02G 001270.1	TATA-box	ATATAT	1124	6	-	*Brassica napus*	core promoter element around -30 of transcription start
Ghir_A02G 001270.1	TATA-box	TATA	1125	4	-	*Arabidopsis thaliana*	core promoter element around -30 of transcription start
Ghir_A02G 001270.1	TATA-box	ATTATA	1128	6	+	*Brassica napus*	core promoter element around -30 of transcription start
Ghir_A02G 001270.1	TATA-box	TATAA	1129	5	-	*Arabidopsis thaliana*	core promoter element around -30 of transcription start
Ghir_A02G 001270.1	TATA-box	TATA	1130	4	-	*Arabidopsis thaliana*	core promoter element around -30 of transcription start
Ghir_A02G 001270.1	TATA-box	TATA	1328	4	-	*Arabidopsis thaliana*	core promoter element around -30 of transcription start
Ghir_A02G 001270.1	TATA-box	TATAA	1500	5	-	*Arabidopsis thaliana*	core promoter element around -30 of transcription start
Ghir_A02G 001270.1	TATA-box	TATA	1501	4	-	*Arabidopsis thaliana*	core promoter element around -30 of transcription start
Ghir_A02G 001270.1	TATA-box	TATAAAT	1705	7	-	*Brassica juncea*	core promoter element around -30 of transcription start
Ghir_A02G 001270.1	TATA-box	TATAAA	1706	6	-	*Helianthus annuus*	core promoter element around -30 of transcription start
Ghir_A02G 001270.1	TATA-box	TATAA	1707	5	-	*Arabidopsis thaliana*	core promoter element around -30 of transcription start
Ghir_A02G 001270.1	TATA-box	TATA	1708	4	-	*Arabidopsis thaliana*	core promoter element around -30 of transcription start
Ghir_A02G 001270.1	TATA-box	ATATAT	1829	6	-	*Brassica napus*	core promoter element around -30 of transcription start
Ghir_A02G 001270.1	TATA-box	TATA	1830	4	-	*Arabidopsis thaliana*	core promoter element around -30 of transcription start
Ghir_A02G 001270.1	TATA-box	TATAAATA	1962	8	-	*Daucus carota*	core promoter element around -30 of transcription start
Ghir_A02G 001270.1	TATA-box	TATAAAT	1963	7	-	*Brassica juncea*	core promoter element around -30 of transcription start
Ghir_A02G 001270.1	TATA-box	TATAAA	1964	6	-	*Helianthus annuus*	core promoter element around -30 of transcription start
Ghir_A02G 001270.1	TATA-box	TATAA	1965	5	-	*Arabidopsis thaliana*	core promoter element around -30 of transcription start
Ghir_A02G 001270.1	TATA-box	TATA	1966	4	-	*Arabidopsis thaliana*	core promoter element around -30 of transcription start
Ghir_A02G 001270.1	TATA-box	TATA	1972	4	-	*Arabidopsis thaliana*	core promoter element around -30 of transcription start
Ghir_A02G 001270.1	TATA-box	TATAAAT	1976	7	-	*Brassica juncea*	core promoter element around -30 of transcription start
Ghir_A02G 001270.1	TATA-box	TATAAA	1977	6	-	*Helianthus annuus*	core promoter element around -30 of transcription start
Ghir_A02G 001270.1	TATA-box	TATAA	1978	5	-	*Arabidopsis thaliana*	core promoter element around -30 of transcription start
Ghir_A02G 001270.1	TATA-box	TATA	1979	4	-	*Arabidopsis thaliana*	core promoter element around -30 of transcription start
Ghir_A02G 001270.1	AT~TATA-box	TATATA	28	6	+	*Arabidopsis thaliana*	
Ghir_A02G 001270.1	AT~TATA-box	TATATA	30	6	+	*Arabidopsis thaliana*	
Ghir_A02G 001270.1	AT~TATA-box	TATATA	685	6	+	*Arabidopsis thaliana*	
Ghir_A02G 001270.1	AT~TATA-box	TATATA	1056	6	-	*Arabidopsis thaliana*	
Ghir_A02G 001270.1	GT1-motif	GGTTAA	830	6	+	*Arabidopsis thaliana*	light responsive element
Ghir_A02G 001270.1	Gap-box	CAAATGAA(A/G)A	198	9.5	-	*Arabidopsis thaliana*	part of a light responsive element
Ghir_A02G 001270.1	Unnamed__6	taTAAATATct	1959	10	-	*Zea mays*	
Ghir_A02G 001270.1	AE-box	AGAAACAA	1152	8	+	*Arabidopsis thaliana*	part of a module for light response

Ghir_A02G 001270.1	AE-box	AGAAACTT	124 0	8	-	*Arabidopsis thaliana*	part of a module for light response
Ghir_A02G 001270.1	TCT-motif	TCTTAC	120 3	6	+	*Arabidopsis thaliana*	part of a light responsive element
Ghir_A02G 001270.1	TCT-motif	TCTTAC	121 6	6	+	*Arabidopsis thaliana*	part of a light responsive element
Ghir_A02G 001270.1	Unnamed__4	CTCC	236	4	+	*Petroselinu m hortense*	
Ghir_A02G 001270.1	Unnamed__4	CTCC	249	4	-	*Petroselinu m hortense*	
Ghir_A02G 001270.1	Unnamed__4	CTCC	975	4	+	*Petroselinu m hortense*	
Ghir_A02G 001270.1	Unnamed__4	CTCC	104 3	4	+	*Petroselinu m hortense*	
Ghir_A02G 001270.1	Unnamed__4	CTCC	126 7	4	-	*Petroselinu m hortense*	
Ghir_A02G 001270.1	Unnamed__4	CTCC	161 6	4	+	*Petroselinu m hortense*	
Ghir_A02G 001270.1	TGACG-motif	TGACG	414	5	-	*Hordeum vulgare*	cis-acting regulatory element involved in the MeJA-responsiveness
Ghir_A02G 001270.1	MYC	CATTTG	202	6	+	*Arabidopsis thaliana*	
Ghir_A02G 001270.1	MYC	CATTTG	241	6	+	*Arabidopsis thaliana*	
Ghir_A02G 001270.1	MYC	CATTTG	670	6	+	*Arabidopsis thaliana*	
Ghir_A02G 001270.1	MYC	CAATTG	139 5	6	-	*Arabidopsis thaliana*	
Ghir_A02G 001270.1	ARE	AAACCA	115 8	6	+	*Zea mays*	cis-acting regulatory element essential for the anaerobic induction
Ghir_A02G 001270.1	ARE	AAACCA	145 2	6	+	*Zea mays*	cis-acting regulatory element essential for the anaerobic induction
Ghir_A02G 001270.1	ERE	ATTTCATA	150 3	8	-	*Nicotiana glutinos*	
Ghir_A02G 001270.1	CGTCA-motif	CGTCA	414	5	+	*Hordeum vulgare*	cis-acting regulatory element involved in the MeJA-responsiveness
Ghir_A02G 001270.1	MBS	CAACTG	283	6	+	*Arabidopsis thaliana*	MYB binding site involved in drought-inducibility
Ghir_A02G 001270.1	AuxRR-core	GGTCCAT	114 2	7	+	*Nicotiana tabacum*	cis-acting regulatory element involved in auxin responsiveness
Ghir_A02G 001270.1	TCA-element	CCATCTTTTT	400	9	+	*Nicotiana tabacum*	cis-acting element involved in salicylic acid responsiveness
Ghir_A02G 001270.1		motif_sequence	103	4	+	*organism*	short_function
Ghir_A02G 001270.1		motif_sequence	172	4	+	*organism*	short_function
Ghir_A02G 001270.1		motif_sequence	249	4	-	*organism*	short_function
Ghir_A02G 001270.1		motif_sequence	372	4	-	*organism*	short_function
Ghir_A02G 001270.1		motif_sequence	473	4	+	*organism*	short_function
Ghir_A02G 001270.1		motif_sequence	612	4	-	*organism*	short_function
Ghir_A02G 001270.1		motif_sequence	784	4	+	*organism*	short_function
Ghir_A02G 001270.1		motif_sequence	870	4	-	*organism*	short_function
Ghir_A02G 001270.1		motif_sequence	939	4	-	*organism*	short_function
Ghir_A02G 001270.1		motif_sequence	104 9	4	-	*organism*	short_function
Ghir_A02G 001270.1		motif_sequence	117 9	4	+	*organism*	short_function
Ghir_A02G 001270.1		motif_sequence	120 8	4	+	*organism*	short_function

Ghir_A02G 001270.1		motif_sequence	122 5	4	-	*organism*	short_function
Ghir_A02G 001270.1		motif_sequence	140 7	4	+	*organism*	short_function
Ghir_A02G 001270.1		motif_sequence	185 5	4	+	*organism*	short_function
Ghir_A02G 001270.1	Myb	CAACTG	283	6	+	*Arabidopsis thaliana*	
Ghir_A02G 001270.1	LTR	CCGAAA	108 7	6	-	*Hordeum vulgare*	cis-acting element involved in low-temperature responsiveness
Ghir_A02G 001270.1	Myb-binding site	CAACAG	176 5	6	+	*Nicotiana tabacum*	
Ghir_A02G 001270.1	G-box	TAACACGTAG	625	9	-	*Brassica oleracea*	cis-acting regulatory element involved in light responsiveness
Ghir_D06G 019310.1	P-box	CCTTTTG	200	7	+	*Oryza sativa*	gibberellin-responsive element
Ghir_D06G 019310.1		motif_sequence	26	4	-	*organism*	short_function
Ghir_D06G 019310.1	ATCT-motif	AATCTAATCC	111	9	+	*Pisum sativum*	part of a conserved DNA module involved in light responsiveness
Ghir_D06G 019310.1	TCT-motif	TCTTAC	126	6	+	*Arabidopsis thaliana*	part of a light responsive element
Ghir_D06G 019310.1	GT1-motif	GGTTAA	214	6	-	*Arabidopsis thaliana*	light responsive element
Ghir_D06G 019310.1	MYB-like sequence	TAACCA	215	6	+	*Arabidopsis thaliana*	
Ghir_D06G 019310.1	TC-rich repeats	GTTTTCTTAC	148	9	+	*Nicotiana tabacum*	cis-acting element involved in defense and stress responsiveness
Ghir_D06G 019310.1	GCN4_motif	TGAGTCA	39	7	+	*Oryza sativa*	cis-regulatory element involved in endosperm expression
Ghir_D06G 019310.1	CAAT-box	CAAT	49	4	+	*Nicotiana glutinosa*	
Ghir_D06G 019310.1	CAAT-box	CAAT	68	4	+	*Nicotiana glutinosa*	
Ghir_D06G 019310.1	CAAT-box	CAAT	142	4	-	*Nicotiana glutinosa*	
Ghir_D06G 019310.1	CAAT-box	CAAAT	189	5	-	*Pisum sativum*	common cis-acting element in promoter and enhancer regions
Ghir_D06G 019310.1	CAAT-box	CCAAT	218	5	+	*Arabidopsis thaliana*	common cis-acting element in promoter and enhancer regions
Ghir_D06G 019310.1	CAAT-box	CAAT	219	4	+	*Nicotiana glutinosa*	
Ghir_D06G 019310.1	Myc	TCTCTTA	80	7	+	*Arabidopsis thaliana*	
Ghir_D06G 019310.1	Unnamed__4	CTCC	11	4	-	*Petroselinu m hortense*	
Ghir_D06G 019310.1	Unnamed__4	CTCC	53	4	-	*Petroselinu m hortense*	
Ghir_D06G 019310.1	MYB	TAACCA	215	6	+	*Arabidopsis thaliana*	
Ghir_D06G 019310.1	AAGAA-motif	GAAAGAA	151	7	-	*Avena sativa*	
Ghir_D06G 019310.1	AAGAA-motif	GAAAGAA	161	7	-	*Avena sativa*	
Ghir_D06G 019310.1	MYC	CATTTG	188	6	+	*Arabidopsis thaliana*	

Table S27 Expression profiling of GaZf-BED in different tissues

Principle Transcript ID	Fiber	Leaf	Ovule	Flower	Stem	Root
GaZF-BED01	0	0	0.03	0.06	0	0
GaZF-BED02	4.24	3.22	17.15333	8.89	8.91	10.55
GaZF-BED03	0.006667	0	0.016667	0	0	0.09
GaZF-BED04	0.013333	0.02	0.01	0.05	0.03	0.03
GaZF-BED05	0.25	0.04	0.37	0.41	0.24	0.29
GaZF-BED06	4.106667	1.37	7.633333	6.8	6.2	6.57
GaZF-BED07	0.016667	0	0.123333	0.04	0	0.13
GaZF-BED08	0.923333	0.2	2.15	1.46	1.04	2.17
GaZF-BED09	7.703333	4.09	19.93667	14.76	14.67	15.14
GaZF-BED10	0	0	0	0	0	0
GaZF-BED11	13.59333	10.61	42.74333	25.48	32.2	22.17
GaZF-BED12	0	0	0.403333	0.18	0.03	0.12
GaZF-BED13	3.62	2.21	13.73	8.06	9.65	11.32
GaZF-BED14	0	0	0	0	0.02	0
GaZF-BED15	0.44	0.6	2.146667	1.97	1.94	2.52
GaZF-BED16	7.81	4.07	24.27667	17.94	12.2	16.93
GaZF-BED17	3.62	4.59	4.126667	13.8	45.8	59.18
GaZF-BED18	0	0	0.013333	0.02	0.06	0.03
GaZF-BED19	6.476667	1.68	13.78333	7.51	5.62	7.4
GaZF-BED20	0.703333	10.18	1.673333	12.28	47.14	42.9
GaZF-BED21	0.026667	0	0.063333	0.04	0.02	0.11
GaZF-BED22	0	0.01	0.02	0.02	0.03	0.13
GaZF-BED23	0	0	0.02	0	0	0
GaZF-BED24	6.186667	1.99	18.01	6.01	9.61	10.69
GaZF-BED25	0	0	0.016667	0.02	0	0
GaZF-BED26	0	0	0	0	0	0.05
GaZF-BED27	0.026667	0	0.05	0.06	0.04	0.04
GaZF-BED28	0.01	0.02	0.08	0.03	0.63	1.39
GaZF-BED29	0.176667	0.28	0.973333	0.98	1.18	1.11
GaZF-BED30	6.553333	4.75	18.82333	12.98	10.5	13.11
GaZF-BED31	0.03	0.29	0.126667	4.08	11.44	9.34
GaZF-BED32	0.94	0.48	2.776667	1.97	1.8	1.87
GaZF-BED33	6.186667	5.34	17.39667	20.87	14.33	20.62
GaZF-BED34	0	0	0.043333	0.09	0.21	0.35

Table S28 Expression profiling of GhZF-BED in different tissues and under various stresses

	Abiotic Stresses			
Principle Transcript ID	Cold	Drought	Heat	Salt
Gh_D09G081600.1	2.342667	2.736667	1	1.286429
Gh_D09G079900.1	11.278	9.652667	14.062	7.979286
Gh_A10G089300.1	0	0	0	0
Gh_A10G007600.1	20.836	16.162	14.85	14.58143
Gh_D10G017100.1	15.81267	11.464	9.676667	10.26
Gh_Contig00792G000300.1	0.536	0.896667	0.346	0.701429
Gh_A11G244500.1	0	0	0	0
Gh_A05G308800.1	0.008	0.016	0.017333	
Gh_D05G318900.1	0.034667	0.129333	0.086667	0.074286
Gh_D02G157800.1	1.444	1.310667	0.666	2.705
Gh_A06G138900.1	1.124667	0.186667	0.487333	0.164286
Gh_D11G000600.1	0.661333	0.503333	0.627333	0.492143
Gh_D10G061700.1	30.536	25.21133	9.528	16.08071
Gh_A06G235600.1	5.359333	5.665333	6.907333	6.190714
Gh_A06G170400.1	1.092	1.952	1.518667	2.154286
Gh_D06G172500.1	0.824	1.047333	0.654667	0.625714
Gh_D06G139200.1	2.44	2.335333	1.49	1.506429
Gh_A08G042200.1	1.568667	2.408667	1.655333	1.724286
Gh_D06G218100.1	0.01	0.022	0.022667	0.005
Gh_A09G253200.1	0.86	1.024	1.258667	1.112857
Gh_D08G156800.1	0.027333	0.008	0.036667	0.010714
Gh_D07G164200.1	0.898	1.02	0.664	0.782143
Gh_A10G049000.1	0.052	0.008	0.132667	0
Gh_A10G007500.1	0	0.312667	0.082	0.046429
Gh_D10G017000.1	10.118	9.622	7.649333	7.142143
Gh_D09G081500.1	0.916667	2.424	1.248	1.862857
Gh_Contig00792G000200.1	0.248667	0.37	0.423333	0.593571
Gh_A11G129800.1	0.102	0.206667	0.961333	0.433571
Gh_D10G173600.1	4.714	4.486	6.478667	4.251429
Gh_D10G061600.1	0.304667	0.756	1.704667	0.342143
Gh_D01G163500.1	5.516	7.184	5.922667	6.670714
Gh_A06G138800.1	0.082	0.312	0.145333	0.073571
Gh_A05G101200.1	17.272	13.02933	15.06267	13.04
Gh_D12G162600.1	0.560667	0.462667	0.484667	0.412857
Gh_D06G138800.1	0.246667	0.494667	0.318667	0.353571
Gh_D05G169100.1	1.145333	1.582	3.683333	1.630714

Gh_A06G235500.1	0.03	0.062	0.014	0.059286
Gh_A06G140000.1	0.018667	0.011333	0.036667	0.027857
Gh_D06G218000.1	0.315333	0.33	0.303333	0.472143
Gh_D06G172300.1	3.196667	6.972	4.496	7.032143
Gh_A09G191400.1	0	0.004667	0.006	0.002143
Gh_A07G248100.1	3.146	1.446	1.426	1.173571
Gh_D08G042100.1	0.694667	1.440667	1.050667	1.117143
Gh_D07G103600.1	15.09067	10.14733	3.834	9.907143
Gh_A10G007400.1	4.494	10.302	4.938667	6.094286
Gh_D10G016900.1	0.042	0.06	0.328667	0.107857
Gh_D09G081400.1	1.304	4.296667	2.9	3.675714
Gh_A11G085800.1	1.824	1.845333	2.02	1.721429
Gh_A10G048900.1	0	0.016	0.035333	0.011429
Gh_D01G015200.1	0.153333	0.112667	0.218667	0.139286
Gh_A12G164600.1	1.576	1.759333	1.098	1.210714
Gh_A06G138700.1	20.12133	10.57867	16.258	9.239286
Gh_A04G166700.1	0.057333	0.337333	0.215333	0.300714
Gh_D10G017500.1	60.86267	62.52933	48.60667	30.50857
Gh_D05G113500.1	34.6	27.42267	31.73867	29.20643
Gh_A06G139800.1	0.008667	0.009333	0.027333	0.008571
Gh_D12G162400.1	0.97	1.066667	1.157333	1.185714
Gh_D10G091200.1	0	0	0	0
Gh_D06G138600.1	3.394667	10.14467	3.136667	7.687857
Gh_A07G228700.1	0.068667	0.184667	1.24	0.682143
Gh_A06G171500.1	8.512	31.35067	12.362	18.95
Gh_D06G173000.1	3.074667	3.956667	4.446667	3.837857
Gh_D06G139600.1	0.178667	0.130667	0.176667	0.085714
Gh_A09G086700.1	0.833333	0.879333	2.718	0.703571
Gh_D06G243200.1	21.106	18.422	16.74333	14.57286
Gh_A10G007300.1	0.644667	0.48	0.791333	0.36
Gh_D09G081200.1	0.246667	0.511333	0.595333	0.48
Gh_D08G041100.1	4.025333	5.56	7.738	4.696429
Gh_A10G101100.1	0.528	0.295333	0.275333	0.283571
Gh_A10G048700.1	0	0	0	0
Gh_D10G017400.1	16.032	20.076	14.504	14.15643
Gh_D10G016800.1	0.104667	0.378667	0.164	0.379286
Gh_Contig00802G000400.1	0.076	0.096667	0.084	0.030714
Gh_A12G164500.1	1	1.051333	0.876	0.762857
Gh_A03G141000.1	3.398	7.060667	4.803333	6.074286
Gh_D11G276600.1	0	0.016	0	0.029286

Gh_D10G091000.1	4.600667	3.528667	9.964667	2.985
Gh_D02G158800.1	8.440667	15.09333	9.326	11.57
Gh_A06G139700.1	0.536	1.064	1.477333	3.762143
Gh_A06G138600.1	2.476	2.916	1.621333	2.655
Gh_D06G139500.1	0.041333	0.012	0.043333	0.001429
Gh_D06G138500.1	0.230667	0.412667	0.243333	0.271429
Gh_A07G221100.1	1.994	4.576	2.203333	2.351429
Gh_A06G171400.1	4.664667	9.409333	4.816667	7.012857
Gh_D06G243100.1	0.111333	0.149333	0.354667	0.252857
Gh_D06G172800.1	12.408	23.424	12.51933	19.14357
Gh_A10G007200.1	0.044	0.096667	0.039333	0.014286
Gh_A09G086600.1	4.396	2.952	2.324667	1.375
Gh_D09G081100.1	0.420667	1.961333	1.153333	1.777143
Gh_D07G246100.1	0.606	0.183333	0.445333	0.202857
Gh_A10G048600.1	0.047333	0.036667	0.161333	0.092857
Gh_D09G243400.1	0.224	0.484	0.846	0.455714
Gh_A12G164400.1	2.484667	3.310667	2.672	3.122143
Gh_A10G098100.1	6.208	8.349333	7.726667	7.572143
Gh_A01G166700.1	10.75867	10.36933	5.348	11.43286
Gh_D10G017300.1	25.37	20.42067	18.34	17.44071
Gh_D02G158100.1	10.95467	17.07733	8.098	13.675
Gh_Contig00792G000600.1	1.584	3.822667	1.898	3.227143
Gh_A06G138500.1	0.163333	0.145333	0.103333	0.139286
Gh_D10G062000.1	0.028	0.004	0.030667	0.039286
Gh_D05G364200.1	0.120667	0.132667	0.286667	0.169286
Gh_A06G171300.1	4.143333	5.981333	3.276667	4.622143
Gh_A06G139400.1	0.04	0.224	0.094667	0.121429
Gh_D11G130300.1	0.055333	0.072667	0.464	0.177857
Gh_D06G139400.1	18.25333	8.818667	16.27	14.11071
Gh_A09G086000.1	4.59	5.093333	6.904667	5.477143
Gh_A07G165800.1	1.253333	1.735333	1.133333	1.927143
Gh_D06G243000.1	0	0.004	0	0
Gh_D06G172700.1	4.525333	2.830667	2.806	1.664286
Gh_D07G226600.1	2.893333	4.722	5.138	6.611429
Gh_A10G007700.1	0.686667	2.576	2.109333	3.253571
Gh_A10G007000.1	0.096667	0.128667	0.120667	0.126429
Gh_D09G182400.1	0	0	0	0
Gh_D09G081000.1	4.417333	11.056	4.048667	7.499286
Gh_A11G276900.1	0	0.008	0	0.004286
Gh_A10G089400.1	2.405333	1.669333	5.206	1.648571

Gh_D10G061900.1	0.016	0.005333	0.006667	0
Gh_D10G017200.1	16.69333	22.78733	21.67267	17
Gh_Contig00792G000500.1	0.23	0.322667	0.666	0.608571
Gh_A05G308900.1	8.853333	13.01533	16.41667	10.78071
Gh_A01G016100.1	0.246	0.346667	0.625333	0.279286
Gh_D11G086600.1	0.938667	1.214667	0.999333	0.961429
Gh_D05G319000.1	10.57667	9.974667	14.54467	8.871429
Gh_D02G158000.1	1.435333	2.766667	2.077333	2.826429
Gh_A06G171100.1	0.505333	1.231333	1.138	1.428571
Gh_A06G139300.1	0.014667	0.104667	0.062667	0.068571
Gh_D06G172600.1	4.764667	19.76133	9.424667	15.89929
Gh_D06G139300.1	3.118667	1.565333	1.618	1.109286
Gh_A08G157400.1	0.324667	0.138667	0.039333	0.162143
Gh_A07G106400.1	6.488667	5.18	1.396	5.295714
Gh_D07G218400.1	4.261333	13.07467	7.752667	8.335714
Gh_D06G218400.1	0.394	0.180667	0.465333	0.215
Gh_A10G006900.1	0.010667	0.015333	0.012667	0.016429

	**Tissue**											
**Principle Transcript ID**	**Ovule**	**Fibre**	**Anther**	**Bract**	**Filament**	**Leaf**	**Petal**	**Pistil**	**Root**	**Sepal**	**Stem**	**Torus**
Gh_D09G081600.1	0.383	0.058182	11.70667	25.265	4.13	0.45	14.55	15.54	0.343333	18.02667	0.123333	6.393333
Gh_D09G079900.1	4.461	0.170909	0.186667	4.475	2.47	1.043333	0.32	3.52	1.156667	0.863333	1.206667	0.603333
Gh_A10G089300.1	0.0085	0.007273	0.006667	0	0	0	0	0	0.016667	0	0	0.006667
Gh_A10G007600.1	0.0225	0.009091	0.036667	0.24	0.006667	0.01	0.01	4.42	0.013333	0.06	0.006667	0.04
Gh_D10G017100.1	0.003	0	0	0.035	0.006667	0.016667	0	2.3	0	0.023333	0	0
Gh_Contig00792G000300.1	0.027	0.015455	0	0.125	0	0.023333	0	0	0	0	0.013333	0.02
Gh_A11G244500.1	0	0	0	0	0	0	0	0	0	0	0	0
Gh_A05G308800.1	0.006	0.003636	0	0	0.036667	0	0	0	0.096667	0	0	0
Gh_D05G318900.1	0.0315	0.026364	0	0.02	0	0.023333	0	0	0.123333	0	0.023333	0
Gh_D02G157800.1	0.0135	0.041818	0	2.36	0.13	0	0	2.23	0	0	0.39	0.226667
Gh_A06G138900.1	0.0165	0	0	0	0	0	0	0.13	0	0	0	0
Gh_D11G000600.1	1.0225	1.052727	4.506667	0.655	2.72	0.216667	0.546667	1.88	0.503333	1.05	1.073333	2.196667
Gh_D10G061700.1	0.703	0.111818	0.02	26.2	0.036667	48.78	0.076667	13.34	0.436667	8.923333	0.923333	2.063333
Gh_A06G235600.1	16.0165	7.651818	22.04667	20.65	22.88	1.336667	46.25667	6.58	2.25	32.59333	7.893333	23.43667
Gh_A06G170400.1	0.0365	0.011818	0.033333	0.31	0.08	0.016667	0.016667	0.53	0.71	0.16	0.513333	0.016667
Gh_D06G172500.1	0.01	0	0.01	0.755	0	0.086667	0	0.43	0.016667	0.163333	0.133333	0.023333
Gh_D06G139200.1	0.0695	0	0.013333	1.455	0	0.496667	0.033333	0.75	0.096667	0.346667	0.216667	0

Gh_A08G042200.1	1.882	0.174545	0.933333	1.225	1.586667	1.486667	3.29	0.86	16.25333	0.88	1.536667	0.976667
Gh_D06G218100.1	0	0	0	0.04	0	0	0	0	0	0	0	0
Gh_A09G253200.1	0.818	0.036364	0	0.03	0.046667	0.776667	0	0.66	1.426667	0.06	2.083333	0.266667
Gh_D08G156800.1	0.0185	0	0	0	0	0.163333	0	0	0.363333	0	0.753333	0.046667
Gh_D07G164200.1	1.0235	1.034545	11.03667	0.74	2.603333	0.446667	7.03	0.38	3.546667	1.18	3.516667	2.243333
Gh_A10G049000.1	0.0105	0	2.77	1.215	0.113333	0.023333	8.036667	0.32	0.263333	0.153333	0.303333	0.07
Gh_A10G007500.1	0	0	0	0	0	0	0	0	0	0	0	0
Gh_D10G017000.1	0	0	0	0.74	0	0.006667	0	3.21	0	0.043333	0.006667	0
Gh_D09G081500.1	0.0075	0.067273	0.42	2.46	0.06	0.153333	0.166667	0.77	0.266667	2.133333	0.026667	0.63
Gh_Contig00792G000200.1	0.173	0.496364	0.07	0.35	0.076667	0.103333	0.17	0	1.286667	0.203333	0.506667	0.35
Gh_A11G129800.1	0.3025	1.246364	0.096667	0.015	0.77	0.13	0.213333	2.08	1.783333	0.226667	1.423333	0.593333
Gh_D10G173600.1	21.2045	2.444545	8.19	23.74	2.366667	37.79333	1.7	13.66	10.91	7.416667	23.01333	35.00333
Gh_D10G061600.1	0.0075	0.014545	1.523333	0.95	0.896667	0.016667	0	0	206.34	0	0.153333	0.023333
Gh_D01G163500.1	13.369	11.83727	25.98333	1.805	12.37333	2.88	3.613333	1.17	15.70667	16.67	31.97667	25.93
Gh_A06G138800.1	0.002	0	0	1.43	0	0.046667	0.033333	0	0	0.02	0	0
Gh_A05G101200.1	0.292	0.24	0.156667	4.415	0.61	1.816667	0.816667	11.02	0.186667	3.363333	6.14	0.643333
Gh_D12G162600.1	7.507	1.940909	13.78667	7.605	12.2	0.113333	10.77	7	5.03	5.356667	1.673333	2.103333
Gh_D06G138800.1	0.0165	0.035455	0	0.18	0	0	0.02	0.21	0.14	0.053333	0.116667	0
Gh_D05G169100.1	0.096	0.104545	0.033333	0.715	0.08	0.263333	0.126667	3.9	0.553333	0.376667	0.193333	0.186667
Gh_A06G235500.1	0.0685	0	0.056667	0.26	0	0.006667	0.063333	0	0.843333	0.026667	0	0.103333
Gh_A06G140000.1	0.1525	0	0.113333	0	0.036667	0.1	0.106667	0	5.106667	0.086667	4.233333	0.116667
Gh_D06G218000.1	0.0645	0.017273	0	0.26	0	0.123333	0.026667	0.24	0	0.113333	0.096667	0.086667
Gh_D06G172300.1	0.0155	0.025455	0.006667	2.29	0.063333	0.373333	0.04	0.92	0.146667	0.366667	0.406667	0.05
Gh_A09G191400.1	0.041	0.020909	0.15	0.035	0.023333	0	0.006667	0	0.046667	0.026667	0.016667	0.006667
Gh_A07G248100.1	2.0785	0.266364	0.593333	5.525	0.22	1.566667	2.776667	8.38	0.133333	0.973333	0.583333	0.286667
Gh_D08G042100.1	1.921	0.384545	1.07	0.62	1.736667	1.666667	4.72	0.53	5.52	1.013333	0.863333	0.67
Gh_D07G103600.1	1.9875	0.445455	2.516667	0.795	4.023333	6.256667	1.613333	2.41	13.44	1.25	12.99333	4.056667
Gh_A10G007400.1	0	0	0	2.01	0.006667	2.04	0	0.93	0.003333	0.62	0.993333	0.013333
Gh_D10G016900.1	1.416	1.409091	0.306667	0.135	0	0.013333	0.03	0.15	3.176667	0.026667	2.733333	0
Gh_D09G081400.1	0.026	0.012727	0.093333	4.805	0.116667	0.386667	0.27	1.15	0.173333	2.476667	0.296667	0.01
Gh_A11G085800.1	0.312	0.070909	1.963333	1.65	4.983333	0.963333	4.32	2.14	4.47	2.36	2.46	2.313333
Gh_A10G048900.1	0.0495	0	0.676667	0	0.083333	0.036667	1.89	0	0.92	0	0	0.053333
Gh_D01G015200.1	1.574	0.194545	0.173333	0.465	0.506667	0.116667	0.43	0.32	0.426667	0.606667	0.28	0.66
Gh_A12G164600.1	20.635	5.194545	8.49	17.62	1.426667	0.893333	4.876667	19.45	5.693333	6.74	2.213333	3.506667

Gh_A06G138700.1	0.2005	0.07	0.31	12.075	0.326667	0.386667	1.266667	16.01	0.26	2.196667	0.38	0.153333
Gh_A04G166700.1	3.276	0.571818	6.173333	0.045	0.216667	1.383333	0.413333	0.57	7.416667	1.696667	2.986667	1.966667
Gh_D10G017500.1	0.012	0.015455	0.073333	22.08	0.68	18.32333	0.27	13.7	0.373333	11.99333	39.25333	2.423333
Gh_D05G113500.1	0.501	0.407273	0.22	7.48	1.6	3.01	1.51	19.67	0.036667	4.536667	9.563333	0.84
Gh_A06G139800.1	0.0015	0	0.926667	0.05	0.206667	0	2.13	0.02	0.376667	0.086667	0	0.023333
Gh_D12G162400.1	3.348	2.544545	24.56333	11.58	23.82333	0.35	39.09333	5.15	4.366667	11.29667	4.28	5.45
Gh_D10G091200.1	0	0.010909	0.026667	0	0.02	0	0	0	0.043333	0	0.013333	0
Gh_D06G138600.1	0.41	0.046364	0.073333	2.97	0.03	2.106667	0.103333	0.21	0	0.296667	0.996667	0.093333
Gh_A07G228700.1	2.3615	2.744545	0.013333	0	0	0.286667	0.12	0	0.496667	0.4	0.306667	1.056667
Gh_A06G171500.1	0.1335	0.246364	17.18	51.465	21.39	2.333333	4.52	26.44	1.31	5.636667	5.286667	5.226667
Gh_D06G173000.1	0.036	0.009091	0	1.495	0	0.8	0	0.85	2.123333	0.166667	0.276667	0.026667
Gh_D06G139600.1	0.16	0.009091	0.593333	0	2.35	0.243333	0.033333	0.05	7.96	0.103333	5.34	1.54
Gh_A09G086700.1	0.112	0.204545	0.266667	2.56	3.193333	0.166667	0.62	0.69	0.373333	3.276667	2.243333	1.366667
Gh_D06G243200.1	9.2275	11.10545	2.423333	49.335	0.77	6.313333	31.78	33.92	4.863333	32.52	6.36	11.39333
Gh_A10G007300.1	0.002	0	0	0.635	0	0.14	0	1.45	0.03	0.06	0.033333	0
Gh_D09G081200.1	0.0015	0	0.026667	0.62	0	0.056667	0.053333	0.03	0.346667	0.063333	0	0
Gh_D08G041100.1	0.004	0	0.22	7.085	0.083333	0.263333	0.36	1.04	0.22	0.616667	0.086667	0
Gh_A10G101100.1	0.061	0.020909	0	0	0.27	0	0	1.61	0	0	0	0
Gh_A10G048700.1	0.006	0.018182	0.06	0	0	0	0	0	0.06	0	0	0
Gh_D10G017400.1	0	0	0.05	10.42	0.183333	2.083333	0.153333	2.38	0.36	1.95	1.203333	0.383333
Gh_D10G016800.1	0.0415	0.003636	0	0.03	0	0.006667	0	0.19	1.516667	0.043333	0.066667	0.016667
Gh_Contig00802G000400.1	0.4925	0.174545	0.703333	0.07	0.193333	0	0.096667	0.39	0.143333	0.29	0.25	0.496667
Gh_A12G164500.1	1.2915	0.486364	0.316667	8.31	0.313333	0.44	1.21	4.6	0.693333	2.013333	0.74	0.553333
Gh_A03G141000.1	32.077	36.04364	1.693333	5.655	5.213333	4.026667	2.826667	2.53	2.99	3.416667	8.966667	5.68
Gh_D11G276600.1	0.021	0.018182	0	0	0	0.016667	0	0	2.156667	0	0.89	0
Gh_D10G091000.1	0.133	0.391818	3.71	9.985	4.246667	0.623333	10.11667	4.52	8.206667	6.09	5.65	1.1
Gh_D02G158800.1	22.996	49.81182	1.72	7.625	9.926667	4.87	6.01	3.44	5.663333	9.1	12.88667	22.71
Gh_A06G139700.1	0.0695	0.01	1.736667	2.815	0.7	0.413333	2.95	0	4.383333	2.32	1.773333	0.623333
Gh_A06G138600.1	0.1595	0.029091	0	1.03	0.02	0.403333	0	0.58	0.02	0.286667	0.423333	0.036667
Gh_D06G139500.1	0	0	0.08	0.095	0.026667	0	0	0	0.103333	0.073333	0.006667	0
Gh_D06G138500.1	0.1955	0.088182	0	0.31	0.01	0.013333	0.013333	1.14	1.326667	0.14	5.633333	0.143333
Gh_A07G221100.1	0.0515	0	0.383333	4.19	0.15	6.133333	0.973333	1.56	6.996667	1.736667	10.39333	0.3
Gh_A06G171400.1	0.0525	0.023636	0	3.8	0.153333	0.606667	0.056667	0.95	0.196667	0.286667	1.71	0.213333
Gh_D06G243100.1	0.28	0.031818	0.016667	0.445	0.006667	0.076667	0.173333	0.15	0.483333	0.813333	0.16	0.11

Gh_D06G172800.1	0.12	0.255455	0.053333	31.97	0.076667	2.186667	0.206667	4.2	0.376667	7.136667	3.726667	1.493333
Gh_A10G007200.1	0.0095	0	0.006667	0.09	0.01	0	0.01	0	0	0.013333	0	0
Gh_A09G086600.1	0.103	0.152727	0.533333	0.99	1.266667	0.126667	0.19	1.84	0.076667	0.556667	0.04	0.19
Gh_D09G081100.1	0.0415	0.005455	0	2.13	0	0.24	0.086667	0	0.056667	0.27	0.1	0.026667
Gh_D07G246100.1	0.0615	0.002727	0.013333	0.095	0.056667	0.026667	0.056667	2.47	0.056667	0.046667	0.123333	0.01
Gh_A10G048600.1	0.024	0	2.06	0.105	0.206667	0.036667	0.246667	0	526.16	0.52	4.866667	0.22
Gh_D09G243400.1	1.278	0.034545	0	0.245	0	0.91	0.053333	0	2.686667	0	1.26	0.29
Gh_A12G164400.1	2.764	1.569091	7.583333	13.4	11.56	0.713333	24.82333	8.71	2.103333	16.61	3.64	4.006667
Gh_A10G098100.1	7.6575	0.517273	0.566667	16.735	0.726667	41.53	0.486667	10.46	9.02	4.513333	15.87	8.223333
Gh_A01G166700.1	33.4595	58.06091	12.16667	0.14	5.98	1.446667	0.726667	0.19	12.76	6.256667	20.04333	10.71667
Gh_D10G017300.1	0.022	0.008182	0.026667	5.105	0.04	0.226667	0.063333	4.57	0.176667	1.966667	0.23	0.073333
Gh_D02G158100.1	0.4035	0.141818	0.066667	2.52	0.023333	3.096667	0.11	0.93	0.036667	0.146667	0.713333	0.063333
Gh_Contig00792G000600.1	0.0135	0.009091	0.006667	0.32	0.006667	0.523333	0	0.14	0	0.146667	0.143333	0.033333
Gh_A06G138500.1	0.06	0.268182	0.283333	0.42	0.106667	0.103333	0.203333	0.35	0.98	0.393333	0.72	0.063333
Gh_D10G062000.1	0.004	0	1.436667	0.65	0.183333	0.053333	6.47	0	0.373333	0.306667	0.023333	0.026667
Gh_D05G364200.1	0.0025	0	0.526667	0	1.483333	0.006667	0.126667	0.43	0.023333	0.076667	0.166667	0.116667
Gh_A06G171300.1	5.0535	2.625455	1.393333	6.53	3.62	1.29	1.806667	6.84	4.343333	6.33	6.643333	5.92
Gh_A06G139400.1	0.0975	0.016364	0.18	0.785	0.553333	0.05	0.246667	0.24	1.053333	0.186667	0.413333	0.166667
Gh_D11G130300.1	0.446	2.001818	0.123333	0.275	1.263333	0.026667	0.333333	0.89	1.29	0.346667	1.526667	0.543333
Gh_D06G139400.1	4.365	0.348182	16.84667	13.585	19.95667	1.373333	19.88667	5.2	15.28	17.86	10.12667	7.053333
Gh_A09G086000.1	0.0555	0.012727	0.16	7.155	0.196667	0.536667	0.086667	0.4	0.043333	1.453333	0.423333	0.26
Gh_A07G165800.1	1.5695	6.895455	6.186667	1.725	1.686667	0.78	7.45	1.22	5.866667	1.143333	4.68	1.48
Gh_D06G243000.1	0.005	0	0	0	0	0	0	0	0.073333	0.03	0	0
Gh_D06G172700.1	0.0215	0.001818	0	1.605	0.03	0.19	0	1.15	0.06	0.42	0.13	0.016667
Gh_D07G226600.1	1.7565	4.08	0.093333	0.31	0.053333	1.96	0.056667	1.07	0.54	0.27	1.546667	0.026667
Gh_A10G007700.1	0.007	0.011818	0.016667	0.5	0.006667	0.076667	0.03	0.85	5.003333	0.086667	0.476667	0.023333
Gh_A10G007000.1	0.4885	0.323636	0	0.39	0.006667	0	0	0.13	9.103333	0.28	2.893333	0.433333
Gh_D09G182400.1	0	0	0.743333	0	0.026667	0	0	0	0	0	0	0
Gh_D09G081000.1	0.057	0.135455	0.48	3.565	0.383333	2.25	0.15	1.02	0.19	3.373333	0.713333	0.386667
Gh_A11G276900.1	0.005	0.084545	0	0	0	0	0	0	3.553333	0	2.726667	0
Gh_A10G089400.1	1.447	1.368182	5.853333	5.05	3.813333	0.613333	5.413333	1.31	6.113333	4.27	3.093333	1.076667
Gh_D10G061900.1	0.028	0	0.333333	0	0.03	0	0.823333	0	0.293333	0	0	0
Gh_D10G017200.1	0.0715	0.047273	0.156667	52.35	0.753333	3.91	1.36	33.56	2.61	13.55	2.903333	0.783333
Gh_Contig00792G000500.1	0.103	0.487273	0.006667	0.19	0.023333	0.023333	0	0.45	0.5	0.276667	0.19	0.103333

Gh_A05G308900.1	1.215	0.47	5.183333	25.425	7.59	1.503333	17.02	9.6	20.61667	10.82333	9.266667	4.87
Gh_A01G016100.1	0.7795	0.143636	0.57	0.45	0.796667	0.323333	0.396667	0.62	1.126667	1.186667	0.68	0.98
Gh_D11G086600.1	0.2935	0.067273	2.283333	1.03	3.113333	1.026667	3.013333	1.41	4.653333	2.043333	2.166667	2.49
Gh_D05G319000.1	3.1175	1.892727	6.79	14.615	6.266667	0.83	12.41333	8.64	20.06333	6.52	6.853333	3.126667
Gh_D02G158000.1	0.099	0.630909	0.05	1.265	0.173333	0.75	0.18	0.59	1.07	0.72	1.143333	0.086667
Gh_A06G171100.1	0.5865	6.608182	0	0.255	0.01	0.12	0	0.27	2.33	0.016667	0.246667	0
Gh_A06G139300.1	0.008	0.007273	0	0.525	0	0.11	0	0.18	0.03	0.01	0.073333	0.04
Gh_D06G172600.1	0.1615	1.286364	0.146667	12.315	0.123333	1.306667	0.263333	1.86	0.886667	6.263333	4.1	1.636667
Gh_D06G139300.1	0.1115	0.015455	0.726667	3.065	0.043333	0.486667	1.073333	1.84	0.116667	1.18	0.653333	0.313333
Gh_A08G157400.1	0.2205	0.009091	0	0	0	0.266667	0	0.38	0.686667	0.126667	0.413333	0.096667
Gh_A07G106400.1	2.1055	0.412727	3.403333	0.195	2.413333	3.33	1.896667	0.34	9.65	1.246667	6.016667	2.646667
Gh_D07G218400.1	0.0105	0.055455	0.086667	13.175	0.016667	15.82333	0.42	1.38	29.09333	1.206667	51.63	0.613333
Gh_D06G218400.1	0.131	0.03	0.033333	0.105	0.126667	0.043333	0.12	0.26	0.176667	0.216667	0.066667	0.09
Gh_A10G006900.1	0.021	0	0	0.03	0	0	0	0	0.21	0	0	0

Table S29 (a, b, c) .Expression profiling of ZmZF-BED in different tissues and under biotic and abiotic stresses.

		Tissues												
**Gene ID**	**Gene_Name_Class**	**Anther**	**Ear**	**Embryo**	**Endosperm**	**Kernel**	**Leaf**	**Meristem**	**Root**	**Seed**	**Seedlings**	**Shoot**	**Silk**	**Tassel**
Zm00001d003128	ZmZf-BED02	5.7440433	4.0077984	6.1974622	5.721817926	14.9419333	28.70843	26.78115033	21.053183	24.00922	6.684023	18.04052	24.18409	16.699377
Zm00001d003194	ZmZf-BED04	5.946761	8.9103423	7.796487067	3.312332	0.37211875	0.332062	0.220052333	0.65074648	0.642569	0.075293	0.11662	0.344665	0.4559504
Zm00001d004256	ZmZf-BED06	0.1387637	13.239256	7.593515567	8.339147704	0	0	0	0	0.045035	0	0	0	0
Zm00001d006692	ZmZf-BED07	12.769827	10.586621	0.3360264	16.74654996	0	0	0	0	0.086691	0.188474	0	0	0
Zm00001d010895	ZmZf-BED08	0	0	0	0	0.673729	0.013884	0	0.03943989	0.066272	0	0.02364	0	0.0156396
Zm00001d011158	ZmZf-BED09	0	0	4.464008567	3.761758815	12.624513	10.27051	9.921863333	9.48281892	11.23541	7.643921	13.5908	15.62274	16.527966
Zm00001d013336	ZmZf-BED17	0	0	4.4041199	0	20.8226568	36.07481	61.19650167	26.5601048	40.03914	21.21163	24.20092	33.83505	54.665834
Zm00001d015283	ZmZf-BED24	0	0.1832411	188	0.273065815	0	0.086409	0	0.07056674	0.09422	0	0.144382	0	0.2747431
Zm00001d016617	ZmZf-BED25	17.220169	16.652897	16.5163911	12.40950033	0	0.011554	0	0.01644055	0.005787	0	0	0	0.0128412
Zm00001d017846	ZmZf-BED26	22.84254	17.844512	18.24697823	23.25255726	20.2923893	31.96753	57.76275833	24.8235364	24.94794	13.73506	32.07862	42.11247	38.783573
Zm00001d022534	ZmZf-BED34	0	11.821449	12.8719304	4.998764148	28.9295063	18.74578	37.44193667	15.3925828	34.05599	18.62426	13.86636	22.37042	28.352522
Zm00001d023717	ZmZf-BED35	36.663116	19.663628	0.015963	2.351258741	1.396869	0.028965	0	0.00210957	1.14724	0	0.02522	0	0.413732
Zm00001d025207	ZmZf-BED36	24.403108	17.413039	9.148176133	21.80306626	0.35124775	1.803347	1.628758333	1.37955106	3.212969	0.083325	0.752157	2.361663	1.376144
Zm00001d025324	ZmZf-BED37	14.335646	9.4088925	0	3.956477111	0	0	0	0	0.015671	0	0	0	0
Zm00001d026358	ZmZf-BED38	7.466803	13.951909	2.393602133	9.986935444	8.37411575	10.44924	16.081971	10.1389445	21.31683	9.454661	10.10165	15.27088	20.90075
Zm00001d028972	ZmZf-BED41	0	0	0.018553733	0.073780556	0.22807275	6.072221	7.756086333	3.9060882	5.307488	1.833385	5.459653	10.28091	10.494491
Zm00001d029192	ZmZf-BED42	7.8597973	7.0342073	4.9276409	6.365267667	0	0.006461	0	0.00888544	0.014166	0	0	0.138764	0
Zm00001d033361	ZmZf-BED43	30.267391	0.0218774	15.33750073	31.75797644	0	0	0	0	0.069515	0	0	0	0
Zm00001d033903	ZmZf-BED44	0	8.3062497	0	0	4.551925	7.047095	11.79244933	6.26182918	7.093839	4.532588	6.510267	24.18409	10.458007
Zm00001d039328	ZmZf-BED45	1.3010133	0.8579521	47.25110207	25.72492374	16.8454385	16.71541	10.31041367	11.4375805	9.180684	26.02441	12.48099	8.745945	14.040166

Zm00001d043354	ZmZf-BED52	0.300663	0	1.935787933	0.037932407	13.168093	13.95739	20.713315	15.6774909	17.62551	7.230768	15.13657	17.88189	18.16608
Zm00001d047922	ZmZf-BED54	0	0	0	0	0.04605425	0.383299	0.110338333	0.66846678	0.70819	0.060479	0.278605	0.312914	0.5332254
Zm00001d049450	ZmZf-BED55	0	0	16.17141547	0.002944111	5.98754125	7.040129	14.74845133	7.92046104	9.430816	4.24618	6.134191	15.51752	11.107753
Zm00001d050879	ZmZf-BED58	0	0	0	0	0	0	0	0	0.021893	0	0	0	0.0203416

		**Abiotic Stresses**							
**Gene ID**	**Gene_Name_Class**	**Cold**	**Drought**	**Heat**	**Nutrient Deficiency**	**Ozone**	**Salt**	**Shade**	**Water**
Zm00001d003128	ZmZf-BED02	3.96987547	4.12063016	6.563725586	6.851316556	19.2417108	9.5561	13.3977352	12.6912358
Zm00001d003194	ZmZf-BED04	1.403797217	0.80467085	0.724878469	0.6095823	0.6432565	0.609303	0	0.8477947
Zm00001d004256	ZmZf-BED06	0.003868995	0.00681669	0.002588697	0.002263422	0	0.005601	0	0.00094505
Zm00001d006692	ZmZf-BED07	0.295107717	0.30378592	0.293641738	0.042011689	0	0.113519	0	0.60022712
Zm00001d010895	ZmZf-BED08	0.101248571	0.02483801	0.025381476	0.014391144	0	0.079763	0	0.17417998
Zm00001d011158	ZmZf-BED09	11.10335202	10.1346384	13.20495419	11.14992999	7.3547965	9.511545	10.7618842	8.65251438
Zm00001d013336	ZmZf-BED17	25.2395308	38.6067154	31.37416633	37.4162789	22.463236	38.21804	26.1409938	36.0285161
Zm00001d015283	ZmZf-BED24	1.282052702	1.36097862	1.338077159	1.820108078	0	0.194326	0	0.34811536
Zm00001d016617	ZmZf-BED25	0.069683747	0.0169226	0.017862386	0.0023347	0	0.010955	0	0.00486403
Zm00001d017846	ZmZf-BED26	25.50453276	30.3719977	19.81753601	26.1478688	25.4090777	24.93061	30.9866034	29.3346829
Zm00001d022534	ZmZf-BED34	11.72035128	24.9196222	21.83823543	22.81124993	14.8655528	25.178	15.7973153	24.9406033
Zm00001d023717	ZmZf-BED35	0.041249732	0.18811276	0.307879897	0.005274044	0.10946	0.042151	0.03352067	0.00942596
Zm00001d025207	ZmZf-BED36	0.315093581	0.51567404	0.426945455	0.364737244	0.41474867	1.849989	0.66381178	1.13619901
Zm00001d025324	ZmZf-BED37	0	0.00310701	0.002179793	0	0	0	0	0
Zm00001d026358	ZmZf-BED38	6.77820796	13.7697486	14.0091103	13.71019346	10.3589993	14.68463	8.11365267	12.0840988
Zm00001d028972	ZmZf-BED41	3.726438929	4.64445173	3.160196607	6.839128156	3.6605315	4.007868	4.29822667	3.42145932
Zm00001d029192	ZmZf-BED42	0.004270636	0.01001085	0.036037166	0.009713778	0.064373	0.043858	0	0.00932119
Zm00001d033361	ZmZf-BED43	0.003899727	0.01195497	0.000905669	0.003131978	0	0	0	0
Zm00001d033903	ZmZf-BED44	5.204657768	7.750082	4.757169076	8.2534069	5.857199	8.843649	7.22632311	8.35241752
Zm00001d039328	ZmZf-BED45	10.66473896	18.486065	21.98783046	19.0209557	12.3761648	15.51148	17.1838017	15.0781033
Zm00001d043354	ZmZf-BED52	4.104452177	10.5892824	8.026772828	5.460174878	11.6122972	11.30332	12.5278756	10.3103409
Zm00001d047922	ZmZf-BED54	1.798990359	1.11454242	1.188661703	1.183090144	0.40841683	0.728304	0	1.1197359
Zm00001d049450	ZmZf-BED55	5.481440152	9.27213526	10.16768865	7.041849389	8.84877083	9.917098	6.04732433	7.70861383
Zm00001d050879	ZmZf-BED58	0.000941136	0.0050449	0.006707759	0	0	0.003893	0	0.00173354

		Biotic Stresses																		
**Gene ID**	**Gene_Name_Class**	**Aphid (30)**	**Aspergillus flavus (119)**	**Cercospora zeina (6)**	**Exserohilum turcicum (36)**	**Fungal (18)**	**Fusarium graminearum (20)**	**Fusarium verticillioides (27)**	**Fusarium virgliforme (18)**	**Gray leaf spot disease (18)**	**Herbaspirillum seropedicae (4)**	**Maize iranian mosaic virus (3)**	**Oligonychus pratensis (8)**	**Ostrinia furnacalis (3)**	**Rice black-streaked dwarf virus (3)**	**Sesamia larvae (3)**	**Tetranychus urticae (32)**	**Trichoderma atroviride (3)**	**Trichoderma virens (30)**	**Ustilago (24)**
Zm00001d003128	ZmZf-BED02		6.458750229	9.555462667	0.040762889	12.6794687	15.21668	12.42581348	14.6546673	0.657075	9.606253	5.006428	8.950658	9.2136783	6.01002	0.391753	11.36068	17.8466	16.66249	12.67426779
Zm00001d003194	ZmZf-BED04	0.380970667	0.232635556	1.033263667	0.165825806	0.51643667	5.361047	0.609122074	4.15708544	0.715768	1.885327	0.063858	0.018218	0	0.05522	0.742286	1.176612	0.087283	0.374571	1.261648917
Zm00001d004256	ZmZf-BED06	0	0	0	0	0	0	0	0	0	0	0	0	0	0	0	0	0	0	0.029474625
Zm00001d006692	ZmZf-BED07	0	0	0.982200833	1.237003056	0	0.036624	0.019181037	0.43225072	0.585558	0.12072	0.07484	0	0	0.062743	0	0.198041	0	0	0.571465542

Zm00001d010895	ZmZf-BED08	0	0	0	0.317110667	0	0	0	0	0.014069	0	0	0.0201	0	0	0	0	0	0	0.02515525
Zm00001d011158	ZmZf-BED09	9.909015733	10.2388614	12.35048533	22.11272892	5.90139861	12.15871	9.838052852	10.0980736	8.328914	6.206493	9.545641	9.112643	9.8182823	6.0141	4.640881	13.01121	11.09925	8.615872	15.94061667
Zm00001d013336	ZmZf-BED17	33.8396006	22.301212	33.8322235	48.31040083	29.0932624	27.40635	38.53250256	30.0508667	26.38561	37.25155	50.48834	25.65742	18.439678	26.95019	23.76748	24.40402	45.28801	29.76181	35.21517375
Zm00001d015283	ZmZf-BED24	0	0.01998665	9.5884985	1.487739944	0	0.21522	2.230977481	3.00203206	4.348417	1.446579	1.745245	0.012995	0	0	0.381779	1.532872	0.089216	0.019891	2.087757
Zm00001d016617	ZmZf-BED25	0	0	0.022479667	0	0	0	0	0.04762078	0.001758	0	0	0	0	0	0	0	0.035425	0	0.05623

Zm00001d017846	ZmZf-BED26	18.55090767	12.5152775	34.50414783	30.04308153	24.2208049	20.91766	23.47756596	25.2647272	36.11748	25.60367	17.42427	18.7034	22.217269	12.2379	30.64043	20.29259	54.21526	19.3784	32.09016421
Zm00001d022534	ZmZf-BED34	23.02219277	40.5587292	24.28042167	25.24538275	17.1681779	17.4231	38.434576	17.1675479	17.03083	12.47341	32.20753	14.21212	11.281659	12.64859	2.420071	17.02639	26.27887	35.20059	21.06123458
Zm00001d023717	ZmZf-BED35	0.237664533	0.50555999	0.019172333	0	0	0.26077	1.243447889	0	0.023337	0	0.137694	0	0	0	0	0.003096	0	0	0.039211417
Zm00001d025207	ZmZf-BED36	0.272424967	0.36754708	0.508269667	0	0.63363633	0.377684	0.848167519	3.63062211	0.032789	0	0.383792	0.617858	0.535838	0.416774	1.047991	0.878574	0.808047	0.220249	0.195821125
Zm00001d025324	ZmZf-BED37	0	0	0	0	0	0	0	0	0	0	0	0	0	0	0	0	0	0	0

Zm00001d026358	ZmZf-BED38	1.23142E+33	13.6579628	15.6072683	19.23287364	9.68329922	10.19862	20.63669667	12.8600618	9.179027	8.73547	17.17047	8.55951	8.6657213	23.23776	0.09707087	9.457797	9.930883	17.33305	13.32779925
Zm00001d028972	ZmZf-BED41	120.25.	2.12010309	6.081291667	5.428224944	4.96267017	4.542406	5.42237618 5	1.28661544	4.257602	2.543171	1.74528	4.11529	2.3107883	1.918715	9.202037	4.365608	7.806178	2.734	14.11128225
Zm00001d029192	ZmZf-BED42		0	0	0.033297889	0	0	0.00594663	0	0	0	0.13605	0.023097	0	0	0	0	0	0	0.021936875
Zm00001d033361	ZmZf-BED43	0	0	0	0	0	0	0	0	0.011652	0	0	0	0	0	0	0	0	0	0.020364875
Zm00001d033903	ZmZf-BED44	5.640088667	4.91681287	8.618567	9.14005075	4.97081017	6.881541	6.67161563	7.93624267	5.826565	8.031923	10.7139	6.290797	6.3492537	5.791695	4.900577	6.130269	5.410982	5.991866	7.72624125

Zm00001d039328	ZmZf-BED45	37.96979337	9.74616864	22.38738167	22.66346736	24.63688	9.286032	9.982753481	14.2931198	24.78718	13.6435	37.02186	11.75734	16.291392	17.82156	4.828599	11.46669	12.29462	22.40118	7.037565417
Zm00001d043354	ZmZf-BED52	15.52260753	7.70459543	20.52856933	6.895809778	17.0267669	4.693134	11.75448426	20.4303885	5.990596	0.59046	8.014685	10.51233	10.044949	4.809694	1.445069	11.91435	13.89005	10.38952	5.962207917
Zm00001d047922	ZmZf-BED54	0.428514167	0.41406424	1.513308667	0.070095861	0.90041511	1.569042	0.747437852	2.908023	0.619514	2.080333	0	0.015214	0.049364	0.081836	1.1004	1.68558	0.223302	0.276781	7.754410792
Zm00001d049450	ZmZf-BED55	9.8928266	3.89305496	6.906807333	9.751904	5.73819206	8.992193	8.576161111	8.32949956	5.580576	5.797141	8.939411	5.679127	4.7845207	3.801459	3.52709	6.98618	14.00108	7.373199	8.877146083
Zm00001d050879	ZmZf-BED58	0	0	0	0	0	0.013571	0	0	0	0	0	0	0	0	0	0	0	0	0

Table 30 (a, b, c) Expression profiling of AtZF-BED in different tissues and under Biotic and Abiotic stresses.

		Tissues											
**GENE ID**	**Gene Name**	**Embryo**	**Endosperm**	**Flower**	**Leaf**	**Meristem**	**Pollen**	**Root**	**Seed**	**Seedling**	**Silique**	**Shoot**	**Stem**
AT1G18560	AtZf-BED01	10.4456695	10.234241	8.031907308	5.35426242	4.9691072	1.313584	6.636908245	6.106163611	5.996539305	8.163836	6.84864	7.152531
AT1G36095	AtZf-BED02	0	0	0	0.00132806	0	0	0	0	0.004231838	0	0	0
AT1G79740	AtZf-BED03	17.1949268	14.971778	21.66002437	18.4079647	15.5720998	1.2563537	15.98220586	15.43074986	17.19243883	18.32795	20.69158	23.92326
AT3G17450	AtZf-BED04	21.586378	20.50754	20.93562154	13.9549416	18.1851794	6.457879	15.98711976	20.12310997	15.38630058	16.93969	15.75546	16.30245
AT3G48770	AtZf-BED05	9.08329745	0.5497011	4.298380023	0.45146214	0.5471386	0	1.164605734	0.092199972	0.386729622	4.260353	0.465479	2.551806
AT4G15020	AtZf-BED06	40.2954745	36.474586	26.81544311	18.6064777	29.8368792	44.116459	21.67747669	40.28374647	18.50599655	27.0873	20.19579	21.20303

		Abiotic stresses																
**GENE ID**	**Gene Name**	**Cold**	**Dark**	**Dehydration**	**Drought**	**Heat**	**Hypoxia**	**Irradiated**	**Nutrient deficiency**	**Osmotic**	**Oxidative**	**Ozone**	**Salt**	**Shade**	**Uv**	**Water**	**Wounding**
AT1G18560	AtZf-BED01	5.19753274	4.9879511	6.928926462	6.25373071	4.71025042	0	3.881891417	6.290828439	8.69435333	8.151122	6.7857249	5.61183	5.985011	5.9696565	7.301319	7.0473409
AT1G36095	AtZf-BED02	0	0.266008	0	0.0300523	0	0	0	0.009236581	0	0	0	0	0	0	0	0
AT1G79740	AtZf-BED03	17.834196	21.97229	16.70978835	19.3182554	17.0162097	0	6.81468575	16.75204664	20.3513487	8.592474	14.392438	19.8449	17.4637	15.862256	24.28617	19.04000058
AT3G17450	AtZf-BED04	13.9986139	16.0298	19.24112377	14.6853542	17.1128312	0	9.556921167	15.92530265	17.1197947	14.68853	14.360298	16.9964	12.60595	14.581849	15.30474	17.06740255
AT3G48770	AtZf-BED05	0.66840672	3.53629	0.473702269	0.5535493	0.46300445	0	2.83014125	0.866563581	0.28150167	0.266038333	0.4817078	0.82611	0.127554	1.0890093	0.142295	0.2622415
AT4G15020	AtZf-BED06	22.9371306	22.65117	26.90915865	19.876494	20.0308848	0	16.17654892	21.00576903	20.5943577	23.10886067	21.937883	16.5901	18.05834	23.200838	19.68593	20.28373608

		Biotic stresses																
**GENE ID**	**GeneNa**	**Bacterial strain sa188**	**Blumeria gram**	**Botryis cine**	**Colletotrichum tofieldiae**	**Fusarium graminearum**	**Heterodera schachti nema**	**Heterodera schachtii para**	**Hyaloperonospora arabidopsis**	**Microbacterium sp**	**Plutella xylos**	**Pseudomonas simiae**	**Pseudomonas syringae**	**Rhizobium**	**Rhizoctonia solani**	**Sclerotinia sclerotiorum**	**Tobacco mosaic**	**Verticillium dahliae**

	me		inis	rea			todes	sitism			tella			sp			vires	
AT1G18560	AtZf-BED01	11.0985587	6.4373812	6.448442467	7.57227317	8.27523	6.217457	7.202936	2.782337339	3.24001175	5.7065985	8.647433333	6.646668579	8.63228433	4.0224349	2.493978333	6.9387055	10.235834
AT1G36095	AtZf-BED02	0	0	0	0	0	0	0	0	0	0	0	0	0	0	0	0	0
AT1G79740	AtZf-BED03	13.0907937	18.046309	19.0180714	16.9543293	27.3950265	16.830982	23.0275375	5.175692075	14.49525925	19.020222	20.49922058	17.08208007	10.9213372	17.444711	3.157787333	26.8838465	21.257752
AT3G17450	AtZf-BED04	18.595879	14.628365	16.63507553	19.1608487	21.694296	18.755313	20.1838235	6.82450454	12.34224888	13.4776685	20.01200558	13.34193295	16.7248965	12.012884	14.756425	10.204465	22.46238533
AT3G48770	AtZf-BED05	1.269062	0.146652	0.3137046	1.45437075	0.078761	1.9073868	4.9008065	0.084895977	0.185966125	0.298652	1.131114417	0.402707993	0.37080083	0.136892	0	0.5276935	0.802585333
AT4G15020	AtZf-BED06	22.804298	20.51311	21.95205547	26.6380483	21.9327165	21.417139	22.501627	10.58134795	15.05736725	16.222036	22.26065442	19.4670181	25.1512197	14.56955	23.41602767	9.9019165	25.591119

Table S31 Orthologs of Arabidopsis, *S. lycopersicon*, *Z. mays* and *G. hirsutum*

Orthogroup	*A.thaliana*	*S. lycopersicon*	*G. hirsutum*	*Z. mays*
OG0000000	AT4G15020.1	Solyc09g005660.2	Ghir_D05G011710.1, Ghir_D13G002100.1, Ghir_D13G002100.2, Ghir_A13G001820.1, Ghir_A13G001820.3, Ghir_A13G001820.2, Ghir_A07G008970.1	Zm00001d000412_P001, Zm00001d004256_P001, Zm00001d010895_P001, Zm00001d016617_P001, Zm00001d025324_P001, Zm00001d029192_P001, Zm00001d033361_P001, Zm00001d039328 Zm00001d050879_P001
OG0000000	AT1G79740.1	Solyc08g007470, Solyc03g007510.2	Ghir_D03G017110.1, Ghir_D03G017110.2, Ghir_A03G001860.1, Ghir_A03G001860.2	Zm00001d000412_P001, Zm00001d004256_P001, Zm00001d010895_P001, Zm00001d016617_P001, Zm00001d025324_P001, Zm00001d029192_P001, Zm00001d033361_P001, Zm00001d039328 Zm00001d050879_P001
OG0000007	AT1G18560.1	Solyc03g119830	Ghir_A11G000730.1	No
